# Predicting lung adenocarcinoma prognosis, immune escape, and pharmacomic profile from arginine and proline-related genes

**DOI:** 10.1038/s41598-023-42541-z

**Published:** 2023-09-14

**Authors:** Ziqiang Wang, Jing Zhang, Shuhua Shi, Hongyu Ma, Dongqin Wang, Chao Zuo, Qiang Zhang, Chaoqun Lian

**Affiliations:** 1 Research Center of Clinical Laboratory Science, Bengbu Medical College, Bengbu, 233030 China; 2 Department of Genetics, School of Life Sciences, Bengbu Medical College, Bengbu, 233030 China; 3 Department of Clinical Medicine, Bengbu Medical College, Bengbu, 233030 China; 4 Department of Clinical Laboratory, Affiliated Hospital of Guilin Medical University, Guilin, 541001 China; 5 Department of Clinical Laboratory, The First Affiliated Hospital of Bengbu Medical College, Bengbu, 233004 China

**Keywords:** Biochemistry, Cancer, Immunology, Molecular biology, Biomarkers, Oncology

## Abstract

Lung adenocarcinoma (LUAD) is a highly heterogeneous disease that ranks first in morbidity and mortality. Abnormal arginine metabolism is associated with inflammatory lung disease and may influence alterations in the tumor immune microenvironment. However, the potential role of arginine and proline metabolic patterns and immune molecular markers in LUAD is unclear. Gene expression, somatic mutations, and clinicopathological information of LUAD were downloaded from The Cancer Genome Atlas (TCGA) database. Univariate Cox regression analysis was performed to identify metabolic genes associated with overall survival (OS). Unsupervised clustering divided the sample into two subtypes with different metabolic and immunological profiles. Gene set enrichment analysis (GESA) and gene set variation analysis (GSVA) were used to analyze the underlying biological processes of the two subtypes. Drug sensitivity between subtypes was also predicted; then prognostic features were developed by multivariate Cox regression analysis. In addition, validation was obtained in the GSE68465, and GSE50081 dataset. Then, gene expression, and clinical characterization of hub genes CPS1 and SMS were performed; finally, in vitro validation experiments for knockdown of SMS were performed in LUAD cell lines. In this study, we first identified 12 arginine and proline-related genes (APRGs) significantly associated with OS and characterized the clinicopathological features and tumor microenvironmental landscape of two different subtypes. Then, we established an arginine and proline metabolism-related scoring system and identified two hub genes highly associated with prognosis, namely CPS1, and SMS. In addition, we performed CCK8, transwell, and other functional experiments on SMS to obtain consistent results. Our comprehensive analysis revealed the potential molecular features and clinical applications of APRGs in LUAD. A model based on 2 APRGs can accurately predict survival outcomes in LUAD, improve our understanding of APRGs in LUAD, and pave a new pathway to guide risk stratification and treatment strategy development for LUAD patients.

## Introduction

Lung adenocarcinoma (LUAD) is one of the most common respiratory tumors and is classified as non-small cell lung cancer (NSCLC), accounting for approximately 40% of all lung cancers. According to the latest research data published by the World Health Organization (WHO), LUAD ranks second among all tumors worldwide and has the highest mortality rate, with an average 5-year survival rate of only 15%^1–3^. Lung cancer also exhibits molecular and genetic heterogeneity^4,5^, which presents a challenge for the diagnosis and treatment of lung cancer. In recent years, immunotherapies such as immune checkpoint inhibitors (ICIs) have brought a new direction to lung cancer treatment and have been shown to improve survival in advanced NSCLC^6^. However, only some lung adenocarcinoma patients respond to them, and most patients are prone to primary and secondary drug resistance^7,8^. Therefore, there is a need to find new molecular markers and construct prognostic models to stratify and tailor treatment regimens for patients with lung adenocarcinoma.

In the tumor microenvironment (TME), tumor cells form a metabolic competition with immune cells. Many tumors have evolved to evade immune surveillance, using their metabolic flexibility and redirecting nutrients to their advantage^9^. Among these, amino acid metabolism plays an important role in tumor cell growth and survival, including providing a carbon source for the TCA cycle, a nitrogen source for base synthesis, and regulating redox homeostasis. Previous studies have identified the involvement of arginase in obstructive airway diseases such as asthma and pulmonary hypertension^10–12^. The latest study shows that arginine metabolism is closely related to TME^13,14^ and is involved in T-cell activation and immunomodulatory responses^15^. As a multifunctional amino acid, arginine has multiple biological functions in metabolism and signaling pathways, and many tumors exhibit exogenous arginine-dependent growth^16^, and can maintain tumor growth through the synthesis of derived metabolic polyamines^17^. Proline metabolism is associated with ATP production, protein and nucleotide synthesis, and redox homeostasis in tumor cells^18^. The inhibition of proline biosynthesis is thought to inhibit tumor growth. For example, Myeloid-derived suppressor cells (MDSCs) that play an immunosuppressive role in TME can express high levels of ARG1, iNOS, TGF-β, ROS, IDO, etc., while depleting arginine to inhibit T cell function to promote tumor immune escape^19,20^. Meanwhile, arginine deprivation therapies targeting arginine have shown positive results in clinical practice^21,22^. In contrast, the molecular alterations and metabolic profiles of arginine and proline as non-essential amino acids in lung adenocarcinoma have not been fully investigated.

Carbamoyl phosphate synthase 1 (*CPS1*) is the first rate-limiting enzyme of the urea cycle, and some LUAD patients show significant overexpression^23,24^, has been found to maintain pyrimidine and DNA synthesis in *KRAS/LKB1* co-mutated non-small cell lung cancer (NSCLC) and imposes a specific metabolic vulnerability^25^. And the study suggests that *CPS1* may be a biomarker of poor prognosis and a potential therapeutic target. Spermine synthase (*SMS*), a key enzyme in polyamine metabolism, plays an important role in spermine anabolism^26^. High expression in cancers such as hepatocellular carcinoma and head and neck squamous cell carcinoma is associated with poor prognosis^27,28^. In addition, recent studies have shown that *SMS* and *MYC* can synergistically inhibit the expression of pro-apoptotic protein Bim to maintain the growth of colorectal cancer cells^29^. The dysregulation of polyamine metabolism and its requirement occurs frequently in tumors, and multiple oncogenes *MYC* and *RAS* play a direct role in regulating polyamine synthesis, which represents a complementary relationship between polyamine metabolism and tumor proliferation regulation to promote cancer progression^30^.

In this study, we derived two metabolic subtypes of LUAD by clustering analysis of ARG metabolic genes, with significant differences between subtypes in terms of prognosis, metabolism and immune infiltration. It was also observed that subtype 2 has immune escape-related features and may be insensitive to immunotherapy. In addition, we constructed a prognostic model to predict overall survival in LUAD and then analyzed two modeled genes, *CPS1* and *SMS*. Finally, a series of cellular experiments were performed on *SMS*, and it was shown that knockdown of *SMS* significantly reduced the proliferation, migration, and invasive ability of lung adenocarcinoma cells in vitro.

## Methods

### Data collection and processing

Transcriptomic data and corresponding clinical data of LUAD patients were obtained from the TCGA (https://portal.gdc.cancer.gov/) database and GEO (http://www.ncbi.nlm.nih.gov/geo/) database. Construction of relevant prognostic features using 500 LUAD cases from the TCGA database, and the sample inclusion criteria for TCGA were 01A (Primary Tumor) type samples containing complete survival information. GSE68465 were used to validate the prognostic and immunological features of LUAD based on genes related to arginine and proline metabolism. To avoid the interference of irrelevant factors, most tumor samples with a follow-up time greater than 2000 days were excluded, a total of 333 samples were included in the GSE68465 dataset, and the GSE68465 dataset was used as the first validation set. The datasets GSE30219, GSE37745, and GSE50081 from the same microarray platform (Affymetrix HG-U133 Plus 2.0 Array) were integrated into a new cohort and used as the second validation set to validate the ARG-based prognostic model, and sample inclusion criteria for the GSE50081 integration cohort were samples with the histologic type of adenocarcinoma. The 54 arginine and proline-related genes were obtained from the human C2 KEGG gene set of the MsigDB database (KEGG_ARGININE_AND_PROLINE_METABOLISM)^31^. These genes were integrated with clinical information from 500 lung adenocarcinoma samples for univariate Cox regression analysis, and 12 APRGs associated with OS were screened according to p < 0.05 before subsequent analysis. Finally, two prognostic hub genes were identified by stepwise multivariate regression analysis. The RNA-seq data types processed for TCGA were log2(TPM + 1). The transcriptome-in-a-million (TPM) values for the LUAD cell line transcriptome were obtained from the Cancer Cell Line Encyclopedia (CCLE) (https://sites.broadinstitute.org/ccle/).

### Analysis of prognostic characteristics associated with APRGs

Univariate COX regression analysis was performed on genes related to arginine and proline metabolism using the “survival” package to screen metabolic genes associated with overall survival (OS) in LUAD, and the R package “ggplot2” was used for mapping forest plot. Prognostic genes were identified at a p < 0.05 criterion. The 12 genes associated with survival were analyzed univariately and then clustered by consensus using the R package “ConsensusClusterPlus”. The optimal number of clusters was assessed by the cumulative distribution function (CDF) plot and the consensus heat map with an optimal k-value of 2. The distribution of groups was studied using the UAMP dimensionality reduction analysis using the R package “umap”. In addition, univariate and multivariate Cox regression analyses were performed to confirm whether characteristics could be used as independent predictors of survival. The R package “survival” and “survminer” were applied, survival analysis was performed and survival curves were plotted, and log-rank tests were performed to assess the difference in survival between the two groups. p < 0.05 was considered significant.

### Inter-subtype immune infiltration assessment

We used ESTIMATE algorithm to assess the level of immune cell infiltration, stromal content, and tumor purity for each sample. The enrichment levels of 64 immune features were quantified by the Xcell algorithm, and the relative proportions of 22 immune cell types in each tumor tissue were estimated using the CIBERSORT algorithm, and samples with p > 0.05 in the results were excluded and the remaining samples were further analyzed^32^. Single-sample gene enrichment analysis (ssGSEA) was introduced to quantify the relative infiltration of 28 immune cell types in the tumor microenvironment, with unique combinations of characteristic genes for each immune cell subtype derived from recent literature^33,34^. The results were presented using the ggplot2, ggpubr, and pheatmap R packages.

### Gene set variance analysis (GSVA)

We used the ssGSEA and GSVA algorithms in the R package “GSVA” to study the variation of biological processes among two clusters. Well-defined biological features were obtained from the Hallmark gene set (h.all.v2023.1.Hs.symbols) and the gene set constructed by Mariathasan et al. (from the IMvigor210CoreBiologies package)^35^. The gene set “c2.cp.kegg.v7.4.symbols.gmt” was also downloaded from MSigDB as a reference gene set.

### Functional enrichment analysis

By using the “limma” and “clusterProfiler” R packages^36^ for ontology (GO) enrichment analysis, FDR < 0.05 and |logFC| > 1 were set as threshold values. Gene set enrichment analysis (GSEA) was used to explore potential functional and signaling pathway enrichment in patients with two immune escape subtypes of lung adenocarcinoma.

### Tumor mutation load and immunotherapy response prediction

Somatic mutations were obtained from the TCGA database (https://portal.gdc.cancer.gov/). Somatic mutation data were integrated using the R package “maftools”, and mutations in five tumor driver genes were analyzed to extract tumor mutational load (TMB) values. In addition, given that T-cell inflammatory GEP and PD-L1 (*CD274*) expression predict immunotherapeutic response, we evaluated GEP scores and PD-L1 expression levels between the two subtypes. Predicting potential immune checkpoint blockade responses (ICB) in LUAD using tumor immune dysfunction and exclusion (TIDE)^37^. Higher TIDE scores indicate that cells are more likely to induce immune escape and thus have a lower response rate to ICB treatment. We also invoked an immune signature-based approach for hot and cold tumor identification and analyzed the differences in response to immunotherapy and the proportion of hot and cold tumors between the two subtypes.

### External data validation of LUAD-related features

To validate the accuracy of prognostic and immune escape features in the TCGA cohort, we performed cluster analysis on samples from lung adenocarcinoma patients in the GSE68465 dataset from the GEO database using the same methods as in TCGA for prognosis, immune, and MHC molecular expression in LUAD, respectively.

### Drug sensitivity analysis

The OncoPredict R software package was developed by Maeser et al. to predict in vivo drug sensitivity in cancer patients^38^. Maeser et al. using the “oncoPredict” package, GDSC2 gene expression profiles and corresponding drug response information can be downloaded^39^. To generate a ridge regression model that could be applied to lung adenocarcinoma transcriptomic data was used to score the sensitivity to predict the half-maximal inhibitory concentration (IC50) of 198 drugs between the two subtypes. p < 0.05 was the threshold of significance.

### Construction and validation of a prognostic model for ARG-related genes

To further explore the association between prognosis-related core genes and lung adenocarcinoma prognosis, we performed multivariate Cox regression analysis on 12 modeled genes and constructed a risk model based on genes related to arginine and proline metabolism, resulting in the risk score formula risk score = Ʃ (βi × Expi). Where βi coefficient represents the weight of the respective marker and Expi represents the expression value. According to the risk score formula, patients were divided into low-risk and high-risk groups, with the median risk score as the cut-off point. Subsequently, nomograms predicting the likelihood of OS at 1, 3, and 5 years were constructed by the R package “rms” based on the results of multivariate Cox regression analysis. Calibration plots were drawn to assess the predictive accuracy of the nomograms. To validate the predicted values of metabolism-related features, we used TCGA data as the training set and GSE68465, GSE50081, and other datasets as the validation cohort. The R package “survminer” was used to estimate OS by survival analysis in the high-risk and low-risk groups, and the R package “timeROC” was used to assess the prognostic value of the feature model. In addition, we analyzed the possibility of risk score as an independent prognostic feature using univariate and multivariate Cox regression.

### CPS1 and SMS gene analysis

First, we analyzed the expression levels of two modeled genes, *CPS1* and *SMS*, in LUAD normal versus LUAD by TCGA database and GEPIA2 (http://gepia2.cancer-pku.cn/#analysis) database^40^, then followed by protein expression analysis in the UALCAN database. Kaplan Meier (http://kmplot.com/analysis) and GEPIA online databases were used to analyze the survival curve analysis of the *CPS1* and *SMS* genes^41^. In addition, we obtained mRNA expression levels of *CPS1* and *SMS* in a variety of lung adenocarcinoma cell lines in the CCLE database. Immunohistochemical analysis of protein expression of CPS1 and SMS in normal and LUAD tissues was downloaded from the Human Protein Atlas (HPA) database (http://www.proteinatlas.org/)^42^. The correlation of *CPS1* and *SMS* gene expression with clinical characteristics such as age, gender, stage, and TNM stage was also analyzed.

### Cell culture and transfection

Human lung adenocarcinoma cell lines A549 and H1299 were mainly purchased from the cell bank of the Chinese Academy of Sciences (Shanghai, China). We performed in vitro culture experiments using A549 and H1299 cells cultured in DMEM medium and RPMI 1640 medium (Gibco, ThermoFisher Scientific, United States) supplemented with 10% fetal bovine serum, 1% penicillin and streptomycin (Gibco). Small interfering RNA (siRNA) targeting *SMS* and interfering RNA control were purchased from Gima Genetics (Shanghai, China). For transient transfection, A549 and H1299 cells were transfected with siRNA for 12 h using a transfection reagent (Lipofectamine 2000), followed by functional assays.

### RT-qPCR and western blotting

RNA was extracted from lung adenocarcinoma cell lines (A549, H1299) interfering with si-SMS and NC as controls. SYBR Green qPCR mix (Vazyme, China) was used to synthesize cDNA for real-time PCR. primers were as follows: *SMS*-Forward: TAGTGGGGATGTAATTTGGCAG; *SMS*-Reverse: CCACACGTTTTTCGCATGTATTT; *GAPDH*-Forward: GACCACAGTCCATGCCATCA; *GAPDH*-Reverse: GTCAAAGGTGGAGGAGTGGG. Protein blotting analysis of RIPA lysis buffer (Servicebio, China) containing PMSF (Servicebio, China) was used to collect proteins from A549 and H1299 cells. 10% sodium dodecyl sulfate–polyacrylamide gel electrophoresis (SDS-PAGE) was used to separate protein samples, and polyvinylidene difluoride (PVDF) membranes (Immobilon-P, Carlsbad, Ireland) were used to transfer the separated proteins. The closure was performed for 15 min using Rapid Closure Solution, followed by incubation with primary antibodies: *SMS* (Proteintech, 15979-1-AP, 1:1000) and *Vinculin* (Proteintech, 66305-1-Ig, 1:50,000) overnight at 4 °C, followed by secondary antibody incubation for 2 h. Original images of the western blot are available in the Supplementary File.

### Proliferation and colony formation experiments

Cell proliferation and colony formation assays were performed 24 h after transfection with *SMS* siRNA, and A549 and H1299 cells were cultured in 96-well plates (3000 cells/well). The proliferation capacity of the treated cells was assayed at 4, 24, 48, and 72 h. 10% Cell Counting Kit-8 (CCK8) reagent (Bio-sharp, Hefei, China) was added to each plate according to the kit instructions, and OD450 values were analyzed using an enzyme marker (BioTek, United States). For colony formation experiments, 2000 cells were inoculated in cell culture plates and allowed to grow until visible colonies were formed. We then fixed the clones with paraformaldehyde for 15 min, stained the clones with 1% crystalline violet for 20 min, and counted the number of clones (> 50 cells).

### Transwell migration invasion and wound healing assay

Transwell migration and wound healing assays A549 and H1299 cells were transfected with *SMS* siRNA for 24 h and cultured in 24-well culture plates with 8 mm pore membrane inserts to measure cell migration and invasion capacity. 4 × 10^4^ cells were inoculated in 200 μl of serum-free medium in the upper chamber of the transwell, and 800 μl of medium containing 10% FBS was added to the lower chamber. After incubation for 48 h, cells that migrated across the membrane were fixed with paraformaldehyde, stained with 1% crystal violet and counted under a light microscope (200×). In addition, A549 and H1299 cells were cultured in 24-well plates and scraped with a 200 μl pipette tip. Cells were cultured in DMEM and RPMI 1640 medium without FBS. Wound images were captured at 0 and 24 h and the wound area was quantified by ImageJ software (40×).

### Statistical analysis

Statistical analysis and plots were performed using R software (version 4.0.1) and GraphPad software. The Wilcoxon test was used for the test between the two paired groups, categorical variables were compared by Chi-square test or Fisher exact test, and the statistical significance of the cell line experiment was assessed by t-test in GraphPad Prism version 9 software. Differences were considered statistically significant at *p < 0.05, **p < 0.01, ***p < 0.001, and ****p < 0.0001.

### Ethics approval

The data used in this study were obtained from publicly available datasets, such as the GEO database (https://www.ncbi.nlm.nih.gov/geo), and The Cancer Genome Atlas (https://portal.GDC.cancer.gov).

## Results

### Identification of metabolic isoforms of Arg and Pro metabolism

**Figure 1:**
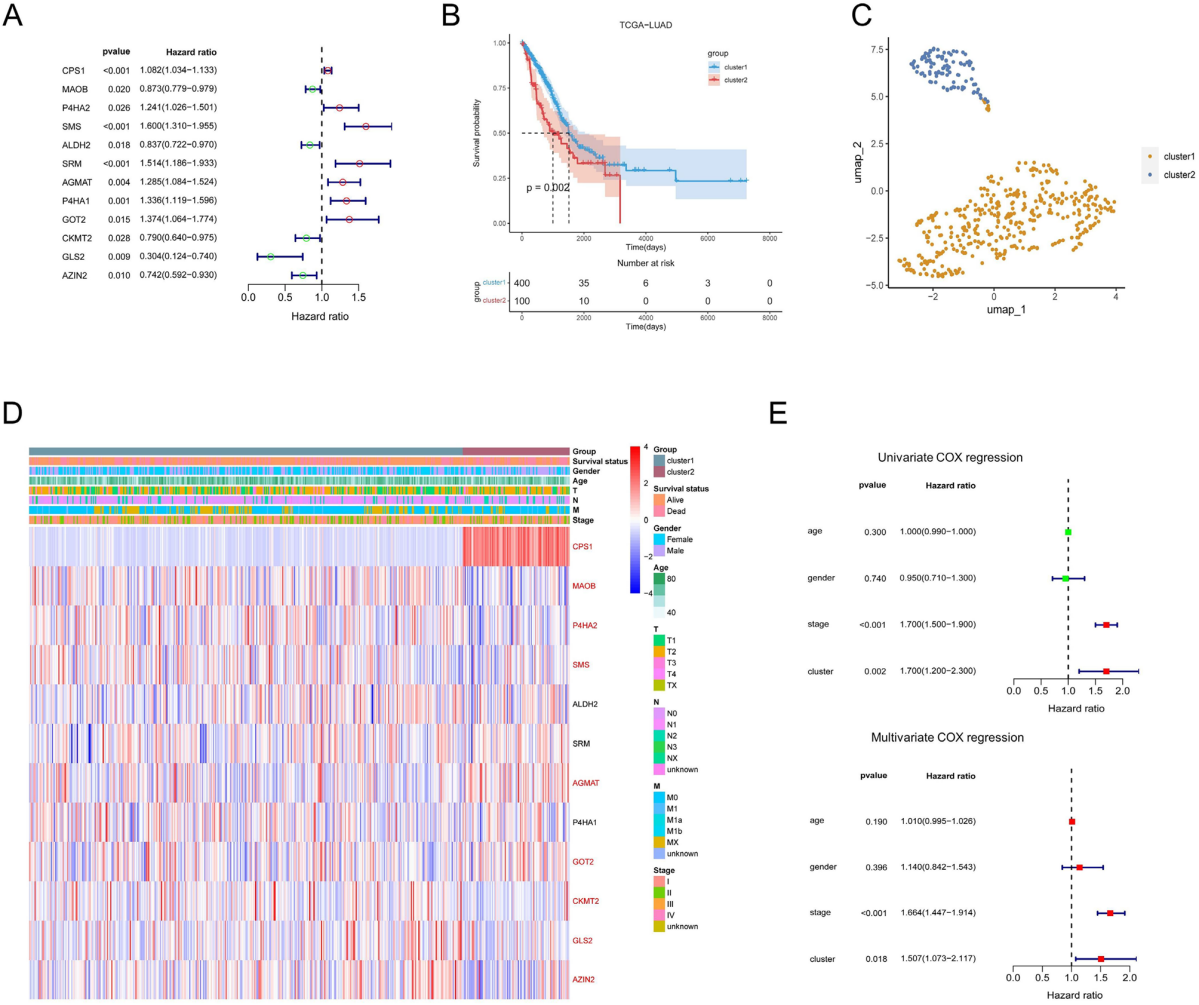
Identification of arginine and proline related cluster. (**A**) Identified these differentially expressed APRGs related to the LUAD risks by univariate Cox regression analysis. (**B**) Kaplan–Meier survival analysis of the two clusters. (**C**) Sample clustering by UMAP in the TCGA dataset. (**D**) Differences in clinicopathologic characteristics and expression levels of APRGs between the two clusters. (**E**) Univariate and multivariate Cox regression of clinicopathological factors and APRG subtypes. *APRGs* arginine and proline related genes.

We sought to explore the prognostic value of arginine and proline metabolism-related genes (APRGs) about LUAD, using univariate Cox regression analysis for 54 metabolic genes, and 12 genes were associated with OS (p < 0.05) in the TCGA cohort, namely *CPS1*, *MAOB*, *P4HA2*, *SMS*, *ALDH2*, *SRM*, *AGMAT*, *P4HA1*, *GOT2*, *CKMT2*, *GLS2*, and *AZIN2* (Fig. 1A). We performed consistent clustering of the 12 prognosis-related genes in the TCGA dataset and identified two clusters (Supplementary Fig. S1A,B). Survival analysis of both clusters demonstrated lower survival probability curves and significant differences in prognosis for cluster 2 (Fig. 1B) and obtained the same results in the GEO dataset (Supplementary Fig. S2A–C). In addition, we obtained clinical information for both clusters (Table 1). The UMAP downscaling analysis indicated them as two distinct groups (Fig. 1C). Next, we compared the clinicopathological characteristics and prognosis related to APRG expression between the two subgroups (Fig. 1D), and *CPS1* is significantly overexpressed in cluster 2. Then, we used univariate and multivariate Cox regression analysis to confirm the cluster grouping as independent prognosis for LUAD factors (Fig. 1E).

**Table 1:** TCGA-LUAD Clinical characteristics.

Characteristics	Cluster1 (N = 400)	Cluster2 (N = 100)	Overall (N = 500)	p-value
Age				
< 65	173 (43.3%)	47 (47.0%)	220 (44.0%)	0.796
≥ 65	227 (56.8%)	53 (53.0%)	280(56.0%)	
Gender				
Male	166 (41.5%)	64 (64.0%)	230 (46.0%)	< 0.001
Female	234 (58.5%)	36 (36.0%)	270 (54.0%)	
Stage				
I	225 (56.3%)	225 (56.3%)	269 (53.8%)	0.482
II	94 (23.5%)	25 (25.0%)	119 (23.8%)	
III	58 (14.5%)	58 (14.5%)	80 (16.0%)	
IV	17 (4.3%)	8 (8.0%)	25 (5.0%)	
Unknown	6 (1.5%)	1 (1.0%)	7 (1.4%)	
T stage				
T1	145 (36.3%)	23 (23.0%)	168 (33.6%)	0.538
T2	205 (51.3%)	63 (63.0%)	268 (53.6%)	
T3	33 (8.3%)	10 (10.0%)	43 (8.6%)	
T4	15 (3.8%)	3 (3.0%)	18 (3.6%)	
TX	2 (0.5%)	1 (1.0%)	3 (0.6%)	
N stage				
N0	262 (65.5%)	61 (61.0%)	323(64.6%)	0.790
N1	79 (19.8%)	16 (16.0%)	95 (19.0%)	
N2	48 (12.0%)	21 (21.0%)	69 (13.8%)	
N3	2 (0.5%)	0 (0%)	2 (0.4%)	
NX	8 (2.0%)	2 (2.0%)	10 (2.0%)	
Unknown	1 (0.3%)	0 (0%)	1 (0.2%)	
M stage				
M0	259 (64.8%)	76 (76.0%)	335(67.0%)	0.0808
M1	16 (4.0%)	8 (8.0%)	24 (4.8%)	
MX	122 (30.5%)	15 (15.0%)	137 (27.4%)	
Unknown	3 (0.8%)	1 (1.0%)	4 (0.8%)	

### Metabolism-related characterization

**Figure 2:**
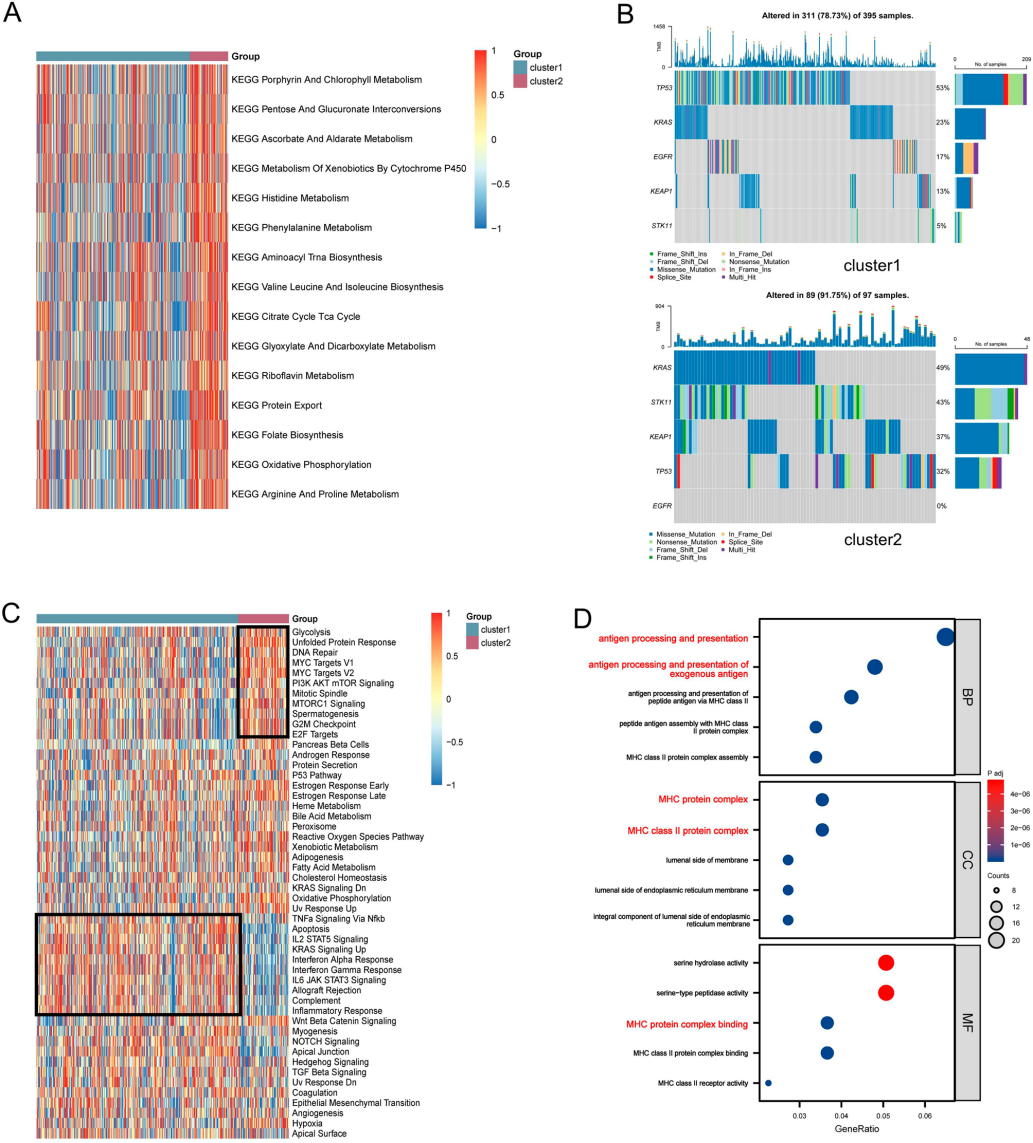
Enrichment analysis and mutation analysis of two APRGs clusters. (**A**) GSVA of biological pathways between two clusters, in which blue inhibited and red represent activated pathways, respectively. (**B**) The somatic mutations of tumor driver genes in two clusters. (**C**) Heatmap shows the enriched biological pathways calculated by GSVA algorithm in two clusters. (**D**) GO enrichment analysis shows the BP, CC, and MF of two clusters.

To explore the biological characteristics of both clusters, GSVA enrichment analysis revealed that we found a significant enrichment in the metabolism of various substances in cluster 2 (Fig. 2A). Furthermore, we investigated the somatic mutations of tumor driver genes in both clusters, including *TP53*, *KRAS*, *STK11*, *EGFR*, and *KEAP1*. The results showed that cluster 2 possessed a higher overall mutation rate and higher mutations in *KRAS*, *STK11*, and *KEAP1* genes compared to cluster 1^43^ (Fig. 2B). Subsequently, to explore the differences in the biological processes of the two clusters, we performed a gene set variation analysis (GSVA) for the Hallmark gene set in LUAD tumors (Fig. 2C). The results showed that cluster 1 was significantly enriched for immune infiltration-related pathways, including IFN-γ/α response, IL2/STAT5 signaling pathway, IL6/JAK/STAT3 signaling pathway, allograft rejection, and inflammatory response; cluster 2 was significantly enriched for pathways significantly associated with oncogenic activation and highly proliferative features, including unfolded protein response, MYC target V1/V2, PI3K/AKT/mTOR, G2M checkpoint, and E2F targets. GO enrichment analysis revealed that biological processes (BP) were mainly enriched in antigen presentation processes, and both cellular components (CC) and molecular functions (MF) were associated with the MHC protein complex (Fig. 2D). By using the subsequent analysis of Mariathasan et al. this feature set indicated enhanced angiogenesis and EMT activity in cluster 2, with cluster 1 exhibiting higher CD8T cell effector capacity (Supplementary Fig. S3A).

### Immune-related characterization

**Figure 3:**
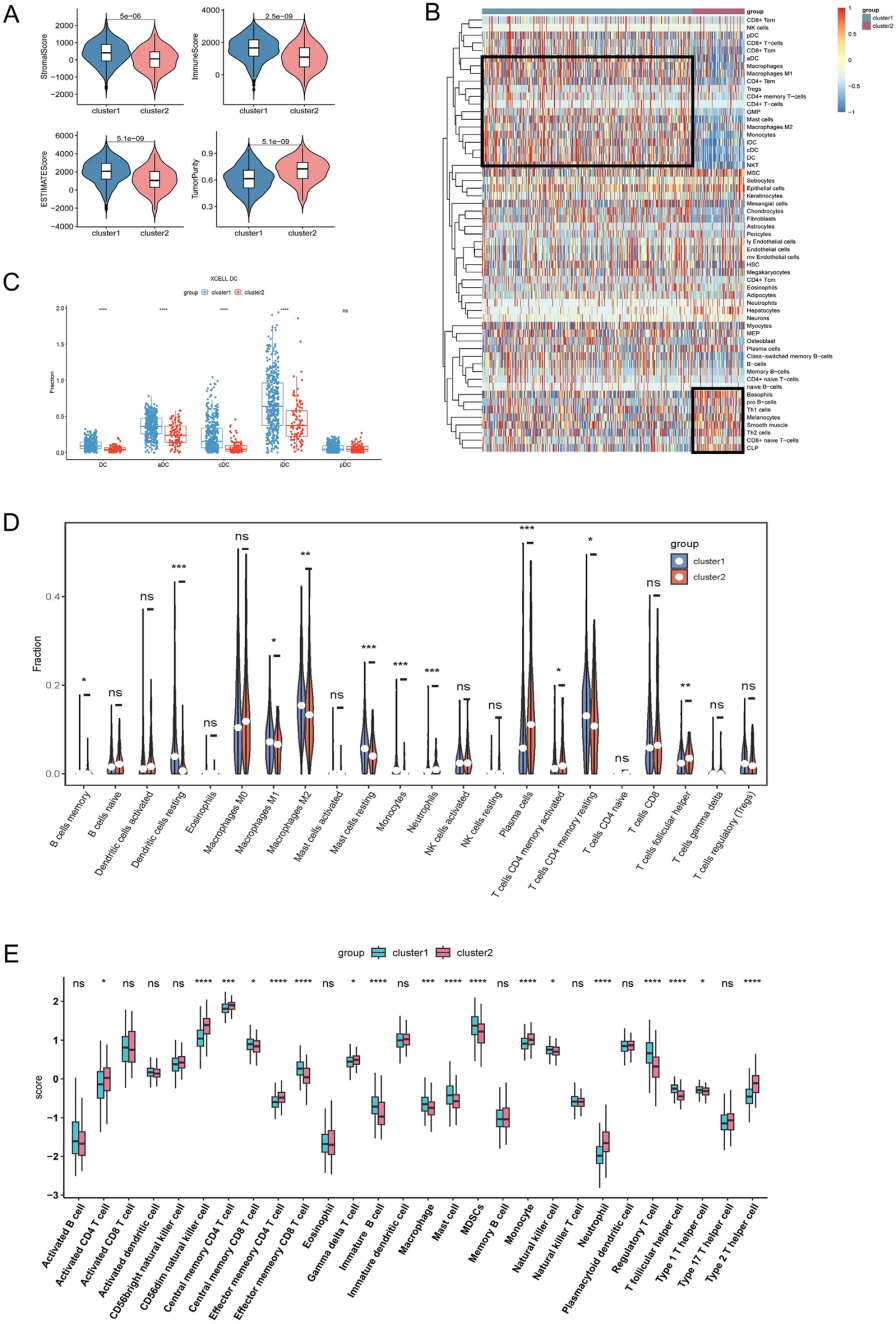
(**A**) Immune infiltration characteristics analysis. (**A**) ESTIMATEScores, ImuneScores, StromalScores, and TumorPurity of the two clusters in TCGA. (**B**) Heatmap demonstrates the 64 cell levels in the Xcell algorithm. (**C**) Proportion of dendritic cells between two clusters. (**D**) The comparison of 22 types of adaptive and innate immune cells between two clusters. (**E**) Expression abundance of 28 infiltrating immune cell types in the two clusters. *p < 0.05, **p < 0.01, ***p < 0.001, ****p < 0.0001, ^ns^p > 0.05.

To explore APRG-based immune-related features, we assessed the overall immune infiltration between the two clusters using the ESTIMATE algorithm, with cluster 1 exhibiting higher immune scores and stromal scores and cluster 2 exhibiting higher tumor purity (Fig. 3A). xCell algorithm heat maps showed significantly lower numbers of mast cells, M2-type macrophages, dendritic cells, and fibroblasts in cluster 2, while there was also a trend of lower CD8+ T cells and B cells (Fig. 3B). As the most functional antigen-presenting cells (APCs) in the organism, dendritic cells play an important role in tumor immunity, and the number of infiltrating dendritic cells within most solid tumors is positively correlated with prognosis. Next, we compared the percentage of several dendritic cells in the xCell algorithm in both clusters (Fig. 3C). Thus, we hypothesized that the tumor antigen presentation step in cluster 2 during the tumor immune cycle was missing or impaired. This was subsequently confirmed by comparing the cancer immune cycle scores of the two and revealed a significantly reduced recruitment of CD8+ T cells and CD4+ T cells in cluster 2 (Supplementary Fig. S3B). The CIBERSORT algorithm subsequently showed that resting dendritic cells were significantly elevated in cluster 1, and the elevation of plasma cells in cluster 2 was also brought to our attention (Fig. 3D). We did not find significant differences in active CD8+ T cells in the ssGSEA algorithm, but CD56dim NK cells and memory CD4+ T cells were significantly increased in cluster 2 (Fig. 3E). Also, we noted a significant overexpression of T helper cells 2 (Th2 cells) in cluster 2 compared to higher Th1 cells in cluster 1, i.e. a possible Th1/Th2 drift in cluster 2, which is consistent with a poorer prognosis and immune infiltration performance in cluster 2. Overall, we identified two subtypes in LUAD based on the transcriptomic profile of APRGs and found that cluster 2 has many biological features of poor prognosis, with deletion of dendritic cells and impaired T-cell activation as its main immune infiltrative features, which may represent a close association of cluster 2 with tumor immune escape.

### Immune escape characterization

**Figure 4:**
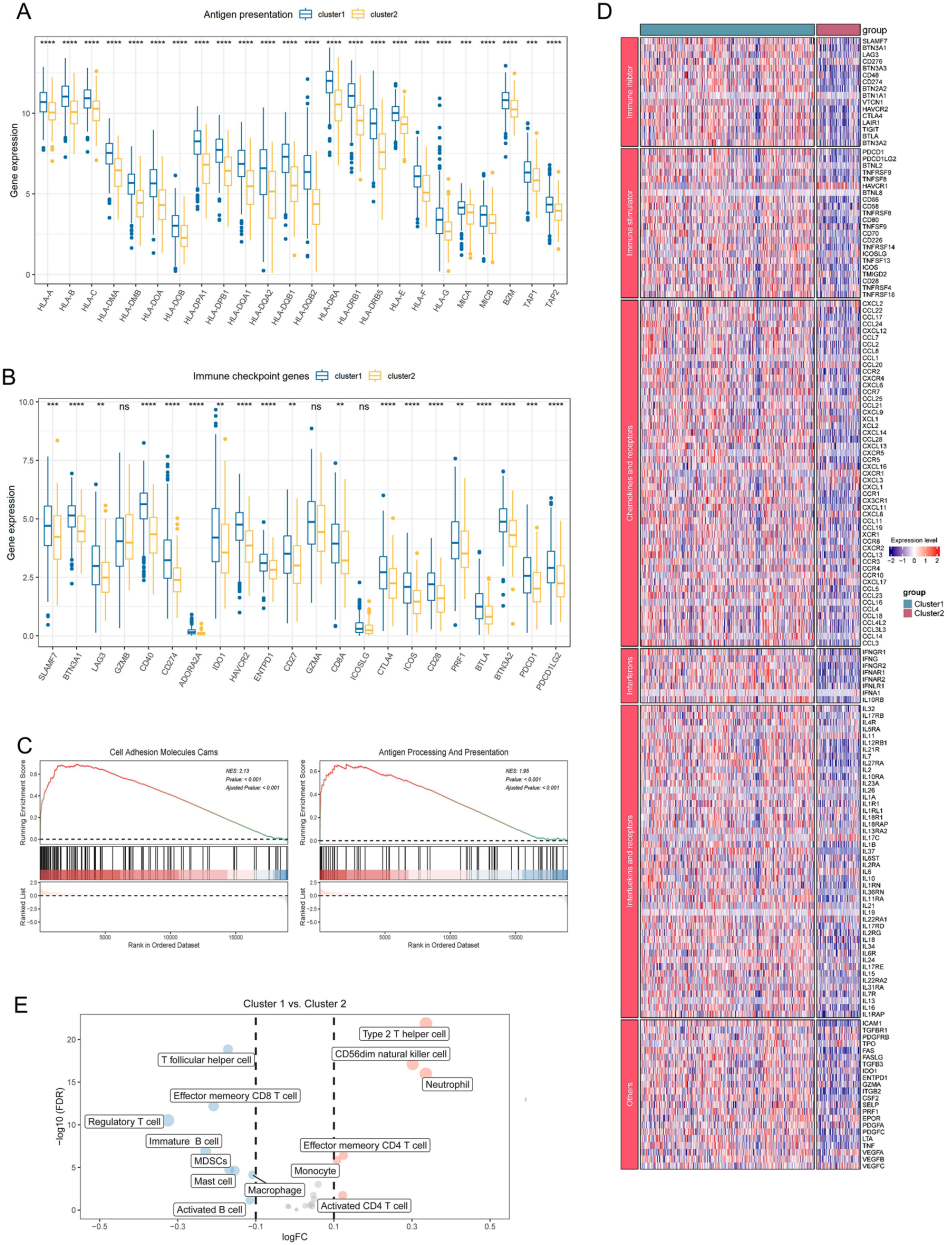
Immune escape characterization. Boxplots displayed the differences in the expression of antigen presentation (**A**) and immune checkpoint genes (**B**) in TCGA melanoma cohort. (**C**) GSEA plots showing the Cell Adhesion Molecules Cams and Antigen Processing And Presentation gene sets were enriched in cluster 1. (**D**) The expression levels of immune inhibitors, immune stimulators, chemokines, interleukins, other cytokines, and their receptors among two clusters. (**E**) The comparison of each cell type between clusters. *p < 0.05, **p < 0.01, ***p < 0.001, ****p < 0.0001, ^ns^p > 0.05.

Previous studies have shown that factors contributing to tumor immune escape include at least the absence of antigen presentation, tolerance and immune bias, immunosuppressive cell infiltration, immunosuppressive mediator secretion^44^. We explored possible mechanisms of immune escape for both subtypes. First, we explored the differences in the expression of antigen presentation genes and immune checkpoint markers in the two clusters (Fig. 4A,B), with significantly increased expression of antigen presentation and immune checkpoint-related genes in cluster 2 compared to cluster 1. Pathway enrichment analysis revealed that antigen presentation-related pathways were significantly enriched in tumor cells of cluster 1 (Fig. 4C). In addition, consistent results were obtained in the GSE68465 dataset (Supplementary Fig. S2D,E), where we also found that the T cell receptor pathway and the arginine and B cell receptor pathways were significantly enriched in cluster 1, which is consistent with the immune signature previously analyzed; DNA methylation and SIRT1 negatively regulated rRNA expression pathways were significantly enriched in cluster 2, which may be associated with the transcriptional repression exhibited by cluster 2 (Supplementary Fig. S4A–D). Then, targeting immune tolerance and immune bias, in the heat map we found that most immunosuppressive and immune activators were significantly reduced in cluster 2, which is consistent with the absence of antigen presentation and impaired T cell activation in cluster 2 in the tumor immune cycle and suggests that the pro- and anti-tumor immune activation of cluster 2 is simultaneously suppressed; meanwhile, hepatitis A virus-cell receptor (*HAVCR1*) as a multi viral *HAVCR1* is aberrantly expressed in tumors as a receptor for multiple viruses and is highly expressed in cluster 2, may serve as a feature of immune escape in lung adenocarcinoma^45^. In immunosuppressive mediator secretion, by comparing the expression of cytokines such as chemokines and receptors, interferons and receptors, and interleukins and receptors in the three subtypes most immunosuppressive mediators were lowly expressed in cluster 2, such as chemokines involved in T cell recruitment and inflammatory response: *CXCL9* and *CXCL10* were expressed at high levels in cluster 1, while the enrichment of *CCL4* and *CXCL13* may have promoted the cluster 1 establishment of the thermal tumor microenvironment^46^. The low expression of *IL-37* in cluster 2 suggests a reduced antitumor activity and potential for tumor metastasis^47^ (Fig. 4D). However, *IDO*, which contributes to peripheral tolerance, was highly expressed in subtype 1, suggesting the presence of inhibitory cytokines in the inflammatory microenvironment of cluster 1; the significant absence of *STING*, a key factor in the cGAS-STING pathway that contributes to the initiation of innate immunity and recognition of tumors, in cluster 2 is consistent with our results, indicating impaired immune initiation in cluster 2 (Supplementary Fig. S5C,D). In addition, we compared the infiltration of each cell type between subtypes. Cluster 1 had a higher infiltration of CD8T cells and B cells, and cluster 2 had a higher infiltration of CD4T cells. In the immunosuppressive cell infiltrate, cluster 1 had abundant Tregs, MDSCs, and macrophages (Fig. 4E).

### Immunotherapy response prediction

**Figure 5:**
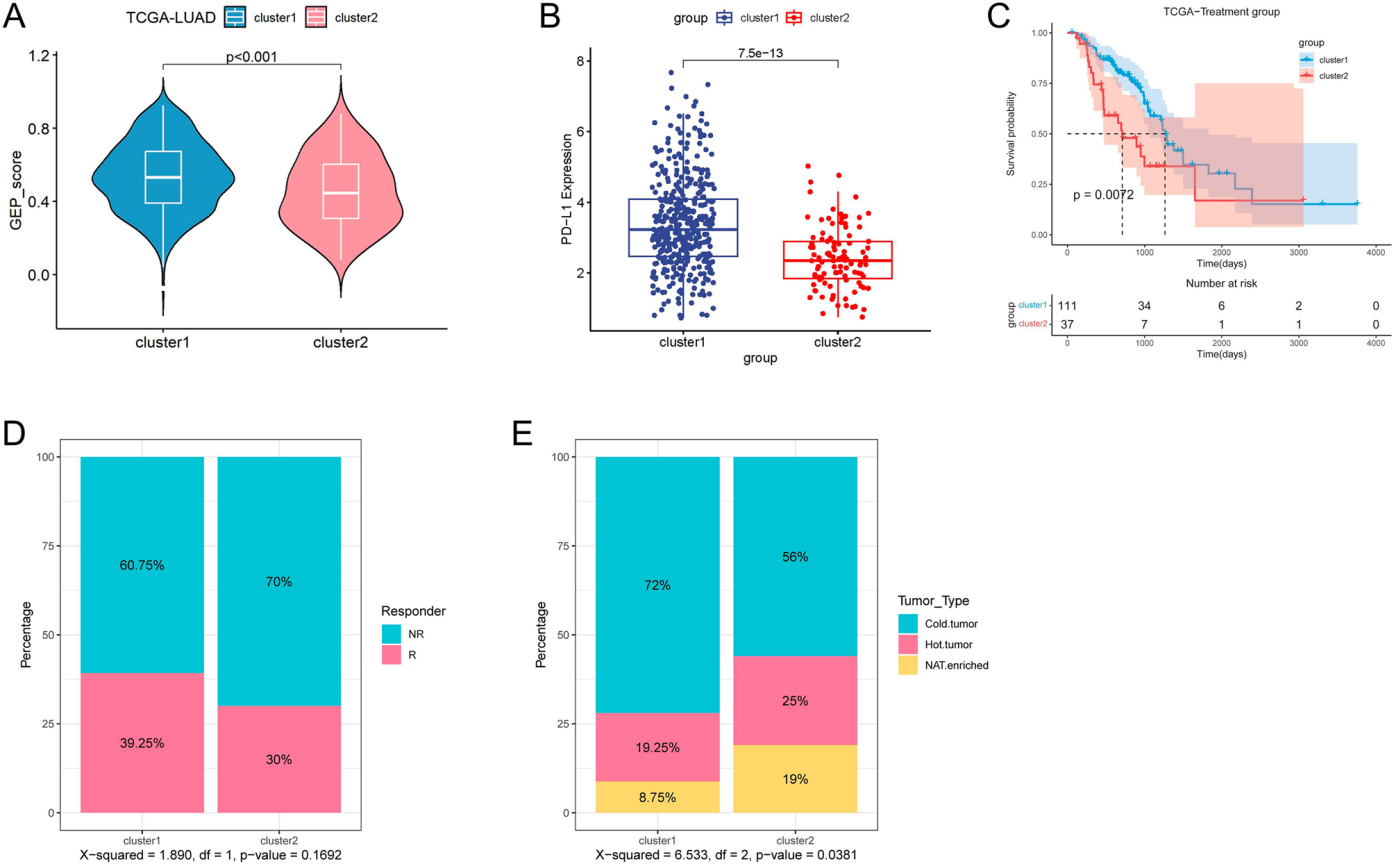
Prediction of immunotherapy response between two clusters. (**A,B**) The differences in GEP score and PD-L1 expression between two clusters. (**C**) KM curves associated with survival outcomes between two clusters in chemotherapy groups. (**D**) Proportion of patients responding to immunotherapy between the two clusters according to the TIDE algorithm. (**E**) Differences in hot and cold tumor phenotyping of lung adenocarcinoma patients according to the CIBERSORT algorithm.

We analyzed immune checkpoint PD-L1 (*CD274*) expression levels and GEP scores between the two clusters and found that cluster 1 had significantly higher PD-L1 expression and possessed a higher GEP score, which may predict that cluster 1 possesses a better immunotherapeutic effect^48,49^ (Fig. 5A,B). In addition, we combined clinical information from the treated and untreated groups in the TCGA cohort, and a subgroup analysis of the treated group revealed potentially better treatment outcomes for cluster 1 patients (Fig. 5C), while there was no significant difference in clinical prognosis in the subgroup analysis of the untreated group. Recent studies have shown that a higher tumor mutational load (TMB) is associated with a better immunotherapy response^50,51^, cluster 1 exhibited higher TMB levels than cluster 2 (Supplementary Fig. S5A,B). We then predicted their immunotherapeutic efficacy by the TIDE algorithm and found that cluster 2 scored at a higher level compared to cluster 1 based on comparing the two subtypes of TIDE scores, possibly predicting that cluster 2 has a higher immune escape capacity and poorer immunotherapeutic efficacy. Subsequently, we found a higher proportion of patients responding to immunotherapy in cluster 1 (Fig. 5D). Based on the spatial arrangement of immune cells, tumors can be classified into cold and hot tumors. Effective infiltration of large numbers of CD8+ T cells and better therapeutic potential were present in hot tumors or so-called inflammatory tumors, whereas immune cells in cold tumors were lacking or suppressed and thus insensitive to immunotherapy. Therefore, we quoted the hot and cold tumor ratio method based on the immune infiltration characteristics of LUAD, which showed that cluster 2 had a higher percentage of hot tumors and a lower percentage of cold tumors (Fig. 5E). This is not consistent with the immune and prognostic performance of cluster 2. Based on the possibility that cluster 2 has features associated with immune escape, partial activation of the Wnt/β-catenin pathway and suppression of immune-related signaling pathways, we hypothesize that cluster 2 is closer to an immune suppression or immune desert phenotype^52^.

### Drug sensitivity analysis

To explore drugs suitable for cluster 2 patients with poor prognosis, we used the oncopredict algorithm to assess the sensitivity of commonly used lung adenocarcinoma chemotherapy drugs to help treat LUAD patients. All scores for each sample in TCGA-LUAD cohort are shown in Supplementary Data S7. We show six drugs that are common treatments for lung adenocarcinoma, and the results showed that all six drugs, cisplatin (Chemotherapy drug), 5-fluoropyrimidine (Chemotherapy drug), paclitaxel (Chemotherapy drug), erlotinib (targeting drug, EGFR inhibitor), gefitinib (targeting drug, EGFR-TK inhibitor), and vincristine (Chemotherapy drug), may have better efficacy in patients with LUAD in cluster 2 (Supplementary Fig. S6A–F).

### Establishing Arg and Pro metabolism-related profiles

**Figure 6:**
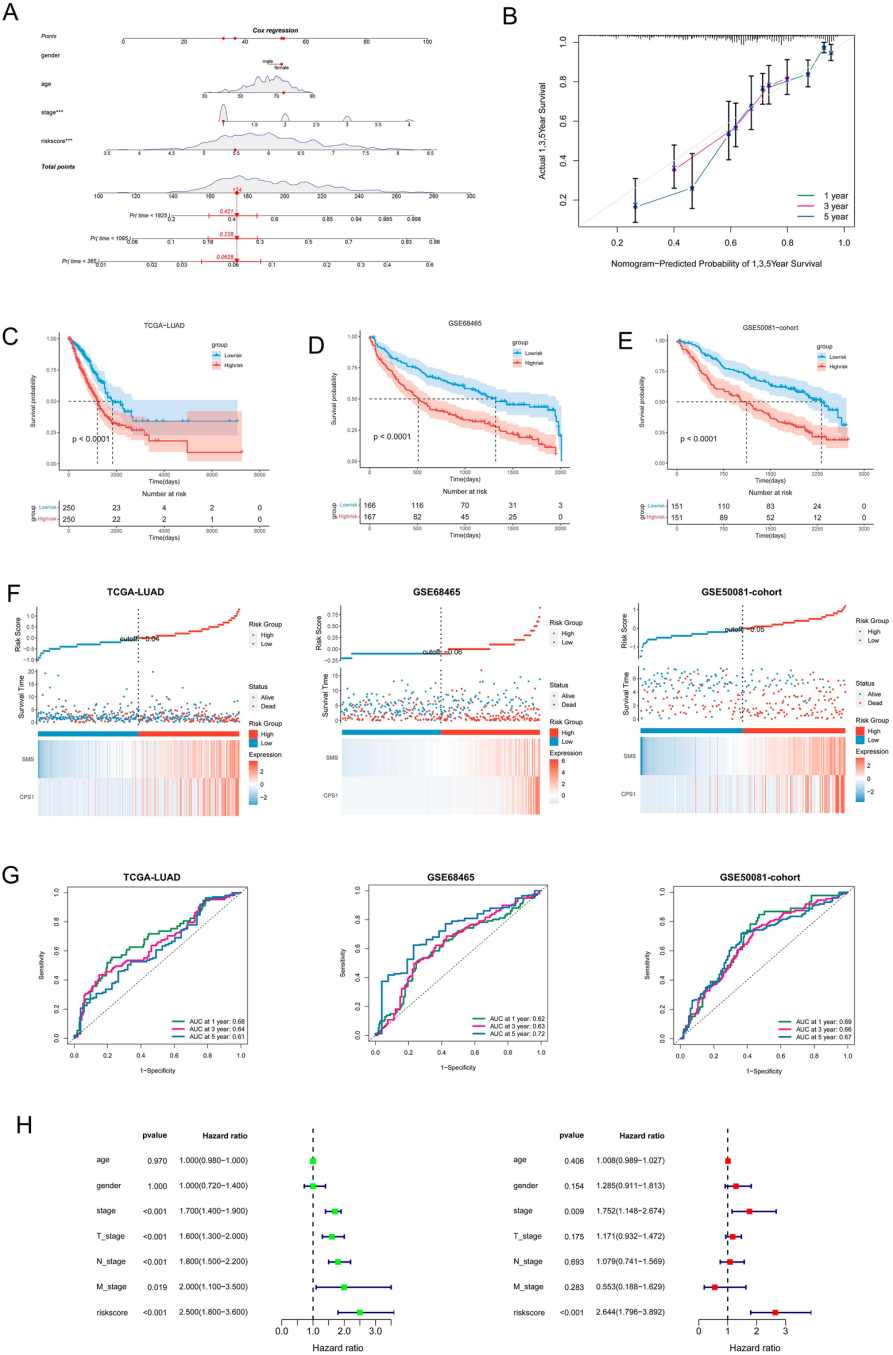
APRG-related prognostic model construction. (**A**) The nomogram based on the signature in LUAD patients at 1, 3, and 5 years. (**B**) Calibration curves of nomogram for the signature at 1, 3, and 5 years. (**C–E**) Survival curves for the high-risk and low-risk groups in the TCGA and GEO cohorts. (**F**) The column diagram shows the risk score distribution of LUAD patients; survival status and duration of patients; heatmap of the CPS1 and SMS genes expression. (**G**) Time-independent receiver operating characteristic (ROC) analysis of risk scores for prediction the OS in the TCGA and GEO cohorts. (**H**) Univariate and multivariate independent prognostic analysis of independent risk factors for OS in patients with LUAD.

First, we identified two central genes, *CPS1* and *SMS*, that affect the prognosis of lung adenocarcinoma by stepwise multivariate Cox analysis and constructed a prognostic model associated with Arg and Pro metabolism. The formula constructed to assess the prognosis of each patient was as follows: Risk score = (0.08383 × *CPS1* expression) + (0.48094 × *SMS* expression). The risk score of each patient was calculated based on this model, and LUAD patients were divided into high-risk and low-risk groups according to the median risk score (Table 2). A column line plot for predicting patient survival was then constructed (Fig. 6A), and good agreement between predicted and actual survival was observed in the corrected curves of the prognostic column line plot (Fig. 6B). In the TCGA and GEO cohorts, OS was significantly lower in high-risk patients than in the low-risk group (Fig. 6C–E); risk scores, survival status, and gene expression heat maps for the two Arg and Pro genes are shown (Fig. 6F). The area under the characteristic curve for simultaneously predicting 1-year, 3-year, and 5-year OS rates was 0.68, 0.64, and 0.61, respectively. Validated in both the GSE68465 and GSE50081 cohorts, the area under the characteristic curve values for predicting 1-year, 3-year, and 5-year OS rates in GSE68465 were 0.72, 0.63, and 0.62, respectively, indicating that this prognostic model has good sensitivity and specificity, consistent with the results of the TCGA cohort (Fig. 6G). In addition, univariate and multivariate Cox were performed to assess the independent predictive power of risk score and clinical characteristics, and the results showed that risk score (p < 0.001) and pathological stage (p < 0.009) could be used as independent predictors (Fig. 6H).

**Table 2:** Multivariate COX regression analysis results of model genes.

Id	Coef	HR	HR.95 L	HR.95 H	*p* value
CPS1	0.08383	1.08744	1.040	1.137	0.000227
SMS	0.48094	1.61759	1.328	1.970	1.75e−06

### CPS1 and SMS gene analysis

**Figure 7:**
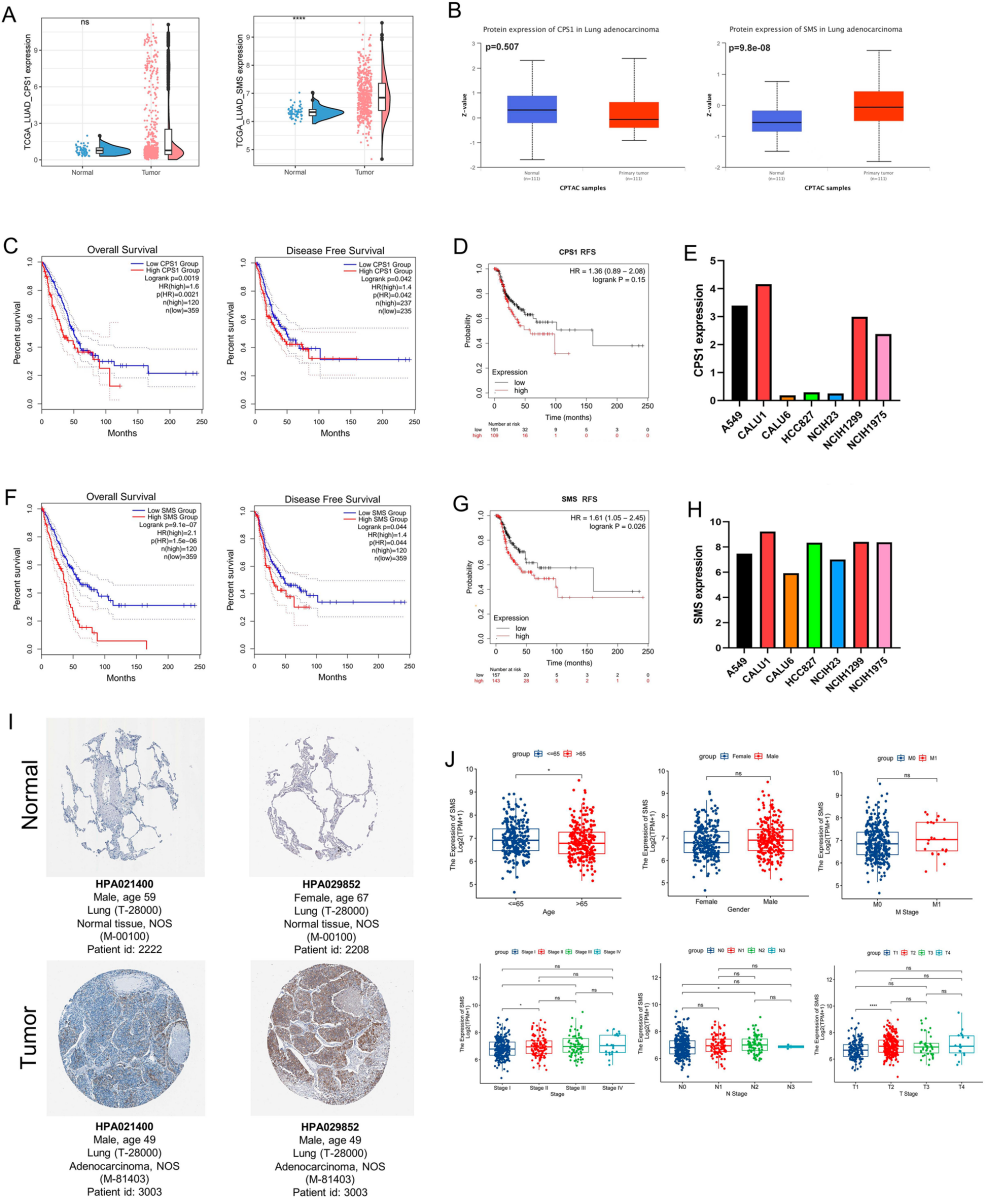
Analysis of CPS1 and SMS in terms of expression, prognosis, and clinical staging. (**A**) Transcript expression levels of CPS1 and SMS in the TCGA cohort. (**B**) Protein expression levels of CPS1 and SMS in the CPTAC Database. (**C,F**) Survival curves of OS and PFS of CPS1 and SMS in the GEPIA2 database. (**D,G**) Survival curves for OS and PFS of CPS1 and SMS from Kaplan Meier database. (**E,H**) TPM expression levels of CPS1 and SMS in different lung adenocarcinoma cell lines from CCLE database. (**I**) Verification of hub APRGs expression in LUAD and normal lung tissue using the HPA database. (**J**) Relationship between SMS gene expression and clinical features. *p < 0.05, **p < 0.01, ***p < 0.001, ****p < 0.0001, ^ns^p > 0.05.

As a hub gene in the model of Arg and Pro metabolism and prognosis, carbamoyl phosphate synthase 1 (*CPS1*) has been shown to be associated with poor prognosis in lung adenocarcinoma, and spermine synthase (*SMS*) has been reported to be associated with poor prognosis in hepatocellular carcinoma, head and neck squamous cell carcinoma, and colorectal cancer. First, we compared the difference in mRNA expression between normal and tumor groups in lung adenocarcinoma with *CPS1* and *SMS* genes in the TCGA and GEPIA2 databases, and also validated them in the GEO database (Supplementary Fig. S7A,B), and analyzed the protein expression level of data from CPTAC in the UALCAN database. The results showed that both mRNA and protein of *SMS* were highly expressed in tumor tissues, while *CPS1* was not significantly different in mRNA and protein levels (Fig. 7A,B). Subsequently, the relationship between *CPS1* and *SMS* gene expression and prognosis of LUAD was analyzed for overall survival (OS), disease-free survival (DFS), and recurrence-free survival (RFS), respectively. The group with high expression of *CPS1* in OS and DFS had lower survival rates, while RFS showed no significant difference; *SMS* showed significant differences in OS, DFS, and RFS, indicating that high expression of *SMS* in LUAD resulted in poorer prognosis (Fig. 7C,D,F,G). In addition, we performed univariate and multivariate Cox analyses of clinical characteristics of LUAD and gene expression of *CPS1* and *SMS*, and the results showed that both *CPS1* and *SMS* could be independent predictors of LUAD (Supplementary Fig. S8A,B), and *SMS* may play a greater role in influencing the prognosis of LUAD than *CPS1*. In addition, we obtained mRNA expression data of *CPS1* and *SMS* in seven cell lines for LUAD in the CCLE database and found that both *CPS1* and *SMS* expression levels were higher in A549, CALU1, and H1299 (Fig. 7E,H). Protein expression levels were analyzed using the HPA database, and the protein expression levels of CPS1 and SMS were higher in LUAD tissues than in normal tissues, and *SMS* exhibited higher protein expression levels (Fig. 7I). By analyzing the relationship between clinical characteristics and the expression of *CPS1* and *SMS*, we found that the expression of *CPS1* was higher in men, but no significant differences were found in pathological and TNM stages (Supplementary Fig. S8C); meanwhile, the expression of *SMS* differed among different pathological stages and T and N stages, and the expression of *SMS* increased with increasing stages, which might represent the correlation between *SMS* and tumor metastasis (Fig. 7J). The differences in *SMS* expression, prognosis, and clinical staging may represent a close relationship with lung adenocarcinoma progression.

### Role of SMS as a central gene in lung adenocarcinoma progression

**Figure 8:**
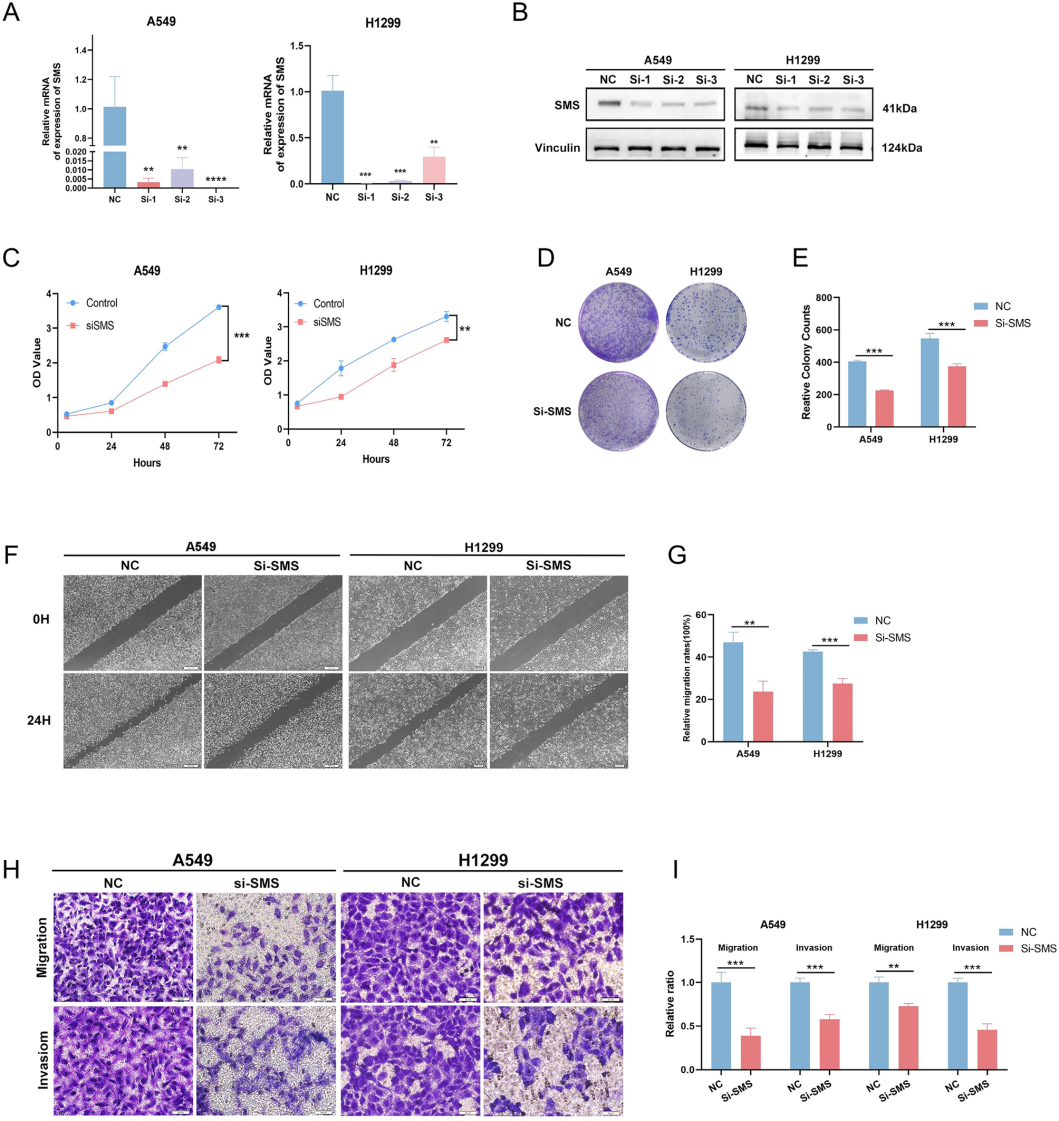
Knockdown of SMS inhibits proliferation, migration, and invasion of lung adenocarcinoma cells. (**A**) The transfection efficiency of si-SMS in the A549 and H1299 cell lines detected by RT-PCR. (**B**) Western blot analysis confirmed that the expression of SMS was inhibited by SMS siRNA administration. (**C**) The CCK-8 assay was used to detect the effect of si-SMS on the proliferation of A549 and H1299 cell lines. (**D**) Representative images of the colony formation assay. (**E**) Statistical analysis of the colony formation assay results after Knockdown of SMS. (**F**) Representative images of the wound healing assay. (**G**) Statistical analysis of the wound healing assay results after Knockdown of SMS. (**H**) Representative images of the transwell assay. (**I**) Statistical analysis of the transwell assay results after Knockdown of SMS in the A549 and H1299 cell lines. *p < 0.05, **p < 0.01, ***p < 0.001, and ****p < 0.0001.

To further investigate the role of Arg and Pro-related genes in lung adenocarcinoma and the biological processes involved in *SMS* as a central gene affecting prognosis in lung adenocarcinoma. We performed cellular experiments to knock down *SMS*, and two cell lines, A549 and H1299, were selected. The transfection efficiency of the cells was first assessed by RT-qPCR and Western blot, and the relative expression level of *SMS* was found to be significantly reduced after siRNA 3 transfection (Fig. 8A,B). To further validate the role of *SMS* in proliferation, we performed the CCK-8 assay to detect the effect of *SMS* knockdown. After *SMS* silencing, the proliferation of A549 and H1299 cells was significantly reduced compared to control cells (Fig. 8C), and the inhibition of proliferation was even more pronounced in A549 cells. Colony formation assay also showed that *SMS* silencing significantly inhibited the growth of A549 and H1299 (Fig. 8D,E), and wound healing assays were performed to detect migration, and the results showed that the migration rate of A549 and H1299 cells transfected with siRNA was significantly lower than that of control transfected cells (Fig. 8F,G). In addition, the results of transwell assay showed that knockdown of *SMS* significantly inhibited the migration and proliferation ability of A549 and H1299 (Fig. 8H,I). These results indicate that knockdown of *SMS* inhibited the proliferation, migration, and invasion ability of A549 and H1299 cells.

## Discussion

In recent years, Lung adenocarcinoma has improved its 5-year survival rate from 5 to 15% as a result of a combination of new chemotherapeutic agents, targeted agents, and immunosuppressants but it is still the leading cause of life-threatening health problems in cancer. Alterations in cellular metabolism are considered to be a hallmark of cancer and are one of the key mechanisms of tumorigenesis, tumor progression, and chemotherapeutic resistance^53^. In recent years, the complex relationship between amino acid metabolism and cancer is attracting attention^54^. In addition, amino acid depletion that also targets amino acid metabolism has great therapeutic potential^55,56^. Arginine has been shown to have great potential as a semi-essential amino acid. In addition to its role in the TCA cycle and polyamine synthesis, arginine, a semi-essential amino acid that is synthesized from glutamine or proline, is also a precursor for compounds such as creatine, polyamines, and NO^57^. Meanwhile, in TME, l-arginine is predominantly deficient or depleted, and immunosuppressive cells such as M2 macrophages, regulatory T cells, and MDSCs can compete with CD8+ T cells, which exert anti-tumor immunity, for l-arginine, while recent studies have found that supplementation with l-arginine along with knockdown of CAT-2 transporter promoted infiltration and activation of CD8+ T cells and suppressed tumor growth^58^. Therefore, arginine and proline metabolism may be potentially relevant to the tumor microenvironment and immunotherapy. Moreover, the overall effect and relationship of arginine and proline metabolism genes with immune infiltration in LUAD has not been reported.

In this study, we reveal the landscape of arginine and proline metabolism-related genes and TME in LUAD. Screening of APRGs by univariate Cox regression analysis in the TCGA dataset yielded 12 APRGs associated with OS, revealing two different molecular isoforms based on the expression of the 12 APRGs. Cluster 1 patients had a longer survival time compared to cluster 2 patients. GSVA enrichment analysis showed that cluster 2 was significantly enriched in a variety of substance metabolisms including arginine and proline metabolism, riboflavin metabolism, and histidine metabolism. The enriched pathways in cluster 2 suggest that they play an important role in the development of LUAD and that there is a crosstalk between arginine and proline metabolism and other signaling pathways. We also identified TME features between the two clusters, with cluster 1 showing high immune cell infiltration and high stromal cell infiltration, which is close to the immune inflammatory or immune rejection type, and cluster 2 with impaired T cell activation and unique immune escape features, along with high methylation levels and reduced expression of most antitumor immune molecules, which is more closely related to the immune rejection or immune desert type. In addition, we assessed the immunotherapeutic response and drug sensitivity of both subtypes by algorithms such as TIDE, which provides a reference for clinical treatment. In addition, we developed a validated risk model for prognostic APRGs, which included 2 APRGs (*CPS1* and *SMS*), and validated its predictive power. Finally, we performed cellular experiments to validate the role of *SMS* as a hub prognostic gene for APRG in LUAD progression, further validating the feasibility of the prognostic model.

Among the two signature genes in the prognostic model of APRGs, both *CPS1* and *SMS* are high-risk genes in the LUAD cohort. *CPS1* is a key enzyme involved in the urea cycle, and our results are consistent with previous studies in terms of expression, prognostic and other characteristics. The potential mechanisms of *SMS*, a key enzyme regulating spermine synthesis, to regulate the progression of LUAD remain to be explored. Previous studies have shown that *c-MYC* directly regulates ornithine decarboxylase 1 (*ODC1*), spermidine synthase (*SRM*), and glutaminase (*GLS*), the upstream genes of polyamine metabolism^59,60^, its synergistic relationship with polyamine metabolism has been demonstrated in bladder cancer, neuroblastoma, and colorectal cancer^61–63^. In addition, the study by Yubin Guo et al. revealed a complex mechanism of *MYC* and *SMS* synergistically regulating CRC apoptosis.

Recently, an increasing number of studies based on metabolism-related genes^64^ and hypoxia-derived genes^65^, radiotherapy autophagy-related genes^66^, m6A-associated lncRNA^67^, etc., all of which have shown some degree of predictive power for prognosis, immune response, etc. in LUAD patients. In a recent study, circulating l-arginine was suggested to predict the survival of patients treated with ICIs^68^. The specific mechanism of the potential activation of rapamycin complex 1 (mTORC1) by altered arginine concentration remains to be investigated^69^.

In addition, evidence supporting tumor immune escape can be found in the following literature and related studies: in the study by Jhunjhunwala et al. we found the most critical antigen presentation and HLA deletion features consistent in this study^70^; Pansy et al. reviewed the regulatory mechanisms of immune checkpoints, co-suppressor molecules, and cytokines in the process of immune escape from tumors^71^; Liu et al. also described the mechanisms by which immunosuppressive cells promote immune escape from tumors^72^; and Nagarsheth et al. elaborated on the roles of chemokines in cancer immunity and tumorigenesis^73^. Therefore, exploring the immune escape profile of lung adenocarcinoma patients may ultimately benefit clinical immunotherapy.

In summary, this study characterized the arginine metabolism-related gene markers in LUAD and developed a prognostic model of APRGs based on two signature genes, which demonstrated strong ability to predict prognosis and evaluate immune evasion in LUAD. There remain, however, some limitations to our study. Firstly, all samples used in the survey were collected retrospectively and analyzed against publicly available database data. Thus, inherent case selection bias may influence the results and more compelling prospective studies are needed to confirm our findings. Secondly, because of the limited sample size, large cohort studies are essential to evaluate the value of the model. Thirdly, to improve the future understanding of arginine and proline metabolism, it is also crucial to validate the molecular understanding based on functional in vivo and in vitro experiments. In conclusion, some crucial clinical information including surgery, targeted therapy, and chemoradiotherapy is not available for analysis in most of the data sets, which is thought to affect the prognosis of the immune response as well as the status of arginine and proline metabolism. Future studies will continue to address the specific mechanisms by which *SMS* affects LUAD progression from both in vivo and in vivo experiments.

### Supplementary Information

Supplementary Information.

## Data Availability

Publicly available datasets were analyzed in this study; these can be found in the GEO database (https://www.ncbi.nlm.nih.gov/geo), and The Cancer Genome Atlas (https://portal.GDC.cancer.gov). The authors confirm that the data supporting the findings of this study are available within the article and its Supplementary Materials. The original R code has been uploaded to the authors’ GitHub homepage (https://github.com/wanglaoji2345/APRGs), and the corresponding data in the article are available from the corresponding author. A statement that all methods and public data are implemented by relevant guidelines and regulations.

## Abbreviations

TCGA: The Cancer Genome Atlas
GEO: Gene Expression Omnibus
TNM: Tumor size/lymph nodes/distance metastasis
ATP: Adenosine triphosphate
ARG1: Arginase 1
iNOS: Inducible nitric oxide synthase
TGF-β: Transforming growth factor-beta
ROS: Reactive oxygen species
ARG: Arginine
MHC: Major histocompatibility complex
GEPIA2: Gene expression profiling interactive analysis 2
MAOB: Monoamine oxidase-B
P4HA2: Prolyl 4-hydroxylases
ALDH2: Aldehyde dehydrogenase 2
SRM: Spermidine synthase
AGMAT: Agmatinase
P4HA1: Prolyl 4-hydroxylase subunit alpha 1
GOT2: Glutamic-oxaloacetic transaminase 2
CKMT2: Creatine kinase, mitochondrial 2
GLS2: Glutaminase 2
AZIN2: Antizyme inhibitor 2
IDO: Indoleamine 2,3-dioxygenase
STING: Stimulator of interferon genes
NO: Nitric oxide
