# Supplementary material for: Predicting lung adenocarcinoma prognosis, immune escape, and pharmacomic profile from arginine and proline-related genes
Source: Sci Rep. 2023 Sep 14;13:15198. doi: 10.1038/s41598-023-42541-z (PMC10502151; doi:10.1038/s41598-023-42541-z)
Supplement: Supplementary file 1 — Supplementary Information. [file 41598_2023_42541_MOESM1_ESM.pdf]

Supplementary Figure

Figure S1

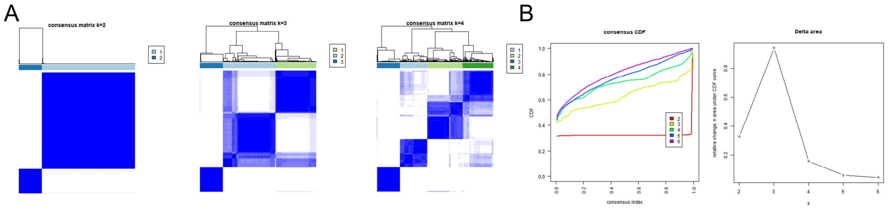

Figure S2

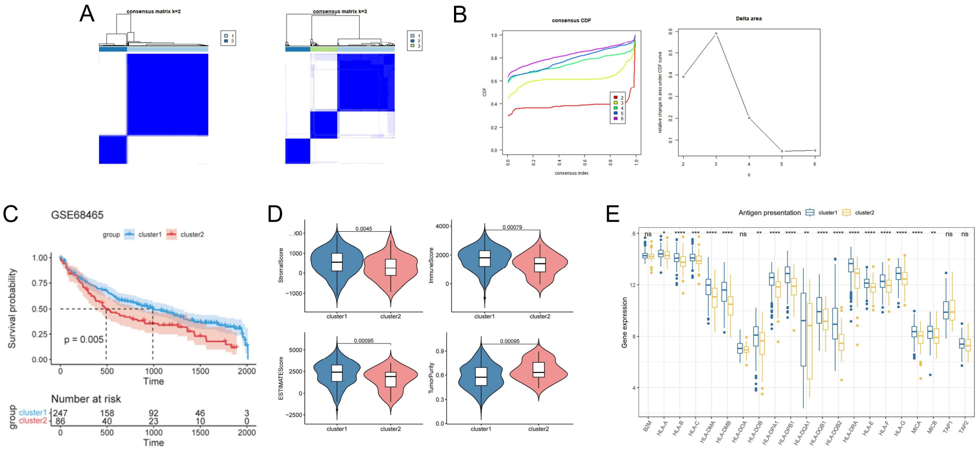

Figure S3

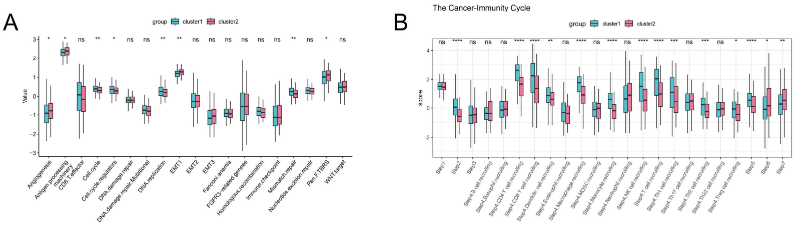

Figure S4

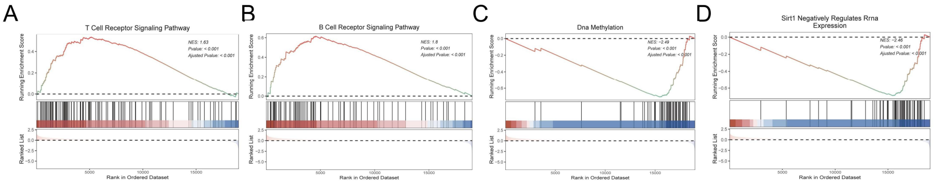

Supplementary figure S1

(A) Consensus heatmap matrix of TCGA cohort. (B) Consensus matrix CDF and Delta area curve.

Supplementary figure S2

(A) Consensus heatmap matrix of GSE68465 cohort. (B) Consensus matrix CDF and Delta area curve. (C) Kaplan-Meier survival analysis of the two clusters. (D) ESTIMATEScores, ImuneScores, StromalScores, and TumorPurity of the two clusters in GSE68485. (E) Difference expression of antigen presentation genes.

Supplementary figure S3

(A) Differences of specific pathways curated from Mariathasan et al. constructed gene set among two clusters. (B)Boxplot demonstrating the difference in scores between the two clusters of the Cancer-Immunity Cycle.

Supplementary figure S4

(A-D) GSEA pathway enrichment analysis.

Figure S5

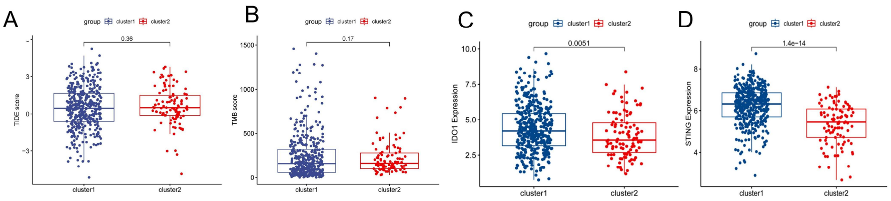

Figure S6

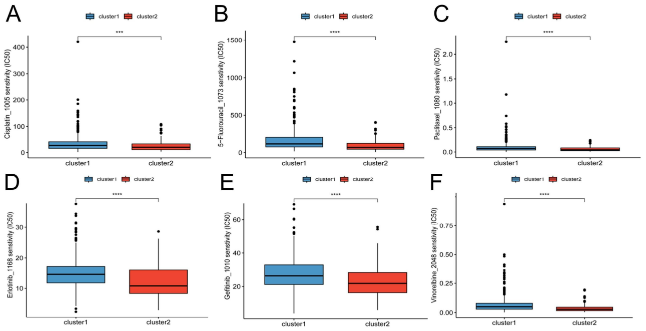

Figure S7

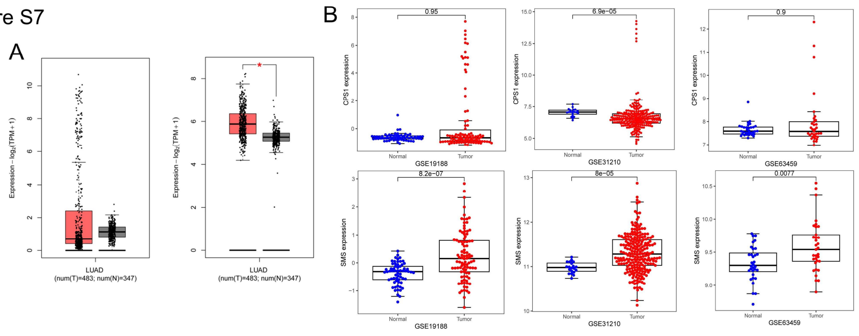

Supplementary figure S5

(A-B) Difference of TIDE and TMB score in two clusters. (C-D) The expression of IDO and STING among two clusters.

Supplementary figure S6

(A-F) Predicted sensitivity (IC50) of cisplatin, 5-fluoropyrimidine, paclitaxel, erlotinib, gefitinib, and vincristine, which were candidate potent drug options for cluster 2

patients.

Supplementary figure S7

(A) CPS1 and SMS gene expression in the GEPIA2 database. (B) In the GEO cohort, CPS1 and SMS gene expression levels in normal tissues and tumors.

Figure S8

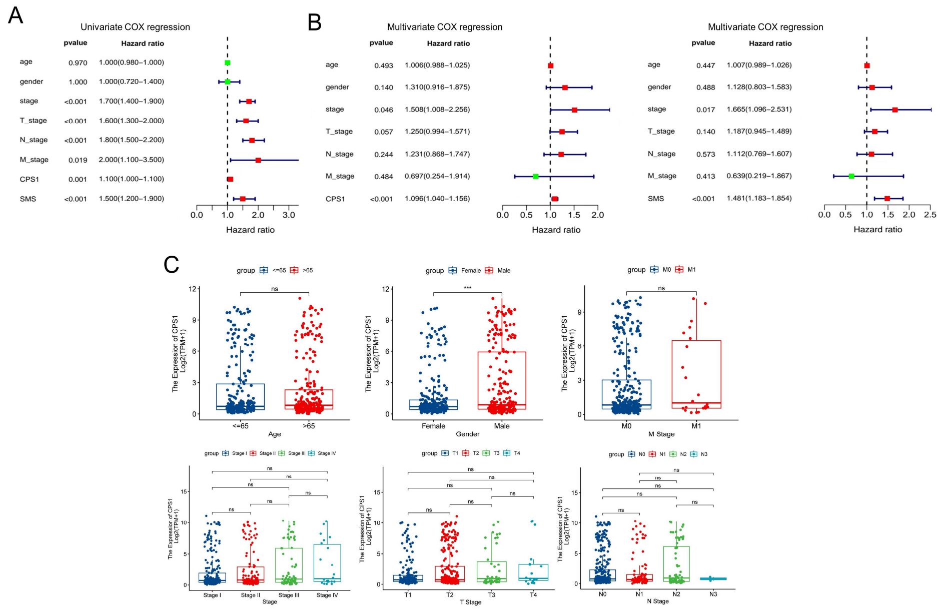

Supplementary figure S8

(A-B) Univariate and multivariate independent prognostic analysis of CPS1 and SMS genes. (C) Relationship between CPS1 gene expression and clinical features.

Supplementary original images

A549-1-2

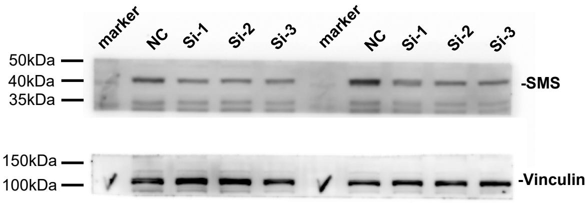

A549-3-4

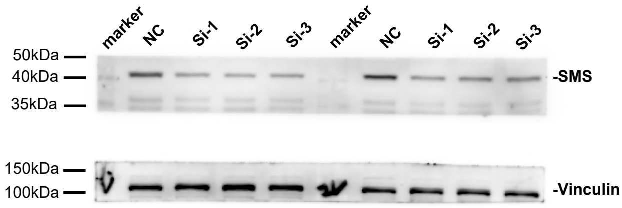

H1299-1

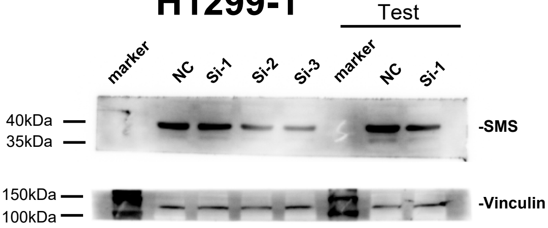

H1299-2

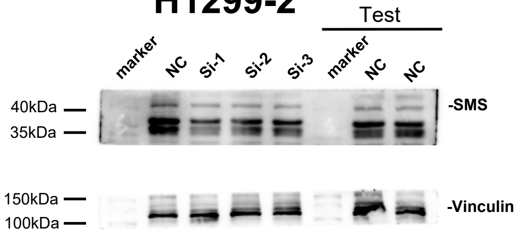

H1299-3

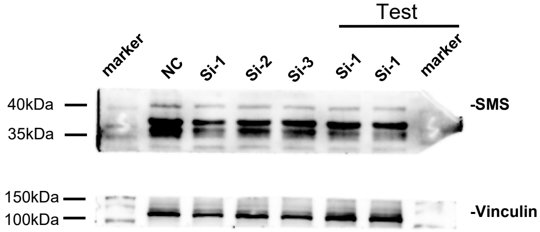

Supplementary Data

Supplementary Table S 1
Clinical and Subgroup Information of LUAD samples in the TCGA database.

Supplementary Table S 2
DEGs between subgroups in TCGA database.

Supplementary Table S 3
GSEA enrichment results for the KEGG gene set in the TCGA database.

Supplementary Table S 4
GSEA enrichment results for the Reactome gene set in the TCGA database.

Supplementary Table S 5
Clinical and Subgroup Information of GSE68465 in GEO database.

Supplementary Table S 6
Clinical and grouping information for the GSE50081 integration cohort in the GEO databa

Supplementary Table S 7
Drug score of 198 drugs in each sample.

Supplementary Table S 8
Fifty-four APRGs

Supplementary Table S 1

ID	time	status	age	gender	stage	T stage	N stage	M stage	riskscore	group	riskgroup
TCGA-05-4249-01A	1523	0	67	male	I	T2	N0	M0	3.283550635	cluster1	lowrisk
TCGA-05-4250-01A	121	1	79	female	III	T3	N1	M0	4.108383831	cluster1	highrisk
TCGA-05-4382-01A	607	0	68	male	I	T2	N0	M0	3.34385665	cluster1	lowrisk
TCGA-05-4384-01A	426	0	66	male	III	T2	N2	M0	3.253435124	cluster1	lowrisk
TCGA-05-4389-01A	1369	0	70	male	I	T1	N0	M0	3.449526251	cluster1	lowrisk
TCGA-05-4390-01A	1126	0	58	female	I	T2	N0	M0	3.759924172	cluster2	highrisk
TCGA-05-4396-01A	303	1	76	male	III	T4	N1	M0	4.17495436	cluster2	highrisk
TCGA-05-4397-01A	731	1	65	male	II	T2	N1	M0	4.226153342	cluster1	highrisk
TCGA-05-4398-01A	1431	0	47	female	III	T4	N3	M0	3.417744829	cluster1	lowrisk
TCGA-05-4402-01A	244	1	57	female	IV	T2	NX	M1	3.214745551	cluster1	lowrisk
TCGA-05-4403-01A	578	0	76	male	I	T2	N0	M0	3.390666713	cluster1	lowrisk
TCGA-05-4405-01A	610	0	74	female	I	T2	N0	M0	3.187989928	cluster1	lowrisk
TCGA-05-4415-01A	91	1	57	male	III	T4	N2	M0	4.49009021	cluster2	highrisk
TCGA-05-4417-01A	455	0	51	female	I	T2	N0	M0	3.765185152	cluster2	highrisk
TCGA-05-4418-01A	274	1	69	male	III	T3	N2	M0	3.986835528	cluster2	highrisk
TCGA-05-4420-01A	912	0	41	male	I	T2	N0	M0	3.793601456	cluster1	highrisk
TCGA-05-4422-01A	365	0	68	male	I	T2	N0	M0	3.95561258	cluster2	highrisk
TCGA-05-4424-01A	913	0	70	male	II	T3	N0	M0	4.010131414	cluster1	highrisk
TCGA-05-4425-01A	669	0	70	female	IV	T2	N0	M1	3.4535278	cluster1	lowrisk
TCGA-05-4426-01A	791	0	71	male	I	T2	N0	M0	3.15312998	cluster1	lowrisk
TCGA-05-4427-01A	791	0	65	female	II	T2	N1	M0	3.807614415	cluster1	highrisk
TCGA-05-4430-01A	761	0	59	female	I	T2	N0	M0	3.649633982	cluster1	highrisk
TCGA-05-4432-01A	761	0	66	male	II	T2	N1	M0	3.712007574	cluster1	highrisk
TCGA-05-4433-01A	730	0	82	male	I	T2	N0	M0	3.566222335	cluster1	highrisk
TCGA-05-4434-01A	457	1	67	female	IV	T4	N1	M1	3.852337357	cluster1	highrisk
TCGA-05-5420-01A	457	0	67	male	III	T2	N2	M0	3.455231233	cluster2	lowrisk
TCGA-05-5423-01A	151	0	65	male	II	T2	N1	M0	3.497210466	cluster1	highrisk
TCGA-05-5425-01A	882	0	68	male	II	T2	N1	M0	4.255919034	cluster2	highrisk
TCGA-05-5428-01A	670	0	57	male	II	T1	N1	M0	3.16246212	cluster1	lowrisk
TCGA-05-5429-01A	275	1	60	male	III	T3	N2	M0	3.483292485	cluster1	highrisk
TCGA-05-5715-01A	62	0	69	female	I	T2	N0	M0	3.411283277	cluster1	lowrisk
TCGA-35-3615-01A	14	0	57	male	I	T2	N0	M0	3.235085564	cluster1	lowrisk
TCGA-35-4122-01A	225	0	69	male	I	T1	N0	M0	3.37617297	cluster1	lowrisk
TCGA-35-4123-01A	182	0	38	male	I	T1	N0	M0	3.410896348	cluster1	lowrisk
TCGA-35-5375-01A	264	0	61	male	III	T2	N2	M0	3.311903281	cluster1	lowrisk
TCGA-38-4625-01A	2973	0	66	female	I	T2	N0	M0	4.439471003	cluster1	highrisk

TCGA-38-4626-01A	3674	0	57	female	unknown	T2	N0	M0	3.380205902	cluster1	lowrisk
TCGA-38-4627-01A	1147	1	64	female	II	T1	N1	M0	3.530972469	cluster1	highrisk
TCGA-38-4628-01A	1492	1	65	female	II	T2	N1	M0	3.595959242	cluster1	highrisk
TCGA-38-4629-01A	864	1	68	male	II	T3	N0	M0	4.246117203	cluster1	highrisk
TCGA-38-4630-01A	1073	1	75	female	I	T2	N0	M0	3.806039576	cluster1	highrisk
TCGA-38-4631-01A	354	1	72	female	I	T2	N0	M0	3.766460899	cluster1	highrisk
TCGA-38-4632-01A	1357	1	42	male	IV	T2	N1	M1	3.35114163	cluster1	lowrisk
TCGA-38-6178-01A	448	0	70	female	III	T2	N2	unknown	3.382696005	cluster1	lowrisk
TCGA-38-7271-01A	800	1	72	female	I	T1	N0	M0	2.939170669	cluster1	lowrisk
TCGA-38-A44F-01A	133	0	80	male	I	T2	N0	M0	3.230896498	cluster1	lowrisk
TCGA-44-2655-01A	1324	0	65	female	I	T1	N0	M0	3.234716421	cluster1	lowrisk
TCGA-44-2656-01A	1429	0	59	male	I	T2	N0	M0	3.759160468	cluster1	highrisk
TCGA-44-2657-01A	1351	0	74	female	I	T2	NX	M0	2.956977829	cluster1	lowrisk
TCGA-44-2659-01A	1367	0	65	female	II	T1	N1	M0	2.572163998	cluster1	lowrisk
TCGA-44-2661-01A	1159	0	69	female	I	T1	N0	M0	3.11487549	cluster1	lowrisk
TCGA-44-2662-01A	1280	0	65	male	I	T2	N0	M0	4.21572899	cluster1	highrisk
TCGA-44-2665-01A	1301	0	55	female	II	T2	N1	M0	3.016949812	cluster1	lowrisk
TCGA-44-2666-01A	97	1	43	male	I	T2	N0	M0	3.293100969	cluster1	lowrisk
TCGA-44-2668-01A	761	1	51	male	I	T2	N0	M0	3.659463868	cluster1	highrisk
TCGA-44-3396-01A	1130	0	74	female	III	T2	N2	M0	3.194040734	cluster1	lowrisk
TCGA-44-3398-01A	1163	0	77	female	I	T1	N0	M0	3.292016929	cluster1	lowrisk
TCGA-44-3917-01A	1183	0	33	female	I	T2	N0	M0	2.525442155	cluster2	lowrisk
TCGA-44-3918-01A	1036	0	60	female	I	T1	N0	M0	2.98450997	cluster1	lowrisk
TCGA-44-3919-01A	1026	1	71	female	I	T1	N0	M0	3.285668646	cluster1	lowrisk
TCGA-44-4112-01A	808	1	60	female	I	T2	N0	M0	3.726040212	cluster1	highrisk
TCGA-44-5643-01A	1013	0	53	male	III	T2	N2	M0	3.797690763	cluster1	highrisk
TCGA-44-5644-01A	863	0	51	female	I	T2	N0	unknown	2.981300374	cluster2	lowrisk
TCGA-44-5645-01A	852	0	61	female	I	T1	NX	unknown	3.61491693	cluster1	highrisk
TCGA-44-6145-01A	723	0	58	male	I	T1	N0	M0	3.668040133	cluster1	highrisk
TCGA-44-6146-01A	595	0	62	female	I	T1	N0	M0	3.243212027	cluster1	lowrisk
TCGA-44-6147-01A	728	0	64	male	II	T3	N0	M0	3.083706977	cluster1	lowrisk
TCGA-44-6148-01A	845	0	67	female	I	T1	NX	M0	3.173261337	cluster1	lowrisk
TCGA-44-6774-01A	704	0	60	male	I	T1	N0	M0	3.215960565	cluster1	lowrisk
TCGA-44-6775-01A	658	0	56	female	III	T1	N2	M0	3.624359824	cluster1	highrisk
TCGA-44-6776-01A	2616	0	60	female	I	T1	N0	MX	3.236297667	cluster1	lowrisk
TCGA-44-6777-01A	987	1	85	female	I	T2	NX	MX	3.216866242	cluster1	lowrisk
TCGA-44-6778-01A	1864	0	59	male	I	T1	N0	MX	3.543690111	cluster1	highrisk
TCGA-44-6779-01A	500	1	50	female	II	T2	N1	MX	3.298550165	cluster1	lowrisk

TCGA-44-7659-01A	691	0	70	male	I	T1	N0	MX	3.057376404	cluster1	lowrisk
TCGA-44-7660-01A	592	0	72	male	I	T2	N0	MX	3.653613158	cluster1	highrisk
TCGA-44-7661-01A	557	1	69	female	I	T2	N0	M0	4.028385584	cluster1	highrisk
TCGA-44-7662-01A	218	0	61	male	I	T2	N0	MX	3.661671239	cluster1	highrisk
TCGA-44-7667-01A	1097	0	49	female	II	T3	N0	MX	3.562816766	cluster1	highrisk
TCGA-44-7669-01A	574	1	59	male	II	T1	N1	MX	4.725791685	cluster2	highrisk
TCGA-44-7670-01A	882	0	47	female	II	T1	N1	M0	3.666274769	cluster1	highrisk
TCGA-44-7671-01A	889	0	64	male	I	T2	N0	M0	3.814542321	cluster2	highrisk
TCGA-44-7672-01A	719	0	52	female	I	T1	N0	M0	3.328279433	cluster1	lowrisk
TCGA-44-8117-01A	385	0	54	female	I	T2	N0	M0	3.112000965	cluster1	lowrisk
TCGA-44-8119-01A	285	0	73	male	II	T3	N0	M0	4.100829094	cluster1	highrisk
TCGA-44-8120-01A	260	0	58	male	I	T2	N0	M0	3.586077786	cluster1	highrisk
TCGA-44-A479-01A	486	0	73	female	I	T2	N0	MX	3.22643188	cluster1	lowrisk
TCGA-44-A47A-01A	466	0	78	female	I	T2	N0	MX	3.345035905	cluster1	lowrisk
TCGA-44-A47B-01A	287	0	79	male	I	T2	N0	M0	3.670872813	cluster1	highrisk
TCGA-44-A47G-01A	351	0	73	female	I	T1	N0	M0	3.521590778	cluster1	highrisk
TCGA-44-A4SS-01A	415	0	73	male	I	T1	N0	M0	3.702767485	cluster1	highrisk
TCGA-44-A4SU-01A	409	1	67	female	I	T1	N0	MX	3.607582128	cluster1	highrisk
TCGA-49-4486-01A	2318	1	72	male	I	T1	N0	M0	2.95454318	cluster1	lowrisk
TCGA-49-4487-01A	855	1	72	female	I	T1	N0	M0	3.898330297	cluster2	highrisk
TCGA-49-4488-01A	869	1	74	female	I	T1	N0	MX	3.459424929	cluster1	lowrisk
TCGA-49-4490-01A	385	1	45	female	III	T3	N2	M0	3.202164858	cluster1	lowrisk
TCGA-49-4494-01A	1081	1	77	male	III	T3	N2	M0	3.444351479	cluster1	lowrisk
TCGA-49-4501-01A	1421	1	67	female	I	T2	N0	M0	3.583829821	cluster1	highrisk
TCGA-49-4505-01A	428	1	61	female	II	T2	N1	M0	3.244487972	cluster1	lowrisk
TCGA-49-4506-01A	999	1	68	female	II	T2	N1	M0	3.810429428	cluster2	highrisk
TCGA-49-4507-01A	268	1	73	female	III	T3	N1	M0	3.49755852	cluster2	highrisk
TCGA-49-4510-01A	896	1	51	female	II	T2	N1	M0	3.372026605	cluster2	lowrisk
TCGA-49-4512-01A	905	1	69	female	III	T2	N2	MX	3.378914493	cluster1	lowrisk
TCGA-49-4514-01A	1700	0	79	female	I	T1	N0	M0	3.607249344	cluster2	highrisk
TCGA-49-6742-01A	488	1	70	male	II	T2	N1	M0	4.186859225	cluster2	highrisk
TCGA-49-6743-01A	1621	0	81	female	III	T1	N2	MX	3.496612017	cluster1	highrisk
TCGA-49-6744-01A	1683	0	64	female	II	T2	N1	MX	3.639406903	cluster1	highrisk
TCGA-49-6745-01A	522	0	82	male	III	T2	N2	M0	3.991325372	cluster1	highrisk
TCGA-49-6761-01A	354	0	68	female	III	T1	N2	MX	3.354038275	cluster1	lowrisk
TCGA-49-6767-01A	677	0	46	female	II	T3	N0	MX	3.958866319	cluster1	highrisk
TCGA-49-AAQV-01A	677	1	63	female	II	T1	N1	MX	3.325331741	cluster1	lowrisk
TCGA-49-AAR0-01A	4765	0	57	male	I	T1	N0	MX	3.393802622	cluster1	lowrisk

TCGA-49-AAR2-01A	2224	0	64	male	I	T2	N0	MX	3.542908491	cluster2	highrisk
TCGA-49-AAR3-01A	1893	0	69	male	II	T2	N1	MX	3.751570347	cluster1	highrisk
TCGA-49-AAR4-01A	879	1	51	male	III	T2	N2	MX	3.476620004	cluster1	highrisk
TCGA-49-AAR9-01A	260	1	61	male	II	T3	N0	MX	4.306537848	cluster1	highrisk
TCGA-49-AARE-01A	1229	1	51	female	I	T1	N0	MX	3.402471238	cluster1	lowrisk
TCGA-49-AARN-01A	1135	1	56	female	I	T1	N0	MX	3.191664845	cluster1	lowrisk
TCGA-49-AARO-01A	3759	0	39	female	I	T1	N0	MX	3.530436692	cluster1	highrisk
TCGA-49-AARQ-01A	6732	0	41	female	I	T2	N0	MX	3.038683607	cluster1	lowrisk
TCGA-49-AARR-01A	4992	0	68	male	I	T1	N0	MX	2.978255872	cluster1	lowrisk
TCGA-4B-A93V-01A	300	1	52	female	I	T1	N0	M0	3.807949532	cluster2	highrisk
TCGA-50-5044-01A	624	1	72	female	III	T4	N1	M0	4.029668284	cluster1	highrisk
TCGA-50-5045-01A	2174	1	57	female	unknown	T2	N1	M0	3.01497821	cluster1	lowrisk
TCGA-50-5049-01A	3094	0	70	male	I	T2	N0	M0	3.090484026	cluster1	lowrisk
TCGA-50-5051-01A	478	1	42	female	III	T2	N2	M0	3.760658422	cluster2	highrisk
TCGA-50-5055-01A	1830	1	79	female	II	T1	N1	M0	3.092435425	cluster1	lowrisk
TCGA-50-5066-01A	1442	0	72	male	I	T2	N0	M0	3.162663158	cluster1	lowrisk
TCGA-50-5068-01A	1499	1	59	female	II	T2	N1	MX	3.315750046	cluster1	lowrisk
TCGA-50-5072-01A	250	1	74	male	III	T2	N2	M0	4.332055701	cluster2	highrisk
TCGA-50-5930-01A	282	1	47	male	III	T2	N2	M0	3.60225221	cluster2	highrisk
TCGA-50-5931-01A	434	1	75	female	I	T2	N0	M0	3.990502109	cluster1	highrisk
TCGA-50-5932-01A	1235	1	75	male	II	T2	N1	M0	3.384734587	cluster1	lowrisk
TCGA-50-5933-01A	2393	1	72	male	III	T4	N2	M0	3.912392844	cluster1	highrisk
TCGA-50-5935-01A	653	1	86	female	I	T1	N0	M0	3.310960901	cluster1	lowrisk
TCGA-50-5936-01A	257	1	58	male	III	T2	N2	M0	4.467931223	cluster2	highrisk
TCGA-50-5939-01A	460	1	85	male	I	T2	N0	M0	4.128793768	cluster2	highrisk
TCGA-50-5941-01A	1474	0	55	female	III	T2	N2	M0	3.090324617	cluster1	lowrisk
TCGA-50-5942-01A	1847	0	67	female	I	T1	N0	M0	3.000228791	cluster1	lowrisk
TCGA-50-5944-01A	1750	0	69	female	I	T1	N0	M0	3.472878183	cluster1	highrisk
TCGA-50-5946-01A	1617	0	62	male	I	T1	N0	MX	3.316911571	cluster1	lowrisk
TCGA-50-6590-01A	1288	1	72	female	I	T2	N0	M0	3.745519633	cluster1	highrisk
TCGA-50-6591-01A	119	1	63	female	IV	T2	N0	M1	4.013011813	cluster1	highrisk
TCGA-50-6592-01A	777	1	71	female	I	T2	N0	M0	3.214175846	cluster1	lowrisk
TCGA-50-6593-01A	336	1	49	female	III	T1	N2	M0	3.411696948	cluster1	lowrisk
TCGA-50-6594-01A	370	1	79	female	III	T3	N2	M0	4.385390566	cluster1	highrisk
TCGA-50-6595-01A	189	1	74	female	III	T2	N2	M0	4.012642052	cluster1	highrisk
TCGA-50-6597-01A	1268	1	79	female	I	T2	N0	M0	3.640143391	cluster1	highrisk
TCGA-50-6673-01A	22	1	84	female	I	T1	N0	M0	3.188163149	cluster1	lowrisk
TCGA-50-7109-01A	308	1	60	male	I	T1	N0	M0	4.162048058	cluster2	highrisk

TCGA-50-8457-01A	1125	0	63	female	I	T1	N0	M0	2.925236762	cluster1	lowrisk
TCGA-50-8459-01A	1119	0	68	male	II	T3	N0	M0	3.307333149	cluster1	lowrisk
TCGA-50-8460-01A	829	0	74	male	I	T1	N0	M0	2.513764705	cluster1	lowrisk
TCGA-53-7624-01A	1043	1	40	female	IV	T2	N0	M1	4.024386957	cluster1	highrisk
TCGA-53-7626-01A	929	1	76	female	II	T1	N1	M0	3.014031324	cluster1	lowrisk
TCGA-53-7813-01A	424	0	51	female	III	T4	N0	M0	3.160487726	cluster1	lowrisk
TCGA-53-A4EZ-01A	1071	0	63	male	II	T2	N1	MX	3.351899959	cluster1	lowrisk
TCGA-55-1592-01A	701	1	65	male	I	T2	N0	M0	3.101066139	cluster1	lowrisk
TCGA-55-1594-01A	1178	0	68	male	III	T2	N2	M0	3.459540359	cluster1	lowrisk
TCGA-55-1595-01A	1479	0	74	female	I	T1	N0	M0	2.792133909	cluster1	lowrisk
TCGA-55-1596-01A	2065	0	55	male	II	T2	N1	M0	3.590246016	cluster1	highrisk
TCGA-55-5899-01A	930	0	58	male	unknown	T1	N1	M0	3.191940754	cluster1	lowrisk
TCGA-55-6543-01A	435	0	60	female	I	T1	N0	MX	3.075824872	cluster1	lowrisk
TCGA-55-6642-01A	2449	0	63	male	I	T2	N0	MX	3.235108612	cluster2	lowrisk
TCGA-55-6712-01A	171	1	71	male	II	T2	N1	MX	4.133195604	cluster1	highrisk
TCGA-55-6968-01A	1293	1	61	male	IV	T1	N0	M1	3.81418449	cluster1	highrisk
TCGA-55-6969-01A	1239	0	52	male	I	T2	N0	M0	3.57513251	cluster1	highrisk
TCGA-55-6970-01A	464	1	67	female	III	T2	N2	MX	4.025507341	cluster2	highrisk
TCGA-55-6971-01A	1400	0	59	female	I	T2	N0	MX	2.86160536	cluster1	lowrisk
TCGA-55-6972-01A	1632	1	72	male	I	T2	N0	M0	3.14640499	cluster1	lowrisk
TCGA-55-6975-01A	118	1	61	male	II	T2	N1	M0	4.179802395	cluster2	highrisk
TCGA-55-6978-01A	176	1	81	male	II	T2	N0	MX	4.145832615	cluster1	highrisk
TCGA-55-6979-01A	237	1	59	female	II	T2	N1	M0	3.233639108	cluster1	lowrisk
TCGA-55-6980-01A	2109	0	56	male	I	T1	N0	M0	3.828994904	cluster1	highrisk
TCGA-55-6981-01A	1379	1	53	female	III	T1	N2	M0	3.587785241	cluster1	highrisk
TCGA-55-6982-01A	995	1	79	female	II	T2	N1	M0	3.795295169	cluster1	highrisk
TCGA-55-6983-01A	2823	0	81	male	II	T2	N1	M0	3.438608163	cluster1	lowrisk
TCGA-55-6984-01A	760	1	71	female	II	T2	N1	M0	3.669939184	cluster1	highrisk
TCGA-55-6985-01A	1233	0	58	female	I	T2	N0	MX	3.049210126	cluster1	lowrisk
TCGA-55-6986-01A	3261	0	74	female	I	T2	N0	M0	3.624025309	cluster1	highrisk
TCGA-55-6987-01A	2137	0	77	male	I	T1	N0	M0	3.521935736	cluster2	highrisk
TCGA-55-7227-01A	952	1	77	male	III	T3	N1	MX	3.191021359	cluster1	lowrisk
TCGA-55-7281-01A	872	0	70	female	I	T1	N0	M0	3.182059082	cluster1	lowrisk
TCGA-55-7283-01A	609	0	76	female	III	T3	N2	MX	3.262118791	cluster1	lowrisk
TCGA-55-7570-01A	824	0	60	male	I	T1	N0	MX	3.693230909	cluster1	highrisk
TCGA-55-7573-01A	487	0	72	female	I	T1	N0	MX	3.000535517	cluster1	lowrisk
TCGA-55-7574-01A	995	1	64	female	I	T2	N0	M0	3.230480968	cluster1	lowrisk
TCGA-55-7576-01A	670	0	54	male	I	T2	N0	M0	3.493946117	cluster1	highrisk

TCGA-55-7724-01A	705	0	76	female	I	T2	N0	MX	3.485079472	cluster1	highrisk
TCGA-55-7725-01A	442	0	68	female	I	T1	N0	MX	3.252340924	cluster1	lowrisk
TCGA-55-7726-01A	652	0	72	female	I	T1	N0	MX	3.294653818	cluster1	lowrisk
TCGA-55-7727-01A	119	0	70	male	III	T1	N2	MX	3.671076206	cluster1	highrisk
TCGA-55-7728-01A	704	0	64	female	I	T2	N0	MX	3.008157988	cluster1	lowrisk
TCGA-55-7815-01A	773	0	76	male	I	T2	N0	MX	3.282276027	cluster1	lowrisk
TCGA-55-7816-01A	468	1	49	female	IV	TX	NX	MX	3.179563676	cluster1	lowrisk
TCGA-55-7903-01A	567	0	64	male	I	T1	N0	MX	4.088545431	cluster1	highrisk
TCGA-55-7907-01A	343	1	77	male	II	T2	N1	MX	3.476649388	cluster1	highrisk
TCGA-55-7910-01A	1040	0	50	female	II	T2	N0	M0	3.210967293	cluster2	lowrisk
TCGA-55-7911-01A	537	0	70	female	I	T1	N0	MX	3.525040088	cluster1	highrisk
TCGA-55-7914-01A	187	1	71	female	II	T1	N1	MX	3.517007342	cluster1	highrisk
TCGA-55-7994-01A	603	0	81	male	II	T3	N0	MX	3.246386642	cluster1	lowrisk
TCGA-55-7995-01A	889	0	73	female	I	T1	N0	M0	3.748716786	cluster1	highrisk
TCGA-55-8085-01A	904	0	64	male	I	T1	N0	M0	3.743369668	cluster1	highrisk
TCGA-55-8087-01A	462	0	59	female	I	T2	N0	MX	3.439983672	cluster1	lowrisk
TCGA-55-8089-01A	702	1	56	male	I	T1	N0	M0	3.597861217	cluster1	highrisk
TCGA-55-8090-01A	598	1	80	male	I	T1	N0	M0	3.218923212	cluster1	lowrisk
TCGA-55-8091-01A	600	0	74	male	I	T2	N0	MX	2.99947583	cluster1	lowrisk
TCGA-55-8092-01A	154	1	75	male	II	T3	N0	MX	3.713512176	cluster2	highrisk
TCGA-55-8094-01A	541	0	51	male	IV	T2	N0	M1b	3.678127528	cluster2	highrisk
TCGA-55-8096-01A	719	1	67	female	I	T2	N0	MX	3.499079732	cluster1	highrisk
TCGA-55-8097-01A	476	0	60	female	I	T1	N0	MX	3.384562718	cluster1	lowrisk
TCGA-55-8203-01A	547	0	69	female	I	T1	N0	M0	3.128499621	cluster1	lowrisk
TCGA-55-8204-01A	515	0	87	female	I	T2	N0	MX	3.314560355	cluster1	lowrisk
TCGA-55-8205-01A	599	0	76	female	II	T2	N0	M0	4.092101747	cluster1	highrisk
TCGA-55-8206-01A	888	0	56	male	I	T1	N0	M0	3.339313273	cluster1	lowrisk
TCGA-55-8207-01A	977	0	73	male	I	T2	N0	MX	2.980561475	cluster1	lowrisk
TCGA-55-8208-01A	674	0	73	female	I	T1	N0	M0	3.18791322	cluster1	lowrisk
TCGA-55-8299-01A	469	1	61	female	I	T1	N0	MX	3.666318382	cluster2	highrisk
TCGA-55-8301-01A	534	0	58	male	I	T2	N0	MX	3.256066997	cluster1	lowrisk
TCGA-55-8302-01A	478	0	54	male	I	T2	N0	MX	4.051905783	cluster1	highrisk
TCGA-55-8505-01A	440	0	62	male	III	T1	N2	MX	4.005710856	cluster2	highrisk
TCGA-55-8506-01A	11	0	62	female	II	T3	N0	MX	3.382440098	cluster1	lowrisk
TCGA-55-8507-01A	418	0	53	male	I	T1	N0	MX	3.304372413	cluster1	lowrisk
TCGA-55-8508-01A	617	0	60	female	II	T2	N1	MX	3.45307283	cluster2	lowrisk
TCGA-55-8510-01A	539	0	55	female	I	T2	N0	MX	3.655067978	cluster1	highrisk
TCGA-55-8511-01A	552	0	73	female	I	T2	N0	MX	3.663565097	cluster1	highrisk

TCGA-55-8512-01A	607	1	41	male	IV	T1	N1	M1b	3.218092742	cluster1	lowrisk
TCGA-55-8513-01A	791	0	77	female	II	T3	N0	MX	3.489030745	cluster1	highrisk
TCGA-55-8514-01A	520	0	70	female	I	T2	N0	MX	3.986876435	cluster1	highrisk
TCGA-55-8614-01A	536	0	76	male	I	T2	N0	MX	3.365850192	cluster1	lowrisk
TCGA-55-8615-01A	446	0	67	male	III	T3	N2	MX	3.674986084	cluster2	highrisk
TCGA-55-8616-01A	48	0	58	female	I	T2	N0	M0	3.474601314	cluster1	highrisk
TCGA-55-8619-01A	416	0	72	female	II	T3	N0	MX	3.067264672	cluster1	lowrisk
TCGA-55-8620-01A	375	1	60	male	IV	T1	N1	M1b	3.831632385	cluster1	highrisk
TCGA-55-8621-01A	515	0	75	female	I	T1	N0	MX	3.179206383	cluster1	lowrisk
TCGA-55-A48X-01A	689	0	63	female	II	T1	N1	M0	3.010730728	cluster1	lowrisk
TCGA-55-A48Y-01A	630	0	69	male	II	T2	N0	M0	4.235954806	cluster2	highrisk
TCGA-55-A48Z-01A	651	0	60	female	III	T1	N3	MX	3.33043248	cluster1	lowrisk
TCGA-55-A490-01A	99	1	78	male	II	T2	N0	MX	3.619567956	cluster1	highrisk
TCGA-55-A491-01A	626	0	81	female	I	T1	N0	MX	3.283501335	cluster1	lowrisk
TCGA-55-A492-01A	596	0	70	female	I	T1	N0	MX	3.678838538	cluster2	highrisk
TCGA-55-A493-01A	28	0	54	female	I	T2	N0	M0	3.65504666	cluster1	highrisk
TCGA-55-A494-01A	481	0	61	female	I	T2	N0	MX	3.409332954	cluster1	lowrisk
TCGA-55-A4DF-01A	440	1	88	male	I	T1	N0	MX	4.434657103	cluster1	highrisk
TCGA-55-A4DG-01A	608	0	71	male	I	T1	N0	MX	2.837131722	cluster1	lowrisk
TCGA-55-A57B-01A	546	0	80	female	I	T1	N0	M0	3.097222659	cluster1	lowrisk
TCGA-62-8394-01A	139	1	65	female	III	T4	N2	M0	3.769081464	cluster1	highrisk
TCGA-62-8395-01A	1216	0	80	female	II	T3	N0	M0	3.014752435	cluster1	lowrisk
TCGA-62-8397-01A	1289	0	70	female	II	T3	N0	M0	2.718641673	cluster1	lowrisk
TCGA-62-8398-01A	444	1	55	male	III	T2	N2	M0	4.525522317	cluster2	highrisk
TCGA-62-8399-01A	2696	0	62	male	III	T2	N2	M0	3.747520279	cluster2	highrisk
TCGA-62-8402-01A	1498	1	73	female	III	T2	N2	M0	3.37128049	cluster1	lowrisk
TCGA-62-A46O-01A	1454	1	65	female	I	T2	N0	M0	4.811391672	cluster2	highrisk
TCGA-62-A46P-01A	594	1	65	male	I	T2	N0	M0	2.880669824	cluster1	lowrisk
TCGA-62-A46R-01A	1725	1	54	female	I	T2	N0	M0	3.655431663	cluster1	highrisk
TCGA-62-A46S-01A	1653	1	73	male	I	T2	N0	M0	3.663920399	cluster2	highrisk
TCGA-62-A46U-01A	2067	0	71	female	II	T2	N1	M0	3.362256859	cluster1	lowrisk
TCGA-62-A46V-01A	2199	0	78	female	I	T2	N0	M0	3.292885479	cluster1	lowrisk
TCGA-62-A46Y-01A	414	1	70	female	III	T2	N2	M0	3.389936439	cluster1	lowrisk
TCGA-62-A470-01A	1194	1	84	male	I	T2	N0	M0	4.394468834	cluster2	highrisk
TCGA-62-A471-01A	1246	0	64	male	II	T2	N1	M0	4.728162706	cluster2	highrisk
TCGA-62-A472-01A	910	0	70	male	II	T3	N0	M0	3.477330602	cluster1	highrisk
TCGA-64-1676-01A	1728	0	58	male	I	T1	N0	M0	3.357340874	cluster1	lowrisk
TCGA-64-1677-01A	628	1	77	female	III	T2	N2	M0	3.256611996	cluster1	lowrisk

TCGA-64-1678-01A	1189	0	70	female	unknown	T2	N0	M0	3.649481958	cluster2	highrisk
TCGA-64-1679-01A	2488	0	58	female	III	T1	N2	M0	3.798626455	cluster1	highrisk
TCGA-64-1680-01A	1126	0	63	male	IV	T2	N2	M1	3.098261484	cluster1	lowrisk
TCGA-64-1681-01A	1167	1	61	female	I	T1	N0	M0	3.696030184	cluster1	highrisk
TCGA-64-5774-01A	2676	0	60	male	I	T2	N0	M0	3.975952798	cluster2	highrisk
TCGA-64-5775-01A	62	1	71	male	III	T4	N0	M0	4.852554345	cluster1	highrisk
TCGA-64-5778-01A	1305	0	60	male	I	T2	N0	M0	3.599681407	cluster1	highrisk
TCGA-64-5779-01A	864	0	61	male	III	T2	N2	M0	4.312809499	cluster1	highrisk
TCGA-64-5781-01A	1559	0	55	female	I	T2	N0	M0	3.61996091	cluster2	highrisk
TCGA-64-5815-01A	866	0	74	male	II	T2	N1	M0	3.584103138	cluster1	highrisk
TCGA-67-3770-01A	610	0	70	female	I	T1	N0	M0	3.063301278	cluster1	lowrisk
TCGA-67-3771-01A	610	0	77	female	I	T1	N0	M0	2.899251247	cluster1	lowrisk
TCGA-67-3772-01A	573	0	82	female	I	T2	N0	M0	3.240860447	cluster1	lowrisk
TCGA-67-3773-01A	427	0	84	female	I	T2	N0	M0	2.81759525	cluster1	lowrisk
TCGA-67-3774-01A	385	0	73	female	I	T2	N0	M0	3.64280686	cluster2	highrisk
TCGA-67-6215-01A	174	0	52	female	I	T2	N0	M0	3.305010172	cluster1	lowrisk
TCGA-67-6216-01A	141	0	57	female	I	T1	N0	M0	3.120688187	cluster1	lowrisk
TCGA-67-6217-01A	422	0	73	female	II	T2	N1	M0	3.276922586	cluster1	lowrisk
TCGA-69-7760-01A	202	0	73	male	II	T3	N0	M0	3.371044937	cluster1	lowrisk
TCGA-69-7761-01A	186	0	84	male	I	T2	N0	MX	3.347117209	cluster1	lowrisk
TCGA-69-7763-01A	690	0	69	male	I	T1	N0	M0	3.310864702	cluster2	lowrisk
TCGA-69-7764-01A	414	0	75	male	I	T1	N0	M0	3.546160533	cluster2	highrisk
TCGA-69-7765-01A	165	0	56	male	unknown	T4	N0	MX	3.1236727	cluster1	lowrisk
TCGA-69-7973-01A	230	0	42	female	I	T2	N0	M0	3.962264174	cluster2	highrisk
TCGA-69-7974-01A	184	0	54	female	III	T2	N2	MX	4.117066488	cluster1	highrisk
TCGA-69-7978-01A	134	0	59	male	II	T2	N1	MX	3.600383953	cluster1	highrisk
TCGA-69-7979-01A	408	0	71	female	I	T2	N0	MX	3.015977494	cluster1	lowrisk
TCGA-69-7980-01A	411	0	70	female	I	T1	N0	M0	3.525737372	cluster1	highrisk
TCGA-69-8253-01A	426	0	59	female	II	T1	N1	MX	3.166292442	cluster1	lowrisk
TCGA-69-8254-01A	409	0	85	male	unknown	T2	unknown	unknown	3.230714544	cluster1	lowrisk
TCGA-69-8255-01A	129	0	71	male	I	T1	N0	M0	4.142011906	cluster2	highrisk
TCGA-69-8453-01A	813	0	77	male	II	T3	N0	MX	3.431078843	cluster1	lowrisk
TCGA-69-A59K-01A	591	0	60	female	II	T3	N0	M0	3.098156904	cluster1	lowrisk
TCGA-71-6725-01A	256	0	48	female	I	T2	N0	M0	3.525822404	cluster1	highrisk
TCGA-71-8520-01A	210	1	60	female	I	T2	N0	M0	3.26339006	cluster1	lowrisk
TCGA-73-4658-01A	1600	1	80	female	I	T2	N0	M0	3.91366292	cluster1	highrisk
TCGA-73-4659-01A	711	1	66	male	III	T2	N2	M0	4.139723847	cluster2	highrisk
TCGA-73-4662-01A	2515	0	65	female	I	T1	N0	M0	3.235355278	cluster1	lowrisk

TCGA-73-4666-01A	800	0	52	female	IV	T1	N0	M1	3.94919283	cluster1	highrisk
TCGA-73-4668-01A	467	0	66	female	II	T2	N1	M0	3.634086029	cluster1	highrisk
TCGA-73-4670-01A	131	0	69	female	IV	T2	N0	M1	4.396206656	cluster2	highrisk
TCGA-73-4675-01A	922	1	59	male	III	T3	N1	M0	3.279761164	cluster1	lowrisk
TCGA-73-4676-01A	281	1	45	male	II	T2	N1	M0	4.279277807	cluster1	highrisk
TCGA-73-4677-01A	38	1	74	male	unknown	T2	N0	M0	3.1753088	cluster1	lowrisk
TCGA-73-7498-01A	1189	0	58	female	I	T1	N0	M0	2.921386308	cluster1	lowrisk
TCGA-73-7499-01A	1531	1	81	female	I	T2	N0	M0	3.174095352	cluster1	lowrisk
TCGA-73-A9RS-01A	340	1	41	male	II	T3	N0	M0	3.885622631	cluster2	highrisk
TCGA-75-5125-01A	2027	1	65	male	II	T2	N1	M0	3.802802809	cluster1	highrisk
TCGA-75-5146-01A	2368	0	65	male	I	T2	N0	M0	3.408641047	cluster1	lowrisk
TCGA-75-5147-01A	1333	0	65	female	I	T2	N0	M0	3.577323666	cluster1	highrisk
TCGA-75-6206-01A	2590	0	65	male	I	T2	N0	M0	3.571760413	cluster2	highrisk
TCGA-75-6212-01A	1516	1	65	female	II	T2	N1	M0	3.086763852	cluster1	lowrisk
TCGA-75-6214-01A	1115	1	65	female	III	T2	N2	M0	3.491563484	cluster1	highrisk
TCGA-75-7025-01A	3305	0	65	male	I	T2	N0	M0	3.050363707	cluster1	lowrisk
TCGA-75-7027-01A	3059	0	65	male	I	T2	N0	M0	4.66013769	cluster2	highrisk
TCGA-78-7143-01A	4961	1	62	female	I	T2	N0	M0	3.694386935	cluster1	highrisk
TCGA-78-7145-01A	826	1	52	female	IV	T4	N1	M1	4.017259367	cluster1	highrisk
TCGA-78-7146-01A	173	1	71	female	III	T2	N2	M0	3.777173975	cluster1	highrisk
TCGA-78-7147-01A	586	1	67	female	II	T2	N1	M0	3.641247957	cluster1	highrisk
TCGA-78-7148-01A	626	1	71	male	II	T2	N1	M0	4.036225303	cluster2	highrisk
TCGA-78-7149-01A	3940	0	71	male	III	T4	N0	M0	2.857490919	cluster1	lowrisk
TCGA-78-7150-01A	666	1	59	male	II	T2	N1	M0	4.025204811	cluster2	highrisk
TCGA-78-7152-01A	1215	1	65	male	I	T2	N0	M0	3.592999941	cluster1	highrisk
TCGA-78-7153-01A	3635	0	65	female	I	T2	N0	M0	3.608687604	cluster1	highrisk
TCGA-78-7154-01A	593	1	72	male	III	T3	N2	M0	3.535053279	cluster1	highrisk
TCGA-78-7155-01A	1171	1	68	male	I	T2	N0	M0	3.886918572	cluster1	highrisk
TCGA-78-7156-01A	976	1	62	male	IV	T4	N1	M1	3.525443943	cluster1	highrisk
TCGA-78-7158-01A	179	1	59	female	III	T4	N2	M0	3.192727932	cluster1	lowrisk
TCGA-78-7159-01A	1974	0	60	female	I	T1	NX	M0	3.876633855	cluster2	highrisk
TCGA-78-7160-01A	697	1	61	male	IV	T4	N2	M1	4.26159726	cluster2	highrisk
TCGA-78-7161-01A	291	1	69	female	II	T3	N0	M0	3.744684111	cluster2	highrisk
TCGA-78-7162-01A	3169	1	75	male	I	T1	N0	M0	3.559541594	cluster2	highrisk
TCGA-78-7163-01A	7248	0	60	male	I	T2	N0	M0	3.533802841	cluster1	highrisk
TCGA-78-7166-01A	258	1	84	male	II	T2	N1	M0	3.497323863	cluster2	highrisk
TCGA-78-7167-01A	2681	1	77	male	IV	T2	N0	M1	3.303296695	cluster2	lowrisk
TCGA-78-7220-01A	807	1	53	female	III	T2	N2	M0	3.957648291	cluster2	highrisk

TCGA-78-7535-01A	949	1	45	male	I	T2	N0	M0	3.103166791	cluster1	lowrisk
TCGA-78-7536-01A	244	1	69	male	III	T2	N2	M0	4.361359776	cluster2	highrisk
TCGA-78-7537-01A	1622	1	72	male	I	T2	N0	M0	3.080051395	cluster1	lowrisk
TCGA-78-7539-01A	791	0	75	female	II	T2	N0	M0	3.188803558	cluster1	lowrisk
TCGA-78-7540-01A	1197	1	66	female	I	T2	N0	M0	3.319693486	cluster1	lowrisk
TCGA-78-7542-01A	321	1	56	male	I	T2	N0	M0	4.366323412	cluster1	highrisk
TCGA-78-7633-01A	1528	1	67	male	I	T2	N0	M0	3.440870525	cluster2	lowrisk
TCGA-78-8640-01A	7062	0	59	male	II	T1	N1	M0	2.903742596	cluster1	lowrisk
TCGA-78-8648-01A	1209	1	58	female	II	T3	N0	M0	3.102548201	cluster1	lowrisk
TCGA-78-8655-01A	2360	0	77	female	I	T1	N0	M0	3.139093524	cluster1	lowrisk
TCGA-78-8660-01A	321	1	69	male	II	T2	N1	M0	3.140515721	cluster1	lowrisk
TCGA-78-8662-01A	3361	1	53	female	I	T2	N0	M0	3.619141622	cluster1	highrisk
TCGA-80-5608-01A	2832	0	65	female	I	T1	N0	M0	3.832537035	cluster2	highrisk
TCGA-80-5611-01A	2595	0	65	male	I	T2	N0	M0	3.794857836	cluster1	highrisk
TCGA-83-5908-01A	824	0	59	female	I	T1	N0	M0	4.48569071	cluster1	highrisk
TCGA-86-6562-01A	376	1	52	male	II	T2	N1	M0	3.475124051	cluster1	highrisk
TCGA-86-6851-01A	179	0	73	female	II	T1	N1	M0	2.648101776	cluster1	lowrisk
TCGA-86-7701-01A	947	0	66	male	IV	T2	N0	M1	3.868747199	cluster2	highrisk
TCGA-86-7711-01A	1046	1	70	male	II	T2	N1	M0	3.857799131	cluster1	highrisk
TCGA-86-7713-01A	1157	0	70	male	II	T2	N0	M0	4.129887453	cluster2	highrisk
TCGA-86-7714-01A	625	1	61	female	III	T1	N2	M0	3.134393556	cluster1	lowrisk
TCGA-86-7953-01A	997	0	69	female	I	T1	N0	M0	3.663188592	cluster1	highrisk
TCGA-86-7954-01A	605	0	68	female	I	T2	N0	M0	3.519626788	cluster1	highrisk
TCGA-86-7955-01A	1072	0	62	male	I	T2	N0	M0	4.63356855	cluster2	highrisk
TCGA-86-8054-01A	1148	0	61	male	II	T2	N1	M0	4.024993575	cluster2	highrisk
TCGA-86-8055-01A	124	1	79	male	II	T2	N1	M0	3.343257096	cluster1	lowrisk
TCGA-86-8056-01A	139	0	63	female	III	T4	N0	M0	3.473482749	cluster1	highrisk
TCGA-86-8073-01A	740	0	58	male	I	T2	N0	M0	3.608220841	cluster1	highrisk
TCGA-86-8074-01A	24	0	62	female	II	T1	N1	M0	3.764540179	cluster1	highrisk
TCGA-86-8075-01A	694	1	66	female	I	T2	N0	M0	3.535846502	cluster1	highrisk
TCGA-86-8076-01A	993	0	42	male	I	T1	N0	M0	3.344549933	cluster2	lowrisk
TCGA-86-8278-01A	944	0	63	female	II	T2	N1	M0	3.238841907	cluster1	lowrisk
TCGA-86-8279-01A	949	0	46	male	II	T2	N1	M0	3.534316725	cluster1	highrisk
TCGA-86-8280-01A	701	0	54	female	II	T2	N0	M0	3.456770125	cluster1	lowrisk
TCGA-86-8358-01A	653	0	44	male	I	T2	N0	M0	3.421510897	cluster1	lowrisk
TCGA-86-8359-01A	444	1	52	male	III	T3	N2	M0	3.48187117	cluster2	highrisk
TCGA-86-8585-01A	353	0	57	male	I	T2	N0	M0	4.167772371	cluster2	highrisk
TCGA-86-8668-01A	423	0	61	female	I	T1	N0	M0	3.08870787	cluster1	lowrisk

TCGA-86-8669-01A	938	0	64	male	I	T1	N0	M0	3.439267018	cluster1	lowrisk
TCGA-86-8671-01A	839	0	72	female	II	T2	N1	M0	3.007340729	cluster1	lowrisk
TCGA-86-8672-01A	19	1	59	male	II	T3	N0	M0	3.652309944	cluster1	highrisk
TCGA-86-8673-01A	862	0	61	male	I	T2	N0	M0	3.300940635	cluster1	lowrisk
TCGA-86-8674-01A	806	0	50	male	II	T2	N1	M0	3.128784415	cluster1	lowrisk
TCGA-86-A456-01A	896	0	78	female	I	T1	N0	M0	3.691572367	cluster1	highrisk
TCGA-86-A4D0-01A	116	1	48	male	II	T2	N0	M0	4.234528622	cluster2	highrisk
TCGA-86-A4JF-01A	737	1	56	male	II	T3	N0	M0	3.410297586	cluster1	lowrisk
TCGA-86-A4P7-01A	415	0	63	female	I	T2	N0	M0	3.135526583	cluster1	lowrisk
TCGA-86-A4P8-01A	805	0	59	female	III	T1	N2	MX	3.263327968	cluster1	lowrisk
TCGA-91-6828-01A	323	0	70	male	I	T1	N0	M0	3.208889191	cluster1	lowrisk
TCGA-91-6829-01A	1258	1	78	male	I	T2	N0	MX	3.706566451	cluster1	highrisk
TCGA-91-6830-01A	60	0	65	female	II	T1	N1	MX	3.76234708	cluster1	highrisk
TCGA-91-6831-01A	310	0	66	male	I	T2	N0	MX	4.507738899	cluster2	highrisk
TCGA-91-6835-01A	79	0	81	female	I	T1	N0	M0	3.561386171	cluster1	highrisk
TCGA-91-6836-01A	417	0	52	female	I	T2	N0	MX	3.9477842	cluster1	highrisk
TCGA-91-6840-01A	372	0	59	female	I	T1	N0	M0	3.766052059	cluster1	highrisk
TCGA-91-6847-01A	842	0	62	female	I	T2	N0	MX	3.840790749	cluster1	highrisk
TCGA-91-6848-01A	224	0	59	male	III	T2	N2	MX	3.51079468	cluster1	highrisk
TCGA-91-6849-01A	35	0	75	female	III	T2	N2	MX	2.94113356	cluster1	lowrisk
TCGA-91-7771-01A	492	0	62	male	II	T3	N0	MX	3.605900541	cluster1	highrisk
TCGA-91-8496-01A	505	0	63	female	I	T2	NX	MX	3.054268561	cluster1	lowrisk
TCGA-91-8497-01A	434	1	75	female	I	T1	N0	MX	2.908682609	cluster1	lowrisk
TCGA-91-8499-01A	36	0	76	female	I	T1	N0	MX	4.168018812	cluster1	highrisk
TCGA-91-A4BC-01A	44	0	59	male	II	T2	N0	MX	3.498310138	cluster2	highrisk
TCGA-91-A4BD-01A	603	0	78	male	II	T1	N1	MX	2.970980586	cluster1	lowrisk
TCGA-93-7347-01A	683	0	76	female	I	T1	N0	MX	3.009332006	cluster1	lowrisk
TCGA-93-7348-01A	531	0	75	female	I	T1	N0	MX	3.162933092	cluster1	lowrisk
TCGA-93-8067-01A	186	0	77	male	I	T2	N0	MX	4.644494828	cluster2	highrisk
TCGA-93-A4JN-01A	718	0	71	male	IV	T2	N0	M1a	3.204160397	cluster1	lowrisk
TCGA-93-A4JO-01A	33	1	70	male	I	T1	N0	MX	3.008972787	cluster1	lowrisk
TCGA-93-A4JP-01A	578	0	64	male	IV	TX	NX	M1b	3.546854473	cluster1	highrisk
TCGA-93-A4JQ-01A	526	0	49	male	I	T1	N0	MX	2.913164632	cluster1	lowrisk
TCGA-95-7039-01A	1272	0	54	female	II	T3	N0	MX	3.546410223	cluster1	highrisk
TCGA-95-7043-01A	503	1	63	female	I	T1	N0	MX	3.515766847	cluster1	highrisk
TCGA-95-7562-01A	87	1	71	male	II	T2	N1	M0	3.303557275	cluster1	lowrisk
TCGA-95-7567-01A	568	0	61	male	II	T2	N1	M0	3.108353089	cluster1	lowrisk
TCGA-95-7944-01A	377	0	71	male	I	T1	N0	M0	3.354336474	cluster1	lowrisk

TCGA-95-7947-01A	477	0	67	male	I	T1	N0	M0	3.753467981	cluster1	highrisk
TCGA-95-7948-01A	476	0	42	female	I	T2	N0	M0	3.716184006	cluster1	highrisk
TCGA-95-8039-01A	830	0	72	male	I	T1	N0	MX	3.471352862	cluster1	lowrisk
TCGA-95-8494-01A	84	0	67	male	II	T2	N1	M0	4.049003404	cluster1	highrisk
TCGA-95-A4VK-01A	651	0	74	female	III	T2	N2	M0	3.197226263	cluster1	lowrisk
TCGA-95-A4VN-01A	553	0	62	female	II	T2	N1	M0	3.807947691	cluster1	highrisk
TCGA-95-A4VP-01A	605	0	66	female	III	T2	N2	M0	3.488947151	cluster2	highrisk
TCGA-97-7546-01A	1285	0	76	female	I	T1	N0	MX	3.336908191	cluster1	lowrisk
TCGA-97-7547-01A	1965	0	67	female	I	T2	N0	MX	3.4629741	cluster1	lowrisk
TCGA-97-7552-01A	1932	0	70	male	I	T2	N0	MX	3.212185965	cluster1	lowrisk
TCGA-97-7553-01A	1870	0	58	female	I	T1	N0	MX	3.467583222	cluster1	lowrisk
TCGA-97-7554-01A	775	0	83	female	III	T2	N2	M0	3.090350945	cluster1	lowrisk
TCGA-97-7937-01A	564	0	65	male	I	T2	N0	MX	3.567661063	cluster1	highrisk
TCGA-97-7938-01A	18	1	76	female	I	T1	N0	MX	3.142737181	cluster1	lowrisk
TCGA-97-7941-01A	484	0	72	female	I	T1	N0	MX	3.449699317	cluster1	lowrisk
TCGA-97-8171-01A	568	0	81	male	IV	T2	N2	M1a	3.086703825	cluster1	lowrisk
TCGA-97-8172-01A	545	0	75	female	I	T2	N0	M0	3.124097886	cluster1	lowrisk
TCGA-97-8174-01A	164	1	67	male	II	T2	N0	M0	3.764620053	cluster2	highrisk
TCGA-97-8175-01A	551	0	55	female	I	T2	N0	M0	3.786969945	cluster1	highrisk
TCGA-97-8176-01A	468	1	63	male	III	T3	N1	M0	4.382527904	cluster2	highrisk
TCGA-97-8177-01A	499	0	59	female	I	T2	N0	M0	3.443219039	cluster1	lowrisk
TCGA-97-8179-01A	435	0	72	male	I	T1	N0	M0	3.883593449	cluster2	highrisk
TCGA-97-8547-01A	657	0	78	female	III	T2	N2	MX	3.326427217	cluster1	lowrisk
TCGA-97-8552-01A	626	0	55	female	I	T1	N0	MX	3.3391398	cluster1	lowrisk
TCGA-97-A4LX-01A	614	0	81	male	I	T2	N0	M0	3.102771259	cluster1	lowrisk
TCGA-97-A4M0-01A	652	0	60	female	I	T2	N0	M0	3.189899569	cluster1	lowrisk
TCGA-97-A4M1-01A	601	0	52	female	I	T1	N0	M0	3.191772578	cluster1	lowrisk
TCGA-97-A4M2-01A	624	0	66	male	I	T1	N0	M0	3.026132683	cluster1	lowrisk
TCGA-97-A4M3-01A	540	0	69	female	I	T1	N0	M0	3.489015722	cluster2	highrisk
TCGA-97-A4M5-01A	634	0	83	male	I	T1	N0	M0	3.945873255	cluster2	highrisk
TCGA-97-A4M6-01A	568	0	45	female	I	T1	N0	M0	3.352498115	cluster1	lowrisk
TCGA-97-A4M7-01A	629	0	74	male	I	T1	N0	M0	2.858535281	cluster1	lowrisk
TCGA-99-7458-01A	747	0	74	female	III	T4	N0	M0	3.106836079	cluster1	lowrisk
TCGA-99-8025-01A	1060	0	72	female	III	T3	N2	M0	3.493006981	cluster1	highrisk
TCGA-99-8028-01A	1118	0	50	female	I	T1	N0	M0	3.100675734	cluster1	lowrisk
TCGA-99-8032-01A	44	0	61	male	I	T1	N0	M0	3.870189657	cluster2	highrisk
TCGA-99-8033-01A	656	1	74	female	IV	TX	NX	M1	4.176055877	cluster2	highrisk
TCGA-99-AA5R-01A	658	0	70	female	I	T1	N0	M0	2.954638766	cluster1	lowrisk

TCGA-J2-8192-01A	739	0	65	female	II	T2	N1	MX	3.574987259	cluster1	highrisk
TCGA-J2-8194-01A	724	0	69	female	II	T3	N0	MX	4.175518743	cluster2	highrisk
TCGA-J2-A4AD-01A	550	1	61	female	I	T1	N0	MX	3.610263566	cluster1	highrisk
TCGA-J2-A4AE-01A	1079	0	77	female	I	T1	N0	MX	3.070564	cluster1	lowrisk
TCGA-J2-A4AG-01A	988	0	66	female	I	T1	N0	MX	3.468690599	cluster1	lowrisk
TCGA-L4-A4E5-01A	578	0	48	female	I	T1	N0	M0	3.015072126	cluster1	lowrisk
TCGA-L4-A4E6-01A	435	0	67	male	I	T1	N0	M0	3.406197808	cluster1	lowrisk
TCGA-L9-A443-01A	193	1	63	female	I	T1	N0	MX	3.153047855	cluster1	lowrisk
TCGA-L9-A444-01A	307	0	60	female	I	T1	N0	MX	2.589275988	cluster1	lowrisk
TCGA-L9-A50W-01A	442	1	75	male	II	T1	N1	MX	3.218922068	cluster1	lowrisk
TCGA-L9-A5IP-01A	58	1	40	female	IV	T3	N2	M1b	3.984860061	cluster2	highrisk
TCGA-L9-A743-01A	664	0	56	male	II	T2	N1	M0	3.299529673	cluster1	lowrisk
TCGA-L9-A7SV-01A	565	0	69	male	II	T2	N1	M0	2.99340924	cluster1	lowrisk
TCGA-L9-A8F4-01A	476	0	64	female	I	T2	N0	MX	3.302621235	cluster1	lowrisk
TCGA-MN-A4N1-01A	827	0	60	male	II	T2	N1	M0	3.122127263	cluster1	lowrisk
TCGA-MN-A4N4-01A	1175	0	57	male	I	T1	N0	M0	3.295393917	cluster1	lowrisk
TCGA-MN-A4N5-01A	84	0	63	male	I	T1	N0	M0	2.879288114	cluster1	lowrisk
TCGA-MP-A4SV-01A	2620	1	67	male	I	T2	N0	M0	3.580748759	cluster1	highrisk
TCGA-MP-A4SW-01A	1778	1	53	male	II	T2	N1	M0	3.232908253	cluster1	lowrisk
TCGA-MP-A4SY-01A	1501	1	61	male	II	T2	N1	M0	3.859440386	cluster1	highrisk
TCGA-MP-A4TI-01A	429	1	72	male	II	T2	N1	M0	3.475518701	cluster1	highrisk
TCGA-MP-A4T4-01A	2617	1	68	female	II	T2	N1	M0	3.358217765	cluster1	lowrisk
TCGA-MP-A4T6-01A	1790	1	76	female	III	T1	N2	MX	3.23379565	cluster2	lowrisk
TCGA-MP-A4T7-01A	167	1	75	female	IV	T2	N0	M1	3.762416832	cluster2	highrisk
TCGA-MP-A4T8-01A	161	1	68	male	III	T2	N2	M0	4.011050916	cluster1	highrisk
TCGA-MP-A4T9-01A	1265	1	54	female	III	T2	N2	MX	3.694184001	cluster2	highrisk
TCGA-MP-A4TA-01A	950	1	75	female	I	T1	N0	M0	4.273338198	cluster1	highrisk
TCGA-MP-A4TC-01A	74	1	77	male	III	T1	N2	M0	3.831603882	cluster2	highrisk
TCGA-MP-A4TD-01A	307	1	71	male	III	T2	N2	M0	3.616430451	cluster2	highrisk
TCGA-MP-A4TE-01A	896	1	56	male	II	T2	N0	MX	4.073540304	cluster2	highrisk
TCGA-MP-A4TF-01A	336	1	58	female	II	T2	N0	M0	3.865350397	cluster1	highrisk
TCGA-MP-A4TH-01A	741	0	70	female	I	T1	N0	M0	3.068451408	cluster1	lowrisk
TCGA-MP-A4TJ-01A	339	1	62	female	I	T1	N0	M0	2.949920694	cluster1	lowrisk
TCGA-MP-A4TK-01A	582	1	56	female	II	T2	N1	MX	3.350544527	cluster1	lowrisk
TCGA-MP-A5C7-01A	2248	0	76	female	I	T2	N0	M0	3.084406889	cluster1	lowrisk
TCGA-NJ-A4YI-01A	4	1	87	female	III	T2	N2	M0	3.166721529	cluster2	lowrisk
TCGA-NJ-A4YF-01A	2161	0	50	female	I	T1	N0	M0	3.692091626	cluster1	highrisk
TCGA-NJ-A4YG-01A	2261	0	65	male	I	T2	N0	M0	3.074796093	cluster1	lowrisk

TCGA-NJ-A4YP-01A	50	0	52	male	I	T2	N0	M0	3.97109574	cluster2	highrisk
TCGA-NJ-A4YQ-01A	1432	0	69	female	I	T1	N0	M0	3.368060468	cluster1	lowrisk
TCGA-NJ-A55A-01A	15	0	76	female	I	T2	N0	M0	3.191524583	cluster1	lowrisk
TCGA-NJ-A55O-01A	13	0	56	female	II	T1	N1	M0	4.050032301	cluster2	highrisk
TCGA-NJ-A55R-01A	603	0	67	male	I	T1	N0	MX	2.85284458	cluster1	lowrisk
TCGA-NJ-A7XG-01A	617	0	49	male	III	T4	N1	M0	2.68482425	cluster1	lowrisk
TCGA-O1-A52J-01A	1798	1	74	female	I	T1	N0	MX	3.639238591	cluster1	highrisk
TCGA-S2-AA1A-01A	513	0	68	female	I	T1	N0	M0	2.638265635	cluster1	lowrisk

Supplementary Table S 2

symbol	logFC	AveExpr	t	P.Value	adj.P.Val	B	entrezID
CPS1	-6.83617	2.2323673	-59.90516	5.44E-230	1.08E-225	502.67102	148811
INHA	-3.521795	1.9321858	-19.4362	4.63E-63	4.61E-59	132.21905	3623
SLC16A14	-2.728945	3.4582817	-17.62901	2.00E-54	1.33E-50	112.66081	151473
PDE10A	-1.640777	1.0696534	-17.38832	2.74E-53	1.37E-49	110.08422	10846
FGA	-5.158363	3.9278116	-17.14321	3.91E-52	1.56E-48	107.46883	2243
DUSP4	-2.526691	4.5924414	-16.77676	2.04E-50	6.79E-47	103.5763	1846
HAL	-2.229453	2.0380605	-16.71424	4.00E-50	1.14E-46	102.91439	3034
SLC7A2	-2.941746	4.2074353	-15.56731	7.99E-45	1.99E-41	90.902155	6542
INSL4	-2.643923	0.8790374	-15.48143	1.97E-44	4.37E-41	90.0137	3641
FGL1	-3.576877	2.5367207	-14.64782	1.15E-40	2.28E-37	81.482025	2267
PPARGC1A	-1.64456	1.3523033	-14.52106	4.21E-40	7.63E-37	80.20045	10891
FURIN	-1.64682	7.4747493	-14.4427	9.40E-40	1.56E-36	79.410373	5045
PDE4D	-1.62134	3.2225029	-13.99074	9.30E-38	1.43E-34	74.887907	5144
FGB	-4.384413	2.6619928	-13.64448	3.01E-36	4.29E-33	71.46509	2244
ETNPPL	-1.445011	0.5892846	-12.95917	2.61E-33	3.26E-30	64.807276	64850
ADGRF1	2.6340833	3.2614567	12.756294	1.88E-32	2.20E-29	62.868243	266977
TTC39C	-1.082749	3.6126416	-12.46215	3.18E-31	3.52E-28	60.084339	125488
FGG	-4.17168	5.2812574	-12.38992	6.34E-31	6.59E-28	59.405916	2266
BMP6	-1.933268	2.6559891	-12.38547	6.61E-31	6.59E-28	59.364134	654
CARD14	-1.265939	1.6049488	-12.24565	2.50E-30	2.37E-27	58.056899	79092
TLR2	1.4478299	4.4345063	12.209682	3.51E-30	3.18E-27	57.721939	7097
SMOC1	-2.525495	2.3916695	-12.1391	6.84E-30	5.93E-27	57.066044	64093
IRS2	-1.500394	4.1576984	-12.12132	8.09E-30	6.72E-27	56.901159	8660
ODC1	-1.797064	7.4295748	-12.01641	2.17E-29	1.71E-26	55.930937	4953
PER3	1.2462894	3.0429506	12.013591	2.23E-29	1.71E-26	55.9049	8863
CRYBG1	1.4383333	3.8554453	11.976097	3.16E-29	2.34E-26	55.559288	202
S100P	-3.726619	8.2338924	-11.89495	6.76E-29	4.81E-26	54.813327	6286
CD83	1.1730006	5.0081733	11.867583	8.72E-29	6.00E-26	54.562389	9308
TESC	-2.759552	5.5136709	-11.84478	1.08E-28	7.17E-26	54.353502	54997
PDE3A	-1.481433	1.9714694	-11.73702	2.93E-28	1.89E-25	53.369613	5139
FXYD4	-2.19457	1.7140046	-11.62399	8.34E-28	5.19E-25	52.343053	53828
FZD10	-1.880175	1.4057455	-11.57607	1.30E-27	7.83E-25	51.909493	11211
BHLHA15	-1.510756	3.2553897	-11.47756	3.20E-27	1.87E-24	51.021506	168620
TFF1	-3.828156	3.2340371	-11.41593	5.61E-27	3.20E-24	50.468215	7031
CX3CL1	1.8409125	5.0786802	11.377377	7.98E-27	4.18E-24	50.122938	6376
SYTL2	1.1647437	3.5415494	11.285632	1.83E-26	9.36E-24	49.304091	54843

DHDH	1.2338501	2.6495178	11.283082	1.88E-26	9.36E-24	49.281381	27294
BASP1	-1.587595	6.4469939	-11.26184	2.28E-26	1.11E-23	49.092414	10409
HLA-DQB2	2.0440795	5.8685273	11.217878	3.39E-26	1.61E-23	48.701873	3120
MAML2	1.1071985	3.4867873	11.210446	3.62E-26	1.68E-23	48.635941	84441
HLA-DPA1	1.4835754	7.8088735	11.183542	4.62E-26	2.04E-23	48.397504	3113
COL25A1	-1.808563	0.9935454	-11.1624	5.58E-26	2.42E-23	48.210381	84570
FCGBP	1.979615	3.4258547	11.078328	1.19E-25	4.93E-23	47.468308	8857
GPT2	-1.385316	4.7251785	-11.03399	1.77E-25	7.19E-23	47.078311	84706
HLA-DRA	1.4189826	11.540705	10.980483	2.85E-25	1.14E-22	46.608939	3122
HLA-DRB1	1.4813898	10.688061	10.976083	2.96E-25	1.14E-22	46.570396	3123
ARHGAP20	1.2153691	1.9642959	10.899842	5.84E-25	2.20E-22	45.904145	57569
HLA-DQB1	1.6276727	6.8468167	10.889326	6.41E-25	2.37E-22	45.81247	3119
C2CD4D	-1.001691	1.9238576	-10.87487	7.28E-25	2.64E-22	45.686551	100191040
HPGDS	1.2358495	2.1465844	10.839764	9.94E-25	3.54E-22	45.381135	27306
FAS	1.0944421	3.5880018	10.827148	1.11E-24	3.89E-22	45.271526	355
MFSD4A	1.9935745	3.7599711	10.797963	1.44E-24	4.94E-22	45.01828	148808
HLA-DRB5	1.7745161	8.9830857	10.788515	1.56E-24	5.19E-22	44.936389	3127
CD40	1.167537	5.2499649	10.784191	1.62E-24	5.31E-22	44.898921	958
CPE	-1.759314	6.4517096	-10.75101	2.18E-24	7.00E-22	44.611743	1363
CACNB1	1.1559577	2.8567218	10.726151	2.71E-24	8.57E-22	44.396946	782
DAPP1	1.1134238	3.5578784	10.688756	3.76E-24	1.15E-21	44.074406	27071
KCNK5	1.516002	5.3546752	10.65962	4.85E-24	1.47E-21	43.823597	8645
ZFP36L1	1.0198966	8.0794444	10.597867	8.33E-24	2.45E-21	43.293423	677
PCSK1	-2.229263	1.2508251	-10.59741	8.36E-24	2.45E-21	43.289507	5122
ICAM1	1.4796043	7.5054873	10.579029	9.81E-24	2.84E-21	43.132078	3383
FNIP2	1.1104771	4.2278055	10.571818	1.04E-23	2.98E-21	43.070364	57600
CSF1	1.1525169	5.143054	10.522991	1.60E-23	4.49E-21	42.653184	1435
FSTL4	-1.805821	2.209086	-10.48195	2.28E-23	6.32E-21	42.303497	23105
UGT2B4	-1.445527	0.7688921	-10.47393	2.45E-23	6.68E-21	42.235268	7363
B4GALNT2	-1.464146	0.9514674	-10.4563	2.85E-23	7.57E-21	42.085384	124872
CALCA	-3.448172	1.8413199	-10.44104	3.25E-23	8.53E-21	41.955765	796
TACC2	-1.272656	4.0751601	-10.40679	4.37E-23	1.13E-20	41.66529	10579
CYTH3	1.0710117	4.7917223	10.390537	5.03E-23	1.29E-20	41.527675	9265
CD74	1.3020986	10.963133	10.370794	5.96E-23	1.50E-20	41.360696	972
HLA-DMB	1.1710177	5.3058894	10.323063	8.98E-23	2.18E-20	40.957833	3109
BARX1	-2.721641	2.4029809	-10.31229	9.85E-23	2.37E-20	40.867079	56033
HLA-DPB1	1.2825731	7.3702117	10.257206	1.58E-22	3.62E-20	40.403954	3115
WLS	1.3207625	5.5149499	10.190198	2.80E-22	6.34E-20	39.84275	79971

PCK1	-1.017148	0.3839064	-10.1845	2.94E-22	6.58E-20	39.795146	5105
SIK1B	-1.453933	3.3426451	-10.13773	4.37E-22	9.68E-20	39.405014	102724428
CLEC5A	1.2090185	2.6659596	10.000526	1.39E-21	2.83E-19	38.267302	23601
RNF186	-1.024486	0.8189182	-9.9617	1.93E-21	3.81E-19	37.947216	54546
IFT57	1.1699034	5.9745517	9.9515956	2.10E-21	4.11E-19	37.864049	55081
PLEKHG2	-1.083996	4.2618723	-9.934047	2.43E-21	4.67E-19	37.719746	64857
BHLHE41	1.147177	3.8321425	9.9246626	2.63E-21	4.95E-19	37.642645	79365
ANOS1	1.2900437	3.553255	9.8879726	3.58E-21	6.67E-19	37.341679	3730
DPYD	1.1453347	4.4319146	9.881993	3.76E-21	6.94E-19	37.2927	1806
ITGB2	1.2319981	6.2291953	9.7922638	7.93E-21	1.41E-18	36.560104	3689
AVPI1	-1.075434	5.5870475	-9.744572	1.18E-20	2.02E-18	36.172553	60370
F2	-1.053664	0.4345926	-9.675834	2.08E-20	3.37E-18	35.616238	2147
FABP3	1.5529769	4.5767165	9.6502789	2.56E-20	4.09E-18	35.410099	2170
LST1	1.0735683	4.3165629	9.6153651	3.41E-20	5.32E-18	35.129067	7940
SCD5	1.2643631	3.3988275	9.6118822	3.51E-20	5.43E-18	35.10107	79966
HLA-DMA	1.0804582	7.2223043	9.5830071	4.45E-20	6.67E-18	34.869229	3108
LY86	1.096645	4.4116206	9.5268448	7.04E-20	9.95E-18	34.419668	9450
LGALS9	1.0182827	5.2069287	9.5156578	7.71E-20	1.07E-17	34.330338	3965
MNDA	1.1814326	4.5623689	9.5110641	8.00E-20	1.11E-17	34.293677	4332
CLEC7A	1.104452	3.9997491	9.4470302	1.35E-19	1.80E-17	33.783917	64581
CH25H	1.5147057	3.8219873	9.3969531	2.02E-19	2.65E-17	33.386927	9023
LGSN	-1.762693	2.733954	-9.374956	2.41E-19	3.08E-17	33.21301	51557
SLC6A20	1.383447	1.5557171	9.3647966	2.61E-19	3.29E-17	33.132779	54716
NTS	-2.300154	1.4562259	-9.362763	2.66E-19	3.31E-17	33.11673	4922
IL27RA	1.0081835	5.1813337	9.2473168	6.71E-19	8.06E-17	32.209466	9466
ADSS1	-1.041979	3.0451636	-9.241545	7.02E-19	8.29E-17	32.164319	122622
TREM2	1.2045424	5.7156388	9.2202093	8.33E-19	9.71E-17	31.997583	54209
CD1C	1.2986545	2.530947	9.2091316	9.09E-19	1.05E-16	31.911121	911
GAL	-1.588343	1.5253965	-9.20176	9.64E-19	1.09E-16	31.853625	51083
HLA-DQA1	1.3504447	6.5064136	9.1413235	1.56E-18	1.70E-16	31.383483	3117
TNFSF10	1.1490523	6.6327807	9.1385851	1.59E-18	1.73E-16	31.362233	8743
ITGB8	1.4082698	2.5254659	9.0968774	2.21E-18	2.32E-16	31.039135	3696
CST6	1.9107421	5.0035738	9.0827037	2.48E-18	2.56E-16	30.929575	1474
CHML	-1.026303	3.9026102	-9.081709	2.50E-18	2.56E-16	30.921892	1122
CCL28	1.2431208	2.9122261	9.0718097	2.70E-18	2.74E-16	30.845449	56477
RAB38	1.0975698	4.7162535	9.0556932	3.06E-18	3.08E-16	30.721126	23682
HLA-DOA	1.2751083	5.3451186	9.0534055	3.12E-18	3.12E-16	30.703492	3111
PLAAT4	1.3020191	7.2268017	9.0269214	3.84E-18	3.77E-16	30.499573	5920

PDLIM4	1.2891635	3.9441654	9.0210543	4.02E-18	3.90E-16	30.454456	8572
TNFSF11	-1.106382	1.3890605	-9.020886	4.03E-18	3.90E-16	30.453161	8600
EVA1A	1.2056991	4.6340518	9.0115472	4.33E-18	4.15E-16	30.381393	84141
CGA	-1.596644	0.6381408	-9.005539	4.54E-18	4.33E-16	30.335249	1081
PLD4	1.0384223	2.4253603	8.9754137	5.75E-18	5.43E-16	30.104208	122618
CYP24A1	-2.297272	4.2332998	-8.964682	6.25E-18	5.88E-16	30.022041	1591
STK32A	1.1846892	2.9906223	8.9477915	7.14E-18	6.62E-16	29.892854	202374
PAH	-1.10155	0.6942248	-8.923972	8.59E-18	7.86E-16	29.710977	5053
COL8A2	1.1906349	3.964483	8.9232646	8.64E-18	7.87E-16	29.705579	1296
CHL1	-1.388986	2.6268958	-8.913339	9.33E-18	8.34E-16	29.629898	10752
HOXD1	1.2043941	1.4757812	8.9079779	9.73E-18	8.58E-16	29.589044	3231
HCK	1.0600367	4.8961202	8.8636098	1.37E-17	1.20E-15	29.251633	3055
CD1E	1.2936909	2.1366705	8.8632566	1.38E-17	1.20E-15	29.248952	913
KRT15	1.6421647	3.8090669	8.8574686	1.44E-17	1.24E-15	29.205027	3866
ALOX5AP	1.1777939	5.8759235	8.8466384	1.57E-17	1.32E-15	29.122892	241
MSMB	-2.861445	3.6865747	-8.832418	1.75E-17	1.45E-15	29.015158	4477
NPC2	1.1016241	8.5784903	8.8103894	2.07E-17	1.67E-15	28.848516	10577
IL37	2.3388134	2.5659907	8.8092687	2.09E-17	1.67E-15	28.840046	27178
COL21A1	1.1578317	2.2180189	8.8088125	2.10E-17	1.67E-15	28.836598	81578
LY6E	1.1810562	8.5705926	8.8016021	2.22E-17	1.76E-15	28.782126	4061
CD207	1.9317266	2.9084997	8.7597179	3.06E-17	2.34E-15	28.466347	50489
GLB1L3	1.7507645	1.7882614	8.7506847	3.28E-17	2.49E-15	28.398387	112937
ITGAM	1.0759401	3.9281513	8.7392141	3.59E-17	2.68E-15	28.312163	3684
PLA2G4A	-1.566353	5.0589269	-8.723823	4.03E-17	3.00E-15	28.196599	5321
TNIK	1.0074763	2.7366616	8.7090467	4.52E-17	3.32E-15	28.085793	23043
CX3CR1	1.0655822	2.0149963	8.7017057	4.78E-17	3.49E-15	28.030794	1524
ENO3	-1.330461	2.5152476	-8.6773	5.76E-17	4.16E-15	27.848189	2027
AMOT	1.3200612	3.0002243	8.6740651	5.90E-17	4.25E-15	27.824016	154796
IVL	1.9922724	2.002665	8.6676509	6.20E-17	4.40E-15	27.776101	3713
CPVL	1.1110219	4.7027931	8.6146845	9.28E-17	6.29E-15	27.381431	54504
EMB	1.0969325	5.5941022	8.5940053	1.09E-16	7.26E-15	27.227829	133418
NR4A2	-1.266469	4.1696556	-8.592088	1.10E-16	7.35E-15	27.213604	4929
PLAAT3	1.1623727	5.5448756	8.5813222	1.20E-16	7.92E-15	27.133755	11145
PITX2	-1.324481	1.4575676	-8.569744	1.30E-16	8.61E-15	27.047966	5308
ELFN2	1.2072095	2.0799775	8.5348722	1.70E-16	1.11E-14	26.790103	114794
ID1	-1.271902	6.6416179	-8.528894	1.78E-16	1.15E-14	26.745972	3397
CPLX2	-1.747402	0.8731584	-8.52257	1.86E-16	1.20E-14	26.699323	10814
CD1A	1.7375781	2.8689549	8.5214774	1.88E-16	1.20E-14	26.691262	909

GLS	1.1151901	5.4475924	8.5202808	1.90E-16	1.21E-14	26.682437	2744
HGD	-1.912223	3.3417697	-8.508607	2.07E-16	1.31E-14	26.596397	3081
TRIL	1.0396248	2.5075291	8.5036995	2.15E-16	1.35E-14	26.560255	9865
MET	1.6153621	6.1137587	8.4995904	2.22E-16	1.38E-14	26.530003	79811
PPP1R14C	1.4866963	4.1231553	8.4894299	2.39E-16	1.48E-14	26.455248	81706
TENM1	-1.088335	0.9088479	-8.457985	3.03E-16	1.80E-14	26.224313	10178
RIMKLB	-1.044349	3.4564764	-8.424598	3.89E-16	2.25E-14	25.979822	57494
BPIFA2	-1.686978	1.8817208	-8.397132	4.77E-16	2.74E-14	25.779232	140683
NPW	-1.648086	2.0462978	-8.388383	5.09E-16	2.89E-14	25.715439	283869
SLC7A11	-1.462177	2.9536181	-8.372109	5.75E-16	3.26E-14	25.596909	23657
AKR1C2	-2.905048	3.7536618	-8.358012	6.39E-16	3.58E-14	25.494372	1646
CSF1R	1.0021616	5.4123532	8.3561251	6.48E-16	3.61E-14	25.480661	1436
CHRNA9	-1.358673	0.889876	-8.350246	6.77E-16	3.74E-14	25.437943	55584
RAB7B	1.2200541	3.9826723	8.3443288	7.07E-16	3.89E-14	25.394975	338382
MAP7D2	1.3719399	2.3908973	8.3408205	7.26E-16	3.97E-14	25.36951	256714
ERVH48-1	-1.332035	0.7895679	-8.32255	8.31E-16	4.53E-14	25.237016	90625
WDR72	-1.41107	1.5781729	-8.308524	9.22E-16	4.98E-14	25.135461	256764
GABRE	1.2897622	2.6290115	8.2903698	1.05E-15	5.64E-14	25.004196	2564
GLTPD2	-1.127291	1.8814573	-8.283652	1.11E-15	5.88E-14	24.955677	388323
ANXA1	1.2972197	7.4698919	8.2107117	1.90E-15	9.42E-14	24.430804	301
ATP13A4	1.6239927	3.5981859	8.2088044	1.92E-15	9.53E-14	24.417126	84239
STAC	1.0510768	2.2032359	8.2077522	1.94E-15	9.58E-14	24.409582	6769
RSPO3	-1.145438	1.4767592	-8.170453	2.55E-15	1.22E-13	24.142613	84870
CABYR	-1.323054	2.2997234	-8.168744	2.58E-15	1.23E-13	24.130403	26256
S100B	1.3300345	3.49122	8.1287452	3.45E-15	1.60E-13	23.84519	6285
DNAJC12	-1.678701	4.0881887	-8.104439	4.12E-15	1.87E-13	23.672393	56521
MACC1	1.0747043	4.1473015	8.0774824	5.01E-15	2.24E-13	23.481222	346389
SUSD4	1.2007408	3.0893362	8.0744693	5.12E-15	2.27E-13	23.459883	55061
ABCA4	1.5192272	2.0708424	8.0696295	5.30E-15	2.35E-13	23.425622	24
ADORA1	1.2258571	2.3793333	8.0690446	5.32E-15	2.35E-13	23.421482	134
LRIG3	1.1039202	4.7185085	8.0616402	5.62E-15	2.46E-13	23.369098	121227
CDH3	1.5073417	5.2349839	8.0438156	6.39E-15	2.78E-13	23.243146	1001
WNT10A	1.1650335	2.2724238	8.0272512	7.20E-15	3.11E-13	23.12629	80326
PADI2	1.0101162	2.7093818	7.9952775	9.07E-15	3.86E-13	22.901252	11240
MMP28	1.6597155	3.7865114	7.9929386	9.22E-15	3.91E-13	22.884818	79148
TMEM63C	1.2674272	2.170919	7.9735782	1.06E-14	4.47E-13	22.748921	57156
B3GNT7	1.3336395	5.6907948	7.9501275	1.25E-14	5.23E-13	22.584656	93010
DACT2	-1.468383	2.0015681	-7.90719	1.70E-14	6.83E-13	22.284858	168002

MYO1G	1.1385089	3.608738	7.8711831	2.20E-14	8.66E-13	22.034424	64005
NNMT	-1.022935	7.2490961	-7.858191	2.42E-14	9.45E-13	21.944281	4837
ICAM5	1.1211607	2.680655	7.8128499	3.33E-14	1.28E-12	21.630592	7087
MISP	-1.090011	5.9091977	-7.801642	3.61E-14	1.37E-12	21.553272	126353
KIT	-1.442001	4.4726859	-7.789087	3.94E-14	1.48E-12	21.466755	3815
KCNN4	1.3766914	5.0756209	7.7853285	4.05E-14	1.51E-12	21.440878	3783
SLC14A2	-1.357029	0.9121521	-7.765271	4.66E-14	1.72E-12	21.302947	8170
DPP4	1.4477253	4.1678134	7.7511819	5.15E-14	1.88E-12	21.206222	1803
IFI44	1.1067371	5.4942983	7.7457658	5.35E-14	1.95E-12	21.169076	10561
FCER1A	1.4838165	3.0598015	7.7415191	5.51E-14	2.00E-12	21.139966	2205
CYBB	1.0890963	5.9136698	7.725727	6.15E-14	2.21E-12	21.031821	1536
OLR1	1.1721015	4.6242899	7.6989933	7.42E-14	2.62E-12	20.849143	4973
MMP7	1.7550915	5.8207599	7.6943178	7.67E-14	2.70E-12	20.817245	4316
TCIM	-1.277079	8.4341963	-7.691015	7.85E-14	2.76E-12	20.794724	56892
LRRN4	1.6205508	3.5212299	7.6837662	8.25E-14	2.89E-12	20.745314	164312
KLHDC7A	1.0906049	2.6093138	7.6479337	1.06E-13	3.62E-12	20.501623	127707
THPO	-1.052721	1.383169	-7.631297	1.19E-13	4.03E-12	20.388783	7066
IFIT1	1.1744008	4.2354289	7.6295611	1.20E-13	4.07E-12	20.377022	3434
CYP2S1	1.1890728	4.3209223	7.6249507	1.24E-13	4.19E-12	20.345792	29785
LDOC1	1.0977141	4.237877	7.6115318	1.36E-13	4.58E-12	20.254979	23641
RDH10	-1.090969	5.569466	-7.60943	1.38E-13	4.64E-12	20.240764	157506
AKR7A3	-1.719533	3.059459	-7.606855	1.41E-13	4.71E-12	20.223357	22977
MSLNL	-1.212956	1.3309092	-7.600816	1.47E-13	4.90E-12	20.182549	401827
AKAP12	-1.171774	3.5133204	-7.594246	1.54E-13	5.10E-12	20.13818	9590
KYNU	-1.152647	2.1453974	-7.562645	1.91E-13	6.25E-12	19.925207	8942
CSF2	1.1803128	2.3736789	7.5510614	2.07E-13	6.72E-12	19.847313	1437
SH3RF2	1.0974592	2.2473246	7.5300999	2.39E-13	7.60E-12	19.706601	153769
SLC15A2	1.0265468	3.4591755	7.5225551	2.52E-13	7.98E-12	19.65603	6565
CCL17	1.3690675	3.1670017	7.5137534	2.68E-13	8.41E-12	19.597084	6361
MAP1LC3C	1.0678297	2.2163488	7.5041414	2.86E-13	8.95E-12	19.532775	440738
MYBPHL	1.3593969	2.0270184	7.4754992	3.48E-13	1.07E-11	19.341532	343263
STUM	-1.17599	1.1796993	-7.46586	3.72E-13	1.13E-11	19.277303	375057
RASD1	-1.554596	6.3295564	-7.448773	4.18E-13	1.26E-11	19.163609	51655
MUC13	-2.220511	3.0926091	-7.446243	4.25E-13	1.28E-11	19.14679	56667
ABCC2	-1.49356	1.3748972	-7.445749	4.27E-13	1.28E-11	19.143507	1244
FNDC10	1.0281605	4.0597377	7.4193935	5.10E-13	1.49E-11	18.968607	643988
HAS3	1.3761737	3.8818555	7.4123331	5.35E-13	1.56E-11	18.921837	3038
AKR1C1	-2.196084	3.7861711	-7.401538	5.76E-13	1.66E-11	18.850394	1645

AKR1C3	-1.802546	5.3274447	-7.388698	6.29E-13	1.81E-11	18.765527	8644
CLDN9	1.4068951	2.995293	7.3867252	6.37E-13	1.83E-11	18.752501	9080
DCBLD2	1.0256096	4.6420672	7.3731129	6.99E-13	2.00E-11	18.662679	131566
PLAU	1.2824775	6.4502312	7.3593439	7.67E-13	2.17E-11	18.571957	5328
BMP4	1.1024507	3.5796713	7.2891877	1.23E-12	3.29E-11	18.111828	652
BRINP1	1.3921417	1.4888872	7.281472	1.29E-12	3.46E-11	18.06144	1620
APLP1	1.1599754	2.7936008	7.2487071	1.61E-12	4.25E-11	17.847946	333
IFI44L	1.1306581	3.2926651	7.2446819	1.66E-12	4.34E-11	17.821772	10964
IGFBP1	-1.092448	0.8065165	-7.229627	1.83E-12	4.75E-11	17.723982	3484
MUC5AC	-1.988361	1.8180501	-7.215186	2.02E-12	5.19E-11	17.630332	4586
MUC5B	-2.298162	4.3090434	-7.214165	2.03E-12	5.21E-11	17.623716	727897
CDKL2	1.0670308	2.9640405	7.2034857	2.18E-12	5.54E-11	17.554564	8999
CRABP2	1.8173009	7.7679732	7.1815076	2.52E-12	6.32E-11	17.412514	1382
MUC21	2.0093408	4.4355211	7.1723777	2.68E-12	6.67E-11	17.353608	394263
BMP3	1.3747795	3.0823494	7.1551009	3.00E-12	7.43E-11	17.242306	651
PTPN13	1.1690063	4.0300062	7.1239068	3.69E-12	8.96E-11	17.041897	5783
SHISA3	1.7535393	2.5859368	7.1235105	3.70E-12	8.97E-11	17.039356	152573
TMEM130	1.2194836	2.7449969	7.1122153	3.98E-12	9.61E-11	16.966967	222865
BPIFA1	-2.952519	5.4236978	-7.104427	4.19E-12	1.00E-10	16.917109	51297
RASGRF1	1.0268569	1.8370826	7.0952939	4.45E-12	1.06E-10	16.858697	5923
AMIGO2	1.0705871	5.3518838	7.0824814	4.84E-12	1.14E-10	16.776857	347902
CTSH	1.0389011	6.4448619	7.0225738	7.17E-12	1.64E-10	16.3958	1512
TP63	1.1513898	1.8567627	7.022028	7.19E-12	1.65E-10	16.392341	8626
RND1	-1.160948	4.1938436	-6.998508	8.38E-12	1.89E-10	16.243469	27289
SLC7A5	-1.006342	5.8937471	-6.985669	9.11E-12	2.04E-10	16.162375	8140
CLDN1	1.1819354	5.6345757	6.9776264	9.60E-12	2.14E-10	16.111641	9076
HMGB3	-1.06253	7.3484263	-6.967448	1.03E-11	2.28E-10	16.047499	3149
PLAT	1.3041531	4.8517113	6.9466933	1.17E-11	2.56E-10	15.916951	5327
ZNF486	1.1960654	3.3130604	6.8459633	2.24E-11	4.58E-10	15.287902	90649
RNASE1	1.0938079	10.054749	6.8099117	2.82E-11	5.67E-10	15.064607	6035
SLC1A7	1.6115821	2.3899994	6.8022216	2.96E-11	5.92E-10	15.017103	6512
SPTB	1.1607885	2.0675134	6.7866446	3.27E-11	6.47E-10	14.921015	6710
ROS1	1.339951	4.1731752	6.7752374	3.51E-11	6.88E-10	14.850765	6098
ECM1	1.1309576	5.369428	6.7670042	3.70E-11	7.19E-10	14.800123	1893
AGR2	-1.347127	8.5890746	-6.760034	3.86E-11	7.48E-10	14.757286	10551
SUSD2	1.5636099	5.4924381	6.7454148	4.24E-11	8.12E-10	14.667569	56241
RRAD	1.2344513	4.946206	6.7230655	4.88E-11	9.22E-10	14.530721	6236
UPK3A	1.0267565	1.6966659	6.6949931	5.82E-11	1.09E-09	14.359365	7380

NMNAT2	1.2179288	2.2404214	6.6824609	6.30E-11	1.17E-09	14.283061	23057
IRX2	1.49938	3.8410698	6.6631709	7.11E-11	1.29E-09	14.165843	153572
TPSB2	1.2081706	4.2980024	6.6627561	7.12E-11	1.30E-09	14.163326	64499
AMBP	-1.379202	2.0508004	-6.658162	7.33E-11	1.33E-09	14.135453	259
KCNMB4	-1.010501	2.8928536	-6.655725	7.44E-11	1.35E-09	14.120674	27345
C5orf38	1.2469092	3.034197	6.6514606	7.64E-11	1.38E-09	14.094822	153571
CDH17	-1.364894	1.6060775	-6.641373	8.14E-11	1.46E-09	14.033725	1015
C1orf116	1.263652	6.3959907	6.6391087	8.26E-11	1.48E-09	14.020023	79098
ADGRF5	1.1647454	6.2048596	6.6222199	9.17E-11	1.63E-09	13.917939	221395
CCL13	1.2392062	4.8692602	6.6149619	9.60E-11	1.70E-09	13.874135	6357
DDIT4L	-1.111189	2.8909872	-6.589689	1.12E-10	1.96E-09	13.721919	115265
CT83	-1.86954	2.6772684	-6.577928	1.21E-10	2.09E-09	13.65125	203413
S100A2	1.3644503	4.8785275	6.5765832	1.22E-10	2.10E-09	13.643177	6273
TPSAB1	1.0682907	4.3322228	6.5636939	1.32E-10	2.26E-09	13.565863	7177
CHIT1	1.4121471	3.7620609	6.555728	1.38E-10	2.35E-09	13.518146	1118
HLA-DQA2	1.4316132	5.9900054	6.5516069	1.42E-10	2.40E-09	13.493478	3118
RET	-1.067157	1.3975457	-6.545907	1.47E-10	2.47E-09	13.459384	5979
CXCL14	1.8785525	5.7119319	6.542484	1.50E-10	2.52E-09	13.438918	9547
ITGA2	1.0891756	4.8978572	6.5298869	1.62E-10	2.71E-09	13.363686	3673
PCSK9	-1.127871	2.1174929	-6.502944	1.92E-10	3.16E-09	13.203186	255738
FST	-1.101918	2.4849703	-6.496972	1.99E-10	3.27E-09	13.16769	10468
VSIG1	1.711171	3.0339812	6.4859125	2.13E-10	3.46E-09	13.102019	340547
BCAS1	-1.212453	2.5437523	-6.484303	2.15E-10	3.50E-09	13.09247	8537
SLC34A2	1.458069	9.4387751	6.4779343	2.23E-10	3.62E-09	13.054704	10568
HLF	1.0715754	2.7324169	6.4760972	2.26E-10	3.66E-09	13.043816	3131
PIGR	1.9943806	7.3846794	6.4725488	2.31E-10	3.73E-09	13.022793	5284
SPINK4	-1.188098	1.108696	-6.471213	2.33E-10	3.76E-09	13.014884	27290
HHLA2	1.7129632	2.4037593	6.4640934	2.43E-10	3.91E-09	12.972738	11148
SLC16A9	1.3095406	3.1315313	6.4108482	3.36E-10	5.20E-09	12.6588	220963
CPA3	1.1658812	4.7163097	6.3887786	3.84E-10	5.87E-09	12.529319	1359
TNC	1.2758782	5.8200432	6.3823932	3.99E-10	6.08E-09	12.491927	3371
GPX2	-2.324262	4.7270139	-6.381645	4.01E-10	6.09E-09	12.487548	2877
LYZ	1.1974031	8.4957472	6.3494483	4.87E-10	7.29E-09	12.299511	4069
MRC1	1.0920513	5.1038219	6.3455758	4.98E-10	7.45E-09	12.276949	4360
CA12	-1.299532	3.3737886	-6.341991	5.09E-10	7.59E-09	12.256072	771
TMPRSS2	1.0603998	5.2711829	6.3178088	5.88E-10	8.65E-09	12.115515	7113
NT5E	1.1324102	4.9852162	6.3150157	5.98E-10	8.78E-09	12.099309	4907
PNMA8A	1.0939245	2.887058	6.2810436	7.33E-10	1.05E-08	11.902693	55228

HHIPL2	-1.272169	1.9130976	-6.219381	1.06E-09	1.47E-08	11.548124	79802
MYBPH	1.0812648	1.5725148	6.2067859	1.14E-09	1.56E-08	11.47607	4608
KCNE4	-1.019603	3.2031613	-6.181336	1.32E-09	1.79E-08	11.330855	23704
HAVCR1	-1.02528	1.4990616	-6.175058	1.37E-09	1.85E-08	11.295109	26762
AIM2	1.1133524	3.2759795	6.1564408	1.53E-09	2.04E-08	11.189298	9447
PRRX2	1.0255019	3.159925	6.146108	1.63E-09	2.16E-08	11.130689	51450
F13A1	1.0321687	4.6400143	6.138626	1.70E-09	2.25E-08	11.088303	2162
FAM83A	-1.255447	5.1778305	-6.092295	2.23E-09	2.88E-08	10.826818	84985
PAEP	-2.13891	3.609624	-5.999236	3.81E-09	4.71E-08	10.306769	5047
CHI3L1	1.1570449	6.3601142	5.976568	4.34E-09	5.27E-08	10.18114	1116
LBP	-1.099251	0.9808984	-5.932514	5.58E-09	6.61E-08	9.9381603	3929
EPS8L3	-1.103846	0.9485706	-5.916531	6.11E-09	7.17E-08	9.8503919	79574
TSPAN11	-1.014175	2.7873163	-5.911935	6.28E-09	7.33E-08	9.8251948	441631
CACNG4	1.213256	2.7074931	5.903804	6.57E-09	7.61E-08	9.7806508	27092
DEFB1	-1.541127	3.7702534	-5.897715	6.80E-09	7.84E-08	9.7473298	1672
ANPEP	1.0255231	4.2151732	5.8387126	9.49E-09	1.07E-07	9.4260026	290
MARCO	1.2314237	5.4962139	5.7921951	1.23E-08	1.34E-07	9.1746522	8685
MMP13	1.4488709	3.1939574	5.7652551	1.43E-08	1.53E-07	9.0298881	4322
SLC5A1	1.0452631	1.7644486	5.7154653	1.88E-08	1.97E-07	8.7638925	6523
DDC	-1.134663	1.6008636	-5.713339	1.91E-08	1.99E-07	8.7525791	1644
GGTLC1	1.4339055	3.7919679	5.6920178	2.14E-08	2.22E-07	8.6393271	92086
ASCL1	-1.618595	1.4579909	-5.654442	2.64E-08	2.68E-07	8.4406443	429
AQP1	1.143447	7.3774724	5.6366611	2.90E-08	2.92E-07	8.347027	358
RHOV	-1.097579	4.6973259	-5.543242	4.82E-08	4.60E-07	7.8594475	171177
SERPIND1	1.2795575	2.4750053	5.533594	5.07E-08	4.83E-07	7.8095016	3053
CCL19	1.1107296	5.7007796	5.5037703	5.95E-08	5.56E-07	7.6555959	6363
KLK12	-1.464332	1.6089036	-5.501658	6.02E-08	5.62E-07	7.6447238	43849
KLK14	-1.160348	1.4749527	-5.49592	6.21E-08	5.78E-07	7.6152086	43847
RETN	1.0102793	2.6414921	5.4868154	6.52E-08	6.05E-07	7.5684276	56729
LEMD1	1.0081805	2.6114674	5.4342054	8.62E-08	7.77E-07	7.2994668	93273
CLDN10	-1.285385	2.7862166	-5.420296	9.28E-08	8.30E-07	7.2287411	9071
TSPAN8	-1.399444	5.0734211	-5.387274	1.10E-07	9.69E-07	7.0614779	7103
MMP11	1.1157076	5.2180002	5.3340857	1.46E-07	1.24E-06	6.7939802	4320
SCTR	1.1286417	3.2636793	5.2349044	2.44E-07	1.95E-06	6.3014976	6344
NR0B1	-1.114391	0.9815352	-5.215699	2.69E-07	2.14E-06	6.2070903	190
COL17A1	1.2399874	3.2505859	5.1996845	2.92E-07	2.30E-06	6.1286032	1308
SCGB3A1	1.8636657	6.9754497	5.1637512	3.50E-07	2.72E-06	5.9532853	92304
BPIFB2	-1.386209	1.9840587	-5.150179	3.75E-07	2.89E-06	5.8873509	80341

VTCN1	1.0382597	2.3481233	5.1118348	4.55E-07	3.43E-06	5.7019144	79679
MUC4	-1.035582	3.5018442	-4.9965	8.09E-07	5.78E-06	5.1516735	4585
KDM5D	-1.042617	1.6543439	-4.969625	9.24E-07	6.51E-06	5.0250855	8284
FGFBP1	1.1152906	3.2244624	4.9063579	1.26E-06	8.58E-06	4.7295257	9982
PEG10	1.2058989	4.3193414	4.8673098	1.52E-06	1.02E-05	4.5488218	23089
CRLF1	-1.47761	4.6059689	-4.817289	1.93E-06	1.26E-05	4.319257	9244
SPRR2D	1.0066506	1.2879409	4.8105982	2.00E-06	1.30E-05	4.2887142	6703
PGC	-2.031677	5.8684689	-4.776394	2.35E-06	1.50E-05	4.1331806	5225
PI3	-1.130439	3.2117782	-4.759952	2.54E-06	1.61E-05	4.058774	5266
SPINK1	-1.698262	5.7512267	-4.738213	2.82E-06	1.76E-05	3.9607571	6690
HPGD	1.0591578	4.8567969	4.7072262	3.26E-06	2.01E-05	3.8217492	3248
FOXQ1	1.0705851	4.5465415	4.7063593	3.27E-06	2.02E-05	3.8178725	94234
MSLN	-1.648607	6.8405161	-4.680937	3.68E-06	2.25E-05	3.7044693	10232
RPS4Y1	-1.922666	3.6117918	-4.63217	4.62E-06	2.76E-05	3.4885066	6192
UCHL1	-1.163236	4.8928139	-4.526873	7.49E-06	4.26E-05	3.0292886	7345
SPRR1B	1.2649326	2.2765783	4.462307	1.00E-05	5.53E-05	2.7525114	6699
MS4A15	1.1267424	2.7395644	4.454891	1.04E-05	5.68E-05	2.7209559	219995
DDX3Y	-1.111928	2.0697019	-4.404445	1.30E-05	6.93E-05	2.5075923	8653
PCSK2	-1.08235	1.2682113	-4.38803	1.40E-05	7.41E-05	2.4386439	5126
ORM1	-1.001936	3.5372023	-4.369271	1.52E-05	7.97E-05	2.3601473	5004
PCP4L1	1.0763664	4.3316848	4.3630232	1.56E-05	8.17E-05	2.3340733	654790
TNNT1	1.1088348	3.6241346	4.3487845	1.66E-05	8.63E-05	2.2747775	7138
PPP1R1B	1.0847503	4.2189625	4.3178518	1.90E-05	9.75E-05	2.1465798	84152
TNNC2	1.0360353	3.212264	4.3129944	1.94E-05	9.93E-05	2.1265259	7125
GKN2	-1.05322	2.3547791	-4.242328	2.64E-05	0.0001306	1.8371493	200504
NAPSA	1.1759444	8.3252799	4.2140148	2.98E-05	0.0001453	1.722452	9476
AOC1	-1.075336	3.5104127	-4.19868	3.18E-05	0.0001542	1.6606296	26
TFF3	-1.282264	5.932937	-4.165289	3.66E-05	0.0001747	1.5267398	7033
AKR1B10	-1.409805	3.0768433	-4.018438	6.76E-05	0.0003005	0.9497603	57016
ALDH3A1	-1.08606	2.8746566	-3.963099	8.48E-05	0.0003672	0.7373629	218
AZGP1	1.1157566	4.5418425	3.7638841	0.0001872	0.0007344	-0.004306	563
SCGB1A1	1.3675162	4.4248902	3.6894113	0.0002495	0.000943	-0.272296	7356
CTSE	1.0535559	6.3050294	3.497311	0.000512	0.0017685	-0.940116	1510
SFTPC	1.0218814	6.4974246	2.3146705	0.0210361	0.0451618	-4.292342	6440

Supplementary Table S 3

GSEA_results	setSize	enrichmer	NES	pvalue	p.adjust	qvalue	rank	leading_edge
KEGG_ASTHMA	28	0.897401	2.248701	1.00E-10	3.72E-09	2.46E-09	1341	tags=68%, list=7%, signal=63%
KEGG_ALLOGRAFT_REJECTION	35	0.854339	2.211044	1.00E-10	3.72E-09	2.46E-09	1575	tags=71%, list=8%, signal=66%
KEGG_AUTOIMMUNE_THYROID_DISEASE	50	0.793725	2.18361	1.00E-10	3.72E-09	2.46E-09	1575	tags=48%, list=8%, signal=44%
KEGG_GRAFT_VERSUS_HOST_DISEASE	37	0.820447	2.153453	1.61E-10	4.29E-09	2.84E-09	1117	tags=62%, list=6%, signal=59%
KEGG_CELL_ADHESION_MOLECULES_CAMS	130	0.689865	2.112684	1.00E-10	3.72E-09	2.46E-09	2860	tags=55%, list=15%, signal=47%
KEGG_INTESTINAL_IMMUNE_NETWORK_FOR_IGA_PRODUCTION	45	0.780406	2.109621	3.06E-10	6.32E-09	4.19E-09	1622	tags=56%, list=9%, signal=51%
KEGG_VIRAL_MYOCARDITIS	68	0.717954	2.054882	1.21E-10	3.74E-09	2.48E-09	2323	tags=50%, list=12%, signal=44%
KEGG_LEISHMANIA_INFECTION	69	0.712403	2.037094	2.30E-10	5.36E-09	3.55E-09	2258	tags=51%, list=12%, signal=45%
KEGG_TYPE_I_DIABETES_MELLITUS	41	0.727199	1.947329	9.93E-07	1.09E-05	7.20E-06	1117	tags=56%, list=6%, signal=53%
KEGG_ANTIGEN_PROCESSING_AND_PRESENTATION	80	0.659634	1.933332	2.64E-08	4.92E-07	3.26E-07	2025	tags=32%, list=11%, signal=29%
KEGG_HEMATOPOIETIC_CELL_LINEAGE	84	0.647985	1.902322	9.57E-08	1.37E-06	9.07E-07	3051	tags=58%, list=16%, signal=49%
KEGG_NATURAL_KILLER_CELL_MEDIATED_CYTOTOXICITY	131	0.5956	1.825636	5.41E-08	9.15E-07	6.06E-07	4426	tags=49%, list=23%, signal=38%
KEGG_CYTOKINE_CYTOKINE_RECEPTOR_INTERACTION	261	0.561019	1.815656	1.00E-10	3.72E-09	2.46E-09	3993	tags=46%, list=21%, signal=37%
KEGG_B_CELL_RECEPTOR_SIGNALING_PATHWAY	75	0.616474	1.79091	5.05E-06	5.22E-05	3.45E-05	4548	tags=59%, list=24%, signal=45%
KEGG_LEUKOCYTE_TRANSENDOTHELIAL_MIGRATION	114	0.584637	1.782648	3.23E-07	4.01E-06	2.65E-06	4684	tags=55%, list=25%, signal=42%
KEGG_DILATED_CARDIOMYOPATHY	90	0.592427	1.757384	5.74E-06	5.62E-05	3.72E-05	3549	tags=40%, list=19%, signal=33%
KEGG_CHEMOKINE_SIGNALING_PATHWAY	186	0.550126	1.744231	6.18E-08	9.59E-07	6.35E-07	4169	tags=46%, list=22%, signal=36%
KEGG_PRIMARY_IMMUNODEFICIENCY	35	0.668565	1.730256	0.000383	0.002377	0.001574	3697	tags=60%, list=19%, signal=48%
KEGG_FOCAL_ADHESION	198	0.531404	1.692718	3.47E-07	4.03E-06	2.67E-06	4259	tags=44%, list=22%, signal=35%
KEGG_ECM_RECEPTOR_INTERACTION	83	0.569663	1.668947	0.000116	0.000798	0.000528	4737	tags=54%, list=25%, signal=41%
KEGG_TOLL_LIKE_RECEPTOR_SIGNALING_PATHWAY	102	0.55234	1.661874	3.41E-05	0.000264	0.000175	4636	tags=47%, list=24%, signal=36%
KEGG_SYSTEMIC_LUPUS_ERYTHEMATOSUS	131	0.538461	1.650493	2.53E-05	0.000205	0.000136	1575	tags=22%, list=8%, signal=20%
KEGG_JAK_STAT_SIGNALING_PATHWAY	155	0.519326	1.620206	2.30E-05	0.000194	0.000129	4764	tags=43%, list=25%, signal=32%
KEGG_T_CELL_RECEPTOR_SIGNALING_PATHWAY	108	0.535457	1.619996	0.000107	0.000767	0.000508	5148	tags=56%, list=27%, signal=41%
KEGG_HYPERTROPHIC_CARDIOMYOPATHY_HCM	83	0.551219	1.614911	0.000483	0.002807	0.001859	3549	tags=40%, list=19%, signal=32%
KEGG_CYTOSOLIC_DNA_SENSING_PATHWAY	53	0.558608	1.548942	0.005244	0.021674	0.014351	4237	tags=34%, list=22%, signal=26%
KEGG_PANCREATIC_CANCER	70	0.537999	1.543703	0.002876	0.013049	0.00864	4548	tags=47%, list=24%, signal=36%
KEGG_REGULATION_OF_ACTIN_CYTOSKELETON	211	0.483195	1.541591	5.08E-05	0.000378	0.00025	5148	tags=45%, list=27%, signal=34%
KEGG_LYSOSOME	120	0.500022	1.531718	0.00124	0.006588	0.004362	5878	tags=57%, list=31%, signal=39%
KEGG_FC_EPSILON_RI_SIGNALING_PATHWAY	79	0.514678	1.502658	0.003749	0.016601	0.010992	4636	tags=48%, list=24%, signal=37%
KEGG_NOD_LIKE_RECEPTOR_SIGNALING_PATHWAY	62	0.526754	1.491385	0.010002	0.037209	0.024637	6147	tags=66%, list=32%, signal=45%
KEGG_FC_GAMMA_R_MEDIATED_PHAGOCYTOSIS	95	0.499895	1.489751	0.004013	0.017359	0.011494	4548	tags=43%, list=24%, signal=33%
KEGG_ARRHYTHMOGENIC_RIGHT_VENTRICULAR_CARDIOMYOPATHY_ARVC	74	0.511013	1.48338	0.008747	0.033893	0.022442	4737	tags=47%, list=25%, signal=36%
KEGG_NEUROTROPHIN_SIGNALING_PATHWAY	126	0.48229	1.472834	0.002358	0.011539	0.007641	4985	tags=49%, list=26%, signal=37%
KEGG_NON_SMALL_CELL_LUNG_CANCER	54	0.527988	1.468942	0.011212	0.040105	0.026555	4457	tags=44%, list=23%, signal=34%
KEGG_PHOSPHATIDYLINOSITOL_SIGNALING_SYSTEM	76	0.501684	1.459674	0.013714	0.047237	0.031278	4938	tags=49%, list=26%, signal=36%
KEGG_AXON_GUIDANCE	129	0.475787	1.45629	0.002758	0.012822	0.00849	5148	tags=52%, list=27%, signal=38%
KEGG_APOPTOSIS	87	0.48097	1.42052	0.014268	0.048252	0.031949	5872	tags=54%, list=31%, signal=37%
KEGG_MAPK_SIGNALING_PATHWAY	267	0.407841	1.320688	0.010664	0.038894	0.025753	5196	tags=43%, list=27%, signal=32%
KEGG_HUNTINGTONS_DISEASE	172	-0.33728	-1.34209	0.005626	0.022264	0.014742	2726	tags=37%, list=14%, signal=32%
KEGG_GLUTATHIONE_METABOLISM	48	-0.48788	-1.69222	0.013638	0.047237	0.031278	1111	tags=33%, list=6%, signal=31%
KEGG_PROTEIN_EXPORT	23	-0.59875	-1.69648	0.009633	0.036566	0.024212	3691	tags=61%, list=19%, signal=49%

KEGG_ASCORBATE_AND_ALDARATE_METABOLISM	25	-0.62098	-1.80903	0.004468	0.018888	0.012507	2285	tags=36%, list=12%, signal=32%
KEGG_COMPLEMENT_AND_COAGULATION_CASCADES	69	-0.50737	-1.83329	0.001022	0.005592	0.003703	791	tags=25%, list=4%, signal=24%
KEGG_HISTIDINE_METABOLISM	29	-0.61476	-1.87707	0.005458	0.022069	0.014613	580	tags=34%, list=3%, signal=33%
KEGG_STEROID_HORMONE_BIOSYNTHESIS	55	-0.54393	-1.87947	0.001374	0.0071	0.004701	1276	tags=18%, list=7%, signal=17%
KEGG_ALANINE_ASPARTATE_AND_GLUTAMATE_METABOLISM	32	-0.61976	-1.91801	0.002735	0.012822	0.00849	524	tags=16%, list=3%, signal=15%
KEGG_MATURITY_ONSET_DIABETES_OF_THE_YOUNG	25	-0.65929	-1.92064	0.001832	0.009207	0.006096	493	tags=24%, list=3%, signal=23%
KEGG_PARKINSONS_DISEASE	113	-0.50383	-1.9375	1.33E-05	0.000124	8.18E-05	2726	tags=49%, list=14%, signal=42%
KEGG_OXIDATIVE_PHOSPHORYLATION	116	-0.49956	-1.94226	1.40E-05	0.000124	8.23E-05	2726	tags=48%, list=14%, signal=42%
KEGG_METABOLISM_OF_XENOBIOTICS_BY_CYTOCHROME_P450	68	-0.543	-1.94984	0.000226	0.001448	0.000959	1663	tags=31%, list=9%, signal=28%
KEGG_NITROGEN_METABOLISM	23	-0.70049	-1.98474	0.000573	0.00323	0.002139	324	tags=22%, list=2%, signal=21%
KEGG_CITRATE_CYCLE_TCA_CYCLE	30	-0.67156	-2.03114	0.000428	0.002565	0.001698	2700	tags=53%, list=14%, signal=46%
KEGG_ARGININE_AND_PROLINE_METABOLISM	54	-0.60507	-2.07618	0.000132	0.000878	0.000581	753	tags=26%, list=4%, signal=25%
KEGG_RIBOSOME	87	-0.6089	-2.25414	2.78E-07	3.69E-06	2.44E-06	3291	tags=49%, list=17%, signal=41%

Supplementary Table S 4

GSEA_results	setSize	enrichmer	NES	pvalue	p.adjust	qvalue	rank
REACTOME_IMMUNOREGULATORY_INTERACTIONS_BETWEEN_A_LYMPHOID_AND_A_NON_LYMPHOID_CELL	127	0.668699	2.053341	1.00E-10	4.26E-08	3.61E-08	3291
REACTOME_INTERFERON_GAMMA_SIGNALING	90	0.669736	1.993258	6.51E-10	2.08E-07	1.76E-07	3487
REACTOME_PD_1_SIGNALING	21	0.816022	1.950849	2.85E-06	0.000158	0.000134	2802
REACTOME_INTERFERON_ALPHA_BETA_SIGNALING	72	0.665422	1.921245	5.20E-08	8.32E-06	7.05E-06	3805
REACTOME_GENERATION_OF_SECOND_MESSENGER_MOLECULES	32	0.737777	1.896468	4.56E-06	0.000224	0.00019	2802
REACTOME_INTERFERON_SIGNALING	199	0.595812	1.893036	1.00E-10	4.26E-08	3.61E-08	3526
REACTOME_ANTIGEN_ACTIVATES_B_CELL_RECEPTOR_BCR_LEADING_TO_GENERATION_OF_SECOND_MESSENGERS	30	0.737573	1.877163	6.11E-06	0.000279	0.000236	4426
REACTOME_COSTIMULATION_BY_THE_CD28_FAMILY	67	0.651486	1.858825	5.83E-07	4.97E-05	4.21E-05	3293
REACTOME_INTERLEUKIN_2_FAMILY_SIGNALING	44	0.681355	1.840086	1.26E-05	0.000521	0.000442	4426
REACTOME_SURFACTANT_METABOLISM	30	0.714553	1.818577	3.87E-05	0.001413	0.001197	2019
REACTOME_INFLAMMASOMES	21	0.74369	1.777927	0.000271	0.006193	0.005245	3340
REACTOME_MOLECULES_ASSOCIATED_WITH_ELASTIC_FIBRES	37	0.675327	1.775525	0.000111	0.003372	0.002856	2942
REACTOME_PURINERGIC_SIGNALING_IN_LEISHMANIASIS_INFECTION	26	0.711448	1.762547	0.000156	0.004355	0.003688	2955
REACTOME_INTERLEUKIN_3_INTERLEUKIN_5_AND_GM_CSF_SIGNALING	48	0.642292	1.75287	6.10E-05	0.002052	0.001738	4426
REACTOME_INTERLEUKIN_RECEPTOR_SHC_SIGNALING	27	0.700954	1.750476	0.000202	0.005381	0.004557	4426
REACTOME_MHC_CLASS_II_ANTIGEN_PRESENTATION	124	0.56783	1.736422	2.36E-06	0.000144	0.000122	3479
REACTOME_FCGR_ACTIVATION	12	0.823024	1.736129	0.000445	0.008623	0.007302	2802
REACTOME_FCERI_MEDIATED_CA_2_MOBILIZATION	31	0.676371	1.729644	0.00038	0.007587	0.006426	4340
REACTOME_DISEASES_ASSOCIATED_WITH_SURFACTANT_METABOLISM	10	0.835271	1.696639	0.00037	0.007514	0.006363	1634
REACTOME_CHEMOKINE_RECEPTORS_BIND_CHEMOKINES	56	0.604449	1.694874	0.000315	0.006609	0.005597	2797
REACTOME_DAP12_INTERACTIONS	39	0.639813	1.693138	0.000289	0.006409	0.005428	4568
REACTOME_TCR_SIGNALING	117	0.552914	1.684374	1.06E-05	0.000466	0.000395	4340
REACTOME_ROLE_OF_LAT2_NTAL_LAB_ON_CALCIUM_MOBILIZATION	16	0.739769	1.682165	0.001267	0.019766	0.01674	4169
REACTOME_ELASTIC_FIBRE_FORMATION	44	0.619391	1.672743	0.001149	0.018369	0.015556	2942
REACTOME_ASSEMBLY_OF_COLLAGEN_FIBRILS_AND_OTHER_MULTIMERIC_STRUCTURES	61	0.59306	1.667398	0.00022	0.005621	0.004761	3748
REACTOME_ENDOSOMAL_VACUOLAR_PATHWAY	11	0.803077	1.663054	0.001761	0.02531	0.021434	2597
REACTOME_THE_NLRP3_INFLAMMASOME	16	0.72073	1.638872	0.002593	0.032835	0.027808	3340
REACTOME_THE_ROLE_OF_NEF_IN_HIV_1_REPLICATION_AND_DISEASE_PATHOGENESIS	28	0.652945	1.638168	0.002091	0.028449	0.024093	3281
REACTOME_OTHER_SEMAPHORIN_INTERACTIONS	19	0.694826	1.633175	0.003804	0.043437	0.036786	2919
REACTOME_GPVI_MEDIATED_ACTIVATION_CASCADE	35	0.625826	1.626537	0.001666	0.024214	0.020506	4886
REACTOME_DAP12_SIGNALING	29	0.640581	1.622744	0.002351	0.031001	0.026254	4548
REACTOME_FCGAMMA_RECEPTOR_FCGR_DEPENDENT_PHAGOCYTOSIS	86	0.548788	1.621225	0.000494	0.009324	0.007896	4581
REACTOME_SYNDECAN_INTERACTIONS	27	0.647695	1.617475	0.002802	0.034135	0.028908	4259
REACTOME_SIGNAL_REGULATORY_PROTEIN_FAMILY_INTERACTIONS	16	0.710379	1.615334	0.003786	0.043437	0.036786	4568
REACTOME_INTERLEUKIN_10_SIGNALING	45	0.593858	1.604089	0.00184	0.026155	0.02215	2978
REACTOME_A_TETRASACCHARIDE_LINKER_SEQUENCE_IS_REQUIRED_FOR_GAG_SYNTHESIS	26	0.641412	1.589038	0.003533	0.041074	0.034784	2763
REACTOME_ECM_PROTEOGLYCANS	76	0.540678	1.577371	0.002083	0.028449	0.024093	2301
REACTOME_REGULATION_OF_IFNA_IFNB_SIGNALING	26	0.635755	1.575023	0.004298	0.047544	0.040264	4426
REACTOME_SIGNALING_BY_VEGF	106	0.520335	1.570427	0.000442	0.008623	0.007302	5148
REACTOME_RAC2_GTPASE_CYCLE	88	0.528445	1.567327	0.00099	0.016443	0.013925	5400
REACTOME_CELL_CELL_COMMUNICATION	128	0.506458	1.557057	0.00034	0.00701	0.005936	4169
REACTOME_CELL_SURFACE_INTERACTIONS_AT_THE_VASCULAR_WALL	137	0.495959	1.539018	0.000619	0.011002	0.009317	4259
REACTOME_RAC1_GTPASE_CYCLE	184	0.485844	1.537659	0.000157	0.004355	0.003688	5430
REACTOME_COLLAGEN_FORMATION	90	0.515566	1.534418	0.002144	0.028861	0.024442	3748
REACTOME_SIGNALING_BY_THE_B_CELL_RECEPTOR_BCR	110	0.503716	1.527452	0.002476	0.031984	0.027087	4426
REACTOME_CDC42_GTPASE_CYCLE	155	0.486018	1.517006	0.000496	0.009324	0.007896	5192
REACTOME_RAC3_GTPASE_CYCLE	94	0.500826	1.496639	0.004371	0.047787	0.04047	5345

REACTOME_EXTRACELLULAR_MATRIX_ORGANIZATION	299	0.458218	1.494914	0.000102	0.003184	0.002697	3879
REACTOME_NEUTROPHIL_DEGRANULATION	477	0.448136	1.487975	1.46E-06	9.86E-05	8.35E-05	3561
REACTOME_SIGNALING_BY_INTERLEUKINS	469	0.441131	1.464693	4.19E-06	0.000214	0.000182	5208
REACTOME_RHOA_GTPASE_CYCLE	149	0.467047	1.45487	0.002781	0.034135	0.028908	4886
REACTOME_DEGRADATION_OF_THE_EXTRACELLULAR_MATRIX	140	0.468373	1.454428	0.003408	0.04036	0.03418	3879
REACTOME_GLYCOSAMINOGLYCAN_METABOLISM	124	0.475034	1.45265	0.003496	0.041019	0.034738	2564
REACTOME_ESTROGEN_DEPENDENT_GENE_EXPRESSION	147	-0.36046	-1.44874	0.004312	0.047544	0.040264	2795
REACTOME_CELL_CYCLE_CHECKPOINTS	288	-0.34034	-1.49714	0.00015	0.004355	0.003688	3404
REACTOME_INFLUENZA_INFECTION	157	-0.37604	-1.52593	0.001862	0.026167	0.02216	3291
REACTOME_EPIGENETIC_REGULATION_OF_GENE_EXPRESSION	145	-0.38864	-1.54089	0.002446	0.031918	0.027031	3529
REACTOME_MITOTIC_PROPHASE	140	-0.39911	-1.57031	0.001448	0.021787	0.018451	3529
REACTOME_DNA_REPLICATION_PRE_INITIATION	157	-0.39125	-1.58764	0.000663	0.011612	0.009834	3331
REACTOME_DNA_REPLICATION	185	-0.38697	-1.59634	0.00023	0.005758	0.004877	3331
REACTOME_GENE_SILENCING_BY_RNA	136	-0.40991	-1.59797	0.000709	0.012254	0.010377	3529
REACTOME_ANTIMICROBIAL_PEPTIDES	91	-0.43087	-1.61683	0.0041	0.046412	0.039305	704
REACTOME_MITOTIC_SPINDLE_CHECKPOINT	111	-0.4179	-1.61804	0.002709	0.033642	0.028491	3161
REACTOME_TELOMERE_MAINTENANCE	110	-0.42283	-1.63021	0.002283	0.030421	0.025763	2875
REACTOME_NONSENSE_MEDIATED_DECAY_NMD	116	-0.43129	-1.65825	0.001226	0.019355	0.016391	3291
REACTOME_NONHOMOLOGOUS_END_JOINING_NHEJ	65	-0.46942	-1.66725	0.003346	0.039998	0.033873	3331
REACTOME_ACTIVATION_OF_THE_MRNA_UPON_BINDING_OF_THE_CAP_BINDING_COMPLEX_AND_EIFS_AND_SUBSEQ	60	-0.4816	-1.67188	0.002578	0.032835	0.027808	3322
REACTOME_CHROMOSOME_MAINTENANCE	137	-0.4313	-1.68321	0.000212	0.005533	0.004686	2900
REACTOME_RNA_POLYMERASE_I_TRANSCRIPTION	108	-0.43897	-1.68432	0.001109	0.018104	0.015332	3529
REACTOME_REGULATION_OF_INSULIN_LIKE_GROWTH_FACTOR_IGF_TRANSPORT_AND_UPTAKE_BY_INSULIN_LIKE_GRC	124	-0.43685	-1.68516	0.000237	0.005821	0.00493	1517
REACTOME_AMYLOID_FIBER_FORMATION	107	-0.44438	-1.68855	0.000572	0.010444	0.008845	2795
REACTOME_EUKARYOTIC_TRANSLATION_INITIATION	120	-0.46342	-1.77336	9.68E-05	0.003174	0.002688	3322
REACTOME_RRNA_PROCESSING	202	-0.4231	-1.77438	3.35E-06	0.000178	0.000151	3796
REACTOME_INHIBITION_OF_DNA_RECOMBINATION_AT_TELOMERE	65	-0.5004	-1.7773	0.001118	0.018104	0.015332	3331
REACTOME_NEGATIVE_EPIGENETIC_REGULATION_OF_RRNA_EXPRESSION	106	-0.47088	-1.79053	0.000291	0.006409	0.005428	3529
REACTOME_SIGNALING_BY_BRAF_AND_RAF1_FUSIONS	65	-0.50682	-1.80009	0.00087	0.014832	0.012561	267
REACTOME_POSITIVE_EPIGENETIC_REGULATION_OF_RRNA_EXPRESSION	103	-0.46946	-1.80319	0.0003	0.006497	0.005502	3529
REACTOME_MITOCHONDRIAL_TRANSLATION	94	-0.49131	-1.83382	0.000263	0.006107	0.005172	3930
REACTOME_THE_CITRIC_ACID_TCA_CYCLE_AND_RESPIRATORY_ELECTRON_TRANSPORT	166	-0.44445	-1.83745	2.29E-05	0.000887	0.000751	2760
REACTOME_HCMV_LATE_EVENTS	112	-0.47285	-1.83771	9.95E-05	0.00318	0.002693	3530
REACTOME_SELENOAMINO_ACID_METABOLISM	118	-0.49469	-1.88076	1.20E-05	0.000511	0.000433	3291
REACTOME_SARS_COV_2_MODULATES_HOST_TRANSLATION_MACHINERY	51	-0.55212	-1.88211	0.001553	0.022833	0.019337	3291
REACTOME_SIGNALING_BY_MODERATE_KINASE_ACTIVITY_BRAF_MUTANTS	44	-0.58096	-1.88352	0.001312	0.020219	0.017123	263
REACTOME_BASE_EXCISION_REPAIR_AP_SITE_FORMATION	60	-0.54614	-1.89594	0.000183	0.004976	0.004214	3529
REACTOME_B_WICH_COMPLEX_POSITIVELY_REGULATES_RRNA_EXPRESSION	88	-0.50668	-1.8985	0.000315	0.006609	0.005597	3529
REACTOME_RECOGNITION_AND_ASSOCIATION_OF_DNA_GLYCOSYLASE_WITH_SITE_CONTAINING_AN_AFFECTED_PUI	53	-0.56151	-1.9136	0.000245	0.005904	0.005	3331
REACTOME_UREA_CYCLE	10	-0.8087	-1.92722	0.003025	0.036496	0.030908	650
REACTOME_INTEGRIN_SIGNALING	27	-0.65866	-1.93106	0.000613	0.011002	0.009317	5
REACTOME_IRAK4_DEFICIENCY_TLR2_4	18	-0.70251	-1.93447	0.002662	0.033385	0.028273	5
REACTOME_MAP2K_AND_MAPK_ACTIVATION	40	-0.61115	-1.94004	0.000259	0.006107	0.005172	263
REACTOME_TRANSCRIPTIONAL_REGULATION_BY_SMALL_RNAS	104	-0.51036	-1.94318	3.35E-05	0.001258	0.001066	3529
REACTOME_SYNTHESIS_OF_BILE_ACIDS_AND_BILE_SALTS_VIA_24_HYDROXYCHOLESTEROL	14	-0.75867	-1.97165	0.004266	0.047544	0.040264	842
REACTOME_PLATELET_AGGREGATION_PLUG_FORMATION	38	-0.62642	-1.9777	0.000155	0.004355	0.003688	133
REACTOME_MEIOTIC_RECOMBINATION	84	-0.53579	-2.00141	4.24E-05	0.001466	0.001241	3418
REACTOME_TRANSLATION	293	-0.46421	-2.01454	1.00E-10	4.26E-08	3.61E-08	3811
REACTOME_GLUCURONIDATION	25	-0.6993	-2.04289	0.000532	0.00986	0.00835	2285

REACTOME_RESPIRATORY_ELECTRON_TRANSPORT	93	-0.55057	-2.05491	2.49E-06	0.000144	0.000122	2726
REACTOME_PEPTIDE_HORMONE_BIOSYNTHESIS	14	-0.79103	-2.05575	0.001932	0.026863	0.02275	53
REACTOME_GRB2_SOS_PROVIDES_LINKAGE_TO_MAPK_SIGNALING_FOR_INTEGRINS	15	-0.77929	-2.06121	0.001539	0.022833	0.019337	5
REACTOME_COMMON_PATHWAY_OF_FIBRIN_CLOT_FORMATION	22	-0.71504	-2.0614	0.001403	0.021356	0.018086	360
REACTOME_FORMATION_OF_FIBRIN_CLOT_CLOTTING_CASCADE	38	-0.65696	-2.07413	4.18E-05	0.001466	0.001241	998
REACTOME_P130CAS_LINKAGE_TO_MAPK_SIGNALING_FOR_INTEGRINS	15	-0.79276	-2.09683	0.000885	0.014894	0.012613	5
REACTOME_RESPIRATORY_ELECTRON_TRANSPORT_ATP_SYNTHESIS_BY_CHEMIOSMOTIC_COUPLING_AND_HEAT_PRO	115	-0.54504	-2.10066	5.46E-07	4.97E-05	4.21E-05	2726
REACTOME_EUKARYOTIC_TRANSLATION_ELONGATION	94	-0.56637	-2.114	2.45E-06	0.000144	0.000122	3291
REACTOME_RNA_POLYMERASE_I_PROMOTER_ESCAPE	88	-0.56525	-2.11797	1.50E-05	0.000598	0.000507	3529
REACTOME_HDACS_DEACETYLATE_HISTONES	90	-0.56577	-2.12488	1.03E-06	7.77E-05	6.58E-05	3530
REACTOME_MITOCHONDRIAL_PROTEIN_IMPORT	62	-0.61384	-2.1451	5.37E-06	0.000254	0.000215	2700
REACTOME_RESPONSE_OF_EIF2AK4_GCN2_TO_AMINO_ACID_DEFICIENCY	102	-0.57291	-2.19226	5.25E-07	4.97E-05	4.21E-05	3291
REACTOME_SRP_DEPENDENT_COTRANSLATIONAL_PROTEIN_TARGETING_TO_MEMBRANE	113	-0.5743	-2.22851	9.34E-08	1.19E-05	1.01E-05	3291
REACTOME_PRC2_METHYLATES_HISTONES_AND_DNA	70	-0.61795	-2.23592	8.24E-07	6.59E-05	5.58E-05	3418
REACTOME_DEPOSITION_OF_NEW_CENPA_CONTAINING_NUCLEOSOMES_AT_THE_CENTROMERE	70	-0.63468	-2.29648	2.55E-07	2.72E-05	2.30E-05	3331
REACTOME_ACTIVATED_PKN1_STIMULATES_TRANSCRIPTION_OF_AR_ANDROGEN_RECEPTOR_REGULATED_GENES_KLK	64	-0.65056	-2.3009	1.44E-06	9.86E-05	8.35E-05	3529
REACTOME_ERCC6_CSB_AND_EHMT2_G9A_POSITIVELY_REGULATE_RRNA_EXPRESSION	73	-0.63824	-2.33139	2.11E-07	2.45E-05	2.08E-05	3529
REACTOME_ASSEMBLY_OF_THE_ORC_COMPLEX_AT_THE_ORIGIN_OF_REPLICATION	66	-0.67935	-2.39894	3.19E-08	5.83E-06	4.94E-06	3529
REACTOME_CONDENSATION_OF_PROPHASE_CHROMOSOMES	71	-0.6694	-2.4401	7.73E-08	1.10E-05	9.30E-06	3418
REACTOME_SIRT1_NEGATIVELY_REGULATES_RRNA_EXPRESSION	65	-0.68824	-2.44444	2.42E-08	5.15E-06	4.37E-06	3529
REACTOME_DNA_METHYLATION	62	-0.70555	-2.46556	5.55E-09	1.42E-06	1.20E-06	3529

Supplementary Table S 5

GSE68465

ID	sex	age	status	time	riskscore	riskgroup	group
GSM1672282	Sex: Femal	age: 74	0	756	8.668515239	highrisk	cluster1
GSM1672283	Sex: Femal	age: 74	1	1944	7.611933411	lowrisk	cluster1
GSM1672285	Sex: Femal	age: 59	1	819	7.890391662	lowrisk	cluster1
GSM1672286	Sex: Femal	age: 60	0	1215	8.157734387	lowrisk	cluster1
GSM1672287	Sex: Femal	age: 54	0	1176	7.982393172	lowrisk	cluster1
GSM1672288	Sex: Femal	age: 63	0	723	8.428669341	lowrisk	cluster1
GSM1672289	Sex: Femal	age: 63	1	3.9	9.083399564	highrisk	cluster1
GSM1672290	Sex: Male	age: 66	1	1506	8.840104685	highrisk	cluster1
GSM1672291	Sex: Femal	age: 52	0	480.9	8.331957636	lowrisk	cluster1
GSM1672292	Sex: Femal	age: 62	1	294	7.458256714	lowrisk	cluster1
GSM1672293	Sex: Male	age: 60	1	828	8.956265633	highrisk	cluster2
GSM1672294	Sex: Femal	age: 66	1	384	7.520415355	lowrisk	cluster1
GSM1672295	Sex: Femal	age: 61	0	1728	8.106380081	lowrisk	cluster1
GSM1672296	Sex: Femal	age: 68	1	144	9.249042273	highrisk	cluster2
GSM1672297	Sex: Femal	age: 48	1	231	9.1012197	highrisk	cluster2
GSM1672298	Sex: Femal	age: 75	1	546	8.375614039	lowrisk	cluster1
GSM1672301	Sex: Femal	age: 72	1	1314	9.163484994	highrisk	cluster1
GSM1672302	Sex: Femal	age: 72	1	177	10.08312217	highrisk	cluster2
GSM1672303	Sex: Male	age: 71	1	72	10.48392093	highrisk	cluster2
GSM1672304	Sex: Male	age: 58	1	258	9.430869322	highrisk	cluster1
GSM1672305	Sex: Male	age: 63	1	369	9.660837926	highrisk	cluster2
GSM1672306	Sex: Femal	age: 74	1	168	7.719730892	lowrisk	cluster1
GSM1672308	Sex: Male	age: 59	1	1770	9.204168997	highrisk	cluster1
GSM1672310	Sex: Femal	age: 45	1	30	9.113039612	highrisk	cluster1
GSM1672311	Sex: Femal	age: 78	1	504	8.078112788	lowrisk	cluster1
GSM1672312	Sex: Male	age: 75	0	1860	8.136926584	lowrisk	cluster1
GSM1672313	Sex: Male	age: 61	1	1869	8.151680227	lowrisk	cluster1
GSM1672316	Sex: Male	age: 73	1	558	8.292400433	lowrisk	cluster1
GSM1672317	Sex: Male	age: 51	1	1374	10.18963802	highrisk	cluster1
GSM1672318	Sex: Femal	age: 63	1	468	9.05906071	highrisk	cluster2
GSM1672321	Sex: Male	age: 56	1	1215	8.484467629	lowrisk	cluster1
GSM1672322	Sex: Femal	age: 69	0	1563	8.447610754	lowrisk	cluster1
GSM1672323	Sex: Femal	age: 58	1	528	7.897252232	lowrisk	cluster1
GSM1672324	Sex: Male	age: 62	1	387	9.690561302	highrisk	cluster1
GSM1672326	Sex: Male	age: 65	0	42	8.027261997	lowrisk	cluster1

GSM1672329	Sex: Male	age: 72	1	570	7.718676797	lowrisk	cluster1
GSM1672330	Sex: Male	age: 65	1	153	7.702354943	lowrisk	cluster1
GSM1672334	Sex: Male	age: 63	0	1914	7.777187114	lowrisk	cluster1
GSM1672335	Sex: Femal	age: 54	1	1155	7.665764194	lowrisk	cluster1
GSM1672341	Sex: Male	age: 64	1	846	8.459506282	lowrisk	cluster1
GSM1672342	Sex: Male	age: 69	1	66	9.300534731	highrisk	cluster1
GSM1672344	Sex: Femal	age: 62	0	969	8.0855786	lowrisk	cluster1
GSM1672347	Sex: Male	age: 77	1	447	8.525304795	lowrisk	cluster1
GSM1672348	Sex: Male	age: 56	1	78	8.469760994	lowrisk	cluster1
GSM1672350	Sex: Male	age: 51	0	1872	8.247033119	lowrisk	cluster1
GSM1672351	Sex: Femal	age: 63	0	1929	7.800912224	lowrisk	cluster1
GSM1672352	Sex: Femal	age: 85	1	627	8.63925904	highrisk	cluster1
GSM1672353	Sex: Femal	age: 60	1	9	8.683573626	highrisk	cluster1
GSM1672354	Sex: Male	age: 58	1	1677	7.734692096	lowrisk	cluster1
GSM1672355	Sex: Male	age: 81	0	1089	7.878274721	lowrisk	cluster1
GSM1672356	Sex: Femal	age: 52	1	261	8.980672975	highrisk	cluster1
GSM1672357	Sex: Femal	age: 81	0	987	8.23600532	lowrisk	cluster1
GSM1672358	Sex: Femal	age: 57	0	1143	8.441759284	lowrisk	cluster1
GSM1672359	Sex: Femal	age: 76	1	816	8.885648798	highrisk	cluster1
GSM1672360	Sex: Femal	age: 60	1	1323	8.35286145	lowrisk	cluster1
GSM1672361	Sex: Femal	age: 62	0	1296	8.874217728	highrisk	cluster1
GSM1672362	Sex: Male	age: 68	1	375	9.34512452	highrisk	cluster2
GSM1672363	Sex: Male	age: 73	0	885	8.293930562	lowrisk	cluster2
GSM1672365	Sex: Male	age: 63	0	1611	7.594389055	lowrisk	cluster1
GSM1672366	Sex: Male	age: 70	0	654	8.246231511	lowrisk	cluster1
GSM1672367	Sex: Male	age: 78	0	1674	8.75678626	highrisk	cluster1
GSM1672368	Sex: Male	age: 54	1	630	9.837938334	highrisk	cluster2
GSM1672369	Sex: Male	age: 78	1	219	8.539443702	lowrisk	cluster1
GSM1672371	Sex: Male	age: 72	0	1824	7.666281498	lowrisk	cluster1
GSM1672372	Sex: Femal	age: 81	1	396	8.354973059	lowrisk	cluster1
GSM1672373	Sex: Femal	age: 69	0	228	8.150156376	lowrisk	cluster1
GSM1672374	Sex: Male	age: 59	0	696	8.681682475	highrisk	cluster1
GSM1672375	Sex: Femal	age: 76	0	1418.1	8.084765601	lowrisk	cluster1
GSM1672376	Sex: Male	age: 70	1	1773.3	9.756116496	highrisk	cluster2
GSM1672377	Sex: Male	age: 58	0	1547.4	8.671238706	highrisk	cluster1
GSM1672378	Sex: Femal	age: 48	1	517.5	7.994583857	lowrisk	cluster1
GSM1672379	Sex: Male	age: 82	1	198	8.122305382	lowrisk	cluster1
GSM1672380	Sex: Male	age: 62	1	1359.9	10.69349537	highrisk	cluster2

GSM1672381	Sex: Male	age: 82	1	292.2	8.504443244	lowrisk	cluster2
GSM1672382	Sex: Male	age: 68	0	1766.4	8.348839869	lowrisk	cluster1
GSM1672383	Sex: Male	age: 58	0	1554.3	8.552710492	highrisk	cluster1
GSM1672384	Sex: Male	age: 63	1	59.4	9.005508916	highrisk	cluster1
GSM1672385	Sex: Male	age: 78	1	191.4	7.933219292	lowrisk	cluster1
GSM1672386	Sex: Femal	age: 57	1	1320.3	8.375851448	lowrisk	cluster1
GSM1672388	Sex: Femal	age: 86	1	607.2	8.910910994	highrisk	cluster1
GSM1672390	Sex: Male	age: 73	0	1690.5	8.885699469	highrisk	cluster1
GSM1672392	Sex: Femal	age: 75	0	819.6	8.487958337	lowrisk	cluster1
GSM1672393	Sex: Femal	age: 49	0	1837.5	8.000890515	lowrisk	cluster2
GSM1672394	Sex: Femal	age: 51	0	1512.9	8.358378908	lowrisk	cluster1
GSM1672396	Sex: Femal	age: 76	0	2002.2	8.527332037	lowrisk	cluster1
GSM1672398	Sex: Male	age: 76	1	597	9.428116592	highrisk	cluster1
GSM1672399	Sex: Femal	age: 60	1	509.4	9.723887799	highrisk	cluster1
GSM1672400	Sex: Male	age: 73	1	1359.9	8.724708452	highrisk	cluster1
GSM1672402	Sex: Femal	age: 55	1	378.9	8.906268448	highrisk	cluster2
GSM1672404	Sex: Femal	age: 53	0	47.4	8.533652184	lowrisk	cluster1
GSM1672405	Sex: Femal	age: 77	0	915.9	8.821399301	highrisk	cluster1
GSM1672406	Sex: Femal	age: 55	0	1247.7	8.946619279	highrisk	cluster1
GSM1672407	Sex: Male	age: 67	1	164.4	8.584905039	highrisk	cluster1
GSM1672409	Sex: Femal	age: 72	1	508.2	9.156879411	highrisk	cluster1
GSM1672410	Sex: Male	age: 43	1	67.2	8.60691554	highrisk	cluster1
GSM1672411	Sex: Femal	age: 52	1	265.5	9.882176517	highrisk	cluster2
GSM1672414	Sex: Male	age: 59	1	749.1	8.164086192	lowrisk	cluster1
GSM1672415	Sex: Male	age: 62	1	31.2	8.248699561	lowrisk	cluster1
GSM1672416	Sex: Male	age: 70	1	366	10.01711071	highrisk	cluster1
GSM1672417	Sex: Male	age: 74	1	81	7.737350853	lowrisk	cluster1
GSM1672419	Sex: Male	age: 67	1	1983	7.865508755	lowrisk	cluster1
GSM1672421	Sex: Male	age: 75	1	1440	10.27968659	highrisk	cluster2
GSM1672422	Sex: Male	age: 64	1	240	10.1868977	highrisk	cluster1
GSM1672424	Sex: Femal	age: 70	0	1410	7.978690099	lowrisk	cluster1
GSM1672425	Sex: Male	age: 67	1	60	8.178623042	lowrisk	cluster1
GSM1672426	Sex: Femal	age: 56	1	750	8.225984576	lowrisk	cluster2
GSM1672427	Sex: Male	age: 57	0	1980	7.692553282	lowrisk	cluster1
GSM1672428	Sex: Femal	age: 81	0	1320	8.297026725	lowrisk	cluster1
GSM1672431	Sex: Male	age: 65	0	1650	8.72840318	highrisk	cluster1
GSM1672432	Sex: Male	age: 62	0	1950	10.49123326	highrisk	cluster1
GSM1672433	Sex: Male	age: 71	0	900	7.255334074	lowrisk	cluster1

GSM1672435	Sex: Male	age: 68	1	510	8.947434309	highrisk	cluster1
GSM1672436	Sex: Femal	age: 48	1	630	9.402784734	highrisk	cluster1
GSM1672437	Sex: Femal	age: 82	1	690	10.58037774	highrisk	cluster2
GSM1672438	Sex: Male	age: 64	1	480	8.67978809	highrisk	cluster2
GSM1672439	Sex: Male	age: 68	0	1200	8.55861803	highrisk	cluster2
GSM1672440	Sex: Male	age: 58	1	30	10.10176503	highrisk	cluster2
GSM1672441	Sex: Male	age: 73	0	870	8.111081346	lowrisk	cluster1
GSM1672442	Sex: Femal	age: 52	1	180	8.780912867	highrisk	cluster2
GSM1672443	Sex: Male	age: 62	0	990	8.214480014	lowrisk	cluster1
GSM1672445	Sex: Male	age: 62	0	930	7.952858798	lowrisk	cluster1
GSM1672446	Sex: Femal	age: 50	0	450	8.417348428	lowrisk	cluster2
GSM1672448	Sex: Male	age: 74	1	870	8.258404983	lowrisk	cluster1
GSM1672449	Sex: Femal	age: 55	0	990	8.061389778	lowrisk	cluster2
GSM1672450	Sex: Male	age: 54	1	480	8.092906343	lowrisk	cluster1
GSM1672451	Sex: Male	age: 76	0	1860	9.147422117	highrisk	cluster2
GSM1672453	Sex: Femal	age: 58	1	120	8.463913726	lowrisk	cluster1
GSM1672454	Sex: Male	age: 70	1	210	8.284326469	lowrisk	cluster1
GSM1672455	Sex: Femal	age: 57	1	870	9.136840806	highrisk	cluster1
GSM1672457	Sex: Femal	age: 74	1	420	8.873728753	highrisk	cluster2
GSM1672459	Sex: Femal	age: 66	0	990	8.517742032	lowrisk	cluster2
GSM1672460	Sex: Male	age: 67	1	330	7.49438039	lowrisk	cluster1
GSM1672461	Sex: Femal	age: 57	0	1800	7.549491439	lowrisk	cluster1
GSM1672462	Sex: Male	age: 64	0	1500	8.163067615	lowrisk	cluster2
GSM1672463	Sex: Femal	age: 59	1	240	9.386069393	highrisk	cluster2
GSM1672464	Sex: Femal	age: 76	0	1110	7.661869769	lowrisk	cluster1
GSM1672465	Sex: Femal	age: 82	0	1620	7.673495016	lowrisk	cluster1
GSM1672466	Sex: Male	age: 58	0	930	8.099713819	lowrisk	cluster1
GSM1672467	Sex: Male	age: 65	0	1140	8.231841525	lowrisk	cluster1
GSM1672469	Sex: Femal	age: 71	0	780	8.095303213	lowrisk	cluster1
GSM1672470	Sex: Femal	age: 61	1	1440	7.780589878	lowrisk	cluster1
GSM1672471	Sex: Male	age: 75	1	300	8.746984293	highrisk	cluster2
GSM1672474	Sex: Femal	age: 51	0	1500	8.880512308	highrisk	cluster1
GSM1672476	Sex: Femal	age: 50	1	450	8.57586706	highrisk	cluster1
GSM1672477	Sex: Femal	age: 69	0	1800	9.922500141	highrisk	cluster1
GSM1672478	Sex: Femal	age: 63	0	780	9.758612927	highrisk	cluster2
GSM1672479	Sex: Femal	age: 76	1	210	7.932325276	lowrisk	cluster1
GSM1672480	Sex: Femal	age: 72	0	660	8.232941788	lowrisk	cluster1
GSM1672481	Sex: Femal	age: 70	0	1890	7.899839647	lowrisk	cluster1

GSM1672483	Sex: Femal	age: 81	0	210	7.582667847	lowrisk	cluster1
GSM1672484	Sex: Femal	age: 61	1	990	8.115568343	lowrisk	cluster1
GSM1672485	Sex: Femal	age: 53	0	1080	8.582031653	highrisk	cluster1
GSM1672486	Sex: Femal	age: 72	1	300	9.81422128	highrisk	cluster2
GSM1672487	Sex: Femal	age: 53	1	210	8.498679495	lowrisk	cluster1
GSM1672488	Sex: Male	age: 61	0	450	8.824084731	highrisk	cluster1
GSM1672489	Sex: Male	age: 66	1	450	8.840854411	highrisk	cluster2
GSM1672490	Sex: Femal	age: 74	0	1260	8.55776433	highrisk	cluster2
GSM1672491	Sex: Femal	age: 48	1	660	7.639721667	lowrisk	cluster1
GSM1672492	Sex: Femal	age: 65	0	1500	9.641464268	highrisk	cluster1
GSM1672493	Sex: Femal	age: 70	1	1560	8.732078361	highrisk	cluster2
GSM1672494	Sex: Femal	age: 79	1	180	9.3775021	highrisk	cluster2
GSM1672495	Sex: Male	age: 67	1	2010	8.424015287	lowrisk	cluster1
GSM1672496	Sex: Male	age: 77	0	1440	7.352533401	lowrisk	cluster1
GSM1672497	Sex: Femal	age: 63	1	990	8.11576154	lowrisk	cluster1
GSM1672498	Sex: Femal	age: 76	0	1110	7.805021668	lowrisk	cluster1
GSM1672499	Sex: Male	age: 72	1	60	9.141924485	highrisk	cluster2
GSM1672500	Sex: Femal	age: 51	1	720	8.920089775	highrisk	cluster1
GSM1672501	Sex: Femal	age: 63	0	1320	7.281333136	lowrisk	cluster1
GSM1672502	Sex: Femal	age: 80	0	900	10.01752341	highrisk	cluster2
GSM1672503	Sex: Femal	age: 74	0	720	8.455284137	lowrisk	cluster1
GSM1672505	Sex: Femal	age: 62	0	780	8.149350705	lowrisk	cluster1
GSM1672506	Sex: Femal	age: 67	0	1380	8.236727411	lowrisk	cluster2
GSM1672507	Sex: Femal	age: 71	0	1680	8.593245797	highrisk	cluster1
GSM1672508	Sex: Femal	age: 61	0	1200	8.250540368	lowrisk	cluster1
GSM1672509	Sex: Femal	age: 38	0	1140	8.281386175	lowrisk	cluster1
GSM1672510	Sex: Femal	age: 72	0	1290	8.424961805	lowrisk	cluster2
GSM1672511	Sex: Femal	age: 43	0	1650	9.182719614	highrisk	cluster1
GSM1672512	Sex: Femal	age: 65	1	300	8.743382186	highrisk	cluster1
GSM1672513	Sex: Femal	age: 54	0	1080	8.468547808	lowrisk	cluster1
GSM1672514	Sex: Femal	age: 79	1	120	8.002110867	lowrisk	cluster1
GSM1672515	Sex: Male	age: 61	1	60	7.772958175	lowrisk	cluster1
GSM1672516	Sex: Male	age: 74	0	1860	7.836505614	lowrisk	cluster1
GSM1672517	Sex: Femal	age: 80	0	1470	8.358876692	lowrisk	cluster1
GSM1672518	Sex: Femal	age: 68	1	1080	8.609991701	highrisk	cluster1
GSM1672519	Sex: Femal	age: 64	1	1110	7.803057261	lowrisk	cluster1
GSM1672520	Sex: Femal	age: 58	0	780	7.59735861	lowrisk	cluster1
GSM1672521	Sex: Male	age: 57	0	1290	7.969122409	lowrisk	cluster2

GSM1672522	Sex: Femal	age: 57	0	1230	8.209124689	lowrisk	cluster1
GSM1672523	Sex: Male	age: 67	0	1290	8.375242196	lowrisk	cluster1
GSM1672524	Sex: Femal	age: 75	0	960	9.176676251	highrisk	cluster2
GSM1672525	Sex: Male	age: 74	0	1920	8.098939476	lowrisk	cluster1
GSM1672527	Sex: Male	age: 75	1	330	7.326362269	lowrisk	cluster1
GSM1672530	Sex: Male	age: 59	1	600	8.963368859	highrisk	cluster2
GSM1672532	Sex: Male	age: 43	0	870	7.856419747	lowrisk	cluster1
GSM1672533	Sex: Male	age: 54	1	390	7.974637473	lowrisk	cluster1
GSM1672534	Sex: Femal	age: 52	1	180	7.78642476	lowrisk	cluster1
GSM1672537	Sex: Femal	age: 66	1	90	8.907256123	highrisk	cluster2
GSM1672538	Sex: Femal	age: 70	1	540	8.926233804	highrisk	cluster2
GSM1672539	Sex: Male	age: 68	1	90	8.30008021	lowrisk	cluster2
GSM1672540	Sex: Femal	age: 61	1	120	8.628827495	highrisk	cluster1
GSM1672544	Sex: Male	age: 54	1	60	9.110549951	highrisk	cluster2
GSM1672545	Sex: Femal	age: 73	0	450	8.015994922	lowrisk	cluster1
GSM1672546	Sex: Femal	age: 38	0	1650	8.578733091	highrisk	cluster1
GSM1672547	Sex: Male	age: 58	0	1080	8.247122027	lowrisk	cluster1
GSM1672548	Sex: Male	age: 65	1	240	7.064487908	lowrisk	cluster1
GSM1672550	Sex: Male	age: 66	1	90	9.056813881	highrisk	cluster2
GSM1672551	Sex: Male	age: 76	1	210	9.748273376	highrisk	cluster1
GSM1672552	Sex: Male	age: 53	1	1980	8.114036648	lowrisk	cluster1
GSM1672555	Sex: Male	age: 67	1	0	9.260012635	highrisk	cluster2
GSM1672556	Sex: Male	age: 62	1	600	8.319235061	lowrisk	cluster1
GSM1672557	Sex: Male	age: 61	1	570	8.92095832	highrisk	cluster1
GSM1672559	Sex: Male	age: 62	1	270	7.723569008	lowrisk	cluster1
GSM1672562	Sex: Femal	age: 56	1	60	7.890945322	lowrisk	cluster1
GSM1672563	Sex: Male	age: 61	1	1050	9.145483492	highrisk	cluster2
GSM1672564	Sex: Femal	age: 52	1	1950	8.775471393	highrisk	cluster1
GSM1672566	Sex: Male	age: 57	0	1920	8.464504365	lowrisk	cluster1
GSM1672567	Sex: Femal	age: 46	0	1890	9.051858792	highrisk	cluster2
GSM1672568	Sex: Male	age: 71	1	60	7.193439692	lowrisk	cluster1
GSM1672571	Sex: Femal	age: 72	1	30	7.993764856	lowrisk	cluster1
GSM1672572	Sex: Femal	age: 68	1	1170	7.752843281	lowrisk	cluster1
GSM1672573	Sex: Male	age: 65	0	1440	7.364554786	lowrisk	cluster1
GSM1672574	Sex: Femal	age: 68	0	150	8.488842156	lowrisk	cluster1
GSM1672575	Sex: Male	age: 68	0	840	8.597970224	highrisk	cluster1
GSM1672576	Sex: Femal	age: 57	0	1080	6.829226261	lowrisk	cluster1
GSM1672577	Sex: Femal	age: 56	0	840	9.243160937	highrisk	cluster1

GSM1672578	Sex: Male	age: 56	0	1110	9.318277314	highrisk	cluster2
GSM1672579	Sex: Femal	age: 51	0	1200	8.230741832	lowrisk	cluster1
GSM1672580	Sex: Femal	age: 68	1	690	7.272027333	lowrisk	cluster1
GSM1672582	Sex: Femal	age: 62	1	180	8.457915874	lowrisk	cluster1
GSM1672583	Sex: Male	age: 73	0	1980	6.947980311	lowrisk	cluster1
GSM1672584	Sex: Femal	age: 63	1	360	7.776638985	lowrisk	cluster2
GSM1672585	Sex: Male	age: 72	1	60	10.22751536	highrisk	cluster1
GSM1672586	Sex: Femal	age: 57	0	1590	8.070132174	lowrisk	cluster1
GSM1672587	Sex: Femal	age: 55	0	1890	7.49591753	lowrisk	cluster1
GSM1672588	Sex: Male	age: 64	0	690	7.766900826	lowrisk	cluster1
GSM1672589	Sex: Femal	age: 40	0	1860	9.0442531	highrisk	cluster2
GSM1672590	Sex: Femal	age: 75	1	60	8.371636376	lowrisk	cluster1
GSM1672591	Sex: Male	age: 60	0	780	7.543636206	lowrisk	cluster1
GSM1672592	Sex: Femal	age: 58	0	1200	7.380436512	lowrisk	cluster1
GSM1672593	Sex: Male	age: 64	0	600	9.930257836	highrisk	cluster1
GSM1672594	Sex: Male	age: 62	0	1200	7.810778376	lowrisk	cluster1
GSM1672595	Sex: Male	age: 75	0	270	8.732792161	highrisk	cluster1
GSM1672596	Sex: Male	age: 76	1	540	8.081905328	lowrisk	cluster1
GSM1672597	Sex: Femal	age: 59	1	390	8.15150444	lowrisk	cluster1
GSM1672599	Sex: Male	age: 74	1	150	8.453919275	lowrisk	cluster1
GSM1672600	Sex: Femal	age: 70	0	960	8.600898065	highrisk	cluster1
GSM1672601	Sex: Femal	age: 68	0	630	8.489674247	lowrisk	cluster1
GSM1672605	Sex: Male	age: 79	1	30	9.425382693	highrisk	cluster1
GSM1672606	Sex: Male	age: 69	1	600	7.740646633	lowrisk	cluster1
GSM1672607	Sex: Male	age: 77	1	150	9.534611677	highrisk	cluster1
GSM1672608	Sex: Femal	age: 66	1	1264.5	9.761548643	highrisk	cluster1
GSM1672609	Sex: Male	age: 79	1	636.6	9.71266826	highrisk	cluster2
GSM1672610	Sex: Femal	age: 72	1	74.7	9.208736559	highrisk	cluster1
GSM1672611	Sex: Male	age: 67	1	244.5	9.537760907	highrisk	cluster2
GSM1672612	Sex: Femal	age: 79	1	15.9	8.623893201	highrisk	cluster1
GSM1672613	Sex: Femal	age: 74	1	312.3	8.837140532	highrisk	cluster1
GSM1672616	Sex: Male	age: 79	1	1704	10.09354314	highrisk	cluster1
GSM1672617	Sex: Femal	age: 75	1	903.6	9.825519039	highrisk	cluster2
GSM1672618	Sex: Femal	age: 64	1	36.9	9.286191752	highrisk	cluster1
GSM1672620	Sex: Femal	age: 66	1	276.9	8.929030294	highrisk	cluster2
GSM1672621	Sex: Male	age: 49	1	54	8.027276628	lowrisk	cluster1
GSM1672622	Sex: Male	age: 62	1	27.6	9.613077086	highrisk	cluster2
GSM1672625	Sex: Femal	age: 62	1	948.3	9.471846349	highrisk	cluster1

GSM1672626	Sex: Male	age: 82	1	169.5	9.000301068	highrisk	cluster1
GSM1672627	Sex: Male	age: 77	1	12.9	9.860532341	highrisk	cluster2
GSM1672628	Sex: Femal	age: 61	1	496.8	8.657867194	highrisk	cluster1
GSM1672629	Sex: Male	age: 81	1	1836.3	9.063146358	highrisk	cluster2
GSM1672631	Sex: Femal	age: 67	1	9.9	9.466335818	highrisk	cluster1
GSM1672632	Sex: Femal	age: 74	1	105.6	10.04471964	highrisk	cluster1
GSM1672633	Sex: Femal	age: 64	1	450.3	9.402211253	highrisk	cluster2
GSM1672634	Sex: Femal	age: 65	1	63.9	9.050211536	highrisk	cluster1
GSM1672635	Sex: Male	age: 76	1	238.5	10.27144509	highrisk	cluster2
GSM1672639	Sex: Male	age: 70	1	824.1	9.220116372	highrisk	cluster1
GSM1672640	Sex: Male	age: 63	1	0.9	10.13704712	highrisk	cluster2
GSM1672641	Sex: Femal	age: 68	1	603.3	8.588689299	highrisk	cluster1
GSM1672642	Sex: Male	age: 76	1	530.1	9.440303572	highrisk	cluster2
GSM1672643	Sex: Male	age: 49	1	1137.3	8.060015731	lowrisk	cluster1
GSM1672644	Sex: Male	age: 61	1	68.1	9.255067227	highrisk	cluster1
GSM1672646	Sex: Male	age: 60	1	934.2	9.531321736	highrisk	cluster1
GSM1672648	Sex: Male	age: 47	0	112.5	10.40338773	highrisk	cluster2
GSM1672649	Sex: Male	age: 64	1	771.9	9.745496421	highrisk	cluster2
GSM1672651	Sex: Male	age: 48	0	284.7	8.283827377	lowrisk	cluster2
GSM1672652	Sex: Male	age: 79	1	1260.6	9.507915175	highrisk	cluster2
GSM1672653	Sex: Femal	age: 68	1	48.3	8.088151065	lowrisk	cluster1
GSM1672654	Sex: Femal	age: 68	1	134.1	9.104162873	highrisk	cluster1
GSM1672655	Sex: Femal	age: 82	1	1437	8.288825262	lowrisk	cluster1
GSM1672656	Sex: Male	age: 70	0	1765.2	9.497321242	highrisk	cluster2
GSM1672658	Sex: Femal	age: 63	1	69.9	9.314776345	highrisk	cluster1
GSM1672659	Sex: Femal	age: 81	1	210.9	8.302688355	lowrisk	cluster2
GSM1672660	Sex: Femal	age: 75	1	51.3	7.896597405	lowrisk	cluster1
GSM1672661	Sex: Femal	age: 60	1	1206.3	8.660980791	highrisk	cluster1
GSM1672662	Sex: Femal	age: 60	0	2000.7	8.54675484	lowrisk	cluster1
GSM1672663	Sex: Femal	age: 71	1	1229.1	7.643512377	lowrisk	cluster1
GSM1672665	Sex: Male	age: 77	1	1064.4	8.510311709	lowrisk	cluster1
GSM1672666	Sex: Male	age: 50	1	81.9	8.406436374	lowrisk	cluster1
GSM1672667	Sex: Femal	age: 59	1	60	9.260359223	highrisk	cluster2
GSM1672668	Sex: Male	age: 70	1	451.5	9.598692943	highrisk	cluster2
GSM1672671	Sex: Male	age: 72	1	330	9.92974762	highrisk	cluster2
GSM1672672	Sex: Femal	age: 62	0	6	9.357393832	highrisk	cluster1
GSM1672673	Sex: Femal	age: 77	0	1170.9	9.777493225	highrisk	cluster2
GSM1672674	Sex: Male	age: 82	1	1566	10.01584393	highrisk	cluster2

GSM1672675	Sex: Male	age: 73	1	78.9	10.25327335	highrisk	cluster2
GSM1672676	Sex: Femal	age: 57	1	490.8	9.742638317	highrisk	cluster2
GSM1672677	Sex: Male	age: 73	1	196.2	9.111497796	highrisk	cluster2
GSM1672678	Sex: Femal	age: 70	1	360.6	9.18761357	highrisk	cluster1
GSM1672680	Sex: Male	age: 75	1	126.9	8.986994214	highrisk	cluster1
GSM1672681	Sex: Femal	age: 71	1	125.1	9.3048844	highrisk	cluster1
GSM1672682	Sex: Male	age: 36	1	326.1	9.481789044	highrisk	cluster2
GSM1672683	Sex: Femal	age: 68	1	192.3	10.45481007	highrisk	cluster2
GSM1672684	Sex: Femal	age: 68	1	86.7	9.376558408	highrisk	cluster1
GSM1672685	Sex: Male	age: 65	1	59.1	9.391950239	highrisk	cluster2
GSM1672686	Sex: Femal	age: 51	1	306.6	8.578539056	highrisk	cluster1
GSM1672687	Sex: Male	age: 62	1	921	8.341944363	lowrisk	cluster1
GSM1672689	Sex: Male	age: 69	1	351	8.585483038	highrisk	cluster1
GSM1672690	Sex: Male	age: 68	1	996	8.306342937	lowrisk	cluster1
GSM1672691	Sex: Male	age: 57	1	1275	8.306267167	lowrisk	cluster1
GSM1672693	Sex: Male	age: 58	1	906	8.559875071	highrisk	cluster2
GSM1672696	Sex: Male	age: 64	1	1956	8.338934233	lowrisk	cluster1
GSM1672699	Sex: Male	age: 77	1	99	8.579323106	highrisk	cluster1
GSM1672700	Sex: Male	age: 77	1	585	7.463173419	lowrisk	cluster1
GSM1672701	Sex: Male	age: 70	1	252	9.35410135	highrisk	cluster1
GSM1672702	Sex: Male	age: 57	1	807	8.512661752	lowrisk	cluster1
GSM1672704	Sex: Male	age: 66	1	588	8.206566713	lowrisk	cluster1
GSM1672708	Sex: Male	age: 66	1	1434	9.794854489	highrisk	cluster2
GSM1672710	Sex: Femal	age: 46	0	1569	8.691891503	highrisk	cluster1
GSM1672712	Sex: Male	age: 60	1	778.5	8.39467444	lowrisk	cluster1
GSM1672714	Sex: Male	age: 44	1	978	9.968533048	highrisk	cluster1
GSM1672715	Sex: Male	age: 49	1	1206	9.497510442	highrisk	cluster1
GSM1672716	Sex: Male	age: 70	1	435	9.085372304	highrisk	cluster1
GSM1672717	Sex: Male	age: 68	1	276	8.95949625	highrisk	cluster1
GSM1672718	Sex: Male	age: 66	1	570	9.605184014	highrisk	cluster1
GSM1672720	Sex: Male	age: 62	1	270	9.182686475	highrisk	cluster1
GSM1672723	Sex: Male	age: 61	1	1359	10.07432893	highrisk	cluster2

Supplementary Table S 6

GSE50081-GSE37745-GSE30219

ID	time	status	riskscore	riskgroup
GSM1019140	162	1	5.053322	highrisk
GSM1019143	556	1	5.200357	highrisk
GSM1019144	304	1	5.38212	highrisk
GSM1019145	590	1	5.345229	highrisk
GSM1019147	61	1	5.401939	highrisk
GSM1019149	595	1	4.832724	highrisk
GSM1019150	1839	1	5.744426	highrisk
GSM1019152	523	1	6.00531	highrisk
GSM1019153	393	1	4.65347	lowrisk
GSM1019154	250	1	5.075694	highrisk
GSM1019158	490	1	5.034224	highrisk
GSM1019160	84	1	5.847762	highrisk
GSM1019162	355	1	5.339527	highrisk
GSM1019163	88	1	5.33454	highrisk
GSM1019164	286	1	5.886946	highrisk
GSM1019165	151	1	4.868129	highrisk
GSM1019166	2108	1	5.182232	highrisk
GSM1019174	514	1	6.042094	highrisk
GSM1019176	281	1	4.843844	highrisk
GSM1019180	343	1	5.296846	highrisk
GSM1019183	591	1	5.162985	highrisk
GSM1019186	2086	0	6.065663	highrisk
GSM1019188	966	0	5.225724	highrisk
GSM1019190	1728	0	4.825388	highrisk
GSM1019192	1726	0	5.63876	highrisk
GSM1019196	6	1	5.635631	highrisk
GSM1019197	196	1	5.218408	highrisk
GSM1019200	351	1	5.754915	highrisk
GSM1019203	79	1	5.325667	highrisk
GSM1019207	247	1	5.839301	highrisk
GSM1019211	323	1	4.839196	highrisk
GSM1019214	2084	0	4.92785	highrisk
GSM1019216	396	1	5.737853	highrisk

GSM1019217	370	1	5.803362	highrisk
GSM1019218	262	1	5.585087	highrisk
GSM1019220	533	1	5.158484	highrisk
GSM1019224	1874	1	6.14819	highrisk
GSM1019225	177	1	5.201843	highrisk
GSM1019230	2397	1	5.26534	highrisk
GSM1019234	2068	1	5.827882	highrisk
GSM1019239	1222	1	5.100143	highrisk
GSM1019242	2086	0	5.017519	highrisk
GSM1019243	1031	1	5.340738	highrisk
GSM1019244	1243	1	5.185345	highrisk
GSM1019247	1123	1	5.279799	highrisk
GSM1019253	857	1	4.911579	highrisk
GSM1019256	836	1	5.590223	highrisk
GSM1019257	1190	1	5.057686	highrisk
GSM1019258	2170	1	4.959995	highrisk
GSM1019264	1205	1	5.154262	highrisk
GSM1019270	1000	1	5.229679	highrisk
GSM1019272	1224	1	5.449867	highrisk
GSM1019273	1463	1	6.077003	highrisk
GSM1019274	50	1	5.528707	highrisk
GSM1019275	319	1	5.001237	highrisk
GSM1019286	756	1	5.327546	highrisk
GSM1019289	1011	1	5.535145	highrisk
GSM1019290	1500	1	5.396719	highrisk
GSM1019292	1181	1	5.836489	highrisk
GSM1019298	1007	1	5.643735	highrisk
GSM1019299	1610	1	5.667458	highrisk
GSM1019300	1367	1	4.872384	highrisk
GSM1019302	2369	0	5.087425	highrisk
GSM1019303	766	1	5.159441	highrisk
GSM1019308	1221	1	5.182349	highrisk
GSM1019309	979	1	5.046647	highrisk
GSM1019311	1687	1	5.32886	highrisk
GSM1019312	2167	1	5.36042	highrisk
GSM1019314	869	0	5.259299	highrisk

GSM1019316	1445	1	5.505856	highrisk
GSM1019318	2060	1	5.655802	highrisk
GSM1019320	1488	1	5.014761	highrisk
GSM1019328	1113	1	5.57402	highrisk
GSM1019332	1015	1	5.55812	highrisk
GSM1213669	1500.15	1	4.286303	lowrisk
GSM1213670	2744.8	0	4.554187	highrisk
GSM1213671	2671.8	0	4.403793	lowrisk
GSM1213672	2496.6	0	4.230398	lowrisk
GSM1213673	697.15	1	4.539148	lowrisk
GSM1213674	2565.95	0	4.385028	lowrisk
GSM1213675	2500.25	0	4.151892	lowrisk
GSM1213676	1580.45	1	3.841675	lowrisk
GSM1213677	2270.3	0	5.070405	highrisk
GSM1213678	1489.2	1	4.126143	lowrisk
GSM1213679	2303.15	0	4.492022	lowrisk
GSM1213680	2186.35	0	4.20868	lowrisk
GSM1213681	1941.8	0	4.110835	lowrisk
GSM1213682	2029.4	0	4.124548	lowrisk
GSM1213683	1832.3	0	4.250919	lowrisk
GSM1213684	1266.55	0	5.12749	highrisk
GSM1213685	2467.4	0	4.308624	lowrisk
GSM1213686	1036.6	1	5.256577	highrisk
GSM1213687	2357.9	0	4.123463	lowrisk
GSM1213688	2102.4	0	4.080804	lowrisk
GSM1213689	1806.75	0	4.737913	highrisk
GSM1213690	1868.8	0	4.342658	lowrisk
GSM1213691	2332.35	0	5.107751	highrisk
GSM1213692	2095.1	1	4.280415	lowrisk
GSM1213693	401.5	1	4.57825	lowrisk
GSM1213694	2441.85	0	4.18305	lowrisk
GSM1213695	2131.6	0	5.199994	highrisk
GSM1213696	1890.7	0	4.599482	lowrisk
GSM1213697	2325.05	0	4.7167	lowrisk
GSM1213698	2054.95	1	4.489607	lowrisk
GSM1213699	686.2	1	4.499462	lowrisk

GSM1213700	1470.95	1	4.546945	lowrisk
GSM1213701	1905.3	1	5.148913	highrisk
GSM1213702	1069.45	1	4.662246	lowrisk
GSM1213703	2175.4	1	4.323598	lowrisk
GSM1213704	2022.1	0	4.547145	lowrisk
GSM1213705	175.2	1	4.756735	highrisk
GSM1213706	2737.5	0	4.241025	highrisk
GSM1213707	1974.65	0	4.306513	lowrisk
GSM1213708	2230.15	0	4.888816	highrisk
GSM1213709	1898	0	5.079377	highrisk
GSM1213710	120.45	0	4.500998	lowrisk
GSM1213711	1686.3	0	4.492936	lowrisk
GSM1213712	2401.7	0	4.757274	highrisk
GSM1213713	1803.1	0	5.135971	highrisk
GSM1213714	2244.75	0	4.696894	lowrisk
GSM1213715	2686.4	0	4.845175	highrisk
GSM1213716	1595.05	1	5.051097	highrisk
GSM1213717	1792.15	1	4.434438	lowrisk
GSM1213718	1806.75	0	4.972673	highrisk
GSM1213719	339.45	1	4.938656	highrisk
GSM1213720	2325.05	0	4.461988	lowrisk
GSM1213721	2230.15	0	4.561651	lowrisk
GSM1213722	456.25	1	4.187091	lowrisk
GSM1213723	952.65	1	4.032133	lowrisk
GSM1213724	2292.2	0	4.181325	lowrisk
GSM1213725	2346.95	0	4.52572	lowrisk
GSM1213726	2394.4	0	4.516917	lowrisk
GSM1213727	2113.35	1	4.246607	lowrisk
GSM1213728	1967.35	0	4.612795	lowrisk
GSM1213729	883.3	1	5.027859	highrisk
GSM1213730	1171.65	1	4.296995	lowrisk
GSM1213731	2120.65	0	5.287641	highrisk
GSM1213733	1270.2	1	4.670872	lowrisk
GSM1213734	1883.4	0	4.635173	lowrisk
GSM1213735	2211.9	0	4.293037	lowrisk
GSM1213736	332.15	1	5.10278	highrisk

GSM1213737	1076.75	0	3.783564	lowrisk
GSM1213738	1405.25	1	4.836885	highrisk
GSM1213739	43.8	0	3.400739	lowrisk
GSM1213740	2131.6	0	4.473478	lowrisk
GSM1213741	2317.75	1	4.328947	lowrisk
GSM1213742	1919.9	1	4.35774	lowrisk
GSM1213743	1835.95	0	3.601574	lowrisk
GSM1213744	1839.6	0	4.652833	lowrisk
GSM1213745	1737.4	1	4.89962	highrisk
GSM1213746	1887.05	0	4.162729	lowrisk
GSM1213747	1507.45	0	4.213684	lowrisk
GSM1213748	1854.2	0	4.905225	highrisk
GSM1213749	1168	1	4.229862	lowrisk
GSM1213750	375.95	1	4.155584	lowrisk
GSM1213751	1792.15	0	4.180299	lowrisk
GSM1213752	354.05	1	4.30057	lowrisk
GSM1213753	1127.85	1	4.277924	lowrisk
GSM1213754	737.3	1	4.25556	lowrisk
GSM1213755	1941.8	0	4.621167	lowrisk
GSM1213756	1923.55	0	4.190887	lowrisk
GSM1213757	2098.75	0	4.355618	lowrisk
GSM1213758	2011.15	0	3.899701	lowrisk
GSM1213759	2003.85	0	4.603936	lowrisk
GSM1213760	1668.05	0	4.337166	lowrisk
GSM1213761	1799.45	0	4.99112	highrisk
GSM1213762	313.9	1	4.315627	lowrisk
GSM1213763	711.75	1	4.447661	lowrisk
GSM1213765	985.5	1	4.612392	lowrisk
GSM1213766	1270.2	1	4.885352	highrisk
GSM1213767	1328.6	1	4.662067	lowrisk
GSM1213768	1584.1	0	4.168969	lowrisk
GSM1213769	1930.85	0	4.568468	lowrisk
GSM1213770	770.15	1	4.477475	lowrisk
GSM1213771	2693.7	0	4.592948	lowrisk
GSM1213772	2715.6	0	4.289767	lowrisk
GSM1213773	1748.35	1	4.133384	lowrisk

GSM1213774	1098.65	1	4.189744	lowrisk
GSM1213775	1146.1	1	4.127173	lowrisk
GSM1213776	441.65	1	4.724965	highrisk
GSM1213777	1989.25	0	5.310062	highrisk
GSM1213778	2069.55	0	4.768782	highrisk
GSM1213779	1135.15	0	4.260927	lowrisk
GSM1213780	237.25	1	4.298639	lowrisk
GSM1213781	657	1	4.106751	lowrisk
GSM1213782	1171.65	0	4.274691	lowrisk
GSM1213783	219	1	4.822797	highrisk
GSM1213784	1898	0	3.911689	lowrisk
GSM1213785	1419.85	1	4.284377	lowrisk
GSM1213786	2175.4	0	3.116894	lowrisk
GSM1213787	1251.95	0	4.317884	lowrisk
GSM1213788	543.85	1	4.250714	lowrisk
GSM1213789	667.95	1	4.467501	lowrisk
GSM1213790	1890.7	0	4.159495	lowrisk
GSM1213791	1890.7	1	4.570726	lowrisk
GSM1213792	1065.8	1	4.361914	lowrisk
GSM1213793	981.85	0	4.724995	highrisk
GSM1213794	1821.35	0	4.367597	lowrisk
GSM1213795	2434.55	0	4.348554	lowrisk
GSM1213796	2044	1	4.829313	highrisk
GSM1213797	2131.6	0	4.481829	lowrisk
GSM1213799	69.35	1	4.960616	highrisk
GSM1213800	186.15	0	4.167135	lowrisk
GSM1213801	1474.6	1	3.971733	lowrisk
GSM1213802	1726.45	0	5.430431	highrisk
GSM1213803	562.1	0	4.680067	lowrisk
GSM1213805	2197.3	0	4.223581	lowrisk
GSM1213806	624.15	0	4.340659	lowrisk
GSM1213807	259.15	0	4.900511	highrisk
GSM1213808	1825	0	4.513512	lowrisk
GSM1213809	463.55	0	3.953428	lowrisk
GSM1213810	54.75	0	4.173304	lowrisk
GSM1213811	500.05	0	4.571237	lowrisk

GSM1213812	292	1	4.316377	lowrisk
GSM1213813	346.75	1	4.087054	lowrisk
GSM1213814	1861.5	0	4.435855	lowrisk
GSM1213815	1737.4	0	4.068343	lowrisk
GSM1213816	32.85	0	4.2359	lowrisk
GSM1213817	1262.9	1	4.380266	lowrisk
GSM1213818	1233.7	0	4.457335	lowrisk
GSM1213819	554.8	1	4.456354	lowrisk
GSM1213820	697.15	1	4.2618	lowrisk
GSM1213823	1474.6	1	5.533102	highrisk
GSM1213824	580.35	1	4.596452	lowrisk
GSM1213825	2693.7	0	3.81625	lowrisk
GSM1213826	208.05	1	4.907187	highrisk
GSM1213827	1522.05	0	4.391455	lowrisk
GSM1213828	908.85	1	4.555684	lowrisk
GSM1213829	500.05	0	4.737243	highrisk
GSM1213831	1872.45	0	4.430086	lowrisk
GSM1213832	609.55	1	5.616334	highrisk
GSM1213833	204.4	1	5.338628	highrisk
GSM1213834	989.15	0	4.523205	lowrisk
GSM1213835	719.05	1	4.307924	lowrisk
GSM1213836	459.9	0	4.114911	lowrisk
GSM1213838	1405.25	0	4.683905	lowrisk
GSM1213839	492.75	1	4.922487	highrisk
GSM1213840	448.95	1	4.699215	lowrisk
GSM1213841	2361.55	1	4.573929	lowrisk
GSM1213842	383.25	1	4.390609	lowrisk
GSM1213844	1657.1	0	4.261777	lowrisk
GSM1213845	438	1	3.125341	lowrisk
GSM1213846	113.15	1	4.461894	lowrisk
GSM1213848	36.5	1	5.142005	highrisk
GSM1213849	1518.4	0	4.424032	lowrisk
GSM748054	630	1	4.922302	highrisk
GSM748055	2580	1	4.566862	lowrisk
GSM748057	2430	1	4.419961	lowrisk
GSM748058	2040	0	4.718312	highrisk

GSM748068	2610	0	4.765842	highrisk
GSM748071	1530	0	4.522468	lowrisk
GSM748075	840	1	4.984288	highrisk
GSM748077	540	1	4.71812	lowrisk
GSM748078	1740	0	4.57029	lowrisk
GSM748079	840	1	4.659346	lowrisk
GSM748080	1920	0	5.122509	highrisk
GSM748081	180	0	5.36786	highrisk
GSM748083	240	1	4.676278	lowrisk
GSM748085	2640	1	4.585892	lowrisk
GSM748089	90	1	4.687567	lowrisk
GSM748090	2640	0	5.200539	highrisk
GSM748091	2520	0	5.28987	highrisk
GSM748100	1380	1	4.933325	highrisk
GSM748103	1770	0	4.745229	highrisk
GSM748104	1620	1	4.780618	highrisk
GSM748108	1860	0	4.452391	lowrisk
GSM748111	750	1	5.062238	highrisk
GSM748113	1500	1	5.459215	highrisk
GSM748116	540	1	5.131635	highrisk
GSM748121	2550	0	4.339288	lowrisk
GSM748123	360	1	4.748306	highrisk
GSM748124	2430	0	5.081109	highrisk
GSM748125	1680	0	4.483442	lowrisk
GSM748126	2070	0	5.139684	highrisk
GSM748127	1560	1	4.915681	highrisk
GSM748128	2160	0	5.517502	highrisk
GSM748130	360	1	5.092452	highrisk
GSM748131	390	1	4.653158	lowrisk
GSM748134	0	1	4.921793	highrisk
GSM748137	1470	0	4.728417	highrisk
GSM748138	750	1	4.55303	lowrisk
GSM748140	480	1	5.394032	highrisk
GSM748146	540	1	4.772852	highrisk
GSM748150	1680	0	4.429725	lowrisk
GSM748162	2670	0	4.278854	lowrisk

GSM748164	1830	0	4.378966	lowrisk
GSM748166	1950	0	5.280258	highrisk
GSM748170	60	1	4.872387	highrisk
GSM748171	390	1	4.83936	highrisk
GSM748173	420	1	4.89546	highrisk
GSM748178	0	1	4.636681	lowrisk
GSM748179	210	1	5.266661	highrisk
GSM748181	1530	0	4.604007	lowrisk
GSM748186	330	1	4.840134	highrisk
GSM748189	510	1	5.555357	highrisk
GSM748239	90	1	5.651145	highrisk
GSM748240	480	1	5.079758	highrisk
GSM748242	390	1	5.369186	highrisk
GSM748264	630	1	4.65881	lowrisk
GSM748271	480	1	5.733718	highrisk
GSM748276	840	1	4.900269	highrisk
GSM748278	360	1	4.714864	lowrisk

Supplementary Table S 7

	Camptothecin_1003	Vinblastine_1004	Cisplatin_1005	Cytarabine_1006	Docetaxel_1007	Gefitinib_1010
TCGA-78-7156-01A	0.15571005	0.032660759	81.74615431	4.683599603	0.017312041	26.32694321
TCGA-44-6774-01A	0.128688846	0.025754243	25.08015475	7.627677148	0.01385434	39.11003087
TCGA-69-A59K-01A	0.076016516	0.044256186	32.65861822	12.72804466	0.027340337	40.13739171
TCGA-44-2665-01A	0.088042666	0.023889791	14.41104142	3.728907245	0.012548925	30.47931241
TCGA-55-A490-01A	0.471623145	0.098394718	49.23279754	33.68715819	0.023121158	42.82141337
TCGA-55-7816-01A	0.047007951	0.016701787	14.71068662	3.462302157	0.016862158	31.71546262
TCGA-69-8453-01A	0.046441442	0.02360214	21.60716941	3.810225608	0.012190993	39.53694873
TCGA-49-4501-01A	0.177914593	0.057423712	79.86763325	15.89735163	0.020554912	41.11955463
TCGA-49-4512-01A	0.235616951	0.192487841	71.68391502	37.86901533	0.039390633	66.39696757
TCGA-97-7553-01A	0.053599483	0.013126301	12.96220225	4.589409071	0.008613042	22.65655447
TCGA-91-6828-01A	0.072051521	0.027088082	21.10600489	3.569555854	0.018879779	23.51462858
TCGA-67-3771-01A	0.226370264	0.031363959	44.53968837	9.27209977	0.014236834	22.93263142
TCGA-78-8640-01A	0.091048061	0.012853338	15.37090963	4.762615702	0.004071839	9.273619306
TCGA-55-7815-01A	0.212569395	0.091574416	17.62803342	7.199628175	0.044832703	28.27246621
TCGA-MP-A4T4-01A	0.069045449	0.035155161	22.91549165	6.427862204	0.01393154	32.90445223
TCGA-86-8668-01A	0.162791407	0.034521021	101.1912463	7.033058368	0.024242444	25.66975866
TCGA-44-7667-01A	0.049896651	0.005039556	8.591853082	1.651055324	0.003129045	25.39687139
TCGA-05-5429-01A	0.13137931	0.043100396	25.78103801	6.987515128	0.010094706	51.35140185
TCGA-55-A493-01A	0.053170535	0.022932457	21.84364031	5.744368726	0.013912562	30.64638349
TCGA-55-6978-01A	0.026966459	0.006399759	9.926010167	2.153275266	0.005749678	30.52824038
TCGA-J2-A4AG-01A	0.058961055	0.021131415	26.75776392	5.979180559	0.015822432	32.97853299
TCGA-55-A494-01A	0.205974891	0.039969616	51.23866902	7.462345954	0.030372618	43.04065734
TCGA-67-6216-01A	0.032074441	0.015854373	24.37323513	2.964846985	0.007554767	30.11038521
TCGA-78-7535-01A	0.095483583	0.059783409	35.12386691	19.96131442	0.013847603	29.27676673
TCGA-55-6985-01A	0.074709161	0.049462848	24.50598471	9.672090817	0.014915725	31.11295166
TCGA-05-4430-01A	0.178370858	0.022285674	33.62387377	11.60517362	0.010200167	39.33823
TCGA-62-A46R-01A	0.382980162	0.066451821	65.40866704	18.41905414	0.044076352	28.46199233
TCGA-55-7281-01A	0.100044259	0.0505992	34.03044645	12.31013025	0.016697664	30.5245176
TCGA-44-3396-01A	0.05452318	0.015701724	18.70420865	5.44386083	0.00845241	27.83499044
TCGA-80-5611-01A	0.088052552	0.016857561	14.29306253	5.575485857	0.007029973	23.24674289
TCGA-86-8056-01A	0.12136751	0.009486922	29.8193452	2.421120714	0.010912855	21.72987421
TCGA-73-4666-01A	0.079707097	0.014689408	19.87846628	8.833010699	0.005398022	16.43853452
TCGA-95-7039-01A	0.730647681	0.068611434	119.835955	22.56548868	0.018732392	33.00782072
TCGA-75-5147-01A	0.052658594	0.018869063	10.30811262	4.114416489	0.007690148	33.78404823

TCGA-55-8514-01A	0.1265192	0.040672777	30.83618915	23.12731537	0.015287863	20.89511927
TCGA-05-4426-01A	0.081364031	0.051751902	69.13852806	17.18275323	0.014235596	34.44573935
TCGA-50-6590-01A	0.038883026	0.014416622	7.069579671	1.970563745	0.00405223	17.34035097
TCGA-73-4676-01A	0.29536335	0.037051101	44.6992301	26.0483418	0.013270784	20.31897118
TCGA-MP-A4SW-01A	0.156763372	0.035401782	57.68457493	5.267070587	0.017261709	30.95598743
TCGA-50-5946-01A	0.284939445	0.017921447	37.13430432	10.83744107	0.007479961	17.62951303
TCGA-35-5375-01A	0.142342473	0.025256193	12.86020254	16.02524074	0.005807326	19.23116196
TCGA-44-6777-01A	0.119384841	0.047430276	22.32673265	10.64498801	0.017089559	38.43829251
TCGA-78-7149-01A	0.136585104	0.024169825	46.28536885	5.779130193	0.010975229	19.78689391
TCGA-55-A4DG-01A	0.271724175	0.058626711	69.28325081	13.27834681	0.020283809	14.87111133
TCGA-62-A46P-01A	0.041039214	0.018346574	14.32762514	1.593538743	0.007754913	21.84710888
TCGA-78-7155-01A	0.486017024	0.002588983	20.04633625	10.65287017	0.003157624	13.88889362
TCGA-L9-A743-01A	0.035487318	0.019471974	11.46125667	3.974992183	0.010888275	24.51485691
TCGA-44-4112-01A	0.109761898	0.025523577	44.16685715	8.27887232	0.010677103	43.73830096
TCGA-55-6971-01A	0.032429277	0.018372064	9.687296101	4.122847779	0.014383399	26.7111048
TCGA-55-8511-01A	0.132182514	0.014082351	16.05550422	5.118280609	0.005462418	13.33287905
TCGA-MP-A4TJ-01A	0.015880571	0.008432621	4.322475352	1.209361	0.006568458	20.19004984
TCGA-69-7978-01A	0.056391653	0.023820573	20.94702642	6.269764517	0.011652354	25.11200149
TCGA-55-7994-01A	0.184364681	0.027720343	46.75949528	8.038073729	0.014409457	20.04080129
TCGA-83-5908-01A	0.025661911	0.008358468	4.972753426	2.235381155	0.005101538	20.62741603
TCGA-50-5068-01A	0.052595964	0.013050679	10.28913176	4.275291952	0.006658893	40.13352247
TCGA-64-1676-01A	0.083411204	0.036884388	11.75025599	8.379504981	0.009148297	38.71560406
TCGA-86-7953-01A	0.03192233	0.005651658	8.396024029	1.326540104	0.0042808	17.98864683
TCGA-93-A4JQ-01A	0.043924274	0.01392958	18.27750891	2.744204367	0.008080388	19.41637959
TCGA-49-4486-01A	0.131794143	0.091011744	87.3146722	10.02303429	0.016618816	27.41476102
TCGA-MN-A4N1-01A	0.093174067	0.019393696	28.94631014	4.884649472	0.006390313	12.756341
TCGA-50-5944-01A	0.580523653	0.099754935	201.0094788	37.19807296	0.048279797	49.93464887
TCGA-97-7938-01A	0.132684305	0.052321872	53.62349743	6.675583256	0.022059884	23.28437583
TCGA-44-8117-01A	0.633624962	0.060592053	119.2167901	48.59489469	0.027861996	28.46824215
TCGA-05-4420-01A	0.085629551	0.012328022	20.90555141	5.967928729	0.004851036	25.37043191
TCGA-99-AA5R-01A	0.044132158	0.021368031	15.18632001	3.6421156	0.013444253	36.32631805
TCGA-55-8208-01A	0.037392707	0.012387433	6.365961404	2.86797992	0.006715539	19.1199926
TCGA-75-6214-01A	0.200695869	0.028285072	53.18014058	6.492033485	0.006917486	4.205694551
TCGA-50-6597-01A	0.317085096	0.600448564	159.1484547	33.01273946	0.067180128	60.14949295
TCGA-44-2662-01A	0.140870465	0.021851392	25.96598382	8.647964539	0.009987125	24.44741986
TCGA-55-7995-01A	0.035451871	0.008172754	11.1254867	2.80241281	0.005197944	13.79458001

TCGA-95-8494-01A	0.075329364	0.016960007	48.43332901	10.77069989	0.006199915	27.07402169
TCGA-95-7562-01A	0.033848239	0.015593084	6.92286875	6.207008521	0.004925394	12.49756693
TCGA-97-8175-01A	0.13018229	0.031525568	46.70989921	10.16486336	0.016104602	20.03989314
TCGA-69-7760-01A	0.233384765	0.26387182	83.60523869	26.75684393	0.049420356	54.95034026
TCGA-50-5941-01A	0.072357057	0.025711569	21.06314763	7.753554383	0.010906571	25.84044936
TCGA-38-7271-01A	0.036700179	0.018886028	9.614550689	3.579165982	0.009004665	26.04278279
TCGA-55-8085-01A	0.061553309	0.013231036	11.62775082	1.974824579	0.005377898	19.29872626
TCGA-78-7537-01A	0.06793462	0.028803133	26.57992929	4.560473369	0.009795268	26.2576858
TCGA-L9-A7SV-01A	0.433114034	0.025767255	40.24719971	15.53024232	0.011797463	19.16376253
TCGA-55-1592-01A	0.159994811	0.028974321	24.41741215	10.91339453	0.012192619	16.73495787
TCGA-35-4122-01A	0.081216715	0.055495509	20.10723251	9.030372564	0.011439182	45.05836269
TCGA-55-6981-01A	0.231512415	0.062692775	43.5385203	21.56571874	0.019005283	21.13690097
TCGA-93-A4JO-01A	0.049547455	0.009883635	11.83400765	5.88761437	0.006647827	19.59689709
TCGA-50-5055-01A	0.0392343	0.013513945	11.87989625	2.397203155	0.007387783	32.89302403
TCGA-L9-A443-01A	0.173451789	0.069532413	32.37712775	10.12233994	0.041589508	31.96696424
TCGA-55-A48Z-01A	0.07751112	0.08026616	34.94857295	8.713698403	0.021576662	27.26354293
TCGA-55-6968-01A	0.045978587	0.012446915	4.937869933	2.428821228	0.00605907	26.86980847
TCGA-97-A4M6-01A	0.075297823	0.016703759	36.39679609	4.824174098	0.013471934	27.15030128
TCGA-78-7145-01A	0.120600731	0.047470813	38.89379996	8.727215521	0.021969068	49.74881618
TCGA-44-2668-01A	0.035892342	0.010445019	7.494193523	2.449588328	0.004778571	29.41411921
TCGA-86-8669-01A	0.077792326	0.035660625	31.13823191	4.242646742	0.017357587	31.53323022
TCGA-44-A47A-01A	0.040951168	0.011741881	10.52814164	1.844072298	0.009524969	24.13167251
TCGA-91-A4BD-01A	0.050142077	0.049738376	29.68139066	6.364828272	0.012507199	38.27718278
TCGA-55-A57B-01A	0.081755445	0.016878062	47.31690017	5.642601267	0.015717498	15.07879008
TCGA-05-4249-01A	0.062099991	0.012051574	17.40641218	2.284041387	0.009834504	21.91639035
TCGA-44-3398-01A	0.063949108	0.015987843	21.38274577	5.625726962	0.008788777	41.23181855
TCGA-55-8203-01A	0.041158347	0.023982675	10.72922727	3.321298686	0.008979293	20.61310034
TCGA-50-5044-01A	0.039797085	0.013786072	16.63887097	2.045579261	0.005958509	60.5108605
TCGA-44-2659-01A	0.161847803	0.07297111	30.63447013	8.942322416	0.017225889	24.78988287
TCGA-44-2656-01A	0.145271462	0.033456362	20.31624612	5.730142192	0.022667302	17.27586552
TCGA-86-8279-01A	0.213110634	0.045201262	37.33403934	13.843376	0.016835865	47.33972354
TCGA-55-8510-01A	0.138144922	0.052494257	34.24572751	11.10329864	0.018023168	20.11788369
TCGA-38-4630-01A	0.067597808	0.004295378	6.481755774	1.030209955	0.004860701	19.40572808
TCGA-97-A4M1-01A	0.065487166	0.020754383	25.92635166	4.239921602	0.011710839	22.79437374
TCGA-53-7624-01A	0.057041568	0.015504786	9.796451399	2.648312499	0.006704532	11.07433686
TCGA-78-8660-01A	0.063467599	0.021465843	41.7476054	5.349008566	0.012045383	24.3345573

TCGA-93-7348-01A	0.101808548	0.049191122	24.95500819	8.612442614	0.017721071	26.84250665
TCGA-55-8096-01A	0.191651822	0.038354874	33.40161036	9.964365455	0.016547942	22.46766836
TCGA-62-A46U-01A	0.033771392	0.011143855	11.18301909	2.940942458	0.008923167	19.53428088
TCGA-50-5932-01A	0.397816993	0.156933628	145.5231824	16.00745836	0.044708684	28.81960796
TCGA-50-6673-01A	0.264145375	0.040654077	109.9572799	7.618804903	0.024133697	17.51413711
TCGA-55-8089-01A	0.062421724	0.01845519	16.95204385	2.916441477	0.008578931	19.09126481
TCGA-55-6712-01A	0.26614601	0.133848887	154.5462204	17.10223187	0.033290371	31.40724192
TCGA-55-8097-01A	0.100374086	0.017160711	45.56226696	3.266696816	0.012199398	37.16773268
TCGA-67-3772-01A	0.1818933	0.062465893	57.4246092	11.19200946	0.017652249	39.6167655
TCGA-86-6851-01A	0.084729087	0.027176431	18.83381479	5.789105016	0.011388074	21.19980094
TCGA-55-7725-01A	0.054840559	0.02941341	12.05078542	3.247886663	0.021442936	22.51577745
TCGA-05-5423-01A	0.131280132	0.060297999	40.84794736	15.23260406	0.024843379	47.06430187
TCGA-64-5815-01A	0.164432006	0.053348097	44.5406489	16.48217861	0.016819429	41.49998445
TCGA-97-A4LX-01A	0.045100576	0.019586113	19.30026882	4.480090739	0.013087286	35.06093067
TCGA-50-6591-01A	0.034849611	0.009264328	9.077916577	1.673966655	0.006930424	31.14997333
TCGA-05-4433-01A	0.14696129	0.027689653	60.01081225	4.175537598	0.014830242	34.71972179
TCGA-MN-A4N4-01A	0.181895127	0.032792658	31.63288441	9.759173072	0.008883015	13.27046976
TCGA-50-6592-01A	0.099323512	0.015524294	17.50474788	3.449439771	0.007229234	18.27508912
TCGA-05-4427-01A	0.079307854	0.008020417	18.05149885	2.603061306	0.007849043	26.05161464
TCGA-97-7547-01A	0.160244896	0.03166243	65.52746198	5.960354443	0.013060609	19.54292504
TCGA-05-4389-01A	0.193964994	0.047105948	38.68890061	6.124159098	0.016563989	31.07183824
TCGA-62-A46Y-01A	0.053039663	0.020344336	26.85348097	5.940836923	0.01002954	19.83467545
TCGA-44-2666-01A	0.060104737	0.014035303	22.54001105	3.643201582	0.007267969	18.03206609
TCGA-78-7542-01A	0.084345797	0.016002876	30.86203994	3.218088276	0.006041441	22.84575931
TCGA-44-6147-01A	0.121689925	0.043837927	24.00251232	8.028287435	0.022113568	20.97603029
TCGA-50-6595-01A	0.065976227	0.017687755	20.02306156	4.889723224	0.007383763	23.05478107
TCGA-62-8397-01A	0.341497095	0.301095162	152.4573633	25.18430664	0.03706851	50.15098713
TCGA-50-5045-01A	0.069108138	0.017906043	13.58448122	6.154448776	0.008352538	36.88576176
TCGA-75-6212-01A	0.053814356	0.028832601	22.10249181	4.513016921	0.012061439	27.57501395
TCGA-55-7724-01A	0.212109822	0.092315196	20.05761467	20.36751665	0.043059907	14.54279043
TCGA-50-6594-01A	0.101937607	0.022761909	42.78666689	5.173918016	0.010524646	23.54289621
TCGA-71-8520-01A	0.116494103	0.033639121	29.25938676	5.378848368	0.017815663	28.83667944
TCGA-64-5779-01A	0.035134244	0.028356407	8.102591352	2.248161274	0.014586191	31.24114989
TCGA-69-7761-01A	0.089416337	0.037223482	29.91131582	5.062993769	0.018118616	30.10280956
TCGA-MP-A4T9-01A	0.100741968	0.018375512	31.13376052	8.007607625	0.014063471	22.02816079
TCGA-55-7907-01A	0.405726915	0.044751131	56.6142217	10.96242618	0.031012283	22.84238002

TCGA-55-8090-01A	0.07054936	0.02820489	55.9508714	6.084900976	0.013324791	41.86928418
TCGA-97-7554-01A	0.168514155	0.057702607	45.77986001	16.69384953	0.020595548	29.10189161
TCGA-J2-A4AD-01A	0.50476597	0.044741494	118.543862	15.39650429	0.016144689	15.39153104
TCGA-50-8460-01A	0.064399971	0.046846164	28.07129681	6.977871797	0.015814599	38.44031972
TCGA-49-AAR9-01A	0.30855796	0.06123384	71.88900669	12.19443095	0.012507677	27.98293036
TCGA-73-7499-01A	0.049124678	0.028567916	8.311388898	2.948362352	0.007634023	25.90259388
TCGA-05-4432-01A	0.083100899	0.036194921	23.98207622	10.6200292	0.018437894	36.20457409
TCGA-99-8028-01A	0.040881811	0.023020341	8.253639395	5.09836802	0.008392495	32.84833348
TCGA-55-7728-01A	0.053368683	0.028776015	19.41218525	3.227550987	0.012306538	38.68111524
TCGA-86-7954-01A	0.076369139	0.023113748	25.08726839	5.182025169	0.014879247	38.42552334
TCGA-O1-A52J-01A	0.146035477	0.12222624	85.65634434	21.00946598	0.049137638	36.28322307
TCGA-69-8253-01A	0.085820299	0.016030134	42.7652797	5.1325573	0.007458814	16.78749322
TCGA-69-7980-01A	0.093475288	0.015915862	32.22246765	4.134195567	0.009722266	30.83820951
TCGA-53-7813-01A	0.077712461	0.146201959	13.44659744	8.674504242	0.032894537	41.91907586
TCGA-97-7552-01A	0.04525486	0.016382526	18.82213197	2.511054152	0.010188922	24.43910872
TCGA-53-A4EZ-01A	0.150004122	0.046131627	26.02028916	5.940696831	0.009374431	25.70114642
TCGA-44-5643-01A	0.017846619	0.001578605	4.091444612	0.714504519	0.0014395	4.238322682
TCGA-78-7147-01A	0.308562769	0.054264645	85.47776959	22.15903919	0.017255462	42.92575125
TCGA-44-3919-01A	0.075998521	0.041725697	35.95893641	8.394089672	0.021533745	25.85655873
TCGA-55-6983-01A	0.035955706	0.012809183	11.55677504	2.444780359	0.00555878	20.35736753
TCGA-78-8662-01A	0.771208068	0.009054172	36.8086917	17.04442857	0.004618129	13.36662711
TCGA-86-8278-01A	0.048727501	0.014553057	35.57981082	4.817762381	0.009268033	24.66918585
TCGA-NJ-A4YQ-01A	0.059454308	0.023553744	13.89093719	5.07562427	0.011151762	22.45999636
TCGA-67-3773-01A	0.073852936	0.039396565	22.88825099	4.382542915	0.01437253	39.65628846
TCGA-05-4424-01A	0.219433001	0.026717776	28.14889519	18.24589978	0.019010299	24.81915113
TCGA-78-7539-01A	0.296127594	0.054525901	81.97629848	8.821689302	0.02616986	29.32778971
TCGA-86-8074-01A	0.100622094	0.019149632	17.83978152	7.442032508	0.008692222	24.74621389
TCGA-44-6779-01A	0.168931709	0.021759521	36.57230383	6.454910791	0.005825844	13.12780568
TCGA-05-4434-01A	0.049417263	0.009295778	15.78983803	2.552858989	0.006649377	19.69198848
TCGA-86-8672-01A	0.103991127	0.004841296	17.37967545	3.642513312	0.00251009	22.36128794
TCGA-MP-A4TH-01A	0.058057187	0.014131284	25.43757134	2.559421501	0.011613456	27.9609635
TCGA-86-8674-01A	0.142307638	0.011411407	30.84464582	4.714897603	0.004206467	15.73018931
TCGA-S2-AA1A-01A	0.033112639	0.021585807	11.38046458	2.796768658	0.009738925	28.7944005
TCGA-95-7043-01A	0.356116012	0.047385177	39.05020413	13.34587734	0.013423498	21.12677934
TCGA-49-AAR3-01A	0.04642095	0.018234997	12.48608522	3.609864283	0.00743979	25.01472815
TCGA-38-6178-01A	0.128367354	0.020158861	28.39411371	7.202158059	0.00693024	19.66474863

TCGA-97-8172-01A	0.058300917	0.012261112	17.13072978	2.385472781	0.012954153	25.05105729
TCGA-91-6836-01A	0.053058756	0.010625937	10.58044202	4.045851518	0.005685674	45.33038368
TCGA-44-2661-01A	0.050969532	0.026503776	20.41626923	4.565771415	0.013178842	37.96914323
TCGA-38-4632-01A	0.132065736	0.02552451	15.89895029	6.241052948	0.009148171	43.39583913
TCGA-97-8552-01A	0.076197072	0.02146343	39.57949086	6.547090199	0.014970759	31.50818067
TCGA-67-3770-01A	0.388580602	0.080305625	142.1148639	17.6697385	0.026741908	47.23985904
TCGA-50-5935-01A	0.197337703	0.106892923	52.20296657	15.23911234	0.03525324	32.60156607
TCGA-44-7670-01A	0.156753343	0.019358758	27.29795562	5.705796681	0.011174575	32.15133144
TCGA-55-8206-01A	0.069180402	0.014198824	29.84380298	4.969382535	0.0132015	24.64030962
TCGA-91-6849-01A	0.121265574	0.034899117	30.63346432	6.663845623	0.012824149	21.99806145
TCGA-78-7152-01A	0.046503822	0.012201833	19.76192632	1.772809769	0.005621877	16.40697439
TCGA-44-6775-01A	0.117309294	0.037338918	28.96990801	7.508591553	0.017307888	18.98698403
TCGA-91-6840-01A	0.168208941	0.066042594	25.21372204	12.54610586	0.027095764	31.97653566
TCGA-49-AAR4-01A	0.044223646	0.037166479	16.70683572	5.787626972	0.014762437	26.42851819
TCGA-55-8620-01A	0.118807001	0.017538347	21.46743699	4.041905665	0.009437883	27.72852389
TCGA-50-5049-01A	0.053848537	0.021464105	9.810474265	5.606698214	0.00852381	28.81551571
TCGA-05-4405-01A	0.149820991	0.019595352	26.6840217	8.548537613	0.010239122	18.82766643
TCGA-MP-A4T6-01A	0.061999938	0.035232321	26.34675109	4.040532924	0.023964706	34.25670846
TCGA-53-7626-01A	0.053383057	0.020282979	14.92905394	3.009679486	0.011178247	21.16425131
TCGA-55-8614-01A	0.071070651	0.016775627	11.22276358	5.05765704	0.006008127	15.2943969
TCGA-95-8039-01A	0.065706989	0.017548597	40.98998842	4.650098527	0.010892747	23.06325437
TCGA-97-7937-01A	0.481190782	0.123247036	84.68317941	55.72273388	0.035800156	28.74576067
TCGA-49-AARQ-01A	0.02466114	0.028322017	6.953313889	5.738801445	0.009018965	15.51324199
TCGA-44-3918-01A	0.058867648	0.033367202	14.50745977	6.572381336	0.01536178	37.99981566
TCGA-38-4628-01A	0.342227758	0.076137597	77.90012343	19.77986357	0.027487072	41.91031755
TCGA-86-8673-01A	0.098288729	0.034357846	18.86632487	8.74090979	0.009865678	27.90516416
TCGA-55-6972-01A	0.175476146	0.059967943	86.13090051	5.48291421	0.012108402	27.81469128
TCGA-91-8499-01A	0.023780152	0.003599347	3.761938071	1.360230465	0.002902304	23.65530304
TCGA-NJ-A55A-01A	0.031848668	0.009899076	13.35947985	1.64421967	0.009797016	22.96549984
TCGA-55-7283-01A	0.065870367	0.0207684	25.75285337	3.294220008	0.010056883	23.05544938
TCGA-44-7661-01A	0.052324799	0.010710872	14.9224215	2.785598279	0.008606316	23.29342576
TCGA-50-5942-01A	0.077356474	0.026199734	40.71336149	3.995014413	0.014403717	28.79904142
TCGA-05-4402-01A	0.180971323	0.052756989	52.57180013	15.11477763	0.017805532	23.08673435
TCGA-44-5645-01A	0.058872232	0.011996411	25.28837362	2.710945063	0.010937536	23.86112006
TCGA-44-6776-01A	0.239542739	0.028735892	62.75312695	6.526253852	0.010189932	15.27697012
TCGA-05-4397-01A	0.106424244	0.043196577	19.91183149	14.56534758	0.011101149	39.52093603

TCGA-MP-A4TK-01A	0.233205779	0.03152084	64.45223435	14.48659662	0.017771567	38.13416772
TCGA-64-1679-01A	0.375922618	0.035396048	59.76933625	30.61761602	0.012291954	29.19457292
TCGA-L9-A8F4-01A	0.102663307	0.024521999	36.13230914	6.735496486	0.013236356	38.38770856
TCGA-64-1680-01A	0.071449105	0.041172444	49.05895043	4.579923165	0.013017034	35.91177226
TCGA-78-7158-01A	0.056089247	0.010040024	13.14432699	0.763067542	0.005086641	19.76773422
TCGA-78-8648-01A	0.034058931	0.014370875	8.511964426	3.59589525	0.006637984	31.36181914
TCGA-50-5933-01A	0.240380617	0.029670807	43.87748796	12.30547194	0.014137367	25.79700116
TCGA-86-7711-01A	0.239034634	0.015969134	17.90101359	15.31406503	0.004963939	10.56798424
TCGA-35-3615-01A	0.124915412	0.013739577	38.38618375	4.82564516	0.004921762	15.56296143
TCGA-91-8496-01A	0.098566056	0.03469869	36.23374096	7.231804141	0.014129292	35.7479359
TCGA-55-7726-01A	0.418156426	0.03050753	37.6467453	19.26861743	0.008798641	19.23364437
TCGA-49-4494-01A	0.108234582	0.039691594	38.78929968	12.16204163	0.008852022	26.62866025
TCGA-99-8025-01A	0.188869293	0.035292401	33.06604083	11.34199405	0.012311204	25.30625226
TCGA-78-7143-01A	0.142237442	0.042051375	65.21525319	10.56414313	0.02460491	25.2822635
TCGA-44-6778-01A	0.045157502	0.016467375	7.771173184	2.917230214	0.01013822	27.36904818
TCGA-55-8091-01A	0.055830657	0.042288814	33.66528812	7.704789168	0.013171512	43.04233446
TCGA-95-A4VN-01A	0.036445822	0.012095072	12.64882802	2.035745773	0.007504134	23.78326979
TCGA-69-7979-01A	0.207995406	0.022354601	34.7209316	15.9845218	0.006894867	23.27059324
TCGA-J2-8192-01A	0.147618728	0.029375204	40.37482397	9.690151873	0.015678456	19.52042635
TCGA-49-AAQV-01A	0.038307828	0.027659133	13.92357679	5.26738424	0.009616608	20.50490193
TCGA-55-8512-01A	0.046012386	0.016478928	24.7822263	3.77992705	0.010091265	21.79861317
TCGA-L9-A444-01A	0.068173755	0.033435534	11.93147382	6.993941123	0.018094759	27.03578181
TCGA-78-7146-01A	0.101835496	0.016383598	27.34479342	4.374406953	0.00575704	17.97595147
TCGA-73-4662-01A	0.133867887	0.040215481	52.30403844	17.0404986	0.026952036	32.71525794
TCGA-55-8301-01A	0.036845855	0.025833838	9.423726334	4.588222034	0.008676946	30.90701102
TCGA-55-6982-01A	0.320612748	0.18256991	92.18556542	20.55654222	0.041686044	40.75219786
TCGA-J2-A4AE-01A	0.047223377	0.033825225	28.08211616	3.888542095	0.015730308	25.49356588
TCGA-86-8055-01A	0.195383011	0.025623522	43.92741381	10.7210944	0.011591675	25.08958051
TCGA-55-7573-01A	0.063463108	0.011386379	24.27982001	3.959401171	0.009170006	22.27113323
TCGA-55-7227-01A	0.056481547	0.018776909	28.37556534	4.231008853	0.010214277	31.20540985
TCGA-95-7948-01A	0.335015033	0.028971069	135.7932325	19.74379996	0.011548638	30.74866801
TCGA-62-8402-01A	0.01929724	0.007995197	6.22201172	1.090653344	0.00741633	17.74592901
TCGA-49-AARR-01A	0.02381536	0.012157577	11.7887558	1.989102726	0.008619498	24.94605258
TCGA-86-A4P7-01A	0.065372403	0.019048164	17.66627851	3.784622109	0.013298941	28.73032217
TCGA-38-4625-01A	0.045582595	0.011188361	7.698355694	3.666446005	0.00462989	29.08775958
TCGA-49-AARE-01A	0.062101509	0.02452215	17.10700293	5.497398872	0.007200341	21.53562381

TCGA-38-4626-01A	0.035242097	0.016835154	13.74161294	3.949993263	0.009368588	29.11083434
TCGA-86-6562-01A	0.210540684	0.06424162	37.03962033	16.26933676	0.024627633	17.35508612
TCGA-62-A472-01A	0.066007667	0.052395108	44.4258952	5.634676578	0.024461241	40.78092108
TCGA-49-AARN-01A	0.087831532	0.053099473	37.89039856	7.799948817	0.019100716	39.88744118
TCGA-93-7347-01A	0.074915141	0.032087078	26.65490174	3.026685575	0.015936979	34.29481331
TCGA-44-A4SU-01A	0.092877448	0.070127877	41.51770305	11.11665831	0.023738831	22.8698377
TCGA-05-4398-01A	0.069455543	0.019045998	18.09406254	7.322018031	0.007753876	20.5766751
TCGA-05-4382-01A	0.116325634	0.028365609	18.26597658	6.348132975	0.014249567	18.59074503
TCGA-38-4631-01A	0.06175659	0.036821919	13.75657489	3.864106444	0.009543104	39.33989976
TCGA-78-7163-01A	0.265359288	0.09495592	420.3476637	23.59242524	0.017800402	38.59629261
TCGA-55-7911-01A	0.050612875	0.014979075	20.54457771	3.027002739	0.01369106	21.72825723
TCGA-49-AARO-01A	0.068984258	0.048130911	26.38819108	9.314395803	0.022900113	33.89199066
TCGA-86-8358-01A	0.117469454	0.011361052	11.37494152	3.296299249	0.006486411	25.25836635
TCGA-64-1681-01A	0.169498165	0.201812515	55.16065471	21.06158482	0.056929761	45.78845293
TCGA-49-AAR0-01A	0.082208594	0.036770478	29.45031339	8.421184842	0.009975834	38.4708665
TCGA-55-8205-01A	0.021020889	0.006371633	8.157862779	1.063577628	0.004311044	16.16849384
TCGA-49-6744-01A	0.071886404	0.022534655	20.63154021	5.648404299	0.01114216	30.26107436
TCGA-62-8395-01A	0.12895393	0.019265306	65.17535389	7.713969295	0.010641085	20.6527759
TCGA-55-1594-01A	0.132128494	0.018330459	33.28504461	5.326881295	0.021267596	30.29354832
TCGA-44-6146-01A	0.117830397	0.019245919	45.43420689	4.013499764	0.009062482	20.11372896
TCGA-MP-A4TC-01A	0.062937874	0.030604333	12.53648736	4.946898879	0.009369063	22.0135927
TCGA-86-8280-01A	0.105684197	0.030359718	33.45477533	7.838311913	0.015200176	32.20130376
TCGA-64-5775-01A	0.083782474	0.006762943	15.9150734	3.363480436	0.00523297	69.08027061
TCGA-86-8073-01A	0.186539688	0.020595633	48.54793585	5.835015957	0.011104337	16.46059722
TCGA-95-7944-01A	0.026734044	0.010335275	5.863181628	1.90227057	0.003741984	11.27791546
TCGA-05-4403-01A	0.1415641	0.026088161	59.80487814	8.645169648	0.008984622	39.79681679
TCGA-55-7903-01A	0.072897954	0.025945424	26.90746157	5.764781853	0.013136695	31.02123525
TCGA-55-8506-01A	0.106392406	0.036988914	32.34767066	13.38387514	0.01243353	30.51033986
TCGA-86-A4JF-01A	0.036692585	0.026581477	14.99832453	5.139819107	0.009497675	24.71938015
TCGA-97-A4M7-01A	0.048702444	0.021384881	16.06764487	2.052041204	0.010710708	25.48681063
TCGA-49-6767-01A	0.17764051	0.065364434	39.23881066	7.189159814	0.017736935	40.96709028
TCGA-44-8120-01A	0.328410504	0.023202117	54.75729413	14.49978595	0.014148992	24.48691189
TCGA-55-7576-01A	0.148941208	0.042146177	68.05282669	13.33329588	0.017968262	24.74520076
TCGA-93-A4JP-01A	0.103832597	0.038247454	27.92449523	7.229014733	0.018682472	20.50725877
TCGA-50-5931-01A	0.022740305	0.003325938	4.91765683	1.193470294	0.002476678	26.78278918
TCGA-L4-A4E6-01A	0.03677888	0.019493157	15.85918476	3.279052854	0.012400138	28.84631234

TCGA-67-6217-01A	0.14106932	0.034173265	43.80630177	6.511416495	0.017699758	27.22570129
TCGA-86-7714-01A	0.073237924	0.014461298	35.42065842	3.305379002	0.009236577	21.62140884
TCGA-73-4658-01A	0.127691497	0.042151343	30.81727932	11.32262364	0.014749792	34.9251531
TCGA-NJ-A7XG-01A	0.091668681	0.041334561	76.57346202	6.684495302	0.015001211	27.04867227
TCGA-44-A479-01A	0.029015463	0.015853492	6.921351324	1.875123774	0.011096839	17.62012589
TCGA-55-8087-01A	0.076265699	0.007704325	29.29299844	2.411575078	0.006324585	24.73194498
TCGA-38-4627-01A	0.12233376	0.036849012	34.72518557	10.59083234	0.013069031	48.27026117
TCGA-69-8254-01A	0.060811279	0.023188542	21.0364101	3.268464821	0.012061643	25.18319748
TCGA-55-7727-01A	0.110939777	0.051814002	26.81261021	5.20054295	0.029387884	27.27839567
TCGA-55-8513-01A	0.036501038	0.012640346	14.79872002	2.587534761	0.009948368	22.64389262
TCGA-75-5146-01A	0.072294355	0.014054903	20.48862553	4.092119219	0.00836736	34.7029308
TCGA-91-6835-01A	0.040309177	0.011838674	7.556680784	2.588207923	0.014449443	15.8616908
TCGA-NJ-A4YG-01A	0.079609339	0.028693268	29.80893148	3.851754529	0.012973005	29.08943812
TCGA-05-5715-01A	0.152223044	0.047918552	27.44769572	6.048584017	0.01909993	23.41756066
TCGA-91-6847-01A	0.163075815	0.012870115	26.64477384	7.898987229	0.007449062	13.45848367
TCGA-55-7570-01A	0.10725455	0.0040308	20.08348799	1.309021889	0.002685849	18.57162094
TCGA-55-6979-01A	0.04641711	0.020314316	10.10034053	3.322336049	0.009084073	15.38590446
TCGA-97-7546-01A	0.077341314	0.029573611	33.37416534	7.131086713	0.020696637	29.54376607
TCGA-71-6725-01A	0.618133948	0.093931041	185.3173418	49.09975945	0.021526583	26.23977134
TCGA-97-8177-01A	0.088357714	0.027525928	19.95932607	7.513690144	0.011681507	36.82656402
TCGA-55-6969-01A	0.054067234	0.029660248	8.109438217	4.052849317	0.007510611	40.00532077
TCGA-44-6145-01A	0.073182178	0.014314481	16.59843526	5.610732448	0.006202327	19.14746445
TCGA-49-6761-01A	0.080440442	0.029728733	28.03765931	6.114919308	0.010636896	31.67165127
TCGA-95-A4VK-01A	0.10308534	0.018947986	30.59452012	2.399011243	0.014135667	21.42113323
TCGA-55-A491-01A	0.08555797	0.041760668	22.64596027	8.073025418	0.01164532	30.94035358
TCGA-95-7947-01A	0.086454424	0.016783161	12.33805196	6.555457391	0.005749727	5.890704106
TCGA-50-8457-01A	0.075473795	0.029403895	32.14532075	5.332831591	0.018028733	35.41298648
TCGA-97-7941-01A	0.102601331	0.020597421	52.24599898	4.012113226	0.015164096	32.2218573
TCGA-55-A4DF-01A	0.025205226	0.013194353	7.362645919	3.259688334	0.008222254	18.45257649
TCGA-91-8497-01A	0.06708761	0.020721489	27.33408392	3.635108621	0.013504291	33.92218399
TCGA-73-4677-01A	0.151251667	0.026339455	47.29642197	4.844363014	0.010035355	15.51417956
TCGA-49-4490-01A	0.328899563	0.063497531	96.98186686	18.85166516	0.014403458	23.00911324
TCGA-MP-A5C7-01A	0.147877471	0.011523291	37.66055596	2.676012662	0.007964162	20.60429944
TCGA-75-5125-01A	0.059171656	0.024773219	26.95833861	4.677508287	0.016205635	32.47037756
TCGA-91-7771-01A	0.056762816	0.028667835	22.09534836	3.708958772	0.015980755	25.00728502
TCGA-35-4123-01A	0.059210491	0.016902334	12.30092911	6.00384393	0.007275297	41.54734305

TCGA-MP-A4SV-01A	0.052747354	0.010908462	16.72233607	2.949664835	0.008273219	23.72924041
TCGA-62-A46V-01A	0.147712121	0.020413213	38.84418085	10.24519713	0.014307324	48.60736931
TCGA-44-7659-01A	0.071950845	0.020954588	30.45701509	4.197454466	0.011892073	26.07542361
TCGA-38-A44F-01A	0.048551652	0.024839772	19.18636497	3.839322452	0.012647167	35.67539246
TCGA-64-1677-01A	0.055711965	0.020772779	12.59097946	5.186365796	0.00665566	24.47833088
TCGA-50-5066-01A	0.090930998	0.028093259	13.56846434	9.71582124	0.008059779	52.25623703
TCGA-L9-A50W-01A	0.139499449	0.071347797	77.76359327	5.996652233	0.020189313	37.67453762
TCGA-55-8302-01A	0.097219489	0.029333348	72.03868223	13.80063758	0.013297642	27.64144932
TCGA-44-2655-01A	0.107636808	0.022605315	43.50369603	4.41419544	0.012383314	24.58456899
TCGA-55-8507-01A	0.051151111	0.015200768	12.30196954	4.723644845	0.008181888	18.0699264
TCGA-62-8394-01A	0.114090136	0.050484862	30.55842865	11.54197388	0.014665756	24.63554846
TCGA-44-7660-01A	0.043591089	0.012488541	8.573141305	4.351154689	0.004937979	19.73728954
TCGA-95-7567-01A	0.104712796	0.034689598	35.58110151	6.967186083	0.011580547	39.65943712
TCGA-MP-A4SY-01A	0.11491389	0.022318444	52.8064242	8.177373629	0.015462298	33.047861
TCGA-44-8119-01A	0.052699648	0.02388604	12.00348904	5.137110231	0.012706467	48.70191046
TCGA-69-7974-01A	0.092389675	0.016499014	26.25759506	6.360921185	0.013367569	23.11719018
TCGA-38-4629-01A	0.076696543	0.043892295	26.17028698	10.39111111	0.012347665	47.13285266
TCGA-97-A4M0-01A	0.046371334	0.022023824	31.48652569	3.08176705	0.010284303	25.13479793
TCGA-86-8671-01A	0.031283679	0.012806688	10.84480965	1.955151988	0.009229522	26.23897134
TCGA-91-6848-01A	0.017513784	0.001285415	2.135979211	0.241670364	0.001652329	20.1671595
TCGA-55-1596-01A	0.116060463	0.029456219	20.97224958	3.149638997	0.008072357	21.232635
TCGA-49-4488-01A	0.20299099	0.042138606	69.19098525	12.62135374	0.011504436	34.09840352
TCGA-55-5899-01A	0.195820419	0.022945423	25.81269441	3.232260524	0.007213798	24.21558483
TCGA-97-8547-01A	0.168737462	0.036670092	70.35116654	10.89404366	0.018681243	46.73091748
TCGA-78-7154-01A	0.178316794	0.013995997	34.75022668	3.790613728	0.004697029	10.85266537
TCGA-MP-A4TI-01A	0.026919201	0.005405187	7.6481717	1.220954769	0.004601575	26.55144989
TCGA-55-8616-01A	0.265368742	0.04596156	54.72400816	18.89712067	0.017377904	22.78904746
TCGA-55-A48X-01A	0.066299615	0.022679528	24.33266112	4.307624096	0.011456076	17.24714805
TCGA-44-6148-01A	0.062385243	0.013316902	21.72130457	3.445683479	0.012958393	27.96380667
TCGA-55-6986-01A	0.031689838	0.006803869	22.43567728	2.1235859	0.004871222	17.28482989
TCGA-78-7153-01A	0.177588727	0.070752324	67.63067036	6.561013511	0.017007958	24.87955794
TCGA-55-8207-01A	0.125049127	0.054970868	43.45751152	17.77484023	0.023944293	42.0823789
TCGA-MN-A4N5-01A	0.055465812	0.031092507	39.22461175	5.080543627	0.017318419	24.28839948
TCGA-05-5428-01A	0.145979993	0.022421025	21.31550501	7.291100558	0.007488592	22.7525661
TCGA-69-7765-01A	0.084860969	0.049196186	15.85533336	4.880874132	0.016577266	27.97172901
TCGA-93-A4JN-01A	0.042748874	0.018881209	18.19339358	4.353440029	0.010916655	24.01783514

TCGA-44-7672-01A	0.084830343	0.068311882	28.32621525	9.276299306	0.019191742	44.59003085
TCGA-55-7914-01A	0.055420434	0.011394037	10.96567578	1.10958566	0.00693929	16.84854105
TCGA-55-6543-01A	0.117567244	0.047246893	68.69407805	19.12832767	0.012015097	27.88952844
TCGA-L4-A4E5-01A	0.085488201	0.033261269	19.18773865	11.44012296	0.011820927	28.18124348
TCGA-50-6593-01A	0.304558802	0.030783455	55.38684894	10.50737537	0.013293031	28.67772759
TCGA-05-4250-01A	0.084460765	0.025813512	28.42409589	7.40650799	0.015650037	35.0855882
TCGA-86-A456-01A	0.13106127	0.054838488	38.0015785	7.570720403	0.027659182	38.66506595
TCGA-55-6984-01A	0.092964249	0.031355194	31.9794799	5.136991699	0.009906809	29.3143075
TCGA-73-7498-01A	0.149146956	0.018687788	51.35344535	4.025078773	0.012901683	25.09248788
TCGA-78-7540-01A	0.272719608	0.100916211	104.3891584	17.03506676	0.022265047	38.71877756
TCGA-05-4384-01A	0.098322201	0.013492083	47.69888059	4.023752369	0.011239017	19.84831888
TCGA-91-6830-01A	0.203506132	0.041972365	79.38124828	16.13229944	0.021779659	30.45542536
TCGA-44-7662-01A	0.13753762	0.03222637	20.87418689	14.08823671	0.009574038	25.05069603
TCGA-49-4505-01A	0.095658021	0.088652828	42.76535378	13.61819854	0.017007853	45.45313257
TCGA-86-8075-01A	0.226184451	0.03808732	33.1438098	14.49958112	0.015517508	23.87019192
TCGA-64-5778-01A	0.092543467	0.028706307	22.35604305	11.68272742	0.011383146	30.07428995
TCGA-78-8655-01A	0.077923308	0.017029189	25.44356041	5.585011025	0.009869138	21.67866717
TCGA-55-8621-01A	0.049260866	0.024527554	18.91871964	5.254804997	0.015977614	32.07968927
TCGA-05-4425-01A	0.133213005	0.035861687	27.1356809	7.67945593	0.013663259	35.4133575
TCGA-NJ-A55R-01A	0.188666038	0.100613904	56.97103997	6.542661612	0.020455487	32.98730485
TCGA-55-7574-01A	0.045971614	0.012799587	13.3717439	3.197251722	0.009542312	35.10181496
TCGA-99-7458-01A	0.087260316	0.023028278	24.58574781	3.592909571	0.016992396	23.51748394
TCGA-49-6743-01A	0.203191767	0.009762378	30.33075132	8.377193556	0.004990269	14.06451225
TCGA-49-6745-01A	0.060735818	0.016509726	31.61877148	4.3415498	0.010340031	19.71481848
TCGA-44-2657-01A	0.077924821	0.033597852	25.85470564	7.122405473	0.015092476	32.83500366
TCGA-67-6215-01A	0.075801786	0.022054287	27.67763136	3.964754854	0.012091675	26.21442727
TCGA-86-A4P8-01A	0.035140568	0.009306398	16.08870829	1.760781685	0.010578217	28.53754447
TCGA-97-A4M2-01A	0.04091469	0.012646143	15.92817094	1.957697018	0.00931605	24.46646018
TCGA-91-6829-01A	0.170551251	0.013745609	22.52747025	6.536609897	0.007900135	18.94647998
TCGA-NJ-A4YI-01A	0.048145167	0.032843538	19.06851104	5.828130123	0.020296894	33.77190229
TCGA-44-A47B-01A	0.053979823	0.023056642	27.03403752	5.13850544	0.019697436	51.94297542
TCGA-55-8204-01A	0.06770886	0.007308294	6.793559328	5.593275351	0.003542025	8.198964304
TCGA-75-7025-01A	0.051212684	0.026195674	32.00625816	4.413468224	0.015756288	30.66321615
TCGA-73-4668-01A	0.24438591	0.074674809	37.98871873	17.24478832	0.013048001	29.88029684
TCGA-44-A47G-01A	0.048887151	0.019935354	18.97378997	4.487403725	0.010757363	23.04293032
TCGA-55-6980-01A	0.185523291	0.092005284	69.01017789	19.19887497	0.022883939	41.3971845

TCGA-44-A4SS-01A	0.131894195	0.01698741	40.74534036	5.427512056	0.010172773	15.14953784
TCGA-55-1595-01A	0.094589591	0.012013281	34.33199744	3.199815658	0.010483362	33.24066094
TCGA-55-8619-01A	0.07071326	0.040249767	28.88290618	5.954532332	0.01561864	32.26922245
TCGA-97-8171-01A	0.041806319	0.013066276	9.319218167	1.178555677	0.004067911	15.85076459
TCGA-50-8459-01A	0.122891404	0.021794089	31.54875975	7.708263838	0.011928414	55.03985748
TCGA-73-4675-01A	0.31469219	0.02923126	98.77093352	20.36375518	0.011091905	25.91912974
TCGA-49-AAR2-01A	0.045241225	0.034666709	24.60954158	4.926178435	0.009239058	24.65487781
TCGA-69-7973-01A	0.045470252	0.013173407	8.696510093	2.39304726	0.008915246	27.93217884
TCGA-44-5644-01A	0.369012086	0.025430307	46.399693	8.379931034	0.012140284	40.89748466
TCGA-J2-8194-01A	0.056353219	0.007086116	16.85805651	1.454061074	0.004923062	15.5944628
TCGA-78-7161-01A	0.081444555	0.03677866	23.16524062	7.178672106	0.019184138	32.33134423
TCGA-49-4514-01A	0.154404328	0.055438924	27.8334399	6.117381697	0.0098334	32.89684478
TCGA-MP-A4TE-01A	0.102821908	0.053142832	24.4258375	3.728491966	0.013584736	26.94620774
TCGA-MP-A4TA-01A	0.063476854	0.011840235	14.10972379	2.860900716	0.005959234	15.62149265
TCGA-49-4506-01A	0.029518164	0.0034892	7.657170046	1.368259645	0.00178386	8.408498826
TCGA-78-7220-01A	0.178966295	0.003965108	32.75848117	1.960464403	0.003946637	14.29714452
TCGA-05-4417-01A	0.076979736	0.024883333	15.57577954	3.151185109	0.011869741	28.66171259
TCGA-55-7910-01A	0.035501011	0.016493577	8.606054745	3.190477785	0.00669298	28.05915979
TCGA-75-7027-01A	0.090871331	0.006675883	19.03335357	1.523914517	0.003250868	21.80526454
TCGA-49-4510-01A	0.240663752	0.062852583	108.5010668	14.39256915	0.02450424	55.39549423
TCGA-91-A4BC-01A	0.024250708	0.011518908	4.386202964	2.276109133	0.005808266	27.9891243
TCGA-55-6987-01A	0.020173509	0.008803736	3.871502277	1.834937953	0.003579091	15.60244709
TCGA-55-8508-01A	0.051416958	0.043607073	10.37544999	8.100571174	0.010263258	21.7853635
TCGA-97-8174-01A	0.075728664	0.013213666	29.03474229	2.360069503	0.010238798	32.08769733
TCGA-05-4390-01A	0.072793302	0.032961465	16.73888412	5.237246693	0.013634604	31.59845824
TCGA-55-6975-01A	0.180291785	0.013254707	32.46313453	6.42523787	0.005466674	18.34960823
TCGA-55-8094-01A	0.570073859	0.038969643	31.57325863	14.56745356	0.01314898	36.9288694
TCGA-99-8033-01A	0.075751198	0.009020151	16.42071778	3.857586549	0.004321889	16.01287706
TCGA-MP-A4T7-01A	0.063155475	0.014208003	34.79117379	2.076273814	0.007798686	31.09397098
TCGA-44-3917-01A	0.04999022	0.023249033	8.316795623	3.28130846	0.009329184	18.22701825
TCGA-50-5930-01A	0.209448179	0.037228839	35.70018247	15.53394391	0.010836822	25.21392712
TCGA-86-A4D0-01A	0.139756363	0.02103458	17.64747125	1.301607715	0.007481269	38.07759664
TCGA-55-6970-01A	0.099601531	0.011310482	21.80554762	3.587322888	0.005307838	13.75578682
TCGA-86-8585-01A	0.056480125	0.016256927	11.96446453	4.456651743	0.006771638	13.61980086
TCGA-64-1678-01A	0.084222808	0.022623788	15.71904945	3.19592584	0.005925028	39.49005463
TCGA-97-8176-01A	0.105485385	0.00979979	25.24568552	3.048711046	0.005058057	20.30973028

TCGA-05-5425-01A	0.102579889	0.01862121	13.63898775	4.180418941	0.005338859	20.94733155
TCGA-86-8076-01A	0.020741844	0.006416487	5.885535338	0.850332411	0.003958277	16.56076359
TCGA-50-7109-01A	0.060676956	0.020368106	18.10347461	5.074165605	0.013118068	27.07698886
TCGA-86-7713-01A	0.142816049	0.006530331	27.17820989	5.354957868	0.007157097	16.11236057
TCGA-05-4396-01A	0.081400032	0.014922247	24.26709582	1.7563109	0.006429062	19.91286171
TCGA-69-7764-01A	0.146840082	0.070088671	40.87654343	12.62580211	0.026537232	27.36996516
TCGA-55-A48Y-01A	0.097086433	0.012029539	17.04668842	3.56605474	0.005827333	18.09420818
TCGA-49-6742-01A	0.106260165	0.018366892	37.26020591	3.576066586	0.00698105	17.73466464
TCGA-49-4487-01A	0.055038246	0.01809857	9.45139668	8.348392493	0.007492863	23.52979065
TCGA-97-8179-01A	0.323569855	0.037649776	49.86495023	9.541460608	0.018642018	19.38874424
TCGA-MP-A4TF-01A	0.095672041	0.011746537	33.02708912	4.274163809	0.006418597	26.76084009
TCGA-62-A471-01A	0.049198665	0.004602546	9.457644107	1.029411123	0.002151177	6.862410714
TCGA-62-A46O-01A	0.195690413	0.010237618	18.55227063	4.032505453	0.005402129	13.63121758
TCGA-64-5774-01A	0.041823581	0.0332456	12.6907901	4.207309096	0.01360655	23.188261
TCGA-86-7955-01A	0.127161319	0.013097063	19.94980034	9.650451355	0.006285465	10.93069011
TCGA-62-8398-01A	0.055607582	0.00669821	16.37096662	1.443770565	0.00448027	19.50567537
TCGA-67-3774-01A	0.093495478	0.016855668	45.58511243	5.652335281	0.013742979	45.7722666
TCGA-78-7166-01A	0.091973029	0.036486249	29.97240682	7.029814621	0.007202652	21.54885499
TCGA-78-7159-01A	0.042653392	0.023968339	11.09441857	2.002899052	0.01138698	28.81099999
TCGA-44-7669-01A	0.03867222	0.006713142	6.603613172	2.130071695	0.005710686	23.76184353
TCGA-50-5072-01A	0.068115232	0.00595558	15.14989278	1.245329855	0.003680423	16.36731061
TCGA-86-8359-01A	0.035246101	0.008726184	9.807370486	1.802882034	0.003886651	12.3582163
TCGA-49-4507-01A	0.086251882	0.012976964	18.93332515	3.570526598	0.003385833	22.16323211
TCGA-78-7536-01A	0.029499611	0.010366431	5.573635283	2.25827469	0.004749694	18.08187383
TCGA-62-A470-01A	0.087583813	0.013647835	21.17573325	3.141258149	0.007683138	20.69915477
TCGA-55-6642-01A	0.081402523	0.038923039	20.50861286	8.864206221	0.013056473	25.17598877
TCGA-78-7633-01A	0.281796561	0.055863996	104.3142963	16.19118028	0.017589483	22.51223453
TCGA-97-A4M5-01A	0.055197935	0.00916879	18.64131359	1.848146543	0.007131453	16.68222272
TCGA-NJ-A4YP-01A	0.027993604	0.003954851	5.799824595	0.586060163	0.002691273	11.28585804
TCGA-62-8399-01A	0.121754256	0.014303758	41.0729596	16.81684671	0.006936735	16.58639751
TCGA-50-5051-01A	0.084182772	0.031488694	25.54748548	4.471459449	0.008476122	35.46228144
TCGA-73-A9RS-01A	0.034821787	0.005660479	7.687800915	0.636902357	0.002255406	20.27656324
TCGA-55-8299-01A	0.024607505	0.005389958	5.281258967	1.070652522	0.003906938	15.66752514
TCGA-NJ-A4YF-01A	0.173477714	0.042105142	38.1311972	19.75232761	0.015307582	54.06973414
TCGA-MP-A4T8-01A	0.093382485	0.009551235	23.75584361	3.808905757	0.005401268	24.60168015
TCGA-55-8092-01A	0.047571188	0.015548149	8.279994516	2.433566681	0.005462289	15.48939578

TCGA-55-8615-01A	0.065174481	0.016557422	23.19463335	2.124692008	0.006762339	27.01213581
TCGA-05-4415-01A	0.031716674	0.002618196	4.419256993	0.605104154	0.002738429	14.884249
TCGA-78-7167-01A	0.244648547	0.015911042	106.3289567	5.365716134	0.010008802	19.95107205
TCGA-62-A46S-01A	0.103871747	0.0344292	42.10307551	3.589733799	0.017093651	31.04758139
TCGA-MP-A4TD-01A	0.083743727	0.022426739	20.30544157	3.72863919	0.009260307	30.11586268
TCGA-78-7162-01A	0.119428647	0.031798264	37.14423811	6.292680368	0.012737332	24.87112084
TCGA-05-5420-01A	0.034885962	0.019026425	5.740195067	2.122817023	0.007532398	29.68773547
TCGA-86-7701-01A	0.044189744	0.01629777	6.921325079	1.35572974	0.007801297	22.08141788
TCGA-44-7671-01A	0.237336652	0.016116973	85.6741193	6.542992983	0.00715428	17.59851738
TCGA-80-5608-01A	0.061420309	0.050408376	21.25590572	4.500738661	0.016628389	30.67841911
TCGA-99-8032-01A	0.059112162	0.035900625	19.99694444	3.447401478	0.011626018	39.97002869
TCGA-78-7150-01A	0.118844025	0.007462283	18.9320931	2.196183526	0.003838773	9.80353454
TCGA-69-8255-01A	0.03607807	0.009525796	6.046757598	1.638163905	0.008804544	29.3507863
TCGA-50-5939-01A	0.087698616	0.01084051	28.90218021	5.26559627	0.00447834	18.4081056
TCGA-86-8054-01A	0.031314973	0.004333899	6.005157627	1.286506729	0.003143806	16.37383513
TCGA-78-7160-01A	0.080349529	0.007602339	18.63530999	1.682211449	0.005013762	11.35589474
TCGA-4B-A93V-01A	0.025711302	0.009805976	4.752851417	2.281614212	0.003427141	14.63891777
TCGA-05-4418-01A	0.070047365	0.013678723	24.02507996	5.951262313	0.005618263	15.11213181
TCGA-NJ-A55O-01A	0.094537885	0.031573616	35.03179452	5.571542802	0.010812181	28.46263182
TCGA-05-4422-01A	0.06828692	0.015005739	17.87100426	1.220336924	0.008550546	17.30522424
TCGA-93-8067-01A	0.12950714	0.022566367	72.53261338	6.138862259	0.009423807	36.55832205
TCGA-95-A4VP-01A	0.031918602	0.035174895	10.96303689	2.57190361	0.013338995	39.30109724
TCGA-55-8505-01A	0.160531849	0.008531521	43.52514886	5.481930307	0.005784716	15.16212712
TCGA-73-4670-01A	0.103575279	0.006887666	20.59628173	4.130790691	0.002482605	13.91155064
TCGA-64-5781-01A	0.079868305	0.016094122	13.82712355	6.29790182	0.003514412	19.79059785
TCGA-73-4659-01A	0.049768124	0.014017609	14.52464818	3.389928764	0.006060433	27.73348669
TCGA-97-A4M3-01A	0.088381033	0.02559206	38.38183864	6.245281068	0.009429137	21.14195562
TCGA-55-A492-01A	0.138974561	0.056098054	55.21688834	6.735201562	0.017348565	27.12623945
TCGA-75-6206-01A	0.194668848	0.01815538	91.15006477	5.975401058	0.010486607	27.31369495
TCGA-78-7148-01A	0.152505485	0.032635647	76.26057778	6.421111873	0.012702969	24.8339076
TCGA-L9-A5IP-01A	0.085154187	0.012952472	31.79699991	5.186822897	0.003477197	6.196895168
TCGA-50-5936-01A	0.104269574	0.011009712	40.91516242	3.908676424	0.005632976	18.47289403
TCGA-91-6831-01A	0.029296557	0.007152679	5.490187213	2.054375291	0.0040936	22.91935404
TCGA-69-7763-01A	0.130692154	0.029244284	62.33044603	9.527199008	0.014924298	37.46933069

Navitoclax_1011	Vorinostat_1012	Nilotinib_1013	Olaparib_1017	Axitinib_1021	AZD7762_1022	SB216763_1025	KU-55933_1030
14.08088846	4.508673393	48.00912175	94.17546352	28.76499413	2.275366074	222.3666383	110.0290374
5.621371596	4.48270655	36.77576413	60.66621119	22.62009617	1.486212525	175.0997809	73.40354284
2.527235834	4.474624396	44.675768	184.9658321	25.35498254	1.455854666	211.6019776	100.0559922
12.0477653	6.766111896	24.49647248	49.82679596	24.6464909	1.317747865	102.7767871	70.69723575
22.57239615	4.043297004	74.13156892	133.1183101	36.54887128	1.8571517	263.3946751	99.71218958
8.52376572	5.516915972	26.2463256	36.56808703	19.81887342	0.712735026	126.7253947	78.87043762
5.035341572	3.713096916	27.13792497	68.20935976	18.14682109	0.561405723	147.4832442	79.50064085
21.60704845	5.481373536	52.71101283	123.8664151	20.9641445	3.215641098	175.486392	83.20154712
12.38686776	7.413898067	68.73101976	136.0463314	27.43732538	2.474673655	242.2283269	92.55461713
2.356024383	3.288747074	16.04929792	39.05358629	15.98867583	0.705293127	130.3762105	74.37128285
2.275762287	3.071351843	29.04608396	47.03058302	18.49370477	1.039667596	146.0753498	86.62227232
7.02190836	6.66174391	31.38873482	99.13595711	16.59561559	1.028929792	161.3310654	80.11095423
6.537030366	3.97365079	19.65980477	93.14038962	23.78397872	0.55024325	235.0472964	95.04073968
8.29273201	8.539872057	52.23743709	64.09724691	21.36339337	3.870667211	115.163655	72.19013499
4.592030169	3.211531645	32.26739301	87.17512362	25.78138066	0.501273189	167.1224621	82.2853811
8.291946481	3.645480564	46.62486033	95.40549748	17.04161971	1.713831357	166.4865282	88.80100227
3.118787504	1.342792254	13.64771922	29.0444522	23.16318876	0.308515803	336.8623963	111.9077603
19.24291004	8.128517085	43.19838592	135.9853191	20.54744713	2.154457565	210.2578603	80.15220085
2.828851862	2.941978127	54.3589954	139.3465298	27.7990585	0.467869285	203.411363	89.55884311
3.0888776	3.006291856	19.81834864	48.45078488	20.78787321	0.281184102	155.2356589	79.20873701
4.408446707	2.885220488	54.66298972	74.08941474	15.89424943	1.755752057	142.8384298	85.95426727
5.462800061	4.216967654	74.29714021	179.7527589	29.75250225	4.937083677	393.1422283	127.3958494
10.0469905	5.34197845	40.79296018	71.08200149	24.06229197	0.923447476	181.0504145	89.74134446
10.11827126	4.473024344	76.32350321	119.7472623	23.68620338	0.78434485	273.5901689	89.50914309
5.315033234	4.26553612	48.86615436	77.34530992	23.77686937	1.050754429	191.0107701	83.33650712
11.60645748	4.208937293	50.01629417	80.02866606	20.85679009	1.26564436	180.4106593	83.59765726
10.31071146	5.262872543	59.13079884	185.5343299	21.49446418	1.976616201	172.0202617	92.05694149
13.61570898	4.844098688	96.27889407	120.2748058	24.19980984	1.483043437	180.2979442	86.77813199
4.621907543	4.346481463	37.14060689	78.00681414	24.35552316	0.695630408	166.7129756	77.7574805
18.26890884	7.281292638	18.95517054	86.5132393	22.05068728	0.586285454	243.8949372	98.11241406
4.900975746	3.522874456	22.70125753	81.73971834	16.25763091	1.885972376	144.753623	88.70963171
8.649168049	4.910843154	38.84215652	159.5473266	26.53208304	0.641083544	184.6797728	83.07937649
16.27225822	4.422988547	70.74534981	212.9045469	26.64031519	1.303585437	239.464485	92.36117802
3.875837986	3.527633977	18.67550143	45.89762489	16.10654195	1.189942976	162.9519183	81.4496672

6.727294567	3.684809467	55.42242814	86.99610906	17.97562714	2.533268006	220.178902	90.88592854
38.62175464	6.108088455	68.25017171	174.3610851	29.25429643	2.102363214	233.667653	98.40165014
5.945445175	4.239599555	26.55859555	45.90222357	32.25519417	0.18372186	196.8857866	81.89388199
28.00728464	5.537855348	72.78268669	217.3635629	24.57571627	2.477735913	203.1413142	83.23954068
7.99360061	4.371202309	35.28202265	93.25988905	19.5023604	0.929610589	169.8881471	85.08501098
5.309239696	2.36265384	28.95800115	88.11139532	26.63482967	0.657096359	361.0422421	110.4374225
13.27665762	8.835878567	12.6325611	119.1033876	30.87211869	0.640360734	207.5705125	83.56597819
7.931691875	4.531509725	49.0022252	64.87212162	18.12213631	0.936509027	121.3025322	65.68742238
20.17818343	3.715676756	34.71423066	82.77285211	28.41205769	1.544984306	236.6525528	94.09819508
6.779231285	4.223553745	35.62251941	84.9369172	17.27654433	1.51351466	161.5304603	87.99605001
7.870253965	3.745486219	29.3101745	45.36474944	26.98113847	1.335981909	246.8939698	99.20451901
0.696040689	1.181696021	8.637640056	32.78753587	18.4226385	0.481258768	283.1579735	109.8362852
3.347210578	2.45100107	27.90308601	56.9395881	17.46194567	0.739896028	149.0750397	79.85379479
14.15547597	5.941020806	40.56049815	96.07822749	20.33696648	1.481611526	171.6196828	86.4724416
3.719838648	3.865843068	33.55574252	41.64858124	20.52296102	1.055304385	152.535575	82.93303691
15.70662243	3.543552868	33.86246654	65.74694801	18.81066404	0.52476314	181.9961478	81.17532385
1.71504129	2.262376341	14.38046375	23.92302533	14.47056826	0.556986694	121.4798317	76.03921311
3.581208089	4.381999125	50.21966714	103.6492267	20.72927003	0.731062167	140.9550001	77.7967845
10.88385106	5.388096005	45.86218717	175.6429849	22.13658044	0.792254021	166.2065154	82.55095947
2.388327153	3.888350828	32.84847795	65.9544929	19.13284061	0.542003123	186.0191871	84.11671667
15.42808036	4.716462587	13.06700727	65.27661325	18.84450448	0.663575667	140.0295304	76.54521887
54.37957771	6.026488404	49.16242564	134.6461574	33.05027187	0.470219946	182.6687856	81.32745012
5.60361181	2.684979311	28.64867594	54.6091235	17.00977027	0.658086445	177.2104369	84.51109882
2.906550921	2.904668453	26.99620645	53.2834346	15.19367992	0.570513581	137.953798	78.93992571
33.75088219	7.800649917	64.70231071	129.2521908	42.57714174	3.281276049	282.1771024	108.4842572
7.461639438	3.332662715	46.91587768	94.07463258	20.84662663	0.871325111	307.796517	97.81099774
19.82691911	8.57429187	89.81782274	147.0092428	27.85787074	4.927977043	204.5790526	87.81896415
6.091765473	4.913911844	49.08569714	80.2059211	21.98750169	2.193043548	168.733128	89.95997826
8.022064395	5.434846719	77.20583117	263.2146765	21.62257406	3.659457592	244.7430517	95.40414162
5.22981793	3.70533492	44.40120584	99.72309712	26.33949681	1.218630539	265.6305866	87.2198096
3.713905033	3.322187848	18.92664095	37.87006999	16.39613679	0.699714492	132.8200401	80.67915197
3.723914001	3.916983504	22.98685198	35.27464719	17.85947495	0.491932745	131.8032283	71.59740879
13.0918545	4.958822258	95.05741468	97.47905731	41.9127239	0.468099524	265.3435516	93.42899845
29.82483228	7.899983405	106.7331083	197.2678846	44.96036556	6.626032688	248.7179691	103.0889103
12.27791905	6.408706623	45.22488843	149.8283364	20.02954623	0.864364439	168.5831638	75.47211426
3.488203124	3.946752772	19.6373772	42.905466	18.63298702	0.512460292	194.7916848	92.53819095

7.232385588	3.085945246	41.07068294	226.9207997	21.8683182	0.685827222	251.5342687	92.89268318
2.490323033	4.575140254	26.69879234	38.9136966	17.39039431	0.918702691	248.8379097	84.96893309
5.811845717	6.995486547	66.08832457	149.1287204	23.31495597	1.586033445	202.5955357	86.41688267
24.0142488	8.583465302	150.355181	178.3767694	47.86195617	3.289355834	327.3111429	107.1699841
4.932770872	4.418301127	38.49829721	67.20084353	24.34188064	0.858008639	164.5748782	78.4397217
6.043298819	3.084441519	35.81415851	32.55974222	18.17052715	0.58392587	125.7057533	72.0450352
6.787077433	3.199059382	20.22959862	41.8334998	25.13640872	0.679427642	200.4728998	92.79208573
9.180172857	4.589724373	56.3325762	64.11156253	27.18705622	1.48548421	217.89198	91.55934692
4.665304076	1.436640337	27.20608739	103.4951793	19.4204283	1.634371848	258.9071712	121.499274
4.23680832	4.590209035	28.57653703	80.774498	18.24453189	1.507054495	202.581519	84.27172699
18.5079981	5.812744334	38.32256343	144.568739	30.83405324	0.695930689	157.1553426	76.24086726
11.40868703	7.930318033	108.8836214	143.086823	23.94219583	2.600143535	242.5586966	80.85254966
1.673719562	2.226038815	14.17812571	61.21336789	13.81097017	0.667182579	153.2805959	83.79632107
5.978627863	3.369070556	16.92422421	37.63649217	15.70798364	0.515492488	118.0985945	70.69858939
10.04196674	3.776262843	57.14772106	79.69661409	21.12581928	2.859409004	199.1613759	101.8603046
3.609752181	5.023887801	43.14991431	80.18872757	17.08519604	0.980214042	218.1876993	91.19228483
0.435190475	1.624457892	17.69746173	20.14159641	13.46712156	0.403866323	213.1188253	83.55651829
10.30677508	5.9585813	39.54028046	52.62007608	13.62908496	1.220934815	150.029594	84.35597648
15.05290623	5.742472716	83.68958632	134.6060435	28.13831856	1.278305118	203.1577013	90.73725902
13.43559349	5.351023708	46.24696691	54.35807622	25.88211936	0.484932593	169.6844495	78.98427243
5.319872481	3.69655737	43.22885969	75.23273624	24.13855476	2.057334948	178.5674297	94.61696736
3.19623303	3.321276716	22.04849376	46.24128706	11.97334616	1.073294573	168.4167566	90.78321526
7.872884433	4.825736432	32.76439813	95.00182162	26.47286976	0.917590512	186.4411749	92.80798545
6.91646712	3.63480463	33.71407558	82.11366266	16.13848351	0.971760375	173.7693441	90.16728557
8.208359982	3.406921325	42.3993812	57.00276143	19.91536515	0.858923238	170.1013581	97.88537335
11.46897053	6.230940474	24.11638481	70.96314722	14.0269255	1.225955181	103.2761498	63.5207029
3.24624686	3.061296584	32.6137697	45.01921365	21.05319798	1.102725869	200.1288329	88.6574506
18.52545169	5.710886342	39.7359036	48.19452306	27.48438471	0.423228578	222.0293867	80.88985873
4.643623752	4.823970083	42.66125177	70.49345956	23.65442946	1.215685578	150.555025	79.77961359
6.353226366	4.184681068	28.50822902	63.55997725	22.2831635	1.557697086	90.248814	68.70704846
9.924116478	4.795298339	48.7163606	98.33914638	23.29606164	2.547269443	305.0551461	101.6472704
5.465816085	4.364914785	49.72004533	91.47022043	21.41155332	0.794570654	184.5116876	84.37192195
0.814915806	2.036554361	13.65134074	26.78933548	16.02234956	0.429721107	304.6654447	118.2978716
4.58860185	3.29880883	20.9097089	42.72206094	15.3343307	1.344299384	164.7354036	94.8748664
13.42193638	4.95326522	34.76045445	44.26266175	56.206923	0.565779078	326.5430847	110.5982335
7.112798686	4.358411015	33.5284584	125.1049657	25.80413477	0.905667545	225.6608789	91.31949735

9.09993942	5.033314262	57.4298336	61.19081829	18.82796201	2.345490372	176.0370834	81.19884661
7.519141811	4.232222207	39.33131925	61.55193072	14.21051327	0.77772602	161.7232441	77.8504595
3.177492672	4.63276411	18.45542802	58.6234592	15.14711459	0.845364252	141.5034984	80.12616411
14.67154153	11.37252674	121.949201	184.6021452	39.38109953	4.887050055	285.5015283	110.3377533
5.017093445	4.509715762	48.78857231	102.2864273	18.28998233	1.530091686	155.4051311	80.0144939
6.797556474	5.938235681	36.64411063	62.12089718	29.09469884	0.373960073	161.335796	77.87352655
10.96515197	7.028609531	111.7700083	166.9808608	24.75956644	1.934555407	166.0218873	78.15402076
3.295658144	3.291315697	30.35035343	69.81189947	20.76159694	1.116202196	203.5001472	103.2345742
35.11569172	6.444626465	64.7742694	108.900221	20.64449291	2.161074612	169.0963875	74.350755
3.334464684	3.598973222	40.12984226	57.04607995	21.50548866	0.924359226	154.4437168	81.93702579
6.242753756	4.26783452	28.58492491	54.11572251	21.83420002	1.241565616	117.9176692	85.57480973
18.31320457	7.023401988	32.14282897	114.7904943	18.21513961	2.348563041	153.7664833	82.66734452
23.77179414	8.511794689	84.84081618	121.2592834	24.61003863	1.107295584	166.1014518	76.52724033
3.745924248	3.365530762	26.63146672	56.37023031	16.83484996	0.593031511	133.2618581	79.29968859
1.426515879	4.738926084	44.83653382	16.87634474	17.21348338	0.688104131	410.4266706	111.8618339
7.895240387	5.530969281	35.76538939	101.1630247	25.89570174	0.738889025	171.3767274	84.47654748
7.683555228	4.019144434	43.44928243	79.12358041	23.98363661	0.525264925	217.5361095	89.08672368
2.632942002	6.555179195	30.37293983	88.80131097	33.73465369	0.641325658	164.1489308	85.72291447
8.541908933	3.307059068	30.79185219	76.16186547	29.65654493	0.540724353	183.1963148	97.49053612
8.423556599	4.633793717	44.91185048	83.61713823	21.91501589	1.129487435	168.8057602	82.63990792
7.257046726	5.068504986	48.24293922	128.3407616	23.17042602	1.930926356	212.1529897	92.88893588
4.912690363	4.361739284	26.47587765	102.9319261	16.75598566	0.990679479	160.0683889	85.09060928
1.953265641	3.591024419	24.09900536	66.29116442	18.88103549	1.046174943	186.4519564	89.3897234
10.62130771	6.290280699	56.1896597	104.8190673	42.85261976	0.538436904	276.2766787	90.30478204
1.958924177	2.661653029	17.59602181	52.95233308	20.76387161	1.366714097	140.3369744	82.3467051
10.56343831	7.485510244	60.86500672	59.10584794	29.14954118	0.459650406	193.1679079	76.86738703
19.78596351	10.7748406	111.354357	178.3765303	32.08363474	2.319808278	230.8059713	87.41219555
8.584533244	3.712979507	22.51206419	51.1408998	17.40582888	0.582702463	128.4737608	71.28764625
13.77062086	5.489236736	47.8004321	70.68075814	18.38757831	1.032934655	142.0801603	75.22342565
9.357980438	7.845093687	68.66488037	81.1573101	23.31475358	1.806851866	128.1741693	70.642729
28.30889701	3.306651082	39.95478077	182.5069016	31.85912177	0.618603188	213.5195198	90.28725877
15.87629216	4.599310332	67.69659138	121.3967245	15.4186642	1.419239391	217.8135666	85.8454137
0.95551911	2.268416577	34.81415798	23.28354863	19.31547002	1.116532709	168.0671816	80.35478685
3.919685514	5.275902057	45.25754342	87.39103241	24.31530156	0.995886939	150.5705495	75.84931101
7.945683407	4.405576811	52.92961664	103.9111734	13.66251637	1.038443438	169.4097264	81.85367752
9.988280695	5.214854261	64.02859018	124.1440827	29.81718718	1.149061068	185.4547187	92.29275253

12.36427263	6.473923352	77.58709027	103.4934637	23.07722849	1.114974596	218.4937968	89.40303994
9.318540573	5.062396835	81.96395349	108.245608	24.93248768	1.793246189	171.8768042	80.95901582
15.21641252	5.777037037	36.85690911	134.2901277	27.8763006	1.551298061	249.6139701	96.65481936
10.05069637	6.117454648	48.94929254	83.35838319	21.7087623	1.279040011	181.4077079	86.73481869
27.56929162	12.87075191	162.8851848	205.0273751	68.83942076	1.886337126	505.4139585	121.8829521
8.619672552	3.561486667	19.1266062	66.78499295	18.58528852	0.624711028	193.6952046	89.72562173
7.683568266	4.63614246	71.01991247	89.99256298	19.90234888	2.785616064	224.350284	91.29535099
5.452098122	3.302432142	30.18287954	46.75455028	20.27346145	0.466098727	131.371665	74.12050027
3.627338062	4.206472212	29.28542945	65.29865059	19.48207445	0.454137436	160.2951016	84.88902885
2.857934615	5.628990791	43.47007045	90.07270084	15.54845805	1.14427148	196.2317752	90.29252673
11.19909202	8.152138717	91.13815172	170.7302295	21.94128789	2.385117855	219.0908948	101.5649083
11.81282198	4.821597145	56.55615357	86.01818231	25.2062573	1.283408242	189.3818282	93.01039701
3.561433925	4.15160898	54.30334094	63.59220211	29.87298692	0.807872796	236.2847701	104.9106493
3.083916124	5.873139296	62.29981589	52.3737232	41.18360254	2.567314322	231.8275223	98.83791
3.150296543	3.322727871	23.20641632	44.60006378	16.44740851	0.7203069	110.8575023	72.03734214
4.254926508	4.211929003	16.70896822	90.37124251	25.87126299	2.35928436	285.4157258	104.7784234
0.948764075	1.685746402	12.56500086	46.36285325	25.68384803	0.392600284	215.6695666	102.2870055
10.63282107	7.953807691	60.92407448	123.969211	20.59413746	2.879394767	275.7739121	100.1915313
4.69308382	4.608932712	57.14756387	76.45123898	19.20436372	1.296998694	166.0809928	85.56321088
3.627711878	3.055094305	29.51401082	35.30453112	14.8315539	0.846770529	156.9594182	79.11624694
20.71834911	3.2173421	22.38136956	83.08532257	27.5465855	1.03087598	297.931553	106.2219389
18.29785063	6.036552232	57.57171557	56.61043377	21.00238959	1.435782705	180.3924568	86.07184841
5.217850631	4.106338992	43.72741158	73.90301283	25.1463921	0.813127123	171.463382	89.91456103
10.346739	6.582012857	23.50006927	74.42286711	21.52894247	1.364217166	149.4065754	82.34479624
6.640525337	3.386321778	47.39061408	82.45584531	26.66424829	1.448879504	201.3850652	90.62898813
22.82900699	6.019610967	62.08401429	102.5087012	22.48669375	2.974538809	181.1152925	101.5421426
11.70034284	5.352242211	46.17321777	52.29798245	16.53256174	1.169651472	189.1080058	82.53120191
41.23016284	6.426095481	82.12941013	75.76808404	32.07695654	0.588826914	192.399716	75.26280954
12.19158106	4.107110506	30.43837587	52.5351569	20.27509071	0.464194694	149.4094727	81.83361539
11.39985544	4.59075128	18.72572339	80.41856508	23.70247251	0.491259021	175.8985852	78.47149972
3.70098003	3.354256944	26.14429627	39.44707896	21.89277206	0.947935738	144.6062288	86.92198186
10.57262614	4.641252411	32.69630908	56.73627292	30.42546771	1.558917281	305.2688016	105.734447
2.767358442	3.129889189	27.75331913	41.05333637	17.57690591	0.847950433	145.9891796	74.73832679
9.322848141	4.135493253	30.90811836	170.9903407	44.1382776	2.149217705	332.8168674	114.0454543
8.106314206	4.262697416	41.7549212	62.63071985	23.62728899	0.381373688	188.0380039	80.64801604
5.590226951	3.959096753	26.86909083	68.40547701	17.32353374	1.377751552	185.3051662	84.82844915

2.577874124	2.501280542	16.59890601	37.95078288	15.05163452	0.843598445	135.1574415	87.75237809
10.11251572	3.766426196	60.67597526	119.4855959	30.73791502	1.253901773	298.6228424	114.4155091
8.533130349	4.982914519	25.2826494	57.48795566	17.79129967	0.953764297	122.0369756	74.67222166
15.07242425	3.935001787	27.24281249	139.2860251	20.99608332	0.877089534	179.472546	77.83257826
4.285735416	3.323956299	23.2559956	76.10546912	15.81689682	1.052448267	169.5124527	89.34769543
41.38993756	7.031120169	51.89984326	155.3920915	24.1197234	3.350650228	181.0072515	84.78223983
8.560663019	5.952984325	85.68598253	101.087457	20.07151987	3.190792941	188.5679878	99.06714353
3.715736076	3.394357832	41.82918144	99.94154313	21.95701519	1.235245669	264.7044851	98.77366745
2.850001028	3.755778928	24.96099788	61.96748298	12.31957489	0.942844092	135.7484377	83.72643245
18.65763689	5.664861132	40.52600707	68.86066476	25.6039782	1.656114349	161.2199787	82.33215814
6.408096717	2.534144415	30.8695474	59.34754087	22.29613183	0.71595457	183.164884	88.92684528
4.559140515	4.539185228	39.59240673	62.86755561	13.79862548	1.00667024	150.378051	71.48759008
2.827316054	3.198080859	24.16172501	78.84996834	35.63985868	1.994667999	272.322572	109.6341484
4.759209085	3.673448528	43.43609892	75.8453046	21.81512227	0.858653276	204.9231739	88.15099233
0.469319329	1.703292008	10.70613349	47.172247	13.58455808	0.4595151	244.2772417	95.52322933
3.990067894	3.545583741	19.4374689	47.27550789	21.07725867	0.50092639	136.69206	70.69199098
9.545275458	2.504163743	34.37931658	65.34101183	15.93003868	0.933428086	160.2050508	81.61063657
9.77654735	4.992238404	53.79864308	87.77410391	17.43327387	2.493997395	269.2565848	113.8331289
4.211082763	2.90779422	26.65110197	48.68915834	21.01106374	0.644787863	149.7167986	83.32657901
2.843740685	4.151234742	23.76815496	47.3712558	23.89358079	0.709006073	259.1165552	89.93341033
13.08177351	4.389342687	57.52961014	78.64830148	19.11112588	0.853656321	170.510274	89.28740076
10.4749248	5.854984954	78.6253104	210.7647894	29.83742424	2.714701807	284.556693	96.79280273
2.715721444	5.381544989	17.73014447	43.18904266	21.6879981	0.484118708	297.4930873	94.30684728
11.34886492	4.540981362	35.4611669	62.75858278	22.80015806	0.70931441	136.5636427	78.34987286
21.79389099	9.8119098	81.12215358	133.0896649	30.63399157	4.298125252	254.5249932	93.78342568
17.99601866	4.407534516	36.10972319	118.7971531	24.61782812	0.806963007	264.5490428	98.12419646
19.24523226	3.318559297	26.92334864	137.8106893	37.35098345	1.705986796	320.8156536	130.1377391
0.359761161	2.139616612	5.824659622	20.70953536	12.01307371	0.591252531	221.2278357	100.9367201
2.559946657	2.291862596	11.8195548	36.72541284	12.29644128	0.851597429	125.2520852	85.38489833
9.805324956	3.443692069	35.67049093	77.90286308	24.10129585	1.139219776	200.5195252	98.59982959
5.808611264	3.810088884	34.72671482	58.70451176	19.10047508	0.529184843	147.9338664	75.25044072
6.056313277	3.597405728	33.74264577	64.20670197	19.72306127	1.319024335	164.3866681	88.56000596
14.99441287	6.714857404	66.19484534	94.19010048	24.32928024	2.03969888	208.4327774	83.98976963
3.701517663	3.771143664	24.73551717	50.52032047	14.28856709	1.125665725	144.2425225	85.1478953
16.02345186	5.468500919	53.13506304	80.14206344	30.19339747	3.309293167	223.9050654	94.95623656
6.552202124	9.313774098	41.47505874	90.1679505	15.08643516	0.928917184	282.6358657	91.28221669

7.051914912	5.97540518	53.42167489	107.9350471	17.20720713	1.01014362	158.1063034	76.27202391
18.97013344	5.631474389	81.2691671	116.714911	24.12007372	1.101770047	193.7994306	77.07709338
3.101855977	3.295365395	22.54151401	70.14680989	25.75350603	0.640587239	220.5941464	97.71974915
17.97360766	6.803336824	58.77348105	153.5505919	29.41100054	2.384276139	210.8309465	95.76822724
2.943675214	2.073287566	14.47225895	40.41315685	17.41098061	0.871911141	216.8131016	103.9293084
4.302214417	3.073629115	23.57506949	28.76966684	20.69718515	0.423978171	139.0856418	70.15576769
12.749806	4.162132078	38.08580608	125.4365522	24.70425585	0.84960653	148.4357018	76.90698289
23.93518249	5.636455946	34.12453825	101.560093	29.85075918	0.804117686	206.9648797	80.42298135
17.76544889	5.836188063	28.26028238	75.04885314	24.60903679	1.463628233	172.0787792	89.15493205
27.94101272	4.956382987	34.23337248	89.66190815	19.33776998	1.147763263	147.6663157	80.27759635
14.38266015	8.036782918	71.17970837	122.5204439	27.01647884	0.959349513	176.841434	68.32858497
17.36511946	7.362324125	74.16418446	126.6122047	23.25809212	1.207469107	261.6698069	82.61384035
5.186168425	3.011280185	45.33236484	108.6133212	24.1447514	1.648433145	229.9804642	96.63451621
4.557412677	5.341900117	40.9979885	98.11893019	17.02822458	1.279206825	198.981007	95.22212882
0.61904307	2.00134216	12.83785172	25.14954983	11.50065245	0.494913031	131.0079596	74.1397958
9.386852325	7.077512814	50.10746482	71.66510964	19.02750546	1.268962964	158.2426942	79.32310371
2.74970977	2.074326472	24.52448994	47.43975758	21.16640478	0.383004971	171.0255264	89.10723945
4.336057407	3.843633534	33.94015554	89.5559972	22.42486987	0.672452448	293.8699655	97.13848625
8.475795588	5.555693693	47.0352749	76.79118029	13.73671129	1.38492218	132.2486848	72.79287356
12.00359432	4.035718244	33.60551036	87.04970473	14.21041807	0.935674875	217.2211182	90.49369954
4.471153415	3.110699444	23.14317801	57.58425792	21.57380305	1.516311305	200.2569313	99.16759682
3.483743629	4.202044175	37.52135713	48.61526822	25.14453291	1.046787979	177.7379186	86.87262956
24.22618902	3.465786082	35.06589693	141.1455276	49.38372603	0.563743114	252.5262517	104.0658243
6.080487704	5.539779256	72.30298892	124.3596752	18.8680044	3.536192405	159.1249902	93.24230558
3.581536891	3.581602246	32.20250838	36.72055762	20.84549225	0.494454781	169.1427097	79.85594505
12.20853352	9.277820996	96.46476648	124.9738245	28.58557289	2.762287592	187.2482238	79.90860729
2.93144125	3.793989434	21.2472045	51.24581211	16.71674487	1.124512198	148.4536076	86.91391398
14.05023938	5.454155578	52.99640264	90.16156858	16.83558115	1.094661497	160.253967	72.23313123
4.708369943	3.9486779	25.59764614	51.23979307	14.40024467	1.230830925	149.6075741	83.7835165
6.194279108	4.114865297	39.1201409	64.56987643	19.76417305	0.746943279	149.2323837	81.20704533
11.46168787	4.361498608	37.74774796	149.1526365	30.93663518	2.105368516	275.0715929	109.9082986
1.684631817	3.716538015	33.68925272	39.01769575	23.03860608	0.867380795	252.5356866	97.68579267
3.136903767	2.287397774	11.11807533	32.94606485	15.24781041	0.776118505	138.3969898	82.88709684
3.745376499	3.112194477	20.61964515	49.07538736	15.67462679	1.278695945	157.6826275	85.05636983
3.304401126	2.848298132	19.51565466	74.78592557	21.57556689	0.298473153	187.5868678	83.30804298
6.958930774	3.709111336	25.43846965	59.24623464	31.67804973	0.708442591	255.7959994	101.7250915

4.590543049	4.266760913	21.96636874	44.4274973	20.90491893	0.939679628	147.530693	76.82969153
15.19606211	6.229980115	87.1385159	102.7116709	19.35408984	2.116493549	196.4821584	82.87201804
3.378339149	3.134734223	27.3719343	131.3986088	24.20976994	0.860062709	199.4749679	106.4799826
7.163032945	5.843568981	64.25517414	107.46827	17.17407542	1.21787758	220.3121902	93.34560647
3.188343236	4.107335816	34.29024152	64.04807231	20.30097079	1.005759121	140.2753464	81.58206325
5.343462969	4.865793239	54.22371394	82.94729618	20.03645911	1.258035502	220.3030905	93.2900992
9.420120015	3.011810198	38.11322372	81.05048502	21.51370425	0.701745366	154.2356242	81.77493758
15.33292214	4.945365815	51.63351705	92.11119497	20.3031449	0.739799726	155.1151192	78.47522575
11.96865485	9.819089574	37.70902786	150.8896004	30.85604377	0.500929584	270.8659877	89.19999886
16.63967471	11.81200491	106.9717403	262.3958793	32.21254543	6.002266261	307.1655517	107.069334
4.339701426	2.895081178	30.80123381	80.27233211	25.65353651	0.696532927	178.7834942	101.4846073
5.923342926	4.8240351	58.87949105	90.78803656	21.06043703	0.876601339	195.33524	89.30086284
0.917243918	1.722180783	6.869067758	24.87081992	10.41727761	0.588617287	243.9173998	87.35755733
9.624559517	6.571167881	76.73876488	134.1810749	24.39781773	2.464881812	222.7674114	90.36826424
2.324982932	4.499458857	24.34876806	68.32470407	14.4435999	0.642739814	213.1935822	90.8243678
2.210916274	2.437763213	15.61044247	58.6613712	20.00660541	0.285331245	142.1234069	78.86020191
8.20495063	3.634716818	39.28995492	54.39813227	20.4268881	0.844523654	141.3116092	75.87353818
4.641782511	3.701738313	25.96290806	66.83677193	18.65768978	2.440366802	209.2007771	94.21592164
4.762909423	5.040372801	53.81677051	104.6369993	25.64130374	2.120621855	240.271121	100.8422481
11.27388203	3.98193171	40.83714838	76.28839516	36.22211352	1.885720784	178.5401856	78.63747328
4.543503855	4.555111835	35.54516376	74.1076541	22.8341277	0.868369389	207.7561652	79.87070233
6.532987205	4.627061662	26.32625514	70.03182194	14.40992948	1.296738226	171.5542701	78.66917883
23.02751119	5.802150459	19.70050737	55.58574945	27.47858258	0.31707987	227.0136048	84.59584374
1.980544377	4.676386781	23.26439165	91.94377781	17.65894682	1.196366901	151.2039607	85.70667654
3.292043463	2.745122459	36.99634377	48.5196617	24.72293716	0.31948881	188.9095841	85.30230669
16.65174667	7.388433186	59.11122546	101.5492004	24.51835224	1.079183628	173.0125412	76.37339322
6.710897343	3.991536506	41.9905234	124.0514638	25.34079637	1.380080114	222.3276323	104.2437324
17.5572963	3.707414519	85.24665629	110.3347895	22.52548769	1.535855311	289.4606495	93.08106596
4.663556499	3.632216678	46.78069028	61.82603828	24.25179024	0.852897851	208.4394075	91.35646264
3.304692957	2.96747186	27.99843176	44.13079921	14.53451009	0.941695476	150.2108488	82.049416
25.92726169	11.88427344	95.28156685	231.0673289	35.09949543	0.721925093	263.6755868	97.67494372
8.633244879	4.745892731	45.29911606	106.6296988	21.42164946	2.08795567	222.4595979	90.60213126
11.71984559	4.914718639	83.90564497	163.8221474	29.38605186	1.827782703	207.8815561	96.6747206
2.99042965	4.668218182	16.93587579	50.6177798	17.42067511	1.378490453	175.0886701	87.0731004
1.282918743	2.237418633	11.2150015	22.18421937	17.01049994	0.580526712	266.5827481	109.0962265
3.996942426	4.118379787	18.81317146	45.81123932	17.5613803	0.748608151	130.079526	78.07573686

10.71885206	4.370650496	41.40444126	67.3396511	20.11270138	2.040690362	170.8603948	87.87306625
6.409756963	3.999858331	34.00880225	68.59688581	22.14539467	1.01443404	170.7548127	88.88519192
16.60576195	4.320694922	52.80355618	96.27849077	20.28567659	0.940844763	153.9875708	76.07700325
18.92419309	6.115852171	65.09447219	121.4248909	32.55035973	1.71308162	280.2820771	115.2712574
1.292703889	2.416525031	23.29406047	25.63940857	16.78535173	0.918477658	140.064666	77.43471758
11.47652089	2.731752455	25.00926826	59.67271644	17.2759586	1.152298017	191.8700727	106.406507
24.41221085	8.33121927	47.83897845	79.19227311	20.20419137	1.270323805	155.2432633	69.29413447
4.334544889	3.929687986	49.80528009	56.76647523	16.78462833	1.288331196	155.7419191	81.68686299
9.69573198	6.021161947	47.06167544	74.25542036	20.59091462	1.939731671	133.8839367	82.81016358
3.157328211	3.256627982	15.2993433	36.15507391	15.54974077	0.737271543	127.3289585	80.8812046
10.31494976	4.273436799	35.74633423	57.30327229	18.09114042	2.456659685	155.0654532	78.44027886
3.266461074	3.660538459	23.20968493	44.38765066	11.47173568	0.852677091	121.2242359	81.20801063
7.354723867	4.530186081	49.25765888	70.52393537	19.98731013	1.082566355	169.1594952	93.40912602
20.96367121	7.467706854	73.19815067	82.38987173	20.23306633	2.893484637	197.396145	83.48902313
1.571291666	4.698264712	27.09013749	64.34553296	20.9084711	0.617587094	418.2085753	114.3152907
1.034047181	2.904967409	15.62286674	49.41530252	14.82357388	0.590514139	252.0584997	88.64130473
4.589821351	3.290397971	30.94900041	49.93689298	15.96160231	0.569356103	141.2698192	72.81473606
5.1912083	3.991958672	40.6954854	57.04606858	14.26129226	1.76054793	130.5277037	79.75279573
11.78024516	5.417945723	99.12206564	255.8778294	31.61576939	3.045085398	348.2462044	103.2105355
9.807851377	4.274581647	28.57749361	63.62873068	17.14027359	1.304289407	145.3660186	79.04681794
1.301988839	2.477637451	19.33432063	33.22069336	20.57485381	0.539647166	178.5690798	75.43948628
6.495705397	4.418892578	45.09703452	63.46265631	23.54672111	0.970862961	156.1016103	74.36830942
7.764854325	2.920968954	46.23078676	84.14092253	27.16312677	0.736632988	184.8941874	93.88810942
8.845034179	5.110562168	51.88236225	79.29783338	17.1390683	1.60773736	193.2478187	96.8044243
3.055890321	4.038910661	54.65498453	70.97354964	24.5954951	1.260832514	206.7740001	86.19296296
3.623351386	3.059394788	22.23583115	49.51709911	15.7528679	0.935020055	182.3899473	85.53918105
5.770268706	3.538957347	42.98167526	53.36207757	19.9248776	1.157715053	143.0230902	89.14589345
5.189583488	4.30088717	40.62430742	76.44026597	14.64760787	1.124121599	162.732458	90.14849601
0.773397622	4.275990884	20.85990109	37.96524008	16.15889889	0.574181658	189.0581144	88.3401931
3.503065937	3.467463328	21.86423857	42.03201115	13.6018476	1.018543452	141.8957354	81.30101284
29.47243435	6.038233441	51.1543708	81.98552842	28.2369892	1.684602719	167.2389411	86.02154786
26.09782599	6.41767055	92.43167668	140.9456211	19.5799652	1.143202902	215.8446748	86.63027099
9.253854046	3.139154596	30.76731217	44.44684254	22.20042132	1.613693067	267.1139256	108.3198728
8.447589563	3.768568176	51.98865816	130.5252369	24.7606342	0.935082554	180.3619695	85.79256899
3.704905462	3.729712933	31.98418864	54.56473922	18.01677937	0.993529533	132.8916575	81.6445542
15.90233887	3.106484066	27.01518481	103.1727622	24.15642395	0.5658526	169.3131966	78.97106149

1.554618698	2.191847385	21.80179794	67.57114418	17.61603064	0.587027734	182.7736775	91.13592006
8.025893619	4.792386945	33.62268354	112.8070119	16.57331835	2.509842749	208.9739921	99.11601677
3.916225538	4.294081609	26.05095503	63.81665856	21.27781689	1.453302511	168.0522756	98.91418836
4.019825011	4.326991328	23.10627103	53.83158217	15.04281182	0.716908042	138.4231501	78.47864101
4.548204716	3.846304118	20.21797068	69.50935887	28.32868301	0.887499644	210.1560702	94.30222759
20.76389299	5.239628791	19.11345575	79.91834981	25.82574764	0.497957829	130.1814436	69.85262228
6.954225046	4.546199623	65.54853256	116.4331081	20.99101916	1.956287698	251.3329281	98.20299786
7.052921116	5.305502163	90.02975452	167.1958772	41.47687894	1.1694118	269.5390919	102.3629501
27.83188225	6.714131193	51.73613805	86.08181222	23.09314798	2.114320962	157.2968757	84.75075362
4.376042386	2.906656624	27.52165156	44.9400182	20.35305573	1.385168177	236.6235604	98.69807695
6.053178998	7.159334481	66.28798868	79.89938281	18.02699267	2.399304259	278.0616181	96.60902604
0.814602961	3.117462101	22.82029809	42.74266655	20.03735193	0.736754196	269.4959018	97.01116093
11.46507584	3.037824865	64.79575962	115.4384872	30.66172096	1.509336036	237.6946916	97.28546467
5.856360177	5.520582225	46.15994568	122.8307056	21.76881833	1.377013677	190.9401765	92.61785517
2.347982278	2.970003059	27.33964217	42.71388405	17.83203381	1.672133901	197.1749289	80.468966
9.335526422	3.993267772	44.52864807	100.8138505	21.01795874	1.226603788	171.6495024	85.91119509
38.75993073	7.143262524	77.66030743	112.0903549	32.48027356	0.900092872	194.425972	77.50489045
4.803692737	2.445294055	38.42285008	64.21643504	19.15123636	1.100474782	164.5272453	101.6617738
2.77274125	2.887355749	16.79202304	31.42985898	12.94325169	0.432858351	125.59523	74.61055447
3.103122462	1.688700836	6.906781505	16.65993987	13.80820903	0.106734242	124.7140858	68.48211434
10.14315328	4.374478793	30.15438173	95.31468982	26.03517725	1.083576469	281.9045539	90.7573893
10.61931663	6.856199329	45.0365557	160.989759	26.30670895	1.257580418	215.7849276	88.17322317
2.160233706	2.718285667	18.11244759	40.38461577	13.62847515	0.994588329	210.6357132	76.23885089
10.64553885	4.816824768	52.10044583	122.2314625	18.19819212	1.971383128	198.1169333	85.34101145
4.573339563	3.527822699	22.4324875	176.7820749	21.42141832	0.376827521	234.8017772	87.23406483
3.497869851	3.287071895	15.72124991	36.89421423	17.28984173	0.209599069	139.3541202	77.38302081
5.777585744	3.089820661	47.76420752	102.7351091	23.66484531	2.371731121	242.3457749	98.16877034
3.715868596	3.596544543	39.23001199	52.1738687	14.94953974	0.899688882	151.550277	83.62873559
3.652745774	2.745355735	22.49303707	44.57339872	15.39880025	1.583537459	146.4862542	88.67604415
5.202023436	3.509284841	24.96004557	56.11217406	19.44210678	0.87066243	159.8499242	89.20676401
4.814998426	3.032491568	41.91249565	99.19662267	29.61565662	1.488517583	262.1083871	101.2303104
11.02606381	6.102662445	60.66165546	106.1372463	20.49404778	2.344245955	161.4778741	89.9845822
6.104431649	4.303759763	43.44851964	100.649065	26.2148132	0.85526582	219.213603	102.6137114
23.49805863	4.981641154	18.61808311	139.096164	20.97129562	0.575828789	176.8225539	80.38375251
10.5778507	3.613551491	53.40782188	56.32146198	20.65133227	1.288578827	133.6929413	73.52147463
1.384117091	3.701060554	27.26820731	35.50239894	14.44279136	0.476113806	208.810398	86.81596763

6.377228018	6.117608388	64.68911214	72.31930572	27.52958513	1.053210395	178.672153	79.12708771
1.445626062	3.199101457	13.98196547	50.68203467	18.33947469	0.649834683	172.3706498	86.64561122
8.164623769	7.054358684	54.34697161	148.3330182	24.24680114	1.993721513	197.5693932	85.76726637
3.713379921	2.913886586	58.78026763	103.4874302	22.82228019	1.795933567	274.7750938	99.65828668
14.21731607	4.350265699	39.26057524	107.3870425	19.52608246	1.198768996	190.6165171	81.43408151
16.08328303	4.204785721	52.19221018	169.3353736	29.62062244	1.143009546	203.2991544	91.77032865
6.859763664	4.753101835	40.17736954	76.04272876	20.23364484	1.319943825	163.9401004	90.30695494
7.76239574	5.361092679	56.85912767	90.55265697	29.12265749	1.049059042	203.1926216	84.84956402
9.058543176	4.089805188	31.30091843	60.37960099	20.35857894	1.35900973	185.5214218	94.18817685
9.141398463	8.852807385	92.1170739	164.3115896	26.25705459	1.99614829	206.4075303	84.23947776
11.44615812	4.696393488	35.8848354	65.89563831	18.2977769	1.5950907	162.6387572	91.351458
6.822776978	5.617883721	45.06667775	97.85852694	20.98332797	2.217929994	150.641832	83.60764618
8.147329522	4.271531837	50.96700622	86.25229573	25.01121157	0.696277405	213.3667609	81.23065703
25.02861755	5.977942133	75.87143112	137.4093554	29.34436238	1.560348092	191.2903845	83.75927538
6.429179677	6.099786558	57.2187815	76.37058292	16.90307482	1.591040031	175.4706704	75.42804541
10.58500073	4.54816975	33.39514801	94.09199019	28.82208969	0.709909985	174.1777417	93.98431304
10.28051225	5.886451936	53.31694293	77.85460283	20.14789319	1.286252117	193.9087702	93.4824705
3.128648992	3.755410747	24.71351437	48.36988229	16.54195201	0.949742758	141.2747887	78.39611532
9.33403616	5.892569156	37.74835795	115.5076807	18.77508733	1.936354471	184.7885093	79.79664366
3.367205194	3.20195405	34.31741986	68.54487933	17.53458671	1.496445711	204.4391383	85.31384528
3.549170299	3.02538442	20.08227829	37.31562468	15.7902945	0.74242979	136.3173917	78.8820287
5.801569661	3.471602147	36.33007272	58.21618339	12.75817791	1.30142631	116.8544016	75.90755771
11.15978559	5.155101303	42.58786134	73.61238645	18.33497523	1.029265783	169.731845	76.67989436
5.971339605	3.744648701	49.95240403	90.66974603	22.85518617	0.878954434	155.6208214	77.65676685
5.195865055	5.076302693	29.87473592	51.26114086	18.80566341	0.793725834	143.1281382	75.93077319
16.91444299	4.882871244	58.93296961	86.69532651	25.00995951	2.077830065	208.1543035	94.52998502
1.669066012	2.753014221	9.37952587	35.68790551	13.70519622	0.518015727	114.1535842	81.83920634
2.361394673	2.685843298	11.68739879	36.04422383	16.47314718	0.644496765	136.427637	84.5595723
3.793247924	3.120187025	30.03994853	43.00724384	18.47877393	1.071490232	190.5114928	84.15672568
8.244150673	3.794413076	67.08017073	89.68579675	19.20651164	1.633099408	207.6899461	107.3691565
7.061056776	4.219503463	54.19339984	84.10714778	23.93677256	2.039209585	218.9009316	105.7235594
4.63738884	3.491791342	23.53789661	38.24258032	20.20292841	0.554228214	177.1370674	72.43064349
6.542304945	4.187332727	34.36153247	51.00179192	18.52736522	0.7019932	153.9299986	85.78756278
10.60197291	4.09690836	110.9986571	133.7525663	35.08149267	1.607107436	251.4529012	89.09496969
5.883673199	3.319854567	33.73743904	67.16292513	20.90608064	0.501658013	135.270019	78.112468
10.77994545	7.242483707	65.91518499	103.6606451	20.81318182	1.80614026	169.5769121	78.59321956

3.490036924	4.909538078	37.64018604	115.2720359	23.89735209	0.645506928	168.5215819	82.26025207
4.871773865	3.137515465	28.15821148	69.30085282	23.64090983	1.375847735	188.2332103	97.81687162
3.710735027	4.365324581	32.31234639	64.28929606	19.05575561	0.834411472	139.7568614	74.47364873
6.975126157	2.488962261	19.49398508	48.71739157	18.59837794	1.103973932	237.247243	100.7612645
6.218854189	4.846464519	33.39521835	60.82492023	16.2666995	0.604918087	141.1575712	70.43612026
18.29278946	5.30136941	58.72323384	153.4186739	21.3690278	2.67076701	214.9383003	89.6709306
2.094348981	2.483790526	22.57449311	57.61488056	37.14664782	0.756121534	283.294546	97.79400791
2.001070523	2.636827744	27.04424902	40.58162158	17.46669087	2.046273289	241.0953963	99.6939922
3.790736919	3.299700951	26.30344157	113.0587573	12.72130169	1.205206503	272.5446747	89.47164026
6.284821964	3.073966696	14.39376393	41.15852831	19.30001696	0.940327232	165.3406911	88.66190339
17.81831721	5.788321975	58.77142317	65.06731523	21.93481949	2.191718937	255.7591255	104.0775805
6.444376037	4.788090753	55.64790598	101.5560147	37.56635191	1.097302181	260.6575245	89.17729734
4.678293145	3.843228193	61.96521791	82.99928896	34.02693061	1.942747956	371.9874965	112.9335055
2.441658342	4.397287744	23.19655487	82.55498389	20.74361922	0.605932013	227.6029985	95.91422518
30.05252876	2.91947727	19.10838893	51.91698141	29.19985634	0.368272116	161.1973035	80.82344586
3.541704702	5.774391018	18.30382733	145.1018268	24.12843931	1.119342464	208.0210201	93.96760379
6.113213765	3.660941144	31.1817709	36.05697101	23.3282262	1.321387873	146.8013563	77.26913344
7.239845446	2.935977145	46.45323882	54.62075744	23.71325545	1.283804544	211.3236305	91.16993197
4.338563901	3.340831033	34.14289557	70.89059465	23.96587869	0.570490768	225.7049093	88.23446398
21.95390507	6.233573115	43.80004863	143.6278791	38.04994688	1.836559008	257.04661	106.5379251
2.68084971	2.447215335	20.74140529	30.44341382	18.16408197	0.312598257	185.425482	82.62086425
3.783510526	2.450978862	19.30492816	31.25755465	20.07372114	0.409671526	127.6852134	71.14890204
3.292361118	4.704736477	60.10106932	41.6708886	25.60508009	1.091756085	227.10485	85.4871336
5.856834132	3.388594579	18.39910265	37.82438357	18.33178444	1.204628128	142.6956237	85.91293117
4.04507414	4.02011983	36.01038015	69.72749203	24.70531563	1.179926911	291.3804306	96.78770181
16.32788501	6.983713653	39.69603488	68.8639378	24.19742649	0.881898797	208.0319771	75.5521334
13.21150146	3.782681246	58.91651541	130.3113393	28.49617082	2.500851837	317.4625185	104.5878816
6.658112863	4.357345031	35.49489579	68.35322649	24.50965967	0.968380093	205.136814	87.90069058
5.615787649	4.985296301	32.06503931	83.90872955	25.25523716	1.134341239	210.7069304	94.96016262
3.932872335	3.57894127	18.29415771	52.37510684	32.77975776	0.635822483	166.0031725	80.45029167
14.76654973	7.956822111	66.21088835	90.23686685	33.12700541	1.592615503	174.136946	78.56768658
1.295131088	1.930912767	15.52523124	32.39223912	20.10320534	1.032333618	256.0972474	99.68900429
5.036525154	3.995893192	22.69909346	54.8920666	19.46661342	0.805030028	142.9301782	80.37736368
6.100439405	3.870270769	25.55668786	70.57587483	25.7594033	0.479586193	207.2696997	95.48212587
5.599678485	3.175504318	18.91395759	69.23343593	25.55796599	1.118957377	266.2508677	93.13123387
6.900386415	4.326536227	38.41837053	81.1104028	19.27937137	0.849959885	212.8997733	90.79890239

8.402500953	4.06498344	30.16423811	81.18018595	21.75969409	0.818719686	174.376862	81.11211206
5.537617971	2.256048827	16.08383418	26.34922132	17.84961413	0.517026129	129.162802	80.81442773
4.197926106	3.665953732	27.41612723	54.36308659	30.08154455	1.642750238	201.2108527	92.97267282
2.808661558	1.968397991	22.35202064	72.55793381	21.80443196	1.969653894	254.0522495	102.1485553
2.705857875	5.51846229	45.37592592	68.96922003	21.31896531	2.309552471	207.2962053	85.25337028
9.303649163	4.899179971	83.095652	99.85652174	25.14842444	3.283411829	227.0431375	100.942088
6.515821714	3.470464965	42.49189735	50.56473895	28.66067404	0.942684768	194.8204264	83.02201825
14.78281785	5.630552103	60.48205403	72.26907974	23.91174573	1.325935301	229.9476828	93.82778208
4.33790528	2.751801276	36.11615157	46.06736664	21.85012007	0.572715704	166.2535902	83.38719864
7.203960955	4.827333155	28.98069256	108.1345417	23.79793663	1.314349211	233.4743808	98.10833649
3.469963237	5.452766088	50.81570246	116.475583	28.84813141	1.420826797	242.5315267	100.8743613
9.223678628	4.980738932	35.94002128	58.68237272	31.1527478	0.600023861	247.267055	93.94997325
14.75872661	6.532603431	35.55588375	91.8051267	28.64103111	1.222407392	266.4668494	98.67340901
2.591148243	3.562559869	46.15651616	65.37607257	25.47503728	1.897230337	310.384522	106.0672427
19.21448958	5.682166061	44.92382015	182.2864377	32.50897423	1.714610109	372.9486311	108.3666581
2.987391311	3.880035187	30.68175794	66.8114877	21.95958276	0.798522641	208.5941502	87.15625947
14.5071941	4.670859263	40.45033978	60.2462928	24.81132141	1.869410743	171.8355204	88.82368921
15.8296905	5.915406788	61.28337749	83.83917764	38.52635329	1.06682937	251.5108455	93.6422698
5.106915446	3.646269899	34.75868061	47.48950893	24.90392609	1.161537535	223.6753896	93.89963689
0.639441649	2.069210714	10.41034201	21.47993171	12.37528727	0.881801286	180.204795	82.70531489
11.66890729	4.447356909	37.79228468	58.07442143	20.97237404	0.716163145	193.8845384	83.1401722
4.901657689	2.486550387	43.30559982	52.64903512	20.31804114	0.749945878	184.3584331	84.70249698
49.66076699	4.839943356	45.36293795	95.35909954	38.71262791	0.469877514	231.1152293	87.9938819
1.100382834	2.356592731	16.2946818	43.22554273	23.18065608	0.842713631	268.2164642	101.3185536
7.425142745	2.904985093	22.31596551	62.93987406	22.69119236	1.453297914	204.0449895	92.48439455
6.598802972	3.765320421	49.2440969	47.12049984	26.05536181	1.43669647	157.7644038	73.82979945
12.58879802	6.839260971	55.28560636	118.9500736	36.74986682	3.754780002	263.0861369	103.5404373
3.023207413	2.807067503	18.17295284	34.21047742	16.52405208	0.656097995	153.8293423	87.2975407
5.877952023	2.069930445	21.6472914	28.28807856	14.31449529	0.498282812	165.9719523	82.29684512
2.780010024	2.808363117	35.31944293	58.21884294	15.97476234	1.099158283	233.2340066	93.63745115
5.222815393	3.105102261	32.04546048	68.78964525	27.03256912	1.361625889	249.5213776	100.623247
3.727153962	3.988565198	9.720137384	50.30981316	29.18460861	0.551276893	275.4107572	101.0079585
4.429843436	3.309305513	15.62135034	31.10011832	16.77720509	0.530722267	136.480508	73.0230343
12.57660355	5.213784988	43.26248233	167.4055935	42.47548359	2.198039274	332.234931	109.5520787
6.745999954	2.677899816	25.34549688	61.1452388	23.86994477	0.988053998	257.6198603	94.36342871
1.931806289	3.267334873	21.06369609	49.61801501	25.96626605	0.425773219	173.5510687	80.94401429

4.210466581	3.24623677	43.85891295	44.39999247	24.90998839	1.690677668	291.2264154	108.3528968
20.66358996	5.447770805	43.42221646	43.54976057	26.35843167	0.63257088	233.8584034	95.24978878
13.10731856	4.01954732	39.41043131	90.80211592	27.39732265	1.705815123	223.9933676	108.8324251
4.294557029	3.501080674	28.394812	75.41507608	29.18675789	0.99359643	199.3735909	94.54805467
3.772455425	2.799170578	27.83729059	45.40565893	18.04534864	1.082531311	180.9940563	90.13778664
6.884436499	4.576398682	32.88403699	62.74520039	23.95081779	1.647229624	164.3188383	82.67026551
5.637328838	4.335441769	13.04779007	44.08408339	23.37323006	0.810799724	118.3003139	69.95633934
2.344450741	3.055510556	21.87897082	28.22316966	15.36192796	0.651127119	127.2706249	70.33972148
14.6348703	4.071645882	35.78152	101.3311291	24.11419472	1.709683286	191.5512416	85.65652885
3.632535445	5.427045935	48.8464987	73.98894782	34.27803387	2.005276355	302.4967555	99.81080373
3.877641429	3.037055857	35.10640174	45.08254336	18.43539914	1.381375213	186.3678468	83.98153112
12.35058884	7.200750002	53.82196909	63.83519243	25.20990641	0.971544139	211.9475057	82.35744307
5.927853581	3.52771831	25.19239819	49.77374861	14.94115	0.87306656	194.1165116	86.88642725
27.36975295	8.967673404	60.56833244	96.20354329	26.34283414	0.733889312	170.096183	75.65038647
2.085774551	2.179243212	15.00559123	27.95960799	17.24784208	0.697530386	248.6284211	96.1755819
3.820351276	2.913134674	22.46698664	45.5798997	14.77022457	0.688171525	119.545562	74.66289968
2.942389786	2.05453053	11.45090586	33.0780749	31.0910137	0.485598869	256.9717655	100.8117976
14.12581998	7.301040336	40.71712404	93.0032529	32.30347933	0.983892878	171.7523855	80.56020301
5.859928318	4.322950738	40.13040682	83.88485857	22.1859742	1.367555332	190.8888747	87.18736439
6.187606211	2.495091693	14.53182258	51.9640937	22.1234918	1.295721939	165.0482292	91.55102147
8.704627262	4.6666187	70.81868924	102.9146717	34.3569768	0.889968776	283.0430527	105.8148427
2.79861506	3.650429481	28.7960783	35.91887143	21.35406292	1.192706942	199.3857851	91.34794135
7.237027548	6.901089683	46.17023901	102.2190629	18.72620079	1.520638174	219.0670127	80.55895094
53.12489756	4.663098462	29.0575918	92.55759153	33.86866395	0.745214461	250.045975	83.36781331
19.98077826	7.343328619	56.8110414	80.40610903	32.5215652	0.745381644	205.1059575	81.40068849
7.550504567	3.431596342	28.53661943	61.17861848	18.99153559	0.974791871	205.7286074	82.90366188
6.926149546	3.857200438	51.66816689	80.52103286	25.37341768	1.459037558	223.03858	99.1040144
31.49114867	6.724817023	52.84500707	117.6950946	26.58734378	2.181617952	234.314461	99.62686579
10.18329634	3.76447851	34.215225	76.73519909	28.43422211	1.591893053	199.6634315	95.8488404
8.192041159	6.890125375	69.58150966	107.9680857	29.18351984	2.089541489	213.5388566	88.09869405
12.4904903	4.598062102	49.19391538	75.96068582	32.35511004	0.457928569	225.4082303	90.28491681
6.635516589	5.598506555	47.17403042	82.03788784	26.56362322	1.327591628	178.1697943	90.3672344
1.092780883	2.261609318	21.62710224	27.8935066	17.29313019	0.787101473	221.1622665	85.0860657
9.370084895	4.96061522	54.22156893	68.12715473	27.05219023	1.133492235	184.7687835	89.52495015

Afatinib_1032	Staurosporine_1034	PLX-4720_1036	NU7441_1038	Doramapimod_1042	Wee1 Inhibitor_1046	Nutlin-3a (-)_1047
4.31792049	0.132812168	113.7460071	16.01855748	81.77785853	8.097098574	93.73426061
14.34112113	0.01625245	53.73518345	7.033203625	76.36919575	10.6238131	45.92985717
9.121543577	0.195393148	162.8482232	18.23050423	89.30840084	7.797094332	270.3078579
8.845592173	0.026544853	65.39316726	7.077125617	69.6650815	10.10841133	57.05165139
13.22470023	0.090256383	171.6512529	15.80418227	95.19820363	12.98163977	181.7461595
13.93961013	0.027002151	41.85180414	7.885207426	77.81883313	4.796021612	17.25031181
8.242515717	0.044988511	55.40677015	11.71197565	85.12907888	5.411897855	53.58934723
10.79908207	0.07284704	143.458629	10.68142878	90.30660944	24.46347275	166.5575228
16.65346633	0.098789056	109.7938435	14.58832651	86.99993341	31.05179601	161.9293838
5.120267056	0.030531457	37.89404197	8.730018608	68.9509806	4.466844721	86.6336611
5.993774588	0.056394587	89.11974159	10.36003333	56.58562792	3.539857612	50.09335311
6.91422335	0.040221107	118.4935852	10.01262987	78.15750208	7.838781474	150.8808799
1.818381539	0.053891504	83.46748744	17.70559081	102.5471963	3.613411779	197.1249519
9.630718483	0.124767549	160.0301632	7.60436948	53.05258201	11.11427327	124.9556853
8.697338623	0.034224958	77.75038041	13.4563739	92.94656552	6.202787931	190.843318
5.295740172	0.04697758	97.71489474	11.46059465	72.52899592	12.44268808	77.64583368
5.933826918	0.029253368	97.51026502	21.28993578	128.9337256	1.40981767	181.0366077
13.05406665	0.059376012	151.4252693	13.17972899	127.8196458	13.40807407	118.1395616
10.53080192	0.062851622	104.219367	15.16903916	112.7522727	4.887114666	285.2630118
8.104425767	0.015880958	48.97401355	9.732955315	101.7178583	2.325389854	53.44441666
7.581937258	0.05047024	102.8296823	11.33893741	69.4323139	7.818980868	109.3091887
10.11706953	0.221591427	182.3089524	21.93153867	110.6107067	12.93353917	94.42504178
3.740976642	0.039320858	70.70411216	15.07241388	102.1451321	6.948230721	70.33075731
4.330287451	0.046415847	99.00794421	16.1090536	103.4693036	23.4197053	377.0893934
4.887174664	0.03566338	55.70191642	12.29898588	79.18629898	8.03291345	106.6976876
8.210932124	0.04652703	81.01055056	11.22105545	83.64696515	9.235764762	102.223727
6.875248503	0.142077599	198.8104435	15.52642364	91.34455003	21.36345446	368.7344912
5.774432038	0.040709086	97.53940653	14.05853849	96.40231202	13.10171973	274.0015501
5.638412926	0.025359112	73.38753648	10.45899192	87.47996413	5.96346293	96.9297609
3.877972441	0.06193711	92.81646683	20.55350813	119.0668751	4.15498641	192.5343814
4.944606286	0.056856018	76.67326366	11.51677839	68.6100232	4.400254513	83.47591323
4.170740523	0.054108292	112.3073127	12.89053309	133.5830406	6.376312365	334.0618012
8.477504091	0.053894011	238.176939	15.59898825	124.0510541	15.98856388	600.9195028
8.161940309	0.054947074	74.92547932	11.53244832	91.84384473	6.888377707	156.8754688

3.804927102	0.079233862	91.60525576	15.08347376	85.10349438	30.40363022	295.3982913
7.68489667	0.107797333	152.2753352	18.05121963	118.164084	16.16057098	241.0628428
5.247546192	0.009767177	45.90621586	12.49274417	129.2283116	2.583074839	66.27437918
5.827733637	0.117789858	197.0620394	14.6317212	127.2400042	18.90076483	453.8525495
5.900956856	0.069772669	99.65473166	13.34729981	83.38667336	11.2056223	95.01063576
2.760403567	0.045951985	108.9911012	20.90822516	107.3955866	2.884571188	213.2316544
9.739814486	0.038516218	123.3259578	11.31384552	107.1655848	5.901570573	287.2406453
12.18073309	0.029593819	65.87538776	6.599763348	70.25106324	10.50097915	83.70102583
4.834444195	0.050326972	80.66030427	13.56875667	89.33090532	11.60708306	34.09438067
2.427135045	0.044570124	121.3373552	12.15602728	67.02996516	16.71452112	213.6288491
6.172397384	0.051630128	103.2795575	16.5034626	89.97307134	8.19269042	63.72508632
5.450141967	0.021108873	51.73218472	14.12635939	97.09321938	1.017712828	199.4726243
5.59152278	0.035169639	60.02259699	10.92568712	76.35183625	5.454428041	61.80567127
10.09392698	0.060768139	161.9337937	12.24113785	89.69522181	7.376977357	52.6596886
7.16413481	0.041937922	65.98626656	12.03699509	71.23504063	6.237054892	33.09426534
2.848447793	0.029200262	66.06005317	13.59248599	96.91378933	5.502139345	81.01069174
5.114319655	0.01915604	32.06506832	9.267919082	63.06543553	3.046838046	14.97558276
7.456510848	0.040418359	81.21147254	9.674970479	82.65064967	6.015637681	168.838832
3.739284407	0.083402463	129.1400174	10.82277889	92.47170246	7.606000115	507.7219352
5.904760016	0.026484736	53.21283126	13.98772521	131.8222599	3.925502645	254.3967759
12.06249985	0.032072175	123.1525654	9.61187357	89.60710652	9.238060824	192.3591104
12.16509465	0.049181283	158.033871	12.4722336	105.6128728	17.42487564	384.0217057
3.156795371	0.041149989	56.78735567	14.37948788	101.2834889	2.767572728	81.41567319
3.739979356	0.019384956	50.6539764	9.425373872	75.18283093	4.850173916	39.91461903
5.230948676	0.171794885	183.8530076	20.61612275	102.6404727	20.68687222	147.7589075
2.196515703	0.03346308	62.98121712	18.88431808	119.2680757	5.689993514	138.460808
9.806059914	0.079847638	148.3669121	11.28768491	79.22616483	37.10764532	280.131444
4.257178452	0.061280147	86.689015	12.11541784	73.81605389	9.494596816	81.3793506
5.450879833	0.079290238	247.7476224	12.933632	94.58896405	21.56961927	772.2899936
7.436093338	0.08811159	136.9829571	11.21592556	89.44004472	5.219836524	186.9193777
9.192132274	0.02501983	48.75406882	8.776630916	70.27318996	6.406443561	36.70648539
5.450798376	0.021543793	33.94854782	8.814123343	76.59660023	4.65889199	43.44667653
2.061902107	0.031943538	142.1325342	16.47163538	168.7201144	5.674448189	375.3176635
14.70435148	0.309838507	219.3702002	21.42102409	112.2303787	96.81483311	363.6569396
7.13298665	0.05478816	100.1893295	10.69953188	100.8536076	8.202627891	250.7206899
1.854912498	0.054052384	43.60544475	15.50179998	85.8327828	3.244266687	69.57039994

6.428669368	0.070876912	122.5693034	15.68294509	126.9687913	5.877264173	403.9080583
1.435266534	0.04085439	57.26533135	11.94479542	81.93308629	4.198229582	133.9417814
2.821956976	0.061784195	112.5934571	14.33353138	98.78097944	10.30858839	279.5728529
14.86950207	0.274795401	298.3145297	23.69075982	119.0991216	24.65562412	443.1263571
5.489966379	0.035608167	67.29548044	10.75814636	82.46528145	6.524444347	175.3030719
6.017599903	0.024141047	44.01835491	8.369362742	65.14862247	5.190556787	32.71653045
5.342312616	0.044545473	69.16394232	15.89607202	91.89476445	3.690024171	52.95828541
4.978615629	0.034216572	103.6957209	12.29913977	77.92017084	12.80326521	35.77003999
4.948169453	0.07144134	247.6353645	20.74969808	106.6495075	8.438894149	253.2964939
4.107448354	0.073270381	71.1387624	10.68953878	71.09316134	8.255914581	155.9421095
21.37494706	0.079454264	175.7295038	11.83703756	118.6730174	10.2948196	242.2642914
3.576968983	0.071850801	98.49777699	12.83967592	99.2497222	24.28924065	422.8817344
3.590561441	0.028423158	46.00907838	11.18420822	70.08816082	4.809601456	81.06379169
6.479939368	0.017517408	50.49428476	7.786533604	75.54684812	5.40376769	30.08052046
5.976483778	0.07557113	186.6410474	17.22059616	95.8568884	19.29307555	101.3799242
3.860852926	0.041661126	106.2489871	14.02936157	88.49391498	13.50641562	150.5044157
11.97967483	0.031165015	106.722119	9.438053848	71.46852337	2.192406524	146.2096795
5.6712395	0.044974364	54.91092022	13.00640216	86.06372375	11.62744209	79.13428158
10.73481925	0.086808467	236.4722671	14.69883034	103.7402207	7.272834237	389.5849151
6.919981185	0.015224986	59.91357895	11.96234669	113.011505	3.505777191	82.04226094
9.963401196	0.135459167	141.7572705	12.76668871	64.3578344	5.633097185	60.4432553
5.614728829	0.049662842	80.44193582	13.99524763	89.3721926	5.861611675	46.20441257
7.479956276	0.077273701	98.25611951	15.26309121	90.87943787	11.49653624	109.6965642
2.649829258	0.033618569	48.61033604	11.0809493	80.06907116	6.105484557	78.31320418
3.346576539	0.052979071	88.73151396	12.570725	66.73569136	4.798737408	62.05055346
9.292704442	0.031443701	76.69039943	5.543021656	76.46782115	12.13750718	67.76068109
6.152258709	0.036185143	91.70091648	13.78086225	76.31452301	5.313727274	39.13630563
15.9923987	0.014473952	47.26906338	11.6773034	138.2426147	5.189533717	37.33779326
6.833998781	0.036302239	76.24562549	9.80693456	78.91768457	12.29909978	99.44998293
4.285529428	0.072951532	113.2238953	7.321583562	59.9221063	9.507232302	175.4829011
10.08982353	0.056598156	135.0291546	14.51694783	89.95509923	16.13683273	237.7984937
5.426046469	0.040404833	87.29227693	13.39306198	101.1837887	12.73951457	160.0177619
5.254850719	0.021792262	48.76366308	21.47925693	112.2608377	1.621125184	136.6804645
5.206183377	0.046458277	70.99515222	13.05443159	81.8661489	11.60269001	99.36506077
9.623561822	0.05160917	150.6650922	21.63683818	189.3232031	3.005366091	241.3364799
5.677752185	0.081375438	128.9916219	15.08523249	107.7868729	8.07103436	142.2438569

5.064839304	0.043029468	59.66806357	11.1684545	69.0243847	18.25091717	38.53498939
4.732940974	0.033196438	69.59867336	10.30155002	77.50235093	15.32565601	113.9859772
6.218152718	0.084900817	74.71482517	12.33832622	88.56330132	4.029128692	133.0653362
5.621126185	0.140943233	314.3230877	19.59430238	111.2576471	29.8990801	161.4850298
3.545384514	0.046378626	78.18570567	10.16753776	84.27027362	8.981516019	95.79349752
6.27257451	0.025977735	69.01795414	11.98899626	111.4527312	4.490162173	110.6280146
6.309617487	0.066999332	153.6586703	11.20981775	93.91233601	20.40402029	741.2024964
6.456281232	0.04291409	78.03805766	13.26520757	73.67078164	6.400077641	53.52558707
9.830417114	0.059225219	120.1613208	8.315289658	74.91702213	28.01288106	176.2134753
6.370708578	0.031275398	72.5436589	10.72347916	68.19710954	5.457869843	131.8709628
5.196977986	0.072176713	85.1676847	12.45430787	70.72008939	6.088190286	70.76152396
15.01221349	0.2076199	177.7587572	11.62210845	77.47579351	22.34025244	376.3801977
12.12727625	0.046377784	127.6345219	9.79173511	95.04980143	13.51833903	105.4973185
8.511332732	0.03969633	59.96137127	10.35313491	67.64396862	5.53718311	72.84669366
6.246671793	0.026541262	78.68628504	13.49887175	144.2292463	3.206081536	131.8785218
8.082566878	0.076523381	100.9257279	14.00107752	90.7038037	5.177253043	123.5399244
2.722904756	0.014574245	59.65021244	11.96234125	88.96165201	6.652025359	61.81795846
6.853966047	0.061789214	77.32178953	13.05766752	104.4483422	4.11147928	168.2620729
5.880766561	0.059541472	122.5726116	14.98887034	86.88397546	2.017186734	180.9335063
3.539764898	0.052928505	78.78354708	11.99030833	76.19384119	8.22634706	65.23731733
7.652342937	0.21585936	149.3847949	16.17353887	96.56291915	12.04449685	212.5885889
3.536005762	0.054746094	80.73245772	13.16573557	90.69714013	7.917055492	130.6774717
3.204409029	0.046424762	50.81008583	12.50584525	90.9237521	5.119466895	30.68204541
5.245531644	0.035256172	89.89843121	17.23022628	186.2740936	5.022363057	396.4643511
3.774909312	0.045069689	57.8091942	9.410155724	54.51915793	7.829224196	68.04767318
5.72145606	0.01360634	58.56298339	10.60621701	158.8832794	6.100496446	172.1877219
12.45352408	0.135501262	194.4385459	14.57785366	90.03474827	37.13518947	236.9579273
9.741594419	0.017506461	61.10238007	8.67198285	84.83570845	7.797571926	82.54193696
6.44523	0.05423609	61.34313254	9.060814284	79.27480193	7.636415639	70.98352066
4.60271028	0.098300255	88.75788092	8.621458092	72.46126039	11.2346246	271.748183
8.418053507	0.12464747	194.5061413	14.58443915	129.2505385	7.024866341	386.1988895
6.577038226	0.054776788	88.27966673	12.67374531	101.4626975	10.1856472	223.2908543
12.38028073	0.030817045	79.06079352	9.105265679	64.6410639	4.909262011	56.14267478
5.71754819	0.040922285	81.47717833	10.61496987	76.11356155	6.927780385	80.37552412
4.565828253	0.042406295	81.20430661	9.901928911	92.20651608	11.59186767	147.5140898
5.042816012	0.053881575	153.8177406	13.72861749	94.13950068	14.81525788	273.0700448

6.940403947	0.037261676	102.4958531	12.84088083	101.1811938	11.55091916	109.2300102
5.506499222	0.035776641	111.6350852	10.02464911	82.10904991	14.96913361	136.0811734
2.070735043	0.035988488	125.3748486	16.46293893	104.807023	9.655092106	367.5748754
8.095960555	0.058545503	94.52969884	12.81076103	85.38270991	13.59734803	55.02903412
8.005523151	0.09199679	233.3739452	32.74898801	207.1619447	10.95628479	582.0317257
4.311462087	0.050080191	92.72627892	15.07986652	91.78004641	6.840280674	188.7880392
11.46662051	0.088317213	165.8404028	12.24949212	80.5634615	13.80566109	194.6138182
11.34676031	0.020332802	55.0152541	10.101453	80.32192453	6.431271811	51.93649989
8.074840398	0.035744314	53.42370561	15.1185025	91.84297501	6.706266496	46.40923339
7.532255959	0.085654575	67.00292721	13.73259626	80.34784291	5.245281761	202.9015441
11.93690817	0.280331358	184.2431047	16.80767871	96.49899382	26.95160346	282.317487
4.171907899	0.040239791	119.5283536	16.74042457	105.7507513	9.933687127	66.28578607
6.32325494	0.038311099	70.16951799	18.62964371	102.2032781	4.892903483	71.62799436
12.89774743	0.096419333	179.5116781	18.91317387	82.19616764	15.11830595	142.4375326
4.860174134	0.032196428	48.00023576	8.12178231	58.62889157	4.057890408	30.49276676
10.01795951	0.147243893	129.5952629	18.3460664	101.5535388	9.877614307	224.7245221
1.153480694	0.045000393	27.66321075	19.17951705	113.939562	0.643941125	64.82059748
6.245825863	0.09654158	116.1852024	19.1761354	113.8195552	19.06466427	618.966549
4.566165052	0.055565859	106.3524987	11.77575484	78.04095232	9.38073249	192.1522294
4.056729481	0.027162868	52.67078651	9.718143696	63.35038229	4.821488579	23.79335485
4.401012353	0.052082581	109.9479065	17.4760281	129.2241171	6.358893944	183.6441104
3.309709884	0.039379492	59.21737171	12.93230006	88.35281331	11.72051302	82.43947552
3.803113756	0.050283793	127.0438207	15.49646666	75.98449582	10.09529678	74.64301008
10.52729697	0.070108664	85.33579461	10.94748244	95.90592863	12.22723842	70.90878803
4.270172877	0.061732004	109.6674704	13.86237814	74.92352812	6.320098363	128.2597623
8.035969189	0.107011135	180.7942998	17.5430463	101.0504733	13.13402417	234.9527013
3.970620853	0.024384403	73.28508635	11.61927188	95.94826501	8.650027277	166.5675153
3.013228245	0.024908032	66.00421303	11.78076053	117.1269553	8.633503616	310.2337133
4.553686854	0.033327904	49.72579623	12.93516636	97.76753451	4.710159666	55.90998772
6.075842381	0.011585119	46.68358932	10.51182859	105.9669549	5.146814953	78.46707014
6.644827439	0.030930296	82.34245406	11.1441787	66.5963083	6.408748608	39.37918623
3.874113996	0.039682962	91.59974745	18.67636616	103.4345302	7.240896918	45.97395403
7.963813908	0.018979481	38.49618864	8.557305737	62.44526608	7.866997691	10.94974016
3.941102095	0.085005089	185.8113128	17.40523378	111.7698331	28.16279873	241.6422595
7.589325193	0.016934879	50.69951129	12.31027867	122.4513446	8.228559033	115.6945646
4.027649133	0.035417542	47.47009199	11.5816735	94.26720083	8.779144336	148.8020826

5.232241745	0.035186969	65.65538628	9.579662246	56.23283593	4.686840881	29.87816652
13.48585053	0.1423822	250.5929277	26.45299565	115.30021	3.421105833	506.8434894
9.484040674	0.042453994	76.7056631	8.462943716	70.68603541	10.66492752	62.33058766
17.86745986	0.101359167	133.7276106	11.21723855	108.4840114	8.862207873	441.5973709
6.110975818	0.062083759	51.5336636	13.24581928	69.48534427	8.256866216	65.19563136
14.41626299	0.169269208	202.5834814	12.63065325	93.69346658	39.07865965	434.1089104
6.694366088	0.154894477	159.5013987	15.83425808	80.38753762	20.89367929	79.61080206
10.20169237	0.077246819	74.04786031	15.07891536	115.8309921	3.639241181	213.9428337
4.933916732	0.048283202	68.34213037	8.110331087	54.47613123	6.353348393	61.68680676
5.792429522	0.055640428	78.1959566	11.26369835	75.73160742	14.8304129	52.85767969
2.732322451	0.033620048	73.60668046	13.71856401	86.94065798	4.928676703	31.98685905
3.733103258	0.025867797	57.44510981	9.161089335	78.31639032	9.038165106	147.8487438
11.00135291	0.082758567	104.0707269	16.19242419	99.47388659	7.291852985	164.7642664
4.027801583	0.031970717	74.2186849	14.87081687	94.14626799	10.55541952	127.4187322
10.96065686	0.05286329	65.22728486	11.34350482	70.25072026	1.785564923	127.4878451
6.978857648	0.016175626	62.88110525	7.47961278	70.28528147	6.907371808	74.41235943
4.293092223	0.027153898	78.76895815	10.16177185	73.79096216	7.888029062	105.6502834
5.704906698	0.209699882	82.25849986	23.98055745	84.32029585	14.19374919	168.5017864
3.939702999	0.029719853	47.48401333	10.22664389	67.64925584	4.303355846	62.18750681
3.258801151	0.019996738	33.04333725	12.88994464	99.95525451	5.363801958	161.4802964
3.414093681	0.063805735	85.2879249	14.23182895	86.91994786	7.454985622	124.013287
4.906657127	0.097072194	164.0769946	15.03564756	87.68145034	32.25621778	692.7616006
4.139098232	0.062062625	51.46625483	18.67145645	105.2368446	5.541463844	271.6495937
8.380918967	0.054908687	109.2776146	10.62703036	75.44829318	7.590990722	76.92651986
12.30575642	0.160346838	258.4494201	14.74448218	111.1193137	42.12666872	177.8681712
6.877369621	0.089002276	116.7251343	18.22490175	98.92861374	8.825925118	173.2578628
6.313973896	0.144013474	223.7009538	27.84009895	106.8920771	6.680609663	262.5226442
4.203698259	0.020507618	52.70760559	12.24906295	81.90981265	1.612865622	151.6001439
3.000300249	0.03035173	30.98579221	9.966022963	60.26128935	3.635815228	27.83592672
4.393298518	0.07373794	84.31875962	15.81582085	94.50603653	5.000462109	50.8684642
6.43843923	0.022650213	53.96837855	9.115222674	110.1548783	3.545011213	93.28141196
4.452904776	0.043314159	51.01475999	10.93536507	70.84572897	8.817090666	36.82208952
3.31676778	0.049669732	88.11006595	12.96730532	90.80394293	13.11883344	296.3545746
4.284504983	0.043886204	42.51530619	10.68316423	65.28803023	5.189471594	39.24315843
3.270255626	0.07586681	108.1874579	14.19496883	92.23829066	20.71467323	83.48392157
10.62573882	0.136170668	94.72903833	13.37402481	123.2244204	6.366279631	583.5643405

8.461914953	0.025858889	90.32012876	9.334962124	90.10307508	10.53309464	155.5045559
5.105449641	0.026162601	65.1051407	9.777001941	91.08944864	9.82546561	246.0940111
7.639810854	0.032505775	94.77307036	16.76786279	89.88941069	5.10204247	140.6921901
7.217713241	0.144411121	207.2653916	15.4139547	102.190797	17.34073397	173.041242
3.400282726	0.035113665	60.37893667	15.61649025	90.26532702	3.124885344	39.86436503
11.20323078	0.011549564	27.52937713	7.196489575	73.84246534	5.241260339	12.70637289
7.790721746	0.028227927	137.2618725	9.008090706	97.66923385	6.100476733	211.433199
3.956163872	0.03439008	97.92148505	11.81378517	98.73668978	7.565065385	143.0788632
4.120365819	0.047835274	94.48105159	11.75007743	93.53042604	9.381501644	39.52301713
10.09276778	0.081526565	109.5915772	10.55589226	77.99479993	16.28758995	149.7688261
7.219026985	0.015145657	71.21225673	6.487323322	101.3373556	8.098422544	344.6010984
4.016656414	0.059519692	119.128877	12.71507423	115.9230097	13.78175214	243.7504144
4.623003495	0.039319061	123.8243284	15.71800169	102.913198	13.72850512	189.737774
3.821130675	0.103326092	124.4860621	18.01841512	94.15697072	6.100389346	219.1633866
8.027917388	0.032543004	59.27629394	6.747566169	57.15374366	2.497147029	49.18367556
6.639201989	0.037012763	61.05818537	10.6138927	78.51420426	9.46798692	102.2803509
4.931750963	0.041800182	70.79510794	15.15940004	96.91060992	2.706445209	54.27993624
3.339868905	0.031768351	69.90405538	14.16790455	92.4823974	5.518928476	196.1514631
3.714107332	0.025488631	55.90735628	8.0072127	72.20049774	10.76772256	79.18468786
3.662612475	0.098206731	88.62860636	16.66770786	97.59707487	9.693159582	134.3742369
3.685618045	0.050902319	42.6413998	15.63206298	74.09831709	5.377880602	42.69216652
7.728727482	0.048208795	88.91284576	14.54304119	78.22321751	11.86178451	58.08472101
4.417142417	0.052628899	135.1221047	20.86345919	146.0200271	2.913583859	484.8798771
7.679733726	0.151411012	209.7861721	13.0135252	70.78543345	13.28752748	139.7307333
8.987874016	0.030952384	67.69529433	12.20609346	79.07554254	4.705136737	84.62735638
10.52389968	0.08184295	143.3659845	12.90390466	106.6771908	36.89622175	340.4274455
4.471497745	0.062450128	82.20713096	12.42218663	73.26406237	5.576548122	79.29243586
4.704282794	0.020546385	85.26279034	7.446184237	81.2239117	12.74476483	120.1008748
4.729874856	0.03992702	65.19044082	10.59242657	66.58712148	5.382100443	97.21002523
5.423043859	0.02513623	77.81005675	10.47401696	84.25743978	7.366316042	65.41708374
7.094207718	0.108247198	104.0823908	20.63173976	106.0891303	13.63599552	112.7827861
5.089707816	0.034277801	41.73011773	17.89761677	108.1593569	3.666471423	84.05962169
3.736686258	0.021919507	24.84461521	9.871257318	61.2375682	3.938543929	13.34440012
6.167293942	0.045105432	56.12039398	11.20339675	73.06423853	9.390318993	41.20209128
11.31933544	0.065952303	57.39538557	12.59763754	128.1165987	2.150199086	273.857894
3.54407543	0.054849627	82.59898186	21.05290234	104.0158149	7.706009483	97.38715715

7.939859006	0.035534128	43.95528719	9.434779852	81.62396516	6.454761645	30.49790343
2.437326132	0.065138449	85.72180472	10.91695094	77.89999181	15.1410598	146.9746403
6.025892219	0.103973946	175.8342909	20.61072029	100.5358698	6.453107381	234.9263929
8.066806008	0.070053789	110.2868773	15.81362224	97.98386208	12.60936534	170.8314901
6.464267539	0.049862032	67.32698339	11.9749413	82.27997674	8.000267612	67.69131892
3.405386508	0.043956615	76.14167015	14.36587152	76.61683733	15.53490531	104.813446
5.168293169	0.055705984	75.60984429	12.29185241	90.27017078	4.233314122	142.8365852
5.190994931	0.023971231	89.86453606	8.923445207	79.82095708	7.717381678	68.36646297
18.93634417	0.058755515	130.5852911	13.40786476	134.3289054	6.535933722	318.5166963
5.383080911	0.216137744	162.3652386	20.3735796	107.9815603	47.4542503	281.5538723
5.401043038	0.073158906	115.9260407	19.70807664	110.4300499	4.146189024	99.43122106
6.055131447	0.033497983	56.04791556	14.77454661	100.5715926	9.661475548	113.9966014
12.48099869	0.044816863	128.8166917	8.964564396	80.37901264	1.932639552	176.130686
12.32893071	0.127841718	95.84492341	15.00275745	86.91703646	18.75708855	262.771002
9.244742301	0.056030707	77.12421296	12.71302383	87.57802663	5.923676846	131.9474411
4.74443395	0.017009074	55.99158532	9.79214536	124.1399057	2.162216372	90.83725981
8.672318065	0.033870012	57.17762315	10.07242708	79.22022951	6.54018777	79.57842687
4.194952999	0.098526577	65.52870097	13.7025708	74.209944	6.989675017	48.64600158
9.55964144	0.11331019	107.6290527	14.03105096	86.01283301	7.142170525	296.2415832
5.36134546	0.040216681	89.4272164	9.231654151	71.56286951	8.767978487	58.48602148
5.00099171	0.017364677	75.5407575	11.85374652	116.9354925	11.1545466	145.6148714
7.889226964	0.056147514	56.01116491	9.476005397	69.2594013	9.236225296	110.1112467
26.14301673	0.008964389	45.3225828	8.148660122	181.9768552	2.167481658	168.1217199
2.721611453	0.040715328	95.72150255	10.15960693	72.89232346	7.041234168	263.859241
4.826136442	0.029824323	36.08423932	13.55143094	117.4225897	3.037019218	51.36241552
9.377161522	0.05445414	96.47711505	10.91843727	98.62461974	11.82568663	113.7005162
6.346763528	0.11452146	165.7856725	19.31190383	103.4477747	6.814900293	172.8949763
5.076546863	0.06914545	118.1789534	14.95629157	91.61993513	14.14346761	296.9932204
4.497279984	0.064925128	88.95854155	15.0608516	94.95353256	6.104957822	118.6747194
5.14757442	0.031520071	45.23371226	9.954609642	70.62348227	7.134551906	22.5453163
16.13851702	0.089808276	151.8546492	17.1635219	141.6324416	6.348375878	1041.125881
6.25485504	0.037186639	86.05383372	11.62026946	80.91755456	12.86043543	149.6317022
3.633259163	0.064997215	232.2041415	16.54717127	101.4753371	12.45065658	412.006837
3.507435835	0.056344508	57.18706229	11.03475714	71.75549818	8.604398683	65.18501418
6.164560246	0.02692462	46.26563106	15.76909687	114.2664672	1.306637342	90.7787198
8.818650928	0.037151747	52.35298259	8.780523959	70.95219383	7.825967901	25.25351253

7.858245344	0.083450266	98.46942894	12.89588786	75.97902905	12.05763592	151.2072318
2.689935513	0.040972331	37.03197961	12.58652692	75.73610764	5.212484528	56.00567536
8.880650982	0.037253413	93.00554548	10.14264121	87.29556246	11.27849389	57.13899934
4.379755783	0.086547838	169.1258492	20.96586726	115.1275092	13.0439722	100.6634655
6.906982246	0.032019529	50.23252841	9.003683426	63.77417481	5.342154745	41.86601334
3.038008032	0.044017363	78.8957418	13.83490045	68.26168122	4.639846065	57.67785858
11.52681869	0.017509038	59.47038645	6.749163568	87.52169776	18.9366041	51.49410873
3.881715777	0.062685874	51.17409587	11.98754436	72.29357234	7.670223579	30.70482623
5.541074314	0.185593168	173.1182673	11.32340312	63.99605039	8.203711252	263.8720319
4.592028965	0.032509743	32.49084473	10.8416788	67.8181011	4.550527198	19.16398088
9.598611971	0.057458728	128.2552982	8.469099772	66.99315277	11.06771744	166.1519134
3.147111645	0.04950807	59.85779823	9.414331805	63.0927216	3.846682621	44.4346619
6.411046521	0.050767621	106.6246304	15.29084867	90.95796978	9.037005528	43.78847277
3.484885287	0.082014771	95.30607957	13.13222286	85.80120326	27.74203891	130.7188562
1.952569348	0.017742717	69.13612757	21.58153682	144.8668038	2.863323457	376.0549627
6.352938072	0.035295277	43.19194839	10.14524692	96.8626061	2.702750723	225.9019031
2.704859916	0.022432515	48.30716297	9.470360613	84.38743486	6.82922053	96.04439555
6.819068686	0.063752328	74.09448709	8.594755751	61.55542192	7.068688933	104.3009264
3.304494648	0.115250157	186.3000176	19.37281462	97.73860599	31.16724246	664.6902957
9.32162158	0.04132068	80.90393262	9.821701604	80.63168596	13.86395149	87.96488279
17.20429652	0.019179078	64.36923473	8.149193493	64.91222378	3.447978032	74.79001549
5.965183143	0.027495123	62.50001387	9.424916474	78.41412973	6.454548295	131.3667382
6.160507056	0.064217799	192.6057627	15.47242738	104.219645	6.512557523	106.1495771
3.688227181	0.057813993	99.78094001	15.72685629	92.51529133	10.27860838	103.5769648
4.906231782	0.038919707	91.55433426	13.69025053	96.37230483	13.67136429	127.9961518
0.545879361	0.054003511	63.38786856	12.72758348	72.92873444	4.65510085	187.9512774
8.3597509	0.0503271	72.67316282	11.78785591	67.92455651	8.38482804	43.39725691
6.084910747	0.060967577	45.64470458	13.79379838	88.74290748	7.79148059	34.0235764
5.459954045	0.059092534	97.33587298	11.81306474	84.20446806	3.056412366	193.6193763
9.983852172	0.049198921	55.28648116	9.879388673	60.48182064	6.009214515	38.12155505
3.385559158	0.062356459	119.503122	11.46033674	75.58327022	10.85274503	67.19363312
3.559982953	0.029221803	103.5653589	12.4344782	111.3689033	18.37472867	279.8196746
5.818730991	0.075592235	79.40585765	18.07906293	82.10184528	4.644595799	59.79897399
8.768027857	0.103413068	162.9865486	12.33013224	90.6558166	5.698082236	426.054378
7.198931817	0.05355919	77.47662158	9.969666148	62.51845049	4.462381882	37.11194592
17.079134	0.026790994	185.9050786	10.8449475	122.7718451	8.094030018	365.5263107

4.460395254	0.039559843	70.3061531	12.32318228	79.97038563	3.164786385	111.6931162
10.25604468	0.077541409	136.2559888	14.35766151	90.57227102	12.08886266	71.05346217
5.050038024	0.082706896	84.88513682	16.21917777	81.09269467	6.971957016	57.82050127
7.104603965	0.028241781	50.97346843	10.15174179	74.57056534	7.033145315	24.88761334
5.703425317	0.041687019	139.8481972	14.02908195	104.3881678	6.052086597	172.8321276
21.10933398	0.0280844	143.1336809	8.087771209	97.01812078	11.01523674	311.9836097
5.467282739	0.114686618	67.05124185	15.68899807	83.63060618	17.63203479	182.0088042
5.108400237	0.082479088	165.2697394	23.30791206	136.3994844	11.127781	444.7536974
6.576826546	0.071010926	124.0244115	12.57316593	89.06699868	14.30983259	90.35609751
4.687852504	0.041124189	66.99902971	14.13998253	86.64360159	5.207273129	76.03826124
5.052331326	0.089542325	84.80857913	19.68826122	129.8248116	16.88893603	588.3681409
3.183335795	0.027216185	59.2734108	15.79505859	104.5536128	3.465490804	151.3773242
7.455019315	0.053662944	135.1212136	17.45713202	100.3834686	7.606972643	369.7563784
6.790109201	0.04436409	88.5911333	14.11926236	97.17920313	9.128514725	55.65187881
13.85208369	0.033764958	66.17085268	8.029600409	70.28897331	6.246418705	85.36048308
5.801356371	0.053128704	114.7467459	13.90436694	101.7320754	8.365148065	190.2382029
12.89316018	0.038055042	104.629624	12.27209985	131.5311055	16.45455293	300.2824698
4.983155364	0.056487884	83.69264459	17.74483022	87.07446764	5.840138565	105.8680798
5.859516056	0.020476492	26.07389989	8.72576452	68.22830398	4.588132696	24.2218316
8.846095868	0.005659203	12.801394	5.842022279	108.2282479	0.614891573	22.74890836
3.942688569	0.086824354	72.60807096	15.73530037	108.5042596	8.185173293	320.1305653
7.190854551	0.065363637	173.0068815	14.7717597	108.8504882	9.766525956	287.5080299
11.02453393	0.040037409	79.82876942	8.011871637	79.29945358	3.711903763	147.5880604
8.402108654	0.044761603	70.9304379	12.37038746	97.48798551	16.66642663	86.22223817
2.11093021	0.059231576	65.07206913	14.01583678	122.3904323	2.879113737	268.1916654
5.959252328	0.01199289	37.07221187	10.10506858	98.48475362	2.363039477	39.75428608
3.331199211	0.063377935	111.4081475	17.56363827	96.55089293	22.09032358	245.7426637
2.94497333	0.033262855	67.2148143	11.08472267	71.30598336	5.981112667	88.62065717
5.312705007	0.040113599	39.03366564	9.714233966	57.07763843	5.568789509	22.55653161
2.301203798	0.024086316	46.01203468	12.56206545	94.3958257	4.370050007	27.26420574
6.053948326	0.068615298	107.3019675	19.86674974	101.2263081	10.90076702	79.84567934
11.31835303	0.06029383	135.3544683	11.06041082	77.32995036	15.74971789	134.1968955
4.071997885	0.077883248	145.4147615	19.27623243	102.9169035	7.465629605	216.0571758
8.07314084	0.044729541	231.1810409	10.20569498	104.832798	9.867158407	386.8631433
8.534445714	0.039633665	95.12038191	8.106045044	64.01989858	9.368168749	155.5594006
4.666928137	0.01914527	50.35501027	11.21310564	81.23361909	3.81309362	64.16373501

11.91698624	0.040286197	88.03795496	12.27741574	85.69704035	11.99293535	69.85767931
3.614768984	0.022648167	45.12695513	11.47380357	85.18326931	4.784401233	42.42505495
3.510066074	0.043016019	72.73120081	12.4098026	87.96507668	14.12583846	87.66867101
4.971909855	0.078640661	157.455108	18.95812087	104.7590033	11.08029013	203.5669191
6.168572903	0.031197697	107.4412212	12.50694012	105.4971597	14.52102238	175.4813352
9.398879165	0.060407973	122.7932111	15.83235762	147.3941243	7.882417705	304.1402487
10.50740508	0.091429685	114.7356131	15.85634568	89.91103815	11.89064778	115.3553011
5.873137994	0.038438823	73.16067409	15.78160901	113.5095686	6.176603015	65.01052971
5.620068671	0.052400362	75.59384483	12.22192725	74.07320258	8.231680925	45.8278693
9.066796271	0.087538522	106.8914414	13.97441727	96.1808903	16.00816203	223.501718
3.96676424	0.07192299	89.37209821	11.69695678	74.43349498	7.857884222	72.72597501
9.017986791	0.066034769	86.05069371	10.15021616	89.69212934	10.59997951	93.49592705
4.979716378	0.015380132	85.81435964	11.60221609	110.4008135	11.00136994	261.020684
9.92278199	0.067010496	125.5190326	12.51450039	87.92848583	22.63071881	268.2740326
5.991087991	0.025912193	76.86562966	9.201697009	88.58061828	14.31198885	179.1614203
7.465039166	0.068176963	160.0397429	17.70584186	95.51448319	8.562513289	436.3316731
2.937470901	0.03918142	106.8900792	13.17138025	89.22633461	9.704834545	69.04160746
7.52182758	0.039249622	52.9891533	9.739490413	73.73064112	6.92705213	53.66458265
7.273043784	0.045144237	89.14940181	10.98006761	94.93556906	15.29462594	86.46122026
17.18211339	0.068870799	101.3894145	10.67344614	68.18706534	7.026861302	34.80033977
8.562054745	0.023054279	53.98577292	9.106946778	67.333331	4.648293627	37.31299759
4.40729968	0.041645927	83.54260164	9.086522581	73.43666603	6.668972203	80.61057861
3.543157465	0.033838439	68.90563787	10.00903886	90.25414386	6.294494611	220.1599996
3.432744255	0.035881534	72.95875922	10.87871689	104.2203869	4.942197455	71.89134684
9.39976119	0.040665043	60.01385965	9.209917941	70.3519217	9.268618757	108.7324449
5.024498004	0.097152465	178.9655239	16.63401248	106.4259247	10.66461653	98.14896982
7.822146452	0.023208382	37.25144792	8.444868107	66.73158973	3.554379092	23.74897231
5.672809338	0.034690477	39.20044302	10.77575512	64.17078046	5.126676983	18.0019372
6.100689977	0.017918277	62.68282815	9.643494284	84.98134745	5.52624475	138.8710639
6.853325153	0.067567663	117.3727609	19.01855877	97.69974196	16.49991779	95.59374739
9.518433725	0.100479903	209.9327355	15.16359574	76.45956899	8.98214586	109.6181066
2.144183901	0.017104313	20.26204871	10.39971193	102.3754263	3.455972662	85.17558766
6.286617546	0.038386807	51.70368182	10.84450888	69.11563595	6.957886325	48.34945583
9.394846975	0.044103553	190.2575314	13.55286723	108.7423488	13.9132208	469.0618067
6.57741104	0.032697315	77.16631989	11.33975243	84.23722834	5.658427363	66.49012703
11.35295794	0.058179504	87.09338668	10.24321241	87.53125116	20.34156907	221.844873

3.099620543	0.024071001	60.24359698	11.95999095	93.07535023	5.161573215	94.96798824
7.738094113	0.06585247	166.2683948	13.68843831	80.98195795	5.551797135	42.70340975
8.805820196	0.033943884	56.24750248	9.607420186	75.25575012	8.973853992	64.35155552
4.640039168	0.066932108	66.13249705	15.71686854	92.41462629	4.937393952	36.58996473
14.18953683	0.013594961	36.56699284	7.357779925	75.25534179	7.966777502	20.90627842
7.904853393	0.093410153	131.0825634	14.2896521	95.43164295	18.33160597	72.1152607
7.558163327	0.044348634	54.65239373	19.35546803	104.7983235	8.010306373	33.88671852
8.250260622	0.062935216	92.29384242	16.0976784	87.33405948	5.914149549	48.1141388
18.67368117	0.063146023	134.2369263	10.13757488	81.99301234	4.902853148	338.9345137
3.879684534	0.028079979	55.78108793	11.74346536	83.15544932	4.245713117	24.87819633
7.466164696	0.062499445	107.6280698	15.41838603	86.07056137	10.60524972	63.75392744
9.368139741	0.095105983	78.39119495	18.26742562	114.9227195	11.89209483	151.1667229
9.932194973	0.15023055	115.0648483	23.04306825	92.47213559	6.917896638	63.17324161
6.123805494	0.044879102	61.71601845	18.51845105	127.6186035	4.245423983	112.9597604
4.789563091	0.022325835	70.11054112	13.88476489	125.7463757	4.287050026	9.999126445
7.708135374	0.05176891	106.816483	12.15110555	114.7953298	2.979543136	123.1351508
7.432688821	0.031916731	72.1569213	9.683218512	71.78535348	6.910816752	40.31836293
10.81436956	0.068727815	91.28337176	12.87051238	78.91159189	6.296428953	174.0251493
8.418566103	0.01586408	57.42778089	11.82889876	101.4975296	2.828528332	40.01500039
16.45942148	0.158596857	174.6542593	16.88591188	90.95617606	17.34822434	122.1299189
10.4935868	0.017404395	35.82680114	11.13780686	76.38222953	4.067178769	24.10849262
4.632433869	0.02009822	38.46859533	9.429707009	68.64689168	3.114976613	36.84826135
7.739142621	0.030711848	68.56916579	14.04173308	77.11302451	8.345265984	27.93276363
8.726790977	0.050332453	56.06657799	8.871551753	62.97597782	5.808308467	23.36821932
10.1465536	0.087791227	114.8303022	16.01159523	90.05456989	7.436377583	76.37860171
4.881168043	0.016553746	70.17230169	8.492771336	104.6022338	6.086567616	84.04113231
27.78086809	0.198636163	212.9701511	20.92622417	116.0962011	9.455366192	402.8294025
6.413034216	0.044907895	109.0319102	14.86998477	115.1017575	5.724380059	200.1088277
8.311221828	0.047549239	83.22572249	16.71339742	108.0816427	5.773057961	51.90893198
6.47955764	0.044573012	102.67098	11.29742617	76.80617676	7.870877504	65.7073253
9.288032562	0.042045683	89.37424605	10.01482062	78.50616721	13.52541657	86.03444611
14.26191998	0.106314595	107.363787	18.30489859	87.52509461	3.127937429	145.2896316
4.124906748	0.037355313	80.85454606	10.91157954	80.52111106	3.582050475	40.6920289
2.938198746	0.029053548	87.61403363	16.85557481	88.82196979	4.777892276	115.8638691
14.62648141	0.079814276	165.7661153	12.7660042	109.7894349	5.65757601	275.6539967
4.528777722	0.051057402	62.50516131	15.3933652	107.4630022	4.878007218	52.12111901

9.481080339	0.069063145	92.43349507	11.50211972	95.79822106	6.08786942	119.3352024
3.346195279	0.020799502	34.93765778	10.61431664	65.45562078	3.046108139	18.56487982
7.487397296	0.059600643	136.8790892	12.51320503	66.46190265	6.042993526	44.41938962
4.392869875	0.093124659	83.61612179	15.9664434	84.68671882	4.923336719	150.0842081
3.566051006	0.065412393	74.67122698	9.913517474	65.21248423	5.514468677	70.93171867
5.683404779	0.126498162	173.2840416	13.88956071	70.84974915	15.77902426	130.9459861
4.709210947	0.023690761	50.30516312	11.21616688	84.0563235	6.777719279	41.78658406
4.27043934	0.063299751	84.1926848	16.74626445	124.0928928	7.453117054	123.85317
6.427232137	0.030427101	85.26229367	12.42963717	81.90529552	4.058881266	69.79335268
6.009979707	0.073494808	116.2342238	14.43143482	89.5581092	11.62225507	158.7424777
8.435039975	0.040476527	121.2317382	16.57621899	114.064623	4.600075983	174.046786
2.890474591	0.039449066	45.15642451	18.7154719	149.5350991	3.429477642	102.5492342
7.434969478	0.088232834	203.8156123	15.13495665	119.3649515	3.795554002	283.5203375
6.063809969	0.086710558	124.7991494	18.1152954	94.75152308	5.068345531	58.97118858
2.59313749	0.125844508	107.7386591	23.11758216	114.5554996	5.736392367	452.5296156
6.852916668	0.03669163	43.10365794	13.37507303	105.566384	3.948541983	27.36388523
10.33994029	0.076435364	109.4644763	13.2417441	79.71085975	9.183101128	60.69196886
6.159726105	0.033357714	93.90109886	17.64790852	115.8691699	13.01834435	87.66668146
7.161410778	0.053862769	95.23036094	13.23395348	78.01389833	5.214472565	38.19387855
8.824392752	0.038300036	60.25198063	9.604069172	72.82547544	1.652883102	128.9012767
5.555112022	0.042283735	73.17539105	11.91470265	116.9651346	3.605839409	46.77083902
2.394029065	0.027987268	48.5949933	12.43238825	73.94365165	5.193599627	33.92372801
6.965717928	0.029602732	90.08665454	16.90475723	143.0907683	8.932067458	153.7923229
3.044016685	0.047543148	102.6654923	15.59891473	82.43876207	1.808129275	114.0732224
5.012626476	0.065413904	93.21465972	14.11480104	82.54790067	6.300132111	46.39735567
8.594908857	0.024666726	71.61237021	7.93078238	61.13924627	9.712986927	27.30040223
7.882213933	0.140316925	195.5222977	15.47506403	99.59062206	13.80720883	114.0785842
3.590770278	0.027508825	44.52229157	12.42983158	75.23272561	4.598589609	16.70733443
2.641312059	0.015022154	34.14714458	10.02969416	87.21685603	3.122774952	10.83898028
4.103781405	0.034803092	65.72987014	11.56648676	86.52005369	4.964464488	344.789969
9.539433954	0.065818812	123.2676972	18.28486836	97.68920266	7.095824327	42.23667345
4.573314116	0.055630333	61.31452184	18.31302658	92.0577841	1.58870746	53.01974919
4.987556014	0.011987292	27.99743608	8.517875077	87.26547597	3.667323794	14.128919
17.18917931	0.24638069	208.4368324	21.65854231	88.11173395	14.50248526	217.3904818
4.704741379	0.029733615	66.28457204	15.82324511	94.25173198	5.095012438	103.8494112
5.787052676	0.017648495	68.43406357	10.60917632	77.85785301	4.44918807	65.98510087

7.50311591	0.07085675	81.37694627	17.70958893	90.52902573	6.163518681	35.65896297
7.43518122	0.037541956	56.6120748	16.58092122	197.60006	2.325152498	136.5384615
3.661364873	0.034135539	150.8793877	15.1805352	81.82538633	7.72545024	99.79885993
7.869970979	0.073287571	118.6978412	14.3121555	71.98134747	7.052975502	40.94789122
7.292872123	0.035583799	67.8579835	12.52595844	73.34387479	8.5451461	20.08793405
6.617266307	0.06417928	75.08172218	9.528560835	71.47476918	8.198048581	60.54066567
15.97141096	0.04447929	60.53658242	8.361617503	76.67524229	8.93678104	45.54891618
7.32933619	0.031805479	56.06045329	7.454915467	59.09861567	3.438928648	57.51235598
2.981285494	0.04077051	80.53852351	11.17284864	81.74517993	8.489406531	64.21068626
8.149829528	0.11369819	116.063306	20.65906075	94.89535163	10.45717218	88.40695083
12.6583519	0.038661123	80.21330331	10.16452031	70.68967425	6.573301057	33.54230455
4.188965383	0.021551997	66.32492817	10.781853	130.2957326	5.432233755	172.4431091
14.80818689	0.131226209	45.51256324	12.67778353	96.89217464	5.956626867	68.13729126
5.008997016	0.023586986	73.94447713	10.50259075	103.1517617	7.98449858	109.2665284
6.907865455	0.039795548	103.5303566	12.06187869	74.51112373	1.622634906	23.67966273
2.668462178	0.028518095	46.04096695	8.728294162	68.0218965	4.226604797	32.65612101
4.696263941	0.041089152	53.2080894	21.43780285	100.3990492	3.204508814	31.10488521
9.232685167	0.040141223	77.97290362	12.62836539	112.7326511	8.088638274	43.34051245
7.753855921	0.062018548	90.30582975	14.69593031	93.0621241	8.536425596	66.85224793
4.474968119	0.054483799	92.43382824	14.10744797	75.95541681	5.323806928	17.24923003
3.846314777	0.0549912	96.952327	22.85373614	97.72671176	5.769618571	189.1489592
13.03409023	0.044201528	54.71929783	14.82562838	90.9246412	7.616800388	37.19719715
4.356871807	0.034861025	49.61830527	10.60823357	101.3866579	9.383026341	42.55845875
4.713227355	0.040735158	59.83213102	12.86170214	119.4785783	4.322089617	89.29248647
8.64078416	0.020322912	97.76477134	13.64610456	133.4256015	8.696897233	191.8987785
6.968267094	0.049891386	88.13442165	11.25088873	81.8519502	6.31786989	60.6178593
5.789493868	0.036066252	114.0616326	14.85556923	96.67928778	7.830674359	41.05220021
6.508461018	0.107174273	139.7976119	16.42029371	94.96145315	22.67593205	130.5044209
6.905458724	0.066666189	95.53011004	13.9282396	74.80420202	6.720838831	66.21459035
4.93220976	0.089551412	120.4070527	13.30555531	90.26097476	12.34599771	107.963888
1.996037555	0.018986269	74.36547857	17.13709698	138.1621452	5.062141685	120.8104836
4.742670418	0.039724303	83.48067255	13.66505435	106.3704407	6.00405283	52.98168326
5.874862353	0.02697988	64.71643791	9.78374325	80.50428863	2.926830938	44.72104841
7.735496365	0.034734279	130.1198598	12.03173735	80.89623705	9.939090195	61.21430044

Mirin_1048	PD173074_1049	ZM447439_1050	Alisertib_1051	RO-3306_1052	MK-2206_1053	Palbociclib_1054	Dactolisib_1057
228.093789	92.23375328	28.99905209	19.17858638	19.88973677	18.39842028	23.52347603	0.113418066
115.284739	25.06803672	14.77534162	7.441547869	13.88308102	20.97079135	45.43922853	0.178087619
133.308668	96.82821228	21.00253451	10.12079235	19.04178743	35.25004915	89.28297012	0.429683358
182.240627	63.99429483	21.1613	4.03872985	9.568622483	30.43698274	52.39505035	0.246794355
216.243799	129.5963405	28.06522045	15.46357543	25.42013149	47.18422684	106.9927336	1.025144704
97.9746029	11.84622319	18.18227878	1.328034259	10.96137907	20.94458418	22.75604992	0.135686928
87.3365004	50.52551621	17.60663468	3.01517966	17.81749975	22.96737374	25.54711538	0.19991293
170.5114	86.10770455	20.67646618	16.91965962	15.6255053	24.84390335	67.69771755	0.373614519
210.445273	154.7740382	23.68767702	22.66378968	23.43607481	45.07552829	87.74415497	0.471746785
69.6257695	25.12408025	11.3753084	2.302125501	13.19350468	10.52823572	31.17086246	0.161486028
108.90865	35.30472914	15.14804793	6.751964475	15.67549615	16.11134425	39.00455879	0.225376749
167.150242	49.53139394	14.43953627	13.34976725	14.56270523	25.13993551	65.98872386	0.308238265
100.439872	40.700219	20.02075899	8.076886507	20.73983785	11.02789636	35.65520996	0.158638251
518.217089	52.18987586	16.70640359	14.11570107	8.945902705	73.1676536	143.5137417	0.556521495
100.147935	64.26848605	16.55181404	5.667331744	21.52625415	24.13540801	65.1436536	0.319797913
106.200554	38.43959824	15.81830361	8.48993491	14.78593624	10.32992814	38.27272324	0.310633375
60.1483516	20.21578538	28.43669275	1.810594553	21.12256279	20.84044512	19.36336869	0.137653225
205.429507	86.03615705	27.9676764	20.28754971	16.8351177	90.77481155	36.90058591	0.378340959
102.340374	82.41926962	19.29619606	5.276454002	19.95378761	40.71137475	71.50738282	0.429203489
61.3304738	31.71030427	16.26377481	2.043042756	15.33132711	24.69643559	26.15552192	0.110745777
105.199032	54.5213394	13.56501903	7.338850855	14.00207067	18.47304633	42.8674815	0.262549014
161.623579	126.3665025	24.75348703	33.53030015	24.24799327	74.74393665	50.93310173	0.354503184
74.1423189	77.24515099	22.22076481	2.737010732	20.4229652	16.50901063	21.14803302	0.129980576
155.488988	152.0503543	16.70370063	14.8802794	20.75920443	21.85483365	62.20034463	0.253843702
94.952076	48.65338325	14.63277008	4.853986197	17.77676976	18.72826253	51.96361517	0.215048565
113.409206	52.11151038	18.11097641	14.83041965	17.37489108	18.43147926	42.77688956	0.154181136
354.79717	125.7080515	19.03310074	21.38101049	17.32839655	74.40527638	74.90800174	0.754309225
157.485908	116.2244494	18.86518388	7.631615265	20.2967271	33.97202363	56.51854829	0.340383118
72.3922184	68.09268858	17.7498946	3.217775161	17.06691407	27.7487629	38.72722939	0.20904762
142.014282	44.45611694	20.10643575	8.933076583	23.40347291	17.32874539	52.69209467	0.213324172
140.160023	48.51394045	15.70994648	7.705379907	12.20772267	14.80305108	34.95232191	0.194975588
183.926049	86.53594068	17.58150145	3.295675758	19.84347775	19.85135365	28.67055172	0.121803531
235.955415	125.2515763	19.59053254	17.25738731	21.44704288	32.32501223	78.36281152	0.261664072
98.9914814	45.63595211	17.809844	5.90168084	15.18929562	24.02677113	60.50538222	0.320909736

231.888763	87.8950934	16.66109987	22.51144757	21.95370736	22.18982617	64.72195921	0.313132993
109.341229	149.9965525	29.61651273	11.1876622	20.84574747	35.40978947	79.87319952	0.254204419
69.91867	36.2792503	18.72498145	1.89606002	22.84314661	20.33450966	39.56148702	0.145350584
243.664364	171.0176441	20.91004865	16.36072636	15.53581499	35.50403505	83.92165888	0.240351095
134.46319	71.05973214	16.85201088	10.21955303	18.90748089	14.13277395	30.01287794	0.215383472
84.8612414	41.26149689	17.44448745	8.298893921	20.19541233	18.06786765	59.20764741	0.400066633
170.949202	85.37173634	27.78212779	10.96562799	16.50335786	15.66078501	90.79018163	0.075357516
140.727763	59.07283712	13.86919818	4.790052866	11.31616564	16.99127612	62.2198634	0.231107796
121.282025	78.82953413	25.11572432	11.039035	20.24461961	12.42842582	16.67317696	0.109458377
177.647172	43.54568649	13.34416278	11.99368356	15.22970745	11.6674621	50.51237084	0.319196661
95.2027971	92.4183907	26.04467824	10.01070314	22.09451967	20.52081266	20.93305222	0.090365737
97.4012305	4.027035448	17.42487918	1.956594518	12.91513325	12.89511399	103.0074746	0.176777552
71.1095891	48.10720517	14.93141259	3.457284258	15.23660212	15.76565416	29.68979942	0.152604661
124.26283	85.41150251	19.90567338	6.878702271	16.30055456	25.19921465	51.8272952	0.21564731
65.8779244	36.2896988	17.24567707	2.431052083	16.46098394	18.34897509	37.04364732	0.243906042
108.201571	42.00730702	15.01958096	7.93996588	19.16750966	16.63072487	32.91833323	0.166628002
48.2889726	15.93808247	12.85154941	1.456632467	13.81252157	8.984834423	15.53376677	0.097532326
109.901662	65.88495209	13.86916584	4.930323267	14.51172737	22.70940246	58.92761595	0.236555793
98.9351007	62.43022218	14.07197587	6.670848188	15.48431132	23.17656633	40.0783317	0.319500244
94.4826089	40.64552758	13.88129894	3.264146646	18.35003991	30.4937714	38.02006034	0.185936055
134.156954	92.93175857	24.8759118	8.632200864	13.03008427	36.32631916	24.72505785	0.115531306
204.706553	205.2811986	30.69935569	17.97684482	21.09109237	58.6965476	61.11309782	0.386689861
74.7768715	39.4300647	14.97567994	3.053113524	13.94858553	12.17536472	24.03661779	0.178740462
60.6685174	27.24502857	11.91054059	2.045651995	13.67635724	15.82293792	23.08269459	0.18965056
208.314629	221.3409346	46.96682624	21.30317939	28.273624	24.27689228	44.70858033	0.171786907
104.931012	73.5969935	18.21442058	6.809971598	18.71877411	22.4576648	29.555203	0.155299615
262.234913	98.54435066	18.46671767	22.2525129	15.74159995	40.60397646	93.16889355	0.490471026
163.398034	42.50023952	17.64380555	9.37939588	14.41397434	22.57903555	61.98065376	0.286983689
299.24198	133.9702681	19.44824942	30.96794121	15.7411492	48.25389281	107.7608619	0.525030696
71.6716628	81.00299208	19.45457251	12.26093766	15.17579981	43.26999292	46.0868622	0.290873845
75.0663394	45.97600276	13.8259483	3.245373669	14.75475843	11.33039884	21.82066983	0.109510944
68.8394941	33.85299297	12.76742312	2.429815625	15.0514937	12.42427268	32.96966745	0.125907941
81.9323753	118.6583807	21.8894969	2.763002063	22.14631158	64.54187982	80.40834545	0.363247969
381.780734	335.7713859	43.64588437	35.07904874	27.4482636	69.32730921	164.0724034	0.579290196
167.151678	102.2138422	14.79965612	8.843770824	13.13908176	30.23141862	75.2569064	0.307302121
84.4316459	36.71988468	12.39892589	3.552230829	20.73067814	5.904244299	37.38812992	0.176581628

120.505575	181.9268958	21.74644208	7.616551509	18.47349045	23.86845148	26.2921815	0.266599074
113.860175	32.87013386	10.24247126	5.790766539	15.00533167	7.175422168	64.98579674	0.228556012
116.997398	120.7045694	17.08966997	8.582077591	21.3600147	26.24585495	63.2965685	0.291140294
336.252162	220.0583932	42.09640801	41.6852394	24.23440017	110.6795669	149.7407423	1.149994736
86.4448709	54.16597483	15.1926834	4.061156261	18.51568941	22.34333937	47.83549294	0.285380673
85.856479	38.04965307	14.87875761	2.349560898	14.68902573	9.520680791	29.96862954	0.128844867
66.4888401	34.9964692	20.77152814	4.854311999	21.78006202	16.37196997	26.15691986	0.203155295
118.61249	89.56327005	20.70282847	8.781826498	20.00962447	15.91151977	33.53646713	0.138930838
179.587516	55.73036559	22.99154987	14.65703514	21.53312998	13.7192164	49.88560876	0.250367835
133.19295	25.52739824	12.98990697	9.595729411	13.92533315	16.3593967	49.60869197	0.246003743
161.436654	180.2938579	31.12429915	13.66605133	19.06293742	71.474301	111.9619255	0.275144482
205.820256	131.7794952	13.99072009	18.09219782	15.79449797	38.98349621	108.739863	0.374953792
77.5187295	37.12554711	11.50191166	3.840062193	14.03896185	12.1352693	16.81403646	0.095545811
62.9827866	47.78952555	15.78197649	2.21161593	13.47502925	11.09235872	19.75754516	0.102725343
156.854533	88.45973965	21.74674949	13.65547445	21.02110805	38.71989652	54.16130055	0.409618545
153.861812	88.73983671	15.36478982	8.406293978	19.01373634	15.57583538	90.01774824	0.286231025
103.241399	33.42782198	10.5411039	3.154904386	9.325464485	15.32992553	53.4280899	0.292712491
107.654142	41.13814201	14.13371221	5.449262868	16.44792159	14.57347422	27.07299409	0.175342361
199.973206	85.60453609	21.94283345	13.5961866	19.49285468	50.45301847	121.3084324	0.588908826
87.1765148	38.63142703	17.87863505	3.154254078	19.73023058	32.94988508	32.18151151	0.196237249
157.826527	58.69566365	21.43887332	19.86587084	17.24155823	20.26468527	37.78404422	0.321360003
91.6099749	51.5090418	14.04386503	3.399697887	15.65909287	16.6323134	14.64094962	0.138054263
120.648646	134.2015527	28.8061095	8.458212977	19.60653392	34.40871851	27.07672603	0.181892026
83.9334715	36.79874689	12.75261119	3.921795638	14.37168193	16.69087004	29.90210988	0.241303029
120.718874	39.71272411	16.72986472	6.412936223	20.17225057	14.04512613	22.40257556	0.130634882
87.0438704	73.23836503	15.66140169	4.323724341	9.749738865	22.15574806	35.46444057	0.163365647
107.985139	42.19903214	18.0378382	6.31906989	18.24766354	13.31721974	23.91779446	0.114441347
81.7470224	67.52764828	27.36123323	3.448707132	19.90621041	24.2178392	25.56482788	0.103513817
207.495241	65.17522286	15.41891017	9.407145423	14.14273248	19.33064039	72.87864485	0.195352286
261.480962	75.91618257	14.7676448	5.668597461	9.447260727	42.60389886	80.21506437	0.391493058
194.112679	62.93036525	15.84566412	28.32442044	18.63165721	28.72201598	59.32508384	0.233840496
146.370269	78.28072831	15.44067294	7.669687371	19.36612368	19.97992269	56.02017694	0.217795926
45.1161174	3.974814416	18.3175009	2.36795055	25.89467228	5.520222422	21.65494129	0.168432053
85.5443432	34.31151173	17.414599	5.614324116	16.82952843	12.09648262	23.47914424	0.13393633
119.820782	35.93864948	31.16655895	6.886465092	25.77250327	62.28246086	108.6133404	0.312122359
139.374499	93.34559755	20.25943635	8.212765715	18.93417179	31.92473433	31.28071474	0.174643552

156.582645	53.00854614	14.37595082	7.275771052	15.56126593	10.94295115	47.8749262	0.170157254
160.785218	44.98972475	15.08992781	14.09749344	13.74084255	12.72521407	40.14526779	0.272479792
87.0394794	48.76508823	15.05299304	2.691684425	14.53782464	13.49746653	42.09171671	0.250884802
341.707607	126.9837905	31.440931	29.3312156	23.17167492	43.29154259	59.95400802	0.365996375
97.7150481	77.80244938	17.9714568	6.871289238	16.19376	15.64708512	53.58949289	0.232031436
103.460301	68.21683605	18.22375763	4.227795366	17.21377605	30.8513969	34.88561989	0.148071068
293.922344	126.4181238	15.8784744	14.34594222	16.52005582	59.31000047	101.0075975	0.530431394
99.909614	47.52538907	18.94784329	7.694599042	19.9604454	14.08702349	22.18211648	0.175549866
249.347229	92.53601398	18.98387409	19.16730171	14.38824901	23.71723766	52.81055952	0.282965009
115.394796	49.70742391	12.69591587	3.251797493	16.99998337	18.45582717	50.47315656	0.274496497
232.818899	55.4326843	19.74295965	6.2329107	12.83750084	28.59326481	35.51746418	0.235138695
218.897512	163.3322586	24.09014235	28.09104475	15.43507517	33.98249911	62.31723418	0.227030723
119.24577	123.2431861	20.2324259	8.987388784	19.01740595	33.83170804	72.364135	0.375446443
66.8047577	41.70864644	13.11878839	4.347838107	16.78236479	14.99857371	32.58422815	0.213522681
87.9973398	18.81073907	16.55714897	1.077193705	15.41447799	21.20647942	93.38603932	0.298361077
151.49237	40.24230654	18.8156742	4.775940792	19.05168896	28.07778184	48.33880029	0.287545898
95.4812192	49.04243421	14.98370034	9.205358638	19.38762947	13.53256492	37.03377546	0.207409794
100.135197	39.41304339	22.58643281	4.213986478	21.84520787	25.7807916	52.97674857	0.204428022
88.7244694	42.71862877	18.98983422	3.812339656	21.58087977	34.65433591	59.58361216	0.441950044
236.628591	58.92824802	16.19543695	13.68926689	14.66489195	7.993243828	23.51383136	0.12041523
184.690324	78.09908232	22.26744589	15.13589782	19.33028533	32.87718143	53.88512394	0.307622872
91.0503678	66.39935415	16.72079115	3.320085709	15.4990053	16.02511524	24.39029129	0.128650542
97.0931136	39.23651216	18.33407752	6.385828614	13.80211414	13.35762399	15.3896951	0.10057745
119.082443	76.23927448	24.24076116	5.047274231	23.54827111	48.30463857	40.37123665	0.192076445
166.065516	31.40740296	15.35868792	9.808836481	13.85302187	10.21857726	56.93207337	0.253646221
98.9012869	47.46392191	18.61584436	2.953826358	18.38640149	33.54702899	42.40499146	0.123293391
224.013176	338.5121876	25.39975066	19.30261218	20.34209615	80.81507976	134.5978104	0.769065655
79.6593697	68.204301	18.25961373	4.021677018	15.44970754	18.38691061	28.39525034	0.13302163
98.1121992	76.10971079	16.93489531	5.996780316	13.80538481	18.40761338	38.52102908	0.19975174
211.68454	58.97967488	13.2678821	6.841103051	13.08589267	35.60513133	150.6100164	0.608901566
132.581808	117.1404144	25.62198484	17.59917688	17.93009225	36.83024883	58.89239322	0.250178303
171.271711	51.79300118	14.10331702	12.26027116	13.81876994	20.52266006	56.10297948	0.203574743
89.9336798	31.87181156	13.64065978	8.322063422	12.07440878	26.36540988	50.86364248	0.246758568
114.525683	43.55041391	16.52375963	6.214606673	14.63469783	28.58432826	43.88711569	0.306909682
90.0139526	58.38720936	12.03690187	4.678236344	14.21402569	16.3336649	35.96587526	0.288705497
192.258364	68.18331321	22.08243334	13.40618773	19.64315538	34.90626501	69.22332295	0.32870966

89.8876462	130.2114742	18.93688747	4.879228898	19.12338389	30.3099787	32.6500289	0.146392701
169.496432	74.97622833	17.9256755	9.308346547	15.8729668	24.02133736	83.30566792	0.281878869
118.433989	71.42985045	18.6079137	14.66572115	19.73569961	20.12728009	51.17729214	0.272692076
102.369529	95.4975167	19.92869456	5.922898897	17.96036087	18.64116659	35.93593176	0.145282733
306.332863	220.5276815	41.44772108	46.41684941	32.25663622	156.7019898	94.43943002	0.49811195
107.187738	56.50072434	18.6908821	10.91220172	21.06852776	9.678141936	38.29687333	0.164263453
138.490678	62.66860059	16.39553299	18.65157535	16.05444958	27.30663593	64.57785193	0.354730067
82.8732282	71.17023525	18.46676407	4.540542567	17.03993307	21.10035434	29.32370167	0.142722594
80.6003084	68.42393279	18.33606355	3.730171881	22.51325449	21.77910061	23.96880411	0.195799176
148.038108	40.19065788	13.48848063	7.71821377	17.90717085	14.26909888	52.73751098	0.2024474
231.874913	95.86668572	24.74239091	30.18359145	20.20602836	44.57284979	77.76244952	0.465377191
136.579717	116.0633122	23.600351	8.329271282	23.85974417	18.30497104	35.93137292	0.143039936
69.7755817	30.94385989	20.34220586	5.909296518	25.26962063	18.06488609	37.24992349	0.214037944
220.977886	85.71305861	27.31593779	15.69616845	22.01452545	46.16918488	108.4746653	0.359562659
88.8374588	21.48509806	11.91903402	2.132721423	12.21550032	10.33379539	23.3759381	0.140137734
184.482525	83.4648259	22.05706133	20.09704202	22.21497541	23.86859269	77.04049358	0.347371026
25.7669399	18.20284099	20.36705919	0.175086124	22.76055501	8.733605595	8.618713455	0.113180399
190.92193	81.42857991	16.66001108	19.48284633	18.50915031	22.74747989	86.09936018	0.399315633
114.477294	59.93265086	14.33262325	5.822542067	17.03161035	22.28749663	69.46942312	0.320791738
81.7427701	24.59426026	14.72429107	3.069657251	13.43665526	7.172473985	14.85674389	0.094939729
113.952055	47.73805602	24.05132118	14.48453629	24.72607206	24.66754523	33.95452738	0.232418856
81.6384643	44.78708945	16.60702804	3.796544225	19.26292476	12.24108673	30.16549748	0.155701108
144.804443	91.12501476	19.82763751	7.918404509	20.73526666	15.77638073	26.14308388	0.224659362
92.4359847	101.1653061	26.63623744	6.735307713	15.93964354	26.99971312	26.74569696	0.158672895
142.581474	27.69576743	16.21507984	12.85168351	20.57734623	15.32821567	65.4820202	0.30448798
251.276098	101.3742	25.19180261	16.10050912	20.97794092	42.39233002	64.06187781	0.483599329
127.241669	38.41003603	13.30010224	6.711601925	17.66718485	14.33080642	50.72313198	0.344407018
109.593182	69.04812443	17.56800548	4.593666571	20.26245722	26.85046841	40.87223338	0.181087643
72.7460685	44.96339606	16.51151742	3.121822316	20.01326938	14.35808877	19.93148328	0.209677819
72.194506	46.18827803	18.2745705	2.178680813	20.27728137	15.50316023	13.86854231	0.083425978
76.3680626	44.57594654	16.97451933	4.665461084	19.36020545	11.0030542	28.42980336	0.130970295
90.0399022	60.88493795	23.21264008	9.18529979	28.16116318	11.46531103	20.14251728	0.083475166
71.3492681	57.68409803	14.61294806	3.13880394	13.93494916	9.274796306	19.48575753	0.104780565
356.743655	84.80522718	33.55861914	14.94473453	23.82585782	19.1004453	67.20012164	0.293517375
102.453577	73.79683319	17.20799486	4.356252784	20.0302478	25.173518	27.18541347	0.119928499
102.937659	24.30330409	14.39511693	5.142129987	14.99734307	11.08046101	47.39813127	0.165363435

81.7688798	26.19014777	14.11760548	4.029424999	16.48492185	7.181130667	16.29208131	0.089366287
204.865624	72.00989023	26.12151816	7.238323542	24.01646672	39.31455691	81.9080705	0.426157868
102.371405	68.26318762	17.67203717	5.97733681	14.07780858	21.7414136	41.91504135	0.170529427
174.724792	146.3321409	20.89044784	20.05316271	14.13951346	49.74007685	48.53615969	0.184448163
95.1963749	54.66043266	17.31977229	7.242683361	16.23313713	9.334990265	21.35058022	0.13829974
219.742444	174.5198422	27.60732616	28.4597136	16.94838735	58.59259825	79.78451493	0.474543244
274.631995	102.2646346	21.39244241	19.805819	21.08058126	14.24628733	56.84957089	0.20945544
100.148931	30.96792243	21.32824209	7.549709558	15.8938438	30.5118473	81.03954799	0.241030345
104.609671	34.70913573	11.42055504	4.524643004	11.92335072	10.94515702	32.65417933	0.163142248
133.886041	73.49950369	21.06329572	10.05929969	16.818888	15.45146525	40.41425036	0.150372148
62.4090243	59.57685767	19.99383161	4.765063856	19.92838627	10.75242089	18.49317817	0.148710102
142.366914	36.91098054	9.812247683	6.877427201	10.85613642	14.14818454	62.72879055	0.319406556
99.5932636	20.62158811	24.70034558	23.63717155	27.57834457	15.56389091	99.91616312	0.227962099
96.8605659	83.52518338	17.06399289	5.268851562	21.36014647	29.22654074	37.10566996	0.240100869
114.665989	28.02452292	15.14463304	3.866424397	12.13381971	11.73304276	30.57121607	0.179871531
99.0277953	43.6451009	15.75778934	4.151313259	14.45061693	14.62906233	38.0243941	0.163582894
103.882281	44.98687814	11.98157998	9.235987119	14.14952676	11.45044662	46.06389642	0.149636534
154.454798	60.94652334	16.28086053	17.56477466	26.65436643	20.83427694	37.16787507	0.290537484
97.5305597	30.92419463	14.74569885	3.621087674	15.91256854	9.23912736	33.68369619	0.136359144
47.9334355	13.37160327	14.20552814	2.717738566	22.64336664	11.32771177	48.3446502	0.114098219
82.5295291	82.38216991	17.97180998	4.363169505	17.91381101	18.81597444	32.51398437	0.228548647
778.374825	121.5402219	18.37366022	45.64447391	19.59164376	34.6228665	113.8213778	0.67487437
88.7587112	52.617377	14.87239631	4.54358721	20.02307581	17.20615449	72.86896573	0.211493959
92.8122648	100.9200974	18.38418858	8.582569118	19.06189036	33.57476781	64.41367289	0.321510845
232.788252	175.6514246	24.33334132	23.87854445	21.14263246	55.81148189	139.2870164	0.542489072
88.3153852	105.3685663	20.21624647	9.868804149	23.31063114	12.28950141	79.64232498	0.190019691
257.608424	131.2131308	43.34991218	15.5974358	28.43494259	16.68634569	38.80829119	0.135312667
82.8591102	9.908291832	17.05584502	0.533067416	13.06905285	8.335021508	17.79973465	0.171595272
61.9969614	19.0898651	12.82643709	2.114431271	12.36506375	5.612562019	10.89399125	0.080314667
125.445659	62.4326212	22.34420065	7.160267137	18.41705515	15.33345041	24.28104973	0.113591504
71.8183603	42.6240732	14.09052191	2.043822814	13.83432045	26.6514016	26.51869574	0.147124793
105.705128	42.04178518	19.23153126	5.287229371	14.49755706	10.06371775	19.58289346	0.137457791
145.029643	58.92392728	15.77918777	10.7060597	18.33610584	27.03670861	65.56072096	0.497232871
108.019775	29.5921045	13.20972245	4.320900904	12.89420173	8.299652522	17.94032049	0.117623936
191.501262	72.18551984	25.35575365	9.897999291	18.8524055	12.26754499	44.44932081	0.11048472
147.71948	74.45866681	13.47223942	14.22633021	17.12035844	28.05633964	117.3517422	0.52379506

132.763376	78.49542141	14.00400967	8.677467249	13.95860058	24.03953079	48.84231891	0.307038112
146.784665	56.74185054	15.53405458	8.530469347	17.62870754	14.18040979	85.48994059	0.35005692
75.0437967	41.40468319	20.81681019	10.67646629	26.65983483	14.23384872	38.7082276	0.189915099
151.261262	153.1266677	30.35180106	10.96752298	19.21791465	36.58816026	40.20578096	0.182425715
85.8342408	33.0189317	18.61029882	3.019206973	17.41174186	9.956415794	8.733227598	0.100838807
55.7135749	26.63786721	15.67152332	1.971841478	13.93115628	10.70317127	27.62139389	0.09922947
160.568673	62.03480327	16.15547784	6.884737792	13.48015753	33.20052192	63.43491771	0.371744984
107.654367	45.47160884	18.08843167	5.266809663	15.54849075	19.95190348	75.85895825	0.314912059
116.133693	92.18975318	25.65567536	7.576224359	16.00847586	14.2980072	22.97556802	0.07888097
141.386031	100.6455149	21.19622979	11.16311424	15.14374854	24.40189652	38.56173354	0.268606825
176.844605	71.8969832	13.40479061	3.296244613	12.3459689	46.57425697	102.199982	0.52691131
99.8481763	198.5654165	18.53956323	11.13442411	20.63721874	26.47236238	49.44934184	0.135162099
146.271882	95.29596444	19.8726595	13.99896391	20.84841524	20.39527296	44.43193892	0.215602719
119.393332	66.89613425	12.45792972	8.574384591	15.54523815	22.05904419	94.5894487	0.334495317
63.1180333	16.12163374	11.13662329	1.367957205	9.006467568	11.23737684	29.24864585	0.193736874
135.919962	71.9994827	17.07198594	5.526210488	14.27047512	21.5748292	52.47135186	0.171652972
71.8893355	48.99780904	16.79138319	2.51369277	21.48784704	20.8194231	28.23026167	0.156700681
153.445811	45.06406209	12.80057677	8.127536577	22.29435055	11.98473609	73.5479024	0.196222745
125.937129	37.71228211	12.30221141	6.704966204	11.28230738	14.5572279	32.20344371	0.238956102
103.638553	93.07214313	18.27482966	12.64859284	19.51476183	12.6467673	37.30520836	0.218647377
68.812639	32.11077678	18.30846455	3.943255923	18.97220939	11.7020002	18.67576194	0.108209892
109.532796	60.21716395	17.95839114	8.973283841	22.69662317	21.61726832	51.51510467	0.358925665
174.914869	82.81988687	28.88754418	10.94009079	26.85032829	19.42844236	62.06306353	0.155621771
182.67215	95.54005217	17.8691279	14.27138089	15.74909569	29.53037109	78.94807395	0.455431748
99.2628122	50.52943587	16.13327119	4.850138455	19.56819308	17.15453811	40.9343205	0.152940179
361.897525	109.727384	20.31732086	25.09746544	17.90883538	54.93548452	134.4682166	0.544840631
91.3802078	53.15447105	14.51173698	3.571523852	13.8674461	16.45786776	52.54362474	0.198643725
137.226325	48.1745807	12.56781603	8.061086112	11.07963863	22.39298675	61.32866207	0.323942095
91.8622765	23.5338744	13.01340835	3.44163254	12.55140427	6.844200972	26.35692557	0.132011876
95.6019862	58.607931	17.17205511	3.701245379	16.40639208	19.81655233	24.56539137	0.139144447
120.758797	92.55041622	22.6007047	21.42806837	25.50927728	24.24084094	28.85521797	0.224732182
71.7215317	25.27529577	22.07040373	3.720703412	24.83418709	25.33410643	11.22746517	0.241359779
47.5547838	20.5593751	13.33799357	2.023190132	13.43665117	6.542825295	11.34421938	0.070745318
96.428198	38.38568518	15.29733936	6.201392691	15.89434077	11.84003376	24.00518329	0.161751718
99.5786746	52.70742739	19.89106342	5.79927576	18.31980246	30.26197603	37.39802086	0.127476279
150.389669	86.55268106	23.67342995	10.45764602	30.35799762	15.65400812	45.04389349	0.130420441

72.4016753	35.89294022	17.78976854	3.465978018	14.82970355	14.67512988	35.04790724	0.134310443
285.789166	67.66179207	13.23191875	14.30316636	15.64856649	20.64693038	72.80220406	0.234788052
107.41006	113.0541709	27.46273787	4.954302831	22.00476226	33.87607522	35.63352404	0.260519815
117.547046	70.15679276	17.12097846	8.038370026	20.84834508	30.15665546	40.70223063	0.246142982
111.414547	61.85006618	18.7019399	4.792595481	16.65294112	13.36775811	28.91539156	0.146419078
128.489133	63.48737913	15.10409992	14.56569596	20.94583393	13.04458417	42.22240158	0.266851693
91.6201574	89.76644706	14.69524759	4.197004216	19.78951228	20.05280041	54.11011011	0.220553514
89.2160096	38.70909948	13.84634008	7.711245471	15.33351893	21.26093646	37.80896912	0.254369184
128.687782	93.93583989	33.40323479	11.51544483	21.17577059	54.40362004	63.18940097	0.257428073
416.518779	210.0452854	28.96727025	40.67215441	18.99763506	20.12774854	56.7309575	0.145301662
79.3125353	60.21559157	23.25487663	3.386667795	21.76270799	33.74211836	23.31874612	0.185790519
95.7257837	76.01133463	16.08422232	8.781739652	19.57526038	33.95136299	36.4470978	0.260176901
79.5279261	22.60592937	11.3892435	2.170348128	7.993226404	14.76414124	48.2268582	0.335101066
232.185929	80.60558244	19.61192016	25.20970697	20.12081443	32.08696495	86.16529912	0.490975938
109.556728	62.48179687	14.88904714	7.901511953	16.01944316	16.94330286	33.46895961	0.141944401
62.7602093	41.43431139	16.18927753	1.72609554	13.44754773	19.89852841	18.40107585	0.100919086
86.5261339	37.70866366	16.00192862	3.614672952	15.2951553	13.40959346	37.14787103	0.196123512
94.7721976	49.60452547	18.95435916	7.096634879	18.8201625	9.959098857	26.08117122	0.157681787
136.622073	47.02840197	24.43699735	11.80937482	17.27796853	29.42780505	36.04976953	0.517635028
165.261948	57.82256344	19.14289846	5.05358211	13.98444541	32.71816576	45.91316837	0.319725546
140.788359	55.22792363	16.65021762	6.104611172	16.69496177	27.80678199	40.62840696	0.149457943
120.261802	38.8010631	12.39266552	9.258577332	13.95419435	11.65823867	37.01556048	0.184989218
101.942308	33.95987751	29.53139711	4.018112382	11.67783878	81.70152983	38.12807689	0.159012431
199.675874	39.01012298	13.34232747	3.698842606	13.92044717	13.34725532	73.86769913	0.171216498
48.0824937	53.17855865	19.12040823	1.107966527	18.86693785	32.93460809	22.29640117	0.155147372
131.243381	86.82880444	20.5268544	7.362551637	17.88915261	43.42862461	41.53854141	0.327527707
108.159676	72.37784164	22.30171317	6.478977229	21.72350899	42.92085738	52.18875914	0.363034552
183.154092	79.51655567	18.57025644	15.64154356	22.08795903	22.42046984	62.8514545	0.294371859
117.491601	82.21215683	18.67400638	6.269930719	21.80419251	18.61128744	42.62935582	0.162406929
76.111805	33.43946535	13.43118602	3.343329551	13.61591351	10.12458022	16.86705206	0.128218366
204.287902	190.2086505	25.82242241	24.10111003	29.26887387	80.17510966	113.5937551	0.553785011
124.78526	41.73399667	16.60587766	19.04367324	15.98034266	20.79819405	39.04740401	0.203670452
214.728604	102.1828558	19.39078641	10.57086175	19.09560648	46.4547353	87.78000501	0.582735276
94.6777049	29.50118457	11.80610223	7.531507051	15.30186136	7.969182669	37.20890791	0.178854745
54.2822435	14.19550607	16.65137894	0.700734648	18.42656613	11.08536083	26.95989047	0.160146512
68.5432256	41.17617972	16.45612602	2.535410598	13.64191192	15.03310503	24.84848775	0.138611058

156.291396	55.63822764	18.48815466	11.86507634	15.28957734	18.73001715	48.47084271	0.27510266
109.870877	39.82809033	18.49454953	3.917544675	16.37777513	12.3735287	23.36861191	0.133915281
121.538555	74.95340572	18.73976401	9.534120223	16.98553016	20.731157	55.28117363	0.175340977
122.598136	154.3959428	30.77206866	10.41617181	24.49269125	21.52616912	26.50747734	0.118950527
73.6427324	25.83442253	13.5883768	3.630678923	14.10217514	17.42090948	32.33414109	0.215079378
100.135923	36.03548049	17.16291201	6.854238933	19.0166015	8.184990553	18.60750835	0.107463773
125.719972	46.98403924	18.5697979	6.44435119	13.69096001	17.65430758	38.43054079	0.132298233
140.62933	42.14210663	14.97486839	5.125974989	14.21741198	16.52864273	25.86934073	0.149573786
386.374057	67.39803351	13.76058216	13.88025014	11.67753951	48.72439532	135.2773858	0.444305292
57.1639787	21.03300626	13.35566202	2.766856361	15.44168298	9.317994956	18.03388142	0.120866182
121.963997	69.42103189	16.81142399	9.196126248	11.39943047	19.89316576	36.88577567	0.108508713
79.9379561	22.88043772	10.00086564	1.909457173	12.76599906	8.358472769	41.93258208	0.209550975
117.636409	74.98316433	20.63185386	6.275959439	21.19424089	25.27610168	22.24421434	0.156167562
247.695041	106.3624226	17.09770919	12.97730258	17.69054425	18.32274715	48.31851443	0.21331911
122.776814	34.20547099	15.64669546	3.696687163	23.196393	17.49388127	59.82275566	0.342266719
62.418419	13.77522766	16.97570454	1.502607641	9.718309694	19.78775452	9.901275058	0.12363877
92.03868	48.5441217	11.20476593	3.670497627	15.38036503	15.2029124	41.97303789	0.187920477
142.811261	33.42066197	12.88867586	7.179774796	10.80712143	13.63039397	72.2597012	0.250255003
338.112547	161.2180799	20.76964287	62.28516108	25.27777345	23.00913013	81.74825808	0.534912874
128.223988	67.55571412	16.76474279	7.753949326	13.9984287	22.10619999	38.5784538	0.154825444
83.6768184	27.14086307	15.25289582	3.511094057	13.18986939	16.58265872	40.75134304	0.198805457
91.8091115	53.00369635	15.01908453	2.134814099	15.09612349	15.98738399	52.06990235	0.195858698
100.178408	107.1984293	24.32555476	8.920087482	22.86858856	24.604593	53.68916345	0.268352125
116.596544	72.97060909	17.74983585	6.053276918	19.73753197	23.31626283	19.58196394	0.303135505
139.599636	107.898195	17.72014724	9.9963335	20.4350042	23.68438775	80.32003341	0.182407015
134.706609	37.41738696	9.105203705	4.647852087	14.58571778	2.858209052	57.39277094	0.211278815
115.984278	52.65307527	18.26550418	6.204852991	18.36020495	12.30175366	32.60814445	0.179517919
119.526997	44.37824242	18.74565364	5.452007095	17.17591423	16.17680436	18.42718582	0.162620739
89.9981161	26.4460699	11.65126101	4.60557903	15.31814928	16.14361639	72.88123805	0.252777541
80.2606535	37.07437103	16.06224666	3.697471512	13.83905643	9.185982388	20.02589817	0.153644741
137.077772	74.81459321	20.78045026	13.65576234	18.5629072	13.94803425	37.73493787	0.095728278
134.625622	77.50145803	17.82049686	6.990452984	20.35200238	20.33477556	36.65461704	0.374939543
97.5686786	47.27053462	20.82451821	12.52004639	20.26623527	12.56183836	15.56274033	0.138285742
122.122406	131.0674235	15.34932986	5.852739775	16.32152812	46.92004019	77.43193983	0.456782358
99.0361441	30.53171856	17.51457135	7.38183289	13.48087713	17.01556544	37.42969633	0.200478693
99.1407017	96.33655164	23.92356039	9.121767552	17.71850948	79.6990319	61.88939604	0.296118756

74.9951999	35.78974153	14.32876607	3.786360774	17.39345958	17.76117606	26.16695664	0.204899276
149.039039	87.10146902	19.89572387	13.69504915	17.09719914	26.02314399	30.73817698	0.167015176
105.066992	52.47461823	20.95649566	6.515073673	17.06570754	13.26720649	20.46874668	0.137635362
71.3982403	42.71324323	14.93725691	3.910177871	15.10500542	18.98608294	15.96085804	0.153344954
109.637041	95.88607584	25.91538676	11.84948249	20.56861002	28.3840858	37.04387256	0.145967062
163.337393	133.861818	26.95482808	9.399217403	14.49847585	54.72524599	74.18212362	0.183753069
129.436629	120.9357291	18.87655646	10.02707434	22.95084217	19.1501429	31.54854369	0.3108237
129.460555	167.3951046	30.34476029	8.595839301	31.68257316	55.75329695	48.59677613	0.303889465
161.328588	96.87166301	22.31236285	13.68476308	18.53619605	21.06428535	31.18583971	0.162295557
80.8614555	25.29390582	16.12586283	5.45977038	20.75910873	15.40888751	25.6692123	0.184679927
151.488377	66.41949897	15.78965148	8.606757812	24.17858847	26.56395233	103.3217612	0.660489543
86.4649537	35.73065098	13.70538897	2.58236382	16.91986563	13.08633426	47.39494029	0.216399972
143.338067	92.58106437	23.65165144	11.37156736	24.46937297	33.01756523	58.55422868	0.260354362
124.987357	51.40549077	19.48804262	6.879573683	17.83681265	30.12432321	33.0008613	0.158022709
98.2123619	45.64422331	15.46997614	6.393777428	13.24974175	25.51110034	29.54593015	0.270185081
120.939465	69.39383755	17.67719394	6.920054938	16.46703622	36.65486201	46.69736557	0.203331268
166.148042	106.5299602	27.27865042	8.163384335	22.09587591	49.49998286	63.31849754	0.262032836
103.595412	51.01308375	17.92854716	3.474281939	21.31216503	11.78754114	22.38048624	0.170444577
61.7537428	23.07714602	12.19141385	1.755686417	15.25832235	7.065168359	14.33318089	0.10462845
38.6382716	6.004787833	13.24064832	0.192310569	7.563468015	10.34072641	10.43866684	0.054554172
101.81198	38.02940752	21.74543997	17.78513065	21.6904806	12.03515653	27.68997798	0.216703937
168.143726	156.0873757	23.66196365	24.73669089	22.1275764	39.48094274	97.44894837	0.210692612
117.217228	27.10484623	16.24902578	3.972785172	9.153847254	27.08329849	20.7553584	0.214426815
114.730422	84.79365563	18.71432292	13.08161677	16.47037184	26.60776575	26.2122432	0.360781839
281.754614	50.9663134	19.00979557	6.46047995	13.14972266	8.453257176	19.13582586	0.077594326
50.3664131	31.38448704	14.45328496	0.928581902	17.26660253	20.43467735	12.99074453	0.10275598
218.768278	84.30713202	20.69457243	15.96373161	21.9969335	23.05718788	41.84075306	0.304632334
90.9325363	33.91063407	11.12764216	5.461674489	15.58235744	9.89313812	29.88843574	0.148658551
77.1894988	19.21427231	15.23304266	2.4634995	12.88274009	10.44422721	15.18788415	0.11362916
68.646716	37.92244573	20.70768637	1.992955371	17.28972955	8.118237065	13.93688464	0.083131918
192.656055	86.19757103	35.57214619	20.20466651	24.79250711	17.81455643	33.59513721	0.196214413
153.00605	75.60405303	19.39646819	8.802510284	13.90726803	35.85668907	54.97696051	0.29852059
90.4676439	81.60149731	18.4606331	5.829030555	28.36613251	28.92256702	53.8007632	0.309007057
174.870834	177.2586582	21.70615663	16.54268402	13.65744888	52.45124017	59.05395822	0.183260552
180.10446	65.6515267	12.77471454	9.85766652	12.43531479	26.11936847	79.53082234	0.192847781
66.742989	16.46375213	8.994510712	5.117916866	15.31553631	11.58014921	48.88457815	0.154015395

131.055538	93.73529839	22.08580419	8.59349546	20.26582758	34.54319209	55.33600331	0.226481775
86.1307925	24.27294436	16.26054841	4.732567176	15.94744801	11.33390272	18.9405009	0.095712652
175.58009	94.68026907	21.45995625	13.37952902	16.41378187	13.97109857	38.36708193	0.132110114
138.063792	122.6191098	18.0624434	9.249233283	21.20050816	31.01897025	85.03044829	0.59818343
159.272449	69.00195155	17.27664908	9.443308096	15.88489452	25.07751772	38.88641905	0.25278725
126.739156	120.940691	26.68605052	11.12128437	21.89959689	56.7355681	42.46640828	0.20077013
119.178765	72.31924143	20.32648544	15.38410922	19.37465303	35.73513795	50.09934566	0.311612555
106.894671	89.10099683	21.54280021	4.853676152	20.00991842	37.406407	39.17653381	0.251909073
122.635722	50.54982723	17.39528058	12.40901054	18.63897002	11.69887814	18.18164837	0.127610943
178.166294	85.39673789	19.67393232	15.62986164	20.19320724	50.83929498	68.73862641	0.636396237
101.974353	55.04144249	16.51055907	6.333960089	16.06851936	12.70190793	25.96065157	0.14232187
121.520704	71.99380874	17.08300106	7.035401489	13.88334292	21.6776247	65.02877148	0.259959056
145.181181	51.70297383	15.91303587	8.159112412	18.65259189	23.64904116	40.58490554	0.181171372
128.048336	126.603291	23.21367115	13.29689197	19.78987912	47.20541203	68.20876453	0.448244368
159.166746	35.8935862	11.27209218	10.89800252	12.37692848	20.18240837	106.4529708	0.497730362
154.019366	99.65507262	24.05198626	10.71828144	25.62119911	22.1652836	62.23963405	0.185104828
91.6877431	81.55510601	16.78298433	5.075978358	20.71680851	22.65027429	22.25964365	0.160360427
88.7491313	41.10878166	14.4782156	3.852239945	13.71787203	15.12580006	27.27119238	0.159065836
128.234824	61.77298698	19.04116393	7.165107214	15.4581098	29.16024768	28.50185094	0.264864275
136.320582	55.44198703	17.95207261	10.54749533	12.16657993	22.37981803	41.63157941	0.317008691
74.3996402	34.27694608	12.85993322	3.65870874	14.97198402	12.44359012	30.69054093	0.139316519
119.548699	48.23296946	12.83863382	4.141761544	11.29515471	15.0069585	30.95449733	0.231860782
116.878451	43.16988773	13.28989881	3.923875676	13.35843676	15.89860553	90.14420668	0.212121747
90.3426453	66.80576635	16.99899072	2.15128015	15.30879244	29.35043625	37.79466872	0.21343695
101.270349	50.90682104	14.86783246	3.883184408	15.14924896	18.286462	48.04574476	0.227132085
161.419701	113.6829393	21.46869555	11.02921136	19.1002668	27.47898663	36.25308669	0.208598302
55.133651	22.70438748	13.38877091	1.656903899	12.53110672	8.167772005	13.3939564	0.094769106
54.8169762	33.77488695	14.99859372	2.580007711	16.26396105	8.990211934	13.91127884	0.106157535
95.6944254	14.76027701	12.29699624	5.437363736	13.05644576	16.5617051	71.64648541	0.168813127
119.312871	83.16130089	19.20875148	7.354702746	22.46530133	30.89697914	34.06052998	0.276468477
108.726053	66.96282971	22.72630282	20.47038791	20.13446377	37.08221337	57.46473448	0.302674183
56.9636855	19.07969568	15.61159696	1.076473549	16.89385631	10.5926425	24.1556364	0.113968891
84.304672	41.27985423	15.97694478	4.800637945	14.71337987	13.4833392	23.04102001	0.12792671
186.28244	87.05220882	20.98030537	18.81387964	20.77095935	54.56889946	120.3891598	0.418334585
83.1050577	56.70618389	16.67624178	2.601079604	15.9647977	18.65717414	36.3698931	0.181346213
150.419547	77.41180382	16.44657765	10.68174621	15.07420956	35.24900628	86.95623587	0.462276284

100.558959	52.58563325	13.71440059	3.708019559	17.40056777	21.37070877	42.34135817	0.135191516
92.6766598	46.1474216	21.73711548	5.969810579	20.47197182	17.79827168	24.17265673	0.303208606
93.8208555	49.61863438	14.45127652	3.696302244	15.35127829	14.53850385	34.82120934	0.204883247
97.8787766	45.15586556	19.47765677	2.380011558	20.17501152	12.07679011	16.93111452	0.213564665
74.6880256	42.87057303	13.50457038	3.159464146	13.72605272	20.70631069	26.14809893	0.195917214
153.986422	104.8694386	22.64682418	18.77872972	17.6363603	20.74406077	31.60697192	0.148224159
71.4133315	57.91938724	30.47347029	3.864549172	28.90774799	22.10928001	21.59810381	0.218507079
103.592856	27.94929859	17.77828737	7.863866617	17.9572654	11.33414381	20.13031222	0.111662657
238.16157	61.58342277	20.03064556	15.66533068	11.56133088	21.96682855	36.04149733	0.356773952
75.7948898	31.25409316	18.75670448	3.961834337	15.29933153	9.928860261	16.67290725	0.098621987
125.579557	70.32075292	24.12204712	17.51260436	20.48691705	23.80566381	36.2486284	0.32576245
226.997932	127.2939594	30.09278355	9.00418904	23.58980348	32.57648166	37.70577585	0.163077126
120.399132	92.9343684	34.806801	22.94800411	24.79182596	32.62649671	26.10466545	0.155878795
74.2232908	33.87115872	19.76344481	5.891467704	21.03502985	28.36088765	31.43651274	0.184153494
55.6560155	117.9941569	34.97452394	2.143959035	21.82414484	20.90016839	9.584130226	0.058329067
98.5848194	29.03278147	24.65203238	2.944968585	15.47011406	25.7467366	23.90749189	0.170288697
93.7685434	47.64275183	18.67280012	6.481747826	15.90126321	18.89802692	37.66682418	0.138528321
147.552083	64.35344928	15.65078333	11.81694716	15.3987474	29.99677195	55.36921302	0.258333029
109.762523	38.00201445	21.30250501	4.08122601	14.37438412	22.03928518	10.93000457	0.063783608
112.569744	137.7802824	35.64712035	22.47967143	24.64332397	29.03700909	58.66625233	0.237592021
62.5601767	32.09103422	16.96611857	2.684482405	16.54555362	15.37115278	21.69868214	0.115524637
57.5103663	42.12741668	15.66677736	1.328149325	14.17170878	9.956719968	26.75294743	0.116185222
103.297765	65.15534124	17.36405216	6.15535222	18.46187417	13.69886571	47.31731684	0.165379153
82.2136949	24.50976649	16.80463589	4.154472217	13.97484627	10.70864848	21.19417085	0.117023308
102.674358	65.32191217	25.16391115	14.14658813	22.0802061	22.90395389	51.50611029	0.207539769
99.6251178	58.28625616	17.00170763	4.922729345	14.35628065	29.98898104	36.34582595	0.140353222
231.118632	58.89838068	41.24711427	17.87664076	21.20015761	105.519186	70.24094751	0.342061277
79.9651207	66.60268796	18.68770529	2.684867589	19.31601834	28.27291272	37.99097473	0.161124632
103.802449	95.29223105	24.7349877	5.590267567	21.10609762	20.34379492	22.20651509	0.119611268
138.714173	73.89914839	29.01672992	4.416682108	13.07621265	41.19834801	64.79406797	0.26577564
126.96131	76.33455102	19.17380192	7.743455893	15.81532028	25.87211251	96.24895281	0.333439441
145.992452	29.29964776	25.1853056	9.116832057	17.07431016	28.9319667	19.2495371	0.15519263
93.0378815	31.74582574	16.95251105	3.650651283	14.53024475	7.188432077	29.51308394	0.080680158
70.098644	54.07678606	22.60238566	7.563676689	27.8321131	13.80363874	23.30005764	0.124708173
135.232731	152.0355865	35.83573818	18.11031421	16.12037744	37.7334163	29.71269154	0.169829186
96.3891444	75.74411642	18.41961772	4.245559604	17.48339553	19.60552339	17.37882397	0.121376525

106.759747	43.85492782	19.81899562	8.055376029	16.1684955	20.7738974	50.54864957	0.151213815
48.8726826	26.24605663	17.07869639	1.614448338	16.31845984	6.440248975	12.3708462	0.074375111
116.407919	54.93597997	22.11174348	13.70286803	19.28901174	12.88418064	47.79664827	0.157338771
97.6961017	32.38309072	19.54646624	10.40833746	15.95590461	19.39590067	21.81225014	0.290719938
99.8238509	40.46776284	15.84695797	6.602228757	11.34686574	12.46498096	28.28841431	0.130290434
234.21756	79.73993317	18.74201139	18.85374325	17.47597397	28.00118621	60.23681101	0.319143738
85.5637162	48.36348139	17.55636502	4.865760184	17.11058982	17.96910584	32.88798267	0.175270225
86.8426489	55.42434039	23.05725332	5.166491773	20.23378076	36.85042814	35.85151157	0.357216084
75.0048031	47.70136339	15.4529903	5.439389107	20.17106296	9.95206601	86.4691139	0.262117974
222.81315	67.33190518	18.86872066	19.66388554	20.36228503	13.40164416	38.52359771	0.129304089
71.275044	59.33571178	25.01252669	4.472982631	18.65307205	26.97138848	53.74129596	0.191265026
61.3560597	67.48328561	24.04615518	1.027432124	23.74184198	24.42208282	21.23366582	0.108228249
105.427668	65.54989356	24.82574739	5.652848774	14.25462219	62.7819081	111.5479001	0.410951588
91.9791912	50.986654	22.50263434	7.911729463	19.28710351	24.19508699	36.57038356	0.206973186
113.076316	67.82715733	26.45458347	30.06438557	21.81506884	29.40873942	50.41352256	0.255305725
71.0593971	55.1020539	21.19066541	3.801350272	17.17482563	23.82660839	11.42419394	0.075497363
71.5278854	53.38570855	23.90221207	8.111738399	18.99163507	23.52818014	48.23702383	0.180446775
147.774383	150.1859681	30.54253694	6.198351221	24.62704136	27.20863711	34.64384312	0.166977273
91.3657295	38.93986549	20.97973882	8.013714986	17.88388023	17.81254973	36.86689112	0.185330787
86.7372283	17.9628361	11.04411612	1.373076988	9.335620285	18.63166555	28.1335096	0.206175296
62.952886	40.62899194	18.52674935	2.11880865	15.0773618	28.22452122	25.35157304	0.213221271
76.2258297	53.5965435	16.95489354	4.24883988	18.50895973	10.37677494	28.65122768	0.132821281
71.9944541	153.4406753	32.81331455	2.928521093	27.96929747	22.30823063	24.27795483	0.123813696
96.8834623	28.51594353	17.02484354	9.779684711	17.31806759	14.00375945	34.49525366	0.154464619
104.400539	40.3071257	21.35482134	5.375184454	15.51955743	23.41347119	17.98897562	0.164279908
124.850245	51.57917713	16.25585417	7.758181781	12.98712898	18.63138463	61.10682438	0.211206156
201.677338	86.29617439	26.66071853	18.0582946	16.80446853	35.74470435	65.47995891	0.199195469
59.4281937	20.6363576	16.64768013	2.527427785	16.61074147	9.294579814	11.94162661	0.089908814
43.4304155	20.93824349	13.97322648	2.125920437	15.44958488	8.175286167	8.115242064	0.083191961
79.569864	26.73180662	12.64625591	3.719681587	14.68748664	20.36782718	72.39816137	0.295902146
120.122749	103.2475176	27.00034699	14.27566108	21.86829478	17.23615916	22.06518184	0.090613244
94.3592662	32.25568472	22.41427201	4.652704635	20.32774762	18.72899743	20.44182	0.068360766
44.8940718	23.02367027	13.817121	0.978655948	14.83274822	11.88463146	13.93555736	0.089009131
210.717174	153.4558146	38.68461168	47.59673102	29.63846556	52.90018705	91.87394828	0.639754414
76.9788345	42.96600745	23.58249664	5.247475921	23.09937245	16.410669	16.30039404	0.136425708
65.493891	27.3286993	18.21858729	1.767483288	15.83704801	12.98880183	23.73098091	0.113791027

83.8618267	56.09945335	27.64776841	9.909048872	21.83068956	22.45581367	23.22293629	0.109661243
63.9721814	34.06149319	38.23567701	1.960276219	13.45877413	108.3874386	25.63458075	0.16931577
158.525019	48.23574155	22.65139153	8.312459422	19.44637749	15.94551707	20.51855989	0.12639458
111.742615	52.07605019	25.33505791	14.31572339	18.40560742	14.69905336	38.20078245	0.123018589
110.593259	53.78059419	19.29894019	5.857595779	19.96731166	10.4325365	18.68142132	0.083541649
132.518767	47.46714968	16.17265379	7.093842535	13.8104334	15.63143392	36.65829346	0.126159841
78.7746058	67.89848236	30.901528	4.143586066	14.2384214	39.52553906	24.07893989	0.1681427
90.8314476	27.42461917	12.72218918	3.307698998	10.39668685	10.97638707	24.96144587	0.135963606
131.107718	65.8949246	19.6570571	8.990894896	16.65547623	9.542599682	28.84475998	0.090910113
150.638251	70.10736972	28.15642963	12.88243249	27.54093695	23.8488065	37.72505921	0.196762985
102.776789	62.58126127	20.84367617	8.32737789	13.87377228	24.10446438	22.85871392	0.202382709
98.2141148	30.19206891	17.85001152	3.530952027	13.48652227	28.84579367	58.37789591	0.232465197
64.6500471	40.73325601	25.69202584	4.063248246	14.05239026	112.5917695	19.46223517	0.300169259
86.0647025	71.90109965	18.26385279	3.063601652	15.6550343	25.91879062	52.51363209	0.193642998
81.452747	21.85819148	18.80566488	2.857125419	11.13422769	9.51717614	26.26399208	0.125908157
87.3539735	27.76385719	13.55608175	1.966649277	12.54621424	7.478591945	21.25011381	0.085681952
93.1463589	48.63871855	33.18646542	10.82022034	24.70243069	8.735848537	14.78740493	0.072313515
73.2362123	110.2772658	29.18307062	4.181517017	18.07568841	64.33090324	35.36777461	0.197288489
122.213481	58.97218576	20.19598565	6.532623387	17.23244788	24.84864767	46.67797505	0.236352663
78.0407433	41.8392045	24.11607962	4.323354221	18.14432528	11.73813079	14.87154633	0.11356701
134.284323	59.92166489	26.46285413	10.70241822	29.0936191	18.70301734	41.41606118	0.212671052
85.6808669	61.20725819	21.23310248	8.059558372	20.01019595	15.3373155	18.97909966	0.07456273
85.3677093	52.73474085	14.78214738	5.169007762	15.12195978	24.64278464	32.80011222	0.198640454
85.0529428	87.57034653	26.9929044	5.624579747	21.88448451	28.60423272	17.87574767	0.114087277
74.6736085	131.9233498	24.95355958	4.325941718	19.53786545	26.1485597	57.15604289	0.103301065
88.4780408	64.08849178	17.63071057	7.739272664	14.48114973	16.45585416	28.67629989	0.132914819
141.34339	69.13209417	19.63376419	6.172867764	18.53973347	25.1788409	34.68828057	0.183095901
220.662865	208.9833121	32.89480094	12.63015078	25.13670852	15.95339426	20.46030995	0.096484879
106.373536	43.1290464	20.71989913	13.89959971	18.20219431	15.4478898	37.57258404	0.111196823
152.994948	89.25029946	24.94907425	8.640307237	18.43544318	24.69733843	37.09828882	0.196464689
102.099912	107.853968	21.79460785	3.557620298	22.8600035	20.51257409	45.43664049	0.111079877
92.7386159	56.67139856	25.57405487	3.781189196	15.74360033	19.69184771	31.90687741	0.117157864
70.1878307	17.68806808	12.86672675	3.467986287	11.25214753	17.78473963	25.66282495	0.12539417
107.963203	63.21977961	20.28423838	7.654671593	20.98451025	22.1501746	55.46365759	0.17887138

Pictilisib_1058	AZD8055_1059	PD0325901_1060	Obatoclax Mesylate_1068	5-Fluorouracil_1073	Dasatinib_1079	Paclitaxel_1080
4.165388379	1.061433637	2.808825476	8.124250938	140.3027524	33.12694759	0.093325022
4.000239977	0.685043402	2.662017251	3.055724188	270.9947101	1.848064422	0.082881634
7.467430436	0.790981731	5.652917797	5.650958213	200.0999455	15.86239848	0.102014203
6.647967235	0.630129642	0.971609956	2.504280101	355.7997843	3.553033779	0.08789967
12.37953041	1.215519308	3.214273961	5.279124511	371.8168179	7.349863763	0.302167829
4.106140698	0.700896401	1.380532792	2.634165719	79.71313192	1.114246922	0.098134369
4.808685798	0.740162906	0.333914449	2.974348788	53.49518494	5.02366925	0.087618934
7.636858494	0.619117767	3.041707462	5.492123348	178.3681158	6.048903439	0.16433788
10.33067486	0.907449195	3.509577927	5.843066748	483.7065121	11.64557848	0.466143456
3.470585914	0.59964069	1.38544112	3.506346397	71.18948573	2.548071409	0.039070673
4.731494637	0.808196047	1.752631295	3.953263128	84.52734126	7.101307621	0.106610827
5.243955179	0.701945729	2.581597202	5.640754925	225.8995646	8.829104437	0.072595578
2.309018206	0.817400296	1.830271615	6.490959849	164.9468726	5.344406698	0.01410316
22.68561105	0.776243306	3.808484093	6.073019985	1066.357951	33.0832865	0.463757373
4.744604683	0.731508294	1.458958901	3.48333796	109.4750764	3.663627807	0.069192257
3.181190879	0.737213495	2.442565616	4.162317962	201.1014553	1.553958278	0.248889645
5.224031804	1.131141269	1.966826204	2.898394162	54.95997821	10.06731083	0.009179632
10.0664154	0.944520536	0.519336226	3.898644774	44.64812431	7.15419118	0.077545539
6.540766917	0.822688626	1.325956048	2.839617412	68.55890184	1.078503791	0.086296254
3.852333966	0.71345597	1.263825447	2.160762485	36.33094234	0.482198429	0.019339406
5.358590455	0.599962843	1.720353621	4.205589225	81.16562945	7.897216605	0.075742011
9.153138424	1.829012435	3.305087735	8.436590763	159.2035787	32.91051982	0.103732974
2.797630323	0.759202536	0.764014157	4.008265451	84.25316115	5.767836309	0.05118124
4.878524166	0.822916965	1.82474854	4.858621466	104.9013481	6.303844511	0.149117765
4.223798848	0.796408663	1.806523941	3.70060733	261.7548514	2.556350145	0.093139692
3.85184548	0.492644132	3.067476849	4.737244837	181.4839986	3.270934354	0.058739744
11.54024012	1.119897728	3.503621882	7.543499443	299.5546591	12.93701912	0.327818236
6.708564212	0.718459574	1.200945931	3.884854358	210.101395	3.801185641	0.148895931
4.73160303	0.538645634	1.078532452	2.390313817	115.138826	2.032590257	0.055234026
4.238068791	0.676955092	4.7077524	6.20639338	52.09853525	6.049298427	0.022905195
2.758174459	0.664512611	1.143641111	5.974682485	124.49166	11.01564093	0.038510891
2.177152721	0.5578899	1.969689405	6.153994731	57.46181342	3.0152817	0.018486275
5.292901283	0.898540715	2.828492878	6.542693241	398.3893799	5.999536178	0.115086209
5.090211406	0.52183888	3.667576169	3.892397982	114.50767	5.942972315	0.042249592

6.032174188	0.748252364	1.948156218	6.359067755	317.291889	14.29218889	0.077390114
4.329259688	0.801011272	2.349758317	6.154480675	194.5003757	3.030740145	0.121383352
2.485710279	0.727427289	1.167662687	1.941317416	36.56485232	0.152708505	0.017159736
4.839577883	0.606535501	4.245889493	6.239292208	370.3908417	9.922973236	0.092452464
3.840981612	0.747882896	1.535536366	5.857807095	75.4421913	5.386787848	0.102132507
4.076224883	1.247122719	2.62298859	5.017649327	140.8693863	5.001518459	0.025546569
3.25033269	0.384559019	5.849560688	3.678786905	288.552016	2.5795733	0.024568998
4.93589452	0.467818909	1.425160711	3.201459265	163.2026599	3.010778139	0.163338102
2.814231295	0.854001381	1.610505587	4.102213616	120.8961877	8.013352516	0.081875925
3.091794419	0.700106884	2.447104347	5.48615221	257.310118	5.849021331	0.11179058
2.958666104	0.794798626	0.933114937	4.434480023	48.56257896	10.87458535	0.077132912
4.212030573	1.169354015	11.09727512	2.41241378	139.9430854	1.988414576	0.005483212
3.376673197	0.613688112	1.063866412	2.455811837	77.1517945	2.422714401	0.060491263
5.600818569	0.530623114	1.867995052	4.046314423	94.67976179	8.212442723	0.067472199
4.719371615	0.700908908	0.936931957	2.545776258	92.48934648	7.651518519	0.094021454
2.852921282	0.707903801	1.926667157	4.557937468	55.7159858	2.990416573	0.027569433
2.771838051	0.524260731	0.773683968	1.993955876	34.53168467	2.238044051	0.025114288
4.411200291	0.613641193	0.999800387	3.150189386	104.9491676	1.479061762	0.065869318
4.150078123	0.654157128	1.701545031	4.639493954	88.45712004	2.910676851	0.067642982
4.380545268	0.778170615	0.891530254	4.043606312	58.45587973	1.639610344	0.019597047
6.519730073	0.308075491	1.68220203	2.349943357	96.36378105	3.143186036	0.041974791
8.874099023	0.494041195	1.599616739	3.422461132	69.51180192	2.689761917	0.101733285
3.413263083	0.695554558	1.707977748	5.660165479	58.83051813	4.030833302	0.013812986
2.651495262	0.639576837	1.555685117	2.552307263	51.87226338	0.639055172	0.037131814
4.606703314	0.924616743	4.185583879	5.829963298	235.7115739	30.34581803	0.220233545
3.237904475	0.869411146	2.211011926	5.205031331	133.8863144	2.712980542	0.0341922
8.980000729	0.87691302	6.501274364	9.070997359	1218.714027	9.593952673	0.40171839
4.658349307	0.949280724	1.864855089	5.933037725	309.1434836	7.516709112	0.095283282
8.138284155	0.808018593	5.052699152	7.251971286	601.5785943	8.640687311	0.14229121
5.156064381	0.644675871	2.668387039	3.559780733	79.39211721	10.50160151	0.032103381
2.813227701	0.592116154	0.875449187	2.498569282	66.96129508	1.486995274	0.092780282
2.670343443	0.55425141	1.172123056	2.741925162	67.24689051	1.568938199	0.031301488
4.089747878	1.946215953	0.643186396	2.869398741	106.0915772	0.929427265	0.050213984
15.56430798	0.754365234	7.973375182	7.581085571	701.224033	9.789424197	2.253968509
5.148846265	0.589922237	1.080972334	6.17721646	60.38103412	1.695770966	0.044608345
1.898919159	0.918448259	3.070485152	8.802490215	47.45470337	2.726185191	0.012183308

3.939557325	0.739231442	1.470099194	4.321207914	68.31653949	1.910291761	0.028266364
3.043292031	0.611155295	2.963818638	4.213004951	93.51556531	6.969897334	0.021253003
3.510746867	0.640531489	1.005973469	4.65954986	204.3540522	2.191800487	0.089481609
24.61953906	1.673580097	4.096693958	5.382161474	1475.953829	27.07356472	0.740245816
5.24080452	0.653540587	1.237009974	3.192458231	99.30518127	2.603574317	0.053715951
3.116729512	0.550133871	1.032496517	2.125668631	114.1661274	3.021264838	0.07544231
3.309485288	0.963780801	2.035696511	3.333212098	34.12948028	6.223318916	0.024319919
2.806977989	0.769882734	1.399833634	5.066370572	127.1806668	11.41338399	0.089208336
3.373216953	1.129495069	7.562236337	7.201351654	277.5390519	17.75477609	0.034512985
4.332572547	0.840826787	3.909934839	6.025429434	221.1177384	7.475351349	0.056831609
11.88003532	0.445337395	2.346423795	2.431889808	124.2350467	2.309494958	0.182820264
7.010169205	0.804694611	2.145058869	6.517375209	687.3284925	4.104895192	0.227707403
2.285941306	0.467627211	0.605321771	2.782442388	69.33567346	2.040251484	0.019591354
2.62697685	0.346711017	1.087560853	1.670846843	82.33593421	0.817409367	0.051857508
6.042704384	0.869493191	3.118377899	5.173263375	127.6690768	11.46232383	0.231492538
3.570354939	0.804806634	3.234425794	6.050303698	114.3713999	3.105271461	0.113505005
6.935414815	0.88597803	2.408163562	5.661115896	114.8897393	14.79327724	0.02295349
3.762362803	0.756225814	1.609895776	4.933081793	105.0609396	5.551385774	0.069906308
9.589004125	0.842894577	2.887711361	5.847438707	230.0901565	6.418031645	0.19056403
3.640202577	0.802568136	0.811235966	2.638550588	30.71007204	0.356438562	0.020581679
5.855891854	0.845181651	2.429056236	5.067852976	111.0406809	19.79510108	0.107804317
2.935848522	0.697645712	0.466670282	4.172695227	31.42744591	10.60112784	0.033092191
5.619392003	0.614448452	0.784479121	4.027648131	104.9659212	12.46341287	0.097689973
2.917369015	0.738641398	1.036541046	4.634385392	124.3946817	1.725794816	0.096619761
2.944817863	0.831445382	1.166532974	5.667397104	90.74903814	4.198238732	0.031706553
4.185902629	0.279090405	1.244398823	2.617039343	74.3174734	2.667703959	0.094802834
2.954871876	0.737235223	1.355752514	4.004942647	95.86887891	8.152205723	0.046204808
4.379794736	0.5930695	0.501993898	1.740996944	51.19285749	0.81604576	0.061884358
3.823635545	0.657616543	2.026554448	3.725831754	499.1811646	5.162736851	0.137000337
10.81098899	0.624246413	2.375967783	4.0167188	395.4384108	11.5528599	0.118420765
4.944643044	1.026534151	5.084928591	7.604229205	193.8234336	5.774905734	0.073315322
3.657981907	0.690515463	1.872182068	5.432968884	192.7737046	2.12554528	0.12673752
1.648732341	1.158099157	5.583580971	2.45111556	42.71100741	1.311345802	0.006886893
2.715614744	0.631907817	1.339125148	4.021903029	82.31442646	2.842995884	0.08492468
4.988844616	1.543784257	4.160475148	2.145373713	62.78097487	1.248392109	0.027433855
4.654819126	0.665394807	1.036430467	4.566135167	91.81106271	3.555061227	0.08836684

3.18095577	0.709230442	2.496637566	4.281474016	234.3525962	7.392229681	0.103655303
4.295148017	0.690752296	1.744397968	4.48602005	219.5583281	2.201421913	0.143472408
3.884701172	0.616628542	1.065199597	4.722819123	28.79314661	8.95834571	0.02832103
7.2568531	1.218319194	2.748300813	9.591602384	387.3455759	17.13798019	0.227993769
3.538394451	0.848730823	0.994841961	3.052917203	333.4850162	2.322313337	0.230883989
3.518430154	0.829535344	0.510168842	3.261874259	56.63811676	0.834136278	0.070947566
9.764899531	0.814422965	2.809425762	5.823933834	748.564123	1.641699103	0.583175311
2.95973112	0.834129556	1.462318814	5.356346124	125.6724818	3.737310189	0.044793537
8.40529802	0.630771865	2.245306697	4.965357565	372.968283	4.743311389	0.240956045
5.027706392	0.83066732	1.485543501	3.383503952	182.7796782	4.149192913	0.057216693
7.265065982	0.8527574	1.169210383	3.743086522	254.9139736	21.67516272	0.113215324
10.2771885	0.444043225	2.986831134	5.704005312	179.9814595	12.55310535	0.223816197
6.138060931	0.630992755	1.021787579	3.561178732	185.5952827	2.710466031	0.17233927
4.026694138	0.656455766	1.56539419	3.060486792	39.86336133	2.83639738	0.062750154
3.565843715	1.48155296	4.995263479	2.338381239	119.93839	4.339584861	0.022473139
6.551573542	1.210538873	0.283500399	4.648339941	82.59280077	8.607688052	0.084458579
1.901837381	0.958245006	1.362854373	4.276664843	124.0767544	0.520520284	0.053003401
2.532804352	0.760948305	1.323799028	3.635582806	59.817122	3.169386589	0.029843899
6.570919983	0.880937502	1.676618872	3.579339276	88.20741838	4.900551608	0.019150759
2.184803617	0.823503276	0.954226445	7.779501701	203.6378178	15.37979957	0.093110477
7.71647646	0.800522363	3.647421775	6.43855556	219.714512	14.33892142	0.163326617
2.837181985	0.547925971	1.098859243	4.68087409	69.73835056	4.947971742	0.044803918
2.008660716	0.825237383	1.163067916	4.123923861	84.31060431	6.343437369	0.033235307
4.260118161	0.99729868	0.674481648	4.152305372	25.3985133	0.627948021	0.026390773
4.409772313	0.84473139	1.9677064	3.756242914	472.3537924	4.78615637	0.068622134
3.351939226	0.610853363	1.090069836	2.406125637	67.44547153	0.161345966	0.041565397
16.71997008	1.313089278	1.773148272	5.92005015	846.7981457	24.59007976	1.174263307
3.641922822	0.333415633	1.030260579	1.957862028	82.8363419	1.105010363	0.0683048
4.485112859	0.602736882	1.244165753	3.394755255	170.9407935	7.343553216	0.119109547
12.85692321	1.068854072	2.340684939	3.277480541	831.9827828	9.893660698	0.461644232
5.651401663	0.751573478	3.446925969	5.722341424	139.3535039	5.81922416	0.057143735
4.125230428	0.989886956	1.764971745	7.127448948	120.4544345	4.460031075	0.118781441
7.219235259	0.922680339	2.039911551	2.602047465	271.3144604	6.825262546	0.122898968
5.115944827	1.132865492	0.631852988	3.100846997	97.90620108	3.47025602	0.119394183
3.545522894	0.594226438	1.256785161	4.908114801	103.7408943	1.912260579	0.094164793
6.876355958	0.994613823	1.93466922	5.360882791	362.5428441	5.028810324	0.278807542

4.175630143	0.855930769	0.489207131	4.359962906	160.204793	3.357767501	0.098398297
5.217322711	0.772023221	2.116775921	4.26983344	473.6372513	3.901360912	0.123746662
2.888749379	0.971716775	5.402724715	6.247590689	338.3462839	2.794236859	0.084548072
3.741242244	0.650286846	1.257061998	3.681125837	172.6815286	4.957194932	0.187962161
7.162408038	2.21136138	2.108344972	11.36237962	171.0114982	5.352081917	0.092305454
3.356820941	0.503810335	5.327386211	3.717043043	45.03750514	5.333564193	0.034890327
7.220415166	0.789280078	4.583413979	5.029388744	176.9704792	12.7805277	0.142326513
4.385013224	0.465094547	0.92575668	2.006596561	72.51918797	1.857973284	0.067004054
3.628273825	0.85134611	0.215179835	3.347552458	63.81822729	4.59287527	0.090207343
3.935977282	0.857423911	3.663342485	5.659250287	110.0377479	7.286595374	0.039152408
10.52065248	1.033367532	3.167590623	12.07521629	347.8309279	15.7419074	0.413286052
2.922638246	0.927628426	0.517489537	5.288832279	65.70203762	11.69588937	0.058082827
3.089881315	0.802480828	1.263586528	6.077305371	111.5471735	2.903186337	0.049285835
11.67101516	1.423577338	3.171820524	4.202065309	380.5204607	28.15435455	0.213326239
3.422146128	0.762349027	0.638226431	2.532706736	73.5473198	2.635363885	0.060757664
5.706415959	1.295460989	4.943198757	7.131994087	361.2635387	25.67316548	0.031695411
1.960062791	1.615878466	0.597556036	1.462991207	18.65626095	2.734556035	0.002583736
6.801300386	0.719040061	5.005240612	8.802780079	299.9808605	10.65168579	0.091554552
4.625675354	0.644420716	2.323429252	3.985405629	181.7696652	3.313592594	0.13220352
2.007997379	0.527530333	1.359419756	3.381692113	68.72967331	5.012153902	0.034815106
3.08295364	0.741109097	6.229437584	5.320604917	141.7278136	2.752333455	0.01959652
2.279529337	0.768618178	1.546393673	4.741618134	51.43942259	3.352219102	0.048567338
3.395360741	0.653173414	1.471871713	4.307107255	97.11640309	7.157540856	0.067868182
5.180458897	0.491687565	1.011219497	3.442289626	163.4057042	9.186040196	0.132184863
4.018635929	0.840535454	5.293108658	5.882993991	259.6980199	8.993925659	0.082425002
10.54737483	0.76755024	2.937475384	5.864383256	275.6324227	13.56247307	0.190728146
3.34360038	0.672869517	3.21177329	5.78754566	102.5907779	2.02130985	0.026285254
3.235890394	1.017152758	1.021918185	4.596557877	72.59383597	0.669484761	0.059517207
2.515370914	0.735412399	1.804305392	2.983077588	24.31396534	1.637757689	0.033849622
1.815133703	0.5528684	0.885112129	2.606106086	22.63111098	1.507814958	0.007141273
2.644917591	0.747013117	0.868655895	3.704554091	69.1214641	5.426944468	0.051517394
1.617577327	1.076548608	2.129476873	5.686340701	40.79701229	17.62399412	0.016864267
2.095679081	0.607784629	0.437173471	2.364294897	107.1460715	3.061062944	0.08292298
3.69308872	1.260205279	7.372553448	9.368322592	606.1615313	6.816953404	0.07616498
3.611154329	0.729667097	1.142503959	3.064156854	101.8065841	0.664762968	0.038968856
2.896866809	0.819951734	1.846409522	4.299633459	195.3215643	2.345930632	0.027554099

1.921991543	0.636855663	1.760930075	3.604980893	68.85381486	4.055964427	0.044180892
7.952144955	1.292741147	3.373170219	4.992693768	110.3461231	23.24660469	0.019963368
5.141713621	0.402454623	1.209334099	3.366996055	119.7996309	3.567070191	0.137233751
7.891255688	0.413729493	2.067193849	3.510999955	96.23415431	3.479063655	0.093607905
3.352166489	0.6472845	1.510996982	3.926442717	134.5179333	6.127943452	0.086381023
15.95892362	0.512389955	5.767546234	5.910172868	419.5186738	9.956298212	0.530379984
4.025049222	0.811966684	4.766694375	6.496110151	330.8984164	16.63060895	0.249207801
4.893667806	1.158349	3.731567364	5.024520993	177.9567987	6.088064713	0.035807885
3.664460492	0.642464231	0.960949686	4.403455321	109.3140527	6.638887183	0.060313936
3.840993864	0.678099675	1.415185551	5.258910506	98.2556611	13.15131711	0.088339339
2.149207882	0.809708035	0.866573865	3.604060104	53.57751201	4.680910261	0.028516835
3.910568838	0.608348925	2.936174293	5.423400713	201.7754983	2.163037661	0.105838145
3.545689467	1.234297564	6.79980241	5.921502064	300.9799147	1.623032897	0.095047315
5.334320019	0.773114517	1.424373258	3.443732101	186.5033744	1.900976536	0.135027298
4.833250608	0.718948994	2.959413991	4.77828903	178.8930312	10.70837791	0.020882886
4.181270679	0.412335551	1.437964501	1.939781044	139.1924239	1.180441623	0.057491668
2.818356997	0.607777756	1.597680687	4.228733939	165.3292236	3.662622383	0.035615783
7.002184138	1.210105676	1.377013911	8.47568637	168.3902908	17.59322795	0.102267941
2.57643662	0.681534252	0.914993429	3.613379617	118.8574821	2.499431168	0.047542226
2.280792561	0.968893711	1.594019114	2.430265598	115.1747229	0.374556844	0.018978734
4.162182965	0.708296263	1.961555896	4.267128383	115.6761528	2.227159397	0.06295931
9.552289883	1.162243875	4.849650426	7.158178267	805.1774665	13.22193253	0.149738441
3.712967775	0.838997733	1.920515662	3.764406484	84.15050989	12.02855512	0.047422866
7.538644225	0.434785339	1.597821742	2.670157354	77.04756125	8.079648381	0.098847597
13.51612175	0.642415342	4.667443509	5.860153924	478.8371233	11.01392974	0.263365123
3.16044876	0.598333003	2.651344647	4.15282285	72.3978851	10.5332374	0.058946451
3.583452312	1.073772418	3.514329668	6.999786456	154.8870672	35.45808494	0.053852032
4.386777974	0.513291889	3.893820292	1.900354136	51.29490094	4.596612328	0.007247092
1.866968533	0.543649048	0.923341574	2.758303537	73.43218226	3.15398903	0.025358483
3.497220099	0.759466091	0.876321297	3.92595569	76.95648322	7.299446796	0.039400183
2.978306681	0.565248988	0.569893047	2.690164143	53.56301776	0.473552716	0.044345573
2.678830011	0.819883919	1.003362825	4.224782557	222.2452137	6.86340121	0.074924455
6.544681664	0.798041577	3.968140384	5.232591896	321.7306001	2.753546766	0.12414127
3.18513395	0.631273469	1.2861269	3.989957052	123.5618341	4.203551097	0.040411843
2.817824788	0.914308439	2.208882204	9.466598483	221.1045912	12.38525664	0.079485582
6.088468806	0.861192972	5.986829485	8.561941983	48.40238997	15.72722467	0.051273753

3.825063255	0.521680553	1.565031834	4.175567222	352.1937335	2.589914715	0.177882036
3.838966567	0.711430575	4.299669581	4.360959825	366.8665649	0.535517958	0.089315302
3.033135196	0.842566436	1.717322445	3.658930818	92.67990098	2.223286169	0.057805137
5.142102852	0.689459292	1.663860602	4.969667583	196.4590364	18.19134705	0.123913786
2.173974477	1.013302666	0.476635524	3.627866169	53.30143784	13.12420466	0.011603953
2.519212801	0.54095009	0.855524326	1.934068253	69.28705109	0.95833872	0.051698716
5.329634742	0.590850181	2.179563186	2.874198514	99.53007355	0.645423511	0.066443254
3.482753738	0.960959615	3.920992795	4.515514068	106.1933977	3.248874547	0.027172835
1.725490174	0.511735646	1.350094975	5.124320466	84.31269894	10.57767392	0.028578351
7.749060362	0.512786982	1.029586873	4.443444308	134.8049455	7.942986343	0.156387151
7.206582718	0.98232823	0.961919265	2.065912882	512.7882381	0.871392686	0.058695772
4.198910054	0.50605144	1.044147743	3.637163855	120.4116049	7.66087848	0.084958664
3.618213695	0.877881481	2.870585709	4.979368528	205.1098775	4.293686089	0.051795616
4.702370159	0.84728838	3.153247378	5.683466358	172.5191853	10.61704414	0.09181576
5.18117134	0.659570827	1.603718139	2.683237378	118.7519265	6.136346608	0.052916525
4.356937385	0.605314405	1.812898605	3.960216284	141.6442448	3.66807855	0.120966294
3.261909862	0.699790304	1.009430879	2.793499519	26.73867773	4.18755788	0.027105374
2.162551229	0.716622835	4.472921926	6.802408243	134.3427761	3.927005843	0.014539882
4.100990106	0.649121648	2.746934526	4.527452639	176.8475418	2.006083297	0.106791899
4.638297006	0.798090896	2.534494917	5.615929784	81.16166758	14.51098388	0.05509538
2.653164881	1.113845925	0.947446721	3.477319217	70.52051576	8.067504587	0.038862646
5.033985824	0.75134032	2.29823198	3.072486379	107.8710693	7.247932645	0.143337627
2.274197701	1.390005012	1.320787339	5.13466884	115.4481614	0.914192325	0.014576822
8.118990467	0.619628441	2.568793994	4.828507465	236.1452387	15.64672698	0.153244503
3.795453445	0.661837251	1.986201628	2.655457316	82.97603458	4.875171173	0.048711264
9.752334827	0.88718663	3.78664738	7.245531506	591.4261635	5.909937405	0.514151599
4.526689601	0.762369208	1.178622857	3.729661302	106.4056274	10.93065657	0.100941069
4.623825777	0.694808499	2.003568905	4.872833989	196.6213344	1.263437463	0.103763672
2.614517978	0.632971048	1.520016529	3.778906277	120.4487024	3.828028161	0.031333983
3.276656139	0.682173714	0.995488663	3.98213997	91.09260505	2.346709097	0.068677428
3.591555669	0.87929095	5.342646174	6.825501873	233.0277822	14.9575528	0.033427861
4.58667102	1.09641488	1.15295547	3.203646715	45.65975937	7.891749704	0.029831357
1.738804102	0.608543161	0.73626636	2.174132442	42.84336918	2.059064177	0.035780494
3.104519391	0.656104006	1.144697547	4.136824727	77.0149447	3.701945313	0.054946194
4.375660742	0.487089892	0.937708312	2.277972438	33.55394222	2.055742819	0.017758572
2.404804177	0.83890618	2.169117717	6.050404382	119.0250412	7.264553561	0.042615645

3.132685334	0.647000437	0.670764005	3.479817217	43.22673822	2.987697773	0.060509777
4.641921338	0.772692421	2.64592088	8.090731306	308.1584311	6.072397315	0.134858591
4.445035346	0.920676822	1.408982204	5.343298196	87.5985725	9.16554163	0.106510241
4.43504774	0.949270162	0.986003255	4.336128539	94.47902331	8.818984804	0.117972961
3.243710536	0.606575654	1.231534767	3.985973354	147.2630825	6.472176618	0.119341585
3.731334179	0.868880633	2.287415984	5.487818784	211.6553697	4.550883132	0.18711318
3.386668234	0.517444064	1.942464402	3.25127777	60.06616944	2.281095278	0.027637209
3.808239766	0.657510057	2.122700675	3.182054639	74.68274739	1.724636264	0.086378796
5.465377596	0.866120953	2.16887884	2.112107346	77.49850891	9.908508445	0.077318999
5.05396408	0.91869286	3.475618116	12.73700882	534.8050122	19.85195169	0.122139416
5.228020911	0.829019762	0.770422259	3.789960532	49.11941727	4.238459683	0.066777657
4.891375692	0.719857951	1.560496697	3.890767514	120.8905989	1.391735116	0.166068358
7.83626772	0.70315769	6.701487716	4.361093912	173.4626714	13.12974275	0.021476591
10.30088606	0.886860712	3.152671746	6.304301432	395.6513788	10.60329519	0.353760602
4.04326726	0.522423429	2.631751476	4.900243375	128.4410534	10.47441364	0.050139097
2.531762442	0.58273361	0.548779825	2.541644012	22.07899667	0.41993243	0.010707976
3.923215659	0.634419403	1.17075905	3.185866865	110.3520369	3.595968797	0.064186702
2.447244138	0.871156091	3.868443906	5.816549598	115.49479	8.80241794	0.046160258
8.854429874	0.98098887	4.191456238	4.616666219	206.650862	6.556244908	0.094625254
6.358122133	1.630184787	0.35681854	3.824919867	145.508594	9.740731323	0.065458554
2.973251024	0.631701688	2.351303267	4.556505482	100.1976104	0.923673991	0.052673954
4.503308691	0.573781091	2.368752865	4.458886886	160.218386	6.503864601	0.071343742
6.671377349	0.61344545	0.453242598	1.560178219	205.8726287	0.330099943	0.031955373
2.988197338	0.69218801	4.906619707	8.867756907	241.5169988	5.76924904	0.022907083
3.785615137	0.74028395	0.330972799	1.736318251	18.70134417	1.584061498	0.029470633
8.347202355	0.883939154	0.371673006	3.781933008	83.44948111	6.481509595	0.151214929
5.717403961	0.875243484	1.901208463	5.588999145	88.29105451	8.023150855	0.062438123
4.359798856	0.848845047	3.999676758	7.96919566	123.6789294	6.640349855	0.077218538
4.021539923	0.71851624	0.994266081	4.312309579	55.64013757	8.706368647	0.049357755
2.515449354	0.642314351	0.751921594	3.586134768	64.66644081	2.010366016	0.070457017
10.32097522	1.157039167	2.042921035	3.971453121	234.516741	3.484799024	0.060629771
3.476113433	0.746797196	3.519295644	5.248160933	241.6904233	2.331821012	0.061360075
6.937393222	0.896274551	2.122172076	6.439789921	299.468654	5.909631904	0.122721286
2.058864875	0.691795088	2.828430504	4.379360175	100.8991174	1.805199319	0.096370023
3.84956344	1.077822345	2.349762985	2.149957911	35.46006778	7.674979107	0.005923847
3.360269678	0.581397058	0.402114091	3.026203699	48.70810223	2.236735827	0.090628062

7.437351581	0.716086682	1.936111436	5.04503529	190.2718829	12.74937561	0.086049738
2.805872283	0.930330587	0.853376543	4.757414451	133.8551981	5.862143406	0.040082721
4.45710571	0.512074568	1.811901423	4.174906647	139.8062237	2.888786334	0.131352036
2.321842747	1.179121501	0.752632332	5.084475426	122.4680171	10.38523013	0.089917296
5.155768444	0.619996759	1.762899187	2.843733166	142.0870538	3.15775593	0.075189086
1.717726436	0.766822734	1.89201801	7.189487692	49.83874723	6.830036938	0.015253777
3.0779614	0.447401465	2.236773412	3.561457057	210.4182573	0.921189463	0.135917223
4.100286325	0.984541063	0.590644885	3.917369476	92.52112106	15.86384618	0.080022101
12.36459988	0.818335325	3.706734722	6.787536469	510.139609	25.31155965	0.132343071
2.062561547	0.659115131	0.509074962	3.018653441	35.11389729	2.753938668	0.043344358
4.447948914	0.468562525	3.37616072	3.664306193	139.5712991	7.502459743	0.058004809
3.127418615	0.533345333	1.546075772	3.561505	71.07988412	3.406212922	0.044079025
3.934793925	0.771802398	0.742413429	3.907706004	68.55293975	9.686697163	0.058126697
5.010360702	0.729760716	1.845156657	6.927539416	350.1324126	12.89701619	0.207903986
3.857263225	1.276881914	1.263675944	2.845423355	221.5440949	2.990032498	0.019937583
4.583057363	1.17882389	1.596638066	2.217601518	77.25172931	10.06448141	0.00832059
3.720735148	0.509625677	1.048906757	3.531266363	72.49113317	1.470827201	0.050785165
5.21158101	0.614857154	3.220622751	3.883698504	205.6780755	7.247239388	0.10318981
7.320911174	1.000560289	4.934116374	9.917343523	370.7265634	13.07450207	0.081588258
4.666183682	0.469068143	1.608119924	3.932057088	93.8691997	2.625403151	0.105992159
6.519102543	0.641064696	2.550719043	2.215641168	120.1902478	4.729884371	0.038107873
3.123157824	0.781165855	0.902522073	3.892806064	81.78110729	3.440684371	0.031911218
4.613899265	0.580168234	0.85587481	2.704935215	124.1454134	8.332143346	0.082112916
3.687063965	0.978205005	1.409652612	5.346352995	82.9668561	9.131267899	0.111479232
3.547058821	0.641100825	1.731469622	4.890184603	191.6455489	3.647390445	0.060245318
1.263708458	0.694042366	3.073731451	7.259009719	65.18361872	5.281960081	0.016859981
4.071966461	0.79381778	1.196306606	3.448614246	194.7254965	8.323039571	0.113008853
3.549565814	0.862033912	0.728868486	4.428922768	75.26433468	9.630035596	0.090715788
3.807242633	0.901177153	4.666897672	5.295482496	91.08377169	6.770140095	0.017868692
3.105636892	0.560994783	1.361674658	3.189020699	122.8227272	9.091172263	0.10799617
2.607340916	0.735872275	1.50846973	5.342930903	93.90453371	12.6538683	0.07288934
4.292271842	0.753752514	2.119727261	5.48310436	240.3223797	2.902944265	0.147717449
2.902519271	1.399900677	2.479847812	6.195514132	90.47183594	30.66298475	0.033551384
8.195866715	0.710046073	3.228075103	2.825961697	68.38375023	4.879309965	0.09041763
3.856170719	0.773334615	1.441880456	3.312788151	51.73848066	8.59628749	0.105644046
10.84967448	0.497412134	1.133919642	2.119009467	84.34389793	1.485412457	0.044504759

3.055730681	0.789655236	1.593057169	3.177146228	46.85452002	3.87498187	0.023581422
3.55314252	0.595468878	2.933040652	6.993746677	121.858227	14.41951881	0.060221732
2.863714179	0.748248949	1.40537472	3.582775706	119.0596267	19.9409692	0.061257096
3.524236991	0.658758739	0.521468478	2.233776392	52.57823629	1.810311024	0.088378676
3.939592613	0.530074064	1.834273543	2.644918042	126.1411014	4.507747397	0.036156147
8.418995995	0.294747215	2.413028276	1.790806609	213.4375636	2.538293213	0.076579403
4.409233732	1.318983266	1.292968615	6.573840374	184.319435	10.38113952	0.116293976
5.372471153	1.054385226	1.770905018	7.323254891	205.3700814	4.108489324	0.120862337
4.181625444	0.638472581	1.687862106	4.78949367	96.23113319	12.09013532	0.092041098
2.928642347	0.921275263	2.353999064	3.952846667	57.45150954	3.390849511	0.03322829
6.530395731	1.024081691	5.744075845	6.25249906	168.5400985	5.757164374	0.069518325
3.724460754	0.976618939	3.2001897	3.219844419	124.5600058	10.83837713	0.016169945
5.486788246	0.911186742	1.611329232	3.983928147	164.4202861	4.389366788	0.051854076
3.425951777	0.77560758	1.754048994	4.407212135	111.6999003	7.005569111	0.064504113
7.610615104	0.761640826	2.151352556	3.217385171	134.5175096	5.814213953	0.063849552
4.517635347	0.77352759	1.440527587	5.212117126	89.64548416	3.139005434	0.075759332
7.111923263	0.581659779	2.36260241	3.520545385	97.64154546	0.973442506	0.139649965
2.317808372	0.801991756	0.937328147	5.498424605	41.41038564	5.214426763	0.032581632
2.099334836	0.543813486	0.736613755	2.617630844	37.35758143	1.270036048	0.053693935
2.123177472	0.43617302	0.608219522	0.881513062	22.25800781	0.320376307	0.005055552
2.742172675	1.09851036	4.237057619	5.282301915	54.14064151	6.117328861	0.03994836
5.485862912	0.518909142	3.239604611	5.066116244	178.5470899	7.388555236	0.091353137
7.054145409	0.524037388	2.962517169	3.822317409	131.0309022	7.975363598	0.049970351
5.295269946	0.74471681	2.324193341	4.376546387	225.680601	5.041290878	0.153794846
1.878453028	0.961685952	1.566271925	4.825298013	103.3855173	3.19374612	0.020406675
2.653761192	0.542698398	0.331887505	1.712972638	17.49694516	0.224827234	0.019966919
4.61926671	0.918954639	3.703720293	6.339301251	425.6975351	6.315348027	0.071742443
2.47435816	0.663983279	1.529860583	4.187338634	94.33490041	2.978601045	0.046136734
2.766683046	0.675471865	1.493079754	2.812663905	140.0378665	4.471314581	0.063050772
1.491998685	0.514980194	0.612181631	3.445869604	56.09329248	4.511340276	0.019031322
4.86820743	1.139238664	1.249721066	3.981250712	224.8326981	20.55746304	0.210277191
7.665924605	0.652296816	1.815589917	5.294321284	205.4399901	9.942769234	0.1714418
3.508128466	0.931818268	1.302234206	5.545959522	71.8254151	5.880928221	0.067633226
5.226558012	0.421342959	2.409948395	3.564666256	145.4698896	5.798947379	0.037711815
5.399452482	0.708079948	1.526756236	4.033836088	250.0464279	6.599068025	0.130264539
2.008537702	0.803137825	1.092887805	3.782161361	79.54208755	1.412364228	0.048082437

6.495456811	0.713178881	1.39946631	2.786502118	245.1386365	3.527380105	0.267609097
2.337271145	0.903329339	0.724231826	2.806472855	76.19565074	3.916617409	0.015782078
2.587312244	0.603168663	1.624542125	6.061504932	212.8320831	5.361082759	0.072874496
5.506497075	1.151068989	3.669190249	6.340479742	144.7017255	19.89107748	0.050672131
4.299506245	0.746579245	2.911967032	5.522600636	256.5289138	2.876931133	0.073504652
6.981834227	0.818343215	1.039885946	2.949654749	74.7013085	1.776757602	0.106800454
6.778063555	0.792616377	2.109446238	4.477921443	95.45730312	9.985608907	0.213831479
4.470471003	1.294197794	0.15780028	3.483060722	56.7171661	6.86069707	0.072466657
2.688420194	0.749046594	1.469414696	5.91192845	96.37190605	11.5413344	0.0562124
11.06270029	1.224865803	0.288557502	5.510473613	214.0799813	14.89764253	0.270164581
2.958749358	0.790493463	1.277540308	5.033037656	65.38162164	14.59951725	0.060316187
4.373900993	0.797724494	2.383379609	4.12802947	148.2436934	2.379219991	0.131086079
3.088006908	0.703994506	2.195096808	3.779931562	126.4827931	0.48309517	0.052975121
8.833996822	0.599789327	1.75386069	4.273583928	312.1573975	3.865950009	0.255037093
5.086310572	0.768247423	3.431133384	6.028077859	227.3318757	2.735418491	0.072489893
4.691034832	0.395094364	2.557301214	3.691447408	115.0148592	5.637452433	0.052028689
2.740268518	0.559323484	1.000512028	4.608477626	124.6373403	5.608450508	0.049600334
3.940163823	0.632139815	1.162915589	2.96064418	95.9609102	3.015614743	0.108819234
5.132408809	0.644890571	2.068527526	4.567633902	89.53576278	3.591526927	0.084028055
6.173671601	0.842908961	2.265899664	4.336588705	215.3371972	22.16010786	0.261098087
2.674190825	0.580486848	1.024951513	2.633232467	69.63306854	2.103372357	0.046897831
4.41846996	0.602396909	1.494440973	4.531076042	108.2841112	5.957778232	0.087013495
3.068973928	0.731323872	3.30863395	5.989774918	110.997986	5.31391484	0.026181226
4.078171394	0.75191072	0.881716616	3.34574779	116.4184984	1.671559849	0.062385326
5.175331203	0.522398655	1.6646227	2.683262757	239.3529135	3.004362281	0.151182281
4.672239949	0.996544958	1.045103851	7.063683477	87.41487716	12.70186603	0.062378667
2.094159569	0.581099804	0.526677415	2.514813794	31.73867522	1.173622255	0.045523302
2.237624254	0.674941176	0.706717967	2.842809629	24.82041482	4.091653527	0.045636682
2.940760152	0.835532867	3.099248825	3.84446161	194.3561272	1.064381959	0.033374267
4.453714345	0.731240304	1.199574177	6.76429533	79.48320958	13.21033062	0.090550836
6.81806169	0.926888569	2.622948756	6.754928839	89.42485669	7.491506432	0.088236609
2.706501511	0.88752365	0.876893923	1.500635194	132.9569916	1.093273736	0.016336869
3.332607666	0.776871515	0.925628316	3.326999749	102.430722	4.192785654	0.102640277
5.473921674	0.91177929	2.394524252	4.540863343	598.0877042	3.588842215	0.115059727
3.549693416	0.682798235	0.839989977	3.008711789	74.64625002	2.238631536	0.073653447
8.198166536	0.69799419	1.86011838	4.57961951	305.8449476	3.225523293	0.232074912

2.118916435	0.796370499	0.836472321	4.519078167	89.03587735	1.726547789	0.047651789
4.018827683	0.757598786	1.974322716	3.769719252	69.18131109	8.858149101	0.043743344
3.677657288	0.80186125	0.507738942	2.853913517	89.81033468	2.532397906	0.16584091
3.697820869	1.41815099	1.121698939	3.083468374	49.35047784	24.3677232	0.010823445
3.624789168	0.592048251	0.547306341	1.938003351	110.7961569	0.901734767	0.105651383
2.751792119	0.76226188	3.107077348	8.719884068	204.8912853	15.66745326	0.095945151
4.47632783	1.12239537	1.351547675	2.972402544	94.81099433	7.277196074	0.066371221
2.998797104	0.802290343	2.435526714	5.474402358	80.54501254	12.96309474	0.03853737
7.936168726	0.867976386	2.561057538	3.684982311	402.3023911	24.59445748	0.081591425
2.065371081	0.746170509	0.977613432	3.864091238	43.60173026	6.317199667	0.014289129
4.91924176	1.092279819	3.046676614	4.14895142	144.3409058	13.18028331	0.155590784
6.003732609	1.027363876	2.715200465	3.656539459	206.5316168	13.3682056	0.1415867
5.384640396	1.360172099	2.033628283	4.474416999	183.8661488	25.16028179	0.106103389
3.205229252	1.123852492	1.373166403	3.317732991	32.45978678	4.298239913	0.02788402
2.002915542	0.630880829	0.196832962	1.570537721	11.27218318	5.8435861	0.013469635
3.331937004	1.165202125	1.240837367	4.660889546	49.42570991	11.63154221	0.014528227
4.705893461	0.684043328	1.358799953	3.417284613	128.4340108	4.386562665	0.092541521
6.0654446	0.965587376	2.330709229	5.293637089	89.55825355	12.92981629	0.04135372
2.352961973	1.265237001	0.515202133	3.82647365	37.59925817	5.185382449	0.013984754
5.776646677	0.675221701	2.524450245	4.533735922	170.883754	12.84794858	0.238558653
3.563175908	0.810683376	1.553315747	1.755072628	46.38981369	3.142842376	0.041327882
2.789031661	0.577931567	0.584257842	1.700219226	39.66547982	2.919632321	0.021615275
3.327361667	0.866947657	1.107095867	2.601639282	89.16422744	9.066610886	0.105572178
3.344246958	0.703701078	1.355494046	4.002568607	72.91378926	6.809435921	0.049072585
5.125349907	0.822353068	3.518614078	3.46171512	109.2099587	7.1277627	0.104645572
3.492491685	0.815851216	1.631113745	2.706107627	131.5191164	1.741389378	0.043155677
21.46446964	1.359091059	1.364105612	5.203889582	234.980346	46.69405288	0.143726489
3.656762942	0.92049478	1.198569059	4.359607469	41.14539452	5.227744443	0.020179856
2.498441541	0.857353009	0.466247981	4.254456484	42.88277512	8.749154459	0.052147869
9.690008093	0.87005944	1.539405243	1.720509542	406.3891351	11.75062049	0.081569851
4.744948768	0.708153915	2.470623371	3.288533798	250.236932	5.879830441	0.139205677
8.700706206	1.129696849	4.606385528	4.360150587	112.6597498	30.47922053	0.030203289
1.629395354	0.634569057	1.101597785	4.332683149	49.09014926	9.043251737	0.020109796
1.913224013	0.756153342	1.507809666	3.517029529	53.5283552	1.495474032	0.048406072
8.498047859	0.631952389	3.555492857	3.027025027	301.1404277	13.91069198	0.046454172
2.395242553	0.820894134	0.917241487	3.791671065	36.84043052	9.643474838	0.026268283

3.767548188	0.579771905	1.744837898	3.683569425	41.69493515	5.455247056	0.02906108
1.821686203	0.628232136	0.528920767	2.021648061	31.70406176	3.732122094	0.016338141
3.706259297	0.756439226	3.262887731	3.764493695	169.931528	9.541304591	0.059478694
4.15720016	1.035608472	1.117363337	4.800789146	115.8204718	19.76478607	0.025343756
1.533217571	0.879483043	1.499732303	6.081479998	67.79339052	22.63980488	0.042165104
6.883647516	1.023689431	3.534101697	8.476181172	319.5345461	25.2045657	0.145135071
3.112699745	1.052497998	1.755352838	3.157221094	112.7372165	6.061599315	0.045622871
5.10287895	1.563606591	0.976984199	3.152602204	56.98446732	13.47931438	0.093814266
3.041818142	0.582776886	2.872805704	2.330232368	82.38156028	3.646612287	0.047731014
3.071912313	0.966613093	2.541435097	6.751894129	219.1557822	8.670928015	0.07945486
3.447594066	1.102594144	0.407036997	3.400500067	52.43844736	4.666804194	0.026013328
1.853891344	1.1793421	1.208806669	3.265224277	19.28569473	4.552035934	0.009971545
6.961770489	1.73710427	2.285547969	3.201707972	45.21340617	12.77526408	0.027682953
4.951457009	0.986122909	2.631152305	3.590762753	125.40451	12.1994793	0.08009565
3.141511667	1.278735199	5.14544006	4.015170386	142.9630183	10.02084031	0.055470083
2.416770713	1.160168228	0.337200944	4.042273404	30.51899298	8.967080633	0.024942383
4.401370191	0.729391038	1.626381253	3.811949139	102.3064255	10.08851696	0.108601364
3.543414442	1.094989211	1.151630904	2.979499792	120.6154319	9.477908171	0.110085065
4.638286848	0.946450682	2.137474028	3.429760988	93.53697601	7.116036003	0.055780463
7.4190996	0.756577892	2.772147088	2.647958479	62.02521692	15.68569096	0.017983868
3.574358462	1.089601076	1.397737081	2.798222377	26.17456394	7.695659099	0.034253638
2.048019336	0.723487932	0.911896428	3.070301604	30.11541509	4.882090266	0.021184005
2.695947829	0.618040748	1.006677019	2.440326488	23.44648073	1.291162188	0.03327341
2.971237686	0.95316377	2.600513578	6.044911774	97.26321771	15.66542156	0.009398253
4.952647608	1.138262685	1.778638471	4.240439624	59.03972534	12.64960856	0.028667537
4.87824927	0.890359754	2.342620166	2.856356977	251.6133484	3.739849508	0.165948275
6.068241554	1.108978428	1.976160807	6.48199416	250.0996267	20.99114283	0.211331939
1.990052109	0.800294932	0.677135933	3.287783581	32.88609342	3.212713581	0.039985496
1.397029544	0.764273557	0.495456051	2.492830914	7.660242018	2.819002041	0.010714201
3.803290472	0.989203423	2.377506003	3.908689288	184.6291632	4.882669785	0.02441859
3.392063007	0.965822069	2.130066632	4.870815947	106.5790197	20.14243462	0.06349432
2.201974533	0.658477917	1.048543583	3.409092978	26.83739454	8.888422186	0.007713047
1.894987131	0.538571054	0.600394815	2.031431929	21.57777092	1.008317397	0.019810882
13.5006817	1.120755169	4.70393665	4.928849344	239.0576967	35.44316312	0.088761462
2.774368268	1.206003833	1.291121655	2.518968626	63.88441163	5.089901537	0.021135131
2.23635396	0.794253695	0.665966104	2.099073385	34.40010958	1.766799412	0.036497262

3.551613246	1.089381017	1.700899656	4.696781449	86.20925641	10.76084352	0.039346824
8.318377402	1.402553161	0.549358149	1.803719544	22.03107301	2.789236871	0.016504713
2.560395461	1.081043252	1.071937197	6.029790116	121.9843181	12.81940354	0.031424049
3.79672152	0.816940987	0.624956461	5.581471502	58.88449173	13.21282074	0.107416304
2.201868646	0.719551119	2.1461138	3.728306313	110.0957715	5.034594229	0.060519
3.86330351	0.692174245	2.288891492	4.309597408	191.3754821	7.360995906	0.104040141
7.903670492	0.44480071	0.436674894	1.542997362	55.30427517	6.675313842	0.090744081
4.276393093	0.621487814	1.665749883	2.256603053	87.12831663	3.594774328	0.073454517
2.115708343	0.853682088	1.611661356	4.776341506	120.2058738	7.593377932	0.042151789
5.096711909	1.179337889	1.835431916	5.010186949	167.5527191	18.32091295	0.163614983
5.487969448	0.726666664	1.528635685	2.762316058	104.632125	8.317629011	0.099236484
2.365822567	1.3621351	1.93648343	3.216067537	62.41133071	1.391842402	0.030120686
13.30846424	0.872129898	1.330082635	1.889875684	42.17614811	21.17380493	0.04805142
2.866085595	0.903916407	0.774701828	3.558001805	47.60449053	1.029756538	0.028161067
3.186618248	0.849336845	1.389925331	3.310867012	52.73074459	20.69619469	0.012658594
1.822924776	0.646889396	0.995989859	4.219304544	51.08091708	4.030845034	0.026671799
2.656740023	0.720087158	0.832099624	2.889571914	55.9767605	11.66531896	0.021845623
6.050265669	0.750252234	0.592372314	2.054378701	48.40400936	6.227106846	0.068478468
5.071356893	0.822115399	0.67881014	5.106292935	65.70970143	12.19368719	0.079044402
2.709194312	0.823299552	1.468230128	3.384105072	49.65056948	10.76831598	0.068730505
3.239170246	1.367611317	1.634242953	5.611368563	68.37045549	5.035597958	0.045447468
2.972000416	0.690790669	1.036636469	2.851923081	69.08329913	7.099209215	0.119109085
2.439988621	0.964027754	1.115949453	4.348514594	75.42641723	4.556734367	0.039270172
2.697901109	0.949747584	1.236617736	3.349124951	30.29994427	3.757679637	0.010888589
2.948014033	0.564011428	0.903195916	2.837449428	39.79412598	1.975805784	0.037090477
2.777835196	0.662449201	1.52063937	4.640724003	70.15058416	7.203562414	0.043102314
3.174624288	0.88367809	1.371208436	5.342151326	61.5167238	12.14986919	0.052796255
3.501904216	0.703649551	1.729149875	5.139831616	110.1171966	26.32931476	0.175550649
3.166840941	0.985439168	1.229211078	6.55474804	131.1002726	8.961186003	0.048670219
4.499437894	0.943887055	1.387966288	5.681774195	187.3839437	13.18888039	0.178642676
1.589361622	0.961418111	0.252885834	3.42700638	40.57604095	1.632461703	0.023806034
2.76097377	0.839403315	0.899007515	3.53303678	52.7415538	3.701056852	0.035704841
4.097696628	0.776357424	2.914644766	3.152706816	59.65761684	5.792105223	0.015067254
4.159377063	1.026752943	1.109165897	3.759175265	136.0614792	6.967578955	0.112192677

Crizotinib_1083	Rapamycin_1084	Sorafenib_1085	BI-2536_1086	Irinotecan_1088	Oxaliplatin_1089	BMS-536924_1091	GSK1904529A_1093
35.7940829	0.091510511	16.20676004	1.164837053	23.47048288	56.68722845	8.19127617	72.41112925
35.97031304	0.134505805	18.02847361	0.738273383	15.28942315	82.70818158	5.167671064	105.2549174
28.75115801	0.170788244	26.83300241	1.584193751	18.47563067	72.8356379	19.75076559	102.1627751
19.63311967	0.217399157	28.44202427	0.11840906	13.86560797	44.25003387	10.70564562	96.25118258
37.47369166	0.682970043	25.36035036	1.145535474	74.94136432	106.8428768	11.673565	161.180966
17.15755265	0.099395366	9.797336596	0.781758366	6.643020863	42.01400045	4.495793646	49.22267703
18.34726366	0.196862087	11.06885707	2.465541508	8.080464428	21.92001266	6.752862536	77.5398926
40.68251889	0.178633928	18.92329199	1.886350648	24.6543775	64.6246382	18.63569492	99.6942071
45.25970308	0.367253884	23.92602658	1.581507026	30.09763127	83.17876114	17.10479554	132.4407402
14.02802099	0.099726783	8.515044173	1.704410702	8.110178007	37.49837365	7.22529357	53.15779853
14.92573016	0.139030627	8.066821594	1.230155085	13.27456674	20.78040927	9.476345917	60.20131606
35.55613453	0.10996322	13.56614286	1.071139538	28.50294054	68.55108852	8.860708979	85.3365518
24.46813633	0.059616492	9.611051544	1.57528195	17.88000442	62.38036865	7.696557259	50.17339028
38.46265024	0.302600347	21.90312374	0.19047695	23.30549981	72.08281092	19.60396972	135.518382
20.79897699	0.132804213	20.23837853	2.103285734	12.66305014	55.77555603	9.760285964	87.69992159
23.75125264	0.133125706	14.10842277	1.564365113	24.76545752	62.1454682	6.330831901	92.89846801
14.01706028	0.092727968	4.85721139	0.307365684	4.771623659	15.13637232	6.67289013	33.99592239
28.36965905	0.247406496	25.74226093	0.917239027	24.01033199	28.79224696	15.81899478	104.2631568
17.99229324	0.214276559	18.35299987	1.84002315	17.10963764	44.17334028	8.354440995	98.87685189
11.72258418	0.085973682	10.94844356	0.938569564	5.174287235	28.71824257	5.788126663	58.10055186
16.82887301	0.117666053	12.49115571	2.087263036	9.358714539	44.57323616	10.25476121	91.28222696
31.60804549	0.210375693	13.84798733	1.095916787	26.761198	44.3368661	21.61922216	132.2167222
13.0056087	0.148760547	13.69085825	2.362660196	5.085605245	22.76668099	5.503975028	93.65318169
34.83903886	0.240400331	24.68378762	1.562602466	11.08044753	61.99124355	9.184106411	128.703403
24.31365003	0.16480148	17.45329198	1.702636813	10.22914858	54.06366081	8.447877864	86.45140477
37.75782804	0.120322915	15.40224268	1.715616325	23.47396603	71.11745825	8.395018616	114.5534707
42.858915	0.232373474	27.11673368	1.144808334	69.04044334	108.5523477	19.2567077	93.74473448
34.59228164	0.274100998	25.00366474	2.040748504	14.80935346	59.34508936	9.761155925	184.9849492
21.56111313	0.0928082	17.09361295	1.48850273	9.640621891	45.81057111	11.4882037	87.3538559
26.75352622	0.061260104	8.739836771	2.53423902	12.65487232	46.93913669	18.81621257	39.66975607
14.35001481	0.109886	8.338152496	1.421409163	21.57709345	46.89543523	6.197872015	78.41161821
14.32169154	0.084901871	19.6585925	2.151072045	20.67941546	60.30837813	6.744190571	69.10845516
35.11613996	0.210297642	34.00110586	1.192959278	89.63930415	184.4316521	10.49876044	124.7769873
27.95572871	0.085619145	10.77493072	1.08862906	9.063626308	38.30829168	12.64780627	40.08717726

33.96661996	0.122517165	18.08161993	2.466056793	15.92662807	75.43490713	13.98791734	93.38705793
32.77719488	0.220013402	23.76261975	2.181404483	15.49129645	67.51432641	7.914813841	131.2495283
22.23078448	0.114728154	11.70765246	1.028946591	5.501962627	22.15202489	4.499644919	56.39133457
45.81157274	0.137039373	28.68897072	0.844153575	50.02773989	254.5324927	11.61900225	177.0701069
19.36015646	0.125354752	16.01131037	2.617641382	25.96130887	34.79092637	11.90204355	81.11508452
25.17461692	0.135326661	14.44871345	0.450779031	39.19359892	87.45031145	8.438958901	61.7522623
57.93945525	0.033052379	11.94565535	0.532177658	14.34002723	60.23124123	26.25191121	79.97125244
27.56000046	0.217083254	17.87190351	1.420726018	18.00009185	54.43658052	5.319307694	158.2083783
44.01661916	0.068798057	16.90863999	1.025561273	13.73728381	54.92205221	5.340344562	79.94396523
23.04011911	0.097960167	18.89570833	1.28337355	27.09602305	68.73246716	10.67915647	77.09275424
33.81167425	0.063763376	10.30680987	1.612680824	4.253078766	12.73963505	6.09794103	69.11966173
13.6499628	0.080505188	4.076585059	0.028739416	44.34792457	39.84305502	4.805699464	15.34107921
18.55780104	0.095399388	12.71937064	1.892173087	5.583626805	27.85206198	6.556750493	94.66538278
17.29577314	0.128760318	19.03717143	1.253522029	18.00118424	52.13375731	12.02768777	111.5384898
14.36595524	0.127119338	10.85089546	1.663554871	4.578410321	17.62918378	8.414372698	77.77019089
18.33244997	0.070857448	14.49640072	2.225177219	19.41138561	47.54133218	5.788345669	79.74053518
10.5951279	0.062351459	6.353365701	1.796564476	2.202631015	7.944608229	5.418525213	42.25431514
19.33807364	0.149118341	18.97235858	1.664855011	12.27453489	58.24387898	6.168870404	115.2579165
12.21111078	0.135998296	20.71509853	1.439392964	40.42740581	65.04956338	11.95336786	81.54254693
11.66426892	0.091173782	15.06549363	1.365198038	4.349515786	32.43235822	4.94842626	64.11068583
36.79738711	0.043473751	13.91158036	0.791857834	6.788057678	37.08919226	18.75081182	72.23235335
46.12404315	0.127670107	35.81750777	0.945106765	15.48985664	65.17774694	21.24097958	123.5111633
11.92543574	0.10058085	7.644070324	1.698093965	7.071412924	32.63547405	5.694503661	51.20114095
13.60569546	0.066286325	11.45422683	1.463433465	7.401658894	23.58339153	6.956586764	53.85092318
60.02788269	0.116196491	23.64907355	1.567918608	18.07436015	53.74872759	16.54173631	85.02602611
37.43150863	0.110121336	18.0601336	1.097766735	10.06561143	55.23425864	9.717305217	88.84014833
45.97152787	0.284247578	27.4132951	1.070327799	63.38744272	287.3287794	15.11699206	138.579423
22.41393454	0.135889237	18.84463964	1.178412528	19.42043701	82.03023517	8.231369503	87.78953991
48.36742939	0.232323093	35.50407147	1.075067846	98.9702582	272.5999515	11.6330147	139.4766555
23.68529187	0.100745477	12.56109305	0.448613273	9.284167823	51.41010324	9.678900079	75.496896
19.63204184	0.079070771	12.19856612	1.835742815	7.041764135	25.12550857	10.10560426	65.2289442
22.00878541	0.08534249	9.457223902	1.520986693	4.7524289	19.23548895	6.019430155	74.70823144
28.02765641	0.226667333	24.89385539	0.534440694	24.68168508	89.057292	4.179791608	71.4046958
104.2312235	0.419496501	38.91108301	2.334198	41.03768502	92.57870577	27.07574798	197.4837159
20.43511029	0.135341477	18.54077412	1.443430604	31.51669639	55.54463344	6.34690983	122.5532302
19.07000633	0.072214105	7.583345322	3.122055128	4.76355855	15.31814362	11.43038036	42.01965136

23.18011236	0.143601002	22.84043029	1.666098368	19.9740217	67.2465383	8.236781004	100.2760718
15.01144016	0.07307625	11.11184444	0.938572209	5.424860566	29.73542472	16.83262318	41.5025301
26.95724366	0.144910153	26.78263646	1.599363202	24.23677144	65.52181429	14.49208461	148.3260512
50.14552177	0.539806104	43.55898563	0.973885608	33.92477535	204.7634719	18.36301204	206.1956396
20.91297431	0.129232737	15.046617	2.44757572	11.977213	45.72763316	10.14262772	78.89286582
18.14989366	0.113276563	12.01722687	2.169389693	5.413634321	23.33714508	6.348102701	88.71668772
19.60034532	0.07686523	10.41247311	1.571375688	10.07393553	26.4322268	8.322813	48.24165623
26.16582854	0.098533535	17.11788338	1.389348955	7.408828269	29.20821194	6.050461292	101.152823
23.73947508	0.076878286	8.999543659	0.565524386	48.88027622	138.8862363	12.78200592	77.19355938
29.25450209	0.103736643	10.98919549	1.287526341	27.80733942	63.37435271	10.66649186	45.8020918
53.58699523	0.176564341	24.66545783	0.8397491	14.93899372	65.30766755	23.10454934	117.6141054
50.22815692	0.144962422	26.81173193	1.585440947	35.83712496	145.2696016	16.8307377	127.0961428
15.41165955	0.0418187	7.657955122	1.683102963	6.398013952	30.07532217	8.061016728	71.98876641
21.87802329	0.060631049	9.471414916	1.715506731	5.313161721	19.84089415	8.004247775	64.02476521
36.8893368	0.122587055	20.29738904	1.246044093	24.46424881	68.37595974	16.68354189	98.2326432
23.2941169	0.128107235	19.66665285	1.358141794	11.68469115	38.40431587	13.49237121	86.47123619
33.86236009	0.139541507	6.853312023	0.302313158	6.338450355	23.7748276	7.366643772	49.40872527
26.34663809	0.098753647	14.17112719	3.705451371	10.26095818	40.40503519	8.388442677	53.64862937
31.76816847	0.202346112	28.65446768	1.072381094	28.19332724	105.337764	13.03182504	143.2639105
24.53816028	0.105746502	22.21344807	1.84219562	6.916018695	30.12305547	5.501523687	102.6838976
26.03582848	0.150385478	11.31388105	1.547000838	17.78599468	29.74930344	15.08049075	90.16432341
13.18653134	0.047135061	7.584184322	1.68233462	6.650809903	11.75726626	5.984239144	64.35556117
32.49354277	0.153863851	14.02540842	1.451956742	7.693838815	21.55736348	9.330433632	83.26586015
17.25430083	0.068832239	13.3518247	1.518694142	14.24679444	53.01245882	8.817814681	49.78892833
17.94595336	0.087713062	9.985596331	2.086262963	9.387534776	20.07858462	9.61297978	97.10977863
22.49872575	0.103132474	17.85821583	0.726713595	8.958394169	34.89007294	12.15968839	84.15523123
20.70876206	0.103351707	9.926371756	1.510960306	5.013539162	20.03474427	6.691057618	76.33321976
33.7888466	0.138509063	22.06535342	0.880571749	6.254482427	29.62659325	7.277747687	89.46009226
20.48036821	0.146948399	17.58956806	1.148050976	29.36487313	57.01502489	10.21138498	89.38388194
20.89894534	0.198573881	20.65957651	0.366072269	25.45946815	70.99735748	15.68038043	84.22520325
41.00017695	0.104115526	16.93548808	1.394493444	27.17465984	150.1788749	9.122155874	95.40369524
33.90404328	0.138518134	21.947134	2.455543477	21.65429014	74.7093953	7.562767798	104.3027086
20.84688111	0.031175259	3.838177449	0.238527018	8.552727398	32.70669721	9.155367267	24.86433427
25.13234534	0.083732124	9.407455365	1.942167029	8.239452625	26.69873342	8.953521448	51.36311605
36.42128988	0.06069095	13.85413659	0.901421594	11.94565033	61.64927252	5.214685922	38.33558562
23.34598337	0.121603515	17.15519105	2.148009664	14.70659916	51.22188069	6.89291249	100.8037164

28.02003124	0.114471062	17.15114637	2.007977111	12.61428945	46.87898791	10.77619422	101.3686771
31.96477888	0.207319298	17.18169795	1.280802157	28.10023848	44.1623333	5.766935868	83.93564645
13.76718108	0.112944071	8.268081231	2.436081886	6.229463147	16.18210813	7.004315619	62.22446092
46.26268894	0.183987072	30.88770894	1.939827174	61.03691418	115.0346998	8.541517166	113.8134096
21.47070691	0.216808222	15.33003152	1.203764069	41.47573587	47.66998909	7.639589781	83.40987993
27.54542016	0.111770461	13.99602247	1.702200679	12.21278842	41.6874115	4.148279513	92.27805227
37.74942661	0.409045586	40.49472102	1.642884634	49.20612644	219.7101255	9.711381656	151.5989597
18.31549412	0.102446521	11.11083138	1.866833114	15.81824993	33.41599784	8.41841029	73.72881458
44.54493593	0.195361006	26.45756701	1.444926638	20.78094689	60.27011281	18.09779061	104.5519677
17.83953962	0.194607298	13.14549435	1.643042868	13.98284596	30.78257098	6.310458428	86.4540778
19.36639254	0.219409265	12.8468899	0.491031534	8.303609297	20.43268181	10.56700979	77.14794119
43.70861612	0.123305942	19.61022574	2.142044201	21.27340608	62.70147324	25.37826399	73.55610978
32.6537917	0.312496795	32.9740241	1.819867436	31.49372981	75.15224624	8.117142158	233.1773505
18.75044166	0.10304086	12.12521351	2.63702428	9.444181957	23.39599159	9.622874762	59.85610997
15.65818785	0.102490084	11.5868033	0.075002984	3.181514113	32.23999804	9.885116918	33.36539603
18.66019882	0.345038494	13.94535587	2.557799546	25.87170148	32.37474759	7.032139192	96.73474681
26.49521539	0.096999534	17.07921914	1.469714703	18.314365	45.04974457	3.522375658	85.4104746
26.81442871	0.110987341	13.30224181	1.359028524	23.22215787	46.9409891	6.599232145	83.96778544
12.2299221	0.231943184	15.56395322	0.954706145	15.01757526	45.1514248	8.937432926	88.28110212
24.30619462	0.151308343	18.31412302	2.377778622	25.49668065	42.36482515	3.804074173	133.7805438
49.39505605	0.129939491	13.7682512	2.265501582	34.03179713	78.62661203	12.72270929	103.866287
14.79495999	0.082506817	10.72208703	1.605696318	7.486507394	26.09396635	7.32427507	73.52581773
13.20856012	0.07186067	9.823956927	0.778600334	10.46850085	26.30221467	5.953479318	68.12605707
27.82593404	0.130026893	30.21729834	1.183931925	16.75785407	60.11815253	7.154600125	69.14290606
24.71225367	0.137691074	14.08594125	0.591959525	18.38752162	49.5713826	11.35093658	58.93485759
27.69285409	0.078354768	24.06492879	0.853957022	11.11579246	53.10289204	4.143490786	70.84038374
54.76759246	0.742123285	35.86983966	1.698444283	47.34769229	73.7553844	16.33237868	161.6617379
27.0043926	0.083624179	13.87480358	1.541960834	8.918161613	33.33949972	8.565432398	84.91859844
27.06066324	0.176501067	14.48754144	1.52991314	9.339349122	20.16788362	9.109572331	77.22758589
50.70189705	0.364174158	23.90501051	0.390302753	27.26200557	100.8438898	16.98137952	80.57496902
37.57840411	0.14318331	30.47820389	1.582877116	21.33778515	85.11274849	12.82443654	112.5136818
24.80341524	0.06868027	20.55216418	1.287521681	18.51082527	68.0795994	11.22581912	93.80197858
27.82070724	0.185658545	12.59369287	0.242134738	4.429757838	30.8202726	11.77606649	61.16674851
18.5828479	0.220468341	18.41667617	1.595705616	18.30397528	39.16926133	10.98635149	110.523923
21.43731067	0.104127449	18.87767546	1.510652446	16.87725745	54.2686043	9.797672177	82.47791263
52.84005271	0.22701318	23.75593755	0.938334747	50.82923286	87.62139832	10.3830208	116.4954705

27.70429743	0.236047739	21.79089824	1.622708949	12.40792877	49.48347931	6.882915685	137.2326275
28.49889127	0.220835207	26.93076656	1.057653278	30.7148412	88.69884425	9.766027186	133.4909181
48.21793699	0.097179957	26.51615531	0.830724184	51.71618211	280.2696645	12.01147852	50.36854588
28.53538216	0.129911562	17.35626664	1.981954373	9.648496699	38.11024946	9.572882139	112.5667235
109.9608957	0.267287463	35.08388839	1.188076612	50.79826889	197.4886449	6.639508841	184.2454615
29.11650874	0.056113874	11.93946516	1.576043055	6.063703713	30.55288944	25.11863147	45.06694572
43.52224859	0.146767443	16.58441537	1.41870966	11.70330188	74.80600488	12.460992	86.84088306
27.84935593	0.131898745	15.29427563	1.737465072	5.936932936	21.02827539	7.569643198	120.2606266
21.20350391	0.345758366	11.36241866	2.964082686	7.439811419	16.24092659	8.869978159	95.05332873
22.12583246	0.102155282	11.68150688	3.233709407	11.96823354	52.20469865	12.1922748	56.79385718
50.64097253	0.334568772	23.90593132	3.603593376	28.33524003	64.28219853	11.7937352	110.2132278
28.60441332	0.110689422	18.40228797	2.638424019	15.40658626	38.61791393	3.582884846	94.41236169
31.6534254	0.146184617	15.68377859	2.501573473	14.55218787	41.45815229	4.923502528	90.44690937
46.23634684	0.230732063	22.4921197	0.625747784	10.26360605	40.22185421	21.81037375	92.09156939
11.99504554	0.21103273	10.50804919	1.645288681	6.438161066	16.28376366	6.298599573	69.38629448
33.16552694	0.112193749	9.953076241	0.76800874	16.71617502	51.48075184	23.16299736	64.64406416
10.16729877	0.061019898	2.157983657	0.609800587	2.375166762	7.214806928	3.844530443	29.55700498
39.15476984	0.14268055	18.26538478	1.338619472	38.75082539	115.8045315	18.52097062	63.8361433
24.19189561	0.188492765	16.69189677	1.659758229	11.67248634	46.89152541	11.98919389	100.167461
20.78904585	0.061301325	7.904588924	1.842705385	4.329090129	15.9580425	4.127996614	57.18922564
36.81608045	0.044935949	9.9971682	0.627244834	48.15744452	67.60821074	7.600757569	60.8888037
18.04759757	0.114906771	18.21962277	2.374588776	7.332560191	25.18240402	5.150774086	68.85673153
35.3621212	0.076571028	16.02593351	2.58000293	10.38445406	42.26758686	10.01937902	101.8027387
20.18226924	0.12293995	15.295116	1.189531842	13.96322142	29.773558	12.58233871	67.65161063
36.74889033	0.120571095	11.31617595	1.72366852	35.05108676	71.45394663	13.38704648	78.02094941
41.20133159	0.249060892	17.97187539	2.771025737	35.36892174	50.14933871	11.71812809	103.1402778
21.89395295	0.101941712	18.35402122	1.405818732	15.24972474	58.77952464	7.952411598	81.91161298
28.83748863	0.166221971	28.84126508	2.808073607	28.78482721	104.4423796	4.816207016	99.88038806
18.25261553	0.114858509	11.57955033	1.964116624	10.1600574	24.93071806	5.390855876	47.48216896
14.04152774	0.066181276	13.73325749	1.509523658	15.38692564	40.53413493	4.257592854	69.16299424
17.84064935	0.10321526	10.4040848	2.685182667	8.396381072	17.29871305	7.187306318	71.50738678
25.3144733	0.045422865	9.981863181	2.00122705	17.00223653	38.52426609	4.624352929	53.32814519
19.06826054	0.08818863	11.50112245	1.83184631	4.496756667	15.255753	6.488280953	85.96131218
43.31517596	0.096920606	17.44242788	0.649960995	57.71566425	91.02951907	16.41748599	91.05639914
24.67111772	0.108888755	20.48241771	1.422861854	6.523415486	54.1332672	7.258707386	91.91711682
19.63732633	0.091724012	12.45442241	1.63721792	22.45701852	72.4532726	5.284734268	53.81101652

11.54915929	0.069206304	6.668273158	2.19333284	9.397940274	17.52804199	7.554050895	52.17178456
17.46272448	0.210707553	23.1430131	0.901611303	10.46507527	53.47471344	14.95406576	114.6737242
31.77121613	0.100166108	12.76539687	1.364858001	6.935289282	26.98442543	13.56125727	95.60492332
42.51046348	0.091901864	18.04335076	0.753353773	18.86952198	52.86405772	12.49306988	137.3205185
26.61452286	0.129431303	9.050023545	2.253447369	10.37099447	26.65276544	8.924374828	51.13052887
66.0036447	0.201523478	27.83369098	1.510450095	48.07902526	102.8256322	41.67244878	89.19396224
33.56555297	0.125477379	22.61036324	2.60025577	28.15526527	61.74522241	11.15265128	109.1309476
35.32718777	0.248944329	12.18495567	0.784495824	23.74476908	84.87700855	7.679014876	54.63235534
15.28119276	0.097457038	7.316563411	1.48406155	10.81022514	28.70078076	8.768331692	58.98397817
37.50550145	0.070853968	14.98088162	2.586159321	19.51944726	48.31840676	7.676196395	93.67421488
16.7476518	0.116686034	11.09424999	1.890568792	6.182018607	17.71639179	3.498250993	70.20040928
24.83285347	0.142591667	15.81014889	1.218031288	17.21321183	63.61596243	5.649907002	72.55584876
60.69784193	0.107102651	11.88596471	1.38686646	28.84915139	73.50671735	14.3908022	42.97100261
32.91851995	0.130778629	21.9201129	1.93880235	5.876485373	44.82607142	10.92516154	99.05969404
16.94841334	0.115323509	4.043440576	0.776685913	15.84344294	14.59967465	5.581676938	48.88700263
29.13431978	0.076162474	12.50680487	0.997489148	7.938957194	31.90785178	9.977919978	85.41132839
17.81834503	0.091905612	12.73328692	1.106565684	16.84088729	67.09680239	7.841884211	102.7615886
27.57853528	0.09142707	14.54765327	4.658201488	8.647328525	39.65071214	19.82252582	54.93285108
16.64460545	0.095215762	10.77031757	2.106221639	8.188235083	33.47637119	6.868559817	83.52425987
25.49209563	0.047248227	10.50573374	1.082599954	9.516956926	46.61185906	7.011994993	40.90931597
31.70921778	0.123885416	16.8540089	2.575169943	11.59121719	46.68109641	7.37338402	92.71400758
44.29858314	0.191289227	30.6967928	0.841627392	63.87816557	234.6191699	15.5424189	104.4419296
23.27192425	0.122711455	10.33047389	0.912269932	2.67076691	11.05644909	24.432039	34.03470579
20.80808053	0.143189604	19.89325975	1.821670701	8.634026315	39.85741978	21.14960515	92.42107917
80.81552875	0.218264002	23.78324123	1.083298642	50.52706583	129.0152681	24.75203924	116.3748286
18.99175502	0.067584787	19.28505439	2.09876658	12.25753252	56.71202963	14.3686218	101.0643289
41.55332997	0.077578702	14.51869679	1.268158652	20.39296176	58.96416843	12.86363294	64.38961474
17.76680065	0.056588752	5.797139545	0.148316678	1.843699873	16.6828259	9.971537881	20.7774648
12.88207091	0.049227218	6.311211056	1.477963999	3.724679995	17.04908347	5.559424359	32.05097122
14.61047493	0.098406549	9.983659173	1.701788204	10.01448692	28.16539074	7.349418993	87.27377007
13.42442001	0.120619709	15.28018012	0.988312908	10.34371112	39.96953502	5.548341742	75.65781217
18.58737102	0.166853698	12.44012164	0.93623472	12.14397598	33.03281804	5.308192534	67.43578606
39.16033908	0.17612687	19.95420803	1.851853935	27.78940428	92.812087	14.03162932	68.26512334
18.38343624	0.102175636	7.75189552	2.207892978	9.021177983	19.09947479	5.328922181	47.8524755
37.94841731	0.091783244	16.67352374	2.141792844	28.8610597	60.61419413	6.352722331	84.06280558
33.60001678	0.098020463	15.83295854	1.04177058	16.16907233	40.19252806	19.85324732	34.83271346

32.25838373	0.156932379	25.64942221	1.055730803	35.64447784	132.4543823	5.422038162	123.1130115
35.61978861	0.193369617	25.37329058	1.378373959	57.48742654	124.3426374	4.890630759	130.1075389
32.77888519	0.119778603	10.51570728	2.24769566	15.99357943	41.44147375	6.439358157	60.59295908
25.24535198	0.137705796	18.39267186	1.363697361	11.4328827	43.89654876	11.81961975	121.7397464
9.133017515	0.052653027	6.2420162	0.584719782	7.574494981	16.01159062	4.954103751	45.5882093
18.68331401	0.113635825	10.11464884	1.383222055	4.984028789	22.12661071	4.48255791	85.48486149
19.80280267	0.174378806	23.4843987	0.710940573	45.09402863	85.92526312	5.972885268	118.7010246
35.90087884	0.071522009	10.18025798	1.049032618	38.07677537	118.2059873	6.536802741	89.93012928
28.87095354	0.051520076	11.58512268	1.311911517	18.69187645	35.9557968	5.541595197	76.03838586
28.08007321	0.176952067	15.87286234	1.314574375	15.14578328	35.40488941	18.11712277	70.21314541
37.6764525	0.319137999	29.90390504	0.266609909	47.33618738	184.262532	7.199246968	101.5177638
30.4224968	0.082209556	23.28266517	1.485990833	14.65632153	56.31956172	20.19811492	99.17092489
32.6348268	0.125431015	18.48345809	0.975087544	20.71119767	71.22144288	8.575896569	102.2819229
16.77382881	0.179689798	12.55629309	1.43567846	19.1880535	58.8631791	11.74350624	72.69405606
18.45426945	0.079150977	5.659951239	0.841583472	6.578868175	21.88077012	7.31307558	41.04869026
24.58529479	0.14068742	17.63482795	1.560210108	9.76843203	43.13238063	8.177604049	82.39149778
9.252017151	0.117394817	10.95524985	2.005414617	6.089286087	21.37362037	7.304214895	76.12890267
17.7824694	0.070254173	15.38421776	1.209532608	24.89576073	67.5572918	8.40171755	60.68073958
21.6986322	0.12626043	14.97623618	1.533521225	20.51685573	60.8431128	7.368519512	80.40977847
31.4426225	0.076864238	13.55658399	3.035124734	5.953801175	39.16860231	13.92115052	53.47195537
20.01077418	0.091932027	7.533067569	1.800009212	4.746127106	22.54445996	6.062101087	39.60181667
21.48976631	0.167747828	15.99057691	2.2503822	9.171463289	26.96654702	9.806047455	94.74328772
22.29008292	0.135244958	21.10092895	1.148404368	25.07519472	121.2745174	4.144497477	88.7995023
27.46744862	0.210019307	21.53417812	1.936368229	23.46950734	61.42673808	14.98388714	126.4648322
21.90540436	0.102688223	13.5053288	1.809256181	5.730055425	24.23919016	9.236092155	78.93203261
62.51214185	0.310785161	41.22733752	1.70674973	44.48494446	124.9509737	12.30140424	154.8276547
13.23585871	0.12724817	10.68207511	1.104596968	6.768029139	17.71033123	12.9974802	45.04102046
30.82751961	0.111864287	20.03604719	0.796001258	34.77700577	85.69529838	8.023742928	100.0418999
17.05163371	0.069153978	10.18842213	1.951498472	8.974051341	36.2082788	7.417531338	51.22112678
18.51759997	0.124103848	15.41657084	1.32719451	10.8384855	34.433056	5.015445331	105.7714016
29.92135139	0.086709467	13.64432881	2.051984036	47.35555387	107.6005824	10.08241266	72.46026639
22.38074569	0.09307008	8.070158778	1.634022808	2.750191891	11.99290259	7.530993416	46.13446952
15.67666629	0.063678507	6.507929233	1.628325062	2.483288828	14.13079555	5.907643103	33.41857715
16.34991351	0.073929419	10.41694775	1.822895484	9.341097083	28.97206752	9.784601732	55.53492877
17.10872394	0.073343731	14.70275618	0.846606184	8.427342664	28.80810054	8.791863881	58.65988484
40.13736767	0.091262453	15.54834409	1.873658045	7.514725247	33.49063797	10.99125481	64.79458399

18.26313153	0.091975063	9.748607497	1.939618315	6.367867095	24.53349608	6.440724915	65.93383383
36.48488133	0.144372253	23.09971036	1.72645505	23.07918075	85.92138401	10.55737359	125.2911324
14.5716678	0.116557061	16.76482603	1.368632533	15.60215305	40.19415684	11.39690491	86.65091344
29.37213173	0.144554147	20.8504249	2.365036939	12.74103283	44.91091181	8.258740736	91.6927877
22.30192257	0.146222282	13.12619124	2.550947282	13.78649073	30.5263705	6.239616037	91.67812425
30.33612589	0.130620091	18.63763861	2.054047321	11.42013309	52.6627539	9.850106707	79.43605871
13.71608543	0.11172028	16.3387968	1.870310448	11.8333449	34.15603288	13.56846173	96.22301495
27.41708554	0.124883619	17.87508266	1.205992812	17.96610211	44.2233133	8.216880617	90.40810625
29.17535072	0.107150471	14.34823742	0.735523774	12.71290148	41.89142821	20.39182334	44.49456601
53.31020155	0.156807507	26.39115814	2.963768164	41.57144244	67.30512049	11.61487977	117.0189537
15.96433866	0.084188694	14.0508084	1.839863751	8.94859476	24.40114657	9.0267664	88.72144678
39.69406882	0.195224353	21.23136772	2.007471024	8.438836193	48.46106941	7.664784299	97.81107707
21.77960813	0.078401036	3.666015774	0.241780231	14.22597377	54.49690364	10.93402565	35.91041693
45.16385101	0.29326105	26.3052809	2.264943964	27.23350548	75.61336525	19.21617556	95.98760234
38.59965989	0.072883386	10.47430536	2.200060323	11.78056997	46.57518986	8.005505936	60.43765738
10.60756006	0.064127567	7.7438579	1.348815064	4.494336018	22.64516623	4.420189936	54.53356466
22.1389003	0.140894383	15.16077725	1.989276629	12.5229405	43.63460789	7.090613074	97.62015499
33.96967263	0.080925055	11.02386388	2.246784986	20.75220056	53.09170339	6.878739437	52.48371039
43.86477566	0.141314907	15.09998599	0.749646938	23.73981747	54.29864914	7.995603707	63.81112568
17.03190787	0.450179562	18.86996799	0.490988958	14.72640224	29.0411414	9.247186374	102.7971358
25.95579957	0.083776848	19.4371975	1.493503694	9.788843764	58.75291039	4.897925626	106.7332337
21.85524013	0.105350961	12.18542535	2.334028305	14.75461878	46.54111465	9.535928329	56.89777715
19.58457979	0.139164193	10.52746395	0.086792072	23.96626425	44.92783126	4.401851858	47.62260481
29.12017367	0.058629844	11.03181919	0.98138807	33.51219547	147.3609103	9.26416476	51.31107431
10.30115653	0.061942273	9.968192179	1.371359193	3.699322985	12.30081662	4.302279887	62.1999098
29.30591988	0.514434982	20.75016238	2.012002963	22.17921374	35.21234533	8.061922454	127.6986704
16.70996941	0.172397195	16.29926286	1.470781979	13.58643419	45.00240826	11.72157146	116.5390031
33.39000887	0.14401207	26.88458605	2.475836733	14.29274293	77.70058613	7.897924316	130.2081822
13.79468443	0.073248073	19.25856224	1.974089718	5.463852501	22.77950307	10.07464351	81.12492279
21.16042196	0.06457075	9.946449053	1.764261037	6.859853515	16.96385673	5.689453753	59.6144704
43.30974661	0.366705231	37.79105634	1.334104323	28.03508369	87.00064434	15.70403142	102.3267683
44.68537674	0.078161862	18.54663519	1.762289722	44.45972276	215.1644112	7.215317445	90.27072191
34.54268281	0.201315054	28.46018008	1.433428098	28.86600078	144.5829782	10.95699981	164.5680484
18.85270746	0.067984834	9.639726808	1.721033305	16.64246267	52.7390454	11.73334536	35.91479223
21.35532145	0.098168012	4.871325881	0.241895576	1.896574381	30.29413577	6.472812549	26.68506022
17.77628441	0.099093198	9.517358566	1.742185843	6.477909837	21.45910396	7.907568544	61.70201203

28.63100041	0.126631829	14.76331938	2.549020484	17.68471098	53.1481573	10.01202995	70.74665452
14.67772946	0.172046353	9.733011489	1.208388052	11.15841089	27.13172934	4.90656036	71.27669615
26.74373808	0.149366219	20.02284987	2.065252313	19.36524005	62.33641713	7.44470222	148.2647891
23.98330454	0.121237535	22.20373351	2.310544618	15.30137645	41.46796857	7.251517977	116.460558
24.38428019	0.095721466	8.703985761	0.906506503	3.912311923	17.80640925	7.344095563	55.74718512
12.20143536	0.060108756	7.796914381	1.855348677	9.984007221	19.94090369	8.427111683	56.27909702
46.82890466	0.111206316	21.09201179	1.278232522	17.73969495	75.86589177	6.499378929	99.99863902
15.68226277	0.166258174	12.17702797	2.074073424	7.956148295	17.36201781	6.465345342	77.50562168
35.06921978	0.243636371	24.00944527	0.5765277	15.19242715	79.51170957	21.25651488	100.5256008
12.44477375	0.105004878	6.617436138	2.335502066	5.283337747	13.31547788	6.340405278	48.34461812
45.00892825	0.070370125	11.39253489	0.912912725	7.449833782	53.9345973	13.05239182	66.70387705
14.30724795	0.060505007	7.067746887	2.178343417	7.472299949	26.66541043	10.58561236	54.965055
19.17381579	0.149198438	14.81717645	1.961391807	11.060172	22.89803163	6.966487404	101.3709027
28.56454258	0.126791216	17.77343772	2.107373766	18.27013914	62.58824321	12.48515789	113.1878455
20.96372664	0.065521663	11.39001263	0.27261359	21.04160572	82.89439156	13.80671054	47.36847325
18.86176149	0.031087035	7.632845549	0.115063274	13.08498525	41.04788723	6.577315485	35.16638991
19.18262224	0.094859275	12.95371617	1.430200027	6.563648498	30.07197537	9.122497891	90.16000523
19.93026177	0.172051163	14.61515177	1.170589131	12.68389387	58.70256467	8.582857791	64.72138804
45.08733349	0.207814892	31.03237049	1.904195055	73.21475209	169.6617424	19.85100207	125.3127944
24.49442477	0.095479374	12.09774324	1.94162069	12.1422567	30.50846102	11.11580043	92.77511863
25.20763865	0.087928883	10.40153366	0.607304494	7.450138663	28.69027913	8.377394405	66.73567101
18.72347149	0.156775526	17.60385106	2.158816315	10.28533659	48.17326799	5.89978518	91.49653812
15.39787937	0.161640915	20.10200245	1.662588487	17.2785011	40.38389114	9.807237409	131.3743493
28.46417896	0.095366905	15.46286183	2.141735563	14.36740257	34.07770086	7.129219798	58.06279564
40.92507816	0.144186415	16.77489308	1.427427606	11.06993542	52.40665747	13.37816746	162.5680466
17.44390235	0.046188299	9.60241888	1.656363361	13.48844547	28.49364901	8.604042931	30.82608273
18.09473468	0.195210353	13.38485056	1.943656041	11.7797565	30.5691652	6.669078712	93.07326455
16.30824134	0.222340488	11.14496149	2.102105074	15.22467616	23.85824133	6.219934501	68.3181719
19.52678925	0.075523657	10.58360794	0.814413544	3.916330673	14.75371101	19.91292026	34.16870007
17.13007157	0.113238197	7.389237408	2.542646634	8.901256808	19.45652557	4.920995378	53.87426591
34.00271504	0.088165279	16.57774093	1.952884759	18.93115688	44.68135411	6.287264114	84.9512225
32.51878306	0.185674289	23.08330113	1.989086973	45.97080378	110.2145825	9.447437345	89.7887697
24.66033651	0.081865154	9.333144506	1.307097056	13.38154099	37.60246083	5.463234084	45.97946804
15.38393701	0.208974931	21.40642134	1.235088585	10.10375834	47.87228527	10.2383146	94.53163297
21.13029298	0.15821392	11.31639534	1.375698785	10.90501848	20.65975465	6.838255016	62.81969138
36.71955888	0.142259088	24.6996708	0.883401068	11.62797319	63.42835118	14.174587	102.4446794

8.802282289	0.087793187	9.898189378	1.183607258	10.10763886	22.45865912	6.869968003	49.96647442
35.54688859	0.080760164	18.06331889	0.804186779	18.53303315	65.21817576	11.93819484	86.62078639
17.66651486	0.087897167	11.81923369	1.462418251	13.92381684	30.10479178	5.761768961	55.1378432
22.5651406	0.139822122	11.92498892	1.757653351	6.245817794	18.02053399	8.928475076	68.2289982
24.68838741	0.092676703	12.99626553	0.559634525	7.198042303	34.3336349	19.66912965	78.96281624
42.38519828	0.092294672	21.20196848	0.305410776	12.94065837	60.1829144	18.661479	93.31927703
24.99094396	0.104407706	15.00438	3.045362241	18.4985557	55.6429913	10.74559045	84.60378959
41.21058559	0.266720883	34.20772742	2.926110081	20.63739957	114.7380396	9.790735492	146.1298741
42.7379013	0.089893821	19.49230333	2.320510059	14.34172205	54.15138489	8.696965144	75.76903895
19.00704306	0.087413981	9.495378824	1.259963141	7.015696782	31.96773047	6.619242255	59.9593737
30.53165733	0.16808633	17.79177886	1.812663595	14.71593467	46.10553071	16.72239084	68.64750325
23.55049971	0.090276942	6.766106014	0.521497258	4.646277318	42.53193815	13.52294445	60.78833454
28.77940487	0.15886939	23.78418303	1.485602465	16.0017986	77.8112661	9.334079051	122.863414
21.85585853	0.141494869	17.3974841	1.029818691	27.18263012	65.65157154	6.527851931	87.08950918
27.8646067	0.146570846	11.32228264	0.524451733	8.07989267	43.34531842	6.330616862	65.53209337
21.69636946	0.170045803	16.07983235	1.485882855	19.58583018	63.56402477	7.520898229	121.284061
43.37166238	0.195065733	34.13872961	1.47321276	15.75757877	79.27446057	11.66181206	124.4223013
11.8306361	0.104521632	11.74816273	3.213610866	8.295241876	23.59830427	6.399462224	82.21684151
12.1670697	0.06938793	7.916001173	2.848311778	5.194060559	12.7278135	5.417198866	52.0288807
8.65962463	0.027111831	3.17408648	0.178808315	2.41291477	13.38766976	2.104586663	22.15687713
45.36142794	0.043296709	11.28942788	1.440113157	13.06379031	69.48121395	13.52898682	38.18163065
38.21878951	0.095487784	20.21726441	1.705286737	30.89235641	84.89947485	11.80830305	112.0243982
30.20168783	0.064793301	5.602842038	0.444550178	19.74556873	42.48781681	6.90668931	44.25269247
27.43452528	0.187161542	18.74357104	1.227911442	22.25364896	59.10014998	9.930041223	98.95664752
20.83461437	0.068436585	10.48934465	0.362878525	33.75204173	50.19751279	5.06485079	52.21699828
13.25762718	0.07233002	9.827113625	1.866913877	4.811259515	17.51369909	4.28603853	56.31777948
38.82921789	0.173290866	20.68724357	1.595038124	31.15495323	95.5444373	10.84197214	99.28961534
23.5284233	0.092410714	14.67520605	2.296218788	9.677126825	43.90748069	7.816837057	62.58007985
19.81564901	0.091619829	5.886401569	0.990442183	6.468660214	21.74125373	5.821060139	51.99232721
12.43933493	0.072312528	6.90376661	1.455946449	5.85211816	19.5265942	3.442996154	56.28098645
34.05185382	0.222460172	14.05527233	1.369157974	29.35314957	28.68322118	7.038631613	74.62527839
32.22071778	0.164121428	23.68481685	1.248354274	19.6610885	75.32066975	8.170990914	143.3032981
23.2727484	0.098206104	21.4221515	2.634528042	8.754312628	44.51524193	8.022386053	100.7446106
44.0293623	0.062892276	17.96150663	0.318060706	20.81028215	72.67369958	14.35485493	83.44686895
23.1162276	0.139456006	23.22848863	0.836923089	10.73021732	71.934151	7.215819968	144.6619006
22.80674361	0.068937367	12.25805296	1.127306169	5.815973957	35.80189252	5.808297173	51.9692087

38.25845627	0.30397157	24.22647939	2.070449332	13.17774334	37.79966164	10.74677822	152.6595019
19.9440277	0.052318609	6.936130057	0.91161573	8.476103622	22.05411567	5.334218049	47.84998316
34.10537454	0.108987258	15.63356063	2.36514218	23.16003415	57.83468643	6.66732562	106.5300753
19.86192565	0.17705268	15.43702695	1.046427965	11.82237531	67.60570272	15.54129054	93.88573246
37.07197794	0.176964688	18.44561545	1.428879552	37.19132983	111.4098117	5.594966142	107.9025992
30.47237681	0.206724216	24.31779381	1.226658172	17.98716523	57.63357933	9.447369153	130.4385937
25.93173928	0.114857493	16.45382069	2.341909441	22.12892795	55.01482042	15.11141296	81.1032189
17.90467381	0.327604367	21.08035754	1.405707413	15.50281337	30.77849089	7.344900506	131.644824
29.6102623	0.08229212	12.19402214	2.392653636	18.43319964	47.71807757	5.71151861	61.80311936
32.15924012	0.689485187	34.68686904	2.060368049	39.27098845	69.86700013	11.08333199	135.3276422
23.33653088	0.088154291	10.31173333	2.072439477	14.29004201	43.17816895	6.124949349	57.49262354
34.77460692	0.128610698	18.19286064	1.407291699	33.32604327	78.77430368	8.612099438	78.66719472
27.73719743	0.088677543	32.68494977	1.079382345	20.85923804	88.51177055	5.552927703	118.9063904
37.13402562	0.230803482	30.94448165	2.674262612	15.42620478	92.32960886	16.20550833	143.1248764
25.49321131	0.133429952	16.12144016	1.319363048	35.47665116	79.02201091	10.2096455	85.9484908
32.27391229	0.087975481	19.52643026	1.935944101	12.28968138	38.00941382	15.55125474	119.8518759
20.08651991	0.082082277	12.07824408	2.775509751	11.48794192	50.31945325	7.934706316	98.10696506
23.88727493	0.126423282	12.39174875	1.78360894	8.050584523	32.86130441	6.919482593	59.99216793
28.39590106	0.125339076	14.38831207	1.481232879	23.48673901	45.81645935	9.021840633	88.86968409
37.3506465	0.136944594	10.42430726	1.352618591	21.40615082	31.12464295	5.389798841	66.40298031
15.49533089	0.08174539	13.06256008	1.456909773	6.931105726	24.3926766	7.26114415	66.59716918
18.11346906	0.1418793	14.59030302	1.444035118	12.54109622	36.87453187	6.636675271	87.94852894
24.12761901	0.110333297	13.65959844	1.023396782	22.55617578	76.51295686	5.409310144	54.74462933
19.01044789	0.224130009	18.74837447	1.213880832	14.7044465	45.48339467	4.791922799	93.72838469
26.99762807	0.140222711	15.14176293	2.052944966	11.92846261	33.90839352	10.90589924	66.89765044
18.79166614	0.187124695	16.19065715	1.644529674	12.72901447	27.00356235	8.148135471	118.0814821
10.86239057	0.059141512	6.874133916	1.686572918	6.352558284	19.21094291	5.596575055	37.32928567
14.69180904	0.071556584	6.064702887	2.230305534	5.640697883	14.69971401	6.342927557	38.74179811
26.04568541	0.080264962	12.29056082	0.440603209	24.99926798	112.0148315	4.811211113	55.16627302
19.86040872	0.126948526	17.03621851	2.067980529	8.260626726	24.96753134	8.539244749	91.49399069
26.47440686	0.165943767	16.3675358	1.154882314	11.11816973	30.22003119	14.87926559	118.5696345
26.571535	0.106124145	9.36724581	0.719774858	6.458739272	36.05876603	3.887681713	39.36888122
23.07180924	0.129190204	13.38331636	1.64640318	6.686481206	24.18057328	7.501471107	60.42425579
38.75007019	0.245088981	39.86874739	0.852114069	37.73183193	161.6223603	6.838148025	164.3415863
19.36117208	0.136413694	17.95031465	1.630907037	8.668029749	28.56133504	6.924772598	110.2212412
38.39410188	0.31099482	22.83220713	2.586316677	31.72679336	91.55487348	8.821651393	108.9433527

26.64763658	0.074878966	14.4912874	2.060609762	24.13164149	62.88781034	7.352060951	106.0835611
21.26342148	0.118981631	12.41693236	0.988817449	17.65888752	52.28305012	8.761161873	83.62791384
18.94304498	0.260062638	14.98831276	2.40383557	12.08626986	30.59527812	6.871496924	90.81079191
9.921713317	0.098800372	7.780060336	0.611687393	5.397260443	8.559046129	9.162450273	30.96507848
27.43115331	0.222252415	17.3672906	1.206575653	16.1247862	42.66074757	4.792523693	107.1969586
48.39246164	0.111663199	15.78901388	2.59592453	41.27713029	90.89883882	5.634293888	130.481257
30.44173257	0.106383534	10.45840356	1.969933693	5.712513754	29.07550982	9.948232796	49.41967105
18.92354645	0.065799188	7.627323851	0.773866182	5.48448867	15.99678911	6.917962552	52.32824455
25.13038876	0.230803193	8.676724274	0.329835381	30.91610971	44.58021707	7.206757723	71.51536037
15.52596269	0.053667915	6.457671785	1.658554643	8.998520921	28.36485756	3.700766047	48.0920643
34.79229474	0.146502949	15.99020589	0.92146972	9.537403331	45.69265785	8.690678227	72.97558781
56.96134577	0.321663262	18.82029286	1.179058227	22.1259477	48.46099216	11.84757846	77.45815978
48.77811197	0.127344928	11.28467305	1.175186297	13.50433577	31.17148287	7.775474573	98.92517065
21.12878882	0.087956501	11.09317258	2.041357513	10.15144717	34.93108928	5.966842261	27.72032585
14.96285537	0.040862677	7.902771245	1.083513199	4.147828537	8.009953974	4.009139045	57.74479335
14.20437952	0.10425705	8.418769383	0.423738917	28.5465314	39.60985231	3.367289309	40.66565709
26.83488545	0.124892729	13.15311498	1.493403811	10.40106743	28.83284117	7.502461768	78.09048038
19.21020275	0.122849566	18.01462413	0.525653286	4.056040929	25.70954376	8.51470036	72.38379392
21.3654815	0.078334507	10.36171158	0.53751764	11.12643302	31.12063742	2.666218847	79.58914743
53.71723419	0.174826298	19.33725387	1.81153953	28.22857928	70.14552041	13.47752237	102.5438644
14.20387537	0.095965171	9.606697713	0.874088688	2.556688367	12.19471087	5.405437315	55.00732486
11.01505231	0.075607951	7.844056004	1.234024535	2.915743237	10.06935899	5.761285651	62.61897851
31.23924957	0.130689784	14.77323501	1.708004334	7.002328408	18.31101427	5.38151934	92.88342043
19.34953798	0.112870819	9.065037371	1.630893656	10.49492884	31.48214275	5.520603068	57.41757961
27.78717259	0.122553157	12.88851836	0.998218051	12.34394064	27.95679724	11.01944085	71.18019187
37.51044429	0.079755751	18.46739143	0.55554418	23.9695155	105.9776626	5.198340095	49.24973836
40.61620002	0.27751149	14.64759886	0.65104282	49.20866813	63.28682479	10.77373346	76.85727658
15.26218207	0.092440944	17.93106876	1.53465145	11.04994814	45.73499827	5.207929241	61.31332089
20.75150863	0.08710935	11.29168336	2.034174507	9.822800014	23.98066019	5.486308105	70.25849095
27.68376836	0.129683511	15.07799181	0.077844743	7.663302109	28.4449323	16.26053284	48.6480988
34.06667334	0.209006794	20.73303012	1.149426946	25.61005061	61.74018074	6.46748626	111.6127321
35.7014677	0.103344968	6.018856354	0.398881941	15.38151727	29.0860888	11.05748351	38.49163888
17.98912104	0.063530398	8.429890272	1.242779927	15.19567837	30.08659203	4.239580017	61.86930998
27.1694428	0.054334166	10.52473874	2.241024079	7.649657294	20.88867687	6.318467462	61.04945981
58.896017	0.111461321	16.04821611	0.090086137	8.693166449	42.15372098	21.26950179	50.43734036
17.96901669	0.099858298	8.970454595	1.468190074	15.90587742	26.06881474	3.983157693	63.28898056

22.06847433	0.07211871	11.5239746	1.757663778	16.33644261	26.46716805	8.465567715	55.38531513
12.10240307	0.070583958	5.523196498	1.576082344	2.43229312	9.053035897	3.526529428	49.36363493
24.66288819	0.084073325	10.84855665	0.990139968	8.348866791	29.526796	10.24519739	57.93368546
20.40489723	0.097321528	6.400688648	0.614087699	21.20315509	50.01321021	7.146655691	44.26394845
17.58947995	0.068544961	10.24781223	1.03576981	9.878724069	46.55762877	5.775169697	54.01980863
36.54336084	0.104733614	25.78269312	1.030376663	23.37190182	82.24743414	19.77794162	100.2556509
23.24761121	0.134613838	15.64351329	0.624228253	12.0540815	27.55015846	5.405526932	65.79486321
24.53228479	0.190384782	13.55519764	1.086383445	12.5431604	34.63777635	6.885328353	42.2466622
21.91351786	0.104429867	14.62539337	1.307665678	7.306509579	26.9119531	9.090939484	73.43972098
41.21835356	0.058609956	15.68263658	1.774531843	35.4050867	126.4543669	9.06580254	73.95558751
15.81742418	0.09682107	19.01157671	0.974728455	13.26122531	47.29444593	7.619113213	59.61313536
13.95928785	0.063173985	10.06521106	1.059659894	6.81702899	26.8929707	3.89351801	42.54592775
22.90780014	0.079348438	15.29989121	0.217220096	29.64457908	68.03985991	8.854546468	27.1352168
26.54408418	0.103050079	9.956274399	0.725090684	6.000197845	28.58188265	9.389411242	64.05715723
38.4407793	0.071376426	14.93020341	0.79115493	22.34257338	129.0961173	12.04684518	39.06575566
15.52620416	0.092025863	9.197856776	1.668862911	8.20777252	17.15569564	3.561154647	66.32041315
33.79049666	0.177169803	14.00109265	2.389356442	13.26754245	34.54813121	6.521168511	71.9755846
35.12115762	0.200766642	19.9200825	1.057239096	10.96938639	32.54072461	5.969492566	92.92102828
24.00652385	0.091014485	13.75830631	0.654826709	5.24848674	24.41756387	9.018693448	72.06746976
14.73326688	0.073993319	4.199666892	0.432565112	6.587724693	26.36924103	5.303562713	24.26554809
18.56401158	0.092359321	10.49368546	0.543900437	9.052686291	30.8225424	4.652101664	32.93701387
14.61689392	0.086110306	10.87798731	1.841670238	4.317626686	17.9119193	6.227145054	69.38721062
33.05714778	0.087067282	22.37288209	1.896365729	11.56758011	45.4108435	6.094548779	74.81029739
24.09281176	0.07673599	5.88444848	0.631237692	3.569108551	22.32254551	10.08440332	46.39043032
22.03379481	0.10686094	8.152750159	0.680750336	10.44160116	20.9172666	8.2444719	45.87267345
30.68401263	0.248023419	16.73599897	0.594296336	11.20323599	39.05598989	5.447588298	135.2931557
52.11152198	0.101824533	21.46793224	1.100255287	37.71647032	134.3021592	7.128742953	118.3357099
15.67497267	0.082605158	6.834947587	1.988027491	7.149937011	15.46719786	3.799483057	39.90367724
7.742189138	0.043021467	7.569761464	0.98735233	3.127872399	9.412521957	2.612798074	46.07419953
16.22519491	0.099364305	12.23135905	0.660416343	17.53582409	104.0546579	5.02619035	45.73587852
31.47472859	0.102387857	13.2539212	1.296204728	10.48600493	33.01916494	6.987231774	75.40844213
14.82932697	0.024754888	6.442123749	1.219355449	3.245146068	18.84291586	5.853673739	28.8239059
11.40346118	0.040663508	7.844066116	1.56307188	3.466463533	19.15348106	3.345027627	44.16043937
56.92307839	0.318905908	18.3557677	1.280459809	22.71437944	56.34234479	17.15802738	98.74761413
20.82202736	0.088865113	10.4738615	1.271257704	11.33151398	53.2348706	4.551489639	54.42812596
13.30649469	0.063454186	12.70075098	1.259955344	6.504539958	14.27651983	5.244959616	47.99436079

29.02730054	0.111560293	9.378981423	1.132225844	5.458932775	18.5577742	6.088345632	76.60492456
16.81131786	0.069781565	7.257494668	0.315300952	6.507371851	16.99619577	3.083968315	29.35972718
29.37489495	0.092971452	12.84603772	1.052281538	25.12237201	74.48026581	3.749045424	62.0401822
18.89603752	0.101465382	13.32160547	1.897353406	16.02056943	26.50023684	9.718012065	78.41311528
27.95999961	0.060625064	10.92796235	1.712621533	9.62008703	28.67923933	6.88467385	76.02162423
34.52134678	0.112545628	14.13278141	1.362907056	18.03174177	65.61838808	7.361292328	73.08818347
27.99942797	0.091107434	7.955449069	0.91410589	3.973430259	12.71145376	13.63360388	50.36737747
14.47721616	0.104679013	11.09359295	0.572119893	5.183998405	15.69668269	5.997200622	45.06697728
31.95416127	0.079347639	13.04235636	1.457194746	28.54808799	65.60899533	3.511776166	85.73866062
31.46861973	0.17729343	17.20491499	2.46528614	8.557673559	28.11116464	11.22017724	81.59215274
32.66589176	0.1068943	11.5490581	0.651468866	7.361273696	24.4721203	6.050061996	84.71729188
25.41415505	0.07876848	14.37156825	0.432897664	19.23698488	46.13138873	3.421792953	44.63413282
15.96293962	0.094350887	4.690077714	0.501642891	5.014391171	11.08237942	10.57532512	25.40903859
27.47662181	0.143923672	19.52321996	1.479940823	15.84658489	51.07549639	3.589525089	96.07676866
12.85579513	0.054410061	4.512150319	0.219742204	3.570328451	17.14903751	3.921599835	35.90790184
12.84273925	0.055416888	7.302258924	1.630185682	10.82315667	32.65992055	3.739886009	54.58567824
21.72718443	0.055711698	9.65549471	1.506260354	3.321858594	14.59588	5.375849275	48.73902656
21.67761383	0.112632897	12.25981809	1.220322343	10.32949126	29.2069132	7.222558142	66.52374338
21.4968693	0.162506018	11.62010021	2.193509178	12.5995892	31.33980264	7.791791208	80.65472615
18.02504647	0.112814738	7.029574208	1.897307716	9.672187274	12.30164749	8.168736797	53.03179542
30.19759615	0.177616634	20.93294349	1.770954957	19.35213495	52.47505951	8.700726084	87.39847185
35.2596265	0.071437679	11.34948346	1.930791779	4.353616069	19.49078812	9.401753686	65.36561809
24.12366667	0.098938698	12.26923955	0.968849899	23.89633353	49.27576975	4.94813172	69.37874578
26.62985499	0.09954663	12.26402811	1.167469056	13.63408743	34.22755996	5.490624306	58.53823118
24.11997313	0.051458332	21.13197283	1.547336492	12.12976142	59.37915203	5.686809204	87.38429902
25.15448659	0.082108886	8.454643634	1.203111107	6.842345691	25.29243554	5.839716083	68.3296511
27.75450867	0.107083439	13.23413587	1.415568272	13.55741163	42.78612561	5.082647609	87.69505455
57.01313634	0.055075352	22.07195984	2.69833226	14.71250763	57.15498286	9.364340376	102.1377753
30.90417403	0.107231854	12.80681462	1.822014392	23.73093922	71.8980255	5.298210645	77.62412879
31.17757057	0.190078017	17.60879327	1.737551244	22.61056147	58.75695689	5.43549527	81.88699817
18.88493368	0.067026523	17.08192262	1.799520758	16.54668894	55.58054575	2.313528611	81.17620091
20.34755379	0.101805039	11.99573484	1.189695883	17.19267029	36.73049923	3.39064496	68.69304005
18.39385296	0.062030934	6.636031127	0.336975437	3.54636386	21.81592581	6.171019949	39.46143526
31.13507327	0.159232997	17.00153933	1.429041202	15.94942694	44.83370825	6.00011415	102.8183291

Tozasertib_1096	PF-4708671_1129	PRIMA-1MET_1131	Erlotinib_1168	Niraparib_1177	MK-1775_1179	Dinaciclib_1180	Gemcitabine_1190
24.29428014	93.77109304	259.0206893	10.65210716	73.48508527	2.783116792	0.076362402	2.335742652
14.77974005	67.79393541	88.60371975	24.56290614	85.11395907	2.936413991	0.044673695	0.943524801
19.06967882	67.2140919	107.4536402	17.63358233	93.59195655	1.978400542	0.151319298	0.755504732
11.47794634	96.51438138	51.10695303	13.80797956	46.75471142	3.066502445	0.076394883	0.279727636
36.80162865	79.70484092	136.7098233	23.35550459	202.4183192	2.669036897	0.1538537	5.253993807
12.91067842	67.37699655	37.44725912	15.56153712	31.48914835	1.3370051	0.038074346	0.191592919
20.54212601	35.78516857	79.24166274	16.39892482	55.94933463	1.117483832	0.0681344	0.14134423
19.56015222	35.0800678	135.1927062	17.80516612	139.6240392	4.912416406	0.057764746	1.27398815
33.13189228	56.29070554	123.8086475	29.57726662	143.7664934	9.156167729	0.207479031	2.233925802
13.02976445	33.4828292	47.11499202	11.29566807	40.59991783	1.17818783	0.03849915	0.210452956
16.93563594	47.04323269	64.18871118	14.4193996	46.04317737	1.254513544	0.051318027	0.378118818
14.50673239	48.37883718	133.1710869	12.38275494	79.41216711	1.809051588	0.052801535	0.758440956
20.94146131	43.84740303	131.0779329	7.096692357	96.04307652	0.669804769	0.045743996	0.207301537
12.02502544	97.35836696	171.8865376	11.77555721	106.5713142	5.930121836	0.075366289	1.084053022
25.38754299	47.08431958	51.37120437	15.98621287	86.8114131	1.516827308	0.083246314	0.385298369
13.72418712	40.3031398	71.45741383	19.82984915	80.1979375	4.162975979	0.070943323	1.473683599
14.33318755	78.04968616	68.9656435	13.05574838	48.08889988	0.391067478	0.052579492	0.405484198
15.18701184	45.36278001	293.1964596	15.29153929	125.2478911	2.182951075	0.091400301	0.392692106
23.32128842	66.29293805	100.2951141	18.05534405	136.208007	0.825067378	0.136682603	0.434389263
12.45408411	64.68806813	36.29127494	16.03635646	47.972037	0.575101242	0.038353533	0.052051294
13.89852613	39.44188229	70.06522312	21.97170931	55.91485078	2.478645603	0.050099707	0.307598683
27.23418216	73.51863577	141.1077931	34.5273164	126.2893529	6.875189789	0.142993517	1.857152236
23.37163775	36.86724882	60.58482046	14.31138342	54.71826251	1.31169008	0.047862543	0.159961145
25.04744773	38.0302326	151.7502984	12.50769013	117.0925655	4.155877146	0.151225541	0.605242708
17.96407305	51.91743134	80.65861801	15.34993651	81.81694607	2.414566918	0.061740772	0.68224412
17.78961099	40.46300641	98.87602522	17.34091403	123.107811	2.251430326	0.051306488	1.072446312
23.50463368	47.85986134	103.0970566	14.39549432	126.0958061	4.966167539	0.139430537	2.692817521
25.18465534	40.97075844	90.97871251	13.85346984	104.7015878	3.111493522	0.056674018	0.486591807
19.16083367	46.92823949	35.06571726	12.76614271	61.89514001	1.447019235	0.049729768	0.19396757
22.00648702	46.99470699	82.02117828	11.58395738	59.47915858	1.112812202	0.044613815	0.192249518
11.54987208	34.62361561	75.08282102	14.87711951	80.56335122	1.056825613	0.047655894	0.339661212
19.9760266	54.48984747	130.088709	8.221548252	132.5133424	1.082445956	0.11330152	0.557100656
20.90286026	55.6402081	143.126791	17.133253	253.8427215	2.836142001	0.106807447	6.375109323
10.70003475	31.84788758	127.991874	11.87658337	65.20243058	0.996973852	0.041692326	0.361322368

29.4200864	27.57966948	114.0788581	13.56524674	94.58258835	5.540126869	0.079040297	1.7572663
24.89038399	57.09049019	94.67539561	24.44008195	172.2797766	3.318837121	0.094853487	0.541596397
23.8945071	88.09112016	53.59363604	7.514433751	67.98975052	0.593843589	0.057733687	0.154721598
20.57822234	63.89305503	235.53607	10.38840222	209.0431814	5.311168809	0.108533984	1.211436645
20.87563211	27.41125543	119.0937574	15.77590588	74.80819043	2.152101003	0.088421121	1.098525002
13.87275984	85.96621309	72.22146679	13.29406979	136.4394494	0.611411771	0.04780514	2.704154661
24.993069	78.63905137	222.3861613	5.676493683	130.3841126	1.538990744	0.05552717	0.870395566
14.75430909	39.1688441	74.82477885	16.02220106	87.22265823	3.193300583	0.055288344	0.515915726
24.08829531	61.86590645	137.1764138	7.801995235	67.79402125	3.592114331	0.05754946	0.835781161
16.72221879	35.86683946	73.80256726	12.80361045	63.58098533	3.704768166	0.056680524	2.17315081
29.10622075	37.49754477	110.1640175	11.32314653	48.37431505	2.255323416	0.07860834	0.180801489
4.512545242	103.6380228	40.88211452	7.144631495	92.91438784	0.235119749	0.027239945	8.761062868
18.50922234	33.27632392	38.64853126	16.15908608	45.49746159	1.401083176	0.059951894	0.121595377
12.04982191	56.94850898	75.50441436	21.3457331	82.78580736	1.92086398	0.053972479	0.495311933
17.91359792	46.25560778	37.16424065	14.86768885	35.69831862	1.964858475	0.042287872	0.207124469
18.79750739	37.17554616	81.23089358	9.65841705	75.39331196	1.165716078	0.039505609	0.345732247
13.86320166	36.06373404	27.09225487	14.00793416	19.56541262	0.911814951	0.034611563	0.063229722
15.22951316	47.45883978	58.92841753	14.36074957	95.37540124	1.321481485	0.078264014	0.266681374
15.7460861	39.05989568	70.30085905	12.57337225	113.8292298	1.481917404	0.05707955	0.836730688
11.39171702	48.81357315	81.97347599	15.0751161	77.55335351	0.625556611	0.054520325	0.059498123
15.14405532	39.01890964	85.03599854	11.14097218	54.24429794	2.345282107	0.04911498	0.047608668
35.3283963	57.49684722	139.9179671	11.51970062	97.30497207	3.562294314	0.063979144	0.118191819
11.83289936	32.2778504	152.2283528	12.51441416	65.27535536	0.543374305	0.032247101	0.108566922
12.00118674	35.53074078	47.03508767	14.88908925	45.27073433	0.977343338	0.046566068	0.278496836
53.22610295	87.13081035	274.751447	6.961822794	107.4567939	7.097122047	0.111522165	0.982377737
15.36508132	41.52908764	153.835291	10.54950075	107.4616498	1.12161304	0.083145607	0.477433863
22.00792275	62.64508984	331.7606929	21.19826336	169.9802459	10.06023756	0.086863861	7.8305611
17.02169543	59.75720573	81.9065124	14.75723866	65.9033696	2.061790605	0.043741222	0.898438014
21.00077865	51.13389531	166.8825709	17.70643216	205.8467508	4.389743517	0.101199458	7.690201705
15.83855875	51.4962593	165.4597384	13.13982281	105.3233959	1.324212196	0.0339069	0.790952309
17.0008935	35.05934933	32.78531272	16.68259211	31.28984565	1.731687581	0.058532611	0.120441687
15.65694743	33.48845396	49.77881233	9.487648846	38.96060646	1.146702657	0.046009425	0.091089042
30.40194315	90.85096725	235.2556296	2.450362159	131.821255	0.940288711	0.051104113	0.886926605
49.90253065	44.33674275	252.9483294	18.06740195	287.7042924	15.13536081	0.405123692	3.415598987
16.72639894	36.6186581	113.1504667	14.38594879	180.8668235	1.221733563	0.075571366	0.39482179
16.62027778	31.30703795	108.7571788	8.208785772	57.87959379	0.57162543	0.046631355	0.239618681

18.36317824	40.47845572	103.6686231	15.96229045	193.1692093	0.97012138	0.116656198	0.318843782
11.04145828	36.32356344	133.7714688	5.609194184	59.83932232	1.161632423	0.035988436	0.633674281
17.20074082	33.75229683	63.04140168	10.87762956	124.2223302	2.238776747	0.07837348	0.515381383
45.77679316	131.4616902	245.4205135	23.22980374	172.0483274	6.451270582	0.119720354	3.83243167
23.65802492	34.14534441	63.55599348	10.75733742	72.06029286	1.586405485	0.042993633	0.434810516
16.80939834	33.84880308	41.5627923	9.676960437	36.11841298	1.53102508	0.037992449	0.108029324
23.09471373	48.85706325	63.01916104	7.90345897	41.49181296	1.038700473	0.032394398	0.353076919
25.9175385	50.66920554	103.0006122	10.57489116	58.49455826	3.380703664	0.064366523	0.430779233
18.72954559	66.35293744	70.91189114	15.06810613	114.0849982	1.840877995	0.07218857	2.027305335
14.93212541	35.83640797	102.9527522	10.83076619	86.53579672	2.213952882	0.045744497	1.029266603
30.40772945	63.18549004	118.1981093	9.25663852	162.3811054	2.151111322	0.083546209	0.170634926
21.57469248	37.7016313	281.1661611	11.54938095	204.9023124	6.967622246	0.126286121	4.932103534
14.04868002	24.43162505	40.10336917	14.98103509	48.8134399	1.310186639	0.0540464	0.245324055
12.07749967	30.33359809	47.72209241	11.04265327	38.4307048	1.384026056	0.039944592	0.085172783
19.62792521	55.4394632	68.14865026	21.60205289	84.98530077	5.612871052	0.101249925	1.17996113
14.12470444	38.87041456	60.43685704	19.96191192	70.12517039	2.089918033	0.085751918	0.675469151
5.592188782	39.68838187	91.02807157	11.68581451	57.77864711	0.573390966	0.085406225	0.165620823
15.53980502	23.66418587	86.7562432	21.17023781	38.93998288	2.145691624	0.042931632	0.488876684
23.23211125	76.02819027	148.3726267	19.840673	153.5667804	1.733461622	0.063165313	0.597305141
23.03816236	66.04414779	70.59562102	15.89839729	60.0976656	0.695128873	0.041506388	0.116414548
29.0718632	46.05366847	157.5743198	20.995349	76.75878891	1.517986106	0.059861388	0.61297975
10.7325919	25.77094552	58.72165127	18.10164238	34.06093674	1.512227077	0.046489673	0.12529181
33.36884343	43.66406588	79.03335985	13.78388604	72.65160196	2.572319117	0.111634042	0.260148762
12.76737136	31.65919565	76.5834851	9.860261569	49.53358164	1.573708154	0.036669117	0.550573216
21.35669519	37.15915383	54.26407196	17.02784886	41.80186913	1.394460265	0.039895109	0.246336412
9.961091706	34.96956208	70.98364592	13.72196947	54.67703117	2.809346035	0.046416964	0.218048268
18.7865778	43.98811878	64.5634413	12.8466783	48.33462633	2.339159572	0.058386672	0.379094611
18.83587067	70.11277037	66.70351333	23.5458546	63.82779118	1.616407369	0.068501411	0.121722995
19.2236022	42.34358452	104.0359897	11.07214407	71.49028616	3.551244992	0.076644383	3.276876272
15.73724262	67.508808	76.38576813	5.542747028	59.01273223	2.718409344	0.063178663	0.594954615
19.25485938	49.49447208	177.0604453	29.68071413	151.1941331	4.27932821	0.072771606	1.668743828
22.61197197	35.37137468	81.79330688	10.80638483	86.48461658	2.673301105	0.094380381	0.967612278
6.323222097	71.26445925	43.5982115	17.27726411	33.78804207	0.365679367	0.020272168	0.582908029
17.93035921	28.62294433	51.36009762	16.56777272	32.60501369	2.607616755	0.057801181	0.36640142
32.71798006	105.4813249	185.0598369	7.597947261	114.365015	0.474404995	0.036495556	0.111742969
24.34411789	38.11276796	106.6680302	14.48617425	106.7899286	1.601529242	0.097135498	0.21085734

14.99396868	38.78529148	98.31830658	15.79299151	77.82698393	5.654723397	0.061310484	1.10681858
12.84487812	35.08632669	115.7517236	14.81508485	81.7117863	2.719141062	0.069445082	1.628434521
13.27206351	24.87470915	41.83776163	12.45852758	44.25956482	1.060512472	0.049337836	0.146212078
37.36388335	84.07751377	226.4619217	16.97630061	119.4859095	5.876804725	0.092214916	5.300543204
14.15847176	42.6112462	128.1807359	8.420725779	94.10104561	2.122458934	0.046873177	2.814497439
24.90175408	54.8737353	93.03859843	8.901344632	76.90954181	0.924978079	0.052787427	0.130572788
20.81405701	58.41515781	210.9966521	12.69132301	184.4356393	4.817679677	0.128995999	2.270936466
19.84806244	40.25637372	56.11840566	23.00754903	56.25977469	1.525464015	0.055943263	0.65384786
18.55718751	38.63268261	189.7954554	13.24890408	102.9552147	6.958234696	0.068404702	1.085590636
22.00302369	36.83147196	79.1262138	14.31806802	76.5557249	1.330256557	0.062873923	0.765126581
16.61026311	68.5253242	48.37715725	8.19889963	50.6756606	2.30662883	0.052705711	0.267011737
22.29289983	39.38768086	134.8265048	16.75145897	117.0902831	6.138858744	0.091616749	0.59639232
22.18729999	50.66539589	116.1562823	22.93838411	113.7006714	3.187937783	0.085039323	0.631150672
17.04107536	33.41258585	38.1141295	18.38275975	43.39520123	1.529767627	0.044677318	0.262248656
3.92431346	104.1560885	40.70991039	22.43838167	26.03208993	0.649152925	0.041644509	0.684836017
23.63912875	39.62504378	144.4471588	18.04845668	111.1424427	0.942043286	0.079063911	0.913659946
21.65868327	47.32077283	64.3515972	8.562407793	95.07408371	1.18019288	0.054762235	1.51563081
24.01189834	67.94187891	148.4853037	11.8075363	123.9114531	0.682120661	0.049540958	0.21053107
18.35677206	69.94932101	43.90583387	16.42029293	65.73158663	0.380687631	0.038279149	0.217334661
21.82868457	41.26322194	100.6170307	15.07821395	104.2160936	2.331965129	0.058590164	1.079173616
24.98660444	35.42508019	294.2976721	14.79409914	151.3627255	2.226626873	0.079007389	0.948551859
13.45438562	35.64498327	52.45364321	11.56974031	74.28153867	1.703799813	0.05410998	0.350596008
13.62449169	44.66338176	63.11164969	10.84641741	73.03282818	1.42415509	0.048166125	0.276802033
25.39641606	80.90485772	179.9071529	12.16703663	106.6712869	1.029946339	0.073499718	0.669016735
16.52281045	72.00297526	54.51986855	10.98438284	52.07410657	2.579503229	0.070904462	1.263836291
16.3806313	82.92381065	110.1898187	12.20314045	108.2818486	1.141263446	0.066600385	0.390990787
47.01802963	46.03992912	251.0778744	17.44529705	144.5517477	8.311320513	0.185984874	10.91902617
15.36695492	34.27113168	55.37376624	11.49935312	57.79011555	1.900314182	0.05028924	0.213305901
19.243486	34.61878918	82.404694	11.91278172	60.08890838	3.012958708	0.045557664	0.527938889
16.04421754	90.65521791	250.9054268	5.244483993	110.2402462	4.650159953	0.073606282	2.598860969
25.73775638	67.93193226	127.4658761	17.18142044	172.0302272	1.397422731	0.069305367	0.448647434
10.71817498	32.67628326	86.69619138	27.53352181	99.51705704	2.471813666	0.076633131	0.559449768
12.62136821	69.80241671	68.78381992	14.73229265	37.48581141	1.861679939	0.0526372	0.202288739
19.88945132	51.35679608	108.4995274	11.94686416	68.02894678	1.32835758	0.080330108	0.22314403
10.39134919	29.24521522	65.14888996	16.06470083	84.26996581	1.97758263	0.05431548	0.449581615
27.88749886	66.27562905	101.3815514	12.85925909	132.1556699	3.056265565	0.104035657	1.400408605

19.13601015	44.66291228	133.3617899	23.5167986	96.66469231	2.643613177	0.074131881	0.313294583
18.2420447	53.84751349	75.68281136	14.00936383	117.3294683	3.358770954	0.078887335	1.27157365
20.42476994	71.80805144	89.14643284	9.046704229	113.675293	2.773833899	0.055981197	2.807320131
23.92775753	38.99575284	77.80082496	15.8324676	59.27776029	3.928149693	0.071877733	0.363967641
49.81651137	123.5617721	858.1905306	15.82382085	405.0902222	1.668510983	0.16705419	1.625132391
15.82472227	35.00762104	65.1114991	10.28404738	59.8597859	1.282257421	0.056036485	0.104202439
19.29395985	43.19827455	171.7906413	23.13175479	92.05346505	3.179631845	0.086589969	0.496430447
22.13633178	40.2691096	46.88357663	14.0581638	57.7514076	1.695058991	0.063761727	0.098444644
25.25104335	29.19649446	58.76349482	16.85679299	43.54694693	1.369238918	0.093545391	0.1253159
14.43837683	34.58033261	191.7531243	26.4852127	71.75253473	1.038515268	0.057396532	0.874881789
30.29857231	45.25546881	100.9003918	29.53255275	121.3072094	5.251632742	0.105602324	1.817716291
27.73539561	47.79728845	108.3288521	10.92893338	69.57087088	1.931968918	0.083823257	0.380569376
20.23118628	47.67211318	85.8856993	23.5419248	79.59711299	0.959583022	0.045713453	0.578790633
35.84422798	95.2476272	89.35911614	16.07384716	61.33375745	4.325861105	0.105342038	1.430183955
13.06769625	34.96069261	58.03800154	10.69607227	37.49347376	1.023100728	0.048310902	0.179808865
24.14351692	55.86514655	161.2081761	12.10322124	99.71687333	1.880521832	0.079961855	1.19617645
13.63440274	76.88964391	187.3869137	3.435505103	80.1836146	0.164563925	0.021881234	0.029257165
16.63370021	35.84765915	203.9172518	26.19185368	142.7665508	3.162186474	0.103319167	4.543882293
14.85073728	33.84518731	82.54988024	14.73162543	78.76929178	1.99544825	0.077544979	0.695285927
12.01502611	30.59211137	72.3363219	11.96836532	38.32017462	1.335748773	0.046993113	0.175390177
22.9081595	45.4468366	193.4346377	7.442640264	120.7333075	1.423646758	0.053388371	3.352911654
16.33111872	33.59031143	65.99366219	23.35520983	61.87977015	2.263411765	0.049280497	0.571895654
32.50298448	37.08302878	69.67568202	9.919024778	67.33559496	2.191420652	0.122606358	0.223542538
20.6441778	38.6970789	69.53739515	11.4914148	61.42831412	3.121059195	0.047222635	0.402320894
23.90403604	66.62476233	90.02447532	13.51762554	116.2426797	2.213410613	0.040231688	1.768421619
28.66164714	32.92668481	142.8517662	20.77799437	107.5131358	2.832638357	0.090720648	2.517358432
11.41897909	37.49207213	96.62313701	17.09233512	54.02937378	1.574579479	0.036399966	0.712250828
33.16788255	56.60416177	281.7728404	6.249027365	96.93322948	1.442525038	0.063964437	0.940552729
21.13137562	40.17044887	89.53971614	10.11220229	57.21773784	0.941061672	0.044218202	0.128811974
18.28595529	42.18585831	87.82715657	11.8129074	49.6299411	0.870093448	0.038761525	0.210443335
26.00626117	34.49260904	36.87444508	17.77984619	34.48632851	1.658884306	0.05276794	0.326327285
32.5897933	51.51661976	103.1174595	7.949363023	50.65779241	2.158475415	0.040797976	1.256202042
16.47250615	32.72772954	49.04842788	13.16624051	43.48653625	1.874849319	0.058430242	0.180631111
31.36233323	70.0646371	193.3351908	8.958223389	143.808536	3.874010922	0.134693884	2.390755077
20.65055387	46.85624489	91.35339268	13.77714269	66.87876201	1.979289289	0.087788528	0.147093586
11.97022429	39.05341549	115.4304871	15.20886448	86.46289789	1.394485576	0.046009742	1.125908935

15.6495497	34.83511222	34.65851408	17.76031191	28.8657226	1.461665306	0.044510517	0.298117253
17.5275056	92.49814078	161.5165181	31.26092175	111.6081292	0.592206824	0.134702495	0.324589955
16.99139038	32.80723944	62.17248403	13.38562804	54.89841763	3.53702642	0.04301904	0.166857097
20.15440089	39.6486976	159.1143845	18.68801595	172.3977954	2.349171812	0.103818888	0.289057073
18.36874614	35.63469166	50.86270953	17.77677436	65.7603829	2.360037257	0.074142068	0.419292726
28.21581753	36.43236122	342.4160902	14.72865192	149.889008	11.8646553	0.102198844	2.665473242
21.7374203	55.47965675	75.66677172	20.45269289	75.79249835	5.113163714	0.091959908	1.877714037
11.36277011	58.69987474	297.2466242	16.91636074	138.100438	0.68290051	0.059280409	2.800429468
13.14984647	27.93917495	53.92419652	17.65152363	41.18804226	1.689383725	0.041431984	0.361664883
32.72972382	42.98246711	89.01077036	10.41240249	63.66019279	4.250149309	0.063042744	0.519558253
20.54361055	44.14077022	82.42162825	7.135992324	46.06269256	1.384374252	0.038361873	0.126404152
8.580134707	32.45721086	82.700102	12.61205288	76.87940864	1.926909621	0.062919944	0.647527743
23.3253752	70.87038957	52.69333846	17.85825609	111.6360285	1.918032148	0.052144984	2.480236214
24.34990583	37.65311786	53.02546191	12.84311145	67.04126347	2.192581411	0.089395277	0.220215616
6.46808693	33.29168043	90.15629637	15.86015468	60.98762499	0.532433195	0.101068505	1.327134773
17.51388141	43.2107688	52.58624666	9.746918848	52.91150026	1.924395924	0.041938339	0.102512757
11.39870193	35.34879697	73.33933482	16.19950426	69.17768931	2.203533764	0.037206292	0.613094909
21.08290547	30.84386748	63.70542551	24.97469092	65.97462152	4.383766408	0.088758612	0.46523487
19.14834787	42.15154802	49.78883292	12.4299609	49.81925676	1.098500654	0.036166797	0.207452273
18.14275183	57.33904141	78.23501683	12.39112385	63.09691492	1.177520801	0.029998564	0.503370039
17.98575319	28.27113841	86.25211905	16.24124655	71.79503674	1.220190448	0.051859106	0.325864106
25.29753245	79.47655256	213.1039674	17.81804746	179.0780409	8.159025662	0.103374696	5.529364329
17.49949879	35.79956064	41.50410119	7.503216891	53.15625764	1.089599533	0.098910224	0.281167599
21.75544813	41.51335807	51.22707721	13.31601265	53.16840511	2.109800524	0.046012234	0.284937509
24.22133599	58.4021634	330.5329912	15.08458607	199.2921049	9.841777808	0.080039302	2.021552827
22.20000789	43.12154422	71.22817358	17.94927024	90.54238568	1.516253021	0.079380743	0.453658879
36.21675242	106.3667397	163.5834739	11.76719505	109.9970746	1.969066077	0.083423162	2.485034104
3.171444145	55.45898918	76.29315295	11.18842683	27.0904007	0.280404777	0.033525237	0.103028036
9.735038541	36.6583614	33.43994212	14.66432445	26.95982854	1.004700256	0.040139247	0.167694126
22.4447882	47.20026676	64.26588064	12.89978269	68.29019518	1.433000705	0.045584753	0.28709348
10.43998743	46.66804187	44.03499358	13.56991386	58.88721325	0.82549561	0.049192172	0.199793711
16.03347739	55.82934468	62.23577677	14.0167747	62.06867963	2.130565122	0.052706561	0.521283587
22.87903822	42.35801267	241.337117	9.749793322	108.0614567	3.102341337	0.051644902	3.51133085
11.61218048	34.16587451	68.50429934	13.70022719	49.84493066	1.399494495	0.04299369	0.406040146
32.14050202	44.48250891	213.6429539	9.620579721	80.3275408	3.62007235	0.081401827	1.715047572
9.987426862	44.74479568	99.03798026	24.15770404	65.69124199	1.321097278	0.056924787	1.890801717

12.07046763	40.4926452	96.81663631	22.42588775	123.5213376	2.504969433	0.094930218	1.428638366
16.97415257	57.10276973	140.1848342	15.81944726	189.9658027	1.738924952	0.045303012	4.266558215
30.66575843	61.79790942	32.06899861	23.70820044	91.02555412	1.437623362	0.061357036	0.388911859
22.59483636	60.54090075	115.6131053	14.51247336	100.3502581	3.677294022	0.075338962	0.251941264
11.82474153	55.41244816	48.88568145	11.32972858	34.11918143	0.951696212	0.029768377	0.216734395
18.20677681	44.71897225	34.60588462	14.7980914	37.06798735	1.587037799	0.037731491	0.101664042
15.33386005	59.72656445	74.60471764	14.15461548	98.75071354	1.66949393	0.056072101	1.174701657
27.786029	54.44864187	252.5949414	7.144009718	145.076722	1.617745594	0.047711223	0.714748041
19.64830326	39.52313383	158.6851615	6.809806535	67.21675241	2.142906625	0.052754582	0.422047639
22.95791654	35.84211395	104.4776529	10.03591921	71.56525719	5.40609539	0.057752849	0.364378872
14.25634291	84.83891189	277.6630242	7.785294302	164.0685654	2.180853526	0.057416923	4.320608869
18.94572669	33.54156688	123.2365057	8.931627065	133.3248967	3.066477612	0.080121878	0.833061952
20.59308833	55.21444246	87.83254888	14.53770093	124.6190676	2.386479576	0.131150969	0.80880282
11.78686443	40.95506502	131.1916392	15.81710669	81.9859734	1.36378057	0.090518218	1.891018242
7.492782253	36.26055269	34.44553819	11.92268269	24.64144081	0.872883368	0.042701784	0.229756304
16.38603159	45.16913629	107.4446161	22.69145417	83.77676973	2.316293284	0.114298849	0.422165563
20.09808861	41.92338075	41.81766444	13.33637505	41.23812485	0.700133561	0.052029647	0.201898805
18.49781597	46.17746395	160.1974249	16.7599513	89.56468245	1.244768168	0.058630944	2.212532978
10.52648769	36.7025663	112.5632655	14.11274708	81.68665589	2.96326911	0.045657824	0.934485176
19.07196825	29.08073227	75.84164376	15.9751706	67.75021315	2.26921412	0.058414951	0.15230496
24.40536317	62.72648735	88.33073964	12.91464549	45.45619621	1.590784058	0.057126014	0.43169529
30.32797436	39.19456058	48.00779048	12.20723454	57.48346568	2.936235883	0.066916137	0.434119488
33.38454391	131.5519611	151.9449097	13.88192749	152.3509444	0.591082984	0.063241082	0.770644165
17.95886886	43.51369634	87.31133116	19.78498433	85.9839536	3.828885863	0.070555997	1.128960979
21.44560886	44.01996795	59.13348526	13.13535445	42.85063235	1.263376256	0.050849618	0.223040421
22.99084667	46.8271017	225.3861076	20.13814666	164.1810071	8.432075613	0.155332091	3.713445818
15.7328751	36.62442308	55.5543903	14.8761337	33.64109852	1.305450155	0.082107815	0.435920677
10.58207866	39.01896754	148.1208511	16.69625505	122.0479356	2.400603353	0.048465957	1.071079474
10.80566802	32.00978298	67.78113769	16.30218095	42.7389235	1.433798645	0.036163219	0.36604489
16.62709382	43.12937569	57.31257146	16.65246754	69.91020961	1.572422383	0.054792165	0.197155916
26.87579165	44.44981542	133.2564596	16.3312403	135.9703857	4.307616385	0.062012021	2.359886361
14.68677071	44.70705589	69.09474902	12.03269511	32.06727357	0.740000478	0.038891073	0.038511543
13.91119455	41.10310073	40.27818528	13.88118734	25.39305447	1.14483561	0.046769208	0.125503935
14.44082353	31.51091666	43.44301921	17.91412638	39.5292912	2.399111755	0.053944587	0.314406779
16.72303882	62.64348875	44.00037038	15.48616653	76.40981549	0.66626331	0.039434583	0.197808508
33.13237821	50.70448981	80.26894004	11.97889379	74.79992338	1.496924085	0.10565536	0.417486836

20.73014165	42.37987767	61.13600832	14.78386309	40.12249738	1.82697601	0.038053111	0.125623653
15.80774181	48.4648373	145.9207631	10.20720572	133.7881952	3.731270296	0.077188998	1.722717376
22.75044355	61.6489402	65.77145068	20.32144497	80.76314246	1.344606375	0.095957206	0.366822303
16.04896573	37.72836786	69.19715994	29.30215399	76.75545355	2.157527481	0.108869592	0.685772804
19.65170007	33.86406998	76.2830413	14.71176329	73.03432844	1.747658123	0.062445477	0.353747867
19.93913045	39.23324826	66.01176872	15.60198969	76.46091327	4.422127766	0.080927129	1.331747631
21.90751128	37.50884083	56.43662351	11.17978541	72.63623518	1.136154254	0.059034256	0.785658915
16.02995809	46.30078845	62.20358242	12.91248505	77.13065398	1.848860628	0.043143185	0.330818656
23.27417705	82.94165755	146.8600619	12.61053871	145.4694261	1.313906175	0.059017308	0.147061246
32.07023154	54.86391483	325.5265602	26.36749362	255.2585378	7.802382473	0.213689946	20.09585184
18.87998478	47.49299161	33.22936642	14.83469077	56.3249608	1.063007471	0.065456243	0.14654181
20.88492734	46.15619942	75.24860006	16.9974045	91.5301331	2.483133748	0.117406601	0.704894054
2.553694061	34.6170605	107.6404924	15.53960925	53.06872806	0.54668538	0.073818493	0.38569452
24.11219199	57.22914945	137.1893987	17.5810476	124.319894	4.684059506	0.090681168	1.846763394
14.40163391	27.61284011	121.6780105	17.29383003	68.86571897	1.565273825	0.100551187	0.786908973
14.22668571	44.56180447	41.15899016	10.16806234	58.3356233	0.543836072	0.032715065	0.077348663
18.28256304	41.87850463	52.7187675	16.38773801	63.75864151	1.602509491	0.037944771	0.258224803
15.19657483	41.13132617	113.4842764	13.68922211	78.18899775	2.178499159	0.048811501	1.449090333
14.64867776	55.9364021	188.1803152	17.81664629	105.2020122	1.370592911	0.058613851	0.949026078
29.5140182	63.21303361	208.172654	7.169944359	75.90582007	2.024785469	0.082440388	0.906371293
18.56663858	43.9500136	100.1424126	15.03987338	103.9291656	1.656340714	0.079900844	0.152716038
14.77641936	29.57118647	73.26721123	18.3309524	78.64621422	2.563061021	0.054397799	1.136353129
12.01797509	163.1208178	100.2570529	37.73246565	124.7869494	0.817543725	0.073638933	0.32940569
12.65648365	42.01571872	133.2742848	11.44530334	87.24337909	1.290019775	0.055182572	1.044303107
17.05709578	48.46300067	39.52685225	5.337287627	42.29631839	0.463827867	0.036144232	0.058670612
24.07169936	32.84695675	236.5883647	12.66122926	123.8395805	1.785100205	0.086189222	0.315406143
20.62325937	50.8616447	52.85518847	16.62283339	83.10411587	1.21712687	0.071376241	0.193174321
22.84003258	44.33599736	150.6487799	20.50662866	118.1232853	3.379967073	0.084776254	1.155450778
23.62368396	43.166363	42.32171085	14.39685649	41.16192483	1.503038301	0.052432079	0.135910817
11.85519024	28.74161878	55.28930664	15.75146146	32.84760017	1.503226435	0.047856195	0.217496298
30.60955371	102.0186685	93.60547156	18.77074938	237.7566322	1.566163566	0.098086488	1.479316649
16.30261391	43.95211503	108.2804344	17.10807834	109.4955757	3.339654262	0.054200894	2.433191451
22.6028077	51.08574641	228.6524635	15.09263788	179.5971439	2.145959273	0.12726316	0.880610094
15.5494136	35.24605887	45.34868001	11.86152124	42.22501709	2.383058259	0.050614072	0.934650224
6.37552612	83.48127328	111.4013823	16.82608856	39.17429903	0.342522982	0.032661883	0.057320659
17.72947398	34.5302064	39.29559646	13.47043979	31.30313599	2.105524525	0.046326063	0.111010566

20.56623527	35.82167471	79.04956107	18.34922855	63.10051228	3.893425563	0.053042438	1.431195424
19.07718913	55.61830876	77.79202347	11.56698053	70.11002407	1.433247529	0.043142265	0.550702906
19.51532114	44.69687594	66.02619508	14.95847177	103.0924758	3.185908122	0.055929908	0.326512354
35.25483769	56.77897826	112.3279687	24.02881501	82.42373724	3.420064874	0.085905839	0.554139113
13.16114748	41.5043929	47.54438986	11.09231224	26.840583	1.7710619	0.048514321	0.134335198
17.42412251	40.74199758	41.77362829	18.77538877	37.66408695	1.270994606	0.036510544	0.342199424
13.14047123	53.21062595	136.5092966	21.12540565	102.1515884	4.189677141	0.057743641	0.880076958
17.80567527	28.87900088	90.69146173	11.8690528	51.42348981	1.802710362	0.070469122	0.334455016
12.78265209	55.81758855	122.628738	12.94706623	99.71802107	2.251228078	0.090000703	0.75159386
15.40779505	31.86392111	51.51953105	13.65301975	26.60768715	1.267237964	0.04501975	0.16034945
14.61045479	35.86565083	195.8722047	11.81569741	80.29441189	3.02210615	0.040464553	0.183941597
8.232245318	26.58934768	36.99876565	14.77043571	41.60540487	1.012536561	0.033399547	0.145129282
22.9615683	39.38731176	67.04287298	18.77617054	44.10482126	2.392686089	0.065731642	0.399360072
17.53870332	26.10265002	183.7575876	12.16437878	99.65722911	6.394021069	0.060065299	0.578653567
9.499635183	65.97541967	94.41824839	10.16427012	62.91423362	0.558428402	0.037317709	3.761897671
5.008683991	50.96880199	178.4594537	13.65693812	50.69485561	0.556305371	0.02726342	0.271086076
13.0672593	28.27371273	55.53640492	9.34370924	53.93590142	1.53425836	0.049781608	0.151053569
9.800530364	52.68218602	76.58555501	17.6578002	64.31622583	2.062866155	0.049725691	0.595010526
34.75303629	49.30710054	166.8153756	13.896722	219.2238436	10.98828776	0.116268237	8.274835887
18.00827734	28.02922673	81.13154348	16.02069508	58.99462131	3.241999778	0.062163337	0.323679081
14.53245638	61.13915357	57.67629076	17.87715348	54.00417879	1.2730706	0.046279002	0.221009245
21.65175532	41.53596111	117.8764687	9.4827447	68.32608596	1.010754589	0.044406552	0.614571441
24.84862511	50.90623454	53.48405499	16.20572213	73.26929928	1.626617654	0.072111621	0.822861279
14.67756692	32.3032375	112.8391365	13.98473561	61.93194276	1.980156193	0.076738904	0.510521069
23.77069804	32.55742602	143.3968753	15.12546532	94.48970744	1.836567514	0.071515805	0.414654233
10.47221049	27.52030584	98.25797634	5.352519699	89.37911006	0.522398472	0.055937082	0.77069555
21.65878281	49.46566008	48.35391714	20.94974783	45.62763673	2.419591053	0.058168694	0.494524008
14.98186452	29.6151381	106.3305975	18.20306138	80.74242365	1.486181251	0.077834482	0.792341386
10.36685945	53.54218347	81.76849879	12.2325984	42.53635373	0.609965146	0.056132187	0.163836582
14.01161737	28.16603153	59.02566181	19.15469602	41.19320571	1.95769248	0.060527363	0.310249058
33.39563704	48.91312079	155.0381247	6.666582223	60.90346469	3.322548373	0.05581009	1.321663501
19.19949143	37.0081822	125.5491363	12.8249908	148.4540331	3.19976779	0.075514731	4.00248162
18.29704946	52.99070063	156.1039499	9.742954238	49.15791091	1.584490357	0.044060168	2.34401337
19.93320253	55.48479586	121.8724455	15.19838574	113.7313246	1.132779681	0.106463201	0.366946067
15.18442186	47.65453879	80.06344075	14.55732133	47.84266573	1.339471671	0.046584338	0.319548526
20.63177562	61.30775469	82.27994523	13.66913622	130.5047123	1.881515575	0.076425098	0.190778128

13.56766603	46.07791833	44.11800176	15.47882185	46.86174046	0.713041807	0.039555653	0.314596059
13.08108938	45.04428491	47.51641847	25.95650332	77.5620293	4.237097286	0.06997717	0.844541952
17.21664693	51.9412206	44.12534871	14.01576066	45.03196408	1.642710168	0.062137602	0.451088229
15.02472391	32.59965764	61.81493223	14.51192424	35.86555758	1.727897447	0.062614539	0.163442349
20.69608958	60.00294722	58.15607688	10.00140186	80.45509582	1.472091118	0.053112454	0.200465614
21.12064476	70.4155436	111.2837654	10.47301225	91.74932419	2.939163525	0.086883677	0.201165109
22.95739824	31.41145849	101.255703	19.68638122	104.46706	2.514009936	0.091026672	0.993087939
42.91235999	54.697154	135.6954073	13.41269996	172.743484	1.747704997	0.229757229	0.533872855
24.87129949	45.00275305	173.7711936	9.615707893	55.78760631	3.721891038	0.053475587	0.730459084
19.64007049	42.26106593	81.33549526	13.42999904	47.73848024	1.204767155	0.043769108	0.233382908
14.05244627	33.14610421	163.0847134	17.51179127	95.07761389	1.885660906	0.090366021	1.580034745
12.02615367	48.16584753	128.1003936	7.825373059	52.60859494	0.615408608	0.057712553	0.350018987
28.6486231	57.8947221	98.59164053	19.04449654	100.2238509	1.610787106	0.065379686	0.58041932
15.39365149	60.29362608	87.53187753	28.67008817	110.7580431	2.808962071	0.078822216	1.034082938
12.30316096	54.26108114	108.8023495	29.31091137	60.50620501	1.693526968	0.070418939	0.177211902
16.24832842	37.97025964	62.5136498	16.07328638	98.35581803	1.750506807	0.066296619	0.215016522
28.63153242	65.5841151	142.1133747	16.51304642	138.2105038	3.919271494	0.103359244	0.237592158
22.28299574	32.87903531	48.76376874	19.96657198	36.65759145	1.064045272	0.050516325	0.246773943
12.86663687	23.05402707	27.82169158	15.68840591	29.72995335	1.089703393	0.035370537	0.099222741
3.986813049	65.65915786	31.77646674	11.60443953	25.11592575	0.209788272	0.019423872	0.024173422
20.33958842	43.60915104	238.8463848	12.98001657	153.9664545	1.492161524	0.052420107	0.333587225
27.88531265	50.29171327	196.6880303	10.10810964	184.6979853	2.333026101	0.083548402	1.883054856
7.72709356	27.45930999	208.0750491	15.47110638	102.2584872	1.726112456	0.063878391	1.79715016
15.37424463	34.02844194	76.42787511	31.63528363	98.44728089	4.241431929	0.076220016	0.99122362
10.05865687	56.54667249	386.8911578	7.055965132	204.2117635	0.555480218	0.073684833	0.286752536
14.80164305	35.98761713	34.89596958	13.93313922	30.68352246	0.502442515	0.033950576	0.064199393
27.47590045	45.94085698	143.8895079	11.74599314	102.8214599	3.419076749	0.098998018	2.697598785
13.13481475	30.07268561	50.36536368	14.07325161	49.64654087	1.619638545	0.051326549	0.542265301
13.06751083	50.31103526	67.19711633	16.66207735	46.04716116	1.826847707	0.053345818	0.394527662
15.14684211	38.2193247	47.86451478	11.96316863	51.37852828	1.2010052	0.0338521	0.131262904
30.55736388	72.57537824	103.4027001	10.3737397	96.88115259	3.066401106	0.113387692	1.633178386
16.42419968	51.28939575	72.15393229	28.36878653	73.61772309	4.414426603	0.07126395	0.700232687
22.6234022	40.08137199	44.28503954	16.41256884	70.13885672	1.520309514	0.058610624	0.367669978
16.67413731	48.44956393	222.7135194	5.456773577	166.7941698	1.909045578	0.062115194	0.321184225
15.74496831	53.36179004	69.67898162	15.8452076	80.97891153	2.854102906	0.053637319	0.47171389
8.360631141	39.38963748	37.98808278	17.16933095	38.2464712	0.805397727	0.049383797	0.32668216

29.14219011	47.91407858	84.99803737	17.85966494	88.59921948	3.400439301	0.133399657	0.426312684
13.83119565	48.58856178	30.11770068	10.08868094	36.87475366	1.403337702	0.030578057	0.130463365
27.65573678	47.49035899	102.2907623	16.70933042	142.508273	3.675942945	0.096843032	1.38309333
20.04689052	49.5665475	170.4118354	15.71419141	121.8090548	1.935501075	0.121436689	1.366292442
16.52468786	38.16485525	189.2347341	15.88684924	152.6446122	2.494451125	0.080072115	1.470894025
21.95185957	80.0640125	70.99324511	18.01033668	159.6187634	1.84606453	0.098501196	0.183743731
22.98115052	42.17377186	87.7783057	18.86932889	67.92340398	3.298626742	0.074985378	0.474016394
23.91077093	52.48990147	149.4746482	11.80988026	83.29022422	1.304664773	0.078755973	0.474316788
22.16951805	38.69443008	111.5869028	15.61344144	58.10296789	2.256690236	0.050156791	1.349835635
23.12103478	37.54836036	278.9881361	15.03147032	140.8312666	3.270770342	0.164771979	2.931223917
19.51354746	42.39520707	144.2688694	12.3283776	52.57359791	2.177811933	0.052805384	0.671185483
17.17353866	57.68036923	120.5461178	14.32393528	102.3514926	2.205982921	0.049644923	2.117755334
19.34737495	47.82612177	83.8809071	18.69994655	95.24519368	1.915466954	0.090957297	0.684673189
30.91347414	44.46121509	152.4996651	13.85176091	120.2184151	4.375781076	0.076319396	0.60011371
11.09455839	33.39855625	122.7978497	17.06829693	113.9664299	2.368279791	0.058886323	2.429034571
28.62918034	39.77183896	58.77984179	14.70476517	111.7848002	1.57819312	0.08618737	0.388556621
18.83232031	30.39292899	76.60929301	13.44594492	64.76877299	2.099147685	0.05003232	0.283313192
16.2710045	39.68967409	52.4520733	14.42818936	39.56433554	1.957459441	0.067210087	0.265861217
16.05730585	34.04859537	66.72687092	20.08388357	105.6974176	3.627882105	0.070709283	0.348594414
14.35306963	38.01165664	108.1082169	16.57340546	84.51348528	3.196553786	0.133308015	3.850858313
13.23250867	39.57418939	26.95020167	22.71520913	31.82013514	1.427984267	0.039206296	0.19402515
8.845911953	38.75476973	56.69577472	13.64382194	52.45774428	1.848560201	0.044335049	0.383993955
13.52044385	46.92162849	129.7479199	7.435320648	120.960194	0.918927111	0.042095161	0.733184571
14.46092181	55.22001795	75.88658003	10.77165781	100.3888231	1.12967055	0.051565731	0.284200209
16.70612688	30.73198644	50.13570959	14.4687549	48.05777946	2.803804862	0.055444481	0.488464219
21.40515129	42.44790048	92.59483503	14.95082966	64.20444371	3.312842302	0.067109141	0.336584039
12.31509874	36.796393	25.74674783	15.72246766	18.10963864	0.929377458	0.036729946	0.092013294
20.94340684	33.04086418	39.95883787	11.15546198	28.43923129	1.479462131	0.046367296	0.170401901
8.597864655	60.8307077	121.1841905	17.6913259	101.6163184	1.251489626	0.02992729	1.504671325
20.89676466	40.03371851	85.58371103	22.12980753	44.68679525	3.260309753	0.09524162	0.34264901
22.27891273	63.08667794	48.39782137	33.87536078	65.79373063	2.845723995	0.07070523	0.182135326
15.66539225	60.68417467	151.876375	4.30540802	57.88995949	1.149965156	0.026210621	0.358438194
17.38975145	44.33254693	70.96208302	17.92869555	45.60199475	1.855534558	0.054743161	0.444360727
26.10922318	91.24050395	142.1892648	16.03006255	183.3210319	3.167933823	0.077201465	2.434072881
18.32605493	39.51090411	40.8502934	12.06439617	50.9517162	1.356695183	0.060327696	0.182195332
23.07077482	34.9632652	105.5334503	21.6801108	131.3019043	3.623796473	0.072868487	2.519136272

20.94718016	38.8952331	145.3230853	10.31522899	90.0477142	0.948306725	0.045762285	0.609499424
17.64337375	61.13828758	71.42094223	23.32505201	66.67622401	1.346984749	0.065610186	0.296509736
19.77199796	32.51576671	54.3922776	14.0800004	51.04543425	1.881993564	0.063889144	0.234777087
17.64612707	57.60528064	70.77111194	7.447050078	32.6149306	1.74505609	0.032264276	0.426548047
12.3116384	43.0296823	66.89265444	25.63012485	59.07728733	1.987754345	0.060792913	0.415881562
23.90824662	43.59242991	259.7791898	15.5256586	158.8724151	5.152776313	0.131776913	1.887332291
46.90171281	50.45546789	69.88694738	9.443252639	46.18263971	1.836911356	0.076991715	0.401916321
12.0386301	56.97137484	107.3090115	24.56102124	45.90027798	2.232408081	0.050557323	0.480432624
9.227650369	44.68461808	225.2421454	28.60637386	164.0755359	1.250692074	0.118703974	3.409396547
19.66398557	38.48065785	107.4508824	11.09702444	37.92238848	1.118505271	0.030809685	0.220068722
20.15411073	60.16437964	162.1090871	24.76926731	64.06940782	2.886426134	0.064618681	0.581219961
36.67837323	59.03982452	305.0027951	7.633107622	111.5741902	2.879295935	0.179391935	1.863947352
37.6277205	58.44661911	175.8905289	14.8890496	84.77542512	2.568019456	0.102363046	1.03028247
15.98866285	48.7796132	129.3757345	9.373065574	75.15175023	0.790096024	0.047215138	0.445843585
34.1907807	50.39701608	107.8505005	2.985175721	34.81397685	1.159706398	0.037882838	0.045486257
12.11019035	56.29618014	353.2585358	11.1129909	132.8403566	0.465400541	0.043488974	0.662230529
22.33547623	46.42378636	81.76082134	15.17290194	53.61643275	2.839091769	0.052357046	0.655806512
16.98013908	68.75370879	112.7747499	19.7426718	45.53377753	1.52270662	0.049686138	0.347296855
14.80167445	69.71573729	325.0534655	15.18938534	74.77444373	0.66755344	0.04095269	0.360398165
44.91243646	63.56586334	165.0906483	22.26144616	132.6198024	3.623115997	0.095892463	1.267539597
15.94075748	50.97919096	31.47782749	15.08401751	26.56344446	1.125607767	0.050116095	0.188849355
18.59639831	39.54772148	52.11560073	6.001640406	34.83200378	0.857735702	0.038756515	0.049861388
26.63343279	50.46147879	40.20750237	10.97433443	54.38553551	2.370372584	0.067081545	0.390128989
16.24922227	39.72864677	61.00168626	22.07144369	36.58214646	1.633946998	0.040174303	0.314381277
24.10287761	72.31190282	104.0662464	18.16604437	74.07650665	2.8641774	0.061418654	0.526488759
14.50760772	57.18283181	206.1414673	9.043508479	91.60302505	1.398081283	0.044878928	1.123991318
28.93416977	70.87630099	301.2835773	20.01620165	229.1917606	2.090983789	0.192458385	6.431274223
20.21204338	45.62197417	161.6521881	9.718553291	60.84370097	0.972826869	0.05904929	0.701385258
26.19104276	46.08705118	105.2980943	15.55243253	58.94633471	1.220221523	0.071431637	0.175219217
21.17638341	110.1335397	104.4845249	4.238332156	44.10400786	2.625349135	0.078764118	0.157107573
29.54302056	56.10760245	146.1223189	9.903871281	123.2494925	2.570727534	0.074432101	1.067224075
16.20455661	58.62318893	365.1443182	18.04088971	61.62222594	1.033030047	0.098506711	1.062049757
16.03517344	43.30388907	97.88490543	7.884202029	52.96077744	1.013132464	0.031087346	0.533388883
34.69776288	45.65730537	51.19809112	9.539600446	70.8477675	1.162378634	0.045035694	0.169022402
13.93820925	85.93057911	355.8338201	9.668035909	79.73422948	1.769951805	0.072891598	0.230980364
17.07415016	34.81053721	179.8399323	9.373003097	64.93059011	0.883267712	0.053876832	0.437102652

20.56294466	33.13177606	141.2041405	10.39478219	102.9288782	1.184154208	0.036365793	0.273839068
18.9740512	40.71214579	35.11687767	8.213548688	23.6445763	0.928013037	0.031073529	0.044120101
25.55099263	64.72240671	104.0844656	12.99338834	62.81782932	2.37688942	0.050806317	0.696298733
16.06593668	51.35953653	119.9187133	12.874559	88.50299275	1.115562098	0.058069123	1.113223418
12.52230462	42.6260997	230.5866045	9.988835272	57.25086573	1.081578444	0.03304764	1.006676152
20.03220795	50.9811419	130.4237803	19.15761329	95.34185676	4.100145396	0.083644193	1.29668631
18.04934151	57.44961653	143.9265734	5.82336062	68.1836913	1.561868959	0.036504325	0.908304457
19.89731762	44.40770962	213.6442921	6.873834319	74.37505892	1.199133801	0.042548968	0.346115392
16.17910046	45.6721597	55.06505826	9.89130225	53.82467139	0.969962742	0.040257652	0.309137962
21.98522737	56.98294575	172.0247686	11.56248192	104.6855131	3.193002629	0.096634828	3.736416743
17.61353004	72.55116516	119.1646267	21.21051657	88.80025914	0.957325531	0.066225296	1.201891359
23.22887835	42.86167085	294.2036281	3.821962716	61.06852166	0.420641592	0.038152356	0.111503634
17.00468958	103.4644338	187.0791663	8.131239115	101.9572177	0.685908749	0.035324277	0.550106477
15.55793385	70.19413078	71.95473786	16.89879761	71.04339063	1.747627393	0.073435146	0.464319309
25.16657643	81.45853429	140.5630609	5.969288184	189.6765256	1.829398702	0.056094594	0.437015543
18.19815237	45.13661537	191.1636544	10.60935306	56.96920584	0.893966066	0.041474371	0.177922149
25.13009808	49.61313952	85.54219035	21.86676034	66.54176253	2.659311051	0.049668231	0.478252359
42.96101364	65.10525606	167.4716517	7.076368247	80.71369269	2.816018752	0.127491313	0.502409735
18.52323763	65.22779934	97.60568382	17.08451075	40.34210184	1.676917553	0.046716238	0.313543951
5.065390872	43.15744972	107.7833427	11.77718208	31.60497811	0.470202264	0.048834948	0.150563727
12.79391542	54.016808	254.3686648	7.457783297	70.72726704	0.623661251	0.032116699	0.209694232
22.69684342	34.11388679	85.21773278	8.395545981	50.95501774	1.065294759	0.034519631	0.254937044
35.43429105	59.52732376	153.6728592	7.71553759	77.95765707	1.014212942	0.086506395	0.180472533
16.31644953	66.83817929	136.4011373	11.9789186	58.76240269	0.654909871	0.043149137	0.343802913
21.1857883	48.15709222	137.4102258	10.35028682	44.80397989	1.638028211	0.048199221	0.755441187
21.48162761	71.26206587	86.1699992	12.00324004	74.0820624	3.288928795	0.066465846	0.745224745
27.59833373	76.4668178	264.2891733	10.11473871	108.0821128	3.91220711	0.085462359	3.4821233
14.66700885	32.95577131	57.50168427	10.83978688	31.91957423	1.078667951	0.043555081	0.205177203
9.800775961	35.23727948	33.27998689	8.109495568	21.75306294	0.773612623	0.023974397	0.046602396
9.468367427	45.59435583	85.47397027	9.604985208	84.1702878	0.827185848	0.04990152	1.638384332
30.25744639	67.9875166	164.190679	20.06212187	60.67040345	1.98004193	0.107737889	1.058921274
21.89325756	70.40073115	89.93063785	10.48646508	44.75132989	0.493863178	0.047031321	0.076888461
12.40118452	37.22608782	33.08027256	9.218419257	29.51833159	0.955218867	0.024915029	0.062834592
59.99840482	81.53320219	149.9135578	26.2523528	170.9386686	4.516333335	0.238728367	2.679239779
27.30986544	61.62892424	106.1037954	17.37765895	63.71967482	1.25298614	0.049289367	0.628717693
21.39822463	50.71578725	49.94532083	8.138376738	40.0848746	0.853431495	0.042074457	0.209694315

25.62400606	61.08254206	109.5824222	17.24569471	58.77469664	1.721847479	0.069693841	0.411545197
12.95898733	114.3872431	120.8237419	7.250202231	41.3886709	0.41986881	0.030343073	0.071329329
26.91715975	72.40969409	207.5907387	12.74177598	64.63135587	1.890790479	0.058118531	3.171948599
36.31455613	52.25383733	70.93321208	16.13915533	64.54721211	1.64944413	0.077915206	0.693085036
19.4331505	38.90021299	78.1279862	18.3556386	46.29648073	2.022327544	0.101210732	0.402594137
18.9756959	46.48271909	132.5012082	9.764027715	66.65799962	2.387929152	0.061379088	1.04551851
25.71600624	42.49296004	82.15231411	7.484368584	43.6614706	2.768348089	0.045991979	0.062104783
8.978675644	49.34306225	62.56436224	11.0795792	38.29256448	1.262972368	0.044089922	0.253316333
21.20407936	54.25964246	178.5905496	10.02356976	96.21186062	1.933416431	0.061816729	2.071836313
45.33793305	53.51770952	171.5403936	15.63459072	74.39238292	2.633253775	0.090656444	0.822677326
14.59816065	48.96391231	118.2684734	21.65370778	52.16876395	2.311163201	0.069958701	0.366294258
13.08389332	61.18398414	355.5344818	5.109381064	94.20382303	0.682204401	0.03661843	0.30692537
13.58689266	35.02414736	105.5292593	12.60832397	48.86772165	1.189383557	0.041819392	0.085589155
21.08545349	63.91969671	198.0663449	8.116864894	86.18777288	1.027597713	0.050443189	0.246131775
7.52048593	61.95881635	76.276156	13.19811426	46.98926369	0.431487771	0.03033984	0.193927226
11.51109526	34.52822086	119.7267987	6.296508292	39.86300742	0.932632264	0.033345351	0.231538603
39.46034516	58.26632211	62.21497038	9.76037201	38.00841299	0.884096632	0.079815041	0.158317908
32.39619294	55.990469	201.9200736	4.166665332	70.8120428	1.642406699	0.042645757	0.177709629
22.64774979	36.30491864	124.4975374	16.03229912	86.80356984	2.06751806	0.06770967	0.648454552
26.38581957	36.46584811	88.33700917	8.713856395	44.60063572	1.378167358	0.053832808	0.359224884
28.78532298	66.32202355	179.3001984	16.04614026	90.60276446	1.031397902	0.083033882	1.179791894
21.90977796	39.16652546	60.56499338	21.03531763	38.25568755	2.417347522	0.085960359	0.180399873
14.87518225	34.28470473	306.0016179	8.522993147	102.5586341	1.773427996	0.046234465	0.548792589
33.17911367	64.61684179	218.8486916	6.387960724	113.6939071	1.041541081	0.031023919	0.4412103
26.94048564	53.09964675	187.3047643	6.893300345	143.0007163	1.299535995	0.068904584	0.180641511
19.76990943	38.56398678	151.4913745	16.96140341	83.6458578	1.90308771	0.066312057	0.260783085
22.19151226	56.12493212	148.478173	11.69310407	53.8943006	1.639719693	0.06837996	0.618741524
37.1259427	51.11541522	224.1216328	10.70652799	77.04532329	5.512501298	0.122122014	0.875542698
26.37289809	55.55268755	127.5413443	18.02974117	85.51735472	2.344765204	0.061122686	2.267113459
25.7481606	51.44705799	265.5330575	11.27601281	99.40319758	2.341129364	0.087784896	1.143084085
32.79924072	60.65300766	266.0946109	3.928911044	104.4135061	0.718361893	0.066831303	0.563554547
16.78821366	58.1305383	187.0196651	10.72184199	88.65734581	1.129969249	0.045546907	0.406189378
9.579337008	56.28181999	72.27133475	14.17875024	52.37049595	0.946472657	0.035761015	0.193879161
25.66376959	60.60441673	79.50835637	17.86723536	62.18551814	2.205883115	0.059905406	0.798333122

Bortezomib_1191	GSK269962A_1192	SB505124_1194	Tamoxifen_1199	Fulvestrant_1200	EPZ004777_1237	YK-4-279_1239
0.012447042	24.88307809	6.374947936	29.47168267	23.27993079	316.276087	14.6984663
0.006846557	16.178941	8.976237827	29.62995047	15.7425454	137.6828557	9.253090556
0.007612898	29.55292394	13.48114039	44.37256504	29.5028006	239.7874955	20.59255056
0.009246448	22.25639675	5.601090786	46.47386855	12.84408983	137.7057749	8.658551043
0.013743601	26.86931456	10.19670269	61.87650672	31.92093627	320.8640318	47.86323327
0.007481932	16.87515175	8.760956164	25.72918526	10.6735603	98.81510536	7.245480034
0.00741174	15.51359358	10.88650774	29.95732723	20.89094445	160.3151416	8.851021102
0.007119656	16.45736247	8.630625929	52.93111551	21.84235395	254.4851378	19.12055751
0.012849215	19.73612446	9.406606836	63.05108756	36.59549609	329.8010748	51.45087209
0.005170589	13.10630011	11.69778588	23.77868756	11.2122468	89.56685358	4.325214672
0.007305996	13.88069515	8.539067401	31.68428452	13.87588427	128.3234544	10.37765272
0.008471747	16.7971062	9.477827064	40.24642405	18.73077017	167.6731697	16.03864945
0.007497415	20.00189173	10.98895079	25.48338311	15.39131703	140.4944188	5.488374781
0.013942112	20.33874401	5.982893434	59.03069484	20.90074837	207.7219795	39.52877397
0.006399282	19.68281748	11.53620599	38.06213945	21.14019108	162.5081909	13.92904425
0.008177831	15.30354396	10.9704894	41.73151322	17.35710045	162.477121	18.61329272
0.00490832	25.05662563	10.36171275	15.95917536	10.51178146	113.5742575	2.412057855
0.007294989	20.00949855	7.197933918	51.07990793	39.66149983	310.7884771	15.2508679
0.00635292	25.57227627	15.6820647	43.24990063	21.83260909	209.343308	11.84111186
0.004688362	18.60666214	14.41984141	29.66258818	12.11380803	104.6848402	2.409761016
0.007799678	14.22342528	11.53654803	38.99699796	16.57155063	152.6947067	8.430388479
0.009173352	24.59412479	11.50489498	72.06092233	27.91867456	305.4355031	25.77307899
0.006580252	18.92746271	10.42638892	42.85976818	20.24007819	199.1443799	6.235626024
0.012638583	17.9601878	10.92075156	48.25997321	31.48808618	273.0669358	21.65719921
0.008194332	19.58064966	11.5832462	45.86936749	19.80623165	164.3738848	14.99528336
0.005184572	15.27517199	10.8103501	42.9468797	17.12894158	192.6041052	8.170642591
0.010376665	21.87274609	11.25494401	51.05478647	26.37680894	262.4674244	26.55424789
0.008242795	19.48677444	10.86295735	64.75785616	36.30161564	313.8851357	16.1681901
0.006111275	17.10551379	11.93187864	36.64023164	19.09950625	157.0386686	6.104768414
0.006926114	21.85954215	11.73462942	36.426106	16.23567224	175.7084069	6.649813347
0.005323218	13.87048451	10.94023171	39.39657252	16.25232863	162.7138915	4.456668164
0.004461901	22.57791327	12.63073348	31.26656109	26.50752847	220.8190602	4.326850255
0.005636544	22.44572374	11.95521013	47.44532345	36.98409057	362.9520289	17.57542069
0.005706343	15.73427343	8.789991799	37.1074064	14.38698948	150.9207401	6.782109465

0.009630488	13.70286154	10.30391327	41.37837496	23.20253786	184.0992511	18.80112681
0.007807136	27.46875153	11.84922321	60.60485456	29.32884967	364.7279028	22.18580222
0.004105552	24.77271437	12.22877927	23.58914886	13.06528907	96.90311885	5.992577381
0.00735166	25.10309905	11.96459809	55.06020814	41.67788985	517.0151476	16.29230324
0.007078392	14.88874748	10.14374926	34.54881126	20.94371012	163.3043075	13.34106919
0.004809963	29.11721508	12.52655495	37.20121771	15.10391723	160.7280822	9.096850092
0.007411691	23.5649683	6.273232136	33.12511156	20.30937539	213.1174851	11.57546711
0.005809106	14.13335573	9.89574387	36.7670755	21.07185775	158.3802772	15.07984207
0.012161992	19.53673222	8.095905017	27.83769993	25.55481326	233.5013607	16.26970398
0.00958269	13.57883788	8.260531428	43.52141661	17.61836059	123.8722691	35.25447922
0.011127597	13.94187424	7.02723915	29.725549	19.89223919	197.8659514	6.840973996
0.003248358	22.608538	10.56151445	9.361801244	6.644245525	60.00510889	1.668443915
0.006471124	14.0732764	12.10306784	29.95255617	16.90585354	119.980811	8.103045924
0.007313879	17.07636605	9.75641239	57.80606582	19.08222925	203.0718349	10.75452009
0.008182232	17.45629107	10.39197105	34.34988045	16.49708533	122.0230005	9.648143235
0.004914639	17.10107099	14.93676393	30.20213384	15.48989294	112.210486	5.211201767
0.00568168	12.79308409	9.879402001	24.66798166	9.790667776	71.07884395	3.763330012
0.005217008	17.21286012	11.5586574	38.69734426	19.39068684	172.9358383	9.095816647
0.005113202	16.66417134	15.08973247	51.86518381	23.44499761	223.5501494	12.3211541
0.004791331	21.30492803	15.22424852	46.37856078	14.5427549	135.2980022	3.003609714
0.005927948	16.30294522	8.076879318	43.53149842	16.29575729	178.1124906	7.378966947
0.00902774	22.29145761	8.387017837	65.45136578	27.68702125	312.4328836	13.65875325
0.005667626	18.66097875	14.72757228	26.45504499	11.50321519	126.0345579	2.190429361
0.006105829	13.21015721	12.87762218	30.46786348	12.49862194	107.2094249	6.597389497
0.015309633	30.63030113	6.114701712	38.90318571	36.34862491	504.9270368	38.08930251
0.007201771	19.52987085	12.53518958	40.85608921	21.91426637	216.2546036	9.56714755
0.014423951	19.57648848	8.630540036	57.26992118	29.08632971	337.5561837	35.02936148
0.00641198	20.66522312	7.934046042	53.08138375	15.99454383	201.0909658	16.21604649
0.007965554	17.89749618	12.06455527	69.00945617	26.06475802	298.5439852	25.60608948
0.007795458	17.29855853	12.05684205	36.68575177	20.07853982	147.2252533	7.650189266
0.008107701	12.77397249	10.26093483	30.6338739	15.65745569	105.0287678	7.988545013
0.005821177	13.95296499	9.97523806	26.59900048	13.22303383	96.06485949	5.418958567
0.005537583	37.60306439	8.404341444	34.85567147	21.407567	224.0636469	7.446010666
0.015324132	26.68742764	6.564828841	59.99506189	53.52532287	708.3170044	98.02641922
0.004828717	16.67308812	13.57711415	35.96688595	23.31108111	168.6529359	5.319790546
0.005183367	19.93327049	11.568904	20.15494504	10.65872114	74.70987329	2.580132899

0.004516191	20.96466712	15.35285203	35.65325249	22.05797663	306.3129141	6.068723871
0.005868628	13.33334272	11.9002887	30.01639447	14.94989735	121.8104329	5.173553708
0.007493653	16.1136944	11.63349381	61.01768313	36.95524882	290.3585586	14.47489172
0.01643656	40.12478176	7.067589767	98.21521978	39.1241089	506.9278573	115.7994657
0.005353871	16.17940046	11.17486343	34.84835854	16.13212483	138.3279763	8.654957652
0.00531466	13.46236841	10.20762882	30.60188153	16.27218123	99.9777439	6.457856239
0.008682501	19.88561583	10.41614918	25.27229057	11.66516028	121.5756452	5.768930897
0.007543415	14.58603651	7.63293688	37.13808802	21.14438223	209.0363025	11.22094196
0.007535118	22.83196969	10.11820038	31.10080331	16.37777548	193.992033	13.55691898
0.005760813	14.35565992	10.00486677	25.50977259	14.35590183	140.3386987	10.08996946
0.006473	24.14029717	9.053982992	49.96194921	33.65952756	287.621556	19.80298847
0.007257393	18.98309237	12.76028842	49.24610058	30.14327763	260.7186651	18.12201172
0.004109456	9.934825311	12.60112067	27.37041111	14.62423842	105.8343827	4.67706962
0.005291701	12.05912918	11.22103384	27.36120929	15.27219568	123.2711824	5.428817942
0.009943937	21.37560197	9.229946727	58.1611066	24.7309662	308.2361229	25.42644219
0.008094941	15.51201359	8.97154641	61.68106469	20.02562289	185.6578223	21.47582451
0.005527145	14.43134751	12.92704867	16.81064458	10.19229039	100.0339286	3.903572573
0.009442686	13.10551769	9.612288636	38.4522253	13.79886655	115.637679	7.565782585
0.006324212	26.5119737	11.03548895	64.42139576	24.03351764	385.4458663	16.34693293
0.004361231	25.00889002	12.85861723	35.8104167	15.19557829	130.8629407	3.961044648
0.010514948	16.57498232	7.113876243	31.04845154	20.50546018	189.389131	17.11571032
0.004515185	12.28998987	10.54759401	28.44118994	14.43770303	119.3509736	5.623418693
0.011878592	18.07546138	7.342347734	39.48856849	24.96458931	249.0472398	19.56116187
0.007682061	15.62102624	14.13829831	39.44038838	17.92342768	158.262451	8.585922815
0.005875574	13.81527348	8.327285449	39.28643576	13.0509068	135.7854155	6.132069548
0.007492118	11.80756242	8.145464963	40.81561827	20.01897873	186.0613584	7.038222637
0.007340097	15.67885864	9.307977801	31.50384683	17.02692634	143.3718468	10.09644557
0.006243968	22.37777911	9.242420591	36.75877509	29.52485425	245.7450869	6.950233509
0.009014953	14.51302	8.707631339	43.57496566	20.17766087	156.5621956	21.38637624
0.007666426	19.2870108	7.490590714	49.18235265	14.91243266	166.082476	12.22273154
0.005761377	17.45047536	11.19627212	36.59216841	16.8949619	178.3124896	16.71035072
0.008937683	17.52023493	11.61533984	40.35008747	22.67540927	178.9942017	18.8079234
0.003016855	20.72030088	11.46522644	16.09131152	8.092422743	74.98418043	3.046469761
0.009345003	12.42176024	7.881365186	31.35056686	14.07919339	144.3535275	9.57893513
0.006813822	46.26343044	9.395937199	22.67774148	13.78798991	167.1072677	7.251488058
0.009337758	15.46826277	11.82889843	37.08652346	27.85369916	219.2764694	11.93182416

0.0078248	16.30753969	10.08556058	37.92856885	20.7385752	149.5249215	18.22027445
0.008072036	14.10650043	8.678999213	41.11515328	16.94287626	142.0432126	17.31874633
0.005523357	16.61771092	12.84200318	34.16780887	14.88691516	103.9014086	4.444308099
0.013607079	25.16602587	7.174610952	62.24077291	30.85254401	518.872491	60.55963558
0.008873922	18.40401613	8.232402546	41.84724197	19.58262905	212.0950897	14.8249019
0.005899802	21.73488715	12.87310706	31.27008081	17.4578355	164.2383361	5.639706009
0.008526673	20.71812587	13.55417523	63.27642576	38.51976856	368.079353	22.04282075
0.006351576	13.40995271	9.550732288	39.68580782	14.25522399	144.0840591	6.968042161
0.009578239	16.42743138	7.582045782	53.10073399	26.19606062	264.0219411	20.27859575
0.006096867	17.6512517	9.493826725	35.15038276	16.93031351	118.3584557	9.549416057
0.007881967	20.52990078	7.197031759	38.13186399	16.12960694	140.6828839	13.56129965
0.006598402	17.15887184	7.769289315	39.91871784	23.17207166	251.8338889	21.36261362
0.0102038	18.37988382	9.021076271	73.51558608	31.00409512	315.6356061	21.49171171
0.005585669	13.47418988	11.87738929	26.86617804	12.44315483	106.0951127	7.563322208
0.007440485	17.48595349	8.793324563	32.54428002	10.11666996	103.3777626	3.710254092
0.007581378	19.03884931	9.058345699	34.77207852	24.54410581	169.4565465	10.08808092
0.005160403	16.94128039	10.93087523	32.35977526	14.27012606	143.1444196	8.282857103
0.006573966	25.04891776	9.736740704	33.66117521	17.95236284	149.7449465	6.981595803
0.006853997	23.55454595	11.82363375	57.14457885	14.57332342	170.6259907	4.093708828
0.004953316	17.59483384	8.36900277	36.13845357	22.03306194	188.3347632	10.30551297
0.011037408	19.56542375	9.098162253	48.09957011	28.3783901	258.9835001	21.10511252
0.006099424	15.88444107	11.20507275	34.11704876	18.13881868	179.0067219	8.478952394
0.005436992	13.65895951	10.27158224	27.10994631	15.50504352	159.2756448	6.581691088
0.004296467	28.67218182	11.45420979	41.95057226	23.97766723	254.9400253	5.31559771
0.006420876	19.36094389	7.411530268	32.80674266	11.89606906	108.5633318	15.06122295
0.004587774	23.07469278	11.593491	33.66796721	19.87936916	208.4500947	5.36158811
0.029502306	22.29845064	8.461431825	67.22469082	40.31177781	321.4795742	61.78165003
0.006078411	13.6364373	10.39168768	38.08593395	18.88540437	151.7053961	7.376352547
0.007087314	15.63348413	9.421053961	30.46803707	19.13679829	171.648769	10.25251163
0.010715812	26.14473132	7.304768053	37.27338784	25.93284145	197.7279884	36.28102806
0.005626481	30.97701452	10.2886568	39.24868354	18.07405639	270.296175	9.603591596
0.005301777	18.51421562	12.12808282	43.66864279	21.6688261	188.5567307	11.03377535
0.010543261	17.83213379	8.168837596	27.06078328	13.70135566	108.6686412	15.28074387
0.007519903	19.28001472	9.158737533	33.63603479	20.19882116	142.1422797	13.06879987
0.00727474	13.60510001	13.32105924	44.76952606	18.55718028	166.9706017	8.49130212
0.009783424	25.9023446	8.582086256	41.44172392	29.24138101	283.3818373	23.89997118

0.01169355	17.69038622	10.91942908	64.57938336	29.57047681	329.0085819	12.75785355
0.006642661	17.80408287	9.367659892	51.98787314	21.30468064	208.971952	20.78506467
0.007845036	28.87028614	11.00662251	40.63042582	21.89591097	218.4401652	16.60651973
0.008356177	15.9130817	10.01058184	34.08863959	23.93571461	203.4522072	17.03065057
0.015693792	55.2416815	11.12665011	77.60265465	43.92762283	666.1592568	22.09715157
0.005190738	17.86764609	9.334700299	36.76532257	14.609748	164.3529705	8.378331145
0.010334011	15.77318919	8.833261204	41.95874343	20.31598705	202.1374053	16.37843389
0.006619793	16.35367615	9.190559972	35.67057636	19.21448528	143.3375751	8.287022351
0.011380242	15.46731162	9.035676529	40.95096454	23.72641577	157.0918252	13.68117586
0.004782099	16.83443573	9.668387307	34.75840966	14.08521199	147.7590471	6.69505946
0.009967785	23.47117289	8.387127214	44.18586442	25.62917653	289.8690876	41.46060546
0.010310661	18.16132894	7.68041911	37.64753681	21.20137329	252.0091091	6.936456093
0.005885208	22.70057103	9.192295068	45.58440787	16.6077611	185.8410334	6.593036142
0.016065111	28.73204424	5.556334672	50.36359677	23.65344262	233.7501359	50.29797819
0.005753164	12.48031889	8.662941145	26.39452418	15.18992426	103.2575497	7.335596375
0.015484677	26.16149436	8.867189017	36.71802719	22.15230527	206.2144147	19.2998779
0.002554355	30.70031404	11.02256205	9.386053848	9.838447121	85.48635527	0.949048813
0.00836755	19.88388193	9.851148194	46.41137158	19.62750695	267.2109287	16.42604977
0.006531176	16.08846507	11.21774982	48.55610659	20.34527363	191.3295086	14.18455032
0.006204612	10.99183536	9.575853133	27.64840086	14.57919504	126.8869409	4.180015636
0.004584713	19.22606821	8.874925314	22.91702642	19.78920192	171.2643804	6.883528632
0.007118307	15.22047485	9.441814406	35.69021084	16.65817349	148.7603932	5.424283806
0.00895809	17.56690512	9.347014449	39.4377844	18.74097818	147.197686	9.606253595
0.007813885	16.02510326	7.832457828	43.40005399	20.82327059	204.7296533	12.25644628
0.00487943	21.0950361	10.83781906	24.40702919	13.7409688	152.5271366	9.230416711
0.008912588	17.86885327	8.278033207	55.97269211	23.64440312	269.5235701	18.18544011
0.006065373	16.68775671	11.79195537	52.96547244	14.03747822	163.6474652	8.749433963
0.006666053	27.1237755	11.49520744	34.84122154	24.62610337	192.6376701	4.763314484
0.005819102	17.72166851	12.1500818	25.37872007	13.01476588	120.0667339	4.217760902
0.006722743	14.81881168	10.65712431	29.12663149	16.16555318	154.3945592	2.79405555
0.007234888	13.02675923	8.861680085	34.51968064	14.58998359	120.7419619	6.825414072
0.009779101	17.03544381	8.097161396	27.84585822	16.46581116	196.6055262	4.535452979
0.006555663	12.96781788	9.513114646	26.74061771	17.52946212	113.94464	7.817659676
0.017008482	26.59243533	6.535495429	35.45104384	28.93264812	261.859811	17.76757973
0.008284581	19.70016819	11.60467559	33.47368649	20.54823051	145.359505	8.050444584
0.004607671	17.94484828	9.592228535	27.34394367	11.91067548	122.7527549	5.393761003

0.006233966	10.19574116	9.828973325	30.04199054	11.77671485	100.749536	6.401889169
0.005603212	36.20915128	10.67108883	66.49963537	23.36916009	222.9653171	4.890399083
0.007944332	12.80858013	8.550964247	42.8049924	19.22476459	155.599229	12.79549284
0.00587728	15.80002143	11.06069028	46.43621018	30.11804221	273.5582915	11.37984712
0.008743162	14.19814004	10.28306305	28.01336006	16.32622878	142.9942964	9.643163582
0.00971826	21.0168473	7.595287031	58.39928843	31.09937947	376.7090361	40.32587949
0.007577609	19.66185694	7.031656153	50.2720157	23.53144395	292.3671356	35.89555819
0.009005846	29.13936698	10.3555533	30.21667955	20.92392897	219.1267075	9.25952362
0.008060343	9.051735285	9.384835496	28.9880217	12.22434244	111.3405195	6.835256873
0.011899644	16.96204571	8.374916214	31.21159336	22.76121421	189.6012657	10.79954566
0.005740579	16.55751386	9.932648281	29.53801614	14.14481229	160.9125659	5.964595258
0.005659258	13.15238504	12.57808531	34.97915392	15.37737345	134.1062865	9.394860639
0.006215523	24.8216067	7.897690103	26.54296643	12.84276615	155.2581468	23.61526663
0.010427675	18.19599553	13.35273222	44.48544011	26.12088927	189.7106484	16.77113033
0.00749447	12.92851323	9.748799178	17.88916408	14.1155403	95.04141744	4.931849358
0.005717322	13.921019	9.321683664	30.88933702	16.98469275	122.0951755	8.963148562
0.005210284	12.77880197	11.82372063	40.1193588	13.83446565	138.6543682	8.593816391
0.010650031	17.65298923	12.28372477	46.07237021	20.89546613	170.3756901	21.32891384
0.005176317	16.87123183	10.77089574	30.8639254	13.20873682	106.0005193	6.140864616
0.005807873	17.72937856	10.76761557	24.13583576	13.0657373	95.47344293	7.455718438
0.00872794	15.72479585	11.02151611	43.4441835	17.87833737	190.9248931	7.852169095
0.009745518	22.9706818	10.14217356	48.836355	23.00417905	221.7513058	46.12924363
0.008855802	17.69645002	9.694175139	34.45109358	21.18584614	115.8204614	11.83982425
0.007295593	15.43674055	9.495450621	63.405519	20.16368683	151.3671415	16.37727383
0.008441929	22.7519346	9.225294134	47.91619304	27.68307538	371.2089334	32.53579042
0.006780361	19.43694656	11.77261731	47.59033176	20.25828878	173.9221148	14.42565939
0.010900305	33.01528339	6.068622652	40.32465636	26.12597355	316.1864341	20.22118682
0.004597889	14.95945178	10.81899175	14.35447388	8.868081498	70.00256889	2.31339475
0.005744893	12.63174323	10.49276392	23.08170561	11.26003405	89.12382111	5.056226607
0.005505115	19.49657451	9.790731925	38.06447654	17.21022173	174.6436159	9.198449529
0.004887421	16.22700944	14.9089104	34.90857268	17.15347317	157.7137833	4.107997994
0.007437343	16.43758856	7.553650134	37.11510488	17.28622218	173.0479231	12.4405911
0.007975474	20.46918571	10.84391882	46.15796072	20.54346733	249.3498954	19.55136887
0.005462038	13.45532849	10.72502178	24.57869786	13.061819	113.3212586	5.4125432
0.011495849	17.65835276	6.358905795	31.18991428	22.86635431	229.0848351	9.711567262
0.005442248	22.03380789	10.95496277	28.9363789	19.59427753	156.2493005	12.02968699

0.008455901	15.0851621	11.59804021	47.75509677	32.08857633	209.1673174	19.97770756
0.005918756	22.6547122	11.44257073	46.51303892	21.20133343	220.3617101	13.00814624
0.006812456	20.09196953	12.06003381	28.99517553	13.98415303	156.1296914	11.03890526
0.007107959	24.21282227	8.184308419	59.61525704	28.04166193	365.2905733	17.80482446
0.004644182	16.08498149	8.434964918	22.68001393	10.34546303	118.7393331	4.404588549
0.006122327	15.28270991	9.661612244	22.42156948	14.00906249	85.27837576	5.252786975
0.005364742	16.64989192	12.25026934	45.2618042	18.85309997	203.3399686	15.27171897
0.007196885	25.37690585	10.22440819	29.77637924	16.64753654	123.2884605	5.746376307
0.012256359	13.63388901	7.087502421	35.16831537	19.53018351	223.5521032	6.038356377
0.009361263	15.32419184	8.058243341	39.08742253	20.46257887	206.0432911	15.67322698
0.00723968	23.17971772	10.03361533	44.12204997	22.03690872	196.1223759	11.34599075
0.005562262	14.77687973	11.27968492	49.09511197	36.62443178	338.2647897	12.13498653
0.007978142	20.34623819	10.54926387	45.13226748	25.99159105	248.8537764	11.26882305
0.007298136	19.20930619	14.01230147	54.23681263	20.25704987	198.4284517	15.01868169
0.006850656	11.43240127	12.06075732	18.57262984	9.456741099	82.97351371	5.225513505
0.007848594	16.09697594	10.12529291	43.69076295	24.62902389	169.2358642	9.912158055
0.00490318	17.80796766	14.08467811	35.22618877	14.72602652	135.7500302	4.97336883
0.006733251	16.29709089	11.62268065	44.46709758	19.03617014	130.2556263	6.694864613
0.006696837	14.34067787	11.07320743	39.65524633	15.13324901	162.5767843	9.57582921
0.006761383	16.24696448	11.97056278	37.33170467	14.79252431	176.1753515	13.08447035
0.009071211	19.08113858	8.945094125	23.34584934	15.8847282	133.8371436	6.81168758
0.006794559	16.34917247	10.66775596	43.15067537	19.39185306	120.2420846	17.08518291
0.004076531	46.56150055	11.01852985	37.61666497	23.37986192	304.9594911	6.43814163
0.007768408	16.74122109	9.791453954	56.24448768	24.3682344	240.0797696	22.53622897
0.005291833	16.49825016	10.07546526	32.21670886	16.50764515	127.3433435	9.600171618
0.011107339	22.20270115	9.058215865	54.09823294	34.61901023	355.2810796	48.16357659
0.009359305	16.36726042	9.640446552	34.23510568	15.23274734	118.4107482	9.391946425
0.006830577	13.92437269	10.75659515	43.99764729	19.21080994	181.0805325	10.17163222
0.006245981	13.91018974	9.981019438	33.69534711	11.05562739	110.4513936	5.349468532
0.006265053	14.60534993	10.4386803	39.73354176	17.40119528	184.9295536	6.818124697
0.005120617	18.32377871	12.35806472	29.04783876	16.3423286	172.9606031	11.74326241
0.005300759	20.21192323	9.478072663	27.55746309	11.26763431	121.6138817	4.99607199
0.007420506	13.16211503	9.884010447	19.96447222	12.82852232	78.01034584	4.894415447
0.006931063	12.27261765	10.75251372	30.57424295	14.28457887	103.9735505	7.732928243
0.002794774	19.0912615	12.54156003	26.77416557	15.91500108	123.9232628	5.135567614
0.008955762	21.78534908	8.742174686	33.35268812	24.27941085	205.8959585	8.499494025

0.007243552	15.86160155	9.506702462	25.41075513	15.252499	115.6011992	6.449614903
0.006248122	18.11740411	11.21518662	44.42179882	24.14741297	199.5382131	23.04697004
0.006955628	26.74503523	11.93538523	50.71948666	23.81198968	306.5239729	19.50046195
0.008329183	16.8812468	10.2726552	51.1574354	26.72115599	235.5527332	17.07418145
0.005663165	16.11979242	10.4423796	33.77529596	22.44004231	178.8830957	10.35285705
0.01051082	15.42158904	10.15214878	50.15706206	17.39423683	165.3118746	31.34571742
0.004930335	17.01452037	13.63510077	39.38848898	18.73972168	139.2128666	6.646760421
0.007209486	15.53628717	10.6806577	39.38987828	17.87485422	175.147602	13.60484977
0.011120854	31.9815732	8.721349912	43.02600944	22.17424059	265.3146177	20.04740865
0.007779158	25.62499443	6.65123439	47.73976696	39.51700679	472.2051946	20.58306487
0.00523331	22.35719538	11.26177676	40.25547728	19.14162857	215.5761559	7.711602969
0.008513967	19.51812565	12.76982066	42.95083355	26.18485781	184.1509726	19.28083467
0.007263608	12.15852853	13.52779797	21.29477818	9.867133469	92.04145197	4.615637002
0.00868503	23.94277406	9.285037109	59.87677898	26.62696861	298.5520676	56.18245419
0.011241474	11.37768065	10.49774826	26.02187956	19.51747822	142.9570903	10.32184521
0.003774695	18.53464329	16.54150626	24.66801681	11.12459629	111.006459	2.189581674
0.005983792	16.51499961	9.587354419	29.23113904	14.58228588	132.4726715	7.268151861
0.008421035	16.74833106	11.03195518	25.47672223	15.95130969	160.1457267	8.850997406
0.011672743	21.02359243	8.065661605	32.220916	16.09473584	179.7049809	11.0030928
0.008977081	22.43892545	5.846936369	33.44051893	20.50219091	152.3604563	9.159246551
0.004909391	17.3581221	12.23633236	46.45995107	19.5114712	211.4732271	10.70682192
0.006366935	11.84370957	11.57059979	32.15639197	15.60379959	133.239692	9.852580914
0.006554625	22.8758538	8.126078737	44.99073096	21.25694586	300.5481271	3.857400507
0.007095138	14.69393495	11.63674883	26.30137165	13.31352997	137.0080159	6.969493242
0.004552042	18.56628205	11.99051054	25.68735814	13.09247903	103.6509379	3.197679206
0.009186365	14.94911574	7.688320624	46.49121788	34.04491464	248.9618339	11.57087857
0.004956426	20.70642522	12.43240817	50.65792404	19.96105987	208.8472264	10.07296436
0.006734696	21.50506824	11.61488231	47.96727897	25.96467433	211.462028	15.47046503
0.005210271	18.05679894	8.929651506	38.15423022	18.86693035	141.5164733	8.722710764
0.007061588	11.52890869	9.228662762	28.26624257	12.97276356	118.0270452	7.517489308
0.007556118	36.85362098	14.37276403	68.35199302	29.98677701	357.0970944	22.89187798
0.007410193	15.49358391	12.63184973	34.29934677	20.8941452	207.4073543	11.57164404
0.009757024	22.40888832	10.60797364	61.21286233	30.14121433	305.4171276	16.33073426
0.007674948	12.19373462	9.882327153	27.93983749	12.11045405	110.2986066	11.16805355
0.003668729	25.18630399	9.428636897	12.58862771	7.539386461	87.51195505	1.424439679
0.008695457	12.94475195	8.927748574	26.31497871	15.61905434	111.1309373	9.052643155

0.008907584	15.88526946	9.337668308	42.76058173	15.21329375	169.411406	14.23417425
0.006222572	20.76569583	8.513587168	31.71502354	15.32269295	155.2521742	6.679684055
0.006840554	16.50468821	11.5184893	46.64532712	24.4329821	209.3080466	13.15660145
0.01037421	21.3837704	7.576888876	43.63345435	27.26156813	290.4288321	19.38087687
0.008680734	14.14987498	7.7248562	27.26024906	10.09727317	96.43427543	7.991565327
0.005707594	13.69451343	8.214584982	36.68837744	10.96521337	124.8205686	4.658607434
0.007107796	16.21823032	8.50124552	41.28195824	21.7119113	208.8983582	10.93115634
0.008069149	14.04980589	7.950480213	33.36446574	23.33561172	155.8692435	8.800343317
0.007913384	18.96888568	8.604028585	54.26966463	19.08379709	180.0742244	21.53984297
0.006818054	13.32189788	9.061081465	23.91914897	13.49332448	96.01135615	6.867340868
0.005365899	14.7004925	8.14239133	29.38169917	17.72056086	209.4535439	7.059637592
0.004990613	11.30304972	13.53808894	31.05508041	11.19720714	91.68845656	5.194842379
0.008500789	14.67687895	7.505974299	40.73714681	19.54056474	192.7171146	12.53158048
0.008506091	14.38622231	8.564523492	43.06888471	30.34871347	248.014142	21.77628292
0.005720284	22.24583582	10.01156786	43.90045462	15.63598902	145.1060713	4.678092057
0.011673778	15.79278262	10.45571568	16.17900414	9.875228177	100.6143535	2.084070613
0.0051649	12.88725766	10.74629962	35.35673199	16.63580555	117.6720127	6.779367459
0.007381239	15.87423063	9.477872621	35.72445548	12.60477639	127.354453	10.05443895
0.008216527	20.21393386	11.57911665	61.03253547	30.73011771	298.5451082	36.86644142
0.006119456	12.97344548	8.223154838	28.94199695	19.36871372	166.7198092	9.610820177
0.00612167	15.20459359	9.666447526	21.76063252	11.49440327	107.713474	9.832180426
0.0071735	21.46155188	9.007964796	33.98908199	18.13708376	140.0263404	3.771672109
0.006579608	19.37080786	10.77727971	72.24312508	23.945668	252.4359146	17.80027543
0.01162752	16.71417903	10.44476665	48.77890689	21.49140856	196.6698871	8.055701095
0.007599248	16.37825491	8.308230067	40.16946006	25.6833531	208.724812	12.27553286
0.003847789	14.12520979	11.84334807	18.35425148	10.71625509	80.53074931	2.936684753
0.008978014	16.46340238	8.405797898	35.97579039	18.67575272	145.5041152	12.73146685
0.009771924	13.81661974	8.470117148	34.53668108	23.01706331	178.6205469	10.45687085
0.005785647	18.55731879	10.15574503	19.70363518	8.76634055	100.5173283	4.578827237
0.008500516	10.90399335	9.248310144	26.9008615	16.08514926	112.2659656	8.474112412
0.009482532	16.59169337	6.726757094	31.75825656	21.48788679	211.51997	11.37972599
0.00715365	18.44583192	10.12715088	46.61113406	23.92312246	269.7828329	16.87747319
0.008049963	21.81566206	8.458810321	25.59157468	15.57260801	169.7893161	6.761875135
0.005845581	19.49517225	15.00359329	40.69900257	23.91562831	222.0931395	9.664459055
0.009655593	14.41149072	7.524510439	32.30914003	14.13094477	123.5180472	11.50376229
0.004768461	21.06848126	12.26043342	49.93397878	20.9179326	220.7542443	8.03965937

0.004948339	15.37432607	12.75626743	30.09140649	14.65618827	112.3806617	5.087161063
0.006963157	17.13163644	11.21168417	46.68555558	25.73155743	270.8909662	17.80498493
0.006799915	17.34481604	8.747832767	30.69125359	14.99612407	164.6674739	9.335652753
0.008296649	12.91921063	10.05562547	33.06692257	19.34866087	137.2947852	10.77399414
0.005921828	19.21037559	7.562643191	39.09787934	20.09832921	200.144603	14.77396761
0.008705522	19.76852164	7.374757231	47.48382293	28.01350788	205.4296661	14.69403956
0.008605083	18.7247885	9.433468768	42.42918192	26.3880765	230.7044863	23.2315571
0.012155097	29.77509759	13.50246832	64.46641212	33.95062412	367.3523641	12.83673146
0.011047673	18.96090762	8.622266017	39.71162247	26.86831146	285.2036738	11.97771781
0.007458212	15.45918215	9.610144715	26.46594114	13.60946257	109.2049003	8.438510439
0.006708187	20.19694356	9.654056179	47.27472922	20.52385752	203.7161192	14.09448133
0.007618775	20.90545551	10.65598051	29.13357857	15.58461567	126.4755015	5.078061516
0.005716651	23.15911096	9.985841655	49.10041094	27.80259128	243.0658001	13.89738334
0.007702353	16.41326197	11.58366578	38.80814623	18.49870545	224.1128414	10.22789075
0.008198829	14.26999656	9.711028024	29.79915076	14.98571809	126.1588467	7.51607006
0.006071174	17.74446775	13.78530459	44.07874744	23.84575781	216.8391557	7.511286552
0.006949517	24.2292886	11.14799639	47.30808293	29.14188198	263.654667	15.7606024
0.00677054	18.37999532	8.870833161	42.61579134	13.52303175	151.3193795	7.860706544
0.004842043	10.05132201	12.01167615	23.83844454	12.11727732	76.64381717	4.444591903
0.002812774	15.59718165	14.53294526	11.73509637	6.797297313	42.79866824	0.654197396
0.006883466	22.51537475	10.31285737	22.0720321	15.13892698	187.092998	11.15610828
0.004998062	21.10533191	10.60536737	58.96380087	27.77900803	300.136866	23.41229965
0.010442079	10.44748775	8.347893092	21.93866356	15.72629214	126.2747135	7.024304404
0.008466169	15.39342914	11.69290034	51.30992295	23.37134947	213.118892	18.42690305
0.008544786	18.5655433	8.941122521	18.07689532	22.39935303	188.0299806	4.401389973
0.00425652	13.5120918	14.33241057	26.20303592	12.89525643	101.0507525	2.399818628
0.008915848	20.83623142	10.50854035	43.3627814	25.01871884	244.2333841	21.71451655
0.005298042	12.86754935	11.66212373	30.2356101	14.47764854	132.0556355	8.331120398
0.008038964	12.68927196	8.554689563	21.98255522	14.58308815	127.9700511	6.878600514
0.005546745	12.69535154	10.35119276	27.65655813	13.68969561	144.2164234	3.715880275
0.011123755	21.9833007	6.287636096	32.2282504	25.27307392	278.4982509	25.42664933
0.00993201	16.50144305	8.311348856	61.82431632	25.25132037	242.3739477	19.95041279
0.007131018	20.03516414	11.9131846	50.07821956	20.63684284	168.8970666	12.74624948
0.005616489	17.70158882	9.557525349	41.15922435	22.49933101	260.746508	9.845438526
0.006398116	15.878929	8.977750875	41.86228265	18.16873519	168.6781713	13.81843704
0.006460397	13.69263515	12.90943639	33.63494793	12.87147891	105.333746	8.775942092

0.009594644	19.2491643	8.861481283	48.00224056	26.26764951	191.9853083	24.74651002
0.004093579	15.83848618	8.972859165	26.0630486	9.577231368	107.0859221	4.807423751
0.007114437	16.9559428	9.970685636	41.73954649	24.15609314	264.4350731	14.1511421
0.008719643	24.71448704	14.3453713	55.98037103	20.26875925	218.5802279	14.42901138
0.006986323	17.90620067	12.15162815	38.34939646	20.44394495	230.4204451	12.29697937
0.007894588	28.44954432	11.70385274	53.38493613	32.23110575	338.6538573	13.13890626
0.008765971	19.02373582	11.16975885	40.97647556	16.99808944	211.8997861	20.49638971
0.008817431	22.08870941	9.089604836	40.06951106	29.27530516	200.4832602	9.375117545
0.009349929	13.86146899	8.719366976	31.218025	17.10413009	181.3903347	9.632139658
0.012194897	19.22667974	7.680684607	62.67013124	48.37252811	301.0804131	32.50314982
0.012568555	15.65950058	9.439626869	31.83152445	18.43869206	189.7677046	7.221647822
0.008020875	21.96271872	9.056916827	37.46368441	21.14828538	229.8797676	13.99621931
0.006400231	17.410995	11.20354237	41.99630472	20.23619464	172.2072603	11.75926832
0.008437933	21.99796347	10.0767816	70.66501691	27.540421	293.3954866	22.70907725
0.006250205	15.27726757	11.70432179	48.20322449	15.35899656	136.8719535	10.62661867
0.006177376	17.96466683	7.536951835	56.61037968	24.63505692	262.907475	14.10747112
0.00670228	14.34872228	11.68396032	56.91267722	19.57573409	225.7611537	7.856813593
0.008180106	14.84169195	10.24921166	28.80560781	17.41570475	127.1193655	10.64344177
0.005825877	15.25246018	10.73891355	43.90538584	19.76039491	216.4386844	12.38845906
0.015081502	13.56388112	7.827964789	31.58754137	20.12771113	143.3842106	23.03895638
0.006709255	12.77157512	10.74353078	32.40559696	12.63324437	92.19187465	5.371774224
0.005256895	14.83335536	10.86758927	38.68195267	15.45117292	158.0093446	7.771102481
0.005383861	19.84142018	12.84191897	34.32643176	15.06504443	135.0204518	3.623126007
0.005326316	19.46342294	13.50535041	34.70611903	19.91871766	202.885327	5.437949338
0.006780804	13.52061995	9.300347785	32.49021584	17.17526747	108.3043196	14.63284421
0.00672838	18.71204622	8.665257334	39.13176244	20.6132118	219.2289408	9.270562881
0.006599072	13.04226841	10.15960731	21.58198092	11.13141971	84.18587419	4.224945421
0.006867226	12.01232051	9.77881778	19.69769512	11.9258087	92.44328916	5.685348434
0.004716922	18.24732417	10.42022133	23.87041782	10.80221401	113.555204	4.473225443
0.00638739	17.6779038	8.782785622	60.20823048	23.03766089	213.3556293	15.72633503
0.008934332	17.63525249	9.274856245	60.57961013	17.58914145	231.0611892	12.71383267
0.005132475	21.20955806	9.883104041	18.80551761	13.76938656	103.7636068	4.065071091
0.008667633	15.62765112	9.182190353	33.23755489	15.52313715	143.1524324	10.34657235
0.008801508	27.25374307	9.469991007	53.74177651	27.84290101	308.4907821	20.41377463
0.007153413	19.58668671	11.06071529	35.95390363	18.99874708	135.9430552	8.707727092
0.008094848	16.21374063	10.00456908	45.49559271	21.38922157	198.8448674	16.7417283

0.006349008	17.05010592	10.89296027	34.62137765	19.32286512	153.0709623	5.627476086
0.007027796	19.07301644	9.401414321	39.43705395	13.91837121	150.036322	7.446661695
0.008382995	13.89345463	9.252813931	31.15461016	21.3189045	137.6743184	13.12349344
0.007148307	16.45847172	7.827384344	21.13797014	10.94934528	112.2658972	6.361300339
0.008645283	12.45301846	9.876365782	35.1503407	22.12753869	144.0598107	9.080297937
0.009596277	17.56563002	10.84011037	34.35357581	28.90687221	344.5772604	13.58942461
0.023847237	23.5922095	8.128555751	25.29220644	19.65140729	144.6616592	22.11617209
0.006267792	18.20403176	8.502175463	23.40416681	12.00130661	165.5832987	6.123654793
0.01275529	16.07267495	6.89032484	46.09491948	20.94087675	251.9362567	21.10873709
0.007810207	15.91350059	9.370956734	25.32368998	9.224899172	126.8246952	3.135743939
0.02041767	19.47975559	8.333074242	52.13559259	16.84460656	191.3069747	19.31871402
0.013535039	27.40222048	7.277343668	27.77976101	32.41029619	296.7999929	26.89442488
0.019470385	20.96555857	7.115119193	39.87767622	26.80660672	232.4129568	15.77039824
0.006764775	23.90417974	12.11515162	26.96022707	12.48262411	135.4430266	5.271990667
0.012274307	20.85281862	8.363593614	23.07830213	12.47313908	137.3346097	2.022592176
0.012289583	22.10173022	7.762432368	30.28235107	16.39379074	137.9006934	3.230711257
0.008685693	17.10649525	8.072377565	31.67832161	16.31331282	152.3478327	9.248219607
0.017350012	23.92747446	7.569641028	36.72673	15.04132988	131.881289	6.918593407
0.011905544	22.77880826	8.729783518	25.15437663	17.10588662	177.8108208	3.053185589
0.014769171	22.5242934	7.681236789	57.64779675	27.08664463	310.852933	34.62334669
0.007824174	16.18408478	9.559693071	21.14945352	13.53364683	70.16937103	5.717323236
0.005555021	14.76672701	9.64733278	21.84039913	12.34830985	75.68143281	3.203525221
0.010279747	16.60808512	7.99594267	37.18353129	20.9047017	148.2467118	14.99473543
0.00756537	12.1331739	7.633888849	25.60164757	10.94656179	97.80713094	5.734665691
0.011087371	18.3031098	9.195166346	40.47037226	18.80663208	195.5014959	18.89190009
0.011828293	17.3173437	10.29627571	30.38698674	18.7405136	168.4653839	5.51295261
0.010204884	30.23186329	5.525747653	38.85097087	36.45170451	377.978805	22.71065404
0.008193239	23.56396549	11.52991416	29.51991871	15.4204234	154.9246574	3.6036431
0.013019607	20.47880823	9.335619491	36.40855863	20.53335211	177.5499839	6.766653245
0.018165166	27.56215034	6.277220967	32.8105916	14.13954134	132.372185	15.61629598
0.013224733	19.27040316	8.176491334	45.75291025	22.1286863	181.5203002	16.5010832
0.011414262	19.45975857	6.793942933	17.43707166	13.8309248	161.2558497	6.485038757
0.007674233	15.98888293	7.963344319	28.00673144	13.00439026	128.8992352	5.41745612
0.00701357	18.04529891	9.393464972	27.36457669	15.3638044	173.2130098	8.131065029
0.016350507	24.5194976	6.353134227	37.02782244	18.50089841	277.6703577	16.70491134
0.009864069	15.66010723	9.434528521	32.62006211	20.18049063	198.7964199	4.767426023

0.005198603	15.74001796	8.490084854	28.8580964	14.55736131	107.8377379	6.356423943
0.005910266	14.22739254	9.042103212	20.65175664	10.8955639	91.42690291	3.052349094
0.009243811	15.48576948	9.2443654	26.51787361	14.34993261	130.1037571	9.717813118
0.00608814	15.38298717	9.652249669	28.71122946	10.7715627	135.8355632	4.344548677
0.008249095	16.50415947	8.853499643	35.95420967	18.94559852	170.4073266	6.772224584
0.012944879	17.57179763	7.247972377	56.70695294	26.72813359	262.0202268	36.53749587
0.008847318	18.06492527	8.678173918	31.40810552	13.7134383	129.8211995	4.997435138
0.017266934	20.6364923	7.359423352	36.227526	23.26098807	205.2359382	9.000132241
0.006329607	16.9430774	9.866042649	32.33212635	15.3562374	100.1218924	6.632545283
0.0114588	18.72701373	7.938339067	33.13007209	20.84381451	205.047343	14.95447468
0.008988028	27.2845707	9.626957716	46.09896751	15.01869939	196.3024468	6.162417657
0.01465841	22.46587371	9.350418168	28.96682172	16.45749222	140.1648423	2.432843624
0.012710439	34.5220179	7.658879863	34.33816807	14.28140589	180.2261786	5.918794675
0.008370105	21.72777089	8.955852311	34.8994858	17.59982305	191.9760928	13.27147594
0.00783517	27.53891185	13.10621889	25.44299423	18.57552928	232.6810962	6.391084256
0.007865432	19.49551843	11.43747716	23.91215096	13.76358048	143.7994915	2.722292087
0.008604357	20.22793194	8.648891842	33.61809969	17.30724477	166.3085972	8.394107083
0.030690447	22.7636069	6.985397037	44.7434705	29.9347312	329.9805096	15.88888816
0.012023591	20.53601045	8.610716191	35.2038907	13.54183791	143.712526	11.40263726
0.007718804	13.34427899	12.15859864	15.83575461	8.434410849	84.34954049	2.361645355
0.011749921	20.48231522	10.13951909	24.54076846	13.94344016	151.642154	3.2087732
0.005902984	14.98747895	9.419831133	36.86210066	17.52051373	117.6497747	4.429604653
0.010454838	26.70035697	8.792400137	39.70458945	22.96792435	232.2038461	5.128721927
0.005514543	23.20797192	9.407828599	24.38590712	11.00301569	116.8413952	4.427923575
0.013944561	17.95955725	7.831137597	25.61231304	15.77027358	140.283345	6.869450499
0.009560674	16.90626323	7.453612891	37.72206204	17.47259009	140.5070599	17.05129363
0.011838922	25.79477012	6.511065063	31.33301351	27.31223658	284.5903713	19.25517662
0.007800258	12.70658619	8.805627186	23.77224567	12.47250256	103.7061862	4.311961729
0.005309745	10.41766441	10.24535876	22.0864446	9.670222617	86.21525065	1.882720547
0.006871906	15.06414026	13.20471613	25.43692023	12.9284774	120.094982	4.024442889
0.013530742	22.32401227	8.312223029	31.35650224	22.00650964	230.9226356	9.730185
0.006084373	22.63472931	8.952467161	22.42882391	12.95330991	100.0466168	2.779766454
0.004873466	13.34477438	11.82790175	22.59975523	9.86674864	78.88414844	2.18634935
0.015432551	29.1216847	8.866790334	37.23480162	25.31768318	237.632459	25.60070698
0.008296674	19.97419338	10.39841869	25.53359485	14.46192414	148.4593126	4.13470734
0.004779374	17.57917497	7.574205575	24.88112774	13.12215554	103.307404	5.851448608

0.010467031	21.55075303	7.352892653	33.08435443	17.46038676	159.3939556	10.57717313
0.012602375	33.59755623	11.10850165	27.43850989	14.24071126	179.6700692	1.744389009
0.014059349	20.75435883	6.637235089	32.17559534	23.42758484	253.3562997	12.01326611
0.008567487	16.14672391	7.031503358	36.66663954	17.42730239	172.8980236	13.16902471
0.010596802	12.88624066	8.095968274	25.33724263	17.00132385	130.149474	8.1552634
0.007791272	14.25138898	7.945863131	26.19714823	18.66908891	167.4436165	10.81941976
0.008859234	15.84494601	5.666069467	29.33452956	15.38962256	135.0185663	11.54022308
0.007748776	13.69930415	8.450138691	26.23400941	11.829237	97.8664989	5.831470784
0.009426641	16.44639615	7.420953455	23.93717359	22.03762367	212.7955273	5.990910601
0.012811321	22.46826636	7.053360489	42.36958249	24.30963666	229.7819756	15.9770987
0.012026548	14.55001639	7.100897151	29.46458492	16.43521768	154.3864999	13.51535313
0.009998172	24.33221682	7.972839048	32.98755851	16.08934255	165.4313091	3.854163835
0.009507967	14.28548567	7.71696334	27.77176648	14.65631052	92.19141282	7.913807748
0.009922994	23.97260469	11.71117607	35.06589168	17.35422698	160.1277077	4.209249766
0.006131987	15.43303221	8.778209808	19.11543874	8.276130339	90.70756713	2.64275752
0.006871229	14.36793958	9.556096389	23.77175614	12.62952465	94.83202166	2.821136305
0.012540878	20.57547236	8.093121822	18.52771891	14.42414992	109.1738088	4.880979866
0.01361744	22.43891444	9.548694803	40.97216372	20.30908707	196.9330079	8.997989094
0.010327543	16.36660706	8.283157411	35.88446146	21.68564427	162.1424827	11.18028749
0.007103076	15.77955401	6.812871876	24.40072238	18.55052378	153.5463269	7.406128461
0.013388448	24.12355644	8.877424653	51.08522659	24.28068987	226.7233096	10.64941667
0.013412009	16.5854069	7.855302686	30.25784563	20.21461157	156.4296396	14.16616815
0.013615056	14.02073328	10.89232966	38.30369259	25.48498394	177.83205	6.671176078
0.010950319	25.44055102	10.1373649	26.6384655	14.86073816	175.5003334	3.262211999
0.007405305	23.95858472	9.710253562	39.38444653	27.67412129	257.1613032	5.083799337
0.008097149	13.26323332	9.595338772	27.16780942	16.20536126	149.234542	4.445663844
0.017182412	17.9718087	8.166255055	49.77507245	21.76751387	205.956324	10.17696498
0.018726796	20.79584087	6.364562147	36.39080375	43.39376046	398.0427775	23.56354221
0.007468853	18.99536445	7.973231496	26.36386601	16.07177804	171.8663393	7.627695462
0.015436834	20.07525993	7.483297979	44.66581458	27.96113506	281.4663861	13.24458978
0.011111158	27.56152294	8.698206782	34.87370209	20.98486796	215.3786783	4.349792826
0.011081622	21.32085489	8.768379928	33.13277007	16.051449	185.9156051	4.585594189
0.005538417	15.27178912	11.96664857	16.1124812	8.457349641	82.97987599	2.983701798
0.011854479	17.66333411	7.341453623	46.4578173	20.58884113	222.8878495	12.74567508

Daporinad_1248	BMS-345541_1249	AZ960_1250	Talazoparib_1259	XAV939_1268	Trametinib_1372	Dabrafenib_1373
0.010475548	23.06807259	16.24696655	24.00055116	99.66824858	2.482505292	149.6452662
0.011097295	58.36498604	4.9435626	19.36132297	81.61496172	3.260415398	90.56723742
0.02105993	52.33023873	11.86994442	58.48371621	119.374033	11.56736854	238.3594125
0.005609366	59.45993604	6.426653466	13.78914904	86.11516475	1.059216018	161.916165
0.011238596	85.35767791	21.25779003	107.5476463	174.7369175	3.414580827	135.208616
0.00608573	24.168718	1.82762311	10.56910077	51.41535198	2.024623998	64.91884861
0.008327066	25.43765646	6.606328582	18.7807598	81.58036571	0.101379952	44.97092663
0.009380548	43.84808495	14.17524831	51.3808765	114.7871218	2.348801237	136.2794039
0.037110831	159.217353	13.92082037	63.5003378	116.8075687	2.254120971	185.2292448
0.007616163	20.32331355	3.167200815	13.8452545	52.6706	1.972446242	61.47610827
0.010825743	24.37253027	5.545762552	15.98353958	76.53816533	2.905131826	119.4841752
0.004467531	21.07590612	10.43012165	42.10937037	72.4237575	2.131051771	124.6321802
0.017283005	17.97209769	9.232691317	39.65985587	96.13482687	2.237055087	110.2914735
0.008006897	73.64537405	16.5405168	21.87000868	105.6096449	8.138014137	217.9641405
0.025272217	41.03124951	4.810449517	40.75244074	77.72803611	2.557750411	106.7008721
0.007787578	34.86812802	5.395570758	31.70682382	69.78885921	2.580430603	155.603593
0.003128814	12.17403551	5.002311414	9.675098692	80.00553516	3.240771026	61.43172434
0.002902108	30.29638955	40.43507255	39.55094313	210.3939501	0.054195092	103.6304846
0.028263017	26.74302998	5.695763678	45.54631976	91.86145463	2.009113464	100.605273
0.006121112	15.79036796	1.757920783	9.903069044	55.81515629	1.034909892	96.5217079
0.012519911	30.20957496	8.130876008	15.5982283	77.51688897	2.14028123	110.5806909
0.026368055	30.13567746	32.07484623	47.10203539	120.5851356	4.611815762	162.379797
0.010603349	19.76769316	5.045668011	17.95716516	74.57086014	0.421070617	80.04370736
0.054535399	64.31125309	14.61814772	45.9378526	85.53143751	1.186187062	131.8159899
0.016135595	43.55041455	5.868822417	28.13509644	71.98186903	1.886861288	116.3566389
0.013349169	38.63688285	7.520229778	33.34673479	96.86602977	1.786940511	133.0510053
0.015752542	51.77602485	13.80036929	67.42038813	101.959671	5.357363135	248.6734586
0.022646985	58.44583487	7.685503769	38.34242386	86.57944501	0.644700551	152.6435681
0.028494013	37.50280358	3.900638416	21.47739061	64.11838997	1.421483133	65.07709313
0.008863606	15.27688482	6.461952463	26.1254854	61.06775117	6.892781873	145.7327667
0.008236146	19.40481764	8.490507998	30.06685664	87.6266584	0.91729605	81.32408675
0.055984428	23.35705935	5.452345678	39.0915495	76.72551048	2.183526428	166.9283568
0.034910094	33.6543973	14.74158269	88.54844417	125.6522921	2.359906387	313.35921
0.006806565	20.52450297	7.411124688	19.25377971	107.839003	4.990238537	73.83885252

0.060199088	54.06530497	16.02449641	32.89838809	94.38220273	3.015610514	75.82844762
0.012734699	23.8996211	10.03677882	63.47271312	114.682189	1.887818331	169.6813497
0.013406052	18.02154992	2.479122372	12.92714294	52.69236467	2.026840019	56.87122997
0.03152413	33.17070679	14.25617048	72.30822099	99.84371304	3.10924163	199.2679413
0.016199495	34.4995545	9.803032507	45.60862266	108.1210038	2.101352333	100.9724971
0.002945303	14.55198134	9.623249626	59.21428234	110.099929	3.770986607	129.8802883
0.007424522	27.72172386	19.84781369	37.07860736	95.27386738	8.203681838	167.2139433
0.013033859	48.87488494	4.964086422	27.84981591	91.00535789	1.488042538	83.88794819
0.010585861	23.95461759	10.0630251	24.50036249	70.64551527	1.030667501	85.40908287
0.00738542	25.23998974	8.731259266	52.40039848	91.95739679	5.378863752	182.7297993
0.016132598	34.80342982	13.36247404	10.31567142	79.94115634	0.612769647	58.86310644
0.001221246	9.616572846	4.03996393	35.48251908	92.28890651	18.71685598	53.95040116
0.015326385	26.42961471	4.078716598	14.06973756	58.99061177	1.775167912	62.33008524
0.011280774	23.95917532	9.264243749	29.7320443	85.71530784	1.624046706	166.1281467
0.012495702	25.27195496	3.821712364	13.0947649	58.18425855	1.501209581	71.14424933
0.036197954	21.55112993	5.140340604	29.38531571	79.93209592	2.447110347	75.95383335
0.007499453	22.22807	2.566785338	4.583118364	44.01479415	2.016936844	32.73037754
0.021487307	33.49679652	3.819949132	28.93510536	78.86557307	1.691735082	122.6188555
0.015616924	14.91535138	5.066228885	73.53291089	97.41665566	2.894977378	159.8451834
0.00886619	16.39536355	3.689928278	17.53466121	68.44339105	1.248069347	75.70653302
0.004718713	25.42231647	7.277472545	17.70435505	66.64165645	1.075450042	76.4586495
0.017367787	37.4954316	8.747631987	35.69939198	98.63325562	1.232171126	181.2687976
0.01217268	26.43458807	5.196349473	15.98675657	68.99448016	3.014072333	71.80792655
0.011707495	21.25909513	2.623877895	14.77531814	59.5277404	2.124894257	71.72113515
0.027091767	31.57507451	24.60305857	34.66526552	97.5257513	2.425443999	152.7855286
0.019222145	40.77946109	10.68818494	29.57835407	100.4369206	1.486180113	113.2600423
0.018683276	83.92922039	14.30894312	63.64449783	111.424705	4.783545052	273.1545907
0.01000928	23.10065562	8.193101233	25.25985868	87.54184349	2.869550949	165.6906318
0.022492111	46.33431589	16.73680784	119.6307321	133.5801119	6.442045929	269.6678784
0.006207127	32.75068643	9.627381739	31.03760976	90.80437793	2.93018182	61.88029226
0.009110078	29.27380695	4.450238887	12.33077451	61.03820831	1.009449188	73.79338105
0.009433858	32.51793578	3.340050633	10.35022419	49.7883949	1.368385351	43.92421218
0.011839426	19.23453125	9.870282892	53.67142182	61.78113756	0.452616836	135.602081
0.032098053	210.0525622	29.69039157	109.7985536	148.5501684	5.653577486	179.1584254
0.026664836	31.30759237	9.022342679	57.67720704	101.7304198	1.80417078	128.7078897
0.008022169	16.31149921	4.924144498	13.72938915	57.38907348	9.150590556	114.1858292

0.026718779	36.2456856	6.746779133	58.96770123	107.6952044	1.417398631	110.2453828
0.024067036	18.5517126	6.327836483	13.38136032	81.11812227	9.2887735	99.04827418
0.056218315	32.77806668	8.871461689	44.65460656	77.24465157	1.042336747	127.8142335
0.026784501	168.8868517	33.49021201	50.61264416	162.4110219	3.2348796	308.1403636
0.022047099	25.25201682	3.982398688	26.27872133	62.37386123	1.976887271	75.37503878
0.011386925	28.98400735	3.584108417	10.59589474	57.31771859	1.179551225	53.78825615
0.008451218	17.18745937	3.680076848	13.65451917	47.78427125	4.769398927	75.20370488
0.016815796	33.51988101	10.91055292	16.46508464	87.24769292	0.896327674	107.8259288
0.003282487	23.82282258	16.2273117	53.24041476	100.3482583	9.589556114	190.3202263
0.01013403	24.19617244	8.113956841	40.1626626	82.53232294	6.254898491	118.1987961
0.016095471	47.89295707	10.57282653	49.34513871	91.69281039	1.373162086	137.5779776
0.065301487	102.5131564	16.0669853	59.72008742	110.9466086	2.601858349	190.880928
0.011532604	21.26288202	4.102322879	18.63315151	61.9056247	0.6848449	60.37824066
0.007128575	27.39303889	3.248058283	9.989996981	56.69869213	0.773717529	38.47867792
0.010493469	43.98620952	11.04914688	26.79535285	77.06486909	2.801768378	231.0766065
0.020017076	37.22936996	8.110114778	22.78678525	78.66103869	6.38628873	155.2397767
0.002728876	26.59340708	7.530404781	7.368572859	77.87940716	5.409286692	73.12037328
0.009678347	21.71490897	5.000585028	18.9357404	58.46227083	2.368105066	92.62950347
0.009973959	35.35618048	13.44733807	37.98456376	151.6326333	2.294788726	295.0978861
0.014351728	23.53998748	2.270837884	18.88789339	75.5105914	1.33671648	94.5577333
0.0155555	38.71837098	17.31855793	21.69091965	135.1965387	4.305258925	99.66508519
0.006768501	15.07667623	4.689072882	12.34513535	71.56428001	0.505755852	49.54326846
0.011371048	38.23517598	11.15814054	29.56552036	81.84713758	0.493217424	71.18254797
0.011341143	21.93512509	4.844699056	23.35981369	62.68519712	0.860368593	130.531858
0.005036516	16.18836814	5.563980831	13.09261654	89.15056605	0.986886707	96.06982185
0.007810568	27.348037	5.425393202	21.12003957	75.69315864	0.631684871	65.39432284
0.0120736	31.65731299	7.162620941	10.89160624	70.2562185	2.230266337	75.96240306
0.020593655	33.07096627	3.020167916	12.96940147	73.91629791	0.081062653	34.25798295
0.01613244	43.52379133	9.728857084	37.71857661	93.1248794	2.623923917	136.7660684
0.008918409	44.24405852	8.904494334	22.4779917	84.15310136	5.815140749	161.6700403
0.008944283	45.90278374	15.31051811	35.94781852	118.1287819	5.68465489	211.5892193
0.023512858	46.89433368	6.766140647	37.26183307	70.83285273	2.384808949	152.4012965
0.000808996	11.65848858	3.389261125	12.05177457	57.87104157	10.10781304	69.46380735
0.004746339	23.93913421	6.2757811	16.00524886	78.24835275	1.357928694	94.50442255
0.003858622	14.82180956	6.093350348	24.2013093	66.200654	3.254569407	73.78319338
0.026921554	25.00166722	7.520793325	38.91645209	76.95192226	1.402412961	99.77390795

0.020390269	59.3408181	10.63106139	20.82473222	80.65766867	3.253426166	95.59437295
0.009323439	49.20019069	9.540199484	33.84072959	105.4112832	1.805181209	107.4613969
0.011633637	11.72744753	3.556347111	16.60102734	48.3422332	3.075397988	63.95061908
0.019256927	19.8016188	19.20385008	61.60230012	99.94657306	3.105390559	265.3697651
0.012313732	26.82060117	7.693145078	49.57923351	89.65317508	0.429576074	68.71869142
0.008444976	26.22504106	4.296491488	19.11095873	54.85291225	0.288540139	75.21698255
0.029632001	77.09848616	11.3417134	80.35618377	116.6578939	1.862159969	302.2725781
0.005686782	26.61017409	6.6157	18.9193124	89.80512888	1.209012438	101.7326052
0.014457022	70.74261942	13.91115058	33.4691943	104.5076504	1.521833355	162.2128402
0.012856743	37.00185927	4.853680311	32.55493565	72.32350798	3.969392891	71.89408232
0.003745013	26.01936635	11.47220748	13.74889147	85.45944544	1.807126468	124.6894089
0.014467944	47.50035977	17.41558657	36.13734782	114.8997124	2.782034583	169.2267691
0.019497932	53.9658483	6.298781153	50.90186538	103.2376079	0.395360509	130.8502455
0.012212841	24.65032837	2.681362868	18.67025727	70.89804379	4.137246294	80.73781464
0.001408795	12.37797334	3.450648819	3.313791975	79.88464294	11.66904599	85.94798068
0.009007356	20.58710089	15.51544862	42.83168858	125.7210165	0.06397232	55.02090015
0.014582171	30.16969301	7.697497946	35.45069865	89.35283712	0.776662339	146.8812167
0.009666553	21.86031566	8.780170723	32.11124983	79.69551963	1.161187969	107.7297037
0.004622284	16.27898145	5.489369654	27.93799016	97.1563795	2.934527043	133.4601471
0.015851257	23.28453979	16.26001154	39.61001672	118.3780809	0.584329251	108.1314122
0.012430277	29.40293616	11.35426535	47.63461934	87.57264465	2.133688825	138.0225371
0.013622394	17.53915353	5.803505271	28.34309215	75.64417972	1.066253684	79.9999378
0.006804129	16.23756851	7.576586728	20.29575413	89.70314364	0.658499401	75.11982885
0.026322459	24.30070888	7.547948441	42.18246663	108.4770083	0.492593724	80.59744408
0.010568068	42.24206116	12.19163586	18.48950742	86.42500272	4.533391184	135.2885616
0.018998643	29.32206542	3.722731712	17.25084401	68.69278647	0.493258995	112.9217281
0.05586633	110.2577401	19.40856247	87.27840554	125.2769776	1.338008632	154.4547391
0.012849132	32.42116488	4.138058705	17.07064077	70.37590996	0.657892876	55.14145361
0.014907621	30.02745297	6.007436157	19.10083658	82.15928088	1.346685041	83.04566409
0.00652554	84.09525364	18.275608	43.28171123	79.24109439	2.533474634	220.7313957
0.015283478	41.0880267	17.82684122	58.36796281	121.0772441	4.385229414	185.0125159
0.014102103	42.35782362	8.736786203	28.85506234	101.6897065	1.423797623	222.3726558
0.005045487	57.36688896	7.308212591	6.588803874	80.3056658	5.299899144	66.14965875
0.005747735	29.03300718	10.23061078	21.31104483	81.97805614	0.366723494	67.99801491
0.010487463	27.9395937	5.650291095	33.10272061	74.13130945	1.117284587	125.3384416
0.009386825	37.36453488	12.07170231	57.9642139	85.48226215	1.152274688	223.4561681

0.015801095	31.35366383	7.298176574	35.79253154	93.98228908	0.120270499	92.85146431
0.031561738	45.90426398	5.783033755	46.87991659	98.85433947	3.109402328	170.2317342
0.008604042	27.29558416	10.68684061	65.11725411	86.74535664	4.496519554	288.3715426
0.013734697	40.24689806	5.624441741	28.92634172	80.51377432	0.742179547	133.3946104
0.010545161	46.24163297	43.02372396	72.43264019	161.8404165	0.45499061	201.955214
0.0064831	36.59257106	7.688122712	16.32188346	67.8867318	10.53242424	123.98255
0.009915071	53.61279438	14.29663721	29.52163071	99.5614688	7.106615762	125.0226667
0.0135394	49.70703368	4.202254888	14.52111148	64.84215418	0.900417438	44.73511937
0.008176634	37.49108222	9.206605973	21.81251385	85.32911042	0.056859308	23.07149715
0.016457435	36.29056506	5.303564781	18.36763981	79.70606808	12.02221024	100.3652571
0.014993586	54.44080132	12.71972245	44.47275649	110.5045239	4.809734401	195.4423804
0.028945546	23.91863648	12.65376983	25.01811848	66.91090682	0.298192775	75.29904608
0.005465445	27.793614	4.715051198	27.2597991	80.38841285	0.916410491	144.962838
0.013901241	95.12094484	17.01954011	17.43202265	80.76855713	8.235175409	168.0266676
0.004259718	17.9807931	6.053923269	15.77479971	84.49253101	0.406898996	44.41804209
0.021554267	36.77680938	21.23614008	45.96737904	98.19177441	9.209332196	106.047281
0.001770965	8.692423489	4.396524425	11.88669788	56.71117364	0.571108388	31.50613316
0.008590174	46.12466726	14.43386315	58.20067934	96.12643039	6.402382438	220.0907943
0.013932273	36.6683838	7.880134092	26.99360914	82.71206494	4.038303932	162.6188048
0.008343627	26.02198684	5.259494558	8.551942896	62.8509098	1.116728485	54.31476412
0.004523747	19.06045896	11.7687763	58.43352755	94.82876574	3.589706225	105.8416356
0.012705114	24.395608	4.581209398	22.86935941	67.88739866	2.147865041	125.7713406
0.038230027	39.93513535	7.803312416	26.25885286	79.59661232	3.051559815	86.96024443
0.008205655	22.82003749	6.379477837	25.9618572	70.6514834	0.688850027	83.5807914
0.010421004	27.50527992	10.63724584	38.93633847	96.29217613	6.56377161	177.5203944
0.021534126	32.55806349	10.11943339	50.67514051	92.92797351	3.664277439	157.8735518
0.006864626	27.05480237	5.039958332	20.60946584	86.33533001	5.517178531	160.1056984
0.02644482	30.13070727	5.729682958	39.5808826	66.22883527	0.730779981	146.3581064
0.008336046	16.85960572	3.376895764	19.26663507	64.14862428	1.650139726	83.83927789
0.011870403	18.19636545	2.670646646	26.92745518	52.92471822	0.65341113	45.12559323
0.009531287	21.14138564	6.276361677	13.32050053	64.41970705	1.195523286	65.58841126
0.025981876	19.70123092	8.957450623	19.70433108	50.83483385	2.218941818	91.19536206
0.014214493	49.09712661	6.118467639	8.66154238	77.18988275	0.313182747	25.8295182
0.033571542	55.43343577	22.70195823	69.80189939	88.19112247	6.604378773	222.7462429
0.031383449	49.3831504	3.308252927	18.37105202	65.54715871	1.263711187	69.11706484
0.009022666	21.85389384	5.210311379	30.19079424	86.39302378	2.310326743	136.2417123

0.007033101	17.3499149	4.694531872	11.54111153	68.63305616	1.783765372	88.19203578
0.017622591	24.58631598	10.89625166	26.39462907	133.4911682	4.428657805	180.9680404
0.00555923	32.79912687	5.185207086	18.37289261	64.6755128	1.046208136	59.03482814
0.012616228	27.79162643	9.913043226	51.45973546	98.93033165	1.067727034	97.43179047
0.012552448	34.86057421	9.369226504	24.68045956	82.3370358	1.571439928	72.25818264
0.01246072	53.00570931	21.28422692	76.0821951	109.3436611	4.927607384	182.8337437
0.01898686	34.15993549	12.90160349	29.57592674	104.240746	7.573354128	235.8757482
0.004545944	25.9199327	10.11655433	30.75809477	82.63986324	3.887439135	100.1559251
0.009473985	19.2936535	5.14973685	13.79149463	76.09902075	1.393248648	86.91170521
0.032092768	30.12078032	10.16760283	22.64478972	63.68527182	1.335157123	80.15455723
0.008469792	15.67247467	6.254094944	15.51258883	67.00921438	0.664191366	63.93241974
0.011244976	38.84064967	6.027039916	23.95564477	70.31158078	3.554309599	141.1956429
0.004061663	23.94987172	11.06528563	47.00028903	115.9695317	10.4990767	155.3465153
0.015066812	47.2261169	6.200947379	27.56970158	69.12200413	1.290006945	75.97722368
0.004362499	17.64366932	11.17278918	22.78899545	104.6101926	4.639768509	34.511577
0.009226523	35.81185933	4.214814852	13.80956375	57.83165741	1.610006568	59.19578229
0.011676712	27.19228278	6.291221095	24.76767251	76.00176056	1.603546287	126.2919069
0.019145416	27.66117802	12.3820408	23.02563312	98.404702	4.253515899	154.14486
0.011174829	26.05091089	5.102163269	13.75753654	63.09111633	1.160212344	96.02619878
0.004650905	21.42101653	3.821912143	24.3572426	52.36526661	1.466186536	116.0352782
0.011685092	25.54980678	4.308427343	24.7729592	80.02017624	2.05449333	123.0427873
0.026369642	62.70715129	28.24970088	68.87480767	136.0493193	6.408029441	280.4451954
0.032218397	33.91282858	9.580471148	16.51968985	48.79302337	5.42825653	89.82797557
0.016296089	32.27332719	6.346546001	22.84576182	68.46133437	3.040041571	111.0500393
0.016777804	62.50176616	20.31864886	52.79517851	135.1925583	3.662808652	213.9883229
0.043287196	30.23722437	10.52981863	42.40619324	76.49785569	3.295481826	186.5175968
0.013903631	22.67519537	28.49056129	35.37486348	115.7845709	3.544563493	221.9446095
0.001219027	10.37231584	2.094623565	5.503142683	73.70377544	14.55312998	46.96777381
0.004627532	17.05838005	4.332765777	8.901902902	60.98120402	1.226659609	68.19119995
0.011728656	16.35710881	8.305781255	18.30014331	84.49364524	1.022499108	141.8011052
0.010666058	19.15632792	2.683320151	16.22237533	62.89774703	0.330133254	58.37739187
0.004804605	21.83528469	8.315553519	24.57553172	95.69961517	0.736126631	73.32326975
0.018676228	40.02615748	8.852732793	45.43291572	90.2690312	4.047773719	142.947233
0.007142614	20.21948858	6.05674403	12.48026603	72.02742572	1.822640908	73.79304965
0.023387364	29.01921333	21.41276959	29.63666119	79.65731811	1.743576726	120.4783663
0.007695024	16.668262	8.82826028	46.37103918	83.69879098	15.54338948	160.3101075

0.015689224	51.1594863	5.972666595	52.46806325	93.0869984	0.759627132	112.5906081
0.015011594	34.06757185	6.459559475	75.2306956	132.5545806	2.289087709	160.7937057
0.005581386	28.34745433	7.708041429	26.04611158	78.1153695	2.561289952	102.2825256
0.009157784	26.60838952	17.5329544	33.07611239	99.96473383	0.948259331	162.396656
0.003459459	11.67342484	4.822537922	8.601423984	68.19818917	0.578686356	58.16755214
0.010197236	41.52131153	2.490617314	9.896181346	50.81985832	1.052120525	31.50942066
0.009957806	25.48111419	4.180927019	59.73525163	96.81252789	2.471252731	202.007414
0.056314348	29.35679318	6.505665418	50.9613058	46.72617161	5.00374545	121.2812182
0.011923078	19.28570938	11.19440757	24.89176701	71.48946196	0.862824447	84.56547489
0.007533209	29.65387983	11.47928293	30.8425908	98.39034462	0.67976829	135.2329405
0.00814257	84.34602991	8.066820022	45.27203011	75.21819873	0.483432461	159.3056463
0.108256428	31.32502323	9.554535711	31.21391134	81.2427614	0.458235264	126.6998581
0.021370322	35.81700878	13.00483783	40.64743075	81.04021494	1.895588291	171.1452563
0.01011081	19.04574748	11.39921577	32.16649269	84.63208683	3.863436836	224.5915865
0.00483326	14.21345439	2.732382096	7.631104598	57.4373149	4.242937021	41.30552703
0.033273881	43.73734924	5.069084136	22.020787	77.40751635	1.526600145	127.4258513
0.018308933	21.42143397	3.642681489	12.0852647	61.08684061	1.293451215	70.93730116
0.028463312	25.56542789	6.194633354	37.08067299	74.50727917	10.07461218	160.3018765
0.012417629	37.69971467	4.873736515	23.30265052	78.3465447	2.257044953	169.0946422
0.036399235	31.29305019	9.900449966	21.03116268	84.57660463	5.776300182	100.4858631
0.007710084	24.08727234	8.672442093	15.41615234	69.27274455	1.019708307	75.68125849
0.019418648	40.45782927	4.693783178	23.76831945	65.59893509	4.482692369	71.81664398
0.009387229	22.55048174	9.294452863	44.49720222	119.8062269	1.434927532	240.638483
0.012891455	27.71649113	10.60628192	31.24906676	103.8582413	4.556927811	178.5327983
0.020058732	35.52655809	4.118008973	10.88720317	65.90311078	3.848498144	88.93919798
0.051788873	110.666547	15.67662453	59.04930314	118.7568284	3.59995412	219.2723326
0.011340179	23.28773199	8.077076814	12.5804877	67.91139924	2.227942422	102.1300905
0.008876812	42.46274191	6.200717399	34.73521418	96.42032609	1.120756394	155.3562577
0.005081822	17.90788681	5.23045736	17.24165902	67.80471575	2.64671769	99.39678505
0.008393876	21.24047414	4.830284344	23.53004343	74.4562334	0.586890707	91.15100106
0.018942044	21.5313729	11.95740579	55.57470751	75.3207548	5.828186911	193.9570582
0.00381569	17.28964831	4.208782088	7.898120664	64.60311394	3.154590621	26.07026301
0.007172464	25.90210998	5.029425881	8.375972406	56.48733986	0.879379105	45.70573654
0.012137673	25.54274507	5.175817492	15.32914682	69.52730684	1.694312394	64.11574636
0.009265434	20.06669718	3.675869639	21.24460205	80.5760206	0.986370907	71.79593753
0.018814049	78.80455909	14.08891357	22.50570295	84.53320412	1.151877468	121.6005809

0.015112677	26.51441029	3.936956376	12.5675175	64.40579326	0.784974227	54.07827315
0.037464146	37.30751678	13.59809809	34.15793155	98.61420273	2.786466991	219.0709925
0.011807186	19.16648425	7.511393724	39.98829495	102.5419734	1.017079913	183.849102
0.010991546	28.77996306	10.59677895	31.57941675	101.4706578	0.653139914	128.6592573
0.014860054	28.25612481	5.212148625	28.46949602	79.17817811	0.779174932	74.44498877
0.014543504	45.75313683	11.14525369	28.88819995	93.47347707	3.700620497	144.2580887
0.047214665	23.62078474	4.786595146	28.7746445	71.90196386	3.568074419	142.3661591
0.007243891	26.92187706	5.419501617	29.36630844	73.60799758	1.977335625	135.655483
0.01144279	33.16502703	17.09704608	29.01608893	90.18332888	1.00483993	44.86161002
0.136682531	46.92623937	35.9885614	101.3189954	159.44295	2.546021468	163.2821124
0.00853708	15.01193925	4.950917982	21.27537051	61.34292724	0.860171444	121.4558992
0.015362833	54.23148513	6.012681589	30.67007717	79.22821816	1.515308276	120.2465416
0.001769133	11.44396463	5.492690799	10.60541272	78.77965158	12.9738478	60.01336952
0.022666346	63.78356383	16.88497546	43.39240225	124.3655985	2.800447647	216.4181762
0.019876244	34.32384055	7.795344106	22.60706307	76.95165021	4.298057083	88.09026723
0.007311856	11.38769556	2.121689936	12.90763936	58.84606178	0.827911443	35.78426021
0.009440232	30.34555178	4.814820076	20.16645013	67.72809073	1.274910899	94.86552038
0.018498836	23.15061114	7.016553342	34.37268056	83.3577955	5.062317481	89.33399859
0.0033622	31.81929527	10.94603778	39.95236265	110.380227	7.444446018	92.33347767
0.006252238	39.47502893	36.72292662	27.93774859	135.2537975	0.102754561	68.96861752
0.026178814	33.11000291	5.663656611	22.35944502	76.91183015	2.197975826	126.4981029
0.017179437	31.27650184	6.917048973	25.07927204	75.00960792	3.563303191	97.42876611
0.004402276	13.14884132	2.948889146	27.09239508	116.9410575	0.048096654	25.91024547
0.021812672	18.58667872	6.513185227	32.37040638	80.13497478	7.958414863	171.5245383
0.017532038	19.92707356	2.966947845	8.28560026	37.97290192	0.263492811	34.03402109
0.005842037	28.02083572	19.77296142	50.49668529	136.4485519	0.025897655	57.18456593
0.008708391	21.58746564	8.033079588	36.00772058	92.93721509	2.587015139	216.5314634
0.022649454	44.31520516	9.39946408	53.8143317	107.8178124	4.654580092	158.817713
0.031880952	25.64727993	5.588936722	14.92004406	66.10279145	1.301821535	106.0013293
0.004169668	37.71820263	3.808509369	10.2779003	71.33727407	0.756298758	55.05558893
0.027588887	30.17820328	8.357091544	60.77581463	134.8859612	1.691265134	170.4397981
0.010114677	32.08530156	7.345370058	42.24179033	87.02756972	3.336809393	162.7069045
0.018931693	42.83863149	10.88524099	65.18768211	110.0756147	1.337842811	259.9322434
0.012477115	22.37897452	5.97476198	20.04382352	63.98491357	4.554208953	129.2603852
0.000681276	15.23113099	3.606147009	3.942015547	67.56503232	4.326211949	76.64299117
0.010607568	27.72938984	4.248257102	13.64294352	59.77886082	0.431896495	44.88145007

0.008813102	28.90078253	9.851727227	27.24358675	93.47901099	3.307805301	123.67204
0.005751014	18.05811936	6.996624887	26.55560959	87.34893781	0.632119762	84.30279031
0.021326452	37.15191417	6.824994539	30.56672741	88.54352287	1.027237448	132.6463731
0.017926227	18.35400519	14.43759032	31.45031294	99.56273568	0.576397267	132.2727065
0.005825693	38.37853997	4.153278897	7.190915248	60.88349188	4.215280177	35.99579841
0.003580086	13.17753012	7.476521562	17.57299221	92.79643503	1.886014097	95.93901949
0.011564225	56.5602621	4.693573602	25.01989275	93.05385206	0.817463478	147.5274182
0.013611088	25.26190044	12.03858352	17.50420333	91.90762525	0.355534083	53.1610827
0.006978404	36.2999854	19.49476645	24.66383863	119.1183022	6.864607123	230.2678898
0.008600623	17.75296501	5.882396457	12.04505487	66.22570238	0.507345814	39.05652548
0.005040075	21.40338156	11.98641016	12.73242637	86.81331987	3.526725357	94.80694057
0.007514492	14.04103288	2.751538289	10.48197703	53.64821698	3.193550857	89.82461416
0.010547104	24.04720108	6.52264248	20.58333967	74.85246141	0.610199379	99.20545057
0.0378746	41.21193861	15.81671609	26.20572902	95.76072099	1.341442369	128.4207652
0.003481834	15.3785345	7.720446303	20.63569265	67.63853539	2.117667672	94.75122742
0.002609024	15.76894027	4.228899053	12.78639762	58.66539222	1.434577871	26.39970759
0.014367328	33.749409	3.84573218	17.26171833	57.18176115	1.212650771	66.824116
0.008684607	29.01584281	7.914247015	15.46939431	86.73688756	6.481109999	169.7058286
0.068311906	45.45074422	28.64040964	114.7324783	114.4771742	6.353765404	280.4843264
0.007515137	44.71099031	9.359204828	20.50866427	84.06464743	1.247655329	87.87956907
0.006230213	40.04050627	5.269538	12.58797966	83.00929822	5.204773541	44.33413467
0.017268063	29.4759922	4.300952456	22.66411024	56.95541059	0.888808763	90.91120183
0.020603792	26.53617908	6.017341693	26.06124498	85.5920699	0.520175605	103.1097214
0.009582011	21.06380976	7.843129943	21.56688207	71.99790702	1.15603144	116.6252192
0.009275584	94.77819405	12.07988474	26.27436837	109.4374658	1.603013935	93.5192638
0.011049032	16.25468289	12.39884746	25.00304713	73.50769557	8.488437892	128.4394301
0.007003376	27.90331372	6.960146906	19.10621957	82.77953088	1.307634646	99.70281872
0.008586505	23.19895351	12.15970298	25.07287807	106.7157413	0.27599023	57.82738072
0.003286195	14.16626679	7.080845027	7.813720764	82.35666765	21.42504577	134.2565343
0.009695976	21.55390925	5.464052279	16.01878773	74.5174415	1.41798446	43.62566916
0.017219257	18.39567273	11.7920592	21.87801664	70.61846158	1.082255297	117.1905549
0.017408946	41.05465017	10.25637424	66.69682659	96.94406622	0.90636937	180.3071983
0.005701118	18.09024345	10.99454135	12.56217245	78.17563343	2.974295778	89.19490708
0.017805739	29.98295189	6.303316474	31.47775215	91.78924762	4.385552987	164.4829758
0.007100908	18.48063481	7.22188074	14.33900254	90.13241261	1.666346396	93.46084348
0.008142545	27.41215032	4.912165049	38.44946659	79.93039968	1.248693871	96.16684874

0.007889963	13.85777939	4.018905734	20.33529021	74.21751424	3.07834248	81.31207502
0.005436175	25.76035119	8.506197307	37.84874441	121.658137	2.094367689	177.4605573
0.008196595	16.35534518	9.445313744	20.46417938	79.57607977	1.552375165	100.5138431
0.006036931	30.08940579	5.983796417	14.92789607	70.79033879	0.209494214	54.08045193
0.0058225	28.20215398	9.669246025	18.75696283	80.46521851	2.383535203	84.48980565
0.00726775	35.76624268	7.595443462	29.08043443	71.60339232	1.348232851	98.01744872
0.022797253	35.70355132	10.15772492	41.03579084	87.28437709	0.78345959	94.27833986
0.039441199	42.61680609	9.919334634	64.17392716	107.9545116	0.91196212	216.4340512
0.014156023	24.48923861	10.83874712	20.19948063	69.33623556	1.063841115	112.7370578
0.009470175	21.59272754	5.880491152	15.48759568	65.78838352	5.271491282	81.19941505
0.014458401	36.83736373	10.28359755	32.78770708	85.94703936	9.159898535	153.7108331
0.00360548	23.93837785	6.308395893	9.210792077	64.87788573	4.851573123	109.8242425
0.018661806	37.68766276	6.759086182	38.93204583	91.87424928	1.449301167	148.4374598
0.028965449	27.78491449	6.122326307	34.7373696	100.9854064	1.944307629	150.463117
0.010450799	38.17588831	6.698270813	10.66082205	88.5412296	4.748764845	51.57329296
0.014200583	24.99337117	7.385145823	36.36845802	78.88869817	1.527331375	117.871067
0.033509302	51.59676952	5.97568233	40.83817284	96.65797435	1.506963505	123.1345533
0.006573101	19.91709164	4.974029369	20.36516891	81.26573631	1.339517311	102.9525248
0.008739462	21.34082024	2.114165511	11.79885276	50.31239115	1.04560742	33.07672159
0.001710157	7.131100282	0.689421847	2.514622041	50.5996565	0.964350762	7.452249478
0.005926311	28.81823937	15.99074145	28.94489333	89.31023423	3.963698767	101.6460494
0.023951396	22.52404091	12.34844011	62.78384491	119.7613265	2.933408438	165.2566457
0.012924757	25.37195625	5.687208908	20.9820533	85.45743641	2.57432681	42.2515052
0.015660366	53.98978725	5.75414808	45.07195736	100.0090915	1.579522573	129.8714325
0.008986015	21.15664882	12.88898972	44.90932618	107.5974285	0.91185819	105.3200224
0.008534265	16.54653836	1.129509935	8.013086568	46.69154554	0.255395686	45.8782795
0.020528731	34.53082899	14.15455694	57.52011131	100.6524	4.714858521	157.365796
0.008774374	23.81181875	4.69190592	19.85422796	62.40267817	3.479162572	105.1585279
0.003177954	25.75055847	6.58160888	12.51975568	79.96567589	1.005039528	61.88106979
0.009852892	13.26769599	4.318801176	13.80351632	74.57978639	0.37874783	44.33526709
0.01645526	36.35416394	21.82267622	37.74075726	105.5735917	0.867944255	98.66977745
0.008184949	38.90077707	6.664109135	33.18361703	101.681772	1.528564742	207.3440037
0.026805201	17.06537292	5.252407796	30.91593832	68.97374914	2.61905817	140.0838252
0.016273302	19.78611899	16.94038307	37.04293939	83.92439034	2.128300511	152.8113433
0.017867607	41.40866808	8.177672966	16.69652702	91.44589267	2.039584742	154.0462909
0.004844566	17.26655453	3.442440669	9.5114664	57.50788438	1.269595516	107.7795824

0.027694785	83.23701307	7.57736475	27.65915457	87.00611098	1.242078742	81.41810855
0.007483612	14.80562328	3.842668725	10.58269493	63.37452384	1.268747108	42.48748656
0.042641677	38.32943628	12.03741638	42.63801209	109.9554453	1.358568051	115.6165974
0.029900189	40.38573133	12.73996799	33.35349127	118.018116	6.327128655	148.2405053
0.012160864	39.8264645	7.602718032	62.41953266	102.0813187	2.395971352	116.543902
0.009411921	21.78691325	8.684579909	39.92889671	90.35312788	0.498828156	170.3040746
0.020920793	34.9662596	9.35296021	31.42475265	88.49762807	2.399202804	158.6438719
0.014702303	30.99690751	21.75503678	27.42072794	113.9536066	0.033212852	58.41468748
0.009487641	19.39512199	7.378133948	21.89512179	77.23049994	1.524851759	96.13495469
0.01734374	46.19383692	36.32757475	83.69003167	147.3010827	0.039999365	103.8781415
0.010472963	16.09039084	7.935446524	21.02747449	74.20195562	0.833782412	94.46816534
0.009759901	22.90171342	6.21768068	43.98385029	94.42467472	2.517478296	165.2507213
0.013362117	45.1301501	4.68736687	37.26901842	80.72803126	1.799561063	154.1304946
0.024596266	67.95800551	11.54746696	49.45942875	124.2013867	1.382743935	165.3010489
0.016148661	49.95461784	8.148684831	29.67460661	72.96377835	4.548774098	200.8295292
0.011288122	24.68330954	9.912220208	34.80628571	76.65009024	1.925586362	118.7802692
0.017213976	19.72226294	5.598788525	20.74952405	71.55191225	0.698053468	120.1122003
0.012334819	27.87550486	4.358030926	17.64241509	62.90346607	1.64709191	71.93590993
0.009021311	24.74498672	5.313225392	43.79854304	93.02929335	1.381390723	85.07137782
0.017884873	45.57040912	14.40530345	35.16178438	102.1493776	2.374890639	65.71461321
0.007227244	22.90624372	3.640837377	12.2798135	65.11725257	1.771908723	61.39738491
0.008135622	19.07251977	5.227250731	19.31813071	81.22543375	2.366241068	115.5599892
0.0082194	23.12337207	6.28556142	30.71720665	65.86520005	3.250890585	123.3107226
0.015913214	26.86419682	3.199156193	28.28788283	76.84996695	0.490043723	118.4703111
0.010120388	37.85994743	5.249919848	27.50689329	67.11127537	2.890267407	76.97600644
0.009165175	21.0531926	12.98650626	23.94123664	92.94624394	1.184773969	119.7975903
0.004743583	13.30651901	2.42960072	8.902450749	54.61617041	0.651993812	48.70943838
0.00927589	17.51578721	3.718824948	11.10830698	52.99557505	0.950840636	41.43412544
0.004492922	23.52456283	3.652598327	17.71812053	77.3942688	3.415285459	121.6304067
0.014969995	26.51237166	6.977894267	26.64953542	95.19458426	1.369364914	201.3647767
0.004460094	28.88878383	12.88911486	23.68911345	107.2826687	2.387998664	174.5253884
0.006317507	25.01414525	4.349562324	22.11632692	52.14447892	0.499361013	52.09507318
0.005311571	28.78139551	5.97534649	15.43002357	76.95951829	0.702053619	115.4619705
0.010946607	56.54601834	11.73376507	51.98638384	119.6028777	2.209689528	235.1514251
0.013634922	29.92379252	3.98941073	21.87914899	57.88237786	1.044985064	96.85672424
0.019498963	61.16619232	8.446224155	53.83352573	111.3880815	1.547694793	189.9072553

0.023689959	29.27059998	5.417522286	29.15874256	59.34545785	0.837679025	91.29376228
0.008313901	26.43442328	7.00355338	21.34910543	86.05552571	3.068903775	134.6549661
0.011458857	34.55673763	6.80727511	25.77150459	77.34414823	0.277975199	53.60030354
0.010856753	9.975763651	5.795580058	9.621259999	70.82140877	3.83316253	43.32753801
0.005410793	45.07740319	3.454730524	23.34891098	81.67676568	0.143953186	47.97705629
0.043410162	54.6940477	19.54930668	52.56650456	97.01912249	1.478613153	167.8777946
0.020363373	44.64017662	7.203114659	25.81049428	60.94885206	2.043337418	22.11627665
0.004313241	28.61700077	7.391571056	8.808007811	82.51387463	4.302814649	82.4345058
0.009156775	50.47250778	24.98554522	60.92136817	189.2174335	3.065039851	67.83178383
0.015678387	13.05748709	4.513174588	8.459086355	53.83104886	1.360598415	57.03782484
0.009538387	30.9417928	11.32806523	21.61550415	99.6120218	4.040945586	125.6727978
0.02649682	104.8266892	20.97878046	51.07079443	98.45396514	1.56410564	81.1430868
0.032134902	57.09911138	21.43208559	18.94951012	106.8860627	2.303011021	104.7454429
0.010789283	19.01786171	6.097479917	25.80590765	65.21393278	1.428692501	46.87389664
0.020671107	15.33607786	5.455549878	8.825795915	40.64987356	0.12550716	23.8379137
0.004390858	9.37389503	12.57898228	43.07955462	86.61233315	0.995854477	52.52651271
0.016185118	45.02880147	5.904550601	15.35042466	72.38534797	1.744762541	79.12179916
0.008736594	51.33340467	10.76062204	12.93162617	85.27578008	4.112312713	90.05858954
0.00548511	21.45010612	6.48311421	13.85423564	65.08352353	0.246955176	48.10993266
0.015087653	40.67555933	24.3489297	51.93336159	111.3467691	1.75946696	173.9466011
0.006905175	36.69487807	3.075842743	9.199218898	53.25203247	3.690641357	32.73707767
0.021422262	26.21316993	2.863646137	6.688840764	48.79996869	1.399930278	36.09513876
0.037177071	57.21609896	8.071674526	12.07700485	66.14629258	2.080219852	63.27524617
0.004010159	19.32223898	5.135415661	12.65243924	74.16532096	1.4276783	80.30691954
0.011339743	30.74613337	10.32704714	21.63960182	85.72889852	6.568860754	122.5818862
0.012346249	29.96724589	5.674491365	29.18179856	67.01545171	0.821975807	94.23393211
0.011684499	49.43441464	40.16555871	74.66116977	180.3082184	1.132149292	93.97158785
0.025024782	19.94085637	4.296479861	19.7468669	45.72947218	1.360701047	139.5712425
0.017315176	23.65908786	8.251922397	16.60585246	63.6329447	0.194710227	54.43097602
0.012748685	66.9859419	10.46687902	14.40893487	64.67704751	4.101973972	69.91168957
0.023888268	47.36694808	10.77224322	43.89433846	78.66256746	3.246002715	114.5507192
0.006733741	40.65919239	13.24267373	13.3833045	114.7637772	7.165342014	70.62538324
0.015818396	10.62473415	5.5338467	18.46399223	53.38941852	1.381159206	77.15809285
0.012484348	22.94012596	6.111020523	19.98065661	70.39074243	1.327954466	81.59542754
0.004759797	49.01988466	22.05219556	21.843851	110.8237446	4.176843118	70.92720783
0.0151499	19.27942718	6.870249722	22.40243474	66.34447111	0.509715675	62.95590999

0.013074827	18.42741919	5.968890624	25.98249504	59.39866324	1.85667059	81.08969646
0.007139447	14.93064633	3.131309841	5.459319664	48.33075504	0.575921633	30.08719101
0.017740552	30.39808748	11.34437278	16.02390055	84.21731421	6.415322192	121.3759154
0.00649109	17.1899236	11.89422256	23.82605204	89.72940286	2.217878926	75.24032635
0.021187711	15.70503216	11.46092165	18.07020734	102.626917	1.481981008	48.82797778
0.013677631	31.09494719	18.07072733	39.12016697	109.3055591	5.521108618	207.9957811
0.015685674	31.96420515	5.803443208	17.9578822	66.0520779	1.421387104	89.69248731
0.005036802	19.63543531	11.71471063	24.11042692	82.97154572	0.351441952	82.81749963
0.007717189	27.79035998	3.929858639	12.48704708	57.6410574	3.720614053	96.39827036
0.028104054	30.02920329	12.85875825	32.39534599	79.89360205	2.512390796	184.7015937
0.017370527	19.13283319	7.019879835	31.16885823	81.60681928	0.741101205	69.16478926
0.013910224	16.26656678	7.369294363	20.50362831	57.63176846	1.506953529	47.6178538
0.003919446	15.59233607	13.13246207	40.02769827	75.61041539	4.513998185	130.1942816
0.01051802	28.82643045	8.989560391	10.33713547	77.41961248	5.514737241	100.2365554
0.015728554	23.52017383	26.38784647	59.78644888	102.7090714	6.44826747	138.2134461
0.008632054	17.13241753	5.953720057	12.44015028	62.49457929	0.169488647	39.34857206
0.00692662	21.76323275	7.32022226	24.9263621	71.32239413	1.250415238	102.670705
0.034406885	54.59206952	14.31471043	27.32452461	68.15976064	0.443530162	62.29700329
0.007209004	30.83733576	6.279236484	11.19940216	68.74427416	5.065544089	92.7353448
0.003835375	16.0370657	3.834925538	6.019446159	68.40400274	9.657138941	46.94606626
0.00531635	15.39185532	4.468187537	15.65038154	60.82173088	0.700574566	70.80694014
0.03679458	25.74375709	5.220922988	13.15619598	65.85899255	1.074740424	67.41748672
0.024416508	36.43251978	5.674563117	31.04785407	51.19777287	0.484711053	84.60034204
0.005240785	20.30252868	13.04084136	7.215844887	101.0501099	13.29227462	106.8397713
0.008224539	26.91747697	11.5942387	16.91860057	75.01612618	2.702287062	60.60154859
0.010080646	67.28233267	6.651557238	21.41574372	83.84467309	3.062806418	87.68233748
0.010829059	25.83807305	15.46549719	42.54582165	92.0724024	2.242656113	207.112066
0.00667577	17.75015899	4.64304729	11.239327	63.60188935	0.446662632	56.91095996
0.005990334	11.1038076	2.701709989	4.483372667	51.14387166	0.414175127	49.26524019
0.015149462	21.89169763	4.690094721	26.5868274	61.23562096	2.714539538	93.01257099
0.024629499	55.40824203	17.0807347	20.77029241	95.01542526	2.057602679	103.5334606
0.010943413	20.97628987	8.542846434	8.951338941	65.39891369	2.061256713	50.05637936
0.011937419	16.4928678	1.774812873	6.114363135	35.43970258	0.937060596	33.92371254
0.036696026	147.0053039	39.33055841	90.31743321	149.5079968	9.447792299	113.0367006
0.01650863	19.71472432	5.312223549	20.31286709	72.40319392	1.462701778	70.1753453
0.006305228	21.75222714	3.589584175	11.16357931	46.30442262	0.813723445	59.35712154

0.009126334	35.07994736	11.34419592	11.88977982	92.45800099	1.9829973	66.0015735
0.003447684	9.630308704	2.457560011	6.600462257	41.3391208	0.41071213	36.9114572
0.008100928	12.77187713	9.338320549	31.57879127	81.05772498	0.918444036	133.0599778
0.017039635	23.88078762	13.70858767	22.15385222	92.33655477	0.590818086	107.0703946
0.019496247	55.86141988	6.093408998	12.88919729	70.85906737	2.307664744	76.62302957
0.012812472	26.83626705	8.227952187	22.894973	76.65788406	2.453485422	111.3836656
0.009059549	24.66318608	6.245409167	14.7286252	56.44580998	0.495499364	23.54824346
0.008155009	33.70127269	4.454999104	8.067135471	71.60067145	3.175824997	56.31585711
0.024931354	20.67290475	10.10869255	31.24292864	78.75255721	0.871767953	92.37396419
0.031705057	41.91577479	18.07590126	19.82158002	94.66072148	2.548954134	105.5270854
0.007669162	46.9755728	7.892967392	12.95348091	91.66323592	1.811357301	52.95670849
0.005420801	21.18321883	6.091237965	23.94317171	54.44054278	1.109994339	102.0272601
0.009186897	12.29990324	4.568147979	20.30169187	64.20054533	5.077521035	30.35598824
0.027530272	21.46833215	4.960978082	31.58023799	76.24123624	1.00388064	94.86815552
0.003051277	12.02109558	6.603999379	4.846263466	69.2567379	2.890182152	50.46493105
0.015465398	15.03052238	3.51908382	11.6028408	48.56321622	0.734786522	74.81060991
0.04125946	26.92309381	10.32736738	7.661998838	84.28887002	1.549804817	37.70268508
0.019458519	18.80853596	7.257099787	31.5504084	48.10562455	0.484311385	31.16077877
0.016086247	32.52364503	16.09621219	33.42128161	95.07367677	0.636163316	62.60800185
0.008825076	14.60705618	6.683153511	15.16869918	68.54165551	1.425726188	58.00077273
0.026029717	33.96820937	11.32063005	40.464712	99.80893963	1.37335398	172.3388655
0.021021973	55.45393069	9.110259996	9.915132739	73.1077975	1.410744234	57.71824638
0.021560614	22.49927742	8.264845142	34.68653452	74.98742087	0.636172033	52.61923669
0.015287418	18.96457622	8.374032713	24.98723709	62.92259842	1.0576281	62.27387179
0.033591852	35.06564422	6.903690757	26.30626945	50.76847379	0.387787043	109.2523565
0.021326219	33.10070205	7.609951231	15.14877192	80.18076436	1.661817096	66.79453841
0.027187471	29.99645	10.24520834	20.04350738	75.67585951	1.4806957	126.2109064
0.040039654	36.19701031	18.79131227	26.6505147	83.55005347	0.924793868	99.78246057
0.015226552	22.83139464	8.841267533	26.2490239	81.40065213	0.997129124	158.2206213
0.020363294	25.54617403	12.51997305	35.55617616	87.70335931	0.63918108	109.3268694
0.164446462	20.86526732	7.335463845	19.57798466	48.28487165	0.147440309	75.85403954
0.006707125	20.68011401	5.660384045	21.78607474	62.3748899	0.357283696	95.77231803
0.00425112	31.19638391	4.085853448	5.028102027	61.67606777	5.946308725	63.28489564
0.010057358	24.87059598	7.53079389	23.8569836	76.25805388	0.441277011	151.3080584

Temozolomide_1375	AZD5438_1401	IAP_5620_1428	AZD2014_1441	AZD1208_1449	AZD1332_1463	Ruxolitinib_1507	Linsitinib_1510
793.0988485	14.08940121	282.7262402	7.489446316	215.4100961	89.15775133	169.8383951	34.38299661
366.7046391	7.16315215	153.6120913	7.73973666	152.4710161	22.51997815	141.5864785	43.19220552
620.6840258	27.13547204	473.8460553	14.18226222	256.1791506	86.7710114	165.9886176	78.52230485
642.6122427	10.19431952	64.48455365	6.99378276	114.9696336	38.30207131	120.1397407	38.33777167
679.7615094	28.7510834	298.0866399	24.70778231	264.6270505	98.92972857	239.4538268	65.91350671
154.9247002	6.556896897	137.7624614	4.709235744	90.74285918	11.26198448	65.60644398	28.27985723
275.0550706	7.892325344	232.4293478	5.7117819	192.6696885	26.57467939	114.8506317	47.00189646
604.7707558	12.98057505	189.7450037	13.1194238	284.4597343	76.2091054	181.0022881	96.14796804
884.7862192	24.91195306	428.9862677	10.19994113	303.8929579	70.85275223	279.3542488	74.87843648
177.8038776	5.516854487	80.1002057	6.348314214	104.4109093	35.26319815	64.59354545	42.60210795
337.2500031	6.697034661	124.7717362	8.300496459	105.2535904	40.53076434	67.89488271	31.7598651
360.111894	8.53404565	147.4032987	5.58556979	155.6607129	38.82651016	119.8439351	28.14631945
228.0638127	12.98697913	135.7863176	5.483878787	184.1117636	52.71597395	158.1971396	29.89277124
690.0922858	12.30555408	119.3443108	17.55779654	157.7331717	100.5349482	117.7794921	89.60711004
468.33214	11.35637779	180.5866747	10.4128762	192.4763167	56.06272547	118.3447563	51.59872545
400.856513	15.95570743	250.4458475	6.450764187	202.8384699	24.12731983	98.98436251	44.44610123
177.767759	5.151584067	94.4818081	8.142739627	85.95286901	37.06150712	90.42087916	19.04203141
588.1520259	10.62192682	288.8229251	8.18592531	434.6392701	61.0736541	288.8098835	128.6426811
415.5643206	13.40667078	303.7717809	16.19779696	283.0482845	42.87068353	116.1835868	62.22017014
220.6383628	4.999637672	164.8262985	7.450329356	181.5852127	22.09295561	65.75098188	53.4263507
346.5322272	8.7579628	218.6354133	10.484301	203.8156234	47.20970418	106.1922173	56.47021719
971.1618901	13.92381803	632.303919	14.14760763	287.1499507	82.58420152	240.6229991	68.8393759
439.8196493	5.472977754	203.9119418	5.006078053	227.4171892	26.54475847	117.4101795	56.30720318
669.1033832	15.87218132	328.0568353	9.457595198	276.2164628	75.10554484	243.6453014	68.19392843
466.6542482	7.296114569	201.1325635	6.092003702	193.6326662	30.1931986	151.8444884	59.78775031
403.8801748	10.1649459	207.8331722	6.052554325	221.0769332	38.94938093	156.7002447	75.69015436
786.3108162	15.27372746	326.549544	20.45170224	287.1679636	111.1050725	167.8258923	64.36527575
559.4701314	11.03221487	324.1456967	8.774144859	300.7104883	34.08332178	173.8364536	81.50166806
310.4065045	5.492599324	109.0169814	5.508756565	193.2586649	39.11861135	136.8982252	67.39381713
373.8332241	8.909908339	45.65561322	7.67894535	197.093795	63.74940084	84.81001843	68.96302233
271.663652	11.87589414	293.3846663	9.73224169	151.3019982	33.17280901	103.7551416	35.88035532
568.020298	14.28124172	157.408071	12.70795336	260.7481306	95.74107704	111.0272569	63.70087135
916.019064	18.75835886	317.5818353	13.69288434	385.7078517	73.7747129	213.4237395	76.5730967
296.0972089	7.165056872	111.299429	14.48508354	190.5391514	60.08001585	106.7719552	41.87526339

489.8767194	12.88474739	141.9154883	10.52743699	246.292468	73.50686303	204.6952003	56.42441391
698.5665971	22.07898682	344.3667943	9.372827841	371.9422017	63.1708233	152.221282	84.82425834
219.5431096	7.005969562	51.2781075	4.590378321	133.7974782	26.34560502	79.16086725	27.58340708
739.4660862	16.1681087	522.7308215	8.982228077	360.0959361	114.3073504	195.9873736	127.2489444
522.3252612	9.923067413	212.4992201	8.600015853	238.9496346	51.88255531	157.2243136	46.88030558
397.5455632	15.06998367	239.1018022	14.03373916	180.9088757	55.1240669	161.0540085	29.79564506
497.978024	8.095323752	50.27833438	6.32618577	302.1563453	94.29176885	161.1692161	165.2194209
395.0990492	10.89663862	174.915592	7.1867516	166.3347856	44.86930676	121.7322793	57.90422763
497.7732995	7.007924702	195.1777808	4.043742728	217.9539261	45.19008485	186.0399505	28.49114066
659.0104085	10.53109765	139.473466	8.170593001	170.7650968	41.27084844	114.3161856	28.58755673
444.2336548	6.704720756	161.5648969	5.746082895	204.8355304	43.85186614	196.1180595	34.1459235
129.0211624	8.781396776	42.85063797	19.24005075	108.1315962	23.63355166	59.5311519	13.28972433
263.3740884	6.017906931	221.5993824	4.767061547	153.5240673	27.24204518	107.9807376	41.27099579
529.1098449	9.187745301	176.3797807	9.173144943	261.5051784	50.65402031	134.6600881	80.7670068
311.2398042	5.385798649	158.0207008	5.279902199	113.6758896	36.40788054	83.47432719	38.12655403
291.3195873	7.567386726	137.4462943	7.949153771	183.3891518	49.26954798	129.8292868	32.86699692
170.2546615	3.556283399	87.289652	4.379710624	83.75613142	16.5835796	52.98212712	21.98069041
398.5536026	14.40473327	199.6112867	9.487585335	202.5839251	42.49348641	103.3721529	61.26864399
440.6855918	11.04470302	164.3505188	11.89162725	232.0041948	69.62130033	83.19673613	67.13740894
229.2958263	8.572773204	161.1849314	14.8101347	204.6731215	43.92881184	70.33502481	48.5783681
462.5745484	5.68833412	132.9601795	8.252926873	299.9081065	62.0951552	137.8696404	117.111689
763.7698904	6.66508121	128.0779746	15.85511034	338.0330176	87.84702491	168.7128348	108.6041603
198.1662795	7.759365607	198.1357586	12.92752596	150.1254955	73.87477658	87.66416005	40.43875631
231.516805	6.934849919	198.3437543	4.192585015	152.4784121	22.7409094	82.73577992	28.15488795
966.5710747	14.47949127	291.5631299	6.295228239	330.5097239	111.0156482	229.15602	66.07251013
309.8259475	9.604574455	294.3993103	6.919450806	264.9781323	37.1107114	255.2995853	54.45006406
819.6209594	23.01064837	329.8938695	15.94320846	314.3285487	82.44641814	182.0651839	81.39962589
525.1502526	9.475151802	240.6148549	8.404203866	186.8999815	46.12045732	108.396079	43.41034373
897.9352475	38.91516011	280.0505971	30.81975238	399.9957153	149.083743	225.8409326	56.47142609
399.3684984	5.372663076	56.98819445	16.63238243	191.4272867	99.26354809	139.4905669	48.08046411
244.7536104	6.976660463	143.7478698	5.021535694	159.8334314	27.31288806	113.618395	33.05461689
176.2828373	5.308758666	105.9389518	4.442815142	127.1359685	27.24462806	81.05561374	40.45678429
335.7622895	8.877965203	213.0748176	10.63516356	279.2589169	43.02803585	122.1758604	25.56298842
1307.170547	35.21559106	503.0334638	15.14886848	591.8909348	151.2430159	372.3690584	147.7642921
404.5665003	10.32180192	163.210814	17.10435335	270.9619631	50.64072533	137.7251194	64.37164967
232.0441543	8.244749891	110.5866552	9.428858961	124.4447186	54.82541121	67.45420688	52.57066724

483.6047378	28.99102857	533.3908524	23.87642302	410.9053937	86.51249129	236.9810469	79.45327282
354.7162404	6.153099753	33.83932707	11.01691702	132.4867916	68.86124913	97.14839828	74.38778657
631.9219868	12.35020929	243.3990264	8.186146633	390.1590781	51.36269108	186.3148204	100.2938809
1209.720495	32.74261935	739.4010955	22.54215058	393.8907903	148.9251015	374.2150705	112.779715
327.4733686	6.340577288	68.86459653	8.197401732	146.2145184	46.29148039	94.35927869	67.12365236
310.4321723	5.498323201	120.3951661	4.746891263	122.3990917	37.35116706	95.23910847	42.32769734
274.2319169	3.658374401	61.07212965	8.574663308	112.8077896	46.59780383	84.8984275	30.36611896
618.1967546	9.98167466	261.6103331	5.337272801	199.6233915	49.46755515	191.4798584	41.09321924
492.8859445	19.3487028	240.2190222	12.75179997	202.8359958	103.8190969	127.1052299	32.06562598
339.1097722	8.5894831	123.1507081	9.990335217	165.1064605	49.31077852	93.70863535	35.94702827
667.0685217	9.206184787	132.9784613	11.76013074	358.3088552	77.54765602	181.303385	160.436307
756.7911148	18.34596806	396.7198005	14.37552833	363.8118311	88.55019855	233.901614	119.0539562
269.3612268	7.128715465	244.4539843	4.954212552	162.2232532	28.27377622	123.1742181	33.12053265
247.5286094	4.097190964	113.5958089	3.711152755	178.1764331	26.60643955	121.0726179	54.85304626
635.9772439	13.83736142	484.1244382	8.338481043	308.4029927	56.7514746	152.8265777	58.06859417
562.2231517	13.82789984	238.8860973	7.834665103	264.1197537	49.92471829	147.9231204	49.11062232
132.479385	10.89730475	107.426316	18.19399001	76.60612212	62.14115926	103.9014404	26.4151918
271.6628102	5.912476107	126.5043822	8.178288539	192.9139501	37.27501206	84.16203814	31.78788838
499.7664993	15.30299616	277.9748296	20.42220575	275.9370646	63.56640012	159.1238153	89.48015025
297.500706	4.81267806	77.35717982	8.556885937	202.0775008	22.96484776	100.2123789	54.62126683
465.9910996	11.52506181	194.2842529	11.90527294	149.0769643	80.31168522	152.1439797	40.48260231
368.5551576	6.651924694	165.0489932	7.139862595	148.9509054	30.6265904	77.92154974	25.93629656
527.4814694	10.02166165	205.6456991	6.328331213	264.8423528	53.17562986	195.2418868	52.86109589
354.6865857	7.86219189	231.6745326	6.362091325	203.7834966	23.73540973	76.79653638	40.45564187
307.1006105	8.759202454	168.9506898	5.693560881	163.4347374	28.47527996	90.26413782	57.76728364
451.7045347	4.964756801	125.3520254	5.464105536	253.9579935	32.92927584	109.9137571	72.82227268
437.8802922	6.836626715	135.1447456	6.549009655	139.976748	44.89068288	114.9554448	27.20300188
274.6072533	7.46056253	227.8193857	3.483401984	345.7304654	19.98330321	170.0944804	71.13431526
549.9984038	15.21225972	123.7550885	7.129321309	145.7344056	66.24602911	154.0428388	50.67950107
559.371859	10.26322194	85.95515271	19.9652735	134.7782304	149.0489636	67.17746768	63.19078623
589.0303117	16.7897679	241.9606195	17.80100487	235.6359349	49.68435771	195.6330571	51.49021906
444.8561066	14.69764145	218.8303516	7.324342484	225.3649306	47.64149192	144.9746225	54.50098377
157.3055793	3.092690942	37.49392089	10.13888043	129.0650227	12.10672721	43.14991231	29.13529239
264.332531	9.650091966	151.4878404	5.413323389	170.6488242	25.6085513	103.8373664	28.55084106
231.9832607	10.09836612	43.28951268	9.626224138	172.3968932	23.66915426	64.07757167	27.08168012
397.506127	14.55473578	307.5916357	6.313455631	257.3722303	44.17890396	148.3682278	53.53804708

450.7280789	8.531709704	247.4144557	5.027133535	203.7562479	41.23606916	166.4525813	55.52254902
439.6038113	11.83536995	211.1353866	9.063371721	238.1144553	32.86983197	145.7196521	29.22659986
214.7429694	6.57973587	92.06466289	9.081341342	121.7653314	38.26307662	43.30611418	42.1358045
994.2475041	17.12687636	202.8705388	16.46443593	361.5651178	115.8464866	173.0075223	43.31150792
414.1667869	7.128033985	123.4877819	3.587565949	177.553889	27.03229425	132.6842338	37.88849344
282.9647717	9.13992587	151.8348382	7.427902953	215.7550709	37.87540897	83.26698464	45.40050478
836.6053652	31.2608682	415.5915984	20.31757965	445.9803436	105.7842834	185.9977384	82.32074011
412.4155001	10.85928033	245.2881578	7.613268992	194.1273615	25.96770277	131.2906024	33.87503869
767.1481892	10.30955816	138.5514945	9.936014807	291.7476319	66.4926388	194.8956443	91.08719483
345.7468641	11.50609205	116.0883392	13.50816073	145.9021427	51.7945391	85.44189916	30.69814376
557.3190546	7.096856607	125.6181295	11.07818874	147.5805586	69.810612	91.25073603	56.36197866
670.3388075	12.72304008	233.8598104	13.19013383	312.1625133	126.7626062	169.3376331	117.7678258
744.4997577	17.07493802	336.9960063	8.297386942	337.7592576	48.71192821	200.6771677	73.58667036
233.3845173	6.077514368	128.3603356	6.839555454	134.5939988	39.21122596	76.08931397	46.1121604
246.4380315	7.685495752	40.48965807	10.05641349	181.0371699	27.71681494	123.9465316	21.44290152
360.6859699	14.73879815	170.6388315	9.237059058	214.252149	62.24885465	123.1831543	50.01442514
397.8446095	10.6554897	145.4766371	5.049742165	203.0699032	21.38182365	186.7678684	24.15132078
281.9083394	8.278889635	179.451216	6.296523222	225.4643315	50.24235776	98.0421605	37.69591323
238.5858048	8.950732239	115.0569501	19.39509566	139.7046177	57.41181171	70.76970613	51.57847759
535.6676356	9.528156822	408.6603726	4.203210908	162.757724	48.00024601	167.3574944	31.91856662
558.2701165	16.86727214	262.3068858	9.949515173	246.4521697	84.10156171	120.0855908	69.76140422
421.609225	6.110188164	179.0902961	6.27226268	230.1627229	35.83908438	113.703633	49.41361686
382.9146071	6.298796072	259.0244283	4.353434648	153.016144	26.86955894	119.2803389	40.50221302
427.8574726	12.04908587	159.3940657	14.07091687	382.0261798	60.77168926	136.474667	55.74863746
530.9157116	13.09266408	130.5926845	6.867191606	97.03507436	57.12323094	111.5509013	29.72014972
338.4900007	9.561556016	179.8007467	6.012773301	360.9855989	28.23625408	118.8323602	63.01833177
1167.977323	21.44669259	244.537281	16.7269939	302.1647476	110.495738	320.0066552	73.07251793
314.8578542	5.34982351	112.9530822	5.304864857	220.0666855	29.96508697	128.9055512	61.63001138
470.8217855	5.743252848	119.8997014	7.59545169	180.057648	43.20304618	106.3627469	56.11092629
557.233399	12.66055709	214.8371092	18.18019316	194.0764752	112.9046729	97.08629725	76.31844217
515.76601	12.08754705	166.4362267	13.68366749	278.9633335	141.5513603	182.1464515	91.88615399
411.8158488	14.35101161	360.4286992	9.778986777	242.6573505	27.78473243	153.5666688	80.3658581
248.1555474	5.654900444	119.2485429	10.45467802	91.47181568	42.70508619	122.8522468	20.61444236
294.9121577	8.673420122	149.1938633	7.108958872	170.6578831	55.56231975	119.4689212	72.52091533
384.6697818	7.138593392	261.3620717	8.506636331	252.9936025	29.19401233	119.490156	46.32213673
566.3748494	9.299927809	362.1933719	8.133327975	319.7387706	37.92350579	150.8382367	65.63400316

758.8619653	8.216192011	401.7282578	6.390039388	379.8883713	32.59262179	167.6210091	61.06463959
753.7055739	17.03959247	169.5052332	7.712948785	210.5083085	60.26120208	154.2635292	50.88532773
572.1634475	10.29043813	198.8200851	6.81872574	291.8229973	60.41554828	179.1149204	41.1530696
555.6878408	8.702981796	253.3571157	5.042790692	205.1890508	29.92276451	165.9427863	56.29502967
756.0108879	85.76343873	600.2788758	19.23161024	627.4760754	125.7043553	388.4688797	80.16759646
332.8906337	8.535160637	94.51040443	7.199348915	226.8087408	89.22696201	107.1113683	97.55184568
412.3250464	17.27455892	153.0325106	14.07988532	175.8145407	73.21960348	148.4967598	41.13980473
324.2296704	6.760963769	180.3790609	5.573551528	170.7337928	35.0217936	147.6258108	56.87042661
258.4695358	9.634102556	231.0039968	6.597558899	248.3260869	48.32839587	162.4040418	43.76869152
361.5359383	18.29610819	118.6598526	19.14096938	192.1526915	60.2703477	88.74402456	68.08341448
518.4913677	28.74478424	290.1978011	20.2029818	282.8305191	69.91604688	133.8192191	54.14382711
629.0806587	9.810301491	213.757344	6.629963173	266.5579877	55.50501343	154.4920626	30.45424661
374.4546443	11.16453632	165.062924	13.12091083	223.2149831	22.95514671	119.1352342	29.86743346
942.1307395	12.04860262	133.7951449	16.53095917	217.3625049	127.2126661	174.4463311	53.0663017
223.9232169	6.641073486	147.4885025	5.077147318	123.8742249	35.43820408	88.12696719	44.25689978
459.2645548	15.21723852	197.2469899	12.18911904	189.332099	82.83644183	153.6747189	49.47584631
102.5804593	4.051222432	353.1235206	10.00895243	107.8510394	45.5195664	63.58139713	17.56130876
581.5693884	21.05063162	231.8700132	16.89522969	339.5369902	86.75313515	160.4741267	93.9906359
425.9116747	15.546478	200.1984516	8.982002222	246.9361644	50.68389368	111.2446551	77.76300011
328.7720802	5.619587327	188.9829403	4.777900132	141.5081075	24.62608696	106.2757165	26.09923747
409.5885842	10.4594827	132.9253293	9.278579579	276.5173403	60.80451582	151.3868719	33.21749951
422.6130633	6.768583447	213.8224774	5.202115413	217.0841086	19.64939195	96.35293987	41.47181062
365.0274276	15.47491581	167.3416125	7.809588783	196.814959	104.0304168	152.2508035	32.27011062
468.3652293	6.959628336	119.0966374	6.052257324	274.6416936	47.00203181	110.1302121	73.06397199
372.6607543	8.3486523	175.7890416	10.00133045	161.127222	48.2521608	105.7920675	62.32082528
622.6268768	19.63816614	163.2858649	21.22061575	287.0311484	91.24136723	129.0026566	51.65616439
345.998283	7.751752691	91.77119995	16.22891482	172.6955806	35.20948167	94.68064834	51.58129134
484.6142828	10.11386756	118.1540554	11.0726593	243.5420114	86.68639033	103.0672498	58.42946566
246.4298029	5.138745544	145.6723253	7.24474486	153.619128	30.84180912	66.05052789	26.80882974
259.5889285	4.368733884	89.28601772	4.86911919	193.431791	28.50841783	111.838641	39.94831496
355.6150791	6.903318416	127.8441896	4.923213267	146.5171725	47.17629756	91.27949826	30.33548637
505.516755	5.211423304	109.9291671	3.190592581	164.2243864	42.59328066	134.230734	30.46452761
352.3235595	6.780322919	180.3628213	4.48712041	149.2010202	34.29930756	169.3385139	37.22019425
1026.269334	19.43405926	400.7909046	6.452198901	302.2449064	79.31719384	177.0727325	76.0159995
335.341637	8.338504569	140.0018542	5.782690756	212.8002308	35.15288865	141.5118338	52.80162268
365.018324	9.148793836	158.7868315	9.371195583	174.7469813	31.98630971	116.2304329	31.23235332

331.2807361	6.144395746	168.3249512	4.153098648	124.0926878	23.86670922	70.0603945	21.5695766
397.1563688	22.63639243	203.0619068	27.66236931	196.6587171	125.4508453	140.0655077	59.3211793
419.4269861	5.429293937	104.9430996	4.663211294	205.2300123	36.60389556	98.55079602	76.87018704
564.0342472	11.28873701	310.8778821	9.246575562	405.5622064	67.36137676	169.621358	120.3595354
311.5600731	9.419220255	264.8673304	5.480242723	174.3009859	34.27013937	145.9205838	37.47305224
801.3562457	20.5469792	215.9866151	19.31035273	459.0471229	149.8144769	185.1214888	137.5097747
808.2462266	19.48162638	347.5857921	9.01508814	260.4378458	85.49113747	149.7230844	50.6524285
332.8116503	8.689606687	186.8018147	10.94926478	159.6405984	32.21294731	125.2426716	32.43699187
270.8930206	6.789146872	106.2656074	6.370889477	136.9481251	34.58148669	77.1003598	35.57989793
469.0099945	6.105222058	162.4809844	5.033271144	185.8198538	59.32705445	129.1253551	45.96189313
374.5032061	5.76612169	198.8203515	5.809834144	163.7274836	42.40594658	107.9348445	28.56965108
295.2686728	9.90421952	198.1865748	8.938747759	159.7257598	30.85830459	97.85902539	44.76592114
413.838168	17.37297747	121.8414953	11.87756579	191.3933629	32.39216155	124.2070971	37.96413616
410.9158051	10.75762176	323.0096098	8.382699417	307.8858284	40.4387921	180.4496322	58.70111397
195.2564081	13.91034174	107.1593864	9.568471061	84.67416197	39.9082069	113.9101614	13.97024338
336.9274146	6.147666498	79.34194336	5.692774494	169.2917832	39.19594483	112.0674257	65.39404058
380.0969122	7.123938767	225.9821714	4.478834	158.3339255	36.57453968	123.9884338	47.97020868
418.91943	17.85992257	197.807289	9.862117437	241.389675	58.00944858	142.3393367	52.13830599
284.2876034	6.193318844	130.4402638	5.864430822	126.4876588	35.17531952	76.88435142	37.01271771
250.9990776	4.96462823	147.4237147	4.937020978	172.5492216	12.88206826	75.52338358	38.33655749
355.2072633	10.06547515	252.3276114	7.74928349	267.7706604	39.17426734	138.9829332	56.94973199
846.1512302	24.76134167	291.9255439	28.44852437	328.4544247	129.1439297	210.6054311	72.92950133
358.5304744	8.2018685	84.92744875	8.879510362	155.4927788	41.74305779	151.0618166	52.29245489
512.5714639	5.339756804	74.45805519	6.20791488	169.8436085	81.19243948	103.8330172	94.19141723
639.9003967	15.54653016	266.6109222	16.07288959	382.5384207	82.91647263	241.7769254	120.0002733
469.3303316	11.54963059	230.4062509	6.698072719	241.6155273	81.56035159	184.4733424	78.10714265
1100.302189	24.03785464	207.1324339	8.694675455	270.6874099	165.2900224	211.3365834	36.76078645
202.0008909	3.798504158	39.5056755	12.45828608	112.7528646	33.27147434	76.5070423	13.95788734
222.1725845	4.844396025	156.6108353	3.441601212	121.8956825	15.62024901	76.85314804	21.45540175
426.2562328	7.099001342	213.6611158	4.638439802	138.0271466	42.63760129	116.0311329	46.80404993
297.2340542	6.436335144	183.0906039	6.419242535	214.8954712	27.85941358	80.19810419	48.37673889
473.7167012	8.178180637	234.2428836	4.888203984	130.4399012	31.83234406	119.4264723	32.99897762
456.9394768	10.81615352	171.5639122	12.49134861	285.8511672	55.3850031	129.7094774	71.99835261
323.5610962	9.107319255	183.7732838	8.124127189	145.592041	42.23548656	83.4353195	34.48116319
559.9653858	12.83978506	161.0336385	5.339329079	240.6494573	68.03345238	142.6020054	36.02551576
299.1017755	8.220155345	52.20090606	12.41152651	174.5469937	67.95637848	110.9399548	58.44251963

655.4715848	12.17350097	310.7933697	8.149324483	254.5525123	32.60420242	170.9658021	28.59718561
434.0897826	12.75978364	174.5124722	12.4523061	279.9934301	29.99450652	136.2182071	58.04119355
424.067154	10.7312969	171.5576398	7.69224933	206.3058016	40.12189641	132.706409	26.62405239
798.1286825	9.936788972	309.1893834	6.158236345	325.4252727	65.75400312	174.083125	83.2724216
389.8789992	4.905962357	125.8044892	5.326596147	108.4011114	28.43280126	76.40620599	18.99329851
211.7082077	5.24294722	96.92967595	3.610797007	98.28697473	18.34316576	98.85413855	33.24445187
445.9858779	10.05750077	178.7279878	10.95141677	216.4857056	35.51907869	106.4764738	66.32356364
310.4550807	10.80042516	82.92019103	9.780406531	153.311642	107.9768685	91.15066073	34.98621398
564.5302847	8.403748272	140.8776181	3.647555199	203.5468168	78.56799627	158.5458085	39.2739068
487.1273138	9.756861606	108.8842181	9.289142103	226.9511045	67.95731005	130.5868893	82.99256608
540.6911206	12.2901535	161.5044858	17.26659609	310.2453201	46.16949521	143.4211673	57.99981272
907.1888605	7.69339029	196.6992847	4.449439472	406.2078325	75.53289143	225.1047819	187.3354891
643.7859857	14.02848126	432.3284803	8.24282489	372.1825008	63.14597569	189.7193978	48.28955291
459.7211079	12.97322776	263.3091114	10.06081285	211.4183123	65.19615943	105.9777447	69.22823792
128.2830977	6.225045664	84.27779264	7.730379753	58.03160353	33.7836929	59.54492803	19.51525906
475.2771974	12.98091397	290.9167404	6.287038495	262.0582732	36.53789316	126.5161604	57.18250898
238.8327152	6.260780428	177.8448698	6.623583913	170.1485038	52.53543818	86.72178331	53.92125053
541.7160758	8.636929662	77.5343856	7.526016531	214.271201	33.28640611	110.6668426	38.88657328
395.7786457	8.598000577	210.3909693	9.419229412	225.8643382	37.72031978	95.37019847	52.06827193
382.1715711	8.349431708	177.6253078	9.787749007	239.6346116	96.87462202	173.0434725	46.55738385
255.2792954	6.381865002	207.4611713	3.921283449	137.7192096	17.84416604	119.7737226	23.82519219
342.5506414	7.858011601	144.0778546	7.572684094	136.0562296	52.05848455	110.736649	32.56716775
682.4957936	14.03555588	134.1685055	11.83831288	246.3476454	70.1831713	122.3006749	44.07169092
554.4722706	18.04350978	230.4188778	13.69745999	202.3898619	77.5979141	140.5164032	51.93119396
339.1897165	8.149959916	150.716941	8.251965551	162.0761528	55.70559386	108.3053567	42.81325159
677.9707178	23.52422901	382.7373497	12.63699236	343.6184825	80.62816248	238.1864513	71.8671392
338.808886	7.722822567	150.0188754	7.3488225	156.1333604	41.97962031	100.6481777	35.6599461
414.2204799	9.463811237	212.1403727	7.916358173	229.8581108	29.39356253	140.8807192	54.71003267
273.6043137	8.322518354	122.1487497	7.486030782	146.6744442	23.3987671	76.47820471	36.41890209
450.8688823	7.561746841	322.7597846	5.974767954	212.9597379	27.58571707	103.5401547	45.21021153
688.3173888	9.190891939	362.6809604	12.04260319	257.7360432	84.25526479	154.7005625	36.5262792
142.0372606	4.378976993	106.8547373	15.11156979	155.8485359	41.44511075	78.525674	35.09543345
165.4316631	4.203744322	155.9795801	2.403182124	110.8971353	11.63378658	104.0540545	24.7611909
276.8579847	7.783486072	186.4431034	5.793154765	157.7377522	32.45554372	113.3488598	29.86876852
286.82428	5.749299742	117.7211208	9.59282432	193.5733413	46.96139217	99.25304641	54.17182956
416.0562307	13.10363121	299.9088593	5.210574641	325.1464888	73.90443075	219.0666416	51.24840986

233.4781353	5.302218106	119.7508687	4.386258168	126.5187986	21.61236385	80.59193598	45.44213003
554.8582187	12.92347951	299.2276488	10.04826767	276.3429437	79.53521699	159.6192912	60.61800253
656.8829724	12.31957951	515.8406533	8.807192006	298.2449092	73.62698743	133.1698224	47.95596472
600.6554562	13.25581245	361.1389186	8.122124174	313.0199554	38.97202515	180.7464905	57.70449846
430.2208353	9.510494126	325.3284408	4.648448948	185.4210228	38.23062143	124.4403527	41.62383822
530.1044703	10.3034538	212.4600989	9.699959687	231.9238015	45.54479481	151.9531367	37.63392149
393.6080465	7.436123168	134.9760087	8.017507572	204.5011404	57.84524453	99.0776149	62.97920789
312.0536652	6.959549138	139.871043	5.621515093	212.9721382	22.95723496	123.1072265	44.76392227
353.7527906	7.34253809	94.94476316	6.672137138	345.1197006	86.66102193	190.386097	60.19780888
1398.540794	34.48467881	349.1838633	11.60494343	482.4731352	110.6034161	246.344521	73.07069133
434.8700267	7.598322481	282.6587555	8.567899028	240.2748694	43.67796612	98.14789608	54.33515408
564.8482504	11.9749179	317.1980204	10.05655462	293.5833319	31.4239434	157.7975159	48.05032433
145.8659107	9.163392603	91.55190577	11.005917	67.49194106	38.24523972	90.20103374	25.04844626
634.5324704	18.36550842	283.9378981	16.22477129	275.3347613	62.21407808	180.0548579	91.98838207
378.2399706	10.34782963	195.4964731	6.495222506	166.1814541	30.47072729	180.400803	34.54527264
198.5990584	5.331438213	96.0552106	9.191871274	142.5323045	40.58055067	53.64354059	44.21787906
304.5665456	6.12696374	126.0003697	6.768177092	140.6315044	33.57374617	107.7799818	48.97171766
388.6706019	10.95969222	394.1716177	8.522445898	179.6384374	45.71819616	133.3308161	32.73005498
273.5782106	11.18174968	125.2826678	21.80217079	191.5910025	43.94759544	103.0317113	30.00972825
375.6269475	13.53552906	164.9361603	9.466042698	137.6540375	89.72780788	153.2309353	53.55909726
426.9416332	13.18946006	324.4297326	7.866053607	286.376416	33.49499279	152.5449956	53.89278364
414.7330678	9.662162547	163.4722708	9.337965632	168.0092748	41.37075369	111.3999182	50.80106701
312.6802095	11.04081459	166.3899278	7.31985007	341.7059733	32.39803005	101.1143519	50.47247456
285.8709444	9.764938706	193.9002314	9.115885555	197.7517256	57.74762177	96.26989915	35.54206133
138.2440301	5.910875186	83.25536887	5.233140946	151.0536038	35.88419987	69.36612242	29.44762603
392.8935661	13.58341018	298.0995193	8.868531026	370.106287	54.47685169	205.6983873	77.05289324
524.1526076	14.14027512	259.6586956	13.24851328	236.5057021	68.88951824	128.7727184	57.19319915
615.7640888	19.0429475	260.7161155	15.36767523	332.5599498	54.5620554	145.5816121	89.43780283
370.6932267	8.611614036	104.6415904	7.208543823	213.8335811	52.0097728	113.0054954	63.28864133
241.3259205	6.435524616	141.4456971	5.518971271	151.976038	15.97107036	97.28867002	36.45470454
815.1982716	18.05174579	142.9527901	16.51410342	396.6110925	101.6133462	195.6751797	88.05860263
511.8607846	10.23253081	221.565713	11.05496914	223.5721197	48.39930272	164.4260509	47.44505031
812.8991661	30.81053854	357.9474262	19.61303567	263.4540346	79.98356671	193.7315233	68.4999502
273.1996125	7.145306508	84.33866752	5.063962326	124.758054	32.80426862	81.4349013	31.5975761
139.0703398	5.620586304	137.7383837	11.79681938	103.8004772	30.7606228	86.08590818	19.88457785
207.3614943	7.033986635	127.9455295	4.363826527	138.1892132	24.241143	87.15749547	35.03275932

442.1516431	11.2320608	152.8921332	9.45148775	174.8781538	58.489951	121.4716431	55.79141277
389.0218235	6.41519328	212.5042258	5.821081344	137.2717162	30.22514145	86.41033624	44.33708982
559.3808477	8.922307874	247.5629542	7.259064978	263.9240661	52.28925695	153.9681799	100.3980437
831.1844965	9.799553231	349.6750913	4.887323852	331.6314738	43.50498396	215.7799877	44.27922546
191.972099	6.19375459	111.4763078	7.910887738	96.29679054	32.96813606	75.43724388	27.39754175
351.3140625	7.367270922	160.8806323	4.569825018	139.7966631	23.52061916	82.87972869	35.12333836
540.1387938	8.856633076	187.6862814	5.647010824	269.0739752	20.36718517	165.7164714	74.30326905
346.9681885	7.307897792	236.8689477	5.146713756	163.4664534	29.350249	138.7118478	49.00461494
587.9711001	15.81278782	179.9624188	23.78192943	210.7776546	139.7636681	114.2291902	93.3574443
182.8251123	6.162127483	146.9809256	3.922203751	122.2993928	20.89460831	77.87115553	29.17325885
356.9195617	6.919839029	147.2376669	9.98227304	275.3810357	70.76583833	111.2131112	56.56290562
208.3013896	5.60146453	121.9207911	6.826410669	111.7549027	29.13763302	52.39105512	40.78800191
521.9387951	7.989274933	208.467139	4.612721948	233.8860709	30.27624915	116.9487393	41.70754831
498.2837083	12.79832105	236.0861581	5.025448359	321.3161152	53.13897796	167.4043221	68.27447968
610.4082695	7.609408184	33.31288665	20.83514529	238.1622652	48.03578225	110.3557986	45.45138479
170.8237609	4.225592856	65.93412336	7.348899589	134.0294028	18.31766266	71.54734096	14.03417618
341.511084	5.173944884	126.1928637	6.225146664	166.051544	37.5053339	88.08115332	62.59153232
306.3567095	10.87765345	145.4935228	11.94270577	132.0298992	42.07066309	84.59537067	41.96835616
966.2980709	29.30983546	383.6268323	15.10476088	401.3353094	175.3110107	293.982098	103.6566904
366.992706	7.455062605	150.4642753	7.427670593	219.2190795	39.36928272	143.5517555	67.09950426
213.2897819	6.354694897	91.30782707	8.259884685	102.7301012	41.36137184	134.9036994	33.98472862
350.1499458	9.24068521	100.5152908	10.39564456	148.9797978	54.61933133	81.26030207	47.60129677
608.584	10.24082304	168.6108665	6.818218264	229.4809745	77.85326298	150.6652902	60.56867881
456.358673	9.716270445	245.6256504	12.86174581	328.391371	44.86399468	116.1406853	27.11324086
489.8800875	11.81612964	280.3597836	9.318759284	271.496937	67.81556413	263.4026876	79.62606655
219.477101	12.603352	71.32477745	11.34090805	133.8613152	99.28097179	90.94304886	24.86576776
460.7113248	8.623116956	213.2519344	5.396066077	149.8690731	41.75157753	116.1566951	32.0854867
355.1519897	10.41754094	316.6521868	5.040623735	230.0913426	27.77354225	147.2832534	45.62483581
233.5262508	7.395425438	60.90815455	13.1103249	121.5819308	62.26989537	74.25082674	44.09433585
293.2809097	6.981655287	183.3192484	4.48050973	107.3100995	23.44740592	111.1347275	17.31283072
565.0454908	6.549705063	84.6452931	3.313061706	162.7309643	59.30699559	112.1003789	36.12713555
539.9614849	10.86569006	278.8577051	12.00206441	397.4220745	37.0002209	179.5934821	50.74926372
426.5160186	7.150580294	112.6444384	8.727024107	124.5950248	58.14353663	103.5765922	19.45882887
400.9696946	17.24095799	172.8370253	18.06895606	269.198442	86.15088515	92.24804192	81.60316622
363.209939	5.471378938	108.1706232	7.092554548	127.2479594	44.10943709	88.00633041	29.02844611
535.9568461	8.974488037	86.67030046	17.45064876	351.6560672	64.45382203	118.4009897	123.9187538

301.3242246	6.235700741	138.9944121	9.141551044	133.9297018	37.17219323	72.80054339	27.10862402
513.979139	10.70349758	301.7114053	8.283923661	285.1532731	41.21285897	205.1073084	60.74101469
361.4463901	7.256108584	253.6089396	4.770022577	159.9945355	39.78074269	122.3927626	28.14969011
295.4494354	5.678576549	233.3130003	4.080260946	222.6450006	19.11571967	139.2860358	47.58476736
489.8143599	6.692045338	83.64357217	9.75991803	290.7452238	86.10463664	163.035996	64.39980637
585.8217441	7.154201658	77.05317371	7.724753718	298.1473458	60.35123239	139.5297057	97.14781827
648.4777282	13.58776782	361.2524501	7.215723098	293.7838136	44.95775585	171.8195909	54.37684637
587.7672117	26.02128229	588.6008933	14.16585369	484.391094	75.06126663	221.2314572	70.47403949
533.2740341	7.272071728	153.1800857	6.190759442	230.7616505	66.24656995	121.6402504	56.3811019
236.4578997	5.392383149	73.37100178	6.645561989	108.020194	22.50118334	87.56352397	21.90400045
358.3804793	12.30594482	155.9879567	17.24857978	278.4716923	56.45311542	121.237245	75.97619915
287.2784053	9.56061956	117.1304265	6.535452697	150.1201295	52.44644082	108.3672918	42.26056188
647.7916486	13.32275138	220.5725002	11.07555764	285.9052251	75.93086875	175.6099573	56.97470429
501.9631624	7.839884008	385.2527165	5.756827504	261.3796451	25.13292707	135.3970057	49.26785011
409.2355416	6.744685956	143.6281725	13.52289833	128.3286389	40.23559803	133.4150314	24.13921195
387.5977731	8.753944319	272.2576889	6.064552939	258.4609513	38.77268146	111.6263814	69.52271601
576.3897149	11.55815077	235.5944597	8.014986034	398.0823602	63.25969383	179.5505909	102.8948222
369.8498602	12.13139578	230.5973995	10.69818566	198.7949883	46.5118123	84.31343205	30.08659594
183.5635525	5.105490547	111.1717721	4.211206446	107.9540254	19.45813799	71.0789895	28.01912661
52.71556129	2.356464209	45.27834711	2.475410639	66.05608689	11.48017891	38.543101	17.53001141
287.1797092	11.46282274	71.75870459	9.323927958	288.431341	90.00217917	135.4662353	52.38988845
658.7380119	11.12592344	143.3444884	8.328562754	293.2297396	72.99018998	158.8472705	100.9841137
201.24994	6.660045148	93.20120806	6.534688771	121.2860309	24.42282833	99.57146603	21.49821731
499.9275873	10.24197025	376.6075929	6.622033898	288.8692763	27.63848619	179.7206769	51.63810671
421.9199318	13.60372583	393.8692478	5.585918755	307.2617852	61.27986095	195.2438452	43.9919343
171.1466851	4.406309079	106.6356055	4.918179895	173.2657721	13.25089413	66.70999583	32.46300156
646.4951444	13.947803	300.3985315	9.793607176	275.7711307	67.65603163	191.0013506	42.36441108
346.4088652	6.341593027	166.8710497	6.512886633	168.3455224	30.09234229	100.6662109	36.45661757
303.4798865	7.352985251	209.0508308	3.839616874	121.7635169	17.16029378	111.4216552	29.65580428
296.4022621	3.751500689	175.6122375	3.611333103	164.1761423	23.05772586	99.45772747	28.15467475
892.3672413	12.38169589	362.3909003	6.564364085	206.98998	95.3185041	228.1287716	32.93342499
616.6172826	13.00025734	328.472175	7.86498231	235.5182244	32.77348181	159.7076709	49.48214685
487.2889749	9.673540779	226.7711028	7.892817213	211.5947616	40.10034744	125.8754006	36.83041131
728.2463983	7.018499915	108.7184089	11.01166013	346.4177935	92.46244538	160.9524217	101.8611918
455.2173671	9.474966477	179.7215504	7.69781447	161.792561	57.38710956	129.2645235	54.96340021
246.5359908	7.52095047	122.264364	6.962709763	117.1645364	9.709064681	84.82371753	29.17950068

503.3537527	13.11253828	219.4128372	7.001801271	220.72591	49.95147038	201.5856558	66.11123682
272.5105977	3.389094217	103.3277684	3.482369268	123.3727312	27.20583912	91.4883538	28.88253632
755.3105785	15.53464165	257.7934759	6.176294152	294.1375246	65.09950608	177.8751744	62.7029161
534.0702427	20.11291083	203.2127193	18.45507896	205.8297856	142.8046624	181.0703727	51.10292561
470.8575729	11.02255387	290.78326	8.397461087	256.2891446	39.13440295	167.4811354	49.66160875
505.8565659	8.886771486	473.7899988	8.05831003	361.614554	49.86516684	154.5183885	104.3012425
325.5132219	9.74023892	247.2924925	12.44557756	206.6889201	57.96239823	133.0955546	56.24697831
442.6499535	8.132103413	280.1634522	6.387252565	269.0550093	52.21096675	224.2438537	60.59673705
476.3586746	7.704136326	174.7990973	5.854789644	168.6081199	51.44673464	123.5160154	23.517486
533.5746771	21.34973609	392.5339324	11.24887171	442.84022	86.65387657	262.3285472	86.39984089
369.4373816	8.189014017	226.0222247	5.762826663	171.0236515	49.06018485	93.40620167	28.4754665
482.4780675	10.54246749	251.6028023	7.971967515	208.5830995	40.12251116	123.8534482	41.30157738
471.3145742	11.07543538	173.0284655	6.868726145	272.2652737	31.67707924	160.5520926	42.14492581
655.9737921	13.10640541	178.6304229	10.48793976	304.0220238	73.13316618	209.6613948	142.970137
383.3371108	10.28607296	133.9137334	11.58747737	193.9577092	40.21917324	121.509527	61.04079831
538.1687055	10.09970574	156.207762	9.622175024	337.4630968	69.07286662	125.0179127	92.4646594
462.5016411	9.002426678	206.0844928	5.576133318	261.6723166	38.04853922	126.2355795	57.82501659
246.4548652	7.621026459	214.7951705	5.09851583	142.3773296	27.26027136	100.7491212	31.38129642
548.6674748	10.73221281	262.147997	7.87687984	255.5696501	33.84073375	117.4246334	76.56296579
474.0566559	15.96384003	139.2053213	5.937762176	105.1263733	32.45998658	158.7455456	14.80407596
265.9736657	5.949858157	142.9900197	5.315901122	130.3355716	29.17149118	92.6540154	30.05313455
395.7864035	8.559026158	203.2689073	10.47870777	158.164753	52.28105755	86.00154988	35.74998725
257.1555747	9.974624401	112.8732876	7.321281036	191.9344085	55.14019101	81.01083385	40.72773687
410.7706565	7.702579626	289.2096588	8.107200289	226.3312887	42.74030959	105.1531371	53.99397072
333.9317792	8.104617398	121.2152335	7.822592724	152.3869826	39.96688771	121.144634	43.13677898
565.7916754	11.12503425	257.861519	9.226594022	246.4792536	60.81594836	130.8774528	56.48538281
153.0198857	5.415595237	119.0352961	4.422684928	109.171287	18.37091118	56.48719339	21.42779326
204.303002	5.103226419	134.3372262	3.791086878	107.7691425	26.49985631	80.4238938	20.04686336
283.6749587	6.814424642	103.2460705	9.042378594	141.7053166	22.63461628	82.77110922	30.80737734
562.3095074	17.97981811	319.5999585	7.362671868	229.2793695	28.32349329	114.7518092	36.15671938
455.1609101	17.25235018	296.571389	12.15931776	254.8418936	35.95580245	124.9445497	70.9782505
173.3186914	3.371013267	135.7926333	5.737340638	169.8991402	20.6469894	80.77964537	30.70936504
314.3796905	6.155715636	217.9643785	5.064716471	160.3316576	17.11489927	113.0331939	38.35573287
594.4630596	17.07861053	171.4118056	17.57627684	308.9031694	53.78516362	181.7798015	60.08876014
332.8260802	6.893624857	222.3410506	5.171196066	173.7875938	27.34956353	105.5650107	46.65675368
537.6182762	13.21132034	236.5470537	13.70416989	269.9381913	43.47134008	173.6612469	63.83837763

304.0406416	7.867770797	155.4713271	5.249885882	199.0432707	36.88929103	106.0378568	56.80730363
423.2189574	12.42088621	230.1384856	10.21366967	198.9992958	52.2176559	126.0179167	32.51586172
293.7633437	8.779504814	190.466548	4.886036305	151.180851	29.34717702	140.3975245	38.62603425
354.9884265	3.315952055	54.94506862	9.882299219	125.8824986	41.24162733	88.5307833	19.41360523
326.2356079	7.458718607	240.0799555	4.073977618	221.279543	12.64835916	155.7686015	40.48622411
679.6066655	19.77457516	559.5229923	5.852295244	361.6683638	59.89224711	225.3090775	52.14356679
320.1241788	8.037394933	94.10370733	6.353385765	147.7737303	48.71255811	152.9943077	24.51659206
456.1537954	5.977197961	222.8489443	9.476608956	151.4553809	47.57329466	93.20488167	27.78150301
425.2181785	27.35983412	302.073978	29.19107483	233.9333901	71.18765735	221.7349641	20.85119603
245.112549	4.38230311	89.73633159	4.600930119	105.8081624	37.95659325	72.16153949	19.57647631
542.8308348	9.19978008	138.1962832	13.30524381	217.2671408	59.92416767	146.2790057	24.34063364
575.8971527	13.85631001	269.9502644	11.03357023	248.3961569	95.30514066	297.5809275	70.42793252
601.0709027	11.98379114	277.0979203	5.483800168	200.7608476	70.63314658	257.4654049	27.82631856
222.2848776	6.786694819	109.4558381	6.990525527	205.5563638	54.72016418	109.3146125	22.99032978
286.1161376	3.158403673	39.2851242	2.285117206	171.6534342	69.12975122	117.8924677	26.83408878
247.8570309	5.38442364	62.97893749	8.702975633	164.2100306	36.73724436	89.15676814	16.25459744
389.0004642	7.359971244	124.0108385	5.462071081	135.4553878	48.67787435	115.7070422	37.68505497
405.8700509	7.583550884	111.7584526	16.32212164	144.5412473	59.33995734	125.2616737	30.13718939
299.8532459	5.027146314	194.0450608	5.404130441	193.6824991	31.20463359	122.9974527	17.00064662
775.8745702	16.78897496	253.1039482	6.880615389	309.9178969	90.77410572	237.912655	54.61708339
236.1110065	4.669771013	93.9847537	4.939943948	90.56252634	26.3545971	116.2308128	21.24904156
212.9244912	5.327561117	65.21959627	3.945523269	83.37098973	53.49455689	85.3602148	28.85376082
376.0415226	7.542468065	168.3229653	4.381891873	121.6297455	41.46620463	200.0618567	25.40528785
240.1397222	6.711716397	137.8281794	4.844142939	121.6738089	32.32232502	87.46432748	25.32627107
451.6794963	6.819481325	158.7396237	8.432054301	210.9253111	55.38577311	126.498561	39.89592613
303.7763791	6.4393573	86.75186902	6.266400802	221.3638888	46.7249991	135.3435214	29.51201509
610.8494205	22.20187173	244.1153813	31.35005116	295.1107748	102.1764333	268.0940366	33.5093571
315.0042331	7.244258608	144.7818185	9.911734707	221.2655432	50.60600385	99.59356037	36.34880208
359.3836621	8.403982989	218.007419	4.401618827	226.0163748	40.27297955	148.9928383	27.81937067
553.952397	7.626602282	60.41596734	8.286406046	99.97357765	72.07114996	152.3732647	32.89766403
540.5899433	12.33721223	161.9713735	7.058698881	200.1057302	52.67105452	165.9303873	38.69669886
369.1164195	10.62751136	206.9208971	14.47084341	139.9401571	90.4280939	147.4167688	22.12434778
297.3475165	5.207513771	61.00645819	4.392395699	115.4622875	60.90738405	65.12071153	20.01523978
317.5397853	8.62315922	122.4384485	5.058686485	283.7894743	48.88451742	105.3126752	28.13013292
557.8535823	9.814457454	108.42174	22.05687911	341.5256207	141.6329992	218.8198652	37.72113895
331.6390115	6.444228432	277.1519731	3.867011584	162.6040447	35.22780012	141.05394	27.65830185

247.6170548	6.152682925	59.77256839	8.021245433	181.1974553	54.09622282	95.84614129	41.25934673
236.2686223	3.648022321	79.41873941	3.235773326	95.97825819	25.37075357	74.71704501	19.75997988
444.4415368	7.190367838	69.71920991	6.625742254	129.4143122	70.61000239	123.6387739	33.37616066
279.7214219	10.49266028	199.2900326	20.39864301	145.1058032	81.47478109	107.1913523	19.35896157
350.5096482	8.5738958	176.6885674	7.434860194	126.4890396	63.4583177	120.747498	30.12559125
717.1160947	22.3078079	292.8039031	15.13858247	259.5927237	105.7878896	165.0181311	46.2923955
427.8821195	5.031679084	140.8102227	5.34402834	147.8847181	39.28500615	136.9937781	33.05089562
242.4693286	5.433798989	138.5386965	6.094062222	210.9636598	43.29577437	136.7589966	29.99028429
329.918887	7.179078072	72.76428715	7.655623635	139.2829885	42.79289156	113.3159701	38.76902242
582.275225	14.70983979	181.1173686	9.196582364	229.5457105	82.52640793	143.782099	33.4857198
385.4215575	11.46849259	103.0579939	12.11186394	309.1679717	73.49474234	121.4101762	31.50618747
172.4201301	4.796396559	75.1379238	5.554729829	211.4182645	52.38748304	106.1942777	25.94874179
308.3714605	7.634746815	71.62794252	15.84587843	210.989083	79.30474564	89.86278845	22.99060423
453.887468	7.464601478	151.1024726	10.08315565	178.4685802	61.92953798	131.3177537	42.18963116
508.9825451	9.20204229	110.6125761	10.7186547	274.2789444	154.9748334	195.7008003	45.85707662
202.3481971	4.724329348	219.0090851	3.978865055	191.7923041	34.81648551	112.0416584	32.94768021
339.3271948	6.044151697	168.6461797	5.416581615	155.4664127	31.66242517	101.8042426	35.0768856
750.5974605	10.74903477	167.9348375	5.694017644	295.9498954	64.32364813	236.4734309	30.3017614
382.4554467	6.027738266	91.95961864	7.957591972	154.3545258	43.97376849	110.1923127	31.22880197
114.2261175	6.949611692	134.286866	13.31141899	67.70161927	35.71158744	56.30260693	14.94839774
179.7849643	3.529905526	110.4141232	5.866219348	184.4588553	29.82037213	85.72503627	20.53107775
368.8243152	4.912183444	147.4680184	6.22867572	161.2647882	55.63898838	120.6798914	39.39853965
473.3400714	9.189202369	105.1577388	5.705833686	315.2337454	86.66485073	159.8543242	41.56800356
307.0612876	7.13399886	88.78347643	16.53049973	108.9699152	106.2302633	108.6873025	39.95836859
341.0156447	4.949660255	105.2401837	8.157385684	139.0072168	47.55840999	108.3406036	24.25202426
458.0083389	8.374923038	136.103944	6.721542033	117.43652	47.05993024	130.3332862	31.94762123
703.1138978	18.6167191	243.3058151	6.413791357	190.8540309	76.76229299	170.8264075	41.99754636
190.7027874	5.922013069	162.2290223	3.142824947	134.6465208	17.04100686	91.9778708	16.24428919
169.4557967	3.223068359	120.4557048	3.113169846	124.9159358	17.33801816	78.90233719	17.69832882
252.7701981	10.90750056	117.5035175	13.23272774	137.669387	34.71718206	98.47581046	19.30131234
623.9146446	12.05895263	329.3480083	4.994876993	195.0813814	93.51751952	194.7838615	29.4083248
197.3151714	5.202833194	53.25381678	6.896447831	179.9639515	50.62977954	98.3038183	29.32421199
183.5327835	3.06418034	77.27761481	4.499816854	117.855303	19.96108077	56.34786222	24.7054722
824.9478012	20.87215009	180.1946998	16.85941143	207.7275084	152.3729113	341.5013752	56.77809227
325.2300198	5.662737646	113.9552447	7.815899713	173.5265814	50.32179791	117.6417444	19.91214668
259.2220563	4.953026168	72.71069521	4.896405013	149.3554043	33.2424228	85.48834951	26.02980335

385.4510782	10.2517474	251.8738207	5.875022104	172.5963877	48.78205914	184.1693074	25.59195599
128.4199433	3.140639474	70.46161201	5.964821718	206.451738	36.30215931	51.82660113	18.50492115
645.4226808	8.077872015	157.0480117	6.663448811	187.2353139	34.97684062	118.2927178	17.61617878
418.9920003	9.846840616	176.1891989	5.15497008	171.4322719	67.97139805	142.0857605	40.88582682
398.7176564	9.714144159	233.3529152	3.451130561	177.3123047	35.40144067	193.463473	25.265487
398.6491112	10.9196006	146.6982729	5.529810444	146.9057345	46.75069598	125.0262975	43.22337317
258.7780056	4.385035867	52.06144684	5.274839348	163.7460199	65.18866637	85.78452451	41.01474241
225.0616767	5.941696503	123.9984125	9.929975075	107.3196369	52.076682	94.51647097	22.96197998
523.2298177	7.998880683	197.035685	3.895464041	171.5133148	43.42683753	142.817843	28.41655452
587.1857028	11.24447433	148.1627868	9.880808265	236.5010576	87.03099137	172.339996	41.87018021
460.8878839	8.469137516	217.8673174	7.428944497	137.0474031	35.50329967	177.9345962	19.68674876
248.5082711	5.922531069	134.2984834	6.51077246	221.0999393	19.38927087	92.6947268	22.59597195
203.9625158	5.337269572	89.83963615	7.198216087	137.6181504	29.3195373	64.91336791	24.14386217
301.309099	7.423623891	110.8236331	5.977768946	173.247434	45.95068309	117.1913006	34.79332878
248.4438107	4.092752229	80.17926203	12.02882048	88.38750339	36.02392141	88.66392103	10.86867194
228.9360182	5.496648388	119.4879337	4.103093231	132.765339	42.49029022	55.26885765	23.58735019
298.3674679	6.569822428	120.7767548	5.265155004	125.2968393	56.23957744	209.4232482	18.9508575
273.1996146	4.253977025	60.81458661	4.175255086	193.6307416	57.43622439	97.40393525	48.1033075
412.395805	13.40584612	197.7269871	8.919766535	191.404245	69.5752646	159.1669141	43.5340865
416.6691649	5.619199804	154.6241684	5.05252285	153.3879666	65.13753507	89.41790214	24.04033034
489.8284292	14.91724065	280.9582021	7.88244417	260.4653711	64.87659628	180.2850489	39.51629553
388.5513604	8.867983635	202.3338776	4.375401567	173.1188513	37.44716722	193.5550006	29.37310398
271.3639766	6.679537497	198.5602128	3.277685477	200.4342785	29.12084544	133.4633116	40.64763346
305.2447681	4.25414893	74.7356748	5.507924812	259.9275298	74.41864099	131.9784552	43.92637449
488.4671058	8.124314505	112.516279	4.405063647	325.4001979	63.70181583	157.4090042	67.76702497
405.5207514	8.246111132	195.77976	6.738753794	155.3166117	42.42168399	121.8043434	33.67840136
519.8998031	12.18108095	217.0851145	8.241185961	179.4808579	42.70420472	136.6745623	31.09082355
874.4877775	13.98865207	234.5860045	4.509372632	360.9333564	84.38450924	240.0503639	36.231176
472.3392552	12.02032636	230.9704959	5.695502682	155.7276882	49.69709481	123.0927176	36.10584173
602.6888868	12.48493957	183.1341773	7.65185565	263.3828098	66.56351738	136.1957326	39.26508484
416.6748995	10.30204759	127.254552	3.679299155	204.4488578	54.29302418	101.7310518	19.22394078
340.7387667	8.999714118	161.3898985	4.086302766	250.068462	38.62600042	97.72917672	25.98299972
149.1697344	4.562212164	67.63543468	9.394211396	94.60769822	29.64409985	79.1641839	30.4115308
532.0219208	8.649505028	153.0773228	4.252992816	199.7967944	34.02442853	135.7929756	37.88678036

Epirubicin_1511	Cyclophosphamide_1512	Pevonedistat_1529	Sapitinib_1549	Uprosertib_1553	LCL161_1557	Lapatinib_1558
0.581581203	209.2942897	2.49108148	40.85793372	22.03231145	163.4552599	13.15217223
0.359088687	158.8246796	4.361502601	120.7203585	29.26435189	207.9730377	33.6346691
0.430363378	309.063315	5.207288131	104.9978294	26.92483231	281.1038937	21.17378273
0.356631693	126.7306315	10.06005987	116.1557358	22.89029908	85.63385568	77.10588838
0.615924456	316.9832847	15.43132661	112.6887575	37.68677286	233.9803007	30.29972533
0.147867813	135.3786936	2.515543475	139.2399804	26.88142676	138.0244967	40.29776432
0.239074482	172.5565572	1.018179085	63.46314657	21.13293853	184.8282772	33.24358805
0.612747547	213.9326878	3.686297729	91.62222923	26.07747592	194.0076812	64.58708786
0.868768383	315.6286918	8.105275483	120.8929497	30.07138191	269.372782	59.42701388
0.2558982	109.5073693	0.995500168	50.68641712	12.79565361	79.71256646	24.29934204
0.27745895	131.0834691	1.749032984	54.67262429	17.3477979	108.9333141	18.7655107
0.694409555	165.9736501	2.383887425	64.03226521	24.2836725	163.4036899	20.77195492
0.389727783	150.1460014	1.571140455	32.98350146	11.48324175	120.1238745	4.640577677
0.427227632	177.8251433	6.480228395	120.2185828	77.57521642	147.6758685	84.08622952
0.337057967	178.2656651	2.765692229	61.21887929	21.45961526	177.0779563	19.07856198
0.608939162	212.6898318	5.46975546	39.00441574	12.89429128	202.7661899	24.44103505
0.171265052	99.21547965	0.423176311	79.64200425	40.67599207	102.2869289	8.993296362
0.766644502	264.2412026	2.209450115	84.99677501	49.9900727	206.4653037	40.54824695
0.338100802	252.3070932	2.574714716	76.21868308	35.73130827	195.1940932	21.06999841
0.148201509	136.7900719	0.520056804	80.66298997	25.97015295	108.9639274	24.55781422
0.311697654	152.1439293	2.399209439	92.06186769	17.40840491	169.6705763	28.44573953
0.712542375	306.3980892	2.450994804	64.72303052	46.2308064	308.6927165	20.38194887
0.174292167	210.5100123	1.147179337	40.87952028	11.08248455	127.9033388	30.5346736
0.554956345	332.661068	3.784639874	41.34721556	17.43289012	200.4739854	26.7872535
0.459512112	161.0765641	2.149432175	36.4244265	13.81450345	133.7099961	24.24434166
0.305724955	173.902654	1.957588371	58.77331329	17.66617345	179.4785265	29.87834678
1.083469001	226.5715179	5.199688305	57.6107314	57.56734522	240.7051106	28.48254742
0.437604941	358.2218185	3.816170737	49.13717123	22.93249261	202.9624249	30.94906576
0.316841106	177.7781721	1.737405201	56.80482625	13.12556282	126.662484	22.3274879
0.497466531	129.6910188	1.318680236	36.20185332	14.01142423	69.74937083	15.10201953
0.249760631	153.0267625	2.21842735	67.71117301	13.41061643	171.5111906	21.1583381
0.549095329	224.8146098	1.407353476	47.69012493	19.8346115	83.20240856	13.6273221
1.203036479	273.618795	2.382611912	81.10473228	23.00919373	252.4418993	16.8817334
0.289844686	111.6240027	1.230464673	81.58138958	23.39255133	103.821838	30.16020793

0.628979232	188.7657013	4.565603349	44.42693025	24.26516271	156.1137286	14.83134358
0.434750403	305.654608	2.038652759	64.46211628	18.4615719	168.3142594	45.45159408
0.127911817	121.3761971	0.714952615	19.37358175	18.76379555	54.2262261	10.05865979
1.256759935	338.5871391	2.430425513	82.89991001	20.22970262	249.6765783	18.48617324
0.547185268	187.2037499	5.909204852	53.3470585	17.2112506	170.8010349	27.08610005
0.340131268	148.7766357	2.398083499	24.29760436	18.38395012	149.4777136	6.646064981
0.896097257	159.9649361	0.647231442	78.41411815	11.87280108	78.45963277	22.01145899
0.28574311	199.3947834	3.165252801	115.2688812	17.74874476	141.1679333	55.71413018
0.560723645	237.8453947	2.914379123	32.57703425	15.06916896	198.9714551	12.13857993
0.631525298	149.8711132	3.92121179	19.56096457	12.13151176	155.7984925	11.94016331
0.158105378	184.3620345	1.460360208	32.77365576	26.79291778	150.7800218	12.94775319
0.181159878	52.13047521	1.02830405	77.2047357	14.8734297	35.41888936	13.56280378
0.179999123	144.6460038	1.718429399	46.23531215	17.14740338	173.6110111	15.81398071
0.279052402	214.5506091	1.800622899	139.9470288	12.21677745	122.0238853	51.2764317
0.170264506	160.525973	2.034699415	71.84800294	15.1963226	145.0858093	29.6229296
0.276502915	139.5732343	2.377325565	29.16861133	16.34136936	141.777526	8.498706375
0.117579487	92.03107988	0.885863255	43.1150475	13.50014923	87.70629519	19.790304
0.292957472	220.3553265	2.044481372	69.39460373	26.52038571	169.0449056	20.73871672
0.490312434	224.8599146	2.316621828	52.16734118	17.35304836	119.7973126	17.38697302
0.199507789	167.6000941	0.717173092	62.92045601	30.98343376	127.1005022	18.24464692
0.332445138	135.1961755	0.431308796	110.9619681	25.45650206	128.5495665	68.82168219
0.557625995	206.2816532	1.954286643	73.4193462	25.79414795	149.7944351	48.73567812
0.168707176	130.9540884	1.242598383	70.41461332	11.48255836	114.3579827	8.69319089
0.210902657	130.0335135	2.384278137	30.58051372	12.05888638	129.9735751	10.58700524
0.672444339	304.9334181	2.006696417	35.44736262	17.44166868	158.6413622	22.41259262
0.243617858	185.2666332	1.313896287	25.68280534	19.36224034	209.643233	7.003900839
1.462526582	268.3172142	6.721043591	77.97846674	33.80973126	200.6718716	53.22721829
0.446350687	165.5995905	4.35685626	41.89303695	18.64068375	144.9580625	24.71959002
1.220969204	291.5070609	9.029310927	52.63746549	46.41760617	199.4551965	22.16459828
0.373781889	203.6617053	1.118759043	81.06051915	19.00252468	93.28395297	25.08960793
0.221244842	137.4200455	1.456119795	69.37924677	12.7873834	137.9091923	28.10391707
0.190199159	124.8553575	1.629129021	38.06196537	14.44942938	104.1306456	19.38229727
0.46174623	202.9696724	2.212691515	3.953767268	37.83898178	122.712936	6.417469852
0.757725597	501.7454839	9.34511	70.64837843	72.22783105	357.0570508	52.68670885
0.435853244	177.4489426	2.243721549	70.08891746	45.3867037	149.3658639	31.61908036
0.432542789	117.6414151	1.416044614	18.32487894	9.48718809	79.97515598	5.050999685

0.40190637	297.7198717	1.567930068	69.24680213	20.35597677	213.3639541	13.94324822
0.194396361	105.3595796	1.265158956	24.88177312	5.497068394	39.38208647	8.370509955
0.425011218	286.1494049	3.333709903	26.3640265	10.55567079	117.1921335	17.10508604
1.23169033	401.0967928	8.70828275	121.5564512	61.88267846	390.8674478	55.4558114
0.353882318	158.1835267	1.963703098	37.31833379	22.72341482	71.90600831	19.86262692
0.173073184	141.1135226	1.300439264	48.75860987	9.389545358	108.6573047	26.98393038
0.306153415	117.6078403	1.49027601	25.39550381	15.61507132	86.25361124	9.639834755
0.197441099	236.5260262	2.257072904	40.47231483	14.28001291	172.2751575	20.05674623
0.36724023	167.4227574	2.666571807	58.53530029	23.26496844	223.9123255	6.020325728
0.580644818	149.5597221	2.476799937	26.27012979	15.36841201	89.56552571	13.01120397
0.493450708	238.7661372	1.323176394	120.4847367	51.04218681	156.5421226	66.12343003
0.91097188	251.9933853	6.41388851	28.80841674	24.37144898	214.8328399	22.72344639
0.234816184	119.3488402	1.095260434	35.24053005	15.85877174	164.6654349	11.29942928
0.156323019	125.8436997	0.653475546	56.05675179	10.48396927	120.5204845	29.41348502
0.483187596	217.6571793	4.223852972	50.06169343	27.07029785	283.9566126	16.39423195
0.407263501	235.39837	4.178272049	40.89935183	11.88834348	132.8959135	18.53750177
0.238385696	103.1626461	0.438827255	140.3277982	29.3910733	141.5871286	16.80447652
0.447289596	134.9884854	2.096809697	42.38391582	17.37458447	120.3689932	26.16270312
0.433060578	241.9503676	2.133846563	103.389528	37.96911545	244.5848654	38.56761082
0.224541874	145.1491184	0.985938312	47.2780025	34.80524845	87.29853461	28.60797363
0.338587254	190.2499928	2.893880417	117.1464027	22.32632297	183.0005024	22.82470572
0.149656935	158.4818859	0.804913921	57.55294118	16.0674037	135.8563935	23.17447713
0.304043475	195.7586542	1.318913567	49.77949643	26.60484588	137.5291625	47.26172265
0.397170166	162.5146484	3.122991179	21.20217165	10.60462165	130.5284378	10.91601409
0.172573365	146.924831	1.853276916	26.90874905	10.75165151	120.5402185	17.65339629
0.308974628	171.2794885	1.471409273	100.8405766	11.73841547	94.25634283	63.73123321
0.210656504	138.76271	1.422831972	46.86874718	20.83050724	141.9964535	14.71868105
0.163958211	221.5819836	1.466267706	129.1506671	17.66984735	150.5912691	41.26187556
0.624446672	174.1925058	7.400017602	61.36101802	12.92845406	115.7746323	26.45479289
0.480739982	128.8526869	3.394299275	81.18742067	34.50606707	85.77817549	37.50461338
0.558962135	156.8904209	1.855552093	81.51315627	37.32858486	181.6604504	26.08066198
0.55842499	218.2632491	3.646670876	38.70012124	19.7273724	192.7786852	18.54922059
0.210984776	86.07377962	0.580293759	49.15435841	12.10852431	71.01690305	5.423232093
0.258241919	140.7532391	1.551997874	40.70945411	12.95889155	122.4713484	20.03368997
0.255261463	139.4535391	0.650416632	45.89027838	70.11074618	72.03127457	13.3364467
0.424039031	248.4566756	2.072992786	50.14620637	30.52090781	232.8982718	17.91865904

0.439913317	190.1725253	6.422044148	49.30720547	9.720904171	182.2381096	22.80580799
0.549139409	166.8872557	7.217135435	39.39883941	17.44321835	165.5694343	16.1632721
0.203497336	145.773747	1.015143492	52.19007038	10.58029576	75.30159501	25.41952254
1.060299705	320.4536555	3.520903844	44.37350306	48.84953355	165.8361745	25.75222136
0.753510024	189.1121161	4.795140585	24.95557545	13.38851986	126.4088847	16.32459733
0.274413994	162.0484029	0.877201923	31.68044775	42.49436651	137.7217591	20.87349131
1.187826425	340.8913796	3.988892096	63.95414197	42.55880804	218.912336	35.47861331
0.272691965	149.4265596	1.729437745	46.44493336	12.52127745	181.0526965	21.77219452
1.251897398	210.2371747	4.876022848	83.55146371	22.66095205	147.2858998	65.29259044
0.291313366	168.8455099	2.995952016	42.56842147	26.91954463	105.8287591	20.68750941
0.258208069	141.1764896	1.366405914	47.18781623	29.3039342	113.623806	29.58963732
0.546549856	164.7714537	1.410754371	110.4349104	29.43916995	138.3174224	78.40022501
0.409512266	358.3297497	4.472411205	94.77523155	23.09752267	199.6908087	50.57801886
0.238623136	136.2257564	1.906394969	72.85505862	17.34378487	115.7623733	24.6443593
0.146957377	92.71796876	0.626305217	85.15013243	12.81080736	59.73027283	27.18664395
0.452843287	206.7485548	2.393972917	62.74505286	26.99244982	158.3252651	28.11933909
0.370422539	144.9235626	3.593787182	14.98075887	11.76569038	133.6795996	6.819340463
0.469733051	175.3267008	1.902031525	29.65466436	28.0887146	143.9542107	13.82201861
0.20897737	171.7042599	2.317808239	102.0985216	20.90852138	125.9936691	25.59965883
0.389309791	212.2080583	3.353085558	44.72864706	8.331446205	292.4656614	13.70930076
0.866171248	251.6249503	2.732152133	69.58770107	42.82226998	206.0416271	25.19182782
0.285248806	186.6963045	0.942500793	36.51229889	12.46113042	131.670816	24.13579237
0.222110593	148.9497897	1.420193831	46.51962417	13.40687716	159.7836333	13.60957008
0.337297652	206.5737176	1.152245081	37.75349559	32.05463077	85.25565265	17.18343657
0.499524935	106.8884384	7.991198636	46.55444421	12.79509227	111.3929913	18.39871466
0.270500627	204.5257275	1.309294435	34.93471852	31.78585277	95.88600996	17.86906371
1.04241011	370.5268851	16.19196713	65.13538141	35.81944813	197.8279705	89.0010626
0.263907882	141.2586207	1.135837173	84.95405581	16.78607397	119.2179845	40.87800421
0.334071777	190.1390249	3.508486268	53.03448956	12.07743158	101.545998	40.30752379
0.937100973	220.3878381	3.509771751	31.88071697	19.8326892	135.1685261	29.72959989
0.382730596	222.8818791	2.339866528	99.90766211	29.63389986	175.8413496	18.51543534
0.37801015	226.1980438	3.399566553	65.17375038	18.07115721	251.9076656	24.05313827
0.25545287	108.6598157	2.681561053	111.8746913	39.04471109	186.4440189	25.08005987
0.471141376	174.4789743	2.721312795	27.63492708	27.8506431	160.2272466	13.7407126
0.298390684	204.5273832	1.786529747	37.68070594	15.87168506	178.3726967	21.01635988
0.754978305	295.9239863	3.176059423	34.68641691	36.31769409	257.0033369	22.3284219

0.280278068	343.8193798	1.093732942	59.39628753	16.30385917	198.2441585	38.02647648
0.502468614	227.24578	6.299780505	57.2769028	18.93223354	124.2937295	27.33828446
1.45149569	170.7987193	4.379937884	13.40385376	11.79795444	141.8495492	6.93019558
0.354781726	241.7786218	2.971629751	49.57144409	15.58414221	230.4555439	42.80449443
0.868694544	415.0712201	3.870252597	36.56421925	82.98296043	336.5231546	12.38472088
0.255341902	141.9453712	1.03747843	34.21402328	8.889040036	81.86246227	15.56461984
0.358321053	198.1046526	2.951927817	97.07188149	44.90817682	168.3779012	33.78209622
0.1667348	164.1598949	1.603814294	77.86235881	23.70853731	176.389911	31.10508417
0.224763565	185.2907817	1.548131678	56.68398507	19.84116016	179.2804519	28.16258998
0.421574429	157.022097	2.379933972	72.75849	27.64177084	95.49221407	25.98799261
0.696779622	280.633518	5.828602146	54.02658571	43.4602249	220.2070039	46.6314497
0.272201806	223.9807416	1.760868345	29.95967211	18.34428675	164.9922073	15.14448688
0.228667374	181.9166327	2.206319882	40.79557783	19.47107532	157.0025811	15.27208835
0.509877564	181.7382442	5.262924959	76.0510072	46.02637672	152.7295352	43.59378735
0.215788424	131.1781092	1.232014686	45.66369332	14.08157088	111.0369782	23.70699249
0.762002254	227.787763	3.345808694	91.9690728	22.92792131	224.9602062	13.14907553
0.085622196	100.3091946	0.10130555	9.740281451	5.270633863	99.78742556	1.560411892
1.357261576	250.9620355	2.32100508	45.72837687	20.21256	137.5826732	36.20992731
0.3165037	199.4062943	2.425106386	50.09860004	15.68324955	124.9223116	30.63173322
0.152549939	143.783917	0.938428196	37.41067978	9.987913013	148.4018466	16.11777991
0.382745166	167.9907385	1.756879184	22.29234675	17.91847852	157.7718477	10.0500686
0.305483969	188.7854075	3.708604943	26.1185868	11.00868344	116.8271777	19.452896
0.26091996	167.8869702	3.025220328	35.56141333	17.80018296	121.4209212	13.99654295
0.437368093	189.0071821	1.099687838	74.34630748	11.7795317	93.00238566	54.89848513
0.41784719	141.4860291	3.827815989	38.24960651	20.42001266	169.12071	12.23218393
0.476294553	240.1693806	3.841449728	55.8527612	47.50061344	159.9047911	40.67654781
0.252626502	147.8275476	2.232449936	49.43233123	12.6596178	101.9000444	12.95648964
0.552311701	198.6394895	1.992876365	17.92987932	26.6175431	88.23908273	13.10174368
0.193694357	144.8780846	0.999016884	27.70035884	15.95285048	116.9195424	13.36356879
0.34115773	170.1292381	1.046113589	58.7568377	15.36085209	97.52242751	12.72886477
0.178595828	147.8948715	1.634446682	42.3158478	11.53586487	130.5259284	24.42342453
0.417481273	167.6998704	1.449071825	19.84784777	9.959703639	92.3089448	7.377175431
0.172097906	152.4016748	2.189962126	54.27656579	11.65314309	173.7156214	18.63335655
1.914330035	327.4769843	7.995618088	23.21361365	17.94100546	191.0633209	7.521526509
0.382987544	174.70458	1.458832437	52.48600664	25.38567694	117.2310182	19.93202222
0.36269434	138.6455108	2.634932555	39.69697895	16.15127275	134.3295818	10.850355

0.164281886	115.8345985	0.982894922	47.4910387	8.308294877	111.9497058	16.16666783
0.193539749	231.4571641	2.237908609	177.9325424	30.97952265	179.2756572	28.57592058
0.338572977	164.1063623	1.409314712	77.71194833	14.66067117	108.2310018	82.87605808
0.438330608	235.6210916	0.968565224	135.9474962	48.72854423	240.283917	59.17546435
0.300593531	139.2609967	1.525890224	49.84305118	12.04147609	178.2304488	28.8723436
1.289510525	250.4446805	5.999572702	83.96177179	37.96516735	168.4668127	99.36908158
0.397769196	262.949943	3.818559149	54.5327816	15.06332031	206.3981282	22.18946551
0.730763607	159.1628793	1.567507707	57.31652724	28.61230649	187.4699208	13.66956374
0.269501592	113.2852844	1.019866782	50.96989897	10.95658579	93.52208874	31.2545917
0.410722948	184.6616791	3.287076833	35.41812718	12.60239695	141.2672326	20.19630481
0.169959179	183.1413053	1.040455216	36.1035076	12.39940143	152.9503924	11.08173576
0.365318316	146.7339901	4.118031836	44.83108167	14.93821466	143.545673	18.91923237
0.474081335	145.4529865	4.676886994	42.86177646	14.95594577	116.6644943	11.11250976
0.255033447	214.0910675	2.192821745	32.82924735	26.15760926	233.4342348	15.85676564
0.550433419	117.2501298	2.011790086	135.1486379	14.14443448	112.2897879	13.81832142
0.211096044	138.7195309	1.455003249	64.87080706	15.32838398	109.9747626	26.33421129
0.223794635	155.0525404	1.893181852	48.9911398	10.34689278	172.2490549	15.10014455
0.448503356	164.5376266	2.067069795	45.15727841	18.7118419	169.4459393	19.74328206
0.225318822	129.3324734	1.78722997	35.54342928	9.214718834	103.521138	16.69740252
0.346903856	121.6443825	1.171256814	11.27524891	5.296817327	104.765503	8.113340887
0.257658711	210.1875486	2.055152039	32.74806698	16.48255139	160.9160741	20.99189548
1.20459254	224.3799916	6.181777428	50.47443782	32.7859961	210.6561232	16.14743788
0.217202307	133.8908408	3.019954741	29.01652463	8.956807774	88.55355016	11.76979588
0.274240459	151.5507869	1.272961503	85.23677302	14.43900035	87.0874497	48.40572082
0.669530339	237.853055	2.454860571	112.6477548	32.04056384	186.4546894	70.93591586
0.25449905	260.7406458	2.739896887	60.34369748	9.358565635	193.4047414	16.13587117
0.701137732	198.8332198	2.318600713	61.21790127	18.26788292	156.9412622	13.0979571
0.299941421	66.07259456	0.200120478	129.6874756	10.74621119	38.66980413	18.54000771
0.174405481	93.89571538	0.654058561	31.04155523	6.344641203	99.15395054	15.21686824
0.22785886	191.7211854	1.109305181	46.10250857	14.78667459	153.3095963	19.74209355
0.185434159	162.0681692	1.12154088	58.11866245	19.13891915	111.1656557	21.27611994
0.303709231	179.2157731	2.122896162	46.88544828	11.49910976	165.7907583	24.08960144
0.681538705	202.3840426	3.211404686	23.35463309	21.1552952	102.4336309	18.73460792
0.24049897	130.5282147	1.066351421	44.1703465	9.84110405	112.9970131	28.28557785
0.534447637	221.6460705	2.448284819	28.66040179	12.59061712	101.8674439	11.86684094
1.347459019	206.0454691	1.840689239	86.34962164	8.896824139	68.023688	33.64617206

0.565760233	247.4541794	5.571822977	84.18055406	19.41949291	243.2098918	28.33374288
0.543551508	212.3380431	7.077732877	43.06415162	14.11460544	138.4142022	19.00310119
0.251390233	159.5687065	1.250810096	51.75618977	15.86550583	163.8751723	11.71064805
0.441645623	331.5760587	1.178538384	67.89430911	22.89700098	208.0621825	66.54077369
0.182911939	117.5912871	0.455331594	43.2807164	10.66067208	97.66244297	11.25176413
0.15354207	129.87964	1.835739607	84.65996078	10.89871482	116.0678982	31.98471052
0.557090778	191.0397966	2.404190898	83.41720584	31.00117964	142.8762087	20.54582599
0.942694017	130.8125263	4.341410554	21.84504084	27.88975791	87.63730674	9.043646424
0.371495163	182.7618741	1.094990998	35.95490706	12.67219433	106.7447829	21.73999104
0.463655092	157.5707817	1.425876369	93.34540426	15.06975668	107.5691291	71.01502766
0.714501019	200.9247976	3.904300694	24.68905632	33.37942549	131.6461807	19.0039444
0.42106572	307.6663841	1.027506239	34.71370799	9.754392045	103.8813285	22.95770855
0.596902183	245.4275336	2.372824034	40.86203396	23.51430952	248.5797478	14.71997728
0.544196015	190.4513334	1.623813048	42.23244678	12.16528539	140.3363569	28.53400579
0.250772226	82.62146614	1.130947356	95.33276184	22.35883864	97.7778777	20.20186968
0.296809494	207.0055714	2.851641031	61.29871784	15.32306352	147.2104207	43.52585008
0.140840246	158.5725518	1.052196713	51.29795056	15.04498327	152.6613579	13.61322497
0.602567629	181.3497838	2.422599076	34.12349932	8.702322879	90.60095458	11.16453921
0.427344295	153.4643093	3.227797174	44.75223642	17.65434708	130.6149622	20.45066102
0.266819444	180.3285655	1.401334434	49.14242748	14.79629908	125.5916589	10.6308615
0.322487599	125.0465024	0.960062267	29.76751747	11.52005999	143.5405299	14.40860861
0.258431488	167.1920542	3.300788635	52.93968238	19.44249245	159.4330554	22.78011804
0.578222895	228.7137129	1.399476434	48.26821158	21.73983574	108.1853808	7.232178129
0.382677671	215.2704809	4.587560315	91.28564882	27.33525281	190.1635813	41.01990506
0.190626843	156.5059625	1.38739386	73.79829266	18.02764424	120.7154115	23.53730491
1.318675429	290.9841486	14.28719649	72.77414658	42.80360161	267.1807991	43.65354831
0.313980457	134.5475302	1.749431215	41.26257874	11.56346644	104.142497	28.94525459
0.412273549	179.4525525	2.945628217	41.04634788	23.05001713	162.7942418	17.93135573
0.303239114	116.4569328	1.591774036	48.2202753	9.823118047	111.4361258	25.66787564
0.216344735	217.5122631	1.015553867	50.81815627	13.76305139	181.8420492	25.92451848
0.901174893	175.828478	1.822634203	35.47755574	21.3665448	200.3704703	11.93759743
0.167028272	125.5347586	0.613038824	37.51258634	28.19124054	94.0459039	10.70903973
0.15209377	93.75068474	0.866689648	33.47229603	7.515689915	108.7026002	16.52245242
0.257043749	126.8842272	2.46690042	54.72859617	13.41255843	144.3214816	19.65445701
0.22674337	128.531894	0.821871843	81.43003955	22.87374007	94.0675409	24.51349251
0.385647871	207.5836298	2.215266591	30.92637917	13.92590335	192.7905719	7.944473983

0.194394224	137.6699943	1.36556739	74.91611035	13.36000222	103.3844472	35.58899154
0.487577944	223.5841417	3.408764991	26.41013453	18.46689287	154.3400943	16.86222983
0.275673267	247.4139307	1.076447756	65.23823437	18.44494415	267.0331342	20.64466097
0.414912302	258.081592	3.101887282	60.02594974	24.4074672	254.0427295	30.41152112
0.243474726	221.222283	1.788812247	61.80459697	11.47088744	216.5137862	29.97322531
0.448483094	179.9371948	4.3967387	25.21399838	11.94630766	164.5105582	13.84783776
0.296925551	142.3434865	1.774427919	46.73098028	13.9792493	89.89081912	14.25522995
0.291013514	185.3474337	2.411648752	33.3454046	20.03941221	134.1279565	15.91207967
0.802091615	169.9311087	1.071105847	97.4021675	22.12529482	93.53357878	34.33727881
1.285130694	353.1979364	7.168357757	48.68665103	18.32453327	211.1400308	33.38662407
0.20978539	209.23441	0.945713454	43.54937918	30.61236681	180.0889197	19.95734095
0.382097014	194.7248349	2.636844314	36.99798332	25.43513008	199.7140387	26.49173241
0.426407238	85.8189252	0.704992129	169.4930376	36.31632446	120.6398412	16.77017672
0.889847056	256.4860654	5.184744428	80.12166501	22.79721216	207.2558662	50.10533084
0.571573284	157.6408384	1.188139785	77.61944993	19.50446476	187.2785511	20.5156726
0.142239516	121.1344001	0.450761199	52.66355162	16.60334933	85.25624866	15.37425045
0.33190233	152.3397883	3.090720528	70.97634411	16.09720033	129.9527888	22.65864655
0.309557423	138.782559	2.876402206	44.82448865	12.16457368	192.5530331	8.888958103
0.76060192	160.7209068	7.034656726	69.18005817	37.96078462	138.2859511	28.93847682
0.386335813	162.0307573	6.11518782	40.30016644	23.99991901	124.3764307	16.39840951
0.184975325	209.2163324	1.516092877	54.90512839	29.34847085	195.6305046	15.05097004
0.38248156	143.431527	1.737159364	66.45323561	14.04494858	126.5727351	28.58347972
0.229438871	180.1445176	0.614841706	366.3631778	81.07349974	91.07420799	99.96986042
0.491741403	142.1820176	2.097097299	41.74120373	13.05343384	97.51731277	9.586240444
0.083370159	122.6434661	0.45318619	23.19418807	31.94332131	118.7933878	11.13010151
0.426152215	273.3463269	1.625124458	61.28019221	35.22461003	177.6046373	50.06413072
0.269183629	211.8250842	1.475810376	59.51258827	32.71107463	190.834421	22.40758911
0.429213759	273.8572666	3.598674241	44.33354006	17.33648109	167.9259946	18.09755638
0.216918311	185.3208408	2.409070601	36.77667346	10.23951442	83.48065135	17.53731666
0.216897727	141.797681	1.708063887	36.90118665	14.62636529	163.274539	22.41690323
0.55876997	331.0123447	1.218705167	86.40869508	39.13700735	145.1405523	46.52072281
0.628157719	182.1072119	3.5792165	55.82860051	17.63748655	200.6963621	14.36195264
0.484848769	304.7246922	3.406609426	43.46610615	48.42351547	231.2843497	23.36522489
0.451447135	113.5138535	2.685898484	24.72949133	11.12772478	75.61208018	10.90160153
0.169539901	84.26789268	0.185999325	76.16048106	15.51334335	125.5778778	7.644488095
0.167910837	148.3635954	1.337996042	69.0891855	12.73269815	109.5444619	40.98610411

0.411675025	163.7658062	3.345284084	75.29525333	20.48732263	148.2036624	34.28338383
0.274976185	163.0106534	1.521469512	33.17725135	10.93411497	114.2299035	24.26419756
0.304590214	231.1997699	2.633281441	92.7269426	15.9300119	188.7027035	33.57653464
0.302759104	254.7377114	1.281939007	29.44579444	14.51777591	198.0922789	17.91455404
0.190728567	99.31486189	3.493388902	58.4189202	26.59174936	125.645205	21.34074173
0.14868623	127.5455264	0.895598181	30.91918695	6.93712709	104.082565	16.73580075
0.494764154	205.6050185	3.232960825	104.6796179	19.37634947	161.7567457	53.91574446
0.215669697	169.3174612	1.798465369	39.48786966	17.38455061	162.8984094	19.84459687
0.322553575	175.1349197	3.77312779	74.65207363	45.56221365	143.4799683	51.69207352
0.173179309	120.3089334	0.95433676	37.04393759	9.883251248	91.49730709	21.65385519
0.285103693	152.9161221	0.556864543	90.61344224	27.73336986	133.9064905	46.42589273
0.131389877	124.1224939	1.060258409	41.59059088	8.223087725	100.4923914	16.06170722
0.339608389	218.4924542	1.954081455	53.12772345	19.46141199	162.8514945	27.74336032
0.439627622	245.1235789	4.712160298	34.80575366	14.10958052	126.3728702	22.14952485
0.580107068	106.5042382	1.099522028	19.66891144	13.61062711	68.58038031	13.23316964
0.645554917	106.9572753	0.615498913	101.2025848	26.54830102	98.47801344	11.28302308
0.173631832	154.7763211	1.61392913	32.06785356	17.82250066	114.1938596	14.96046761
0.384819592	126.980196	2.456111213	75.99444391	15.55095016	119.4071643	40.65827999
1.155785654	282.451882	7.261565375	27.8218705	22.45976889	182.9423549	15.67503195
0.361991284	162.5470184	1.793865793	74.115661	22.87114297	134.9285685	34.99525555
0.2446537	104.3257871	1.166642652	137.379338	26.36120744	131.2223252	25.959362
0.449836947	155.8529821	3.124240004	46.84701714	16.20888455	96.46824951	18.18038388
0.225070025	245.4004584	2.16420129	79.00857988	12.90823526	161.6285716	15.80706569
0.322578768	197.7871339	2.873028257	31.36920699	25.39125577	166.821667	16.94848788
0.254856547	208.0269334	3.588780738	47.06390018	30.50316835	251.1929698	19.4808488
0.288485823	122.6048672	1.293467913	8.837372463	2.814395549	49.37659567	2.52859515
0.321698315	185.7656316	2.919120434	81.00392933	13.84945533	168.7451037	37.30479118
0.306592322	193.898043	2.790882095	53.61878164	18.37416685	213.6265934	23.31964468
0.426323481	101.7727128	0.857995868	59.81485313	16.09770856	85.30361588	15.82567132
0.216949429	147.251377	1.820750178	80.04928998	10.68693706	171.1814408	30.68920611
0.386406401	182.7636811	1.743736652	18.98843802	10.22458803	85.82046986	17.10844511
0.843682568	276.946316	5.410426435	19.56424595	17.69539058	203.9129828	10.85890611
0.386639876	148.4129875	1.480562211	43.60864032	14.06225776	139.535139	8.563377348
0.233253659	234.9928497	1.171861084	87.75789893	38.27052189	139.2661466	29.72981958
0.382502399	150.2019123	2.940840174	62.12784741	16.71406426	117.6698498	24.21861859
0.262860503	163.5013492	0.577690027	139.3780297	60.90197093	116.4420223	40.18002832

0.226559238	134.5100789	1.656613877	45.51009949	12.93149913	111.4473967	10.31790124
0.32968484	224.1722597	2.198524172	82.18035055	22.78711598	271.876403	35.15668292
0.240841991	154.3857276	0.880745306	44.27509742	10.97011662	143.7566121	21.99928421
0.261353771	149.6725027	1.361011809	42.5251032	18.51936402	164.3684966	28.77994505
0.246169453	146.1905234	0.883103725	45.77270173	16.7456027	90.60782435	19.58336338
0.53534785	172.6115758	1.345048884	166.7468114	43.96999757	114.8852641	83.52453463
0.597211281	290.1389904	3.381066368	30.0220038	12.10014511	210.0551589	19.44830599
0.408784614	335.0928956	2.594923421	34.5469441	42.30255428	264.7128569	13.59954357
0.421878128	217.6174357	1.958983808	44.46397284	12.95658052	129.1867646	36.37756744
0.245305415	117.8362766	1.058884972	39.66700259	19.7951741	83.32688662	10.77638617
0.696689545	258.6905466	4.606940335	36.38991795	25.32993626	125.8612458	21.84259845
0.444594073	140.203276	0.50150162	42.2818325	11.05471027	131.4145141	8.593832606
0.41871047	263.769431	3.247175836	64.18446308	33.33810208	171.989283	23.70121309
0.444333354	190.5782156	2.009397236	66.84591827	16.43740552	198.9517331	19.21529427
0.24952204	114.0435137	1.808412553	134.4932531	51.65131613	129.7541345	25.95465423
0.354483357	229.5204948	1.358356193	55.38044399	30.34721634	150.0536904	25.90750185
0.392491366	248.7625031	1.522578525	84.30762568	35.28017356	128.8928943	51.87867084
0.179512333	162.4829455	1.141562985	41.4823083	12.43116692	134.6955132	15.18324764
0.131230392	117.9739413	1.224555871	46.05187118	7.779382848	109.225389	19.45090533
0.061161958	52.09682739	0.214048714	122.5143646	21.43729154	53.72882248	13.61440158
0.616943939	156.8140917	1.103229243	32.53970604	19.95451823	87.9287825	8.934032456
0.723644394	244.2565764	1.167718456	62.81854689	23.35317539	152.0093741	35.30390913
0.558069559	125.2083213	1.38868191	118.6760541	39.64107915	98.52494102	15.22953088
0.501382493	237.764138	4.595493533	77.91363637	22.51110058	247.3042283	34.96948062
0.973771428	211.0288331	1.490785064	30.22305481	18.13336905	233.0079042	3.704877268
0.104589421	117.7368524	0.570147192	45.52660429	17.03597741	95.69568488	20.48858792
0.749632345	211.5223074	3.323892325	36.20919602	26.64160398	190.8909298	14.16745953
0.28949879	144.0154562	1.698904026	23.63451909	10.50000113	128.1287536	14.06599884
0.24062094	125.5942013	1.056049612	55.32352867	13.55694349	136.8800502	25.30864358
0.156387813	145.1677013	0.935496451	31.40726466	7.410259217	104.4729887	11.33025601
0.581487589	273.5877469	5.189671521	57.76963297	14.92254801	244.6560091	12.80325846
0.475351798	225.9103322	4.409889947	101.302177	32.60635411	223.1517039	59.12900912
0.283457723	229.6107995	2.214006397	27.57819096	17.19516749	170.6533956	12.92739737
0.847706988	168.6755308	0.553646573	65.91669153	31.39546977	114.5402399	28.13703568
0.275805668	175.840238	2.810959671	80.48330555	21.49778583	165.6167635	23.21574096
0.196104344	116.4329482	1.617999871	31.76368381	9.766339754	166.2726882	15.14191708

0.329834823	224.3883187	5.442775328	73.30696837	31.05722461	185.9994954	50.21583519
0.238260776	115.3143971	0.837925362	33.04246089	15.54183637	121.4691896	12.75863959
0.380522709	244.9260023	2.743123509	36.14219827	14.42559385	142.6936077	23.18334397
0.333935966	242.318921	3.212351541	77.98379969	19.23373211	196.1530605	20.17799546
0.654825504	223.0927959	3.199453477	67.02625522	29.40453798	218.1814214	22.60840457
0.439465535	328.1455509	1.459567257	56.3323093	55.31013517	244.6752156	26.67257486
0.418648727	160.327198	2.234187568	85.75803697	29.02771668	203.5551578	30.38077377
0.443593378	238.7083061	2.72415907	33.62248431	25.31229946	195.9924257	15.62213622
0.314223588	164.8629442	1.711055234	44.91914387	15.0646025	174.3416284	17.67525197
1.310287872	358.5085185	4.889431479	55.50919523	23.16126241	195.1125035	36.5438725
0.275404738	177.3627339	1.137568436	40.97115357	11.94031452	144.1034697	18.69830947
0.857188226	199.5981298	5.22933726	61.27182548	37.29450224	208.1071936	29.83985899
0.426506172	193.2112444	3.384176026	41.90766998	24.1856567	149.0742658	15.1482763
0.677182772	277.4516171	3.402013116	72.8068598	25.58980044	183.7748728	48.96166279
0.61196572	152.1685505	4.594090937	51.77672382	19.86305551	132.2324946	24.4447345
0.314272035	219.6195207	1.50057376	56.65294704	22.59639369	141.710759	25.1997724
0.242933958	209.1389294	0.650325526	34.22891205	12.55630866	122.922949	15.5187181
0.264328809	151.9728164	1.672963561	63.11776859	18.2537501	145.2453733	32.56501142
0.420814789	177.7836056	3.069486842	64.33448771	25.33021386	161.9008734	30.53282486
0.492002946	148.5029054	8.124479488	80.24941766	26.16470105	173.1093273	20.28231714
0.189274723	125.2785294	1.859971168	77.19232909	12.08982007	132.6799849	25.97387152
0.276497222	163.5153788	1.618949672	64.24506337	14.20903656	148.4019427	22.54280242
0.505490393	165.2220008	2.958920346	30.32901725	19.37633428	86.32131318	15.2430024
0.218114457	219.8899533	1.599670467	49.25069317	19.4960369	149.2191247	20.17077148
0.380046656	148.3814872	3.598328451	57.24055873	15.73376282	131.1152728	42.90536294
0.311650736	217.8941669	1.516802832	43.92316091	22.4311364	153.9615674	30.42237714
0.170501738	107.2760636	0.669741644	76.05013861	9.955980505	95.14303796	27.7120258
0.161524119	109.2369368	0.931783272	40.87099154	10.57301611	109.0343658	18.01131255
0.357766035	100.8252419	2.073964291	64.0486337	22.81490729	110.3290054	16.27712765
0.271760522	229.9159224	2.303133723	50.52788561	21.24871608	200.5763388	26.79195214
0.196230693	204.069961	1.81238288	97.59171035	28.46163021	198.8399503	34.79151716
0.365363359	101.975339	0.960017395	12.32572496	8.452139881	95.63564602	8.448435229
0.317612549	141.3806172	2.378213153	41.91768877	13.101955	181.2702317	28.93049604
0.788653625	236.3338037	7.369733377	51.49071906	40.9993708	237.7118667	17.30856267
0.243724096	172.4939047	2.223430299	43.24905115	15.33375602	177.7505181	24.74512412
0.648556618	199.8914878	3.985799903	72.43615293	30.86689983	210.5139941	43.08525996

0.522957789	190.6657438	1.755818532	18.3302882	23.243418	157.8991815	5.072537983
0.180537189	163.7957964	1.549319113	79.42998377	23.51180179	180.4663425	20.77355985
0.316156041	186.4543922	3.028648366	58.75401732	16.67400487	161.5785247	28.05991904
0.166637197	130.9857506	1.001543543	36.66587826	11.33536832	59.4941424	11.05054956
0.263524143	179.569049	2.749263686	97.59080179	19.40540553	208.9422224	40.37111601
0.602711229	295.1810041	1.927837475	59.76295127	23.07999751	336.6373361	15.39618426
0.492289209	163.6441328	4.075655243	45.38147824	16.57296626	123.5931097	8.800778025
0.236902363	156.5350171	1.333328066	84.53587141	30.84414997	202.7093027	19.72964706
1.04569448	191.1904743	4.728346109	267.1799691	52.56416375	254.1738605	35.00814153
0.208008708	98.11909814	0.984788602	45.60420987	16.84928456	91.33124718	10.06754758
0.3272116	200.1115892	2.867300957	75.55091785	27.55278029	157.665839	24.605764
0.853544608	251.4591701	7.537101796	48.54800267	48.12199177	235.923629	17.75156302
0.464215851	217.6622193	4.948596326	61.09611473	35.80074389	214.2193322	14.73040737
0.622064699	128.5278857	1.948697467	32.90805921	32.46904293	101.8250988	14.31841139
0.154904503	109.0904954	0.540900808	26.38594367	20.11246133	71.47093943	7.797719674
0.591553686	151.996559	1.126471765	56.1184131	29.37218955	60.98146124	12.33140821
0.26844542	157.0037314	4.087608202	77.76211364	22.45634657	118.0367312	23.1948195
0.318044674	181.4432715	3.386138788	77.07789903	28.97725756	147.4088019	24.88988267
0.481854754	164.3382238	1.019289731	69.66540329	34.47496758	168.8024644	10.96446381
0.420963546	254.3932452	3.158081398	81.78567924	20.86275135	251.2521998	42.29347124
0.176432582	128.4735281	1.653448189	82.68309011	17.58415721	124.9771262	25.76834059
0.114923144	99.73502244	1.134336436	42.85289095	11.77752247	75.40844817	14.70302817
0.263894806	164.7396376	3.291461015	47.74972958	16.79657096	190.460351	22.47703221
0.171690401	124.2990968	1.261735604	84.23106035	14.43511797	115.1843736	29.99009624
0.378828293	165.4153031	1.528667738	81.97128053	19.75307057	158.9589808	21.49066852
0.520217004	165.1988209	2.208451364	32.53188919	26.52684552	83.23525185	13.5480474
1.061653296	237.1554249	3.549432163	209.0106863	136.4720357	183.0729768	25.45975222
0.520107079	128.8658389	1.027800623	34.70562162	35.46430493	114.2768923	15.02766675
0.229612531	182.3457321	1.537981615	39.76956112	22.34450909	187.5962479	24.37852075
0.438182728	116.254187	2.929382364	69.38309412	33.75824348	94.09834733	27.25007305
0.454174961	220.6899347	6.21910054	58.47036262	20.30537042	138.9698056	32.56332719
0.491679126	110.4291043	1.329774602	167.6537541	54.24674237	187.3070981	16.94870872
0.257191142	114.8976683	1.325762831	30.70248613	10.56549198	72.95114766	13.50741659
0.222115054	151.9060707	0.901124732	16.93409629	14.42077554	88.85559481	6.954987076
0.710657575	157.0339031	1.471917317	133.0637602	33.90946643	151.1925973	32.89593092
0.351643725	171.6133826	1.422619528	38.84967682	19.37692584	155.1467838	14.25286904

0.292220092	121.5939674	1.273539238	43.92100529	24.79384834	78.33750239	16.90068605
0.095398044	103.8817936	0.619993838	30.75063613	8.665144962	73.6536605	12.41420954
0.276373228	132.0341057	2.14388767	56.42570868	14.06998832	93.97337323	17.57723879
0.360660213	101.8685771	1.805376196	46.4156459	32.50585898	175.0139108	8.12773551
0.200273332	160.2180569	1.981045768	43.15145337	5.99302789	98.89516768	10.97753359
0.589899922	220.2935288	6.498101689	55.71338736	25.14053608	199.7082349	18.07306182
0.375686606	154.7490515	4.007783035	34.69445505	17.79030825	134.0746452	14.55611842
0.555266711	177.338397	2.054268108	17.39522272	39.44698522	110.4155559	16.15741332
0.156904484	146.5820692	3.269204894	40.65487376	9.993491471	117.7587283	24.2345332
1.061470673	187.6501651	3.351623723	39.54912939	21.69989434	187.5267352	11.75279418
0.334002436	146.9816944	1.034019554	65.30731664	27.24036549	84.38981921	23.08048819
0.211912513	138.1523783	1.10447177	12.27699291	24.02341499	54.50281379	5.712942704
0.443926269	129.2050345	1.263338214	60.49089023	55.98927258	61.19971893	20.26958886
0.245640114	191.9964606	1.363574471	54.64632899	32.68064467	127.6222312	14.31092469
0.868977724	178.206393	2.058784592	25.5247216	24.07198863	85.89217719	4.536277752
0.284765332	141.5739202	0.857107511	42.02637911	25.31336757	162.5747292	11.3881523
0.288083553	167.6404064	2.074105056	62.61384647	18.65803004	166.4421289	38.88391833
0.513216582	269.2315008	3.40396869	31.15282885	25.34512	129.3442496	16.07229432
0.290238109	160.819877	1.819324491	67.83530692	22.8873074	123.8267569	24.71540382
0.297825802	74.05344572	0.605993995	150.1783573	36.50206326	103.39488	17.3543706
0.385197905	135.5041241	0.854711096	32.56342505	31.29717449	98.74250297	15.90990346
0.161270228	145.9843383	1.471492578	28.66278263	10.63665647	108.5618444	6.765700239
0.33182459	210.2042052	1.571802282	35.5514168	23.83385993	112.8141035	15.64420677
0.170315444	93.70530112	0.422842629	57.84082219	25.71155743	91.10839687	9.098367143
0.483645206	141.5618188	1.934580318	56.71177723	22.99235173	103.0549131	14.54033071
0.279852549	174.3009586	5.508435084	65.54788431	25.56598682	158.6017232	25.898
0.814782732	263.3164192	3.192307804	38.52149526	32.73294825	201.15306	23.6781865
0.167575688	112.624872	1.44972956	31.1067552	10.94893738	106.5732541	12.82687755
0.087492411	104.2919349	0.671423378	24.78708719	10.24956359	100.5005482	6.896454936
0.330705752	106.5649501	2.566823322	33.048972	30.033128	105.0623489	10.79062795
0.367290889	237.6988782	2.327104235	77.83369239	13.69987585	234.6024312	18.51931815
0.174504431	113.4349072	0.467069298	59.57624896	21.51373438	51.13054979	9.291185126
0.118281908	102.474703	0.662887473	32.94869889	12.74165092	73.46933806	16.40665219
0.593265278	263.7701177	5.720768734	116.9294135	51.90615428	203.0687726	25.10861124
0.223478719	131.3877034	1.26825518	56.00249736	17.71456848	134.9031528	7.329396004
0.208854115	133.2025423	1.566468194	27.55112974	17.64351508	79.31806514	11.50366545

0.155372336	177.9785782	1.269400521	51.29512889	29.18126697	212.8696732	15.41090213
0.145585387	135.2797787	0.324261435	52.82603162	99.17598454	59.46272774	34.32513633
0.560379394	192.0564489	1.526843113	28.0305083	17.62448468	139.0288727	12.2748952
0.306848845	156.6781692	4.19050137	57.98149719	11.57132276	155.4383817	17.1455036
0.273328084	164.6413529	2.748221625	62.63691246	14.91314608	204.3086785	16.23844988
0.425440492	181.9853975	2.277778749	51.12523149	17.49710967	128.2334843	22.1855255
0.351701834	113.2557904	0.973857847	105.6168825	28.40730456	68.68821968	72.2416063
0.205745852	116.8515834	1.916519573	66.49008522	15.8982451	104.7103997	24.70916341
0.468896108	223.326816	2.720766384	27.5543364	10.13711983	150.6821276	10.31384701
0.414358406	208.3366894	3.339948737	57.17038391	29.36606345	149.5977325	20.95940532
0.236298106	161.5125146	2.935395042	110.9602602	28.52482845	219.2559678	26.32332398
0.585366798	154.115707	1.68096375	17.47070619	39.17739201	81.12050461	12.64198208
0.454078513	135.8728004	0.670139273	88.3605067	74.26670503	99.51856883	22.49208543
0.371353781	209.912903	2.677721479	34.11833429	25.78357058	116.2717004	27.76226857
0.086780604	79.89233035	0.348485114	97.4587746	12.95326536	78.71703548	13.02336562
0.246129941	130.2128999	1.130844756	26.88947587	9.41485129	84.69952842	9.580693562
0.162288416	120.3002113	1.690312059	48.52065781	9.78922993	124.6035792	4.488858491
0.343413199	149.1089913	1.168627137	31.22139944	52.35684684	77.46659203	25.52288278
0.278677571	188.5819026	2.301793346	67.04913197	23.74936669	151.8910961	28.36359271
0.231403773	143.2347659	1.259678785	29.70646231	13.55827772	119.3630472	9.945914274
0.520610446	212.3585438	8.819052683	32.66484852	13.9068671	164.5103243	11.52420141
0.257758999	190.5721014	2.581380179	93.6828447	18.49784599	203.1615575	24.84314397
0.585360636	199.7336087	2.783052246	26.9533376	19.89493438	152.1721491	9.917719864
0.414051298	152.4813495	1.287496133	25.48100711	30.37040047	69.56149666	7.04996386
0.317151024	234.0290258	0.857951922	38.95888885	22.57348034	97.84063528	20.79116183
0.263795179	158.7917404	1.117700878	70.32413707	20.21155335	151.5374812	21.95998263
0.357607673	202.6699996	3.065701856	44.72432785	23.02225585	188.9049851	18.73990278
0.523185111	322.3501939	2.377923969	45.45492437	14.72656715	181.1516065	20.59759405
0.40918222	162.1797564	2.578833033	42.33769058	18.14387717	173.8673642	15.36694183
0.662987672	275.6820778	2.575453276	42.34591204	20.18565194	126.0140827	22.85671657
0.47022382	149.6220668	1.311280622	11.02108597	21.73804487	83.10683263	3.730944763
0.368469428	179.2264084	1.635188578	42.52009664	24.15051497	118.9114476	22.01947396
0.164138131	89.95319105	0.460253878	74.11027143	33.75493531	97.38896013	12.59521637
0.315039714	220.8602994	2.574106106	54.53769256	21.84086738	166.3738605	37.33749177

Luminespib_1559	Alpelisib_1560	Taselisib_1561	EPZ5676_1563	SCH772984_1564	IWP-2_1576	Leflunomide_1578	Entinostat_1593
0.21153965	43.25385608	15.50773259	470.3242297	13.31335787	23.96540998	160.4222745	16.4433724
0.059690135	33.6283087	6.6747164	242.8270377	15.15786416	17.81471934	224.3199032	9.248701085
0.339552794	71.35878092	17.08549239	349.4360684	15.87025539	19.77923374	177.4395108	11.68324695
0.082933401	36.22747537	13.04258237	187.7045404	14.3500691	14.5289089	206.2884696	5.329500047
0.259085893	69.21872214	29.07458929	446.8400768	15.02264651	20.81487007	303.407305	7.463434467
0.043395787	31.73911403	6.869873199	171.662016	14.0023123	12.87589237	118.0009142	9.444783576
0.06270291	32.67763109	6.058921377	249.3135667	9.845683863	15.96488595	138.5894021	7.611558028
0.204063998	95.36852062	27.26095021	352.4817135	13.9948755	24.12168413	212.7764477	22.10382366
0.361107781	113.6106002	32.14604793	477.0645814	15.1034609	27.05735793	252.746225	17.84969999
0.077137207	19.57680816	3.722300218	140.3314066	13.19945766	11.23908374	112.6837296	5.388941511
0.10149799	22.06105748	4.966544408	176.6634125	13.27613059	11.484226	136.1140338	5.561586621
0.140553371	31.20760261	9.481802693	291.8156856	14.10629184	16.44571869	178.1955265	7.360216248
0.129627628	20.67585902	3.96263991	229.0453318	14.33761997	15.53337022	187.2793363	8.172905226
0.514880631	139.0828057	53.08913081	330.3436413	15.44726134	20.13289243	237.0621813	7.011615476
0.0865225	46.70168846	10.0431648	271.1803009	13.28987063	15.99190481	144.2063522	5.113045938
0.072926579	22.49973227	4.356868823	256.1409659	13.83426077	17.09477347	189.6624653	12.96201688
0.077552122	39.27296612	16.86039683	142.884973	18.30449226	10.97353357	120.6108495	3.194284986
0.262221755	97.10510173	27.64153497	481.134647	9.527958299	28.31968383	228.9006204	25.19987582
0.065733948	50.57268144	12.31776585	309.3581172	13.56717089	13.2672486	177.6772782	6.705928772
0.02485938	23.62874109	5.938703029	164.6936943	13.3404506	11.43804709	112.4005858	6.089114402
0.089477554	36.46668936	9.109270758	298.5647211	12.91299477	14.90750239	118.8008372	7.21419622
0.330940566	81.92461223	25.50868783	524.3851595	14.28228623	23.89794506	199.3989294	15.91137295
0.075752976	34.32209071	4.949791823	265.4949397	11.15976067	15.88345413	96.5343647	7.899207077
0.155309573	70.91812402	12.36748261	372.4427961	14.10588797	26.46833886	169.1756532	7.751932567
0.091946577	25.2907866	5.456260042	253.9693676	14.83910782	17.17932128	161.279923	7.523966511
0.070644085	29.87588918	5.358680864	313.341206	14.59839354	21.31876996	208.9555537	10.69622636
0.304875027	105.7923401	27.0437012	379.1367641	13.03905805	24.50654068	175.4097385	11.72682031
0.135549546	58.62562421	10.68492718	427.2643412	12.50435527	25.30863646	171.7685773	8.22381015
0.073855226	39.16638335	8.407335657	244.8238898	13.03889513	15.58431254	128.7282087	3.586575061
0.092635943	32.31079323	8.292111885	174.5408905	15.53203461	12.9328471	113.2423446	7.85632012
0.142319444	14.71597136	2.533733673	196.4652958	12.11156988	14.39392984	134.9698991	12.93264979
0.122315085	30.1077706	3.894748418	269.8473201	12.50558178	16.47091382	136.0400513	5.848427022
0.253154768	70.88192866	13.50939098	549.8682242	13.75561205	29.59441144	262.1427795	11.88988648
0.120476421	46.96727098	13.23140585	210.694773	15.76327442	12.82312169	136.2804914	9.192619028

0.452095278	84.50369404	16.54388541	325.3937909	13.02316294	21.94517196	132.411437	7.878031274
0.076235223	63.66624012	9.081320192	404.9927309	13.11257654	21.12248785	174.7059188	16.79689869
0.030751849	16.7309995	4.043064552	117.483422	14.2285616	11.34979276	166.061047	5.383014949
0.32787253	85.0078512	15.37101621	603.4068539	14.0573052	31.34527443	194.385046	9.393191188
0.096872971	44.12017875	8.104257865	264.2703536	12.50435058	16.05155186	152.2981904	9.256406107
0.081953618	18.2257462	5.142220968	243.1249156	17.53929055	15.82773619	185.4934693	13.94252012
0.285241337	107.5957938	24.20069195	293.6875419	18.34913836	19.09680389	139.7032807	12.26209097
0.09410923	43.19465432	6.903336218	218.9960026	13.10196792	16.23415336	217.5170934	6.108408877
0.145373648	42.0121904	10.53872674	394.9959125	13.00798117	22.69088965	165.146997	11.91981799
0.136541273	31.03709087	5.868067906	212.1546703	14.05166347	14.7511387	146.8106063	8.443207982
0.128369456	53.19126592	10.53119585	243.1743579	12.45025348	17.97957236	110.5274665	11.53231203
0.028745788	17.07167779	8.488530882	105.9509219	22.44919543	6.853286616	116.7224304	5.944126381
0.055245731	23.4578998	4.829227799	198.1197927	12.90116495	13.31015192	119.8292739	4.844149779
0.084227693	48.70010805	13.10239779	293.184147	14.27534587	13.7551366	145.1272684	9.982238641
0.09325948	27.39684237	6.003088766	171.4940127	13.3737021	11.88918539	116.3240788	3.395471122
0.05611839	21.65261258	3.577709423	189.5038461	13.08183911	13.32038843	147.810405	5.959127692
0.041540905	12.63406665	2.627656523	111.0568265	13.05732662	8.686300809	75.36072346	3.300707083
0.075187756	31.68355398	6.430451213	258.5837652	12.63936417	15.33494497	168.2749068	5.564176402
0.126455748	45.86542696	7.471513032	295.8646006	11.64826133	14.65000842	135.4063141	6.166562922
0.05931893	23.4550553	4.426030575	194.1539862	12.96135669	10.86214839	121.9242966	9.070316424
0.078160281	161.1245793	43.91222126	218.5484108	13.58486556	19.40881505	88.5156202	9.339410669
0.100149951	224.4868828	49.43922143	368.0898374	13.17604113	21.49967809	182.5968478	8.950137649
0.059120803	14.88280047	2.652155512	160.2813913	13.80296569	11.69098087	128.9863253	4.772046839
0.029571547	12.3028355	2.612508707	172.1109963	13.15487342	11.83502597	134.4358381	6.078097107
0.468273495	116.1074581	35.00817817	556.056995	13.10620573	31.6891051	173.011832	17.38284281
0.062836687	50.965899	9.152355519	260.8228321	15.54435927	20.1714365	169.9097838	17.56888443
0.490309064	72.62246481	22.10373567	499.1487898	15.51264175	29.0957616	254.4088278	20.11350269
0.134105209	23.33037281	5.980386453	294.0701576	13.75530099	18.56281404	177.9667627	14.63631446
0.222760753	80.22709398	20.43708844	457.6149849	15.33315579	26.98165065	232.8104358	12.57298527
0.067212534	31.94730065	13.71252119	211.9113909	14.71268911	16.46872237	133.3894365	9.451171267
0.067368597	25.57801012	5.342783918	206.710523	13.12582154	13.68105974	111.4486623	6.874843698
0.06729493	16.59678338	3.320860926	157.8613775	13.28253083	11.42700778	116.0637034	4.477155969
0.038213367	24.52183044	4.662491475	292.8159579	12.34801306	15.65911868	156.377741	12.42261618
0.886168229	218.7753562	69.82009728	705.0275013	15.37747745	44.26735495	292.5120514	29.02295212
0.145337844	49.4743798	7.495555552	294.4778897	12.61382803	14.31087225	169.8492843	6.974542061
0.086975589	7.958680827	1.298276576	156.561447	15.36303146	11.16061654	136.2567227	7.077259487

0.093817477	63.25945371	11.02593692	388.8740123	13.08436989	22.01404497	168.9804162	13.10281102
0.065776349	25.15237824	3.814838792	130.0917266	17.87104438	11.89900478	139.6346683	4.693875893
0.162634489	33.52809898	4.280960122	387.005933	12.43101871	19.1772567	137.2491212	8.862414625
0.663883442	179.6367091	79.04714821	638.2536505	15.18749191	36.9602529	360.7353043	19.86844811
0.095435216	40.48467272	9.969430491	190.4674542	12.95300915	13.81177835	136.4424512	6.94679139
0.063840158	24.72758429	4.165448202	157.1181451	12.73370936	13.17083886	113.8401012	4.09410678
0.095304659	25.86945467	8.84611228	193.2345793	13.61492775	10.82319238	109.3139046	6.884534725
0.114704155	35.518486	6.34819035	284.905412	12.87842171	21.16670321	158.0766842	9.654485011
0.307606953	37.2194754	13.41104884	394.1708754	16.81488126	20.39993669	143.6324125	6.96475242
0.112216564	28.81930633	6.872259964	216.0616431	14.99058807	15.84400966	153.4467021	11.60450594
0.17859151	244.7032263	87.72479347	340.3643256	13.09348438	22.96509106	169.6459973	8.012992128
0.241807803	103.392151	18.8453294	429.9987684	14.5428695	26.97268797	249.2422172	17.62845333
0.061420994	20.83325812	3.72957737	174.1846998	12.71733674	13.63670102	90.68627217	6.131899402
0.038721721	29.00737734	5.243336576	179.8555652	12.70905542	13.20863017	104.5268742	6.758388767
0.165982998	41.25294842	10.27042623	535.8516001	14.79103574	22.56306999	157.2378128	9.039892766
0.072406772	23.36143579	3.882134218	231.8086047	16.59601142	17.98015135	180.2828673	8.200954191
0.099172941	38.74194373	15.4350099	153.5664382	20.75040815	12.92105424	145.8555445	3.20472499
0.076606571	26.69987903	5.703564338	208.7829898	12.6188857	11.82808995	103.0681493	14.42308141
0.19181187	92.32425379	24.58241137	511.8414839	14.01035438	24.5588244	259.1421458	15.7071675
0.029485214	21.22198694	3.673431467	207.7228857	12.67646807	13.38578537	157.9148351	9.738450004
0.45608144	38.1184185	9.770233139	242.3699088	13.07328809	16.97714077	168.3995613	9.37760901
0.052086638	29.46125493	3.891014638	166.9627456	11.18006923	12.5891895	94.86819442	7.459088085
0.168251381	84.3939835	20.65342669	285.3695184	11.50948184	19.27490452	109.8166452	8.360340773
0.058161531	22.06084091	3.831569371	251.3245801	13.0172054	13.7384757	141.3905454	8.117254648
0.048156773	22.83020461	3.264628438	215.3787393	12.70702333	15.53782975	135.1389067	13.04483046
0.074476827	50.70537875	9.480586399	249.2076431	12.38555849	14.67339805	122.1542611	6.294222903
0.086735994	36.74324545	6.514025793	214.1928172	14.30485093	14.73488632	122.3865754	4.182934212
0.02586042	69.90877001	16.18928537	301.2938458	13.61386788	21.4010879	137.9527664	11.37784227
0.16099608	48.98398973	10.47974313	161.1184118	14.08284271	15.09875163	220.3104711	7.41044172
0.31673401	81.48291185	31.15472283	199.2003313	13.17313478	15.06953889	159.0889709	5.199784699
0.123292404	60.35800609	15.67388018	378.3367723	16.5813519	20.23512234	197.6518022	32.54242634
0.150373801	47.92562631	8.286599377	282.1438999	13.55833701	19.64746048	189.9922181	8.012010321
0.049168864	10.47737554	1.949934231	153.7655913	18.93728158	8.803358336	60.28621061	9.34937171
0.072881574	30.06922307	5.484159505	198.1420425	13.47549453	15.45904532	134.1136147	10.5543144
0.045259281	33.2495938	15.02227125	199.707833	16.50698538	13.19836203	137.2682382	17.76598202
0.114903034	57.10580881	10.01787172	303.1545561	12.38211219	17.53716632	155.9790877	8.270191289

0.101949947	27.26942437	4.451247311	295.060692	14.30361196	18.56943054	152.6528151	8.323622914
0.0774949	30.26754644	5.849800972	280.9140445	13.59919007	17.84132954	233.3535206	7.736319657
0.079447751	20.29682657	4.190905773	140.8873564	12.48774451	8.043998817	87.86292199	5.845406246
0.296208863	76.10158122	24.33072719	513.2941837	12.92039761	24.79282485	161.1758421	34.55976411
0.088705051	19.38458149	4.002083947	284.2317301	12.26347035	15.49480388	241.8571101	7.721446656
0.035853907	38.95798358	6.371119393	208.2419535	11.16459826	12.52333681	115.0766389	9.281675523
0.181324228	106.7408028	21.58361247	549.2194112	12.68533055	27.27136011	218.6403617	10.70060924
0.084519352	21.09652974	4.969428197	265.0516257	13.38785589	16.5813463	146.3319557	13.64197548
0.154378686	122.9650492	31.63040912	376.4256246	13.45546069	26.22971242	207.1331971	12.21970985
0.144363274	24.23448983	6.063230581	179.4715623	13.2789891	11.59810005	131.8108276	5.312581458
0.197805546	58.84532383	18.95690526	243.8718175	11.73532302	15.60951531	110.5537118	6.514518479
0.189401905	204.7537161	51.60004114	328.4441236	13.35686812	22.64906824	154.6853673	19.03410656
0.069172391	41.85119671	7.113649712	389.7874624	12.20939051	22.94972808	265.8597763	10.71370392
0.072950779	30.10429984	6.5139949	174.4829618	13.04193685	11.03503843	136.34511	7.814249752
0.028617366	15.63089362	5.08994679	152.3050346	24.0417797	9.102595301	121.2174894	14.57047518
0.10390392	36.93832933	7.311492327	247.2099257	8.908666401	16.48068899	186.8057189	14.28429954
0.035059718	13.07775959	2.31338578	289.1058073	14.6713272	17.28397364	203.8360701	8.672588042
0.050479045	16.66116149	2.561722044	273.123235	12.66168889	13.32975261	151.1367177	13.8585134
0.075116847	26.87270946	5.938466503	198.2559098	14.13528764	11.68175844	136.8051186	6.518438341
0.095817689	22.80421819	2.056608326	332.3216363	11.53819127	23.91789236	227.639338	11.11927602
0.338048721	77.54355782	21.14457931	341.9476599	12.9820124	20.79422708	187.3526679	10.77448327
0.101810261	41.28077069	6.130177231	257.756138	12.15520449	15.51457495	96.38152672	7.281139047
0.085280919	26.25409043	4.261780311	215.2443103	11.80180686	17.31229239	129.4853311	10.52193078
0.026747031	46.32721402	6.197420695	264.8811146	12.7038747	17.60438185	127.2556595	15.3859317
0.170704424	21.83735943	5.760259515	157.2014675	15.48375657	15.74880272	200.310663	5.821135324
0.027801193	24.50698205	3.989417507	248.4290413	13.34308877	18.57097101	196.3847241	15.44277767
0.616419274	164.1728046	61.58956322	500.3079217	12.55532004	28.50390004	281.9239231	13.89682776
0.055423918	52.943797	10.5566403	218.780814	13.11281127	14.71019469	132.6230158	5.889908501
0.11670486	60.4045706	13.79394655	225.6649197	11.83656517	15.6694102	149.2080888	7.258203028
0.306279894	58.15802547	13.27088257	366.5337778	13.70575069	18.38839742	214.7831317	9.204637813
0.095711873	129.1870846	19.10382342	361.3733711	14.13754734	21.67724397	169.0474493	10.77421774
0.091233161	47.9061401	6.502852371	320.0331328	14.70563732	20.3821201	208.7675226	15.76113825
0.11989009	40.22503052	18.92963941	176.0902282	17.03924034	13.57931856	156.8124215	3.997401821
0.086657663	25.48172095	5.913692221	255.6060788	10.68495833	14.79432644	160.0397998	8.650950701
0.07243451	36.57442327	5.235607115	316.6734406	13.18067703	18.3200579	168.7763566	10.05383045
0.138026355	84.02141868	15.29407743	541.1444958	13.3833003	24.72783893	153.2344927	13.63878803

0.054993867	42.01095246	7.369079069	453.392923	11.71476276	21.87415799	142.8050104	11.40060606
0.092451605	38.70838969	6.32171633	272.0516289	14.5243545	22.14062767	253.3618243	8.482270369
0.089161776	26.11710626	6.195679092	489.1854748	16.5063079	21.53928329	188.6385994	14.86188501
0.091783368	67.15446349	11.45309111	328.1180636	12.92068595	19.68631623	142.1267021	8.665374196
0.117121583	84.26053492	16.82240141	1109.204881	14.40675109	45.0078342	352.1059627	103.2300066
0.075831838	30.86115927	8.211786079	216.8170873	17.10409229	15.1233449	122.1207428	9.220936071
0.265284445	54.466286	14.8227444	324.2068937	16.46455282	18.18463579	150.6093631	10.60690572
0.071330852	46.96742732	10.08834107	208.2845706	12.83376798	16.97955495	148.6608084	5.705752368
0.087132354	35.48217895	7.117158375	257.1220102	9.259963461	15.37764235	123.7228776	6.187499601
0.140184853	17.95349137	3.946654699	230.1013557	15.06332289	12.24312711	128.7999615	18.59012119
0.319965634	116.8203865	29.58466917	425.3689143	13.70291977	23.40475367	219.7783864	23.67514208
0.13736528	30.75027021	5.557951363	370.34932	10.79851929	17.46522952	129.5331442	10.90829499
0.056355061	14.0175508	2.902266394	309.513523	14.23049267	14.49579349	147.6261036	20.95434365
0.433786462	81.6803533	46.24739216	409.6827648	16.6871436	19.48030317	149.4887662	6.345372883
0.059356801	20.26670306	3.482020135	148.7970166	10.50492913	12.73539401	130.0997888	7.237813936
1.682706404	62.651699	20.34584004	273.9306734	16.16537101	18.83882799	178.5956275	7.034122419
0.022102157	2.96622756	0.465695346	144.6814933	11.64807311	9.035597342	80.23897019	9.665144304
0.261406649	47.63807643	13.00849934	368.643347	16.33223716	19.68131635	144.5374323	32.48552389
0.111183545	32.23921252	6.451728593	253.6759366	14.34187988	16.30359608	148.8107412	8.224543142
0.055337101	18.91639038	2.659181288	168.5569426	13.62877199	14.17694692	111.6682469	7.136256581
0.070385984	38.48066166	11.09405427	233.3492029	15.78463446	20.45619956	138.4423468	16.26697158
0.065403992	12.76508775	1.482102705	230.1438632	13.03005767	14.08950408	117.0978775	12.95871709
0.130715269	28.33130952	6.123646253	251.6628536	13.75981782	16.65230771	148.1817542	6.025090445
0.140811022	82.1460888	22.30140209	256.1868776	11.72366179	15.33526631	119.4182175	8.606840281
0.147701455	21.53733393	3.68588552	309.4728242	15.80565858	17.34426574	189.1906949	5.879153638
0.292705916	81.71114093	23.62798144	315.6156551	12.82057137	18.2012017	168.0758404	11.75527301
0.065254317	20.49886988	4.05712877	239.6006627	15.98698024	13.4912943	176.1422053	14.64899778
0.09247797	24.83330478	5.240097998	268.4315828	12.11130706	16.93052535	161.4213275	7.894020455
0.046325387	17.42945763	2.983471223	188.1912136	12.30634287	11.38538628	134.7279404	9.496323696
0.062298793	18.46602632	4.082824236	196.0360014	11.75244191	11.86715355	102.4554348	6.914528562
0.075413531	19.49613261	4.102798847	213.3280501	11.81019611	11.80464617	97.66305669	6.89485087
0.206544263	20.50405029	4.703170725	310.3646987	13.70990351	13.12143094	102.57773	9.350650314
0.067215257	25.13364525	5.047849062	192.9121151	11.82226617	16.37399096	140.8068322	6.391543981
0.719069811	40.58354246	7.210346776	463.328083	16.51781755	27.99204698	187.9164079	5.585271871
0.051212734	40.84455205	8.832762651	231.4479756	13.68966027	17.1231306	154.1832781	4.739252413
0.069441034	21.66355421	5.273983257	193.8617535	14.38313079	14.22832462	200.9628749	18.06857419

0.044394527	16.76373562	2.234804699	167.7390524	13.26754991	10.42054058	104.5987593	6.018948716
0.168211925	48.76628141	12.9228606	282.7393801	17.05621145	14.0920972	190.8855364	14.63277779
0.094594608	61.91963243	16.11632835	212.9035414	12.25720333	15.99899482	112.5294416	6.545565365
0.098988192	165.5515732	40.6040808	355.3016017	13.30941978	21.87893575	158.3557371	11.6768617
0.08373438	37.03390498	6.180031141	250.1964933	13.22316426	15.95648405	117.0115562	10.89143374
0.307130735	356.4266514	129.3805199	454.9319826	14.02945546	25.37395324	211.4950527	19.93572543
0.17853597	46.96482691	6.939426433	423.6277979	15.10955942	22.66478621	182.0482805	15.95477656
0.177735022	18.71672626	7.122023154	354.4355209	16.04717893	15.3870849	167.9092352	11.57186035
0.078710274	28.11857837	5.091369079	168.6264094	12.37966792	10.86349539	103.3997797	8.166585461
0.26437332	44.24398768	9.395063361	333.5662554	12.0441645	16.66503032	118.3753247	7.106150556
0.045970157	19.5027288	3.687178836	201.1505163	11.34167596	15.82778309	121.0357927	5.645782156
0.067192082	18.34328979	3.059048964	201.5944971	14.82285402	15.25755729	170.4676074	8.280496113
0.076855703	14.61737314	4.95146208	282.1276515	17.36038093	18.40108371	179.8848001	19.32647671
0.090359829	46.47389189	11.07264769	364.2852384	13.72865977	20.21510414	127.8113638	6.28234853
0.110861922	27.11306018	8.63324384	139.8475129	19.64431557	8.790039265	120.3400619	2.229217522
0.061490002	45.73760531	11.20529753	177.1076018	13.81557937	15.60893696	126.1843671	5.622715434
0.062202169	23.59211432	3.477596785	211.402097	13.77998922	17.85117844	185.9340321	6.334330306
0.183552696	82.28880567	23.24674927	344.3972552	13.82245796	15.48840102	135.5945986	20.06376186
0.055190246	16.53489112	2.307305292	165.9226492	13.11625499	14.17981551	129.6810031	6.034482007
0.04579732	13.81814985	2.156493896	197.5526575	15.1122119	10.56498511	114.9479876	15.32760689
0.052803743	31.02172506	5.650528389	269.9993151	12.84512362	18.4716201	153.9082455	16.71267848
0.353719492	75.19632074	19.19724149	423.3157418	15.33808746	30.31683169	223.7176128	22.4588141
0.163777912	37.75786397	9.5517981	145.8562353	17.36335035	12.38880338	102.6668082	4.478901146
0.083110642	55.49664004	16.23730873	181.3926827	13.44734453	14.39288393	125.4990339	4.201456128
0.254593124	160.7090247	46.90770204	447.9464658	15.13876967	27.73494139	257.5217717	34.95948437
0.120257696	43.51416065	7.908437629	301.793822	15.44393506	14.8857305	141.7606097	6.732768941
0.327124395	67.59616553	25.50779798	441.9781597	14.80463919	21.933937	145.2627848	16.14785135
0.103094748	16.2934482	6.039906549	112.4722206	20.19744486	7.29756638	92.64364466	3.330862445
0.037893364	9.66469843	1.49316042	159.0037332	13.88551357	10.88587177	84.87680934	6.954230729
0.083393415	33.49441615	7.054959524	203.6358331	12.17290302	17.54049847	135.7784848	10.78783461
0.027482079	20.56564865	3.320236417	225.896118	12.02839106	12.90645801	131.0043136	5.624166877
0.103262503	22.23435251	4.020588674	234.8505782	12.35136295	19.58266222	147.9545591	8.999784153
0.157525213	47.3267673	11.72360036	310.8152899	14.68862892	20.18314868	196.4356942	15.0749344
0.046797269	21.05003792	3.658106052	182.5675607	13.25131237	13.37945222	114.7903747	13.95089522
0.241432209	32.03921304	5.507288544	307.2676696	12.98601768	21.5734326	160.7506285	16.23798384
0.131871909	25.45004116	7.960764966	219.2291718	18.15006436	12.83082455	138.7076266	13.50252424

0.061774263	34.91526323	5.344675736	373.3943253	14.18298896	18.67644403	197.4128202	10.09513235
0.071930339	23.7031257	4.058620014	388.1635297	15.19519681	19.47332951	270.3829332	16.31398042
0.07968184	21.94223095	8.716515022	319.1443496	14.22395382	14.19345001	141.0627614	10.40832219
0.165770008	83.26157273	18.47488631	432.7248674	11.96384671	25.44709101	132.8309396	15.18392107
0.054102023	24.46743906	4.383782658	143.3977193	11.7996828	13.58944077	111.5322774	6.894777514
0.040466174	19.30439715	3.479133704	131.7404623	13.54935175	12.01994285	142.6627639	4.065463736
0.056717642	38.05112365	7.337528697	245.4487983	13.19048458	14.80695667	208.5413828	8.380482069
0.080787831	21.13073291	5.631730555	199.0624433	15.03545065	12.67562294	166.4477303	6.391703705
0.205531695	36.97010254	7.687561543	249.4134595	12.03723939	16.26819809	118.7220839	10.39351295
0.12099366	156.327514	34.82334118	274.6648452	12.09804933	19.53299207	159.6195735	9.01151357
0.050826439	24.97997242	7.121867412	337.3938538	13.44892771	17.57357211	227.2198593	9.559720712
0.13121496	86.78879078	16.07816659	311.7222673	12.8622856	23.36844621	148.9452587	8.168257665
0.157147099	32.83053186	7.24192024	434.5206382	14.87567509	24.22757367	145.1454858	9.580134495
0.081268305	27.52099753	5.797343385	236.8785598	14.6381279	13.76508298	133.9552365	13.27737612
0.09709976	25.92585209	9.098683168	151.5089107	16.49676964	8.041919693	108.4547115	2.621993511
0.06439399	30.72627001	5.561938124	266.6868322	13.79350543	16.90287216	170.9327551	11.30207704
0.045221276	20.95560446	4.829993181	192.2630057	12.48702627	11.91289025	114.8915119	3.694151392
0.086229646	16.79657668	2.824845856	213.7039422	17.31500564	13.4771462	149.0062242	10.61805359
0.053085229	30.98711106	4.947402764	252.0085803	13.79377266	15.46226552	183.7110105	10.45533676
0.103938827	58.62093731	13.51978651	235.8137635	14.46032123	16.71713088	128.1259813	9.103856757
0.062042147	13.8300337	2.696826849	229.9346216	12.87599641	13.96828089	116.3086355	10.54989824
0.131301964	42.32168674	10.06972698	184.5361918	13.91245687	13.57939933	138.4949807	5.610085639
0.049174693	13.8376569	2.923121123	388.4607386	13.32910473	18.57820601	205.8600882	11.58471077
0.218836719	52.23464945	14.17408242	312.3120827	14.0974747	17.14808472	160.8489789	14.63086309
0.079580083	49.76428803	10.40145022	163.919302	14.17681491	13.65751892	120.1723952	6.31865844
0.305462905	90.54246423	18.96160762	455.0323954	14.09622543	29.45945988	319.9385647	21.62257917
0.071386321	23.15701211	7.436076072	173.8958193	13.32326093	10.73648358	105.4390323	6.629953725
0.064826609	31.63467693	6.039656505	278.1882856	14.29186011	19.87132731	265.225888	11.62857645
0.069177588	19.41928054	3.299107436	169.5497383	14.10454465	11.82305974	112.0746523	12.47685042
0.065737512	33.21087262	5.236767972	244.2306921	11.90881064	18.52551736	138.1738687	8.537246831
0.168999161	51.29214318	14.41986528	299.7847366	14.0816792	20.36925264	145.7240491	17.78008014
0.077845063	27.6947877	6.65603017	159.0826113	13.17801364	12.07015273	92.13103309	7.618223173
0.032900343	8.882918778	1.41167925	147.6064076	13.65185969	11.53287129	93.13699411	5.718145574
0.085800054	22.64528379	4.017050324	183.2581604	13.42597714	14.23850167	130.5553291	7.196269158
0.029360807	38.86026884	9.61807835	194.1690452	12.90981514	13.89563512	97.5448646	5.771465842
0.141998454	32.86616765	5.35807523	415.9183326	14.92288436	21.60648981	128.78146	12.44828818

0.062644997	26.33709624	4.015554003	161.770095	11.55626689	13.36108103	141.2122574	6.478413873
0.101216835	44.75128811	6.204381777	317.5788075	14.67667356	23.30851086	200.7501599	11.96751986
0.081125797	43.23121531	9.427186511	369.7664327	12.50968214	20.31003011	152.4269905	7.379898573
0.138535022	47.39471298	8.160030519	358.4869505	12.33714041	19.41395417	141.4782142	18.66666875
0.089519151	28.93214988	4.019406532	248.4583814	12.0358821	16.54956833	154.3411571	7.005747557
0.12459792	34.74467428	8.373020891	302.8409362	15.10560899	17.49876509	166.2888618	10.87922465
0.09463871	28.32534473	6.689000008	215.0177362	13.06189927	12.18527522	117.3339518	3.830827902
0.071808598	23.29795036	5.883457102	256.7495549	14.2278328	16.64980014	178.0985045	7.628117867
0.078843924	76.59613805	29.49503792	382.3063474	13.69905372	20.17839004	135.9555542	18.19210707
0.254180725	98.02345051	17.46065496	489.4307745	13.82128851	28.5884578	194.1637858	59.89133871
0.105871386	40.86075336	9.649071873	242.3737671	12.02538825	15.66325998	98.12591537	4.715254524
0.073750121	36.97390921	9.280955377	422.127027	13.81794566	19.71129956	152.0799814	11.17119793
0.133561854	45.02385949	22.42405682	149.3431446	24.22398169	9.785689593	139.7314025	2.388968573
0.239464601	90.04484646	24.71638176	436.1176228	15.32597884	25.95330514	218.5542355	22.11180583
0.362131029	43.12386378	12.5839666	278.4993498	15.89444058	14.50967918	123.856241	7.7405525
0.024670661	18.11717855	3.31792042	128.6739282	11.15394095	10.99452025	103.7659731	4.003767992
0.09501138	30.62833897	5.716927428	218.1751594	12.92474542	14.83349356	139.216232	7.500448743
0.19881354	24.35918129	3.929981868	287.2978641	14.27198803	15.35336043	160.734914	19.88844579
0.163197699	29.66774946	10.28103117	313.359056	16.38627705	13.05140902	145.0601657	15.32067979
0.149742886	42.78834235	10.81527255	197.4159256	9.79917262	21.5930806	213.870013	8.090839234
0.055423993	42.73801645	6.05403675	266.0526169	14.40112003	21.67285146	174.5743613	11.58902824
0.123877697	45.84681465	10.42775035	226.1364707	14.54376206	15.83353986	142.3052691	12.57697576
0.013772129	81.24822653	27.18259286	286.5585725	12.89159991	17.41685743	155.1187048	13.42152546
0.141658207	20.18808591	4.331747615	229.7299137	15.48097129	14.43937565	156.6674348	12.2404711
0.049052973	26.95425598	11.24895055	129.431233	11.06664555	9.61430496	93.59193346	4.1627513
0.148612953	83.46964883	15.94949457	354.3260794	8.570166916	24.15221698	216.4394414	18.0643806
0.162606183	56.20441916	11.68697726	284.9807919	13.7241009	17.03998313	150.5666345	8.612719926
0.085382754	41.28333734	5.394930667	371.4460473	14.27680511	23.6259684	176.7970773	9.942349251
0.084923335	32.76412835	5.896306251	208.4472806	13.5694719	14.72187872	118.4598579	6.036978621
0.044861451	19.07198811	3.702461307	199.2660016	13.0925238	12.24282749	129.9025253	9.096038041
0.054582027	115.0195951	32.19751042	503.2306449	13.22477574	27.26770009	257.3934832	26.66383054
0.137953966	48.35923445	10.56252619	332.4190898	15.09436732	20.85069085	167.2472094	27.00785854
0.183842408	49.97124925	10.49954352	377.4932646	14.08093261	27.34360959	237.4828626	14.42554464
0.071526162	12.14032664	2.009179032	155.9956307	15.21832557	11.61531153	131.4149885	8.189281115
0.072570166	8.398766477	3.155486419	206.6673254	18.57816959	7.728300174	102.2603627	10.6224459
0.066449429	32.66295008	5.334061016	164.9181283	11.20844785	12.54278159	125.4369838	5.724791669

0.137962815	74.00572739	18.45748682	262.99102	14.12285822	16.82752576	144.4443543	14.34842958
0.091409583	20.19482444	3.048823438	191.1087298	11.73284209	15.49300664	118.9557197	9.33232938
0.088206933	44.72523533	8.35354724	357.5681769	12.5491246	19.82306178	194.265967	5.869432656
0.069664448	42.93271344	6.38881747	404.1938335	11.9170929	21.13944013	133.1923864	16.92119465
0.081300392	28.96131173	10.34293543	160.3297972	15.01499205	11.83550624	129.0123555	3.48642653
0.057080478	16.29025474	1.928449999	215.0747144	13.49553978	14.34732085	109.2566738	11.09136007
0.063785858	34.4809236	5.658315852	325.5338579	14.20725032	22.6880892	245.1836261	16.16029069
0.101507442	29.29348885	4.96783147	225.0314533	10.63042407	18.38459476	135.4242986	9.983482964
0.201021344	100.9677159	29.78777782	278.3842028	15.13873879	18.19005745	142.6354349	11.62175922
0.068858458	13.5955391	1.862165987	135.4337615	10.96574681	10.6576183	101.7287849	6.634241719
0.096441476	72.71339793	19.11768343	293.8282811	14.22715579	20.08804361	103.5900857	20.55896227
0.065262548	15.26766957	2.532349343	149.4937494	14.28081553	8.821324477	92.52129105	6.481686138
0.082655856	35.84937393	7.978043976	290.4821093	11.14906786	16.76451077	119.1737959	9.742291149
0.174886718	61.66135177	6.455516905	377.0650389	12.80524299	23.07103005	173.0852065	11.4315217
0.043258507	24.19418258	8.020043692	153.5458746	17.1706355	11.6014582	104.8304937	7.231853495
0.067214097	41.47248224	20.65089688	179.7686525	15.63510736	11.16282874	113.0614285	9.058267788
0.067054629	26.20873472	4.335894827	170.4246105	13.44390394	14.01863334	126.883844	4.147464211
0.077424402	24.33733411	5.773910073	195.5022245	15.22586465	15.37540307	151.1623427	8.830895811
0.225669763	81.23444601	18.03290163	492.1980866	14.80142986	37.55255032	242.8811864	18.75874474
0.118953864	66.96029851	13.15877339	227.9612795	13.00304885	16.21260343	123.8170882	9.193650142
0.101537965	50.84498156	18.49740807	182.5147149	17.15485711	12.4580218	135.9808745	3.882085414
0.104327293	18.88061493	4.059632192	229.1874423	13.05836535	12.13569478	121.3450913	5.684043434
0.041512326	38.95437992	9.364020454	245.1929985	12.64787531	17.81643476	198.5881306	4.251945494
0.109758724	28.08256451	7.85400377	393.4947825	12.69192975	16.44894985	103.293842	13.85908684
0.182748389	42.43015443	7.630526701	337.6993979	14.36281357	21.40366573	174.304513	10.47216215
0.054735343	4.031929487	0.394909989	135.6132866	15.02087699	12.47769766	118.926346	7.475852665
0.107441536	31.30650985	6.709393671	243.1648509	12.69759242	15.2476525	131.5176043	6.567002101
0.103663773	30.61527926	5.370270632	315.9469296	10.36832357	19.2614233	158.0958948	16.36429605
0.104222545	23.26572127	5.310358799	153.9518441	18.30852566	9.640848968	133.9141306	8.659953496
0.10387673	27.56602944	4.422534573	191.1016366	12.79456256	12.25719406	111.4456956	6.184432552
0.139798818	27.05861153	5.301522481	265.5438429	11.57155108	18.36461733	144.4863748	11.22455068
0.105989782	63.70310638	11.70409008	505.566855	13.01382227	26.78642207	233.8347964	15.14983929
0.129577651	26.33784411	7.433083555	286.2326707	13.57279345	16.0908316	124.8479781	12.38151279
0.068429437	65.2590317	18.13509398	302.5941279	13.31110993	15.31385159	139.7382446	7.488353492
0.081134807	20.75312839	5.569119326	190.6728115	11.78884833	12.57429702	129.0607028	9.890524364
0.06144906	151.9667307	66.59308435	278.4927258	12.96431329	18.70084245	190.6956989	5.562854256

0.054974731	18.58520133	4.756944021	164.5813468	13.20993746	11.04224527	115.6848131	4.403525458
0.19027641	76.21006787	13.94890799	484.6954033	14.55385439	21.92479002	146.2517846	19.12901429
0.114278928	26.58669973	4.389404996	215.4262917	12.36468179	11.96114777	93.81908999	10.39113196
0.054586827	33.40832259	6.671982661	247.1371269	11.0839829	14.9714026	109.8835646	10.17155555
0.085883287	62.74653019	16.29932291	208.7783651	14.85714954	17.51878256	113.2279728	5.342591088
0.087468338	206.6102523	81.89472445	272.3163286	14.94307832	19.25034083	134.7989307	5.56012021
0.096088057	39.83086801	7.998253541	376.4763971	12.48826145	25.4823781	162.4412277	21.19924309
0.122737898	69.68460619	11.71809726	534.1857286	12.62351843	24.88181048	220.0510232	16.47382327
0.161623857	46.87406944	11.31773098	370.4421379	11.96357123	19.07748432	146.3619368	14.62499265
0.126209815	16.94261236	3.993428263	181.4625373	15.11467942	11.42020579	113.2777707	4.900713098
0.130952153	32.00694303	7.65235834	257.743839	16.97806881	17.18189033	183.5843104	24.56651194
0.172781984	15.20427288	6.624805846	217.306419	16.64720855	13.62532816	132.0149628	3.18490856
0.114802131	40.41773442	10.94844261	297.4806563	13.40905692	22.86351096	174.4486868	8.528826823
0.085316064	28.63026046	4.778940377	334.2632031	13.17184941	16.24488988	174.6212669	12.47315964
0.094057781	31.02967688	13.20663627	226.7269334	16.39222215	14.61862568	156.3301631	5.44527196
0.136780203	37.6671967	4.975366365	313.2615757	12.41626765	18.08860375	144.9339992	7.89305111
0.067756953	104.4115595	18.02364044	328.1056756	13.20627643	24.54222727	211.7581893	8.312967843
0.072903432	14.68169314	2.759560226	183.4992976	11.91456798	13.35083856	106.9103864	8.632353161
0.039404812	15.51803946	2.341222866	120.8535781	12.3413761	10.14342454	105.1999839	4.821044698
0.009189927	12.61621683	3.681007596	71.65058454	14.40786158	5.413403705	85.73011345	2.81093227
0.099377721	19.72416814	6.175161823	289.6667619	14.09964372	17.82131247	145.7554687	15.63945588
0.117790132	100.1196609	24.78252503	303.6270178	13.70094457	23.62137948	233.6621637	22.99581899
0.144520072	83.73545634	22.31620776	181.5208947	16.39585467	12.99371267	184.711124	3.963921798
0.10105876	48.72027115	8.863364657	368.0630907	13.32567344	20.73432134	229.621554	9.43424007
0.124347576	18.24933398	2.196913827	279.6914368	12.8752466	23.19127476	233.7265274	9.498376893
0.018001765	18.70399957	3.563976987	151.5515019	11.74271641	9.187009341	89.81793453	5.80489749
0.228462058	47.68900519	8.605623653	368.1249946	14.79150902	24.99093473	163.7851736	13.78059446
0.081585886	17.75908661	3.007181686	225.8990316	13.93987684	14.29480789	103.6350262	9.937782919
0.052958381	15.34590194	2.542183429	192.8748168	13.7400555	14.89018097	122.3275179	11.26259227
0.046344591	16.54655697	2.134302368	173.5321777	10.72235066	12.89239663	89.18801562	5.638998635
0.187652442	48.95391254	13.63266068	298.6110526	12.42171939	22.87705309	218.3347316	5.7709576
0.242898158	56.44239861	12.69051831	355.197181	13.72260509	19.34942417	168.2375053	12.80322454
0.079836058	26.05378834	4.388459395	268.7269308	13.33661337	16.71855268	148.0220091	9.671584032
0.194503719	139.891157	44.7922887	290.609484	13.16541341	21.13204022	154.3200034	8.040056842
0.119272571	49.36510451	9.809019808	242.7959393	13.67281902	20.00179635	199.0515392	6.20653666
0.037987411	13.9162146	3.102225712	167.8730674	15.86962821	11.55183978	120.8990908	14.44351569

0.083748834	50.20025695	11.93750549	269.3150539	13.87418749	20.3076951	212.9256705	9.380477535
0.062986581	30.06022024	5.917380946	142.3628525	12.42470832	13.56329731	100.9135478	7.482559461
0.094417795	36.68363817	4.414101444	309.6487466	12.31694996	23.38392106	181.0479559	14.73531777
0.154150954	45.18313025	12.94946886	303.0936492	16.50770648	19.02115347	205.2521384	10.85900808
0.092647831	41.26237163	6.862494888	347.6221007	13.52579025	21.5200033	218.5738809	13.58778208
0.103935971	75.18021013	16.09576151	550.3324299	11.89224431	22.20829639	160.6398091	11.02313205
0.170688281	71.24007639	20.44157288	288.2546354	12.59915527	16.34677393	146.7646627	10.54279991
0.173736216	32.92644177	7.146449376	357.0757797	9.286895289	17.59406666	184.6810807	9.488379358
0.107207178	24.81472787	5.628174922	292.3941217	12.8695896	16.77046269	131.1343314	13.0652656
0.48877433	71.33313683	17.40217974	458.5209069	9.120612064	25.01642778	248.8251944	17.33525176
0.125874269	22.82210559	4.437044331	282.0057055	11.89196968	14.44899414	130.56114	10.70739746
0.134376989	27.19822366	7.667462441	384.6080088	14.01634078	19.89442617	206.9508831	15.25578776
0.047856401	24.65250804	3.447886437	292.9075098	14.80327484	19.49431085	201.0299703	8.400111225
0.230133812	135.177163	35.89719804	385.6285036	12.97678156	24.8654006	253.6759114	10.65984546
0.09299855	22.15825228	5.390155217	209.3281962	15.72726264	16.94763646	206.5208333	8.335527895
0.151440924	75.16944395	15.63499196	353.5691824	14.02154441	17.38158288	95.4152701	10.82348907
0.081412889	27.17643338	4.640389722	303.3188821	12.04319851	17.11177071	127.9910933	9.2104173
0.081833576	26.66534155	5.434498354	199.376375	13.28850644	14.29321324	129.5271844	6.405604618
0.092637267	47.31819834	7.95168283	256.0099212	12.01773214	18.98670861	169.7888901	16.97153124
0.290858715	66.33117993	19.14103282	322.6578907	16.47827441	14.39346386	213.2472464	4.824526288
0.063958895	16.56106327	3.513119737	176.8177276	13.4535574	11.25001742	113.6114784	5.559904803
0.079134815	31.77483772	5.541799411	213.3931554	12.83771453	15.44081106	159.9829888	6.895581152
0.064535901	13.75922596	3.457534875	306.1923054	15.57936127	15.08476574	162.2206103	6.45283584
0.041302515	24.78696211	4.333765078	249.7146093	11.37012148	16.96314649	166.3054726	7.790775589
0.11791931	54.02719427	12.85308707	176.017131	13.77676264	13.31304	122.8365199	8.443941003
0.111443369	42.95246208	7.25642555	323.8772558	11.7971133	19.75462266	139.2509454	10.78960264
0.043751575	14.46327687	2.597137566	133.1668686	11.76911129	9.307703534	89.42064074	5.428404304
0.052984535	19.12759925	3.816839032	149.4339343	11.67653493	10.25584011	94.80914688	5.044547035
0.058343915	19.3544414	3.839704215	174.6884221	17.67175371	13.03644097	192.3573787	12.92783045
0.093825569	29.46038595	4.769547152	303.0654417	12.9592245	18.55115918	160.9347327	8.830516282
0.089360299	41.33136503	10.48079856	377.6360919	13.90448362	18.68168711	143.0291573	11.9660799
0.04194072	14.10266847	1.971696679	188.1326261	13.01430733	9.836723436	97.00170924	7.173891367
0.051689506	30.71809764	5.934300083	248.4045074	13.14152979	14.66349628	130.5506986	12.97027659
0.159317175	65.35132004	18.19430785	538.3604835	14.54641618	25.63899982	247.1926419	12.16058634
0.076270069	33.44546908	6.30664745	235.0975071	12.15995949	13.94320713	120.3526342	5.610229434
0.128009359	79.40387446	20.71241824	327.1288466	13.7212383	19.67449158	240.1899801	17.66293153

0.070505917	13.16145548	2.248843194	258.2559205	11.76451245	14.17789185	164.3829776	9.309991636
0.094764124	19.87887636	5.88499761	233.1101275	14.66663465	14.09296704	162.1164188	6.964031692
0.104102266	32.39064629	5.330792163	229.0912091	10.73097424	15.95525112	153.92245	6.37504061
0.110560347	36.93956201	15.50009435	162.7831354	11.95684605	12.63380711	109.1715956	5.034915174
0.034626056	27.75188103	4.768392656	297.1517839	11.86806529	15.91897289	171.2363879	9.003855253
0.178329081	55.76401589	7.72600987	626.4024667	12.5645406	28.55668692	210.5356595	16.43870025
0.166341104	50.25250493	22.45959515	256.8829992	13.78613495	14.86759837	108.4334819	3.206639782
0.074004624	22.7250403	4.39133437	214.6248553	15.97777158	16.31456522	126.3322536	6.583705444
0.373982381	76.67851177	28.37958455	462.5306925	16.62367374	17.98203489	178.3762484	11.21765285
0.070614098	14.41210234	3.989352614	176.0776627	12.26695896	11.32968057	121.0441453	6.740650991
0.097993832	34.49186204	10.4297703	297.4375893	14.90257995	16.88201696	149.0823595	12.38835698
0.378568736	86.95115219	28.37864062	365.4182418	13.78571463	21.810924	181.9004709	9.899472605
0.257641968	54.77412136	14.73109634	338.1032838	14.77197501	21.60700106	172.1846502	6.364853887
0.078884214	21.96382273	7.398957655	222.4621589	12.56684823	11.33141012	113.3890055	12.97261448
0.062838083	29.6497096	10.61944547	122.2851	9.519258891	9.716033072	104.3221187	2.079459627
0.110334478	10.04191518	2.321236185	213.8985902	12.73484625	8.958380052	155.4828047	12.88488642
0.083952962	30.81561441	6.582709258	210.5272588	13.39959771	14.42562102	175.0911381	5.228346187
0.240720361	33.56777625	13.26076749	247.2010203	15.68565779	14.78488476	125.1558458	4.738162078
0.049328779	20.47634857	4.868938774	322.4867821	12.57273781	15.22477898	158.3809789	11.4520531
0.232107265	71.55057315	28.52032964	518.0158441	13.77672051	20.56767678	164.0655353	14.75891536
0.0532918	21.87941144	6.521418362	125.5310274	15.30839805	10.65206911	107.6138991	3.094603506
0.05054585	18.94346358	5.074893616	92.94905331	12.87626921	9.669046777	119.491791	1.856713639
0.190101695	33.38182167	8.375927278	260.5627002	14.93172144	15.63063515	132.3670488	4.271516587
0.059213583	21.53153093	4.981595941	176.6208455	13.11370069	12.5099138	125.2640766	10.63699279
0.121586471	59.68320427	15.02057612	266.8779396	15.70148959	15.57386364	149.5762454	6.630422797
0.054128035	27.74791409	9.109630758	217.0189652	14.64718811	15.07597387	188.0128763	12.7236856
0.403510575	171.4750128	114.3278083	492.3082651	14.83618196	22.45365646	184.5715276	11.20169459
0.141072184	34.86835518	11.93400966	250.1898732	13.18534341	11.01018932	121.5269399	6.554309214
0.106429601	26.41167594	6.024006247	312.1204496	10.87163871	13.90163977	103.7199126	10.81638051
0.22352462	111.9956629	55.70262182	130.9338172	16.38845703	14.20661911	155.8474763	2.644802362
0.164929997	38.35202657	9.135998144	296.0522382	14.24301346	15.97290072	192.7321306	7.8568636
0.160007439	59.68526166	30.87927227	216.5691563	16.78371338	16.15104309	149.2559311	6.449106009
0.109029144	16.56589118	2.958238205	161.1082602	12.19449963	9.67368022	107.6913072	5.687680588
0.043874661	32.13440808	3.876483359	295.1690934	13.46398391	12.99660926	110.1908101	6.659455845
0.132500607	154.4270671	103.2783677	315.532204	18.0272071	20.60551076	118.0542932	6.677039073
0.102041714	17.77095319	3.962268608	245.8201821	12.00538946	15.1219918	143.2187487	7.329020411

0.090427469	26.28912059	8.064732831	167.0718458	12.52717175	10.74374099	120.491526	8.858919351
0.037267731	12.6900246	2.674023126	124.3653928	11.6204793	10.34394624	82.34899558	3.177482551
0.127379522	24.75698197	7.210038731	185.2599309	15.77932249	14.42099031	152.7947522	5.167048318
0.112486109	17.29456868	7.937156408	193.4829747	14.22156155	11.86627009	124.6593851	5.665288648
0.138688198	12.52515822	1.785391469	205.3212271	12.75068323	12.4015542	133.0472425	13.48206802
0.335154261	62.3715892	16.84484687	413.4119662	15.73292531	23.3427548	173.9176527	17.75756417
0.105361891	18.65775382	5.377518088	261.4798267	13.66921504	15.31827871	177.8142406	6.346132315
0.121097332	36.85599882	11.74406464	332.183577	11.98594652	16.7178735	150.0323956	13.14376084
0.057114136	18.76455762	4.311156727	187.5395732	16.14682003	11.78952812	124.6381564	4.582971505
0.213821256	45.27365384	10.51038787	344.4848384	14.84349896	19.25764983	176.7980587	13.88707286
0.094660211	31.15289436	8.64567811	302.9371946	13.00582394	10.22671145	107.0424659	16.4518036
0.100237334	14.27148617	2.912646546	197.5765813	12.84929594	7.606008072	101.0535954	7.535104832
0.159045172	39.16343404	28.88874298	228.2320499	15.61750012	11.04834596	115.9496809	22.54305804
0.107548802	34.39224724	11.26309343	276.7464019	17.23745825	16.04000164	127.079767	5.191118957
0.184055786	42.65672375	11.77757814	358.2645981	15.71234383	16.03865379	144.4314133	13.05194743
0.054275833	24.29645308	5.894093381	250.8955894	10.45619668	12.40293515	133.3396278	9.601927716
0.078764835	25.51359438	7.178888446	298.0813254	12.79138787	14.60290232	120.1759879	15.64155249
0.23290704	62.27355088	18.29996109	416.6384341	12.66317897	20.6917087	142.3097862	6.554127543
0.099503285	30.17144236	9.029174183	228.2101011	15.32655632	14.25836492	119.2736537	5.893520686
0.228495795	26.37284638	15.8684999	113.2676084	18.35111316	6.820884651	91.97039737	2.675438959
0.054630215	20.06680442	6.269235218	200.7276244	13.01177472	10.98286171	143.51354	8.757899983
0.050367159	15.44300534	2.899221089	185.1952729	12.49827524	12.24862197	115.2297172	4.538652638
0.083974989	24.15636152	9.652458109	262.8765381	12.55348258	15.76518148	141.2286719	5.573260525
0.137634116	16.73836792	5.597180977	142.1979359	16.38959529	13.51745154	115.5603041	6.663409134
0.18030894	29.55181334	10.28062931	230.4393447	12.81109031	12.9114582	109.0539692	5.360508315
0.096052562	34.86906777	9.75999847	213.8424826	15.07508096	16.59198628	193.5351325	3.990551487
0.331282872	58.61007235	19.5363408	423.2264326	14.00085834	22.76093279	202.5967295	19.7917765
0.043143542	11.15085079	1.890207192	160.0261825	11.99261289	11.20025712	113.6479133	8.653545376
0.030817166	10.03580109	1.641802001	154.2468775	11.36119873	10.3706636	97.94397462	4.046410959
0.126357944	22.90619129	8.257930264	236.8245596	16.63290316	10.638202	132.5802375	10.16711231
0.165693307	31.48327311	8.359390665	329.2057312	14.75941962	20.27394734	183.376957	6.822296735
0.065296775	22.34077706	6.649701325	142.5647753	14.08648975	9.256748802	65.45293153	12.17533072
0.033941879	13.47237945	2.65777153	120.7083218	12.35394745	8.541025464	92.12944686	3.912744453
0.986348744	134.2167218	50.88756095	396.1280113	16.01050486	21.03479058	223.3197372	13.32797534
0.042647926	22.9495962	5.770382071	208.9364502	13.33709524	13.11930774	132.3573926	6.620390603
0.064171046	21.60586997	5.149989464	168.3911782	12.666017	10.76619065	93.65322515	5.331895703

0.06476955	40.67946486	10.03418117	247.8621855	14.50254796	17.61888327	154.8514066	11.9442436
0.029373668	69.77118105	53.6344492	161.5011716	13.09043662	8.852463721	91.39564431	17.19529619
0.162731614	21.90640622	5.871592337	345.4439156	12.60400301	15.96967576	124.9174684	11.51802264
0.105705578	30.70925452	9.354723045	272.5989114	12.19287282	14.77524292	132.1411509	7.164167514
0.090830847	21.3314198	4.774759669	235.516408	14.93075804	16.65803703	158.2454285	5.788688941
0.176272525	34.60931602	8.582805778	239.6345503	13.74351191	17.9354376	176.3393221	11.56372018
0.077011461	104.3926486	46.81980718	146.7090996	11.34760052	11.96761451	94.03759467	4.482885151
0.080496914	29.6740875	6.768881574	124.1154622	14.79556999	10.87549103	134.709048	3.827956878
0.098641488	16.8546347	3.124722293	301.0944315	12.67054936	16.91080718	171.9393705	10.63334563
0.227531615	50.47774742	11.95631092	321.0019001	13.98682258	18.26874763	140.0906088	9.192594334
0.094455928	40.4519966	11.99771987	267.1353461	15.14519936	18.38278555	159.8370361	6.499421787
0.058646963	11.39160994	2.857437715	240.8970407	13.86487172	12.59874647	168.7086815	16.25174669
0.126262326	68.45376229	63.72426595	171.9552217	12.94737291	9.793427898	83.08768899	6.062786908
0.059141137	20.04979651	4.139297281	232.9345798	12.06186394	13.70340711	164.985897	12.33868426
0.052563266	19.04649997	4.892413103	115.6427653	17.77843524	9.667047928	88.88750883	5.393020844
0.060688355	12.27340059	2.055646869	150.781084	12.14855473	10.9763479	130.8432858	4.747399455
0.113400785	38.80573683	9.655830002	156.8463422	14.47079889	12.94299261	130.8632124	2.387634623
0.150307722	75.0410898	36.27734634	215.5371111	11.00818891	8.809489004	108.7051947	6.053181198
0.22131778	50.92704505	11.85058835	239.3905654	11.27285761	15.91581726	145.0243116	9.481331834
0.070877063	28.18011265	6.503373742	200.9736709	11.4427149	12.48955241	103.2039018	4.893233845
0.114847852	13.43967218	3.13108116	350.090975	13.33278502	17.92289182	199.3042855	17.37861429
0.104231692	40.39678266	8.209716968	260.9060597	14.26791657	17.48606646	119.792694	6.506140818
0.107888473	21.3816944	3.385727059	310.014307	12.08537618	15.31916763	180.0209542	13.67539647
0.09407868	21.87163981	7.653616658	251.4570332	11.83725148	13.80731901	135.622571	7.519521271
0.077647413	44.14983703	11.0715242	308.9510259	12.83512951	15.96184813	136.3246644	9.309255183
0.081906395	28.73019921	4.708085418	226.7710007	13.80575248	17.73781575	179.6710952	7.602768518
0.247979098	29.04457938	6.710629509	324.8523185	13.00941953	14.94775984	166.2367811	9.349639967
0.356352662	76.49629404	17.13233133	582.2590357	12.53696403	26.59905285	141.2962164	14.42655676
0.116539888	26.90567011	6.567803158	282.6237203	12.35009897	16.13741667	160.5588976	15.02371432
0.149095551	37.95523878	8.725841431	322.5236842	12.24677172	19.07341076	178.6768974	11.60632196
0.075391096	14.99916472	2.953098332	248.5426293	11.36643547	10.76549889	161.2609137	4.834255495
0.063765316	22.18893345	5.100750635	278.2793012	12.05915158	12.64796431	143.7613361	16.39364529
0.063844353	28.69146379	9.943021188	128.3853004	17.67944538	11.21200065	134.88394	6.266808732
0.094632724	32.47946932	7.491760472	320.9110569	12.61080016	18.17132144	151.0654905	11.62710804

OSI-027_1594	LGK974_1598	VE-822_1613	WZ4003_1614	CZC24832_1615	AZD5582_1617	GSK2606414_1618	PFI3_1620	PCI-34051_1621
109.9559493	100.0063663	32.38519192	51.40207213	266.4572863	20.28848695	48.03477157	280.74955	111.2251199
163.6426885	71.86091602	53.71453087	28.20091719	123.5036927	13.8475861	33.44529199	157.55288	61.01780101
181.2075058	89.30392959	45.96013915	53.73304857	198.2152804	43.02807887	92.29831522	235.55654	159.3350837
203.7931215	135.5926144	18.1386773	40.05925183	122.6043489	5.287256371	52.58864289	155.23409	48.04929076
170.9737134	134.9499794	49.09871884	34.04080451	240.3972338	10.36052956	103.6902216	303.51023	216.7313928
183.0698888	56.07711332	28.89068604	29.3587517	110.8745567	14.223841	18.01380678	127.07392	38.41091923
149.9125084	40.28803759	18.14554536	32.48041628	157.0894653	14.09942558	25.57225431	186.33408	79.63208643
115.6600633	66.24941199	128.7721473	49.49364647	179.0568555	14.35119319	50.42660774	226.03233	97.6509591
105.9038867	111.7665488	101.4232625	63.56400279	235.7302601	25.06925232	88.129135	307.07448	184.2719238
108.5215367	31.99830521	23.61963819	25.01147777	85.33651031	3.407995081	17.52098177	109.39673	42.26864884
117.5875323	31.24173753	21.86636768	36.63140458	122.3608881	8.878406611	26.71131705	145.66328	62.59840281
177.2415615	53.97885368	19.91336623	43.9332159	175.4913908	8.538576683	55.12794691	210.48568	127.0806821
101.346078	46.51583554	14.63608449	40.07574128	150.8008335	4.110436829	48.10530589	198.38597	122.9616357
331.1637889	90.17903103	52.42030176	76.76218729	190.7637825	12.31390907	67.18618702	205.71866	61.4150839
99.79123658	70.03554346	23.85307553	37.08388906	136.0665199	6.61113371	54.68406251	189.61235	104.2179434
195.1506162	91.07807015	52.14746303	42.9129115	166.5852493	23.75983703	39.72897918	177.30852	99.87467604
64.08166561	30.05032052	12.58254084	29.3244791	123.7859176	7.469575081	31.75249027	141.13232	76.84718334
213.760375	51.06551359	29.54001077	119.9649636	306.6059093	18.05158739	61.57303184	321.39332	165.6997178
195.709036	81.68258165	21.21218178	34.89153	137.1305843	21.55248272	61.54067735	190.22806	167.1476432
162.8274138	46.98839251	13.59252117	32.24155898	88.91235259	7.829454794	19.90238274	119.82027	48.61495301
171.3897945	60.81184588	40.78908487	37.05733263	148.1807124	11.59846416	42.65200009	192.84081	75.83255802
190.4431655	57.7078444	84.4306869	109.7447581	287.1456084	70.54180022	97.30167408	301.14498	192.1795115
130.3427581	53.51804895	24.06926403	36.27285489	175.7104453	7.926635059	25.69982772	216.3842	84.71457132
68.9198476	107.3875736	37.49222549	37.8381328	185.958629	8.73652669	65.17314673	270.95479	173.0821961
138.3263068	57.4851926	35.34252108	27.41971854	143.936737	8.64360158	37.88106611	187.59913	68.32320679
102.3790938	64.56389174	59.2326654	32.21505177	149.6883807	9.08834835	35.26184817	198.62341	87.7076945
214.6842593	105.1475678	48.2762247	80.71883389	201.2058069	20.08153692	80.71539727	229.02205	143.8889121
108.6239363	126.8882473	52.86954901	32.63604312	193.5734813	13.76385281	46.52077636	249.31983	131.1797856
152.174208	83.71156127	24.79611545	32.62849766	125.8227586	3.642630844	33.15638265	168.20028	78.62817605
26.56452492	38.22326257	23.75186903	54.05451906	132.7092638	1.226567968	24.02367432	154.40644	109.3786658
153.1272386	41.02527528	37.07057816	52.66183929	158.2914225	24.90585192	24.09613601	180.9187	87.31944649
108.9932152	83.18410687	28.51969561	52.22162966	149.1797305	5.621117403	51.6343469	210.88675	157.4994311
130.557789	127.6572294	71.52554345	52.97600255	255.6181921	15.21596747	80.64265143	292.67438	218.7942432
78.64025197	34.55778364	31.91693254	72.20131802	119.596673	9.179149511	33.94682999	149.11443	73.09234132

111.936091	69.69099764	62.40406325	51.95532375	204.6268578	3.832163298	62.29669977	240.20034	119.4239708
126.4805656	92.60138182	64.88577075	72.31400773	216.9161581	17.19247011	48.42140095	257.03094	161.1410551
71.29188214	49.21557486	10.22505087	22.50591889	89.50651073	1.615991047	27.56769661	123.36425	78.47089724
150.8011283	137.3638625	58.52989966	71.22359789	259.0319591	56.51417502	72.23407452	279.67774	244.6658626
96.9884735	57.97553036	26.58105029	38.95696209	164.6448999	13.53296905	59.53857602	226.85203	120.8111612
91.79005675	73.28035899	22.12849448	59.66005042	167.2650189	10.95268721	62.54386753	193.30516	94.61077901
63.39437511	80.93743999	16.91760805	97.06815656	158.1481344	3.091252515	76.9760696	214.05105	159.3466962
164.6938421	80.78444554	43.526191	20.94054835	113.5658955	9.133140979	36.68896601	151.68919	85.08379111
97.41636974	84.16976765	34.78821646	29.79974205	227.3940531	15.40813042	48.93719376	266.67658	96.8653576
66.53440828	62.28793666	29.95040609	39.40931677	145.3333685	4.020242864	51.29886662	173.33964	80.71094218
88.84449	48.94899767	26.66867654	58.07905172	213.3069482	8.726618122	47.58679915	247.23842	112.7772547
59.90194286	26.03708827	13.41486545	34.83721074	103.612915	2.36899164	21.76823734	101.01086	23.25101653
137.5263193	50.69504326	18.8783566	28.09182061	114.1328606	12.10907737	38.27648395	157.58561	66.93239745
90.2388757	85.68477633	29.59718338	56.01245981	161.7378079	6.319801464	44.18581818	212.40598	84.8788885
200.5988617	44.31680028	17.7836503	28.07771013	119.8447742	6.830635781	32.94890793	146.29953	42.78276055
65.24353327	53.63185844	27.50415558	31.71999231	126.3321011	1.697711644	33.08238968	159.59445	102.4591083
115.8892801	23.9711587	11.83378716	24.03506663	80.72420921	5.477876233	21.4069844	112.33785	30.62256845
186.7069026	81.60011865	27.23979492	31.22526832	140.0680242	7.560448609	41.87295435	179.22308	106.1645402
189.6101231	69.45834365	27.66022914	46.54149028	152.0198881	3.309579236	37.64954573	170.2332	136.3378765
194.4137181	40.91085878	23.37175855	56.01165403	119.8023026	4.893457932	27.31462696	141.75681	92.83178827
81.3938418	67.8589108	18.00719074	58.1433085	130.1902022	8.034877333	45.77903651	158.58811	47.60069748
68.16152275	130.1272211	28.89370318	36.99737751	153.3740739	3.49857812	69.21357504	214.92317	126.5678221
146.2211012	32.30350126	23.06924628	26.2400657	124.5165186	20.0315714	26.09513491	153.69867	88.77587182
122.8108813	51.45982483	13.06192092	28.79488094	96.94981938	9.244280535	34.89608067	129.86473	63.70082421
69.05716488	106.1453514	44.12897079	69.39867142	368.5514908	18.15606772	64.33839125	371.07539	148.6823549
88.22416362	92.11138837	29.86865068	35.27895162	213.6906257	21.79024551	64.65164367	238.5105	150.3978292
217.6852852	108.8334702	116.3149032	60.21034816	239.7825025	27.19485142	58.3382189	237.76397	132.1902669
149.0367987	61.58418317	40.35785134	47.43599673	167.8375063	10.05176056	36.4977111	177.48761	52.09694737
128.926987	136.0468913	109.6452304	67.66755619	227.6534505	11.46900128	90.16321881	248.15148	153.7138421
98.14505311	49.08640776	39.76364736	44.39445153	172.442073	4.092066126	48.67488963	168.20258	94.90266461
96.08081132	53.05884024	16.861404	25.64765873	105.7369108	5.895683275	33.6518586	144.8407	48.61829417
132.3385592	40.49085327	16.4627772	24.5722106	94.16682623	5.339640221	31.41481437	145.11222	64.12980113
71.63692451	66.97912839	20.05097894	61.23921249	195.8966597	14.74275293	45.70006947	211.00772	204.9562016
128.6940259	197.4245415	190.8681041	87.12405794	339.9922789	70.51074725	154.1699358	419.86313	227.7160678
169.0099692	74.48018798	31.59710236	32.06876995	121.0903514	4.601558638	45.93195832	199.48917	167.805275
70.48894371	27.4996551	14.7272279	42.0347293	104.0589233	5.232272633	30.19349559	160.61645	97.54021096

137.1930048	84.7609988	33.40975574	57.98839593	196.9896887	29.08734484	69.11693006	255.95503	218.7487159
35.96501288	37.94390062	20.43073112	33.54608959	93.04717971	1.437589597	25.58036528	129.13037	52.79043605
149.4710613	100.4741864	40.81144567	55.16840318	209.8684149	5.341346278	40.0199209	254.81406	154.3706982
341.7431069	253.8910987	91.44260417	104.5888721	348.7033348	123.649519	203.6933006	387.99696	272.9074757
77.55156912	48.62055618	23.65268221	26.435435	104.0850145	1.855290235	27.92619713	134.2505	64.17823362
103.4854351	55.26198568	16.65463006	18.01986959	88.28177686	5.474964106	25.66724842	126.03363	35.06698176
73.2415654	35.48926548	12.73230972	27.13686593	111.2141453	1.894743086	30.431403	147.21748	61.34616333
132.388489	77.42159928	43.16379314	40.74050845	193.2772342	10.57518273	49.58487854	244.59025	95.37469019
96.73724339	55.92586525	58.25228342	65.07096994	240.4347369	16.2085723	76.61235723	239.47688	165.1343803
114.1332216	32.36777515	44.97584673	34.60330324	130.0593781	7.469712405	33.24010877	166.41604	75.81431614
127.8221603	136.2045583	23.40233999	46.1267689	189.0892293	9.507597149	73.08568809	233.05768	118.4956834
236.5351197	97.59400278	93.24387069	44.47021227	201.6678905	29.14418914	76.21249913	254.42493	152.6812051
96.39541362	40.79529564	17.58151872	31.17973277	96.89925535	6.848603687	37.07862676	142.5608	54.49453964
93.17733118	52.53732451	16.22050613	22.195654	86.1806473	6.684890605	24.26425455	124.8818	38.70106221
142.3649408	83.08743681	47.70997043	61.56828769	233.2887987	37.01810252	88.87760549	265.94889	122.1701981
89.01728284	91.28743976	29.1581208	89.25774183	168.988107	8.726500277	88.21898901	227.58944	104.0117793
169.3067617	37.22006061	20.13419118	59.11097262	137.952218	18.2263106	55.41705456	159.41569	78.18862893
133.418171	39.29020591	35.87405009	37.81109911	121.3010042	6.585838509	33.1072786	166.69732	75.35704072
170.5038205	96.61579728	54.06237725	57.10112252	228.2701045	26.6967311	64.1972509	230.79589	171.9107761
92.70198412	54.00934983	20.36510143	25.02930725	116.620614	2.172666065	41.26833787	143.65158	103.0622754
137.6733167	42.68014081	32.61104455	53.93764271	199.4139355	24.12451457	58.4041246	214.16834	133.8319635
75.28430404	35.35745016	15.76810735	39.97207683	122.090658	11.84257278	32.02112028	151.95648	76.68924365
98.8098725	60.86286105	26.36211847	51.34860006	209.5923575	14.53116752	57.18357615	259.79815	119.3870799
130.8065867	63.52039076	29.53201076	32.39183575	129.9141012	11.02462199	19.17243387	152.42812	65.55781012
86.64020713	48.14341129	30.45858062	34.02676108	137.6978205	7.256242086	29.65505738	174.59979	64.34736537
112.6585789	63.35542043	21.93650019	38.80108714	121.5721557	6.55015452	28.8688262	166.17707	57.99806505
104.5413353	44.74423933	23.69674763	47.3852024	132.0148371	8.521628003	49.43624291	195.51217	62.71472456
97.15258435	108.5458867	18.65648408	30.11697853	194.2348099	14.36636864	44.97309554	251.36702	96.39109506
114.2018091	80.78484948	35.5324935	31.53057857	113.7090213	9.432843873	56.55105809	156.364	78.36902631
245.8791856	76.4763133	21.44640963	56.05884166	122.8388536	10.07110873	49.40342686	130.64642	50.38578296
71.05219843	65.25715827	88.26328795	44.32983961	145.0447352	7.95947212	51.33469076	196.87039	146.3267638
153.8501466	91.58052838	34.81082544	33.18416662	148.8931193	12.31879057	52.8801108	205.11297	125.7304102
40.37890566	23.45312732	7.034082829	35.13284484	132.5172131	1.7535145	23.09739503	105.40804	35.9553986
72.49356057	47.71581785	38.9845236	38.8436155	135.8573884	7.869579956	29.83395855	169.14382	72.41304075
68.3057862	32.51797359	14.31939526	70.27066643	167.5213916	3.053764781	26.31239542	158.61932	119.600841
125.4158008	76.90990897	19.2580389	40.79780729	175.2231296	16.33333715	45.05825941	238.89642	159.1775451

163.2126828	56.94269929	46.61746816	32.34034587	166.5260239	16.34521566	53.41790581	218.27313	82.69089694
182.7774558	74.30985353	29.08015879	27.49510656	158.145326	8.018798606	54.60051744	199.6837	96.48488311
93.34229332	26.89028558	17.81343585	43.89161929	97.34635823	5.272823427	24.98672112	127.47614	78.09162254
202.7734176	98.35595051	81.54093529	77.96376603	286.0390986	9.312739502	68.28871636	305.46261	127.438754
136.0106496	68.98458944	19.69556622	35.63518658	169.6363764	13.41431493	30.08321303	209.39737	97.139892
178.1971488	56.9912309	18.56418343	31.97236251	130.4992197	6.552100581	28.70848146	166.01962	105.209455
257.1591187	178.3532925	71.35278961	65.16853704	251.9526813	25.80199333	61.52440816	263.3418	219.8865024
98.32407882	56.37728347	26.44099604	40.98477082	150.933471	9.722042438	38.43512281	180.44447	75.52730855
126.7412895	110.9012918	57.34944985	40.86176308	184.4871437	12.74656064	59.59750258	247.42557	93.23265633
159.8631406	44.69393179	27.78397526	35.86343646	127.6656796	5.511208891	46.83086668	155.80458	87.53296124
167.560561	64.4842316	16.11398049	50.86020083	150.16034	8.658150174	45.93849765	157.6737	43.80748709
102.6395723	77.96393628	51.65783437	62.68837743	161.6707312	16.3976479	59.34473219	205.21256	115.9732944
128.9814694	138.1580214	49.59076523	38.28578968	207.1090676	11.41470066	60.67874237	251.29403	150.6295327
115.3007731	40.9014551	25.36566267	25.1648329	94.42352817	4.922628942	29.29782586	129.80969	78.65790032
125.4501185	66.71854035	15.72355545	33.79711385	102.2740838	2.631400773	49.59245935	124.96599	35.6349606
242.4561121	44.79979545	23.30806107	57.84088645	195.4815038	10.21717721	37.30083218	198.36901	91.33909895
72.31902639	84.17762271	24.26523668	29.67885266	150.480873	2.802515668	54.99371056	203.42503	140.3521727
157.5533454	34.77894582	12.89747715	47.8463701	162.7113103	12.31980042	34.44591843	172.90978	151.2404064
128.7648487	61.22758011	21.37354267	51.27948696	141.5316242	4.021676884	28.04946634	138.34553	59.61741326
138.8695855	64.54085384	42.179331	35.23014051	193.2975381	33.66364132	50.41318467	262.14951	101.1324098
99.21396816	52.94775806	36.06100773	60.78412876	227.2418518	28.67228548	46.66127775	244.93648	157.4941792
107.0896343	46.78460824	26.97518659	40.2016661	128.6523257	7.617211745	28.6559318	179.0148	75.22176445
141.5849823	50.17389648	24.4752739	43.05586224	187.7794322	22.55207459	27.84445483	189.66916	66.38673436
46.01434192	78.67483892	42.80790068	52.08283283	198.7132776	3.005943361	35.72069132	204.74314	149.2791547
91.64004828	56.94929534	20.55844782	45.0173812	127.999534	10.05760187	44.744796	145.91394	54.15751403
133.4004106	84.93143296	40.35492678	36.49453685	141.4966404	4.820748296	26.16745709	171.99989	102.0371455
166.8830485	133.7159358	61.14218402	53.57831187	281.554916	19.55518348	97.50613288	337.78135	175.2883644
83.36182502	73.00584202	23.07980544	26.67438336	109.422798	5.825296827	32.70687982	155.60584	65.05416178
114.3921204	54.67180519	25.13297539	25.7524802	147.5515124	10.04406931	29.63998994	165.31129	67.85029482
236.8934447	72.56176554	24.41055996	57.25001918	187.6569138	26.06424414	53.13320463	159.9	88.3859019
168.6201005	100.6890139	50.0818614	44.78778445	190.1312622	20.45757628	93.43726178	228.92861	186.2366018
259.9511389	88.66522826	60.64888651	36.56896596	155.556456	42.36896974	50.28939771	206.8529	132.5094263
208.803616	45.05863718	20.22305727	37.83930624	144.549212	14.80829997	55.28424576	148.42048	51.69563282
289.592834	42.17412394	13.58353235	59.76377501	136.3587548	11.12093987	43.50860386	173.48399	97.16647445
158.9770431	59.97733491	51.05928428	32.19253247	157.5532266	12.11695638	36.36648719	188.88404	100.6448106
280.9042128	103.6491772	41.27881408	41.05588769	249.7613459	35.4814947	67.20793217	293.19582	131.9465741

203.3645629	89.70331486	39.47777594	46.10123774	243.5854211	24.51030897	44.65835661	301.07491	140.8750075
121.1290588	133.6797219	72.27395909	44.64901616	164.5611992	7.498244182	58.17985374	202.39398	74.26078214
102.0371779	84.6853463	40.23108875	63.34623703	190.6871735	5.0191206	53.32673469	233.10366	133.2696394
95.36114048	79.00400526	28.11856097	24.00759581	151.6903115	14.32603551	43.17069219	222.06277	103.0746124
190.0095182	120.863508	78.86721537	144.9513827	487.5388603	39.19229509	130.9104322	524.82092	459.4562454
30.79142179	37.19175733	37.08358939	71.44945642	130.8025627	3.337694161	44.87297008	175.15389	93.77802967
196.264056	47.68704662	55.48770403	42.6748543	186.6173688	12.83114677	51.46627882	222.43059	93.44250473
141.2819192	83.80790918	21.09770821	24.90732135	117.7570581	9.376496218	53.16472732	170.69977	74.9415803
156.297579	42.19458237	13.75129596	33.45276005	168.5414273	11.41667422	44.88793264	231.5125	105.7851508
106.4965835	35.57506114	36.62719348	45.22009474	111.1817219	10.81166727	33.57951099	165.31766	108.7915592
235.9151907	69.62842372	160.5839186	63.83292398	209.2084825	35.02149316	64.25964718	257.18767	209.7102922
120.0932727	69.48286746	29.48989313	42.9607223	206.476311	7.360838489	47.96734905	284.58229	110.4339052
190.4959429	49.10927588	42.04831929	29.61857136	167.2587296	11.23463672	33.56576092	205.86447	75.43252478
229.3038956	71.44179664	25.50420466	81.90892724	209.7823091	7.254865636	133.4752188	240.38623	68.88156297
186.7307591	35.29241667	11.87149641	32.0733483	109.7464967	9.83681219	22.37731729	134.36346	42.67351441
79.5770412	37.27146611	30.56567754	117.093254	241.0492206	10.78199929	65.38270895	249.86811	181.7997135
80.08080986	17.36929183	5.64662911	34.62251204	141.8309898	56.63886542	15.69275592	112.27252	77.016731
82.03272693	42.73114342	100.6522038	78.38426718	226.9745657	12.40021011	58.73798421	252.00656	189.055908
146.7372429	61.68658521	45.31824883	48.38022532	146.4901398	9.949519716	41.79958699	177.8638	92.12030228
102.6298543	33.4298809	25.83707551	34.89890209	131.2986578	17.57844993	25.58423634	170.77726	45.46299152
36.53410955	43.22974181	35.35752077	46.97662216	201.8780794	3.864486128	42.08733385	185.07332	101.5522137
137.5906508	48.71299997	39.80774219	41.83672042	147.9927954	9.12883505	25.33537289	186.57676	94.7777147
133.4580809	61.93132832	27.43387911	48.00893601	145.6162876	5.719276731	70.53043439	202.34606	96.96619417
92.89983215	59.41454415	19.89692586	30.42463634	145.4048611	6.27768202	36.17759318	177.61186	60.2815202
105.1330939	39.1299537	39.72711044	39.85572651	141.4008418	7.60012363	31.13760717	171.33846	89.86483299
103.3503953	65.08070057	87.24223193	64.49772825	195.1815089	7.251700977	49.57338419	217.61182	112.6662789
67.64574538	53.51097362	52.09462889	35.75867364	109.6931105	3.41435756	30.6676272	144.37019	82.24381737
139.5165048	68.8114258	27.93428144	31.61100596	129.5412568	5.541889072	28.63155942	191.20177	131.4491089
77.44882173	32.94206134	16.42600936	28.75796488	115.6214039	8.613061857	22.98277987	164.16678	84.2610026
81.9449505	45.148006	16.45158588	25.08985592	128.3479783	1.611304279	29.28068676	164.14192	77.50929652
135.4743692	37.50416617	21.37626812	37.39729482	108.1237823	4.552815304	37.05294867	152.93084	50.21823656
41.51300117	35.30334183	22.90725375	40.14985289	186.0283946	3.507005272	34.58246389	249.13265	106.8022598
194.8836396	57.70621031	20.89243475	29.61969265	115.2520444	13.86923574	43.20815924	161.21214	55.13447527
118.8813928	100.7551398	54.35553128	103.2992392	314.2019248	38.4355854	85.36151366	328.55278	221.966637
136.47912	98.73848109	15.07095573	27.41562112	123.680606	5.804098535	52.39999007	194.30852	117.6790002
111.3556795	44.92578598	47.55742043	25.81401114	116.1076099	5.587939678	26.6831922	159.9163	67.10143223

82.73761031	36.48540463	16.14946933	32.5467281	100.0201013	8.175002654	22.86242849	126.37147	47.2976055
111.0929383	86.28296972	37.54390099	75.10514338	201.7672244	5.988668514	77.06930755	225.96277	164.0342224
113.5421114	63.01008274	22.81409474	36.26261366	124.1341329	7.406852294	30.35762517	158.04163	55.45996133
100.8926561	89.22963301	25.26446468	45.61899644	180.7111103	18.1985511	57.15070056	217.10172	134.5126447
130.8259756	44.69867642	32.5880169	37.1209299	148.1649151	26.20140145	38.41708129	197.18797	86.21774314
119.938438	95.33342475	94.9900023	71.89550214	246.2623844	15.77771385	66.93552651	258.02879	124.5265803
105.9478189	87.80965996	73.76927815	56.9898605	200.0677022	20.51069308	77.80563871	263.28538	109.8859829
171.3525252	34.28012551	35.35673544	34.01148674	243.417556	25.54492472	42.55293032	208.46436	123.8993446
121.3726841	34.76522524	24.63590977	35.61475944	104.8028004	5.709222667	23.31640286	126.44766	69.93748563
126.0560716	61.43391186	40.60454618	32.3484531	153.8854393	8.202487547	41.13263653	214.51947	76.99156684
78.57010016	51.55197357	15.82331221	30.46338742	158.7599438	8.966984073	30.09459485	196.2487	60.80427288
171.7059999	59.87123989	42.78038009	40.93538389	119.0417102	13.33025732	30.18953516	156.91397	86.71451003
139.3697706	45.48340081	36.91661029	61.79081929	164.6534643	6.556879006	38.47614714	171.87897	81.32846823
167.2946438	76.542704	27.68875079	40.76067323	175.6170326	12.27768284	51.18974091	229.77346	107.9032079
71.11413616	28.18702171	15.364543	56.57608259	150.7854228	12.89430079	38.35921318	153.57459	78.94923809
89.13547801	67.72743858	17.42611021	25.68464051	100.2915612	3.13173509	32.61400556	134.93919	50.78135529
124.2056513	63.57659722	37.23950752	28.3721646	127.7410818	8.97632263	34.49656026	155.0334	75.28469929
190.9491538	50.55517013	66.1666714	41.15252202	172.5025132	8.588491232	44.33242771	227.27348	142.269245
86.97883895	47.52809714	19.58872803	23.06362549	98.69062548	6.061755085	27.23749942	131.10215	51.15459335
65.70758	33.70431089	15.02261847	25.80778297	125.2620067	3.360862309	17.58321447	130.48527	68.77787571
127.9111357	69.12815756	42.52570227	41.06718554	164.1797887	16.606778	42.02414936	206.74263	95.32026962
159.5158032	139.2465272	58.85170947	72.88459726	222.5109739	8.310800113	99.59340806	238.35261	163.14545
58.25468783	39.36291637	9.015344663	59.6875114	142.0701721	7.344280931	56.53267746	205.24121	104.5674108
70.39454694	72.63529648	16.61873809	35.21135032	121.4390698	2.595643754	54.07957376	146.36393	64.41329831
120.0749355	96.6944348	121.2447674	59.33118664	219.096378	39.74268923	75.03931707	267.61409	174.1712023
76.08027389	71.64734289	37.04280916	34.93296807	151.0194174	4.987908798	64.08463699	217.17322	131.528582
57.83230699	94.71400356	31.90444147	86.54995742	293.9584217	15.73544359	84.28370845	311.37324	122.6604555
52.88833944	23.18697176	10.19195148	36.03085779	100.6430828	1.739439305	21.13623416	110.81809	29.74371648
87.02863494	28.7800667	14.24651866	28.61963423	92.58742733	10.86557016	19.21110782	124.15465	32.04320385
76.32096527	52.6350482	28.8156654	35.54167635	138.1642803	15.0389075	30.53636677	168.99384	60.48304712
161.1181271	65.87426265	18.16313919	26.87073764	117.2963995	5.377646571	22.3763676	144.49415	67.4311273
160.5883697	62.03036729	24.06133965	31.19196006	173.4391157	25.47501069	30.00831191	185.71903	56.41250164
161.8665331	65.12246993	55.22616784	40.23260881	174.8772289	7.239166188	31.10248398	195.8724	97.48455243
133.8298072	33.65461145	26.7062802	33.62145186	99.21916882	15.52229	20.39148082	125.99461	45.5054936
98.10882887	66.81927551	59.20346298	47.28018719	227.4695128	12.51640959	46.62185359	269.89783	114.2766865
39.01756831	36.05476216	39.11019948	77.76015081	150.4959071	6.719424336	63.78096725	178.65206	181.868358

111.9518827	110.8394198	34.33993138	31.08466703	167.9559752	14.2987129	52.27235421	208.6764	107.7376859
149.6718612	121.022221	60.71699261	22.21388184	148.4479239	4.472388966	38.34722718	181.58157	124.6176839
88.91926116	44.5305285	22.18714842	38.39493015	140.2500629	4.260587656	46.81036686	174.75383	112.5016021
139.0464337	82.51719513	52.78177965	83.08278904	251.0951082	23.14348817	52.65458011	289.83842	129.3632997
45.40008905	39.51273343	13.180281	31.67179582	133.1499183	9.42480652	24.90194748	146.0689	50.93848712
133.6913155	50.47188169	19.95476985	15.21876884	84.49639272	5.711672981	24.66539839	124.25381	49.52809775
99.4145559	98.52933623	32.19080814	32.25616522	122.6318431	6.451553144	34.0201618	152.1611	105.0182217
80.78290193	49.06036942	23.29749945	31.44980018	112.6503774	4.909624879	50.52318641	152.6012	127.9777642
70.01271547	62.15963145	29.13436847	48.57773755	186.0286378	7.371225573	38.95753536	232.17182	84.44579043
83.32689713	69.44130631	31.05877245	39.43984071	148.806194	8.36818722	42.17740543	190.34047	77.32498979
212.4402185	111.1841044	38.11753735	44.69107872	155.3255182	5.658067299	53.97377447	180.99789	129.9166254
78.94829946	98.8269344	28.28667249	47.41760239	191.443613	9.209519253	46.91639844	258.35259	140.9341632
115.6086246	70.4306494	49.73259129	67.91269764	207.9790664	17.84940465	71.21232922	266.34606	111.7939522
137.8375085	52.92234691	39.11892514	76.68265906	169.3219406	18.28258568	50.95833976	185.23928	120.1020509
165.7820955	29.7228974	14.21624954	21.09654193	76.96977647	4.059180637	19.42696425	106.16882	25.58929241
174.165633	68.00906998	36.79607632	52.59690155	166.1109145	17.36102291	38.19534589	226.04259	128.657386
60.45500956	46.92502547	11.70001509	30.41970058	108.630997	6.269434737	34.0310827	152.37122	73.0365142
24.51501141	54.37669374	33.81300032	45.9895843	139.9683091	1.965070232	41.77433005	163.78139	93.51603966
155.2727106	72.51327165	53.8667694	35.74592543	124.6222173	8.26015649	26.09243882	166.00212	80.44834284
69.6056513	46.22906757	37.16942492	48.22768143	157.6424404	5.096978194	59.70217721	227.82643	114.1663428
104.8784112	32.78583949	25.6811122	37.86028851	170.3221121	21.13228526	29.1374886	190.80768	78.81963932
68.55287173	53.13131152	26.26108708	37.45279757	120.9734323	6.315743747	49.3598562	166.51966	80.08098615
163.7503576	93.92155955	19.12739017	48.46621916	199.2781627	5.574062041	68.74296176	221.31657	207.8649469
147.6708227	89.77478094	62.79030109	53.22762943	154.1961394	11.25220597	54.68636451	191.67309	89.29874709
60.44516923	54.5232435	13.71889092	34.37916146	113.8450775	7.244766179	44.84415468	145.29336	65.63645114
219.3284638	116.3393983	106.5520097	69.85191021	238.1232643	33.78813586	86.5633514	267.54538	204.502279
116.0455345	39.76607763	18.11461757	61.49892632	138.3025706	10.78957216	42.57452227	161.60284	65.82055609
174.9376485	89.36071901	53.65508948	35.81678129	140.316181	10.65634445	44.26767571	184.35556	107.5509717
105.5772903	36.76489132	27.59474928	30.40013666	104.7871444	7.826359731	24.69380908	128.83689	46.54591053
135.8378263	68.45113935	25.92342318	40.94766307	144.469063	15.84024986	34.18009287	179.60874	79.8468221
45.66806322	45.97380898	63.61075999	65.66367333	173.0589529	24.66213551	39.27795517	192.24103	125.136829
163.9534385	24.89537243	22.6799329	49.52646689	138.1028013	11.90035532	22.14536525	155.30639	53.60808672
93.14316011	28.16190906	14.75311996	24.62934159	107.5862959	13.97074013	21.10762723	145.04194	40.44162904
120.8163208	42.88191664	30.21887537	33.65498326	120.6492328	8.025615653	35.52277365	154.54629	61.08942754
39.22937421	61.52636652	9.68931543	39.02503646	122.9927219	5.987421033	37.91015365	142.86299	81.61444122
73.33889515	61.10201949	29.76188792	67.73682785	223.4297201	11.86328073	78.34560362	306.88458	177.3838519

131.5619502	43.55638359	27.89816163	20.85500279	110.1101618	7.261194871	20.89926823	146.3622	63.66119201
111.206996	84.28777942	66.00697441	36.91188312	164.3323537	17.64526423	46.7969457	216.09487	101.1666883
118.8414316	77.72019378	18.03997707	75.54343834	194.2389157	30.04413197	56.04412868	222.75603	122.8314491
170.1288655	64.01691693	42.82637565	72.18424738	220.500046	19.79296799	60.03713357	273.4303	129.3639129
98.35653224	56.15922696	25.70143782	26.66138598	136.548679	23.88058113	32.40999149	185.27941	79.44122659
129.9485295	76.35130143	38.82566332	43.47562172	172.0493335	8.11272075	53.84599811	227.73146	94.35340696
66.4735936	63.95973635	14.66671334	30.2945602	114.7093948	3.210954414	43.6602691	140.82081	93.93616239
139.0545304	86.73094633	40.23813664	23.509764	125.8839774	3.388679277	41.58696089	183.28982	80.77272068
170.872777	64.651354	12.56734002	84.21482221	215.4343104	13.42097012	74.21829378	228.68102	169.3256484
132.2535347	79.36218401	216.1551969	147.4706072	374.7250248	39.65812833	70.04943209	416.39316	261.7082747
174.2490213	67.77571229	21.37725542	51.09193172	149.4752911	11.62301579	39.72305376	175.25907	65.90392716
147.0257655	84.84956522	41.67848479	37.45653027	163.3176609	15.35969749	51.46919399	236.18425	118.7103844
129.4590273	26.32868157	23.36685518	86.25845595	133.9458653	9.256263387	37.37637332	147.23888	70.69563621
103.1509511	90.61993396	73.1467431	49.46920606	193.9550397	21.94025395	60.84286402	244.86973	142.7382391
93.49164873	42.65657558	26.60111903	34.30056369	164.6035156	10.9262688	50.22114082	220.15331	110.566204
129.9625804	36.83702097	11.34182106	34.77719651	85.70961343	4.416543913	18.80112783	105.21388	58.84544379
119.2270338	53.74514322	30.68377428	24.00791615	117.4768523	10.10680968	32.88037112	154.00167	62.6483081
121.0588804	35.86251454	49.71400641	35.03015914	192.59513	27.73278017	24.12721776	209.87035	75.9402048
290.1105792	54.39232584	34.81596752	41.7442986	188.1110746	15.27476761	30.27061055	185.47495	90.52202896
283.1387651	57.06623417	26.99221093	56.02226578	236.9880351	22.99070641	52.32268068	207.33908	94.11518995
101.4114121	80.59961774	42.08441051	46.93280807	142.6947582	12.97153654	36.92866879	198.58847	101.8204347
113.2972096	46.90907668	36.04930076	36.18749445	112.0698197	10.1815387	36.11846583	153.10394	68.87582551
216.3940006	122.4633746	15.75005277	46.39396858	182.1711887	9.109576043	30.57170987	191.79377	106.9672785
140.5748738	50.10964793	43.06360381	50.6451859	166.574778	10.74680083	31.35908143	156.40312	92.97643104
121.1057328	44.44369313	14.95313687	21.5387844	102.7677445	2.579324117	27.29018107	137.68435	59.82015786
249.4484944	54.13048842	38.84526336	58.84746561	262.2803811	18.25677304	32.75151893	278.3525	118.9223808
89.22404743	60.96374197	37.94085259	52.40209938	164.6395983	9.28881519	53.41387673	205.35213	104.5497921
125.1754192	99.80936626	54.45838566	47.84054577	211.2214476	9.849360414	60.48462337	240.75558	163.3938622
65.12986247	57.04536045	18.1430366	36.84093764	139.2697325	2.23882304	41.42559732	195.88445	80.46117886
149.4913103	41.34197616	25.57262767	27.0710594	117.7788445	18.15933598	30.78210203	177.00513	77.71297556
164.2236906	121.3452387	54.38503176	76.84821946	224.7194393	4.274243957	86.41156818	241.71293	237.7580319
151.8752764	67.43458362	89.77070235	30.44589897	162.2305422	6.596918146	34.36765194	173.93704	71.68915239
176.186501	126.8019221	63.77960828	55.89380138	221.8510401	24.09795052	56.34734519	257.12114	137.9933753
52.10856891	34.18923344	30.49657966	50.35062305	118.2764814	4.953047857	25.85562505	138.35291	63.44268667
58.68128378	20.90746594	14.63162047	34.29596712	116.5882349	10.55208596	26.69207846	132.53188	81.56637707
138.5253251	44.81631763	22.01157322	22.80971656	108.2625536	7.691047099	24.5567516	142.1044	57.18961402

136.0764977	65.93905958	61.88441867	38.48778577	136.7290593	9.267446042	48.04888621	175.26719	78.2105666
153.0790508	47.33691127	20.42159679	31.90724688	154.2762128	14.07448375	22.69205281	166.42525	59.7585695
142.2201247	101.570897	41.29877812	28.30188773	142.2627327	7.033412381	40.05337812	205.81548	89.9110001
122.1455321	81.65848538	32.13121554	57.77567963	238.2766236	14.45631398	80.46276318	323.40804	151.6158638
243.9124977	38.97300289	18.2915534	36.48635665	108.7753191	12.62590934	34.64942906	144.03702	43.65040012
81.89961159	41.62698883	27.07525151	42.09519182	134.7572415	5.444421875	24.85698183	151.99733	68.22953464
188.1235195	97.7423144	69.38940289	31.50900698	146.0976257	13.98711362	30.08315963	206.50628	93.13142142
224.796335	45.72882319	23.41421268	37.17600792	179.1742896	24.57056731	34.66597767	209.27847	67.84983925
191.1676138	84.93818276	42.41080729	93.23080463	170.2291597	11.55556985	81.92737011	172.37709	68.00411733
119.3174927	29.62870712	17.02418018	26.38151367	106.0801737	8.569004719	18.33258956	131.41269	48.35975261
106.9221507	40.54785734	47.19717265	61.83514078	180.4021732	14.69610439	37.29826816	179.1503	70.51320097
135.6328835	30.50438716	23.56527103	23.25266341	79.66113723	4.528863501	18.90484369	108.1152	35.32671233
84.31357676	57.95533775	25.12541684	40.35007806	184.2081046	7.686839705	51.26560868	232.97776	96.36771853
147.9888805	58.03422299	80.02614791	40.92802252	204.150632	11.87318851	36.41377752	247.56948	116.3461786
57.50533999	57.76193698	13.00655316	57.73694946	161.5047415	1.355667291	31.66426237	150.75423	62.28067764
107.1761913	33.07961948	16.58742975	32.97240892	153.6182368	3.784389608	30.28243193	127.43485	60.31305308
113.9130259	62.11145679	21.13292868	29.32593116	107.4349946	4.696120146	34.5190061	151.64001	61.18674431
153.9715657	63.10931024	32.97801786	38.38694896	120.4233715	13.91542253	37.72282066	134.49435	50.57950308
84.58706196	124.9499996	112.2873751	107.8046393	272.4254767	11.53763933	88.24699859	318.21782	223.6511201
92.60714714	54.20492282	44.2917266	28.19487642	131.7392902	9.393606694	37.56497003	180.27678	85.22279918
104.3906405	43.15773423	14.779173	29.20422077	98.79113658	5.659224228	47.33362576	139.27249	57.5053686
105.4684272	43.44020757	23.5721682	29.05107681	118.2260763	4.342114716	35.06939611	163.17097	75.98827956
43.21695048	140.3709612	20.74334889	36.57479774	170.5944238	3.651268241	57.65779499	186.31284	91.97809061
125.9130874	49.16663838	43.19074929	61.72034003	194.5941151	9.624908959	47.24105002	238.18821	78.51403629
176.2740769	71.29673076	54.6537792	42.16418374	203.8943154	21.70794312	74.60842182	277.72888	124.497347
54.55430721	29.93672255	31.23607625	51.51344407	110.8658206	2.416118658	34.64779493	145.40833	79.18001939
130.0593607	63.59493885	22.59622605	28.91715376	137.7475831	14.89479627	44.055998	177.65329	68.0251598
185.0095642	45.23056955	25.95780186	44.40407383	214.6794532	24.41322633	31.47572397	232.21379	83.93803413
74.24088791	24.88885489	15.8881031	110.6883272	115.4051281	7.275972352	53.68918944	142.9825	91.82494117
129.7558271	35.68445369	20.97242169	30.96116533	127.512989	19.28299614	30.60747849	162.65245	52.70212936
78.13624997	57.15201678	34.60046608	36.08626873	176.0225876	2.868043822	32.30268541	206.80898	81.12715298
95.80169342	97.95883246	75.2394109	34.68355406	208.6413204	7.744817805	55.46384961	272.005	135.6269487
120.6128929	35.53671413	28.6915434	48.60578562	212.627896	12.65640318	33.95346634	212.74996	81.4038015
156.3813502	79.19899941	36.75168986	57.40341291	151.2990771	9.013251257	48.83182192	174.02649	162.4500394
175.2936756	45.08262868	14.3337628	46.5731016	138.3446067	8.177080531	33.51730267	150.06038	53.69190893
134.5910224	107.9604075	30.6534704	47.060066	154.733657	3.840274013	54.10632158	172.16395	89.9593149

73.87883874	38.52333406	11.22947819	32.67429311	110.296666	5.712992642	33.57183382	133.04688	67.51886105
133.9612777	60.39810748	59.30881996	53.2176759	206.7695574	15.83451885	48.44163004	252.08211	114.4209294
115.6400637	43.61233721	18.29026152	50.11692996	161.7378226	22.3238201	44.61645141	186.44622	75.48986181
144.9111196	40.16306146	15.91501719	37.13731595	150.1266889	15.03556943	31.94474405	193.96737	61.87307326
56.90370825	63.29150953	13.67080573	63.08689093	165.7674803	2.475797136	54.34712633	193.44737	76.46768495
85.05108356	117.0878256	14.48372339	50.35659208	146.3368523	4.891025541	63.98078723	179.30653	80.10670191
167.3144122	58.56371956	38.050503	43.77588961	219.7759332	28.66921906	50.24249501	254.53382	157.3180196
224.9962807	124.5040886	46.13851835	66.98070065	340.0287573	33.22272532	59.81162417	351.51417	246.7614127
116.0108166	66.45823266	46.90621858	46.1650142	180.6137636	9.349025979	32.31072569	222.0915	85.54332545
102.2586981	33.04769884	24.56915405	26.35089304	130.2223457	2.731806483	29.6817579	163.17916	64.89204028
89.97373337	43.8640848	83.33111851	86.96390074	211.3870003	14.24721896	51.42502125	212.44852	186.1106278
65.37648808	35.65298121	18.95684046	43.3781055	153.5819962	9.282218327	45.31138	172.99221	122.3869264
92.97128347	91.48509683	36.31885418	40.2387756	193.0229618	5.304941615	47.97565798	212.24111	102.6877064
181.2987262	63.54295997	47.92150705	52.31495704	193.3854236	23.16611557	46.88745592	227.21535	148.5922783
167.1896839	57.03805281	25.44360169	37.47971165	120.9866646	11.47576676	40.49542124	158.87878	50.68330073
215.3645376	59.74422224	44.24912272	44.39167622	175.7125205	16.81811542	37.2463543	202.71743	137.5064467
118.1543983	139.5522382	44.15770168	40.34201184	196.0596656	10.56837152	53.11062691	227.18396	163.1679125
95.41163562	42.72356229	21.96384366	36.20675826	129.0140345	9.438303263	39.06985363	178.92969	95.33852217
89.06801849	35.36827398	14.28872399	17.47299842	74.11560807	4.495938131	19.38686339	116.36166	39.25890256
145.2409469	29.0929491	5.511876888	12.40682385	55.18241654	4.332408103	13.92411459	75.824212	30.48888455
218.7493105	26.18941153	35.68953996	59.04991935	206.3024404	5.672561764	32.9144608	209.5317	164.8868421
31.72446123	81.30174415	38.40627872	79.90441772	177.0901925	9.448353047	46.51537494	216.15794	157.5351393
88.0273587	25.98064121	26.84040508	61.91229087	153.2793408	14.46114098	25.50254891	153.90207	90.12467419
169.0542512	77.14048513	74.14626342	44.19908952	185.4567795	19.50338549	47.36695507	238.48678	155.1770298
282.2057669	61.77101703	9.365525183	46.71967249	252.1310327	50.93168413	36.31962911	257.52469	147.3672498
78.8550345	43.38201232	8.185708551	19.45830684	84.03445036	2.277361735	17.65216806	119.93625	55.11749785
54.86372794	64.04576908	55.59858219	57.74465922	221.5563064	15.41717265	48.30978861	250.47887	127.8073841
99.12250482	45.70100328	29.90483164	31.43317673	113.4689408	5.304913199	38.32955814	155.16273	54.57530972
172.9245175	40.55817758	32.23282204	27.38319525	139.6181297	27.5900445	18.78199733	141.14992	42.32479533
130.3039026	41.05952074	19.77358539	26.38994867	151.5885531	7.661799678	17.59021214	168.81687	52.94465744
96.99016177	70.62039599	24.08764678	61.02541405	275.8364988	46.9684724	61.26791698	314.02534	120.5507793
175.1435194	101.3846763	54.94651157	43.352037	186.2086093	14.73854375	57.07862187	226.0955	103.0846839
57.51772649	61.11607914	26.15001634	61.36111106	153.4399458	6.384888308	52.07790373	179.99009	100.6300076
57.3890235	81.84311478	17.75992133	65.4053905	161.5223117	5.664032796	61.91918991	192.82396	119.2653116
201.8397355	107.9858921	45.74327479	30.7993155	134.280124	9.163324539	51.11898484	153.41346	79.25948137
119.4909612	45.60683053	19.52312344	33.33014591	103.8912492	6.7047954	28.85969615	151.15331	49.44083348

133.1977451	98.4787027	32.20130887	40.15613917	155.4841102	10.78852387	60.52070775	220.37586	97.73712241
109.2071044	37.37892089	10.45584268	28.29298591	119.107632	5.398272104	25.69787153	137.55919	47.78099726
141.6508983	78.28070944	83.48624656	63.09005168	208.7053342	12.23485756	41.04295282	271.10442	131.5244448
88.4019576	61.63192364	71.71619013	72.12810685	185.9289157	12.52404146	87.11739147	224.34995	170.4904784
109.9552847	69.22078263	50.60685509	39.00435228	174.0348236	18.10557598	44.85052503	210.13059	143.2122942
296.2118718	83.27279555	37.68539353	56.9480518	249.306368	28.53871949	48.75105306	274.17413	141.2149773
151.4121293	58.41675861	38.28713837	39.41640464	162.3297556	13.58011879	42.05630336	187.51423	113.1426481
191.5006297	57.12562094	22.75397313	52.4278371	238.9046489	12.55895554	59.60307782	289.77997	108.9573591
88.92669493	48.01095605	33.76270251	34.68194107	156.7285167	5.610187253	35.04513402	202.27665	68.52540938
388.3257957	67.26989046	56.7545931	77.39168271	341.4603295	20.20878042	61.77147264	341.25527	180.0728366
131.1753247	41.98603379	35.36735899	34.89365294	173.5281562	11.7692317	26.11058904	200.06088	79.98747158
165.3303575	71.63332717	45.80786326	44.20616081	165.9137055	20.10499541	35.92933372	193.98024	94.74583149
142.1259534	111.6766833	42.85590654	30.3722747	132.2853882	2.675378765	46.11025468	190.94	93.41746169
132.6672716	101.1625798	76.54051112	30.44392447	183.4047375	7.541138521	55.71183135	241.08764	120.6920315
136.7148018	59.85600737	80.52785971	39.83195406	125.9073065	5.182665481	47.7227204	177.11979	94.53233659
55.52078722	82.09791983	30.98744988	74.96780718	170.9182716	3.504465077	65.55831689	205.56173	102.788985
118.2805094	55.52194309	45.54795129	51.64544517	157.5423203	6.610757411	35.95958144	207.1627	83.06800174
131.322479	48.77054195	24.12683675	29.47387409	115.8153038	15.09773798	29.61042297	156.96411	54.7432101
142.2255725	64.88647536	46.2840158	50.16741269	155.4970232	11.68992219	31.17023308	192.71062	85.31257499
132.0042113	46.13330987	29.92380475	44.89054935	164.5556069	22.5608079	58.33215857	248.76696	86.87877585
115.85386	45.52617547	12.70080862	27.19022457	98.55321774	3.398501871	33.91223927	128.16705	42.28321632
123.6390805	54.9073202	33.28662616	37.36085925	120.2001452	11.2579341	31.97140851	143.43628	46.70410206
132.4332799	38.90844986	35.99233698	41.1592622	144.2898453	5.375595825	34.92558252	186.90619	131.8888979
190.2516116	71.60832126	35.35961894	25.72871632	156.7825534	23.81558683	28.06510491	181.75405	73.41174297
116.4325865	54.40509473	19.84368585	26.09652736	109.1217394	6.885361841	43.29679135	140.68871	47.10836535
126.8204557	53.9823832	39.74297269	54.75538144	184.9319811	13.33627142	51.58280743	230.60762	116.1705535
101.8826906	34.10612695	10.84137669	22.31916946	83.54302046	5.949500749	15.84698114	108.16114	33.03629805
85.30247366	29.14178518	13.2537033	22.14922067	104.207357	7.37993735	22.93133996	139.54068	49.75073646
128.8446111	53.73935216	43.85982886	26.69734509	107.106315	10.47675112	30.60491785	115.90304	62.07272818
89.06931823	63.5643258	48.63507736	44.66751878	163.1812748	19.56163292	49.2658868	231.64883	122.110187
194.4312241	62.15525694	34.57922793	82.67611382	223.677723	13.18057707	55.11272163	242.16575	133.7111577
85.99639783	23.00024208	13.29508042	26.6059711	132.9020167	11.51930963	17.41992319	135.57569	71.39593306
155.0818899	58.25402801	23.89388778	31.64951925	139.8130052	19.62937	36.95727091	177.23555	74.41154318
164.4447074	125.7749708	84.0530308	34.88736793	227.7063643	6.513110559	72.42396315	249.39788	167.479857
132.3864959	61.00005572	19.41762145	28.91143853	122.0377933	9.864451729	38.52476083	173.20918	69.35679245
118.3644479	71.14906686	91.08843153	32.63812029	138.7979813	12.64157775	50.98311189	198.5298	124.8642389

97.56792661	43.07883233	19.02928739	29.065058	120.8237266	9.072824233	25.87010208	184.88097	126.0281122
113.75456	70.79113703	28.86678937	51.11452951	143.0131448	12.1816908	59.79725341	175.97394	73.56652722
136.1199286	53.44592173	22.18049279	30.83480402	136.9496253	11.6163584	36.85325336	187.93827	75.62338217
83.42167801	42.03629535	16.32513809	37.32946086	144.4758339	2.874248118	36.99809651	148.72122	52.70776483
176.4708333	69.71323827	27.86062334	25.07690646	130.5982965	10.8499974	30.93877992	188.94832	73.66353322
162.1971559	84.38137863	71.29296869	55.33879835	291.5657785	58.14861475	75.74273797	375.03436	252.1798137
90.03579963	46.93842657	10.77363443	28.18595369	164.1131475	7.033747228	49.69557225	211.58436	97.40467829
137.1016627	32.99040686	32.61129263	59.68253028	176.654396	19.35525353	40.77832187	184.90753	63.36221281
308.1079958	42.23063334	39.07695451	160.278904	299.4017208	45.02794387	96.09088671	302.47664	154.6393024
79.03382758	30.11350869	18.63806694	22.95416068	104.367135	5.401700953	16.82493702	149.55466	45.956894
191.9306115	56.5505239	36.53762859	54.41971438	214.9512421	8.280569294	49.84960027	233.64571	88.37502088
87.06407058	75.83043955	20.0959503	39.17335628	282.2413346	31.11660486	98.12198898	323.25236	153.9768312
162.1565889	49.43639029	23.35274123	58.94343748	315.7072547	50.24956658	81.73863452	315.91826	162.4252991
115.9508058	24.89873865	16.96255034	47.59334661	148.5215985	5.563332437	32.78635881	172.72034	91.19899737
66.14758029	47.58985939	5.081502277	24.94141755	139.429335	1.928243884	37.68524735	187.22	87.51488399
151.6208164	25.15377298	8.302722412	70.84028182	204.105346	6.574113211	33.07484648	166.36629	103.0945607
146.8918571	57.49082867	27.72539816	26.50250397	142.242402	6.665185699	37.09599052	168.70663	65.45585853
197.2390048	54.5639211	23.71142215	50.24033681	148.2172344	6.409561467	76.31723956	180.2772	78.62324482
168.1689047	45.98584652	11.29236988	41.63085095	176.7750944	15.11452494	36.91308991	212.30228	90.94493884
100.9533093	77.79412281	51.2175861	41.1059583	235.2326889	13.38255636	80.33636917	287.48074	124.2942507
140.0037897	42.73888651	9.52678816	26.10521561	91.53608045	6.05979155	45.0474774	126.43541	47.03366748
78.95855298	41.58897828	8.801001231	17.97158218	72.76697764	3.873313061	33.69665582	109.74001	44.12799451
129.1203044	60.18689061	27.10931445	30.94579266	158.25279	12.51732319	77.91675061	245.73206	82.46580868
126.5624857	43.46826146	21.77175853	24.38590135	125.6447978	6.332498231	33.78053045	154.3838	52.12681311
107.9468267	45.06656077	20.81705483	45.50296057	197.2227969	9.869214124	48.32266534	208.07678	102.5112082
103.0189135	53.16759629	21.7435887	36.6703933	151.670226	3.407595042	31.95810445	159.69486	73.44201385
206.6410562	63.73879741	46.39887437	77.05800157	319.5025589	38.09568192	96.75946713	289.79098	174.0749732
138.4031502	49.46928839	20.97532561	28.76501326	129.5544151	3.684688592	46.51052471	174.80865	88.97979323
116.9971041	44.39755291	14.72090256	42.83439938	206.5069029	9.205358463	43.53832374	253.89794	111.8176689
200.678888	97.23850558	5.281027158	33.80821359	129.8187388	12.167839	51.07893534	147.77453	38.40198288
153.466492	76.85883541	43.48597622	35.72470565	167.0463187	7.029920982	58.91148872	212.55317	109.0581424
61.26197554	24.34201425	15.8896369	85.07179895	216.2216163	45.68550778	58.53709442	197.95523	105.6220751
54.41738607	29.74533434	11.48552922	31.53600601	122.5510953	1.390505236	25.3468205	140.98721	45.88129351
46.80686972	46.00207126	12.25679837	40.57354164	140.4470163	1.229517301	37.01302598	164.57447	115.7379047
57.84235099	58.34131302	12.26935869	112.1700081	261.1929613	17.20160847	76.66350017	251.91778	79.25884677
99.87846116	42.81771597	11.8912271	36.47050297	181.8952956	18.51097673	36.28646602	234.71057	122.2481085

78.56701264	31.87044716	13.47050371	31.31439833	111.6292706	1.663735817	34.51917185	144.68385	75.39668985
79.53278704	32.84342763	8.563664843	17.99990207	93.35324657	5.114634885	20.09328595	132.97741	35.02206744
105.6936163	40.04991756	30.21977227	48.3637079	147.4322558	3.253906886	43.37181524	149.00642	79.96550646
137.3220636	28.52397482	20.58619203	42.93011871	172.0615446	17.02498022	38.89460521	167.52583	65.63558442
176.8209084	32.7575832	28.69597427	71.06148678	166.9302474	10.53981909	24.51139764	158.81138	75.09121154
148.9208461	91.6175367	59.21665452	74.17484094	224.2029993	11.75721992	104.4743296	240.6423	77.06625762
127.4950929	53.3334177	23.2222174	29.83148698	152.9592378	6.621787961	34.70713294	183.78316	68.05791957
159.6326416	37.39971421	18.8134336	55.48101016	245.8845027	12.71191723	38.94379037	235.14292	132.1748736
49.63334292	58.14006115	19.16560109	30.469379	106.8158549	1.909703565	48.73999456	148.61674	61.05793184
70.91865726	69.90541882	40.96579995	50.53344956	171.0386026	10.61537109	55.50377652	222.32271	132.2344008
226.8559247	46.53199509	32.14863629	58.74446777	191.8309962	0.830326399	45.58597438	194.33796	93.91355684
104.9527207	25.39797646	6.551350968	34.56873847	175.8386819	2.215787788	28.19606978	182.80223	134.7497357
194.8517186	41.52856816	13.24668343	73.84249009	237.803486	2.111638246	67.49465094	166.12563	98.19921761
148.6041176	48.68668366	37.08937213	60.88761258	187.6642042	16.64101328	54.95625056	228.28839	82.69609765
124.8682069	53.0164777	49.86010308	82.92798752	235.6372923	5.269689763	46.01816498	210.78534	200.9924431
204.3003764	30.8668322	11.67679939	27.72194492	163.5318394	12.00128582	26.2999974	208.41774	87.94053151
151.5627271	36.70756046	37.79175968	32.97585488	150.9859073	11.77630966	35.49774891	189.10478	64.5202333
98.83137542	90.57593758	17.34985639	60.34233177	272.080126	7.124604657	90.94868384	340.79825	134.9593151
145.464134	46.74416548	21.9873224	42.79125569	170.9434916	5.127370642	48.34665889	178.40816	64.10078289
159.9830363	19.53177357	10.11438682	33.31226162	91.09436613	14.48741111	20.07070582	113.40656	39.80060332
136.0621596	27.79747225	10.13178725	33.90560087	181.3025209	7.353261461	30.07139841	163.88701	94.71775772
83.07586375	37.66670221	16.42366551	25.41330939	129.745028	5.987924643	37.68644684	180.34482	76.50315583
108.0171518	71.22482735	18.38933274	24.15266251	177.0062092	3.377542003	57.45503774	237.47883	160.9589354
78.64678348	26.18428497	17.20059916	71.67745386	135.3192568	3.934575634	40.01355169	147.42321	68.44697671
157.2261743	36.43871799	13.80702894	38.90278643	187.6305532	10.85750403	42.98607265	195.79349	85.41376645
175.3103051	78.442895	28.53522653	34.92523476	139.5883741	11.31154441	58.60426982	188.57515	65.06674994
145.7685072	77.88075825	68.17349837	47.58400024	243.7568898	29.88785394	64.81932801	279.56341	131.7406675
84.99878717	29.1337666	16.22227484	30.07661794	127.700819	8.529051759	21.5524846	156.50369	46.70357807
73.41499815	32.08325316	10.32395541	22.4751633	103.9641537	2.885283513	21.34004216	142.07303	40.51002414
83.67561102	41.33326536	34.6013494	29.46937069	126.3286538	3.748704256	25.94626801	149.88685	62.72043425
99.19492452	51.41753249	34.47897038	72.99969574	233.6319394	34.63149432	75.65734731	282.18233	129.7390775
73.44404299	25.29657684	8.367372624	60.73300453	142.8316988	2.870543192	30.12489349	151.39002	86.93606757
85.62555405	36.27802688	12.9364737	16.99165186	69.94226902	2.490488448	17.49451265	102.17975	31.73301402
77.49528933	51.16030922	76.04491363	60.40125791	229.6584975	6.465286848	127.581733	230.14224	213.6123229
59.86319743	37.58120901	23.3409868	30.74575838	159.1968345	4.826275157	31.06162152	167.40584	80.02680549
109.8616684	36.64641722	11.11553401	33.05614695	92.41479245	1.64978444	31.38647761	134.38003	49.29251544

105.0941234	47.23392356	35.99751142	41.89713219	218.504409	25.26184494	56.05382268	242.02242	95.55169549
304.9771947	34.29047752	13.07676178	32.24474917	175.0705235	3.329374892	20.15256319	132.37988	68.4772663
124.6174564	58.53704751	23.21115244	35.74261051	211.7823175	6.864597781	39.43844562	228.04181	95.08376015
143.2564383	54.27098085	21.83104262	51.18274761	183.2776973	8.116989556	61.7322812	222.14833	83.19552882
93.02283815	52.88123553	25.44754791	34.47030454	153.7172857	19.85265246	56.16784621	233.57647	93.57668187
116.0796059	60.43910831	39.38710972	28.44460233	158.8688758	11.02934111	33.48330877	182.56515	59.59812385
119.9252028	36.3967265	8.048833647	44.68689338	113.751957	4.944553448	30.61171271	137.52563	50.14910099
174.823638	41.28086335	11.41071728	40.60105091	112.181553	8.093004201	37.31543558	125.88659	40.80340082
78.77112872	64.85341866	35.71290329	29.31918612	179.0793738	14.28271538	31.97194137	232.9421	92.77179505
109.1667573	43.91796627	30.89140031	64.69033222	249.7839948	9.473307015	58.68696235	261.29801	116.0765083
147.8547465	51.88978573	27.17388384	45.3011493	164.8227625	23.64819777	57.86771282	208.32299	61.84018157
177.0539788	37.59293693	11.89371641	50.46678168	171.1077779	12.92085772	28.54856082	171.80985	107.6384052
158.7187184	17.7309118	5.851462162	33.96572724	122.3687393	14.1929708	20.96120729	108.31148	54.63932295
172.0049062	61.18483028	42.61148348	28.29581944	149.8245731	4.13358437	33.0323159	184.9034	126.4339072
113.2920559	25.72180507	9.710263554	58.09459908	108.3859094	8.041282232	39.52526918	128.38511	33.18060944
85.54839787	32.93819297	14.19752446	24.22180716	104.5730851	6.604431617	15.60968297	132.66993	44.70876115
70.7074422	49.07958059	8.028010792	26.13401445	139.7565149	4.461928735	41.04267857	186.26287	61.26534842
157.1544378	38.96820037	10.4540624	41.23162787	157.4867411	3.530069643	34.2987581	188.40607	90.91845694
140.2267661	43.14890237	38.29845509	42.25782618	183.3015892	8.180507	45.77437587	206.73653	83.97637397
87.51748223	34.26637401	15.77191677	40.90783032	158.412895	12.69561848	26.25795216	185.29217	67.3441377
106.5936146	54.24720337	18.74288068	50.59263232	273.2462144	10.07532894	41.73187265	280.20695	141.2700548
76.73059442	43.25838222	19.49362559	39.1808034	170.0389763	14.88797864	54.36755711	223.92019	74.97385171
157.1774642	39.67016837	19.683624	43.75907947	195.7164195	14.19687172	29.77769193	228.11994	140.6124946
72.07484624	39.06491963	19.68550918	39.49947891	152.6921985	1.516196922	41.05157312	169.35192	119.0381217
85.46244044	62.43670018	30.25908451	35.3208833	152.6010713	3.903935704	47.29745052	196.67314	138.2008652
106.2288685	37.77962465	32.68130031	42.75704619	157.0190377	17.69722433	37.59990106	201.30174	112.7011889
104.7164624	63.63606242	31.13185081	48.48930055	210.7281231	11.60097248	41.91460175	258.02006	90.15201788
110.4658572	95.09390473	42.16240699	51.25126977	276.2816739	15.38292285	76.27913214	367.16018	163.4693425
108.1260579	52.90392694	45.01985531	28.99769863	160.3803728	9.457474436	32.4559912	193.37777	83.20396861
184.2636298	62.88479224	29.48828566	55.33072244	266.3376183	14.6043066	44.16478019	266.69876	118.4135557
83.75759542	64.98901135	6.640663926	27.15275265	139.8270147	5.023576275	39.57928397	216.69742	168.3004797
186.6503107	46.09999402	29.94413906	39.92540308	196.349999	6.653925948	31.59345323	203.8806	98.10571332
107.1772604	28.4325019	21.12804563	34.69545377	93.92328366	8.610413162	31.73479115	117.89386	49.92238625
118.7007178	66.43579272	43.01419218	43.14262617	211.0240125	3.936840784	42.74126795	226.13687	74.32770282

Wnt-C59_1622	I-BET-762_1624	RVX-208_1625	OTX015_1626	GSK343_1627	ML323_1629	Entospletinib_1630	PRT062607_1631
73.899643	30.1589948	174.089225	13.51752728	23.21383599	103.1638765	57.67566571	32.13474553
105.1136561	19.13109317	80.39363089	6.199701877	16.1981021	121.0900454	36.49905014	26.42823039
87.38016747	27.46994579	143.5760966	13.17928353	21.41591623	99.37251434	75.44501134	38.75027051
97.42253392	22.12315626	79.44969145	13.5845501	12.88564626	149.0014104	40.15567853	23.21795401
126.0014102	49.86271781	186.5978788	16.96033802	23.19213679	191.7040163	78.31886821	35.26878848
65.91794721	11.51086837	72.17439305	4.896165977	11.99999037	90.60265093	24.46799747	15.82175062
65.58590858	23.34801241	119.3858862	9.508803769	15.43157477	87.23510092	36.11423915	16.92980072
93.60040315	35.03072824	161.6293187	16.7526368	20.39571122	105.059488	52.34756319	50.27968578
137.3859925	48.27246276	198.2752509	21.7232443	23.81666679	177.7932207	57.10681918	41.69945894
43.84149444	18.90558706	73.88780606	7.875027376	10.73483136	64.89220381	23.388386	16.32565341
40.24807221	20.84958101	68.37318418	10.33679039	11.48083114	48.25457264	34.25253312	18.525032
62.37990654	35.03536793	121.9727276	16.81808454	16.08950118	91.47919915	52.21874925	20.99457947
51.2407	29.61025572	119.6408783	12.70908996	14.69699162	72.35437494	32.14920081	20.5115854
86.0493765	61.10394175	126.5765165	67.73506064	18.2210111	105.3167701	61.73570702	36.87121485
74.71260655	14.92362628	88.71307749	4.640696963	14.41448593	109.2494042	36.70626336	23.14073952
111.4624906	27.18056586	111.8514393	10.66921165	16.24535754	151.4602774	39.51298842	21.97386396
49.37461737	20.01363166	79.42412811	9.881681297	13.24193313	39.2219613	59.99929569	25.49374531
100.8988147	33.76405135	247.0561881	15.83638608	23.12016174	101.6651671	75.73436748	55.3920044
105.957568	23.13078763	113.7331166	9.714745686	18.94704517	122.8943595	47.2747489	26.80985796
65.02220189	14.15149066	58.09753311	6.217581688	11.61565633	71.97492825	23.01855386	19.39236155
74.6756705	25.35499443	120.9508387	9.106122513	16.31490222	108.012635	41.50425244	20.88317672
111.6067961	89.49723375	207.8711481	34.61303835	24.87278914	74.04912221	111.0771803	44.97179902
74.93813797	22.87711643	124.243713	8.548524163	15.97515305	99.95574207	26.58671245	17.79104476
86.77666472	45.10758766	181.7444201	16.58375715	21.92263674	158.0479996	37.53261456	24.48958457
74.622922	31.17501492	118.4747415	12.03978408	17.20666055	111.3082981	37.81547862	23.32916057
78.1968982	28.29170152	118.0199256	13.35433726	17.10125886	101.6540941	41.04813734	28.34361681
104.6237639	41.01033133	152.3162362	18.18985481	19.78425417	124.3443193	59.80252412	33.35917101
109.2197614	34.5788138	169.8186651	14.07253496	20.48808871	205.48407	34.99692581	24.80545603
83.5291804	16.57317746	84.23458769	6.056421407	15.48689559	120.2453544	25.86579509	20.745327
45.47188999	27.7577223	86.84452016	19.61281573	13.31453131	59.75196012	30.35993373	28.61852622
54.37194046	30.58443303	122.0737364	12.6845329	15.30794522	69.50609567	37.23616778	22.21854794
86.4350205	12.8800915	79.01026262	6.171923149	17.39439991	101.757541	32.06613462	26.95013586
133.0136238	29.46677706	164.0752877	10.31058352	25.43089832	142.6136314	56.62265988	41.66629147
40.66727741	22.80345393	109.775789	14.92001361	15.25802077	54.83479261	48.31515408	49.89381133

74.07084675	38.2078025	153.9683168	12.34340195	19.00004196	100.3309919	45.41378205	25.57702791
95.38918712	34.01963312	175.6274716	18.88791911	21.77779173	108.6710591	45.61689327	33.37973532
70.78675839	11.86460551	65.43154825	6.189910286	11.21016249	89.36598175	24.58601782	16.31486189
133.9176992	31.05361169	168.2977054	13.10666139	27.88986049	160.4060936	68.35720541	45.09730347
88.80795174	34.26188855	133.5379749	12.32455857	16.78339252	101.3253487	37.01826171	21.50483499
61.78162535	68.26673636	203.0981744	28.47968745	17.19802937	63.52867029	52.98325643	32.54828721
61.63604113	38.00672226	169.9229626	29.12445129	18.48820494	91.0325566	53.82072607	52.39449416
98.727299	17.78569304	89.43474564	7.7578877	14.51323861	161.3313523	32.60807762	18.60250403
86.7605455	25.42592748	141.6947204	8.776776561	17.97153165	103.910076	43.10277055	25.95488914
49.30085095	29.32184503	124.584221	11.26948015	15.04204995	87.70530645	32.79563831	19.13890174
59.43220323	41.60459507	135.8312834	17.72981914	18.04710756	63.91353008	56.63290236	26.83557434
26.06152788	19.4874903	82.58634998	6.192156282	11.07447826	33.46147695	44.86425036	41.82587571
59.46766788	18.53636718	86.99203107	7.014018292	13.14183531	93.85147627	30.14359554	16.23836443
87.31520333	30.21228571	139.7570638	17.11438016	16.56635131	101.7458229	58.34646783	38.17802413
53.91769238	20.53705706	70.26301209	8.366670445	11.9313791	81.75281411	27.33493704	13.28271458
70.47622491	23.94105919	100.3608664	8.683184987	14.30612338	84.72089065	27.9840244	18.80679484
37.15702325	14.3132264	54.43105161	5.845221929	9.348403439	55.80026382	23.49550507	10.97611944
89.77749833	18.46806989	100.1522382	8.050571495	15.57723769	138.2659935	34.32024815	19.00738912
76.87881381	16.26762252	90.6082162	6.291043932	15.708226	81.68740744	29.73659766	18.71119126
64.36686938	22.53923775	89.67492093	10.50777625	14.61256007	74.58073268	37.63756882	27.09839772
49.99298455	22.26568823	106.3983942	16.86123226	14.14045657	71.53935997	26.56176914	32.50317232
71.52922677	16.65847305	111.3915962	10.34125486	18.14455409	107.5597768	33.31280994	35.53488005
62.55879752	20.37202133	81.65772755	8.385795081	15.0139286	75.48516551	39.20423784	19.64843515
66.53630806	17.27159764	72.6187208	6.431393308	12.1294773	92.3492556	25.23341259	13.43503788
76.65321236	37.6034881	220.2232537	20.8887073	25.97212767	113.8076173	62.8536414	48.50168832
91.4930994	64.34481289	219.6063594	21.97706034	24.14923472	97.30276519	55.71459697	32.23795406
126.9776808	53.98878706	185.8007131	25.55462571	25.6097413	172.0202911	58.88655927	46.61143033
71.11627509	38.30130454	152.409211	18.04501293	16.49276424	93.39336727	39.26749907	24.88676129
112.1415324	51.72863714	186.0452496	21.81135537	25.31391492	116.8269784	58.65760665	48.3631883
80.50380501	31.65621929	149.9395406	15.71457208	18.87663032	69.14571361	54.19384818	42.31663142
51.75110451	20.71267845	99.26446969	7.499621041	12.24837077	94.63126844	25.33120042	15.22961027
54.89627095	25.09172539	76.68579902	10.08906027	12.00352849	98.43305682	24.82059709	13.35503186
126.820896	37.28937234	187.2783649	15.46144141	20.27374944	123.915135	52.03300346	37.31249355
159.8249105	81.9804456	348.9131245	63.28022109	36.02310575	233.3767614	104.4858269	74.81315047
98.31654607	19.60213352	101.1259271	9.772236402	18.08484752	109.5248183	38.04938755	24.58506938
44.02443817	27.96841991	75.08974639	16.42945204	13.81624366	73.07724948	37.65014765	21.53518396

131.1892042	23.62583746	139.5811674	8.416660241	21.9655529	128.2309902	49.85875392	39.54880326
42.97030794	12.77078681	69.61775817	7.148904422	11.58080481	46.11557328	32.10782509	31.0678614
87.31696538	37.43868074	151.1558587	19.70126268	19.82867885	119.4894595	28.55311432	27.79492384
245.6183628	108.7360984	333.8986534	61.41532142	36.61725043	256.0146411	145.9026626	85.63689337
55.11112657	17.79571774	79.47770998	7.272170671	12.84620636	89.31838676	26.38557782	23.88808582
50.5786488	13.06993925	73.34666743	5.157666704	10.66616339	98.5671253	22.45854813	13.33124792
37.30541435	19.36760683	76.11565024	6.549947185	11.97724588	49.55355761	29.64946583	20.42506575
85.28945749	33.63360542	150.4124033	14.72253043	18.34404617	117.3117178	48.55777412	28.10704232
58.58519808	36.67559539	179.6950969	13.87031335	21.32024117	70.95984293	91.82898346	37.7904543
44.13807376	34.89019416	107.0830976	16.15983829	16.19235909	62.0720985	32.5226081	26.0470823
98.61532463	26.19049224	145.5839739	19.18754895	20.9460989	131.6189302	55.0484587	47.24336785
133.5255466	63.40121065	184.7005658	22.21659768	27.06216932	172.7665147	45.69249781	35.88710459
48.91533737	19.01928177	85.41148298	6.170342205	12.24368981	66.95879976	27.49601305	13.78031927
54.71618487	12.46345982	77.57548643	6.520922688	11.88253782	84.30224606	21.33334806	14.63527449
82.9004532	43.90751183	164.3705283	15.67545091	21.37721787	124.1718327	58.37130169	27.13206415
88.58656105	36.93290838	130.7261081	20.91727884	19.24405459	128.9412711	50.7373813	29.15672692
51.39204954	24.5892287	85.54338166	11.77702041	14.13148454	65.69033963	87.25009963	22.38567106
53.73110514	37.36446211	99.62485786	16.15728515	14.92837096	76.29828686	29.69346134	18.90779783
117.5356096	36.15722615	176.2979607	17.33073769	21.79770381	133.6805651	63.12608677	47.78345647
98.97301772	12.72144715	71.00205138	4.595271187	14.27592332	116.1992792	29.07976143	21.41341696
68.33781427	33.23906854	123.0322856	19.48116956	19.12424407	74.71362699	72.13147693	35.00351912
48.54106724	15.67686844	82.79441857	6.211667136	12.74545507	64.74373831	29.14599969	16.99668325
72.1800754	34.37627557	174.2410498	16.51116367	19.28605512	107.1929956	47.03320015	25.11778503
57.85989917	19.15186823	100.3699064	6.516806771	13.1383165	96.39218996	22.75375314	19.18813492
59.67256966	22.30794511	96.56357881	9.827686326	13.15642046	87.15974373	30.65057036	16.6265865
67.79624055	16.54182032	97.2819466	9.542273576	15.159316	95.98126251	29.76928342	21.40498661
60.27705778	31.51470721	95.70582157	13.77564315	13.82247982	63.40531088	38.50694193	19.91943456
120.9953549	18.7456064	117.5285858	5.541197738	18.00342432	165.3045602	35.87323997	24.80189898
83.77093986	31.40044847	128.1568414	12.42513869	16.12338016	120.1832993	44.90080291	26.64378884
71.01471398	22.4285318	85.10427043	17.56548633	12.41423891	91.2440796	47.02230184	32.38995287
73.21785278	40.37176206	172.6627174	15.66037029	20.25451371	78.905124	55.8160107	47.26592538
88.37052742	29.06037351	131.1051766	10.61701688	17.88185854	155.3898834	34.89480145	19.56720815
24.69923658	16.26117501	71.45999903	5.521900239	11.26775693	31.41335681	52.69647432	28.61689494
51.89115744	26.39053151	123.4569046	10.05635049	15.6209284	81.82605955	34.19772356	18.89175834
43.54755283	29.75081052	133.2424636	14.86243595	17.460442	64.1366079	44.40393505	55.6355278
71.96303734	22.88273746	113.3779735	10.7914664	17.29811815	113.7365149	33.42270343	23.97359428

90.03361108	42.39162236	148.9670593	16.22846838	17.42986231	129.7401679	49.42346813	23.03219639
85.64912596	35.66304322	127.3499487	17.65956022	18.50445983	130.6823398	35.67579542	16.39720984
41.96060572	23.41293	74.34439732	11.86646021	9.920887067	61.9011829	24.7803031	17.26564674
114.1582838	57.00364566	209.7617812	32.24374736	28.54401305	106.5792132	81.46155701	51.02080973
76.19279427	42.60514831	143.6784054	18.21949083	18.47855898	111.7564277	37.16836145	22.61351678
77.90919208	19.40703803	88.94949412	9.214491737	13.77102225	93.85416913	29.43795019	15.51342131
179.8968524	33.49394029	146.2052761	15.52814434	24.06022343	188.107021	42.33956093	29.46059725
64.0411887	29.46877422	123.7440492	10.75875069	15.4045385	78.07575891	37.34706712	22.14401983
121.7873972	41.14677818	161.7270654	22.83789802	21.21623392	156.7563773	46.84123313	39.25945155
65.65404497	24.57296262	99.18040562	10.5192329	14.8730422	101.7667567	40.99807255	21.94355823
60.16462056	24.62109396	115.4143166	15.04730559	12.06171247	78.07220987	47.88233893	23.47498912
64.81090569	34.18245059	152.2829173	32.96959604	18.20914914	87.39424231	47.51558507	44.8904449
148.048041	27.84491948	147.6788089	13.52946247	24.00036535	245.0249449	43.45197074	30.23679015
57.55804569	14.7356263	68.34092344	5.943871685	11.87051191	82.91901484	33.75813452	16.45200355
56.38025971	23.86617719	106.1895245	9.035020145	15.48993193	75.04710033	68.78778349	40.85870693
80.80954868	33.4318914	139.2299927	14.7551015	17.37306107	87.47788104	46.43905645	25.43456715
103.8709204	30.05320083	136.3879081	9.200168968	18.29759338	138.2016718	34.61326669	20.49495007
56.59757212	34.05913274	109.4090425	19.60008915	14.99846566	68.35686341	34.07280806	30.37553814
54.65361763	21.36949902	87.10537994	9.406659983	12.45486303	67.42998378	28.4506639	30.67530353
102.0555507	37.78940475	157.9821358	17.45915401	21.393719	140.4103253	47.90251016	18.64593555
53.11258527	41.48494616	140.2165358	25.67394001	21.21132918	76.71178665	57.57273118	44.37079451
59.50214743	21.26831599	122.6904105	9.254035296	15.08254925	70.32703363	31.06366443	19.68719926
64.72793711	23.89903219	123.8169119	10.28018727	15.0076501	78.03922367	37.33049507	21.78226202
123.9295999	27.83394524	120.5886628	10.99296205	21.58309239	92.23785483	36.22417194	29.90455862
61.58311154	24.12498259	94.98551093	11.80075143	12.61366383	86.30655552	54.77890999	26.19496461
127.6959838	22.46887016	97.22177828	11.15471694	18.2648133	123.4373028	32.82327224	27.51449543
151.0047801	58.7576228	234.3095549	21.66733158	28.8976908	233.2592446	65.48831306	35.08717312
65.39784868	15.40027197	93.39894154	7.597377995	13.78010908	100.3657321	24.76568932	19.46288305
68.71279451	19.51499504	100.9751508	7.215350921	15.1580808	115.3368082	28.01139618	16.917775
83.64864203	38.269723	129.6722946	27.79557507	16.51141984	127.4370465	62.5700831	38.31747243
144.3007693	27.03173234	141.0407577	13.120168	22.27774986	135.3701422	74.15733826	39.30600909
118.0652633	46.33337652	126.1989669	17.62073155	18.79412515	144.997422	32.24264999	22.69743049
66.180942	29.42983658	93.00513536	11.23314783	14.48119659	83.95982387	63.15099346	20.21750175
71.84010241	24.38786599	99.58711466	11.46404448	15.54823254	86.88769864	49.20201445	25.50444437
82.49392817	29.64300168	138.8294942	12.23874637	18.97321037	112.6278839	36.08409748	24.41078158
103.337636	42.52022729	163.6380794	16.77344599	22.04487942	131.4403522	47.18591268	23.37907773

108.2339938	39.61774591	182.7233366	15.63721693	25.35504843	132.8238832	35.06673179	21.12084435
116.5436339	28.52712136	103.8290482	11.7909211	17.68097809	134.4306139	38.15681385	28.02184182
74.44473213	44.33293658	147.5163388	15.52503456	20.12600622	100.6376335	34.9869649	27.64744816
72.89052326	24.24070611	124.0958151	8.895189755	16.76728326	131.7197097	32.86166029	16.77126356
191.727715	112.205674	419.8136867	49.83121491	48.70820727	244.7632573	98.35833468	73.42417218
50.55622093	31.23831269	113.9714868	20.35448396	16.43565187	61.08479426	57.76807054	49.40922917
64.46229398	40.21816933	106.4717339	18.81304311	19.30722237	78.18628489	65.39379623	33.47342022
74.09562813	22.00253938	103.3141556	7.851611219	14.73347498	147.1162757	30.36188514	18.06404326
67.61801797	33.65930445	154.0025253	11.81582603	16.53501207	114.1204937	34.83480109	12.23828599
60.65254745	38.56156297	105.2232882	23.44158844	17.21487727	74.69971762	51.54219592	38.23869093
101.878583	56.20183455	210.8644157	34.38278369	28.49424593	175.1721708	57.41306218	39.72960221
69.02106423	24.48967892	131.7712558	9.354512462	18.50351764	98.80463629	49.79096068	24.52677665
72.42558386	41.96286726	150.5220516	16.39119105	19.55690607	104.5644713	32.25997398	22.47669898
80.29718744	52.79028578	134.6873781	25.9812159	18.29399847	99.95852933	89.60322061	37.50375268
56.76334654	18.43867734	88.09305217	8.717490686	11.35576305	75.88996738	31.17381381	15.13981773
65.89626744	62.59659728	169.0222624	29.03722282	21.02077243	72.83962927	78.19571696	50.706995
32.81445482	13.81100732	68.3090385	9.42324692	12.53143938	33.99514859	40.12418686	29.22540354
73.41680316	47.22533373	186.306202	22.30814002	23.39188358	87.36710117	69.91008155	45.26865644
76.30929278	26.98346283	111.0209471	13.46601506	15.58765875	111.776635	38.02225806	24.03794712
54.07726663	22.98876463	88.65168396	10.68320807	14.05249652	69.36440156	35.89093642	19.25182727
60.20043755	35.82112404	152.1886291	10.30061712	17.72303396	67.6574339	40.85945207	41.13891876
72.51849798	24.4498026	89.75932328	9.761821887	15.26064304	94.45880311	28.779228	20.71801613
71.28416894	23.10769354	102.2712355	10.67090505	16.51906933	113.168657	50.24780201	20.40859855
61.9293061	20.70522802	117.7211646	10.61086766	13.29629722	82.58686873	25.21416573	21.67837138
53.66475889	36.38870332	99.6726477	17.41876573	15.50042214	83.79506751	44.89720492	27.47182403
67.01283986	43.72564657	131.3993952	28.48943118	18.57670416	82.4318553	53.26189987	32.72982837
64.55608949	26.43599637	100.8742926	10.17898516	15.28807649	83.24980043	31.69378113	30.04140718
107.9762019	20.05664989	80.28532653	7.356982941	16.41382015	105.2031806	18.99511393	21.46411381
54.30921994	18.26741527	80.16451421	8.761151046	12.29205562	60.25963791	26.79075017	18.03635631
62.90191204	14.23617951	77.64084047	3.78107109	14.11236236	70.16016533	24.34994194	19.492904
46.94272369	20.6414143	81.97622203	7.2458464	12.13353374	69.12509211	29.61716468	14.50510955
44.96224408	27.10927044	114.6938747	6.874789957	16.63220997	54.09821489	35.88616447	25.41962969
68.46959759	16.00476955	100.510146	5.084967197	14.45672821	109.4586333	35.98748893	17.85708016
91.40844755	29.58005686	158.8001084	11.31183418	28.40081132	121.8055077	77.37697202	65.06840597
106.9929253	17.2069662	84.66477954	6.268216248	15.53575546	158.6796418	25.87509016	15.97281443
75.28407274	24.13268011	114.0928357	9.310719889	15.33087633	87.74660202	33.65948107	28.92889762

42.14484803	18.84873975	78.1684851	8.51164777	10.95777528	56.00215207	26.35289887	13.41490025
74.99784879	34.55578935	120.0248563	18.12126165	20.78177371	95.52791572	98.13766675	53.73269843
59.38476081	20.44329588	103.6840685	10.75256737	13.08771062	101.988363	29.99386767	19.11033387
75.40769895	31.44522409	135.4485482	19.37814057	18.39366263	76.84306085	40.48868319	36.75013419
64.58493218	37.46874308	146.3050876	15.24678769	16.50674364	83.11048494	38.26806243	17.78394714
104.6180793	58.5398731	205.3707368	35.04085907	23.07652769	134.4267318	56.8970838	50.73006234
84.04941002	47.22875351	167.8018602	32.40424283	21.92005989	119.7380088	62.75629474	32.39547396
52.54669782	46.31203924	171.1956625	22.10756169	21.16125949	63.04464446	64.51239155	33.88618453
42.04440884	20.01495263	93.34214553	8.984649542	12.44303773	67.59663006	30.31714211	17.34722955
55.89476614	26.79461697	117.9757528	9.158500182	15.87351893	95.78997089	32.92823262	18.40012309
68.72515597	16.80384906	98.70712048	6.771476239	13.74765114	84.40098586	32.50647557	19.58583153
90.64130331	28.30527294	94.82219801	13.91798864	14.88592707	121.3519177	33.80164004	21.05984485
48.53318144	32.64846369	129.6883021	12.65829224	16.62467062	73.116628	44.19568751	34.65282416
76.96420269	27.76468199	134.7644433	10.53460009	18.24341087	132.0299476	45.73339072	21.14832438
39.5587879	28.67895766	84.99910807	19.56351542	13.98680686	42.69575916	56.26479687	16.40017428
61.65979333	13.04461729	75.22639258	7.103548115	12.47769782	104.4362249	25.29656808	21.7697536
73.24687866	26.43044459	115.0263947	8.287921575	13.76862934	102.8287926	29.54408566	19.10911407
56.34232827	50.9667611	190.2265725	21.63399777	15.63598745	68.48575817	43.13565922	21.3674565
55.96178948	16.76180628	81.39576581	7.613268065	11.57523271	85.06837845	23.98521107	16.62812468
39.68336113	16.9836271	76.68315965	4.663408917	11.62882897	61.1578176	23.83808963	20.92308968
94.03234955	40.53227416	150.4915851	17.71665265	19.58733685	130.9412285	33.74225269	24.47101123
105.9110454	66.84475484	202.7993799	30.7611071	21.53310763	124.0778365	59.72473258	52.88104855
49.00289583	65.64326014	184.4509989	31.28379435	15.92602852	81.13432332	44.02381702	28.57742906
70.00511419	16.88517138	80.23755669	9.064821493	12.61461187	92.19841839	38.77460743	24.8743776
114.4704422	61.73703143	238.3401339	36.76791935	27.87922849	134.8587359	76.57084007	67.82393489
71.74093541	22.76746646	122.3168421	9.395211344	17.21762735	90.4054363	60.69255866	31.61292032
58.98061965	37.72677932	230.7227718	22.0759757	22.52433541	71.11708877	84.76191399	58.68115372
21.90951487	6.271497523	51.06830848	4.817513719	11.09018854	23.2241401	63.33458779	31.62671367
35.74522492	13.90097176	78.08084186	5.610385109	10.59069859	53.15456611	24.89463527	12.32986981
66.51103751	25.74277433	129.2984307	12.4081847	13.11664143	72.00609183	38.35260771	25.09688503
80.97095455	13.43401531	66.50909299	5.119637118	13.24438029	100.1415595	25.06841221	16.44546687
62.75710534	21.8545623	122.5529643	9.243382008	15.55525227	91.32397825	34.26769091	17.53801798
78.83778855	39.18546682	129.5226413	18.12885275	19.56560537	113.9898771	36.37639639	33.90971325
47.54996863	22.20192957	100.9228219	11.38314061	12.09141462	59.94280026	29.07013347	17.90601381
89.65693252	40.41451419	166.5261793	16.63096387	20.49752553	104.8786573	55.56682928	32.81890946
48.86782606	34.5865309	101.3635814	31.91763953	21.25561171	65.17762408	52.92473717	44.03583388

97.39120948	30.52132106	125.3951227	12.07624268	19.59575524	157.1747029	33.29285369	20.23981234
113.1167906	30.71792377	138.5549772	12.7480121	19.22764336	162.575332	29.66777482	24.95425993
53.15970525	22.95069369	109.5457569	7.819148022	14.94526188	67.67891812	45.97556459	18.82379944
103.4648539	43.53930589	238.7360629	27.09972019	24.30467576	111.6853265	67.88900964	43.36238036
42.73068483	12.25739927	90.63625223	4.325928258	11.00275291	49.77055042	35.35137982	19.54837619
66.08113401	13.12054502	63.67078197	3.81252756	11.17689192	129.0584992	19.89199792	12.00870873
100.9834244	12.79648632	77.45071266	4.811220396	14.82851249	118.493574	33.83917823	27.12773255
83.54978761	32.54369142	96.05300642	14.23104665	14.47808778	117.1229399	41.52411746	35.14714111
58.2139229	24.5399397	137.0557101	9.606602623	17.33717937	68.55303318	39.38893049	27.49052863
66.27646953	26.10290265	135.9275514	12.99449449	15.4331592	89.98071053	37.63106356	27.95799882
156.0369263	30.000526	138.7987999	12.31561253	19.38459515	187.0367223	45.63625223	37.8919487
100.685544	38.39308286	169.7950685	21.41341597	23.2722574	110.59522	40.53882505	35.10550554
85.74652476	34.61977092	148.3339041	12.86496181	19.85533241	93.08463249	57.83092443	34.08791111
76.84028784	47.44911961	162.2167999	25.3675704	16.6012955	87.65825434	49.11730239	25.16351749
33.87658999	16.50330424	57.4114231	8.084021185	9.9280602	54.52143514	33.26596192	10.49397915
95.04555933	31.47074502	115.5780611	15.07233188	17.11964658	128.6314403	41.89908624	24.71559235
61.94752522	22.84250138	96.13354335	8.203239651	12.42119665	80.60086717	32.84627676	18.99544511
55.87021697	30.53344223	120.8619083	13.40739796	16.39088645	70.07727235	42.96586984	34.27677213
93.7157231	35.98947423	113.213853	16.76943224	16.65011274	126.8861512	29.00338933	21.26746174
65.5883008	41.69074124	151.4151084	22.04689581	18.06211367	81.85107869	54.87003932	32.73290545
54.14201403	34.61607395	138.2062359	11.9140758	16.13732332	70.02194163	49.28413092	19.14542612
66.32850003	33.75166145	102.9496998	12.24349149	13.2431335	91.72001305	39.76136522	21.96383586
123.2311887	18.24472549	111.8153007	6.849196084	18.09957565	123.920944	41.49340771	32.1827198
78.431896	43.06678121	151.3594292	23.102848	17.12940706	112.0679951	49.22915123	28.41890757
59.37594991	18.17705284	77.99933923	8.772761806	12.97147254	83.36384179	35.07278371	21.9713221
160.8570222	54.42594595	174.7768753	27.57977097	26.28358525	216.8886503	55.85353107	41.84278726
55.34893429	29.16805864	128.1265448	12.03261477	13.47104152	81.32128318	44.06380933	21.5185789
97.81624655	29.75183912	107.8134218	12.27948932	19.47075264	145.8583457	34.25445769	25.39255573
44.07892236	25.66018455	94.87773175	10.706407	12.1278713	70.60851902	26.6755177	17.68045676
75.83861303	18.11329467	93.78440838	7.089258196	14.59773801	95.98568915	28.995575	19.00192523
79.48852506	25.51302678	133.4251588	10.4989831	15.95871879	65.8756215	72.57026033	41.79205935
47.02817612	42.32578008	95.78296834	20.64459119	14.65500051	48.55953962	39.08589998	27.12437872
44.15085725	17.98160834	84.8541298	5.932410019	11.67526584	67.23322781	28.71361167	12.97891154
58.2744169	21.45982983	91.68713666	7.64203062	12.33786227	80.36789385	33.41350811	17.93247313
65.27199297	13.31272346	70.38004901	6.82145279	13.18994603	57.95586979	28.68916319	28.30669171
78.21590219	54.08980732	186.6391002	20.17543452	20.48824624	109.0767935	52.66278986	30.73432051

60.82446223	17.5300492	84.80456127	7.305377182	12.82988819	96.98374541	22.44805688	15.98845158
90.13327068	48.01262666	157.5227893	32.97302656	19.28656511	104.3140131	39.90350695	28.60556486
80.79557697	23.60135007	145.5509024	11.75936398	17.67772665	78.00160238	44.70959959	28.33528133
88.51276702	37.12552956	186.612039	15.43241418	21.56632291	102.1071296	52.60281632	30.46472753
74.35950939	20.63663782	101.1474483	8.82823729	15.00762769	97.38097406	29.89797147	16.21580794
76.56308204	42.56986671	169.6466806	18.06534278	18.61712575	130.4829926	44.21752671	23.92121286
64.95440591	15.19609345	79.25051372	6.045006007	12.29702968	80.96948631	30.56648042	19.47367883
76.10611013	30.51666324	107.1031478	13.63432485	16.4037863	121.9281174	30.73735773	17.54056196
73.45177332	46.44024067	188.6415001	21.9466396	24.10371809	91.70617028	64.00568142	55.15508518
134.2535784	96.54125432	324.6760058	73.252699	39.31719529	146.0940614	93.81017339	69.23496417
59.63040049	21.08458628	91.14231551	9.901144706	13.51338347	72.18396853	30.56324563	20.33843284
94.84543737	35.84720858	138.6355585	13.84327654	20.12059031	135.5889716	31.32101658	20.4182349
39.85942487	34.53260403	87.62763275	16.43352174	13.9076281	42.93768635	108.1151466	26.54204015
97.01146218	66.20081772	211.678432	41.70429354	23.80699114	143.8172289	53.99594034	48.34097328
50.13365194	32.58247061	136.0978261	12.5936072	18.38098417	80.06633866	44.74956677	17.49607587
69.26985153	10.01034901	57.28272068	3.719087561	10.6432676	67.41087615	20.14754542	14.12978272
66.06113361	21.8584476	91.89154523	8.388062921	13.99518199	103.1896333	32.0728281	21.62195926
53.20945927	33.21643144	132.9110501	12.83794777	17.68230732	66.9660748	32.34509264	27.62725861
64.98585644	55.15187825	126.0556972	37.01658157	21.49412927	74.22555077	53.17578996	37.61506637
109.1278223	33.14673438	159.6062454	12.07521753	18.97510798	131.3020465	64.62528747	35.04006484
93.93241462	19.98868142	106.6323911	8.488410257	18.86075079	113.0208534	32.48916947	27.59476743
58.60961	24.78481807	102.9525608	10.66266529	14.2485041	77.78033164	31.01584684	24.86267726
78.9030702	9.934536967	76.31713645	4.143982828	16.30980581	101.8998636	28.65195963	30.58805692
68.00707212	22.61093443	102.1783241	9.106254975	15.44635299	66.89398058	40.62101978	27.15309075
59.03187207	11.5402355	71.34726588	4.800360158	11.28885859	63.92762128	25.6668912	21.4835299
102.9189623	37.59950766	188.59404	19.12334107	21.08780107	109.3903877	49.82429398	36.27609061
63.68985283	25.13365078	123.2257759	10.65092457	15.50929131	71.3150221	47.00463548	33.54539705
107.447752	32.77281021	142.7612931	14.72835616	20.88682584	127.5653646	36.06496308	29.61021693
65.86594536	25.6538154	99.6996148	13.09728923	15.19894459	106.8409692	35.31224114	24.89346562
57.79752215	24.43024578	110.8233029	9.167096567	15.23044154	101.9995331	32.32026205	16.39910711
163.0407949	41.14987434	202.863623	21.79498239	25.81115409	137.8581275	64.78774887	54.18365515
76.12773335	28.51382853	123.2990621	9.604365816	18.28056148	94.74032139	27.79746348	27.13946792
115.6744439	33.11899357	154.069267	16.14699818	22.37626865	151.3905279	49.2282411	43.10466136
56.03476706	32.63835159	101.6236057	16.84424823	13.12528523	67.2875563	38.60771458	26.69904661
31.83066328	10.55064073	67.09953343	4.582797265	13.76365058	37.47402456	97.39413926	32.09320241
58.43305387	18.18220152	90.38098983	7.029201616	12.26377073	104.0593235	22.26551677	13.11839319

68.2475343	36.68371392	145.2097011	15.43251469	16.72830699	96.54231433	45.79071056	25.25012899
59.37455499	29.72180486	121.430618	13.55998211	13.67370526	81.72367377	25.62813084	16.55843594
97.71769847	19.67200182	102.7514046	9.617788993	15.65685564	132.7980292	32.60543688	23.4026783
80.84240357	40.23851504	206.0822181	14.95291487	25.13987367	105.5462289	49.73678744	25.14451778
52.62242194	26.46894854	68.55868927	13.33907215	12.70476978	83.29796453	35.2205939	14.88496727
45.23832175	26.24735669	121.2723776	10.86227043	12.69880012	56.28017823	37.04932256	18.6102931
127.7117278	20.52464252	107.727123	9.730784952	20.29901234	169.1465275	33.95649097	32.99600917
63.36526612	33.06094226	134.7889442	11.73873317	16.24295904	99.59697962	39.54282176	18.177407
65.53088483	48.40572625	141.1658472	40.29871528	15.02391374	86.92353469	63.71196342	39.19078838
45.84499517	20.27871916	89.9837138	7.979473701	10.9258695	67.79583162	25.70534042	11.78911555
59.29917968	38.83541483	141.4372957	23.30505192	18.64442708	73.4421675	47.55452684	37.46541712
37.46541126	14.52171436	62.53201264	6.640588297	9.843765455	59.6871084	23.10923745	12.9669972
68.64699715	23.68960176	118.2310688	8.896035123	16.81977114	87.57209361	43.87441136	20.24860382
88.84958653	43.7540679	141.8544133	20.83897851	17.5107883	110.1799713	43.70686188	28.60864839
61.04027938	23.1150189	121.5632978	9.278180686	15.34224678	55.81121719	41.70927387	44.29393955
37.2673128	22.35381842	105.0608698	9.398926709	16.00546463	37.01152867	46.41007515	23.86077722
67.51831907	19.7424273	86.00255651	8.970330148	13.34027139	108.4566784	28.53354041	17.0003881
63.06503823	24.20421755	100.1322018	13.66751769	13.30684238	104.5354029	39.20284781	22.75343111
120.1753445	79.37210161	244.5426934	37.16921787	25.81950054	112.8891599	74.74999566	73.12120014
64.24539288	25.60981308	121.0815471	11.91185087	15.86532127	98.00084576	32.15204178	23.6092694
53.48164825	16.84125614	70.13626914	7.3151178	12.88054692	70.28756514	49.107924	20.72559859
58.9595561	17.69995889	78.36483137	6.90367182	14.54761447	79.69057658	28.41595968	22.51023058
102.632146	19.89764677	121.8883564	8.677198922	14.7762404	135.8393219	53.0541749	28.2895446
53.0110949	32.48525249	129.7932269	11.40966766	17.84493172	81.16304621	38.56126102	22.22927409
85.23635874	43.65460319	183.487387	14.43706559	22.75847979	133.6991455	54.44587038	32.31395726
41.99008972	19.99172778	81.96068617	14.37939101	12.84704164	51.6384024	44.28813814	33.1050424
63.82792599	20.14961995	107.9248951	8.401480561	13.63169675	109.0076471	38.50950403	15.51546005
67.70316158	31.22499596	160.4732317	11.02378036	17.78305859	86.28795505	42.96494884	20.42197039
39.16625462	47.3184642	100.3915334	41.60911711	15.22414511	53.89772447	66.53064886	30.45491408
50.40789057	24.25812954	93.59241701	10.20291046	14.10821794	71.25731386	30.98632679	12.13332937
58.97483397	22.08022144	102.5419888	9.308012806	15.71239627	80.94878923	37.82929841	23.24282942
117.9510376	34.00843163	176.7222536	12.57475185	24.12378506	181.154682	47.02901723	36.54086033
51.06623964	35.18827596	132.8470415	12.86737844	16.46722444	48.55123828	50.19648062	28.08688343
90.2013493	20.981404	100.2204162	10.63988485	18.45213338	103.5407534	50.1720386	39.33046276
50.54033949	24.23811271	93.557288	13.20481476	12.64221793	74.74074227	37.92336869	23.25188063
87.9505134	11.45821811	94.53131203	4.825922844	16.01512037	110.6783819	36.43626629	35.46038676

45.607929	14.90857822	80.44919771	6.51590995	11.95750477	58.25421369	32.39603549	19.37236515
69.56851791	29.43966757	157.7256796	10.17288702	19.19123644	79.47547944	41.44302239	28.40911249
54.90264219	29.17250666	133.753752	12.02323166	14.66652986	74.09693894	38.67576564	18.20182007
66.36776509	25.87542389	126.5452404	8.691729964	15.03388981	92.24304069	31.52988464	16.79791765
53.94903678	26.09393745	136.4538588	15.38525141	15.35156915	73.09960139	50.72205988	36.39184699
64.53467613	20.018851	119.2677528	13.94648849	14.8421091	101.4821981	38.44201777	35.68354957
90.30498979	42.53202001	186.4045875	16.96503709	21.29654675	104.2465544	42.7096375	32.63985045
147.0870588	52.71109343	178.5907523	23.11905964	26.87465783	169.4834645	45.81220581	33.41827546
67.81153505	23.81948379	127.5573893	10.97401231	17.70781839	95.56888637	35.43689076	27.37510174
45.53753613	21.9611024	85.62338934	8.337110386	12.74267862	60.20395085	32.30191458	19.11438028
84.2271762	77.3217129	190.5909183	44.37598221	24.39745818	100.0151842	62.40605585	62.18808879
50.89286106	25.45292836	110.8392663	11.7150658	17.54386205	72.06796175	86.74783659	32.92044957
87.56830463	28.2719178	124.1713011	12.84855432	17.8859438	95.46068167	46.18631109	35.07920771
87.21630904	24.84263494	130.7210198	8.758716159	19.774527	91.9986161	53.29142928	26.20152056
66.90299876	23.46421304	73.1447215	9.882890704	14.80015724	56.8954905	46.43397835	22.2141959
79.72012907	21.63875422	102.4835019	7.193028522	16.14848878	96.35737594	31.05353816	18.46658566
133.2652757	18.82164349	101.1313573	11.03479425	21.29793119	147.0828059	40.46975873	36.28838419
58.55780043	19.18713622	112.0621337	7.196950112	14.30214266	85.73747736	40.39159946	18.99482738
44.19613029	13.08534478	60.83673808	4.894311508	9.471691534	75.01477237	15.97629572	9.459993126
38.06926448	7.362090163	39.32753427	2.292065247	7.911420879	51.35755166	17.85964856	10.93624977
52.06187525	34.07715809	107.9916851	20.43304038	18.6501151	43.20426943	50.24403736	50.49824122
80.99718422	40.92349932	168.3271063	28.69300649	18.78562479	85.35480737	46.97517112	61.42838308
43.43565334	24.05108933	76.94325881	13.43051426	15.19536531	46.51935032	62.82582717	24.8445633
124.756123	41.83923809	136.4963674	13.41972733	19.87365927	125.7196494	38.10971802	23.84092765
60.93026461	29.59717292	141.6190084	17.45106868	21.47712273	62.13279012	48.41146572	34.00908785
53.88235569	10.19600299	53.43378151	2.991602959	10.25366214	78.23063962	15.76457516	10.48267833
78.77269585	37.15496634	174.4718814	14.89026282	20.81262206	88.3774127	48.59719465	38.90594921
52.08844666	20.93576121	99.82152655	6.914043143	14.07886372	85.8558592	32.5539454	15.20724921
51.69654094	17.97755418	104.3965893	6.982297566	13.61493689	72.43560911	41.25868775	16.96289216
57.68542557	11.61741765	83.65777188	4.264443553	13.29848985	65.74677154	27.08977755	15.79275009
77.16895138	20.89683307	122.8930328	10.25827149	19.52150288	83.06549364	71.66666253	35.9783525
98.99221668	37.727862	182.3112564	14.83029202	20.61172126	181.6924929	47.89117369	25.78619257
61.09576044	28.40661871	135.1074034	11.41348266	16.98376492	93.5001775	43.47965369	31.39602185
71.7300313	23.62511955	145.0590763	14.54081756	17.95006195	82.91736509	47.79178066	50.16412191
85.75069517	21.07813384	99.5353206	9.188744126	13.97420267	119.6086038	41.51162278	21.80646765
48.02062698	24.67683275	102.3577864	8.289412459	12.44369885	91.43956352	24.41475602	15.92629026

99.97155086	36.58909922	110.9849569	17.1728429	17.57101494	158.03316	41.60504484	24.16441867
54.57640613	16.15985866	82.10422934	5.627870611	12.8656297	68.13175753	31.51283446	17.39756386
113.7239441	37.44455238	158.1000518	18.70943585	23.52107169	123.3358261	45.92614745	30.93929065
94.4581098	59.05379428	204.6901541	22.73797396	23.9270673	96.97905778	77.04641233	48.23765425
115.6461063	31.55765426	142.1015178	11.07790867	21.05394595	132.4842318	47.68997189	28.43295871
94.91170537	27.99049166	136.4116234	13.39903326	22.06906153	113.7437571	48.6899678	33.45938121
71.41174204	37.3438575	131.5407118	13.58680391	16.59426391	89.29459519	38.74897075	23.900758
88.57090629	22.68690111	147.4016054	6.946138243	19.46670433	100.3435645	60.56747523	28.92548225
58.27810464	27.76347494	117.1221541	10.02016637	16.66909199	74.59251498	35.40635633	20.52379831
132.9703719	52.55605269	268.4510682	21.38066746	24.85926493	144.4180316	69.85898113	38.15354157
49.64855289	25.57149256	114.5404154	9.705534384	17.20751135	67.6210756	34.5577796	19.6730692
84.05561003	29.55547861	141.2134563	13.60354119	18.87883705	98.26465048	43.515505	32.0364161
99.9683763	17.31187249	84.73451368	5.909456587	17.29320249	140.8764232	29.48719373	22.80945968
106.2855405	29.89713807	167.5418076	15.2177941	22.89927663	157.3831848	45.15748176	37.63080618
101.5468934	38.30439262	103.7839642	14.90585227	17.53705028	134.5467163	43.73093826	31.64962083
56.34202374	36.58704736	150.5279499	27.20083746	17.37301607	89.82595485	44.18689282	36.25423284
74.36112224	26.07126807	139.9211332	13.18025954	18.88155142	100.818265	31.92878009	23.16297168
60.59259428	22.87727958	89.14573889	9.90667217	13.16343367	95.86414239	30.3954156	15.22736194
87.18115576	24.60319022	111.5550817	11.80516008	16.53400615	98.52092138	33.21431495	28.6591752
53.6614355	35.29059566	104.8620006	13.08162633	17.90155468	77.28243978	62.23637922	18.40770811
47.78875897	17.80931569	75.1156216	6.183384218	11.29567471	82.96847818	27.08409608	14.29542676
61.80731958	18.23249687	86.18560241	8.62199615	12.94875908	79.97041597	33.90299712	19.94698903
68.71608205	36.38736635	96.29104038	14.75909317	17.16276071	99.69322932	39.00406854	26.72918392
98.50006152	14.10388568	88.77821636	5.44132388	16.30527161	106.8234425	28.27243523	19.61353557
57.5753224	20.78807119	92.69195742	8.642507673	13.0743834	102.9442745	28.05411361	16.92532113
71.84518794	25.8386523	117.5320367	9.27040794	16.78986695	82.45219681	48.57521848	24.29675725
40.19823782	13.60827011	74.42031951	5.05668172	10.14686284	70.22459251	20.1952271	10.66506981
41.82204783	15.2193735	78.26591821	5.12882302	10.93027112	63.38446252	26.92055904	10.72046355
62.13372208	24.3641219	84.79855743	8.920880341	14.88428455	80.76213617	34.44959505	26.815589
75.01468937	29.08237614	151.4319589	10.13304291	18.27673153	127.7688374	45.59507193	19.69099161
68.42048943	44.01644406	136.124924	20.10807777	16.83447668	92.14732437	56.91947196	31.32952563
49.46387072	17.2512904	80.47276542	6.793210449	12.00896857	50.8442321	27.65399639	20.50192235
65.3053944	34.59959584	137.3258974	12.70530687	15.1458289	113.477295	35.1848327	17.25509728
97.09231591	30.39936105	177.8497259	9.818285573	24.75841386	176.2554759	55.36824018	39.549173
73.42121293	20.32141986	96.48916372	6.743942243	13.40991452	126.3315352	26.64099541	14.6506057
101.0868939	33.75430774	147.8310392	11.74757275	21.03081988	153.5458097	45.53964615	34.85815181

59.5990695	31.39342749	122.5105522	12.64245545	17.63627196	90.65919896	31.55232368	22.65466567
73.50142051	24.67848898	98.53432919	11.53594499	15.90328321	79.07592334	49.68764632	24.54476443
73.91923716	21.0910468	112.8897966	6.983843613	14.5780862	122.6201977	34.44555145	16.36784193
43.26585725	13.07434192	96.433323	4.282382098	11.74334099	47.58990104	42.86455097	24.21160268
98.52306553	18.6405058	103.4523202	5.27596101	15.63855226	152.6123178	27.7024241	14.33260748
116.2311514	42.35582172	187.4658296	16.30674509	26.95643142	130.0324339	59.16626836	25.60727381
47.53070127	23.22649761	105.0459813	8.568813779	15.941112	77.01752142	35.97483845	18.91356751
64.38998862	35.35640255	90.74984075	15.9976029	18.29583234	49.59907299	62.35448876	25.20152564
77.67418649	95.41518613	208.8766595	46.24881837	30.02300821	60.7539343	131.1244948	40.26553609
46.08586274	21.35656128	82.14408607	7.955986717	12.33849584	54.53290934	24.78716731	18.95224192
60.56164318	44.74655146	136.4740331	19.37881498	18.99509434	69.96353526	53.79414666	28.63535043
102.416976	72.53112923	224.8530218	32.61240875	27.6049828	137.3794713	76.60009279	41.63815663
87.0776904	65.98149154	161.3039992	29.7144813	24.47573804	73.44015423	81.2559804	32.51839504
41.66604474	22.00827661	100.0608572	7.23195008	15.31575099	56.14467613	33.98481233	26.92673334
57.49934193	9.101014389	70.74145582	3.600179538	11.45633885	63.87425442	38.29879204	18.15460573
45.60682819	36.98216054	106.547677	16.57155075	16.73658405	43.57290207	37.3316911	33.07976392
82.44743971	23.45212325	78.24813385	10.57029254	14.76984567	105.1493917	33.68699179	18.0574339
55.04030433	31.60490997	112.3420645	14.34570844	17.3368191	74.32626161	53.18497678	26.85663954
76.4791302	36.37977237	120.2416997	13.62185325	18.64177933	79.97373562	43.0416354	24.69059346
77.59506492	66.039625	244.7939056	31.89375729	24.98013104	89.72289366	66.05301737	37.01403237
54.75916611	17.16697094	65.6879431	5.75963736	12.23339806	81.62646291	31.66811807	15.05852508
48.10260083	10.23004062	52.37254947	4.239951437	9.576883559	73.80668038	29.62793147	13.01160827
78.55818542	41.00191603	128.2104384	14.15133317	18.25568856	113.7297861	58.88237248	19.40944816
58.51019228	23.90370232	99.63490615	8.412451866	13.59468573	80.90077827	34.5556049	16.38023907
60.47600403	36.81712354	102.4474855	18.7918505	17.26362437	52.81178932	42.84103164	29.35699183
79.09031953	22.86365771	89.96526735	9.272355242	16.87485176	86.25501313	29.94453391	28.41462074
66.7912674	77.86621165	222.2944149	36.22999221	26.40419162	61.60177037	121.1418227	63.23800713
52.71677953	18.0748206	77.40170826	5.64002275	14.63851996	67.76737639	30.69358748	21.19057777
57.6245974	34.89861313	130.1926839	12.40612455	18.7580546	77.60238277	44.29863635	22.94387424
63.68124407	21.81872387	76.89074289	12.23936136	12.89612335	84.0681952	48.24115418	27.8210093
81.55236504	32.15022173	129.2121762	12.24435749	17.11831478	123.5048122	45.20273393	27.7102883
52.77799808	31.63147365	91.66147821	17.6064713	18.75475887	35.90433753	104.6344471	40.29963183
38.41252713	18.03383104	69.75962881	7.520037984	11.1168803	44.85174557	30.80514504	18.61493064
43.25694023	18.96633442	91.32514323	8.531129701	14.04473715	65.85422557	27.44079058	17.45810292
54.4599076	69.51045965	179.9430502	50.73175256	24.46930038	48.52473694	94.98202497	66.55072982
62.98628945	28.80585733	128.5322817	9.119712921	18.17249392	79.14673136	41.00610618	21.54835912

47.16136797	28.80852515	108.9337149	14.21980843	13.71575929	62.99657504	35.06429442	28.98242841
39.20244642	10.14679472	61.96207735	3.599907613	9.577213816	58.66343434	24.68080077	11.83050348
55.83329688	20.10353305	67.61565358	9.079622533	13.21634616	56.56803944	47.0088768	24.54582175
48.53335897	29.03708245	86.59693636	9.404024938	15.34934417	36.20480101	54.51971861	25.33172614
43.80154304	29.23782169	124.587968	15.61472839	17.42797854	52.39572133	42.87611525	33.2531141
65.75773766	52.64611094	180.7473222	23.30368301	22.92236256	109.784383	54.49689901	35.55875755
61.72184149	24.52726081	91.92848147	7.931093093	14.61522849	78.23965306	38.52595081	28.24853845
54.90011605	48.35762251	146.1525583	17.47397928	17.81085941	73.39048616	38.31489275	33.10970893
55.48861522	23.47461902	90.37307619	9.989171045	13.46582899	109.9918069	37.15532567	23.62821676
67.47683437	47.48378777	151.2732043	22.57440914	19.99141312	82.19551241	46.11013663	35.88553068
59.02613824	26.3747089	125.3526462	10.03838273	20.77461383	68.5869642	53.42850424	34.59664079
43.56382158	25.94513103	92.01011803	8.494219832	14.74162983	42.54034121	29.77724553	21.99062359
52.44587482	39.32687273	137.421099	11.02008664	18.10954133	63.5461372	50.83124464	49.41773947
75.49181352	39.8332227	115.7637234	24.1787163	18.89622801	67.86243131	74.72253953	32.75817961
49.38678679	42.94305174	120.582508	18.80496043	18.06060951	50.85316895	54.0040861	45.11560408
60.71632112	27.21492495	89.95840401	8.742273214	17.58754731	54.98018422	30.43649098	16.44072308
50.39874015	30.82944341	105.8434288	10.91061576	15.87898825	76.00203291	41.28279831	22.5432961
77.94877063	39.95908368	162.8704021	11.53958315	21.99216013	119.2956798	53.9275429	27.93676553
67.07390605	27.32357263	94.88258872	13.05188748	15.50913104	61.97092329	54.24096485	28.72515369
29.51419509	25.2587125	57.96265517	12.67425267	13.07760593	34.85325804	54.3362661	15.19724312
51.77114246	29.53549493	102.4599583	10.96941212	15.92734811	57.41617296	35.52155436	24.74087482
55.46579271	25.28526774	104.0196251	10.19757617	13.61208205	75.71235578	34.59181761	24.22522102
80.28101059	17.44834045	100.2014238	6.977453925	18.33685973	101.0048133	29.55343874	20.81521166
45.58580113	21.32228794	91.32210622	13.05687837	15.69302213	45.05019513	63.33560633	32.31012092
55.93165884	40.42676757	107.1449712	14.42082898	16.07749631	55.97574412	47.28575702	21.28075629
95.52581075	26.53031829	90.35822293	9.78648284	14.53266418	122.8959489	52.44356705	23.00462979
98.87922192	48.25741975	198.0517326	18.80691268	24.48824953	133.6867769	63.46137905	36.92696681
49.1369025	24.13555507	84.89153399	8.232101203	12.02931775	66.5891139	29.92098124	14.44378851
47.47716348	9.981110945	63.29191583	2.985892492	9.934597192	53.2476157	25.45918355	15.04595825
44.90102574	31.10117097	121.9283083	9.485225114	16.40421138	68.6042708	33.45033405	26.23952154
84.93890111	43.72271502	135.7484284	19.81283622	21.10311677	79.52847617	61.67662821	26.41364764
33.62349504	23.17017047	96.69884591	12.55624539	11.86253662	41.83042771	46.18849746	35.83531948
43.83044563	9.891259583	48.94696345	3.352606894	9.328253117	56.70310779	16.13274394	13.33037658
76.99905811	51.64723102	160.7232237	23.72439132	25.12696923	79.54685265	131.4373388	73.95525166
65.62736804	19.37695161	84.65869269	5.592303733	15.42714744	53.59365966	39.00226284	26.22716614
38.40263172	15.52651339	67.88778809	5.854414457	11.78297496	59.59299224	30.31191938	18.89097767

82.46541671	68.66148071	171.7894635	27.94975431	21.52500932	76.9495082	54.75012951	26.2608885
40.4779188	24.60266021	75.18638589	8.172059997	14.6730255	41.83927164	26.20071055	22.91426996
60.16459927	22.49339365	126.8029622	7.033798321	19.12824356	76.13824963	39.10480298	19.22286744
62.18157401	32.45610256	113.9761	15.01183829	15.54696896	66.59175911	58.63708344	25.19290695
74.89686285	25.49744507	100.8327804	9.253670217	16.85787907	95.12726154	45.78774611	20.5910346
64.79603751	26.57253958	112.5564589	10.96917335	15.62066224	94.93016933	37.56367497	24.75325421
36.38307328	15.63050654	73.83869803	11.75228534	11.21467134	55.42455964	26.92187436	23.22689725
53.02505935	22.13627319	63.54104765	12.22761407	11.69745501	57.82856372	38.24101686	16.63114753
76.16347035	22.18491731	114.5142125	7.426496252	18.55030535	84.43611883	37.30089843	23.83178918
72.49720283	57.74418745	132.8637787	29.46855351	20.67567325	68.59324897	59.48517041	35.08240739
67.11382041	28.75201173	101.1289484	11.63151019	17.84174619	77.54536348	55.56313546	22.87860012
54.91249701	35.98403398	96.5543615	12.6614638	17.64088777	80.36388823	31.75672669	35.45687171
19.50549857	11.78171876	71.01562396	5.22383593	12.48494696	27.55448846	27.86063955	19.89959034
92.51766075	16.86852499	96.80735507	6.98256519	17.70256917	116.4166628	27.64112917	19.35782077
35.65794099	32.49600424	93.14051896	18.05370765	12.87670981	30.83878486	66.99984757	34.61248345
48.79002798	15.73126052	60.95408496	7.246994732	10.56094028	56.70997041	25.36158514	16.31784066
39.49180038	17.31566465	75.9197033	7.662781675	12.70749829	58.3797023	31.82949615	14.68328762
57.17741523	27.82735542	106.3311484	10.89458286	15.73521644	65.08098561	34.20245074	24.52627053
68.28202198	35.65561598	128.2838161	14.22948305	17.34297587	78.98592132	52.5806452	27.31250878
36.38632204	16.39373619	80.51515544	8.070083875	12.73611927	47.04074874	35.84261876	23.064557
76.92973106	41.02216226	130.6266205	14.97226425	21.06881343	83.76129664	42.43277573	31.15400519
56.21382294	43.75987859	124.2123388	17.44314122	18.42797727	85.71302168	39.15720715	20.60894896
66.32185509	36.98851116	136.3511816	11.46556145	19.32910381	74.31078226	31.28291207	26.94601804
62.66325469	25.49711597	100.5689822	9.398358963	15.64498358	53.08121118	40.75737774	33.80812819
73.14506681	18.42394698	112.6394892	7.804653693	16.28310017	85.1164167	40.91274438	39.49883488
70.92207538	24.4805846	93.08297346	9.752055625	17.7576748	73.84251991	42.86643455	24.98142436
71.99311282	34.06503281	140.5503285	10.45271156	19.11802441	104.940357	43.2153994	30.61429521
77.60706519	40.47413267	203.0061007	18.35376171	25.56424102	114.0404468	52.69114724	31.50336766
62.60918567	27.53310175	101.8589071	10.22072608	15.87984611	76.05582193	33.16993742	20.39937176
80.19874213	38.50229325	125.5522302	17.71930008	21.49129629	93.61456362	45.67924136	29.8004938
59.56445255	15.31761182	87.44067277	6.672307827	14.58801785	82.86927982	29.39496255	20.74896951
65.71105937	29.69806834	119.5649708	12.46718473	16.80410685	75.28234702	37.04607693	24.66361301
48.39978581	20.06837383	67.22400945	9.948209347	12.22839616	49.27633352	45.25022856	25.10683118
88.3769404	33.77465753	138.272121	12.12162251	17.766664	111.8542342	53.55069516	26.8487967

Ribociclib_1632	AGI-6780_1634	Picolinici-acid_1635	AZD5153_1706	CDK9_5576_1708	CDK9_5038_1709	Eg5_9814_1712	ERK_2440_1713
50.35950556	65.44704648	237.8552058	5.563131933	0.71604401	0.133865306	0.037880882	33.13555395
48.13329086	74.68669127	139.9037746	3.660520221	0.504672388	0.056088899	0.078336861	10.03011668
51.45492471	61.91991973	243.1027854	5.397447857	1.803879131	0.182193822	0.110983454	48.92914782
52.04650658	57.93737628	118.3380452	8.46138884	0.727607323	0.155256259	0.064087695	10.4597033
59.16762521	147.7373126	329.085969	8.051029089	1.814854317	0.204614223	0.119911887	13.6897266
42.36795197	52.66059433	111.4514865	2.493931324	0.405169865	0.050732944	0.062374898	11.19004063
36.4765859	55.19895591	160.2346649	4.251370713	0.610779941	0.095356342	0.04638714	4.725483544
41.73697071	79.11525511	192.2744943	8.539548649	1.01261166	0.146499702	0.11625923	27.94693422
51.81937048	97.5970303	286.212612	10.67615893	2.386151719	0.312513272	0.157678518	17.43351126
36.72943787	38.27264589	106.4537649	4.207312577	0.434363876	0.053216389	0.03153136	15.10725007
39.41037693	41.11495762	114.2044948	4.332535013	0.380468871	0.060547565	0.066358686	16.70070878
39.06307694	70.90139533	146.5619526	5.360322205	0.562920584	0.065658186	0.070472255	30.52364627
49.1371934	60.95043426	178.9508846	4.151118404	0.771718105	0.075898354	0.024319202	11.84339579
46.12455655	68.46790711	186.1136351	26.1081609	0.688876681	0.112253591	0.254994383	38.95150442
43.53612631	69.89360888	186.6900307	2.568808981	0.966783019	0.116564696	0.061095953	12.50582778
40.34219122	95.82207608	194.847816	5.549746671	0.902768946	0.115810246	0.067686469	19.05775584
60.86266561	36.80610352	115.4639044	5.620529452	0.56703895	0.068101305	0.024620047	16.9332476
47.33648315	75.05024397	266.7740001	6.987071855	0.836876944	0.285973545	0.073216501	5.39886432
44.68566836	105.424426	193.8917618	6.569336149	1.546013818	0.239735468	0.117926871	14.54047881
43.58709522	52.38170212	143.7529496	2.646516569	0.403409704	0.055108691	0.023656859	7.48761239
40.12631758	68.86178982	168.3944599	4.144914604	0.57694229	0.080353167	0.035934704	12.16643348
65.32293716	81.21765435	252.6073932	11.90935068	0.945771273	0.134745635	0.057943729	17.50106144
43.06150979	62.03142393	157.4343457	4.052929502	0.466468661	0.087882882	0.029601498	6.268702671
54.04312634	83.0145383	243.6170482	11.67093773	1.31403746	0.168280822	0.094451535	12.13721215
46.87045318	54.8070841	179.0107187	5.371207499	0.63585888	0.078401913	0.065403865	13.52193052
42.55029333	74.72487719	188.8130273	7.046679011	0.767827525	0.093325427	0.05845833	17.24716551
48.99786976	78.62053829	262.563138	9.880027502	1.455788326	0.263759257	0.120158353	42.97473496
50.01426539	86.89290684	221.4088707	7.116879337	0.939847846	0.132319057	0.051572101	6.533014354
45.85772192	56.01186155	139.6724301	2.712311428	0.507851997	0.066462826	0.027079873	5.913346097
46.83062377	45.13293656	149.1769352	9.385688246	0.669954236	0.075504202	0.024200121	27.4686119
41.76747242	51.4470409	166.3356399	5.706843733	0.703770611	0.104233166	0.032817577	16.46770597
38.83114407	82.61636944	211.5884243	3.793951729	1.418274561	0.209381026	0.025413003	16.4489732
54.05637438	109.820562	299.8825275	5.78237613	1.484429643	0.212369976	0.077800876	17.37819857
40.89395315	36.17266525	135.0831882	7.726270247	0.623335809	0.116819109	0.045901632	32.77440359

47.88951552	84.83767651	243.6874072	5.179034281	0.981263899	0.126609419	0.036979827	9.247250282
50.47477946	101.2630525	232.5112792	8.678281795	1.363808937	0.273982554	0.109148844	11.88315096
48.69298948	63.9456796	125.413757	2.805403779	0.615379663	0.064100217	0.035602913	9.514291445
53.93943087	91.79403049	342.0794116	5.870631044	1.407893655	0.28725171	0.050967309	17.62463619
39.55179102	73.43653412	181.400049	6.827904421	0.725751667	0.136991811	0.052005589	19.88776877
67.43634999	63.32140455	231.7623678	13.52806269	0.878356247	0.092952558	0.058020938	25.23273498
50.20247023	70.82252292	143.0740744	13.4747554	1.112874186	0.135681694	0.08198258	64.04089553
37.86768664	67.91949757	153.6770511	4.896633735	0.778401013	0.091206633	0.116753458	8.375282091
50.08393908	68.32001603	204.1422724	2.593244688	0.460560097	0.062670303	0.034231138	13.92080855
42.64593011	66.21545219	151.6279572	5.027444524	0.657512685	0.084103426	0.057691854	19.97362361
47.95757448	68.32490304	176.0986496	7.356994976	0.543768003	0.083349518	0.062308691	12.17575044
77.66034157	49.08677702	130.3053223	2.413181164	0.488303112	0.046887936	0.012124511	48.86657092
39.76291185	56.72003436	140.5973947	3.144574914	0.514867946	0.078848989	0.043798733	8.519318887
47.17829006	86.34766299	182.1600996	9.0304632	0.674645475	0.099767438	0.052693069	16.19570692
39.70339819	45.14474201	118.2390194	3.256647231	0.356554608	0.046834315	0.039046815	7.986871059
43.1831902	59.77923324	174.7208419	4.687862144	0.507739103	0.051597689	0.018802129	12.14383385
34.93787814	32.50490024	83.24297234	2.306048218	0.297912322	0.037047198	0.024513295	8.247736924
40.07394353	84.42726206	184.5126555	3.743972318	0.979285093	0.159165462	0.075041424	9.86786309
38.57647232	62.3539727	194.8653931	3.313203576	0.739657529	0.096188089	0.052263905	14.32386883
43.18591521	71.31144178	153.7182869	5.771985449	0.826807451	0.111838851	0.025217632	13.349491
39.0598359	38.96613372	137.0270932	6.76017287	0.60566813	0.101236426	0.040887862	22.96354753
47.37812728	66.78006575	201.5940801	5.742782039	0.793753085	0.086237053	0.037560394	12.05351581
41.76457899	49.5748507	153.5128037	4.013557063	0.45307653	0.049399755	0.011330657	15.17814644
36.64206911	59.84452424	120.4376097	3.189112103	0.513076185	0.062674946	0.033232066	12.35684804
57.05653839	62.81999506	258.4599558	6.431109008	0.927842873	0.21805886	0.061101793	24.99019491
62.07086898	77.47114972	251.9984267	8.561666645	0.845898036	0.084911085	0.049858229	13.9816541
49.966056	98.48743109	253.8609152	14.04789358	1.516917552	0.216957876	0.10789111	37.25473272
49.86610392	61.99323618	162.064462	9.055704087	0.541030157	0.08811912	0.063484552	22.82071805
53.59009584	123.0002027	357.5306265	12.36782159	1.941804899	0.216122957	0.090100982	32.20794826
51.54775986	48.50484866	195.7839896	4.883164689	0.406637392	0.043694821	0.032820641	23.23567341
38.00427374	50.14425167	127.5057462	3.204275483	0.503850183	0.063117166	0.038436987	7.125701589
38.39749141	47.09006386	122.1819905	4.172766308	0.43199862	0.048294346	0.033762999	12.33650394
78.04051448	83.99751067	144.1790528	10.81277916	0.831844317	0.136718412	0.047461855	12.82850357
56.37803781	121.3340937	337.6383744	26.36406295	6.515176974	1.069101752	0.35907925	47.54922294
44.72506112	91.74305793	185.8547721	6.890070591	1.015574681	0.168920696	0.044617806	9.411481084
38.07991106	49.94043102	131.5151909	7.915533039	0.607644567	0.071931914	0.018700123	36.40563353

45.93600177	100.2001963	310.0108235	4.904989799	2.223404464	0.29723867	0.050663692	18.20107765
49.81698427	40.92332165	141.7241905	5.173615732	0.452488046	0.062507591	0.026312222	25.64824289
50.12557945	89.75494543	241.3712378	9.999821263	1.032216278	0.159832684	0.076275384	11.22866448
74.16152475	143.0193542	451.8694285	22.79472478	2.363448594	0.459361966	0.374601065	23.46970825
39.1023368	53.28983314	127.1017727	3.87022262	0.578096174	0.080944922	0.049204499	12.97385754
35.91944192	41.20852802	111.3701513	2.509658979	0.413086059	0.048998915	0.042664073	8.45068934
42.96545691	46.35317451	130.320025	2.895738671	0.292092495	0.033020398	0.026424669	21.37782123
47.34546349	77.44347165	178.2667706	5.634351719	0.558021291	0.07150561	0.051810095	12.47001424
60.52460481	99.20332577	296.170979	3.255555732	0.994061222	0.102161009	0.030459804	29.36467187
42.72073939	45.6686713	165.3953881	6.330042673	0.564693619	0.075212873	0.032601747	29.62269368
47.27592598	63.48886603	212.4285435	7.728936354	1.221436848	0.228484226	0.183685294	27.77913444
51.33401492	109.8832882	303.3867768	12.67225369	1.52664606	0.206169635	0.086763246	15.27829791
37.94371155	51.88360616	140.9994791	2.448440558	0.51611588	0.061473633	0.030556444	6.77372184
34.00660711	40.25470614	110.7851748	2.64670785	0.456682784	0.05067581	0.037255404	9.936882888
52.95114804	75.48414922	245.6433494	6.420383018	1.044424218	0.164135306	0.078371037	16.44518865
49.71995411	73.8959634	190.8133262	12.02154239	1.146153287	0.181355822	0.088583585	21.5551584
44.19940973	39.72597436	133.7903728	7.393021143	0.94788817	0.120442264	0.034867562	38.06180204
35.43773294	56.81636635	134.52519	7.643954393	0.456456074	0.067347344	0.030761671	19.38059437
53.6422328	108.313709	279.1502614	10.94233125	1.241354687	0.188752859	0.147378274	17.9659905
45.31297635	77.54891136	145.7965219	2.35527478	0.383218131	0.063706396	0.029985886	5.904230385
42.74922129	74.04782875	167.3168539	5.846870592	0.636015124	0.095404783	0.056470598	20.60459442
36.68088427	55.19964543	126.4702798	2.685786536	0.421766854	0.073583542	0.032108747	5.474863738
44.43857358	61.67196562	193.4096064	4.942037712	0.863211967	0.177131701	0.061236794	9.057034466
42.71481856	54.75967025	146.9493814	3.29014632	0.460527254	0.059512342	0.03157218	8.178854762
41.78440594	72.17761212	141.7277922	3.92125833	0.48998624	0.059925079	0.032672007	9.598575362
35.66762326	48.03100122	127.4288182	5.510914875	0.48296284	0.076918466	0.045244715	9.75588983
42.06035793	57.15901808	137.9852159	5.796321583	0.50822323	0.074419528	0.054765161	15.75379057
53.62879854	85.31973509	205.5208353	3.322441357	0.596869149	0.101370832	0.040409848	3.288635238
47.64600462	67.47203692	160.9950838	7.89750907	1.204386919	0.145831756	0.148476956	22.86035275
40.90682305	51.45970165	143.9541485	9.112661228	0.683498586	0.128519036	0.079856585	32.68989235
55.33505026	86.85692594	220.7745416	10.85056042	1.218445195	0.137157931	0.110952865	25.6062316
43.11786938	93.20045222	208.9703368	5.018085702	1.045073439	0.121370808	0.066073062	13.16030968
60.68634175	27.20567186	91.12693208	2.743569785	0.255544901	0.0314626	0.02611164	29.84637139
40.66998092	66.49941981	145.6277549	4.719310351	0.602869595	0.078334079	0.037439748	11.00473232
71.50855347	50.10208481	126.5341861	3.943683459	0.671500297	0.109347886	0.04879162	29.00483682
39.90913158	95.37666984	191.5981114	4.905122893	1.018736293	0.174880524	0.061223662	12.32275418

42.5731944	67.85118392	182.3008336	6.707721938	0.598131375	0.075681523	0.052751959	17.01162271
40.20060466	80.66830963	186.2954251	10.8801851	0.876497095	0.098284222	0.096574997	16.7608896
36.26137476	36.51652593	102.5048476	6.613941238	0.545714379	0.088890428	0.031556456	21.4387217
53.54931908	118.4431064	248.8549364	9.719608863	0.898431778	0.174636252	0.058746553	17.16397106
45.42987337	73.53945835	150.2503847	7.348498541	0.591446856	0.091927321	0.095139744	8.883489356
40.64306964	56.55120591	151.0806361	5.06598738	0.749236402	0.097536751	0.057859545	9.425726262
42.44489339	113.7544656	310.0346546	12.04312597	2.144231479	0.326140263	0.165437044	26.27072035
45.3442241	66.95826235	162.0572417	5.197962716	0.690809647	0.078864221	0.034566351	13.09064674
40.35413809	77.46100745	194.4217903	10.34807093	1.007757218	0.14719837	0.09262401	27.92637698
41.95668039	56.34712331	137.758632	4.882284553	1.106148984	0.143757729	0.05417976	22.64335362
45.39665054	43.7864363	136.0140213	6.870813694	0.606274603	0.124223718	0.133636214	22.34534737
36.08190034	59.69605837	190.8575616	15.22477619	1.205595306	0.232285031	0.116535279	44.77243712
44.71970547	114.71355	235.3852329	8.008192911	1.090805183	0.147939368	0.129551968	5.592484716
34.09888716	50.64485831	132.3237644	2.634572193	0.45493637	0.053139022	0.043457277	14.5009448
87.14379043	59.1817029	134.737927	8.802749877	0.556240643	0.059567594	0.029760341	20.71932141
39.67467475	78.28013999	176.3366585	7.897762573	1.081850288	0.201401011	0.079198193	7.797116153
52.05911116	87.52669067	164.3913223	5.110267417	0.795456448	0.081743684	0.057633576	7.994642825
47.62258673	52.43794764	142.2447763	11.04389223	0.713042463	0.131155511	0.069667153	19.98498978
58.21032382	55.35701209	147.9404284	5.781581255	0.622207947	0.081292007	0.028494926	11.07550998
43.84672307	93.11033501	193.3887434	7.69447723	0.512316196	0.098216102	0.043165459	10.80113649
40.21708377	72.73648667	201.4488656	8.204640634	1.167347563	0.212118505	0.090239819	29.80527512
40.66212636	55.46331636	152.7316902	4.109808217	0.550921193	0.094519979	0.036804358	9.960604679
44.79940956	52.81482538	160.3126234	3.942268555	0.392441954	0.080038202	0.039351086	11.13417192
60.61253883	93.08794854	272.2741728	10.78702144	0.990748422	0.191021151	0.025120502	8.464035474
47.04387604	51.11964294	128.1242693	6.002560201	0.760077047	0.133376659	0.105673529	20.5749295
52.16559308	96.61337176	180.7147654	10.33579768	0.992822232	0.160202546	0.057960919	9.62035954
52.14050677	121.1231059	317.8792897	10.13462252	1.77283864	0.224449175	0.108527319	13.16265698
37.39723454	52.02147122	131.6423307	4.032475992	0.61880733	0.071638245	0.051351206	7.7040392
37.63392347	57.81416973	153.3327232	4.271162461	0.448093563	0.061603213	0.044195035	11.84132529
46.00717561	50.92780423	155.8897933	18.41069887	1.080663345	0.150450996	0.327135154	49.98660451
49.5163363	98.85960968	202.5351213	5.250885156	1.090825326	0.110610293	0.065815304	21.17397325
47.74209964	91.92555925	235.1318888	9.422386206	0.889226375	0.125434353	0.081299693	13.51576707
50.41364688	42.28354536	132.1456906	4.196124261	0.425486748	0.039738484	0.049952315	14.9657371
39.85087007	62.26575242	150.7845431	4.383338006	0.624843529	0.132335639	0.067390983	10.62756779
40.8137731	80.79206268	200.8260504	4.800332631	0.576056367	0.05745303	0.03439122	6.741988436
51.4807686	75.91054718	239.5523613	6.293732655	0.830958732	0.123552943	0.067737379	16.34430337

46.33065006	88.66797786	252.5423022	6.439899574	0.576420003	0.100849088	0.043896	3.291594209
49.46764185	83.40519939	211.0430453	6.044902557	0.924098032	0.139219146	0.066770974	13.47589466
64.52445002	69.23244287	250.2365466	9.056984441	0.802991998	0.095990183	0.029809392	23.86829085
41.21048978	76.16558582	183.0212101	3.702452032	0.700454807	0.098522443	0.042195827	7.315055324
78.39041441	228.0623318	502.7163971	19.92809501	3.938946087	0.627274815	0.045373246	17.40101735
42.60231179	39.9717357	182.4043833	9.375182316	0.769638552	0.093286674	0.036076101	44.63785622
42.58408161	63.97227966	193.2140499	6.771713614	1.112760453	0.123238427	0.099133075	35.07933254
41.04529863	58.47841291	156.9421358	3.693759919	0.626311227	0.079001504	0.061043309	6.613945234
38.33754102	75.36044344	169.1881135	4.534559521	0.792059103	0.090441509	0.050534833	2.9147463
37.10006526	63.4606818	168.6481547	10.58584451	1.170847789	0.203712968	0.07161704	57.96530121
40.93819771	127.9792187	247.2059945	13.4999213	1.512052456	0.197476319	0.105031338	26.49616787
47.62625614	85.15664874	168.7818223	4.382540614	0.647432879	0.107700513	0.030310933	6.890778072
50.67226498	76.2958873	182.1450239	6.086208026	0.758352132	0.071171703	0.037350833	11.35729387
62.06044478	71.58778314	192.1487203	12.66988348	0.880563205	0.144139366	0.17130538	42.57930215
33.65751565	47.52727648	121.2403871	3.546819656	0.457852461	0.084729185	0.053272131	10.81142244
56.03375269	67.50697877	220.0559236	7.418487359	1.041119066	0.138535342	0.040068293	41.72250297
50.3174557	23.31326369	106.0653757	2.979246731	0.429056986	0.050584809	0.007934867	14.35361714
53.8280118	68.81670848	227.5740167	12.04798813	2.175094677	0.36326267	0.050788342	34.05648044
43.42351545	69.37860606	172.0668498	7.680977772	1.047344667	0.15649793	0.108960778	23.86135265
35.92204703	44.18269766	131.9948933	3.742220023	0.40982604	0.055177481	0.035236205	13.58304858
63.61460301	73.89764588	257.3607212	3.531150129	0.701149571	0.058355666	0.015041889	23.63804939
41.92371986	62.92878594	144.7520552	5.223349075	0.491531197	0.079864887	0.029518578	10.80282847
40.27592434	69.18246935	181.340523	4.973614626	1.468161694	0.153279931	0.033610227	18.95283785
41.36799153	41.73482711	138.0977685	4.561968226	0.564299066	0.098543767	0.04027168	10.09068127
44.55028322	64.0325772	158.8542353	8.713005715	0.514190808	0.063792845	0.049394804	26.45540591
44.2151314	84.34211355	173.6441267	12.60460367	1.599633344	0.252184802	0.058984948	28.07377454
46.82715276	78.95010447	162.4896243	7.55314226	0.50339783	0.052126875	0.048112878	17.28978581
44.17532273	76.09483097	163.4965478	7.400894861	0.619642514	0.093493905	0.039271706	7.721034365
37.85285722	44.77490039	129.6111257	5.266875642	0.458774424	0.061111611	0.036886374	19.06041211
43.27048884	49.54124429	145.5869851	1.718728868	0.375839206	0.042747117	0.014844169	6.465908986
37.08485112	56.7348651	119.1892827	3.169947345	0.448450741	0.055649195	0.03152234	11.52171734
55.0572958	54.57467252	159.8841822	2.914167292	0.32361621	0.046458723	0.011000386	12.94973577
36.78344052	53.00571539	141.131061	1.816705992	0.495012469	0.05909563	0.042256351	4.272587769
66.99386557	72.28263359	257.0229263	3.301575114	1.330131941	0.173739883	0.031712007	31.34245939
44.85241655	83.96196019	187.1548707	3.417257984	0.877973669	0.106178491	0.02727646	7.804916097
46.65734446	78.59609659	159.9063308	6.331913116	0.686250138	0.101055291	0.051391271	15.34482359

35.1897531	43.66281161	106.3492143	4.052625695	0.408013904	0.053494523	0.035486541	12.86802956
63.12120896	105.2220177	238.9583253	11.53628459	2.351458489	0.251650646	0.039407066	35.71845878
35.37845074	46.92902298	128.0016419	4.264103273	0.472568048	0.058699666	0.063094823	15.16932443
44.13724582	57.12092158	215.2027672	9.930227717	1.193659014	0.257095021	0.123986187	22.00922214
38.6615724	57.53002161	172.470958	6.053951517	0.696466444	0.087913226	0.042252534	15.03288294
43.49099213	81.39020173	263.5202897	18.21215959	1.701697621	0.238528012	0.10227357	66.07140409
43.49828332	100.5057597	223.7614791	12.29416849	1.082161135	0.218403817	0.115222059	26.56751032
52.03552671	55.95016917	168.5677377	9.557469995	0.76430083	0.087224243	0.059215241	37.6497294
34.51798801	55.92263209	109.8483646	3.80817998	0.423549144	0.048484143	0.03277843	12.08344051
40.90627097	58.54062886	136.6108763	4.215297729	0.446560002	0.055144275	0.024372218	12.52375761
42.30159006	61.24967756	157.0061163	2.614890595	0.383599132	0.05510977	0.034949929	7.08420116
42.1307833	60.32822314	148.9089169	9.677240668	0.725083785	0.093166862	0.074493172	24.04300007
51.45891839	70.15464201	166.9652033	7.441814005	1.081943673	0.122974042	0.229481062	51.43342972
45.27181874	75.90857254	210.935798	3.872313461	0.801477264	0.112554637	0.045371934	8.896011418
41.83274406	38.00234879	111.371426	8.138417322	0.965982901	0.170520189	0.039019179	29.34322435
39.45038426	43.76095822	115.0399153	3.443888598	0.529799696	0.05860912	0.053926354	11.7122633
45.14779945	56.88642177	155.2369336	3.952527932	0.462311134	0.049059062	0.046067025	10.06130719
40.8948577	77.41225605	199.5710736	9.829980406	1.229140037	0.136846042	0.07328748	19.77168563
39.96239101	47.25163907	116.9165558	3.969524336	0.434242956	0.053058504	0.043804427	9.53594544
50.97263471	50.45143308	103.4900877	2.395386897	0.383910617	0.029352799	0.023224148	8.302148883
41.39564689	89.84433011	211.4458573	7.086946678	0.600762478	0.091501781	0.036630129	13.71958824
59.26518527	92.51853787	270.7899091	20.07450852	1.614587063	0.209593627	0.154415473	45.62564682
57.80683287	59.94966667	163.3340686	12.67337386	0.735159377	0.082818194	0.036309812	15.03812886
40.35175616	47.0891724	138.9634825	3.630559825	0.442166143	0.050738657	0.053742214	12.46268308
55.23813145	86.15589244	235.9203652	16.36675222	1.866257989	0.199259285	0.13806067	30.77078739
52.02873861	66.129531	204.1768061	3.343994225	0.862780608	0.076055148	0.053679751	14.46047548
64.28831148	57.0623243	258.470441	7.304595275	1.209961027	0.196125228	0.09399576	46.04997504
47.57770091	17.45710779	81.0513476	1.797367383	0.548699673	0.073520008	0.015239544	48.3007157
35.83766806	31.75696495	108.3004842	2.235217212	0.375794201	0.052815651	0.020313358	10.2381085
48.01141806	58.41341131	145.8919139	5.412216971	0.553609476	0.104680897	0.059928186	10.35411003
40.96746259	62.51529654	146.3282712	4.282264199	0.553927057	0.094996992	0.043489712	7.059807119
43.92084543	55.64947906	151.8301583	3.307386228	0.495513833	0.07772097	0.050644921	7.255853371
46.22133524	69.08485138	215.7513956	10.77282982	0.899126799	0.123543389	0.071806491	30.9647012
35.94964262	49.17013373	130.1473953	6.58713004	0.637164078	0.09736188	0.037612763	18.06264909
49.18996036	80.2345615	177.1528681	6.153950776	0.746004235	0.134819733	0.032502235	18.79882662
44.81354745	42.78118338	166.0038294	15.33430563	0.664525268	0.070654029	0.026885735	50.21596906

44.5670621	86.26105751	198.2223628	7.969211854	1.108509519	0.128166905	0.070050132	9.459102048
51.08537372	102.2080271	229.7398654	8.904130491	0.804069007	0.097288337	0.074893633	12.91164734
44.93297064	69.34977092	167.7025178	2.952589387	0.756512237	0.054697418	0.050421419	9.798025857
50.82782753	70.90393056	222.1016859	10.58503158	0.860896328	0.202235472	0.061629548	18.77774902
48.66192245	46.01742501	119.742484	1.381605238	0.324517896	0.050079492	0.016128395	4.430225871
41.11049457	46.24266232	111.4606595	1.813556486	0.390726922	0.033099972	0.029991831	4.397694869
48.55813934	81.61015439	166.7890999	3.120527599	0.835542311	0.107316091	0.078323158	10.50706551
53.82606283	61.94893415	150.984451	7.126579348	0.834335659	0.100324408	0.025788079	30.19386072
44.97034889	63.63725312	170.4035261	3.475960418	0.4642835	0.07762489	0.0236013	13.4023225
39.88131554	54.39069341	172.0948641	5.983296457	0.71053642	0.098226356	0.068413772	11.48375184
55.89472451	89.13376964	168.6544201	14.91031478	1.183110311	0.127016496	0.104037729	15.54732174
46.93006978	75.74902221	238.9520857	8.169810436	0.765086545	0.139092293	0.062387903	8.961420481
58.73014071	73.23257496	254.0031598	6.185061733	1.175470588	0.207525474	0.049689051	18.30132824
47.74332672	65.72376535	207.9745845	17.20780123	1.167073435	0.194484075	0.085350076	34.4817844
33.92655188	33.24671533	97.67928933	4.026760848	0.422633465	0.051524311	0.029998733	18.56993905
41.93633633	82.34439143	195.3357381	9.179871857	0.933828077	0.162969101	0.054476816	17.02699148
44.02254922	61.83426631	157.4800381	4.946497669	0.49419551	0.073346055	0.034740262	9.294108678
57.62963985	49.20255563	180.9260035	6.860704852	0.72491976	0.070027719	0.020269092	28.45508709
37.87853581	68.96005091	174.5312352	12.67527688	0.668593304	0.098026416	0.063138332	20.4418065
40.63674568	62.0871558	215.6336761	7.127086801	0.643900646	0.086218493	0.040221195	21.62389727
45.44415764	53.63776665	154.9958289	3.904881127	0.416583692	0.065677081	0.020201375	11.06702903
45.33913137	61.02269343	159.7159922	5.066328953	0.732509144	0.090815643	0.048577127	15.24357518
62.12004139	86.41669363	268.1664082	4.592227311	1.336776587	0.191517866	0.080516332	15.4609533
43.31133383	89.67830653	185.7816806	11.22880629	1.104516824	0.183162332	0.066921958	19.52554997
40.68770201	58.32102416	161.7739599	4.402190726	0.578303465	0.08198227	0.057574827	16.35196204
48.24319796	115.1047515	273.7882065	18.29794468	2.160770009	0.378987446	0.240232371	32.0347462
44.72252342	42.81882611	133.5911859	6.4679504	0.71992648	0.119335657	0.048024277	17.4454
43.73869588	83.15537547	204.6986209	6.786168919	0.680110229	0.086318289	0.064377167	8.907803704
37.84765812	52.22783395	116.3497011	4.592052959	0.484969066	0.07390321	0.026694113	17.65025447
42.24938075	62.67221377	157.5126133	4.084166858	0.499965578	0.090564606	0.046307583	7.929615886
54.20527472	58.37195743	196.674912	4.424243326	0.8393238	0.098335959	0.02799011	32.23230209
43.2765384	47.45168647	125.9279265	5.702378418	0.33623602	0.048772888	0.026111144	18.98769002
39.19698497	35.86902772	115.0246958	2.131166555	0.307845169	0.041277273	0.020541869	6.821854666
37.86473998	51.83173535	135.8283575	3.052271508	0.528016512	0.060566457	0.035552147	10.82154352
45.20714088	52.66481944	160.726696	2.434883904	0.519631112	0.084146164	0.032765962	6.004956161
56.33450057	57.32303908	241.008266	7.747350721	1.253409366	0.166591099	0.045844663	13.88257683

40.302659	50.17212389	121.0398069	3.127643056	0.350770238	0.043339616	0.033423986	6.264798959
47.75500836	76.83489972	206.1761402	20.25033104	0.872727966	0.126397804	0.135318139	20.28271697
47.6657552	68.35033074	232.2562794	5.914135166	0.970992798	0.197326595	0.101045358	13.77505252
42.05705784	87.61625539	193.9806882	6.110662399	0.97838678	0.214380346	0.12558363	10.304871
38.67747333	55.19867417	151.9166157	4.354475633	0.746875604	0.114469729	0.066129423	13.11764935
45.30998572	84.38924877	208.7057599	9.025314197	0.875414034	0.115228633	0.083548959	15.12472478
45.28358814	53.39609711	153.9448376	3.741103042	0.649617551	0.083043307	0.05239145	10.71943816
45.01122442	65.5519097	189.3121183	4.59809039	0.492460574	0.049380208	0.049127463	10.42082894
54.7333141	47.1462403	201.0804228	6.39290643	0.788742289	0.105640857	0.05516378	18.56467669
52.07726315	149.6809843	336.5763903	27.69691158	2.520753103	0.625593408	0.059376595	46.4640524
44.75185343	61.54665043	145.6138293	3.945403719	0.511844194	0.107013327	0.049067496	8.158184896
43.98076691	83.40186061	226.6644349	7.029458661	1.117805279	0.155651478	0.068995521	10.69084149
43.73757327	36.10221428	113.9919397	7.820386459	0.863287095	0.142095957	0.034233838	63.9456939
49.26384567	92.38559453	259.8332416	20.2886072	1.354132195	0.201828402	0.196707349	24.35341732
37.99257894	56.4757092	161.9959956	5.520204456	0.908627393	0.12541868	0.055017566	23.52096599
38.38552512	49.52077381	112.4789092	2.296494753	0.465089862	0.059866229	0.023292967	7.736349285
40.28905163	54.86149827	130.6877003	3.580214949	0.422861434	0.05765744	0.047798149	9.450842146
42.28267541	48.57344778	159.2170101	4.712655139	0.475801575	0.070838365	0.04502474	29.18659172
46.64290962	59.15445536	153.2603688	12.76853176	0.973505151	0.117184202	0.080032488	59.38872702
55.35381024	71.03263967	186.1339143	4.782498973	0.767421134	0.169371996	0.051502915	6.501732506
50.63318553	83.7241837	192.0000763	5.286649645	1.016725281	0.170543437	0.068466556	9.70588269
35.09278212	54.40660516	151.7067061	5.782590702	0.712492604	0.100262183	0.055430635	19.55037274
61.92887346	79.77795994	198.3287288	2.581225818	0.782745278	0.128957646	0.031559669	1.825562579
46.03961231	51.70182971	169.9106591	5.784051102	0.799133981	0.095891608	0.029997409	72.58396645
46.6031674	44.6378199	112.5545746	1.329547018	0.465063939	0.060544366	0.027233069	2.257175824
39.86651636	78.2436028	224.9768557	7.072974063	0.953878107	0.192320592	0.07026677	5.191383003
49.79524785	75.24853607	204.6219547	5.3224491	0.984698201	0.173470499	0.05426299	13.14141876
44.78081549	94.883415	243.7094555	7.695613126	0.909280686	0.098577537	0.059978636	18.30441613
45.28703794	67.97204535	170.6463743	5.525208169	0.592204651	0.092483624	0.02924348	9.789390091
35.23852455	64.56715431	140.3897409	2.985880106	0.423556362	0.054646906	0.043716719	6.69017383
57.8585413	116.0510983	374.7568908	10.06090332	1.693528192	0.182561277	0.076723933	16.35489743
47.85348202	73.62057715	197.8351006	5.086966371	0.6799413	0.068710679	0.0419889	18.49105662
53.22340299	105.3722061	271.6605617	9.777980808	2.152295969	0.38077289	0.131855813	18.45974827
41.7819837	49.77427156	128.2467333	9.981241267	0.524205417	0.071825085	0.047300865	23.24361715
62.12515477	26.19485352	115.4143392	1.517078372	0.500906105	0.045795187	0.007430218	14.91304998
37.54531502	53.28987993	123.329116	2.83405396	0.463968	0.048457552	0.036836743	4.939515693

41.23871292	64.11442896	163.8414512	7.404365237	0.686054288	0.101998871	0.053505224	18.3675363
45.80567344	51.61969845	137.4158023	5.523234784	0.446489134	0.084857248	0.045054064	10.14506911
40.65760334	90.29517938	189.1803197	5.036640904	0.624274123	0.072074503	0.085373353	8.224618982
56.91724962	112.8306247	261.003156	5.078513691	0.548206733	0.120366608	0.042982171	5.588224275
37.70902978	46.3459227	107.7782494	4.530876484	0.434065936	0.043492482	0.048074267	18.89504758
45.57955296	55.71344084	137.6861778	4.243695449	0.380140889	0.040796376	0.018097627	11.12819717
41.58047901	79.38147628	170.6964966	5.406662201	0.727900027	0.103483255	0.109558847	10.24767788
38.34045167	58.187973	152.1957557	4.192833493	0.507383749	0.107518515	0.041358919	6.542902131
47.35226701	58.31957979	200.1498605	25.34430066	1.000134548	0.189727652	0.249660599	58.04612729
35.72891949	44.18747939	121.3275219	3.056479067	0.392217443	0.051611514	0.036232753	7.461779668
41.12501365	44.06269653	168.9739293	8.568861482	0.527928158	0.081583917	0.036300011	36.74610899
33.0522406	40.33525571	101.2669846	3.032597375	0.411562498	0.04511559	0.034143766	14.87487967
44.32989592	72.64967433	161.3144015	4.101936227	0.513746686	0.091998334	0.042791933	6.599210377
42.41471719	68.87924542	188.256507	9.996167215	0.952310475	0.138217541	0.058309796	16.08084523
86.90993105	71.75891536	159.6400477	7.597663886	0.591521673	0.08645809	0.02624984	11.51922583
47.93130686	35.36663379	122.75565	2.666361992	0.347450828	0.038850751	0.006373567	12.09809844
40.45217012	51.60193259	133.4385308	4.857389497	0.520173248	0.064521713	0.054319387	9.091007111
40.8604587	53.98613684	134.5137298	8.499716202	0.679202484	0.100710838	0.05665465	24.77817014
59.65551579	115.0802362	402.7671533	22.08600991	1.476873778	0.180257271	0.081483613	32.57321685
37.34024016	60.93797061	142.2063462	7.036671565	0.869969748	0.121871642	0.067974259	15.54319331
44.45552508	36.86424874	119.6254865	3.210882806	0.541317724	0.064319292	0.062592873	17.06238181
41.37675864	50.46706267	133.172428	3.386454131	0.592754401	0.08507947	0.027485324	10.81906792
49.96589839	94.94250488	204.7360427	5.787242899	0.864869351	0.113541864	0.077190794	6.51431375
43.63029854	49.18261597	185.3306149	5.639972586	0.604023026	0.097695817	0.041744476	12.95978442
49.98954076	77.62443047	230.0263274	5.026904272	1.03559511	0.119505516	0.081018944	10.97735343
42.78329013	37.13320772	144.0995026	8.574377701	0.878868139	0.121372215	0.022582656	40.31217279
41.90376391	64.75016388	140.0456598	3.38731288	0.568751343	0.082398611	0.045099191	7.873149184
39.31611077	65.05241582	181.589524	4.783276099	0.780880343	0.171609479	0.064392155	8.850168656
43.01255839	51.35281357	137.0933114	18.30437461	0.651403222	0.086225564	0.036805151	57.31957095
33.16942966	47.13711603	116.9581479	3.113820531	0.474002818	0.065709234	0.034436794	9.148732795
42.8988309	60.22969004	154.8400116	4.11495237	0.43112821	0.062189777	0.034848319	14.35306542
48.35442611	93.81604364	255.7059965	7.930727902	1.153240582	0.153243588	0.064715008	9.835238895
49.30564183	53.36498974	182.2203786	4.283893722	0.368984812	0.048310129	0.022772821	23.33012358
43.52782807	74.43349277	245.9316385	6.949529968	1.685262212	0.20698094	0.079071929	22.26130675
37.08017427	45.12940088	122.9098214	4.999990703	0.366679025	0.059299446	0.076329568	16.02952207
43.79168716	72.99565925	175.934498	2.782388312	1.090229766	0.147010087	0.07979567	10.92164297

40.48374801	53.37241309	143.2919152	2.614677672	0.434794221	0.057494311	0.029373146	13.72001939
47.83337828	70.21445556	228.9415547	3.163661412	0.67959419	0.077746375	0.046225961	11.70139299
46.44308326	58.87113794	150.4309792	4.162294135	0.468620351	0.085251327	0.028800062	10.24114851
36.37043438	51.89292055	151.360771	3.354447997	0.46647535	0.073912463	0.051091481	7.371215946
51.72883817	62.27288019	166.2111218	6.177050619	0.716094108	0.095681123	0.05530676	19.10382864
45.64851722	48.02310233	170.842126	6.757834019	0.91706402	0.141440368	0.086247851	18.6818861
41.95022096	67.03662326	231.4129863	5.614735148	0.895175529	0.135117337	0.059464232	11.76149197
52.82661726	145.7534181	351.9574477	10.12491112	2.094823074	0.373085929	0.057448247	13.2588072
39.96369756	64.37391859	175.0466065	5.151086805	0.536979289	0.091002729	0.037930662	22.01174809
48.37310802	48.28579021	129.4049371	3.574777452	0.422813359	0.052362355	0.04574123	16.94409297
52.83556537	74.64734853	227.8529105	24.38685638	1.156628952	0.202972958	0.048064601	35.97426987
56.42179975	51.19265031	166.0916479	4.220177143	0.839227593	0.084606432	0.024648401	22.64608093
51.58746617	73.10735668	224.0836654	6.141115515	0.896376688	0.139479176	0.089641649	15.59233454
49.40161198	77.55327149	201.3053281	5.063998054	0.765993916	0.148842196	0.084773717	11.32317559
46.03656001	41.57069554	139.8702295	7.252169496	0.655988168	0.080510754	0.075685036	14.05876138
42.56895751	75.45637582	169.5039082	3.050372897	0.648815168	0.099530768	0.035647366	8.354101699
46.92576985	92.02991244	258.9540244	6.213397718	1.197455127	0.164480241	0.078502138	11.63313439
41.22724614	71.10812241	163.9046738	3.417400566	0.775632857	0.105461746	0.046196798	9.227741036
31.0210663	42.81173257	98.20453412	2.338158879	0.387895869	0.042280194	0.03173055	8.088187806
40.6681614	24.17795969	71.10574299	1.139984774	0.225592129	0.022218574	0.006748029	4.511320556
43.16095547	40.08147979	218.2608723	4.932264111	0.74571404	0.08791666	0.050320433	44.28487954
47.84938644	73.6045552	236.5990338	14.68655935	1.073233602	0.222808004	0.104013179	28.59681492
37.11153421	38.36614255	112.8762945	4.718526829	0.693379952	0.124642427	0.033901022	23.02840328
46.29193037	79.90102582	226.5564236	6.671619359	0.82962549	0.117641375	0.061145271	10.92219953
48.00571654	50.30687915	231.7448569	5.854192736	0.997832384	0.241176706	0.043120948	29.15608556
36.76867905	52.06901903	111.4681434	2.249913994	0.394451259	0.0510222	0.021797196	4.491389056
54.59640002	75.23235098	230.1820126	6.503001357	1.128411805	0.178913922	0.048978811	19.31555863
38.64034931	53.38313888	139.3595126	3.544478862	0.47317785	0.064147301	0.029483321	15.68434128
40.03186603	41.43247245	134.3815015	2.782257668	0.494521286	0.08181134	0.028637142	11.12735101
41.9429585	48.67019136	135.9694395	1.491200503	0.286180776	0.038465853	0.016938793	4.00991244
51.0712849	70.65480679	212.7672287	3.066350898	0.731433497	0.149383435	0.10187708	10.91253813
46.54602154	90.07324962	188.2506425	7.06272362	0.957014473	0.142335285	0.08714182	11.64626234
48.01797395	77.88891343	175.7110202	5.28941863	0.66704289	0.110899781	0.046948245	12.79843361
49.57229311	55.59047352	174.5320748	7.572543101	0.835419516	0.153892224	0.067644967	39.54945081
44.26699265	62.8091719	160.6799015	6.009403232	0.646210799	0.081788248	0.102852466	11.91835336
44.6473363	76.45360105	136.8939199	3.140334064	0.420332769	0.046335772	0.058846091	9.01717359

44.98844239	87.39721005	194.9954423	8.754476918	1.064789655	0.161172804	0.122796674	10.01593558
45.21586426	44.55483656	122.7028861	1.859227438	0.311502198	0.040766727	0.028179844	8.984752472
43.91732957	100.1714123	232.6910177	9.249730133	0.966258561	0.184733338	0.091576417	13.59364765
60.55185797	89.41781634	309.653222	10.41894982	1.419201814	0.144742526	0.050075664	20.61102242
46.09448806	91.49489146	231.5354531	6.409261509	1.010021174	0.137761867	0.064934065	22.0385858
50.98570897	82.6486803	224.3256245	5.148522356	0.897793759	0.201598769	0.118632212	7.898050441
43.08688395	68.86570047	192.8998498	7.031989737	0.780478925	0.10960515	0.095373716	21.28556918
51.82123472	71.24750804	206.5690155	3.250984267	0.570354889	0.111244607	0.049182805	2.762074828
40.51437791	61.41774621	161.8341886	4.430468283	0.440933494	0.060563277	0.026611154	13.84290642
46.38640544	90.27706435	264.9425946	9.535427393	1.590499573	0.374522616	0.121616813	7.026767375
40.09517163	53.63514965	164.2838483	4.29832761	0.438217085	0.064354868	0.025032897	12.14905005
41.47967779	53.82123171	169.1033252	5.34408611	0.643852398	0.108319081	0.091113042	19.14787703
51.7859911	96.3701899	198.8405251	3.549606543	0.86023757	0.111490209	0.049134663	8.810068129
44.86163702	86.95786987	229.0565509	6.770816242	1.138684287	0.142872057	0.117855112	12.46287027
47.17002292	84.10622932	184.3543265	11.00506012	0.866576743	0.104804581	0.05517585	22.26219286
47.97946455	63.25115735	166.0498321	10.92038119	1.187219199	0.250292867	0.057182975	22.27867361
42.86399307	73.7322653	173.3220939	4.69125448	0.616649728	0.09432913	0.02721772	5.340733385
38.28597633	49.91123932	136.1555831	4.370034559	0.584247313	0.088808586	0.046125875	11.40540827
37.64834106	63.20404925	157.0280303	5.199784512	0.748450561	0.1265969	0.06705235	11.64006392
40.24825396	65.3398165	151.7706048	5.54900737	0.79863158	0.155893453	0.066646901	15.34710697
37.64455715	47.09921576	111.1549529	3.065891233	0.398869711	0.043222722	0.038214718	9.512343006
37.15331955	53.73371818	140.7182473	5.391362126	0.546000553	0.093106509	0.052076471	14.42505917
43.74674738	58.02477902	175.7159149	7.324570901	0.602824728	0.072868904	0.019444332	21.27553564
43.55917712	64.52321068	186.6885251	2.698361966	0.562368387	0.101449372	0.055134803	6.764527894
35.83498946	46.42955471	116.2195763	3.69553122	0.658981332	0.070733223	0.067830793	23.44200406
47.59686294	82.08447902	194.8910764	5.356139543	0.565889176	0.1129175	0.037010891	10.80193021
36.23930602	39.66386511	98.46493576	2.085513839	0.380481184	0.043615311	0.020709509	6.084308083
35.19317601	39.95629478	113.0213642	1.938306231	0.370594507	0.045546704	0.022902966	8.286050146
53.55802049	54.61608007	144.983648	5.508823114	0.550262199	0.057649393	0.038347149	12.7456144
44.6491866	122.531454	194.7737176	5.044858766	1.129350131	0.139669706	0.044528104	9.004310501
48.54598243	96.89130647	203.9615312	7.676192632	0.691847683	0.091949481	0.056282343	12.7728756
48.14892028	32.03677523	103.816349	2.721910909	0.2996418	0.036561488	0.017797051	8.496640672
39.63407055	62.30915376	147.5979748	5.253292805	0.49948004	0.073897098	0.051395182	10.8449812
61.13603002	111.9115444	274.3868176	4.970221848	1.28523525	0.136813561	0.085192723	9.269966021
43.42305716	62.39473853	150.6771132	3.694969548	0.615223844	0.088824064	0.04661407	9.842713599
43.2826481	91.2448464	200.304307	8.661482426	1.134913059	0.172020707	0.099400582	12.01432978

44.23241562	59.52135846	163.4610334	5.610226317	0.600163608	0.101194995	0.043354473	10.22922904
48.90973487	69.22732587	155.4984646	5.624847603	0.776618969	0.105489508	0.040988673	15.00521931
37.95544182	62.79205604	151.0680405	2.677467338	0.556568224	0.086208224	0.053618136	4.230033388
48.93308176	46.04269184	134.246249	1.58952497	0.249895018	0.030639912	0.014018325	7.971276392
40.48010647	69.50726061	154.0481845	2.676674566	0.542068873	0.061834191	0.047874283	2.879565546
43.14489535	109.5987651	292.9107289	5.695691234	1.084256044	0.159242404	0.055075282	17.16130986
47.89263729	49.07219427	138.0575745	2.477544636	0.615600446	0.085171664	0.023155002	10.36800743
45.19116056	53.38976692	161.407469	6.267642452	0.414847182	0.061490303	0.039501451	18.85623024
49.96471207	70.81805905	261.5660812	12.08363851	1.365415219	0.179533233	0.085679516	34.1405881
43.23542667	48.87293408	111.382007	3.879100298	0.311301908	0.048303639	0.016631826	9.841057389
55.54711662	54.53041193	194.8318392	7.652811032	0.51510738	0.080023686	0.049725389	13.90172488
55.82978024	70.90717089	264.7691657	10.19440086	1.501575115	0.280580392	0.097953885	29.60602142
57.63061361	77.77938499	235.9258807	8.433178228	0.801012343	0.158723665	0.066151011	15.43560176
45.40048884	37.68193565	120.1652581	3.138340388	0.554398867	0.069438209	0.031379406	12.73033784
43.62628188	42.70855722	105.0717485	0.81641447	0.337468282	0.044609953	0.011821592	3.382343155
50.24520621	43.79370471	123.1028905	5.902284189	0.466210089	0.088235437	0.013822634	21.99356327
41.72873611	54.76468945	131.0779506	5.20615967	0.586714602	0.068938679	0.056634563	11.08695117
50.4773852	50.69693677	153.3045186	6.294172798	0.55324467	0.050056503	0.038899612	20.27743961
51.71763924	58.79938873	150.0851481	5.783757216	0.392009133	0.058804012	0.01603162	7.6634556
54.31001036	79.71667037	234.34978	9.453967578	1.24204852	0.117939298	0.099999965	16.47907206
43.1661946	43.91614274	115.9701694	2.095509934	0.353188921	0.039457334	0.036639879	9.910212989
39.2357444	39.13967112	110.8868413	1.92867989	0.370126836	0.047216557	0.024047253	7.505929459
52.20432753	73.63021994	167.9473407	4.221023235	0.468787855	0.068144933	0.057131171	4.725236105
38.23402169	53.9286952	124.8159515	3.258396717	0.3550779	0.042227686	0.026596186	10.43941047
49.14544546	63.36439661	167.3829324	6.115508186	0.489175359	0.054688278	0.082394006	19.53764829
49.44656484	53.90847738	167.0994369	4.867014676	0.539231379	0.058026081	0.024042613	12.18461452
62.33380707	54.46849787	258.4531592	14.93171682	2.227558536	0.786185624	0.185659744	25.60432242
46.92122796	60.00386118	153.5002619	3.160122515	0.604423713	0.094369313	0.020594919	12.86050451
43.92042428	60.33931613	184.507734	4.269118658	0.544293819	0.072256612	0.035118279	7.166337903
59.21335697	36.91072605	125.2068175	4.615338209	0.673207334	0.105191578	0.070519661	16.62507426
50.24827697	60.64917957	154.0139447	5.814182787	0.942036659	0.097699746	0.079046042	18.97228018
50.42429702	43.12692422	176.7453177	5.830608464	0.754501948	0.153738116	0.033414453	46.55862037
40.86282461	41.04450319	105.6834183	3.147615105	0.298074945	0.040597495	0.028521159	14.37579324
43.70089292	49.9850772	153.4360442	3.719887765	0.669360795	0.057275001	0.025089389	9.051098668
58.42038745	38.90093787	196.0857188	16.07292809	0.997213971	0.111094763	0.049075204	55.50140758
44.72648909	57.28552873	170.3292817	3.021744246	0.41245501	0.077942067	0.019849757	7.700308246

39.40403675	43.41856037	118.3289105	4.960074457	0.566498179	0.071724457	0.051973912	23.32915686
38.21588696	34.72687414	103.3848589	1.265554961	0.25613575	0.033547955	0.01768322	4.87341894
46.51907663	47.1585822	157.7866859	3.977123142	0.542731024	0.064311324	0.033220416	25.67899427
50.7638722	51.58516214	176.4797045	5.002666831	0.550611674	0.103637514	0.025209635	18.71389355
45.09339823	36.23483956	146.0718987	6.387477008	0.360636255	0.048405961	0.026816397	19.12677396
53.02035588	75.09711481	230.1958765	8.862228385	0.969062276	0.129423761	0.106077921	31.02758055
54.04094609	42.00504693	138.5337018	3.810889322	0.329020197	0.036734088	0.031606175	12.10070063
52.32784244	48.50817107	186.8672094	5.784120196	0.411450159	0.088647723	0.031084744	7.671674512
46.19995419	55.7437568	175.166016	4.406875784	0.493683464	0.047741267	0.045740223	16.34705411
48.61043176	86.13332724	203.7110745	12.35174775	1.003286239	0.180496776	0.066258937	26.55333842
54.84720508	63.1262037	156.6714864	4.98281943	0.897297137	0.117158654	0.034901725	10.05731993
57.3835921	41.01308791	152.966097	3.425826038	0.403921446	0.061684629	0.010163001	16.74728934
72.16842953	33.71772165	188.238136	2.309479356	0.464722241	0.059447202	0.021188305	23.84880008
55.60468783	62.52669908	174.3126194	7.854589282	0.609297685	0.109557787	0.058953332	18.20628987
65.53696496	51.04555848	275.1447652	6.762866284	0.570148734	0.079703286	0.019257396	36.08366893
40.70722976	50.28746796	151.2725608	3.345092864	0.35094913	0.057650699	0.016738734	6.748080698
41.85881308	51.99550221	140.1828558	3.88270368	0.425579432	0.053478243	0.026524763	11.27774359
58.64749107	72.99750707	251.5240178	4.009157037	0.79109706	0.13773453	0.033358059	7.007886949
49.86200989	52.64189086	152.0460126	4.665867025	0.399362288	0.055097717	0.060992647	18.62133371
40.88741946	29.8315259	108.8003738	6.935812062	0.488791233	0.090557988	0.019574475	43.30565966
46.09616688	34.60474612	154.6354557	4.086777585	0.299196199	0.048634003	0.015368403	12.1437287
43.68935192	55.57471742	141.2336902	4.429811235	0.334606537	0.043795277	0.030846016	7.125344547
48.60346649	62.09828902	175.1477776	2.403658356	0.908504719	0.10929474	0.024951239	7.457984884
50.86700741	43.92452669	145.2612962	6.383108643	0.542270378	0.06490318	0.036750698	31.17393844
47.08574729	48.55227595	156.8541742	5.064970477	0.401329423	0.05778649	0.023663706	20.62619222
50.12114369	66.02467537	156.9772359	4.366928513	0.528196361	0.075683749	0.09301871	12.1624978
56.07265192	103.0039224	240.1695651	5.628823934	0.838425923	0.191803783	0.041986622	25.19947363
38.34481322	45.37606565	113.9432847	2.804366557	0.395426294	0.051007195	0.025534249	8.628700468
40.82922561	44.24908543	101.399331	0.935749791	0.176971669	0.02180569	0.012813824	3.013547697
54.56326657	59.64712015	163.8922974	5.073334517	0.748984004	0.079760052	0.021710553	13.90511351
51.43367025	68.8789187	209.595977	10.84512579	0.867931138	0.143469515	0.033327708	19.20009862
50.6754307	33.49374385	128.0363529	3.186888376	0.485765029	0.055093495	0.013683719	15.05302254
38.23979138	33.86875518	90.69262034	1.926864637	0.260970458	0.032981213	0.017840189	5.475030517
64.69720399	61.77714323	246.0834642	9.325748237	1.733583125	0.148209333	0.092472489	33.71535116
55.90339544	53.55404809	172.6644683	2.816534432	0.432972769	0.0605041	0.026248845	7.456385611
43.24367559	37.46179612	98.29134684	1.896061817	0.462918991	0.057573577	0.024811543	9.504952615

55.26959238	76.91062151	200.3680788	8.053520242	0.606660543	0.072551814	0.059396189	12.92459056
61.80036479	35.72708608	151.8393547	3.264375462	0.302768551	0.037493436	0.008775087	8.384753642
52.92939598	78.56886411	159.5703958	3.230271735	0.540734207	0.082547101	0.021394332	12.03890202
44.93718627	59.918958	159.9394515	6.025889547	0.625329781	0.095271145	0.07270228	16.93375066
42.66619021	71.73330653	180.3762762	3.600960617	0.699027309	0.09571304	0.042556856	13.17126462
41.14369814	54.05864256	157.2869072	4.319994974	0.628228054	0.111767648	0.051257132	24.60442306
34.83128719	25.48339658	86.15995096	3.737252468	0.434408876	0.078221193	0.039455598	9.645800562
36.63265192	34.64121526	115.9497233	6.497684178	0.403871718	0.048122322	0.047959224	22.98049306
48.1576054	75.14198371	180.3989277	3.709549498	0.494251857	0.089714173	0.038465088	15.41493571
51.28407866	63.03729299	189.8333459	12.60032744	0.769289384	0.159895341	0.06795991	22.7138794
43.93475546	54.0666455	152.2548898	5.061636855	0.565045976	0.080087886	0.052499576	10.05716664
59.15695276	45.74198009	158.4000918	4.960204677	0.409695262	0.062186948	0.017742204	17.04675158
36.82767821	22.64449003	106.4857627	1.942195341	0.424930445	0.072354728	0.017925584	11.96189145
44.04348616	80.62563811	166.5698244	3.478873691	0.488858299	0.060715441	0.023991836	7.125159228
51.96400545	35.51324862	121.8599453	7.886854657	0.329277727	0.047470182	0.018138101	19.34068533
36.02815326	38.95844603	115.8316608	3.537525824	0.329200293	0.048664142	0.015882813	15.08715996
52.07378633	49.67429375	128.0276171	3.747526061	0.523585532	0.066182001	0.016091925	10.99355014
45.21289689	41.45731638	125.4366292	3.306523182	0.411963224	0.066418461	0.024278283	8.464121125
42.82106372	64.35005138	183.1304952	5.665464374	0.764986398	0.109421103	0.057299134	9.809785559
37.43389135	38.15777966	139.2259893	2.250363514	0.440133708	0.085157957	0.02827633	19.37683901
53.73112506	85.52678233	232.4217588	7.800559843	0.706776148	0.088429006	0.060354176	21.60726415
44.54809694	57.45780943	154.7657828	6.136266061	0.633175123	0.095162772	0.06822661	9.512213104
46.80269132	55.92430199	193.2853958	3.940948809	0.407016824	0.062859028	0.018133084	9.546101897
49.16053188	37.38268544	164.9286088	3.701400669	0.346776086	0.035119609	0.020497103	8.514074225
50.11313195	54.21112323	151.3279077	3.52847594	0.897252597	0.122601	0.035957587	7.706213741
40.85211144	68.95566011	179.0408513	4.756618588	0.499151599	0.067728046	0.034154069	11.87757147
53.16842124	87.2860526	171.9676879	4.21037453	0.633556674	0.083760404	0.033492841	12.11784764
44.54106749	71.80431643	239.1344321	6.215118206	1.078948929	0.207519054	0.045840805	18.07645002
48.37096879	73.78633552	173.0894732	5.158377306	0.615349821	0.083818935	0.053766039	17.26748315
44.47852671	64.93476446	199.981864	6.861406577	0.789145112	0.149721937	0.049720401	16.18336593
52.81398183	70.35616979	147.9640358	3.284980465	0.749906353	0.114182038	0.020277703	4.427159328
47.91572434	57.41498347	162.7154132	5.199898228	0.58141927	0.08864284	0.025439075	7.643572406
48.06905798	37.45362695	111.1769809	4.51365334	0.400865859	0.041407627	0.027243908	17.36220531
49.73939958	85.78011995	185.9523625	4.317683797	0.492333933	0.067221986	0.059949364	8.309595153

ERK_6604_1714	IRAK4_4710_1716	JAK1_8709_1718	AZD5991_1720	PAK_5339_1730	TAF1_5496_1732	ULK1_4989_1733	VSP34_8731_1734
49.01575904	171.9663868	53.34730335	103.8499829	14.22435114	40.66836919	23.24953416	11.98333242
34.74202659	135.6929258	48.13965515	66.61163681	13.48800335	71.53905621	8.345081127	12.07698244
71.64029614	243.2694881	138.6425694	83.78423072	15.78304241	63.14952118	15.70798045	17.94156953
24.89060021	176.9774915	94.66233744	38.59357514	16.31211913	83.75584297	14.3153389	13.33665928
35.20525649	254.8421203	105.8066771	133.7195011	14.2948511	113.0149201	14.81041124	19.27379394
43.84650763	108.3473153	29.86461198	44.0501606	10.73011871	61.68800271	12.99792518	12.77375329
10.80572635	101.5221764	52.69986309	179.6255747	9.884270219	34.07381633	15.51098186	13.43011975
57.02309092	196.107361	102.1775581	70.15470305	15.57281418	52.15131438	14.03693967	16.12504654
40.57036195	203.9658624	112.7198209	179.2735112	20.68679356	93.09587038	24.06460501	23.3664047
46.068785	89.77117861	34.40557029	23.30693047	7.673137764	31.47112273	8.110362079	10.30836424
32.07203349	101.0870668	41.64232428	23.52236656	8.929592281	13.3359966	15.61293168	12.94920096
23.04605837	120.4553306	66.2086542	93.42717519	10.82800643	38.34593888	4.604093774	7.382567545
19.92213238	152.3788608	58.68711374	68.35248149	8.863998017	58.79641015	5.860463643	7.7595729
62.51242741	173.7242916	66.58890182	82.68676464	13.90380829	29.62307617	15.52174767	17.0917481
32.91643579	159.0616706	86.12317323	81.70387545	12.72480669	63.78321126	7.535603954	11.1255212
53.95165417	184.7625942	64.20035184	137.4781943	10.44381767	89.51122132	25.83889223	20.90147297
34.42638605	69.6444593	45.89287831	7.019741998	8.546198111	26.21961262	4.21140984	11.69264673
7.621937084	189.2210354	188.4461546	316.3125564	16.26278782	40.66198552	13.67601696	8.308081655
30.36196097	220.6044726	132.6103135	202.9242689	12.94898645	123.0556501	19.52298632	13.41677792
21.1092638	103.6027068	60.480706	69.56421401	8.159759179	82.32362924	6.677082668	8.816779835
27.73486175	167.933247	65.15808424	86.81568716	11.3168725	49.0248049	12.20778393	11.6740353
35.18144452	180.1401578	140.9762616	86.54330948	14.09184738	38.63818967	15.54029009	9.884093131
15.48042596	110.3624031	48.86502779	112.9580992	9.773625888	61.02833289	6.824899115	7.663181877
31.75218202	158.4513063	72.93439291	178.8893441	15.2147249	71.96369978	9.146023878	12.22057392
36.05649355	141.8481402	77.23408705	87.00522135	12.43293152	54.19530092	8.958920349	12.31058601
36.15257657	176.9220928	78.44553353	114.8984753	13.39022061	53.5016598	7.29954801	16.67186865
81.12350546	191.9537711	137.3198652	105.7215809	11.99352305	79.54325214	13.84994064	11.0304818
16.16015507	165.4725395	74.4235545	221.6690966	13.49966943	104.3211097	11.329852	15.11245072
16.70034178	123.7686586	64.17213411	84.47743516	11.43079391	77.60169568	4.889074806	7.704935082
60.58281881	90.42992043	80.99845894	22.81992031	7.343146082	48.02199131	4.731144994	7.705485549
33.09888386	136.7929416	64.56836957	48.4646634	9.529252424	24.16097134	15.14551336	15.60747524
21.04686804	143.5151534	90.297453	108.9450427	14.83801692	136.0528705	11.57530147	12.42877975
37.25978503	220.5801367	130.8105662	217.6757382	15.74862006	113.1687868	12.31861602	11.77668726
56.37686813	124.1368631	85.65393185	27.65831538	11.35158781	20.4454503	9.956578082	10.11177395

25.06401334	163.4822166	71.06650117	134.7182605	10.53748364	56.9012447	5.890892645	7.391963925
34.03818074	228.4039971	127.5929695	367.1319921	16.40914219	108.4249591	15.87968552	20.11062689
32.89463169	106.3982297	38.05129011	48.49621829	6.43051807	99.52727992	2.589241183	6.975473068
32.04365411	194.1755781	148.1075333	143.4873207	18.33179096	110.7562165	20.8764264	15.59028745
39.27669081	150.790097	73.58318755	125.8664877	12.32025959	57.48553571	16.20656693	9.338526934
55.38077628	153.652567	97.15914486	46.10818487	10.44777514	51.955846	5.765283529	8.662365295
68.80568461	137.9335322	156.6615195	57.19250969	18.28729267	59.84214148	4.653634067	11.82283418
25.86359847	188.0689738	51.30345553	103.0509288	12.92469669	96.77905471	13.90533831	20.00173162
28.26158885	126.6742406	37.32218336	102.0117206	11.66896914	52.67336192	12.8531084	6.62036097
54.76563763	157.7845204	47.89926397	37.94582184	9.134625288	44.37308487	9.676792943	7.998270386
30.69515758	113.0269966	41.31739947	78.51983268	9.710139866	18.82624853	7.79658191	7.071992947
99.59390761	76.60070142	38.12433902	1.061111227	3.896414971	33.79791658	1.980424471	6.597181375
26.16761565	132.5352434	56.06719027	91.3989728	8.473028115	37.22275612	9.194166748	8.43813323
31.03038658	207.395824	89.9219287	59.94112767	14.38111168	76.43823837	6.158448557	10.68662255
21.02944273	96.07457667	28.93611171	21.8312725	8.706817197	28.9632432	9.004383497	10.19357589
27.33420993	105.2376685	58.11865644	38.84429452	10.2930828	76.70000223	6.535386042	9.788527414
24.38320182	73.48574201	26.8707467	18.7880374	7.265250678	20.03570609	5.662122685	7.319325507
22.35931292	170.270096	77.20891206	165.9221504	10.58528857	92.56863143	12.67803321	14.68916872
21.35315718	135.0384504	65.77518034	102.3516647	11.55130464	122.5636049	10.64392659	10.24214343
24.32361667	139.6703275	70.19842825	79.08548035	11.99682159	72.68110397	8.967279649	11.38772689
33.52031562	109.9421909	94.72920019	71.71145376	11.97523803	46.76476039	7.244836433	10.55403837
19.70603465	149.3090914	76.14626284	43.5980594	15.35770124	79.45840261	8.985600845	8.305693376
25.16322348	114.7335466	63.23507563	37.33151316	9.431994474	42.20333551	11.10477741	14.26494024
27.40071754	136.7962703	53.82283096	65.7245012	8.181766357	76.25104241	8.834794332	7.464843231
42.36679692	151.9986598	59.38503181	162.9463346	15.77700479	40.79890999	12.57721911	11.58139911
48.32985611	159.98463	92.40370862	189.3793934	13.0266413	80.67144492	7.673447668	7.079082456
84.25517726	214.5586746	108.7436241	153.302016	18.89193272	77.13823738	31.02395875	27.73521213
48.00410154	168.0291794	58.81228469	63.17100277	10.61651466	45.42437012	12.43488825	10.70637482
65.85117235	252.0694022	132.8698051	90.83635656	15.26846644	106.9654708	12.89608867	19.65232858
43.14963486	129.8138969	67.43292858	57.01044048	13.97890367	43.23760832	4.648493429	9.237598531
32.41475027	123.8060468	39.0335135	79.76906448	9.160027168	48.72075681	14.6645328	11.41030534
24.64996822	92.53910745	34.20700158	55.22814746	9.215482526	46.07640419	6.62263557	9.07185069
19.14450509	104.4186167	89.0911004	119.791842	9.353308706	67.49724819	5.595662663	8.097994042
120.8293588	323.7396197	173.0675197	792.773284	19.5140737	82.69094324	32.37644501	24.60904078
21.55678441	186.7998371	100.6002973	123.4264194	12.62438495	97.28715893	9.701348135	10.18116183
75.19113168	104.404728	63.0298115	27.03310292	8.981454727	40.61881412	5.041245772	13.29332461

41.26448579	270.2896462	185.2897812	160.6281395	15.58660047	107.5722904	17.0166112	20.84568286
56.61938278	87.19559759	56.83548742	7.053143833	7.976760627	27.02346214	2.547376248	8.189136159
25.10084652	193.1160848	102.0810517	227.5174882	10.74006632	137.8822756	8.081561457	10.03350974
45.86858897	327.3640736	212.4556095	341.5406411	28.64463885	104.4464285	49.45449768	31.03129307
29.48132246	117.0143303	50.12601728	22.69197817	9.595845512	48.71342336	6.235968169	8.613525559
23.12320958	116.3327465	35.40250248	42.69786898	8.526303764	36.83264661	9.558422093	12.40035374
37.41950945	86.99586948	39.9219333	8.089505787	8.041500076	20.19556822	4.727568354	4.374315798
30.0806888	150.2898421	43.75501073	78.98229173	10.79719242	49.69172133	6.42983444	10.4961607
72.27582135	188.0252742	99.51666339	49.59131048	8.799377829	23.72441666	9.814731267	10.75723428
51.04299537	121.4508246	50.04813881	23.93018494	9.088551632	23.77004087	9.432961474	8.130506938
38.88351441	191.718125	132.0870418	284.1775012	17.04772449	99.16346047	11.46833192	11.54495234
35.54426543	204.3853664	125.6556193	239.5586957	17.43563751	120.5828368	24.86287409	14.8992173
16.58179852	108.2172129	52.80493665	71.18779588	7.945623458	42.82058437	6.355838712	9.201798204
25.9666668	117.732472	45.54795596	62.3664891	7.69008888	59.03428749	6.146965312	9.05935579
32.99714947	201.1526306	89.21578792	97.66960609	13.21270151	67.35574327	16.54983521	12.40661467
68.05235659	213.8205458	122.9397368	162.5496329	13.02740659	108.5676555	10.69407504	13.13673711
56.46139167	103.2035044	62.99815425	28.99197938	10.20583723	13.53238353	7.597773746	21.36899149
40.04225435	128.3505092	51.64635121	48.96151354	9.750025646	41.93956907	9.265588946	8.576112179
37.11856006	176.4044989	123.4289554	119.1293293	16.15291983	65.66185524	17.81743407	18.95003341
18.26099235	110.4610047	65.30809445	44.98771697	8.774282419	92.87231608	5.298346353	6.260134406
41.12059455	129.2077726	56.40810601	74.44109753	10.02818772	18.99059795	12.09520508	12.55674173
11.235851	98.31134326	36.0332892	29.99458286	7.483657595	26.15055329	7.568486758	7.470896046
23.44884149	147.6989729	56.83779928	165.521936	13.52034712	44.45351636	13.5309895	10.57917242
27.24572554	129.8980503	44.78997267	51.04441157	9.731748246	71.1969437	11.66180885	9.004021489
20.67266654	129.7787559	55.73446724	88.45361586	6.755510225	48.71861567	5.300048245	10.12938565
20.86800195	157.625774	63.27182508	83.05086357	13.0084607	79.81361567	7.146407778	8.589870473
31.0127546	104.0604145	44.00576756	33.40779886	10.08611495	22.53556266	7.558953066	9.555000286
7.301005498	129.5471482	59.23030233	339.6554649	10.22011487	181.6631463	9.081689391	10.1191903
63.16304298	183.2852223	66.09391365	58.11656848	14.85207275	71.40482379	19.65434076	17.52971634
67.56341579	158.4392423	68.57521495	21.68803108	13.99480951	39.54051062	20.18896307	13.55109812
61.95032817	173.5057516	116.5248713	64.51213764	12.49271912	39.09250372	7.834902603	13.74437138
37.83894235	172.6824971	67.43608595	201.065524	12.26594967	110.0026961	13.6888205	11.23818518
57.46784222	54.80994499	61.33901753	8.559766855	4.903731809	11.13471562	1.710027586	4.762079542
40.27992685	121.8989793	35.55824002	71.2078358	8.966779819	44.13271541	13.66939265	11.08975207
58.82384437	102.7934135	55.04080834	24.17007506	8.262261199	36.95268858	8.3678282	7.519692496
21.727539	165.5101169	66.64277709	157.0579322	11.33131697	88.00768524	14.69798082	10.76524965

40.34158432	165.4628548	74.50958358	113.9161487	13.03377267	37.26501417	12.20590499	11.36072748
34.75641871	218.760943	61.20572467	140.5396628	11.60662448	91.97267705	15.01229455	11.06114441
35.47627081	102.4698795	37.31129979	23.72997761	7.748798914	24.86090771	7.045943052	10.29334772
42.15748783	205.0413613	86.05568166	180.6024986	12.56194228	82.65881929	9.903102108	10.03540683
21.22329893	161.5786204	56.92451716	118.281558	8.863424052	49.28021289	12.58233861	8.642977751
20.67294551	115.1320539	49.57128226	162.1035456	8.884046982	90.49204098	14.8799309	12.38166562
38.93527995	217.8223461	108.2000591	435.3887348	15.39477148	259.8812663	26.75502871	23.75541385
34.62984925	161.1176081	58.37590882	75.89937932	8.562910959	48.87735301	8.55728616	12.23130803
47.3107417	180.3353074	98.80173443	117.6737449	16.40443431	79.19697298	12.73377992	13.85362018
48.43881259	143.2991479	61.65349751	51.26076352	10.47540584	49.81430193	10.16305961	13.61488019
47.95531916	162.7009601	63.10469956	46.95580909	10.32827851	31.97705888	13.76392937	10.01959017
53.99019904	204.7181225	107.1548389	95.96257527	15.74990725	43.4052072	20.40555555	14.37301763
12.84644381	235.4750715	116.4373567	281.123459	16.15988429	168.356452	13.78039695	20.431217
39.36136205	126.217681	45.78672679	28.66403908	9.149906407	57.43948063	10.10668937	9.623779367
77.18647957	140.3772057	76.42285988	16.31948572	7.3398636	83.2293092	3.290315079	10.15046484
13.85433571	162.7120335	93.31283955	194.5393908	11.83182079	34.94751642	18.38764264	18.23366558
24.24790939	190.4562058	62.95124649	70.8172403	8.728421097	118.5931636	4.382256639	6.152632324
25.79949693	157.8084169	98.65986047	62.79689957	11.68998745	34.14591835	17.63958246	11.93495411
29.44640016	115.3351703	68.23807289	50.31258215	10.33325862	59.02813023	5.547104371	13.35101323
22.34006849	198.4378152	56.80256515	125.1822311	11.40648171	47.78704268	11.39375585	10.85213272
32.08539277	143.1399884	84.53433411	181.1121395	13.77606564	34.796241	21.73315064	17.18260939
27.59228346	141.5260755	63.07970024	93.29182184	9.525907013	52.07350335	8.236858855	6.878820556
24.45339539	114.3952136	52.15083123	108.7435604	8.965765302	37.08587549	14.60977173	7.588874151
21.1692383	122.132524	98.25335256	182.7055951	10.99380762	183.0538338	7.714025497	9.969619678
66.11332583	146.9750262	75.23562005	14.29947462	11.09664696	34.29301345	16.41842206	14.46331396
24.33957351	169.7422414	89.80046031	318.3441822	11.48936463	228.0994954	7.499632226	11.941473
37.23589383	193.6666008	77.8475006	314.3342195	17.59047868	79.70973764	24.64627172	18.22511893
20.77316022	135.0293775	49.20352083	75.40511256	11.25175313	80.28983796	8.262659751	11.17629469
26.58156428	113.2631475	41.11283736	74.5048052	10.52910569	41.64743613	13.33973236	10.48796594
56.50388222	147.637044	71.10459464	106.5815545	15.48904704	42.3564713	21.85917933	22.90466331
39.89117995	226.0361734	143.4379951	114.5488887	17.21538363	112.366665	14.26578171	12.32655052
32.71497771	162.6503361	110.7506582	177.3016984	11.56048149	72.55185555	16.58335418	13.3569158
37.39137252	100.5878804	36.36641668	23.98145004	11.60607471	24.61765338	8.244208274	11.51763361
15.32988652	130.9216119	88.86117538	166.8329167	11.01236621	25.2592111	15.24328368	8.974984758
25.21763793	169.4904529	70.35387487	111.1305796	9.546877811	64.50154206	9.816692617	8.439358546
23.10671526	171.7690754	91.69252537	256.8442667	11.88854721	82.42811806	14.66697814	8.870842527

7.827492568	176.9485111	80.79543924	436.3875939	11.63556279	102.2885129	9.509933164	10.67284546
37.35001841	203.9167616	77.13202201	96.09268739	14.94211475	119.9592876	13.10435511	14.34772439
42.88532939	160.1331214	118.1902047	83.07871992	12.08642458	98.45935395	9.263511581	7.384094356
17.34377201	118.6826265	39.17844887	132.2326233	10.25027353	47.20893257	14.56444006	10.71666392
18.07685996	265.6182906	230.3295333	641.7861581	19.21903563	205.8474119	35.72609399	26.19740291
74.15811278	123.0881091	99.13234054	31.57387612	12.46954528	24.03006328	6.090673161	10.59333114
50.92239141	123.797489	67.14019762	100.6297042	10.06685937	24.64992255	8.023223673	13.13953313
19.72350864	138.5472332	49.89819542	123.6966303	11.41768185	67.07065434	9.959475048	11.63812541
6.408857627	126.1275714	59.3163579	336.359203	9.679419093	56.67160457	13.73941255	10.78808331
78.08141763	165.5755729	84.51213404	30.95045599	9.488079976	35.93961159	13.3161594	18.12606119
61.1703602	209.414464	84.50523595	165.2223585	18.74316765	51.95673849	41.81242956	20.77491717
15.43605539	182.0488994	60.17502763	136.0589299	10.39613213	39.37871375	8.665876584	9.026099282
29.77489513	175.8102901	45.70988652	118.5485362	10.61994671	71.76950941	8.122261815	11.93663371
64.46651854	162.8603946	85.66932222	69.36287261	14.96145696	26.55790878	10.16938745	13.1089317
20.93278214	103.6828258	54.13342432	87.25927693	7.568536688	32.51106625	12.24279464	10.87851607
67.86742828	113.0842982	84.49515177	49.46646107	13.60524587	20.5670378	8.373871084	13.74594673
11.00532066	67.30294501	46.91350508	4.867895476	5.180067665	14.10543956	3.642756068	14.18042127
82.54213146	181.8355662	152.6907374	174.4805872	15.72501176	55.85076692	12.8347631	18.29919149
46.27405066	166.2130538	80.83851716	132.8966082	12.24479202	76.46483583	10.39530995	20.1056095
26.30120155	97.07429692	38.20889039	44.83356256	7.802729626	21.23129567	7.552943384	10.71624654
44.82623582	102.4167291	52.42521138	34.71001089	8.364109806	44.39988508	2.53549775	8.15595463
30.01068544	140.5962847	54.87707284	73.77582542	10.55310506	47.36072107	6.125237213	8.717149722
35.05881337	183.1773485	72.73642665	83.33127523	10.3524467	69.23774121	11.34798614	10.49368241
22.40108769	152.6399832	43.43993316	65.69493256	12.56963617	59.06595862	11.2550671	9.913298622
43.13479133	117.442323	73.4053634	14.83421436	8.098539385	30.18229414	8.155346675	13.46616363
47.97055423	177.1543484	83.53776956	110.8366509	10.39826703	53.73267438	13.6631772	18.04605834
46.7979186	128.3645326	69.99627653	45.98556946	10.60904844	49.35612936	4.425649966	8.280281786
16.36630609	102.9698951	52.02403938	97.67606849	10.88888173	84.6577611	9.283573173	10.43365572
33.74192985	102.0529385	39.97443041	34.65758766	7.939729616	38.11282374	7.717507039	6.925037457
9.602388207	92.47440811	54.5533442	61.71428606	9.676057225	60.69171837	2.885699391	4.08711361
27.07200989	128.6596292	42.58672856	53.43356299	9.151469657	27.85672828	7.148300895	8.888787997
22.44500022	100.6636244	32.11212772	13.2933784	9.970162972	17.18475213	3.800334591	4.507404948
15.80980888	152.1616719	52.70167485	148.3128146	11.58110493	34.89765215	16.67470865	10.77156727
50.15378672	186.215471	104.4576589	70.5758155	15.17945498	41.59311467	11.44130052	12.09388461
14.2605092	130.3557928	58.44664129	191.6218282	10.14500418	170.8100523	6.923492933	7.5694583
38.25263136	150.8198234	61.5741119	34.36859207	10.11308924	48.76050828	9.350393729	10.20505385

34.77335198	107.9518978	36.29003869	39.41907532	7.556148473	33.41631165	9.965829886	11.47319488
43.12351959	185.4163469	148.2439463	40.478978	14.08880776	38.41173511	8.172804526	26.04783628
31.89837212	132.0449929	48.93143749	86.90797697	11.12752871	55.45258353	7.563389876	9.542213023
37.43539703	202.6318775	160.9764205	209.4936905	14.17351643	58.55629313	14.4336977	15.17476663
47.13727447	134.0997895	56.93901788	108.878688	10.91327096	33.01029567	16.5459867	14.86831229
83.44402094	189.5423296	121.1165843	205.7898889	19.82855479	75.5658793	21.02514727	24.09688145
60.79882373	220.7930292	89.47035006	155.2214798	11.0697545	52.62194713	15.58781439	14.48234365
35.62507072	107.2290273	85.05719769	109.019621	10.10378748	18.8004898	10.75152386	10.12944062
34.37246454	112.1571909	39.45845618	20.43745821	7.239741755	22.16059565	9.175953163	10.75478426
27.80391996	125.4901664	41.47546029	50.56400483	12.11591139	25.56627302	9.055644001	9.391598509
14.6708559	124.0269898	32.95655204	64.09772451	8.022434511	51.47336178	6.465193544	9.79185967
53.32271103	147.8336551	67.64393786	104.2405884	10.82488196	65.72957686	9.532413037	14.56910799
87.08270082	146.4783649	59.72087526	44.30444645	8.945374086	39.13880893	12.50190411	11.63671995
25.06551703	158.7951196	90.4787966	274.4613038	11.23173953	86.76758746	8.476803083	8.844782325
50.50700759	95.47183268	83.77181523	74.70996518	7.520582999	18.30741323	16.10702304	22.19995889
31.05864939	125.4844158	55.89580281	50.88480458	9.514940544	60.31105931	5.485255352	11.94733137
25.15824551	142.352594	56.58625373	65.61766868	9.878656695	54.32661028	5.857079312	10.31142361
50.08668147	176.0856645	67.16328244	88.76939532	12.16534872	55.29370925	16.38304359	9.431562431
32.56648586	121.4416911	45.2756094	24.70175331	8.913305607	37.22470055	10.69208969	12.31990544
19.0927285	85.93704228	38.14341301	31.6110475	8.489599626	30.66329362	4.212931196	6.458441351
38.37777771	169.3261097	74.93725342	191.2848136	10.90673703	84.07463644	13.31272811	11.8240902
74.86873149	206.1369569	161.0033627	125.7064645	14.27687984	78.14347353	7.802108151	14.56684717
43.56874501	89.49855544	44.94146737	39.96483956	11.47809204	22.59964169	5.014445362	7.582889321
23.43718183	137.3659804	64.85490643	35.19513292	12.98624357	60.26484686	4.935833687	9.090775178
59.55788421	194.5310412	122.8789914	139.6214187	19.02006393	70.68175058	12.31906222	21.17378292
32.03746819	196.3790289	88.00473712	112.2669832	13.27783214	42.84239922	5.4627395	8.542406297
74.05068281	183.1291819	97.4864727	65.82224791	16.4146268	36.76966684	16.17087837	13.73394954
67.32982487	77.09798218	51.19605623	1.24149448	7.128800049	18.04830778	2.296611811	9.618012011
31.59529131	91.73280777	40.3159715	38.18637372	7.288649292	30.09975573	7.36405395	9.933199882
25.84553996	122.6786349	62.91682053	81.46671364	9.609298593	36.62523831	9.485759426	8.060006335
15.98092286	140.1680541	64.59332545	124.2020134	8.982598742	153.0476697	10.09964137	10.97389378
20.08107076	133.2273313	40.24431537	100.7517312	9.488406402	37.63165412	14.5494547	13.61879261
52.41940202	144.7880503	80.92719924	93.85628163	12.3530535	77.64336455	7.711450803	12.27810587
44.21524042	129.5486479	59.84782582	80.51931818	9.198631398	33.51459689	13.53528868	16.11230439
47.72787257	158.7119505	47.73370317	108.3110038	12.45126572	50.56793333	10.46650735	10.4958192
96.58387356	105.1059158	58.53704066	13.00588785	10.51856153	30.9820144	6.966461767	10.19968853

28.07505092	196.8854428	97.01387375	166.2866209	13.48934913	118.9496711	12.84918924	15.09001961
34.20311466	210.9284472	103.9906219	228.9261352	12.40653615	137.4004634	6.492261594	12.49995994
27.89054387	155.6303289	59.42043599	82.69522501	9.918834446	51.39700483	5.65727457	9.205704773
33.99640909	204.5742853	104.0924681	229.3775119	16.37677764	45.17042592	12.56062565	11.75569628
10.96530855	80.80698929	29.29768974	15.66849941	7.243674372	26.373809	5.964037376	6.203708471
20.73394107	94.18043292	24.79743241	39.01788004	9.522048568	46.91029826	8.015214986	11.71685281
29.33212087	193.3620609	87.19642191	62.82988609	9.974924545	153.076643	7.646709593	12.03947925
49.74358432	123.1822453	58.71524118	16.38031784	11.92818734	40.66517333	7.989055908	11.89139308
21.70092414	164.8680179	49.15317589	64.35462739	12.94543567	39.01172251	9.772448644	8.261028621
27.02741956	138.9009701	50.36041365	52.34127536	13.55266076	45.19596929	10.46710158	11.87067304
23.57460853	186.7327206	138.0836164	141.1385236	14.67406882	95.62520229	5.367331247	21.72222845
16.48723851	157.9498994	84.04274017	244.3241251	13.5217832	92.38314611	5.492391265	6.99058258
41.0406462	198.252033	134.8789077	180.6777909	13.50062036	60.08172808	9.253870533	12.9465523
61.60518035	155.8858924	120.3739454	107.8386986	12.5024511	63.97533808	11.43437356	16.06552869
41.58248026	85.79053892	27.2222203	14.20177232	6.206903976	18.06555639	12.34333504	12.84117624
37.26046268	165.7112767	72.87899603	164.0811203	14.26663047	63.12219092	14.42494865	18.59826792
23.19044587	111.2162441	59.62121	56.98716569	9.605051474	60.8051473	5.884041007	9.329213047
80.10066672	123.4372061	76.43694264	32.19731899	11.11864272	51.26488881	3.128673662	9.640000511
50.06500385	162.2859209	76.99107904	84.71458694	12.52421391	98.33202019	13.84989419	14.21836986
50.6240781	155.6268551	90.35724502	68.03658085	11.69714932	37.28769674	8.778319378	7.520175882
30.10834793	93.27340725	41.07795282	79.9124633	10.3918257	22.57288517	10.61563496	10.10275608
42.40630596	122.2741095	36.23149755	27.77914656	11.19725561	45.22831828	11.03393547	8.892079947
19.42057901	188.4353992	125.3160591	144.6091968	12.31757919	176.2893606	10.90330599	14.13695129
44.92830464	187.4762542	80.30808468	101.4814063	12.1144617	85.62694159	13.92177422	19.7101756
38.70990135	112.226837	48.12902756	58.4968614	9.619310428	44.49110824	7.482526599	8.946577252
70.650527	221.6195393	124.1842203	245.9276865	19.46727356	124.2633835	29.09155694	21.57836271
52.99668186	113.4066733	54.15323013	91.40025924	11.70762882	30.28238761	13.19259861	12.50436239
27.46646419	187.2200304	101.2064675	126.0405946	11.37790593	98.51213638	10.85862142	11.74144697
43.28493239	117.8122783	51.02933424	32.05963348	7.577747215	28.34165831	9.20222054	11.31564902
19.51844988	142.7597381	59.33116907	101.6658995	10.30211943	54.42773645	9.775978091	10.03548903
71.25000294	126.0088113	75.34970797	21.59507932	14.23517727	26.69270043	4.88618474	11.41014314
31.40640048	77.25226827	26.59075596	28.91329827	8.105972758	27.06169511	7.345539033	5.691275361
29.93777019	79.88024552	30.69207739	62.12704432	7.71443906	26.26342649	8.619549028	8.626926465
36.8677737	138.0571542	45.21149849	37.34455191	8.385439943	44.19932016	11.5701345	9.10068834
17.19506471	101.4338248	73.90880714	67.08931179	8.213690453	65.07849123	6.775416411	9.903725833
33.66490149	176.1211973	107.2101069	202.4407797	15.3261498	54.16488372	8.3999824	11.00452953

23.65836635	104.4033304	24.52202222	49.27722291	9.596425559	43.43355667	11.11481814	9.570447155
57.67205804	196.7589731	91.34194246	124.8053306	14.19038647	83.55791372	10.1780465	13.02173762
25.67140093	172.8434571	103.2719163	102.8318506	11.39419132	64.11328474	12.88355347	12.02403143
20.11932421	193.3903071	116.6164731	296.9942578	12.78214689	52.72135831	18.83235687	12.35171831
24.23319701	149.7510994	49.37399806	122.4438571	10.09935269	51.08954484	15.43148338	16.55729233
48.13860134	167.0291031	65.51988883	145.686221	12.8708458	72.36253116	11.96822149	9.010950815
32.52505919	143.3794212	93.09622355	40.99879154	9.944093322	63.07800068	3.965358195	7.792915323
27.01503449	145.3690844	49.47663292	77.56194813	9.863061527	95.23933252	5.02621607	6.237163311
13.08130005	177.4085753	125.3116767	117.4536407	16.09250077	35.08241044	10.19034925	7.701179877
76.07818043	251.3561212	154.5633908	294.441869	20.96996036	56.06445959	31.52410831	26.89749343
19.60258852	149.7307383	72.4653668	80.91915943	9.676561061	67.33409833	12.36487005	8.725648808
24.87981249	183.9005316	74.42674547	220.5590095	11.85511097	129.840066	15.39333518	10.73768809
98.79784888	94.82189992	90.45953798	13.75765389	8.577882126	9.196320243	7.232195993	20.56359549
47.56005872	177.0757045	89.81416524	157.6165766	16.60208086	64.95723728	20.18412213	17.93346064
53.30850441	129.164657	61.39295954	115.8089332	11.30156817	28.82170242	15.21687526	12.6929773
19.57164654	85.49628407	59.84849256	61.92973343	6.667575641	82.5055983	4.364227608	8.381260902
26.64235676	139.9872374	50.47561838	47.24844925	10.77061012	36.53989323	11.80911236	10.30541364
76.16147065	147.1352571	50.99216563	37.33776248	9.780795175	27.53972285	18.04389794	13.453618
65.85783234	157.6734833	54.38895652	67.82108937	9.835676685	50.99834403	13.86022754	12.26517747
13.9455908	138.9757586	74.72253339	118.1588779	13.71038275	30.09775073	13.6686168	12.19656
34.58207335	163.606207	83.06037482	298.1507081	12.03233884	112.0936393	6.260976056	11.16680646
50.26715696	140.6154342	56.07506033	47.17848646	11.1222305	39.5188759	10.931672	11.93263425
3.141881188	141.9491666	77.31917289	168.6278073	8.83997927	216.1146592	7.838955361	21.82752033
103.1352105	145.4407503	76.96460372	52.85033311	10.52230835	47.55949593	6.804957248	10.74664203
11.0767052	99.16178248	30.93084147	66.44974011	8.210416657	40.42578946	6.017883748	9.131352349
7.717511813	162.6371166	81.10992588	477.4207384	13.13205511	56.13388791	15.57137332	14.29784662
37.8469502	163.8351135	75.92424909	46.97320119	12.5672632	50.75825933	14.03337632	12.90569144
46.01442446	200.1076724	72.09387064	172.7831851	12.30534939	92.91283904	6.504474709	11.85213524
24.11439497	151.2122376	53.12098708	47.67870802	9.197071023	49.95539927	6.536534152	7.088613043
22.41353675	117.8779683	32.21136847	113.8957196	9.413043773	41.59316333	17.88002527	9.984595441
23.77803127	224.0323556	162.3066114	291.3751118	14.05384687	151.7282913	5.781408399	11.57339216
50.8224597	144.1006543	70.6881581	62.89701913	10.87456232	78.70503889	12.84044073	9.932840476
33.41564943	197.0305281	130.2981731	146.9362844	13.14504294	80.07189702	16.61459354	23.82875022
93.31894595	115.6607708	44.72677295	24.5724219	8.81703734	36.78073763	15.83950264	10.70204987
24.12810712	91.70071062	54.85315959	4.173005148	8.193640277	12.54452192	4.201729998	16.51829307
19.35345057	109.7452463	24.16878024	70.70427034	8.336540947	51.43427991	16.88135077	11.38989739

50.39613995	159.2837666	66.85728707	56.88470756	12.01165747	46.2860589	13.16997281	10.11958086
23.17206184	109.6075478	47.41273519	62.52610756	9.303335779	45.74626702	11.54234081	11.8911254
20.82863594	204.8276365	66.951284	193.5321386	13.32674098	104.139714	10.00085319	15.04563651
12.85512914	202.875126	82.43910665	220.1640901	12.7966845	68.10699893	9.005987828	8.197362187
35.45331541	112.4896097	29.54665898	38.77182534	8.14776125	24.8100618	8.295928383	8.768166829
28.9833367	107.2491985	50.36493204	53.64814445	6.173896683	30.76808521	3.088140286	7.655203671
24.50981687	187.1369876	84.91899922	199.1507209	17.14242638	139.2581792	12.16009133	18.89411824
16.27847743	108.8571488	44.60594449	147.8923952	9.781746993	27.54355257	20.47651925	8.601504171
97.49507717	191.3255502	127.2050753	123.0841071	13.24878047	43.45126801	13.69899828	16.77464612
21.257477	82.20624044	32.37161321	68.50356419	7.729173628	31.48984302	10.60655081	9.444097919
63.23644217	120.4934991	54.26236468	71.50060567	10.19789635	24.63248516	6.169644441	11.57616844
30.26695337	106.8072605	51.2155123	16.47595686	6.061139409	35.76973068	8.081893488	10.71518061
11.61599676	184.9609989	58.54865011	163.5282781	11.06141839	46.85766185	8.598083341	7.67509004
31.82504064	166.6355802	64.01866276	129.4049976	11.70196202	71.91468802	12.34720456	11.90867198
37.34759826	86.77616092	111.682153	25.34526777	7.008362792	48.11950444	3.237693971	9.867337916
14.2828457	84.01893837	49.92808211	34.23296352	8.613708869	17.95995298	5.993738053	5.133123511
24.1367071	127.161134	55.44762268	89.37147771	8.387638556	68.90380998	6.032478114	8.111357392
69.92983019	141.9710365	52.23328917	47.06252089	10.53711197	35.16576404	15.50831003	18.50729778
72.17267954	255.7424675	148.0104673	102.1725075	15.19960694	73.98664024	7.94946535	12.34849961
42.15984764	133.8063302	65.893693	61.40435555	10.52238818	43.71622488	13.16688151	11.01080703
40.32586649	110.4174463	54.68283581	19.02679854	9.827660751	22.34037813	5.447232813	9.73982063
21.00426392	119.9654411	55.24333306	43.02906713	10.79197765	33.20153711	6.606385573	11.38645936
13.29703428	201.0970197	93.16927401	135.2846314	12.23480039	114.8605291	7.09353227	13.37078663
26.25338533	176.2579496	76.97856378	135.6337558	11.13799923	31.7694834	14.77808563	7.9537874
22.67404919	140.7360599	102.5928533	394.1774492	13.44758689	62.70044503	10.57091815	14.37889997
109.6011469	143.8007783	71.15017421	18.93956083	7.882246626	28.75912214	5.676663449	13.17863193
23.31479551	158.4143638	53.20475798	86.57082039	9.680247435	47.35713312	15.27655428	12.03500165
18.33445209	145.2530541	79.84602001	375.2473683	11.82442643	51.79310083	32.06731355	12.29229073
100.5443601	86.13399661	51.61300232	17.75529049	12.32355468	23.79273989	5.273029651	10.14321696
27.58224228	108.054069	32.07344041	58.23254517	7.114867264	22.41266853	15.61698391	11.44856158
25.80050526	114.3293236	36.70546364	61.37721238	9.622086179	36.2012214	5.710548239	6.747295772
22.9690094	210.073048	116.3240254	193.5968922	13.09972653	102.6740951	11.15340582	7.816745302
36.71138581	93.35852881	34.86969149	34.46429394	9.780849435	11.92411134	6.032516876	7.095605849
38.80030035	175.9664047	82.7019251	127.8783937	14.4414695	88.52636916	9.260146059	17.53318645
33.69292989	128.1218718	41.36493801	35.05653341	8.605962211	26.63335852	12.01230251	8.114581317
15.73361512	156.0914336	106.4640942	82.52851352	13.89826205	127.8327593	5.473543177	10.67340496

28.36864234	100.1263309	50.3449829	26.3906417	7.282551605	38.37222823	5.072581676	6.021714258
31.31483469	145.1201713	61.10171402	137.6121864	12.60255496	49.86474437	7.708382936	10.68749429
27.88723498	127.5199656	44.47514963	67.73849844	9.754093868	30.41450804	11.429552	7.98759078
17.56122186	107.3292336	52.73052507	229.6460707	9.09548126	46.77251358	15.02926028	7.865977066
36.15341088	125.724217	72.3033028	68.2956271	11.98748058	56.82112031	4.308858827	8.480431873
30.217838	132.8521068	99.52566423	106.8554111	15.03024931	72.09402605	6.120644809	10.67038167
30.24186453	166.4433037	74.45136674	225.1120724	10.9046951	38.64871397	17.42555968	10.16818298
27.20456416	228.0208501	135.9415503	623.6150288	16.47245404	219.466212	30.56423207	16.90602454
27.56270019	139.0328914	57.74438883	108.2205887	12.58256064	41.93990735	10.05387428	9.44558264
45.04785524	91.31781721	32.82113429	17.66883048	7.518393568	24.15851535	3.395091122	5.94482369
95.42716159	156.6088335	96.34193885	115.683413	13.90623877	39.90937628	12.16765971	12.09866028
37.16892892	92.74328153	69.33967316	24.87972404	9.613385457	24.62307409	2.13598256	12.5369498
26.02735224	158.3533335	99.25787825	121.7603709	11.78621071	84.07695383	7.875053545	13.57273265
24.35993674	195.0021936	102.1968645	117.8395026	14.78162469	78.93717699	16.97789664	10.11834782
36.12327309	113.0987727	47.66188498	41.77802418	10.24089584	31.5325806	5.569459716	11.93280506
22.97146379	150.3350238	63.65709244	112.161861	10.24530543	99.20245994	13.26741676	11.57336249
24.00924672	180.3730873	94.07158844	208.9729148	12.46612088	210.8379331	8.212236514	12.15252534
30.09913344	158.7638292	64.73308545	57.06672198	9.050573883	55.09323431	9.447767956	12.75924908
27.2572545	96.58293997	24.95731719	35.77631168	7.195437972	46.06493632	9.406084692	10.54653313
11.32245763	66.05146443	24.29972135	5.276680074	3.947218878	39.21250906	2.970078074	5.741561223
49.77495773	163.2751445	89.47230117	57.0834127	9.169277814	30.43440259	7.250243161	7.490535897
49.39052851	145.388712	103.9755714	116.4991823	16.6674391	47.39156341	7.136638016	11.68769056
29.77410231	111.9876308	70.33677156	52.63429981	9.981665451	15.21841967	15.48334951	10.28788812
34.85986955	188.5519751	89.62474692	179.9128781	16.11890006	83.31900307	13.55555078	14.85267505
30.69202445	217.70239	135.1739619	270.2605082	12.47131863	52.35624193	41.07090901	17.02961333
11.07791215	93.61114909	39.48163069	60.29272246	6.19700894	105.329298	6.500572218	8.177037804
53.23537999	132.5443787	81.8868067	67.27653324	10.73247202	52.20586464	6.977018769	11.48548597
40.07318973	134.5987191	65.26913365	55.7031095	9.141771314	47.55117202	7.867024631	7.796270023
33.12999888	98.54803274	46.64692908	67.56630422	8.30396366	25.82265512	13.36354806	14.03097274
10.72174666	112.225766	35.89337644	89.29342816	7.448608117	37.00006666	6.528065533	6.423566594
16.48018285	161.7917386	63.46911918	133.9703257	11.19862188	23.03506075	18.85076105	12.30153588
35.47128637	197.8739167	75.15118479	107.598751	14.99173657	102.757442	20.31218287	15.68589242
39.3671441	184.6650344	68.76572985	38.62023296	10.71203438	57.18578665	7.08921659	8.030157141
43.40354591	169.7710954	142.6447782	37.17549775	17.01259296	49.50755691	4.882168952	8.304448702
28.39248611	176.076178	70.31290303	117.317054	12.14730112	66.55155652	8.142917722	10.41906925
32.58944724	96.28949479	39.59247829	69.98327389	8.935285759	42.24530284	6.716272704	8.804866584

25.89548344	163.752446	73.23630682	254.3338613	13.2107328	85.84859301	14.61616406	16.9827415
23.90679071	99.05293818	36.56225929	18.17479572	5.325142363	32.93133199	4.579136437	4.022469019
39.93120209	215.3567308	91.89288136	242.7723477	15.97880762	79.12076843	13.92188094	18.39376696
54.50263198	188.735448	105.750321	60.94914098	15.20263342	55.81453097	10.81111781	14.50959898
41.32564442	179.8023857	96.92193665	156.6521057	11.96720639	89.16677191	10.07149196	13.70139254
13.33591965	186.6910042	166.0932816	371.0837728	12.97914888	105.9025635	18.42806549	10.91154863
45.34599764	151.1946303	58.10646256	84.40605005	12.27970623	44.45730328	19.82521622	12.76849957
5.264778077	151.7275794	92.14620045	254.5175034	13.24185659	38.9615804	14.33417477	8.436353202
31.78217731	133.2091685	41.19173242	59.24238114	9.62869067	41.87603489	8.459007541	8.96876151
9.954334904	228.7875713	126.7589039	546.4757365	19.95746801	77.80445879	30.8284447	14.41868098
21.32824838	125.5822646	46.09840071	62.60913882	9.716700824	35.14627208	13.72372345	10.44216854
49.24818863	169.2202597	80.40695201	134.6246526	12.60095323	66.98896476	20.45916681	11.66873221
25.62665893	181.4254176	85.71745765	195.0741959	11.64036712	158.4394193	5.573256154	8.908066842
29.11295125	224.1042405	82.73618567	148.0521446	18.9454816	82.19057424	14.50498178	13.96250423
61.62554218	180.2508128	101.021915	53.76916659	12.82696771	68.80702419	8.463569404	13.82894046
37.69559286	179.6942294	133.8021307	177.8380625	13.31317905	62.53759927	7.677277328	9.811621677
13.97854753	146.6590859	52.20405225	100.5223494	10.83288272	67.44769209	6.393066132	9.283512357
37.69366636	111.5303627	39.10119464	82.22845042	9.384822526	52.27635818	14.98392998	12.88753752
22.78927357	139.3241945	82.31197849	143.2501304	9.584520066	78.44270758	11.57890045	13.1367264
29.35312324	157.7429978	49.51256971	82.33238806	11.63940462	14.69510923	27.43877444	12.64562637
30.45548389	130.8932793	44.15935717	28.43436133	7.997852343	50.65919684	7.458635069	9.909995618
39.443174	157.0198376	53.87118358	42.37111694	9.773610936	60.61343606	15.47565468	11.34954953
30.0369665	171.8275271	64.47459637	44.40142805	12.06037759	38.13580072	6.775119819	10.13046248
18.05088114	168.5901816	70.50745361	169.5958912	10.7865231	105.9667131	13.3466337	12.66779505
54.68294802	128.2100826	53.32155991	47.19046627	10.20980549	37.09965479	10.51936985	13.13690186
20.85696533	154.0874526	68.65180536	74.23383204	10.30238716	37.51290883	8.544716697	9.583032955
27.10851545	85.66106298	23.93950905	40.62250728	6.515961576	48.78915782	15.05500118	10.10768247
24.64066026	87.49877973	23.63611788	35.79649219	6.738120626	27.81977443	10.72485581	7.87763112
36.07441551	110.4943234	51.47592073	33.10564212	9.527615688	59.69429286	9.443110041	11.16721955
19.10936255	156.5570923	49.61217679	66.7115533	12.85956342	58.43949322	11.78708062	13.76841494
22.53178914	171.1206141	70.23944621	134.0599556	10.70609949	58.77560683	8.876395035	15.25424638
13.56453453	54.12384414	40.53008918	25.13786044	7.631567819	17.92864557	4.29364587	8.10961949
26.59538824	118.8264694	49.32857814	103.494349	12.1195311	48.94683737	16.90875517	11.5613643
22.93694823	198.0279364	103.3396199	203.0427696	16.66074284	82.76993405	8.642645865	13.06545514
26.68992739	131.1251011	56.03197864	94.17352631	10.75712008	55.23518021	10.59569037	10.26357894
38.10969927	220.4380468	92.71704604	106.1655661	16.67490326	63.31438178	21.52908778	17.51997903

24.35789546	138.7543202	74.57397313	145.2155125	11.45637248	54.26815879	11.10735124	10.00637901
34.46831501	191.7395346	72.6760154	41.0739116	11.30406577	49.04506437	9.234570781	18.92896279
13.54396836	118.7732414	42.88215757	193.4239244	10.05263154	45.11050473	16.81067359	10.8371403
20.70337767	78.55892837	29.55383766	7.282499861	6.414068504	21.65402988	3.237178819	3.23926287
8.492533343	136.917388	53.137506	247.1279892	9.842234657	104.7358436	8.683124412	12.04404522
24.46173779	177.1826867	72.06099598	314.9646868	17.23448546	56.09219868	11.66651032	16.79415456
21.71470098	102.077086	35.2652301	53.10142506	10.47866672	28.44884353	9.397271562	4.160376525
36.07098098	119.2232488	58.50570809	22.96931145	9.268051846	18.44834782	10.30518389	11.87403155
39.43437048	214.441561	123.0449824	144.9446423	15.3905015	15.04848846	34.00537657	32.24399913
21.48947795	92.31971259	30.91703008	15.35469041	7.208249001	22.42161634	5.781447933	7.017819542
36.39373421	144.2597487	51.46150854	86.07517163	10.57604641	27.57690467	10.9937349	11.4835255
41.64287266	144.0801961	95.60394836	269.4224938	15.57970011	30.55353386	34.44296135	15.00850886
24.73645467	120.4839379	65.01741749	162.6385786	14.43372119	26.24989191	27.4510366	13.79660859
19.32133389	121.5256657	62.1739373	30.46041498	9.483002642	26.98819658	11.48078431	5.377254351
5.706309609	92.04825822	34.42091758	30.76405122	10.00581197	35.2536598	4.124526094	4.860114322
26.42384644	131.6446446	66.63431601	29.63532918	9.611447649	20.61992619	9.495594074	5.503764933
24.95556433	127.9517291	41.8247431	64.01400425	10.34101357	56.3023046	7.551358455	11.2967841
51.35093574	118.2493458	60.79718186	33.41042722	12.54811888	20.84310921	7.392573752	8.167381116
8.850175524	104.0934406	69.90615892	125.1034679	11.35680677	43.3454847	8.635353251	6.80933963
43.71699005	222.3377414	70.04986727	149.9230342	17.40465583	42.03069167	10.88119964	17.89421427
28.08114003	90.35797895	34.29766558	29.26173846	8.274616627	31.09646785	7.68017932	6.684793411
20.83622546	92.23723775	30.67405458	18.47547071	7.777286733	24.15187415	6.641441574	9.362675225
20.45264363	130.2797822	41.37461759	52.9568691	12.60261341	26.18796034	11.20988156	8.94583074
35.50837959	135.2034403	30.07209591	35.36543177	9.022697327	43.76778411	11.23048747	11.77332775
42.08926422	112.6052815	41.32028715	45.14004639	11.00988136	27.18020205	6.923861625	8.559633333
22.62508329	130.911714	67.35293463	62.7980471	10.10923718	71.75255423	6.576928278	7.141701841
30.63311651	177.3477907	142.7366972	135.4847513	17.39501702	24.21353732	52.34862065	15.29046484
21.84966367	118.4182403	53.00536591	40.67928925	12.30310551	40.33743395	7.931612048	7.671093677
13.87043471	149.253483	60.94246127	223.9165667	9.500749037	35.85857413	10.23359209	9.007224797
34.6616472	97.71263497	54.67891428	12.00435474	12.1131123	28.82255348	13.02193826	8.205396777
46.12822366	197.2152642	63.45078697	78.59690219	14.74707792	39.93876199	10.55767139	11.56876705
47.13514272	89.72011269	72.01528223	46.35104405	10.07277225	7.294716081	18.86768126	15.59207183
24.07361475	95.71990827	43.86500543	16.49407046	7.75585116	20.23217815	5.540542013	7.17442306
22.70481332	131.0265072	46.43570506	52.91097588	7.275793534	66.24626298	4.669473045	6.628447158
55.44639113	130.5537204	134.3073829	51.04884707	17.50667697	20.47563218	7.505828363	15.45998867
10.62855834	110.4572287	57.65191566	84.7100703	9.319661988	29.66304164	8.729078106	6.798784083

41.20214026	130.7880922	54.23296463	41.31747294	9.474865218	29.16696655	7.644446344	8.448093654
12.90574029	77.55871794	23.55539492	23.10059888	5.790358242	20.15281104	4.615045341	5.689372467
44.32825027	111.4244396	54.78265843	15.6425363	9.498279577	20.12293635	5.133174415	10.57437587
34.31319085	118.0476879	48.37669993	10.78254098	8.244514625	15.40983976	9.577515178	12.37748528
27.88787292	126.0035088	45.63408499	48.77350861	9.924050959	16.59266636	10.13691742	10.30424447
68.9857205	209.2164525	95.13662807	104.2816316	14.20910807	47.4467549	15.96813726	14.09564474
24.28034875	117.9519122	39.68018762	21.18913014	10.93127237	25.51486081	5.407882753	8.632516504
10.87989488	112.3875366	55.67890189	76.90263697	9.139515372	20.58130374	11.44676331	5.068896286
38.25530717	114.5903998	52.379049	26.00930297	8.072608346	41.47698559	4.803035179	10.61039257
58.13771159	176.3874743	85.09137906	84.38498967	13.51199189	48.25070735	18.10082193	12.34456334
15.85402027	173.9009342	123.8124634	68.80628097	10.00711949	76.07016673	6.72326409	8.914193832
23.12088041	112.6323876	51.49646036	56.86147623	8.852501927	27.72924037	6.260901409	4.51222549
47.95521429	140.5717272	82.116494	23.38945868	8.297375039	38.64163392	10.87306919	5.638829678
48.71815538	165.9244132	83.92765294	50.56431834	10.5986895	28.27493318	8.887238216	10.52273964
61.82634733	134.9701166	90.79017842	23.99784402	12.06134224	40.30261574	14.74309768	7.117890159
8.758749266	114.7865607	49.78027601	123.5330877	9.297258461	37.38023951	11.69675922	7.925098545
24.22897823	119.7946955	44.06031664	56.37959611	10.51725515	21.09725454	7.65173087	12.31759406
11.16921138	149.3404141	64.67725565	205.7903363	15.24589557	48.43918432	10.99394382	7.14302139
41.65210818	115.6334727	49.09480876	35.7103438	10.0207186	29.5601506	6.361219978	7.920466729
62.99784425	65.894034	32.35918433	9.744336947	7.273857921	12.15077735	13.11013521	14.92415608
16.01286696	95.50141791	45.81512619	30.18793567	9.458465588	22.79629881	10.00294151	5.237964445
16.81062142	127.8855197	49.79493933	35.53416671	8.219608571	26.73328432	6.152858672	6.311687591
12.09242865	143.3860666	61.31978347	166.8585712	10.99203775	68.73877461	8.183658599	9.436728276
66.20277587	104.1672012	81.75144066	18.09109573	9.934704868	19.67909657	3.318263056	12.49137302
32.26578339	89.45756349	38.01667452	33.39847275	9.422694451	28.88404452	8.356652404	5.5155791
39.03906348	143.427672	48.42918209	50.96553575	12.13197295	36.27057081	8.385455026	12.27396874
43.44670657	166.1127504	56.86482833	64.83459518	14.5402193	43.85079012	24.0046612	14.65467256
21.34269081	95.42420169	30.92597998	38.90587021	7.175049321	38.66399206	10.12012539	8.372453045
8.588258866	96.68530584	24.17475472	22.46613881	7.434435809	34.38857985	3.791720256	3.902955518
47.56299076	140.7600901	35.56475325	16.33803971	9.528176246	29.27974516	7.822224254	9.30422952
27.16664116	130.118862	76.02815524	121.5832071	16.26011111	23.11279557	17.59248054	17.84839191
33.40480669	96.74711618	50.13841631	37.21145857	9.117919774	21.01590284	3.900497672	5.258869557
21.39096204	89.38092406	28.29746256	18.40206643	6.744555361	34.91599276	5.209773658	5.346179794
70.46994227	182.1349683	136.6674854	61.18905196	19.06031058	28.71986436	12.53890428	14.6831506
21.20457673	103.786328	59.63088917	36.16605538	8.819942253	38.73660122	5.142674283	6.590266061
19.98861757	108.6564131	47.27281289	21.41277175	7.74564186	23.55821025	4.500268383	5.938855693

40.37150208	135.5005664	55.29022398	110.5455441	11.56634882	35.277199	12.96098544	12.29941442
10.71764693	94.07702519	46.8657709	23.37322257	7.106849229	38.27087135	13.4919338	6.551230832
20.17714999	117.9664244	47.42534538	44.82946643	8.974300179	48.095871	6.439753815	8.064901073
26.9340741	187.6444149	58.83199336	80.02549318	10.56163927	24.29799085	10.56171621	8.730599132
30.9345111	151.82061	62.07130598	96.88727977	10.69027165	36.35072094	14.35063631	12.48025405
54.58897945	126.6889764	43.42883292	75.63620993	11.80253666	40.75027815	18.92589622	15.26219038
12.34479383	95.02720412	36.32775807	31.35269699	10.40057463	18.47939556	9.276968965	7.36965055
46.37344321	96.46316682	41.71976456	21.11332042	8.657453712	22.77346185	10.4216092	10.63789354
28.21543161	138.8007121	53.54888169	98.53665389	9.960459034	39.5478032	10.35150694	10.06443795
41.46598372	129.5756833	65.96418168	69.8193643	11.92193636	25.17207269	9.985510181	11.4681957
24.91494398	121.1213721	54.58873643	89.28495639	10.23613198	25.87079081	11.28658565	11.86631599
20.61266909	139.219621	60.74179587	36.46123089	8.429564709	27.28410643	7.391789774	7.227205397
25.76916718	91.3615657	23.3249014	13.27396332	6.819717301	5.070884474	11.7649114	5.590271707
17.07219752	133.2944494	49.63068331	114.3867816	9.239568937	89.62523908	9.052200888	9.164259815
46.96833065	91.32311795	49.92900224	2.95698769	7.502125327	9.837674781	6.819631954	10.28702512
23.1180149	107.4967441	38.65027618	40.01598283	7.365418501	26.08960099	9.867103085	8.916585059
25.06777426	100.5430763	41.08003199	41.6360949	9.087043125	23.17968298	5.578683993	6.156894736
11.02155207	110.8933575	40.71187774	52.48279436	11.34327047	28.59777592	8.489075106	5.726590889
25.56189718	166.8752092	72.30358725	106.2216712	12.24534175	33.63256241	20.14387212	12.73283726
27.58762841	90.16333488	43.32855302	39.09540882	6.904152779	13.18636513	9.771190678	7.02704336
25.13561488	149.6325258	87.4176826	121.5938876	11.23999376	49.61521327	12.02403196	14.95421462
27.70846498	106.8736174	43.21517882	135.3160215	12.01311195	31.42014068	19.13494537	10.37786753
15.27816139	134.8864573	58.92856941	109.731671	9.816380731	27.41400666	8.176840838	6.166696187
18.28734221	101.1481761	76.49688693	34.74209519	11.29865092	46.0024724	2.740252423	4.546052769
15.72861399	151.5565723	88.0702869	114.7682908	14.11891408	45.90320055	6.603480697	7.854368843
25.04973194	124.0126727	43.37039545	48.03681461	10.83657559	21.98834497	7.985941825	12.17590817
21.46221794	133.0927451	52.0012175	61.9754738	12.03051916	31.17535482	11.10144777	12.04254432
29.74220365	173.4140151	64.86659925	289.4627161	15.31353562	60.06044479	16.09712932	9.093863259
32.21328088	144.2704414	50.03105059	57.06171128	10.29587638	33.6352978	10.11920079	13.41928551
22.76042464	160.0633315	61.88998525	200.2980892	12.55636312	47.4626089	16.44927211	11.12269262
7.444950409	143.7205347	48.52080784	73.45570892	10.61161412	52.96446279	8.850903584	8.344777101
11.26047099	157.1265189	66.21567682	169.67712	11.20157301	72.10223889	15.17699529	9.315331011
67.19526854	87.49593085	37.0376159	4.821649657	8.809797015	18.23118418	4.46909247	10.05915888
21.93370092	163.2989083	57.20015934	126.1043147	10.05351814	55.23287536	7.341386603	10.92984974

Selumetinib_1736	IGF1R_3801_1738	JAK_8517_1739	AZD4547_1786	Ibrutinib_1799	Zoledronate_1802	Acetalax_1804	Oxaliplatin_1806
151.4465651	6.857005559	27.63226222	19.82489112	49.5999802	52.52749125	206.5844972	203.4938599
79.68093104	3.430023065	10.27876158	10.79444519	168.8959572	36.7032056	153.8532779	161.1968389
228.7109366	14.58674227	60.30788226	45.84105512	236.990875	59.32703617	314.2954094	236.6415124
36.62130703	3.451485646	9.749495226	42.92793339	177.3647423	41.92309878	307.349637	119.4868836
87.35415951	10.31136829	59.21275266	26.33652765	268.5962302	85.37707207	310.4179829	355.9081655
89.30784486	1.294434103	4.449708349	6.56286437	86.93961651	25.7296575	183.5781538	98.31476586
9.377666484	3.176395779	10.21731074	17.24373438	90.6397731	44.51552902	94.22279891	85.56213155
124.0139319	11.45042267	49.17910838	28.57015221	195.7770418	57.05738672	116.5970196	198.5995535
77.39118133	12.09042536	37.94899449	41.25379555	340.5806865	86.98341862	147.8487262	309.4607821
57.0134886	3.957257728	10.17812223	11.33335697	31.5589539	23.73194262	139.235653	114.8596516
79.51397602	7.546218459	15.05016102	9.764904724	63.63692724	28.74243192	122.3017844	95.42817787
80.14065404	8.882923915	19.11552544	9.339401236	78.19894947	54.37337956	72.80661834	286.5981433
47.28675012	6.820363285	19.32536143	12.63271977	37.08313695	50.67124398	83.44278273	193.9450103
168.7442411	18.7009358	22.62403308	10.47235335	110.8211138	54.7255794	619.160518	260.354924
45.90873352	4.774401345	21.37099663	29.59391502	141.4278734	43.37370663	75.58649269	153.4101961
80.50919144	3.373381322	9.807997511	17.8216727	64.32539713	42.1901824	134.1141441	168.6360554
135.4908165	3.64348967	13.06934288	4.537225281	77.83645099	21.20083254	101.6909676	63.11768084
12.07625202	12.98184723	43.76075726	20.11729153	208.8706986	78.17546881	74.32128353	163.9267125
62.28786395	5.702105711	33.10630119	35.79278995	216.7182484	48.53410672	337.5372813	253.252487
58.67548841	1.313606645	7.93645238	14.29818892	129.1622381	24.31760872	126.8339173	129.5374179
55.10678169	7.267579053	18.12961719	28.27273662	134.5965658	39.97988419	199.1178444	142.1501685
124.1289593	19.40525916	48.54272413	27.87851624	168.0316941	54.29065781	40.31615632	143.9152911
23.95190885	2.719923945	11.96722023	25.6141838	85.91566899	42.49474171	193.0150654	74.42291003
42.15731165	13.54679264	31.14035766	39.6550637	139.8035104	67.13334784	223.4211911	217.4654031
54.70319185	3.971253173	9.359471816	22.09836631	79.08934079	40.54627389	88.98742173	192.3588485
76.59862487	6.078420731	15.83993533	16.14796113	103.8620846	46.3669274	88.34937733	204.1449854
134.6029132	13.98428305	65.38970975	55.04699424	152.8450943	59.56648	165.3421436	271.633244
27.23898921	4.900695056	17.38765805	41.40932849	127.6247237	67.76964584	208.0692314	160.3756705
28.25005135	4.117963225	14.56445999	21.79830464	112.4586821	43.90193685	291.676144	111.4375282
247.162649	10.67742587	31.43137425	10.45634372	46.24513101	44.45109135	123.0657419	191.422211
48.49017004	6.090000511	21.14335521	18.04401796	61.97744179	32.97607277	93.33452721	174.0327144
102.9374807	7.553230491	36.62958332	30.98161238	118.0249606	51.63627454	239.7489785	268.4228181
70.92994574	7.378648288	42.47105985	50.37633948	179.4204938	85.11678108	115.1346417	446.5209478
176.3476356	7.948547933	30.02607349	14.1532686	102.2623939	38.81519113	242.6145944	180.5233631

34.43684306	15.42995912	33.80884546	32.70253323	66.30981203	60.25165345	149.6272931	167.566323
88.811459	6.501014403	46.99020866	48.59562985	143.4223263	62.19333839	390.4585315	323.856546
72.9323572	2.286158034	10.81093564	12.91497198	60.32337015	30.38736961	148.1383732	118.6222024
131.4035691	11.1313454	60.25452286	69.01422338	108.0912914	77.16942865	361.1746558	599.0740163
59.73214701	8.812912407	26.78455764	30.05975248	141.7546519	49.33790649	114.1476993	121.0820974
139.9418743	5.355467645	25.73369786	19.33392478	27.68884703	42.93419919	24.25261522	234.2872286
441.7619563	18.1482999	45.83015503	11.47848512	132.5593776	59.03475903	111.3355775	335.4350938
35.66531916	4.373959372	15.92268831	19.57599987	172.7707841	44.46588235	389.9845305	166.8788737
59.56753583	3.817781917	17.2993109	17.99455541	66.6239074	58.08463682	144.8053746	154.6801212
82.59304912	7.110089466	20.97262357	31.56075275	28.10665715	42.00897034	71.16877644	170.1784402
46.29565026	9.258458623	25.2487523	15.68628499	97.74303912	43.37613784	84.36529605	66.28930088
708.2574656	1.373180776	9.037464491	2.489118141	38.51563438	15.4558293	80.81406081	211.1485358
36.27714491	3.248713759	11.93335269	26.29869492	84.333945	35.29395968	225.2494965	101.8300392
81.51154775	5.519059418	20.33048848	20.55960807	208.9451829	42.82357428	152.0885662	179.0119773
42.46935168	4.019760908	10.71310877	9.209769148	92.42653122	33.13121917	206.4634084	66.37456934
53.89745325	5.23316817	15.84510384	17.35484304	68.01925131	47.64241839	48.05627435	173.4559688
45.99394838	2.620929955	5.276212911	7.270605895	54.00793795	20.79015965	114.5866293	41.2865979
40.46052132	4.196726954	20.78740907	28.56370911	159.5522026	44.27912895	272.6417131	269.4520738
57.4367905	7.72478227	29.69468337	25.71352042	82.64336129	42.34764589	112.7906944	232.128371
58.27055576	3.720160598	14.64897489	24.21014529	106.0795855	36.36997187	159.8740927	230.3919751
97.37688553	9.116580827	25.19690495	16.98547207	168.8717759	42.51811452	191.858229	132.3712807
56.46854187	13.80691488	47.87823128	53.92283095	221.2921932	71.73280784	301.7275223	252.1005529
84.8066544	5.350066114	20.81756238	12.46737003	102.0639657	32.20390308	315.9603735	135.6966827
58.00645259	2.178268888	10.23668371	14.36688637	53.06988101	33.83545739	98.59238338	92.62878001
163.4618017	14.07719375	58.48428675	29.17270463	90.53389822	83.41660159	163.033019	187.2512052
89.19883947	8.277891666	27.02864767	31.04256677	80.58031229	56.51786505	74.64838572	172.8431639
209.0268032	9.06760755	29.28618889	27.02728631	102.4703382	67.46845487	197.7961326	479.1452744
65.24849748	5.271442417	19.24698604	20.91134801	44.58132527	37.47043083	99.76056671	223.814953
166.4756962	16.93554688	87.31378292	62.39178901	112.7787316	76.61575543	96.82641659	739.7016467
111.8905218	7.7142227	34.95009711	12.86353599	135.8985695	43.96424566	107.1297059	183.6743318
38.78315015	3.084241081	13.04144859	18.80449203	85.43009839	30.17232072	103.5762968	81.60258
43.82207671	3.30099569	8.954330844	10.11302351	67.13263481	34.02933379	222.2523435	102.8078464
28.09524528	2.001308649	20.67700684	29.26348904	35.72336769	53.92963327	14.28681135	339.8942859
241.1744194	21.66139137	96.0742235	50.29376804	371.0649067	143.4373989	732.4409301	418.4161889
32.95422316	8.255357245	40.68072776	34.19208693	201.3872921	58.87722389	695.8807533	246.3307213
172.0187575	6.620799219	12.29087987	6.923902702	32.22222229	33.17265382	171.0097785	115.780097

76.58710055	7.570682188	74.01860838	62.6348332	214.626393	72.38285367	419.1440362	274.0140175
174.8776953	11.5759811	18.5853173	8.82766999	25.05151248	24.74714594	94.73974543	126.9998471
34.74199315	10.45299044	40.2843332	37.44048337	62.8307465	61.83848994	336.5839053	185.3373945
96.1273571	15.84428745	72.17285784	70.02569756	294.0816024	117.0446653	639.3700897	511.0515362
43.32194608	5.699565879	17.34951497	16.63427929	56.52044923	33.19246403	67.0241301	145.753581
29.60998144	2.871319451	8.710179787	12.30343439	69.65000744	30.85586602	171.5545847	71.00230232
89.79285489	3.410865286	15.39900816	9.70606104	26.24429647	28.89865646	95.83328901	68.98128595
47.27580855	4.850370216	17.42214222	14.32897304	103.8051504	49.51577373	93.78963843	100.3991791
353.4921242	7.29242782	45.57583123	24.28701225	63.16218944	53.34458071	39.16110857	456.9530412
147.1375671	10.63303954	17.87562247	11.86463454	31.70632594	36.14330729	83.76096159	207.0975745
96.12725085	11.20891621	58.87117942	34.69491539	326.4492305	81.46839091	486.9812089	347.5364202
49.83451189	20.03102355	42.6088581	43.77205422	105.8968276	72.67042987	214.9420399	363.3352686
25.40204575	3.670656094	12.60251131	17.40501855	47.71188908	33.26959364	86.18406642	108.9059782
50.06669754	2.91309604	9.950219742	11.78716391	84.23279268	31.01093995	219.1071908	78.44133177
108.4386079	6.67081791	27.30305458	38.24181469	88.25093242	48.79719032	97.22063477	256.4918878
142.7106795	5.367848602	25.40578346	24.6197288	82.73536225	54.44895585	209.1713044	174.9999825
185.0257507	8.615798738	17.54515381	6.493349451	185.9275002	30.3652001	409.328304	163.554521
77.07055514	4.525796086	13.25993968	17.47384443	48.53751017	35.57187044	189.4050141	126.189704
80.71468905	8.281967715	39.63002929	28.32844266	217.2827235	61.75783331	131.8692409	333.0397948
36.51453704	2.606188433	12.45507192	18.80083574	125.7011029	35.8960434	262.3165567	143.2025729
135.5439861	18.35624592	43.33414918	12.90371533	141.9199664	45.55687991	236.2866875	111.3946972
18.23228546	4.218940671	12.89849864	18.21730535	72.51850539	31.9606559	45.6496939	42.55364452
32.23881735	7.100509701	31.12311483	28.8221439	136.9474113	51.69922775	245.2977435	99.10632777
37.8915586	3.430808792	14.41533026	22.72530627	31.67185124	33.96389263	61.48431562	139.1204505
41.28408103	4.365325247	13.1467766	20.89882381	39.8866913	34.65799054	75.88331483	68.66215772
46.90736124	4.519722988	14.50307578	22.27936746	130.130524	39.88255807	291.6724067	125.0869589
67.95363551	5.744550154	13.1180677	9.698183642	101.0360847	36.30800197	133.9029296	72.42720287
16.96324824	1.429760368	9.920287459	17.95942905	257.4362135	57.1584716	363.3841371	87.79657815
58.18860829	6.963331145	24.4005297	23.38157762	60.5552925	31.94386153	113.6224869	161.2789301
98.74339209	18.62341132	34.78616743	36.02497983	85.47037724	38.78576379	243.7090905	300.9902888
152.796326	10.38443883	31.81306172	20.85525572	97.0498479	47.34493481	84.18621999	368.4801704
46.19872114	5.32179157	20.34896759	37.02212885	92.9428357	59.10085802	230.5775237	213.8073738
486.2397364	1.786495427	9.737750291	2.386822929	17.12195235	18.9176234	8.322068855	89.81721874
56.3494819	3.846880826	15.06825199	16.52956422	41.82538727	32.8435112	64.30819009	73.98768345
203.6794667	2.996022414	19.8057245	8.891230803	47.22752823	36.70395964	16.33589626	177.8453417
36.51178627	7.985435611	29.58508439	22.89264464	113.880741	62.766666	248.6391409	185.6842183

81.37140311	7.025008294	12.98798675	25.55177517	93.15066582	45.63878195	138.255469	128.7670805
48.09450696	8.726211219	17.81823523	20.74723884	80.54821453	53.22260617	233.9160868	186.7493889
54.07254151	5.827196443	14.63647238	14.36136765	71.0676774	30.48024522	255.7237196	78.54358363
92.37677245	8.633545226	55.00440623	36.70529935	106.8154664	61.45813244	199.6061987	205.5294093
22.19477739	3.743664305	14.0497202	27.30550865	43.23502574	40.25188711	61.54757768	184.839323
27.00301028	3.422429751	13.41398691	20.11605975	74.53212861	45.45768916	169.0655348	193.6946669
91.79936596	10.77748754	52.59834028	58.00513906	192.2726392	87.50094007	280.2026341	765.5392788
49.62013748	3.394650094	14.64317596	16.05810769	72.27820174	34.43895559	54.76838997	86.32288506
88.14175161	11.47628243	29.37724328	24.52658326	182.9658819	65.13658571	166.8455255	185.8208408
80.83320914	5.0096372	15.34355858	17.80801606	105.2349536	43.82575149	168.5461068	153.0939405
62.79683407	8.187952304	29.74476072	17.51236697	52.93746864	33.54329698	151.1596217	82.62424476
128.1220725	37.5120114	91.03535013	45.71697681	174.7309594	53.65864795	292.5949406	260.1433057
26.06748849	3.005344428	24.85908273	33.60585181	210.1510749	75.88790342	239.7956288	212.8130157
78.26945636	5.214116017	18.03906736	20.20945222	91.91071613	28.6136754	161.4204754	108.1016728
274.2225677	1.296283833	9.791840959	4.628049179	38.30472565	18.37927027	204.8724294	112.7876808
6.255358632	5.960868234	18.30066779	13.98455161	111.6165301	48.49288703	77.78674198	119.0763505
30.701795	3.589198356	16.82417979	14.51088796	54.07800108	54.31129594	31.13411288	200.4448299
67.44285568	4.802056838	20.85529557	11.12288631	59.1755804	40.17858642	33.64176914	244.26056
70.12408615	3.645644059	21.88574549	18.0529558	120.1755773	30.08181628	113.0218535	125.6504693
18.99770378	7.428036498	14.64127798	19.32967964	93.73287363	42.72037342	127.4187888	129.7099712
124.7181463	11.91966569	29.14736581	14.28376845	95.95440539	69.72924043	235.1457303	351.9943029
43.87089358	3.940575298	17.02944216	28.19325769	79.85764923	36.07220222	231.5040351	92.42160044
51.10846559	3.540586791	17.70761475	14.51199786	51.10576452	34.24443279	43.45476571	89.55424528
49.2645589	3.389803605	25.81330456	27.10496877	87.42373003	40.05240552	96.13057189	223.9527459
91.89938426	10.2233675	16.87196082	22.71890955	47.0442525	31.92428942	29.96710915	213.7542389
46.88962575	1.941782808	12.79600087	16.31543222	90.98835279	46.86604975	134.7179633	241.2847451
36.40746679	12.39958836	40.66360051	73.21143227	221.1368174	85.37365309	502.4160011	205.0898919
35.53008335	3.575475761	11.43384383	18.32227373	139.3292157	43.54493165	229.7090629	128.2488639
42.93578643	6.060079918	18.91794135	28.00762367	80.94744392	39.36349485	194.242243	56.4954244
115.9574572	15.87023177	35.92471388	19.40323076	38.70499529	54.75637028	163.0613182	397.2536323
102.2128479	14.60815139	142.0534046	55.45315458	185.4739583	66.19943679	273.8171083	387.6741284
48.43259655	5.82048207	22.4423253	33.97780229	116.5211949	46.81866554	119.3183055	175.0643492
127.1799764	5.56328242	13.86366485	11.43291634	120.8993495	28.09261538	152.5583552	108.1073246
16.23969596	7.448251701	16.59927924	20.35454912	62.98502698	45.6821134	198.8442524	149.4927315
38.08192793	3.57740731	15.5462272	31.22777006	61.74915866	44.4359842	81.59542777	133.3559705
51.26397995	6.739573688	21.56299146	26.85852128	84.9004129	60.24700371	62.59683563	240.7515483

12.04383337	2.283396305	13.97889017	42.59023892	157.1731164	62.98811115	178.404541	125.6447302
62.4149292	4.900730316	21.72394592	31.08599786	115.2634372	50.25923036	133.666949	224.8052053
120.3716567	8.991886	28.36362939	39.25807083	36.49491352	63.34385856	95.76793271	504.5857808
34.69795165	3.055733477	11.5820761	37.84627143	134.9625692	49.10783063	228.7495604	86.35151326
83.2766386	18.69944005	87.67963571	42.49850507	193.4466628	162.7988454	344.2618804	1281.630946
298.3135647	12.51205442	36.14229401	13.70451458	49.81991527	39.91949133	97.19203782	159.8835026
248.6642084	14.1789059	48.68963044	19.71195498	178.1191076	52.35240986	230.1005941	254.4346848
30.51076982	3.853688222	12.19566663	21.51688695	160.6035094	42.31941748	365.9423912	99.18174203
3.808795724	5.156624359	15.19460815	26.4914836	135.6193065	63.33929287	288.7995611	70.09778802
251.119036	10.69889299	24.71185169	21.66319841	85.43881218	39.33152276	305.4899386	335.7341575
84.0752554	17.70655353	51.76692215	46.7364893	127.8713133	67.67192931	541.2054403	222.3142123
16.96848548	4.009093238	22.42110003	27.18218041	84.52721328	49.96055327	97.47978101	110.1735196
48.86370633	1.958590467	9.421915607	16.14282554	80.06043778	44.1940987	142.771427	162.6550758
158.0156845	15.72605792	40.59612863	20.69919529	176.610004	45.1138996	337.2566107	145.5849646
22.57379284	3.420854294	9.2896217	10.4500877	62.53132202	29.00116994	119.1747355	78.54238411
334.5694879	16.83645645	54.76974479	14.07160484	146.0090155	64.87055364	100.7468969	235.1109919
59.25265976	1.408265757	12.9238793	3.202693944	17.21901018	19.72383401	8.009493113	40.51612388
241.0932039	9.508627402	38.96675822	29.40670201	94.53169336	62.52082553	232.323558	462.504296
95.10421951	6.213775692	21.88413302	20.46659073	103.503679	42.12431831	295.925523	193.8549887
62.67915387	2.871934044	8.589622947	6.512215857	76.41197422	29.5150836	92.32803377	59.6334809
173.0166388	4.688851099	27.95992106	10.94715236	31.20702987	44.71959496	11.3979043	126.9715348
46.88324464	2.770759617	7.056140149	16.98555975	48.93361891	35.9804792	94.9349264	79.09254826
58.69879272	12.23875196	31.93159925	22.43190575	98.090181	58.89824179	300.3776014	200.4495289
38.22253144	6.192961325	25.57231334	20.53806797	100.9690749	38.13357397	274.0397139	87.69028312
171.5841437	10.96338969	17.70905285	9.991012599	33.62966999	35.09342067	48.84113914	154.7754267
113.7585171	12.15862292	51.11753581	38.1373651	131.1413233	63.19819234	282.2965645	173.003186
126.254684	3.852857695	13.74281226	19.36116087	55.6954195	35.92748489	79.81694876	198.1796328
22.72625247	5.35728618	19.54268161	21.65169981	61.52035516	58.52276027	299.7013499	368.3128201
95.47308793	3.203212002	12.84706498	16.18688488	51.10144679	36.00979948	72.19028478	127.8390282
30.2064953	1.817740644	11.64977371	10.49467255	109.3280973	38.90250711	85.69934212	95.15268346
29.38986391	4.463128456	15.42553452	13.81252537	84.74738063	28.84269597	82.91099586	44.58873432
71.94071289	4.384454719	13.84501185	10.89842789	48.49676132	37.93527406	25.66157134	116.0087917
11.23742598	3.88409918	10.98999415	13.65496046	108.609091	39.28823142	174.2374727	60.75739162
202.3708845	8.702876076	22.12059892	11.0394466	65.7937125	95.97618732	61.1847455	400.2265332
41.93334967	3.353796419	12.57138274	32.88956003	144.9134234	52.40384984	411.5510938	201.9073134
70.10343873	3.353103363	15.55285905	19.15263947	64.60361197	33.67379034	41.51301095	209.684016

72.85697425	3.136816887	8.810188344	8.953116381	41.10472804	22.41250929	37.77884972	49.22925384
197.9051931	7.175543009	40.52611961	23.08119734	218.0903382	47.8368741	188.7950552	255.3855313
44.55221937	5.535628878	12.52733198	16.93047502	92.27336413	40.34649791	242.2552575	86.88327594
92.64117003	15.0227351	59.60236685	34.6449825	312.5764453	67.58966538	273.9125209	262.8988759
62.99899365	5.650581391	17.19130127	23.68157513	88.55251154	37.88439036	164.7620471	95.54827582
244.3316426	32.23054945	78.76673401	37.40779047	170.2976106	79.8794436	324.3237422	292.5402788
186.405592	9.671212232	42.29963658	35.47835856	87.20239836	59.82111852	261.3765857	233.3509266
157.2785105	3.937723458	13.95629379	16.83334666	57.42468291	48.06690693	181.4742205	450.160951
41.81535428	5.267440652	11.6719745	15.14321286	35.50242239	30.78314652	104.4648625	87.13690018
40.63842731	6.440703978	16.98494509	20.00599971	68.54679422	45.15979183	142.7332599	117.7463424
28.61375585	2.741007548	14.25463263	11.46436758	80.70753286	36.22372635	60.06088592	64.70812053
83.97173546	5.04686172	12.19809091	16.4934663	62.46276434	40.15000533	197.2657139	226.6364603
306.663039	6.96719565	35.19633282	14.41589288	31.98829146	35.89644731	16.98350279	205.0993558
44.09977219	5.368605553	19.11059661	37.69556172	121.4644523	59.40084798	222.7054319	158.8470012
235.1740476	8.668838077	24.33008685	5.366092396	214.198099	31.41225586	374.9894582	86.29337665
55.71550804	4.750432093	12.635819	9.899362641	86.8283845	35.91839203	95.9607238	95.46620592
40.46206963	4.340857063	15.17994531	22.02954772	50.5097033	33.52261116	35.07383293	174.7464759
72.27857537	18.54958332	40.41383458	40.73802089	84.85720988	54.79018886	65.00128126	136.8781289
35.76342116	3.864031385	12.766109	14.55560445	44.38526456	27.45352561	32.14251104	101.0033179
50.27664651	2.104774565	7.407370454	11.4899489	20.12659009	27.16087727	10.38037155	122.0063052
68.08376459	2.421825793	16.15022862	35.91500683	57.20015359	52.68302281	310.6027388	172.087885
159.4733424	28.90376037	73.05921556	39.26419889	85.31476906	67.15981068	44.4107301	459.5047707
72.90506815	9.669771618	14.69797252	12.63207711	58.66751874	36.08791613	151.3434637	55.35424798
57.28908905	8.412127358	26.51128938	18.73523632	119.5951333	39.68052496	173.123408	180.6835206
186.6914471	18.42303061	54.08985914	33.01805375	225.8468268	77.60153483	126.2122538	416.205948
77.02114264	6.095892596	48.08111523	29.846684	180.271794	57.83447656	159.4655199	217.4473454
213.9014363	13.36948794	84.31376472	30.47373731	123.9112051	65.73466151	95.47127708	240.7627627
530.7698201	2.194205105	9.58630582	4.535624854	103.03979	18.71312926	324.1651662	117.9521008
54.17651263	2.018678646	6.982860583	11.78084427	30.11339803	20.3129372	69.62407774	74.52279757
30.83448668	3.731318958	24.83793529	29.5391334	76.92930786	33.79404814	62.62430769	74.59911943
28.20472121	1.909755966	15.3182605	23.23734109	88.11092672	27.78636843	173.2348361	111.8592489
35.01950158	2.188818944	12.20659449	14.98664241	48.47968879	32.65338136	129.7716399	101.8152367
145.2118612	10.58835828	25.61474754	18.04251869	40.89278352	52.72581268	100.8369342	254.8466331
65.14598021	4.590023213	15.10812271	15.72185323	52.67122236	27.21708686	174.7090076	72.07403156
67.50240857	6.56600363	23.85826264	16.93550848	56.27750065	47.39271437	42.2322562	160.6464707
291.8338842	10.49057364	27.12868732	12.19154972	93.12670949	42.87399493	348.8676863	349.1675417

48.20497635	2.666928085	15.88250098	28.89527112	145.8839952	54.28755583	219.1500086	226.60674
82.16154039	3.407291253	14.66783454	22.60013084	79.61773757	70.17811695	204.0048301	315.8251821
66.59725712	5.243110561	22.41018581	17.8362823	102.745842	45.33477574	46.05147312	165.5719255
72.86749	5.283871854	28.00643093	28.16105715	180.4235892	54.10062765	193.2307596	129.3217298
24.00797371	2.056652869	11.93755896	13.66047041	33.24536834	24.59659554	13.63919528	40.95425494
25.3723092	1.637768571	4.983690514	9.097600578	115.6178132	28.29290608	233.3954761	63.71339212
66.76075715	3.305702228	22.26860919	26.18889168	136.6381861	38.04857709	153.412803	271.2649328
121.243316	6.836345606	18.78290415	8.682955257	47.07058933	56.14641849	190.9451592	567.6191967
50.22160708	4.384319649	24.41168276	22.20188067	74.88706085	44.76652337	104.1650447	108.4130529
35.94992371	9.850156712	30.98084397	25.41745181	131.4634697	47.1393338	130.1267522	94.13626248
34.57194353	2.709079866	14.747776	19.07541905	81.66912585	69.35264052	96.37792705	475.2426409
32.94216263	8.936278185	40.57468806	32.46299526	89.1761418	65.4470228	190.7035521	210.8238096
95.35778183	6.022816304	33.70666882	31.25114656	154.4210747	56.93242936	127.4497905	233.6754149
121.3852828	7.595462963	25.27222997	25.07996365	64.1083649	37.91725301	137.4813168	237.8657145
98.5354574	4.004573368	8.436883004	8.857054929	65.498638	20.18680262	401.6276992	85.28288619
71.22122315	4.246833235	10.77028058	25.06556749	140.5931534	48.35510005	333.1829621	206.0347731
39.7284665	3.688981906	17.7824844	20.58751027	84.29772305	29.80429927	76.46214442	97.81649876
164.6853026	4.477607542	9.841643553	13.3449	33.75987154	39.51143653	14.96085626	245.3953899
106.1134001	5.693859049	13.32174152	18.09897476	65.31684535	45.28073737	118.5992506	204.1986129
115.106868	15.5414453	54.93389457	33.1147928	92.20263489	55.36657324	181.7972421	221.1317302
51.57880881	3.49388262	8.871081766	14.92373192	55.93887804	27.63434599	47.08170141	86.52534118
71.01320914	6.134215017	17.62952051	18.5197268	100.3164048	35.28663178	104.0732867	97.42769896
56.4580671	3.732855988	54.8199203	30.20752701	107.9320866	52.48077157	126.6688634	594.3155642
103.337858	8.347002492	34.59482268	42.32776366	126.7582979	44.8156161	337.3812703	167.2940758
89.11710203	5.2511538	19.00704302	17.86821466	114.8066536	36.02743533	137.0951752	94.34476872
106.6495629	12.71317816	43.11483376	39.69173579	195.7692906	84.05834683	368.8591714	396.4448093
52.96910217	4.363071622	12.21045784	18.79344095	61.69159411	34.61914987	155.022173	84.37003249
51.83832438	3.830169739	13.00824442	17.33148595	56.68543735	55.10969855	123.4202793	255.8342567
75.52353248	3.391805912	10.07978446	12.97573379	44.82380538	26.00446511	122.1412546	101.7523405
27.17962236	2.383577073	11.80348775	19.3118112	102.026009	38.04881203	111.5465719	83.40164105
212.0001246	7.080325281	35.64755399	23.91439376	65.22195776	38.84582967	29.20581968	217.9023402
102.7693814	9.127336521	16.35613799	4.229231118	54.11603559	25.55558381	222.1865566	57.61481543
37.30797651	2.314745295	5.983855754	10.15293035	49.76429073	21.01538071	88.76728776	70.63370864
43.68406205	4.769693267	14.04925237	16.21512344	70.43121729	32.96094175	77.99314078	104.8887652
51.87176989	3.162119097	19.65392634	21.46476346	114.9010184	30.30698124	87.00428469	116.5976182
71.48708328	6.963066481	28.38447561	21.43371999	79.37561695	74.37422556	46.40028647	212.2851154

18.56039858	3.564433111	9.116712328	14.34754889	66.82224765	27.22850661	174.5424351	78.62389923
99.3238035	11.03526443	31.27943953	26.65320276	56.71744323	54.63559873	62.06399505	247.7338218
53.49994574	5.36435691	40.60781844	39.90738578	163.8532762	46.93613823	84.30404031	154.3594761
32.5288601	4.56343764	27.00414797	34.22686113	146.7289915	60.59071819	109.4501662	187.3917268
34.78926896	3.683732676	15.60556536	20.42517408	110.7491852	39.00863583	200.846953	100.4809566
80.82034715	10.1741134	22.76877575	28.42770423	71.43367628	50.51019923	112.4281508	180.8456848
62.28228609	6.165116389	32.47300526	35.34028511	110.4309878	40.90557979	77.7510678	135.5085504
83.27753541	4.977296997	16.43746426	13.75213812	63.92530466	48.31731508	180.0034096	145.8823644
77.63351255	9.526963669	51.11237498	17.52830868	202.6742716	61.84315671	101.2258745	208.5383221
145.8967457	29.64986598	64.52529981	56.78120368	184.8973177	94.08011513	386.4584398	356.5418554
40.3405069	3.607619733	20.7368176	26.2250797	84.35135712	39.46874244	146.4550347	94.33876613
55.19839242	6.660557247	20.21499138	38.38184162	152.3525981	54.9425565	271.2494184	227.0589002
464.1026581	5.187622355	12.20962921	4.440257658	108.1555596	24.03528937	207.1151197	162.1821013
106.5741048	12.62625035	36.32353044	27.27027011	154.4496397	60.70653455	195.441935	287.012155
132.4381004	6.850158852	18.63158995	14.29164854	107.5384506	43.71539253	328.3326433	174.1657406
35.47069384	2.71937413	15.89122531	17.05538488	85.51489304	23.23085233	175.0999846	79.29277334
36.9446881	2.518437912	11.75473002	18.54131966	93.06346646	35.7528258	120.6666684	123.0833435
171.1205835	5.93001828	17.81785103	14.59464865	51.15641127	34.16818171	74.0077903	181.325964
282.7991726	13.17450109	30.24731629	9.594680019	91.32225049	44.43401342	412.7685791	245.6130718
6.816886878	7.805287686	18.81457239	16.58979491	87.56255179	47.61618098	38.11478259	119.7927946
79.48738641	4.016213561	18.44472644	20.16110653	129.8775124	49.63653388	121.5644047	187.9771067
87.88647422	7.412910676	19.04998388	16.75730409	78.75053029	35.38756265	130.5292777	134.4479492
24.83712548	1.435363869	13.56441306	12.41296717	419.4528342	42.57429463	485.8936852	182.8718033
274.7183715	6.190316364	24.10829196	17.81566924	28.45058513	38.0092962	59.34113083	445.8143173
10.76044578	1.289601708	10.0757818	9.338493974	77.46392871	37.55799066	141.6190711	64.84000503
5.862980139	6.772374747	22.87139144	15.6174619	159.4062209	73.66279838	121.2947403	162.6955397
78.42445941	5.585276971	38.10825071	25.47727852	154.8594423	43.19753127	89.67831645	173.7366617
92.64171381	11.83519662	23.44741336	23.03020897	118.0261786	66.59813572	89.78148099	315.1174323
35.96530454	6.432699175	15.78720546	25.31362262	77.23148697	37.33129945	52.28087071	81.27932816
28.36195679	2.770121704	6.425981747	17.24026502	63.5337739	34.24038521	154.7417271	84.44342842
89.49872693	7.31838807	79.3575036	61.51349849	258.7564619	80.99496309	149.5481243	307.7041607
110.3688702	6.172250327	32.53192332	20.64943301	78.86700985	39.36009828	114.9264244	348.9297628
64.67580232	7.452706437	34.72323148	39.25398408	104.3140804	63.84498212	131.5943308	369.5114934
139.2864273	4.889954839	14.86094849	12.14705528	20.23205683	26.29084002	33.83835381	157.9742937
152.0435206	1.210011875	8.899192732	8.092415563	66.47286861	21.96567977	131.6439729	136.1364274
14.24839042	3.504678219	11.23012968	16.65800806	71.74994242	29.40026814	276.5370725	70.13310275

59.89029143	9.829304089	29.39947697	26.31911948	89.21420267	38.65989525	148.0222213	140.6754235
38.56192044	3.279082422	12.92644913	15.19994992	41.15424967	31.14941205	93.80286307	95.55773085
37.89750674	5.867489074	22.5445271	28.52614145	190.3056374	56.15630792	274.7494216	203.3389219
18.64746622	3.973865444	24.05365739	51.22195808	88.46444314	51.35150516	153.8743266	103.2114848
94.27869542	7.275137596	10.47587891	8.708945308	73.28346811	32.69467477	198.34356	87.38072247
66.76660443	3.422604302	11.52407217	13.00177317	36.11459963	26.50041473	27.19822798	49.27464005
63.98337654	2.81572397	9.396239283	14.33571841	207.5716137	55.6631101	226.6865468	265.3696816
17.62712618	4.676268612	13.39858164	15.3453726	54.23745801	38.91674508	119.1446778	90.57366641
161.210911	20.28816731	54.47890608	25.08725967	75.76385575	48.03039268	292.2891107	304.2447627
19.40659631	3.73988498	10.16866751	12.040221	42.67192921	24.63021819	87.05371318	63.49611757
205.4029856	9.449747806	24.98337735	10.5653918	109.4475067	42.34539207	148.9447461	173.9202458
75.29182012	2.972771398	11.24558943	12.17786128	28.8968446	23.274447	104.299276	92.49355139
20.29737613	3.05259843	14.15928257	31.10848485	110.5685413	44.39268336	80.80653856	65.0990838
54.06361948	12.87465523	28.03703528	28.3158848	65.5200641	70.89741114	100.6316476	154.1030855
111.7299065	3.629668138	20.32462493	10.30746655	18.56189998	26.35125158	47.49593362	176.5401221
128.8924456	1.605906297	4.764286497	4.797370335	68.77788861	38.24859765	110.5268625	160.878816
34.44039488	4.698786664	13.02652729	18.73939471	44.24758589	39.31415672	202.274461	129.3883842
141.2919974	4.746621814	13.66418289	20.14139171	66.1425627	30.00845153	213.1956565	173.8081622
113.5930543	44.34868444	84.87722727	39.84237813	92.09689465	79.56275953	24.27369686	490.7761758
70.61487056	8.094700403	22.98195992	20.11388883	98.28895092	40.31439527	105.6604877	106.216301
147.4347488	5.142514186	12.36364709	7.138654666	192.9148765	29.77369187	227.6459641	95.18319613
30.70949706	2.780852493	12.21918587	12.84409244	97.20980946	48.46126139	212.1511492	197.9306657
18.91591939	5.406570006	30.09881258	30.61548589	131.8071205	42.49466249	66.7023065	126.2676511
63.26447717	4.158121425	17.57542674	22.19477925	93.86005272	58.62620992	143.1657823	123.8991149
44.7161034	7.486647849	32.07172194	27.97452126	116.6021631	66.32767744	308.1310354	244.9027525
165.1128329	10.14977306	30.06010365	7.358371427	19.81377517	32.3557033	41.52123551	199.8096304
35.80848825	3.343810586	16.64659384	22.07401643	107.0734105	37.04712389	172.822097	82.58098117
29.07931537	4.228529898	19.23619472	19.35688704	85.68371404	51.33321326	89.50526383	130.6056997
428.0246151	12.6374785	18.4768718	8.62987807	51.77052686	25.18556388	169.0394299	101.3167298
45.85239869	3.226169582	10.36962081	10.49687269	94.90564639	30.21397226	203.0630424	53.46342375
40.19045889	5.836649106	20.68014093	15.52042207	39.21256793	40.07054822	95.8064724	107.3756961
46.2290454	4.612075257	20.37026921	31.80225238	92.15531673	88.36258624	131.9046994	248.628318
103.942815	5.847823494	18.0382847	9.676045576	58.67506066	36.4949181	53.17786094	136.0156144
122.6196222	9.463988774	40.70116756	30.86139677	173.3085681	44.42761997	246.3125462	300.1732915
58.43836004	5.555878475	16.63362578	12.52860263	65.37626287	26.60805133	125.6057789	92.89240392
49.84105493	8.73699176	34.13748646	22.94064589	270.5767657	47.18571658	197.3191894	328.5240074

71.24222645	3.669621739	16.23041666	17.20572918	72.41643447	28.26394391	57.18389175	88.64755347
92.37062906	5.237166669	25.00186176	39.72061969	128.9194638	56.27828393	121.5225977	180.3210226
49.39414556	4.574645168	18.81821368	21.50429056	57.81566407	31.27374733	110.5615037	97.64690163
18.68899917	3.311174722	10.9530477	17.13794557	89.16129696	39.00222134	144.2376575	80.18952576
135.1349809	10.15637565	40.26972728	16.36401525	77.7035366	45.64384047	150.0728729	146.4841905
113.489555	6.665050676	20.94209599	18.68159452	230.7836265	62.51654646	357.752838	254.4829174
39.35899123	6.335899434	26.9569777	28.05080425	73.36029165	60.69568062	45.60378836	191.3090979
57.78728057	7.71604475	43.41276965	52.76441661	193.4652812	85.95547782	240.960703	371.0395157
60.8550559	8.35612774	26.03210237	22.23635159	70.44502617	48.44528089	210.8898561	157.6277976
147.8071084	4.585029171	14.31449235	8.436338816	57.14457836	27.46371316	96.60708707	117.556448
299.1668628	7.420309451	23.41980972	20.12566554	53.51977201	55.02699352	54.10555804	271.1847702
149.5889654	4.497362001	13.24772558	9.992431987	36.09206861	49.51263542	118.4176148	192.7538649
56.87976771	5.366008699	37.53529684	34.34130377	139.5081411	57.24140125	40.16153877	207.5515497
77.92341845	4.323510335	15.25891311	28.77499603	102.3326413	50.79798909	212.8267288	200.2337276
136.1800736	5.265427013	15.13273346	10.20578584	169.4851173	33.57979363	215.5140113	135.2196755
41.03685443	5.533509929	24.45159056	30.36552555	76.44640971	43.860358	223.06338	248.2842646
75.58173587	5.548068057	31.16498262	26.70386717	290.8174473	66.19113461	414.5049007	285.7400569
48.29820243	2.50869602	22.13559241	30.19449322	96.37898796	34.63354245	74.80335979	86.36587548
33.0114059	2.430763617	7.350132032	9.027687724	56.75323706	24.23529355	129.2062574	49.93706678
44.93679542	0.469090626	2.701536969	4.024723673	72.65372755	13.3789148	193.5988539	48.85055928
181.4945139	26.00545623	69.36916655	13.3165424	76.29711862	56.85075071	106.4652656	369.8987052
90.58696626	18.60718175	50.26938414	28.01138455	133.7463688	63.21622076	73.18608122	416.9617543
217.0130986	6.969495369	10.86263673	4.634907153	120.6974448	40.50138055	331.1784523	135.8021747
66.18422451	3.928212774	15.41306871	32.60033982	142.4846333	67.85122338	168.9826302	160.4254222
72.83087041	5.993171469	26.3458158	13.01793835	44.96934141	75.83602365	239.7860059	230.2113256
20.87130896	1.110871261	6.154192329	13.6247673	68.67735524	23.34975961	108.9102378	59.84842792
111.2054127	10.6163055	33.19558705	31.61782133	89.01897471	50.37683529	30.83839839	282.4942633
65.66315095	3.954532813	13.2850607	24.5005193	36.50233466	29.69803554	98.76272307	125.0247744
78.88917997	2.091129307	7.943200545	8.34750821	48.38213644	26.07049806	131.9979659	79.60410757
20.47095276	1.687467765	9.445456718	11.19587235	42.40153191	30.14756054	91.72623484	48.59616213
41.8561429	10.74880287	46.69606569	16.51727678	158.0946684	55.31048072	100.639396	87.54041238
55.23397961	3.99622462	16.07183896	33.14051025	151.9587258	45.54431909	242.4346703	198.3615392
47.90176761	3.081220268	17.13128835	30.83762199	56.72444042	40.8454598	79.15645445	134.9312402
119.6971791	17.19075433	70.13944347	28.58806617	137.5338532	60.20429445	87.68190158	339.1891196
38.39945162	5.961090516	22.52559673	26.10222964	106.3374616	40.61348042	185.6591385	190.4506437
43.42991432	1.718652873	5.056111756	14.58233093	36.42959932	25.86102957	59.46072655	111.1780718

41.19936891	6.101017979	19.66091038	26.11703384	198.1795134	59.79949469	404.5802375	123.661579
27.8256973	3.307764534	14.22532122	9.456491919	35.76664283	22.23826367	18.02314649	53.86859992
58.81948784	12.9147274	30.75836934	25.54498905	85.12200098	61.40892762	127.4338023	245.5364037
130.5424698	9.802862903	43.02642871	38.15165513	152.3848641	56.51036832	95.87183402	281.1680011
81.26156555	4.736808538	21.72991478	25.39058947	143.7590317	51.16049872	150.5918689	277.4228
42.78922121	5.103083556	41.83073861	54.40702493	175.8457214	58.11646068	268.6187039	247.6217776
77.37614143	9.621216085	36.68092199	37.05308162	122.8654354	39.57325159	124.1020646	183.7325447
2.11017233	5.313202925	21.30314945	30.22897811	139.5429594	63.76204508	74.17452332	104.8173768
52.50615788	4.342380113	18.26133258	13.27976209	77.23945828	34.87378799	82.31274731	102.6916833
4.420489862	15.70482087	43.32482754	24.61070282	183.4118046	93.44121572	203.9952246	276.8397265
39.89195132	4.566910177	18.89331127	17.04755941	43.17285344	38.54857273	122.4682554	138.7767249
94.74515338	3.774193144	26.17493704	32.46743186	82.21650878	61.03672138	166.7738655	306.6020455
52.83012824	3.007356326	15.37272284	27.56762489	109.3530497	52.08557427	173.9425254	231.8821497
38.32936747	12.70550106	35.50193446	36.47002593	233.2791933	70.7466062	273.6195639	288.5856275
119.1197531	5.409039318	16.25382735	12.86298201	72.86098857	41.84636283	134.3679407	254.007789
123.1557283	6.31024031	34.90635369	24.77161048	129.7374054	55.11581537	217.8917698	174.3134339
25.72013785	3.209452718	13.05401027	16.61379167	63.46458218	48.11907373	94.3684211	159.8013384
54.76343549	3.867361745	11.70148912	15.46196499	78.67791451	30.62529045	196.2326433	107.7175607
71.89128864	7.293554596	23.5515272	16.80686799	104.8931213	47.41700656	88.50841829	166.2964007
92.80330799	7.554285053	16.43631124	14.85374822	205.1446594	42.46095761	350.590235	80.91578662
47.07677758	2.764634822	11.29702336	17.4440343	104.0892319	26.70042123	91.56244302	69.15852724
54.58876498	4.477662053	19.64170719	28.66994352	94.61270145	31.37881011	158.5124956	114.3938984
82.45983014	4.715982595	15.09000946	12.1451516	58.85027145	60.21155413	85.6916262	389.327088
31.97137778	1.941669681	14.10171336	29.93309634	98.70056155	40.98455572	189.0484171	164.6774605
67.33775877	4.812680215	14.16919231	19.00660768	96.72731452	35.72013191	151.5541019	90.60102976
26.97826289	6.919679516	25.82610498	36.7591851	96.07054523	46.0460441	142.1866926	65.23069103
31.06088841	1.877124499	7.860196881	13.24895489	51.06679263	20.96453075	127.9556195	61.62783166
28.69832933	3.183186476	10.80187384	11.30352246	51.13030379	24.19467794	95.15773823	47.91940731
117.978974	2.22725748	9.027295134	10.01722121	31.12862018	25.9889332	120.0481319	273.0410528
30.61712122	4.127493729	14.69412744	35.26696627	120.1872758	42.21429932	66.48062639	119.3503288
83.34579635	9.330135214	21.70267768	16.43471531	154.7315825	46.03565855	131.7339838	134.047939
44.32571164	1.908702675	5.85684109	12.12575243	30.93407904	32.26223222	23.40772113	139.4048564
33.20824175	3.08434993	8.320002352	23.05576446	76.67793539	33.02714198	111.1653563	96.4282172
46.04221909	5.495774422	20.81781126	38.01772907	138.1515038	65.28973531	121.5731377	326.82963
28.44470767	2.599755607	10.30722057	30.96628042	115.8569261	38.93975365	339.2977433	96.7833826
45.5293777	5.225268867	26.88895864	43.43040094	189.1513826	57.13453439	257.09932	260.7185154

40.98407852	4.306085469	13.41843256	21.87051903	34.33323062	49.81168184	167.146699	194.3910708
99.79024913	3.467876962	14.37682736	12.89381199	104.5528273	34.81894159	116.2083227	123.2305911
8.955336793	3.32338465	12.46968303	21.79965465	105.9484075	44.21010662	161.256989	97.09769572
64.85382794	3.008364169	13.44411254	13.07987335	39.97139624	29.09741673	36.00309619	25.24982975
11.65757798	1.402223602	5.855094668	16.98415201	186.5126398	50.58161559	236.5895713	131.1725229
89.31037778	7.34790932	24.88164994	21.0579091	171.6358567	82.36686434	114.8670628	238.0038791
49.58466778	5.642553754	14.48982396	15.84668685	102.5597654	53.39273472	182.3433189	86.65881994
161.2709253	6.167038299	16.40935777	8.9090131	130.164221	32.43935399	45.90836388	55.03466566
200.5658839	15.03906495	52.69828232	13.66659276	448.3786622	76.62613208	507.9559319	369.7182702
35.41591216	2.72428614	11.89572905	9.018838143	57.53799779	23.05860991	79.23501161	76.51602064
129.8007581	5.210100964	25.0058451	15.69499218	142.295847	47.81927683	129.6684182	119.3192106
102.6028192	9.531840963	26.93009216	22.59886379	176.4261431	94.47477091	269.5192321	279.8941182
99.57583187	18.30120594	49.5095953	12.45029189	216.8328314	67.00111461	226.1816368	126.8244482
56.43595155	4.963436916	21.86698168	15.20272395	84.08539268	40.47951545	83.41406853	193.8403238
8.219465575	2.640225774	16.22316009	10.72271088	79.08633577	47.21047821	91.64467987	32.36882269
92.37140878	2.675702638	11.76669715	5.858944327	47.84699059	34.17635545	138.3809565	207.1326722
42.33527743	5.894677171	13.96030907	12.31571165	114.5550292	37.53885529	156.3056218	113.0679057
124.2645713	7.948387034	25.32593825	17.01672865	146.3366309	49.64722788	212.4723215	90.74685698
28.50580985	1.764339421	9.044657362	7.605157207	124.2774884	42.00844122	179.7354042	90.28263057
91.83568465	7.174823831	45.92539403	24.09000185	237.6836656	59.22383199	142.5809452	185.9185011
70.56422286	2.700694444	8.788224577	9.435982485	139.0827229	29.25775873	338.576134	64.73895813
25.04319381	2.811844205	10.63004699	10.11222071	68.25662058	28.61171942	268.6854579	43.46601153
31.8372362	4.245631492	12.32487293	16.95149815	129.1516638	43.36656878	212.3832997	54.91259234
60.32012265	1.753675412	9.550878012	8.664882888	84.78757587	26.80464228	107.5723939	83.83101048
170.0990018	12.31203012	28.09463954	9.260337145	130.1672946	40.74844769	79.81219465	113.5932903
62.77273538	2.940571063	15.41122895	15.3236229	83.68860177	49.16006655	60.18920728	274.6763648
100.3695776	14.46635	60.93489362	12.14456994	368.4410875	71.48158916	375.3721683	234.8040067
46.43388142	2.559290772	10.86103637	14.04321785	72.33543421	47.44192972	441.9870572	213.5358912
18.44527383	3.045568689	21.24098558	21.72394475	119.173798	50.55942354	99.28300051	67.7186209
62.24999001	8.809880729	17.40611022	15.82083939	86.74537058	36.22382878	206.7947084	86.99609848
74.52626878	4.982438107	20.46961693	13.83400283	183.3063597	63.93453719	207.3419739	229.0063718
361.2557155	11.67271728	26.90534839	7.456435431	209.7407064	41.03487658	221.6680688	126.0556994
50.25697345	3.01796494	13.38998075	12.02153138	42.14908701	28.54880103	42.71026364	90.72160672
75.70403476	6.210230082	21.19486879	12.20949909	45.91201405	47.47467575	48.11959217	102.8872333
385.912308	17.18913356	50.83520458	15.61825317	269.163438	60.70756392	94.9242431	135.7596693
29.39270197	2.354731138	14.80954652	15.66450797	63.72046612	46.36761916	185.213063	103.8194754

83.99823863	5.068881476	24.3465019	10.07054079	59.56236397	39.615856	136.5747783	158.4631945
19.94621223	1.726324788	7.296264675	9.417885486	49.39477275	24.72153395	100.8943353	34.0741647
165.4456128	10.27217722	28.1278436	7.605790718	77.10959122	30.96943443	64.77638493	88.42387731
92.81546346	6.773800036	29.58484701	10.89744438	64.57175767	27.93340047	41.66045023	142.5166059
47.96953126	6.49545331	20.22602516	9.447413281	47.09558545	33.49759956	103.3531753	156.5485919
167.4372638	10.49204311	38.67957885	27.47717531	69.00439612	60.03394871	275.1213295	309.6872162
43.22045407	2.65015107	9.425674089	8.762534911	72.83089972	38.21617634	78.61814068	93.29955958
26.71961977	4.052010433	16.36504026	22.61678653	42.79247445	47.36226773	50.84587235	157.6916524
117.5573359	2.5513434	12.6904375	11.37633423	47.87238428	35.04693394	86.33535032	90.85832791
115.0785632	8.30536867	36.85064507	24.37094524	62.6967526	48.0473755	105.1989722	290.6493277
29.69230727	3.880051048	26.95933332	25.44647978	123.8734291	41.99833549	163.3907641	198.1881368
83.67178546	2.41601012	18.89213515	15.03639447	43.75903391	41.1718166	158.4398295	115.0331136
170.9200243	5.049798379	43.32250727	12.1933699	65.02893016	41.28214257	65.83887661	227.0462927
188.2682029	6.991890791	25.89724421	8.274040597	136.9177374	39.83101711	160.5091396	86.71861637
240.7973719	32.57678713	130.9952829	18.66307163	61.29921851	49.6188299	64.93221381	474.7004285
13.01126079	3.218809061	13.28888893	9.274189711	97.37434292	41.16257073	119.35317	71.65251155
44.14632292	2.997954634	9.849740057	14.20049918	97.27737725	33.65427605	159.4280545	85.34327088
27.62973323	4.071323839	20.55288976	24.63875304	171.8404675	89.8104168	145.5264953	112.3947867
114.3670138	5.017540866	16.59410259	10.91721208	132.5680254	39.35723602	175.6271153	94.47136757
349.912161	4.571102358	9.864624405	5.450593871	87.2227457	17.87962895	582.6757505	92.90915018
60.48003256	2.228604876	9.307777887	8.83429978	50.07780415	41.22229509	54.16195704	141.4714774
27.43103476	5.063167477	15.06857142	10.99138994	74.19082212	38.32364197	65.09789022	99.37949123
37.64676907	2.397047034	21.38458128	16.84176072	175.4426894	88.22739467	458.185801	266.07839
225.4434581	19.47177223	36.64787384	7.866777435	61.39076978	30.09374923	98.86990824	114.5870947
104.0039004	6.517519724	19.18501222	8.500228426	51.60866205	34.36967403	209.970297	84.29795516
73.33385651	3.62033934	11.22366447	13.35658462	140.6928792	40.14623086	179.8184834	130.9258413
89.92776099	5.935574332	29.14526052	17.1788399	73.12577472	47.01031785	191.5286971	286.7163053
29.86874781	1.984245624	9.055022838	7.825927645	38.86023261	24.63782421	32.53132517	55.88419953
16.52156629	1.271141122	6.958619314	9.500281917	55.6317881	25.8766135	21.53534392	30.98051674
109.4000193	2.533396467	11.37270242	19.16306903	25.20757011	30.83183537	48.89213864	414.6019373
93.21894625	8.969299083	32.70966099	20.29737072	223.9381328	57.73922035	196.8716642	116.458824
81.50233995	5.919160842	21.11648526	6.568280466	99.36612149	34.75339018	33.47329643	101.5062941
30.03287282	1.361585388	6.250065831	6.718069149	41.65007238	22.54629645	98.80224223	55.97458627
155.4063115	33.27043859	108.4150245	19.0837832	261.8914561	60.46728071	178.8677303	280.4740699
57.36690454	2.828547147	15.33082949	11.63975009	92.88174466	35.25330054	30.00758144	107.3980132
22.91657347	2.442748213	8.338917633	5.876079296	49.6961256	28.46217915	99.31586937	55.05243209

83.92976343	5.59954428	23.73460872	12.47558752	124.1019123	49.67850979	101.0219735	82.69141254
48.98227546	1.256842764	8.656638046	9.8318618	55.66322845	28.09992927	86.11890078	83.95826805
50.10419742	2.482947451	11.53921142	13.50981657	46.29693137	37.82936087	100.7070379	131.0011775
24.92089397	10.08412263	24.79412394	12.65383047	139.3669118	39.76267934	112.0972631	89.68020863
83.7246409	3.507801675	13.44258016	12.81101729	144.4654929	43.33434159	221.0772653	106.1735802
100.6297913	4.613704627	16.80220488	11.56605761	55.41123951	35.96710349	171.9433915	188.6493372
24.37132016	8.368510766	17.51373804	9.096834784	183.4377664	38.08066948	286.7276067	80.12861435
125.7223277	5.783568237	14.51340227	7.842990493	105.5245763	29.14295188	161.3366308	65.90359973
54.32445758	3.497971965	17.24694171	15.95322135	45.47285122	43.39010153	109.6133051	156.6448351
84.23339541	13.15278534	32.23143602	10.81758639	171.8948204	49.41395659	91.83133706	95.74490821
78.53069241	4.330247358	16.45107299	11.50882768	217.6179631	44.0685894	265.7483231	75.85773661
53.61823354	1.406512992	7.78081218	6.387922678	40.5262408	36.58819493	24.57276892	169.4503891
73.42406069	3.352824078	9.388232739	5.795016704	84.08052332	34.2319657	281.3743682	93.35002541
22.45290362	2.315880626	17.67703337	17.8685747	111.0186432	51.63358647	506.5246554	230.5437591
156.9602308	2.643008461	13.51624321	2.684238489	50.50597955	16.38947061	108.8090232	45.92809634
42.37956501	1.961348113	8.429746306	8.168217979	35.86987499	31.48244211	73.04251689	102.768927
49.70877863	10.23290935	18.88341998	10.15498123	98.74891023	35.96823041	227.1289897	46.16205626
26.67827133	4.4829917	21.86978698	14.26636999	109.4660957	44.49942147	146.9519489	125.8048088
22.58806326	8.450652598	33.93203019	17.61885692	128.7034593	40.92658029	72.93414377	91.68763771
73.58936302	5.248996406	21.62307979	9.79621283	51.43824049	40.42243399	88.54628315	49.82719705
66.75213889	5.856134978	23.06318473	16.22382151	95.52193699	51.84445283	51.17640411	221.9472886
58.59754517	7.112843986	21.14566436	19.41757349	197.9173949	45.30011669	175.7639258	73.5248459
28.51200414	3.051399671	13.04633173	12.34942809	60.37711727	57.97186516	80.59128259	180.6105762
49.25185783	4.952761182	34.24257437	10.94280907	82.09225004	52.65613373	93.09355983	101.661747
32.05993856	2.928806139	22.75189819	17.1285538	133.9649373	61.57995427	189.3081264	217.2595936
54.51724896	6.494321481	14.52639876	11.1786955	126.3002546	42.2460081	169.6812256	104.9690677
50.22996898	3.480570067	15.31387872	19.72970022	128.6781408	45.31454893	70.80625019	157.0879324
66.67869788	8.241806494	42.22706161	30.62691366	112.162631	92.03285366	329.9065452	181.1717071
40.93995014	5.412992217	21.717306	13.15511471	67.85347981	35.32102551	89.21158198	190.5278788
44.23946699	4.736030312	18.56852795	13.89555753	99.84367528	66.165852	211.3914494	210.0487512
7.321709595	2.931272204	20.18174709	24.80088937	39.95531798	61.44117126	140.7968117	237.8741061
27.97081326	2.2861611	14.54519789	14.1052844	100.7821199	50.01951712	123.9688089	162.1405727
189.4516842	4.37852546	9.628756599	4.24052987	75.58910286	26.33789995	124.0278671	126.5944568
29.48576791	2.083890428	11.78526028	15.33568234	97.71101434	40.14432661	38.90627419	117.8519297

Carmustine_1807	Topotecan_1808	Teniposide_1809	Mitoxantrone_1810	Dactinomycin_1811	Fludarabine_1813	Nelarabine_1814
490.7218706	2.030345489	3.807676697	1.946432195	0.101872134	225.7956239	549.9292256
437.7029578	1.56975491	2.277304686	2.357171952	0.094208112	118.3573471	302.7501282
578.6513198	1.048983609	0.716948296	1.286430505	0.1424117	174.0882365	563.831702
239.5165339	1.382333998	2.27850947	0.96086906	0.068210348	51.8279923	245.8974667
917.9409646	5.410894434	4.719185527	4.097978503	0.183883109	305.0086868	682.7177148
324.8456512	0.434657591	0.667620993	0.382469578	0.045191595	88.37235127	217.9398343
505.9759241	0.523315256	0.73164047	0.784512533	0.062048249	132.8164629	411.0387016
640.4387127	2.572398628	3.083477537	5.441613165	0.139201271	234.2475869	566.0623215
941.0261492	2.280659334	6.409351313	4.900604561	0.23416458	318.4635994	797.6477558
297.5520182	0.487181881	0.675261593	0.976153791	0.059510477	73.10532168	202.9185865
259.4165125	0.900852997	0.923178276	1.059908022	0.061737449	126.7446281	219.0498004
544.5376926	1.522892191	3.377556535	1.593729276	0.140534588	191.0307978	520.7335196
377.4767312	1.226856492	1.60942217	1.828737964	0.077581824	167.954981	430.460499
355.4964891	1.552825388	2.644721459	1.500021998	0.12648832	118.2455557	402.5599514
506.4770354	0.775390923	0.952300717	1.513909085	0.091015178	109.5031262	389.9362611
561.027451	1.807204299	2.871321595	1.906320854	0.103404995	195.7406448	407.1565871
214.1268204	0.977641752	0.678889212	1.835824088	0.048602083	133.21855	303.813983
693.141069	2.52184342	5.497217308	4.115900943	0.097708617	333.1755244	895.1410847
595.2696321	0.986711925	1.200774136	2.134094877	0.117125564	142.125047	476.3642805
280.6911583	0.367322958	0.550326447	0.532812892	0.038551864	59.86850229	216.319643
507.2219293	0.780616894	1.183701898	1.615687575	0.088330275	168.4149287	412.8535073
619.5736589	3.106108735	7.497269918	6.968175363	0.171625931	515.6158155	800.4181808
464.9829326	0.33221033	0.626916789	0.50930282	0.033274962	111.5439171	408.0590328
902.9767965	1.030657763	2.850640178	3.282836745	0.166743409	202.5025714	708.9292831
547.5380183	1.118327243	1.735524013	2.77943835	0.125840591	189.9287604	382.3957712
580.0223435	2.341878725	2.185736762	3.977511546	0.117742877	144.6614987	479.4646286
656.9242244	3.211519452	4.826257399	4.152143838	0.184534843	189.2753034	579.1588692
877.4157929	1.28019588	1.80995046	1.990951369	0.131770295	185.1471572	658.3336563
469.0455975	0.545400276	0.88067087	0.96247036	0.05364428	92.56316668	395.8541521
363.4884718	1.224138955	1.328158866	1.877207211	0.062513036	96.00203758	294.0928816
375.9229334	1.694264901	2.24448565	2.749123391	0.076579784	164.9588002	333.701971
457.7407084	1.291520739	1.655295424	1.567417009	0.059384109	130.4103084	381.6220986
752.950952	7.621421621	8.411500452	16.36893138	0.203876726	296.4597097	757.9278312
321.6301577	0.461432411	0.658687953	1.652871505	0.092454223	144.2721568	410.3958183

775.192217	1.144154937	3.672697491	3.74305255	0.141687377	330.6242623	737.6556804
667.9819185	0.96949724	1.937043016	2.528912988	0.078630693	244.1316429	752.4743017
337.648642	0.684943618	0.547602071	0.411567541	0.062141761	47.85236724	251.0241293
861.9580322	2.837708595	4.125396617	4.65600277	0.176858831	230.193429	891.8450155
566.8139327	2.193468927	3.346261297	3.142825048	0.082275562	149.6093723	522.151373
412.0319535	3.336418219	2.838237815	8.263016309	0.094359634	352.7021005	398.7585782
642.7189657	0.854334848	2.705182007	1.816631556	0.128285472	240.6663879	661.5172101
566.9268981	1.528883564	1.430698392	1.202558029	0.121776718	77.12936588	417.7698894
599.6946474	1.59535946	3.085680205	2.458538901	0.089985253	169.8451703	590.4687426
459.9429016	2.792463824	3.199719244	3.784760617	0.106768849	181.7008069	416.4848087
489.4708732	0.539961869	0.957258889	1.135505423	0.062775032	170.8016898	542.411908
194.2224766	5.281023403	2.186137183	6.134634603	0.081334072	139.5824531	188.4077347
433.3995803	0.412759983	0.490173431	0.84492633	0.061651336	97.51419576	344.1216864
500.4816004	1.528951544	1.456060851	1.441134046	0.065200061	124.5803969	512.1241708
409.0036292	0.267125849	0.443491686	0.2282873	0.057411721	108.7760029	303.4268322
506.3494192	2.449380965	1.731122524	2.976412213	0.08088997	91.730348	319.6158285
236.9185377	0.162994157	0.213563934	0.222903317	0.031567844	84.52799105	155.8505877
490.8049465	0.700496393	1.172985337	1.022598534	0.101913605	125.5113256	411.9600528
400.9953864	1.368040008	1.550639468	1.809893122	0.07005792	131.1766504	362.7258876
334.2329395	0.374445074	0.529861692	1.396980544	0.056742853	96.72624813	237.1064393
490.489239	0.407123822	0.706538167	0.997242551	0.064176325	67.03518288	524.437961
729.3591221	1.39370908	1.14042125	3.327294805	0.14084817	52.25242404	571.0935116
347.9579855	0.465852203	0.521532026	0.579402201	0.042923915	119.8876596	290.2555302
349.5691413	0.474626167	0.703547984	0.59739577	0.060036824	98.67268316	286.5130035
898.4663954	1.380217944	3.370794741	2.208321631	0.125516731	260.0914972	1021.023919
668.8050152	1.725130159	2.270162507	5.706517422	0.087311193	223.257926	598.829925
838.7985393	3.648969286	10.18214097	5.988549341	0.274892246	365.7548079	625.8101724
366.304179	1.423984027	2.698506188	2.552384762	0.104977343	208.9574675	374.8971382
863.0074752	5.972696862	11.73581662	15.27523992	0.272054829	280.4896492	782.2042532
491.5763264	1.238837678	1.834518122	3.599866936	0.092072348	172.020883	486.5804376
396.7317611	0.380363399	0.51502911	0.463538302	0.063433728	83.95289852	286.264809
378.2279317	0.378473977	0.488979659	0.433981978	0.065039323	84.52438493	271.7759409
478.3086386	1.571645285	5.202264529	5.386917404	0.097190063	209.3019542	475.4325472
1826.151111	2.877071631	3.863253954	5.116908014	0.329137472	433.5817333	1291.450294
564.7572262	2.22040884	2.465181907	3.519888936	0.078600804	125.9592577	504.9986725
360.2198297	0.46244184	1.017723415	0.526774387	0.069702255	176.0524385	262.5362775

656.8509437	1.197685348	1.706010381	3.873532614	0.089762486	241.5258407	680.7572081
323.4572587	0.540351441	0.885570857	1.413321932	0.050268992	135.8929757	240.8489099
679.4020341	1.239641291	1.916736845	2.734461806	0.077679491	147.9420976	624.7602536
1015.316183	2.737091607	6.798798379	6.362930009	0.281230927	554.0068397	988.3994767
382.106111	0.71209002	1.052789603	1.948568095	0.092348991	118.0935857	315.360681
364.6559575	0.450008716	0.498418564	0.392574506	0.069813343	63.66448345	248.3792138
266.5934615	0.616116186	0.753771178	0.993702191	0.056610612	99.63000894	261.6078917
547.8893407	0.978684848	1.777011306	1.299753633	0.075065261	176.5444069	533.1292399
474.3927004	4.339484103	3.431297681	5.920104017	0.1891713	329.6072665	601.5263534
390.3627553	2.202170675	2.884894593	4.866110528	0.116767345	173.0292897	336.0964052
851.8951645	0.861470201	1.372876201	1.647244125	0.147401436	129.1789674	933.9125691
813.2204112	3.31331077	9.209365243	9.101599574	0.195479211	364.2537286	739.4599974
389.2945973	0.580571519	1.292882378	1.736936254	0.065820748	142.5371854	362.3508573
449.7402867	0.380685863	0.478169757	0.437901472	0.052731923	55.09636493	342.4444918
674.3680794	1.5474143	1.535545542	1.480466972	0.154771652	248.9524401	632.0140238
753.7750895	0.974334521	1.58615731	1.455141183	0.114813711	216.7323322	529.1056126
341.2972524	0.606350307	0.750834052	0.804139942	0.122938152	208.019403	331.5841396
414.3607686	0.623444985	1.399418793	1.487263226	0.078175287	140.3905833	333.7891351
650.9436393	1.913282467	2.240356141	4.705698174	0.173318024	165.4491181	626.4891578
368.0608882	0.792160794	0.714591737	1.465602461	0.049022062	74.94044543	292.4275759
442.9143064	0.814685063	1.434438323	1.363449678	0.088585268	244.8532298	405.0247978
268.2535845	0.464500132	1.005064283	0.912625834	0.039175617	95.40612322	357.0519479
588.1513431	0.401360882	0.860405852	0.623083799	0.058679088	129.4014222	593.318177
371.657438	0.965487271	1.5466092	1.921104177	0.062012416	120.4077768	286.8102598
340.0458902	1.206027213	1.304470349	1.572426262	0.050771146	113.4058671	297.2056063
492.6554867	0.542621504	0.945819399	0.773640339	0.060044291	59.74144243	454.305769
351.1592408	0.674633405	1.100150278	1.003591053	0.064537697	149.1000702	319.7144494
605.192338	0.4902798	0.749701647	0.636791825	0.0509788	115.3129111	537.171035
380.9013047	2.877014439	4.237523298	2.83065183	0.121448741	195.839727	333.2290161
299.1815096	1.318003663	2.093834527	1.345874767	0.10253673	60.1010835	275.6459955
483.3061919	4.204355857	9.21104091	23.62717253	0.183173613	245.579771	515.9785126
661.4679518	1.394484598	2.059856264	2.165901678	0.135134467	134.5100972	470.9383344
198.9455586	0.620163963	0.394061778	1.473429843	0.051790925	108.8368497	186.6388445
385.6202768	0.53305466	0.839646427	1.197466091	0.066521733	142.7808367	336.2747719
270.1419726	1.074153146	0.826530418	2.766899035	0.077845813	165.9615994	373.4373467
568.8459975	0.907957019	1.443338791	2.11238615	0.078314745	144.2924087	527.3049493

618.0627716	1.67911516	2.52189629	1.839884581	0.099075297	223.8823614	434.0256267
604.1200487	2.174043408	5.358965383	3.185252981	0.141832207	185.0483558	493.0734099
331.1246175	0.374462575	0.479515251	0.41883714	0.053804048	75.0684066	270.9265699
559.3500065	3.748798904	8.563574584	6.986346822	0.112073882	281.7948683	674.0326727
477.0295277	2.320846799	4.462161293	3.451416231	0.11175634	164.3902543	364.3029482
464.0327748	0.726400618	0.92075168	0.942178586	0.072334181	79.81527301	333.9351567
1018.87908	2.589316603	5.885031682	5.047044406	0.223742144	171.6651494	737.6919709
385.1805239	1.304840529	2.139891221	1.987117827	0.078432166	191.40372	375.9434084
709.6580567	2.722582694	6.82707422	5.367869842	0.140540142	200.9363513	633.2727604
469.4337526	0.829941841	1.241820821	0.955124229	0.105255861	191.9043574	302.3436739
307.5070293	0.592107069	1.132197482	0.525703642	0.062014387	115.7383485	385.5435501
621.3138158	1.165567764	2.552911332	3.625001998	0.141590983	168.7376481	627.3309516
673.0195732	1.810938389	3.040198681	2.327872152	0.100304295	183.764718	663.6452951
362.2424674	0.583632473	0.783037754	0.949208161	0.081163082	97.20806556	279.0162454
215.0024369	0.412698172	0.838652668	1.36939911	0.035407802	227.2707915	274.2465559
528.6476532	2.253282573	2.926076314	2.717752408	0.080568078	206.6569369	423.4954507
546.6966765	3.249196503	8.043064329	7.469196697	0.103547316	170.7561384	473.0874323
394.2691919	1.373671375	1.284776797	1.7956497	0.124415549	112.5628442	423.1256144
277.3365014	0.867173457	0.565587865	1.27060771	0.041910181	78.09926659	272.6152562
571.2834624	2.881335109	4.156448398	2.33430885	0.103404586	223.9784179	518.9607535
602.6484371	1.444538179	3.158501516	3.318964769	0.199771499	176.5162792	598.0032858
420.9424341	0.516175921	0.83044492	0.999428579	0.047963296	138.9812299	408.7685336
313.8130279	0.785516566	1.166495302	1.462113084	0.048795719	155.3862956	373.9825807
435.8202198	1.758941121	1.853772535	7.181691879	0.052476997	108.5531338	355.4493008
291.2884214	1.315635421	3.651739128	2.459434445	0.139251352	154.0206812	263.3608738
483.359741	0.995029631	1.388154918	1.920778196	0.077550308	134.6092148	443.507153
1039.475517	1.724717798	6.622458771	2.481656193	0.164901816	269.7328375	854.1853427
548.1220162	0.696658249	0.792310546	1.028983874	0.075556955	57.98554471	444.171458
403.135447	0.654152283	1.213023572	0.676718271	0.056996462	84.91394832	385.7598629
445.3106066	1.161847116	5.860548343	1.786110912	0.190134864	182.2333729	475.6790238
622.1173342	1.502489107	2.664380494	4.429222523	0.083529655	165.9961948	644.964553
512.0655256	2.321441913	2.72771712	3.941603521	0.092977299	247.1443177	407.8035309
311.0430375	0.445577892	0.669133359	0.580087255	0.097017701	158.0878645	231.6528902
428.0968976	1.112112836	1.936046116	1.168666113	0.091439427	161.0689119	316.1118274
502.0498343	1.014704523	1.486290928	2.638524515	0.083567226	136.641121	400.2650425
685.1762887	2.75356409	3.944181947	2.96866246	0.141609893	256.3222191	604.715471

710.5476081	0.737885456	1.391311137	1.392908952	0.061658132	149.8540047	573.3459693
592.9683602	3.443509848	3.548377939	3.657688171	0.144038459	221.681904	457.1422298
600.9083131	4.749464501	6.49337836	12.0073214	0.185189179	184.0060485	534.0546115
511.8803008	0.762290651	1.095123632	0.914797435	0.060057828	97.28569337	431.5515947
1447.4113	4.440575679	9.823340981	13.45704892	0.271198426	489.3170145	1404.467849
449.8916932	0.586352739	0.644037568	1.627438125	0.093762646	158.3997061	349.1321488
525.8692656	1.240197883	1.955328022	3.038575236	0.139750567	282.4300223	511.226706
572.3344932	0.394646212	0.492831756	0.490747167	0.069462735	97.75481596	394.9046967
707.766019	0.45389012	0.955204339	0.416794709	0.071324457	156.4115038	485.0399458
418.2924708	0.924415756	1.81916564	3.046317652	0.149657525	232.2297179	317.5074669
585.7540186	1.190210098	2.67269658	3.302642891	0.179958363	370.2531889	527.948511
546.1332685	0.995278807	1.224916193	1.06369951	0.064259322	154.2727672	531.8591515
483.7249435	1.318470605	1.316559486	2.331330146	0.07985871	182.0146161	379.5207313
430.9761272	0.744282081	1.775410136	1.080884401	0.131848426	312.1393892	553.1865776
348.0717527	0.562434732	0.800569717	0.460083612	0.052819195	103.7164113	223.3615508
534.3338526	1.185340008	2.545124202	1.909171024	0.128603332	306.0473381	589.5222994
180.8446532	0.176962697	0.295886043	0.392288374	0.026621485	105.963896	255.793565
655.7585291	2.872060321	3.64630328	8.822248193	0.192811593	458.4814741	624.7155188
520.6310119	0.787040719	0.841335879	1.32725106	0.085610129	151.6071935	405.1751546
306.7088926	0.581210072	0.637492755	0.680450449	0.050193219	102.5496962	276.0011985
380.9428363	4.621557923	4.03317613	8.274089406	0.077385479	236.5757286	576.9058867
389.8820076	0.907640194	1.196159154	1.527752638	0.046523431	148.1969463	329.6541228
551.6476234	0.832570581	1.519305686	1.095738659	0.125645991	149.4898578	466.4844863
416.3159852	0.706669521	1.011986802	0.617936111	0.054030948	103.7660728	487.5079548
436.5331761	2.106847418	2.344479119	2.299042926	0.118284425	182.2193234	355.8451936
621.0853979	2.657746722	2.505017289	3.362818678	0.10780099	226.361531	534.0741929
372.1133163	1.481714136	1.641738499	3.435059116	0.072498825	138.0750511	284.1881414
521.7325117	2.762904761	3.403902379	3.322708828	0.110923184	143.1924335	350.4496132
349.3133896	0.762455292	0.583927151	0.881998649	0.068436391	99.6731646	313.4449127
341.1703691	0.865403627	1.113006816	1.330201691	0.039215086	90.56949013	341.6272745
346.6580469	0.524489935	0.742293291	0.518291114	0.052687204	126.8881607	302.7428561
441.7072829	1.928968134	2.761971436	1.984671439	0.061624152	207.3039673	484.1101973
511.5022638	0.45145909	0.906501726	0.464129429	0.070121653	161.1414421	330.1503542
752.9128286	3.508487807	11.73792071	3.754969418	0.250965479	417.0906922	715.7742183
556.8575644	0.689202016	1.360479158	1.188272233	0.08482095	93.34768738	360.4269796
338.3083156	2.23640864	3.093643293	6.106843511	0.096730802	175.9213041	306.8981944

291.9292736	0.59539681	0.863702488	0.499964811	0.048138317	80.43144544	237.5453855
439.6447287	1.416488793	0.790667487	2.785803116	0.103880052	171.5380679	397.4098612
490.409212	0.437565326	0.787229589	0.531124875	0.067384128	81.26738602	418.5924143
848.7071492	2.366993799	1.805395512	4.58754554	0.126150276	103.591779	826.6182907
523.7115821	0.593129251	1.19791047	1.094135077	0.073551952	184.8815619	398.1923911
795.3560067	2.037333133	4.100470452	4.682724442	0.174473511	182.3733352	780.2333864
624.1418511	2.135246461	2.804694904	2.429549249	0.138021855	267.4923805	517.8913063
425.4384859	1.789424885	2.929712584	7.382024068	0.20978085	296.7530249	385.7240105
332.962037	0.565640063	1.319800173	0.999583601	0.067825822	103.471083	231.4572072
534.795361	1.167552713	1.563027872	0.949162403	0.082843684	170.0624775	459.3063483
407.4102541	0.512595278	0.754752247	0.598867757	0.041144542	67.95064827	390.8090751
451.2347043	1.452892699	2.205609004	2.4133806	0.108536999	127.60272	353.9671814
300.5907965	1.322263404	3.602368529	5.312010591	0.15594771	301.3990802	352.9301866
617.0113946	0.385819418	0.733323989	1.098835519	0.08285455	159.3852659	488.7148173
294.6756199	2.374886946	1.997221292	2.03809209	0.074917538	189.2833288	279.6692511
487.4179307	0.539215244	0.794889194	0.734758152	0.084879395	67.41058402	345.7513715
529.0487583	1.894401123	2.670129683	2.689640178	0.10649016	197.1609632	404.5228434
501.467476	1.245367169	2.928369566	4.386432495	0.102344161	322.0085504	479.9927389
339.1264509	0.728615309	0.872216142	1.096003508	0.073860075	74.35025407	241.7894873
269.0029221	0.600897566	1.37216193	3.011180568	0.06807799	137.1678841	244.9557842
574.5870927	0.678273217	0.895910865	1.375469258	0.069720523	150.4891927	468.5629535
709.6936789	5.309965142	18.06151667	16.27759864	0.267598912	393.2913729	734.4268065
431.4326004	0.179896742	0.375448371	0.298380063	0.054635013	175.7232159	390.6055235
512.8136437	0.583185092	0.777720816	0.983182714	0.081777964	95.37771897	429.6307541
921.8865181	3.55428815	5.106661464	7.190969938	0.278535133	332.8463752	925.3378321
626.6301104	1.690060271	1.494465313	2.927389967	0.117431277	237.7297603	540.747549
677.3490826	3.23759398	4.874101353	4.463960724	0.137743436	282.5584054	842.2798456
262.3252518	0.363073508	0.305505396	1.168801607	0.069222524	36.26977051	270.2710869
277.7219554	0.323559508	0.468182195	0.457768815	0.04602212	110.1579336	199.2473552
334.4684073	1.169587006	1.236092319	2.177244077	0.047372618	145.018062	356.0343904
344.7159744	0.613276106	0.613978507	0.76171344	0.040191774	52.72259004	281.1336494
376.681121	0.731030771	1.297880993	0.744879434	0.068177539	129.160578	335.8440231
586.3113294	1.351166239	3.087419076	3.870571468	0.139966589	257.1074133	538.3025015
299.1170976	0.722476842	1.182505844	0.956827347	0.054451201	112.6476616	229.4252903
586.3065743	3.084402255	4.663295261	2.516684453	0.089245359	258.4521997	489.7686996
372.3033682	1.485723231	1.656421848	2.698005912	0.110497976	283.9122959	434.1784806

596.6255317	2.090645817	2.99262919	2.899500883	0.126347304	128.2305777	518.3426832
653.6845372	4.713437273	5.724192676	7.043664111	0.153151305	209.2917912	503.6972902
513.6903175	1.217922188	1.556811931	2.06819244	0.100033568	180.7377234	476.7673785
627.385529	0.869809373	1.52505286	1.754932791	0.070370626	185.9523642	749.8530309
223.3123904	0.827131114	1.204620717	1.117071906	0.036758225	82.6979779	282.9618513
371.9901476	0.410509409	0.516822473	0.326216063	0.050583705	67.28626833	218.0814824
477.358139	3.446258303	3.18172159	4.140041447	0.104697102	87.51133481	412.3321624
564.4042076	3.536117635	5.616460017	3.693261182	0.210846009	157.9407743	447.136031
409.746252	1.396682596	1.946607732	1.096027997	0.052203549	123.3939851	502.0349963
575.2145038	1.086711343	1.918692153	1.458775104	0.081476058	113.7975585	553.0721427
550.5854788	3.189247726	6.493821377	4.68178223	0.14417364	185.7378913	462.644764
747.8166674	1.140542998	2.93028602	2.418725358	0.078742166	269.2856317	795.9578652
685.1841399	2.353918844	2.881161521	4.463181846	0.145891057	267.4732749	663.6392497
514.0698187	1.281063081	1.70712594	2.105905523	0.091606922	225.9875645	431.4098001
221.2460378	0.389227883	0.462239916	0.432037209	0.058562173	61.36202855	147.4611817
629.9293435	0.825009579	1.275327378	0.845702068	0.090032547	135.9079817	433.9015429
375.8594449	0.496223999	0.545775619	0.702203497	0.04809533	86.59974281	320.5072286
547.7640656	4.030619037	4.435654063	7.433015524	0.120992564	293.99458	396.1030429
473.7416067	1.604082882	4.004925708	2.405371051	0.087656005	128.3556004	315.9203646
523.689174	0.462171763	1.151873159	2.372368079	0.094940372	211.6639352	509.2604437
381.4135021	0.532742433	1.212556373	1.273423183	0.05477858	240.0428244	326.4063385
448.7109425	0.790461012	0.997738736	0.8139441	0.103441122	132.1802001	341.7963613
501.5509846	2.436368496	3.758073365	5.386522387	0.11942238	184.1555808	523.1108029
526.0018081	1.672036566	2.121899247	2.527563434	0.097849198	230.5360326	486.4385536
389.2928498	0.40875736	0.644268536	0.593893456	0.069892671	120.0598902	364.7380176
844.6618121	3.444353008	8.418260565	8.344251796	0.257826922	218.6703698	822.2816763
342.9937781	0.425486988	0.753749633	0.686638963	0.04869698	158.6122568	310.6660333
538.6620526	2.403469663	4.108119735	3.445317963	0.122440455	194.6013476	408.4665852
312.3165589	0.772970504	1.024787007	1.434942936	0.066181295	148.5713147	251.1873032
370.7738893	0.648510323	0.80440111	0.736418413	0.055323837	94.67501558	341.044047
510.2058903	3.772186597	7.398380741	7.432461623	0.135021085	269.4854686	726.0091909
255.4880926	0.178015619	0.303460931	0.520305187	0.03808141	121.5037583	282.4245366
314.352542	0.266079492	0.410774261	0.430717747	0.039180692	128.0350117	223.9842245
394.5253103	0.680989841	1.123611469	1.197633975	0.072902075	128.3793568	304.7673618
286.8435738	0.720367338	0.831650606	2.289672849	0.050646828	62.9219125	372.6244481
814.2315327	1.172406998	2.770845225	1.768603009	0.118220355	301.277271	689.9015756

343.834232	0.450277114	0.544764953	0.645231406	0.045933816	84.31327416	294.2220891
664.2726644	4.091401321	5.658244383	5.787347592	0.132494786	213.521386	530.4525445
478.3297809	0.60090236	0.803230989	1.075833184	0.073069504	108.3870569	538.2367872
542.4186611	1.013076593	1.667979783	3.104348675	0.092808312	236.5901748	574.6574909
478.4190261	0.815607985	0.859061368	0.678101626	0.088545196	90.04161814	382.6029599
617.1065542	0.978001075	3.146404732	3.116993212	0.123921649	214.9119464	477.7128734
448.6883007	0.927572887	1.330533938	1.548010228	0.071830501	132.052172	424.6432812
552.3481572	1.896024298	1.795311596	1.775810941	0.108486499	141.8557327	422.9938607
627.7400254	0.834493791	1.90637461	1.928234204	0.149083227	199.1764743	703.4893472
884.7129213	4.749738376	14.63199187	10.10746176	0.135940422	555.1282468	739.4690009
337.7572096	0.489509775	0.553900765	0.687683177	0.043521685	104.7864781	397.0083024
697.6434684	0.871317826	1.779954959	2.524078635	0.13335168	133.1893202	549.7183179
225.7770061	1.235446552	1.539138866	1.501493288	0.116020942	257.1489442	222.0975417
673.2683132	1.638672208	3.605568418	4.904440317	0.211716076	321.183485	617.5917496
531.4319008	0.808519021	1.578765179	1.930913239	0.118395666	271.9209832	481.1119391
260.7142346	0.227278643	0.362410682	0.414550588	0.026355871	47.0570892	221.4234346
425.6628753	0.954374864	1.064528023	1.289579232	0.088770797	117.2836133	314.8852582
430.7683569	1.37028502	2.364826681	2.804904486	0.090356036	205.3733098	430.6031694
338.813924	1.855766069	2.639186957	5.422919445	0.110195228	188.2573227	390.6265206
510.3827257	2.605402324	5.655460509	2.976680811	0.096462486	220.1561778	398.0321172
556.2200986	0.943302517	1.183989103	2.331788268	0.089330973	107.7173522	515.5742442
466.7678135	0.960970012	2.066536883	2.229164393	0.086441252	173.9432431	341.7519082
381.1558165	0.77389683	1.551256799	0.824057248	0.027746912	83.95659335	503.8542881
479.2260929	2.261834789	3.008120597	3.886876991	0.16013317	169.8248592	418.7767547
328.2406881	0.354238594	0.366078643	0.409463238	0.03751956	61.59695913	275.7092067
792.8498016	1.814866634	3.203403266	2.693821919	0.099646508	237.8888995	680.9649242
422.2322405	1.083860345	0.736207354	1.661095468	0.068064221	114.5188198	446.9662814
785.5313066	1.999356832	2.123490938	4.480948804	0.125748807	183.9612573	631.4616744
377.7589743	0.568925513	0.835023331	1.054355837	0.054406888	117.2394643	388.70585
340.8786876	0.637636516	0.972507034	1.114542882	0.05703884	128.3463031	254.5763118
787.874642	1.983280379	3.32923111	5.511755736	0.149122852	186.4525285	730.5779366
425.3729305	3.809915855	5.4470173	12.10852242	0.149922252	182.3984244	393.6571492
676.375801	2.348264598	3.295467612	5.82140374	0.180529626	208.5646054	640.4801338
301.9913635	0.785142759	1.434541993	1.700628134	0.066480006	114.119061	245.8438498
201.4899799	0.359021316	0.476286756	1.453196062	0.069050186	122.0404157	279.2282209
370.3555742	0.348562824	0.492258297	0.349774185	0.04401267	72.49433782	291.9316919

449.615016	1.668775831	2.921976245	3.72009846	0.088954794	263.1989689	398.4302822
321.2655109	0.790512576	1.360795455	0.983007916	0.04762435	99.65724513	279.5135224
657.5471091	1.743500151	2.180794941	2.079919188	0.100657966	89.14476067	551.3125324
520.5283674	1.05078295	2.172484245	2.168987708	0.056102762	270.2545439	609.6531576
297.4033017	0.29405752	0.65570796	0.489463659	0.073623315	123.8673983	240.9229679
298.7726434	1.027057545	1.226459218	1.067510884	0.037045314	162.1800386	301.83635
590.7317346	2.007111705	3.284541962	2.924213917	0.121333479	141.5785941	503.0099161
494.9008338	0.7065082	1.211745086	0.865211736	0.062628404	207.4252853	382.2479564
368.5038535	1.227303623	1.411615744	2.427713844	0.111222869	132.5868295	441.0705968
293.5511088	0.379898273	0.581067218	0.509645785	0.040507558	115.8252104	231.8517529
509.4844931	0.749307413	1.212582329	2.040434921	0.101900311	142.20294	595.262121
282.6014047	0.481345019	0.401901167	0.54417838	0.05830161	90.45348039	174.3567729
429.6377606	1.01390946	1.322876291	1.430381632	0.060287884	157.3921158	498.2620944
627.0735999	2.147216837	3.888739024	3.253010008	0.108580877	206.3216554	549.4781739
270.7320484	1.160863024	2.505551425	5.592455383	0.041973758	197.2582866	325.7684637
256.0924737	1.175339749	1.726562293	2.851726557	0.052238732	76.58759691	244.692975
468.0193216	0.500962728	0.716464172	0.745788139	0.06347072	92.05143069	309.9474622
363.3384483	0.760477837	1.121061078	1.081505008	0.097191109	150.2414234	277.9039567
985.2570701	5.637983406	19.33786549	19.86148338	0.315792908	525.2842328	985.3773285
512.7404798	1.162874794	2.004984254	2.176510163	0.080276672	130.8042513	439.6868754
331.860867	0.670093108	0.741297864	1.129811896	0.083101093	134.244789	296.2232053
400.0595538	0.828200669	1.284832652	1.195543149	0.089526523	154.9478309	286.3929498
531.6124107	1.203509298	1.505254861	1.92738177	0.061442277	136.2643499	570.4098225
445.7342774	1.049384673	1.683319759	1.775170504	0.078302984	173.2634641	504.5332718
776.8218289	1.063866092	2.216977158	2.223738213	0.104593854	279.6404892	666.6285546
402.0352951	1.706858887	1.653025698	2.250334122	0.063195437	200.3036398	298.8345786
420.1287305	0.802686356	1.188978341	0.640446462	0.077079644	120.1882222	354.1959128
594.3266535	1.214525615	2.275365626	1.683666354	0.083390598	261.0175702	550.7767434
249.9602184	0.33893437	0.619419695	0.99702917	0.07843572	201.7869989	278.7380802
368.6862932	0.570228545	0.751758518	0.509241176	0.058175936	115.2794898	306.2739625
395.1304569	1.987696756	2.428996092	1.958029979	0.068997172	131.6146868	366.9672431
880.3035136	3.381575538	4.480689297	6.470798171	0.168837642	285.0095468	711.6708611
367.9429638	1.85818943	3.754017727	3.065906562	0.082818498	196.6557026	383.1252294
629.6909002	0.792968843	1.04697213	1.780329116	0.098162027	113.3778157	539.829356
320.4204716	0.856196499	1.617475522	1.141340418	0.078534775	108.1887214	326.1415153
610.8082877	0.774339638	0.978215506	1.717287896	0.098621072	131.8133906	694.42179

280.9758858	0.607542943	0.71019542	1.212800423	0.053795875	89.76301349	254.4668195
448.0470596	1.31143187	2.316927969	2.475709222	0.091022327	169.5007656	622.0805953
321.7891554	0.680581217	0.950660505	0.757855182	0.049381777	132.5082653	363.265531
457.127042	0.473610567	0.84972991	0.795126319	0.0574211	114.8555019	383.3685223
429.1267439	0.456537825	1.011940217	1.207479924	0.060231555	130.8811731	488.4746517
651.5704524	0.662139059	1.024718594	0.901535361	0.113136263	77.08254507	709.7803017
700.38102	1.446601797	3.600079306	3.211182787	0.131844255	269.1667039	570.9578481
944.55721	1.1420195	1.937715588	3.380002701	0.11890874	221.5725702	953.7529644
524.9625566	0.999254591	1.514476415	1.287768102	0.077566062	125.3562905	496.3687397
324.7306781	0.723908902	0.814503802	2.012452539	0.062226468	149.2416569	287.0600411
619.1742679	1.398867551	1.521283122	3.608051006	0.149139852	325.5280995	524.6490575
424.3471898	0.425305844	1.094379974	1.071599444	0.144432032	152.8495554	496.2007515
563.8555188	1.75724201	2.133059731	4.051348667	0.11634155	193.0723745	637.4232147
442.5802626	1.547204588	2.253373538	4.117480182	0.106902994	174.9656391	491.716578
336.9981245	0.580611287	0.883901002	1.348620458	0.082913354	134.8678286	296.0182656
528.0036236	1.147560026	1.218878344	1.393391311	0.083449894	138.0178466	461.0006799
826.4033967	1.420620394	1.60359574	2.287909918	0.114660578	88.93796997	647.0856022
427.055794	0.603920572	0.528676532	0.562005769	0.06327296	146.7489827	348.2162985
324.0651272	0.308658423	0.38127997	0.300677109	0.045870834	62.26449286	208.6602317
143.4523951	0.248480069	0.160770534	0.17219543	0.019376123	24.13042969	93.79673014
463.0885945	2.379454952	2.51225873	7.215693043	0.125253246	155.0316501	470.6996696
738.0646275	3.814808633	4.086437493	8.351690836	0.184383395	125.5337273	662.4387338
330.3317965	2.517185189	2.85536174	2.440417119	0.096891832	153.5324113	277.1869673
676.7144097	1.846820033	3.523014587	2.824099713	0.117689535	205.1502513	551.9806927
547.1796995	3.73146362	3.753481569	3.216646176	0.095517816	229.8510262	521.9082073
266.5812097	0.275160502	0.262888613	0.322755929	0.027320101	33.63053353	207.794281
680.637744	2.540838537	4.236651235	7.786745827	0.158303818	232.0392812	668.1904254
360.7621386	0.819522733	0.983060642	1.585550578	0.082616633	183.9615198	298.7658441
327.1883391	0.586921319	0.777979611	0.716031325	0.047055601	159.7268491	276.0909408
327.9548044	0.408306452	0.585495456	0.501994294	0.027733107	79.06187932	337.2555263
494.035867	2.198342475	3.417282269	2.012104437	0.08579631	201.037165	673.1379995
515.9123903	1.254007137	1.753973242	1.789819851	0.111335111	189.5053949	492.3311834
484.1237732	0.556803454	0.819842999	1.799847709	0.101309859	145.4194576	486.9968477
680.3094916	1.653992537	1.982448495	3.876767025	0.165238273	84.40661596	870.6841178
429.9163319	1.538790904	1.992608022	2.367683764	0.112229493	125.2942322	394.6456847
270.8628843	0.462797141	0.798903975	1.529751259	0.060042494	121.6078347	204.6357579

583.0145279	0.954942809	1.300043368	1.380590073	0.100837156	127.8178384	457.294567
209.6406655	0.610880644	1.022351562	1.130626704	0.051462439	79.857109	264.7190724
721.2237117	1.475357918	3.774356051	2.720633541	0.098509604	254.7353042	594.8947249
626.4798934	1.649006011	1.781926235	4.642644619	0.162861256	272.5232068	525.7759724
604.0516732	3.051596207	3.853615683	5.823662493	0.138245605	156.7095925	579.1962462
570.8394985	1.021211542	1.478440112	2.427494048	0.088137794	144.3692673	623.3191268
492.6698189	1.444357971	1.883916418	3.22070483	0.111680351	146.2902764	483.9909642
573.5058634	2.014438283	2.053724842	2.205495919	0.082864997	247.869521	535.8790658
403.3170332	1.337055736	2.162820132	1.628697019	0.0747363	140.4117515	357.3168428
1031.499624	3.765025359	11.03215867	7.209819105	0.206022286	493.4646257	821.3669888
405.628963	0.900304964	1.316432155	1.121419976	0.063691659	133.8812431	359.7612371
524.8870349	2.122977254	3.297131157	3.816822178	0.141140293	242.7058715	501.7482773
527.2183073	2.131945764	2.352801724	5.748532221	0.089416897	135.8274694	426.352131
770.8843897	1.455376937	2.198498697	5.093634833	0.174506045	168.7679037	708.2653096
594.5357419	2.805352827	3.581627286	3.964902882	0.145745461	286.2730648	421.9756361
711.5439521	0.95693059	0.92111978	1.96074873	0.101482719	183.6871827	669.7324285
567.7098174	0.724354151	1.492227882	1.51058628	0.083346861	152.9786498	504.3107112
405.9352565	0.40140341	0.623660405	0.56633765	0.069407317	98.87139788	309.5669447
434.1342048	1.258255084	2.372251054	2.318312109	0.091717697	131.9171048	494.6363117
409.3475409	2.457945157	2.786617923	1.779666921	0.103883049	178.358003	395.32536
296.2008419	0.486739544	0.678062906	0.608513988	0.056074711	81.32863875	250.3016995
340.9591733	1.016733104	0.949518457	1.050321382	0.074500982	71.66692748	256.4557
533.9896801	3.376709964	2.583988027	2.732955771	0.148606865	152.790797	462.6482984
429.5271624	0.989845465	1.056781504	1.408480008	0.052134854	90.14539714	363.0057671
423.7137938	0.657096965	1.037058211	0.950061122	0.093598088	115.788233	344.510258
397.2793283	1.300433255	1.863391344	1.614064352	0.051460863	177.4782411	470.80731
260.079197	0.26518831	0.35869018	0.263462982	0.036283365	56.70886392	180.3977553
307.6926413	0.360864347	0.528416894	0.421265982	0.042895353	70.01476636	247.8773609
282.979558	2.527777846	3.365972315	5.140303346	0.127948087	223.1354795	243.2317276
490.6262803	0.638544298	1.34047101	0.975571457	0.087738939	171.2542019	405.0732139
499.6737343	0.631208551	1.226345094	1.20646284	0.065472807	184.2246081	509.5441792
303.4625401	0.433203647	1.16910697	1.510588907	0.066408613	110.1137167	259.4921259
392.3418485	0.659744182	1.318431474	1.247854159	0.067968925	151.923904	319.8605743
682.9633364	4.513010849	5.114000478	13.61312379	0.209593421	241.4610104	621.8723452
467.3154227	0.565959262	0.495888912	0.554729151	0.058302529	91.13568935	322.0049777
665.3372152	3.070820151	3.935054662	5.450890114	0.173567681	215.0866218	487.8215827

521.2604464	2.269146579	1.696871932	2.401582416	0.108168766	137.7579314	345.6906945
366.1011394	0.99332759	0.624994047	0.775303922	0.06591887	132.4060625	373.9272754
545.897352	0.708220148	1.116407653	0.723688033	0.082500872	116.8215227	363.996273
244.0794349	0.454731508	0.991470494	0.690168508	0.032679295	161.0907841	345.7667163
542.1523071	1.302161168	1.725743074	1.055135945	0.077861214	105.6826454	389.8334296
1025.521638	2.748756025	4.505956371	2.103824049	0.131957405	372.9917811	926.6857307
538.2309531	0.375401142	0.954168724	0.784421363	0.076851735	189.858201	445.5719335
299.1464563	0.797775726	1.295543569	1.271811163	0.056681237	179.3170299	322.1247756
567.6123709	4.017949788	9.337648493	8.477161622	0.222134993	509.6050828	631.6214423
266.1057491	0.758108476	1.075329094	1.148596756	0.040861545	64.85593929	253.9968194
391.5481379	0.949053703	1.663443512	1.639680315	0.091747607	263.5745928	464.5365867
825.4130491	1.609906236	3.862621861	2.836171616	0.218268217	187.7745678	636.6053148
509.6844898	1.735921102	2.941034272	2.611767118	0.095706878	263.2606109	609.8266934
285.0705059	0.653816455	1.573011616	2.768132752	0.09659516	201.2138906	373.216252
355.9525314	0.391756743	0.573811299	0.320264132	0.025341113	58.07983924	391.7654052
255.3446951	1.641856683	2.322070243	1.972084058	0.078009198	113.774059	270.578268
398.2201426	1.084221537	1.312372405	1.066457206	0.06739748	91.24737535	287.0274814
366.3656014	0.533978182	1.05396296	1.214708452	0.084265924	215.6799552	361.1441148
320.1608089	1.525270567	3.052654972	2.544282737	0.047480721	95.82236068	348.9498115
857.9047743	2.127344014	2.215023447	3.38032881	0.151683727	207.4423558	803.9996981
285.6146353	0.254442135	0.493433979	0.35064631	0.048148654	98.0789332	194.0153743
295.3805801	0.279183123	0.265155875	0.202555754	0.042052637	65.21333073	203.7346078
424.0565095	0.763761477	1.263253613	0.985731818	0.064778594	278.5106031	450.8119735
295.2530584	0.675711596	0.900259249	0.650486211	0.047265438	102.6314367	262.4661274
416.7668713	0.972635271	1.905308003	2.212056376	0.097421114	187.5404664	474.2105651
359.0660787	2.076036357	3.521876193	3.661195362	0.096501187	121.2631528	350.1898966
497.0370955	10.22484105	6.205772324	17.35932846	0.153045398	661.0849691	806.5206675
367.5835652	0.705698359	1.370744602	1.77658503	0.073063348	196.7607113	366.4278489
393.9669127	0.612904809	1.168375232	0.988060991	0.045529283	118.7890524	492.3041931
247.8375691	0.459408086	1.161798763	0.615877257	0.08923422	85.68115612	281.4139147
651.5848029	2.72339764	2.654258008	2.427234951	0.136390745	226.299218	500.8413295
289.869133	1.709511476	1.743469848	3.665614742	0.095264915	142.2674267	370.3241255
245.3945162	0.982910633	1.16828506	1.100979946	0.048900772	55.96257343	232.1334195
430.4726609	0.490970112	0.952230377	1.431015515	0.060556282	112.0620558	425.7195736
479.5451019	0.942348692	1.656263334	2.616480109	0.117239651	139.0119409	690.547512
399.2542916	1.009086634	2.001161668	1.374412602	0.05794283	143.4141526	440.5405766

353.0849832	1.319973047	1.703082886	2.652829285	0.092204189	147.4101829	348.376644
252.5371553	0.257328644	0.315722595	0.22993336	0.026849891	51.50086632	213.395466
319.3879153	0.844350492	1.415441429	1.259261344	0.069784639	152.6039627	334.2295066
271.376106	1.604878106	2.05437434	2.440901188	0.076703369	301.5984632	422.3047701
296.3499206	1.082698262	1.756457271	2.042455372	0.067141043	154.7840781	271.2209598
537.5584341	1.799896829	5.115482049	4.71115772	0.17173879	544.487245	601.7025423
356.3421956	1.459606499	2.144997155	1.937884251	0.08969387	115.9035314	333.2297696
429.2370873	1.289561352	2.021679049	2.304257742	0.096921265	197.7113536	419.2562633
419.157826	0.638748142	0.6516407	0.644872122	0.079012459	123.0894759	296.1450064
458.5223376	3.902038679	7.421642483	7.813931429	0.173227671	173.588874	422.7478027
347.6365633	1.033477329	1.750289773	5.780351337	0.063148552	293.1761594	387.7195074
292.7821033	0.664447559	0.483361146	0.931078107	0.035233192	86.464919	270.7960947
257.8167628	2.076503844	2.479690962	4.252023048	0.094470944	221.1720446	345.8458293
407.1387184	0.82503079	0.835038345	1.2543911	0.065985882	211.3269671	396.2855709
401.933495	3.456355866	5.953389245	11.51568848	0.112070558	195.1874005	436.6184904
319.1684697	0.849478845	1.686570595	1.419308055	0.038812569	97.67574832	333.9060553
382.3748123	0.950366186	1.028637249	1.434712861	0.069513159	146.0814457	370.9073694
728.6871751	1.006464678	2.450438316	1.06581487	0.092753019	192.8707746	660.3054536
354.2536775	0.510816059	0.833680432	1.217576588	0.062490273	149.0370771	355.8456573
181.2459187	0.486482291	0.665657864	0.723160317	0.049653393	142.9682796	187.842149
304.6259982	0.882334559	1.07534731	1.198244921	0.060914068	100.1552017	304.758174
431.5094672	0.626164029	1.061773369	1.030004006	0.063186303	125.809603	314.7442784
743.4017378	1.052689761	0.791126051	0.953057364	0.08717189	91.75665802	551.0766312
239.2598823	0.345716695	0.776266027	1.29206295	0.048799862	178.4249419	273.6027279
351.4469445	0.839209483	1.932822241	1.021705901	0.048941055	199.411012	380.0347766
414.3703218	1.175170413	1.876136595	0.861951805	0.097434556	151.7700584	341.2804946
547.2144174	4.046267898	4.838449336	3.678690463	0.152305587	312.5953998	616.8480516
264.78758	0.646452104	0.922517	0.873674654	0.043519196	98.0481209	243.2057516
218.0312151	0.453416116	0.581445448	0.628038464	0.023895679	73.68791934	243.242652
387.8716093	1.761881227	2.657225995	6.486778159	0.138179021	267.8982059	347.2288364
520.8603115	1.06862093	1.461329284	0.979774406	0.085491333	252.6576193	529.0738689
237.48978	0.819279339	1.019701583	1.819970898	0.034916579	78.32218437	283.8372772
225.3517405	0.242618007	0.284089742	0.351160495	0.030373556	48.15315716	166.8053939
589.094444	2.440136832	4.702082957	8.612972473	0.169200328	331.2987052	729.4602537
320.3496889	1.178221422	1.330474815	4.133592508	0.05218281	90.27032504	310.0284714
300.7882233	0.580926312	0.773994412	0.744895096	0.050605517	130.5182233	253.0901093

452.134543	1.268465905	1.522231516	2.105794444	0.060619175	219.9571746	502.9844097
165.6823793	0.450790744	0.314225576	0.279847622	0.031721858	197.5916747	315.9003788
459.2808705	2.272902612	3.422161032	1.990929552	0.077485657	203.0695772	451.0894733
444.4943076	1.770392174	1.961740408	1.854810589	0.076286542	214.8237026	427.4406963
448.7038318	1.145832659	1.347130508	1.018779	0.083866153	154.2024931	403.0650132
410.6594649	1.373471941	1.731656495	1.569503223	0.103725929	152.8344405	361.4967171
366.8607369	0.316067044	0.621593753	0.459723319	0.06338351	52.95121978	405.3290694
249.6597692	0.532687908	0.695684221	0.485513499	0.054024393	87.97779039	174.8355188
426.1533153	3.075039924	3.467281825	3.305998286	0.06994989	163.3790316	404.8082204
469.661782	0.971272497	1.638791721	1.999718851	0.084470995	258.6764455	469.7344869
402.4212833	0.695211375	0.916193766	1.194058787	0.069327272	139.1777224	440.599568
279.5796154	2.032365659	2.34829579	1.677428242	0.092597155	126.4698626	309.016509
297.6449238	0.349086671	0.464118114	0.540785321	0.062290588	125.5565726	397.3522533
466.2734933	1.283176512	0.961055844	1.786131756	0.07298391	119.2465748	403.512305
154.6028763	0.414524644	0.416093766	0.788281522	0.022514224	117.6636343	187.1945256
283.5479136	1.275452372	1.065269263	0.789260405	0.056380556	47.71669783	200.0302024
388.8875987	0.498919896	0.648645606	0.610512039	0.04052323	146.7123411	353.1150436
394.6830654	0.581547939	0.90717966	0.677712802	0.057442531	104.4249154	403.0556149
488.4195917	1.509089506	2.064942544	2.428753706	0.081096706	225.2687876	437.7856082
369.3701474	0.802415763	1.549164483	0.752835878	0.048420547	110.8842811	359.9044217
455.3019495	1.916775362	2.477181757	3.457136751	0.110383028	211.8362981	457.4891174
425.4238799	0.350752384	0.760627413	0.806246721	0.068972689	147.6701632	448.0641393
465.5365139	2.113017819	4.357223542	2.747139374	0.09640331	188.3408092	452.1289716
401.6563689	1.545817607	2.356823093	2.692233696	0.051985475	122.1993752	459.2706322
564.4194599	1.250973175	1.322668194	2.061339552	0.101597438	169.1454767	501.8336823
387.681096	0.814602437	1.438850078	1.549347762	0.064486402	148.0752482	421.0560937
523.2288707	1.248931956	1.723155576	1.211058983	0.095121255	162.9613608	441.82938
1020.827365	1.379674335	2.324259172	1.674114398	0.119017609	226.2157459	877.8264035
369.3721049	3.244059724	4.682911533	4.797920524	0.095555079	186.6971037	392.2205351
560.0967528	1.660829676	3.456839162	2.130016358	0.093430794	165.7036075	490.101245
462.7069793	1.77552796	3.259560533	2.544012623	0.087814282	144.8555426	455.8363077
376.2137173	1.561346046	2.405353001	1.809022828	0.067243405	136.9107971	409.3778134
247.7313824	0.523731236	0.896888959	1.553006619	0.06910926	123.3376607	214.7603415
421.3999417	1.18859669	1.987684688	1.566038327	0.074038186	175.5387791	449.1409148

Fulvestrant_1816	Vincristine_1818	Docetaxel_1819	Podophyllotoxin bromide_1825	Dihydrorotenone_1827	Gallibiscoquinazole_1830
102.3392606	0.290632785	0.113348397	0.655032962	2.456495107	17.63278337
125.1628716	0.126517063	0.098781907	0.40171506	3.495719324	13.5792651
101.1383021	0.302837808	0.278120031	0.923864773	4.148478865	24.42501247
109.7776374	0.268761328	0.163505637	0.528848356	5.029971501	11.3354567
135.8978635	0.721943934	0.431430016	0.923334178	5.623286054	23.62582426
71.9678205	0.092105716	0.099241605	0.320062512	3.082838064	11.02367611
104.0718875	0.128825443	0.112169258	0.535187339	2.68375169	16.03524443
150.7598645	0.585275047	0.256738537	1.011881423	2.644421154	14.39277305
173.8413477	2.010347752	0.913384248	1.476771589	4.484420775	26.04488846
71.48971067	0.046921462	0.043404183	0.271916723	1.89093411	9.359320213
67.01483776	0.106319917	0.157686404	0.463173188	0.90554408	8.317777505
118.2721094	0.24160744	0.241880556	0.768843864	2.219278519	15.45152694
72.87777595	0.07641264	0.032013616	0.293715431	2.265672281	12.37653377
127.8866556	0.809452292	2.479271179	1.310746092	2.775588683	15.03553387
99.93915444	0.215555677	0.11110348	0.576460812	4.111246517	19.134492
121.3124855	0.235206192	0.361920641	0.65982757	3.687704102	13.22294838
41.48487902	0.029679024	0.014244843	0.216494845	0.911184716	7.45355997
170.6442904	0.481634873	0.139111964	1.284754766	3.205507769	19.12622467
112.2891648	0.187486095	0.166436185	0.61445025	6.261181419	17.51096277
67.60352474	0.02239918	0.021140046	0.203167492	3.332722496	8.805396786
96.16679689	0.118947438	0.105771078	0.507809289	2.25051304	12.81597636
109.188082	0.562999555	0.517828866	1.441446611	1.882002982	17.38552688
86.49839833	0.101008863	0.047681624	0.431044208	3.091213805	14.59264868
123.482595	0.710404058	0.203201898	1.005077136	3.793196411	26.63599089
109.0847992	0.292468243	0.185948264	0.645229572	2.655766478	14.26225265
123.5585043	0.12385291	0.098667822	0.533587483	2.367959165	12.16358759
126.0336153	0.719768652	0.828148983	1.280241961	4.835933938	18.17871272
155.7470683	0.270345489	0.117892839	0.580939365	4.561907605	21.50720864
86.24284313	0.070237981	0.032649776	0.313108597	4.021959717	14.24572998
63.65203571	0.054783214	0.059711202	0.242859158	1.502729297	7.035033743
82.36925669	0.069968394	0.089995318	0.388349119	1.985649265	11.50918067
94.20371693	0.108454725	0.03458376	0.406056159	4.129136581	14.1528475
163.8238618	0.652341381	0.304848692	1.442088344	4.048819662	23.62788541
73.8715587	0.1120296	0.040408106	0.435138641	1.361587988	7.255535235

123.3084243	0.404338298	0.157262145	0.799037814	2.476632429	17.14006298
162.8840675	0.374395211	0.330429987	0.907002876	3.838311733	15.05164015
106.5587868	0.065645164	0.031407781	0.358263837	3.714145101	13.6126407
139.7672315	0.274964721	0.09819967	0.566495784	5.558080817	20.2214638
119.3567207	0.356728243	0.211982234	0.745388796	2.991481172	15.25003374
57.85794121	0.109509309	0.070379459	0.464040267	2.163859875	11.17371951
177.5775041	0.496679311	0.13417563	0.56347624	2.19752124	14.196538
149.5579892	0.267508584	0.220594165	0.663692669	5.778653615	16.14614785
109.9147132	0.285618066	0.099947458	0.564811661	3.052443143	18.60473796
105.3893955	0.460970723	0.338687946	0.876270337	2.683185247	14.45594339
95.772283	0.280282046	0.13611842	0.713202105	1.339556875	16.30787255
35.42922304	0.013849025	0.009033113	0.203065696	2.1814085	7.549015154
93.0401337	0.101694955	0.086506512	0.410476312	2.721506375	13.23033139
90.17269791	0.127372619	0.117948285	0.558136011	3.621926823	12.81257311
78.81842311	0.07932156	0.085410102	0.335848862	2.316404992	13.52675311
85.70151047	0.075398061	0.034507433	0.327815343	3.448493136	13.05452829
48.94890146	0.032798627	0.031598775	0.206597687	1.322907795	8.011793357
115.5787142	0.125044015	0.12922566	0.558799418	4.772754301	15.82886278
92.06095607	0.096767803	0.078739877	0.477597372	4.792374713	12.08612909
72.57278571	0.03657477	0.021103888	0.292097556	2.717853319	9.919015885
98.43910199	0.149424296	0.060944507	0.387927575	2.502073699	10.98956092
129.995734	0.441162542	0.104902298	0.559522496	3.986194184	14.6955588
57.04156651	0.021393422	0.012962072	0.217878576	1.787140787	9.505759885
78.41486876	0.075706866	0.046733939	0.337945452	3.420226229	10.89784141
130.2121493	1.025678991	0.216272647	0.99452588	2.404535429	24.05170611
102.1661669	0.234658513	0.061435204	0.594736149	3.851173158	19.01234837
194.7341775	0.873171087	0.58593108	0.98209764	3.949480234	19.79085572
111.1758856	0.20986151	0.175477507	0.676995709	2.464206588	14.52032676
164.5661642	0.488127316	0.461664892	1.04161399	4.96511129	24.69026114
79.11615876	0.096551647	0.022574133	0.446535234	2.117551259	11.38833623
86.1295492	0.148864361	0.100753173	0.394963966	2.670618953	14.26457956
85.44441217	0.069419018	0.047509189	0.286035113	2.599218721	12.16167748
149.3771529	0.157168833	0.04288244	0.669847229	2.402018422	15.58266203
248.0789661	5.975408278	2.012073078	3.533802938	5.166050682	29.61878576
127.7139408	0.134496692	0.080603363	0.525234317	5.02305515	14.24571171
72.43418398	0.06703445	0.03653092	0.340858232	1.235478165	9.528693044

91.48764132	0.177599195	0.054145576	0.577014389	4.679111452	19.2039096
58.21192813	0.078790951	0.031674481	0.339338021	1.492589988	8.97464409
124.2774564	0.269745348	0.171181145	0.904627268	5.177361413	18.78415557
163.0619235	2.608695554	2.010384621	1.875635512	4.851523884	23.42230927
87.86126627	0.12603516	0.080263024	0.396907789	2.379609496	11.53941279
88.36001315	0.074236847	0.046435999	0.275086751	2.73440613	11.49719447
48.14515147	0.085568665	0.028978857	0.330841746	1.233919837	8.163449331
130.4876036	0.255732801	0.143926235	0.681989972	2.907985266	17.78606774
67.26758166	0.161807287	0.145050528	0.52451012	1.246123237	14.05594994
76.71715399	0.194676051	0.143997412	0.493982606	1.23793413	12.199554
172.2958261	0.487023814	0.189916725	0.737589301	6.340184189	17.07547236
189.6945983	1.104357981	0.327144368	1.094864084	5.519717429	22.06362026
85.61921676	0.090546549	0.050594832	0.370953767	2.364811732	13.67071878
90.32474691	0.070288651	0.040590802	0.309030755	3.488706092	11.62540706
95.06380058	0.6223438	0.628437821	1.07498813	3.062090303	18.30090071
112.8640393	0.582654004	0.337174058	0.910927941	3.961710727	14.73222399
74.13095353	0.08084232	0.107054994	0.513690407	1.039179704	10.16788969
83.21415089	0.133531697	0.102793067	0.474973821	1.76852906	10.01371745
121.7892949	0.307835404	0.305429521	0.753588331	3.942825395	15.07211896
97.12332946	0.060989842	0.031344867	0.316450111	3.646245318	9.9473151
86.39907937	0.166853835	0.124440918	0.596394696	1.115744671	12.6498911
69.470386	0.125311968	0.115241369	0.583278295	1.534291012	11.59200795
102.730078	0.490723745	0.169641703	0.958616146	2.794047153	17.23319661
84.09766687	0.134082296	0.105481675	0.416710283	3.368806443	12.57053042
87.73045772	0.079719242	0.135866902	0.407049486	1.721640983	9.221397062
110.0396926	0.150694339	0.056890687	0.439050072	4.424641908	13.19315556
85.05433	0.175312507	0.144607435	0.534252913	1.311513303	9.895626141
108.5913173	0.09671321	0.042170406	0.311508163	7.167495753	18.14406564
130.0421882	0.610468235	0.29545424	0.716589244	3.75617167	12.41085552
100.2628021	0.270895174	0.279019519	0.54105923	3.626615096	10.97098838
138.6874393	0.526879767	0.410158701	1.144760576	1.448248704	12.65882216
140.2834216	0.435186058	0.252156753	0.778712201	4.975337026	18.18396789
36.83805164	0.011902651	0.019915506	0.170763115	0.33726449	4.733204915
79.51743457	0.214673059	0.123210037	0.553860488	1.979207572	12.26152493
69.86289196	0.07399369	0.031474894	0.3730164	2.089265075	10.19727686
99.73539767	0.274048939	0.153898883	0.643934491	4.49151002	19.4739075

127.0518475	0.276051974	0.298418217	0.606057594	2.292228619	14.66343951
133.0545114	0.397787873	0.284845982	0.867209436	4.450761022	17.52436729
70.99289726	0.068171955	0.051952701	0.424818201	2.116578392	9.814836686
142.8485166	0.752813103	0.632802091	1.728545051	3.011169268	14.93270194
111.3953269	0.255788191	0.270529883	0.682809615	2.968111425	16.19904864
115.6142438	0.123217963	0.06937222	0.406215802	4.357883125	13.53339619
222.2184404	0.713363915	0.432596112	1.142649351	7.346102384	24.43334008
81.25332367	0.139175334	0.095218265	0.581514969	2.033850555	12.41523695
194.7925161	0.779713528	0.389260308	0.834782978	3.662065358	16.24124254
113.2779869	0.194222963	0.094331767	0.601357564	2.771714522	14.74402061
109.8563589	0.285382831	0.322740383	0.689708725	3.110619068	14.27066165
115.0930873	0.683255485	0.637661535	1.097315958	2.734958786	13.61784444
172.1149802	0.371804341	0.208968556	0.7481006	5.657771228	19.46423876
77.64273154	0.100395765	0.085098086	0.405604208	3.130202996	11.67409841
61.16738219	0.041543991	0.029334278	0.276713691	2.805584433	6.628850533
127.8692709	0.179631254	0.24514118	0.840288057	2.761463404	16.16927665
163.832927	0.27940118	0.176261476	0.66050853	4.164954166	19.12616697
103.8120644	0.11242931	0.04735047	0.445825077	1.952498164	14.28247572
58.6544166	0.024111872	0.023263273	0.24662008	2.667765485	9.003904283
150.4253848	0.236505017	0.195451394	0.717167437	3.695548835	20.44520003
101.4827735	0.33558481	0.323128087	0.604496796	1.523553954	11.90132267
81.88214728	0.12605997	0.075864795	0.542897683	2.862044637	13.13181481
78.88182352	0.10267522	0.067138228	0.505825763	2.524478145	12.07514112
93.06899609	0.090481722	0.03173848	0.43862179	4.644288625	12.20367403
92.83674414	0.319846038	0.554153784	0.718152353	2.084607016	10.53633052
146.4046817	0.108718682	0.049042939	0.441047401	4.91515281	13.38627524
204.0551103	2.749954019	0.619685283	2.099706939	6.316324358	34.78272584
103.1170692	0.127181263	0.063287347	0.364546911	3.825216283	12.87667537
121.7024925	0.165196904	0.092548148	0.511532206	3.317580629	15.03237495
167.9446186	0.988602863	1.369381257	1.153193136	3.092610255	23.70379595
107.9079138	0.168701396	0.141772326	0.531565814	5.296638257	14.2195262
121.9176356	0.356677267	0.331635781	0.79691882	3.178449261	13.8557416
70.12180107	0.135994201	0.108909103	0.512048809	1.493616033	9.994385017
113.8672123	0.218664407	0.233332386	0.717111094	2.497719423	13.97774675
94.44411058	0.12621472	0.107195582	0.512316822	3.005820249	15.11426243
121.9782274	0.484718696	0.422662045	0.799865067	4.161308288	19.65095191

104.1027572	0.241635255	0.12571977	0.750764124	3.458808432	17.28673412
156.9868906	0.297473461	0.210049429	0.691316522	4.426616907	15.69030227
125.4933014	0.372327547	0.142729419	0.705689186	4.786447901	17.72091219
106.216191	0.316888621	0.219370137	0.573487217	3.143653779	17.31905082
263.8044217	0.752595478	0.186329349	1.279605753	6.711219922	30.07296482
66.02533634	0.141881672	0.065196615	0.398673029	1.035451322	7.977928581
97.49821426	0.275944953	0.266319068	0.819845981	1.519166419	16.02566843
106.1369779	0.16120717	0.070399874	0.436968909	4.217792149	16.5581479
102.9846269	0.223414368	0.153886344	0.751990402	3.682193258	23.78178421
86.78820047	0.111626919	0.150489186	0.49228762	1.359316987	10.94150965
148.3556545	1.180623174	2.012232519	1.616593792	2.865537829	18.03733814
96.67637112	0.143676161	0.038671003	0.493525336	2.011482319	19.88317621
78.36273734	0.109698912	0.074060607	0.511225618	2.821209754	12.11787844
111.9233134	1.074396194	1.024122176	1.231473338	1.966256877	17.00376863
92.10198798	0.073042989	0.095678189	0.415409271	2.806113033	11.5548076
98.32526937	0.314123236	0.111135649	0.70267976	1.562834253	16.35771872
31.5853429	0.00660012	0.003430984	0.106842165	0.350189984	6.95485791
99.82544562	0.55189368	0.12500021	1.083075291	1.873271837	14.26982528
112.5018	0.218976695	0.254428213	0.70780567	2.821595773	12.10207611
66.04308759	0.07269439	0.047774773	0.347600545	1.464542869	9.053264759
62.7431427	0.073995678	0.035166768	0.385663319	2.081302078	10.98029352
82.28912921	0.10331372	0.049428512	0.409395486	1.861810282	11.33229215
101.9741201	0.232366812	0.127075876	0.553514315	4.388685585	18.51766076
96.13743652	0.261380089	0.123122584	0.490304191	3.372156278	14.46968326
85.8713073	0.089225424	0.153533986	0.443143949	1.542329432	11.19915966
108.7361373	0.292111646	0.288646401	0.846177124	2.223575761	14.22022326
100.7061043	0.098508549	0.06828458	0.470650753	2.230434766	8.85737621
136.7122555	0.168879148	0.031893356	0.416255665	3.174194127	16.44917332
76.55391678	0.066533403	0.038472936	0.355397976	2.115575348	12.45121046
63.5400847	0.032368831	0.007536886	0.200614969	2.540274817	11.56808669
83.34336979	0.089885663	0.062721557	0.393190335	2.137577841	12.15610963
62.90758188	0.072834986	0.016760241	0.285141651	0.898011217	12.21325847
127.8256825	0.181970256	0.115391713	0.477209577	3.31614262	16.44678388
123.7101862	0.520749484	0.199630298	0.58794918	1.731425849	22.73439141
125.7366944	0.156114301	0.056014094	0.420284376	5.962612133	19.58671463
96.94118237	0.137003895	0.058691252	0.497757634	2.071774109	10.21532617

65.42782734	0.071816247	0.094860239	0.371383992	1.758877932	9.599902339
78.11772889	0.059159712	0.055782598	0.457669282	1.64076567	13.66006102
112.491803	0.186678294	0.143625785	0.500760753	3.299957192	13.54906006
144.1215582	0.239073368	0.126155138	0.627011272	3.651506359	17.84196326
81.95675054	0.165944141	0.167768325	0.547061825	2.280304432	14.067272
186.290633	1.089602761	0.414719204	1.362501623	3.843746323	16.39165671
115.0567671	0.659935579	1.241413015	1.318994063	2.371455405	14.44450252
91.85136102	0.161172595	0.091860905	0.713650093	0.99931576	10.37520761
74.79424385	0.100954962	0.118325515	0.457381121	1.496200056	10.82511305
104.4519735	0.177776033	0.058893332	0.453322104	2.283724073	18.08480358
80.95446001	0.064171115	0.045283533	0.357436568	2.304919183	11.41757195
121.1011398	0.175874164	0.162444355	0.658073914	3.398159311	11.84523419
88.92375177	0.559949043	0.658323793	1.095395203	1.709727844	9.780155256
95.88347145	0.277401964	0.16632699	0.643266122	4.915466391	19.74711143
60.21152879	0.078698385	0.120340248	0.396474049	1.060052635	7.44221139
102.8556157	0.124755206	0.068705455	0.357557732	3.201737141	12.27631112
112.0631048	0.1136376	0.10695397	0.498114348	2.502550357	13.84459689
91.40192451	0.635721907	0.869109748	1.132787746	1.941830118	12.47003543
94.57113111	0.112456515	0.113138414	0.338122309	1.685264926	10.67857202
80.22225946	0.077554014	0.032992886	0.300888672	1.294890867	10.17094134
106.9233988	0.121795502	0.064773842	0.448746748	3.19027047	11.11053574
173.7902919	1.43537453	1.251753513	1.924685863	3.020922336	18.95014261
63.13441962	0.237194974	0.090891846	0.581373487	1.527254399	13.89341504
92.75237212	0.144931852	0.140033265	0.47498118	3.273652486	11.43805371
176.5402981	0.88639933	0.552950568	1.522850775	3.114999808	16.60962919
90.0443083	0.198817696	0.156418815	0.543228598	2.770687842	17.9503694
97.25784875	0.787924148	0.222563195	0.891404715	1.757198635	17.83193078
42.17964212	0.013216003	0.018103587	0.186737628	0.819117653	5.848024093
52.28360756	0.051689706	0.064108985	0.282078817	1.765475859	8.533699922
87.28338658	0.12940249	0.116834302	0.463599108	1.895425771	10.02180455
81.2753736	0.050860494	0.034955218	0.348606041	5.269355015	10.41469547
91.17467314	0.140171233	0.116943945	0.538941152	2.662652634	14.29545859
108.1178942	0.317545812	0.170132187	0.731062093	3.396617594	13.90543327
77.73261584	0.079062859	0.088149394	0.391776678	1.772648086	9.140465655
113.8619621	0.325051244	0.092679324	0.668876657	2.210722829	16.01472434
77.05171436	0.194145591	0.119863924	0.648952429	1.333274086	9.051951233

138.2860164	0.213753355	0.144372506	0.73968432	6.865423332	21.03744874
158.6594323	0.142647684	0.118811619	0.518441183	5.238902051	16.21569846
79.37968472	0.12027442	0.086471095	0.455690085	2.290895086	16.02072176
130.3064573	0.332167861	0.169681034	0.711156058	2.474630906	15.01590719
50.00779664	0.062676912	0.033601085	0.351191915	1.215171844	8.010854844
87.8493214	0.061353523	0.033987116	0.261943895	3.410867126	12.75324468
111.5606043	0.125539475	0.117896011	0.48155507	6.611853144	14.00148108
129.235658	0.072494872	0.033052744	0.277276797	2.638913418	18.02182266
88.44397044	0.11786184	0.024463055	0.363355349	1.7898108	12.65108898
118.6069953	0.44081616	0.267743528	0.658994492	2.839350789	13.37103159
208.6508274	0.256274801	0.09729106	0.555402175	3.993605074	18.85050796
135.8103698	0.367662055	0.114961985	0.934157098	3.508460367	19.73805833
113.4829764	0.259663813	0.116676413	0.625163014	2.441148421	19.19220873
100.7144161	0.241978833	0.314982113	0.989077849	2.758524621	10.19734091
57.07201463	0.046459951	0.072602859	0.248446495	1.541779167	9.428759361
126.9924931	0.248699282	0.120461016	0.681735372	3.096817548	17.35626759
63.23321189	0.053021482	0.044454117	0.327555471	2.84921373	12.45747663
95.45971941	0.145879155	0.072320847	0.510139954	1.972861363	12.19335207
123.5811471	0.181612466	0.150054617	0.512377084	3.553796313	13.10965874
76.40264394	0.173350487	0.141282408	0.48992925	1.880234071	13.92082773
70.39582489	0.108908407	0.096383962	0.449903334	1.438785296	14.0700526
93.76472428	0.176503692	0.152240082	0.488648308	3.573756278	15.16986568
116.2369105	0.153513697	0.060804278	0.537097722	5.165665188	14.0933924
111.864134	0.217138916	0.258596473	0.807203665	3.295231435	13.24517079
86.59957344	0.121488265	0.089285524	0.419693132	2.738601205	13.14743347
240.8519921	2.167103725	0.971679556	2.184213268	6.488726584	22.27004141
82.6665611	0.237745116	0.145407184	0.722232845	2.098699273	12.11303127
134.3678096	0.228572014	0.127831435	0.586304445	3.930529922	13.55818083
71.33812022	0.078872062	0.067881357	0.387672465	1.856969797	9.739827427
90.62918055	0.135057004	0.06529365	0.471170971	3.208647475	13.01717231
91.67613348	0.129022554	0.078533321	0.596283368	1.090929738	12.4744421
48.18337157	0.04131974	0.029864955	0.291733055	1.126611585	8.365880327
61.37378497	0.065483342	0.06705331	0.304456666	1.981928669	11.79094234
75.56242772	0.151002039	0.146965126	0.499200048	2.124892394	11.53256267
68.45833588	0.042742914	0.029233829	0.291647969	2.222023716	8.647322645
110.0901282	0.392681636	0.139024649	0.53199308	2.254888585	19.04826983

93.53091856	0.087781964	0.069370702	0.339475136	2.575129747	14.22806191
150.1528368	0.584236895	0.990199119	1.080797124	3.318491487	16.26186644
83.5301654	0.323054339	0.338295028	0.958465166	3.26573874	17.69521761
113.7800879	0.485557551	0.322247069	1.039825641	2.350950885	16.90870768
117.5214215	0.161941954	0.119537062	0.622027777	3.449405353	16.44680425
106.9499339	0.753520514	0.540130627	1.072432327	3.457604075	16.67987773
100.9146944	0.102654218	0.043505645	0.397893495	3.085466097	13.69998925
102.4740131	0.173460583	0.154888535	0.46884359	4.367626689	16.29583788
94.88011079	0.189615079	0.06737599	0.439341312	2.383231367	13.85773394
204.5662778	1.468036792	0.450313011	2.287225657	2.431471259	18.7658225
64.25861356	0.124129785	0.11445857	0.507016183	2.422387077	10.63767844
132.440474	0.457244474	0.389208317	0.900172359	5.337367253	21.72516204
44.37568475	0.043207083	0.078010504	0.292857896	0.562853973	6.043171649
157.8145752	1.156790598	1.573790166	1.672936855	3.516754249	19.15627821
89.41421628	0.29615557	0.1026388	0.659962204	1.673368722	15.45865228
68.50756679	0.023300055	0.011974731	0.249861867	3.227049215	7.935144023
101.9711562	0.100748927	0.069050278	0.356573923	2.055546761	11.31170202
81.69033708	0.166784657	0.083226018	0.565145096	1.641248016	13.39270769
68.80475894	0.140654436	0.166294505	0.538380477	2.032321314	11.26017566
161.9818231	0.281078535	0.153877691	0.73673131	2.861950734	16.29875415
135.9409015	0.212434198	0.112878796	0.674107498	5.161916743	18.17687742
89.83054142	0.229669528	0.168279545	0.587241442	2.62195142	12.98318492
102.066679	0.027963083	0.017580109	0.229247309	5.108916311	12.44710729
118.6040556	0.11624394	0.06016534	0.507425465	3.048854735	16.27343212
69.83220673	0.042665052	0.012111475	0.267207155	1.881198355	11.68628061
153.5771263	0.239400105	0.12151994	0.855594607	3.333905094	21.11755836
75.35892555	0.159754299	0.14526292	0.560235634	2.550113217	12.33211285
135.573091	0.358348534	0.240676774	0.89709116	3.274263587	16.09460123
72.91308344	0.148926477	0.09736099	0.553971743	2.671052847	12.68147292
81.95036692	0.238328063	0.175434365	0.584664704	2.269761907	10.48362298
171.1148812	0.224631264	0.147217912	0.668096346	4.775070168	17.10325659
97.66926462	0.206168039	0.11550337	0.543513438	3.417073817	12.3485502
148.8910977	0.490685638	0.225972411	0.949019534	4.35810472	18.73931192
75.21135183	0.174599877	0.210113379	0.486210844	1.623515701	8.035834689
36.17433896	0.014847682	0.007823638	0.180884809	0.399445477	5.692568664
93.20350134	0.127589294	0.110627189	0.429730591	3.490295033	15.89034145

108.1918807	0.341068337	0.242608133	0.683879616	2.837284229	12.36222694
79.87298273	0.118492577	0.083173499	0.452617944	2.706465256	11.83911836
146.6083754	0.213278625	0.176032205	0.588438005	4.608601503	18.45461248
103.5622258	0.45136861	0.224843428	0.962612136	2.509314748	15.58997201
68.62655611	0.1018708	0.150884933	0.355493206	1.673108916	10.64110684
59.7126156	0.04579594	0.046321599	0.387719292	1.5217303	8.665552256
189.192471	0.374710892	0.201001043	0.624373406	4.79467568	15.40503976
101.2561086	0.209260503	0.170414979	0.662652816	2.631181765	14.58630466
124.3808989	0.569347433	0.947513284	1.16454928	3.312596974	12.28237696
69.41071962	0.064380552	0.089440031	0.382112966	2.161523698	11.32203089
112.399187	0.140188861	0.083563816	0.471739982	1.441644962	11.72695226
55.72016765	0.044411775	0.07065636	0.275214047	1.952562652	7.858733566
95.42502946	0.198158328	0.116442112	0.570970119	1.867641162	14.03738563
134.640885	0.384202436	0.415271661	0.768725355	3.120723888	15.02420922
72.48788543	0.068214835	0.036105046	0.382605809	1.640243714	8.988343617
41.88647208	0.024017736	0.009353856	0.161118561	0.855724602	7.352699895
100.843448	0.12982234	0.064845764	0.416642343	3.11382763	12.71787688
90.9671092	0.155273753	0.191880584	0.526252468	2.137145746	11.09911844
167.180848	0.899544492	0.546562392	1.511894839	2.781561712	19.72433448
118.6015479	0.321545811	0.172716848	0.621738259	2.322411052	14.09593301
74.56181609	0.104203052	0.065403775	0.333074037	1.643754684	10.38850133
102.7834709	0.087789839	0.028318802	0.310340261	2.191664876	14.06336246
105.5144048	0.102339262	0.101280517	0.524945513	5.356984153	13.97095809
94.61408301	0.190433121	0.11815982	0.501844462	1.770678112	14.4985215
160.5377955	0.381419453	0.168767072	0.856623764	3.361487839	18.26174376
65.11149374	0.082209325	0.040674528	0.453887122	1.493212629	8.763779332
93.11420222	0.144491458	0.120843509	0.48013784	2.572395791	13.90102381
117.8571952	0.232598149	0.183201082	0.807783223	3.340938468	18.71741064
55.89998663	0.122749715	0.109093264	0.630093946	0.822207677	7.261289544
68.69144882	0.099057554	0.093967269	0.400646087	1.789109982	12.09378326
107.6206676	0.180546134	0.085285161	0.55498115	1.965611928	13.35975268
146.89252	0.543174253	0.134122462	0.711203231	3.772322116	19.07752607
75.88906249	0.105087266	0.081682255	0.473420801	0.711663245	10.60848054
111.25679	0.155491777	0.154302857	0.583573709	4.495311968	17.14391978
92.32586137	0.149652091	0.142774659	0.524057735	2.127343566	11.78136774
129.2438611	0.229786483	0.091839754	0.644751863	5.659036461	14.69690201

53.37453865	0.05447921	0.052185467	0.353342716	1.903844505	9.646454861
92.74243365	0.287695794	0.327015731	0.985615289	2.948771999	16.46085831
66.21950824	0.13381598	0.082876532	0.584152267	1.849406984	13.03542198
101.1260074	0.179775998	0.136825247	0.575977204	3.299323515	16.00210531
79.36228308	0.202947111	0.077256986	0.63464475	2.029190967	12.07772194
140.0955036	0.261938196	0.077546746	0.419946654	4.468135495	15.43086462
109.915314	0.782717428	0.377503984	1.175636971	2.038650679	17.88641133
128.7189491	0.290035096	0.127413406	0.803847721	7.566561521	23.70830913
120.2488542	0.19712716	0.084946905	0.530976408	2.697741527	15.28092531
70.18918512	0.09153314	0.075164544	0.389529295	1.028870178	10.56272455
103.6395201	0.376423686	0.145800217	0.911216448	1.494171632	11.311484
84.19939875	0.062633036	0.046395169	0.330640708	1.094641696	14.62275733
117.8943546	0.276045545	0.165370274	0.673363511	2.77973813	15.51134154
92.19316687	0.253966779	0.172667046	0.721784042	3.504986216	14.08780236
67.47176349	0.132238141	0.125822138	0.480316068	1.389152745	10.33953337
98.10401203	0.107002904	0.06841391	0.534058576	3.109539193	15.92555805
164.3701345	0.223754994	0.149947928	0.623543113	6.809962688	16.68231678
70.66602589	0.180706418	0.084412082	0.652482628	2.257661504	11.67910282
70.37088117	0.063115795	0.047155869	0.305324398	2.669467946	10.67444075
46.30305935	0.002859707	0.001555916	0.071134971	1.955275316	5.174024195
67.98128065	0.215469143	0.148144254	0.549546925	1.187429189	11.25802663
145.2388513	0.467467802	0.206384763	0.9950221	2.664743473	15.74553662
66.93260237	0.131535904	0.087140825	0.415901257	1.014022442	9.017359478
132.6901708	0.276061942	0.189824526	0.789816918	3.523338936	16.36439259
93.37459405	0.144759781	0.04155915	0.440186643	2.82649215	18.40694225
60.20852931	0.026519374	0.015666053	0.205488482	4.089256923	9.477632274
129.4201893	0.413287813	0.221304761	0.865700043	2.649633856	19.22526294
80.16844318	0.139554665	0.073202482	0.489673246	2.290496487	11.54577977
74.15152018	0.076578673	0.0901019	0.351894235	1.968273224	10.48548482
67.43584584	0.031773334	0.015646494	0.226314886	2.062382561	10.8084691
90.19300571	0.519744678	0.289405938	0.82995757	1.891839023	17.82889861
129.2008412	0.423171093	0.281395611	0.851093635	4.996344348	17.84210255
87.91063727	0.221024757	0.158276169	0.743706696	2.732963787	13.33365796
152.2052455	0.350733235	0.091705672	0.68246661	2.91752519	19.65569303
140.1456891	0.292535178	0.298290747	0.703511041	4.043854488	14.09504234
79.124116	0.151122397	0.128815293	0.58652393	2.010600212	10.18976497

135.3814203	0.432380147	0.247551958	0.836893383	4.678157098	17.57078081
59.77089394	0.051792474	0.060873818	0.292906202	2.006551357	9.397957241
164.5695806	0.377465029	0.195496992	0.920518694	3.264280267	17.43162564
105.597129	0.303830534	0.139146428	0.734291998	2.811930059	16.93968199
139.0770491	0.227281157	0.120460263	0.683607229	4.419591767	17.71565103
110.093074	0.226707047	0.159100081	0.687226198	3.812824292	14.82230858
99.71180358	0.393226131	0.301767855	0.821590652	2.626913784	16.87023684
113.0881499	0.277772891	0.075598773	0.73448524	2.710174091	20.37661078
78.20284515	0.149924523	0.079364933	0.560027159	1.995623534	12.91023985
195.9079228	1.173281625	0.532044147	1.890543637	5.105377732	28.34149762
73.75904311	0.102392845	0.057589506	0.400228483	1.674882072	13.64972709
144.8447042	0.307249287	0.226261759	0.658180237	3.213918752	16.40004339
119.1359565	0.208694432	0.104151436	0.573999905	5.021965206	14.75651464
169.1050932	0.767726676	0.331823696	0.914536435	4.176390463	17.87560504
133.7888201	0.187401691	0.142846654	0.617006551	3.141992032	12.15907197
113.2401321	0.233515138	0.092687783	0.582923379	3.136676366	12.7336651
92.95428009	0.143529546	0.06114502	0.446420928	2.186683972	10.76549944
85.35405457	0.129020002	0.118728972	0.44585482	3.422113382	14.14096375
113.2309846	0.297649244	0.171682699	0.710718836	3.153739441	12.11152129
86.68720623	0.669750543	0.369511004	1.041123202	1.946813515	16.48471748
75.14474997	0.07319592	0.053321118	0.347593089	2.842512777	12.57472309
88.81665748	0.140659583	0.135038657	0.51178011	3.064522907	10.91928707
107.7285694	0.054491556	0.032338933	0.26675882	2.17435512	16.73182078
99.84434265	0.066943524	0.054281766	0.379623945	4.29930721	14.87196841
107.3834623	0.199798607	0.118994836	0.502801221	2.811043876	13.13633608
102.3930021	0.222605796	0.134038796	0.809895195	1.764696445	11.83726938
62.64385016	0.049108268	0.052166844	0.279296293	2.674432944	10.99458008
56.82118345	0.074687024	0.054524013	0.316443743	1.943919237	11.68551696
84.18703482	0.064084301	0.046584003	0.35244016	2.426754057	9.225020762
107.6333879	0.335299575	0.327366984	0.833518652	2.286667931	15.93808037
92.47728066	0.202747547	0.283692241	0.846547195	2.095846597	13.04038676
73.42863324	0.039626849	0.019761043	0.20620999	0.942337843	10.92255437
93.95310969	0.284996505	0.224764475	0.592424612	2.973462177	13.82817492
162.5348193	0.511698383	0.216254236	0.989605096	3.806766663	17.78730759
101.0551316	0.112932052	0.062226605	0.460894292	4.421206021	16.38522218
162.21027	0.663921804	0.221265603	1.030103749	4.139912809	18.39488964

121.4071491	0.143011438	0.054034846	0.441045349	3.518580842	18.30211865
57.18485162	0.077500453	0.065730609	0.405821627	1.868080554	10.67570249
111.7632539	0.206919518	0.139815398	0.562820548	3.295496447	18.51872266
50.96834425	0.074375561	0.035066199	0.378652916	1.559409682	9.812447875
128.7438995	0.12467836	0.101178868	0.478971568	5.70290357	18.7072359
147.3403115	0.431920978	0.266409721	1.020713469	3.229245902	23.50650237
74.9635962	0.29763316	0.107677057	0.478935082	2.428788128	17.51626519
62.19841782	0.092018544	0.112197721	0.378958808	0.749671663	7.889894576
82.88710124	0.222722053	0.207936686	0.662097828	1.254042689	14.9809459
65.31149061	0.039218951	0.017088893	0.242496905	1.195296763	9.856822648
68.4816557	0.214065064	0.176482833	0.609417389	1.334995926	13.09783484
141.9560713	0.723508061	0.159691433	0.71230342	2.242706121	22.32974228
86.04152544	0.33673768	0.282913213	0.628264501	1.157205381	13.66622067
60.34086329	0.092105911	0.022609793	0.390946197	1.29213665	11.00767252
53.95120819	0.048918831	0.007277577	0.209902894	1.794219535	11.24219536
56.30115484	0.030684673	0.007997606	0.300217393	1.125603982	9.811908826
101.424783	0.14165848	0.107575485	0.441899903	2.917672673	13.23952913
82.99694582	0.149728901	0.042279062	0.421579563	1.480055405	16.0623371
72.68844393	0.058765439	0.018068287	0.276363847	1.951244262	11.0112219
114.4602176	0.52358088	0.376677466	1.058370607	2.171978767	22.0159375
60.83859556	0.075036792	0.044447019	0.282368881	2.389756673	11.17010585
65.15033179	0.040296577	0.016100499	0.199251007	1.860967466	11.13433477
78.12548095	0.23625429	0.14051313	0.537419961	1.801310444	16.69997245
69.76518873	0.08405317	0.071011864	0.351217277	2.131849665	9.637949796
66.20466475	0.186818499	0.306393827	0.573347094	1.379856475	11.76311285
103.1019916	0.107775877	0.031448029	0.347269573	3.43308146	13.93357987
88.48227941	0.772691403	0.269684893	1.207334796	2.488776906	17.78004086
69.70507592	0.085846756	0.018893755	0.337056754	1.72068751	13.11304232
72.14439464	0.110490112	0.055621554	0.452509963	2.195643063	17.82332612
65.80482814	0.332610359	0.16615978	0.445761367	3.451800632	14.39892834
138.8480456	0.214481319	0.115193853	0.519919682	3.537919055	20.1395425
47.02350094	0.1407422	0.054932739	0.455084793	0.367315758	8.327640711
68.65917014	0.044911101	0.023936119	0.282413449	1.085083853	8.526746855
71.0368168	0.086954606	0.064327212	0.306444007	2.427048506	14.67602674
63.31648321	0.284796963	0.081136822	0.621056774	1.083610129	14.81606683
69.89847379	0.063341476	0.019256455	0.324055138	1.682810229	13.52698355

92.70242658	0.094383808	0.031230025	0.396961923	1.728112052	9.88980447
51.74789027	0.028219844	0.017241333	0.185671685	1.365650385	9.211173591
71.66340116	0.082541214	0.104649568	0.353533488	1.212576103	8.143220765
54.18185959	0.046136893	0.044287017	0.341189557	1.048160566	11.38405437
71.35970793	0.080311389	0.037572558	0.423387489	1.529408679	9.136362406
136.5590201	0.791510175	0.517849159	0.943288084	2.692637955	14.74777358
86.39806076	0.071453469	0.02350943	0.292900407	1.39034899	13.19561621
71.36949153	0.126300989	0.039666005	0.430326295	1.146280351	14.01368051
74.91292582	0.068435382	0.041655667	0.290795619	2.424804309	11.54211868
101.3822543	0.535911596	0.437057876	0.762342944	1.962418946	14.29412978
60.80139257	0.107773766	0.034763797	0.471753998	2.388977566	13.91921637
49.29546513	0.026181411	0.003698707	0.183975707	1.236776791	11.16075741
45.18053381	0.073368577	0.013754808	0.264627181	1.658077047	9.996169915
60.85462149	0.1363562	0.16330698	0.477796987	1.122484837	9.68423991
61.65916246	0.074490905	0.035733382	0.291763495	1.776973439	12.38155188
59.28085665	0.058238333	0.026429912	0.298008285	2.027741819	13.47524054
80.11034176	0.098121444	0.06633018	0.461998033	1.473822324	12.16744522
94.99115775	0.520506288	0.068380698	0.556821039	3.075459367	23.03123891
80.4835443	0.138966011	0.178601246	0.461163612	1.456829603	9.974536841
36.77719831	0.020212568	0.017010654	0.21084197	0.695298418	7.40441268
47.76136407	0.041224471	0.010798437	0.231770503	1.275511197	10.73786593
82.29584296	0.066861704	0.023032066	0.294512953	1.432770866	10.87442605
88.29699455	0.141251562	0.016315043	0.304592694	2.658249811	16.87891925
50.91783711	0.051023443	0.029923035	0.357858743	0.633364674	6.282121792
71.34133781	0.139913819	0.06770344	0.477230141	1.536703094	11.96236505
111.5090517	0.179512492	0.181330181	0.46002104	2.782001827	14.73776194
124.2411071	0.6478118	0.245689592	1.123421334	2.877942821	19.36249081
57.66678446	0.057300075	0.044775449	0.305160018	2.049605067	10.14812996
47.62540075	0.019713338	0.009560463	0.185852163	1.365328087	8.265789061
81.925327	0.070907755	0.021110475	0.318609464	1.473524216	12.93343033
88.48981118	0.298901589	0.09160236	0.63473976	1.342431752	17.77176331
36.52653606	0.053509771	0.023658504	0.247895356	1.380608705	8.904517622
58.60621136	0.027088336	0.013136306	0.189119763	2.094700988	9.116896015
97.88437852	0.54436428	0.23375482	0.967453642	1.807882658	19.45473745
52.31300659	0.062473	0.026892778	0.263018381	1.50909602	9.713444619
57.71843606	0.0672238	0.030149843	0.285613945	1.609364892	11.35515876

87.20088794	0.163026194	0.102034179	0.601943581	1.921604336	11.66027328
37.97659573	0.016124365	0.005745297	0.139106808	1.090195422	9.151622796
83.4314628	0.222691683	0.05294935	0.746671295	2.132283637	14.83845042
92.90281145	0.252749119	0.197277589	0.671792918	2.578266519	15.98069391
85.82355628	0.228062545	0.129258904	0.454363229	1.901315234	14.1372192
112.7336775	0.232672521	0.137594139	0.561495687	2.542017229	14.42534116
74.94435469	0.186320202	0.07668934	0.340593645	1.679289704	11.6430124
54.37763277	0.068537888	0.049962437	0.287769002	1.66769576	9.117532221
92.58345349	0.120824677	0.040050547	0.400430246	2.2774171	16.12594222
98.30084626	0.353022788	0.309967295	0.761486185	1.549277862	13.9017044
76.07969967	0.212612482	0.099681937	0.544745941	1.826437998	13.09939256
58.2989627	0.038704364	0.00988229	0.23964069	1.196135859	10.95372031
37.21404643	0.046117684	0.017478586	0.244602042	0.650276429	12.72238874
118.5624315	0.071556176	0.019799165	0.333351712	4.151765093	18.44474876
28.71155333	0.023056079	0.016780974	0.210132102	0.642974212	4.445080807
66.502436	0.033470289	0.02093636	0.219699676	1.55215185	10.09971226
49.71411534	0.084453563	0.021668459	0.287054151	1.651541558	14.84601557
70.78390418	0.098354064	0.0192812	0.35308389	2.078982357	13.79096392
106.1706495	0.321583726	0.157639777	0.810545582	2.825631366	15.84284775
62.32543219	0.10948179	0.077445163	0.386319065	1.304313369	11.28228969
80.16599703	0.199795217	0.07249579	0.646330758	1.790214783	15.22712755
83.41777288	0.29118547	0.176115689	0.496028601	1.663120714	16.52374217
100.7130062	0.071710316	0.029682978	0.352436809	2.11956418	22.01206949
72.21030067	0.053960801	0.010698744	0.231813122	1.60545844	11.78962128
97.29607378	0.158204773	0.017527987	0.367439601	2.030722345	14.93000851
88.05787093	0.125783219	0.057987933	0.454202244	1.3043443	13.30381562
114.0991598	0.17259285	0.059520842	0.551440447	1.726876304	20.15650046
118.1168199	0.846064905	0.223862192	0.844227158	3.128337268	23.63347434
111.4261914	0.171142978	0.08951772	0.595550885	1.956507086	14.67696294
97.31189006	0.302816375	0.144413254	0.711711052	2.651471452	15.74465158
84.15347658	0.12306329	0.014773273	0.370568944	2.392540911	22.29829422
79.77890061	0.092729664	0.029025383	0.36062612	2.495629045	10.95117406
45.75801009	0.043534758	0.024219004	0.285663799	0.931666712	7.896886409
112.4184778	0.148418402	0.115514414	0.511635464	2.364377912	17.07672363

Elephantin_1835	Sinularin_1838	Sabutoclax_1849	LY2109761_1852	OF-1_1853	MN-64_1854	KRAS (G12C) Inhibitor-12_1855
57.34680271	33.79045537	0.858825438	148.5301882	43.8053116	112.33171	47.62212919
27.57325915	37.60654877	1.140169594	157.5197198	72.5978357	112.2760777	122.8533866
67.48674619	65.98729132	0.864923645	291.1960095	87.0344364	170.2527635	119.9479626
37.55254376	46.88344516	0.402195805	167.9478452	80.9271887	100.7831067	69.24090441
42.30538931	77.94646479	1.681467254	323.1823914	167.742089	310.5101375	478.0827916
18.5906502	33.25509544	0.52070425	92.43379205	57.1795967	59.20960022	72.08015149
26.97532551	39.3698948	0.799243249	123.7305857	61.0270808	136.4791137	72.47006337
42.65663284	45.81859576	0.942436491	235.5375053	58.8256388	165.2452804	169.5620279
46.13933677	72.89218135	1.150656404	381.6320498	146.878304	251.9856848	357.59117
18.9043273	21.82711353	0.536023903	82.57886923	41.4131345	66.47709706	47.4075116
26.20102265	23.06978011	0.596273665	108.9601297	28.9832165	83.20852155	71.8866506
75.98814253	50.7177214	0.627524769	165.3136428	56.418724	104.1688736	93.40437941
36.15927874	26.84852457	0.547679573	214.6756544	71.6659103	98.92035618	53.88895079
59.84886247	48.0400419	0.561500043	131.3434735	45.2545979	110.2470587	85.1365453
28.06010971	39.18231704	0.772161479	189.7154221	91.4389346	113.4078134	104.5677786
38.52578006	39.29774193	0.726030154	123.2756445	87.0945155	95.20625543	116.6625077
16.41037886	19.5909977	0.408165908	105.747014	30.7043929	53.18891941	30.48709514
64.5311843	59.5702036	1.562856227	257.6503304	68.2393031	281.4526033	72.154056
42.66217935	66.54945265	0.960800347	193.6542087	150.273371	146.9010481	212.5140473
18.50334359	29.86427997	0.487225643	110.2959738	75.5349172	74.53685599	58.30501149
31.53294815	27.32598512	0.810191289	194.3692027	61.0664276	128.1042922	68.58676694
31.42546721	35.51906434	0.747123384	273.8013687	55.6276424	167.762636	154.84762
17.07025808	31.43998267	0.394998529	189.551666	71.2193705	120.4855217	57.22603275
33.73797241	63.2737497	0.841198369	367.063792	111.102177	198.0766688	141.7206877
25.713957	38.20675835	0.53608295	157.7445003	82.2028289	97.13145894	74.65963159
26.7533293	40.17355794	0.693870537	216.332661	60.2366956	141.1790992	103.04002
54.99392847	46.83668526	1.011884696	242.9654913	87.243879	128.6411307	110.3746075
33.81310142	44.39110917	1.099826321	312.3951849	110.14541	181.5952836	172.8564068
22.12393488	38.932013	0.573313017	222.7128059	96.0850561	112.2209891	80.52586015
29.60015304	20.9627421	0.647223457	200.8180775	60.3019149	68.46906955	62.52598343
36.03587634	21.90807431	0.831228502	143.4077337	40.1343964	97.93145284	76.30847297
52.63316718	44.60857526	1.06560565	248.537032	103.136485	124.9429275	148.5897678
55.12546413	49.03581919	1.486130237	444.2169323	109.669838	182.1467971	199.2327848
47.4977869	38.12463904	0.626295707	140.8261017	26.6826523	100.113184	56.23050727

25.96701899	36.08908394	0.973922232	279.5015042	78.3517523	123.4637613	85.2536994
28.8210001	46.98647859	0.666458989	219.1094588	96.8982134	191.6252303	118.5360676
24.8149013	41.50247939	0.680188789	128.9145867	83.7148944	63.27859918	78.81028723
110.0704104	65.04525919	0.83243779	462.3513283	117.178786	181.7029551	191.7862275
37.21697059	39.37842425	0.895602999	199.863748	80.5609936	147.1940884	105.3137323
17.13084176	20.12872494	0.402241732	159.2014614	53.38713	71.32029811	37.49566767
65.47423303	72.98599317	0.290982849	288.7693906	49.3278889	169.0360733	91.25325357
32.17047664	53.2218648	0.881869105	164.8840898	95.7224363	120.3406331	181.7924008
32.4550616	41.78604256	0.659645189	193.7206415	52.5148157	124.6054433	57.23531859
28.87465968	24.90717138	0.72058176	153.1531281	58.0488315	90.27776498	53.78997197
20.47840781	38.00046975	0.56240602	205.066668	33.9450492	121.0597175	58.58704521
14.00136451	12.28744862	0.410457836	87.06467048	30.4504134	47.75578658	30.32536797
20.73552053	33.10525923	0.462428129	130.6592591	67.1035572	105.5398312	52.86124712
36.76328045	44.70649582	0.455918866	248.068774	67.2367679	119.4625467	101.1421696
18.10646827	35.64431223	0.285596504	120.0309937	52.5833038	86.86201576	76.73684292
27.65558416	27.86093786	0.702579479	267.0794582	96.8803865	109.6708985	107.4197998
12.5992964	22.51648785	0.269533151	82.96030237	36.6031133	63.23234205	30.94097095
36.28628542	45.606684	0.809326501	177.8057384	109.52916	104.5670706	158.9154113
34.45935769	37.06615181	0.724577479	149.9392254	84.5897578	102.8248324	102.4491332
25.85224416	30.43562566	0.788773331	185.989992	89.3609942	107.6399808	62.23179019
31.7911912	50.72064869	0.255409936	255.3330109	40.0905013	127.2581608	34.47341568
35.98654481	63.7879781	0.631548822	445.1454735	90.7016364	186.1430026	103.99503
43.70266231	24.31527625	0.527767509	159.127829	59.3645449	91.82696794	94.83058264
27.99839471	30.54520051	0.409823093	112.3476923	78.886361	82.25693355	55.12509675
54.63802975	51.87341145	0.657448909	301.2798184	49.8155111	187.7381921	75.10092538
28.74957016	38.30172649	0.657037213	352.8974897	98.6632219	162.156524	77.89345564
80.54075738	53.62688285	1.298890438	184.9313867	80.8548994	142.0220789	207.3188705
42.43816832	24.95874312	0.710872315	157.9279307	52.3731452	87.08007215	66.99956316
37.98069745	43.00513431	1.398324701	367.4075379	110.207646	163.7497588	171.9534561
31.14629025	37.31935362	0.675198709	193.0388544	39.491356	132.9694221	57.53446335
18.65733313	30.41409258	0.507026441	132.6817359	61.4037689	102.9464106	73.20627386
27.16038996	34.70137407	0.40171737	121.0648963	56.166573	82.13029102	72.57783812
62.32571882	38.31432525	1.256841381	142.4484695	80.2988981	102.2670448	121.9900963
72.74915495	138.7085916	1.137292497	561.0884636	148.342885	459.8789077	251.426036
51.67998701	54.23335741	1.369619289	261.8958809	128.457815	143.0964094	175.6431116
43.43036325	21.05298422	0.649277881	110.211795	53.6562964	66.22706487	94.33219809

34.92029157	57.03732461	1.033104477	389.7371378	133.255647	199.100758	146.4053463
25.96335407	22.43982612	0.474192979	125.3057615	48.1833915	60.73602284	51.93382722
42.12511212	50.39343173	1.080877242	395.7372619	122.315349	126.9646115	136.1673513
84.47789833	70.99967534	0.856403324	442.7453433	141.334411	239.6474192	240.7953038
23.1567178	28.95998084	0.651510139	127.424007	59.1116309	84.16939052	88.50948962
15.48499802	31.07435727	0.340869691	129.3887118	55.9531308	93.90093856	77.04678615
22.83564859	23.30015632	0.358832407	106.8359458	31.1300016	63.45826713	42.99955835
24.82424172	44.69612668	0.655034074	200.9033523	57.5112736	121.0636153	80.85041919
21.94522858	24.08097203	0.716911527	242.9651133	50.4775278	106.9260786	86.08764626
30.0749134	25.36977613	0.919519154	128.3550332	36.9375828	87.36159377	59.85221229
58.96271886	102.4736625	0.659801181	353.649595	79.1083587	216.6187153	134.5380623
70.82900918	51.97440008	1.161133692	374.0285024	141.433346	141.2016863	264.1804834
15.08867995	29.65687731	0.378501326	139.946711	65.2685118	84.42843358	36.88241432
20.93249403	44.75391789	0.283926366	127.3245145	58.3487089	105.8694096	46.41484294
46.15309434	40.02501137	0.473547765	253.7048737	74.9995433	102.9861056	99.12461824
30.04473076	44.03977459	0.575388937	200.8385422	114.015034	115.9753462	126.5939074
27.89921755	34.43099402	0.516958899	98.42351188	40.1468674	94.19545656	59.04348027
29.51932833	23.00899436	0.715145313	117.3385864	54.3187126	90.91112682	58.05179141
52.18558162	37.23817266	0.999713825	238.0942132	88.034149	149.8524851	128.515179
24.86305909	33.18586651	0.740101039	150.3938469	111.398549	101.9247877	90.74886829
39.35746762	35.81095295	0.974542273	167.7193483	38.6562345	176.2917156	113.5631924
17.88405237	22.23322692	0.480534102	150.1600855	49.7444364	79.52794611	49.56256971
29.1777708	50.24203337	0.451800603	215.9714414	52.6631591	120.2530879	57.23563491
28.04221159	24.17948542	0.833563214	134.647144	79.2782089	97.96319431	87.71738203
14.8657807	17.95849061	0.758782399	133.289276	59.5757418	96.91112824	56.20936135
32.87320379	56.24321223	0.41733469	174.5787481	53.7949775	110.9471353	55.95428638
18.34759979	25.3558803	0.480121598	154.9246142	42.9340055	97.3452961	72.96952887
25.14556202	52.51087167	0.628353937	260.9301889	151.203032	183.9923694	122.9536091
40.46679611	36.46490148	0.674532771	145.7569962	64.200995	82.26471106	106.9594366
49.99178237	39.83438105	0.477496057	139.4006161	43.70753	80.999051	63.1081312
31.51892779	29.65888612	1.478048344	219.6787767	67.8669156	136.9059416	94.53633802
40.51027895	44.22020452	1.074407234	247.6315326	120.977359	133.4872254	141.8814745
12.02761294	12.8401155	0.283371293	72.26671622	15.4817138	35.15707271	21.8372694
19.7624655	24.53073753	0.590466944	118.9443115	53.2686201	107.0997833	56.67204298
37.02951786	25.55037605	0.671653629	145.7735541	69.937272	106.3363536	67.43095396
39.01828418	48.30862533	0.894173903	249.6431201	121.400809	172.5667052	121.4956199

28.61846668	34.97651423	0.540917707	185.2694118	60.3766803	116.426837	84.95284215
32.72672117	53.58107709	0.806342006	198.1620419	105.414888	141.5667106	101.089218
33.29626382	27.98989649	0.569635632	104.0267784	34.2910277	63.4632424	67.67485673
51.11380288	34.54593741	1.37951719	215.2753643	67.4514927	147.6690081	166.0279763
41.5040822	36.08892324	0.957061571	122.4456599	64.932353	99.58644528	90.0147687
32.35560153	41.64747257	0.843124868	144.1291717	87.127486	91.71554443	70.22931571
70.09423292	73.52163931	1.626699235	254.2815212	182.263243	221.8052002	224.5807119
22.09785217	26.41621376	0.75553378	147.4021893	52.404469	101.9401794	81.40659234
47.34336676	56.17091761	0.919773622	268.8220145	71.8105407	182.6102793	133.8942644
33.09232404	38.0739665	0.741022518	157.9424273	67.5974195	108.7101923	144.6374516
22.37596077	28.67886288	0.391793893	137.4044567	38.420621	88.81389535	37.14498187
41.89740935	52.92839889	0.692906941	272.1064707	55.1701615	183.2632871	79.04569205
35.35417882	63.00503327	0.729133484	280.8274197	171.357957	201.8420142	289.2627035
17.3008556	29.91859546	0.493007675	99.31288311	65.8670057	87.47187376	75.24111572
14.69257281	14.8393723	0.400981008	95.13037223	76.7788423	69.03740447	42.98530991
42.29715456	41.46199834	1.881101156	122.5433233	62.4217411	154.8334828	141.5592321
23.70549414	41.88416293	0.908277674	250.7290405	106.149537	107.9914836	122.1854756
79.65298254	48.61064539	1.250483791	153.9309418	47.5679386	103.8963763	93.84455668
20.84486494	18.34370779	0.675589315	132.687012	59.4416828	101.6051522	66.92377874
51.59967542	34.72867078	1.15719747	212.3274379	80.3808138	156.1737179	149.9250217
80.89968178	50.28834078	0.856338859	226.720159	49.9695429	143.3441928	131.0337341
21.27240585	30.99769002	0.523458811	212.5298493	63.9576985	107.8319654	53.02946199
24.33976984	25.0814883	0.47887496	123.2716573	44.7317056	91.88529923	51.62834514
25.63177521	28.38807179	1.514235376	170.2920431	140.201729	114.4216013	109.3533597
24.50172048	28.39949869	0.419346547	102.7761309	42.9112218	63.2812756	55.99938377
35.22299361	50.12675244	1.014301347	188.0112932	140.888149	107.281951	103.4678734
55.47445745	71.94909467	1.142492707	262.8341567	110.088433	252.152244	249.0867635
25.06124981	53.41763551	0.391403686	215.6401225	65.4292929	109.7493005	68.98762249
24.72516769	33.40066053	0.552613874	151.0153524	52.5431882	105.7805925	76.3766292
95.75372018	61.21327506	0.732937083	135.8881743	54.2599581	109.9535752	138.9889056
35.82266783	44.13876929	1.038802564	224.2474467	118.407993	169.6477042	109.0188256
28.2184787	28.46163384	0.977849766	197.1549266	104.573935	121.5416866	108.4387364
24.6482647	29.61626279	0.460117665	102.8101572	43.5714194	93.6055158	71.502244
44.57849304	37.90463707	1.415938146	95.47779135	50.6901602	122.1391531	75.74171976
23.01576413	32.02114034	0.692248098	201.1883683	96.2804625	111.9963253	108.9105179
40.3595141	45.95396316	0.903001197	226.0948273	81.1834618	114.3154056	136.7712431

30.23658943	46.85893845	0.578595926	267.0624968	120.925085	179.2943724	132.2584005
26.39366274	39.88050112	0.861683969	264.066965	95.2507561	119.6148338	186.8521566
62.46393997	37.30414305	1.20683071	219.7840233	105.602396	106.0838149	63.18410696
23.3628289	40.07778415	0.560307299	199.5960614	84.4911211	154.5515618	87.45819147
200.6983335	79.3649715	3.035992498	661.2325688	239.67923	436.8158574	326.9380877
26.35684827	30.65676522	0.38660705	202.9571231	35.3369464	91.36197559	41.0461644
40.49663002	43.75947649	1.101016235	202.6553221	55.8844527	183.9170968	123.1907306
24.72798323	53.87416391	0.442125289	195.9141085	83.2388421	129.6635091	84.62799296
24.32240208	51.13177807	0.957671388	184.5962427	89.9676973	176.1599978	95.87588701
48.10902068	29.97770732	1.351277881	141.0577879	46.6657859	95.30230204	69.26935476
37.20559305	34.18702844	1.70909924	238.1193149	100.91858	221.1989815	227.9164113
39.47577186	40.2336717	0.979944316	284.6754435	48.8333571	101.1263966	119.2968725
27.39617926	27.94880188	0.974809301	197.0271326	69.4253688	104.4266748	94.25827762
49.19837403	49.84935418	0.577589436	186.7336462	42.0955582	116.6433075	100.6642702
22.5962747	30.24883859	0.709674848	69.70968038	44.7680786	94.14701548	48.85089069
54.76761101	38.00702483	0.77624862	250.8702651	45.7576121	169.2938659	141.6704346
27.43912366	19.50401754	0.202616735	65.42020519	17.5686505	63.0751513	31.49639269
63.06068712	39.7779375	1.026582478	260.0571692	65.8870359	145.6926439	81.8007469
40.86990305	37.25756874	0.622443421	170.03595	76.1595145	109.8784355	89.50059799
19.43163477	25.86152715	0.374470766	137.8370003	40.1221173	102.2856692	57.18566277
30.18018423	25.26366861	0.501186833	226.9024742	63.1148757	143.1406351	69.37057503
22.02086432	25.31728951	0.581405698	149.4133824	62.5824611	87.46436481	70.22580722
41.61138148	59.45669743	0.583094022	256.3311478	105.321729	129.749742	113.6499033
30.67123518	45.23335445	0.358025781	224.7545774	40.2223258	104.7175544	57.64115529
30.2015553	22.78672931	0.785043646	128.9054908	41.1707934	76.56840012	77.10187195
45.66304121	33.33675622	1.161975059	246.4012795	60.9456891	146.3719279	140.3786525
24.24755346	23.95487136	0.870645287	175.7248031	63.8101637	71.67200419	106.8121872
56.53197777	43.65151262	1.236438932	173.9755871	103.835139	134.4239957	169.4883224
34.29828815	35.76173013	0.452740917	116.167911	52.7339454	83.30344487	55.80672048
32.17259152	41.10779066	0.645124664	200.7740199	55.730876	88.75503379	67.4916615
18.63695706	27.36402776	0.58449058	120.4903836	44.456291	108.7524685	72.89964394
27.09428373	27.14170618	0.535674107	210.9774736	31.8254278	81.26931202	51.94342294
22.91836929	49.04242396	0.52894713	141.2492656	53.2440812	134.8309373	89.77246392
67.34740248	60.31559572	0.805935014	392.5483183	70.0646241	168.4529741	219.2833152
34.89577888	58.60899887	0.95903998	223.0756388	167.142116	153.4135843	90.16107991
28.65579444	24.87464547	0.76047712	121.1192006	65.9932699	87.99718759	77.40708443

13.9309682	21.31855294	0.401084868	90.24729509	38.5349189	68.1223187	63.35805834
48.23380804	39.19791565	0.766242075	288.4648419	70.6772003	163.711724	127.8991505
25.13733089	48.52592625	0.394252604	171.5450022	49.2920809	100.4300581	59.53480743
46.20943212	83.91339167	0.683130176	390.2526322	65.5702637	188.9540889	86.27147205
21.05334981	32.96496398	0.513334895	124.1600189	52.9184194	113.6860151	47.52029321
120.3561529	69.3106828	0.797925863	250.8568352	61.7713871	202.986231	114.6022426
26.08519366	32.47114029	0.741876494	226.5001939	74.1249765	150.905794	139.6947344
62.99619403	34.48886241	1.092725476	156.0137409	40.8528154	100.6073983	96.56949513
15.84215296	24.02903663	0.775170261	97.41509041	38.3427173	70.52632662	53.00176022
37.80726651	39.46747696	0.535941313	197.4025851	46.8553007	122.2911589	92.45302162
24.44431397	29.11839525	0.374225862	124.2521829	42.2564833	82.2275556	48.68650247
40.2391398	32.60060414	0.940611944	138.6848261	74.6547769	79.35827271	96.6050737
19.94239066	23.86004103	0.670656258	114.0952772	46.4647624	148.2511362	50.42041504
22.97955093	44.62016314	0.588248873	260.8004712	144.255449	169.8230046	89.52177381
23.06319634	28.69174878	0.786571453	144.0037808	51.2103051	148.599617	101.6515513
22.08628776	48.26246512	0.453704755	158.0529168	60.7295458	96.33957767	63.02945184
18.34518965	25.98374813	0.465157495	155.1746409	64.8883243	75.47583353	63.08237432
15.56810996	23.09601608	0.696355033	191.9768547	84.5011224	124.1484564	61.23295496
16.71849093	21.83599604	0.490524627	114.067022	48.093473	71.44725848	63.6239849
18.24712803	23.51814433	0.478955399	104.6432609	47.7685103	68.22092772	44.02667981
29.03201631	29.00023423	0.460056332	162.6655932	90.5116254	123.6040545	61.03657158
33.63930689	38.82381055	1.605762606	270.2875921	82.861746	126.0158196	117.2611859
25.85889789	28.11818442	0.332685117	194.249238	46.7338103	70.14104518	56.7584497
24.42815206	47.15255152	0.39528417	179.3031051	59.5176601	94.37684903	77.60378306
77.63291128	65.7316946	0.87291482	339.9297481	65.7811567	159.2530456	162.7911721
25.57613422	47.11830452	0.45087933	345.4640898	88.3670929	184.5420097	106.4569363
46.27368343	41.29662416	0.716160833	283.0580832	42.4247268	146.9129254	64.08304017
29.83552434	25.23847139	0.232522685	87.68033366	22.8479355	50.78879001	19.63237214
14.5580558	19.34988618	0.267515989	66.68799871	38.4718294	57.44735965	19.94869671
18.5144358	22.13955066	0.485486734	145.0814913	48.8389004	89.80024771	55.62923676
25.60658124	31.56971361	0.734074996	131.4931879	110.868489	94.17202597	104.3514195
22.13775674	31.4794361	0.495635029	105.0931415	42.8560363	89.41234714	54.30311966
59.24987487	40.12420248	0.965308301	150.9223966	74.5964863	107.4701169	95.7974043
22.93067977	23.01535235	0.633028633	84.64218956	41.7234812	85.29696993	42.64904864
40.96187751	30.2956483	0.838320811	195.692338	49.4943539	116.005351	111.7920321
38.15949822	25.19530994	0.640442484	144.9129405	43.1518978	74.26003424	55.59405141

45.97319203	56.73516838	0.906183324	239.6311265	134.664755	140.4160277	204.9502946
33.78232591	57.33328181	1.668902053	204.1934998	120.307146	142.2655664	198.911419
16.70493923	35.86014984	0.716428951	202.1787612	71.0904784	123.0085697	77.57154121
36.25821776	46.00128708	0.47237349	253.5345316	46.9468206	174.3446555	71.5474206
12.71148242	12.96195736	0.450846444	109.2252769	28.8783224	46.12485155	31.51999728
17.51469189	34.78178646	0.434198759	112.5950915	63.4919599	91.44443666	103.7583243
37.82642841	43.53444806	1.15757527	170.9606696	128.483531	97.67464826	116.3858982
132.5369999	42.65959006	1.292111631	225.0590032	55.9073813	80.18158064	139.0743509
47.4457101	40.3416021	0.413788189	237.3349898	34.4807699	103.7109387	68.94154977
27.37478139	40.33193997	0.52195279	217.3129821	50.9660954	136.2395806	70.36014041
69.89996453	58.56186424	1.130040825	139.6787717	92.0776611	113.8457587	144.5281892
41.62347221	67.19660147	0.515822651	475.7927088	83.4868107	127.7397628	101.2494265
41.5075984	44.73935976	0.800392459	326.2937188	73.4710642	154.686461	87.65902713
56.83537094	33.37778448	0.416135698	137.4998509	67.6692515	86.48363465	50.7269494
17.24932939	24.69375064	0.387766289	70.18843482	40.98711	65.43551096	38.02776319
53.32994873	53.42202788	0.651680089	187.4108148	87.1084281	112.9716662	105.0746609
20.34200861	26.07960975	0.402124887	142.3378135	73.3634232	79.0961928	55.47638882
35.6028915	30.22925464	0.945201967	169.4852172	58.1440804	59.23486947	90.13191257
35.75106194	32.8925506	0.765288582	140.4921205	85.5109492	96.4237616	115.9891314
21.53082874	28.88362685	0.419383555	240.8075765	78.8162297	134.872822	72.15302035
27.67592338	25.1988802	0.524752082	88.2626361	41.7667926	90.29658023	35.16147083
24.69160929	34.3371111	0.566863582	196.8094124	62.2612669	91.64717658	123.2979812
75.38559143	45.01926994	1.18553719	195.7550489	115.241994	117.9305074	120.3713919
34.9151048	30.5157284	0.861232914	192.8017978	86.8966798	137.0084777	115.5757245
22.19779485	34.45932471	0.477307815	159.7786782	58.7449847	93.51046211	71.36439441
82.70628818	73.57205226	1.53123824	252.9645188	141.993701	200.6398956	351.6153785
35.16025332	28.07422534	0.37099945	99.93893506	43.1118783	92.71769515	36.85527031
45.53584144	42.58313577	1.02578437	154.369217	96.1735408	92.53840805	146.037422
23.14801942	22.0231951	0.600216325	92.22367775	44.0813559	82.57230418	46.95850075
27.54256257	35.43281002	0.544340574	165.1801257	69.2592205	109.3637211	89.64724182
30.84415222	30.78435005	0.594620627	149.8657327	32.3179117	80.30885008	55.15000582
17.90529957	18.56752123	0.495651745	125.0269156	31.0003929	83.53173147	33.65266637
17.70950397	24.45596005	0.343949684	72.22784602	45.3784151	80.52043154	22.53384229
17.7244811	26.63168378	0.470682948	132.127095	59.2833517	92.18703123	61.69417647
14.72968691	24.71883635	0.699934293	147.4732753	57.6989039	79.84661563	53.92697115
31.16334712	47.43068147	0.547049617	374.7902571	82.9526091	145.769212	106.8433223

23.89512041	27.22858094	0.635464922	115.589113	47.4131467	86.22735764	71.91783575
26.80038366	29.85226927	0.728488181	240.3330769	86.7510159	95.76173194	126.7067771
36.7640979	39.03744959	0.398224904	221.8630126	90.268748	122.9453926	72.95820235
25.64209268	36.13613344	0.94989708	249.5886903	80.1801028	160.165475	86.25539523
42.0182692	45.23280777	0.64352202	181.3290038	60.0685242	106.4985577	102.469144
23.91123304	36.42988141	0.720808853	198.1205672	105.212809	126.5859326	92.89167296
23.7570308	40.28412256	0.48873923	203.8254664	88.2970138	92.36642771	89.03538287
22.4185933	41.25761461	0.646057301	188.4320826	95.7183336	114.7404592	100.2986627
60.39689718	65.01070042	0.883685977	314.0168347	44.3632103	168.9973014	78.46594707
89.5616934	53.40650372	1.970963818	398.4730055	76.6503064	211.9491449	253.4360322
16.78730193	27.5137228	0.327920998	174.0149394	67.5768971	95.61695841	67.38307713
36.61895889	53.95367381	0.973448154	261.1328552	144.34461	171.7574611	136.045459
32.64431004	24.70497436	0.335387328	77.69399005	36.2148077	68.44229322	57.54756139
47.41122339	46.89399311	1.150347295	246.7308418	91.7667978	189.533896	131.2622821
42.77948353	45.85203226	0.607049371	201.1538412	60.52705	134.0578693	64.90550001
21.76096356	23.5406326	0.646405437	111.5789691	65.7241003	79.78652314	66.84806666
23.19824142	27.69348647	0.70047713	152.7962256	47.4731811	90.69862827	76.98405773
29.07146848	27.66997701	0.654929041	140.3360375	42.2222676	100.3129787	81.74276273
33.80088037	30.87314073	1.199677155	163.3428291	52.6531577	144.8992437	66.39809289
37.34629159	36.56740941	1.606886127	114.5187633	42.9548873	165.5875568	89.61185537
31.79030121	43.88313682	1.045572674	258.6555341	109.721087	134.9936277	111.2150319
22.52223792	31.48438108	0.730893722	124.4475499	56.1011951	106.0036406	67.3946926
38.71102982	48.36193083	0.809560528	186.2367613	113.639355	156.4949947	167.0627533
65.97352708	32.9280241	1.061689943	149.4698785	51.1255586	65.81617889	67.74601441
11.15910338	27.88793347	0.46446039	143.5216016	55.0777887	62.85505057	48.41858165
47.92339053	62.41313815	1.829788371	196.0575656	69.8498455	262.4767801	144.7694892
22.63695826	27.3157344	0.470653479	211.4495663	70.8675898	110.2387309	90.95329764
20.97448907	45.68574538	1.036013217	383.9076834	113.618783	189.3313916	139.9784592
15.88998949	25.65048517	0.55032036	209.7396737	73.1733168	94.69744908	61.19665118
17.63408583	26.53533161	0.523780402	115.1158676	54.7393305	87.70040527	55.87505864
37.79565374	49.55456708	1.588941525	313.309017	153.736523	242.4416482	120.6440607
35.38900897	29.75234088	1.200933685	208.7943632	78.813165	125.4197382	152.2610597
56.59270779	47.22099802	1.389053326	227.5372307	104.044108	130.3026711	161.4163281
22.47144911	18.81437999	0.545134578	79.72456429	43.1524831	55.90141242	54.23900679
25.77097872	17.97014104	0.355376304	65.25481816	26.3157344	65.97256746	28.726543
20.23826415	33.22097509	0.518312686	109.5920853	58.1892819	91.53834865	75.37503076

22.80635082	27.49337076	0.638642764	155.1526744	67.5579036	131.1693417	80.26060743
21.7105235	24.49750685	0.514406371	96.03521357	43.416399	68.85820718	46.08195949
27.31981207	51.82331869	0.72062744	281.8145825	108.603854	169.3129828	148.5855348
27.33454461	26.54829281	0.630736132	195.9034124	83.0643086	161.3605697	70.97521871
19.36196601	30.17468402	0.428960769	103.4937418	37.8681904	86.89101827	47.9033451
11.62631234	16.75463724	0.536519766	116.1248143	41.1192137	73.59007661	42.19131448
54.59561408	68.39287494	1.016618959	227.4652062	96.4168901	150.538333	168.5029421
24.44432976	30.3689762	0.663495624	114.3549833	47.9086328	123.8691734	58.74208628
50.33676664	32.73740499	0.545953004	151.6485085	52.1384074	105.0585946	64.48434899
21.249528	24.05513605	0.575988116	73.4658024	42.1991824	82.11770237	38.5388376
37.66215956	38.88207888	0.586330861	154.2796883	22.9570043	127.7237185	46.18822092
16.87332595	19.35601428	0.324274196	77.26544147	52.5841757	56.72833556	52.48985358
23.43036922	30.43976282	0.659744629	225.8167307	53.5661216	110.1378643	67.35590848
33.3644998	36.25249875	1.038928491	292.0342945	86.8669425	144.8981916	131.9151406
25.9310738	17.15103047	0.723222867	115.158072	48.2602802	42.28683492	44.81708299
30.17373433	25.1499886	0.463632291	125.4937514	38.3479243	79.70249222	36.54295914
23.18036797	36.11522959	0.487750136	154.1029418	74.7903267	77.94898384	62.00361053
32.6596491	24.98322561	0.615356754	103.8791835	41.9829108	89.01704808	57.1858557
36.11899841	40.11688802	1.414147603	360.1688745	84.0624821	141.7215229	138.1073292
24.26139907	40.01568461	0.649266307	181.5259618	60.9307774	129.0578311	92.68625069
17.21854522	37.3226357	0.521187519	128.0980864	41.0154182	109.6054863	62.22099765
46.66314391	32.3469455	0.970202306	178.294077	50.9546067	106.0374452	118.3951747
21.24260394	43.49811157	0.39740905	254.9494621	122.987885	105.1178761	120.6441201
38.68681856	33.37245379	0.652836983	196.079262	53.7202383	132.8347616	66.12020565
35.42895513	56.57775077	0.824630039	215.6171018	85.7319464	154.5138078	130.8351422
29.36538238	17.12050182	0.566303637	107.0698633	45.4533543	61.37378845	51.47603848
22.11309834	32.39672727	0.606465841	145.4929631	61.1150978	115.0417434	81.78537236
35.90957127	41.16088447	0.98334408	148.3475927	71.4423974	175.7256907	77.10586033
41.38958391	19.06495645	0.469421888	82.06964736	32.1944564	63.0419428	46.57464429
20.87546754	28.69003991	0.521055725	97.0098179	41.8179265	106.064577	57.60410455
36.71583385	31.32814591	0.679559472	148.6572315	38.187776	94.40901567	72.26182355
41.16153946	49.97683672	1.161757827	355.712599	119.198502	178.527528	182.187072
25.10750105	20.73612726	0.693991493	129.6522184	22.5767562	94.70609881	45.91831823
45.45889673	50.77465269	0.758163506	175.9191825	94.3873263	133.7848118	83.55204981
29.44493398	31.17233662	0.649986589	97.8805868	35.5549379	113.158236	51.2947008
39.03080304	65.94973164	0.56281552	261.5275497	84.504409	126.282154	93.84250991

20.49281134	21.93899815	0.612029188	118.5170122	49.6380909	77.4124859	40.11739455
17.8915952	31.4582889	0.615724243	202.0547189	65.5231225	120.4123792	64.92560501
22.6568999	22.7948243	0.409192729	124.4605822	37.7568486	75.15034908	33.05856735
22.89576893	38.16106869	0.685182439	115.9939102	53.6707495	122.0803241	41.34943257
21.86672926	35.16975007	0.341999686	263.252182	41.7953012	115.8806254	51.59047972
57.97891918	100.0933745	0.326417439	274.5126386	54.0903864	129.36722	67.81454697
27.79447494	39.16564325	0.941453007	209.9525049	63.4643888	134.5863511	101.9325186
48.75038317	69.14659005	1.267578855	331.2620461	194.760481	227.0389924	247.0094383
62.56765284	40.80268537	0.813214423	175.1814919	43.2382627	114.2818047	71.42599414
21.20493529	23.34884902	0.575386246	121.2017698	44.6569887	94.08282997	51.98852204
48.23507878	32.27107262	0.906019976	203.8048706	62.2515626	112.1561265	123.5855637
51.57158836	35.15431715	0.416450255	137.5296836	43.5030291	67.02312818	56.71508665
24.07983164	36.00339821	0.872846455	249.4631934	72.5409365	111.011396	90.94570708
39.36423934	42.71738517	0.605702739	166.9539356	78.4055654	98.12152958	91.32411921
27.13372779	33.89559249	0.686034877	110.1855544	43.9717778	79.47387043	62.99232555
30.78653785	36.88640861	0.85682826	261.6360199	89.4044985	130.6933738	98.53641854
48.34901559	77.83519293	1.042050247	363.0642465	185.103219	178.5782434	208.7417428
20.30693296	25.16662017	0.626699581	165.9253371	62.3362367	101.8144263	79.89536518
13.83400763	24.73025512	0.395101289	91.96077507	58.3269672	75.33215785	56.65927102
14.12671859	17.3162916	0.318420968	69.24909822	54.525851	47.06781899	26.14191932
57.48414379	48.75074195	1.449664324	204.1762658	39.7971374	132.550941	42.07853379
60.55253822	55.42318075	0.876234496	301.9774495	46.6005117	131.2476069	91.14603478
52.05192237	46.22856624	0.80632467	128.6465299	31.6694329	107.9334961	62.16682593
31.36194923	48.22293253	0.690017488	250.2223383	98.5485622	147.0562968	136.3378688
101.367584	60.1326357	1.102298164	269.5463007	70.8285479	162.7682304	68.437872
16.68143533	26.68750907	0.415945114	91.79416956	92.2385498	68.84745342	47.09850534
33.97123738	35.66667806	0.907027523	260.8465739	69.5822723	126.6025531	107.2502187
21.02067844	21.00553625	0.566134235	127.0812649	64.0892803	82.25771208	63.46361124
21.4845932	28.95955845	0.408839979	67.47703896	37.6831756	89.28672618	33.65656038
14.1327203	25.57305577	0.388628253	159.5860545	47.8897464	77.86029116	41.80394808
35.67755677	44.74658988	0.915918984	255.7170824	41.0047732	161.4871435	89.80710383
38.16254704	42.34043515	0.771675848	208.4402859	110.42693	133.3203252	129.0445302
23.12230368	29.61684063	0.512592844	151.2219328	69.1244231	77.02078826	75.98584553
96.48601324	75.40071283	0.566202371	492.6145176	39.2672184	113.2069826	53.33452288
23.96784854	37.20084702	0.770041492	193.8668314	84.2476341	110.6413963	104.8973912
16.39611791	17.84463806	0.534393481	85.25943814	54.7836309	55.16228925	43.61753283

29.1419683	55.18588601	0.770668873	213.1824444	100.336923	170.3872033	166.0992357
14.05382375	18.59211512	0.400650062	105.507614	34.6079384	52.38555481	34.85056392
37.36263106	50.19698947	0.87367895	213.3908662	77.4174907	137.1149904	153.0375776
36.96086489	43.06762004	0.690364571	231.6755046	76.4798031	127.9977511	137.7122901
66.66147009	49.10451582	1.133030973	231.9599262	98.2992516	140.242834	137.9968843
32.21305252	54.29875903	0.853614068	244.3666841	101.651165	174.0334225	121.7192164
39.76127243	33.28213063	0.715460123	167.0186389	61.6122313	126.0906162	77.68591045
39.26285204	43.72772201	1.266564859	245.1039806	85.2800153	235.511815	133.4821721
30.45745429	25.43656728	0.848711128	126.0805606	48.7043058	103.5909716	88.70534879
65.63843957	70.65670985	3.393445087	286.6778459	102.355967	309.4025106	226.0565122
39.54725644	27.27497796	0.643544395	114.6084245	43.8805115	87.15995213	63.93243088
52.80562627	50.39412648	0.99216362	184.5736647	72.0079576	143.8850952	114.0108692
27.68703574	39.10636342	1.142476047	229.8551635	161.19648	134.4707612	96.53714134
41.91462134	64.41656027	0.77597855	344.2428551	108.733075	251.1657173	153.6974453
58.07993614	33.58854875	0.93599654	152.0469501	73.745839	75.49709221	149.3165634
30.98184904	49.68386637	0.662260269	301.4759565	60.1576211	125.9452123	75.60335482
19.61876601	30.85057209	0.408356036	228.2312934	83.0465866	106.1540147	78.96839349
27.02159922	34.59487579	0.578626084	103.4823975	67.0417982	97.98988779	65.71702438
34.28935625	45.70838087	0.792992263	191.0457398	68.4152615	124.2544814	76.66927426
36.68533041	42.84789246	0.859879441	131.6442432	45.1883126	147.0420478	122.7744103
16.47622242	28.11707159	0.454515249	121.4133628	62.0425474	97.4487656	61.04960175
29.36335638	25.05115921	0.632647849	150.0573315	62.6674253	91.12963457	75.44733849
56.88518707	36.78421322	0.792418446	186.6062726	57.3327604	84.62211861	91.47578475
31.63510609	35.70249873	0.738694848	166.0779918	88.0383304	98.99907672	84.82519945
21.13950362	37.42695012	0.469517787	124.2201678	59.0415001	109.9048025	70.366705
26.39802757	25.96160803	0.87312505	239.0422024	54.7222188	127.9105372	97.42456283
18.68840903	21.59430491	0.503724976	74.27889302	45.4759164	65.05171436	39.47322076
17.98960649	24.02056589	0.363298863	74.79263072	40.2730519	68.66747047	41.30338578
34.69789126	23.29778915	0.963361381	91.18003515	43.2875193	46.80465569	75.06318876
26.78266062	33.69795044	0.758907708	281.0765305	84.3812501	116.3197974	126.6906454
21.23001001	29.85038442	0.620373062	205.0058097	59.401479	135.1070752	99.11543215
23.13362968	30.21244316	0.343557045	101.2651469	34.2854019	74.60303068	35.01301697
25.47311549	30.88019302	0.517785088	107.0142531	62.3629676	100.2382898	49.61874567
37.74204015	36.39992448	1.619816544	315.7552283	106.242639	186.9087779	219.4281225
31.97961806	37.13205568	0.542183365	167.4773535	73.5463047	104.5013863	81.94566395
43.53825603	47.51711091	1.458152663	213.2955286	101.037209	172.9965516	180.6093625

65.05288304	32.30736624	2.347198083	169.5373449	68.7845537	84.86625234	80.44663647
29.90217814	33.99147402	0.378725888	132.8815573	47.1511757	71.19431673	63.17338551
24.89593361	36.78583354	1.137416503	133.2304976	73.7727141	150.585468	123.9907081
13.73367383	16.16408626	0.359912358	99.38267932	29.0528897	61.81063648	29.0881293
28.3704412	53.92919848	0.917403558	141.3684436	115.935037	162.4829933	129.1410836
72.71151963	69.29896574	0.778804056	296.4649306	75.1671308	183.6016862	193.6471167
23.8080452	50.67963222	0.53651813	207.6344313	52.6739855	164.5318233	76.90309584
17.44481619	21.41055453	0.436091201	139.3415046	36.2029909	86.17405238	57.14980516
45.01055206	78.3084617	1.053714648	245.2804728	41.2214592	310.3328495	135.5843167
34.93010801	21.23411866	0.581016686	97.36841361	30.2121233	61.85839631	44.10682699
32.83853898	28.08892913	0.505600634	180.774616	50.5372351	164.3785366	77.90435864
61.6560222	73.0070247	1.001840284	338.707829	60.2783793	199.4262792	152.1843977
35.52150341	42.41577071	0.662273981	261.7309403	55.4388172	184.2812454	110.4042159
41.7988717	33.48716225	0.855221507	177.1919132	49.7136448	103.027239	51.44391398
24.13469909	46.8368483	0.182424195	203.2535151	41.448706	72.63566959	46.65189808
75.71972599	38.01650437	0.778827329	152.2493005	31.3731985	98.47767251	50.59068575
30.44613966	36.83286856	0.635977698	166.2285938	63.0610097	108.414105	108.5370286
21.30764484	30.04109266	0.735920041	216.423688	49.8194399	184.821836	74.21147682
45.11343859	35.27674703	1.120729342	166.8621876	62.5670818	126.3405173	98.53670467
46.58384834	60.84952045	0.434317703	258.5949091	56.587001	200.2807718	94.94689027
14.07242861	33.50440832	0.421297546	107.6465243	57.1606525	97.4847345	45.76788766
21.33555162	31.57994562	0.254002479	111.6116038	42.2773817	68.37743813	48.45646312
17.92575317	34.22176756	0.419732233	216.4135684	64.0262896	123.8825269	110.664355
20.60352967	24.92212908	0.413475372	77.80909211	44.0669185	101.7035633	45.53431058
25.64706019	36.05052745	0.43422682	191.1420759	43.3170726	103.0427259	104.4558799
58.51376035	43.69024768	0.79135957	182.5666145	90.494908	116.9634614	88.33988873
50.22944668	60.0272527	1.376659274	372.4522761	41.3764624	256.6219221	158.2112014
49.90106027	37.08189241	0.600178475	144.6357944	53.7532855	72.14110032	60.02100633
27.66494647	38.97100836	0.69831069	215.8185545	59.0111473	119.8172127	67.79713818
36.206155	52.97598318	0.206392615	141.2955179	38.7190368	76.31852796	33.74851256
61.58111076	73.21536562	0.719231412	258.7138388	65.011229	158.1361764	169.8807681
47.66765676	28.92620996	0.747336356	148.8461229	21.8277478	126.4654821	47.36173385
27.48687791	23.98054032	0.433045794	104.5912846	26.9000712	57.45576203	47.74673721
19.24951777	39.52954778	0.521270429	231.9223789	81.2631126	139.4073194	63.80477547
52.1642154	60.1933279	0.331283311	363.1070839	26.8973925	219.4904035	62.45793021
38.0691039	38.09689593	0.48676895	191.8826594	51.6190049	116.9756104	59.87447421

31.47493137	34.85335535	0.715297006	166.9641666	33.2270999	86.40297433	58.42012232
12.05445217	22.31080287	0.218577789	85.89149275	31.2731651	62.39585195	27.03519099
24.40096046	29.30198546	0.398297905	123.1064178	34.8846474	91.78653906	52.32028217
32.06226248	19.18563051	0.461238007	96.38462907	23.7994116	58.86954474	39.03318323
48.68935714	27.22666209	0.995623351	126.7056925	25.2788255	115.5104429	58.69472108
31.02021729	31.86065857	0.685192171	196.6000883	69.995423	139.6969644	109.7911044
33.9488369	37.10166317	0.523964413	175.2094594	39.8644964	85.96021813	71.01824569
43.9265675	34.88603718	0.841329751	140.4179502	42.1116227	120.9443391	58.33414118
19.10164866	26.96529083	0.341428853	147.7202405	53.0703974	79.8565768	55.2214769
58.68422293	29.63750522	1.374352759	194.5986794	53.5809949	103.2199828	133.8309042
29.16619467	42.1736234	0.82805721	168.1185471	78.3002323	109.8986764	97.74613055
49.7345975	33.86675904	0.540623427	176.6317078	52.7997815	121.4987485	45.19179612
55.25159347	27.95468658	0.647331969	183.6514089	55.2809097	118.5023109	48.24132919
20.78275628	29.80424762	0.390884024	167.7741056	51.1721141	116.9862294	67.56016518
48.79258757	26.56307028	1.345227743	305.2678989	62.6621858	137.233942	79.30319521
28.16494682	27.91010594	0.725486367	126.4102041	62.7362603	110.025726	53.05598509
36.06199789	29.54079292	0.66294957	113.9600629	35.0961817	117.940182	57.50122159
49.69690724	71.30830751	0.619665031	391.1916755	88.8664105	180.7276852	149.3650643
26.58256945	32.44722266	0.359879377	153.7675698	43.7178977	100.674502	52.34477005
29.76458858	19.47752953	0.432053306	59.89028449	24.1720797	59.8933732	28.24210389
56.04105086	38.6095557	0.512619446	124.2270335	37.4285984	77.50499604	41.27542833
21.5839393	30.85186558	0.469023816	192.0991549	48.0988824	90.66761858	63.2428125
65.62129549	83.93487335	0.544441196	365.3031958	95.0609734	146.3377925	95.87720702
24.35580313	16.09280354	0.453310374	125.5472831	27.7274285	72.52582457	31.01354482
33.65269501	30.16665034	0.507191536	129.7108813	29.9703337	85.03441677	43.94185494
27.1038942	41.28124193	0.487179864	117.7457266	53.3197009	108.6697212	140.6885436
88.41549124	37.95848394	1.165749096	162.5215795	55.3506546	129.7272713	106.1444426
19.62170722	23.74122526	0.481949253	86.83970155	45.7605373	78.24927154	39.8885112
10.33723399	17.13542538	0.296324104	124.1441938	38.7585209	65.88996138	29.40856589
21.28550975	22.45924337	0.779301654	161.8269986	51.1921328	68.25901838	60.06549349
62.78363288	45.22501349	0.66753712	235.6855873	52.6200911	175.1632588	121.7784067
20.00690821	23.42912399	0.41334839	156.4627809	36.7478497	93.93173823	27.81697955
19.38529001	23.58008116	0.35015005	97.49318063	42.1059879	53.1564454	34.69336783
34.83067914	64.60158192	0.863237779	260.0007384	62.4029641	198.2062967	204.0105885
23.00447943	19.68976318	0.637632636	149.1764473	57.9465709	83.305132	56.67808829
20.4507355	30.20690512	0.636553282	107.0748301	37.9422886	69.7220801	47.67958049

17.31464826	28.38293852	0.483593454	191.1547323	53.8562042	133.6407	66.18078553
35.84152529	27.84984019	0.158863642	158.2632382	35.3603575	47.93993357	41.18574995
61.95970351	31.87792023	1.015932309	128.6138796	49.86382	76.46728765	63.5793698
30.02334441	37.0827358	0.814053836	180.2854393	40.8520134	139.3990797	76.98845159
31.69685068	42.17075192	0.454490013	175.0527581	64.5424255	118.5695009	89.86731094
44.42232042	33.49535479	0.780270423	117.9192055	45.9190923	98.70937679	77.06494377
32.22189165	63.25105321	0.313263888	176.5644031	26.9766826	116.1331569	47.48994782
18.29078191	30.12733257	0.320101185	112.4859787	45.4384519	121.2743896	54.21460884
54.11047104	36.97417211	0.972506441	134.8617168	46.8115335	94.83014852	59.6871898
40.6976422	36.15161874	0.733930914	236.329321	48.0678057	179.1321819	126.6902387
30.56329276	43.03545235	0.577378241	163.2922641	52.6556699	149.9142076	77.71586482
99.83602277	34.64677027	1.027284774	155.2348526	39.5831688	64.36928401	78.36288838
20.75020717	39.21084739	0.302144974	129.299785	20.1386298	122.6141694	20.2759913
71.68656583	52.90082075	1.048613723	181.3288403	96.7575885	137.7459132	106.3955034
15.43450663	13.02768764	0.268600286	71.44132252	16.6030108	47.17798425	29.88915699
31.74185714	24.97274769	0.529071633	109.5241617	39.7558008	62.73486583	58.4480498
18.02554532	29.03769278	0.304237237	218.2427479	56.9297132	163.053557	48.90020941
59.0310925	52.8486595	0.47302259	162.139006	44.6060615	103.26878	81.69135977
32.60541645	36.06002098	1.017189758	195.3852208	58.1571931	163.4268059	129.8854141
27.84281033	31.99282592	0.519984028	129.6844341	29.1677504	146.8453868	47.74180405
60.33398217	41.9675445	0.895600592	228.7348678	77.1981456	116.3114373	99.10681818
23.66148959	36.43100964	0.572024901	230.198866	55.0466718	190.1303777	79.48952014
68.78560901	46.05850846	1.195327944	176.3350904	58.6444089	136.6022069	89.54338142
29.87100484	37.94622223	0.543536666	207.8102448	53.6243899	121.0082903	78.40831869
52.11689693	64.81867544	0.724648901	352.5402784	69.2460053	128.9329244	142.3826419
38.11237692	35.23352985	0.596981952	168.2278722	44.9676088	120.3179562	80.34563715
56.57330036	38.18183158	1.042514295	259.7065714	54.9486908	135.4878613	125.34968
52.9146925	63.63948269	0.904626883	313.1751323	64.2455195	201.1808761	87.20484441
42.72115588	27.92789416	1.356766302	125.0182721	47.8461624	99.88919963	80.56587968
65.24399773	56.97850427	0.922444573	222.220544	61.9394397	162.6919561	131.5083609
94.21796639	57.16268721	1.321282094	250.8715363	93.3811443	115.8493439	104.0912806
46.76055945	44.56488416	0.601667473	188.8400205	82.7869821	104.0036252	126.1439752
15.80706355	21.60486676	0.424240469	83.93054989	34.5371353	61.35813877	41.24668685
25.64287743	37.59616505	0.700142327	155.2366653	58.8032907	103.5295435	101.1450532

MG-132_1862	BDP-00009066_1866	Buparlisib_1873	Ulixertinib_1908	Venetoclax_1909	ABT737_1910	Dactinomycin_1911
0.371310815	10.08008326	2.451433219	13.05561579	16.13773977	13.97331429	0.010053155
0.154408069	9.347645579	2.358617321	22.42379657	5.7573564	6.69751469	0.00850281
0.216872746	26.89586487	4.201295591	33.50369006	17.01305419	6.979252499	0.02062967
0.177971727	10.64232077	2.494301775	15.62161776	6.915229506	3.466320681	0.010708058
0.213297864	31.74047672	5.114736191	38.33593484	18.41561782	29.74753351	0.013718422
0.198998564	6.031622324	1.896822933	11.99018318	6.33213553	11.88990191	0.007385831
0.206420379	8.525015194	2.433069535	5.248731262	7.702763866	9.013339216	0.00660253
0.191124405	14.62156531	4.000602481	23.80131161	9.074325562	23.49411599	0.014738257
0.234243341	32.85720106	5.517626193	31.89761561	11.64229097	10.45861623	0.018330519
0.169437313	8.845876727	1.967319891	15.7108798	4.023123196	3.501586148	0.007145608
0.199277748	9.264365965	2.579512733	14.48781246	6.770711289	4.405144495	0.007386834
0.218418643	9.446119943	2.837361113	20.55638514	6.676204524	7.277975718	0.010735917
0.195750725	10.27359066	1.897172712	16.38640422	9.315695716	9.50875294	0.006346041
0.324238442	14.89206664	5.404497196	39.64166504	7.894687229	5.12379433	0.013867445
0.173084813	14.76685802	3.473678554	19.14616718	10.81307209	4.158977023	0.010391468
0.176638497	11.45148214	2.768204832	22.94745755	11.3619578	13.15974991	0.014419994
0.125938302	5.544719887	1.656968598	9.328428192	5.892532995	5.673899297	0.003618624
0.188909866	8.193110151	4.080272054	4.76289497	11.1210969	23.1563768	0.011428612
0.165667306	14.25656238	3.992129726	19.22455277	16.46894268	7.401493742	0.013198284
0.13085747	6.425316221	1.873857946	10.9758176	6.027988618	4.932915102	0.00520501
0.183306925	10.78632057	3.297516147	17.45584683	9.259584678	6.723938914	0.009303831
0.232902204	19.70772259	5.024229476	18.91978048	23.14213479	12.62036691	0.01673267
0.181120856	8.527130252	2.199495487	9.350410322	11.80020832	12.28119392	0.004178661
0.236542975	14.17011111	4.17298609	23.34267675	10.46934904	5.783255902	0.009570426
0.175015511	11.08737318	2.995267408	24.83319336	12.64942449	7.299480017	0.011232949
0.141963861	10.24608777	2.278098863	22.47184262	8.48980858	11.68933366	0.008505479
0.256840642	20.11119906	6.026732444	26.07124078	14.34727312	9.143761842	0.024361305
0.184344433	14.34573144	3.791327586	20.7444748	13.88448656	15.96233873	0.012463851
0.156172092	9.290400073	2.634404666	15.9521048	5.626348712	3.555805897	0.006410446
0.263491136	8.162577207	2.559864949	35.38734242	11.63896419	30.69680201	0.011233913
0.173567676	8.670514258	1.859295174	8.920782858	9.248515739	9.135328863	0.008351023
0.172588208	12.57040836	1.882932902	16.75449761	13.96633946	11.01738339	0.008096798
0.151707063	15.45008137	3.203003162	24.2088044	19.79026422	14.41326517	0.012592806
0.188143145	11.1087215	2.757212993	20.33607108	4.026718976	5.873829538	0.006490711

0.224037885	17.52068265	3.825796322	26.23835969	7.532211307	5.815663755	0.010388898
0.198653964	13.23572491	3.202273369	23.92183897	20.75239758	34.476367	0.011786859
0.107736859	7.642008995	2.063887673	17.78896643	6.540080864	4.625683832	0.00450228
0.216413838	16.90821012	3.091010307	25.75414902	15.86451064	21.91616902	0.031100957
0.200124424	12.37545692	3.214001717	22.07884973	11.37978519	7.858833518	0.009283894
0.122874483	11.03087458	2.843955632	22.8864319	20.96795089	16.41497544	0.006925264
0.18850619	9.653372871	2.479957519	49.99172591	6.530326569	11.3877801	0.008051869
0.155574645	14.02053078	2.621059592	22.91407364	5.293143362	5.928443359	0.00885646
0.266140996	8.071078469	1.998888654	10.19131393	13.61384253	15.75786498	0.007003464
0.204238917	12.84436094	3.521146682	27.02858204	13.0942545	9.411856939	0.011616626
0.254005415	8.519524929	2.039078008	8.022248185	6.962594843	6.960017949	0.002676588
0.069479765	5.918929107	1.433895016	36.05605899	4.098050892	1.845570486	0.002369626
0.170457358	9.548450371	2.246509743	16.83040936	8.093960705	5.439059149	0.006215079
0.190851338	9.391106572	3.360111205	17.87410391	12.20051668	11.92417129	0.00704771
0.211821833	8.251178723	2.362740918	13.43612264	5.184126752	3.780579211	0.005569721
0.14768753	8.373389046	2.221224141	19.07225909	10.37562349	11.99632212	0.005629111
0.157649945	6.489273043	1.78113628	14.480929	4.583930044	3.821643967	0.003186957
0.148287775	11.95840985	2.813979711	18.96110559	10.36195625	4.928678403	0.009947099
0.149674454	12.82738598	3.208357684	18.52356901	19.45518036	19.24806634	0.013825143
0.136684527	9.375311265	2.466742168	11.75278423	8.298389889	5.860741026	0.008384728
0.168859271	8.383564142	2.841660046	14.8995083	3.324161904	6.200236642	0.006015198
0.22098526	12.45276941	4.172299067	17.78935383	7.667209849	17.34086456	0.012553328
0.188781223	6.494240008	1.928335474	15.74341602	6.822099422	5.88428372	0.005606003
0.16068423	9.421293973	1.998348295	16.3963604	7.682528937	5.200625802	0.00509227
0.327326946	15.41243101	2.892969982	16.92289358	13.93853045	19.95920709	0.008668224
0.169056311	9.429894177	2.971785667	17.46578628	14.93072594	8.781156643	0.004355635
0.292550286	26.93998956	4.269494095	39.12676584	11.4032507	16.38873944	0.040937305
0.185251271	12.61531123	2.722845055	19.46274852	10.3130863	7.067855777	0.014874473
0.205136672	30.54499631	4.472187155	39.47678703	16.4885328	14.18792024	0.03075726
0.177015039	9.756397599	2.943116384	16.32235785	7.368322242	7.822755337	0.006710103
0.212996298	11.14230376	2.156950136	13.336734	5.094086116	5.828750761	0.005580113
0.1762832	8.613953411	1.773862976	19.284267	3.194078613	4.817556407	0.004827746
0.137821917	11.84009643	3.126423909	9.040941276	17.22815369	8.11610726	0.004318508
0.26963634	66.00556757	6.47852318	47.32413604	11.99518538	12.58951192	0.024134953
0.146477068	13.76953853	2.950324624	23.27158473	9.456134284	13.60478192	0.008307041
0.231957739	7.706189261	1.786079095	41.30212385	5.04701904	7.908565616	0.009731261

0.14854569	15.70444455	2.653447937	14.69267512	15.73563434	17.01909319	0.009287317
0.144895767	7.007893308	2.461899738	32.93548785	7.022736645	5.815988389	0.005289834
0.185561475	12.85795957	3.363059402	20.6222891	10.18863954	9.469088539	0.013116456
0.275238678	34.50789789	7.525166718	33.90751201	24.53408607	24.90094301	0.028307237
0.16079138	11.48507719	2.849096617	18.83929767	7.832912668	7.670088666	0.008258145
0.151396222	9.283712012	1.908279408	14.65651906	3.827058254	4.353296124	0.004167425
0.299050383	6.928517538	2.243029114	17.10459816	6.488237957	9.906859404	0.005382012
0.181959441	9.306330397	2.162785551	13.17232507	9.242442709	8.161333643	0.003711742
0.206750104	15.59922867	2.639671059	25.55153095	13.69866543	9.202149481	0.009704833
0.188989892	13.02291852	2.731959305	28.81003152	6.754908139	7.643451759	0.014154338
0.170392891	16.47229901	4.959550633	27.05122753	8.088600663	7.643755114	0.011020237
0.1621531	24.82474301	3.959848911	27.04183499	11.98079814	10.07852081	0.014257043
0.122826302	7.853581676	1.712319008	9.942864535	5.406041007	3.088275182	0.004631559
0.151051757	7.550957998	1.891766068	12.69855129	2.953045126	4.783414176	0.003638806
0.221183085	16.05568254	3.562913841	24.95241171	12.25032874	13.59565417	0.016317295
0.174356809	13.9902961	3.566333165	46.76304981	8.485313135	5.166402852	0.011675811
0.143609078	7.367099305	2.586977429	17.99471812	1.470729976	0.222884749	0.006660597
0.28621184	9.070383669	2.572718406	18.78195577	8.437171104	18.30177523	0.010530854
0.14574668	14.86520989	4.020452549	21.20804294	17.79830601	23.8949342	0.015587898
0.140174224	5.92352622	2.055307818	16.29787533	11.95617778	24.08368619	0.005688931
0.266770267	12.07093301	3.600294761	20.16613884	8.584106674	9.035268809	0.007506059
0.14904162	6.618145815	1.842212057	5.485748777	4.592243129	6.16394706	0.003019519
0.273593961	13.50062701	3.150164985	12.9017591	10.18320356	9.218897659	0.004881728
0.216636566	10.04890135	2.625801234	11.03389577	10.89939035	12.52798684	0.009761712
0.150697443	9.148683833	2.206261914	12.25552219	9.035939015	11.53441032	0.00586641
0.208581429	8.896844282	2.432133407	12.78800908	4.516752209	8.706482742	0.007805766
0.18492202	8.230017133	2.432551701	16.65540298	7.013828096	3.594696472	0.004144028
0.127977964	7.467019257	2.243976076	7.689085476	9.570186032	17.65374485	0.00398231
0.225697194	18.43973163	3.003099595	25.05369254	9.825256024	3.992906456	0.010661633
0.217307179	15.83030466	3.4219159	22.65855879	7.501045035	2.394605247	0.011337366
0.140538288	12.16322109	3.774454413	28.75380004	12.95213961	13.59743927	0.009232265
0.217658989	15.74933441	3.183966889	27.65462632	9.367629364	5.820722819	0.011856806
0.089759929	4.732072734	1.711960491	19.34173008	3.818961737	4.645143643	0.004991789
0.226808935	10.98843361	2.252742363	13.70153385	5.864327607	11.36321706	0.006613596
0.175377203	12.28999886	2.332660275	13.80789177	8.942282448	19.01424479	0.006917048
0.24398804	12.07093195	2.739331244	15.18707972	13.46601986	9.382189673	0.006857052

0.167036051	12.11413614	2.772388711	29.40765434	9.719303685	8.451224293	0.008507456
0.182915051	11.26359906	3.137227456	24.52283098	8.835722172	8.780202952	0.01112495
0.230448784	8.943020302	2.211796054	17.5626914	5.073701696	6.098249209	0.007445861
0.292955442	17.26656613	5.108738007	22.64144504	19.81571598	15.65365983	0.018741579
0.206185776	12.84499991	2.673295417	11.92281296	8.392484271	7.879375466	0.009786223
0.184118546	8.020106121	2.317351374	7.173540561	7.622488921	5.878247852	0.006158358
0.195195031	20.51966638	5.326336419	27.05100676	15.49606946	8.296914765	0.025675169
0.162214386	10.46403103	2.376546256	11.60372027	7.384854581	6.458742511	0.006384633
0.230979787	14.27788873	4.196898153	26.72825814	9.242318586	16.71357293	0.013960285
0.159345088	15.98265205	3.051556952	29.17461477	8.383362704	4.631209738	0.007967656
0.235843668	10.44039131	2.988398488	11.5172764	9.486693568	5.19135478	0.005639996
0.200073182	15.00976308	4.560385288	23.12180514	8.690215741	12.45199489	0.016526279
0.221851255	16.16918562	3.093760057	18.01215428	14.24779407	27.04405277	0.011357941
0.180755759	9.347901349	2.839667634	20.45777853	6.833040046	6.807794139	0.008948302
0.140372576	8.212675774	2.296068038	30.51812461	3.361249198	6.470569058	0.003518615
0.252840607	9.267062378	3.064824771	3.894570511	11.6770416	10.27941009	0.011667966
0.137471806	8.865053206	2.290187816	15.85641502	10.60977188	6.937998007	0.004867947
0.214663717	8.305956119	1.934204874	11.67287274	7.901605724	6.247046903	0.012761812
0.184312174	8.767097637	2.703628463	17.77695029	11.65473547	13.65916797	0.008241296
0.175962326	8.586999148	2.151351328	10.81983276	10.93067556	7.808690695	0.007610301
0.361020614	16.01105563	3.464053983	18.07986565	9.950448915	12.39968054	0.022224118
0.166289585	8.846817247	2.694689925	11.96930755	9.533606428	7.797455423	0.005734576
0.153772409	6.309141548	1.837681512	7.561871412	7.638471025	2.496558738	0.00409411
0.122380169	7.667588572	3.197553871	10.36736056	20.36009214	14.34281393	0.004519607
0.15409341	12.55574633	2.689299012	20.93845819	8.117447739	1.444793129	0.010920532
0.116033051	8.672622429	2.023735532	12.44460765	9.109952728	10.39932929	0.006082922
0.488263114	53.23648771	6.540222561	21.77198376	13.14953104	12.16544627	0.014641965
0.16109158	8.531487869	2.317410394	14.15219788	3.867286169	5.743371697	0.005141915
0.205334674	10.29239615	2.796967062	14.90392483	6.419886837	9.985120116	0.006358157
0.223588353	20.88120981	5.128275261	20.97001292	7.713442364	4.812012196	0.021447164
0.213628746	12.02116784	3.431359724	24.38697687	19.25788074	29.79732172	0.010226497
0.135945229	11.85162815	2.919107247	20.26610365	13.07740947	25.34373582	0.013017302
0.183931718	10.18130422	3.678406967	23.51840945	1.958839718	0.723375194	0.005867566
0.215329554	8.633647147	3.309456417	8.91861952	9.635822568	5.208665087	0.0146983
0.172287848	12.85652951	2.773523922	13.13660062	10.28133101	19.00988048	0.008014495
0.196836998	13.85237698	3.955530611	19.94086683	13.92159528	14.71826381	0.020271864

0.22615889	10.39870468	3.063500998	7.394756292	15.96686009	14.88153828	0.005872158
0.159964781	18.60751428	3.300650372	31.74535356	10.12535269	6.202844842	0.010891025
0.214861783	11.93556586	2.606760715	25.28513725	14.22041998	16.73914822	0.025027504
0.169621861	14.69702772	2.858250526	16.87661608	8.85104097	10.08856384	0.006648713
0.348086519	21.19894807	4.148426629	7.002679281	23.65858948	26.7400728	0.01969388
0.169457726	12.16859801	2.608871537	47.35035833	8.162569102	21.01748152	0.008618997
0.196156735	13.50626901	4.305863122	33.06182583	8.18747771	10.39381028	0.015124503
0.159306716	10.5497207	2.465826031	17.32413887	5.437387081	5.513747718	0.004260568
0.276703637	10.36214508	3.176894825	5.955718455	5.958166984	5.289315014	0.005783705
0.178801512	15.7512515	2.781958269	42.16534485	11.86433153	13.30792776	0.012332558
0.283693202	24.26327448	5.270584516	33.24355091	13.23820954	25.70531893	0.023675585
0.237430731	9.017106639	2.100264983	6.594984683	9.640579542	10.3147805	0.004617317
0.153691769	9.78717858	2.419178179	17.46082585	8.890878568	8.554155929	0.007686416
0.261542916	17.770705	5.449570445	39.16419727	6.960576361	2.333058869	0.010772725
0.163836849	6.404990672	2.218065794	7.778707923	6.68165664	4.974180559	0.005653262
0.430806959	20.18857631	4.348290156	31.21261677	11.68613256	8.975341157	0.012309248
0.084702073	4.497819634	1.052966292	3.885614181	3.495951877	2.832017125	0.001726671
0.185734153	21.41511321	4.133187973	44.69767721	15.94753694	23.24800601	0.027134209
0.165780958	15.12759071	2.967737964	33.16653959	8.189638093	7.21784076	0.010048554
0.169896875	6.677997817	1.650356729	11.35924039	5.643768905	5.385300935	0.0040502
0.119130832	14.33893918	2.09078389	24.81292023	8.663028981	13.97433654	0.004751331
0.184197969	9.55568082	2.117671303	19.04922071	10.27719863	20.80236293	0.007718164
0.263243178	16.22777099	2.538238316	18.3311643	6.855091526	4.065976741	0.008631334
0.229330309	11.82002895	2.83863526	11.85964119	5.418916715	7.195485149	0.00646812
0.14617763	11.16188302	2.309093143	29.47109888	8.227254654	10.61399982	0.011139533
0.224919208	19.78591901	4.158168259	25.26662482	10.92712374	19.91819758	0.016748172
0.152124737	7.565680278	2.882689429	33.32900519	12.81882638	19.0369329	0.007129823
0.236152536	8.558631598	2.08736335	11.62383678	10.24992266	17.66626869	0.00883816
0.193672941	6.766015649	1.998569286	11.31412179	8.210687193	15.30480428	0.005676924
0.18407836	6.492761022	1.472551951	10.45877743	7.931827251	15.6176565	0.004886179
0.202643719	9.442285826	2.107770308	12.30108846	5.819354709	6.029283167	0.004836771
0.293988958	7.234609814	1.489971189	12.99694877	11.98486953	12.5433387	0.00477579
0.174346083	8.466027757	1.83481753	6.800432589	4.667403127	2.652486285	0.002913094
0.443125709	23.92283984	2.61826841	40.5730684	14.27614175	9.547343657	0.023296932
0.180541723	10.51916784	2.759817728	19.76757945	8.834873535	5.881843415	0.006493439
0.122722189	10.23224024	2.336308256	17.78917238	10.47030623	11.83721182	0.008133333

0.179153687	7.633649895	1.920764736	11.94011112	5.869821293	4.218269782	0.005490545
0.157142588	12.26354257	3.082347935	22.09938348	16.16708313	18.85256794	0.012570645
0.199520869	9.390936455	3.212949334	16.43672385	3.507650855	5.1629269	0.007329083
0.152846701	13.29809554	3.366675922	15.58859674	7.795591539	10.36429204	0.013831187
0.214803848	10.55796478	2.431915593	15.19057186	8.741114389	10.15448343	0.006418791
0.270706718	24.6429941	5.635898239	34.39153867	9.290294386	27.56932863	0.027427807
0.198087205	18.8668958	3.383628846	36.57419702	12.267742	12.79432929	0.012476906
0.249976586	11.0175407	2.50071447	19.58093096	11.34653042	10.28716563	0.018307809
0.22706723	9.253203771	2.360378441	13.18164699	4.341024681	5.261698575	0.006250789
0.345440368	10.70783732	2.393633964	15.05785732	9.990513985	11.92134029	0.008338431
0.152074823	6.400782768	1.867302357	8.316677829	9.454459172	6.739254797	0.003048317
0.151307672	9.497406083	2.741724633	30.48851753	6.461064642	5.75056053	0.011843386
0.141638326	15.81546219	2.866900567	42.25646465	9.635380245	11.17193966	0.018567187
0.199950933	15.03632416	3.745299001	20.37470579	10.45152063	6.629665095	0.007781023
0.222652082	7.309916884	2.809807749	18.42374169	1.932882339	0.273616354	0.012127966
0.145397713	9.305142756	2.480234628	19.68306135	3.124580952	1.964938117	0.005398757
0.134034625	9.313678112	2.240181712	19.90183108	11.47317167	8.816088943	0.005653217
0.21846246	13.57993871	5.020761541	22.73042098	19.30234204	27.0072957	0.012025011
0.166288051	8.531802078	1.783804606	13.14476554	7.207499373	4.406442979	0.006422998
0.118237589	8.853820491	2.381769863	16.3045954	7.226476006	5.227599784	0.007459834
0.219127645	11.25218509	2.554522782	19.80387696	12.02585051	24.68993183	0.008127613
0.205130927	17.18420465	5.578104675	36.24856149	16.16049102	6.069265379	0.021975827
0.222019862	13.05926104	3.239378799	40.18131497	6.333756093	5.329301551	0.004672172
0.204247775	9.727332422	3.747696826	26.15257707	8.526423533	9.843637111	0.006410864
0.183565595	22.41811238	5.487956362	32.41588682	8.275068466	14.42267929	0.014238215
0.177986451	12.12820317	3.018792117	37.4783957	18.01417688	28.53876471	0.010014243
0.330303268	13.18403906	2.569932781	13.69895403	20.56600045	15.31698512	0.009693647
0.10618945	4.684026883	1.621546288	25.48566006	0.513462557	0.547636641	0.006826387
0.163977611	6.114563749	1.615442605	9.904750432	7.966515227	8.368334424	0.004430337
0.157745257	9.575611088	2.264059291	11.06485144	14.64382533	11.60598715	0.004137961
0.136657412	7.498890787	1.939243106	8.033316806	7.960166338	8.173696384	0.00676367
0.174400089	9.098073034	2.125781756	9.750703757	8.402328878	5.693141605	0.00585819
0.193209409	15.26732751	3.560136061	32.94771572	12.1981301	19.38415204	0.015606412
0.160978295	7.563625842	2.061687567	11.90433473	7.269101381	6.725519219	0.005618113
0.268136691	13.71223403	2.174371497	15.80924624	11.33351698	12.61858795	0.007969537
0.219408277	10.83867474	3.478407865	56.46662507	4.40823052	17.0136668	0.042267673

0.199955147	13.15648679	3.038539534	16.04739269	9.986740558	5.931544178	0.012494366
0.133076334	11.69505968	3.078160433	32.6185959	13.4732629	19.1206573	0.013946029
0.165918766	10.8546937	2.523633737	14.2988358	9.270132046	5.281439638	0.007132958
0.180336873	13.6480416	3.190260931	13.78224388	15.58140367	21.07874968	0.008908536
0.136333004	6.230910818	1.634364708	4.144762915	6.930922738	4.719360902	0.002387368
0.159795551	9.012761278	1.642406178	16.1658293	3.005906407	3.972850325	0.003383954
0.143719774	12.40676298	3.640491426	23.0289439	14.23196804	15.85425947	0.013519853
0.236152384	15.99569048	2.182927772	36.10308383	8.387111992	13.57840487	0.015703061
0.362162302	8.857166339	1.59560576	9.593601769	8.4063806	14.50657869	0.004329386
0.240440209	11.93171395	3.56477027	12.4361845	7.199377373	15.97503221	0.009455667
0.159195081	14.52527448	3.394390531	14.12341077	13.25195784	11.79076569	0.013955232
0.151370058	11.82999035	3.142869172	17.22337908	9.644022309	11.59039592	0.006102479
0.161752289	15.56734221	2.747498382	20.65797923	11.18518474	7.225246522	0.01128635
0.201001939	11.58560561	3.032697361	28.27826338	14.80923209	12.26926668	0.012260506
0.201406328	8.214457925	2.166783059	15.17573246	1.782017475	0.76558192	0.009343445
0.18172986	12.23049802	2.630778569	22.09725104	8.965727543	8.905415346	0.011406076
0.169331315	8.05141532	2.131446345	11.76767794	9.260402324	4.790946458	0.003894606
0.177334936	9.863324323	2.386128568	44.03996787	10.4236074	4.118816509	0.010457451
0.186487967	9.757409046	2.960140426	23.05438585	9.618363925	12.30816626	0.010395925
0.202383399	11.21338027	3.481422574	28.644103	12.98349597	21.46213222	0.006050022
0.237311257	7.161505939	2.041110169	9.062715645	11.7108839	10.27062207	0.005354514
0.183984244	16.44264913	3.4924142	27.82146089	6.470820388	3.35923215	0.00665511
0.138876014	7.410613457	1.884511864	13.88960055	28.30552769	31.42021737	0.012007355
0.186509819	17.34361369	4.46195993	32.0689346	10.80964127	11.99755635	0.013663159
0.160621607	11.05295129	2.480566201	25.22084249	5.929977577	3.370948085	0.004560974
0.278914269	24.43304872	4.868359443	41.18592584	13.3613757	10.35092955	0.039014617
0.240431254	12.70398047	2.468528148	15.54098103	6.912550167	5.317553931	0.005238877
0.155295206	9.711396156	2.7581894	18.33676464	8.932695643	16.32634566	0.008100964
0.173169665	9.015882193	2.191359361	17.21136338	7.496491025	9.972661941	0.00702865
0.168630826	7.826555281	2.289546301	9.372267963	7.720874635	6.140965909	0.005188342
0.164518606	12.21124686	2.959110193	21.92646469	11.46346145	7.508117951	0.010534966
0.157719562	6.425596584	2.566339892	13.55763742	4.888346812	6.049250648	0.004441012
0.206513761	6.302652385	1.569113792	9.97727223	7.063501695	7.554313214	0.003548409
0.181162135	11.16500203	2.228600698	13.81760817	6.0342387	5.722377768	0.006345949
0.091930196	7.786967112	2.131282475	14.41699917	8.291558411	11.63949413	0.005062426
0.215756016	13.82513507	2.319834259	15.71996682	10.988816	7.309888357	0.006440405

0.211032528	8.315535498	1.988106587	11.65754667	5.14064276	7.284539833	0.005162968
0.156774928	12.23967859	3.167779305	30.18020788	13.9377002	11.87319434	0.011032286
0.199424658	11.01257045	3.105420574	8.9210712	16.05532103	6.108039732	0.008709664
0.191785995	12.75953336	3.41759677	11.84320074	15.3573265	17.07782621	0.010483579
0.17377484	10.49970291	2.112497347	11.43342081	6.419440256	3.360567859	0.007409936
0.183791716	12.24621896	4.553546923	29.4334653	13.37031374	6.385625974	0.009525538
0.174345602	10.91621299	2.59673889	26.487864	8.577640445	6.709383595	0.00733993
0.173046987	10.24756329	3.004392272	28.5219233	10.68463924	20.2471639	0.008316837
0.259962615	14.09447687	3.832516786	14.65371788	8.669246612	12.56192978	0.013943872
0.225731078	17.29922746	4.325311504	27.77310871	25.88821839	17.549473	0.012010321
0.15550438	8.15498258	2.401409866	9.758929178	10.25630031	9.296985204	0.006201293
0.208332527	13.61919712	3.69019648	20.60016698	11.07614623	8.61933761	0.011187877
0.191360535	8.908339773	2.793456937	25.16292217	2.464578309	1.428432203	0.01306133
0.208095782	17.19559214	5.96294499	30.8530919	14.51046233	16.19332202	0.021217422
0.318607791	10.40838255	2.821671552	25.32581973	7.443775509	3.826197261	0.01118536
0.138966601	5.853751605	1.750285104	7.788056871	5.682897312	2.889465295	0.003741426
0.168209786	8.381934991	2.3371365	16.99714263	6.76613566	9.337724321	0.008617966
0.280156215	12.20357962	1.992092504	15.01901779	10.02520181	8.939259385	0.00776437
0.239354488	12.70402104	4.144707118	27.084681	6.296704876	9.688370352	0.022674764
0.214777094	9.984936773	2.847605457	4.344535369	12.27108083	4.774828337	0.007087208
0.126031429	10.08852533	2.58507269	21.88615744	11.23625071	4.750489733	0.006422628
0.185445845	13.29847276	2.903028784	23.46575612	9.593652778	10.45953344	0.009320486
0.111072937	5.189714671	2.023813844	4.727106918	11.51716168	37.85390912	0.00384389
0.2503071	13.25207947	2.134562176	24.49786271	7.157650653	2.767587188	0.019803661
0.116642598	7.845656726	1.710077507	5.646948696	5.175135151	3.154334942	0.002467761
0.230069453	9.54215745	3.412547505	4.532393496	8.388875051	15.28852662	0.008503971
0.152493173	15.48340597	2.961947633	18.01480662	13.641066	11.86530188	0.007418021
0.176857946	13.67487375	3.403456222	34.69245452	14.26568956	11.93227817	0.013047054
0.146620748	12.15863578	2.665937199	20.63547334	8.477449721	6.813863229	0.006050672
0.162638692	7.469741569	2.044580794	11.01983837	6.531319657	8.564924107	0.00404924
0.174808	17.84816841	5.044764076	18.20056368	25.81442721	52.08088365	0.017200738
0.191952877	10.36921736	2.676898605	20.38946065	10.03395884	12.02856805	0.015538923
0.203134939	18.27066816	3.953233719	17.05939177	15.87074561	13.15982113	0.017045918
0.236251117	12.52110278	2.202460043	23.14315863	7.592452476	7.881218388	0.011821055
0.103698366	7.524882781	1.412782478	12.9657588	2.297263533	3.107567583	0.006866382
0.240310912	10.9591349	2.170475248	8.183486551	4.027862972	5.325262553	0.004948265

0.228447063	11.91802954	3.778552121	19.45289744	10.98204719	14.12413141	0.010607608
0.167934798	7.44496722	2.061853012	9.623394298	10.50933562	8.46473681	0.005304147
0.178670793	11.21815039	2.703485152	19.40759476	8.278456657	10.15446791	0.008711412
0.209561244	8.508997679	2.804521335	11.63110994	25.53784488	34.61418651	0.004835989
0.200199271	10.80901116	2.714295229	21.81566122	2.416758718	1.189198623	0.005303542
0.159402769	6.877940071	1.990677852	12.14172152	11.23498958	24.54202297	0.004511119
0.174608603	9.734996771	2.184257901	18.77436436	7.043728502	16.59657845	0.011340185
0.22749648	8.717361674	2.479988771	8.184652814	9.241940353	6.06266698	0.005383996
0.22336231	14.02549298	4.503330822	31.51879679	12.69980141	7.47967148	0.010861817
0.205756623	6.610184668	1.809438917	8.599986692	5.857729627	8.152986121	0.004095519
0.157629667	10.09660097	2.249805238	18.27328479	3.97460415	6.564959547	0.005719388
0.149512436	9.164092499	2.231451219	17.47912831	5.09469084	9.207909083	0.007346986
0.206981511	9.952457573	2.732321289	12.19034537	10.58093013	13.9238578	0.006238692
0.193411554	18.05085302	3.2763058	22.77830436	10.09686295	20.67763641	0.013214099
0.128509736	6.2718209	2.734531734	14.21983971	5.335631141	3.773124793	0.005668956
0.217561102	6.672565834	2.288578951	5.590990597	5.752465318	4.950424963	0.008343266
0.141419939	10.13300375	2.723188839	18.79304386	4.612961109	3.600256363	0.005359449
0.182724435	10.91178273	2.643436821	30.70510995	8.079423035	7.188473843	0.011377907
0.195528965	22.0093858	5.444303521	46.19099546	18.67320593	12.7108713	0.021754545
0.168839413	10.53532445	2.732438596	19.65078	5.57084737	9.264564456	0.006691859
0.133584332	8.910439374	2.93676109	24.20177158	3.626192795	1.549923348	0.005315625
0.240447621	11.63708245	1.882188316	13.86672565	7.047213177	7.058518089	0.009217755
0.168697483	10.67826085	3.250525471	11.73061059	16.29680233	8.880459547	0.004481513
0.284196211	10.73515883	2.449809539	8.409821871	8.702253041	14.91277695	0.007927838
0.172824613	23.33215825	2.833431808	25.49902931	5.932206115	4.791333838	0.0046667
0.136137708	9.19776126	1.55724882	25.73023176	5.24201371	4.964074638	0.006468811
0.203277869	11.59146472	2.598464436	15.29692277	7.519554745	5.360185255	0.007269669
0.274355563	7.7234215	2.292214203	5.761783419	11.25988026	12.08767254	0.007098276
0.191405081	7.120506453	2.47707506	48.45375965	3.998735831	3.037491248	0.007008924
0.219172737	9.27044633	2.338717229	10.87371348	4.756312196	3.693109808	0.006253233
0.274170716	8.55011603	1.954652262	11.86231791	10.47043417	19.85812335	0.006294662
0.16653555	15.75845827	3.724714434	16.39640403	15.01341029	30.09639508	0.012377837
0.271564225	6.495386021	1.82298948	8.745818642	10.04677608	10.17397325	0.005125132
0.164745707	15.13310016	3.366284315	20.45576721	12.29509288	10.95463116	0.009141684
0.280931931	7.950239557	2.430766855	12.57177517	7.104876176	5.6140864	0.009941438
0.140307369	9.240022622	3.754870229	15.94442176	7.496060729	9.417510005	0.006993501

0.138501743	8.322564799	2.195110246	13.85565887	10.00534978	4.79267936	0.005528311
0.183993541	12.703596	2.623971541	16.394753	12.76099292	15.67255573	0.008035148
0.190199574	7.935202083	2.167665753	11.01441681	10.45107583	7.113928899	0.005037207
0.20623373	8.085866269	2.590327138	8.092337217	7.290866238	8.09819194	0.005746665
0.130924934	9.515845177	2.900418914	21.31703689	7.557743961	5.629025608	0.003943612
0.191640226	11.11146621	3.298635232	24.13470246	4.919593972	6.609928949	0.009302937
0.228622813	16.63122964	3.48663227	10.47675322	13.12518684	10.49434029	0.013202844
0.283648202	19.31510601	3.434243125	13.65398042	17.50397806	9.38443795	0.011877058
0.340484601	8.767024762	2.285985562	10.81150424	10.55946198	24.41076391	0.009252613
0.171922653	7.661359144	2.093297661	25.31760133	7.105684819	11.18260753	0.00604808
0.188390034	20.98664961	3.784419353	59.20509369	8.850523755	16.68240837	0.018773773
0.210169961	9.298506972	2.159470511	22.52897451	3.081291617	1.800606388	0.007054195
0.146920582	15.19666391	2.883827084	18.33826809	13.74075791	10.36244374	0.008768877
0.192727217	7.660203365	2.488839114	12.73212023	17.10126222	15.66002558	0.011098286
0.182535776	10.2441319	2.696609505	21.79142135	4.956096025	5.492087133	0.007481515
0.174735539	10.75388929	2.706998771	18.000583	10.06016489	8.634884973	0.009497111
0.167340424	12.66509542	3.669409683	25.76543389	11.46708218	19.03800714	0.011464161
0.185717689	11.36267151	2.409747155	12.88230987	13.05211167	11.6534173	0.007004351
0.156699641	7.313315499	1.805159334	12.31489703	3.767235854	3.489410228	0.004169913
0.096084062	3.67626243	0.883535596	4.871835636	2.908335381	7.050116536	0.001731057
0.19531286	10.81929735	2.275189784	19.6972655	8.92683292	18.95672364	0.018850909
0.165713447	11.06700152	3.687930072	19.1989585	11.85558937	7.027759777	0.009582505
0.285936711	8.572497751	3.646311502	10.12852542	3.352010218	4.843420963	0.013792189
0.204724803	14.0663793	2.997828846	20.35513068	11.51227927	18.00533374	0.010840724
0.239412603	7.258842708	1.706234122	8.683969162	9.600875081	5.840176313	0.015411103
0.139660785	5.36977227	1.625729066	6.900854253	5.798967109	6.829585051	0.003325331
0.213380788	19.30955764	3.387650988	26.11800822	13.94477916	7.134926347	0.010342944
0.152372493	9.482816806	2.515464778	21.95617662	8.354001034	7.694132643	0.008248017
0.19145208	7.908960051	1.776367433	9.385636587	7.684246697	10.36951293	0.006189201
0.144921413	6.062274374	1.453463203	5.464786846	6.72442164	7.033449148	0.003225805
0.235787396	10.51993928	2.686255872	9.28661986	10.23855772	4.36914599	0.007290278
0.214706033	16.26545061	3.480120746	23.27837438	12.38644743	20.24528564	0.015214686
0.232086907	9.904431061	2.980556409	15.86507105	17.74746731	11.0424293	0.00758477
0.187365547	9.670566296	2.943494291	17.35013139	8.393072359	7.893914687	0.007400768
0.161253216	10.604609	2.837068414	21.33555184	9.675855452	5.431940841	0.008539615
0.135989516	6.735640257	2.294761697	14.6556425	6.557025452	5.08372971	0.005418025

0.204178008	15.90530215	3.670240949	23.42864171	7.41663051	5.63919758	0.007790139
0.124667525	6.396946973	1.829847327	8.462009531	3.829152837	2.170405382	0.003114784
0.192393142	10.96858947	2.412898033	16.98790827	15.64722197	11.20496468	0.007725833
0.244913025	20.0300221	3.547782148	25.28168412	11.57161226	6.885062149	0.006571961
0.180576443	11.53873164	2.837906223	21.11005137	12.51608221	14.21784975	0.011480703
0.197777145	10.5208093	3.239641154	11.68381418	17.60872216	29.7773836	0.015666006
0.242602461	11.82712808	3.656208634	17.66204662	9.663033524	9.763451395	0.010682192
0.219168299	7.477447335	2.773391108	3.652164364	13.55552087	10.19411504	0.006478053
0.267610206	8.971695025	2.145008513	11.2437004	9.658455925	14.39849545	0.006355273
0.266687103	17.50223453	5.254323017	6.551788882	13.61527624	11.56007758	0.030187244
0.348596706	8.614865659	1.960464845	7.903982341	10.45878668	17.76097688	0.007022016
0.236161415	14.16689499	2.408804316	17.97051034	10.55985431	11.61537951	0.016267209
0.138742533	11.03721932	2.799633629	26.32219028	9.430853621	9.803426731	0.011384976
0.202854676	17.84045856	4.303266759	22.99840338	13.21884107	15.59999597	0.015170923
0.170494054	12.66723149	3.14849126	41.63175966	7.706442483	9.167870264	0.012876975
0.164046661	11.59883628	3.498965153	29.28750349	8.873874567	9.901364421	0.009933897
0.185253983	11.13995012	2.025536719	12.0672617	10.43700547	15.73527495	0.005933232
0.213094171	10.81253761	2.297509191	16.38219256	6.442482163	4.930975473	0.007799427
0.1748174	12.35309125	2.73375766	14.00803039	8.188102577	10.04151104	0.013353407
0.361648007	11.94068017	3.629802376	12.31410828	6.58202574	3.320438343	0.01045609
0.171824003	8.514419817	1.960623695	14.45019801	4.967101131	6.232930203	0.006119211
0.157934304	9.222560164	2.411054463	13.97902483	7.33356009	7.429145498	0.007306273
0.174705804	11.46685866	1.618223335	25.99658955	6.844708603	15.34518584	0.013326068
0.154945206	8.91318795	2.061875755	9.140608275	10.46664029	8.002082568	0.006004319
0.184328059	14.00570627	3.254502427	25.52619091	4.678229387	3.644085987	0.009196162
0.174490655	10.67293955	2.845191827	13.33176144	9.95771438	18.20056483	0.00676828
0.20184707	7.351803094	1.582222117	7.419872354	3.825513787	5.207884444	0.004889487
0.208177416	7.42354282	1.744270067	8.182344869	4.62958258	3.897479762	0.003975596
0.122381428	6.564828375	1.907472276	21.81812541	5.584661447	6.53666707	0.006616383
0.151220713	16.65258096	3.063586541	20.5936511	13.67604749	21.10415094	0.009851676
0.170450221	10.64276016	3.439733297	19.87824972	10.60677109	15.48152414	0.009439657
0.109544727	6.8470809	1.813600093	11.38529124	5.89893737	5.867715591	0.004841592
0.210817739	7.986343435	2.567142333	11.28682503	9.63137956	10.8454333	0.006214972
0.164907826	17.5312939	4.06534543	23.28079423	14.09446333	12.54024222	0.014642328
0.19209576	9.436148015	2.563913925	14.91450351	9.032570871	6.314439711	0.0059516
0.220434848	19.1303825	4.440775068	23.28689037	11.53062074	12.8203582	0.017905387

0.235506562	8.551771199	2.16018636	11.64850905	11.48704744	6.976081893	0.011381617
0.211410563	11.28142108	2.047713408	14.52037011	7.91311142	11.18417807	0.00561474
0.211926885	10.55355275	2.878013559	9.00911672	7.331945535	5.255145357	0.007712241
0.203863077	7.234760087	2.161594409	9.150359163	8.961612622	12.69304583	0.002415901
0.172067086	8.523265415	2.504384983	8.173905882	5.890331877	8.896855125	0.006807543
0.27731922	10.87817963	2.217872948	16.12380021	14.19607091	17.92712356	0.008261289
0.435481741	10.34233887	3.409375801	15.31665576	5.481536278	1.991310227	0.005940219
0.147776224	7.547412103	1.999873222	17.64385129	5.038849766	5.29580522	0.005730387
0.236094442	12.40030896	4.062027066	17.3607269	11.34470553	7.354931822	0.016421322
0.263451518	6.331804577	1.44156108	7.656784419	8.15794885	8.864440953	0.00353771
0.329507409	11.32796535	3.248106701	19.92272944	11.77889849	25.01423618	0.008052632
0.305059266	20.58373622	3.371916344	22.62075516	10.85729853	5.471467398	0.010554756
0.345951167	12.57289481	2.866363977	15.35104397	11.1618511	8.352485246	0.007845272
0.210049489	7.416022235	2.006181443	10.03256277	8.12502458	7.236796588	0.008428663
0.340040174	5.034620802	1.18652626	3.607603226	4.828301077	9.79612244	0.001579312
0.420618838	6.794520198	1.518517311	8.253891503	7.69276673	11.32461469	0.009922372
0.213271023	10.67138664	2.496185248	18.81424955	5.467008736	5.160247496	0.007593552
0.309850768	10.24857377	3.09365306	23.07755325	8.361189322	6.041391853	0.008739604
0.299088655	5.046943582	1.755197384	4.286260201	8.14842937	5.831483569	0.005536138
0.328474179	17.08102417	3.200706878	17.08464059	15.71264378	24.76454164	0.010910928
0.16563538	7.556944481	2.028744999	20.41220864	4.209655705	3.250022942	0.003632602
0.156809065	7.552129074	1.497478125	12.86017871	3.417443679	2.459109876	0.00297816
0.192140442	11.44348132	2.572909125	21.19995488	8.004723649	3.60253732	0.004368192
0.198112521	8.705339304	1.8642306	12.04000238	6.365074621	11.61997467	0.005076685
0.222484919	9.974422744	3.397843625	27.08416397	7.526879818	6.905001336	0.006058275
0.263827177	8.969241192	1.895616933	10.74815465	6.692363554	13.28770781	0.008844244
0.236059923	16.62330497	5.028526728	12.88994489	20.29162134	18.72785631	0.013397597
0.26345208	9.849405172	1.856678463	13.4702831	7.397377494	11.78353021	0.007916405
0.329366426	9.109311195	1.861432099	5.0862849	9.792949102	9.229285668	0.004798225
0.288688679	12.42270805	3.510030969	16.14881551	4.83776456	0.614275191	0.006176964
0.260755905	15.95213218	2.643283839	27.93980999	9.095489211	10.01283848	0.007605973
0.295911967	10.25729726	2.944820872	16.24847478	4.490152775	1.874940783	0.008124478
0.248937263	7.57369935	1.45584318	10.18823357	6.318623479	6.220794885	0.005347332
0.178155291	9.075679815	2.058656145	11.81960341	6.596596339	8.603525878	0.003831528
0.303037172	11.1567888	3.869265804	21.29170698	4.491948479	1.922984064	0.010036838
0.332274317	8.035847873	1.61533768	6.832307914	8.821928314	12.30142017	0.004274403

0.177023214	11.79848771	2.416865266	19.59391707	6.261880854	9.890023986	0.007582941
0.150588546	4.764579465	1.332070058	7.25277697	6.253655561	7.867865476	0.00211498
0.199441387	8.687698021	2.222400615	21.60265019	7.203460711	4.618092898	0.005646044
0.159328217	10.58738473	2.031147504	8.999327321	11.13046331	7.43779787	0.008138978
0.284524409	7.61413182	1.697527374	8.269287179	10.3979063	4.513855537	0.005660264
0.279802899	18.31357228	4.195579485	31.55836375	11.62867246	10.01485839	0.014705249
0.222837074	7.618216673	1.755392683	10.51015898	5.378943605	3.976306987	0.005487865
0.412041524	9.097226327	2.472337014	7.502453027	9.125879083	24.77584013	0.011443835
0.145517061	9.503904267	2.077892215	29.79857917	4.582675234	8.447374443	0.005257244
0.319314685	12.41568363	2.72032343	18.4962682	12.89277361	10.10934581	0.015407663
0.202379891	10.16419708	2.989154875	7.750938447	12.55874211	17.53109013	0.008611812
0.416034908	8.018058289	1.435899517	10.30299093	7.071380047	15.9417053	0.004594054
0.281966637	16.8233984	2.366185269	14.23633285	9.292682052	26.40330231	0.010876021
0.166324267	9.035655181	3.105173718	23.36344273	7.777109217	4.041183546	0.007515848
0.20155074	8.239788913	2.347026896	19.25450128	12.74559055	15.75013206	0.013026608
0.291990259	6.000198528	1.507417734	3.755721002	7.2297635	6.074114226	0.003688757
0.242847536	9.276166117	2.105507958	12.12507499	9.130458808	18.76962581	0.009157616
0.484084845	13.28759358	2.424319941	10.78763279	10.6052908	9.550917736	0.005858725
0.212991976	8.298217678	2.874312181	22.94613897	8.515701538	9.012189882	0.005691489
0.209300327	7.345476666	2.36813115	16.60855089	1.40511779	1.07563532	0.009312314
0.304825934	7.584073695	1.613476904	7.173344424	6.865405115	25.61161184	0.006849173
0.154768546	6.848380737	1.698000884	12.258091	9.172534222	8.113223251	0.003564818
0.261202788	11.23678513	1.770003717	11.53795801	7.790713276	27.07298789	0.006679534
0.180493379	6.798641372	2.130238223	26.74661484	7.657747925	4.0214558	0.005089702
0.402493693	7.786620293	2.358967703	12.76188847	8.566201861	8.771636717	0.005217036
0.171107128	12.56569063	2.838760718	25.47465081	6.870751423	4.27581976	0.005657804
0.318108608	17.63712223	2.528958962	16.32145369	14.17484462	14.82941096	0.014501415
0.23020654	6.55428024	1.540626223	6.6852375	6.114371756	6.018985124	0.003983994
0.146446066	3.972449639	1.202583632	5.165073917	6.67588001	10.64722007	0.00219197
0.166910303	14.74575246	2.362730068	15.28380645	6.415287867	3.58520582	0.008404546
0.3269794	12.18904365	2.111208964	13.55185649	10.12543561	6.52585352	0.006228843
0.16292078	6.15702176	1.559668775	10.5945395	8.776729815	6.186416698	0.003628437
0.163491024	5.383541668	1.277893606	7.983230175	3.716494933	6.873028931	0.002858253
0.330700629	20.47928298	5.534721968	25.81035093	15.72340364	11.44001585	0.015358076
0.200697488	6.585366384	1.70124464	8.11664566	10.46355922	11.62463614	0.005274713
0.13963358	8.851901825	1.982559829	12.11589234	4.572164741	2.68204913	0.004243815

0.21871224	9.807909667	2.199434391	13.96168217	10.1027246	6.252817233	0.003302047
0.231619617	5.083865483	1.540307278	4.307064484	8.281441511	40.30711846	0.003067158
0.306184218	6.544998428	1.907026112	7.885270614	15.74778167	18.4792679	0.009157282
0.254854727	9.727310078	2.621633467	9.770197799	11.49039371	6.244108997	0.005969604
0.23027256	10.96189678	1.757099641	17.50426853	5.710504187	5.850867289	0.004804309
0.234004534	12.6803597	2.017776017	17.91493723	8.781076472	8.380412828	0.008039331
0.242082651	9.558495795	2.780163558	9.681925702	1.813031647	1.580961106	0.006446241
0.179539296	8.083351913	2.275737288	17.59258696	3.449979886	1.790708566	0.006422303
0.263430406	7.975010188	1.555094338	8.432252551	12.56327408	15.53263687	0.006376492
0.299046814	13.04821296	3.397271852	18.96250499	10.42758464	5.970404535	0.008099436
0.223162241	10.02780481	3.016380511	13.34222952	5.165659588	4.438797256	0.00602104
0.240041912	8.228490235	1.684629064	13.44214962	7.063122805	16.57502595	0.009396581
0.25474899	12.64962938	2.934362927	16.87566632	4.367527294	6.204695349	0.006614758
0.258049046	7.899657048	1.871835367	11.15350064	10.98809819	18.09705679	0.006393363
0.143137272	5.408906278	1.451448943	9.013071992	4.65232288	3.779241228	0.001858763
0.250817353	6.996948334	1.377656856	7.952254835	4.752953544	3.838438914	0.005655932
0.272198196	4.873345952	1.797929458	10.48188448	6.692640419	1.441607476	0.002761521
0.335161526	9.187733578	2.466906319	9.280969316	7.665474059	11.02681367	0.005391998
0.260669522	12.12271378	2.828633112	9.358252835	9.373577989	6.26845194	0.007334516
0.272170118	7.335083368	1.997919368	9.903298862	7.487750694	6.811715072	0.004880046
0.323690786	8.709661055	2.425783417	15.1450732	17.32181127	18.8563725	0.013547762
0.273996653	11.20412976	2.461378036	13.88283104	5.644689396	3.679775429	0.005890821
0.405420882	8.859420319	2.000321816	8.940186936	10.35060859	10.278398	0.008566664
0.35968727	6.613147325	1.664688156	9.43192379	11.60815858	35.96738404	0.005743377
0.176850884	10.66906169	1.806921407	10.81535539	9.001812613	14.97210535	0.005733229
0.222498274	8.902779484	1.768289633	11.31195623	6.092403662	9.106505963	0.00498858
0.388446628	11.43765077	2.313240912	12.14554128	12.85802634	8.301162904	0.00648923
0.395203755	16.2608026	2.634311096	12.5591877	14.73673901	21.54310615	0.008757178
0.235064612	9.01511659	1.910790399	9.768268707	11.91590366	11.60917798	0.008335251
0.376768326	11.58586208	2.571038438	10.90076758	9.556000302	7.900593293	0.011964698
0.326330073	7.835916162	1.513951363	4.816581621	10.78319355	6.020157461	0.005412997
0.239246584	7.925011805	1.633086798	6.860994176	7.344861211	13.10869533	0.006586341
0.138958054	5.790681029	2.048289501	18.74366954	3.016786331	2.321141027	0.003608507
0.241316253	10.66194543	2.443023111	9.760912571	10.02206694	10.50025181	0.006817189

Afuresertib_1912	AGI-5198_1913	AZD3759_1915	AZD5363_1916	AZD6738_1917	AZD8186_1918	Osimertinib_1919	Cediranib_1922
17.29654177	142.2634563	13.0042654	32.18231691	11.89223728	34.88996072	2.796453471	12.2632561
11.32572047	99.3144913	21.85325391	12.41967949	12.12114741	15.29207648	7.983786691	5.032748716
29.32964439	123.2092319	23.45101287	54.21131975	12.88622214	55.48369196	9.865888159	19.5161971
10.95402873	113.5172307	10.73989568	17.19775156	8.883646916	16.17803153	10.10313632	10.18294066
29.56533633	136.5145574	28.69501381	56.2459995	20.66056964	88.53330207	15.36348834	17.17524849
12.73916757	79.56111197	12.95861687	9.329921557	4.741785556	16.02086752	5.720854566	3.78052144
13.04881072	88.36807412	17.37289368	22.12509383	3.822767633	28.34755172	8.828455747	7.295374922
20.16972108	111.714035	16.77066327	29.99622847	40.27941918	28.93512199	8.496929166	8.840455413
30.81308446	153.945417	32.13901062	51.16242671	31.21969705	46.76428236	20.08856827	20.96138559
7.412796029	68.09391626	10.79775566	10.18909643	5.562440784	15.24747171	3.31985995	4.769334411
9.566111418	90.86884277	14.92491853	15.02552041	5.697404175	26.25715218	3.641570523	7.323503824
16.73561136	111.6305835	18.3574518	29.79577842	7.664827058	27.20456313	7.07265119	10.60848744
7.45506041	93.00036043	9.119686716	11.98839885	5.301460859	28.01781972	2.771532096	7.682123971
28.12374192	90.95998058	15.70034238	49.46347642	29.1665162	45.20226352	7.592482212	10.64005464
16.89790473	104.3037706	17.52106945	25.11729112	6.805967141	25.11519277	9.253225168	14.31546949
8.809786884	130.5661134	15.82432001	12.01906317	11.00637079	15.29097555	3.38472775	4.240885555
16.33445564	64.05718765	21.24339845	30.64086504	2.076846874	75.23022441	3.989028868	4.373403459
22.92225244	143.9248132	15.70401743	48.23924004	7.215273011	94.27794795	17.12057783	9.040475571
33.8217274	128.4662326	18.53274398	42.83859905	5.108322844	37.96786288	8.167899698	10.6236888
19.87840039	84.46044531	14.79317064	16.55336105	2.005682254	18.97261551	5.363860889	4.459259405
12.65766796	106.3651449	15.33937789	14.50649771	11.37423724	27.76767325	4.815247147	6.933724425
38.08798779	154.038451	37.74298834	72.8828847	21.56601898	91.97752505	7.303995699	14.05751827
8.320981725	104.5141566	15.24715433	11.9904098	5.958955255	18.44703483	6.554716569	8.616447218
13.86896596	162.4696495	15.63746729	16.32133416	14.16234707	25.95593996	10.03069357	16.73482211
13.53106967	111.9066079	15.02082519	20.57078	10.38386129	22.70040111	6.825152125	7.511989173
10.21215245	95.35563749	14.94378385	11.81989245	7.611092768	20.21554422	4.891094894	4.609596677
38.58016567	120.2990882	18.09726197	80.62069552	25.66405921	61.78370114	8.60596824	22.17217959
26.87097031	154.8473706	15.18594998	33.40043079	15.6639066	35.92488504	8.277684855	9.561216969
10.70087522	94.00113715	12.0862043	14.59603729	6.391594316	24.23604986	6.7999306	6.987917768
14.25709223	90.61374183	11.73816585	12.99322966	5.342187471	23.70859879	4.336295007	7.117466295
7.514939388	98.23723555	13.09350333	15.40842102	7.358028143	21.56608906	3.386098664	4.732219787
17.00555673	123.0172672	12.48785937	18.61090418	7.522732399	16.35409501	4.159609278	10.43523324
20.52346187	164.4655226	16.94846657	27.33503895	11.85928957	31.40465488	5.116482833	10.8529409
11.46944089	82.52152821	11.54553247	19.25799982	8.902413193	32.62610081	7.597945761	6.351369997

13.45759803	116.8978862	19.39776555	20.21940482	29.2383137	36.14809528	6.968026715	18.81126584
15.73734118	152.3439841	21.22430891	22.61933649	17.02178039	25.73548329	7.353853547	9.760810024
12.59121503	80.80298181	10.30988111	15.58296944	2.511439979	15.88067728	4.013660116	8.645445179
18.01784602	144.2917012	12.11457146	28.63596729	20.08431057	31.13051586	5.136215416	11.97734276
10.53982996	103.7252038	17.62620396	16.05196227	6.851058356	24.13289661	7.225807026	13.02207188
11.27208283	142.055991	12.88183374	19.90577463	4.291507953	41.99610883	2.268519542	5.552600388
5.379060395	100.5808942	8.561274005	11.64840013	6.915675285	28.77686574	16.19707907	16.40292468
11.67846491	95.42291765	16.77094673	11.81269302	9.89845486	11.63898596	8.938314101	6.665117988
9.552743513	126.1160568	12.40135474	17.23984031	8.915830328	22.21740738	4.817027671	11.05674472
9.702731962	136.2418418	10.2627025	16.06589377	18.33237293	19.42845913	2.501822104	10.53895107
8.323041555	96.74006993	15.83150889	16.02371901	6.16929268	28.27984575	5.178311425	11.43478634
4.450297765	55.98094373	9.477686618	3.863041758	2.630585513	28.17621091	1.625460367	2.409348073
10.09032158	88.70780712	15.60073559	13.54161705	4.258248268	19.55318797	5.983489836	8.559294752
9.973757133	119.8192369	16.80169244	13.66640018	5.869965328	30.885299	7.722557486	7.20362068
10.3258207	76.52141687	13.7113987	17.10062386	8.420165552	24.9082544	6.271474995	9.450865214
8.58614933	87.24542651	10.74180032	11.812167	4.775728473	18.75022197	3.559615097	6.605648722
6.831574331	67.11593562	11.74071414	9.52486117	4.346748997	15.6119958	5.911564242	6.077771392
15.885204	111.4729535	12.57629055	19.04298232	5.53010334	18.96178927	5.864330969	7.75350921
18.26860657	134.9747106	14.26167899	32.97151091	8.409670135	29.83997378	5.2789976	7.948873969
20.74643461	121.5786572	11.7470373	24.01223835	5.156457887	28.86876049	4.250021257	6.840249042
14.04865828	90.52990093	14.49380059	23.90113723	5.591013385	34.07940606	19.23668706	15.07373489
32.44167681	123.0894236	12.44005317	51.03961018	7.750385788	34.44957364	20.88580493	22.81675558
6.865510176	80.79201625	11.54032813	10.85607619	4.261152255	25.66936926	2.778673417	4.502249854
10.68528711	98.66589329	10.79502386	14.3373144	4.273742336	16.08824724	3.369388073	5.251140101
14.15842438	142.5104069	12.45279323	27.74432578	20.54673801	32.25557442	6.822352792	26.47240584
13.72935891	139.0268194	11.49788101	19.64788815	4.799228163	32.82692482	3.236920284	7.751783954
31.29299295	147.3852599	19.62555267	38.59282543	38.55254837	29.52546366	5.504785692	8.627517601
15.91812217	122.3281893	12.03405429	23.60723163	12.95439239	22.59099195	2.879563325	7.58156862
38.74600384	164.5213633	22.52582252	52.66076216	37.24349186	49.71461148	5.231629681	15.12969253
11.05596797	96.51884126	19.68375035	27.27309764	7.115152098	60.52071315	7.81514922	8.976832785
9.619863752	86.27583885	16.23920338	14.78228171	5.625360216	15.89829049	6.835066412	10.57861743
8.037608622	72.54500272	11.83713168	11.8916259	4.297034534	11.48187275	6.128671533	7.279518611
25.76835062	131.3799882	3.306922763	40.88173189	5.179602787	30.44291071	1.999122945	7.844234005
43.44668767	150.1153824	20.7638213	90.32653807	84.42891751	70.56319047	15.95180955	21.27193055
20.17368445	104.4053548	12.35494277	23.56120577	6.420872523	19.00797689	6.670730849	6.048551497
3.94385977	72.48025408	10.42968504	7.360237752	4.385789325	12.03214224	2.034882634	5.809743988

18.85229758	133.5836593	15.32777974	22.5963245	3.762029112	25.406921	5.414879874	8.90711392
2.989520421	88.42430646	7.169137001	4.126615779	8.340180349	17.73612335	2.53290461	6.507169682
8.596236529	131.9962182	12.92639095	14.99927373	9.160509674	23.03611175	4.036927866	8.223726854
69.17521616	216.7253898	31.32284581	119.0739303	24.14227466	90.22017215	14.39302483	20.90011159
19.80479007	101.0639709	12.35805356	22.79992807	8.400175038	20.48505754	5.688948809	9.883426085
8.353298582	77.15017615	11.0622813	10.22133354	5.882952742	11.50837701	6.424101363	7.921863665
14.60055512	79.89383168	11.22909514	21.20098053	4.798126324	19.08889555	4.050036605	10.12317881
8.983207452	112.3998342	14.57495712	14.82845061	8.319636389	17.73099098	5.074934638	8.489382708
12.15342377	105.0660297	15.91938309	23.07024416	14.71446314	48.59902644	2.491965102	8.503709396
9.972378728	87.16674391	11.63182156	15.34450799	11.86446606	23.55181599	2.697660555	6.258677475
35.89736994	108.7951467	15.18363813	68.45998121	6.535875279	54.03086032	26.47507547	16.18139117
17.79446412	123.951859	14.10131098	29.29581285	26.7542537	23.06075971	4.338599813	11.49615263
8.995431208	92.27416322	14.63731804	14.54839946	4.875391556	19.80292901	4.628643949	7.74283603
7.392197758	78.24676546	11.46193993	11.32425671	4.861182233	13.05570587	8.355420599	5.963200009
22.32092962	120.9007781	18.29918272	47.35989747	18.17421084	43.10518681	4.639701408	12.98459762
8.735791877	135.5426152	17.93763945	14.86118528	10.00200627	26.56196718	5.857263957	10.18950228
7.30507404	60.24049355	15.41323282	13.43888586	3.785696734	39.26229277	5.656063518	5.660103114
14.51337048	100.1376747	18.79289153	14.46486271	11.11745043	18.12806961	4.451080943	8.371059418
36.14172612	146.467291	19.72395645	45.67993606	6.241322762	42.36385041	8.684276107	8.213515581
22.92963448	112.010122	12.38613519	20.39535552	3.354624422	17.07047842	6.042675582	8.685172358
14.43289401	115.4518534	24.84964731	27.61302426	10.77783359	46.35228543	8.168523541	13.18798181
8.884621451	76.80716972	15.83980523	10.04437316	4.448178224	21.60573647	4.787619969	9.787669347
19.48485694	115.669864	16.94502588	42.48211409	9.737897077	35.30914176	12.88985439	21.32138967
13.17653931	109.6836569	9.895849517	14.44617975	6.943217943	19.14070689	2.153182553	4.736078347
7.647349363	97.93448055	13.03697807	8.147620222	5.280911667	20.99531371	2.657053983	5.502653636
7.846480133	90.89884047	14.40482367	15.24790369	9.217983375	18.05056828	11.05550717	8.195630954
8.426470346	90.31649977	15.20130626	13.93737166	5.894709081	24.92390837	5.17195096	7.892507855
15.65669475	108.9090445	24.04527261	15.05338573	3.040669624	20.77412735	15.80396632	9.740309417
14.45023027	126.5578056	14.24449464	28.72880279	20.4178433	20.86296331	7.247719257	11.16904415
26.67355184	102.0640659	7.510286266	42.90546226	18.87925522	26.19008252	5.604618616	15.90654143
19.22506522	133.012727	29.18568759	22.56501909	18.14973079	36.14100755	5.824826471	5.802636507
15.3179354	109.6902246	12.3904836	22.08380378	10.01136629	19.52897196	5.079441789	8.818667749
4.684096468	65.78479051	14.54302356	7.003481517	2.556189055	30.53884513	1.928757649	2.968121701
9.240956685	95.18859005	13.41711138	14.95691481	11.07162782	21.57716556	3.388094334	6.594565277
20.96049123	82.1913274	9.593343766	32.14085504	2.843419021	38.75737316	2.766372697	6.656827679
23.08134916	129.413182	19.29665973	27.85826212	6.189367575	36.63550776	7.619060898	12.27410372

5.822489909	112.0639425	14.77104098	9.357148309	21.01310052	17.49362563	5.090584881	7.716118876
9.369076267	115.0420176	14.72754452	14.20640781	8.154449394	18.25954899	4.493051814	6.077350902
6.106789398	67.68289779	14.34777853	11.17300117	5.937034711	14.96370889	4.911602485	7.47261933
44.60656845	228.2698856	17.70050871	63.13373361	38.99170482	53.14614545	3.098116575	12.24486296
9.444110065	115.9196478	9.00766539	16.34979187	7.809360358	19.76154607	3.862749745	5.685255565
20.38276948	90.23040043	9.441245974	25.65980344	3.274162319	18.67647068	5.652496777	6.181380865
37.6159944	179.9890117	15.56331901	54.63704517	19.55847504	37.94553025	6.554058813	9.211613948
10.01174426	106.8228018	20.27659606	16.50842367	6.358151907	22.24654258	4.348468253	6.71834238
18.3705234	120.7651768	15.50819977	25.94861428	19.34501669	30.71712051	12.09973587	10.55108906
13.57901296	103.7041006	14.40352547	23.29823996	12.28275771	29.37785642	6.68833431	10.58575347
13.85319218	92.11969388	9.528581168	30.33891643	10.7003054	33.64455747	7.129459635	12.46888375
20.67196236	104.4009951	14.8882047	32.51644874	18.1894428	33.53190407	10.96405106	14.3792847
20.43409354	162.5736934	23.38650291	25.91563423	8.149082445	28.03213384	10.53184299	9.201251049
13.65337935	76.65164666	14.2734883	18.37758196	7.075357328	17.9773406	6.24502159	7.877969722
8.354027971	98.96498845	14.04415852	9.687904281	5.192544333	25.33480791	2.765632506	3.569485946
13.97798357	114.0866172	20.36970677	25.51758682	3.866540064	34.62867226	6.660826508	6.762341684
5.477649477	105.6878318	7.439805416	7.625547318	4.632732951	13.78364374	2.353606756	4.306444982
12.37919675	104.6797925	12.70292313	13.63742085	3.081241617	24.14566236	4.984994212	7.7477801
14.86496783	120.1188783	17.38295127	25.71306886	3.101210782	55.18973663	5.791290946	7.23444186
6.272451776	112.1466512	12.97257212	8.26355264	7.747208135	13.12253379	3.207495894	6.063104965
29.07007557	119.4846592	23.2098591	43.27527895	12.21087129	52.26555311	7.799509133	13.33328551
9.593931118	104.7555095	10.34871356	13.67177708	9.054506451	20.27551988	4.849748674	7.107429179
7.154122361	90.32162977	10.59199757	12.33961739	4.136877791	17.53263689	3.369327136	4.980082183
25.51399612	155.66134	12.81986711	34.93690523	5.899450014	38.24611373	5.792497394	8.465919953
7.272539968	108.0207861	9.660756045	14.70077429	12.25625471	18.68927225	4.807369508	9.640670347
17.27652451	128.591163	11.42387051	17.63726938	4.720954394	19.06643422	3.585094973	3.608966711
35.01461374	148.3765978	24.97610749	73.08068144	30.09492076	45.13199716	18.02944736	45.28112393
9.065081565	86.52911155	13.13847351	14.25315673	5.556179862	16.72937555	10.23752992	7.940486197
9.430708606	84.80651264	11.94390603	15.46664963	8.747869679	11.99635241	7.052323978	11.18822134
14.85037258	102.5283526	7.107191386	29.68442228	22.11674428	33.33684325	4.756972858	11.91830662
28.87435032	107.0799533	13.35789111	37.30884009	7.108445151	21.4187217	6.93946246	8.994869373
13.14709877	110.5097476	18.36793365	14.79664561	7.916862894	20.4312684	4.783339948	4.770789417
16.96953924	83.56104313	17.8200686	33.21850521	9.264300158	43.79408786	8.93683138	10.07484233
12.40693811	125.5670106	11.51797791	18.52767524	4.234274998	23.36987668	5.561381357	6.845409855
13.39786363	97.67683322	12.93546728	17.18496025	11.30400639	20.37529926	3.431978813	6.029300456
25.32730815	131.0880732	15.57483125	38.35350269	14.51647608	50.22307496	6.005117796	10.25601377

17.02156199	146.5411305	23.81865556	27.50714546	7.625408136	32.82084607	7.632187927	8.478086882
16.20308868	127.0199364	15.88069338	24.17740647	17.53562913	27.50436844	6.16025063	8.821066334
10.03768044	117.606548	9.430969963	16.11443196	9.408324368	17.81048658	2.017144381	7.136194113
14.94276405	99.56036319	15.74473794	21.33924942	8.772551179	21.69580665	9.895341009	10.99309988
44.57427762	184.5447503	17.90949542	70.03485951	11.32274123	82.55038898	5.747332044	16.5552914
6.757396778	94.07752038	10.06403657	11.56246846	7.812622479	28.92269187	5.859612355	7.4575462
20.00599307	104.2054389	22.88922269	25.57049325	21.32903976	47.35689075	8.178756275	8.830336795
13.67913329	86.24709745	14.12188464	20.06516699	5.210679924	17.46533457	12.29297332	12.24193075
11.66452519	108.7316374	21.24974239	22.80594321	3.721086458	28.31384261	11.49082773	13.35127211
13.14080699	113.6019827	17.1701466	12.02163852	8.768971142	19.55316831	4.717732379	7.124014101
38.75401688	134.1892116	21.63934161	46.68280434	35.81584037	33.44564214	5.86461989	10.35577822
11.03479746	118.7203443	11.10251515	19.88252463	10.03216856	25.70927529	4.127931276	12.62903414
16.65324241	128.7144655	14.35618721	21.0695931	5.103522949	26.87176734	2.956856394	3.467718615
25.45615062	124.8402258	20.50787784	55.13679241	25.83789635	58.25910978	14.36382949	25.29006775
6.834046858	87.17680641	11.01583962	12.66535151	3.98661573	16.54479663	5.133778886	4.874281318
14.12524954	99.92304251	19.00785413	47.8118173	18.29376756	63.85015936	10.68906405	16.68917585
2.561344201	56.88713437	4.3240756	4.500978715	1.741970359	39.13257508	1.347659743	2.841766253
13.51325663	134.8272429	17.98818291	23.26921615	26.91973364	44.43692923	6.88305317	8.21932976
10.70048069	113.9981094	14.96713977	18.69685152	12.86062125	24.578615	5.211794391	7.673492755
5.473244336	75.52279749	11.16413799	7.548550587	4.973153715	14.51065944	3.799622778	4.173770927
13.23673329	90.15190093	12.19193085	16.03264193	6.065948433	39.74535535	2.890303194	6.781100011
6.140064124	120.8481656	16.1997484	8.571309046	13.16830445	13.70583486	3.64583179	4.990164385
12.5690763	92.88846405	11.91784085	19.58706995	7.300050663	20.43617981	5.459032123	14.10950825
11.95576365	93.23702037	15.16607836	28.30630304	11.20710291	30.04931772	13.21807457	13.92842189
10.31873233	72.35779315	12.48865113	11.15883009	9.837586519	17.357857	1.748956217	4.568996025
32.60622725	125.8990597	19.53100163	47.82480766	27.50765308	42.93237915	5.7353618	10.55935267
8.277298776	111.8182032	10.74672374	11.09281164	11.48750104	18.81643143	3.346341495	4.418874823
16.42656989	125.7552705	7.896921135	17.70469154	3.999783657	11.79487362	2.706627433	7.105823504
9.252829829	71.21629244	9.667375183	13.05743703	3.18418215	15.30718817	3.409513713	6.334581256
8.729311257	89.05253741	15.12054864	9.519149585	3.830961707	17.07699379	7.121267666	9.623576496
8.907231107	94.86160232	18.46478397	13.33427541	8.178370313	15.75260199	5.457278769	10.68673246
6.483911135	100.8920814	10.52578006	11.40172874	7.490517833	20.48164879	4.485858435	10.80495542
4.546837269	92.9472924	13.54394843	10.21670231	5.91760067	10.39769551	9.313500318	6.882442364
12.03260388	123.485956	17.17501379	35.93409509	25.53636495	44.36897633	6.867841778	9.663652393
18.25983701	99.67128512	14.588943	21.42484841	4.577558438	21.64643067	8.801495844	12.50203247
9.853462731	101.3839726	9.848758809	13.46023668	12.59982553	18.48879967	3.110140047	3.513790499

4.840647228	79.25348865	16.65016908	7.446992779	4.86609119	14.92348831	3.160849188	5.852340253
24.59432303	129.2994977	21.54514857	30.25929456	5.289176585	57.64629902	6.833642646	14.47517567
10.64503832	87.19994423	16.14789343	18.54827756	9.928672139	25.34834887	12.54643782	10.8386135
23.26077534	106.161446	23.98840958	44.16542526	5.369639369	46.02020933	19.33211787	16.75020751
7.203705505	87.80433638	16.30762865	13.39443761	8.496076398	17.80948068	5.572732911	7.374084171
31.55110321	105.8096351	19.12224738	52.19479153	36.96037897	45.68194072	10.81790903	17.20143535
13.66443591	152.6722803	17.3307228	15.71204958	22.66656336	23.86245354	4.088855428	8.775634512
18.50633035	102.3366849	21.22823232	37.81490962	6.570329273	53.92476864	5.524362858	5.371428238
8.409180166	91.58980507	13.76765583	9.97248643	7.165532996	16.78323119	3.578693969	6.835447104
9.616958182	98.50174139	12.95487255	17.09622304	14.51240306	17.92163812	6.263125844	13.97530764
6.940057793	85.30909884	9.362024624	11.59808482	3.877784192	16.00447152	2.46434668	6.435667245
6.81521732	92.68400052	10.43241553	9.818128792	9.566700481	13.96060117	2.850689477	3.098506318
8.320225213	107.3601826	13.45609587	16.96986113	15.82272738	29.8340451	3.12525851	5.203966509
17.37051797	117.6271783	15.10863243	30.50093645	9.161390163	34.44304268	7.493421291	11.91220227
3.483860166	69.99292095	22.11616515	8.180378971	3.491112167	55.77810757	7.759416855	4.745384608
9.451193557	82.65272306	11.83062734	11.81415287	6.686536584	18.49794071	10.21338818	8.772980794
7.966164579	91.31470733	13.50328148	9.380043706	7.158280165	19.32433602	3.558496403	5.516860707
19.31517622	137.3213767	20.59475459	30.34819542	22.89689754	55.61945175	4.902497189	10.16919791
7.221605691	91.11877457	10.54895155	8.768553721	5.765754078	13.78315922	4.266444767	6.957991074
5.423060963	109.8227219	9.315477966	7.360544398	5.17471777	27.01218563	3.934381403	4.674189519
15.08883418	106.327574	14.02718869	18.67598404	6.200281775	24.62098828	3.742703565	7.499672296
19.03860786	155.5452772	19.30490776	27.29157673	37.41030596	53.08100571	6.385912199	15.09269785
7.381177467	95.71029606	12.75524023	17.83835112	5.829390788	21.20235892	8.804916689	16.67548093
18.84017662	104.5728672	16.87061939	28.29861377	7.768502438	37.72589057	12.88467133	19.56465467
24.03974083	121.2150108	16.96222493	35.00806115	37.90560554	70.0375793	7.866729866	13.74074487
11.01284803	123.7149263	14.88335903	13.68828714	6.200838875	24.05097697	8.488097976	10.11135276
15.51722817	140.675494	14.93205492	38.01455115	11.34481227	44.20071935	6.151040943	17.94670509
2.928207434	67.87024018	10.93728539	5.979952412	5.199916317	53.4901163	4.49844155	3.748182037
4.35562139	75.56457971	10.59444456	8.475582108	5.386794241	14.60767342	3.732032915	4.277127035
15.8988966	116.7318079	13.11907825	19.03928777	5.357349061	29.28651541	4.543254205	7.484380751
14.69882387	89.30742024	12.30724429	14.80227625	3.137421567	16.55485013	3.66128562	4.799276467
7.36719176	113.1874402	13.69432969	15.38209745	9.106746161	19.23230577	5.239426074	6.442117145
18.94046455	119.5151008	10.18378589	23.07183311	20.89610667	24.54121348	3.306447004	6.733199348
7.139587724	95.73087763	11.75127209	8.665035839	8.836148673	15.51459299	3.160597292	3.868278204
7.189609425	119.7635871	10.69306987	13.9849458	21.29468888	20.55224689	3.159800077	9.475623797
13.00668598	104.3717751	19.80907101	24.25262593	9.516869145	40.8368252	7.388821823	12.84231376

13.03812499	136.039152	21.72057764	17.30725697	8.440435766	23.83346866	7.79680243	8.329783338
8.302964631	136.5969221	12.74732316	7.856017518	9.484181111	13.68974741	4.032180667	3.268566062
9.826251694	100.6228863	23.07267534	16.94842211	6.47088057	25.23125157	6.821063006	8.159149483
20.52478415	140.8268092	17.46927639	38.63062349	14.84157312	49.6468935	9.96770078	12.20871873
6.029265571	76.36797364	9.632216116	9.691415973	3.625581811	24.97469692	2.765243178	5.934478785
6.152304426	66.04565208	15.61318318	8.021764293	4.784798279	9.16165738	8.821251501	6.157452931
25.63928905	140.5906281	14.59746062	26.8512128	8.73530313	28.87860019	5.424066654	5.718504198
11.61209001	88.20467718	8.887911359	13.33405172	6.929047711	18.03691091	4.520332828	10.52090276
7.059447218	105.4170922	10.39054332	14.5182627	9.114519803	16.86815829	4.665568354	11.03985106
14.0865067	90.75284168	12.11558335	24.26622676	12.36844857	32.59617088	9.195056732	12.60897856
21.3504753	122.8582541	9.85796674	31.01157982	11.225847	29.49082361	6.000414644	6.643780769
12.99864642	117.1973147	11.59344133	17.18400424	6.707153103	23.22703891	5.47123661	10.46004846
14.60014691	138.0257474	17.82274336	29.41331756	10.9843283	35.30261337	5.790507282	11.60465619
10.94471798	125.4973066	14.08509208	16.3199594	8.447697257	27.33615574	3.336837231	6.379018028
11.61109016	59.34239711	12.23957336	16.92951798	4.999635528	21.59483861	3.661675126	5.06233568
11.77997118	112.3818402	20.09199389	14.14757991	6.803039424	15.76967332	7.528431562	10.13487672
13.66817046	83.12625626	14.83018488	15.42403852	2.295933416	24.64521654	4.849894824	8.677990618
7.804263719	137.2070775	15.67554942	10.00887679	6.15521105	20.8000749	4.100714217	7.066547561
9.885568254	100.0945982	10.24286572	11.21857504	11.75707829	14.41311201	2.706392043	3.223043632
12.43637852	98.3655398	14.32400323	18.6370707	9.445531495	30.03974043	6.605548586	13.38708658
7.699619297	96.22905036	13.53074272	14.81880995	8.157460406	21.48548053	4.111533702	7.329123488
13.71965972	104.8056136	18.14695176	29.18960549	10.58925828	31.96384302	9.309710637	18.25925075
11.79638037	145.7723946	16.5321757	19.86583113	3.927162971	21.07362455	3.536877157	6.979171068
22.72741353	143.3152597	21.32947107	31.77417153	21.9017271	50.96349923	5.952389478	12.46780613
13.91923167	98.22601762	16.09818494	13.81533485	4.172616275	16.31693909	9.222457174	13.85684022
32.08283752	157.0317929	21.52170634	40.73298716	25.63173096	29.69633263	9.630424413	12.01939573
6.500820516	93.45539166	15.02923755	15.66767353	7.553915229	22.10854937	6.088470513	10.57282873
15.42284529	110.8145888	13.31847488	19.98034946	9.866977425	20.01463718	3.836300665	3.391241676
6.613409314	98.66223853	12.17724469	7.974063345	7.320112691	13.11483641	3.967249739	5.179044855
9.993653404	108.0238744	15.49983526	14.56286226	5.85118779	18.60422289	6.109212327	7.32828644
18.52148077	109.8799195	16.70221221	26.04723098	15.69379038	41.07210476	3.553368402	7.792830776
13.41110605	83.61678946	15.60734096	24.10640935	5.601827789	40.76089062	3.268487962	7.019967935
3.759342492	69.21796429	12.19013038	7.472579475	4.643531242	12.65302635	4.880972532	5.721362365
8.734333636	84.73990707	17.07623499	14.23597163	8.82399364	18.02995205	4.733844311	8.565270929
13.79771303	95.27150479	15.01659233	11.38605355	1.443761526	31.23574353	9.878604345	10.76918203
7.384566153	109.1217356	15.33581698	12.95315163	6.431979728	32.0229202	6.447492925	12.06529297

9.876041247	73.44624597	14.88786563	11.31962147	6.951955749	13.17090674	5.105036311	7.406474738
13.44296144	138.5633721	11.65057099	15.90698341	18.38068142	23.66337194	2.350526466	6.490819006
21.20635457	119.590865	21.56746797	35.67893722	5.475592964	39.71541088	7.025678405	16.23852622
16.53428313	146.1558761	22.37183721	33.60920345	8.741180498	37.42424095	7.948659597	9.581952216
8.610647981	94.80274727	16.19668645	15.82647258	7.368545036	17.63777147	7.304531823	8.822789866
7.350929802	141.4659298	12.70300677	17.23882806	13.37732571	27.45983507	4.889932174	8.085008752
12.46112548	89.81320132	12.55517608	13.31400723	4.005800126	18.07042213	5.164010374	11.8989936
17.12521167	113.627708	13.01013937	22.01296011	9.724187971	25.03104654	4.634251186	5.391800574
21.32397297	108.5624889	16.89145948	30.2186051	4.584200011	51.35287393	20.3905696	21.76150511
12.51042071	214.5798085	19.76005379	25.28349559	64.50662592	32.06580352	6.980152035	12.0994454
23.86770634	115.3710553	15.45345944	39.98304109	3.676978539	34.08013763	3.767426858	9.172999897
22.0242814	119.4708278	16.96111097	30.91591438	7.426465386	27.21871901	6.088002853	8.463813037
10.24501698	55.96063235	19.62291955	15.09258279	3.981501529	63.17289124	4.67866548	3.590212937
26.69526571	149.7404909	16.62547337	36.74733379	20.87899978	36.83734624	9.450889246	9.897049906
13.54057428	89.13575835	20.23394587	20.23810451	5.947455075	29.93526655	8.596115247	10.73633463
10.36605921	74.90665823	11.11993122	10.53632354	2.132536389	13.64749483	3.618753562	5.973934096
12.0916884	86.41509414	11.45686851	15.52972501	6.668689858	14.74913358	5.798906262	5.950257438
8.332304396	89.42894475	13.25658471	12.01715834	11.0767933	19.47468503	1.981084376	6.814632657
18.0506352	117.5494338	20.82714214	26.28949266	15.47841933	59.06250362	6.671787744	4.702095292
10.36389571	115.2044159	11.18248397	27.14756715	8.145676123	33.40777447	7.020203305	9.724931249
14.93969797	132.54632	15.16733489	17.45196155	7.065884199	21.79608057	4.951513354	5.656486005
9.793079211	89.5023362	16.02831475	13.61335805	10.55390184	16.90000121	5.99638018	6.831140574
51.35750781	122.5480218	43.28581615	42.70815733	4.160106912	54.73344638	19.85342983	10.50344082
7.828218344	87.28569288	11.79903879	11.41291377	9.465129599	20.38525398	1.955873433	6.845122825
15.81083261	77.21787734	6.234741687	22.34257734	1.797981941	35.58231987	4.421855219	6.603958961
20.00339303	118.1329354	17.96580906	37.46747703	5.714822543	46.70923389	10.01089457	7.298993762
28.61010494	108.7086489	18.64349588	53.25325265	6.626356354	44.19658674	6.637331576	11.12252843
10.73966443	138.2131347	21.29155987	14.12064085	12.17240294	23.07374501	4.6836866	9.84219477
11.50512987	106.7526816	10.55946306	11.30625443	5.093251985	20.14918071	4.739287442	10.11971775
6.736972353	86.76584531	11.7498098	9.967946971	5.342466689	15.33669391	4.225869087	4.494621734
48.64312464	140.6402977	25.37311147	60.87191366	9.509865799	55.45229621	11.21520813	19.26236045
16.6176854	117.356044	17.33267635	21.61264414	15.05666695	25.79409403	3.454233111	6.113501699
34.75708685	160.7387571	15.03278141	46.63391943	17.3556378	46.0127044	4.426010011	9.136306465
5.371579385	77.66569367	9.998480622	10.07430534	9.600079243	12.70782503	1.747729475	5.343237018
6.466147071	63.23512689	11.83364166	10.54358027	2.195002063	45.19944816	3.828402319	2.63585431
10.13756486	71.19508142	15.1146024	14.66462749	7.166132227	15.6445357	5.91360331	9.404646099

16.74872345	112.2522568	15.4206017	20.91524556	24.58410331	31.17683142	6.410458019	8.294315525
6.681581018	108.1772906	12.02741862	11.40371576	6.783716968	16.90967053	3.82904348	5.913083121
12.43282201	110.6603144	16.53709444	13.37169514	6.634271749	18.00483632	6.451350118	6.139924219
12.04266469	168.5962245	20.50919212	20.06290383	10.35257741	22.19904901	4.722666966	11.19094474
10.88411441	72.56122885	11.67486005	13.06280469	8.471072201	20.78676367	5.572387832	6.557629417
4.545505826	89.81032022	16.23647828	6.090130217	6.307158852	17.71568045	2.439523934	4.536989428
10.24545572	113.3378674	19.29275831	12.05646045	11.81330402	11.22460437	8.343684358	4.381230868
10.26865524	111.5612901	12.36812783	17.3791557	6.348734288	26.73725553	5.329496203	7.279954404
22.06825254	104.061818	14.82424811	47.21763233	14.53683028	39.345964	6.117025425	12.07847872
5.831175271	80.24418343	12.3931103	10.02518691	4.738274295	11.10239058	3.987156933	5.150170237
9.688372024	83.09929162	15.84461173	14.79172719	10.35088195	25.95473555	6.783254262	9.260011597
8.251239281	77.92640637	11.06206061	10.77009101	7.459730549	13.94535254	2.38563919	4.612253602
16.10931098	134.6371306	16.73425171	22.02455407	7.433680397	29.86526988	5.600993292	11.01556597
10.96290204	120.3167917	12.46613718	12.71522658	20.10097592	27.29586964	4.167892878	7.584515911
9.45456976	101.2564005	11.28650557	14.05561578	4.617005484	41.12503853	2.861476624	8.088069559
14.8682281	88.62444815	13.77544815	21.19958093	3.866030269	56.44180257	5.437601829	6.084754358
9.156999653	84.32744189	9.318804789	13.90762259	6.975516603	19.02163071	4.271464734	6.300808216
8.774497657	103.8530922	11.98508912	12.1155019	12.66385538	19.218194	4.28340761	5.957227681
18.40394767	172.4718772	19.09953437	27.69176953	42.19064861	42.75800735	3.897073299	13.81905014
12.99248716	91.24755782	16.06156879	16.28847387	10.33868379	23.02903644	8.146257357	7.688304176
10.83338681	79.79300859	17.99517598	13.91354741	5.246265025	34.63478883	12.81146544	7.313981853
8.181182839	94.32715489	9.409139326	12.82070164	6.647516354	12.95739669	5.435147067	7.616125069
16.63669394	143.4034674	18.22064029	25.37862907	5.716928058	41.39952763	7.993141352	12.02253597
15.68323679	114.4277301	13.20020246	26.84018118	11.35224182	32.14645597	3.411529352	9.757780875
13.15249086	115.9453168	20.41184399	23.03715252	12.09306018	26.10078324	10.07606579	10.95633117
0.775904606	69.9488383	5.060245816	1.994401532	7.194626688	8.409656298	0.55626128	3.95754827
9.049326477	104.85998	17.32362467	15.65380652	10.07309345	22.65838452	7.544794536	10.15028052
9.347546036	109.6426387	17.05793725	16.17734651	5.266347286	30.08891593	5.946679853	6.44370954
6.606161176	85.16081005	12.32521089	12.42617704	5.605146071	21.53017858	3.388324032	7.606873603
6.235662675	78.49776196	19.01235756	11.17236076	7.161563196	17.3962901	6.452158994	6.893605843
6.787956356	113.1890517	10.62135637	11.61400633	9.191281306	16.29355092	3.243071425	10.42079592
17.37912549	122.1234597	10.62109864	25.21160712	15.80686758	23.5909721	4.119940671	5.858700891
7.676224713	87.53975049	14.50596654	14.50158806	6.24483057	25.89407179	2.920050441	8.282539211
25.79089873	105.8577549	16.76126757	26.23434897	5.717003675	36.89892672	5.347356857	10.02615849
9.567661529	107.26495	13.9625487	14.53607878	5.508167823	21.69924753	5.438272116	9.223696382
34.02987138	103.6427107	16.6844028	54.64225189	7.181467985	45.31767993	13.3583228	12.67623599

10.6084514	101.3188649	15.14657604	14.95619256	3.797878293	26.16199634	4.312908092	7.520802926
16.6955523	122.9258392	29.33557801	22.29927456	10.86574096	31.13117372	9.288527006	10.66112332
8.11386466	108.836571	14.98353969	14.96897865	8.226942338	24.10426959	5.300404656	11.2253378
11.1436728	91.29284131	14.19433132	23.21891627	4.381810669	22.79557319	8.716436809	7.449767912
9.227608595	113.8944122	12.28615109	21.9511267	6.36024291	44.16083104	8.407512729	12.99163358
22.36423728	88.97503741	19.86041714	44.10128959	9.904272071	39.6265116	32.43211987	22.49777361
11.84625221	121.9072618	20.61698496	22.01278726	10.39897831	30.02272034	6.236648847	12.94943047
37.28432293	141.3838208	17.94602321	50.12594785	6.714759701	31.92449723	5.174797373	12.78672033
13.43434869	113.9370343	12.27957375	19.43361622	14.98255352	21.07529755	4.884150457	12.43941702
8.717320079	86.41298567	14.73943836	10.69488936	7.647775369	33.08931723	3.950639199	6.564388013
15.03323227	118.5239212	13.09625127	26.00204561	19.20093576	36.61382619	3.98617524	7.825036807
6.779676681	78.85970562	10.9174319	10.24583465	5.051710489	32.79691206	4.796598406	8.832957217
29.38520602	161.5421111	24.18924259	35.11690802	8.399698517	44.27640207	8.738984342	14.23273501
13.88271381	132.9388022	20.14095909	17.51921979	9.32167047	24.99666584	4.289495851	6.053134344
16.29131272	85.3608321	31.80430492	20.94902985	11.37982205	39.93013942	10.62471775	7.251447154
21.61674448	103.1552011	16.59260457	28.54069622	7.065935445	29.16942805	4.487168575	8.437222198
27.29191047	116.1115292	19.79661216	28.37934036	8.001185819	27.50972607	13.10867758	10.8515377
9.950723744	108.8400539	13.17581727	13.38797911	6.745639495	22.31270654	4.078598562	9.100332698
5.747764702	65.56407607	13.75667304	7.7113691	4.21157751	9.514145099	5.114642283	5.810865494
6.371547626	38.85539915	11.55819696	5.851904637	1.060212458	15.18236867	4.430352015	2.275502202
7.636458812	88.00595095	15.11855928	14.97778612	5.850083356	22.42061899	3.639326315	5.796423283
21.1382462	132.8927556	17.77599327	36.53941422	9.023649142	38.66601286	10.22943231	18.54958105
15.87777583	66.64953729	21.41866055	17.66737541	6.777569087	47.42885594	5.238199917	4.231956674
15.79734904	109.7517524	26.32095934	23.9856512	11.25154162	25.98757298	8.858184529	7.571137388
5.009518825	109.0503682	10.60695982	11.72878084	1.82027274	28.09312326	3.192492005	4.520290209
14.9646594	78.46144257	12.7221827	12.37179264	1.717352161	16.44899472	5.498882242	6.049037936
15.9478775	129.8895002	15.52082599	26.44879396	24.64115103	46.7166005	5.274167179	15.92440558
8.699316303	101.1058762	9.984517573	13.93677707	10.24353172	16.19688328	2.612789614	6.070222553
6.233050119	83.70229103	12.87198765	11.7879867	9.33376388	22.67312898	4.103501021	3.887190024
5.246513324	88.52466427	12.53270083	7.088467072	5.491184212	13.9140632	2.857868551	4.692852133
8.603472367	132.1616878	15.80289415	21.78575899	9.840760594	34.43160415	6.735221868	11.61342572
26.29573045	138.6450393	21.59482525	38.5963415	19.62947964	42.55708367	8.781024225	10.17734439
16.13017495	124.8960336	17.16596528	24.29989333	7.476438455	25.28395486	4.256140699	12.5210833
16.38225343	91.83427372	9.601240951	36.54856747	5.685455791	33.5099397	11.23294432	21.93153459
13.4508518	106.7136048	13.69645541	17.41667062	10.29725602	18.05403363	6.226500455	7.906901259
7.439696603	100.0004846	13.34621839	10.08419221	3.75298849	14.70463144	3.297403089	4.33843312

20.62055958	128.235118	20.7683907	28.80985197	8.092189276	24.74540713	12.74458649	12.78323329
9.563276742	74.43606778	10.67831272	15.0164923	4.56349724	17.20765305	5.739356344	7.82028446
8.264229841	141.3096414	16.90850207	13.566238	16.93314036	15.86979109	4.762864963	8.616490835
16.57530658	123.5349121	19.46440376	32.7324698	17.1911611	47.12342838	6.487812129	15.22014434
14.14628489	110.0637912	16.9244933	18.95333547	11.20014115	24.6396888	6.415030156	7.42343816
42.18743177	147.5448421	19.02171414	56.22588072	6.589574349	46.29513122	7.757666613	7.690426631
25.38001861	96.14146331	18.46233963	42.3892408	9.422628707	30.91192488	7.031192397	12.04515541
14.45894804	128.3069703	12.29966396	32.95003346	4.348416836	33.32503186	9.789070035	10.90806138
10.63170594	108.3679883	17.26231143	18.35076901	10.53160906	23.04770184	3.460913944	9.735503007
29.03649167	177.1352637	18.77880784	63.50891092	11.92973482	68.30844269	10.9271435	9.104231658
9.227605091	98.73012292	13.38577421	14.69870728	10.7911175	20.42998288	2.355738946	7.79138496
20.79371954	111.6975091	11.19264703	28.81921812	13.70520906	20.24816063	4.138748776	6.087248453
17.15992318	146.3145149	16.19009684	16.12568148	8.624269953	19.03658506	4.796305876	5.378144335
32.658123	131.7769638	15.15176812	49.00164191	19.51907012	33.15388619	12.89860697	13.52934906
12.38169218	102.4979814	14.45580705	16.10832392	17.69820975	20.03939355	4.10145197	4.70685817
12.45562372	129.7619521	13.59904006	21.70250138	7.01783	37.80886814	10.83690643	12.18311275
13.1445897	128.5985312	13.16388335	20.07042323	8.2641833	25.10034435	3.401303155	7.86297577
14.24984644	88.58517475	15.84205629	19.59494838	7.489456948	19.61496793	5.932741515	8.712899761
13.10275813	89.51372549	19.20883878	17.71203345	11.86917732	26.053487	7.357722854	7.18800576
10.24853667	83.33491484	19.22766313	19.56355921	9.396579432	32.62815871	7.446005552	7.678059445
7.626836578	80.41135658	17.09010395	10.70469368	4.429201643	14.00727768	6.179559973	9.510307016
10.5534645	98.81797766	11.2130682	15.75457424	7.99414918	17.61004356	3.718834875	6.352342333
9.177416674	88.59476323	8.704842559	7.398817799	7.057614087	13.5658134	2.597148448	5.747087573
19.26758259	100.8035144	11.65804743	22.29109577	6.198783519	19.7562475	3.291265276	5.751777964
13.21278858	88.84485098	13.2147806	22.24319033	11.49893367	19.87933665	10.6841459	12.43606265
12.75495586	104.9652079	17.17793776	18.18015348	10.41060128	28.70519454	4.096416228	9.741089082
6.810940775	72.41392219	13.66547151	9.695138667	3.850808619	13.34380886	3.830021851	6.135977641
6.781535594	69.17963492	14.10756025	11.6634633	5.022604113	13.47240004	4.335367593	8.696977839
10.27040385	85.77158444	10.76076155	9.505985182	7.895853363	15.41819526	2.094017266	2.485750462
25.08914098	146.624452	16.17543771	24.27820416	11.3515396	37.57062364	4.680594082	11.56449982
12.92068367	116.6025769	30.56047044	22.47528199	7.780507146	45.36557865	6.168767318	8.44785111
5.112055998	77.5194456	5.659867036	6.80391373	4.931488613	22.95494283	4.021981322	5.466791834
11.51530582	104.4304606	14.34869162	16.41064225	6.092028287	16.13036634	6.01277883	7.439259225
27.61912605	178.972396	14.83235404	37.40887321	18.18208633	32.65019697	5.592410992	6.506586455
11.04407551	95.93320135	12.07989576	16.73362256	5.031201879	20.50303203	7.847122585	9.26733059
28.34396671	110.5121536	15.07448501	37.61892634	21.97095873	22.91467513	8.992377322	8.131291315

10.26774376	102.7341375	8.812698479	15.64612063	4.006857618	18.34033529	2.770379272	5.552622761
12.15842149	108.790983	22.32816969	24.00784198	5.526365653	33.54257605	4.733604674	8.205422555
10.09662389	92.42620306	15.36905601	18.71438202	6.408685617	19.14281456	9.531305909	8.078207967
8.015421325	71.17242753	10.20232578	12.44569357	6.958734831	22.15700271	2.99433024	11.46598148
10.20864911	101.4884976	21.76153908	13.88218655	5.386399986	16.62301749	10.71592927	4.60523987
12.62490676	107.3246483	18.645832	18.2096506	11.87673203	23.72279157	6.259096397	8.145920872
13.98398213	107.8898999	14.77322126	25.96112834	6.813768986	39.51663987	10.29136794	17.9046644
8.345689517	84.00437718	19.94888168	12.37513299	7.620042199	32.68396963	4.170769663	5.59610556
18.07185041	143.3440321	32.24417771	44.76338278	10.05334374	110.4156625	19.06337348	11.35908494
7.507438095	70.11524263	11.63501368	11.62681533	4.956835609	15.04412998	2.839287119	6.048275747
12.86037882	130.7504799	22.05946307	20.9960176	11.56344807	41.54235582	8.005681511	12.50453792
22.68568311	122.4968592	14.5401344	51.07945403	7.962657077	49.56177057	16.90440711	25.24808599
14.12994717	112.4576803	24.33076523	28.4826226	5.677607109	63.69425444	9.065626123	13.77184568
13.85996428	99.52918449	9.6797997	17.14641824	4.010687181	33.09941081	5.036120038	8.022022974
7.316172783	59.9817356	6.12776393	13.96717025	1.954323929	24.38114866	7.570294079	16.13631824
9.699839903	99.21126097	12.03144387	16.81211275	3.160842337	42.35954114	4.6737109	9.241068356
11.15201978	82.11633837	16.83072889	17.08481391	9.839328328	22.51994209	6.508469685	7.965545648
14.13011132	102.3269652	18.88937921	22.19933111	9.178036435	42.72754122	9.826090626	16.41881799
12.65359052	106.0559423	14.73468646	14.70232179	2.7041272	31.88204827	5.34250329	5.590420409
22.20382281	141.0751548	27.23683414	48.16688296	13.88141126	45.33200469	12.01965739	16.57727072
7.237538036	71.12791093	17.47775276	11.12904319	3.876234069	23.01773173	11.1376706	11.23535303
6.531451972	60.52577477	7.21986812	9.389591067	3.236578331	10.75340298	6.399948866	10.22757001
9.012336911	110.8847765	13.98019486	12.54114137	8.451317986	21.33178314	7.892897522	12.54946184
8.867004912	82.03858565	17.3424842	12.52440317	5.744216411	19.28942648	4.509306662	6.073372256
13.59039628	105.6829849	21.967647	21.14629363	6.040913347	39.93274759	7.703483111	9.101618706
18.31023848	101.2748483	11.33777768	18.18410877	5.147161348	19.95845824	4.447685982	9.052845313
42.06699679	163.2961148	25.02109178	101.3260618	11.5914487	173.888357	19.00701302	12.82415924
22.05227411	107.8483609	10.91057978	26.85983647	5.119605185	22.18894432	4.549545601	10.92969096
12.16098151	103.8570855	17.99677406	15.81404589	3.944786426	20.82926698	7.008573632	13.65065554
16.56356254	101.0516253	9.86492981	37.15226362	7.568665118	42.10033486	14.74986879	28.15730785
12.29780211	108.9567323	10.63468622	19.13319419	14.1077443	20.26752144	9.343168502	10.4451448
17.01957439	79.03286836	28.87859908	29.61237921	3.670358696	105.4076934	9.311213757	13.08216146
5.881365852	79.04580708	8.667215409	8.28701832	4.51110863	11.12586458	3.226979019	8.940683919
6.005323816	90.18530374	8.351353989	7.532747904	4.337583389	19.99610939	3.756624426	7.977798884
18.85496454	92.36854764	21.19033627	53.96960853	6.242242055	108.6963576	20.26342406	21.49741306
12.66506126	87.38041162	11.66491128	16.18400293	3.230901024	22.86699941	4.049233626	9.055102546

12.13393402	80.97534206	10.04389871	19.08611065	4.354134029	26.76324589	5.17116098	6.958021829
4.305185635	70.93200859	9.103109422	6.309443508	3.322674401	10.58866358	4.380258966	6.122984195
6.110748252	99.63292366	16.33677496	10.0043841	10.0312652	25.25961048	5.977051694	9.061315415
17.02799587	95.61201929	14.93880765	32.95762141	7.610371415	55.44463921	3.098537031	8.054596797
3.837785638	118.0285547	9.707294865	7.631322039	7.278579326	14.14886053	2.784154623	5.708068383
23.25424653	163.5779418	16.47525656	41.75894052	24.63797694	56.01367362	4.25680251	14.16230694
7.933314403	77.35338755	8.586005786	13.78585341	6.549989599	20.17760077	4.872588651	8.122342819
25.96522479	98.79689654	7.950643085	30.97466464	5.045345843	42.60461004	4.250480975	10.01708748
7.248042905	80.31941341	10.51919005	8.606476827	4.982330427	15.29636591	5.207721297	7.389453275
12.89928005	123.5529052	14.686292	21.27353095	9.503107656	26.2419535	3.631720103	9.487460331
15.9573143	173.3794249	14.80680582	27.48549195	7.914696929	41.3206498	5.55144031	9.199304922
9.429767926	82.27424405	5.71669187	13.70223001	2.3271372	28.62183887	3.636816238	11.75582517
23.93541589	87.70107326	8.789859886	48.1133119	5.358592671	58.37150878	5.794328838	17.92718469
15.14657437	107.1725704	17.12141026	26.29199123	7.497015027	51.3057573	4.368442679	6.337301627
12.74229114	108.7945477	8.222732967	23.16664283	7.585799956	37.71983155	3.070907148	11.30752643
11.46297747	80.74413384	12.15575228	17.43229642	2.539077521	22.62452992	4.654050128	7.046981731
12.57628361	92.12166864	20.9111384	19.43697276	10.92648594	23.83697574	6.450243445	8.589828538
14.87872377	139.5369182	12.33921908	29.93573211	8.175604846	25.16084804	12.74700872	26.35429777
10.04840834	103.158024	18.88346525	17.95464606	5.871386281	38.60206228	7.11160122	9.619761014
13.40910684	58.97412951	16.12597101	16.58307851	4.975815357	53.28829849	3.393341832	4.511771819
18.92848798	74.47773025	9.73651335	21.60672589	2.562500946	29.56484616	4.168211897	9.000282886
7.484315754	103.2755	9.271119727	8.846710545	4.401281413	17.01296816	3.801114589	8.822780499
13.15726266	102.4823417	10.85547918	23.89019623	3.247521561	21.39324878	9.874001793	14.30165489
7.744495669	87.34557053	12.2340728	10.98132078	5.056643724	32.41922716	2.773647102	8.955699058
11.55420274	93.21836113	16.95033656	21.86973953	7.193559989	43.86410239	6.142693599	11.01966879
12.20321129	103.4158225	16.05159754	17.19590387	12.37101682	20.87125692	8.290693672	9.078017646
31.10496394	136.8578493	13.57813565	45.2308359	17.03934673	33.56982495	3.478553767	11.23104846
6.324426151	80.66870569	10.56508846	10.10492836	3.797262389	16.06347016	2.659351314	4.27484236
4.482333854	73.38472712	6.982449586	4.840093312	1.87076208	11.01633877	1.909496665	3.549745511
19.05410452	100.2387665	7.984026612	21.68947103	7.613608021	35.85003902	2.246826621	4.468707615
7.617372135	107.1337809	22.33102324	16.1062891	7.663878892	41.39130743	9.565437107	15.02020035
8.862203166	92.49071689	14.72405977	12.89378456	1.873049013	28.88280374	5.464253986	8.234126618
7.828742993	61.87331368	9.152884567	7.702744345	2.833798573	9.143234685	3.849019645	5.398467289
22.53618675	137.8256004	36.68742409	69.43302901	28.84929974	101.0590766	20.24621893	26.5926023
8.47957363	88.1459682	17.72319566	12.82029936	5.306394055	23.16319088	4.91966486	8.157214307
8.537504553	96.97058657	7.884330504	13.67496197	4.386083256	14.98104767	5.353833302	5.801844601

10.7313452	93.63103965	20.91567716	19.03569078	5.700150753	40.5859157	5.046624945	8.394647194
62.79915197	91.53599779	11.59334061	56.04359611	3.427015588	87.37856119	6.844857709	14.79820916
12.30611851	165.0011797	15.65828519	19.14563266	11.63204591	30.48042512	2.761797346	9.76756912
8.828680053	121.5644587	18.92944343	17.54788058	5.014914305	24.35818354	6.806886488	9.805075294
6.909029604	91.7523436	21.58619568	10.88909125	6.153660192	21.36796525	7.743025156	8.778206793
13.43391817	98.13253484	12.05203051	22.21910229	10.90753114	18.61060926	4.444243963	8.206395656
15.83759955	76.27572113	11.08077124	31.82155116	6.832689139	39.512629	24.8576089	23.40554419
6.480372064	72.34913504	11.2421499	9.473768547	4.621524118	16.62507998	6.477845778	7.582334751
5.181528315	112.8021897	12.67813502	8.595535264	7.7747943	13.80640894	2.936190232	6.100747695
14.13108829	110.8329775	20.51871556	24.21909316	8.345927086	39.20631237	8.788554192	15.24626743
11.73633196	86.50413184	24.36565509	19.04362369	7.551026478	44.26669591	10.49360387	9.306226443
16.80778485	97.5638917	6.91914721	13.97308646	3.303277359	16.61874287	3.45345845	6.006185342
49.68545276	87.2298167	17.40992427	65.42842505	4.655993082	99.01328507	14.02305483	21.31630214
14.86951733	108.5245468	8.662796443	18.79711224	5.829329872	16.52839482	5.789889883	8.740073804
4.0356639	67.30796657	14.8542285	8.084620608	2.470106059	38.3406565	2.491769372	3.721392133
5.392093634	68.17300242	6.877893943	7.403333806	3.357075641	9.964427325	1.987860098	4.065989266
3.797187888	86.5401176	14.98455215	8.194680567	2.756982036	26.22402306	6.992167371	11.96029831
37.11206313	83.39330457	7.165528913	52.05538978	5.370155829	45.12269315	9.88201945	20.32602757
13.58063126	95.57787765	17.0175904	25.45761636	9.604765309	33.36227943	6.971711979	10.12484819
7.099849305	82.64341059	10.89365729	11.97203088	6.465685241	17.37108954	4.092545999	7.572991608
13.99243588	146.3557979	19.46480453	23.86653502	4.312890233	35.36525268	5.102006656	10.92196789
10.42087364	97.43238182	24.7866935	15.7357227	6.573544036	31.09234203	12.23007016	17.05972042
9.607974642	103.4207558	10.79220387	11.71407135	5.740674139	22.63594523	4.853152287	7.655212355
11.21502114	81.08297681	11.10843087	18.65950046	4.449375725	26.6671405	5.139176971	9.501177786
10.96302521	120.4009133	8.278432077	15.8085612	4.912119631	21.58204185	9.47567941	8.858793825
6.905809452	77.95235918	17.40327045	9.239778266	4.84982142	15.4752777	4.677502846	7.066531281
18.96361144	116.1811277	14.09011405	26.30774879	11.12957147	27.26563145	5.800962176	14.00214762
12.16490308	150.5056942	18.0193593	21.17759513	16.12696863	28.34258293	8.477322434	22.69567719
12.28490888	110.0683828	17.94625724	15.9324551	9.520201955	20.35675211	3.450385699	6.614430203
15.78325516	129.0489944	13.69749275	24.05448137	8.139612604	27.80956451	5.0742327	9.868639524
11.40394605	110.4868581	5.207952699	13.57050041	3.039552886	11.62198018	2.506719061	10.88492107
13.43060136	102.7667456	12.1056803	15.14664622	5.326976735	25.65862154	4.096301162	6.178101128
10.47236648	66.70241719	14.01876201	12.94936997	3.616510305	29.03695356	4.085394197	4.033656305
15.29341573	124.8545027	18.99957354	23.03340794	9.33014151	27.21307461	6.188157541	10.25332581

Ipatasertib_1924	GDC0810_1925	GNE-317_1926	GSK2578215A_1927	I-BRD9_1928	Telomerase Inhibitor IX_1930	MIRA-1_1931
45.22650969	133.582662	1.082487201	157.9743459	93.59669557	2.636409684	307.152616
27.56995308	145.7212328	1.333012089	122.2026499	79.74604011	1.656657637	196.9820199
78.88119514	292.4363584	4.263375336	186.9532821	93.76073561	2.516798618	287.1617127
31.86713064	157.0837147	2.860240846	97.59779659	121.5791034	1.373001143	212.3332268
105.9667613	271.3665068	5.089657196	165.9316603	169.1001552	3.585068407	379.4498818
24.94497346	117.5685079	1.435610592	116.75032	40.46823885	1.654006614	157.0689858
46.64785225	154.035992	1.426910126	150.7889886	73.63662409	1.78720149	260.0560062
49.67896774	151.5933414	2.940427497	157.7721445	113.5618541	2.497383651	417.2798901
99.97779096	267.7311975	3.539135295	219.9516609	204.5387935	4.365741997	489.7957408
21.280173	107.8605947	1.406422785	96.79067349	44.8074114	1.254855162	141.8350114
30.27975859	128.1644658	1.856192565	120.1677036	56.25536959	1.385263997	166.0455109
35.84421425	149.292851	1.894794085	154.6626118	84.8007607	1.907648858	303.6010729
21.34941105	137.7645575	1.324416901	112.9317454	68.52934094	1.427370993	152.7925358
79.47133381	176.8597032	5.107502412	141.7894298	125.8658422	2.17639282	289.5346201
46.8899875	189.0474224	2.464560197	136.2483021	78.12311909	2.024058523	176.8424291
25.30844443	168.7378627	1.819739592	149.5769458	74.68852912	1.700601967	216.4409186
39.17940824	66.26738379	1.920641073	60.72294025	31.85327022	0.565155269	80.44674977
75.3141779	162.547141	2.955933056	199.2061151	152.3433458	2.479860512	454.0944448
75.08156025	210.0820654	3.484618901	172.3561158	99.90624373	1.591862511	227.0871111
32.7461541	111.3903158	1.166309101	115.2741744	44.04878182	0.767040448	128.2934252
31.4149111	139.1180893	2.014866091	147.0138939	66.93389165	1.200696279	221.1617173
69.07375541	233.8831761	3.472409665	224.6427459	112.621004	1.874514	361.1507415
27.64440133	173.8950158	1.317085102	170.3358007	71.98503016	1.521976606	257.1626189
37.76514555	267.9749026	2.190452981	192.6744224	170.5895178	3.466974802	522.3031263
38.75125016	176.9494662	1.570633284	161.7112082	96.0696468	1.52393687	233.6990082
23.11290177	138.1658464	1.148114229	121.5983525	80.75045545	1.172402924	270.2081917
104.0601752	242.3638738	5.568436717	182.636411	130.6825406	3.867399837	375.1276194
73.47581386	285.558612	2.25728865	216.3672467	161.5405077	2.329038953	482.8885537
34.56617618	170.7363634	1.761176409	156.9510798	74.29571772	1.228245265	230.3025849
22.68556289	109.4466855	2.284565036	117.8055227	63.04569041	1.502552206	212.3863323
18.85224168	137.1239812	1.572452778	147.2573608	47.01739437	1.378307382	222.3847415
27.46221647	158.0979656	1.378270091	193.4466311	75.42098485	2.503817141	286.9810996
39.19618636	232.1376963	2.181407896	244.202365	138.1125693	2.532351596	488.0048489
43.67835565	74.55485065	2.537086962	87.9778218	64.41020036	1.496425743	180.2904459

39.63071566	191.6025665	2.937242445	150.5264088	96.49003672	2.929202199	335.4334532
38.79653081	222.8609229	2.256923075	203.1070905	98.22751433	2.131603203	417.6870026
24.04747847	90.42623244	1.455893607	92.65557573	47.04390855	1.204275006	109.7081557
54.82184097	250.3438451	2.329486614	196.8341338	123.0909209	2.554233228	575.9431119
27.91457129	162.5325017	1.721328893	148.8855366	88.50133621	2.848941457	227.165226
27.25397191	134.5177866	2.379995632	93.55519271	68.82650194	0.704427662	140.2074885
21.50458054	87.92712561	1.238412753	108.5243488	104.2264892	1.609629848	253.0815621
34.49707717	218.5175883	1.68374576	138.2968353	99.03664754	2.098317119	290.0949268
32.87513998	140.8218383	0.959072861	125.0894768	95.74585818	1.836747229	308.7939847
24.57559505	173.0685244	2.325976059	126.7466465	75.00462729	2.201786272	193.9372469
22.66277329	128.7984321	1.034707508	117.0712071	80.45020953	1.259960763	328.8924603
4.763488944	34.55686632	1.85086874	36.14518294	17.09160453	0.417995739	54.80191477
32.10730924	156.8814057	1.31392871	109.0631883	64.42042394	1.23809758	151.444996
26.64123615	176.4801119	2.31139116	146.927153	87.64834061	1.331525311	331.4449006
38.63632908	148.3989968	1.67877469	102.6580338	67.4980669	1.266184452	155.1175248
27.40565928	153.7499009	1.618293543	118.2630239	64.48856487	1.208111698	191.4385368
18.16864232	90.15248299	0.983747142	85.39012065	38.38555176	0.75953738	79.96442769
35.6325074	195.2282177	1.82451882	155.3263859	83.47328308	1.48997201	221.1206027
43.13226119	197.9913099	2.743034345	170.4311334	64.1690695	1.973593283	247.8047677
31.70424119	144.6146335	2.367255257	150.9353839	59.22018047	0.995882579	144.18476
44.26618796	97.0762095	1.503014529	106.151004	98.32764571	1.292422011	297.7805927
93.00805383	165.4368191	3.660197503	135.0679688	138.2763117	2.895742153	431.1412841
15.04322644	99.23828218	1.534637611	108.3633649	36.31962228	1.144966516	131.9806259
28.3011704	115.3706856	1.279151064	102.1921618	56.63937862	1.08587568	126.1685908
50.34521419	152.658151	1.534654801	190.7722404	151.4556348	3.599533302	572.1970973
31.08708069	178.4659765	1.58948824	136.8614064	108.1031757	1.120553667	264.0847062
78.93710591	205.1260229	2.940095285	241.4994795	159.5742774	4.787331095	461.9759995
38.97329105	186.2973599	1.969615504	194.2209801	73.81233171	1.57525283	258.4084847
79.34563686	282.2425229	3.790206945	193.4234773	128.8542601	2.425080472	430.1524248
41.66328333	93.77034911	2.104152435	134.2116839	80.46266799	0.932239959	258.3574115
32.83032408	143.8272915	1.102045338	117.8118161	73.43455454	1.557341151	153.1558961
25.56227486	110.4700685	0.983091359	102.6031897	61.87002925	1.21158664	141.9283728
76.32082659	143.7238053	2.272011965	196.3622083	87.70663291	1.479630317	347.6859559
142.9869351	255.2406939	3.596509256	208.7647726	313.0423437	8.371300478	854.5857582
33.31909616	166.9615772	2.606702585	164.9377199	76.71731329	1.704424054	255.826467
7.644610385	72.52029566	1.027128517	96.54966234	39.07842544	1.984692948	116.9149071

35.00338955	205.4107375	2.351505959	172.4782324	81.11459389	1.750872903	330.859926
8.955420894	91.5686095	2.45154334	91.46476297	39.14073706	1.269516339	147.8398543
21.21573834	228.2366306	2.247330728	194.4043753	105.1043972	2.048892013	460.2953132
187.410056	361.2598106	5.769535147	245.6091428	279.3883049	3.942630048	632.9855072
47.0089613	135.5849498	2.117011659	132.9347187	77.36607218	1.558957156	214.8313762
32.75391903	151.0721437	1.030930753	122.7783312	73.0898951	1.436319307	177.0879161
36.35883039	72.41810531	1.585300189	80.04683764	53.50455161	1.049497014	125.6574476
22.96016947	160.1688014	1.046688212	156.6658815	90.04687552	1.691969039	366.8951767
33.4039475	112.3195018	2.25172771	99.24981145	64.85811727	1.357110788	153.2498365
26.14771025	116.3516702	2.242968883	107.5404377	63.25673909	1.7056285	188.8377711
127.5956046	140.4171006	3.443577024	140.3128084	184.659123	1.960990021	461.7448723
49.57046927	235.4239837	2.714486874	179.581929	181.1206369	2.824772609	451.7076841
24.83757794	124.234035	1.013431775	107.3717658	46.87615141	0.948363425	141.8657121
27.60133727	99.68011433	0.905319844	102.5474675	68.63087626	1.072290213	193.7425741
62.30004491	174.4954094	2.754904803	142.2980811	107.6113184	1.726735608	323.1413256
28.32443636	181.5950145	2.131494554	168.7336698	92.5165191	2.557379658	265.8248549
20.58549486	83.67597516	2.247302631	86.50921085	36.00703093	0.763290408	122.0240877
27.99593144	121.1368891	1.45377085	129.2055759	72.72796666	2.632804051	182.3729053
79.16993828	217.0523054	3.751533756	164.302056	125.0590862	1.690089016	385.8499327
36.41858497	126.6829448	1.465389332	147.3169951	56.8671388	0.896237303	164.7644184
43.09835696	154.3275336	2.299955243	172.8593345	92.73776368	2.344247872	210.9978382
17.87660685	108.1929051	1.309494798	117.1565588	42.13926014	1.097948322	160.3515885
58.44408159	153.8921826	1.79919163	143.6968166	104.6189056	2.453001361	305.164959
34.75579141	145.5044596	2.067427331	137.5161213	68.13470246	1.559586415	196.0328084
14.60051076	120.7593282	1.172735678	137.9253846	44.18115783	1.004408347	160.0655425
30.70570893	128.029061	1.739111661	119.9015206	86.35112807	1.475833146	333.5929436
23.54751234	111.5744071	1.097428507	108.3835787	56.95901343	1.116219384	154.5281954
38.46059273	145.6234106	1.247394709	137.2753263	85.54799209	1.147091487	342.3113145
52.07498389	155.9436161	1.785475736	141.3140257	122.7843888	2.665240869	240.6513515
73.08702907	173.6201234	4.561089621	114.9480413	105.1544246	2.376907657	202.1493743
38.33326217	134.3736816	2.184851803	158.2845289	96.43019829	1.418292268	261.2952903
38.97756192	203.1768956	1.715561951	149.7474464	114.0780654	2.509740417	277.5646496
9.134434052	42.85160445	1.28945945	45.78476441	20.45814645	0.426060438	53.44658207
28.90462671	118.2886929	1.503779249	98.21358787	59.11154219	1.802592422	183.247899
67.24174619	89.60398462	2.457605485	110.9793138	113.9089839	1.445031927	193.8936614
54.79734428	229.9496719	1.755105282	182.7918899	104.1359371	2.191610296	327.0637148

17.14533409	168.3463377	1.282809769	128.9602744	88.16505379	2.086008641	230.5355014
23.27733291	166.6579548	1.923152117	107.3242308	88.95579024	2.355012742	223.1584286
19.02753819	135.7557825	1.992148248	111.8866688	49.5772414	1.279312185	176.9090214
76.66749517	253.1610047	2.215353338	302.4140458	144.9170981	3.402079801	494.2360904
35.06026463	156.1879661	1.62920456	153.6374588	102.3432316	2.246600828	299.3365225
48.55415406	108.1006839	1.249450213	131.1024605	78.93045672	1.440211255	234.0152477
108.5788116	268.8899292	3.951691344	275.1139697	151.5825117	4.4445477	580.3723027
24.63966109	123.5393691	1.361549014	143.4747543	57.67051346	1.354006906	169.8692743
51.57961054	159.732347	2.456360882	194.3840527	164.0407158	3.189363085	447.9445671
37.21141081	140.2277801	2.076798809	149.4834151	86.77082785	1.950136721	174.6270456
52.468362	138.3974706	2.384593577	129.7532291	65.36443561	1.443721684	181.7428614
50.45828108	123.3513668	2.454477091	136.6647454	123.251977	2.25201018	399.7362811
61.34958873	288.9221742	2.288440347	215.1577493	150.5502582	2.202799111	449.5165829
31.51955346	128.7624328	1.775735547	103.0458534	47.89026911	1.341905715	143.1466705
15.18997166	67.12447332	2.725473268	76.54146201	40.768658	0.558704197	115.1224159
37.85046826	158.6995216	1.939206154	167.0997958	82.80746591	3.132112449	273.9867005
15.14138423	124.8731008	1.318025274	151.8218863	76.39557708	1.004446036	187.5503098
25.976699	134.6197517	1.250459449	139.9979682	84.34038344	1.598294647	205.7659408
40.1535972	147.0018196	3.492324487	123.5260125	57.02202498	1.001244719	135.481272
13.59912925	257.4956405	0.872451446	206.2042569	75.05561786	2.63547271	345.3536832
70.85330136	148.3209535	2.025712703	153.6239242	127.8104849	3.622133578	388.6962276
26.07054498	156.4023603	1.492373019	137.8506169	56.24190874	1.377192439	238.1509709
18.18566593	124.9926444	1.02575836	135.7900777	50.34499639	1.129527043	187.9497739
39.56037598	120.4522206	2.023320522	190.5984259	53.32975434	1.222144975	257.7415906
23.26010019	145.6547731	2.243974281	105.337043	72.67584514	1.565864504	141.1400498
36.23728726	131.5154207	1.140241549	157.9533514	86.1074496	1.17411124	301.6872948
132.8709302	268.712295	4.438134958	228.9992493	296.5333073	8.117827615	577.609649
35.17560662	110.7571441	1.317191233	107.8038699	91.58914693	1.183087464	254.2058669
32.73005254	126.1901667	1.84147267	121.2671183	80.32372199	1.715532028	248.45945
65.13301045	179.8300291	4.186236212	153.5313553	113.3808419	3.344750403	370.6532414
55.27888618	208.1769872	2.736785371	159.5575508	80.23083644	1.269488716	260.1491022
26.18022015	173.8217103	1.735857178	142.88305	99.47528131	1.571424325	249.4675386
59.63127986	111.6946575	2.19474111	112.4476397	71.62664207	1.216243391	108.1151455
43.17216745	154.9664923	1.600192791	176.0930425	86.00884515	2.717820832	202.8574193
32.45116471	193.7684425	2.294166779	157.7591178	78.16731942	1.393369138	212.7661727
65.73191357	266.0709731	2.264194751	190.4172006	139.0883895	2.364567714	367.1913377

49.18984919	257.9426022	1.523587686	252.8268447	121.8963767	1.917603618	417.5329526
45.40617701	239.3360951	1.981378706	187.2685456	106.3578339	2.233816172	332.6898179
26.70553559	163.7373419	1.550377509	134.1551567	112.3531683	1.849916651	302.5304295
44.36874047	167.1833501	1.192465259	144.8875474	96.56502406	2.099471436	302.6892241
88.54903163	219.2050866	2.701575805	339.597798	285.4916182	2.19062947	645.5090014
19.11791577	91.50396494	1.640050041	92.38593923	63.77630766	1.547773348	177.2924695
43.98534087	137.5955052	2.595724398	128.859863	89.97084081	1.821714385	247.465433
48.13637084	165.5130482	1.319420618	120.120683	98.27871101	1.349932846	222.4729029
41.00722338	185.1624152	1.390033014	156.3303038	103.0383209	3.103253446	248.207165
19.22888919	125.96151	1.784774392	132.4618601	65.98083092	2.380639937	191.2658596
74.66245108	230.276049	3.253860619	165.3716039	108.5252181	3.870644058	280.4090368
31.69678546	169.1314307	1.309608837	160.3252164	77.91057494	2.048788403	353.7232491
28.09588024	156.9393105	1.33211512	132.0282554	66.55825279	0.931850365	169.4883409
67.86193043	135.111251	2.670428683	143.2218558	114.8338539	2.426443178	242.6293459
23.28946133	131.8441603	1.042307286	149.3773112	55.74466289	1.768230652	168.4656321
41.03340912	132.3556032	3.607610275	155.678421	112.7103564	2.899156508	224.7243031
8.183733056	39.8092031	0.786125057	64.14357721	15.64618279	0.417263428	92.34500115
34.06052875	153.4415751	2.872921333	179.4614473	104.7756321	2.548591489	322.2882422
33.43527794	158.7918811	2.207648845	154.7945709	79.11231358	1.845200658	237.1348374
15.103876	117.0060485	0.819992815	111.7604931	49.89044667	0.898696766	158.556018
40.35226413	111.7433229	1.854126144	108.3773379	69.96580296	0.922514612	228.8006189
13.65225074	133.3377169	1.239985738	153.7609197	52.6681647	1.530216086	218.9100185
32.21790687	182.8964374	1.500226918	153.6510042	98.8737642	2.291876784	211.3166108
49.86418132	127.2767616	1.669855621	149.0341298	97.5292969	1.630918028	281.9955721
20.92449678	114.4710682	1.675306572	93.73567177	55.9089397	1.527641835	180.8198113
64.01468637	165.1009553	3.199892006	163.0455697	127.3077481	2.882673431	384.3750167
21.98524076	123.2112508	2.887713087	108.320965	59.23698364	1.218750537	169.5928842
49.72863041	152.9629149	1.42602397	201.9791033	105.5761796	4.088334542	338.8257701
25.93203644	99.63482282	1.638130366	109.7899787	52.40503654	1.604957122	179.1038924
22.44916818	113.4379884	0.908761597	133.4749745	69.99157854	1.383025782	208.3830608
25.51138414	119.4169938	1.031427256	129.4239605	62.16299347	1.595742879	145.8770274
22.4164257	87.02513849	0.821734069	108.9562035	63.15529093	1.389356414	221.1083504
22.90766089	140.2787783	0.745705717	131.392778	78.36641327	1.421257863	167.7713088
34.03046887	175.1035986	1.820778806	154.9422492	101.6895355	6.257038158	439.7995464
33.50417505	157.5137976	1.389527309	132.7756786	96.54982286	2.249851176	192.9037119
22.3537764	121.8872208	1.670160507	122.6633252	57.19617095	1.296411883	161.797059

16.06941599	97.89845258	0.95043852	112.8920906	43.81657137	1.152015108	113.4588258
34.25065177	155.3582425	3.878477015	151.9660648	81.89821972	1.469840227	221.397275
42.4111604	142.6630481	1.619719811	122.387994	98.18257239	1.905471557	296.9559725
73.27662593	175.0154312	2.394265363	127.9049929	131.7625762	1.355629445	344.3220719
21.41042746	135.5792782	1.309211953	121.326302	61.34406305	1.635639828	193.4905376
92.38037396	154.26881	4.988921045	145.1929886	179.8368706	4.791964483	513.7788206
27.45503261	196.4251119	1.612260345	208.0557025	116.1796091	2.473163038	383.267215
43.30928753	113.7359955	1.996370237	115.0749165	85.50352765	1.269281511	199.1348774
19.87732337	111.237339	1.620750323	126.4298583	48.13596596	1.571757322	155.1752017
40.62684197	160.9241808	1.219504494	116.4637259	92.04584235	2.744845895	273.3421176
17.34771778	135.7151344	1.155428654	133.1781142	51.1843467	1.134974232	220.3280234
17.23981264	153.2473829	2.116434572	121.4839799	63.19174363	1.679517144	217.8138901
20.4587128	104.2740164	1.914248444	72.27951241	55.64812975	1.274298083	132.747909
55.61731937	198.3026499	1.864135072	149.2628214	103.2626183	1.661770823	235.4583924
14.45149153	72.66913798	2.053169497	80.3406147	30.95696903	0.985902417	115.1846992
33.07916859	126.3691996	1.461913538	118.4417747	81.8916721	1.295697827	198.4544226
20.98441927	162.9709613	1.196694961	134.4370482	70.16429383	1.160009778	199.8150281
29.92463427	139.2785807	3.257091224	140.8495294	79.5660651	1.742528113	176.8242203
19.22179887	124.6874035	0.996171654	126.7346499	51.06843019	1.180681333	131.2626182
15.95614274	90.70202671	1.161432135	84.60636028	41.37971719	0.973010576	98.62372711
38.19143323	172.6155244	1.543957074	150.3012817	89.53128523	1.709300625	286.3525629
43.81842908	192.0088071	3.835341225	214.841364	143.1463795	2.894859772	345.2372282
19.55854687	142.1466089	2.111737452	92.70939216	95.61336757	1.777844982	175.6748393
55.90282394	161.6011678	2.809730139	139.7975962	92.42003376	1.681299819	262.4646513
62.72334962	154.4294126	4.318750084	176.0820842	204.8797638	2.762289662	584.2101822
24.96110771	146.8152027	1.577472241	138.5001344	71.69646573	1.443176327	267.4676227
44.81491245	95.32156318	1.270434053	113.8939016	99.87473789	1.816485767	279.4573414
6.837226876	53.37211786	1.866108394	53.50259172	16.96352029	0.398581256	84.26746258
12.90797189	89.99577053	0.784791938	102.8650777	33.91453704	0.737196117	90.24809986
30.21564278	162.3980491	1.215982526	146.2826929	70.50070166	1.048922207	257.8475307
27.48056755	120.6445807	1.369315254	121.7723821	44.67716942	0.881047376	196.8720863
27.02620688	166.228052	1.125827683	170.4655618	62.12700415	1.731614927	204.1849583
45.38156597	131.7837101	2.997174479	152.9145052	109.9139557	2.350103816	309.986674
16.23479795	111.78872	1.115034242	123.958847	42.00294744	1.12384148	151.8228111
21.84768565	142.1054182	0.996174161	172.316128	83.88329365	2.984571443	306.9066269
22.09357417	95.50049713	3.922542409	108.3837271	54.13486085	2.368760805	349.4992016

39.71041833	216.0247722	1.924007468	167.0997298	145.0671389	1.908503866	326.025993
24.48186525	171.9799901	2.092448438	170.7751621	109.3258425	1.707681633	332.0670904
29.30719227	123.9111719	1.5054511	107.0279135	64.49627179	1.20720976	164.0922188
62.1837866	187.7537838	1.96905687	215.2524622	119.8415617	1.97440303	473.2170182
16.20518095	82.18350496	1.12104073	107.4506835	31.95042752	0.691604322	107.622656
24.26814506	137.4409028	0.955243628	99.66720287	65.39857761	1.219155498	138.1585777
51.01035153	173.4815811	3.099387028	162.433366	73.97757375	1.323061829	246.5751881
37.41857435	129.3127623	1.950371445	123.8472096	87.37020986	2.386003062	205.9598712
27.38946013	113.695694	0.956263827	113.5784604	78.75619065	1.581678802	301.6535673
50.46010163	133.4036165	2.779915703	124.221155	88.38022018	2.024776547	302.5536187
73.61132409	128.6287663	3.175443209	159.2318743	149.9003825	2.031248916	275.4131278
30.75106118	158.7588328	1.404981869	193.0644365	154.0673978	1.863803489	632.5494846
42.80776112	185.9182915	1.510162785	189.6140021	101.2119659	1.579354672	313.2005235
28.89241109	172.9979769	2.458211848	153.2118882	78.65574189	1.429596091	270.8312116
33.81357524	79.71289025	1.68673242	62.20267833	32.05079298	0.786229131	80.22503333
30.30124421	159.1585973	1.46092125	162.4655136	88.55664579	2.231629983	284.2982659
36.99365135	150.5227855	1.351110831	115.6051264	58.89553208	0.96961167	172.7056698
13.43042979	114.7459312	1.58348759	137.7578404	56.8082291	1.353857886	231.5532706
27.52505326	138.4165995	2.046762377	140.3519738	76.75018871	1.621286316	232.2946149
32.19395252	167.0905478	2.258691564	125.4917158	70.01229382	1.905041409	211.7058069
21.62526956	109.0696387	0.991746226	131.0043253	49.86704818	1.187853144	162.4112408
51.81571945	180.7533647	2.565995519	118.7619007	102.3891317	2.330620822	181.7934163
25.73203003	130.3982061	1.447791332	162.6085909	71.02440587	1.491530295	229.6500896
59.8661172	198.9413569	3.547360712	162.4780604	121.5283673	1.916122624	295.6961614
36.05949301	127.1174847	1.361234678	113.7752558	75.06069697	1.594097844	173.430143
63.02314758	246.1539649	3.223842762	202.2258841	171.1122445	5.020879274	524.3735983
28.29402519	129.6163915	1.799365199	136.1684514	71.80419472	1.528894065	175.1453369
43.86485977	152.1525488	2.124299488	145.5098232	90.57718861	1.68571675	249.8918995
16.08479191	119.748748	1.188546041	129.3548044	55.79698753	1.002339918	127.0748858
31.17952155	190.0598086	1.337591261	164.1478351	67.64046868	1.580176532	255.0874235
39.12541499	120.4404053	1.906810749	151.5004206	54.77486805	1.892599599	271.5759499
27.06432578	87.66923385	1.753784266	98.92981207	47.39083062	1.101716164	116.6074089
13.55244966	96.09485435	0.673642584	98.04590438	37.13416657	0.899141786	101.0032236
23.62816272	130.9572245	1.400410895	120.5302658	51.22495344	1.49620652	155.9261226
27.81941769	88.0453818	1.55778208	89.76222348	33.59186621	0.804766964	146.8826763
24.74239471	138.9961273	1.104456087	146.386609	122.6289622	2.1221494	302.2563192

24.12527977	133.429119	1.209415792	108.1545985	52.6276956	1.638551262	213.7766841
26.56692645	202.3508216	1.743255754	178.323598	112.8460653	1.882034177	344.0784115
53.24399392	214.1804005	2.234735578	189.7238906	77.95027785	1.911083645	278.9599746
43.54555395	204.7880602	1.917784598	181.1797148	103.3397132	1.870873688	338.9860044
29.07481076	192.9058124	1.066517515	157.1441908	81.62451665	2.146024394	267.6049214
25.82057173	164.6937555	2.415096627	141.8970577	93.72616342	1.877624412	185.4808252
32.2631088	115.2198933	1.867023225	130.1115722	80.66035538	1.402877857	179.7712168
46.01937926	159.0459799	1.626410197	132.1724092	82.0283594	1.094896318	206.1275801
51.99767731	108.1880751	2.123270858	132.0687339	138.4050111	2.139970059	270.9176837
21.39322293	194.1331062	1.551728835	286.0775909	129.3097357	7.01292133	566.9373182
51.71174736	180.4247376	1.832303134	135.394366	65.626561	1.13327969	207.9740634
47.46219258	170.5547189	1.941045889	153.1168147	108.507251	1.628392688	241.7263612
31.72415355	60.85056386	3.457133587	72.02380059	32.58378971	0.544574855	89.4533182
67.85127779	206.4906243	3.51786383	198.0827867	152.6943498	3.078051955	381.7869
32.09271803	118.8877792	1.393947555	104.1068823	79.43413311	1.828658706	193.8923823
17.32832166	88.20894654	1.168690545	105.0547826	32.7441496	0.879982676	114.8254528
30.96126594	135.8824345	1.480027863	113.1825955	69.5675252	1.510597167	223.4528932
15.29326043	120.7056269	1.275568257	125.3991781	60.37389549	1.974522344	184.6307019
39.37197401	94.18710995	2.58665321	111.8491913	89.70335469	1.527380178	174.316493
43.10138674	162.9798456	2.19571425	167.4235724	113.0533502	3.460452472	237.5047698
32.49600136	185.7906314	1.539200525	169.7692527	79.3201221	1.57583396	273.2512138
26.24232023	120.5820967	1.876199133	122.627176	66.50727016	1.631345563	167.6439442
114.1786841	113.4816185	3.535042058	135.9494823	56.62436967	0.754862523	278.7571434
19.94005069	113.9957026	1.628875632	124.6194948	46.94206388	1.663100439	196.3998752
57.30205611	81.77993787	1.371358358	111.5041453	50.23163352	0.984491044	136.3913176
58.8847973	206.7818585	2.165691328	264.8629607	142.1932968	4.417833034	574.6502541
68.09330199	184.5561013	2.804502464	149.6305243	71.67650836	1.382687746	226.0532534
23.7707451	211.9828294	1.931475233	168.2304076	90.29824325	2.459163949	365.9125257
21.29825491	162.203244	1.413444181	139.1362248	77.11820137	1.433551032	237.2455399
22.43754131	115.6960867	1.043875596	116.1375107	58.82261836	1.005569876	147.627004
85.21556205	162.2527546	4.032027209	204.639479	164.3307492	1.740756389	361.1371542
40.76073828	157.8922126	1.77841577	140.5047686	81.86002312	1.492129515	261.4986115
68.32688397	250.1303248	3.192857778	215.2453513	141.8931457	2.284555734	449.6011642
16.33337956	102.2446137	1.604371654	110.6550193	50.73680089	1.732199106	155.5133558
16.01225592	47.79355039	1.436285313	59.40026275	16.88834244	0.407400779	67.95731991
31.2513368	148.9913194	1.41026393	108.139602	62.45957227	1.78747728	189.1482021

45.5111885	155.4637507	2.507115097	134.1367651	86.25729833	1.438066612	214.4806767
19.83154619	136.2775184	1.337205548	149.8088417	61.92542337	1.503369437	210.5686099
36.1801102	227.3414599	1.438049844	169.7864802	89.35449168	1.693483047	360.2747644
27.57377957	183.8821271	1.058641613	233.2619657	83.05098621	1.825616981	351.4422376
31.62981731	97.91363939	1.678658441	88.89420966	62.49346869	1.188213994	109.0637425
8.127842658	93.97836712	1.089394097	116.5225606	37.70558347	0.937036013	143.5117677
29.31250249	155.9816035	1.182504492	160.8734095	114.2351828	2.005822005	376.396862
31.56714539	171.6953035	1.233736407	174.0776603	75.9035458	1.937928109	209.9682624
65.13983159	171.4398159	3.84922086	140.3837937	98.74497433	1.94853834	255.7812084
16.03558921	111.1128129	1.030206541	113.6181105	41.88608747	1.300966888	144.8629891
31.09943384	92.98088102	1.12239413	136.0494909	76.47107809	1.3300524	263.6463658
20.91591389	116.2810363	1.810338947	104.2195352	41.42208446	0.821526035	117.7077515
41.50331438	165.1677675	1.697349538	149.2331651	71.93337233	1.561009448	273.5246572
24.02014151	210.6253235	2.136198138	192.6065579	128.955152	3.678944089	455.3877552
19.90061559	87.58603586	3.235241883	127.0766768	63.61999327	0.964967252	137.8822451
33.9245901	62.36038935	1.724174961	93.03460346	49.19427029	0.88843443	87.44036278
30.15515963	148.6103445	1.817773587	112.9296613	75.47763186	1.491635128	198.9564817
25.05796679	128.2138721	2.021276741	121.786083	60.32194168	1.422199258	163.2086539
31.32248304	232.8552468	3.139670138	231.6505597	114.1531928	3.651167723	558.0023397
32.10028063	116.6827946	1.502259308	112.8171147	84.41098623	1.827845913	244.9669738
30.75061637	87.11087188	1.657292197	80.98088614	55.12024268	1.023981167	114.8687657
29.95443515	119.6363996	1.467596204	165.7932047	86.18345203	2.403596384	221.2973954
47.75095013	203.1996023	2.018626012	147.036262	113.0290144	1.304672796	292.4077064
47.34460401	135.9583113	2.069799693	164.7583801	111.6349002	1.927266315	221.2010368
39.53694223	171.1476312	1.185327312	180.4027796	127.8471219	2.545349933	339.273921
1.897620949	84.5847823	1.045191082	100.6511163	37.22969278	1.634801569	164.6822259
34.5839741	169.0990979	1.4678668	163.5332962	88.39510315	1.706666348	201.1375435
28.6865494	161.4609657	1.401468768	173.819744	77.47847638	2.207618876	263.7866406
13.92031548	72.66282319	2.042907942	104.8554176	33.24192934	0.863677366	106.7475955
20.78158028	133.2120387	1.219910307	124.3714771	65.61473398	1.56605055	173.0663496
22.83464517	124.5184583	0.898799079	141.1924572	79.80705346	2.351263395	320.6137419
53.83508958	193.2429452	2.518987878	157.7658257	133.7930757	2.443262013	373.9526506
18.65386627	86.82771928	1.053315027	108.8471572	59.68689623	1.202894804	137.6434207
60.72253506	142.9913428	3.284906599	164.9780205	88.65166191	1.690805333	281.5019825
34.65421842	127.7283355	1.40058774	116.0776641	73.30313933	1.503105483	201.8733158
76.12272987	113.0625135	3.546286054	109.342157	86.37089643	1.17773287	260.1432295

28.04472059	107.7082602	1.796740673	106.2721896	40.78194031	1.047578086	122.8954769
37.50665343	166.631369	1.502323742	145.6023073	80.38663044	1.523350085	302.9907178
24.45372615	144.9603148	1.247352469	146.2130841	63.72597606	1.244156174	180.3505879
38.61126464	128.7708702	1.149258551	137.9316135	70.73658327	1.590754859	203.1640117
28.46684108	91.68672139	1.630600263	102.0919044	75.52501339	1.14353701	225.0753373
103.8679341	108.2819894	2.453337451	99.63468134	157.2138287	1.635758903	367.2814794
34.43157073	166.6259719	1.96481189	232.77282	126.6213671	2.878857667	308.4983916
70.55696719	260.8636075	2.186020801	245.4364255	152.0898359	2.696780837	399.9734014
36.23349692	134.9005596	1.331791398	155.1157978	103.8830136	2.69355715	373.8911873
22.17399369	91.91308203	1.561580829	84.6557973	48.02043985	1.228418229	121.1749422
41.41881209	142.0253014	4.170459916	157.538113	121.7266284	2.340932776	302.6142882
23.70015649	87.13702839	1.379507926	117.7439069	52.21953475	1.102503298	157.7941056
57.99450874	187.7726571	1.67229142	169.2928366	122.1554882	1.411012576	362.3419832
27.5202444	173.4201665	1.851876512	144.9342831	57.34559924	1.307047104	268.5003514
41.96519064	106.0481566	2.270299627	106.9673144	52.38638229	1.078234688	160.9212606
54.03222435	241.2481601	1.907044306	178.3989248	74.55617322	1.601387929	287.4964602
51.52206672	185.5701816	2.385573899	157.91447	104.6162825	2.345038274	415.0141522
24.07816305	151.7666242	1.399866712	143.8541737	58.60237038	1.709927822	166.1701137
17.57492988	100.9452015	0.970951419	90.99454568	44.14205509	1.442258771	113.3204615
16.05967055	46.59789957	0.819256417	47.22485425	19.78043233	0.288349824	43.35828378
16.67207684	99.72728658	1.400176738	103.9758131	77.37263252	1.966093145	186.9133277
66.86450698	163.8279635	2.039206384	171.7586392	146.5904689	1.69506516	470.7228699
35.44714744	82.33530254	2.48272678	79.79247089	49.05934265	1.164000558	172.2220419
49.68025246	212.0442861	2.610205398	155.690671	103.6254331	1.955972078	299.5843984
11.97692696	134.1228327	0.692534297	137.8630968	77.93682277	2.746379797	317.9271843
31.15985075	90.4280101	1.110319169	95.97573636	48.48461219	0.876282269	111.4119957
48.57927428	224.9371582	2.268912357	212.6190594	107.5965433	2.861643836	335.519811
22.51214279	139.5661919	1.236148846	118.7066587	52.63329263	1.139103523	152.4853405
22.96014584	101.5921531	1.00782746	105.0860337	42.71473893	0.956416177	130.5876624
14.12160869	106.9336156	0.827188743	107.9010105	34.40483116	1.058987176	193.8596096
31.00211627	179.1344068	1.564266755	144.7542266	86.58787779	3.099385726	364.194996
67.66126758	269.4460753	2.722428385	184.2373523	128.9414433	1.800224443	326.5031695
40.6867483	211.3456007	2.229016339	150.5317118	76.25587139	1.564804248	231.3651544
57.15502114	132.9210614	2.47332354	144.622774	146.0214937	1.331391252	406.056114
37.55127048	167.8928995	1.797683538	137.8756638	95.22758614	1.495473331	234.2782007
17.20560932	131.6906843	1.345078054	126.4505051	55.9597063	0.838388752	118.0472683

57.60302453	207.8638767	1.661970727	166.2062435	154.6220946	2.411916932	310.370781
22.75511772	99.15234994	0.960271909	120.6086785	40.81975545	0.816958752	112.3663446
21.94538605	190.9883668	1.162407209	190.4415821	84.00908312	2.48634356	356.3259721
53.08382466	185.1501745	4.007025789	179.1319126	130.4209302	2.080037361	260.1395513
34.40546065	191.8860096	1.948232003	161.1757332	95.81678413	2.142762691	318.5329324
77.01373558	205.5455488	2.051618714	196.5724576	107.9707142	1.13943627	367.0213065
63.85979312	150.1994333	2.525644671	123.5164872	86.38165106	2.017903817	231.5845055
58.87309323	189.2699029	1.451715711	187.013722	86.40317504	2.192652383	365.4889247
28.2318904	123.989524	1.157413151	147.4240934	70.15225694	2.078714977	211.6621865
77.7195234	316.4145189	3.149436752	321.8120149	183.4686272	6.35392816	636.7694224
27.04044589	127.8130948	1.323922942	145.6056869	70.64149586	1.902344712	246.7925385
54.93870487	149.0330011	1.585632459	155.2697857	99.42223841	2.043351601	367.2961267
27.49535769	169.235651	1.5261743	144.443742	86.63048062	1.718358159	235.9162392
97.13387015	241.5324531	3.264042004	203.326324	167.6906041	3.087728897	448.956617
32.60373134	135.7003908	3.029475331	141.54439	86.12359891	1.803361494	206.5230667
42.92125197	142.6104173	1.778852969	133.2363561	118.8898837	1.540193737	287.219058
42.88224419	164.4688623	1.345540963	187.7880339	94.00259513	1.301780201	292.7081257
35.05705279	149.0620685	1.292879853	126.5898375	66.84777256	1.845529315	186.5911061
35.17682677	142.9058159	1.917967447	156.0122648	85.62240692	1.749828066	273.2109545
33.68872795	112.2215081	2.174255927	90.11407363	72.39862789	1.66710298	140.5644628
21.70378825	118.9928874	1.260833942	102.2358755	53.94325776	1.095101439	135.931825
28.88884471	166.8785654	1.849430571	129.4138044	55.02470139	1.360224895	222.8242492
23.00774223	124.8317627	1.372365557	114.3457904	73.47569747	1.483268262	243.817705
43.41346621	181.0638916	1.596207292	179.5990249	68.94320013	1.412694543	298.4442297
43.0982808	135.7670594	1.847930467	112.4524212	82.29492838	2.178727971	174.2369549
29.17376703	154.7700409	1.840315095	145.8654409	64.81440319	1.738050487	297.3780182
21.7643046	118.5433417	1.010766714	104.4094362	38.8050234	1.232810387	118.8714531
22.36509476	93.68030468	0.96427334	95.09703897	41.96573145	1.377414338	122.8358812
19.52226773	86.51217031	1.445542084	90.78883154	49.0981164	0.812770562	150.9586081
46.88436824	232.8112938	2.215424986	155.5832742	66.88461218	1.769628852	247.015027
34.06370518	175.5303695	2.550680292	148.4945678	61.88292791	1.34285527	222.4313322
18.77414073	87.92662149	1.039268944	87.62671298	44.24830152	1.049973385	132.8167096
33.83850348	141.129191	1.153743519	133.724347	80.15619581	1.317453192	173.5269823
68.71636081	197.417587	2.467181154	177.1411703	130.0528222	1.44973156	345.979363
37.97894792	196.2323914	1.554548731	130.5650848	80.36781918	1.518587821	198.9202166
89.02436606	186.0139011	3.247638391	171.7474388	127.3127496	2.473323614	319.131703

33.39376134	140.8102421	1.036940557	156.9657318	96.45470594	2.228921427	228.8470702
35.95879668	127.4212822	1.765664737	117.6413985	60.91456207	1.111437839	126.7273403
39.76804043	196.2106973	1.344128233	153.6015682	79.74410702	2.970614485	256.3730999
17.1742877	67.98856198	2.200161584	83.01523758	39.94349212	1.130267152	116.1812419
35.0053905	164.175749	1.381819588	146.3782443	82.08546907	1.523651996	239.403414
35.98240318	217.0310827	1.027848825	192.4378323	109.1340097	2.413233588	451.1959923
47.29636902	116.4817218	1.871079229	86.76541535	91.47773886	2.986956334	176.562042
15.70603489	87.55286702	1.091817039	96.54914897	39.63304527	0.817686494	132.2126457
42.23772605	171.5641446	3.482591806	167.5119116	90.85086625	1.819439317	238.2050701
20.13499324	85.98155055	0.986019245	100.6736795	41.31248056	0.939318689	118.74013
39.67680822	132.5726316	2.344087234	143.6652809	100.5267238	1.649428353	232.3692885
71.08886213	163.1677327	1.46416706	141.960745	187.7579068	5.898302249	391.470999
37.71950031	148.4507347	1.218965644	155.7208076	105.8789195	1.4910938	240.8320066
36.43984898	99.28303409	1.596311515	116.0192765	69.63910394	1.489696081	156.3299451
30.70102545	62.75346829	0.786834195	85.90617347	68.23292486	1.166993614	220.8469221
18.94074292	104.9131437	2.091991429	131.6434316	80.87774462	1.570456343	161.7600399
30.80210429	134.832681	1.271257863	121.7334888	87.69183853	1.533970185	233.1047484
35.70782446	122.773999	1.95954836	146.6121648	108.3543528	1.814412453	189.8362769
25.38094284	103.9287625	0.690508292	155.2022949	89.50434758	1.078267433	203.6765483
75.56627731	182.2027683	1.75721421	161.7020182	134.4252354	2.659914058	376.8931239
21.25899633	112.1167278	1.234479623	89.03450435	65.91989936	1.106799417	101.1423682
27.17158577	85.85752552	1.051355062	77.26950349	48.56345435	1.120384783	106.488247
31.15484893	166.200938	1.208589807	136.5154402	111.5378675	1.606737538	213.2163251
25.10012141	97.00884708	1.186229663	100.7740829	45.88017755	1.338974532	128.1946527
34.59163685	125.6060711	1.970375455	137.7327	59.47439514	1.262569524	213.9821394
40.24293491	107.4822122	1.331359694	149.1115766	130.5149246	1.932061561	275.6885436
131.520341	176.8514981	5.156384641	114.5411766	139.6593633	1.709394263	379.4787569
39.99978543	97.90193024	1.382124632	150.6570474	77.06323127	1.49350159	200.0957633
28.35386283	117.7060268	0.979693895	143.3213958	84.17260712	1.551994715	257.7266559
63.51719841	157.8075941	3.486199625	76.86907587	140.5394683	2.114924273	178.8378318
48.6456734	193.9630447	2.152508926	161.5012997	145.6221207	2.328563804	295.181252
43.05742151	79.7360724	1.772898756	99.35857784	51.10242884	1.667585631	126.2532579
14.5852885	101.2057101	0.747856795	102.8165383	45.76922751	1.322924653	160.4573191
21.93650102	102.9506723	1.031176958	114.809671	58.40422232	1.141756234	178.7203545
57.45840782	78.35041142	2.328627392	103.9859219	119.5419272	1.710421656	247.9583834
30.5974224	116.9909636	1.050567183	135.9290037	85.38736194	1.435541935	240.3367447

29.05032933	86.26613432	1.362406227	99.50996555	79.54738111	1.496594324	179.5769975
14.8615061	83.58732512	0.700209879	85.13134541	34.26621771	0.753847675	107.2782331
16.61669155	86.17040115	1.266350415	110.6423678	53.60971774	1.230796572	162.3064671
47.28167979	87.29996673	2.178297028	97.65565545	39.01994938	0.992025632	115.7422343
17.05625033	120.6970814	1.004411791	146.0706513	67.28988605	1.900638666	232.5080416
62.02723999	179.0043871	2.53358358	196.377217	132.3010714	2.615856995	232.4724419
28.82755812	106.6018486	1.278897561	114.8276327	93.89412319	1.511723121	208.07384
62.68458174	120.7274292	2.264221535	120.4990376	114.4954073	2.138385497	285.7639963
22.37482498	101.2752137	1.408048373	86.16582817	77.5961534	0.980742609	163.1385097
30.95551369	121.1404739	1.391640838	140.9633178	105.1916275	2.428528105	232.493413
32.66200607	84.60392767	2.037732267	148.8717352	61.42423759	1.006573384	189.172497
28.42430831	77.25097493	1.369867575	113.2547308	65.96749901	1.674804657	148.2723899
86.58899533	95.19757855	4.202889594	129.8430683	111.9657279	1.309419726	155.440297
31.48576035	110.7870452	1.839271667	127.1765349	65.16610164	0.771881866	187.5599297
28.60634396	131.7220779	1.746202834	161.663751	76.40706246	1.404800815	246.0509452
26.71965795	80.89741132	0.777195225	150.1992299	56.65411827	1.116318642	181.453255
39.77229216	130.1443674	1.244607007	122.4536699	68.02822399	1.538345497	218.3340774
65.8537519	168.5171352	1.393505059	161.640856	198.8265036	3.280495954	400.424049
26.19575093	113.7700881	1.673976674	109.6801961	59.08452244	1.106950314	179.751829
34.0077707	61.12183517	2.460748	73.47591973	34.9143293	0.785829699	96.00168257
47.01904287	93.77268444	1.595296262	111.3477118	91.50331702	1.2078859	203.698587
18.65389984	133.1264942	0.968263463	139.7136336	73.50987655	1.320084701	211.8218995
48.69721262	92.71517313	0.952976326	142.7755358	142.6503925	2.995875247	316.5452021
11.32351878	66.95964139	1.589353144	92.47486074	38.49420377	0.661870032	91.03672714
32.97362711	94.00426385	1.518383368	105.2252853	68.21803738	1.678815734	183.0360481
44.94549728	165.6414748	1.596810954	142.378545	101.3571727	1.900949459	202.8574552
70.35180119	191.242826	1.473652962	171.7511522	121.827836	1.977513016	357.819739
16.61769478	103.1683921	0.823439865	100.7537165	43.5148892	1.017946189	137.4300456
9.040105989	72.46575002	0.816884633	73.65458511	26.34837925	0.562359765	108.6601116
47.03554172	98.59708199	2.690888562	94.25006242	63.10021137	0.863490861	137.6298728
34.14143532	143.1565952	0.817909347	146.2855255	90.91634278	2.305454992	252.1524585
15.66525765	69.33880478	0.808247781	113.1418725	55.97248767	0.65733972	129.0271992
19.28412986	83.06133287	0.917560763	91.98033955	41.11476937	0.898851282	123.637542
72.07895545	169.0288688	5.031151726	170.1070671	130.2674427	3.055654004	341.5930384
23.97470242	83.50876051	1.360285935	114.9425366	48.09455509	1.265270171	155.5787484
25.18446623	85.6575684	0.904823386	113.505866	62.26848116	1.310143785	126.2626535

26.34080019	128.6573921	1.113187951	110.0282162	78.21924067	1.006157958	185.6034877
156.5003539	95.10790541	2.61839916	94.06961314	70.73205092	0.514344302	195.6378152
29.21808934	137.5501939	1.037900875	180.107081	77.21715835	2.075043106	227.4609957
26.51866602	149.4175728	1.10690225	126.6392637	64.7222086	1.655872785	204.4713677
24.28111118	122.0657844	0.782589997	118.2567561	85.21929821	1.719530711	161.3050673
40.59786085	132.8553117	1.090713203	134.7064643	80.93717691	2.05147191	251.7743997
75.33694949	84.87985145	2.145244544	87.32335323	106.7700051	2.103937146	216.1388117
16.94732433	92.19413441	1.219467517	90.29974726	56.90846641	1.215989487	126.8368789
17.34229173	128.9530281	0.827470797	154.0762955	73.4652634	1.868267308	327.5265533
35.40200686	146.9542202	1.616669193	166.1361258	91.59239754	2.526055315	274.1257589
40.1756797	115.0541623	1.581965348	120.5323673	89.10433848	1.309995273	204.3844397
27.26611833	101.5042376	1.605924904	121.3499032	101.8420723	1.45431039	234.0549436
158.505399	93.9963889	4.255577083	74.71889963	55.62103854	1.605603069	147.4872456
40.1003349	149.3584832	1.37380257	172.6893658	77.63738474	2.120375607	294.636876
8.947100302	59.57911226	1.498367587	69.14493196	27.40510703	0.479064004	75.2545663
16.93303813	89.52035998	0.791165398	106.8627531	47.41061525	1.414610019	149.7536082
13.00816781	89.09845222	0.916985905	84.29542953	49.13558772	1.061270234	121.0159495
114.3607655	112.0636762	2.306625627	140.8214773	117.6042713	2.144400351	275.3375899
38.29623741	155.460481	1.923427537	148.0545805	84.90313154	2.450476757	273.1489958
25.22183267	78.060351	1.141070919	98.56508747	52.53205559	1.60252707	164.5798164
26.55786094	159.2013751	1.305942501	188.9831091	78.61232492	2.374049786	303.8320331
31.94775741	144.4468292	0.821208629	122.2440644	114.3022822	1.870925369	228.2590927
25.76531204	149.683363	1.55649309	171.0694587	98.21672564	2.284771578	284.766994
38.25996938	84.37037902	1.108350559	135.6285084	67.6936936	1.464915167	235.9584402
38.97592521	121.6749802	0.970862202	183.7036508	145.3097625	1.649882286	381.3244287
17.84571405	113.1891361	1.173824165	127.0650257	56.598785	1.172877855	218.1704132
49.37754673	179.3639517	1.344128393	169.418712	98.18326221	2.120706099	265.8303251
38.85248316	165.500991	0.923105154	177.7450651	194.7817156	4.082238173	495.4009249
24.96073028	132.9269265	0.95511226	147.4073128	60.35590165	1.544526754	236.1247876
49.67522169	172.4618404	1.518215776	217.404743	135.9240607	3.135869695	429.3134187
34.78597196	112.7930399	0.945620673	168.6491557	96.48200834	2.306682525	297.0665971
37.60944101	123.7349347	1.037913277	156.5170399	97.40598548	1.286495481	279.1695895
20.5349221	60.19244231	1.383476219	83.67542733	37.13461966	0.65364149	89.66404361
45.30427207	187.0210333	1.356661682	189.6566048	112.3028841	1.833445461	314.6768454

NVP-ADW742_1932	P22077_1933	Savolitinib_1936	UMI-77_1939	WIKI4_1940	Sepantronium bromide_1941	MIM1_1996	WEHI-539_1997
15.14338622	122.2110067	19.17612046	22.90057812	52.7001859	0.019957111	88.2016352	56.9418258
10.46926447	93.17860771	16.52204705	10.94596591	42.6564065	0.016795585	55.830612	36.60141918
36.72613053	160.3630949	18.07199676	17.33198804	42.0581249	0.00877491	59.416479	37.97347776
22.45955076	100.6161653	9.02573916	10.4741788	53.1793247	0.022715583	40.0355146	29.49018436
14.76999421	209.7634425	18.7425973	19.77371671	49.8704298	0.01614239	119.615795	108.4895638
15.52534293	91.27030231	13.78470772	16.41968306	40.7294527	0.041356779	30.5975088	45.06931394
12.50945914	124.8456036	12.55891302	21.06926847	39.949806	0.033751504	53.6556139	33.32066285
33.42452491	122.3732922	13.72183349	22.49046561	54.1003735	0.013815263	78.4539832	58.44306285
35.577298	205.2617446	28.49760733	25.88666157	54.4733819	0.016502139	114.095378	53.44336103
11.10669537	58.67307901	11.4366327	11.49170219	32.8058164	0.017138856	35.5919306	11.77859618
11.73307621	58.41502246	12.48464995	10.37797089	39.4696367	0.023014254	44.687664	33.29448166
13.49604986	80.17539091	13.88819333	17.24522843	37.0134328	0.015178499	41.3401197	39.66115129
12.33067742	73.41581993	11.83775547	11.05881558	39.7330515	0.010003323	43.0905486	33.18741654
43.04040077	83.29180443	13.60914737	10.86167443	51.8998202	0.014838578	48.7226662	45.17767734
19.6588335	119.9371108	12.39191305	11.20433315	36.3053854	0.012554283	48.619701	22.47746888
12.1785553	128.2499047	19.42981003	17.34940197	38.1555174	0.033237062	70.2346785	47.31231394
5.231645401	11.03628556	5.822101346	5.727503572	33.9532962	0.004302259	20.2022581	19.77803371
44.16555186	192.9936913	8.739978005	27.38825787	66.6636756	0.038593893	66.6144727	69.20424928
19.12844998	123.3411594	10.21196698	12.37808818	36.013093	0.010368951	43.1432125	30.15668698
13.06884605	45.31795679	6.505381165	5.912340916	29.6990076	0.006432191	26.3827466	12.03968109
18.15910607	121.6278942	16.0264325	13.69640845	46.4199791	0.015672999	53.4007943	27.59312604
41.55360374	107.3200954	19.96643623	14.1070044	49.2806039	0.008017876	85.854047	111.2773584
14.52331135	64.91791865	14.50768649	23.71010071	39.2597982	0.011971956	49.8135671	36.61252316
22.71382694	195.4331257	35.11760897	35.52212068	44.3860081	0.022163508	85.1969434	38.73456641
17.12511534	117.8863209	17.74706426	12.55876016	44.3508407	0.014037818	60.0719121	27.87009486
17.86424958	87.76073045	16.57444263	13.20018983	43.1637235	0.010109915	58.0572026	31.11173457
47.81970152	137.2809925	20.73152793	20.35715631	40.6297275	0.012013489	72.7895449	61.06912456
26.07276549	228.0371332	28.31596308	27.68934444	49.7400385	0.014658646	85.3697369	43.05317147
20.06736532	95.71923542	10.56973378	12.92513471	38.1710935	0.012170881	40.4991786	23.41782525
32.68275524	50.5113695	10.21341286	16.50851929	38.7428109	0.010043856	42.5788525	56.60950255
7.516291662	69.90126676	10.54057289	12.84163673	40.571372	0.013505526	48.3983685	31.09433493
16.15034937	86.34767294	9.551870368	10.55534431	35.6804438	0.005933829	54.5882066	34.22905033
17.88956358	110.6298797	16.04068708	12.76420545	38.3380503	0.0115896	75.6828425	33.94153863
22.56106593	39.66943989	8.214174032	14.45393813	39.6165342	0.010621111	26.2025591	17.66307336

24.04095815	107.1563634	26.40172832	25.65910874	43.8277387	0.012442195	60.5975797	32.60949012
16.94873825	119.2466201	24.24382845	24.12196789	46.2236781	0.006822551	62.8310671	40.78832859
11.35455299	54.8223541	6.331442327	7.435940289	27.7054513	0.006395891	27.6510243	18.73801305
35.02768041	224.5261315	19.2067744	23.60299743	44.7675845	0.011943961	106.37783	73.21736135
18.97636359	89.75341291	15.03243889	25.56811662	45.1081278	0.032850286	61.4482326	72.7595551
10.70469167	30.18627862	16.62236864	5.648581697	36.5876618	0.00385671	40.9300765	25.08821416
45.28238678	72.32117803	6.970727713	21.62767597	57.1426903	0.012616687	39.7490266	49.86642272
10.03503726	116.5103359	13.25570999	14.50309217	45.6911644	0.018734983	70.0380054	40.82588982
10.75163024	130.5373023	18.03805598	27.58024701	38.5636737	0.036040863	92.0791983	63.12325997
19.59134415	83.55960709	17.76942917	16.51610093	40.6626733	0.013565718	62.7981456	44.49548549
12.69485212	52.93191093	19.37768644	22.05928563	40.9730805	0.013565017	40.8876319	41.59633759
3.912555104	4.62345584	5.857738029	3.214355311	23.6567098	0.001391091	13.9388676	1.799671483
11.77607963	75.70253519	13.03270453	16.44906711	33.9526771	0.013120631	46.1693644	19.05620984
21.53507866	101.9516234	12.13349507	13.82857263	46.7900655	0.009695618	43.8080367	36.58924844
12.13381476	81.25851018	9.729950868	13.32363595	38.8486649	0.020252165	35.7354327	30.70181966
9.531687887	84.85852721	12.62770558	9.083573525	35.7084384	0.010937132	45.6404317	42.4053093
8.530113654	51.25289356	10.65218461	9.779677921	35.8443448	0.01522711	26.8573015	18.85320855
12.9487655	128.5140378	12.82075221	10.70694968	35.801076	0.011421511	45.3300801	18.93361745
23.00509117	90.70196717	10.04604907	10.9989546	37.8348347	0.007492091	45.6836334	57.70857102
16.72511456	75.26348501	11.60960324	6.768003592	35.3946131	0.007762725	34.7563817	13.65154425
53.18627958	62.9717774	8.169186572	22.30296691	48.8518913	0.012490493	29.6186079	34.10833784
79.33000874	215.9994309	13.91790945	30.06372503	55.0180269	0.015256148	59.6727027	92.75532557
8.030934485	61.24310186	14.18088542	9.12883529	38.8482508	0.019984283	28.9290666	37.34216112
13.43849068	73.11569381	9.290267874	11.65517374	31.4711963	0.017498888	28.1020558	28.63763864
37.69557053	131.5130744	18.65486867	48.98400815	51.6388861	0.018820942	95.5365217	65.6987344
20.35396249	53.92359206	20.56025957	14.96681101	41.9954519	0.011669181	53.3136776	30.2924178
27.43392658	211.920427	21.09419204	27.24964618	53.3438211	0.023507839	115.781072	66.29077111
17.69296991	80.45319488	16.32460303	12.49891957	44.3136735	0.011653059	58.8439155	24.04681839
25.74637469	99.75566078	23.55637456	11.61564949	46.0188763	0.004495138	108.725158	51.55441655
15.74256032	51.16723379	9.870615647	11.32410748	44.526949	0.007867033	37.2726788	27.65319763
12.36111216	96.0044253	15.36973996	16.05804151	40.1408802	0.019606366	42.2270233	24.35012094
11.82863722	88.90315168	9.917628606	16.02029316	33.7138011	0.020378408	38.7613906	26.07146008
11.49077831	134.4990229	9.347690445	13.34852186	33.9559502	0.015300709	43.477942	22.86232252
70.20407434	203.3192845	22.10597066	79.03264614	60.6671772	0.017770747	123.836221	61.30147864
15.00762758	101.9010296	12.00581693	15.48508684	40.2545014	0.008591742	58.6054866	45.71056206
13.54949657	62.27772548	11.42785958	15.62939662	38.50264	0.058486825	31.0268068	26.46475589

21.52933368	100.1831702	16.22545509	13.10806291	37.8470382	0.005375883	73.1487458	48.9267434
20.70878291	30.33062656	7.825630717	8.695338319	43.6882769	0.007104346	27.5164562	20.88425426
28.5218165	113.7494141	15.12104754	20.14107567	42.1902703	0.011178745	57.1203591	32.97439974
58.94481953	242.1699177	28.90695762	21.81901152	59.5452671	0.011201234	160.80747	69.89612869
20.00897211	77.44163399	11.03827145	11.23220237	34.8254802	0.011361188	44.406143	16.9882377
10.85468473	88.82902412	11.08204507	13.94369285	38.7378914	0.018859511	43.9271742	27.9860388
15.33832217	48.10915479	8.207578721	15.54640463	29.153385	0.009965857	33.4050929	26.45987436
11.06629555	89.22144388	16.12113408	21.26513501	44.9233935	0.01788797	62.1056396	45.34506644
10.51356341	51.83408312	14.78996356	10.78060669	31.1032517	0.004392633	60.9660062	38.51362493
18.91832656	57.98027027	19.25360468	13.20882427	40.8136456	0.011440117	47.2271023	22.88895989
66.6332211	100.2591713	8.436359051	22.87136721	45.0966241	0.010320725	48.7428057	32.38369481
42.0901743	194.5083998	23.38225563	17.86889437	47.7670572	0.015406399	80.681756	62.09233959
11.46049786	54.71936124	13.2107931	10.96605051	32.022062	0.009098099	35.5110315	13.30592791
15.69340952	68.21987454	7.458836237	13.8588458	38.8544836	0.015502176	30.5184769	28.94247886
40.32744834	158.2951212	20.13159427	24.09286264	40.9313616	0.022176796	58.0024275	54.23830911
26.0451235	98.97143124	16.39524701	15.13110001	42.2832762	0.010799906	43.4482977	25.27277181
5.916660938	27.79838701	7.893257832	6.181578345	34.5310654	0.012459455	20.4179434	5.321494181
19.50277691	99.2515901	19.92533259	26.4082853	44.1960236	0.027188132	58.4574128	41.90329301
26.66117674	83.92890013	14.59265028	14.28407446	40.8984902	0.007592028	67.9511976	49.29617012
15.87416497	77.03915573	11.67500449	10.22767023	37.8605019	0.010844768	43.515225	41.20850275
12.98303791	74.20486607	13.33799846	17.23868694	56.0153098	0.024070893	67.6514758	62.080177
12.60841238	37.49753918	12.24222836	17.18539797	36.3842188	0.016706058	30.1655745	29.03494876
24.44979055	74.02439449	17.02478891	35.83026254	45.9922669	0.015963082	64.0587882	37.40343368
18.94469299	89.95325238	21.81403402	17.63818467	39.1614869	0.017673664	52.2214698	47.35072252
15.551324	74.73645012	18.5360868	11.98684422	44.8289276	0.008036174	55.6714436	35.06517724
28.11992675	115.7137599	9.700715821	18.78246246	49.8843653	0.017008876	30.9838089	38.11990472
10.6753181	57.10947395	12.26933005	10.82764863	41.130162	0.012715881	38.7679143	30.959021
22.30536378	186.7057698	13.10951831	17.67139748	44.330149	0.021395686	48.965113	34.19936704
16.76901235	77.78390643	9.905123489	13.38547817	50.454919	0.018618834	54.0043556	32.41282263
35.70120864	57.36402117	6.596983118	9.761565521	40.4593018	0.011265613	46.3661569	43.59847662
16.47951749	82.03667907	21.37073308	12.00664851	40.6557562	0.006333739	63.5844131	18.23703066
16.87461281	138.3037277	19.92450817	23.70709752	33.7392086	0.017137193	75.8989545	37.87235457
10.65841938	20.44490641	10.13640201	5.761213805	24.3604452	0.003014211	13.5152362	1.887721559
13.70628944	68.13432204	19.24315448	24.59211001	43.0487314	0.018619826	46.2785558	26.90181955
14.54476397	28.21825475	9.559919636	10.45244569	26.5785493	0.003898654	20.6795472	33.5213489
18.87532535	107.0567507	13.0486836	21.2139913	38.7686665	0.014484665	65.0645539	49.78575836

19.29432243	166.6191056	19.88775899	17.90450404	48.3505253	0.022914682	74.1220324	53.3767979
11.85331366	121.6104729	23.98804254	18.88775429	47.4174602	0.025275447	56.6872114	54.59298722
12.32931441	53.34798946	7.985665298	16.61432601	29.9752087	0.013116174	26.1205927	11.87353283
21.9394534	148.2439161	24.7980631	14.3853618	52.7128232	0.006240543	110.723367	50.42492459
12.25450805	99.73233446	13.08017402	19.99462872	40.6068763	0.023965163	71.2879852	39.79439621
16.41651805	107.9049287	8.87809279	11.97134968	33.8708341	0.023726141	43.7896503	25.23361001
30.48937959	215.5196963	22.37757398	15.36840167	47.0184247	0.019764082	93.6126439	41.41298077
12.11193739	63.0266187	15.39176705	14.61916654	41.0438109	0.011247763	49.4955662	21.46651613
40.37967084	164.0344723	16.68476628	28.97448355	69.9172995	0.033974592	86.1985359	82.44060905
9.971704225	83.48450856	13.21741171	12.77839406	39.2730685	0.017610671	41.6914316	23.85559227
28.02400361	60.45104725	12.83322821	15.99852629	40.0742663	0.011770135	45.407121	24.86276706
69.93538278	84.6146963	18.8397648	23.72835356	56.4598868	0.010830326	56.3378032	33.30691199
16.75050803	220.2452186	17.75194054	16.36387572	53.2139288	0.016107927	73.2235327	45.87014445
15.90975604	81.04101025	13.16559313	9.922369254	35.5553429	0.012936205	38.997356	29.41031361
10.77539176	24.13744321	6.822006591	4.936901112	40.7850573	0.002926312	19.7498649	9.528449074
11.78723965	131.4404498	9.877387322	18.34492251	49.6173303	0.04986713	65.3977182	39.50791423
8.874211386	67.71631091	19.00997316	11.40350903	35.8199882	0.010491563	46.4489035	20.95970964
13.51846107	101.0725084	5.936507438	15.45670436	33.4342736	0.023548635	52.1594063	21.82375791
11.58491089	53.07992161	9.609532247	8.210238033	38.321401	0.00518773	28.9119635	24.16506139
7.115429838	183.5649558	23.50448484	22.78193572	57.5007236	0.06073805	103.100243	64.11661502
25.47925766	113.8143566	14.51430693	25.90570363	40.8731233	0.047721184	76.5685089	50.04536273
15.33298369	66.91474392	12.10362202	21.65249799	38.3574524	0.009779585	48.2191255	31.28876304
9.384496742	47.15683005	11.79165853	12.6350948	35.9099891	0.016584187	52.0069446	15.96040486
20.86876613	108.8830699	9.510453418	6.137517529	48.9488647	0.007392534	51.1614184	11.60714016
13.77022971	56.58278921	12.58750044	8.147583807	43.6688	0.018216959	48.1882793	20.27188518
15.07215742	110.609178	13.46952509	7.587324801	35.6858246	0.008046263	36.9122338	15.91805929
34.14142859	192.1330131	26.99761086	43.61073138	54.8491761	0.020212456	117.631643	76.47664228
18.20877285	82.05112182	9.648226981	14.61742764	42.7087326	0.018484617	32.3362615	27.32311586
19.42157367	88.71189595	18.90347894	24.04441299	45.4660388	0.015937539	54.4626281	42.35746011
35.77695521	257.4392125	19.82626765	16.36685645	52.0206942	0.045641232	73.0038292	49.98787907
25.49248398	111.333623	12.55499511	14.39968035	41.362932	0.00630589	57.7839192	69.73130246
23.25774886	94.7180414	20.20660133	17.78624389	44.1168283	0.014286127	54.8440201	73.28255486
13.32548194	75.38286985	12.74055411	6.519728735	41.2941307	0.012150358	24.1983045	20.23442312
21.27575584	192.324645	8.468390998	14.48315614	38.5295157	0.046005005	56.6024783	28.19421787
20.16121925	106.6700981	17.12657364	17.77144183	36.719088	0.007088246	48.8180418	37.16067581
28.37448989	171.8014763	21.67519482	18.49770097	37.3706062	0.016734807	70.3611104	68.36209539

17.73716668	200.4878776	25.03883986	19.28372796	49.5401861	0.012057542	60.3608503	40.78286225
17.80628587	125.7044198	18.49888743	8.368346681	45.2051545	0.008305552	64.3590696	36.89076217
30.5847476	122.9430359	20.8849569	15.2787511	35.2787384	0.014018487	62.7863964	39.34392086
19.95300166	166.1335101	24.21416231	31.46947091	45.1519184	0.021731985	81.211917	52.64359094
35.01667344	232.0112563	20.4532903	10.4082639	43.8716152	0.008406621	92.6473792	70.76841598
63.91146572	65.60703765	9.951186664	17.42503043	41.152401	0.008767091	40.5185168	30.40261662
18.28228414	114.2317427	25.49743161	12.99671862	49.0462693	0.008750449	53.3255012	50.80618033
15.63974665	118.3593793	12.22362082	17.76014587	41.8396419	0.016168308	47.1767177	30.11469872
15.61988956	153.7936853	12.92670233	32.1547999	44.4910144	0.054259751	60.0068426	52.63405084
30.80614976	91.97500977	18.21539599	14.04489209	44.7839781	0.013126629	62.8179166	28.68704728
31.39323635	139.5212685	32.84433999	31.0067773	56.0731051	0.009517672	101.708883	46.53797244
8.220107798	154.7730452	18.04189604	27.6862207	39.4570245	0.014774452	64.1294497	41.36173968
10.78861378	87.45402681	19.87414631	10.18542118	39.6334339	0.008248553	45.4827054	26.19585055
42.34481711	127.3110446	18.27758449	15.3968904	46.5500656	0.010730953	57.3096824	39.36610563
9.094583793	94.79801352	7.537437675	13.68506658	44.782754	0.049824307	47.5252929	27.68465987
32.26535418	68.70145393	10.96409756	21.66356261	38.0824525	0.010270283	46.2617736	73.40300015
3.573085954	34.3858097	4.61817772	5.449196799	35.2965172	0.018024028	19.7913495	9.224426047
47.94792576	142.3768483	26.60327304	27.13518036	47.4112778	0.012192295	75.9351216	30.79335802
20.09434545	92.55329774	12.10550786	17.58814423	41.6258009	0.014487337	48.8506544	28.94088168
8.164927931	64.23236106	15.12142389	15.07450533	42.0615236	0.01872583	39.2300012	28.18799846
10.65923139	36.66428885	11.95201807	12.41348963	31.0330922	0.003783766	39.5298267	36.6973806
15.54948023	113.1636203	21.01925436	16.0466389	44.5719323	0.01521833	55.3582374	44.57908193
16.42496702	129.2859771	11.94887616	15.43313483	41.6260914	0.015373656	51.0032551	32.71311867
25.4187945	71.25951254	9.958193454	21.95904603	46.5072786	0.016496348	37.3592715	35.95537939
16.5322848	75.41639125	17.59047233	12.22085453	35.291111	0.012012823	54.9675719	27.88562982
22.59425261	89.40611051	22.24900245	20.923029	42.807995	0.007538806	70.1162578	51.66324308
16.52814191	80.10046786	13.28661174	10.79858178	36.1356967	0.008400196	34.4932788	26.07582244
16.91509638	166.3447109	12.93601399	17.93649385	42.9324641	0.031430728	63.498358	55.36575625
14.57260018	104.0989203	10.08488935	17.85690746	30.9856949	0.020619161	33.338405	37.43044525
9.780601211	101.7178025	6.644485395	14.75362462	30.2663427	0.010039226	37.1964114	24.17523454
10.53286163	87.29013009	12.51527791	15.32077965	39.4574657	0.013415884	39.4574462	32.39380483
11.35440871	91.46675201	13.20870045	24.82777953	34.3656643	0.010861483	51.4636131	43.15292371
7.44774719	98.46764005	10.52226794	15.03482356	41.7912707	0.028313455	41.4158907	23.97865983
22.77954317	274.1486179	13.97733764	37.21248965	45.1219756	0.039261125	80.345795	72.88356425
21.4253668	133.6945764	11.34057318	13.08714323	37.0117002	0.011114772	62.8556351	32.4197005
9.559340007	65.88293103	15.66273159	10.26041981	37.1716698	0.007205622	44.3910342	23.96469407

7.720077164	50.0977554	10.57814156	11.68941431	38.8547305	0.01674391	30.8294816	25.03538475
16.86048167	102.7359663	14.29972822	6.762136855	46.3836713	0.003967125	51.9880175	25.04265573
30.67150179	106.3429834	12.79080011	23.89983106	45.7787131	0.024884113	41.8862541	40.20882149
41.99484171	82.9724689	11.06545538	19.6198079	41.5136471	0.006878782	51.977768	34.79646739
13.36591326	92.05966487	17.59702629	22.54529894	42.9225967	0.01904027	59.7284612	34.62479231
98.73352729	151.066655	16.411282	41.34064327	57.2323504	0.021830299	78.9303907	101.8521045
21.44879498	99.79871217	23.16850725	18.33521655	49.1468655	0.009490539	78.4576887	42.52682958
10.59694745	83.73235861	16.4041921	10.8154163	36.846895	0.006319891	47.5656282	19.03708536
13.84424485	56.99278979	13.06385247	17.03365764	40.5881515	0.014971952	47.0274732	27.38263363
14.23208434	178.5147149	17.85424346	34.46890984	43.4038553	0.023112206	67.5810059	51.62636047
6.764537317	69.01484196	12.48111438	13.53650742	34.3449355	0.015949949	43.4167522	24.4315581
11.7381729	98.37416792	17.8835731	12.67748429	35.6928717	0.022603585	41.8200463	18.60228677
17.56904452	33.40690416	20.08931396	8.504089054	37.8937186	0.005355423	41.8172252	18.42528072
25.74023286	122.032715	22.77476368	18.74602765	38.4608094	0.012371625	49.8663251	32.3819572
2.985545152	26.77430641	6.59735478	9.160454376	39.3254313	0.021465815	19.5288755	10.09369671
17.30852544	78.39867399	9.316927201	11.26112883	38.0219717	0.013392246	30.3893851	15.96378089
11.25988087	80.32861125	16.18541291	12.10459716	35.7556054	0.009116988	45.7892765	31.62644883
41.0626882	72.04194535	24.51742648	17.00141291	47.7089899	0.010886492	58.0723621	74.21010529
10.04247845	71.48216317	14.97008108	11.874931	38.463677	0.020115029	41.8058238	19.39626812
12.9751236	85.79406469	20.60836803	8.720351768	36.156785	0.010319381	36.4314933	17.38146067
23.24132216	108.2172532	22.20399974	22.12433501	39.6785418	0.013943484	64.3314883	42.58115738
32.83839797	124.9767723	29.56472247	11.17287318	43.5125469	0.005925716	63.5056914	26.84350327
34.72283398	33.68787201	10.31933308	24.65154573	37.5532229	0.010393684	28.862868	25.92205498
45.27561923	95.83029234	6.517672919	14.83288107	45.5942225	0.011310339	38.1119285	53.38193695
50.50498082	144.5458279	17.15044519	19.53099462	54.8893325	0.018501219	85.5157202	68.5383413
23.62732234	172.1244172	19.23352422	24.17426062	37.9961385	0.011103446	50.6148911	50.10052407
18.04680034	60.91238716	14.30863486	18.97821778	44.1609047	0.007647811	65.7995163	37.20153401
6.05063519	15.32394461	8.260969663	5.135943121	48.3104628	0.004730096	18.9916506	2.848004218
8.147030606	50.5626375	11.60661462	10.35544647	37.21059	0.012365059	31.0856539	23.37523947
13.75417386	57.87277028	14.02663592	13.53588269	37.7708267	0.007753874	66.0727035	28.51974167
11.27423173	67.83186648	9.27780779	8.222909196	32.0271041	0.010894063	31.180372	17.68176127
7.73744992	85.3211898	14.37904863	15.99170827	46.9065383	0.026177726	60.691785	33.39778534
32.19549401	89.42471763	18.6247315	17.4710863	43.4529337	0.011421146	60.6260694	54.05689781
9.416801018	63.8530759	14.68718929	11.53875669	43.2758631	0.012781141	43.4436346	20.18257656
11.73767504	127.5617503	16.06271931	23.97402576	49.2626745	0.019911221	78.778712	45.91840021
48.34415689	70.1680954	14.69920835	19.15299998	53.5336795	0.018203166	36.078197	29.58857252

11.49684586	150.5661189	19.58182066	12.57997099	40.4947678	0.021155696	53.0349453	26.55974337
9.963371049	157.8725579	22.38851464	13.11685489	46.2631693	0.012141593	64.5007564	32.93112651
10.09817212	73.00221505	18.24673736	10.58551117	33.0561902	0.005178607	34.6629857	18.71128917
33.24018105	116.3286918	14.89765113	30.11746563	51.2190921	0.010738301	84.6170548	55.87883885
7.965147219	24.41549669	9.782759838	9.933886765	30.6034128	0.006956748	34.9762037	18.76979823
6.235436319	85.11912066	9.741385582	12.80674124	37.4611437	0.021539278	36.9144677	28.97925288
14.13176304	104.5629292	10.03761198	8.430223425	35.1310714	0.006048638	53.986404	35.83941868
13.15388132	131.488376	11.55489255	27.75222657	35.1121701	0.037483815	76.2399786	62.89940653
12.31547245	96.99236212	10.79177501	30.6873642	43.0906157	0.013891051	57.5388063	40.96310188
39.46328972	97.8924801	13.88636365	27.36760598	54.4159713	0.019548724	52.1915471	77.46659007
18.60987949	274.8070701	15.07618667	9.208086868	44.4764008	0.014579454	45.9766025	23.86450656
53.48652847	81.85079857	12.09384886	22.60115736	49.5927561	0.01196658	70.3417277	51.04518488
16.73606146	151.8187095	15.45043422	17.18279256	39.985119	0.009050787	62.3583034	22.20892826
23.87124933	72.20949775	12.65688573	14.50283677	38.0643274	0.01208533	46.7531859	27.45398236
5.831562442	36.80505432	10.83150738	7.299563653	35.0092164	0.017472243	24.0290128	10.15579579
21.93059669	117.4158113	18.62345703	17.95405783	47.3853019	0.027803974	62.7814413	43.00533204
13.11625607	64.5478516	7.103226573	11.88985618	29.3611285	0.009543991	38.8303097	19.2131557
11.66767725	72.65534746	16.62057216	10.21388437	39.4283581	0.00713322	37.028444	21.53183005
17.05181776	103.0394403	17.46661772	13.77963408	47.56217	0.024496456	51.1234915	38.79734669
31.32321166	84.77630416	20.22694418	20.48859465	40.6463986	0.008592488	49.8458818	49.01687958
8.48099439	79.72412111	16.47615262	15.12340906	43.2101365	0.021281492	60.8270142	37.01206639
17.77010941	84.91349468	10.54136943	16.40172873	33.5906424	0.017313263	46.7907352	26.41111567
8.156453184	90.74294852	11.16828951	10.01847519	33.3108009	0.005327248	49.7045317	37.94664734
24.93526717	97.29779687	17.68242084	13.82706544	48.8663735	0.006268205	65.3600246	38.28268489
17.24618414	56.28733406	12.16772598	12.45299379	33.4218677	0.010773814	37.9730167	17.92830476
31.86815893	207.6807504	19.62133988	22.10598003	49.1858529	0.027175431	112.044041	49.67127379
17.36305199	55.39240132	11.3002024	16.0913054	39.6522845	0.013124402	33.7212899	17.34529638
15.53188294	133.4937814	17.32095887	12.50795294	43.6998304	0.013929707	45.0202858	37.82780829
11.22137315	72.79690048	15.70624438	13.94170046	41.9116485	0.012579588	46.2060951	26.37314545
10.58666843	89.41081765	13.77378146	16.61631197	36.4425869	0.02068348	45.2396077	24.8765817
16.15591392	69.28176619	16.58539847	15.14244612	34.9660847	0.006531448	69.7458067	32.41244507
22.52792413	30.62416245	12.03188282	9.864051162	37.5371165	0.011792097	33.1321683	26.29343366
7.22517811	73.33877952	13.77743615	13.88506945	41.4927127	0.026335726	39.4861351	27.00084962
12.7580064	63.98165082	13.29145025	11.35929008	39.2809327	0.013701528	40.0142423	33.33356642
25.40538362	44.21813973	9.600318243	9.304828134	38.5544869	0.005915161	37.1711481	12.39502704
23.52947091	141.8942586	16.99181486	24.06400475	39.319875	0.02003354	48.3525373	26.01723892

10.58982973	68.22004362	12.84206698	18.48966962	40.4533668	0.022938561	49.9878116	32.77148568
23.02216266	106.8372169	23.48889644	12.23274988	46.4416211	0.012577424	65.3742553	44.20530923
20.50278375	59.85031791	9.690087204	19.66841975	34.3369883	0.010251516	43.5579838	31.71185588
23.3135727	126.1555364	24.38071327	25.58103557	45.0645992	0.014832406	63.9193289	35.38060186
9.756772723	93.88121323	11.3828509	22.42021421	38.7535054	0.037380699	54.2372799	25.20492324
19.65663616	108.4432928	24.99647022	15.39727129	44.543521	0.011014262	53.9923528	29.39693868
19.59379618	59.03418145	8.720828901	12.80334205	33.3927314	0.007003206	37.5986458	28.04842319
15.86200886	117.0998904	19.1404042	11.55136312	39.4407984	0.009457186	50.1982494	62.62045049
53.07385799	135.1721046	6.802117889	20.08984013	48.9804183	0.010228918	48.1933148	59.63988485
22.3663163	212.158702	29.86796286	30.4653399	80.1169958	0.015188427	115.687786	49.14934191
18.2121579	53.6747835	10.47652199	14.35112356	31.9502747	0.008159455	41.1184287	20.70323209
24.50744531	146.0754697	25.93927736	15.40103917	41.6424187	0.012346967	67.6194201	33.75405682
5.589431309	18.96219339	4.721550999	5.30273656	33.1007451	0.012472757	17.2960029	8.99363441
47.64239743	159.9893065	26.31740404	26.34536526	57.6502406	0.018519425	92.0189868	39.54075498
11.35032511	70.74622126	18.92175685	25.41891576	46.5772706	0.023718144	51.1534209	21.39060523
7.983861254	33.06522737	5.315936366	6.835921371	30.7807938	0.006157877	22.3597163	12.58524559
12.40475783	122.1607991	15.6564342	15.67555845	39.8990846	0.027711414	52.3573287	21.83329506
9.108589955	58.16200785	14.57654487	15.58584046	37.2249146	0.01777255	51.4512546	30.80359402
18.45644842	65.96885836	23.46542206	10.90304222	54.0872881	0.008743461	41.4183796	53.5220951
11.86696942	141.2819276	7.969414559	19.85881713	60.8761092	0.083650332	71.6116569	55.28470559
13.19456081	85.73935829	16.2784307	11.42206579	34.1749033	0.010341505	37.3279926	19.04526729
18.28968724	66.01001473	14.52715677	15.96141053	42.2583055	0.011241257	47.2957199	26.45789261
9.884839851	64.54948838	8.724435975	7.425069376	53.0998419	0.003377532	26.073237	62.89504373
14.10061136	60.10688388	10.84564909	11.25780252	32.8605273	0.014522655	36.9748424	14.11997286
13.90162281	50.94881995	6.399332492	15.62775973	25.1435592	0.014005888	25.9068921	5.844071297
18.26517445	215.2081371	12.4942943	33.28081795	57.9698132	0.059116562	86.2039833	62.66055989
21.1038559	64.20958712	11.51621476	13.55126613	32.2498137	0.007829304	60.5832942	34.65634911
22.06005586	112.138517	28.82801044	19.52429316	48.888015	0.017923241	70.026737	69.33063877
25.51921211	72.67179007	21.36589176	13.15933325	38.9807942	0.008822514	52.7119319	22.32965032
12.86197882	71.20059366	18.34947919	15.10949308	39.4618443	0.026655025	42.2269245	31.82383667
63.03912737	115.8853988	11.63950107	9.504784466	41.5356577	0.002263008	56.9948463	40.84903137
10.30452337	97.25754382	17.62510147	12.51436077	37.8023286	0.010272405	53.5955414	27.27464311
19.38145908	127.117272	20.23676524	13.74317735	40.9212	0.008780743	88.2889514	47.77831737
18.98187811	58.71212267	15.06559145	13.59101272	34.3806801	0.022551728	35.3402878	15.29822004
6.224267978	61.67122248	12.65933651	6.424506283	39.9834847	0.009864961	29.1919236	10.33076944
12.90544823	80.00172264	12.56063059	19.77836495	37.8419914	0.021775651	39.4257034	37.43564169

19.75020457	76.7351319	21.09725394	15.11660741	49.4260911	0.0094685	57.3331891	45.80478698
10.40915853	60.99438447	12.83350518	13.61091791	41.8045626	0.01978635	55.1522325	33.28892257
16.1299501	126.6266044	17.75777005	15.346308	43.5322197	0.012226706	68.1753167	42.48276475
15.16650559	131.3859258	24.5519908	25.36159081	50.4120744	0.007712171	78.6540809	63.61907827
15.58007572	60.22873008	12.30799468	8.90384199	39.2956576	0.015009573	28.8659996	25.68562139
10.0494278	44.45648849	15.88672674	13.365507	42.6496537	0.00635294	37.8364479	48.58797942
16.36335398	164.7700817	17.06211382	16.80528238	58.8813465	0.02891871	64.1287992	50.76106945
10.85256191	88.14545524	13.92966139	22.92670933	48.1565003	0.036487534	70.9387747	46.99574667
46.29364604	81.40712702	14.61041696	11.61869042	42.724053	0.008599445	43.0441077	20.03226491
7.74137333	79.89287652	11.25884285	17.92998294	39.9456391	0.028992866	45.7741083	36.29549524
22.96098713	66.34055276	12.22636916	16.20518087	47.6729029	0.011025312	36.956887	26.0032593
14.38360722	58.74274966	9.644038541	9.595774339	30.7582218	0.011022232	29.3981502	25.20824358
17.6474063	123.9782588	20.15547504	22.60349055	41.3645736	0.015009658	68.6218335	47.41999604
25.77927699	157.372776	20.35843679	27.36926389	50.6011229	0.026284681	89.029849	103.201034
16.84567804	24.26389022	7.2813235	7.257574679	34.2302418	0.003660028	34.7490804	7.112247025
8.140914737	62.58368069	10.43545672	7.581413784	35.1319321	0.007688531	37.6595764	16.05064035
19.0609586	86.25876691	12.04403305	12.97121439	36.969713	0.016957901	33.1420294	19.15604974
14.24834485	90.42436153	16.67080438	12.24484143	46.1732534	0.013763067	44.1543323	16.73880288
41.89780579	165.8284822	35.11077283	14.09650258	49.6779749	0.007529157	113.318373	43.08946661
18.92439748	69.2154434	11.70767912	26.23652694	46.5733956	0.019811625	50.5603155	33.7710765
9.691684445	50.02562151	9.726471535	8.375136801	40.3852921	0.01073646	31.3754718	16.59214669
10.89848075	162.8835209	9.663747751	21.71510302	39.7814348	0.044158295	45.401842	19.8914735
17.59068471	73.06770176	9.060471799	11.03598495	37.501995	0.005489912	43.1326963	24.37278773
16.39947455	150.7048125	21.66724199	16.91070933	35.1216666	0.01294777	42.0269684	35.16694977
20.29380448	100.1293514	15.78598814	21.67552316	40.054005	0.011913125	61.0700975	33.55046545
8.439798197	37.52794734	12.45751668	10.33172983	31.8261903	0.029395731	23.7058219	9.805434647
9.962675379	132.2901868	17.84070074	18.08404322	44.6607926	0.021196957	59.0783807	32.61341688
11.17949582	125.6754536	14.84113204	29.91203307	47.2297477	0.049189608	70.4901448	50.370251
23.99119406	29.58121408	10.54254007	7.79839282	41.4061019	0.008751464	21.1132087	13.43837045
6.674205897	74.41593675	14.03276962	16.39155024	42.0711072	0.029871784	38.7361405	34.16736033
10.65268569	105.3690095	14.70688466	21.12866846	43.9088572	0.017217484	64.4685765	49.12797335
23.0200668	197.6138276	25.95799665	28.75211114	42.9569001	0.013802056	83.2760806	64.8134682
8.153856789	43.01050265	11.76556429	14.85797905	34.2138198	0.011302123	45.3924044	45.58100661
29.39411707	77.97346441	9.926521885	10.69476512	35.4054321	0.005892615	38.9869058	24.18344218
13.36172915	92.17683073	12.48783096	12.98588993	46.7265153	0.028568844	44.3387542	28.95145428
33.45908411	55.9455942	7.080991559	9.085745091	40.5780194	0.005552041	23.613969	30.33877966

10.45800726	36.57059461	9.143731818	9.24744182	32.0833207	0.007465452	33.5939546	18.06786856
22.25406976	61.26681983	14.51758807	17.79419288	36.5305545	0.005893033	54.1109983	38.74428168
11.83741608	48.84939721	12.48972237	17.959693	38.0321668	0.009352184	45.6231878	24.08788581
18.33114244	126.8014925	13.97184953	22.64054776	42.5194522	0.037040586	49.6393326	32.14730883
32.84739744	33.94653486	8.438385655	10.58518025	43.7258718	0.004262955	34.1199638	20.12477429
51.70067446	103.7369744	6.043173527	17.08933943	46.1484355	0.009985551	27.40525	33.46410917
23.42108059	82.21030475	18.1054445	28.20786108	40.3234767	0.029926541	71.4506639	57.46422996
25.11829218	136.0608749	16.64277871	16.73609005	34.7960562	0.009398572	67.2959823	31.27974221
22.31029107	168.2325635	15.64382075	37.08572161	43.9621167	0.025050412	70.9465365	62.52930424
8.513898193	44.59716281	15.0162138	11.8299646	35.794647	0.008744655	37.9713025	29.67692936
43.92546253	98.84617157	18.63842365	19.33036408	41.2151449	0.010717094	59.897666	30.39295711
17.45921192	59.30857346	10.8490235	13.12556204	38.4809229	0.016706006	38.6958343	17.68219427
18.17724146	81.92867131	20.43374495	10.85509474	36.6164913	0.0054428	57.0227425	19.70618715
15.91202282	124.0176914	14.24200236	13.32317164	38.8331314	0.010547246	66.4607233	38.54952316
9.277970039	53.0403359	13.77369676	6.682652108	43.5310326	0.012446471	29.3843863	28.41110666
15.74687616	107.5522945	12.16551824	14.45173765	38.7115726	0.010941643	64.1589727	36.85323963
38.43943965	146.4789452	10.48559561	15.2585395	47.0244378	0.009833199	69.8847569	46.85691237
10.39122848	66.63008501	16.79083848	21.0014962	37.8541486	0.013781412	53.0288454	37.90760263
9.150290703	53.76544745	9.921982969	14.08246858	35.0349658	0.029426296	28.6091953	18.14442513
3.771165258	22.57051276	3.901965749	4.96430319	27.1834221	0.005733124	12.6869402	13.03453237
26.89337722	72.58177433	14.06211389	14.50222325	36.4412399	0.016655718	60.5254445	50.39725782
39.33385832	55.17001359	11.23405889	14.45586315	40.6051028	0.006725702	57.1303288	34.58384145
8.648934746	37.48852743	7.61605352	9.770183882	41.248913	0.04097972	33.5088808	51.82966637
22.54267459	123.7693821	17.44040587	24.08624779	43.207689	0.018987128	74.1262164	56.70104182
8.214203684	104.261785	10.09911077	19.28129677	46.2006316	0.053414207	76.3238444	47.25895562
10.7995202	58.68407526	8.580124998	9.57859487	28.5991611	0.013971106	23.3597292	17.7417979
18.66081263	88.73138204	20.57704209	17.26672477	38.7576272	0.009011347	76.1553457	42.909744
15.12849773	99.24010151	18.48602831	11.71838866	37.217248	0.008745306	43.4310628	25.20593425
6.67664442	66.67998401	13.97844605	13.88365655	47.7365404	0.028188193	51.6877794	37.790162
6.57966378	54.53079205	10.84968593	14.96208729	36.2354918	0.012275194	44.041945	27.71287479
9.596521828	85.59503872	17.51802928	25.99370365	44.4584613	0.030225701	89.7214418	49.18120723
20.37032343	142.9153809	27.17200955	24.67072831	47.5189798	0.014298416	79.9895668	40.03813196
18.97314486	73.76077851	12.189241	16.01192553	29.7619123	0.007591674	37.65439	16.40837381
38.39570829	69.14277305	8.486761505	23.91672317	37.7489473	0.007810475	39.8005335	40.39499878
12.44026902	79.04024227	16.73454551	10.62942535	40.5289744	0.011090263	45.9882355	27.13738185
10.22344664	46.19432134	16.54775424	8.732734492	29.6924603	0.007487334	27.2678274	8.408142199

22.70985407	158.1184175	16.82145573	14.3372167	46.3219157	0.016170415	74.350855	30.61004809
10.42967583	37.36147474	10.39262986	9.35091955	30.9484857	0.007144976	29.688252	13.80204825
14.44030802	90.80420795	23.83157708	15.88501725	52.4127096	0.011067404	76.2931569	42.08971699
19.23586186	74.57720691	10.61213694	11.89659015	36.5306162	0.008048977	51.1947626	39.57661331
11.60723472	138.0514582	18.27290074	19.85186226	36.8263342	0.015437687	73.8446572	52.54761033
26.4869736	168.1815136	16.14427195	11.39256667	38.9253131	0.006678505	59.211663	32.69036692
27.89239129	82.34191735	15.67546467	21.79166331	38.6151904	0.011615352	55.9476025	39.12001545
14.65110553	275.304512	12.56962821	23.36017787	47.6013098	0.03647221	89.2324807	50.79908441
9.109308124	79.10297739	16.80171419	18.18168633	40.7849355	0.016096635	50.5762232	42.70259027
24.27710703	415.2603934	16.90012749	37.75738492	72.2519723	0.120608994	140.104784	67.54465986
10.32975996	107.9833462	15.0922721	20.60740568	39.655848	0.018018406	46.204894	45.63128264
13.94417008	122.4921681	16.39502232	19.73814775	39.1616939	0.018921204	63.7022875	31.08656994
13.40606052	104.9437974	18.96941006	9.937057612	36.0733379	0.007426934	52.0960734	23.93460086
43.83026709	190.4532558	20.65069917	26.18339356	54.4091892	0.019174629	88.9111401	64.11268696
19.70809036	102.7984268	14.27340091	12.72945513	37.1130783	0.013201575	45.7222401	23.23404142
38.60782927	107.5714844	14.53941267	24.07991396	37.7732443	0.006733507	31.9016645	12.61056249
22.35720822	86.57501626	13.67277273	15.40197922	39.3562492	0.00785695	45.1283431	40.49929247
10.70252471	90.98304139	14.43277837	16.01431665	40.2434291	0.024938651	48.1791292	25.35335248
16.70178191	84.59629313	15.91812162	14.64662907	42.7428524	0.024674995	49.5715921	32.99955281
7.3799797	73.94334233	17.09227831	16.58582555	42.0788078	0.059722215	41.8492804	45.47805608
11.26178559	73.10383372	12.17931449	10.06141884	39.0873422	0.011905231	30.3367721	19.91241889
10.48129545	74.16060694	12.68680229	12.89038374	38.2156778	0.020199038	48.5846027	30.55406419
15.50209298	90.99998736	13.90130838	12.3273022	34.4681935	0.011145147	53.2882982	39.22701504
11.46108535	93.6774676	10.99253539	9.739699416	36.3540374	0.012166099	53.6419839	22.62294874
19.47886663	99.32279324	12.58485958	17.31500446	42.9747951	0.017961009	42.1430139	22.65469133
14.41497663	104.8991557	16.61491645	19.41396164	44.6160266	0.008757919	77.0973251	63.24169695
7.483388428	49.55694689	10.85078172	12.57842032	35.54375	0.022427341	27.6869554	19.88049743
8.494367419	55.03898813	10.30510715	15.45990508	32.09823	0.027033526	36.2187062	27.8898853
7.772493523	48.86926532	13.0337473	6.399187303	37.3020116	0.008821243	33.6296895	13.77606181
20.09628651	100.6132221	24.21397041	19.52469453	45.1296666	0.011309184	70.9712307	81.16698105
28.12520419	73.96866233	19.55204963	12.27731703	46.4962179	0.00807377	44.4102919	46.61446734
8.998238723	140.2300305	14.30773318	11.98243395	41.8699319	0.030524845	46.7739577	31.06221531
16.29613598	115.7735025	20.54963765	18.70220832	49.0409674	0.022336589	54.069392	40.01633621
15.27446953	169.3110893	22.87089215	10.49163502	46.8579246	0.006426894	66.8025577	24.34474892
15.32856183	133.6557065	12.53619708	24.01432381	33.8439789	0.019911042	48.2046179	28.04341874
25.60632064	172.0860311	19.04616808	21.49409294	50.4175789	0.016200509	78.5321138	30.41167507

17.38918059	146.1585561	9.90961001	20.61833332	34.8255799	0.03863644	57.9491199	25.96999311
13.14523969	71.18286345	9.640654359	8.20471395	36.7018585	0.009689995	41.9480889	28.51645113
11.41704625	192.8927005	18.43463479	26.26134317	43.3774224	0.040573049	58.3856274	29.40654688
10.29412534	30.18400293	10.10014978	11.62056454	28.6378092	0.005079689	32.314401	38.34583378
9.943794679	225.5863062	14.77459062	13.54008008	42.8476413	0.036328838	46.7289975	34.95291087
9.017977169	244.7105824	20.92294704	34.45000394	46.7757745	0.019723409	105.899546	81.45063412
13.69673505	66.75485486	10.77596579	27.96131176	38.2973938	0.050000576	58.4640991	49.74360613
10.64844939	45.38467877	17.5650863	8.180921385	43.4379005	0.008189359	34.0943505	39.34060852
10.74896093	87.04808198	15.75610273	9.822431662	54.5616533	0.013535451	52.1239895	135.10835
6.39146798	53.45525322	9.780643093	15.57611778	31.3789857	0.009897878	36.2077573	29.15604706
15.06721784	108.2950764	22.33296303	16.34902218	50.4537964	0.015326932	53.4536244	84.90053063
31.23319557	231.9006814	12.20486863	49.53681444	49.0072766	0.090315559	122.072635	54.90163073
14.99179228	72.95839141	18.45199837	17.97033673	48.5214308	0.023394005	73.3906182	102.075478
13.67236941	87.22022642	10.39640931	13.5393184	32.8425221	0.014189959	40.5018366	26.99870078
9.07634907	65.52445371	3.6615301	24.75655953	33.4396642	0.022266466	32.6213691	62.04648655
8.457779393	71.31616122	3.956253044	14.10891147	36.7609166	0.018635001	35.6970688	45.82289293
12.24750064	82.76223458	14.33493804	10.84697691	46.8155334	0.028983015	42.5850446	42.65942446
17.29174588	102.2163724	23.65407267	15.92170756	50.9457151	0.011624434	35.6764893	38.57901855
6.551700058	112.76015	11.21947469	11.31688886	41.9961309	0.018901029	43.626651	39.87134494
27.61705997	172.0613138	15.88361929	28.73077096	51.5573705	0.011209954	71.3331071	74.12802619
8.817127097	59.10880741	9.99355925	10.12024682	34.1633257	0.013246147	28.7410446	26.88608752
8.50563345	54.38297057	6.053122072	11.87735587	33.7359463	0.018430957	31.5406616	25.08554804
11.37385272	114.6412017	18.33303196	16.81587869	43.7855625	0.012702793	56.7419691	26.40588979
7.576647641	76.57052718	11.36301809	13.0363027	41.4529073	0.022337645	39.6864234	29.40695505
15.1774308	55.74606417	12.28967927	8.460819993	44.6374189	0.0100369	43.733401	59.31258805
12.29631413	107.3713638	10.8380699	9.821178382	39.231875	0.014482905	39.3226544	38.85018206
15.90977049	68.38753964	12.55872524	15.64288366	65.5092546	0.022123726	68.406928	86.39736627
12.04976927	88.85893755	6.957276332	19.81498064	31.227906	0.020299932	45.2692281	33.75671528
12.64378094	95.68535936	16.10710809	21.03595685	32.3768517	0.011493144	46.9789373	23.06241004
26.17645101	53.43653956	5.005255703	11.03495888	40.9403526	0.018518019	33.3611485	47.3981239
12.24122683	195.1547925	13.87628929	18.83346723	42.7256555	0.016105787	64.4358414	57.98570311
12.5118186	46.88811539	12.01581891	10.16694827	42.885625	0.022587873	41.0167274	44.72242266
7.267571896	69.21710023	8.423648195	12.76090852	30.9786883	0.010599199	36.9822419	19.382321
15.69919177	87.95045567	15.4877239	13.04589152	36.7566426	0.010655976	33.3984934	28.91884114
33.33708199	62.80101444	11.82340688	13.1084381	61.5782932	0.009460893	27.0951357	29.20674821
9.64986154	96.98242631	9.32417667	28.34941605	32.3901864	0.015680856	58.4486263	38.97856665

18.97620461	62.70913335	8.23218111	12.57771847	37.3217925	0.010238939	41.8933827	22.31348915
5.741197798	54.82007884	9.451262343	12.71242027	32.3547576	0.015502112	31.6394915	28.58495327
11.89998349	57.61025543	9.425450528	8.774692179	43.4028568	0.008803248	34.1760683	26.87502807
10.7507791	33.06489576	11.69640958	6.624390297	32.6001908	0.006074718	38.0334668	36.33632913
8.691872174	76.36933979	7.802351301	13.56006054	45.2187172	0.023101695	30.5660159	21.96861472
25.00658488	134.5971961	21.25652069	14.50691788	54.98281	0.010858567	70.1616422	33.04248754
10.99366132	108.8981593	11.04822613	14.80667983	35.0186931	0.01902108	48.8049274	23.85166069
25.46298673	152.5616195	13.35419993	26.55390281	39.1015683	0.025030985	76.3760229	73.46215635
16.12147759	62.99293217	12.84646608	11.43027289	32.6162842	0.008597318	31.8588384	16.04048963
13.65922507	116.1978832	18.3827632	20.00281604	38.3316724	0.017303905	78.5598153	26.02254591
14.07302334	84.08959286	10.938172	7.244900228	46.2060289	0.004820701	34.1389604	25.05455701
13.12469744	98.9291322	4.877104819	24.79731335	31.4890624	0.016057085	40.7986677	65.30040395
26.22086389	79.48434387	7.055250431	10.69687019	40.5513732	0.005001668	33.2514714	67.27426442
17.62629403	44.75436204	17.40758406	7.460414788	45.2743855	0.006544107	36.9080162	28.65547819
21.3885981	103.7582475	17.10586561	10.18080629	41.348817	0.010936064	64.0599147	55.85792732
9.076186435	85.88012043	8.224186069	15.17753363	33.3079379	0.028126169	43.0117316	26.46946789
13.32149557	140.7760677	17.42751594	18.96083357	45.4850927	0.018921691	50.6957979	37.54584855
17.61708765	245.0279817	16.83600262	40.35152491	39.7220848	0.024507439	70.4920801	55.41191949
13.73546713	72.67999748	16.26254163	11.2843285	44.0115863	0.014029399	41.5792511	52.0098269
5.875664047	26.2266013	9.342711462	7.139885485	36.0673692	0.015578927	18.5661659	14.22783765
13.1237483	77.91407372	6.530447834	14.7981471	30.7685517	0.021606015	42.0723315	64.65081404
12.74719387	82.61321359	9.180347341	14.1562592	40.3311304	0.022515202	44.7408796	45.22187408
18.45063026	230.7977553	8.703885504	36.81445234	37.6709284	0.03719591	50.2973471	75.90451673
11.60420166	15.8687525	10.21663166	5.514318908	42.7444477	0.005185279	26.4676466	16.24537798
12.27525199	48.20192638	11.07447524	18.69656388	41.6691315	0.030329233	48.2537013	68.64705667
9.336533938	111.8903095	13.16926565	9.363097306	43.5959465	0.015296713	56.9439817	36.48172497
13.2936417	136.2864812	18.32435927	22.05902159	46.0652354	0.014919822	113.782502	51.71857311
6.888215401	72.14249926	12.30058532	12.54507928	34.6919014	0.021574595	35.3127908	22.79150262
6.492890744	41.55831121	9.067533755	10.47607951	28.9169512	0.013026944	27.1365357	18.30148517
8.995102964	51.21049097	17.85667153	7.846936128	31.3746108	0.003108644	34.4823906	6.574474449
10.28496443	124.7026187	19.52037671	21.17034344	48.205667	0.018796984	58.0917045	62.14208162
12.04124937	34.44337085	8.194615491	7.466800948	36.0705384	0.007085717	25.7263391	19.94114883
7.344732526	56.10113735	6.900910461	12.20544059	28.0455743	0.020410546	28.0756385	17.46538126
25.58260498	110.0755837	17.91384662	15.40671497	51.035629	0.007353131	49.8589115	46.29865689
7.370136875	51.76999818	10.5743951	11.4209334	33.5305945	0.009085621	36.4462027	29.79513718
10.63854021	85.43935156	9.330872104	10.47085296	32.6067188	0.011853623	27.8997131	12.51666777

11.82665848	42.17027884	14.36221929	12.44834844	41.0444827	0.009970694	43.4793564	44.6118644
15.3741384	66.20477633	7.832178859	10.68211457	28.0395936	0.003453281	15.4888877	48.37497145
6.069329901	84.46123878	13.88876225	20.03043436	42.6017836	0.016805101	61.6085024	84.86633778
15.59644883	115.5407544	14.51238789	14.38538163	47.8737345	0.01961549	46.1152227	39.45137916
8.72563383	89.85181353	12.41953713	18.6976973	38.6519092	0.024072233	55.6142743	45.3907585
10.83448332	101.654341	13.38994137	17.22215882	41.8995275	0.022414572	65.3834882	24.21996242
28.10562907	74.54238451	5.320563915	16.03883158	46.3724566	0.035682277	30.0922764	37.61150309
10.63312956	55.05421027	12.84872296	7.669686741	43.4099985	0.022971437	27.7063819	27.16047756
5.900506545	125.1936201	14.09714764	21.86796736	39.4366287	0.025510342	71.9725107	31.95797095
18.71280107	100.0186342	16.56946072	18.31910623	51.2454368	0.025300945	62.1840436	51.44900469
9.094554719	78.65055435	18.6147015	11.56206004	45.3781056	0.023656628	38.8849868	45.69255648
11.74859367	110.5567167	9.051540681	17.7068832	30.9490265	0.014587729	37.379075	37.7281229
21.38712135	41.02894228	5.76660075	16.79137336	34.9950919	0.022128871	42.0388321	71.36207768
10.16087772	200.0080467	13.23591588	18.03963865	40.2127468	0.016214521	57.0144596	47.41283493
4.88626706	15.30722764	7.309656108	5.581349703	35.2269919	0.00510557	12.59555	10.47190851
6.763868291	85.31456536	8.334996493	13.3442936	29.3279941	0.028532636	36.5958982	19.67526701
5.856448912	44.53253205	13.37729795	13.09297402	44.9302897	0.020388963	26.5981226	23.15981737
23.85085512	108.9531728	5.079074898	22.45116953	36.8080108	0.012646032	42.4705161	57.96681373
13.87930839	104.4883704	15.06768968	19.52881489	46.0845262	0.016525361	55.6369356	34.82074337
8.377269792	59.31731982	9.478085932	21.98671895	34.5456214	0.023479626	39.4140771	41.83525534
14.62772231	226.2238824	15.3785486	22.02602916	44.0218215	0.038991294	79.0716011	47.86730822
15.63684325	95.40437233	24.81828748	19.23124755	46.5986213	0.021668086	49.8942778	35.61534525
12.69984101	162.1687953	8.624985166	29.11932571	35.4571146	0.03544707	58.3642328	57.05099861
12.95496283	104.1636764	10.8365556	13.73105447	40.4136335	0.007909359	45.6299121	71.22793483
19.59749305	146.7478837	7.762304539	28.8709782	34.6155812	0.018767887	48.0665972	33.96161577
10.03144301	69.7299635	15.3940895	12.83294304	41.5652177	0.013634783	44.5963183	49.99542295
10.18725276	154.2888084	16.25468367	27.38096181	38.7397956	0.011778718	71.9601887	53.01947818
18.76367662	188.5808656	22.97442229	75.91007659	53.9978912	0.034549857	99.399979	79.15123165
9.00607485	86.55144129	17.92023762	13.82921268	37.8920626	0.013177706	58.5002561	29.1536401
13.56705036	162.239006	15.7687777	20.42297335	47.6845589	0.042671343	72.4640803	51.99634916
7.488513635	162.1005729	8.206047245	24.75231725	28.9927222	0.027073822	46.6605748	50.51042213
9.329882723	116.1226968	12.28116265	12.59343617	34.6296371	0.012758559	48.8474136	29.79017876
8.495590341	19.1582663	8.331561318	5.814435777	34.3516101	0.007587687	24.5472847	13.4543822
11.64904571	121.9915564	18.92440027	14.01876135	41.1143655	0.010410517	59.3548449	24.30775919

BPD-00008900_1998	Foretinib_2040	BIBR-1532_2043	Pyridostatin_2044	AMG-319_2045	MK-8776_2046	Ulixertinib_2047
163.7081114	4.267310538	171.8012441	41.35098538	229.4172622	60.44649441	13.17923585
70.27394944	1.703572281	125.0969624	33.91070553	101.7568686	30.03690401	8.940643589
157.7342693	6.974458273	263.0234409	32.14163296	145.2325405	36.7383625	12.45489618
87.14210511	2.291022842	116.2803915	14.44443376	64.94913487	13.02277634	8.671244248
138.3685335	3.100410474	216.6072686	59.7334081	240.3535949	35.27044452	16.77833781
41.6487776	0.938557584	80.68570503	18.04886545	57.59436175	9.00731177	4.870912265
74.49790253	1.264279063	139.0787234	35.12094164	126.2877804	9.079800163	6.164593203
144.1914956	3.171282151	202.6684571	48.45740997	177.1853706	68.15304745	9.977332058
157.0897443	4.037103322	234.763791	52.55386958	204.322986	73.96105123	18.36502811
61.63415485	1.790253303	104.9358219	21.69501456	66.16618297	12.43865677	8.584241875
61.75125189	3.219598796	93.57627302	26.62129476	102.8748847	12.69099936	8.034390297
109.2750562	3.94016365	172.1763896	34.50366525	135.3749172	20.24895927	10.66197671
69.87334667	2.575771909	149.1806263	16.62058956	118.0300846	10.71884472	7.035568112
137.1435546	6.773042771	165.152561	29.41404678	125.1258789	33.72156926	19.04449187
88.00642614	2.591710148	158.5083556	32.41921821	81.17221035	13.8741979	11.99079486
76.00671912	1.960938173	122.1689209	37.88978061	116.0973246	26.48710174	11.27168074
42.77874257	1.773984809	64.74252732	16.10706388	114.8674532	8.34239206	4.056805855
96.75950614	2.904030776	202.1562155	43.19949517	384.7823699	34.97968052	4.840698791
72.91241299	2.161152809	178.3358578	50.05179395	101.3080152	13.22456246	9.590965237
44.03769948	1.124990551	102.3896451	20.51173047	55.54721059	6.468206991	5.950224507
91.60725098	3.094558256	138.8362198	25.38130188	128.3268651	32.25781203	8.225272156
139.249567	6.867364516	198.2849251	64.75752796	323.400839	86.40488398	7.399473844
85.90702316	1.801208393	175.2109879	33.70046954	121.0097308	28.67756187	8.178135349
144.1022653	3.506180269	245.0985994	40.66837371	206.4575816	64.77666016	26.76509719
95.7051453	2.62967527	163.5643561	30.33072912	110.5535243	26.42172415	12.70634015
95.29999049	2.819975132	193.2721416	27.69518162	156.584483	33.46069646	11.42114345
158.0665627	5.70538562	197.9673331	51.26387737	189.8603675	60.8951827	14.42302464
137.9086117	3.093601454	257.2459044	56.32794772	225.4495923	52.21956087	13.7233889
64.65577264	2.656520305	168.8135835	32.2916732	109.8839542	15.72638528	9.636677355
78.28716292	3.890020984	150.8722136	16.92313541	107.4867314	13.55486032	14.03262267
77.16756504	2.998087064	118.7055584	30.76887016	160.6108825	24.89017844	6.061188525
79.3347844	2.254622883	277.7918948	43.38843675	121.9565166	33.60058215	12.21321664
159.3084093	3.812770057	284.4259911	54.01242472	300.9625265	54.42484004	17.48221652
85.03689762	3.264495959	106.673135	21.92230423	91.89955179	19.83829932	7.739973234

155.7546362	5.183954864	179.5957551	31.44290506	168.3471354	65.72702456	10.62293274
99.83039468	4.879334786	246.1051329	53.37113238	197.0852061	93.70116625	10.58085996
55.37401899	1.734104127	88.52721761	16.01172264	42.58452422	3.074610007	8.617083831
147.9164355	5.28989111	334.1240804	73.96733674	270.0153189	104.0956189	16.11794961
87.05155706	2.419833599	164.3860524	37.16639581	130.6958913	21.29914422	14.00121493
101.7059788	4.42464084	129.4213532	23.30287392	195.5878619	25.83973343	4.834945903
95.62427823	3.996374504	161.4289173	16.23683849	100.8146881	15.18567708	13.38549664
86.22257349	1.600945831	150.3093944	39.31533261	81.76867475	19.71135412	13.73413157
128.019913	3.011897343	200.9925053	37.01221249	225.0270334	42.80897114	9.567585239
124.9725342	3.421535292	105.0109347	17.17311898	102.223552	33.04864338	11.73007236
135.7438977	3.833434839	125.3866734	25.37861061	192.7160287	38.50871017	8.691032571
38.48342936	1.961337757	32.13354933	9.672719045	84.54702808	13.22104066	4.371610033
78.455198	2.046673851	122.0328822	26.31960601	90.51995217	14.32989523	10.47740522
99.98368301	3.293729997	180.9329469	23.68139205	201.9329189	24.22624763	12.09984949
74.02268593	2.044185876	124.5061085	24.09423594	74.48346296	14.19092169	8.953957152
63.46522723	2.266354671	114.3540379	21.96006949	108.6723476	13.94784214	8.953103636
48.35157783	1.579877023	73.91207449	13.30963901	46.39432228	8.00492908	5.790564519
80.12008449	2.018566448	174.8607476	32.4601973	87.81195704	17.81332765	11.89278359
69.47898163	3.345677333	222.0423499	48.70144084	119.4989848	23.35293774	8.680223787
69.63766139	2.208683465	136.9637393	19.93340232	76.53810924	18.22292811	5.790556558
106.8802187	2.851349177	126.3980536	19.53403125	131.742423	20.22012805	8.835279344
156.0404276	4.759072377	210.209984	36.91283721	139.2598657	24.38865503	10.65148048
45.96071283	2.203667266	127.2661189	19.87933622	76.74091517	11.3579599	6.332485118
59.15948053	1.47711775	103.1017852	22.85978563	69.29637697	9.062243026	7.593385382
299.1377361	6.038577087	243.5559516	53.9777285	311.0405345	95.93598095	19.17424767
123.7741137	2.518599117	156.0098519	22.74919724	200.5553849	23.97752543	10.38145605
172.5106759	4.213726005	234.8873259	73.64788646	218.3669211	79.68512158	19.27318913
111.292422	3.698306749	154.0071564	37.55595258	147.2155864	51.78561357	10.67296481
180.2783797	5.46980745	212.8199505	53.42968986	294.7822779	134.1585976	13.37959796
92.28082565	3.767024824	152.8128492	34.70178137	229.2613832	30.48952404	4.682204381
89.29672215	1.435188334	96.42264185	18.77258404	72.80711385	14.5526638	9.577539716
67.61094057	1.722604209	116.6031503	21.7879683	56.98888332	8.605793715	11.61739086
108.6086892	2.823630788	143.3390834	30.92235821	249.5780743	24.15506437	6.629841313
471.0838454	4.968268063	379.1446282	122.3373036	321.3576314	121.9076445	51.73573448
71.26134677	2.022322567	196.7415343	35.79093524	92.42552659	23.25936629	9.216741786
64.0135231	1.93875557	113.978394	14.9765411	52.06333685	7.8670053	15.97901369

98.81277714	2.184021043	245.6403256	39.05461969	171.6352177	38.53028406	8.469200477
68.87997015	3.780834687	88.43644934	14.55824494	76.67614817	17.87386116	11.56888348
131.9024476	4.291081619	276.0690442	46.60127339	209.0688992	38.13561575	13.3743499
269.9189718	8.601351509	323.5555837	74.18494408	341.1569063	66.10390131	12.23773828
82.05501442	2.858663456	143.3910769	40.97716252	86.59314247	16.52550924	9.27898828
85.30478009	1.634327887	113.4118526	28.89225753	69.58231514	14.12401422	12.07554971
64.8049878	3.794125935	108.4372087	26.33250838	82.03646592	14.1721386	9.532520505
134.2699183	2.784734259	182.8105841	32.09370702	177.1615428	39.69074938	11.68132867
123.5327902	3.626916886	124.0836186	27.50903392	171.9890112	67.60134919	9.792659494
91.3272869	3.737227353	127.8843865	28.0500829	99.95667151	27.50864742	8.127238077
180.9781172	3.67571942	227.163522	59.28909063	170.6690294	16.26542399	16.88098104
168.8484847	5.102374646	215.3648427	56.39723717	156.6057236	63.30263531	13.47325414
68.56402199	1.787553811	104.0812076	18.91791276	89.88179195	18.83545488	4.906087798
80.73870952	1.330783387	108.7631636	23.80654469	69.2446046	9.631584512	7.78184472
136.1694436	5.718037248	195.3925785	37.37038075	191.3759134	78.9593202	15.44335668
85.5350153	2.79169879	141.058504	28.99501775	103.933029	25.98275573	18.87100458
79.66615185	1.893035005	75.29187417	11.72378248	80.24311498	10.39859918	8.909071623
82.31483964	2.789803667	119.2863181	24.869903	90.87642587	23.40394908	8.415384234
113.5433432	4.793408845	216.353632	54.93070665	166.0129888	23.59838309	9.897465662
54.58530665	2.596254087	150.1449561	27.60043584	75.66298147	11.21955185	7.264377989
103.4313426	4.506219468	150.6834377	37.0999785	196.0552187	19.06686521	8.938177624
56.21401125	2.144358668	108.1221618	25.05858805	99.30667393	24.08996251	4.271729754
139.3226067	2.789127766	175.8235269	34.93046427	139.853529	33.19310702	9.829834636
96.18401986	2.219576775	137.391972	31.1367162	116.5101022	26.29968463	7.039423123
73.439243	2.479376959	119.4864622	22.7587435	114.933301	17.46755946	5.33824191
99.48616004	2.319605934	167.945178	28.08269301	114.4478667	24.31417722	8.405641251
76.40292549	2.615700833	105.7100426	20.6520726	109.4625553	20.1568999	6.898928459
74.87694927	1.604295604	172.2663737	26.96319871	142.3632575	11.18901891	9.802791691
123.4598197	2.554604535	119.2044271	42.91694493	104.3209094	25.74508578	13.9764032
129.0793027	4.072185973	109.7786109	26.48956158	68.41069168	25.22131757	13.92242789
97.41732395	4.380070902	137.763474	25.91004096	203.5712952	73.12857769	7.927679048
124.9846363	2.054893959	191.5516705	34.91656479	115.7331581	29.19240569	17.12676209
44.23668301	2.246220701	58.40973528	14.3463008	53.08006946	6.307789932	2.404152199
86.87029902	1.814456771	86.46121462	19.58949855	96.58278467	26.18159977	7.55018177
103.4187164	3.368154596	72.95942536	28.33502051	158.7459068	11.66492955	9.10038535
110.273038	2.16263825	191.8719694	46.25102119	195.8028515	23.38386967	11.83822711

103.442675	3.121169914	147.399036	24.92459566	113.7796128	32.88475598	13.49036278
76.17592044	2.071245609	103.7415897	27.04681033	78.8960085	14.17552051	8.549597488
52.88929893	3.136329238	108.6643673	28.73241544	78.22927862	16.68880075	13.25332283
196.9514393	5.264602173	249.0154169	59.10758819	344.8807581	97.28526054	9.368096649
119.0094372	2.489355364	146.7170257	62.23335842	136.2312049	18.99224156	8.655987895
82.09412974	1.600269209	124.7486116	22.21221546	87.0062026	9.193191081	6.222325285
176.1775795	3.247727155	262.7266	76.61366075	198.114436	60.93238819	19.70662927
82.46981499	1.744441767	102.9694664	24.13616381	120.0172409	24.04119498	6.132491958
174.019669	3.436323867	209.9079426	44.89607185	177.9589222	34.61759373	11.51990212
94.11454195	2.635871852	111.4470336	27.79664207	78.47226334	16.12370906	10.81802508
127.9225618	4.019734046	110.0793609	26.08404093	90.96122831	25.03248445	8.239926385
158.35285	6.783846063	189.6349082	36.34506112	160.6083785	51.94539061	11.8199044
97.85690894	2.088508128	278.1891444	56.13284381	183.9884572	25.38629587	12.1636627
56.94699468	2.715267241	106.9226313	23.48092724	51.53050276	10.61083955	8.006969337
59.391289	1.87957911	57.71688937	7.896551677	77.56736398	10.73033771	7.002873726
75.26458631	1.549024376	159.9479451	47.34325314	179.6408879	8.239402836	6.329245832
79.71992233	1.60818557	153.0801011	19.25532051	114.8707975	14.79568966	8.457327016
64.93807195	2.281275564	151.980452	28.97910987	132.8904481	8.562165018	9.741955583
57.30159863	3.55182519	142.7184228	26.52301508	134.9597952	11.64134019	7.067073265
107.7624573	2.333418252	161.6758602	35.70851317	154.1624276	25.28475294	11.20174527
148.5610572	3.933738734	240.0937887	55.01892844	230.1899252	29.21267528	11.94589017
74.20579418	1.885503835	161.4627792	26.10634365	99.93625885	34.41248933	7.250613156
78.69174174	1.580258521	139.0765604	25.75761571	132.6162052	25.31955637	6.982572053
65.30890483	3.145779224	157.9238014	22.15899066	135.4034931	20.58065701	7.89500274
95.05834765	3.488611702	80.98319596	17.10703095	63.95126278	16.57477274	8.803346137
64.97678021	1.690637849	161.5189871	28.15301235	91.48809296	15.8208316	9.463075189
274.1878037	5.204307413	254.1050755	106.3962597	262.7807446	50.78005679	18.67960665
88.51934229	1.708170975	132.0176167	24.5717074	95.66694544	14.84917681	9.08646878
95.45820535	3.085703094	136.1644585	53.81011394	83.56239581	20.5397195	10.0556249
169.1517701	6.969584125	163.559146	37.81201444	128.3384186	26.35901209	16.46008097
111.1626295	5.592611936	241.9507247	56.25016805	121.2493782	26.29407669	9.158689798
81.68763718	2.8018328	183.7545202	29.89883083	108.9947567	26.54999703	9.028068937
93.40520082	3.518195603	71.26379558	21.51364656	79.89020277	22.36861048	7.057720226
59.81235712	2.507238582	131.7682677	34.02712366	94.75159104	8.31069049	6.854628427
93.48531167	1.782031682	164.1969023	29.42005914	93.58548525	29.73285461	6.513671332
144.5724864	3.170782403	230.5352652	46.69493746	189.0700055	29.65627786	11.16478711

113.9300844	2.080980693	223.9906519	42.10418972	198.2892671	43.56530734	7.94252916
105.9037407	3.518858943	183.7160412	43.27316811	150.1254014	48.01327865	15.44672367
124.6859242	6.186621284	182.133187	31.63733511	184.1313124	44.67393023	10.15150299
116.2847059	2.131341242	190.0663837	35.95342402	112.9269786	28.45716819	11.27417535
250.1022271	7.410244616	310.3423372	37.42025815	513.7417692	67.7671104	7.809720713
80.56958581	5.137706766	125.2167733	18.17801816	86.63305575	15.07022777	15.01108037
127.6683046	4.362486892	186.8858215	32.86895883	181.6415024	44.41020961	10.56489781
91.87965365	1.756152679	138.9301004	24.27067713	78.86707955	14.13917368	11.59045275
85.08367682	1.182129263	129.5187781	30.28752107	108.4755443	7.941532606	7.595188265
80.16290448	3.796482781	149.3007606	24.7407043	76.38884369	18.11667821	8.608015694
117.7284214	5.450822778	227.4514344	62.80584333	160.7387682	47.40460761	11.18443224
121.9389807	2.607149701	179.7866647	35.51259848	205.2160043	47.11207967	9.268425967
87.21467577	2.221663651	174.5048011	26.20869955	123.1318463	16.24933659	8.160292966
177.2206365	8.358271401	138.6306347	25.36065378	142.1281679	47.33862272	18.81646926
64.93424905	1.226188376	92.05352384	23.98564344	77.87154724	7.885882578	6.808999141
151.0866494	3.920116728	185.1820933	28.59463905	170.9451186	45.21704485	15.57797399
37.58828737	1.417542905	58.45016299	9.408930888	99.16477405	3.285519351	1.331615883
113.5620975	5.0163992	220.631741	36.37128662	162.4341487	82.20019407	14.18519488
105.9573341	2.814565161	162.0785036	35.06007024	117.9664814	32.14402733	16.2577013
68.26636553	1.420803751	107.3477643	18.1690656	85.54042693	18.66162137	7.273630575
102.4395447	3.417869652	127.3986104	30.71695144	311.9355397	52.9864582	10.48505166
69.47480265	2.378113205	158.1202556	25.27020564	103.9294001	31.30564968	9.862404606
117.6763243	3.484087878	145.974029	23.92360786	100.6130682	16.10154431	12.5670648
116.6989858	2.905402431	151.177904	45.53050705	146.4461522	34.32123806	9.902558923
85.32606278	4.172838917	141.9073468	35.6731183	98.66149193	20.38705328	10.5948975
137.8115938	5.648373669	189.4505531	57.30727386	216.1556973	55.750926	15.26871736
47.97670574	2.554946535	127.7006658	14.91014772	77.23925783	23.51892349	8.930635078
88.70481632	2.215874089	180.4562342	42.27921264	115.0874394	20.04705825	16.23628406
63.9161015	1.84442168	115.1480066	24.98724371	79.6321057	10.89043468	8.049491143
38.98962578	1.115333481	155.3556715	24.40692175	122.0103299	16.70715798	7.283802972
77.00146687	2.539697034	99.82828042	27.97883041	81.73826879	20.77679325	8.438595413
85.47927762	3.491478764	157.798543	26.21280171	157.9929221	48.47258521	9.975601956
82.59819739	1.490201503	105.1018046	24.34944749	67.45463932	12.87687051	5.739618705
158.014607	2.5259678	301.8275805	41.34826166	263.132024	46.69524614	26.2983953
77.361549	1.739990257	168.3943388	23.56498897	77.77903265	12.16377599	11.20571497
70.52366559	1.726306944	104.4116084	22.65344004	79.22744869	32.18840364	6.716351036

54.84939672	1.383308157	66.25384524	18.70245347	70.24717689	11.02199611	6.924590851
88.44637564	5.675672484	186.3964018	22.58950112	182.8853642	18.44510779	9.895124949
108.5477813	2.619359181	146.1636871	32.03682126	114.0962411	20.09705894	10.85050475
131.8339875	3.419631073	223.7292825	49.42857001	186.3008912	27.6346167	10.00390052
98.80349042	2.044735803	121.5931903	21.08441251	98.38568021	21.89994317	7.513579385
246.6249999	6.043199603	229.5502629	60.85260117	250.1783329	64.50344616	20.173352
155.4046375	4.863492105	200.2297194	36.44100163	164.8761634	69.79109089	16.62262426
80.40402213	4.875906194	186.3440641	41.33349736	205.9990501	26.317588	7.694850151
70.42723044	1.918467547	87.81093524	20.71817065	75.56023553	17.15540478	5.96435689
113.942667	3.921961986	165.9468343	38.54899932	140.1505283	39.74405189	16.58541368
78.12740329	1.665000044	134.4415998	28.66102301	137.5545103	24.21191014	7.118169648
71.87929569	2.368958532	129.5662906	23.65600527	87.1238477	20.5516554	14.10802444
110.6210601	4.991932929	100.1524835	28.42586031	118.1335922	32.37748507	8.158317129
137.5216137	2.387969372	179.2411887	25.33775476	119.7081034	30.24302369	11.84013419
42.17820823	1.339394611	78.31718325	18.06637674	120.2290315	9.817899008	11.77282642
77.77035166	1.682648656	115.3132947	23.41719733	74.44281737	12.30459145	11.58951559
72.92344698	2.256869421	139.1438264	27.81385659	117.657234	28.56570001	9.746027734
128.3619408	5.803869644	141.4396803	20.85616379	129.1163889	44.1838224	5.966645274
76.52188842	1.853003654	111.5406344	24.6350595	72.50062722	13.15584274	8.754440506
52.64496874	2.780065575	80.17383611	14.52007779	73.08898401	14.80664555	4.98959989
103.3931642	2.172941246	178.0154969	30.69328186	134.486738	22.06260901	11.2474237
168.9202959	6.340715573	203.2890993	34.46506636	235.7620025	88.55496074	13.26940932
132.7655105	4.196338129	112.2056047	16.18881517	97.74538135	15.88330271	21.60694849
99.51193151	4.636158392	150.4161537	28.90948618	105.9661206	12.13041596	12.30616876
233.8243383	5.836042224	246.691844	54.56383721	352.9330864	72.64286499	16.2853632
89.13075806	3.062865242	222.3448313	25.89739759	114.7334509	18.6200811	16.23981933
236.0511683	4.709048378	197.4437429	27.43468181	293.6210173	48.89593313	9.761262463
47.76041384	2.260290478	61.51752954	7.060562053	85.95566216	10.21552682	3.764461377
56.70892106	1.786649004	75.09148583	11.72506399	61.5713613	15.94977959	4.765367424
97.63872827	2.546477479	160.6339674	33.04019616	167.4154119	30.61783055	6.678876926
51.07473913	1.387932392	132.8826611	30.07707565	83.57639248	13.69072741	6.452185338
94.84600317	1.793134382	127.0936842	34.57118545	116.9576496	36.17728112	6.856741071
125.6513288	3.903579362	177.2574737	41.9726717	136.8524437	38.89720189	12.49534245
72.07752978	2.05593442	88.91474455	17.49533689	73.85133631	24.55747478	5.629993476
147.7305395	2.517292401	159.7144733	33.93784723	192.5219336	54.8037325	13.36421601
82.24495419	7.873508252	172.5099511	25.28127039	119.9127298	13.99617066	14.52769557

92.51176992	2.027063573	161.2957236	35.37427005	162.3074589	23.52690888	11.61353154
79.21358687	1.857589275	199.7985244	34.08736546	118.7739314	25.16596993	13.16730591
74.27543028	2.549915025	110.5775338	24.79858733	79.84175025	20.07202629	5.570449964
151.2645338	3.576596384	255.0020652	48.90920712	265.0856759	63.12782612	9.136836211
48.74037675	1.826498952	79.2155028	16.92435608	99.01539686	26.55268929	2.240894141
56.48613279	0.940303626	99.49070145	21.44043855	57.97641744	9.743331975	10.79558672
59.60030739	1.945556807	165.2473306	42.02837989	126.6101541	20.50852479	11.27396068
81.49536497	3.371873452	157.072819	30.9289283	111.7023327	13.0586125	15.16779138
106.4062187	2.466205247	155.5718402	26.77460566	164.3710034	53.87397648	9.399298056
133.5741744	2.581474	164.4006697	30.23317624	143.3889919	31.81306375	6.710264823
89.6695446	2.447936853	145.21146	27.14710785	148.4172631	19.4850174	8.742977496
178.1415531	4.217170838	326.7369483	50.00950609	245.4326551	47.13662405	15.59332548
130.9409983	2.739134483	199.686305	31.17682124	219.061121	70.34340245	12.74824677
98.85235765	4.126895447	156.1826183	32.61671607	149.3763759	35.54872152	14.52767115
66.31344583	1.490818472	63.3909857	13.47330482	40.33017373	6.713883529	6.904982032
99.14747141	2.275654311	147.3976373	28.31331699	103.2578255	25.31162793	15.78379988
59.7385362	2.131333627	130.1910656	28.55941762	98.16222356	11.07386687	9.96839759
70.27028465	3.124329069	135.2115105	21.71626912	128.5604453	18.84480903	15.43701325
67.64113831	1.844785751	119.9102652	22.32744284	77.81384542	21.76555516	9.534657994
96.12751605	4.75309736	139.5373399	23.71480181	85.46514752	19.38146239	7.375477657
86.78355318	2.598507995	113.6289579	15.34963214	145.1472443	27.02350773	6.359914093
99.09021627	4.111220838	116.8889625	32.91202119	111.3272253	21.79676602	16.23025818
54.65459474	3.487524395	236.1690751	56.92178543	157.8545801	22.61721513	7.06168243
124.9569702	5.602663784	181.2912963	33.35431821	238.4818578	59.87516468	11.64655455
85.1142265	2.769506281	103.7258983	24.89195563	66.85386484	9.686471009	13.46511965
157.8316481	4.658853484	268.3512505	75.46010654	227.5463393	52.29499628	22.15332627
101.7414314	2.73824039	101.4460131	23.92388867	101.9289481	21.3672512	12.61505522
61.5173845	2.165834418	144.5305989	26.83270533	88.85117482	17.26939736	8.15242518
74.31927557	2.171849544	84.56980496	16.59797622	73.63817353	19.84662167	7.398914974
77.39039058	1.535509242	137.9577257	36.22281935	111.6366437	30.73099926	10.04696028
115.0323338	3.423300821	162.07494	39.08825205	195.8363639	67.91786079	8.220350344
67.75278444	2.794336053	80.09163303	24.87429603	90.52669503	13.13978952	4.134970669
64.4899876	1.435227889	78.66077148	11.7091835	67.36170435	12.91820743	6.849562763
81.59343522	1.997192411	105.5383315	19.645409	87.1337283	23.93731098	7.37061043
50.26226605	3.222068458	127.7979914	24.27418879	104.9651496	5.266236456	3.708383273
124.2544487	2.717605742	209.6022165	25.13134259	170.5431017	27.35759048	14.18948183

71.18986507	1.601899275	127.4557856	28.08870043	87.91617341	15.29101651	8.560124747
122.1743701	4.25685841	196.7643135	34.13244526	164.5514321	50.13603147	16.2036806
92.94493371	2.631328096	183.9150824	39.82169826	147.0819918	33.79941139	7.441627637
111.9031103	3.10434854	206.6708169	38.86434995	173.9303844	41.50302715	7.260776463
97.59363516	1.840722169	170.2627677	45.72013053	123.6172765	21.62930351	12.17252393
97.5291189	3.564394182	119.1668115	21.7438533	88.57765203	29.53027078	10.2467696
81.10854051	2.650501816	146.9666142	40.08461472	101.0149424	14.23238862	14.00493403
79.05709623	2.24423305	171.7769116	27.59406066	111.3776278	14.85762789	10.99609221
127.8048685	3.549457265	184.9660005	37.61343217	185.693342	8.261446972	7.204029479
223.9817235	6.908936715	268.6082734	52.11440385	271.834192	167.9231131	14.32410053
74.79469525	2.964531826	168.4672329	36.22738174	119.7906265	20.2833953	6.814118384
113.3405645	3.47370221	185.5930493	36.09862047	135.0931881	33.19381539	11.76600777
56.43622859	1.094390205	60.5454597	10.22362557	91.36429932	6.152602271	9.526903113
151.1078897	5.305409987	224.0533473	39.89203442	168.466776	42.90702968	16.72232436
106.138722	2.212514055	147.2573288	23.27714214	126.2036776	20.72894823	16.7523264
37.04782797	1.081931039	111.130488	32.34133647	69.13191913	10.72079659	5.09306876
84.77055779	1.8995511	141.5166687	28.64701875	78.74190996	14.59511449	8.601606496
117.075422	2.211756995	117.1083976	24.25831886	142.1641981	32.50750753	10.13598503
104.2694265	3.822407814	131.6637126	36.53307187	185.8269837	26.74203027	5.04572267
96.6976788	1.982999842	114.827449	35.5673854	171.1310138	16.17759444	6.447188906
82.25437397	1.533751972	158.1940672	23.80926251	114.7512327	27.19997921	13.31202924
84.30543239	2.759253264	118.8518047	26.43766857	78.46420082	25.27350554	8.964885202
31.6931358	1.325198924	107.7621219	21.33162628	101.0192375	16.96446018	2.8221861
104.5621792	2.535399559	135.1030308	29.27047044	117.5888756	22.82173304	15.33536844
58.30224779	1.10113742	96.87791566	18.83066589	71.04435778	4.324387349	4.809552248
127.4222365	1.421536713	200.9152166	56.03961955	310.8554391	17.81195496	6.113233663
106.1217303	3.550570175	164.3699365	29.45762229	139.6086152	39.14692706	9.258520946
125.5591875	4.109092625	232.5751643	43.57653938	188.737585	61.20655329	16.7970216
97.47359428	4.597222149	155.7808434	27.15640062	94.98269205	17.74905088	12.06292047
63.87090027	1.653517159	103.8361059	18.03725992	68.21113397	14.19465013	5.939446563
136.3221176	5.377688414	310.1531441	67.70929097	210.7097523	29.65624859	7.450542929
95.24008088	3.010770971	168.8081944	37.6294768	190.9490509	73.3755233	9.413683647
147.2548633	3.325079778	234.9657675	51.4571535	245.3149543	60.97590097	14.77082526
90.678177	2.295122719	80.38356839	19.83839619	71.88474004	19.01944942	9.778706712
40.09617435	2.31436694	65.7180149	7.151445129	78.16123325	6.266588165	3.087826023
86.48317963	1.26631935	108.1645757	23.65569759	75.41815179	14.86314292	7.682464738

101.1118336	4.007203164	131.4177917	26.17697935	118.3813678	43.39882124	9.515701829
83.39405936	2.053925203	123.4514494	31.13080234	110.3610989	28.3199558	6.142798928
99.44303502	2.291380817	215.9975546	44.05285534	142.9506935	31.77350113	13.05725471
109.0141284	3.360067602	191.2457574	30.40930179	240.933897	71.66987723	7.630750333
82.59355456	1.951219023	72.99698389	17.82731051	51.82574137	10.72848513	6.843109433
62.67148346	2.130704959	97.28761923	14.55562021	105.0995394	26.93990853	4.293312507
85.08542899	1.265040422	194.4323549	34.77784525	115.4558288	25.17920679	10.44152393
101.5395388	2.233571858	143.1036547	28.37384996	142.3889523	23.46602794	8.34890098
151.2989203	7.495677097	150.5152178	28.42734034	149.6170419	39.36021655	20.22869186
56.57555944	1.356799269	87.63602647	18.30922037	71.31935282	11.48879663	6.332429994
144.4948795	4.017324892	123.1324937	26.99375403	179.361127	39.07989269	10.60584003
63.98870054	1.982780992	96.86991524	19.5119903	55.53663166	12.53278217	8.146463656
81.63284389	2.808606542	171.9204045	33.01968341	166.1116371	37.87431031	8.232320356
154.9770752	3.233746586	217.0049421	42.90906072	200.7178356	38.51558663	11.04841005
64.09745243	3.163182184	61.38285216	14.18045076	107.0890224	20.50227072	4.008864367
60.42051821	1.286437069	100.1345719	9.959927009	107.107568	10.39003764	1.485098648
80.0750602	1.711301357	121.6754558	24.50540579	64.69442121	14.7788806	12.5490228
84.66128614	3.060266196	101.2845665	24.09825955	85.80217458	29.74672307	13.43208158
185.2919491	8.511858434	273.7906374	47.37254411	328.7641777	130.0406218	18.76386385
83.04544114	1.815134094	135.5676	32.61679639	112.9605939	22.95262164	9.76832781
60.03702113	1.821267456	85.78989731	17.38842373	66.38598224	8.259209256	7.25158445
78.48591859	1.957925238	164.4002355	27.66631296	106.3067909	19.84994708	11.92919063
81.2603744	3.508505105	136.0513411	43.22294131	155.5712969	22.16754828	9.668515184
103.8713969	2.714471054	154.1395751	32.39294035	170.781496	39.7394165	9.88745863
166.916768	2.442582053	182.907757	40.22019048	134.9995355	32.04743718	11.92518033
74.15653409	3.365032408	98.7873494	13.90256787	83.48435887	14.68785451	14.30411833
96.85710507	2.468767362	122.9216854	32.28153648	112.3096458	26.91954846	9.934030054
91.71694632	1.270980092	145.8610962	33.59293687	162.8758902	17.24768701	5.954864426
83.99711483	4.56659444	87.49909838	11.77258056	51.23002038	10.88419178	11.33420034
84.71955177	1.553793275	103.786484	27.81066115	96.5392983	14.44797742	8.078105312
111.7611086	4.675580026	168.8974726	42.45907906	212.1954517	35.54379791	12.447971
114.3903695	1.769948854	190.2513795	51.86569495	131.2673493	31.78352979	9.305456601
102.8497347	4.265371352	122.5119481	24.29308669	182.1658791	32.49516484	5.552998957
92.96331547	3.505448624	185.3467007	52.12725108	143.0886634	24.5817422	11.36972161
79.09746762	2.907026367	100.3498552	34.92108925	120.0847234	14.20712522	8.680026575
89.21757747	3.537576284	158.6154256	40.33469117	122.9279472	31.93065789	9.755100727

59.0554293	2.196278072	102.674967	23.12940485	84.51122991	14.87490388	5.410213039
98.80450869	4.00136185	218.023718	31.71312437	178.0022661	63.1043814	6.427780425
86.67261088	3.684431827	120.1865128	22.20479537	137.1478287	35.8313344	8.10284314
83.83856676	1.527206487	114.5119769	23.24175919	91.90286224	11.81433915	6.008123909
111.8306257	3.79592837	123.5107529	22.01720593	173.4948218	25.16613757	7.414467377
112.6426204	2.605111647	154.9703287	27.14118872	119.028909	16.68319779	14.57914587
141.2020389	2.423579295	194.9439239	50.06573536	196.4768807	37.75737796	9.054388586
170.2187962	2.463937013	247.8176017	46.29242378	213.6034474	33.30338031	15.17550479
127.2517439	5.122588285	207.6419187	43.35145524	221.5816851	50.50024506	10.26195497
68.77104629	2.283461019	109.7623035	22.55623967	133.7529547	23.08163817	7.291336592
124.5334464	4.230065535	169.3524115	37.31651345	144.5710228	49.33773873	18.43509117
89.42139565	2.711869903	121.7181801	17.72158183	95.22810693	12.29849015	9.307782917
116.5254389	4.020318126	208.9429335	50.70368503	215.6745898	50.62867231	8.51366581
65.84792872	3.299444504	183.1308641	33.48496054	156.9417369	35.20804626	6.726436498
80.61864435	1.956480577	96.48816818	19.31864086	101.4701339	26.11906895	5.461068094
78.84883181	2.361524879	181.8526243	42.70878553	167.0553557	38.9484484	11.72077811
107.7062154	2.487453321	241.077898	41.0279044	135.5946688	25.57974235	14.86672854
70.23521991	1.826122959	124.3406333	24.45871196	93.52974526	28.70049082	6.534250369
48.23291296	1.063123655	82.6503472	19.64676105	45.68952768	7.331705628	7.672010355
16.99394746	0.838879814	34.37427828	8.678660905	25.83194416	1.573972714	2.223751807
95.68040867	3.55608025	184.1604866	28.93868257	138.0686746	19.98071523	4.502336618
148.0767859	6.315386383	194.2889048	47.52171392	215.6198909	42.26470599	14.96532015
71.0803173	1.250805902	84.58770497	22.27131903	121.9689957	9.026949254	6.063469591
95.2301341	2.112619751	204.2166141	39.95441167	148.1637072	38.5019231	8.577870173
81.20249103	0.990812877	188.8646478	25.37102171	165.1031294	8.873169364	7.164158989
37.0516931	1.152013974	93.60313345	17.38184786	58.4276938	5.420599682	5.303620296
151.2337082	3.46444016	157.0746113	35.58136854	236.7761176	91.33770506	13.25293239
78.42828031	3.187490472	121.5305416	23.11261206	86.83621202	26.88808246	10.54664629
73.968699	1.324633259	89.07353077	16.15730429	92.31193609	21.3612585	5.955275089
56.53698108	1.122589192	109.6957997	28.91018966	99.0675129	20.04509072	3.740060924
105.080358	3.448431919	151.33917	51.69423615	215.2867822	40.67177157	10.97902564
117.6327196	3.478469711	195.1495829	37.62143835	189.0251007	59.71547325	10.44142
88.91025903	3.683930812	154.5682847	33.82685225	138.3043226	36.67828466	12.13147778
168.3791656	5.193253923	194.6405187	42.44144925	229.1051977	34.69833742	11.48762714
101.9949026	3.517319745	151.3086224	32.57233425	106.9369592	25.38378544	13.25055964
61.12783343	2.073412715	95.37557711	13.1680015	71.53579955	13.40537868	7.615289733

124.2550714	3.032543641	173.5964462	39.55984304	138.5383348	19.9976252	17.42718761
59.49464462	2.124492041	98.37713216	24.65486734	63.4969652	14.5113974	4.4580278
108.4230245	3.382337916	193.4190513	40.17401638	137.8792166	63.01401588	10.64135622
125.0206404	4.852801688	191.4215398	37.13336037	186.3121278	63.16486622	13.70150764
100.2588876	1.96441267	192.9152505	46.03656416	172.4604762	41.42937196	12.86760788
109.1351796	3.17159512	269.5520371	63.8215366	207.1697184	45.12016783	7.107099016
113.3495045	3.845223819	169.1884368	36.3477142	130.5358428	32.65377386	10.34195964
82.50232676	2.207144408	195.6349175	42.97284516	203.9990323	18.64102588	4.825812882
86.63551955	2.846021231	120.3539566	26.74106157	138.0794274	37.18636511	7.704550726
152.2943854	2.912307942	254.4166725	83.94425922	349.6780674	32.96346615	9.818722028
100.0436822	2.706077054	135.549579	28.08663211	139.1728408	36.53385777	7.470142977
128.0382081	2.462305604	208.611778	41.59389684	143.6337687	40.90003063	9.373799019
71.83987355	1.81718942	171.7712227	26.64065465	121.4944222	24.27250609	11.9331205
165.9908135	3.951210602	272.6738849	52.02192785	146.1358115	38.62304162	14.36662336
59.73772919	2.666067675	130.4311884	28.73191598	84.14647008	26.16158177	15.5039638
102.0309301	4.31412354	200.25936	34.93456156	151.3746458	27.30806397	17.39081895
92.00869486	2.494828749	166.8578916	37.20940483	140.0990575	38.80861732	7.280639969
92.59550888	1.909156573	118.3481534	25.90459434	89.35949085	17.37866535	10.4485038
78.9027128	2.412576348	165.5471182	41.57284604	148.0272401	40.84162603	7.667869444
106.9861848	2.594284219	120.4322183	24.81966698	113.4776759	19.34896724	11.98235221
67.74662392	1.833312467	90.17722239	18.07561208	67.575091	14.05825108	7.546654734
79.66539401	2.375864611	115.3762227	28.17824839	82.33758222	30.4907687	8.177467772
75.3815335	2.556138034	165.5057269	22.04764988	96.91974558	16.51446081	13.57334656
88.89196093	1.545799065	193.3193924	61.57536579	140.609475	35.73776416	8.857216901
115.7465228	2.254396931	112.504152	28.18486496	75.86492391	13.95331278	11.74102555
84.8123704	3.477797955	173.6099609	33.74531638	173.7626408	54.82064143	8.93245012
62.03199527	0.944335397	69.00888002	14.48986409	53.8571247	9.442388878	4.870250943
72.31730728	1.39182655	78.65592265	18.4583627	67.35708418	13.47920573	6.737755704
55.19899844	2.466586275	91.52713622	22.0422004	95.1798522	26.33501466	7.051619071
87.35695376	3.264632936	206.6911617	27.26428091	157.1634031	53.88521328	8.125920094
84.55915611	4.087224306	160.319073	25.08395644	176.0802102	44.1271197	7.141604206
56.35060713	1.844365533	88.4625824	12.14100815	85.57831628	12.09806041	4.436443563
92.91358093	2.117394386	110.2798554	21.14886426	88.34599135	18.2886901	7.377088973
122.5312509	4.039396909	200.219192	44.23341678	218.5289617	56.36296767	10.3778504
79.11413394	2.008341442	149.2800551	27.67371605	94.45846806	15.60404864	12.1882267
119.885473	2.130981681	167.5764889	40.71427343	108.4857922	41.0751247	12.18968523

69.11889385	1.832237048	171.7938795	28.46591759	103.6876179	11.18127067	7.737686487
74.12836141	2.691304183	113.3782681	26.02116395	141.0173246	25.87618239	7.973934465
79.54771115	1.42930514	136.3102579	32.25156028	97.7316689	13.61148286	8.675192147
60.97955429	2.864720047	78.98142352	25.53903263	114.7323987	28.38682179	4.263163938
65.83011851	0.923061209	139.2279446	24.65348851	105.8713932	12.61176052	6.412499151
121.1116145	2.736873156	240.1341996	47.9351538	239.07331	67.88906882	15.01237507
101.271694	1.967841308	121.7957476	26.21598669	117.9264382	9.590544562	11.66123576
73.3345172	2.221381399	110.8377584	23.02850928	105.6674327	36.50838992	4.549419914
117.293792	1.820530213	123.9621596	27.12461718	211.1134272	21.30765974	6.222642683
54.09806802	1.646729291	81.64640726	19.74054441	80.14165302	17.58373785	4.724525106
125.8616075	3.198813628	134.019558	28.28469416	189.6754532	39.60910535	7.775186107
203.7619646	2.307851912	231.6664409	41.16824815	219.6949686	19.62220432	20.08711387
151.3013709	3.672492278	174.5697974	37.38004633	286.8584461	28.16076574	7.766742512
57.77593592	2.763164817	129.0551003	26.40362903	125.4101655	18.54811374	4.979403623
72.58869933	1.435275153	90.43463377	21.64315995	127.0993915	7.432997121	4.705814379
69.91313306	1.66502358	142.6299086	22.66920494	196.0901802	12.24412067	5.944078741
95.72932232	2.39924803	128.0339368	32.23148092	103.9247229	17.34064173	9.511911557
104.4902461	3.830949122	160.6565953	25.47660176	169.0150411	21.39766691	8.824507349
59.45208723	1.218274783	116.1547669	18.30611002	172.1250564	12.19672603	3.84837836
217.1146227	4.006187395	222.2085962	44.56107326	252.6858153	46.00255691	10.8383438
57.37238501	1.470710794	91.63707559	16.69541985	64.80550364	10.19603325	9.427896459
63.0211018	1.642208882	86.21722968	15.50765102	43.8845107	5.628861847	9.839766933
107.8348852	2.94491173	131.8360366	21.05783462	122.8381455	23.6966529	11.44394579
76.90090852	1.273281294	81.34784941	16.91259858	76.31202539	13.13728884	6.665507027
88.42805615	3.200528947	112.9672996	29.17540519	156.0321902	16.67559223	8.482390349
95.14486889	2.062619144	132.649644	26.95417619	136.8420076	16.06316061	8.357504187
111.2899996	5.178188279	164.2128471	42.85502244	378.5541142	43.31483422	7.077895895
76.77920875	1.940232911	148.0440969	24.07993735	126.2849994	22.53046819	9.798064548
117.2411122	2.464868117	164.8881056	29.35594664	178.6534452	17.99562162	6.420486299
123.1400927	2.910332637	90.29414018	21.5907451	99.20773047	11.19817861	10.02729837
143.3069943	2.482037107	205.3146743	33.21565396	154.3478532	31.91203758	19.58158863
72.60653207	3.332903262	87.54518279	22.42753116	171.4105457	20.57259964	7.98134319
74.26219593	2.401158925	97.85765388	21.01175158	81.81218044	13.18536631	6.772109331
85.22737248	1.986405553	115.609147	15.13402744	103.0308969	11.21134928	6.043783137
170.3589846	4.162613888	113.8649331	24.27878539	253.180719	20.87791139	7.819577045
82.31093277	2.012871882	181.5160126	30.91972468	194.681622	23.48194706	7.553273547

97.87295837	2.572687672	127.0799234	29.97668543	131.584734	11.97691982	8.78218655
55.46357821	1.336219763	80.32457097	15.87392892	56.87502324	10.28981026	4.883954991
85.80525208	4.263741496	100.2647388	22.52997392	110.7256483	25.96500498	9.898682449
66.32432405	2.667453864	60.23777758	19.06040147	119.9772634	23.00683831	3.085192445
85.93044174	3.597509248	118.1331621	24.42147888	166.9810715	22.89103119	6.370626502
147.0577667	5.127009843	158.7198723	34.26658908	217.8875516	52.13692715	17.82138018
93.95356317	2.167300341	149.1452305	30.17481525	140.5706566	25.6868515	10.74726466
115.1629398	2.975992375	172.5379935	34.77890785	213.3639855	25.67156123	7.711055427
86.2420988	2.93395814	109.0645673	21.52760571	83.52611779	10.49419245	16.72984276
105.2658459	3.63166331	156.2191882	32.32832152	166.3979366	34.97647383	13.41514817
65.87438559	4.307442821	123.9619442	15.40250079	105.3789272	18.08515582	2.946653528
80.71814617	1.547018728	123.3560469	12.58914387	124.9675434	10.56293449	7.961729777
143.8366565	3.433532154	97.03980833	16.46084497	155.187941	19.67350458	5.117227444
92.22427961	3.297938493	139.3744614	25.15463392	145.751227	32.26050428	7.316315635
126.3198058	8.199931187	147.7142941	30.80299124	205.209856	50.40761958	8.070762101
63.49110485	1.529483537	120.2672118	27.37266127	112.1964409	10.70105086	3.846183095
105.7225476	3.639266598	154.0341174	34.06554278	152.7816273	31.24035321	8.885595584
168.9479095	2.362197413	194.8733141	34.73676597	195.1088354	35.25577249	17.5504273
83.01496776	3.428939632	120.1437989	20.08564591	124.6586857	19.21185623	6.246912609
53.69541461	1.773469869	54.27895824	13.29720576	90.15763241	11.83068268	7.62118981
77.56516585	1.848142209	117.0486873	25.90879102	142.6697743	10.58589128	6.635378383
71.0960186	2.025362568	125.8083713	21.51490758	108.3108856	17.52571782	7.539325306
121.2707289	1.51339418	221.6468869	30.16002392	136.5989148	15.1069446	14.8328125
63.77729056	4.308771302	70.4667541	14.01507491	95.65327656	16.76544798	5.65303557
89.57883262	2.674398673	120.7835336	22.89993972	182.3409237	26.39119848	7.504633032
101.3298839	2.293449409	113.5774956	29.16085989	100.1341157	22.21523882	14.46216814
189.3616937	4.730786498	197.6307924	49.90387415	273.4490791	74.09258717	14.50287281
63.34872222	1.106278124	81.32543862	17.13600108	96.9772066	11.83675769	5.410841965
34.41865078	1.150610593	84.20471413	12.03860538	85.82487864	12.05896574	3.375361935
88.24337032	1.774180059	108.9756648	19.11388484	117.5319352	30.96827058	7.571483161
105.9388084	3.153837145	159.4343345	32.39133907	193.1605304	39.14680757	12.57233838
86.63490626	2.323721522	90.11532842	14.68808917	127.5480018	9.088333501	5.096087395
46.34712738	1.053979997	82.16920801	19.5568302	53.04324489	9.543816925	6.226057927
142.9107433	7.561014208	211.3660966	40.64239394	234.641223	36.56871092	11.19695883
55.99736669	1.915900982	111.2417734	24.716193	129.4286389	26.73635251	4.461034479
56.39625208	1.810850915	93.39322875	16.18159074	67.1666752	7.297619437	7.047960706

117.4646813	2.617722971	119.9217267	23.35861025	174.8487571	32.12725416	7.193942784
51.50956921	2.804262991	95.48014961	16.52591777	131.3339879	16.54599866	3.551416017
103.0121691	2.565792303	139.7084255	28.89334691	226.7591254	56.13347529	6.765618987
91.39146369	3.742511991	130.8594051	26.71207623	133.682269	15.34488418	8.26359867
83.21899358	1.440014088	124.5461715	20.62608412	118.3838222	19.76197177	13.72436966
130.8181271	2.444047491	140.8878459	37.76151806	133.527163	29.13761118	14.10233709
82.88840971	2.216744374	106.3303291	27.0298323	101.0693816	11.0188359	7.519673417
71.7493073	1.711669125	83.89227342	18.60515459	65.96557092	7.53510384	7.940805942
109.2805956	2.520095449	168.8138735	39.53101271	210.1542699	40.03164405	12.14712045
141.8222802	3.562473048	149.4010697	36.811993	204.3462881	28.00312993	13.02627395
92.55798338	1.988180993	109.7338923	26.92657434	163.2394176	25.09755852	7.815997917
74.41172211	1.656969441	125.8534071	23.69708929	138.8285054	14.286563	10.1447273
61.84397106	2.327825984	108.5540957	28.85809139	126.059347	11.87172579	7.481675209
73.85174536	2.13943044	185.7750884	27.9415977	117.6959034	22.87667903	10.73489251
52.29487728	2.213894201	46.02653132	11.55095066	124.7263646	12.38709236	2.887992757
59.22344384	1.1281939	101.1294065	22.56828921	65.56178649	9.139139236	8.761048349
62.55632561	2.228627809	94.00228376	12.56301935	116.0191737	7.898440794	9.712871721
120.4105105	2.742176791	149.5674639	36.21906127	177.227458	17.0702733	7.1185361
94.35997036	2.131413409	116.5475903	29.44650385	137.8516312	22.35649237	8.098973459
71.25721134	2.114561284	108.6970553	31.07167426	123.1871	17.51973481	11.00623425
95.88124276	3.743184224	198.407686	28.08446556	192.8757877	18.74603031	12.89734899
100.1372093	2.320439507	119.0047557	27.79762499	142.5134479	22.43266893	11.77282639
84.32670927	1.804990039	200.9397498	31.99553415	213.3423371	25.19366081	8.399184095
57.35225793	2.357573986	144.7567232	29.94306617	177.6887604	16.79587193	4.472948154
96.32747401	1.960214527	206.2946791	28.27848938	154.2969947	27.7775215	11.56566585
79.48743868	1.82651767	137.2311736	27.2480296	128.6154482	26.445253	7.312273438
126.7801039	2.197915417	161.826456	26.24080902	178.6259677	48.08161268	11.20100118
225.7798082	4.068982282	247.9320769	53.14226781	269.9007013	52.67064138	16.33400868
91.35102346	3.100001856	144.7585391	32.56354246	147.9754691	37.7669403	8.569912096
125.038582	2.406010715	216.4945934	54.13825137	214.7014609	32.14660062	12.85479042
87.03556761	1.866694919	149.4463036	19.8480799	124.7515718	10.77168926	8.540815323
69.7413868	1.905861255	143.9756475	28.58587834	155.1665506	24.69058036	5.839350238
54.4194633	1.963939975	73.06435324	13.20246488	75.02579293	13.83509616	5.488245408
125.8087344	2.408022618	170.3869622	34.43902344	189.1352746	32.42135925	9.365029931

Vinorelbine_2048	VX-11e_2096	Uprosertib_2106	LJI308_2107	AZ6102_2109	GSK591_2110	VE821_2111	AZD6482_2169	AT13148_2170
0.119029392	26.05895865	21.84685509	229.8767334	12.49598348	81.37810348	88.28443061	26.0050674	66.46106649
0.043942436	27.18226496	25.4281547	134.2555292	10.33642498	125.9884837	85.22055032	26.70009	22.42140232
0.093535638	40.93955804	28.90991453	290.8784453	24.23970944	199.0082499	82.18037937	25.36049816	118.3282211
0.03549981	18.22311953	14.53044893	78.39991876	8.827297002	68.19753498	63.79365073	14.85857999	21.09516216
0.250940356	30.64200658	60.25425428	301.9493765	24.18781306	156.6044054	142.2557122	40.32838564	141.9406598
0.043454505	22.60496822	26.91931084	88.76815812	12.19851897	87.87863334	42.40133624	24.39661829	30.31882209
0.047226119	5.723983828	27.4262958	162.1176728	9.682452381	104.7134771	38.4471194	23.33940651	81.3582975
0.105045076	24.03729486	25.35631708	209.5296065	12.434692	96.80590736	127.8242824	19.59336088	42.47123748
0.212402234	28.00150148	42.64456269	287.5353748	23.15908249	168.4928429	260.7269894	28.51781315	119.0814838
0.024124282	18.50927333	14.79154996	88.44136052	8.646066568	73.73588643	32.73241111	17.85614153	23.55437717
0.057766734	20.92095386	21.62537459	143.6489932	10.07378801	82.69610432	58.25684837	22.88793899	32.12607958
0.075840499	24.89811932	20.06949837	161.1654645	10.04381316	102.6902895	63.44038815	24.79397718	44.17361116
0.02348837	12.17474248	8.835576142	148.9788798	12.90401496	66.27414982	36.96448804	21.35264644	29.98080934
0.299529695	70.26734799	76.61606277	168.5159091	14.08555637	99.64036149	164.3419658	17.23129924	55.02574119
0.053133885	17.47019241	21.98644625	141.179226	15.07252656	99.12415055	50.95348591	23.64386932	49.14791174
0.113348848	26.23139351	11.14726203	218.1426226	10.07456168	118.0597327	82.12462712	21.95050445	61.46965691
0.014458486	12.91325688	29.34877084	113.5885776	12.07295709	81.92152916	22.62930548	58.67306254	30.06520557
0.062965724	4.814636404	50.07071457	367.5220481	13.27798709	114.4528542	55.69293641	26.87184882	72.96806971
0.042340075	13.36710267	27.81909923	203.0782106	15.45923639	158.3839185	44.81798134	34.87426491	83.70519824
0.014182417	10.44443747	30.95178532	120.5955966	7.626203117	86.04692617	22.42973464	29.63985086	30.97483241
0.040131465	18.15826068	14.57231415	145.0115283	10.42809359	77.42641749	57.619076	20.00626145	38.47942191
0.152648944	24.5126474	54.5862347	385.3528209	15.2774938	268.3778056	139.1978836	61.47966357	76.48927015
0.026551642	8.169256507	10.25295262	202.8762221	8.993785738	113.9184481	75.2959069	21.86047375	47.20893115
0.08350205	23.57042293	11.44003764	198.896097	20.87615834	136.5790742	126.1588421	25.05410966	60.81012261
0.076326567	21.01052657	16.03969088	144.2292162	11.91331377	115.330638	72.11744368	24.5514444	38.26682501
0.044178935	22.59183798	20.71506829	165.5386547	10.98737522	120.8726076	70.66990859	24.70701706	27.17244079
0.204898257	45.70044758	52.27876597	200.9214166	23.99505029	143.8841517	134.5414146	27.81707608	85.12677761
0.066515446	17.39083624	27.94456757	227.3826065	12.72719906	139.6943636	115.3358184	25.76420797	75.09232946
0.021050894	14.05295267	12.08719312	118.8117063	8.543625624	89.21564797	58.97843689	19.12495974	25.04228946
0.035246142	32.93239922	16.12183476	152.0254263	13.81878791	68.14828107	40.79587633	23.45722159	27.21330899
0.026390882	10.3934718	11.83419048	165.4686932	7.420238532	106.1227085	41.37177392	21.32990278	39.91109408
0.024704008	15.80604306	17.48182287	208.7522233	16.3680476	91.06401789	46.71738672	18.46726167	41.02093632
0.135031798	24.05040363	20.49093178	293.8809586	14.21530351	161.2269774	91.3780015	29.50995278	52.86814283
0.026770956	21.6707524	13.41831566	131.6175893	11.40835897	69.25521781	50.26046923	16.53622252	26.88008869

0.071647969	29.32229347	14.08207994	187.7592348	17.13833629	107.7007214	166.7291201	24.10314795	59.44335092
0.094180383	22.12740281	29.20091455	418.2252868	18.87415945	195.7099299	151.2612763	28.66345967	89.32739276
0.016341692	14.66787831	14.87471439	87.94075306	11.85970565	59.74336141	17.61450905	36.89877991	18.53898138
0.123869806	21.43521368	18.59967548	227.8054259	19.1822142	153.0762948	124.7304113	26.15106118	60.31240483
0.075257441	17.68912625	9.163931305	188.4239066	13.95724812	119.9495399	48.96009003	18.16658157	51.23706099
0.070155026	22.53894722	14.65359039	182.2845768	9.900422481	98.73404049	35.46332575	36.96808795	46.0040226
0.030594765	19.24062037	2.985309955	110.707251	13.26048733	42.42333897	50.54228384	18.08473723	9.140444585
0.099535468	22.34801298	17.17253466	129.4956393	12.20223935	122.0774957	83.00384017	18.17062354	46.30835534
0.064550397	11.85708925	8.875624789	167.5295537	9.46372941	100.2206386	69.43650977	23.7442126	27.66973215
0.087884936	33.54592828	7.179135797	126.9366202	15.15607328	84.44675172	75.72638834	17.55271985	40.55289647
0.029190826	11.20485554	12.28535449	156.0727111	9.916957148	98.241487	84.55585668	28.32086506	34.2917155
0.011095187	72.24421812	12.19451808	93.76873442	4.905186837	47.68897579	23.91502547	45.04760251	14.19680275
0.03095716	12.62889512	11.82653401	106.16964	9.257468778	93.63140499	44.2711521	22.67735169	36.51142517
0.034453736	15.62707667	11.74996825	202.1456838	10.81887863	91.86096833	49.87199601	21.82579232	31.47431437
0.032365227	15.97742899	16.45805275	99.30433682	10.38438214	72.86283192	53.81235266	23.92862707	43.80209392
0.025249421	17.54823172	11.63127143	108.7581267	12.51361626	76.64000658	38.47085422	21.89052753	44.78088234
0.012346847	12.58593694	10.05747524	67.82501047	7.195345012	45.0569433	23.8817059	18.35468346	17.47988072
0.046280522	17.86258008	22.35792896	161.8794634	13.27207461	100.7389719	43.83384945	22.04192834	37.89652174
0.071462315	17.14620363	22.27658998	238.374237	20.80210333	113.6945592	40.4962902	18.24601604	66.4861377
0.01123887	11.57301975	37.97325363	153.4613422	15.06849229	75.76753375	30.00986821	27.18321067	47.87594679
0.016013849	13.78925898	8.745935843	79.44661616	10.73137755	46.53039591	35.87265552	14.52188562	9.676514778
0.031719958	16.2074537	26.63028872	134.8407407	23.43766853	67.7274897	61.59872554	18.03171016	23.63995521
0.013532643	17.27087052	11.73254455	153.2187007	8.793975349	95.85833314	30.78302211	23.40392911	32.31095925
0.020353406	14.24818343	10.78780352	103.9105327	9.291273704	78.066405	30.6888108	20.84764596	36.16704533
0.147906984	21.73041746	12.85888352	227.2458709	15.71210777	91.08966031	185.3872797	26.44255834	43.31600203
0.05316054	13.88551618	9.640952147	155.4857897	10.60340825	133.7863148	45.99499671	28.87912245	46.69058866
0.3429509	48.92344343	50.6447877	307.1726869	18.09966996	192.8374867	203.5760964	25.09529201	97.64484803
0.124693961	21.40710702	25.82796397	169.3077423	11.42668684	112.6068106	105.5008173	27.03336526	45.31977816
0.185429892	47.74372118	48.29679712	298.4947969	19.92805481	183.6054565	198.9422328	31.34565847	67.22743086
0.030616264	21.97976674	14.79203723	164.3492511	12.59196155	92.8444367	78.77059146	22.74713058	22.09850679
0.030512584	11.86311996	10.64621005	89.96888386	8.945254345	69.93535562	38.87038318	20.0262267	26.90074407
0.019428168	15.90692978	11.9556487	93.54311743	8.466120486	68.14245101	37.07191211	18.27899763	22.09123331
0.035055504	11.04174958	29.61910097	160.4398221	12.95101192	127.0346037	52.31725405	37.97722959	70.31081711
0.484480073	56.07671709	66.30174104	433.688139	27.43615463	242.2601259	455.3738528	29.00804736	141.554292
0.032425499	16.42690633	32.65396075	229.1145718	11.4988252	127.4885474	57.49659245	19.9577679	56.83085337
0.018762017	38.10445252	5.860549985	157.2824743	8.988529387	69.9766593	33.72978441	21.89988184	23.49508854

0.045111948	14.13223903	18.4155992	238.2422604	16.63874058	128.7777704	61.24613818	22.84610877	68.10398454
0.022616433	32.70445294	4.499553938	118.541709	9.941443151	55.84393431	45.60252193	20.08999021	14.79835409
0.05352423	16.48626238	12.34655971	251.7644733	11.16860224	136.9846652	85.19243642	21.18444316	48.9876023
0.938016161	39.50720121	82.88492188	342.0620889	23.03009399	217.0091176	368.114193	39.81838527	255.8484338
0.040717265	16.76705441	29.25470953	136.5057496	11.60091707	93.64385247	58.13959119	20.2018374	34.89681501
0.027711734	13.67890474	10.14636277	82.10698567	8.435113385	65.32048223	48.02347356	17.41092603	24.63271523
0.022868547	17.24641077	16.13971311	131.9345655	9.01529353	57.72993739	38.2438858	25.14517601	26.21918181
0.041389263	13.18306879	12.37890179	205.8683165	9.102738425	106.7112668	108.7283664	23.75640001	44.80668831
0.080559431	36.14702451	12.5390054	162.7927288	13.63081447	111.0256994	74.54512205	43.77001306	21.27799065
0.07518583	43.98805711	18.20347505	156.4827384	11.52905468	97.8132036	65.06179232	17.43760774	23.50835491
0.101079146	14.68363289	35.53455047	137.9710778	11.57006957	109.108531	69.22227864	21.81426368	37.75729963
0.181554958	33.69089222	27.02316561	284.2058618	17.83870294	150.1441276	171.0514437	21.27406549	68.51123781
0.013807488	8.64613441	8.787412435	84.38239766	9.222757641	74.05943481	30.69323007	18.9928479	18.56425351
0.015851127	9.484676349	6.117500249	75.58195188	7.125421024	57.39759807	26.47292114	14.07663499	16.55792518
0.153124133	29.22035252	24.20922951	194.7268305	14.03584802	139.6185081	113.8776106	37.73960263	51.27165435
0.094960207	33.07940065	7.618272669	162.77357	12.9388312	131.368385	89.03839508	28.86115929	40.5390484
0.034880242	28.01178464	13.54709394	92.67038511	11.02623222	94.07015521	33.71183785	24.45081154	27.64755713
0.033692044	26.16234793	17.73775069	174.4613187	12.02428305	91.27254263	68.11988734	19.68414172	36.02660092
0.131001551	21.1391651	53.01103845	309.3605824	18.54218905	143.0745671	80.50357581	30.43323848	76.3698486
0.015818988	14.44582449	34.86634539	155.9913391	11.51401551	78.60448123	31.20667961	28.93896989	34.66516325
0.058897671	26.29926061	30.06375138	186.5989856	10.21516503	117.438367	94.0331567	25.2295937	63.67339844
0.017309195	7.542266309	9.902094301	147.0198464	9.43643247	72.42999578	33.75767205	26.24745203	28.5518763
0.06715282	11.75234997	20.36401163	150.38891	12.89558641	87.46968036	102.3522682	21.31071063	47.0380514
0.057862163	13.15477553	11.49883952	185.8199229	10.40977031	117.8683883	39.20355987	18.6162896	32.80107445
0.026874272	12.23670602	14.96169018	150.9382282	9.152711703	83.21812878	45.89485205	27.00483751	19.82501316
0.021349181	10.80115574	7.946633916	121.9358921	9.915887825	71.15568641	53.2028301	11.01395132	19.02197084
0.037143426	17.4439397	10.74506754	151.303922	10.13573684	73.22858335	54.91980435	24.66413726	28.47173049
0.024331358	6.027900845	15.08851423	198.3765506	13.16602392	94.10684122	43.96809398	27.48267343	36.78285053
0.126328374	23.05873447	14.00205976	146.6980262	10.8176004	109.3996789	111.195124	20.00266295	31.64489225
0.102530488	39.12228646	35.36273434	100.1696302	12.83749958	68.0464413	77.90239427	14.31376596	35.22300205
0.074503013	35.36865348	31.6427734	287.9191357	16.77013366	150.8909396	99.99785745	28.23855081	46.28580815
0.107740929	23.83862526	15.6920344	168.7600445	11.79842665	111.9195678	72.51141715	25.03864314	50.60202005
0.009151146	19.49242848	5.54008588	96.6476497	5.680024757	48.53400208	5.625096278	45.20623645	7.968116926
0.036047693	18.6432373	11.82521477	145.3478532	11.63488231	69.95344641	60.50750945	22.22998503	35.74756425
0.030262512	25.05819173	46.37006936	127.3596456	10.85847138	148.8762304	20.25818575	50.10355477	62.29906092
0.047572082	12.97305071	22.28409699	235.0349387	15.14036689	128.5514306	61.50872007	26.66549822	73.66774669

0.074804615	25.154418	6.382569688	149.1071579	9.444192261	93.25948503	81.62994022	17.7312603	33.09510969
0.075457107	28.21942372	12.35926239	166.9150774	13.49062988	112.941745	53.43930504	19.59477324	59.63651531
0.027727103	21.01259002	12.63420535	137.6957118	11.82657912	95.08726313	44.39382332	18.95458339	39.57947667
0.330948767	29.74063823	68.6883352	404.7838909	15.36302863	185.528847	220.3350614	36.16805101	91.48742675
0.132870741	11.64800801	12.99082094	170.6507147	8.570063808	151.7002456	76.33294524	27.28299589	68.12786224
0.036264537	10.21755478	42.54381923	132.7855153	13.38324261	87.39486057	32.81738771	21.8754721	62.40085234
0.285453022	36.78761477	65.30396406	371.0113711	33.44718103	160.8975115	120.5220028	18.94569231	210.2036889
0.028873291	14.22144744	14.3342698	184.0903402	8.427212562	101.3999363	64.03287985	29.37646467	31.4599725
0.118157891	23.59632236	17.88271842	172.620342	11.29148655	81.4126351	102.9234781	13.85342521	35.93153767
0.036351432	26.50201098	16.55741474	127.0395973	11.45739362	92.63184565	61.16300352	20.92084604	46.25735438
0.101061101	18.76624705	31.40926954	122.2424445	11.18562093	60.42741522	69.59525611	20.94577257	36.63150199
0.113363516	28.86707254	24.17759117	173.1365856	17.7586179	67.39110833	92.33156735	14.85867741	46.91222888
0.081378955	12.51861615	31.32262821	244.8585876	12.78929858	169.3709888	116.4949731	24.81091257	105.8404362
0.035136723	20.6108376	16.75305831	121.0384407	11.65308077	74.81218779	32.57733239	19.21034549	39.62891004
0.018661083	41.66308899	7.875101082	122.3446315	7.847767756	64.84344829	43.51698043	51.28419765	15.62082592
0.08890391	5.696545852	37.23468298	276.4807837	12.6925226	137.8162878	47.69760729	23.79998934	137.9992621
0.049441053	13.13303952	8.965874755	134.2293495	8.690349204	137.406463	54.32550744	24.82400103	29.08023528
0.026443345	12.9956201	20.95888822	162.7122333	12.69282147	121.3151014	28.19864557	32.62211518	36.69741489
0.016366342	21.03117037	27.73274129	195.1377021	11.32998813	101.9264418	38.77000155	36.71117837	35.97698508
0.076010466	15.00895091	9.019707826	186.4193731	10.25899702	160.5044506	80.70115789	25.6852467	62.92917214
0.090057882	21.96032382	31.03049942	199.3757285	13.18018189	124.977869	72.34011548	29.29815493	38.63702912
0.035553629	10.79469559	10.13881944	158.7945827	10.33173777	95.32105721	65.62611317	19.31432818	34.98831007
0.037246248	8.064209055	8.848409997	132.0921435	8.322650227	100.1581332	52.4718625	25.64280064	25.10892085
0.026088447	15.55408052	48.89095231	265.3605482	24.15259421	105.8150363	41.96688172	26.48089242	94.50737673
0.097996012	25.14769085	10.72437921	77.69484914	10.13147769	78.77903497	57.94105395	21.23258388	29.69410269
0.02926409	14.01273435	28.85018429	183.9473345	11.4321397	97.00861935	51.63190166	30.16663318	30.7109073
0.412981659	29.51280637	60.81304321	336.974989	18.70951941	208.09303	349.7763644	26.07354478	198.6172509
0.024420964	10.62102896	10.3063128	94.7519045	9.7809715	68.48145182	37.6515845	15.77334276	22.57314866
0.061157374	14.98561826	13.96656315	188.6946866	9.852594452	78.90880877	73.34893692	15.76655305	37.61182317
0.36415089	41.52688904	39.5613569	170.8290724	19.01824687	98.45089038	107.5078054	19.24650795	71.34213469
0.073306553	21.02383109	33.94240258	241.8656116	19.82854265	207.7795403	64.7833587	20.83255282	93.52346643
0.104691813	19.18692531	13.77417929	292.1368373	11.2377395	151.6367554	67.67667913	25.34707	42.20889003
0.047337406	32.60262738	25.92976114	97.18001865	11.41248665	87.75179076	63.33393916	25.48427857	39.06436242
0.069367989	10.22481724	18.25706529	163.5357319	8.976198414	103.9032074	46.36666836	19.50898312	65.39782313
0.038798849	12.37523083	16.33266475	194.9902459	9.52876103	127.6388361	71.3738105	21.98195622	45.9828814
0.193981614	19.46555586	25.11670561	212.0083758	13.0264208	163.2351256	86.82747714	32.77819939	52.05792081

0.058851841	8.073829268	20.30918675	307.6512428	12.30078561	170.8013783	105.3984978	24.00050819	98.38108496
0.114936823	27.01661559	23.18760768	189.2926257	12.37762597	151.2754403	141.258194	23.96678122	39.82797449
0.099266661	33.18976657	10.57282217	214.6404473	17.46670369	135.4324695	76.37990688	24.32596915	43.18200489
0.064488746	12.17267046	15.55921102	219.8560291	10.83891634	112.9059673	83.09729537	21.18690158	56.41871598
0.153706816	20.84283444	210.6831599	527.2512212	33.5588703	239.4191728	147.2488434	62.1315251	444.5680725
0.027262987	32.45849539	6.008899834	132.8648528	12.70614678	53.832602	47.50343469	16.2177453	17.49928061
0.06893389	39.70573209	37.8376572	206.1518655	12.77057661	119.6783663	93.08359559	30.74649519	59.84552212
0.025724995	12.75400638	16.24504342	89.01353944	9.689417351	74.19044371	54.07020833	20.71163185	34.38760458
0.042531022	5.097828356	15.72460609	159.5098337	10.81148861	116.9486138	45.0282762	24.52861057	83.25866229
0.039721204	36.30597519	18.8312665	177.727786	14.24643478	87.19667011	43.14180051	23.52568366	26.48454593
0.314511581	51.13545083	83.72493666	359.8820431	20.75578663	141.2001921	238.8998738	23.22658178	197.3020144
0.023794824	7.326093683	15.57108845	171.1818469	8.799842701	81.47909057	77.36640498	28.88875442	38.93245208
0.033598204	13.21771079	22.34125388	194.454483	9.75347035	126.4399825	42.32205524	30.00465541	42.90316662
0.155764534	53.41683676	46.42560125	177.6743273	15.66919199	88.99985079	195.2349179	30.7015036	45.83145684
0.040938241	10.62264369	16.63813043	133.9342732	8.539964285	85.41212942	28.58541665	16.4293958	53.89558573
0.067505105	27.87074206	15.37795839	196.0712899	14.79920629	116.8384303	94.53310405	36.45444676	47.50554233
0.002657973	3.57080484	2.781021478	72.84709228	5.053391831	52.75570719	11.40618193	39.27421045	21.72453109
0.129941786	42.444658	19.19408302	297.3845381	17.31508273	116.6472473	135.8557885	24.36107821	51.74786575
0.080521079	35.30190979	18.25146611	183.8089781	13.0670676	109.184192	80.55197524	22.45075784	46.13406334
0.019819328	11.5936265	6.880667544	116.1384622	7.580880351	68.40210612	43.13303823	18.73985861	20.15970278
0.023514103	22.58407571	11.7093096	152.3814788	8.848850386	107.1158431	63.19709096	32.14960569	20.58260112
0.028443164	23.39051482	10.23216384	236.5983022	11.22351956	105.0952054	99.63776256	20.0662543	53.59428092
0.032701867	21.60435748	13.46622908	117.8353932	18.16663655	90.2866736	57.09603891	19.41592911	61.37998524
0.06382517	10.9494639	17.13314303	152.4935768	10.02427526	79.16606013	82.19188725	18.27667484	25.93233241
0.074693987	40.71730457	23.59516437	174.4788379	9.794322769	120.3976576	64.07728778	30.1016992	41.05650754
0.101930725	39.74497518	64.84799208	243.9257482	14.18760725	134.5498365	128.9915424	30.29858927	70.9881001
0.039837411	28.98601427	10.71417291	169.5786165	11.21915897	80.28008519	54.1674983	22.42704595	20.76944343
0.048206515	17.99680759	37.62477804	207.1084299	25.18043044	96.93749508	59.37325167	21.66101158	69.24265146
0.018102895	12.28581011	12.67670845	109.573596	10.02048094	79.94244207	22.3331706	24.57309032	31.01881583
0.006479988	4.926261918	6.945249968	116.6258979	8.270008775	60.91486514	28.66456055	21.35229979	15.4309492
0.02245028	12.14317548	13.58418248	145.1912198	8.645621775	79.418113	59.78125711	21.62822088	42.08928715
0.017511426	13.26395953	7.580395049	193.426322	8.247081126	80.41100326	63.32475052	32.11763373	17.50039192
0.024891693	6.840217132	6.216213228	85.36825495	7.074053807	70.74846947	50.63884352	14.59396245	35.2097418
0.092287626	39.87627235	14.18789389	333.7244415	16.82028915	173.7272182	179.6292247	31.28290686	26.53570112
0.02722522	15.61260771	16.44957432	141.1501058	15.79589735	91.2648429	34.86833037	25.49337267	40.78756067
0.035218195	17.3146948	14.80512883	173.7484124	8.788057092	77.23841365	55.02447459	24.19499142	28.78119617

0.023590324	14.79490117	7.556955547	110.6664715	7.813834402	85.6480084	33.8559252	22.89869828	21.6353425
0.013443674	23.15657874	37.60083063	217.8762675	20.34758717	106.9668498	40.33595952	46.75440055	45.19517494
0.041420812	17.05310911	11.70942437	108.8929265	10.16393497	62.44839148	60.53832873	14.42022096	22.97080771
0.062262672	11.64175212	29.55748486	147.059069	18.30324104	124.4407085	53.34576795	22.85319873	41.27118959
0.05367979	17.75374319	10.87772828	142.9424361	10.20312009	83.37356763	62.5092825	19.58692863	57.28038865
0.208568143	48.86397485	37.34016446	255.9973795	18.74846551	111.0906249	133.3364258	16.74782196	66.76254007
0.181135482	42.2615707	15.85149605	228.6386675	11.86099002	132.6563099	163.8799801	31.85723154	36.63197705
0.085029376	28.20115001	44.7510778	249.7208737	10.86068875	158.5240889	54.22447283	30.81627347	62.47275857
0.033915372	15.44262906	10.69383995	124.6339413	9.159945787	65.66446992	52.64490767	17.13447327	25.50228732
0.062291258	15.02725245	10.2196687	128.0939113	11.23484138	77.84563713	100.1488163	18.76939717	30.91837311
0.026101431	7.411568727	8.790470722	127.2639178	7.541956764	67.87103987	36.73131982	27.87479036	39.38212363
0.0756007	37.00491392	11.98435977	145.0394561	9.604609825	102.4853705	57.00695845	17.72165783	40.34980571
0.191877362	41.87341208	27.70393407	235.3952356	12.81272119	84.39882837	73.2644974	37.51171162	45.68038685
0.051990664	17.18112471	17.66008864	158.2996369	14.78562737	124.9538071	80.87947466	24.00424186	68.39362644
0.050492743	16.18404683	6.573082601	109.5185379	9.886944341	102.4601929	28.02110637	25.4088569	16.65501092
0.02495917	15.75738911	8.000470538	65.81175234	8.463833506	62.88867351	39.9122893	16.85563574	12.48956861
0.044203073	14.502205	6.883737702	113.4441184	9.435932021	129.7706376	59.6505855	25.45076734	21.66357739
0.079840626	21.9754287	17.6164569	258.3698453	19.31259435	70.35382843	58.79557408	24.70977714	77.10507427
0.035461918	13.61375514	8.128628968	85.94162696	9.075722365	79.77292904	34.82076486	21.76197499	18.2159411
0.018592765	11.55176954	5.174206167	144.6585286	8.49937285	70.76057038	29.55372389	27.90577317	15.85853057
0.050770329	21.4868437	17.95763831	192.4746586	12.55398201	102.8304843	83.66940777	20.64309228	51.40671371
0.24796692	54.7554427	23.5686582	238.3139749	23.41339569	118.8855164	198.8555106	27.64666884	57.24389057
0.0336958	36.43971832	6.351627988	150.9841766	11.78707555	88.39646237	77.43876696	22.7577176	29.52595073
0.048289313	22.36881784	19.55027234	109.1566659	13.92024334	85.68906702	52.24963582	18.85554315	29.02422216
0.205696161	33.61394587	23.71144153	180.0780941	17.13192369	115.2666572	146.627035	28.63465715	36.7976383
0.046525945	11.87041711	5.204993983	212.6517451	12.40150278	136.6329163	58.23792413	24.94356958	33.74176305
0.117041414	16.7394977	10.97564356	212.27848	15.07687775	70.5673957	76.28297111	32.05718377	43.474143
0.004168541	25.29323003	5.631844759	62.82514322	7.85443222	31.00968312	17.58307864	27.0247804	7.675001059
0.02300327	11.62601988	5.292740302	93.96485276	7.997820198	58.08760754	25.51243117	16.62644693	21.10754197
0.060665482	8.979183544	13.98959996	154.0799768	8.851190141	92.57270736	47.60036995	33.75620233	29.41136201
0.024405441	8.678127115	25.05866303	145.4973647	10.01029073	106.0076252	27.17428288	23.65746028	31.19585347
0.065929051	11.90073334	12.26122586	152.1183497	8.579140441	99.62535409	80.45065451	25.38413421	38.22051013
0.120160569	41.30102954	27.41593389	214.899051	14.07072025	112.661317	86.05325979	22.20272707	42.74933219
0.031570876	18.94615738	11.0993309	149.9908644	8.611104223	69.20276383	46.70420335	18.0270494	31.05619785
0.072155458	17.33241375	9.222337829	200.5468348	10.52887565	93.98052425	111.7214826	26.94654214	28.32633675
0.103227918	97.74597541	14.00687912	237.9233494	23.32350121	111.3457373	63.99120659	21.59359119	43.42422607

0.065375528	15.06098554	14.12882179	168.1586769	12.53508264	147.9923196	75.16565199	20.28981915	47.52579847
0.09250914	25.00713093	14.74102208	248.2396177	12.61056908	146.1905357	89.65910751	22.94613739	49.68676459
0.036792746	16.83231385	15.19050079	147.3696865	12.66980919	94.20056062	52.0800732	31.91295396	47.34206371
0.062510942	14.48993592	16.78529643	239.2521305	10.48627293	107.0926527	105.6708499	22.62835956	49.6039255
0.016745794	4.725474148	6.427364813	113.5312327	6.072453915	52.38856723	28.23883431	32.40216841	15.20594485
0.01764236	12.37637859	9.426091729	75.37700793	7.26486591	72.88526906	47.34307483	19.67387752	24.00846239
0.073245114	19.89805048	34.99117131	189.4835806	13.26813805	148.8766895	59.97235544	26.09300379	39.99317859
0.028603607	29.24822445	16.82644146	99.50003373	12.84643861	97.72874926	54.97634376	28.28824724	24.65511493
0.022607104	9.060189431	6.23622424	156.615144	9.303537893	59.09586241	70.55201966	18.01819634	15.50735088
0.085915868	14.58183053	18.78675842	155.1032739	12.35980085	60.31091081	73.45552173	16.95237393	34.04443439
0.051478041	16.96744955	32.46779595	167.552215	14.72762297	81.88044726	82.70302296	18.92109386	54.3558288
0.069649672	10.6775491	7.748677463	233.1560573	10.50999643	112.6050947	76.4417856	18.56702778	24.45416725
0.054609731	16.9530004	16.22981016	204.2615942	13.43994869	160.3762199	100.4621186	34.33733113	40.81270257
0.155529091	32.08001645	11.64787757	221.4795118	12.77865443	109.4220601	68.28054752	25.4523851	64.36135897
0.026784937	20.67395628	18.18341843	70.78699466	9.706573654	55.06647284	21.42139637	18.87015539	21.24221057
0.051945713	25.59193226	17.07673023	196.6946803	13.56568066	91.79206552	78.31374951	20.3580649	45.41685435
0.024562876	10.96495984	13.91271181	135.1484906	10.33555596	96.09187515	29.23143732	29.86941797	39.51077505
0.029157202	30.86496037	7.445344954	209.8686232	11.07665004	143.0587724	52.65065343	30.09697921	21.34357476
0.058954532	27.39960381	12.95018681	145.8276256	9.773952562	92.76055093	62.07022635	15.96765914	36.47370168
0.051481906	20.79851451	9.464698446	158.1165162	14.75258046	97.16864125	68.82862703	21.23807228	64.25749348
0.038322868	12.601427	11.00458614	140.4978515	9.59321573	90.05480403	50.38597621	26.42131648	46.01671487
0.06154468	30.19430833	19.59149868	110.6748495	12.08210435	117.5979175	79.8319703	25.16586275	60.13380311
0.04013635	10.89165056	12.14569552	268.160657	16.31368336	125.3528298	40.09033533	45.13259332	45.09257162
0.113781447	36.86329448	28.62114076	216.4210693	13.75129066	127.3730236	112.840311	24.75410294	69.79073127
0.031936593	20.74892417	15.37982873	118.2270511	10.47348932	82.60328989	42.44006711	21.80588509	40.026576
0.49947488	54.05360698	53.08877436	324.2912718	25.04791118	219.3345587	212.2340645	27.2024304	161.5090365
0.064825853	19.53398001	9.077637304	118.8819714	10.2652359	72.07320274	70.64907969	17.87662154	45.79168168
0.058923548	18.94046023	19.15031805	203.1563368	9.6253977	122.2645179	70.31620837	17.37266992	43.60134056
0.028100304	18.62816001	9.634131459	136.5572477	9.774981074	76.62961887	43.10853431	17.10518994	30.30326042
0.037552486	10.41648721	16.79331829	177.0545918	9.883072244	109.9595082	78.40771976	22.97083185	42.63094481
0.039278227	25.10528815	23.67715393	207.0426676	11.03242728	141.5859394	91.31694972	32.48988377	33.67708706
0.014458542	23.73287464	23.58080171	134.5840218	8.97724664	66.36568365	36.91754528	31.48316943	22.11179475
0.022948617	11.01632655	5.779914248	86.97684696	8.153097955	66.58483245	27.22824363	18.29114984	22.85988704
0.036606094	14.12899989	10.98502893	117.2838685	10.48371955	88.68386538	52.39066342	22.27049578	32.01461339
0.022402444	10.3159621	17.56259425	111.272698	13.98953405	66.08093574	21.79124715	28.99420216	30.68462133
0.050474679	13.95983928	8.312989948	183.8419463	11.80402153	135.5609056	76.82059325	28.35626824	38.35935962

0.031040668	11.67170593	15.4243214	112.3660565	7.916905232	79.18177014	50.09479974	22.42404352	27.73004419
0.170889209	30.35914631	19.1173643	192.454902	12.05541441	131.3139241	87.73710536	23.86334387	32.81239901
0.088867637	9.794906614	16.01986251	231.3629003	17.67221251	145.3319566	69.10156227	39.27252226	82.87765566
0.084503318	11.12860936	21.30177899	290.0289628	13.79180599	125.7988437	84.37753848	27.99633929	66.78012846
0.052053111	13.39771201	11.82883987	152.2335382	10.39988115	134.0368728	68.61135659	23.24441837	52.01817287
0.118669819	28.22112152	8.518225606	180.4548124	13.27003819	92.40371544	73.59988077	21.0588526	51.02206761
0.03422041	19.61902056	15.92657029	164.6428016	12.58186472	113.6079899	42.73533957	25.05341064	33.33662664
0.045200927	23.54624168	18.69323798	126.1631033	8.598014849	105.4109822	47.08352564	22.19352	24.00871009
0.051965949	12.09786967	11.58632615	130.8926311	13.59578046	84.93271376	34.42646231	27.2066837	27.88044509
0.122705858	39.18068965	16.17943806	551.5629189	17.75772868	144.9413275	307.6714863	19.97880447	105.6898532
0.034372447	10.83905097	26.07153161	182.4503899	11.42756456	109.4848033	48.47798831	29.6161665	57.19046228
0.096462147	23.82146263	31.4384913	213.779828	17.69053384	133.8605111	55.56235521	26.00848532	70.3647046
0.03286329	27.47969568	15.08364241	94.9522754	9.59300512	85.96777159	21.36249178	22.21748134	18.56573698
0.399094732	40.69025925	45.46255599	275.0119184	17.95279312	135.7888498	124.2727693	25.10149273	82.01351917
0.050899634	18.89243042	12.59163894	160.3599534	13.77911544	82.11783976	57.65213462	22.27462257	33.70857888
0.014685568	8.524713572	13.41356746	103.9817301	9.614632946	66.17687821	17.27972394	25.96005495	20.11134975
0.040761859	15.18829657	17.5243113	115.7907274	9.19695121	72.4968271	48.75873434	19.66742212	28.29248473
0.054169497	26.46925246	10.99675772	158.6813735	10.65360445	124.7577727	65.96113018	25.56987558	44.31490631
0.059144437	42.03799314	36.44241352	278.7741285	13.17247785	116.7824948	74.21921794	29.39442665	45.47367627
0.062244664	7.68655046	24.16249005	192.4963616	9.269740215	130.8433921	69.25099547	22.78142979	70.56545541
0.035422798	18.93454707	16.44197766	151.6167003	11.3101788	136.8435037	45.71748701	30.44389485	25.92775782
0.061241746	22.05175676	14.40398243	170.3104143	11.27422849	89.08878872	58.97191322	17.79199156	42.69363705
0.013682947	6.814040216	80.63843033	163.6177107	20.24758853	95.80018513	36.01493934	41.19235488	20.70940587
0.059343407	28.77809249	8.932101868	122.6979887	12.36341279	109.6970001	47.37758195	23.51644577	15.68138471
0.00891533	5.217174505	30.00224717	84.77244154	9.010508641	66.50345983	26.68976881	27.65398283	18.73003684
0.05222767	5.659825292	50.61725281	288.1602369	11.26138153	148.3340603	59.53598082	21.56560558	122.6881816
0.050996961	17.35323282	38.40674717	250.9693002	15.96017517	118.8267184	71.25771873	32.73553657	73.03408351
0.093242856	52.40985673	18.02165935	334.9530527	25.87013627	159.5349652	100.1541246	26.91694512	74.61985874
0.031446188	15.42815124	10.62684093	141.2138257	11.49522879	84.16651344	54.44508052	24.26190064	36.41828202
0.036292655	12.22311346	8.608884152	128.4927839	7.322136045	69.13279023	40.3472692	15.83539509	25.19112626
0.0863907	24.32771697	92.25666986	331.0564533	30.40255423	162.2671379	91.26707794	44.69915492	126.4440561
0.056653762	20.79998044	14.15420874	178.6693222	10.03880597	132.2342328	79.89715368	23.6362937	32.24607997
0.09611563	18.33660761	43.73751417	233.7679937	18.84881688	158.7105061	102.1008837	33.41598633	68.30621872
0.07923786	29.93211391	9.729000969	117.6130398	10.07007263	85.0011383	54.35375354	20.39138454	23.05415715
0.00432609	13.25911208	10.23585641	134.4701371	7.904305385	66.87556881	14.85010536	39.09399491	23.01872871
0.033750506	10.45166628	14.39363369	96.4520891	8.99728685	84.08765861	47.69638777	20.16123482	34.28367094

0.103001403	26.95684868	22.06455863	176.9384432	12.74220492	95.90477461	103.1062634	19.94806447	61.70350571
0.051998483	12.02916797	10.72619872	159.1639877	8.471579803	93.54774027	63.08897444	24.24536496	36.34429536
0.076542748	16.30645327	19.01961439	179.3631372	10.38963724	112.1362697	80.42399757	22.42307229	38.54948675
0.064421398	12.41506433	12.43200061	323.0546072	11.20042549	153.7119161	127.1204308	33.51510277	56.72902528
0.031074719	26.6206179	18.49139339	81.5513632	9.439663864	59.81205026	50.3309556	20.81774442	25.78627058
0.015557216	13.89801157	6.259899511	196.8252589	7.309721933	89.70120519	50.17520853	30.30169876	16.66390517
0.061249167	16.88314388	15.09289795	161.2847014	10.03978895	120.0724925	98.35228557	18.00295951	25.17520364
0.064967794	9.610039415	13.68415833	170.7595596	9.866086947	100.1647822	61.92529946	19.12226155	57.66242227
0.19963847	51.88163562	49.99716726	194.3044065	19.28970294	89.6959084	105.4106757	15.94199081	77.33591631
0.026669638	8.991036145	9.154084819	109.3502226	8.907424573	78.31176887	27.52759539	19.30331	38.14201142
0.030896684	26.70835652	12.88515231	133.3926957	11.14024332	92.21106358	49.47585381	15.81111253	17.96121185
0.029950976	16.60140189	10.32075548	95.12846938	8.17769276	75.71531568	29.5777528	18.47312612	24.31303764
0.045146543	9.689457287	20.76747531	193.3464644	9.077633866	88.81311991	73.0988558	28.3941515	44.04369009
0.107566908	18.76539586	11.72595242	218.0674689	13.80862608	147.4605018	93.72594027	20.08168496	46.87165663
0.018044174	12.72459426	8.623108369	108.9480316	7.120335541	52.54772886	29.35323826	40.8134822	9.798414454
0.006174873	6.346397028	10.95532253	90.76238262	7.172496802	46.4319813	16.23651622	24.81083449	17.23672938
0.033939612	14.54554208	11.08561051	102.8035388	9.261141462	83.03059217	46.38919082	19.06015694	24.0849105
0.092124187	45.69071309	18.83012308	136.4120811	10.36508144	68.85372431	72.89768071	18.64115817	43.78077164
0.261658724	47.4615294	21.12807503	355.430491	24.10530799	154.3544099	243.1060601	28.45266165	77.91875902
0.043089045	15.58227951	12.95133821	136.6132215	9.275464557	84.99780505	58.72307036	17.80926902	23.60425397
0.027652836	19.2633408	14.32433196	79.61676059	10.25659407	64.02108294	36.36418268	23.59387532	19.23098765
0.021808816	13.9911511	15.65251145	143.0748771	10.9104117	78.28766644	61.05485923	17.79838011	30.02335723
0.047843049	8.652072546	12.48623951	168.1757395	12.37763272	133.0226776	73.04306916	32.00984108	47.60898081
0.037300108	12.96015916	18.04807103	189.1953524	11.22132841	100.589087	69.12907272	22.63389431	46.54822708
0.052907056	14.86000036	11.57389386	145.5810191	9.324515826	188.6281881	135.205918	26.1244372	43.37097759
0.028396148	44.62615005	2.182678811	138.3047876	11.78555801	72.21321196	53.97088301	14.90323375	18.22034533
0.052021999	15.56656482	12.74772718	136.6377618	9.709016213	83.26820541	72.21807495	25.38774122	50.87667835
0.06333008	7.182797972	15.29599462	216.1002544	9.843224322	136.5680598	49.24498291	27.123419	57.04691938
0.021669835	69.50881976	12.38114343	153.5430874	10.41666917	61.32758256	38.97249946	22.75347658	21.8672483
0.037167915	13.29993765	9.726905659	113.1409075	8.689378	87.87002884	53.42460543	20.08941696	41.61289498
0.056542495	14.71047515	10.39499684	177.7320667	10.32847892	75.78329262	80.30160993	20.09264878	33.76449985
0.099804284	15.66856902	17.89059523	244.6451383	11.57685462	137.5479433	87.22701483	20.95802408	49.23409942
0.037766997	19.96281591	10.89308962	181.8235393	8.469621491	79.57063521	46.88313427	27.42139653	27.05583823
0.049763526	32.9260591	41.06988644	237.7040505	18.27167685	164.8467868	55.59107996	26.30307823	85.63254331
0.06689571	18.14560869	20.43658278	132.4286994	10.6898711	73.39614102	51.27006922	21.98368279	39.51162797
0.028464282	14.55873319	37.657501	129.0250432	15.83473843	71.79120908	39.76567386	26.24310899	20.28076762

0.020357567	14.05924697	11.42830165	136.1637276	9.932095807	72.21645067	30.25638174	26.75562638	28.11749335
0.068744808	16.66503948	10.99272227	269.012623	13.03497593	162.1935864	113.4822097	26.96367379	47.76375219
0.04128185	14.81740866	10.40289711	159.5365812	10.20300971	93.2567012	73.45791469	24.33121361	38.30015559
0.045348169	8.200082139	16.55243121	141.3218989	9.426669154	79.22227056	33.3896937	18.15944281	49.43834801
0.022112418	15.46359412	7.670659012	124.6960056	9.188926612	45.50529166	51.95864668	23.72289219	16.96267105
0.030274415	15.57623934	16.50684526	88.52174704	13.97926423	57.87206634	53.06384631	15.13057873	12.63973097
0.10092815	12.28567493	11.50213716	230.4143847	11.96230333	153.2168231	103.637846	27.28614124	53.87939186
0.07684914	18.97527431	74.36706229	374.8602097	28.96387892	238.9164123	99.43222441	32.32416522	269.171352
0.04893823	15.81037748	13.86639407	194.7545394	11.68879092	71.96913784	80.25090404	18.89147875	31.55624612
0.026525524	24.50365293	19.54073073	133.5653776	10.11811688	92.71182764	40.93605491	36.42804615	19.95045275
0.093445907	52.66698496	21.46535823	212.8908952	13.693027	101.727973	100.6619466	30.76511399	50.93068755
0.014636596	22.85967851	8.250421331	120.2237788	8.614673119	69.23247593	41.45197821	30.06304504	10.62503529
0.070029422	15.41270972	26.78159843	216.3938887	13.06982391	124.676592	89.9293253	33.99253207	45.3498557
0.055106801	14.56288104	13.28384857	199.8924828	11.3708254	125.6273005	61.20458696	32.33203512	49.07845137
0.030998127	24.46846644	40.07219656	133.6456631	10.58273564	88.9528901	70.45531374	26.50689187	39.95035127
0.051573492	20.0736837	34.26984943	218.5506489	14.9013618	143.4180382	62.22008461	27.90439713	62.13362333
0.062155332	23.33306229	41.6254277	205.6948377	21.43723667	132.3290335	83.70130308	25.81993947	86.53124565
0.032265821	11.99150079	9.751167801	142.528736	11.3367204	76.6992893	48.98492697	29.31093881	39.39176229
0.018186423	12.9036703	7.637927431	88.87238357	8.022472834	70.2772256	26.14811247	18.41343276	22.94632777
0.00261753	6.037826512	8.233135394	47.05516977	5.322551175	39.81974622	5.229882945	18.50827333	6.612284874
0.051627421	27.8689545	12.78306401	171.6019682	17.02465543	106.3234212	42.73295591	19.68138229	24.15627469
0.092114334	19.67705994	17.45849668	209.9452509	18.35633224	114.165726	71.86838446	24.98401576	38.30301983
0.051744065	14.14212374	18.41112977	117.1299347	11.32291083	94.54318636	35.88387692	19.23178289	24.10092187
0.080592762	21.10411515	20.77163329	209.0303633	11.34139823	154.59752	118.3407331	22.74042795	61.64002019
0.049550049	8.799350139	7.232062828	245.1770922	11.42012413	94.35544561	35.62464661	26.52365391	32.7792776
0.009386007	6.4658531	15.46778857	103.1172482	9.17288798	66.84071719	13.73024371	22.97820751	23.22682729
0.096049071	32.48944689	18.32609015	167.3800843	18.11064959	169.9427172	133.3618521	34.89775694	58.63197758
0.039588689	22.18150689	9.933190686	138.7754029	9.141830246	80.48948952	45.1467979	17.84454936	34.35152996
0.037782075	14.1227857	10.82214111	102.3313364	7.975514572	84.2779534	47.8157579	22.74022953	28.97823159
0.016119587	4.975714838	6.173254756	138.2329795	6.777211167	81.42442901	54.07542285	22.21806665	19.17964225
0.137321648	12.44062909	11.44289916	200.2769467	11.63496283	108.0988761	120.9332574	40.325262	45.3247391
0.09619241	22.525915	34.69916262	202.6307115	12.62592929	115.4906738	126.623471	25.33853648	78.27250141
0.043275859	19.25888824	19.24828875	233.7222597	16.77511044	161.5050486	91.25617743	31.97541373	77.90573787
0.031602896	13.43802901	9.995316897	108.6773776	13.30472505	69.35240461	38.54435671	17.03477261	13.53676813
0.080846767	20.44089917	17.31352546	132.7791576	11.71452136	114.8024893	84.94914248	19.38300287	32.10715155
0.03276266	13.69987206	9.005146155	128.0003345	7.993087122	65.40830637	31.09945404	21.633727	25.70250993

0.098280905	24.56711604	28.98618914	171.2457945	13.40204492	118.2737805	98.60737184	24.300084	73.11516257
0.018982693	6.746459968	8.817151966	99.44323844	6.921213332	66.72872096	35.76689386	28.49206059	15.37660474
0.077563094	20.00850949	13.75841576	219.0343716	12.04504287	107.7932115	133.1048653	21.16710558	37.21838913
0.066731074	31.08416087	17.07948142	194.6144946	17.69663244	156.1541395	111.9593804	33.0407694	85.97583876
0.077800393	25.28552173	19.29714935	185.4343015	13.51353578	168.5768196	84.78230666	28.11981696	55.13025967
0.070821232	10.77846928	69.08622527	271.663551	13.23466782	150.6565274	72.26138057	38.48735288	77.50489999
0.120357745	21.30049883	31.59079891	164.3995346	15.46626878	111.0776678	63.98292535	26.45961597	63.0857089
0.058967031	3.751400065	26.94950469	272.023205	12.78541516	128.7995518	72.10895935	25.95410832	122.9179901
0.040293625	16.77467012	13.62919579	181.3721556	9.94628627	97.73396913	68.34342381	23.76334754	39.40157184
0.333604126	7.406765849	70.41568329	493.8747806	18.35611081	190.5799081	111.2553791	22.03414354	330.661108
0.035958756	12.8514031	10.48056491	173.8133608	10.66983137	96.11317629	61.37233101	22.70533154	37.43708848
0.099305497	19.71275201	26.1122233	160.6609657	8.970362055	114.5786802	68.84025224	25.32197562	56.77483826
0.037981837	23.60344612	23.63304286	196.4561039	13.03698331	124.4764302	70.53653768	25.2159845	36.45103507
0.094418509	19.64825858	35.8764315	222.9092429	18.92619957	124.392637	103.7137785	18.57198496	55.10314492
0.069110515	36.54450013	17.0122481	154.1099243	8.871892934	109.1506747	72.70159	18.67462646	28.65533041
0.031779448	16.28042923	12.53294492	159.7036048	12.26944954	96.07862806	65.03128959	18.74661971	28.02700151
0.027662807	12.62188943	14.5877761	190.1806102	10.41206492	107.1906771	72.26836901	22.60010583	34.34923524
0.051976117	19.82635349	20.22554328	119.8609211	10.89019727	95.4660549	44.93876065	22.73158269	45.4815758
0.06031225	17.02988308	22.83974264	182.9421348	12.41103783	109.2699343	83.34420489	25.46348492	36.52988312
0.137954864	19.0682676	13.89572464	171.0066572	11.64150272	107.0653187	62.90660334	22.71664913	39.54029997
0.02003876	12.34247875	10.48552597	106.0337894	9.515494958	70.06462559	35.04963406	22.20068624	27.25238342
0.061814387	19.58323479	14.44586117	145.1353177	11.01079367	88.27126243	57.594818	21.23574023	42.79883614
0.029258352	31.78619682	15.34895564	154.1791246	11.98839625	106.1765167	54.82577304	19.24665223	38.8452994
0.052815587	10.86128152	38.31636556	166.024305	9.441366127	129.8916434	64.87311419	24.15481658	44.96020219
0.057208605	25.11374551	16.21771665	103.7256394	11.51324381	69.76397426	56.63981254	14.77595503	37.92654731
0.060939606	16.60969274	27.55576847	340.1807704	12.27289452	127.3526874	127.9970977	29.21414908	70.78563216
0.017726115	8.785189406	9.901093909	79.08544675	7.009006363	61.04774353	20.88461626	21.50125852	25.51012869
0.025457244	10.64973367	8.079351537	81.41290025	7.933259649	71.82393364	34.13840363	20.76610038	27.86003999
0.034988511	21.70685672	14.20510362	125.9557446	7.627215141	105.6555951	45.3538096	28.98575957	14.51768641
0.066046099	14.86126793	18.25476271	246.568822	12.74982126	103.5478105	74.98959614	32.5639435	49.50315513
0.060497785	29.50054484	41.20294356	336.4083877	12.85894581	139.1128183	94.17305316	35.73158161	73.95114717
0.014429297	7.765434448	6.546751306	103.4870662	10.23067737	59.12801123	34.04421058	21.67492104	24.31411605
0.068342748	13.97858482	14.13279066	149.5137775	9.750307126	89.55649463	55.61837447	18.17845844	47.27458583
0.113328194	24.6706806	40.75799484	259.5087961	14.99069544	136.5602121	161.5861378	28.18755161	58.4800446
0.032702866	13.27346132	11.92480073	118.9833936	9.227695402	85.98474566	49.81570967	20.18114359	45.5260406
0.161133243	22.46516713	52.2780687	242.7601733	16.7582135	133.7047499	149.6609475	19.80550076	81.23079982

0.031723262	8.662055019	12.43354333	140.4747655	8.811318797	108.5280188	30.31624321	23.82600187	33.59550512
0.024473058	20.600027	17.16549503	140.17963	10.36549257	100.3442884	56.06120241	29.83637397	31.28811529
0.061837879	8.874590178	18.54465537	164.5725697	10.4184656	123.6743307	60.30081758	20.52653295	80.89285004
0.017526614	11.19087685	7.916896862	118.9025065	6.531057218	43.34940558	36.80364556	29.43430816	13.94179085
0.034495841	7.693229852	18.18577892	139.6001297	8.109900898	116.9610849	45.56863067	19.77565413	58.03542019
0.067271265	16.91412424	16.25169767	240.5796082	12.840343	155.6868179	104.2350011	27.27635741	66.89242374
0.033339599	12.10805157	8.876532179	111.2521409	10.54400906	81.88934857	46.32731041	25.60629761	37.45941697
0.031093111	18.98301364	13.75037689	160.5185455	8.986526039	80.04787205	49.95045459	35.41265638	34.65409234
0.069448286	17.75685083	17.36914194	222.9756341	17.36558228	112.6418981	60.75661796	29.23546306	61.53624554
0.016588524	8.866029801	8.812303463	110.2136858	6.816170341	75.72688717	33.11260795	28.03350778	15.23582305
0.093116336	28.65539832	24.66464674	218.2483572	15.33547739	105.5033455	136.8389323	34.06744337	45.22456146
0.126153449	20.49540502	18.06865883	186.4749833	14.42706362	103.5220447	88.35836328	24.86582316	51.17489844
0.098775067	14.23745306	20.65203029	193.0259656	11.86823577	105.7450991	92.42878604	45.24160236	60.78159061
0.025993596	13.99495241	21.73345818	140.8181506	14.17449641	84.98856283	27.04564589	27.49234657	37.68298136
0.00435213	2.28610286	6.57960815	69.79952071	6.635027172	38.82931736	30.18495802	23.27768253	17.14940075
0.009136139	8.717668308	13.91073134	180.8890432	8.636583012	90.20616853	23.71752985	33.74372791	27.78434344
0.0520668	19.83356988	18.40354921	128.5158509	10.77713645	98.71989782	64.22162452	23.72162979	33.02445046
0.029043716	26.99197149	24.66380037	186.1487302	14.34520289	82.82021512	90.24855496	24.26487265	49.72367863
0.011044342	4.628075811	14.34180475	162.4624249	7.527046059	104.7909089	26.06412424	32.70901837	27.9771076
0.095160393	17.75777782	24.37790439	246.8675806	13.92601038	125.1886412	132.9551609	30.54070776	65.48527326
0.014571053	20.41767893	10.04206159	88.91848948	9.554725508	63.9906619	41.416004	25.98683559	23.57496691
0.010269054	10.48250437	8.4037033	62.73877912	8.091840069	43.75296215	28.76114528	18.00755289	17.32852617
0.047989905	18.7921949	11.52176805	135.4983777	8.841381831	86.88879424	93.09991108	24.59262168	31.63636818
0.028863823	15.41973044	16.50750263	135.550168	8.613645625	74.20809338	38.32589854	20.48177144	37.76036045
0.066497037	26.66293261	18.28935958	183.4761937	12.80047874	93.17588197	68.17899299	35.88559996	41.73444249
0.024516224	11.339454	22.48414666	150.358282	12.0710831	100.5758736	48.03064629	22.54225838	34.03053503
0.194862674	13.32815704	72.05884255	260.0267503	23.86645264	134.1136168	94.34005225	43.46532644	105.4287933
0.016309689	13.14042862	27.88671689	190.3562838	11.42093992	84.75481136	49.91813318	25.2830552	36.39810298
0.019216243	5.297918947	15.18580314	174.3862599	8.395806036	101.7693634	49.27734792	27.84137953	49.4793781
0.047135271	21.99364604	11.27816406	52.67202437	11.15937501	55.36959146	61.02263999	23.03249405	20.73411319
0.055004965	23.37714575	21.52208703	135.825965	12.70892038	111.9340045	113.6280883	19.43761936	44.72786699
0.040681911	25.83457236	28.086653	139.5774474	14.91666688	66.6606219	48.11743885	35.80415248	32.2368831
0.018783107	9.383439777	7.237752612	99.98226009	8.297025641	64.56489706	36.72191955	21.60545738	22.58008297
0.016549367	12.60583191	8.847927656	119.9006697	12.95167278	76.75796647	38.55774688	26.17017091	38.67704219
0.027762034	19.73763863	13.8636454	140.4416433	15.82224517	57.16528754	61.74498296	21.55068608	28.83572354
0.018856643	6.676195149	10.78852964	165.5169295	7.859178954	89.81900997	35.33060843	28.69863638	37.29504656

0.029280234	14.36719887	19.89402592	109.6330613	8.302512932	59.58618626	36.11116056	20.68179529	24.40513345
0.010481865	5.975407315	4.969324898	74.76905553	6.273755263	46.83168441	28.18549428	20.2971593	16.40910222
0.045767474	33.86469503	14.20282554	137.8137358	12.56090977	93.99871318	87.16848772	23.82970233	26.64130055
0.024085957	16.10281931	36.86412103	136.7001019	11.10698184	79.46023389	47.23471326	33.32694904	36.96471414
0.024950565	12.59889073	4.306764042	165.0881392	11.13316728	119.1718556	58.01530787	17.00272048	32.94760396
0.137995803	34.87653028	31.23157273	207.6079795	15.10819587	128.8986271	155.6040173	22.42377738	65.8307293
0.026076605	15.24842939	15.3742358	123.8992961	9.630176316	85.37365788	76.31751317	24.8851811	21.59642513
0.035180986	8.822010028	26.81813305	238.6630924	12.32686203	102.047669	43.61985527	32.27660356	38.73210166
0.022302166	23.18549009	8.75458599	103.4291328	7.627735714	51.6504817	46.15261268	21.88457355	24.24532515
0.098931246	17.93310805	10.23740894	188.9223566	12.73971209	105.2728714	70.1296749	26.04007359	31.07870782
0.019303553	7.270274472	21.20166863	210.5493509	16.15755818	88.15582975	38.95988594	27.75751689	54.46802168
0.005734536	8.989564708	9.222337991	137.0897161	11.73856829	83.10793036	21.30664343	30.58708283	26.79933597
0.032465244	18.89942603	37.49853615	120.7112734	12.69727927	82.48526295	41.72664539	35.48545239	53.281945
0.071519061	27.34962314	21.14542195	175.98353	11.17759531	97.68670544	58.01717858	39.50134634	24.70593862
0.029928486	39.12156985	22.7582804	209.3349236	19.61549292	105.3393143	70.54729434	26.2755597	52.21232605
0.012648573	5.077363906	23.64804597	170.7547909	7.337100184	106.9487736	32.29743495	26.52159692	47.78053536
0.0421298	18.02105161	29.08895247	199.2864873	10.58734137	102.8053618	87.46704867	24.27327352	54.57305945
0.033977401	10.95551827	14.35507	200.5038092	14.42465089	98.42105151	111.6264071	24.66684044	51.01670594
0.046252224	23.60416206	16.37196911	166.9553977	12.31967619	79.30484406	61.82924936	28.08949511	35.37405376
0.012857428	26.18413888	27.3400306	90.50261606	8.543548258	51.60472818	19.97140685	22.83140972	21.11525205
0.015323512	7.044898332	18.98092375	127.4806422	8.905799145	83.25570888	22.61089074	27.46464371	43.50391866
0.018583624	9.589030429	6.476060312	131.5870844	8.421606434	83.77421959	50.56689173	25.65367168	21.20281407
0.013657395	7.356565971	12.37530812	133.6982146	14.4768261	62.63120533	43.57821071	24.4770001	25.28483996
0.023932638	31.48439239	10.98114939	93.68405629	14.02748844	43.26830541	28.87026332	29.58327048	18.73714723
0.035519629	20.04870177	16.23075963	148.027724	8.002519533	73.4056179	60.91071202	27.41599091	24.73224467
0.085759253	27.61396775	21.52917764	129.116938	10.24199148	106.8740433	108.1055972	23.01191552	42.20640261
0.188891866	26.27767882	46.98428593	262.5031077	11.69917549	118.2636189	142.3809323	31.22727766	57.58286066
0.02061414	9.03517732	9.935109331	112.5311694	7.95994766	79.76368008	30.59314976	22.78582866	30.71166901
0.006464908	4.076807846	5.009715331	97.86219957	5.879838028	43.74565185	17.08007076	24.33156136	14.28208824
0.020915813	20.98728069	29.64474634	117.7310313	9.60906237	90.99386452	41.77096116	28.68351141	26.9274403
0.044184661	16.59796444	9.638590462	218.8544609	13.13292457	114.4838768	103.6118455	35.19115552	40.9076104
0.010107843	8.66322117	9.103850633	121.5486154	8.789992902	49.65485457	21.07144655	23.63868688	22.50624234
0.007518434	8.234719078	10.14572326	83.54219829	6.443834904	55.87770293	26.6531762	19.79513754	13.21187339
0.083196641	38.08276499	47.45207521	323.9887574	21.8310109	202.2120072	225.6892809	32.89016213	212.1941857
0.015551797	8.022321815	9.972841674	137.1079102	9.847619321	87.46121189	53.46416265	40.73175986	24.32939418
0.014758106	10.29831227	9.986111058	108.6714751	7.807350674	68.15420472	34.71268508	16.89649299	18.11015495

0.037152582	15.21244993	13.70482663	155.4663752	9.691533209	105.492356	66.50263863	44.69820973	33.89755929
0.008402909	7.024888658	83.02888853	102.5401578	9.529710444	70.71327337	29.27690282	49.37116018	34.68014175
0.042177194	13.33574598	12.28840207	222.1133479	9.953089559	97.46273801	76.21249401	31.11404535	31.89826371
0.077808287	10.40055792	12.28585715	186.1907632	10.2900426	111.3509525	80.00326317	23.45592536	43.01751982
0.02876803	14.64143866	6.571480678	107.5150923	8.362864969	84.77452652	65.61825828	26.20957767	24.81336399
0.083211098	21.82071507	19.7749315	150.0239104	9.078445402	87.35710622	73.66872957	20.73179752	30.86061991
0.021536909	8.556808717	14.64611288	61.25724082	10.71890133	42.89953647	39.74155254	15.34207492	14.74210625
0.030391053	20.91832126	9.742129126	103.9835788	10.85852167	53.50449064	34.72804131	15.82198095	23.29626202
0.037405227	9.542106641	6.864089245	182.1662666	8.768775454	93.33379794	70.95085272	23.57845176	27.77852958
0.078658472	21.21092029	23.37562866	230.8811283	14.16754532	119.8890499	111.1372791	34.70576909	59.88303639
0.042028878	13.90298094	15.90637714	131.1793881	10.08805764	87.09542264	78.07122346	25.72130304	36.73734736
0.014125441	13.88726776	15.98997252	211.22	8.340549571	105.2162935	28.65370468	31.20510662	27.82287415
0.01976513	14.3017558	50.55023518	111.1068094	13.09357065	55.62879667	33.77529241	33.43272689	35.51100777
0.017853299	12.76672595	22.25160495	163.8551657	13.98951891	80.54724893	48.86308958	22.28776417	51.15652106
0.009387399	10.38835769	7.018990298	96.09255478	6.344757098	57.21836434	24.61488773	29.88197586	18.79244762
0.016530351	9.046495928	6.537092429	97.17885119	7.333853938	70.24464745	25.61428015	17.49736858	16.22576231
0.011980066	8.732975742	4.773703819	93.65401922	9.990512078	58.30487527	39.05313489	27.90007613	25.75161023
0.015941243	7.666688958	41.36932338	122.6920958	9.891970912	88.69326325	34.89073561	24.93668266	43.50009223
0.05947832	8.669193362	19.76300985	175.4697635	11.08726739	100.1720399	66.47827624	27.23267747	59.41114407
0.030821242	10.76777484	8.127229873	124.9537072	7.168463668	73.05651596	42.32326727	22.9080466	22.17341649
0.046292235	13.96297388	12.77932347	297.1600489	16.77040769	115.8065285	71.38706411	36.55071503	71.85258299
0.03876224	11.72595364	11.31684924	141.4150427	10.92826729	83.29101269	61.40936941	26.7690253	34.60607999
0.019708107	8.767390965	10.02652343	233.0915644	8.767092872	148.3246266	52.26266448	26.36820365	35.25421992
0.013083613	7.807361477	18.47487965	172.0914194	12.52204498	74.04955395	59.15969933	23.61655199	35.60567607
0.015639636	5.963796298	9.671924105	133.9621996	10.43366996	67.30413693	54.6046741	21.53921343	23.2237876
0.027829874	14.48940745	13.52246487	157.0702554	11.08625361	93.41120896	65.5332064	24.46131125	30.61212526
0.038026649	14.37235023	21.89655293	193.079483	9.708300101	94.76238225	86.96742936	32.37766703	34.92321849
0.077908054	11.21022786	6.90649925	187.7169355	13.90974505	92.51358483	119.5223776	22.22424939	40.24828205
0.054754156	14.37432566	23.57066421	203.9122423	9.718969128	117.3506388	85.24832581	29.56732913	44.68267067
0.076824428	13.35429259	21.24337921	267.8185667	12.73875965	127.0794035	97.89862962	22.64203699	57.22112106
0.012674293	4.532395774	12.02207619	138.4215668	12.89828732	103.5317108	35.04106033	30.3902932	47.19286869
0.019561651	6.96015822	18.34010495	179.4972007	9.016936691	119.3020965	52.16150731	26.86468572	42.25103748
0.018287477	29.77837361	16.48232849	107.2191753	9.069503377	72.80102494	34.08728918	26.03221282	19.62711645
0.052965813	11.64975697	27.38392391	207.7460929	9.579058392	131.7415585	101.1566249	30.3225475	51.20694559

BMS-754807_2171	JQ1_2172
1.481805138	16.310514
0.467143362	4.0175733
1.79303182	12.143664
0.988026463	3.4866016
2.654308178	14.885715
0.157294564	2.9365282
0.607639801	6.1656934
1.008912654	9.5778915
1.58598099	20.376407
0.442785978	2.4097305
0.598771539	2.6114721
0.609045161	3.7004731
2.951107997	26.843532
1.013570504	2.1290874
0.856568373	8.8910223
0.465408081	4.0362817
2.072374087	15.403148
4.957393094	36.368543
1.399904817	10.36702
1.061626984	3.7503544
0.422669298	4.9110087
5.282552345	20.827345
1.424332405	15.682509
3.95075726	26.726225
0.989678274	6.5475068
0.724890875	7.1259035
1.983579525	10.128943
1.08408904	14.907219
1.251939248	8.7494049
3.027218613	23.968641
0.478959137	4.9059508
2.794163698	18.872742
2.188117369	17.632384
0.890688121	6.9158601

1.985671302	22.801567
2.912567304	25.307298
2.997684506	12.69099
2.437102322	28.256599
1.363054875	8.8628377
5.358503022	12.886858
2.942043814	25.675669
0.204486117	3.7101435
1.74896703	16.436885
0.759663218	4.7714247
2.660924025	24.038944
5.593277326	8.846536
0.585148994	4.7834116
1.110912639	13.819041
0.43509346	3.4856946
1.717158335	15.629087
0.220165517	2.2319573
0.641210763	4.1012787
2.203851176	6.9033552
1.675250912	8.147742
1.476050739	8.8983776
2.828329764	28.281494
1.027190647	7.4448266
0.283958295	2.4501944
4.924765	54.635137
2.892272788	27.556226
0.883820546	7.5707975
0.793794062	6.216901
2.705507122	14.862141
3.919293025	16.201365
0.299690149	2.6366368
0.685832712	3.9985474
5.158935048	29.354673
2.884410522	40.127394
1.530420982	11.655883
0.707807387	4.6542284

3.691943696	24.381189
2.125970384	10.419276
3.13277528	14.063955
6.217944986	49.463069
1.089796779	6.6299895
0.217825882	3.2141519
2.230618694	8.2719597
1.223330066	14.831226
2.787322848	13.519666
1.527870495	6.832919
2.909895059	17.797087
2.637077448	17.717481
0.709354815	4.575423
0.27813414	3.4515568
0.664752635	8.9490706
1.147051259	9.0866319
0.579187514	1.3698359
0.479346137	5.7370099
2.152954289	17.812927
1.621369171	7.6054625
0.916142861	6.1537845
0.755092038	6.7376611
2.133253425	17.702143
0.560411464	3.9385062
0.976356733	5.0889223
0.359117897	5.0332007
1.116595404	7.430608
2.496447478	23.249265
0.626509193	6.6932872
0.928847882	2.3975038
2.652213781	14.433783
0.998729463	7.9091534
2.382022348	8.8713143
0.654007229	7.6521531
7.993556755	26.748303
1.817326229	11.99008

0.666661616	6.1942388
0.36208905	7.4075848
0.923793725	5.6192684
3.978353591	22.486636
1.178955077	9.8591486
1.650166186	7.8874026
2.103983759	8.6342361
0.655043013	6.7912013
0.952744892	7.8673085
0.930963197	3.9249298
0.964451683	6.2828533
1.223488871	12.729406
0.739651903	9.2318881
0.358485433	3.2781263
2.851544187	8.0403203
1.88322644	9.2224837
1.64555256	10.673913
1.200497542	11.649928
1.868291415	8.6681083
0.40981861	13.792671
2.012443829	10.695126
1.245090103	11.286056
1.684322617	11.754331
7.720189756	46.819608
0.360614634	2.3848663
2.075557043	15.441413
3.870534711	22.456461
0.474420322	6.4019889
0.797403361	7.5345131
0.561701156	2.7147281
3.199755221	25.061268
1.015332891	7.9657523
0.817851815	2.187953
1.957160146	4.6261284
0.615177435	4.759295
1.219900283	6.9860285

2.284253809	10.774132
1.403659213	8.0969675
3.485937351	20.49801
1.325431557	10.18977
9.578863334	64.008869
1.958464205	11.642856
1.092685612	4.4785668
0.527908296	6.6668602
1.612042837	10.550696
0.830651566	5.4030408
1.545335254	15.955408
1.25239608	28.439142
1.558081774	9.5004196
4.295459054	10.597172
0.326069082	2.0952048
3.973779196	13.171591
1.491078207	12.606848
3.299136934	23.586471
0.950313543	4.5361816
0.219288285	3.849494
6.648755038	35.638977
0.680044882	11.184662
1.228404903	10.879506
1.419196971	12.32625
0.669530231	5.8996754
1.759942981	13.094547
0.953119473	8.2704967
3.169892489	16.443304
0.71676064	7.651177
2.319899063	17.576607
0.41196826	4.0390561
3.567199749	41.760622
0.499368896	3.916026
4.262327021	19.958673
2.438303549	10.68798
0.73027799	10.33324

0.12210694	2.1698861
4.685497083	20.590223
0.727076168	4.1798211
2.376515374	16.921851
0.440524105	6.9037596
2.13547967	14.851552
0.565182612	9.6989203
2.987227181	7.6761813
0.28846413	2.2119167
1.133194563	13.219226
1.208579443	12.446514
0.51172764	5.2587123
2.977323257	7.0123103
1.505990294	7.6512774
0.675289239	4.7275222
0.61072944	3.851522
0.536647841	5.9722376
1.300344061	10.343125
0.335313412	3.1696403
1.4918606	8.5512604
1.170242957	8.628924
3.998586255	19.085833
5.029300521	21.122731
0.731204973	6.3390206
3.106337488	22.423612
2.220634155	23.008157
5.690003116	47.39991
1.105033636	3.7244029
0.103645953	1.9873475
1.641225244	16.128118
0.706370014	4.1614669
0.513622335	5.8362104
1.379643356	9.3149085
0.265798636	3.724718
1.373541104	29.004238
5.006964534	14.919609

0.415055512	5.7582199
0.769136033	11.181115
1.613964574	12.490313
3.565719221	26.463665
2.21896802	10.02765
0.437796012	3.3325505
1.224304116	7.8351636
2.139671696	18.371837
2.156389378	21.313885
0.919603076	9.525425
2.442770475	6.8382097
5.494985208	24.727575
1.83138647	15.338742
2.180434578	8.3552175
0.156747696	0.5415865
0.561551901	6.9644095
1.286527255	9.3880249
1.938169934	14.968927
0.147441337	3.669069
1.592165542	23.790168
0.563862933	7.207134
1.114048871	9.185108
6.502854111	33.883238
0.896502744	7.5930068
0.995666051	7.8022385
1.481603521	14.781536
1.591013306	5.6700136
0.479361124	4.5492589
0.231036411	3.3047541
0.726565871	6.3067339
2.676457193	34.008505
2.129427362	10.383385
0.170983541	2.6324838
0.503226836	4.5731357
4.271140356	24.869145
2.202324334	30.497533

0.657056834	6.6047478
0.787963443	7.406147
2.056100415	13.47496
1.509902877	11.081775
0.320634259	5.8767535
1.004713842	8.8932811
1.167059877	10.378143
0.837442071	3.9404462
7.355029386	29.270544
5.223895738	63.412233
1.940863243	11.482033
1.03788355	7.8492031
0.351740567	1.3203542
1.126637639	13.168175
0.442812911	6.4927305
1.645626307	5.7928627
0.379706557	6.4405491
0.412361469	8.5204659
1.453705907	6.3998836
3.277830469	10.095959
1.175848284	13.163201
0.304662576	4.3739982
5.656668721	16.697991
0.66142756	4.5752772
2.609605129	17.746278
1.819398676	15.480662
3.253679334	13.609179
2.161252739	16.996692
2.304944013	18.497187
0.38138541	2.9667466
7.469971	24.663141
1.101743411	7.8631802
1.191662122	9.5620632
0.74919277	3.9583647
0.942555413	8.1307344
0.490574788	3.3702178

0.511875885	8.8717977
1.043042242	7.8411962
0.728242466	9.5802642
3.366144652	31.931886
0.445122523	2.2685666
0.731035679	7.4414348
0.322647923	5.6295539
0.836942749	6.8616904
1.961338211	3.4792987
0.353347409	3.2566642
1.020581806	5.5362937
0.126231445	1.1070783
1.30239606	13.780192
1.268551024	14.863908
9.384680078	25.651073
2.845029949	7.2916053
0.683615304	5.1047216
0.34671899	3.2051206
5.803713101	42.224071
0.559679235	8.0821258
0.675845523	3.870674
0.929366752	7.2307319
1.965094332	17.566541
1.110636388	8.9997202
2.185171785	10.483214
1.596819941	7.469556
0.285525117	5.0385608
0.550959627	10.073393
0.863988457	2.4214624
0.096066537	3.3358315
1.678297195	13.888612
0.950914297	20.516439
2.400249547	11.837694
1.700710056	7.7776502
0.610746917	3.9696563
2.340223059	9.7842149

1.174807531	5.3060426
1.655202035	11.703067
1.100725255	11.463201
0.644038071	3.8800068
4.23857755	17.147398
1.996155958	9.5208529
1.202729709	7.249775
3.705649074	25.584843
1.236084722	13.624093
1.48638511	7.6968859
2.626530366	17.764999
2.0880254	7.5582886
3.270599933	18.556224
1.025874347	12.530588
0.59042042	3.761384
1.370514217	7.8228092
2.589979854	17.905476
0.706706854	8.018709
0.151589085	2.2415785
0.522985404	2.4123147
7.084666306	19.564845
2.765289814	27.251415
0.71213706	3.641312
1.014407572	12.242723
2.227022593	17.61491
0.605012688	3.704079
1.21261285	16.861753
0.434831681	3.3560019
0.095122835	2.3618106
1.189404115	16.184801
2.750686602	32.463003
0.305527634	6.2044429
1.369098631	12.131643
3.865682566	18.372478
0.563731856	3.8087723
1.307643855	2.5313186

1.416848694	9.9349575
2.003415507	8.7645298
1.463677257	18.85602
3.360609348	15.760568
0.905953507	11.439317
2.752675217	16.019486
1.362862315	11.264796
4.322501921	26.633636
0.402056983	6.5452653
2.974195798	24.914597
0.569484448	6.2836498
0.692419099	5.2672374
1.962993992	10.812612
1.404597939	12.879358
0.790286154	5.5534091
1.625058814	21.202524
1.023480512	10.736552
0.247238921	3.4198946
1.292590923	9.4564262
0.365706699	4.5280406
0.302129313	2.5871986
0.20819638	3.4262
1.627322229	8.5436216
1.163396179	8.2292453
0.521264143	3.8121624
2.809531368	19.337976
0.156846616	1.7458518
0.357318349	3.2214189
1.102206849	3.4800082
0.986667111	11.94254
1.817901876	7.0756226
1.340768797	15.071285
0.350783598	3.9404788
3.230621252	20.440629
0.72766479	6.5273466
0.53987264	8.8756657

1.33124647	7.927247
0.986900398	6.5774472
0.658288226	5.2299257
5.173233689	16.588058
0.279329972	3.4532714
1.041451926	22.35387
2.995055842	19.69497
0.774914108	5.4334045
1.480874631	7.5800592
0.586633778	9.2264001
2.150606325	11.832864
5.97054647	42.55459
3.870357086	24.551437
4.102984237	23.593304
3.355344302	39.234063
3.100116996	15.189033
0.473268257	5.2603484
1.757517266	7.282073
2.082476884	9.467805
2.291998711	24.574686
0.876196409	4.1536372
0.709553489	5.5798531
1.329135378	12.666123
0.208529061	3.0188292
2.184972023	13.861125
2.106817418	11.79222
4.150258023	46.476334
2.783739358	14.415091
2.629601067	16.026704
2.565255504	6.19071
1.497256138	9.7413818
3.718548073	12.065496
1.370598527	8.6877156
1.635262462	16.095983
5.805097615	22.816627
2.33509859	17.210574

1.986024185	11.082554
0.540990702	5.282471
1.534486461	6.9291068
1.465423652	9.5603706
1.847797336	7.6035196
1.301627769	6.2934748
2.515156437	9.5594607
4.473310603	25.122865
1.138882472	7.4499376
1.64835744	13.84358
4.486700361	16.87735
5.579215653	45.196141
8.674040298	24.881826
2.472149053	10.358615
8.381083864	66.59684
2.142748473	14.198708
0.881827413	8.0412277
6.432221078	40.238941
1.747073861	5.7318378
0.364516864	1.6884196
1.676503975	9.7771272
0.803860689	11.879012
4.872064719	42.88108
2.977018256	7.4862544
1.889951269	8.1105263
0.855313023	4.2320481
2.393927754	15.815568
0.387861255	4.965884
1.071045335	9.9168824
1.642524253	7.4466818
2.206509503	21.156125
4.155515926	28.481545
0.439366778	5.0116891
3.793306548	38.518255
2.27392487	23.223437
1.947324913	8.2629724

1.850179616	13.814075
5.362591221	29.985919
1.144287792	8.5469921
1.566547846	11.385787
0.549197038	6.9247461
0.62868823	5.2400394
2.177079248	10.045532
0.512535498	1.9755071
0.886040146	16.082241
2.176480084	22.16241
0.485287054	4.9459006
3.146398086	16.28392
3.148899069	13.946923
2.539753364	10.835493
0.728329742	4.1070328
0.433737993	3.7895431
1.453180083	35.028447
3.377667958	21.853132
1.298100544	15.47231
1.559632707	7.7931835
4.651735023	21.427113
0.971031502	8.9757966
2.139559994	14.95575
6.507059245	38.863033
3.388944937	37.859893
0.842774767	6.9381037
1.312882861	14.662537
1.766748582	36.803548
1.253209567	9.2810891
2.205640737	17.341097
2.059715091	35.197156
2.078128196	15.792645
1.406898893	4.3309248
1.119558978	8.1502916

Supplementary Table S 8

KEGG_ARGININE_AND_PROLINE_METABOLISM

https://www.gsea-msigdb.org/gsea/msigdb/human/geneset/KEGG_ARGININE_AND_PROLINE_METABOLISM

ACY1
AGMAT
ALDH18A1
ALDH1B1
ALDH2
ALDH3A2
ALDH4A1
ALDH7A1
ALDH9A1
AMD1
AOC1
ARG1
ARG2
ASL
ASS1
AZIN2
CKB
CKM
CKMT1A
CKMT1B
CKMT2
CPS1
DAO
GAMT
GATM
GLS
GLS2
GLUD1
GLUD2
GLUL
GOT1
GOT2
LAP3
MAOA
MAOB
NAGS
NOS1
NOS2
NOS3
OAT
ODC1
OTC
P4HA1
P4HA2
P4HA3
PRODH
PRODH2
PYCR1
PYCR2
PYCR3
SAT1
SAT2
SMS
SRM
